# Asymmetric Aziridination of Allylic Carbamates Using Ion‐Paired Rhodium Complexes and Extrapolation to C─H Amination of Phenethyl Carbamates

**DOI:** 10.1002/anie.202507532

**Published:** 2025-05-29

**Authors:** Arthur R. Lit, Shotaro Takano, Christian Zachau, Ioana Băltărețu, Robert J. Phipps

**Affiliations:** ^1^ Yusuf Hamied Department of Chemistry University of Cambridge Lensfield Road Cambridge CB2 1EW UK

**Keywords:** Asymmetric catalysis, Aziridination, Chiral cations, Nitrenoid, Rhodium

## Abstract

Aziridination of alkenes is an important route to chiral nitrogen‐containing building blocks. Here, we report that carbamate‐functionalized allylic alcohols undergo highly enantioselective aziridination using achiral dimeric Rh(II, II) complexes that are ion‐paired with cinchona alkaloid‐derived chiral cations. The aziridine‐containing products are amenable to a variety of further reactions to generate useful groupings of functionality. Furthermore, we show that the carbamate group is effective for directing highly enantioselective benzylic C─H amination when it is appended to phenethyl alcohols. Intermolecular C─H amination of phenethyl alcohol derivatives has proven highly challenging to achieve asymmetrically yet it gives rise to valuable β‐amino alcohols. Both processes result in rapid access to versatile, highly enantioenriched small molecule building blocks for synthesis and highlight the effectiveness and generality of this chiral cation‐based strategy for asymmetric catalysis. We report studies that probe important structural features of the chiral cation and demonstrate that the ion‐paired complexes can be formed from their individual components without a separate isolation step.

## Graphical abstract

Aziridination of alkenes is an important route to chiral nitrogen‐containing building blocks. We report that carbamate‐functionalized allylic alcohols undergo highly enantioselective aziridination using achiral dimeric Rh complexes ion‐paired with cinchona alkaloid‐derived chiral cations. Furthermore, the carbamate group is effective for directing highly enantioselective benzylic C─H amination when appended to phenethyl alcohols.

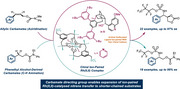

## Introduction

Aziridination is a fundamentally important transformation for the introduction of nitrogen atoms into small molecules, enabling their conversion into a variety of building blocks.^[^ ^1^, ^2^ ^]^ Serving as versatile intermediates, the aziridines can be manipulated to introduce various functional groups with vicinal relationship to the nitrogen, often with high regio‐ and stereoselectivity.^[^ ^3^, ^4^, ^5^, ^6^ ^]^ Therefore, the development of enantioselective aziridination methods is paramount to realizing the potential of this functional group for the synthesis of chiral nitrogen‐containing molecules.^[^ ^7^, ^8^, ^9^, ^10^, ^11^, ^12^, ^13^ ^]^ Although aziridines are often described as the nitrogen equivalent of epoxides, enantioselective methods for their synthesis and manipulation are less advanced compared to their extensively studied oxygen counterparts. This is primarily because the preparation of chiral aziridines usually demand mechanistically distinct strategies that differ from those used in asymmetric epoxide synthesis, making it challenging to directly apply key advancements from asymmetric epoxidation to asymmetric aziridination.^[^ ^14^ ^]^ Nevertheless, great advances have been achieved, which include pioneering reports that harness transition metal catalysts to mediate nitrene transfer to alkenes,^[^ ^15^, ^16^, ^17^, ^18^, ^19^, ^20^, ^21^, ^22^, ^23^, ^24^, ^25^, ^26^, ^27^, ^28^, ^29^ ^]^ metal‐carbenoid insertion into imines,^[^ ^30^, ^31^, ^32^, ^33^, ^34^ ^]^ organocatalyzed methods involving alkenes conjugated with carbonyl groups,^[^ ^11^ ^]^ as well as significant progress in biocatalysis.^[^ ^35^, ^36^ ^]^ To highlight recent advances, Darses, Sircoglou, Dauban and co‐workers in 2022 disclosed a highly enantioselective intermolecular aziridination using Rh(II, II) tetracarboxylate complexes bearing chiral carboxylates.^[^ ^37^ ^]^ More recently, two independent reports on the use of chiral (Cp)*Rh(III) complexes in the asymmetric aziridination of terminal unactivated alkenes have emerged from the Wang group and the collaborative effort of the Baik and Blakey groups.^[^ ^38^, ^39^ ^]^

**Figure 1:**
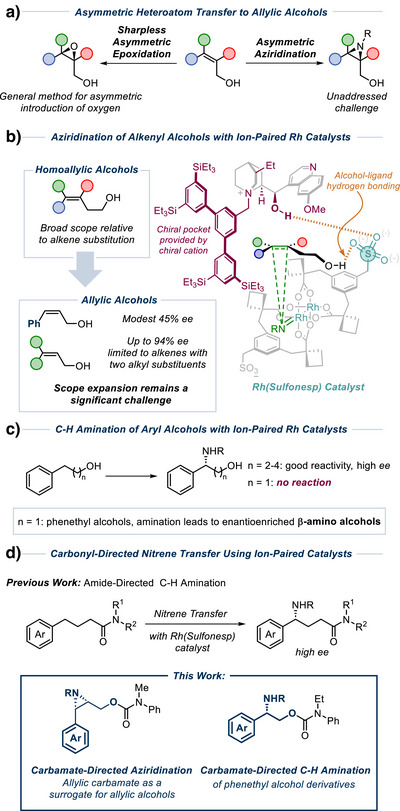
Background and relevant precedent in nitrene transfer using ion‐paired catalysts.

We have become interested in the challenge of enantioselective aziridination of allylic alcohols, in analogy with the Sharpless Asymmetric Epoxidation.^[^ ^40^, ^41^, ^42^ ^]^ Considering the impact of the latter in asymmetric synthesis, it is notable that an aza‐equivalent remains undeveloped (Figure 1a).^[^ ^43^ ^]^ A number of studies have demonstrated that directed aziridination can occur in allylic alcohols and related substrates, but this has only been harnessed for control of diastereoselectivity^[^ ^44^, ^45^, ^46^, ^47^ ^]^ or site‐selectivity,^[^ ^48^, ^49^, ^50^ ^]^ not enantioselectivity. We recently developed a family of ion‐paired Rh(II, II) complexes which are doubly anionic and paired with two cinchona alkaloid‐derived chiral cations. These catalysts were used in benzylic C─H amination and aziridination of substrates that bear a pendant primary alcohol, which we believe engages in hydrogen bonding with the sulfonate group on the ligand to provide organization at the transition state for nitrene transfer (Figure 1b, right, for aziridination).^[^ ^51^, ^52^, ^53^ ^]^ One of the constraints was the minimum chain length that could be tolerated between the reaction site (benzylic position for C─H amination or alkene for aziridination) and the alcohol directing group. In our first report on aziridination, we were able to achieve excellent results on a broad range of differentially substituted homoallylic alcohols, but enantioselectivity dropped to non‐useful levels with the shorter‐chained allylic alcohols (Figure 1b, left).^[^ ^52^ ^]^ Subsequently, we were able to tailor the design of the ligand to successfully accommodate certain allylic alcohols with high *ee*, a limitation being that the scope was restricted to trisubstituted, non‐styrenyl alkenes.^[^ ^53^ ^]^ Although this was a step forward, substantial scope for improvement remained. At the same time, we developed benzylic C─H amination using the same complexes and were able to achieve excellent results on hydrocinnamyl alcohols and those with longer chains between the hydroxyl and the arene. However, phenethyl alcohols gave extremely poor reactivity which we attribute to electronic deactivation of the benzylic position combined with the chain between the alcohol and the site of reaction being too short for effective catalyst direction (Figure 1c). This limitation was disappointing because benzylic C─H amination of phenethyl alcohols gives rise to β‐amino alcohols, which are important building blocks in a range of applications.^[^ ^54^, ^55^, ^56^ ^]^ Towards this goal, several enantioselective aminations of phenethyl alcohol derivatives have been disclosed, including using metal nitrene chemistry, but all operate in an intramolecular manner.^[^ ^57^, ^58^, ^59^, ^60^, ^61^, ^62^, ^63^ ^]^ Very recently, Zhou and co‐workers disclosed a rare intermolecular C─H amination of benzylic C─H bonds, including a protected phenethylalcohol, using chiral copper catalysts.^[^ ^64^ ^]^ Nevertheless, intermolecular, asymmetric amination of phenethylalcohol derivatives to give protected β‐amino alcohols remains extremely challenging.

In parallel studies, we discovered that tertiary amides were highly effective “directing groups” for enantioselective C─H amination using our ion‐paired catalysts (Figure 1d, previous work).^[^ ^65^ ^]^ This was initially surprising as tertiary amides are hydrogen bond acceptors rather than donors. Their effectiveness implied that a different type of interaction with the catalyst complex was in operation compared to the sulfonate‐alcohol hydrogen bonding interaction we had previously envisaged (Figure 1b). Based on control experiments using an *O*‐methylated cation we speculate that this could potentially occur through hydrogen bonding of the substrate amide with the free hydroxyl of the chiral cation and the α‐ammonium protons.^[^ ^65^ ^]^ We herein disclose that carbamate‐protected allylic alcohols constitute excellent substrates for asymmetric aziridination. Furthermore, the carbamate group can also be used to direct highly enantioselective benzylic C─H amination on phenethyl alcohol derivates to give β‐amino alcohols (Figure 1d, this work). In both substrates, the incorporation of a carbamate allows us to overcome limitations of our previous protocols whereby the shortest chain lengths of alcohols were incompatible.

## Results and Discussion

**Figure 2:**
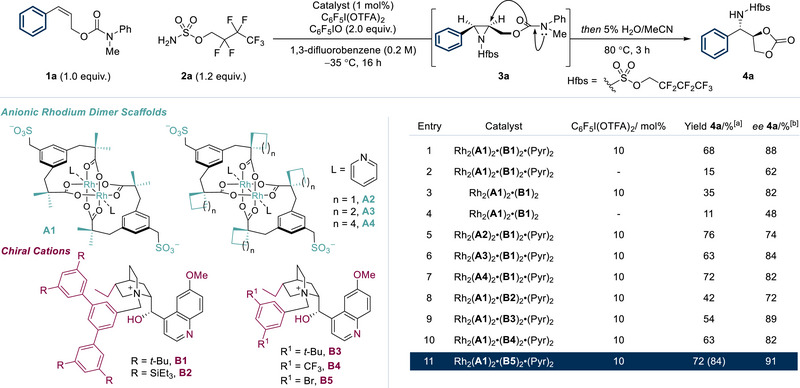
Optimization of the tandem aziridination/cyclization reaction of allylic carbamate **1a**. ^[a]^ Yields determined from crude ^1^H‐NMR with 1,2‐dimethoxyethane as an internal standard. Values in parentheses correspond to isolated values. ^[b]^ ee was determined by chiral SFC analysis.

Previous efforts at allylic alcohol aziridination using our ion‐paired catalysts had resulted in poor enantioselectivity on styrenyl substrates (45% *ee*, Figure 1b, lower left).^[^ ^52^ ^]^ We therefore selected the *cis* isomer of cinnamyl alcohol for evaluation and converted it to the corresponding carbamate **1a** with the intention that the carbonyl may engage in productive interactions with the chiral cation. In previous studies on the amide‐directed C─H amination, we had found that substoichiometric amounts of C_6_F_5_I(OTFA)_2_ as a weak acid additive were crucial for both high yield and enantioselectivity (Figure 2).^[^ ^65^ ^]^ The origin of this effect has not been definitively established, but we speculate that protonation of the basic quinoline nitrogen of the chiral cation may change the conformation of the cation leading to higher enantioselectivity, and separate mechanistic studies showed that removal of this basic nitrogen from the cation was detrimental to enantioselectivity in the alcohol‐directed nitrene transfer.^[^ ^53^ ^]^ Aziridination using 1 mol% of pyridine‐ligated Rh complex Rh_2_(**A1**)_2_•(**B1**)_2_•(Pyr)_2_ with 10 mol% C_6_F_5_I(OTFA)_2_ afforded the desired aziridine **3a** as observed by ^1^H‐NMR analysis of the crude reaction mixture. This was heated in aqueous acetonitrile to give 68% NMR yield of cyclic carbonate **4a** in 88% *ee* as a single diastereomer (Figure 2, entry 1). Whilst the aziridine **3a** is stable to isolation (see later), we found it most convenient for optimization purposes to analyse at the cyclic carbonate stage. Importantly, this two‐step aziridination/cyclization protocol constitutes a formal enantioselective, diastereoselective and regioselective oxyamination of the alkene, which remains as a challenging transformation.^[^ ^36^, ^66^, ^67^, ^68^, ^69^, ^70^, ^71^, ^72^, ^73^, ^74^, ^75^, ^76^, ^77^ ^]^ Omission of C_6_F_5_I(OTFA)_2_ was found to be detrimental to both yield and *ee*, in line with our previous observations (entry 2). As a control, we evaluated the reaction with the non‐pyridine‐ligated catalyst in the presence and absence of the C_6_F_5_I(OTFA)_2_ additive, but this also proved detrimental to reactivity and enantioselectivity (entries 3 and 4).^[^ ^78^ ^]^ We also evaluated the *trans* isomer of **1a**, which gave poor yield and *ee*, as well as an NH variant of **1a** lacking the *N*‐Me group, which completely shut down aziridination reactivity (see SI for details). The poor performance of the *trans*‐styrenyl substrate is consistent with our prior observations in alcohol‐directed aziridination and suggest that this geometry of styrenyl substrate results in a very poor fit in the chiral pocket provided by the chiral cation, relative to the *cis*‐isomers. We also evaluated a substrate in which the carbamate is derived from morpholine rather than *N*‐methylaniline but found that the yield of aziridination/cyclization was poor and amination on the morpholine, adjacent to nitrogen, predominated (see SI for details). We subsequently evaluated catalysts that had different geminal cyclic substituents on the anionic Rh(II, II) dimers (**A2**–**A4**), but in all cases the *ee* was unimproved (entries 5–7). Having identified the archetypal *gem*‐dimethyl complex **A1** as the optimal rhodium scaffold, we evaluated four further cations bearing different benzylating groups on the quinuclidine nitrogen (**B2**‐**B5**). This revealed that two (**B3** and **B5**) gave marginally improved enantioselectivities over **B1** (entries 8–11), and with **B5** the product could be isolated in high yield and 91% *ee* (entry 11). Neutral hydrolysis of **4a**, followed by treatment with base furnished the rearranged carbamate demethoxy‐4‐*epi*‐cytoxazone, from which the absolute configuration was assigned by comparison with the literature (see SI).^[^ ^79^ ^]^ Several different aminating agents were evaluated and were shown to be competent, including Tces, but the perfluorinated aminating agent indicated gave slightly higher selectivity (see SI for details).

**Scheme 1:**
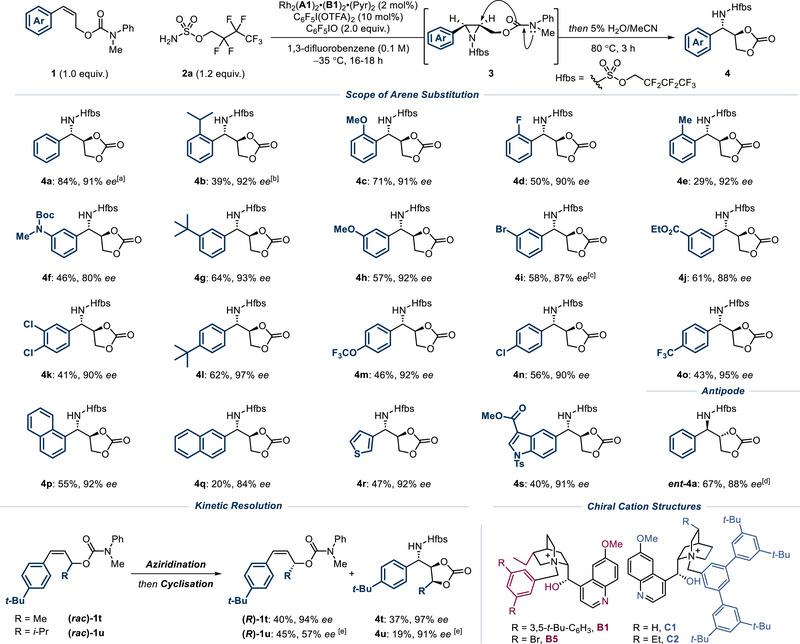
Scope for the enantioselective aziridination of cis‐styrenyl allylic carbamates **1**. Yields and ee values correspond to isolated values. ^[a]^ Reaction performed with catalyst Rh_2_(**A1**)_2_•(**B5**)_2_•(Pyr)_2_ (1 mol%). ^[b]^ Reaction performed at −25 °C. ^[c]^ Reaction performed with Rh_2_(**A1**)_2_•(**B1**)_2_•(Pyr)_2_ (1 mol%). ^[d]^ Reaction performed with Rh_2_(**A1**)_2_•(**C1**)_2_•(Pyr)_2_ (2 mol%). ^[e]^ Reaction performed with Rh_2_(**A1**)_2_•(**B1**)_2_•(Pyr)_2_ (3 mol%) and sulfamate ester **2a** (1.5 equiv.) at −10 °C.

Before evaluating the full reaction scope with respect to arene substitution, we carried out a preliminary survey of three substrates with three different catalysts bearing cations **B1**, **B3** and **B5**. The solubility properties of the **B5**‐containing complex were found to be poor at low temperatures, giving reproducibility issues. Over the three substrates tested, the **B1**‐containing complex proved to be reliable, with 2 mol% catalyst loading preferable (see SI for details). Therefore, the full scope of the reaction was explored using Rh_2_(**A1**)_2_•(**B1**)_2_•(Pyr)_2_ and we elected to cyclize the aziridine intermediates by post‐reaction heating to give carbonate products **4** as single diastereomers (Scheme 1). We were pleased to find that a variety of *ortho*‐ substituents were accommodated, including isopropyl (**4b**), methoxy (**4c**), fluoro (**4d**) and methyl (**4e**). *Meta*‐substituents included a Boc‐protected amine (**4f**), several electron donating groups (**4** **g**, **4** **h**), a bromide (**4i**) and an ester (**4j**). Similar tolerance was also observed at the *para* position and this encompassed dichloro (**4k**), *tert*‐butyl (**4l**), trifluoromethoxy (**4m**), chloro (**4n**) and trifluoromethyl (**4o**) substituents. Isomeric naphthyl groups (**4p**, **4q**) gave high *ee* although the 1‐naphthyl isomer gave a low yield (**4q**). We demonstrated the effectiveness of several heterocycles including a thiophene (**4r**) and an indole (**4s**), and showed that the antipode (*ent*‐**4a**) could be readily obtained using the complex containing diastereomeric cation **C1**, which is derived from quinine following removal of the vinyl group.^[^ ^52^ ^]^ We also investigated the feasibility of operating the reaction in kinetic resolution mode and tested two racemic substrates (**1t** and **1u**), each bearing an alkyl substituent on the allylic carbon. With methyl‐substituted **1t**, the kinetic resolution worked successfully to give aminated product **4t** in 36% yield and 97% *ee*. The recovered starting material (*R*)‐**1t** was isolated in 40% yield and 94% *ee*. With isopropyl‐substituted **1u**, the reaction appeared slower, presumably due to the greater steric hindrance around the alkene, such that the product **4u** was obtained in a lower yield but with excellent 91% *ee*.

**Scheme 2:**
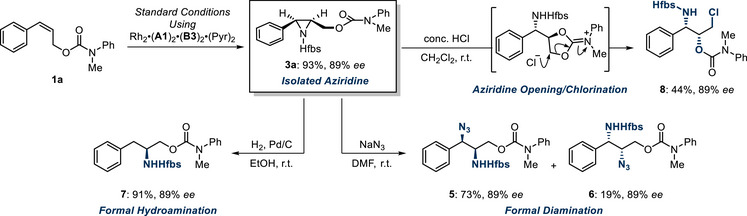
Isolation and synthetic elaboration of aziridine **3a**.

We demonstrate that the aziridine intermediate is isolable, if desired (Scheme 2). In this case (using the complex containing cation **B3**), the isolated yield of the aziridine was significantly higher than the NMR yield obtained after cyclization (Figure 2, entry 9), suggesting that some material loss may occur during cyclization (the *ee* value was consistent). This particular aziridine was sufficiently stable for full characterization. The aziridine intermediates leading to *para*‐chloro‐substituted **4n** and *para*‐CF_3_‐substituted **4o** could also be isolated with ee values matching those of cyclized products **4n** and **4o**. Whilst crude NMR yields were between 60% and 70% the isolated yields were lower in these cases, which we believe is due to partial decomposition on silica (see SI). These aziridines also proved to be unstable on storage and would be best used in immediate further derivatization (see later) or potentially telescoped from the crude reaction mixture. We also attempted to isolate the aziridine leading to thiophene‐containing **4r**, which was present in the crude reaction mixture, but found this was insufficiently stable on silica. To demonstrate the breadth of possible further transformations, we intercepted **3a** in a variety of other processes (Scheme 2). Ring opening with azide afforded the diaminated products **5** and **6** with complete enantiospecificity, bearing differentially protected amines, allowing for possible orthogonal deprotection/functionalization. Furthermore, subjecting the aziridine to hydrogenation conditions enabled a formal hydroamination process of the alkene to be achieved (**7**). Treatment of the aziridine with HCl furnished the highly functionalized carbamate **8**, which is proposed to arise from aziridine ring‐opening with the pendant carbamate, followed by chloride attack of the resulting cationic intermediate. Specifically, transformation into **8** significantly increased the functional and stereochemical complexity from the starting material **1a**. Together, this selection of transformations illustrates the versatility of the aziridine product functionalized with a pendant carbamate.

**Scheme 3:**
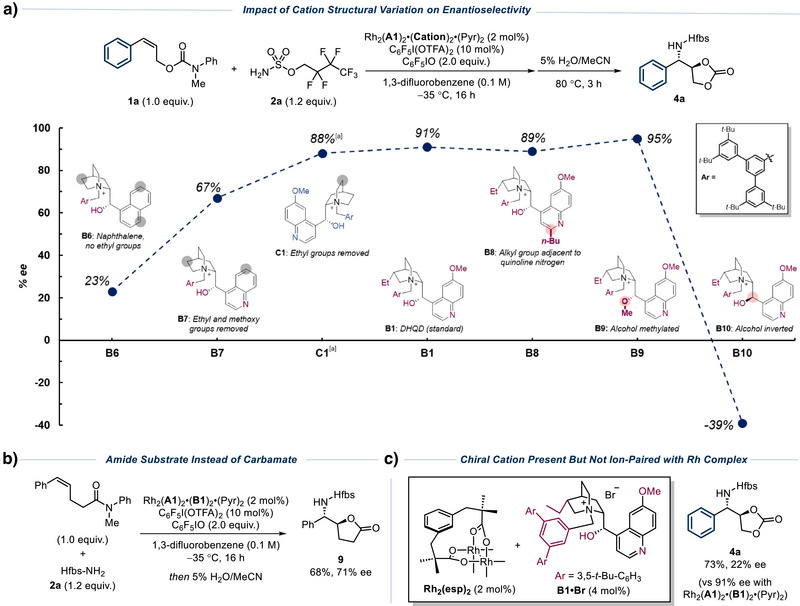
Investigation of impact of cation structure on selectivity, assessment of amide directing group and disconnection of cation and Rh complex. ^[a]^ Antipode of **4a** obtained due to **C1** having the opposite aminoalcohol configuration compared with the others.

We next carried out experiments to explore the impact of structural features of the cinchona alkaloid‐derived cation by knocking them out in a systematic manner, an approach we previously applied to the alcohol‐directed C─H amination and aziridination (Scheme 3a).^[^ ^53^ ^]^ Cation **B6** is a simplified version of the optimal cation, which lacks the quinuclidine ethyl group, the quinoline methoxy group and in which the quinoline nitrogen has been replaced with a methine unit to form a naphthalene. The complex containing **B6** gave extremely poor *ee* in the aziridination/cyclization at only 23%. Systematically restoring the chiral cation features proved fascinating: addition of the quinoline nitrogen (cation **B7**) boosted *ee* significantly to 67%, its importance in line with our prior observations in the alcohol‐directed system. Returning the methoxy to the quinoline gave another increase, allowing 88% *ee* to be obtained using cation **C1** (the antipode of **4a** was produced in this case due to **C1** having the opposite aminoalcohol configuration to the other cations). Return of the quinuclidine ethyl group in cation **B1** adds only a 3% *ee*, indicating minimal importance. Given the evident importance of the quinoline nitrogen, we evaluated cation **B8,** which possesses an *n*‐butyl group at the quinoline C2 position. If binding of the quinoline nitrogen to the Rh catalyst was important then this feature would be expected to impact binding and potentially also *ee*. However, **B8** gave practically identical results to the optimal **B1**, suggesting that the quinoline nitrogen is not operating in this way. We next evaluated cation **B9** in which the free hydroxyl of the alkaloid is *O*‐methylated. Complexes containing this cation had given very poor outcomes in our previous studies when evaluated on the alcohol directed C─H amination and aziridination^[^ ^53^ ^]^ as well as the amide‐directed C─H amination, implying a crucial role of this feature in organization at the transition state in those cases.^[^ ^65^ ^]^ Remarkably, this cation proved to be just as effective as **B1** here and actually increased *ee* to 95% (compared with 91% for **B1**), favouring the same major enantiomer of cyclized product **4a**. This is a striking divergence compared with our previous observations and makes it clear that hydrogen bonding of the catalyst hydroxyl to the carbamate carbonyl cannot be an important factor in the high selectivity obtained. Using epimeric cation **B10**, which possesses a stereochemically inverted secondary alcohol, was detrimental, giving the opposite enantiomer of **4a** in a relatively low −39% *ee*. Overall, as the quinoline nitrogen proves crucial, we suspect that a change in conformation of the cation could be occurring upon protonation in the presence of the acid‐releasing additive C_6_F_5_I(OTFA)_2_, as we have previously suggested in other systems.^[^ ^53^, ^65^ ^]^ Evaluating the *O*‐methylated cation in the absence of the C_6_F_5_I(OTFA)_2_ additive also gave a reduced *ee* (50% vs. 95%), indicating a consistent trend. Another possibility we considered is that the trifluoroacetic acid released from the additive engages in hydrogen bonding with the substrate carbamate group, resulting in a buildup of positive charge on the latter and enabling it to engage in electrostatic interactions with the catalyst sulfonate group. In such a scenario, an amide instead of a carbamate might be expected to be superior since an amide should be a stronger hydrogen bond acceptor.^[^ ^80^ ^]^ However, evaluation of the directly analogous amide substrate showed this not to be the case as *ee* was reduced to 71% (**9**, vs. 91% for carbamate, Scheme 3b). At present, the exact nature of the interaction of the carbamate group with the catalyst in this complex multicomponent system remains elusive. Additionally, we carried out a control experiment where we used Rh_2_(esp)_2_ (2 mol%) as the catalyst under the optimized conditions with the optimal chiral cation **B1** (4 mol%) added as its bromide salt. This resulted in only 22% *ee*, demonstrating the crucial importance of the attractive ion pairing interaction between the two components (Scheme 3c).

**Scheme 4:**
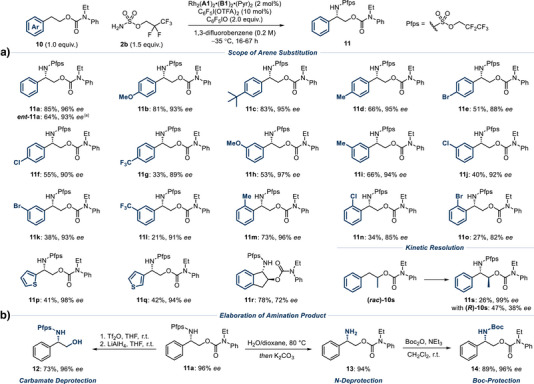
Scope for the enantioselective C─H amination of carbamate‐protected phenethyl alcohols **10**. Yields and *ee* values correspond to isolated values. ^[a]^ Reaction performed with catalyst Rh_2_(**A1**)_2_•(**C2**)_2_•(Pyr)_2_ (2 mol%).

Given the effectiveness of the carbamate group in directing aziridination, we speculated whether it might also be a proficient directing group for Rh‐catalyzed C─H amination.^[^ ^12^, ^57^, ^60^, ^81^, ^82^, ^83^, ^84^, ^85^, ^86^, ^87^, ^88^, ^89^, ^90^, ^91^, ^92^, ^93^, ^94^ ^]^ As described above, previous attempts using our ion‐paired catalysts with simple phenethyl alcohol resulted in trace reactivity (Figure 1c). We hoped that by transforming the alcohol into a carbamate, productive interaction with our ion‐paired catalyst may be restored and that the electronic challenges of the C─H insertion step may be overcome. This was evaluated on substrate **10a**, and after brief optimization, we were very pleased to obtain high yield and *ee* (96%) of the desired amination product **11a** (Scheme 4a). One optimization point to note was to use an *N*‐ethyl carbamate, instead of *N*‐methyl due to a minor by‐product (∼10%) resulting from amination of the *N*‐alkyl group of the carbamate.^[^ ^93^, ^95^ ^]^ This proved particularly troublesome in the case of the *N*‐methyl substrate as it co‐eluted with the desired product; in contrast, switching to *N*‐ethyl facilitated separation. Additionally, switching the aminating agent to one possessing a less fluorinated chain (**2b,** Pfps‐NH_2_) gave slightly higher yields. We also evaluated a morpholine‐containing carbamate but here C─H amination occurred exclusively on the morpholine portion, adjacent to nitrogen (see SI for full details). Similar to the aziridination, the antipode (*ent*‐**11a**) could also be obtained using the complex containing diastereomeric cation **C2**. At this stage we also evaluated the complex containing the *O*‐methylated cation **B9,** which had given superior *ee* compared with **B1** in the aziridination (Scheme 3a). Here it gave **11a** in 49% isolated yield and 94% *ee*—only slightly lower than **B1**, further demonstrating that in these carbamate‐containing substrates the free hydroxyl of the alkaloid is not playing an important organizational role. We proceeded to evaluate the enantioselective C─H amination of various carbamate‐protected phenethyl alcohols. Halogens, donating and withdrawing groups were successfully incorporated at *para* (**11b**‐**11g**), *meta* (**11h**‐**11l**) and *ortho* (**11m**‐**11o**) positions of the phenyl ring. It is noticeable that when the electron density on the aromatic ring is reduced, reactivity is also reduced, thus impacting yield (e.g., **11g**, **11l**). Although this was anticipated, we nevertheless found it remarkable that product was still obtained in these cases, given the proximity of the electron‐withdrawing carbamate group to the C─H bond undergoing insertion. To illustrate the profound effect on chemoselectivity of our ion‐paired catalysts, we struggled to get any of the desired racemic amination products for many substrates using Rh_2_(esp)_2_, which gave *N*‐ethyl amination as the major product in all cases. We were able to incorporate both regioisomers of thiophene into the substrate, giving excellent *ee* outcomes in products **11p** and **11q**. The carbamate derived from indan‐2‐ol also gave the corresponding product **11r** in good yield as a single diastereomer. In this case, the *ee* was reduced to 72%, potentially due to the restricted conformational freedom of the substrate, which may hinder optimal interactions with the chiral cation at the transition state for C─H amination. As in the aziridination, a kinetic resolution substrate bearing a methyl group adjacent to oxygen performed very well in terms of selectivity, delivering 99% *ee* for **11s** and highlighting the potential of this chemistry for accessing elaborately substituted chiral β‐amino alcohols. Finally, we demonstrate that both functional groups on the reaction product **11a** could be readily and independently manipulated through either removal of the carbamate to give **12** ^[^ ^64^ ^]^ or removal of the Pfps group^[^ ^93^ ^]^ to give **13** (Scheme 4b). The enantiopurity of **13** was preserved following analysis of the Boc‐protected derivative **14**. The absolute configuration was determined by comparing **12** with an independently synthesized sample. Given the excellent reactivity that the carbamate directing group conferred in these substrates, we also evaluated a substrate bearing a linear aliphatic chain to determine whether amination on non‐activated aliphatic C─H bonds may be achievable using our catalyst. Unfortunately, no amination on the chain was observed, with amination only occurring on the methyl group of the amide. This reflects the much lower reactivity of these non‐activated C─H bonds with the rhodium nitrenoid (see SI for details).

**Scheme 5:**
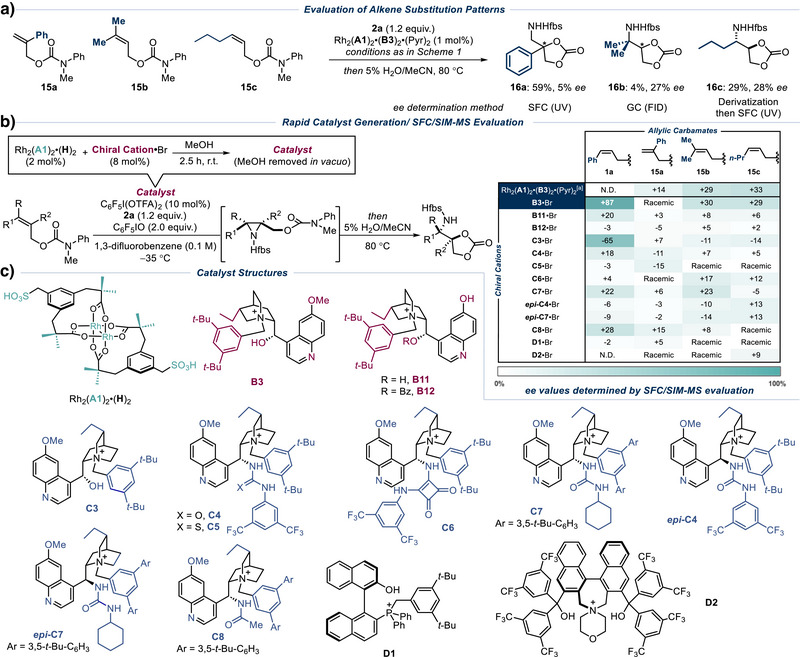
Evaluation of alkene substitution patterns using rapid formation without isolation of diverse ion‐paired catalysts and analysis using SIM‐MS (selected ion monitoring – mass spectrometry). ^[a]^ Reaction performed with preformed ion‐paired catalyst Rh_2_(**A1**)_2_•(**B3**)_2_•(Pyr)_2_ (1 mol%).

Returning to aziridination, since *cis*‐styrenyl allylic carbamates proved to be an effective class of alkenes, we aimed to explore the compatibility of various alkene substitution patterns with our reaction system. As an initial exploration, we tested one of the optimal catalysts on *exo*‐styrene **15a**, trisubstituted prenol‐derived **15b** and *cis* non‐styrenyl **15c** (Scheme 5a). Unfortunately, the *ee* outcomes were poor and we resolved to examine more structurally diverse chiral cations, in the hope that a more fundamental change to the structure might provide a solution. Accordingly, we assembled a collection of cations (Scheme 5c) that included two cupreidine‐derived examples (**B11** and **B12** with the methoxy group on the quinoline demethylated); variants where the hydroxyl group of the alkaloid is replaced with alternative hydrogen bond donors such as urea (**C4**, **C7**), thiourea (**C5**), squaramide (**C6**) or amide (**C8**); and urea derivatives with inverted stereochemistry at C9 of the alkaloid (*epi*‐**C4** and *epi*‐**C7**). Finally, BINOL‐derived phosphonium (**D1**)^[^ ^96^ ^]^ and ammonium (**D2**)^[^ ^97^ ^]^ cations developed by Maruoka were included to provide non‐cinchona examples. One of the potential advantages of using ion‐paired catalysts is that one can imagine a combinatorial approach to catalyst screening if they can be reliably formed in situ without tedious isolation. If viable, this would allow rapid exploration of a wider range of ion‐paired catalysts than would realistically be possible if the catalysts all had to be prepared and isolated discretely. In important prior work, Ooi and co‐workers demonstrated the in situ pairing of achiral ammonium phosphines with chiral phosphate anions to identify the optimal catalyst for the palladium‐catalyzed asymmetric allylic alkylation of benzo[*b*]thiophen‐2(3*H*)‐ones.^[^ ^98^ ^]^ We sought to emulate aspects of that study, as well as incorporate mass spectrometry as a means to determine *ee* values from crude reaction mixtures using selected ion‐monitoring (SIM). This novel enantioselectivity assay method was recently shown in reports by Jacobsen, Kwan and co‐workers,^[^ ^99^ ^]^ as well as Watson, Kelly, Sampson and collaborators,^[^ ^100^ ^]^ to be reliable for high‐throughput asymmetric catalysis campaigns. We were keen to explore this assay method in order to make *ee* analysis of the crude reaction mixtures potentially more reliable and also to overcome the issue of a lack of UV chromophores in **16b** and **16c** (two different analytical methods were used to determine *ee* for **16a**‐**16c**, see Scheme 4a). In particular, the enantiomers of **16c** did not separate using GC‐FID conditions and necessitated further derivatization to install a chromophore for *ee* determination by SFC. After considering factors highlighted in the previous reports that can affect accuracy, we were able to use SIM‐MS analysis to obtain *ee* values for **16a**‐**16c** that were broadly in line with those obtained through conventional means (Scheme 5b, inset table, top row). Continuing with the SIM‐MS analysis, after some experimentation we developed a protocol for rapid formation of the ion‐paired catalysts, which gave similar *ee* outcomes for original optimization substrate **1a** and new substrates **15a**‐**15c** when compared with the preformed complex. In this protocol the Rh dimer is protonated on Amberlite IRC120 acidic resin to give Rh_2_(**A1**)_2_•(**H**)_2_, which is stirred with four relative equivalents of the bromide salt of the chiral cation in methanol for 2.5 h (Scheme 4b, above reaction arrow). After removal of the solvent under vacuum, the remaining reaction components were added as normal. The *ee* outcomes for all four substrates were very similar to that using the preformed complex, providing satisfactory validation of the procedure (Scheme 5b, inset table, second row). Prior to this, we attempted true in situ generation of the catalysts in 1,3‐DFB prior to the addition of the rest of the reagents, but found this to be unreliable, suggesting that a pre‐stir step in methanol was required to enable formation, prior to solvent exchange. With confidence in both the ion pair formation and the SIM‐MS analysis, we proceeded to evaluate all the collected cations against the three new substrates (**15a**‐**15c**) as well as **1a** using this protocol. The results were disappointing as none of the cations was able to provide high *ee* on the new substrates. For **1a,** moving away from the natural structure of the alkaloid was clearly detrimental. The only significant *ee* value obtained was with the pseudoenantiomeric **C3**, which is derived from dihydroquinine. This gave the opposite enantiomer of **4a** but in lower *ee* than with **B3**, indicating that **C3** is a less effective pseudoenantiomer for **B3**. This “uneven efficiency” had been previously observed using preformed ion‐paired catalysts (thus prompting us to use the desvinylquinine‐derived cation to access the product antipode) and serves as a useful marker to give confidence in the protocol used for both the catalyst formation protocol and SIM‐MS analysis. Despite the lack of positive results obtained on the broader substrates, we believe that this demonstration of the rapid ion‐pairing protocol combined with SFC‐SIM‐MS *ee* analysis adds value for those looking to potentially exploit this or related methodology for high‐throughput optimization in asymmetric catalysis.

## Conclusion

We have demonstrated the highly enantioselective aziridination of carbamate‐protected *cis*‐styrenyl allylic alcohols. The aziridine intermediate is amenable to a host of valuable transformations. We have further shown that appending a very similar carbamate group onto phenethyl alcohols enables benzylic C─H amination to occur to form highly enantioenriched β‐amino alcohol derivatives. Both transformations had not been feasible using alcohol directing groups and the scope of enantioselective nitrene transfer using our ion‐paired Rh catalysts has been significantly broadened with these findings. We also demonstrate that our ion‐paired catalysts can be formed without isolation of the discrete catalyst from individual salts and that *ee* can be analysed using mass response in selected ion‐monitoring. These findings lay a foundation for high throughput experimentation to be applied to catalyst discovery and optimization.

## Conflict of Interests

The authors declare no conflict of interest.

## Supporting information



Supporting Information

## Data Availability

The data that support the findings of this study are available in the Supporting Information of this article.
