# Supplementary material for: Asymmetric Aziridination of Allylic Carbamates Using Ion‐Paired Rhodium Complexes and Extrapolation to C─H Amination of Phenethyl Carbamates
Source: Angew Chem Int Ed Engl. 2025 May 29;64(29):e202507532. doi: 10.1002/anie.202507532 (PMC12258697; doi:10.1002/anie.202507532)

Supporting Information for

Asymmetric Aziridination of Allylic Carbamates using Ion-Paired Rhodium Complexes and Extrapolation to C-H Amination of Phenethyl Carbamates

Arthur R. Lit,† Shotaro Takano,† Christian Zachau, Ioana Băltărețu and Robert J. Phipps*

Yusuf Hamied Department of Chemistry, University of Cambridge, Lensfield Road Cambridge, CB2 1EW, United Kingdom

†These authors contributed equally to this work.

Corresponding Author Email: rjp71@cam.ac.uk

Table of Contents

1 General Information **S2**

2 Optimisation Studies for Aziridination **S4**

3 Synthesis of Achiral Rhodium (II,II) Tetracarboxylate Dimer **S11**

4 Synthesis and Characterisation of Chiral Cations **S11**

5 Synthesis and Characterisation of Chiral Rh(II,II) Tetracarboxylate Dimers **S44**

6 Synthesis of Reagents for Rhodium-Catalysed Nitrene Transfer **S49**

7 Synthesis and Characterisation of Substrates (Aziridination) **S50**

8 Characterisation of Enantioenriched Products from Aziridination **S99**

9 Isolation and Synthetic Elaboration of Aziridines **S123**

10 Investigation of Impact of Chiral Cation Structure on Selectivity **S130**

11 Additional Optimization Data of C-H Amination **S131**

12 Unsuccessful C-H Amination Substrates **S132**

13 Synthesis and Characterisation of Substrates (C-H Amination) **S136**

14 Characterisation of Enantioenriched Products from C-H Amination **S156**

15 Synthetic Elaboration of C-H Amination Product **S185**

16 Determination of Absolute Stereochemistry **S188**

17 Rapid Catalyst Generation with SFC-MS Analysis **S195**

18 Chiral SFC, HPLC and GC Traces **S203**

19 References **S266**

20 NMR Spectra **S268**

1 General Information

Reaction setup, solvents and reagents: All reactions were carried out under an inert argon or nitrogen atmosphere using standard Schlenk-septa techniques in flame-dried glassware unless otherwise stated. Reactions performed in 4.0 mL crimp-top vials that required cooling were placed in a Polar Bear Cub (by Cambridge Reactor Design) featuring a deep-welled heating block (IKA DB 5.2). CuI was purified according to the protocol reported by Kauffman and Fang.[1] NaOH was freshly ground using a pestle and mortar. All other reagents, unless otherwise stated, were used as supplied from commercial sources without further purification. CH_2Cl_2, THF, Et_2O, MeCN, MeOH, toluene, and hexane were purified by distillation on site under inert atmosphere *via* the following processes. THF and Et_2O were pre-dried over sodium wire then distilled from calcium hydride and $LiAlH_4$. CH_2Cl_2, MeCN, MeOH, toluene and hexane were distilled from calcium hydride. All other solvents were used as supplied by commercial sources without further purification.

Chromatography: Analytical thin-layer chromatography was performed using precoated Merck glass backed silica gel plates (Silica gel 60 F254). Visualisation was by ultraviolet fluorescence (λ = 254 or 365 nm) and/ or staining with potassium permanganate ($KMnO_4$) or Ceric Ammonium Molybdate (CAM). Flash column chromatography was performed using silica gel 60 (pore size: 60 Å, mesh: 40-63 µm) from Material Harvest® or Fluorochem.

NMR Spectroscopy: ^{1}H NMR spectra were recorded on 700 MHz TXO Cryoprobe, 600 MHz Bruker Avance DRX-600, 500 MHz Bruker DCH Cryoprobe, 400 MHz Bruker DPX-400 Dual, 400 MHz Avance III HD or 400 MHz Avance III HD Smart Probe spectrometers. Chemical shifts are reported in parts per million (ppm) and the spectra are calibrated to the resonance resulting from incomplete deuteration of the solvent ($CDCl_3$: 7.26 ppm; CD_3CN: 1.94 ppm, p; $(CD_3)_2CO$: 2.05 ppm, p; D_2O: 4.79 ppm; $(CD_3)_2SO$: 2.50 ppm, p; CD_3OD: 3.31 ppm, p; C_5D_5N (H–C–N): 8.74 ppm.[2] ^{13}C NMR spectra were recorded on the same spectrometers with complete proton decoupling. ^{13}C NMR experiments referenced as such were performed using a UDEFT sequence to increase the signal:noise ratio for ^{13}C signals of carbon nuclei along poly-fluorinated chains.[3] Chemical shifts are reported in ppm with the solvent resonance as the internal standard ($^{13}CDCl_3$: 77.16 ppm, t; $^{13}CD_3CN$: 1.32 ppm, sept; $(^{13}CD_3)_2CO$: 29.84 ppm, sept; $(^{13}CD_3)_2SO$: 39.52 ppm, sept; $^{13}CD_3OD$: 49.00 ppm, sept; $^{13}C_5D_5N$ (C–N): 150.35 ppm, t.)[2] ^{19}F NMR spectra were recorded on 500 MHz Bruker DCH Cryoprobe, 400 MHz Avance III HD or 400 MHz Avance III HD Smart Probe spectrometers. Chemical shifts are reported in ppm with $CFCl_3$ as the external standard ($CFCl_3$: 0.00 ppm). Data are reported as follows: chemical shift δ, multiplicity (s = singlet, d = doublet, t = triplet, q = quartet, p = pentet, sext = sextet, hept = heptet, br = broad, m = multiplet, or combinations thereof (^{13}C and all

other nuclides except ^{1}H are singlets unless otherwise stated)), coupling constants *J*, number of nuclides (signals for all other nuclides except ^{1}H refer to one nuclide unless otherwise stated), assignment. ^{1}H NMR spectra are assigned as fully as possible, using ^{1}H-^{1}H COSY, ^{1}H-^{1}H NOESY, DEPT-135, ^{1}H-^{13}C HSQC and ^{1}H-^{13}C HMBC where appropriate to facilitate structural determination. Assignments follow the numbering system shown on the structures. ^{1}H NMR signals are reported in ppm to 2 decimal places and all other nuclide signals to 1 decimal place. Coupling constants are reported in Hz to a maximum of 3 significant figures. For cinchona alkaloid-derived compounds the appearance and chemical shifts of the peaks in the NMR spectra can vary significantly depending on sample concentration and other factors. For spectra acquired in C_5D_5N the residual water peak is often visible at approximately 4.9 ppm in the ^{1}H NMR spectrum.

High Resolution Mass Spectrometry (HRMS): Recorded on a Waters Micromass LCT Premier, a Waters Xevo G2-S or a Waters Vion IMS Qtof at the Department of Chemistry at the University of Cambridge. The ionisation method is noted as positive or negative electrospray ionisation (ESI+ or ESI–). Measured values are reported to 4 decimal places and are within ± 5 ppm of the calculated value. The calculated values are based on the most abundant isotope unless otherwise stated in the chemical formula. For ions bearing more than a single unit of charge, the masses reported are the mass/charge ratios.

Chiral SFC Analysis: Performed on a Waters ACQUITY UPC2 System with DAICEL CHIRALPAK IC, IG, IJ or IK columns (4.6 x 250 mm, 3.0 μm) in a mixed solvent system of supercritical CO_2 and MeOH. A system backpressure of 138 bar was used in all cases.

Chiral GC Analysis: Performed on a Shimadzu GC-2010 Plus instrument equipped with a CP-Chirasil-Dex CB column (25 m x 0.25 mm ID x 0.25 μm film) and an FID detector, and an Agilent 6890N Flame Ionisation Detector Gas Chromatograph System equipped with a CP7502-CP-Chirasil-DEX CB column (25 m x 0.25 mm x 0.25 μm).

Optical Rotations*:* Measured in spectrophotometric grade $CHCl_3$, MeOH, or C_5H_5N on a Perkin Elmer 343 Polarimeter using a sodium lamp (λ = 589 nm, D-line). $[\alpha]_D$ values are reported at the stated temperature, with concentration in g/100 mL.

Naming and Numbering of compounds: Systematic names were generated by the computer program ChemDraw according to the guidelines specified by the IUPAC. However, the numbering on the structures does not correspond to the systematic name.

2 Optimisation Studies for Aziridination

Table S1: Enantioselective aziridination of allylic carbamate **1a** with discretely ion-paired catalysts.

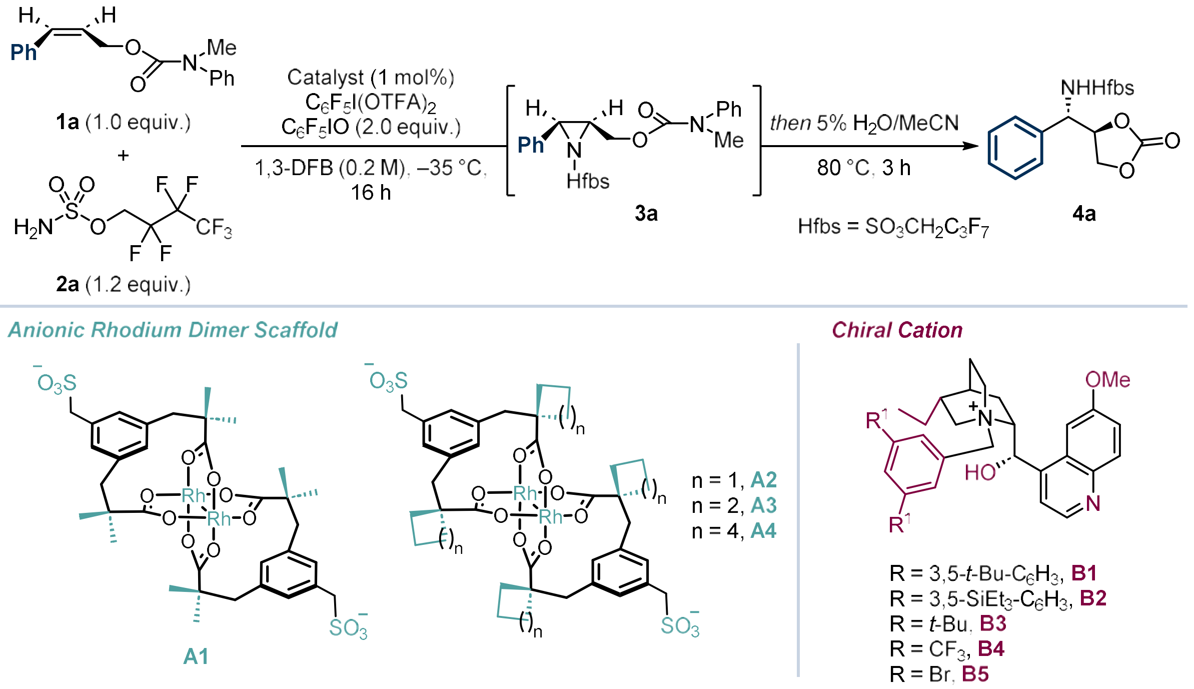

Entry	Catalyst	$C_6F_5I(OTFA)_2$/ mol%	Yield **4a**/%[a]	*ee* **4a**/ %[b]	Remarks
1	Rh_2(**A1**)$_2$•(**B1**)$_2$•(Pyr)$_2$	10	68	88	***Control studies***
2	Rh_2(**A1**)$_2$•(**B1**)$_2$•(Pyr)$_2$	-	15	62	
3	Rh_2(**A1**)$_2$•(**B1**)$_2$	10	35	82	
4	Rh_2(**A1**)$_2$•(**B1**)$_2$	-	11	48	
5	Rh_2(**A2**)$_2$•(**B1**)$_2$•(Pyr)$_2$	10	76	74	***Evaluation of anionic rhodium dimer scaffold***
6	Rh_2(**A3**)$_2$•(**B1**)$_2$•(Pyr)$_2$	10	63	84	
7	Rh_2(**A4**)$_2$•(**B1**)$_2$•(Pyr)$_2$	10	72	82	
8	Rh_2(**A1**)$_2$•(**B2**)$_2$•(Pyr)$_2$	10	42	72	***Evaluation of chiral cations***
9	Rh_2(**A1**)$_2$•(**B3**)$_2$•(Pyr)$_2$	10	54	89	
10	Rh_2(**A1**)$_2$•(**B4**)$_2$•(Pyr)$_2$	10	63	82	
11	Rh_2(**A1**)$_2$•(**B5**)$_2$•(Pyr)$_2$	10	72 (83)	91	

Reactions performed on 0.1 mmol scale with respect to allylic carbamate **1a**. [a] Yields determined from crude ^{1}H NMR analysis with 1,2-dimethoxyethane as an internal standard. Values in parentheses correspond to isolate values. [b] *ee* was determined by chiral SFC-PDA analysis.

Table S2: Catalyst reproducibility issues encountered in the enantioselective aziridination of allylic carbamate **1a**

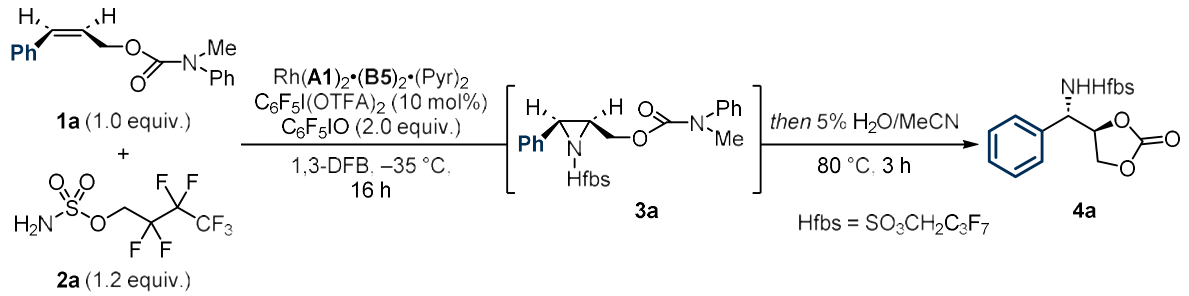

Entry	Rh_2(**A1**)$_2$•(**B5**)$_2$•(Pyr)$_2$/ mol%	Conc./ M	Yield **4a**/%[a]	*ee* **4a**/ %[b]	Remarks
1	1	0.2	72 (83)	91	1st batch of catalyst
2	1	0.2	42	86	2nd batch of catalyst
3	2	0.2	53	91	2nd batch of catalyst
4	1	0.1	75 (64)	91	2nd batch of catalyst
5	1	0.1	57	90	3rd batch of catalyst

Reactions performed on 0.1 mmol scale with respect to allylic carbamate **1a**. [a] Yields determined from crude ^1H NMR analysis with 1,2-dimethoxyethane as an internal standard. Values in parentheses correspond to isolate values. [b] *ee* was determined by chiral SFC-PDA analysis.

Despite having identified an effective catalytic system, a subsequent repeat with a different batch of Rh_2(**A1**)$_2$•(**B5**)$_2$•(Pyr)$_2$ revealed a reproducibility issue that saw a drop in enantioselectivity (**Table S2**, Entries 1-2). We attribute this reproducibility issue to the poor solubility properties of the **B5**-containing complex at low temperatures. We attempted to rectify the reproducibility issue by first increasing the catalyst loading to 2 mol% with the problematic catalyst batch (**Table S2**, Entry 3). In this case, the enantioselectivity was reinstated to 91% *ee* with a slight increase in product conversion. We also observed that by diluting the reaction conditions whilst keeping the catalyst loading at 1 mol%, the problematic catalyst batch was able to yield the desired product in 64% isolated yield and 91% *ee* (**Table S2**, Entry 4). We repeated these conditions with a third batch of catalyst and were pleased to see that the enantioselectivity was maintained despite a drop in reactivity (**Table S2**, Entry 5).

Table S3: Optimisation studies for the enantioselective aziridination of allylic carbamates **1c**, **1i** and **1j**.

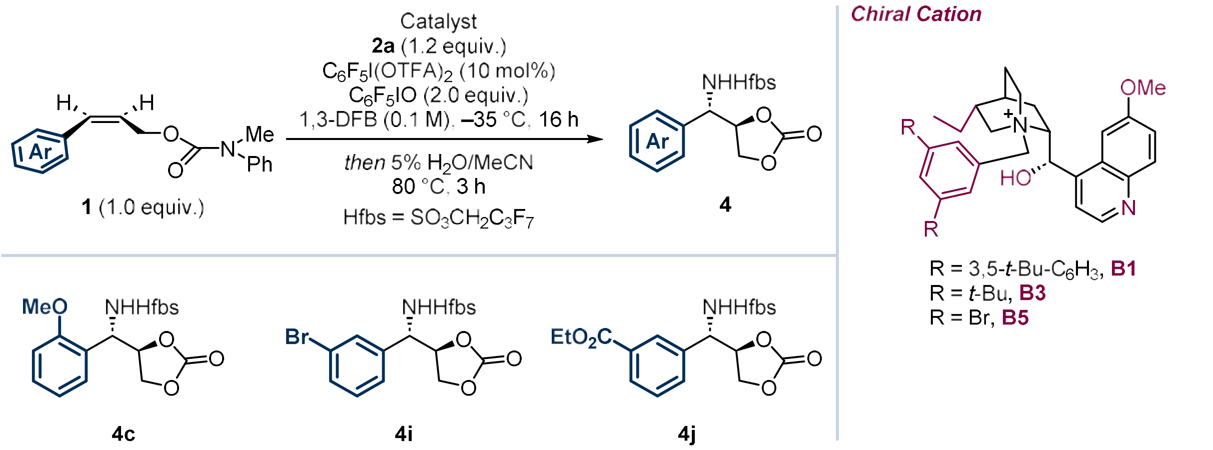

Entry	Product	Catalyst/ mol%	Yield **4**/ %[a]	*ee* **4**/ %[b]
1	**4i**	Rh_2(**A1**)$_2$•(**B1**)$_2$•(Pyr)$_2$/ 1	62 (58)	87
2	**4i**	Rh_2(**A1**)$_2$•(**B3**)$_2$•(Pyr)$_2$/ 1	56 (48)	81
3	**4i**	Rh_2(**A1**)$_2$•(**B5**)$_2$•(Pyr)$_2$/ 1	56 (53)	88
4	**4j**	Rh_2(**A1**)$_2$•(**B1**)$_2$•(Pyr)$_2$/ 2	63 (61)	88
5	**4j**	Rh_2(**A1**)$_2$•(**B3**)$_2$•(Pyr)$_2$/ 1	59 (46)	82
6	**4j**	Rh_2(**A1**)$_2$•(**B5**)$_2$•(Pyr)$_2$/ 2	66 (56)	86
7	**4c**	Rh_2(**A1**)$_2$•(**B1**)$_2$•(Pyr)$_2$/ 2	69 (71)	91
8	**4c**	Rh_2(**A1**)$_2$•(**B5**)$_2$•(Pyr)$_2$/ 2	62 (67)	87

Reactions performed on 0.1 mmol scale with respect to allylic carbamate **1c**, **1i** and **1j**. [a] Yields determined from crude ^1H NMR analysis with 1,2-dimethoxyethane as an internal standard. Values in parentheses correspond to isolate values. [b] *ee* was determined by chiral SFC-PDA analysis.

Before embarking on a substrate scope evaluation, we carried out a short optimisation to determine whether the optimal catalyst Rh_2(**A1**)$_2$•(**B5**)$_2$•(Pyr)$_2$ identified with allylic carbamate **1a** was also effective for other *cis*-styrenyl allylic carbamates (**Table S3**). With the *meta*-Br substrate **1i**, Rh_2(**A1**)$_2$•(**B5**)$_2$•(Pyr)$_2$ gave a high enantioselectivity of 88% *ee* (**Table S3**, Entry 3). The archetypal catalyst Rh_2(**A1**)$_2$•(**B1**)$_2$•(Pyr)$_2$ was also identified to be an effective catalyst for substrate **1i**, giving the product **4i** in 58% isolated yield and 87% *ee* (**Table S3**, Entry 1). With *meta*-ester **1j**, Rh_2(**A1**)$_2$•(**B5**)$_2$•(Pyr)$_2$ afforded the product **4j** in 86% *ee* and this could be improved to 88% *ee* with Rh_2(**A1**)$_2$•(**B1**)$_2$•(Pyr)$_2$ (**Table S3**, Entry 6 vs Entry 4). Finally, we investigated the enantioselective aziridination of substrate **1c** with Rh_2(**A1**)$_2$•(**B1**)$_2$•(Pyr)$_2$ and Rh_2(**A1**)$_2$•(**B5**)$_2$•(Pyr)$_2$ and were pleased to see that Rh_2(**A1**)$_2$•(**B1**)$_2$•(Pyr)$_2$ gave a high 91% *ee* (**Table S3**, Entries 7 and 8). Since Rh_2(**A1**)$_2$•(**B1**)$_2$•(Pyr)$_2$ consistently gave better reaction metrics than Rh_2(**A1**)$_2$•(**B5**)$_2$•(Pyr)$_2$, we decided to proceed with the substrate scope evaluation using Rh_2(**A1**)$_2$•(**B1**)$_2$•(Pyr)$_2$ as the catalyst. Furthermore, as we did not observe full

consumption of substrate **1i** at 1 mol% catalyst loading, we elected to use a higher catalyst loading of 2 mol% during the scope evaluation.

Table S4: Screen of aminating agent for the enantioselective aziridination of allylic carbamate **1a**.

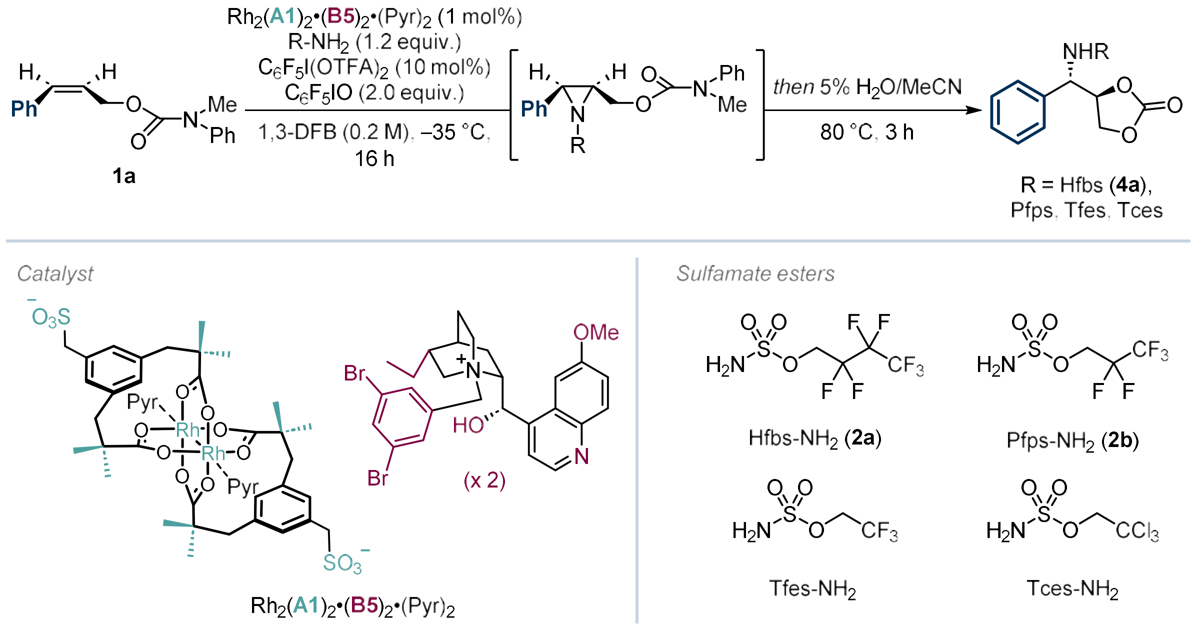

Entry	Sulfamate ester	Product yield/ %[a]	Product *ee*/ %[b]
1	Hfbs-NH_2 (**2a**)	72 (83)	91
2	Pfps-NH_2 (**2b**)	58	91
3	Tfes-NH_2	9	N.D.
4	Tces-NH_2	56	88

Reactions performed on 0.1 mmol scale with respect to allylic carbamate **1a**. N.D. refers to not determined. [a] Yields determined from crude ^{1}H NMR analysis with 1,2-dimethoxyethane as an internal standard, values in parenthesis correspond to isolated values. [b] *ee* was determined by chiral SFC/UV-vis analysis.

Table S5: Solvent screen for the enantioselective aziridination of allylic carbamate **1a**.

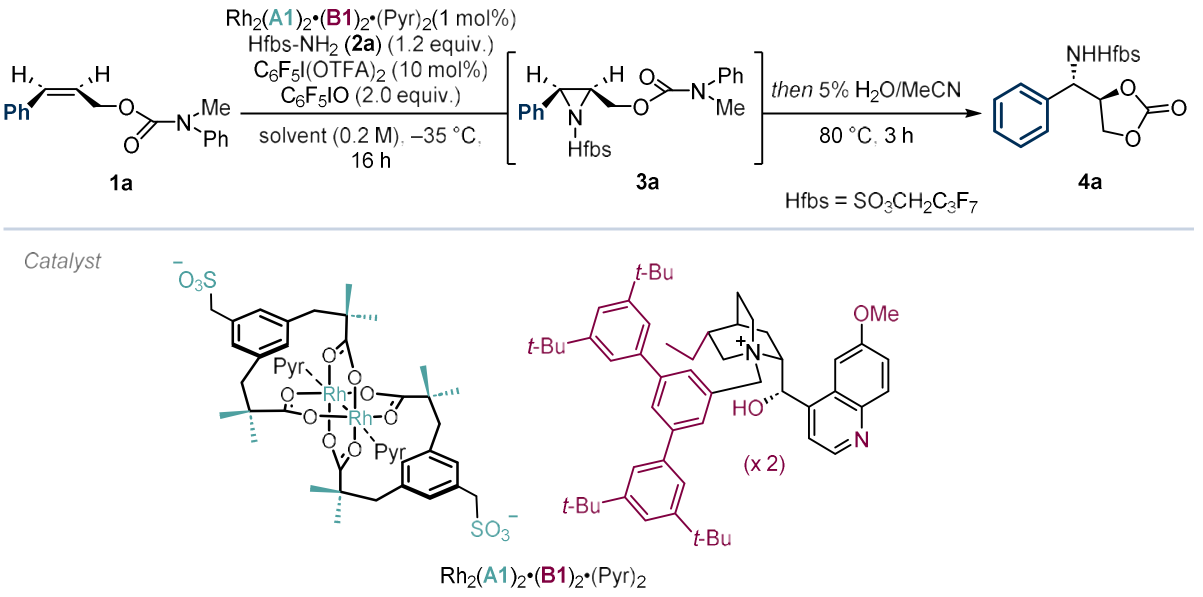

Entry	Solvent	Unreacted **1a**/ %[a]	Yield **4a**/ %[a]
1	MTBE	94	not detected
2	*t*-BuOAc	91	not detected
3	Toluene	82	not detected
4	Et_2O	66	not detected

Reactions performed on 0.1 mmol scale with respect to allylic carbamate **1a**. [a] Yields determined from crude ^{1}H NMR analysis with 1,2-dimethoxyethane as an internal standard.

2.1 Control Studies

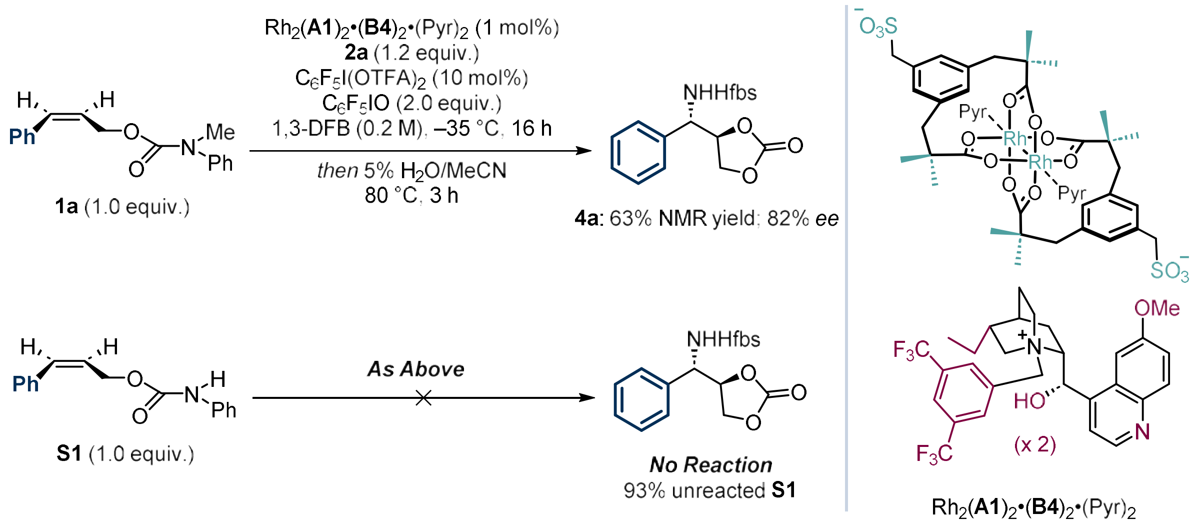

Scheme S1: Stark reactivity difference between N-Me allylic carbamate **1a** and N-H allylic carbamate **S1** under aziridination conditions. Reactions performed on 0.1 mmol scale with respect to allylic carbamate **1a** or **S1**. Yields determined from crude ^{1}H NMR analysis with 1,2-dimethoxyethane as an internal standard. *ee* was determined by chiral SFC-PDA analysis.

It is noteworthy that a stark reactivity difference was observed between *N*-methyl allylic carbamate **1a** and N-H allylic carbamate **S1** (**Scheme S1**). Reaction with **1a** afforded the carbonate product **4a** in 63% NMR yield and 82% *ee* whereas reaction with **S1** gave no reactivity to the desired carbonate product **4a**.

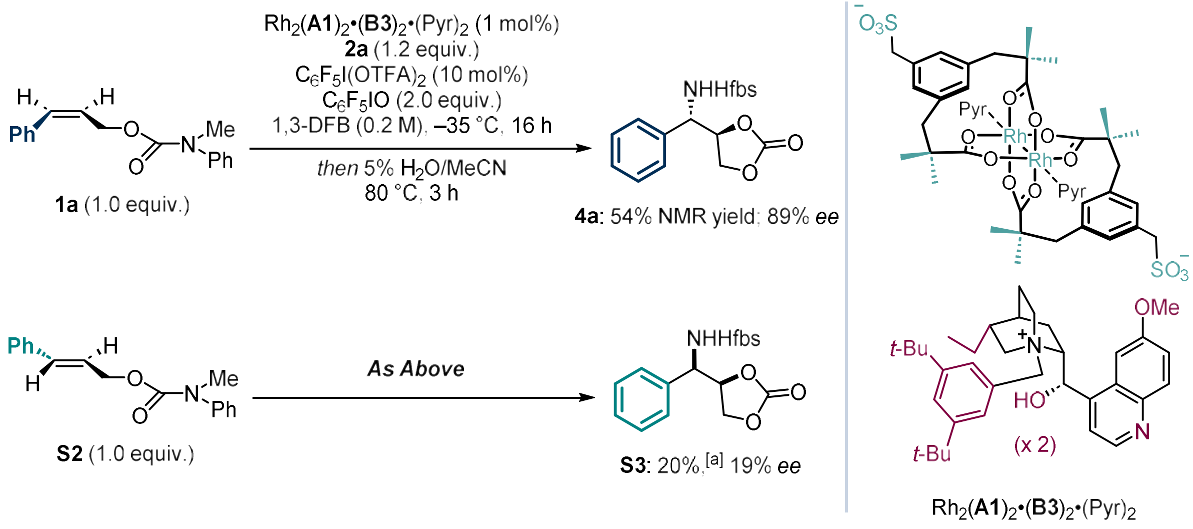

Scheme S2: Reactivity and enantioselectivity difference between *cis*-Ph allylic carbamate **1a** and *trans*-Ph allylic carbamate **S2** under aziridination conditions. Reactions performed on 0.1 mmol scale with respect to allylic carbamate **1a** or **S2**. Yields determined from crude ^1H NMR analysis with 1,2-dimethoxyethane as an internal standard. *ee* was determined by chiral SFC-PDA analysis. [a] Yield corresponds to isolated yield.

Subjecting *trans*-Ph allylic carbamate **S2** to the aziridination conditions with catalyst Rh_2(**A1**)$_2$•(**B3**)$_2$•(Pyr)$_2$ gave a poorer reaction outcome when compared to that of the model *cis*-Ph allylic carbamate **1a** (**Scheme S2**). The carbonate product **S3** was isolated in low yield and a low 19% *ee*.

2.2 Outcome of Substrate Containing Morpholine-Derived Carbamate

Attempted aziridination/cyclization of this substrate gave very low yield of desired **4a**. Analysis of the crude NMR spectra suggested extensive amination adjacent to morpholine had occurred but attempted purification led to a complex mixture.

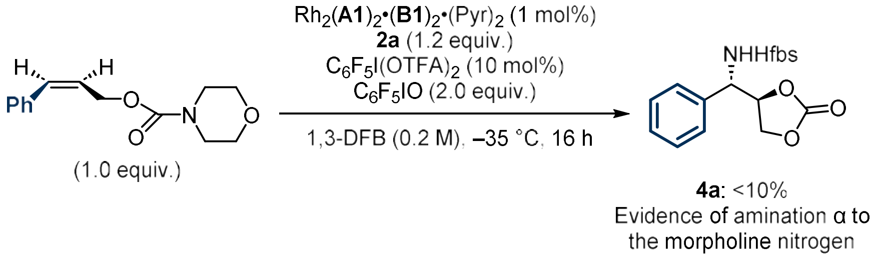

Scheme S3: Aminal formation from N-morpholine allylic carbamate under aziridination conditions. Reactions performed on 0.1 mmol scale with respect to allylic carbamate. Yields determined from crude ^1H NMR analysis with 1,2-dimethoxyethane as an internal standard.

Synthesis of starting material:

(Z)-3-phenylallyl morpholine-4-carboxylate

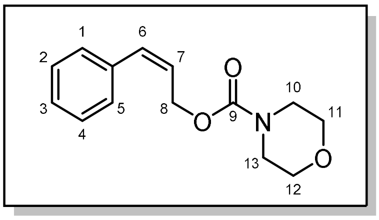

To a solution of *N,N'*-Carbonyldiimidazole (324 mg, 2.0 mmol, 1.0 equiv.) in anhydrous CH_2Cl_2 (5.0 mL) at 0°C , *(Z)*-3-Phenyl-2-propen-1-ol (268 mg, 2.0 mmol, 1.0 equiv.) was added as a solution in anhydrous CH_2Cl_2 (5.0 mL) and the resulting mixture was stirred at room temperature for 15 min. Morpholine (0.17 mL, 2.0 mmol, 1.0 equiv.) was added and the reaction was left to stir for 16 h, upon which the mixture was diluted with water and extracted with CH_2Cl_2. The combined organic layers were washed with aqueous HCl (1 M) and dried over $MgSO_4$. Purification by flash column chromatography (SiO_2, 0-20% v/v acetone in hexanes) afforded the title compound as a colourless oil (203 mg, 0.82 mmol, 41% yield, 96 mol% purity).

^{1}H NMR (700 MHz, $CDCl_3$): δ 7.38 – 7.32 (m, 2H, H-2,4), 7.30 – 7.26 (m, 1H, H-3), 7.26 – 7.21 (m, 2H, H-1,5), 6.65 (dt, *J* = 11.8, 1.8 Hz, 1H, H-6), 5.83 (dt, *J* = 11.7, 6.5 Hz, 1H, H-7), 4.89 (dd, *J* = 6.5, 1.7 Hz, 2H, H-8), 3.71 – 3.58 (m, 4H, H-10,13), 3.52 – 3.41 (m, 4H, H-11,12).

^{13}C NMR (176 MHz, $CDCl_3$): δ 155.3, 136.1, 132.5, 128.7, 128.4, 127.5, 126.5, 66.6, 62.5, 44.1.

R_f = 0.34 (20% v/v acetone in hexanes).

HRMS: *This compound did not ionise.*

3 Synthesis of Achiral Rhodium (II,II) Tetracarboxylate Dimer

The syntheses of compounds Rh_2(**A1**)$_2$•(***n*-Bu$_4$N**)$_2$, Rh_2(**A2**)$_2$•(***n*-Bu$_4$N**)$_2$, Rh_2(**A3**)$_2$•(***n*-Bu$_4$N**)$_2$, and Rh_2(**A4**)$_2$•(***n*-Bu$_4$N**)$_2$ have been previously reported.[4]

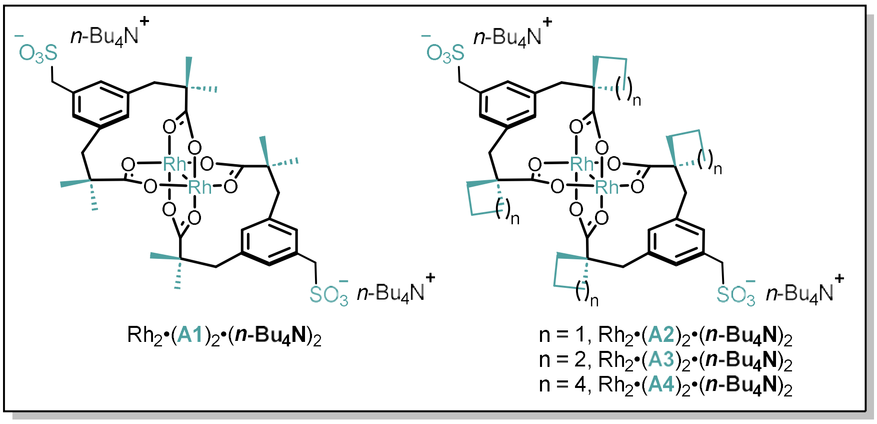

4 Synthesis and Characterisation of Chiral Cations

The syntheses of compounds **B1**•Br,[5] **B2**•Br,[6] **B3**•Br,[4] **B4**•Br,[4] **B6**•Br,[7] **B7**•Br,[7] **B8**•Br,[7] **B9**•Br,[7] **B10**•Br,[4] **C1**•Br,[4] **C2**•Br[5] and **C3**•Br[5] have been previously reported. **D2**•Br is commercially available.

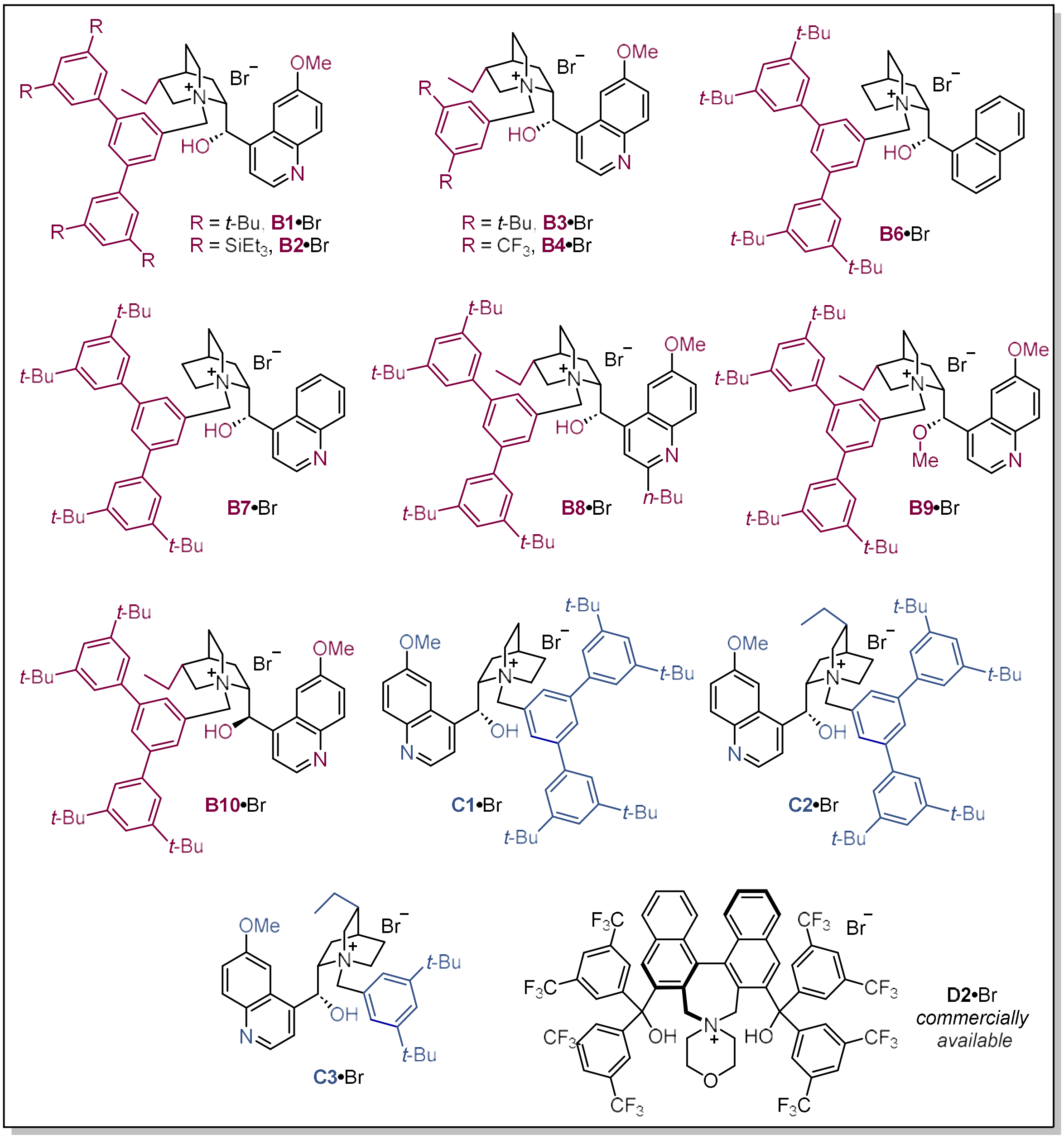

R = t-Bu, B1•Br
R = SiEt3, B2•Br
R = t-Bu, B3•Br
R = CF3, B4•Br
B6•Br
B7•Br
B8•Br
B9•Br
B10•Br
C1•Br
C2•Br
C3•Br
D2•Br
commercially available

(1S,2R,4S,5R)-1-(3,5-dibromobenzyl)-5-ethyl-2-((S)-hydroxy(6-methoxyquinolin-4-yl)methyl)quinuclidin-1-ium bromide (**B5**•Br)

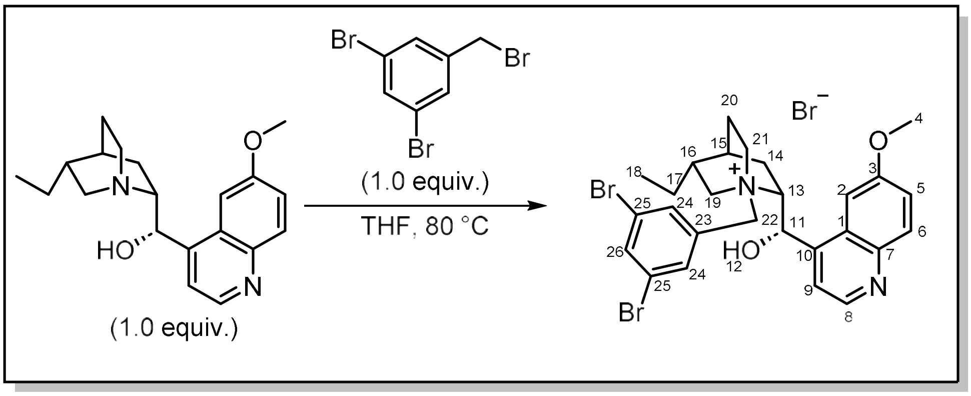

A microwave vial was charged with dihydroquinidine (211 mg, 0.646 mmol, 1.0 equiv.) and 3,5-bis(bromo)benzyl bromide (199.7 mg, 0.607 mmol, 0.94 equiv.). The vial was sealed, evacuated and backfilled with nitrogen thrice, before anhydrous THF (9 mL, 0.072 M) was added. The reaction mixture was heated at 80 ˚C overnight. The reaction mixture was allowed to cool to room temperature and the white solid was filtered and washed with ice-cold THF to afford the title compound (321 mg, 0.490 mmol, 81% yield).

^{1}H NMR (400 MHz, $CDCl_3$): *δ* 8.38 (s, 1H, H-8), 7.82-7.73 (m, 5H, H-2, H-6, H-9, H-24), 7.64 (s, 1H, H-26), 7.03 (d, *J* = 7.6 Hz, 1H, H-5), 6.60 (d, *J* = 5.0 Hz, 1H, H-12), 6.37 (br s, 1H, H-11), 6.01 (d, *J* = 12.0 Hz, 1H, H-22a), 5.53 (d, *J* = 11.9 Hz, 1H, H-22b), 4.29-4.22 (m, 3H, H-13, H-19a, H-21a), 3.73 (s, 3H, H-4), 3.22 (dd, *J* = 11.0, 10.8 Hz, 1H, H-19b), 2.78-2.71 (m, 1H, H-21b), 2.18 (dd, *J* = 12.0, 11.8 Hz, 1H, H-14a), 1.85-1.76 (m, 2H, H-20a, H-20b), 1.74-1.63 (m, 1H, H-15), 1.61-1.45 (m, 3H, H-16, H-17a, H-17b), 0.93-0.87 (m, 1H, H-14b), 0.84 (t, *J* = 7.3 Hz, 3H, H-18).

^{13}C NMR (101 MHz, $CDCl_3$): *δ* 158.0, 146.7, 143.8, 142.8, 136.3, 135.2, 131.4, 131.3, 126.5, 123.6, 120.7, 120.1, 103.7, 67.8, 59.9, 56.6, 56.4, 56.3, 36.2, 24.6, 24.4, 24.2, 22.0, 11.5.

$[\alpha]_D^{25.0}$ = +102.1 (c. 0.44, $CHCl_3$).

HRMS (+ESI): *m/z* for cation found 575.0749, $[C_{27}H_{31}Br_2N_2O_2]^+$ requires 575.0727, (*δ* = +3.8 ppm).

Dihydrocupreidine

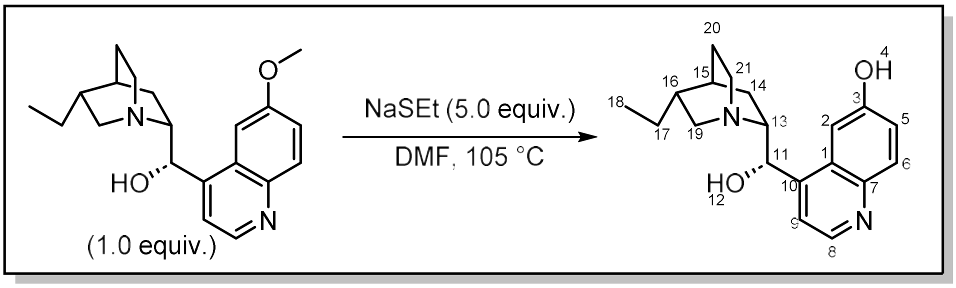

To a solution of dihydroquinidine (2.72 g, 8.33 mmol, 1.0 equiv.) in DMF (40 mL) was added NaSEt (4.26 g, 45.6 mmol, 5.5 equiv.) at room temperature. The reaction mixture was stirred at 105 ˚C overnight and cooled to room temperature. The reaction mixture was quenched with aqueous saturated NH_4Cl (90 mL). The aqueous layer was extracted with EtOAc (180 mL x 2) and the combined organic layers were dried over Na_2SO_4. The solvent was removed under reduced pressure and the crude residue was purified by flash column chromatography (SiO_2, 0-75% v/v *solvent mixture A* in CH_2Cl_2, where *solvent mixture A* = 70:29:1 CH_2Cl_2/MeOH/conc. NH_3 (aq.)) afforded the title compound as a yellow solid (1.73 g, 5.54 mmol, 66% yield).

^{1}H NMR (400 MHz, $(CD_3)_2SO$): *δ* 10.1 (br s, 1H, H-4), 8.59 (d, *J* = 4.4 Hz, 1 H, H-8), 7.86 (d, *J* = 9.1 Hz, 1H, H-6), 7.42-7.40 (m, 2H, H-2, H-9), 7.27 (dd, *J* = 9.1, 2.5 Hz, 1H, H-5), 5.72 (br s, 1H, H-12), 5.19 (d, *J* = 5.7 Hz, 1H, H-11), 2.99-2.93 (m, 1H, H-13), 2.88-2.81 (m, 1H, H-19a), 2.72-2.59 (m, 2H, H-19b, H-21a), 2.55-2.46 (m, 1H, H-21b), 1.89-1.84 (m, 1H, H-14a), 1.64 (br s, 1H, H-15), 1.51-1.41 (m, 3H, H-17a, H-17b, H-20a), 1.39-1.24 (m, 3H, H-14b, H-16, H-20b), 0.87 (t, *J* = 7.3 Hz, 3H, H-18).

^{13}C NMR (101 MHz, $(CD_3)_2SO$): *δ* 155.2, 148.2, 146.6, 143.2, 131.1, 127.2, 121.2, 118.8, 104.9, 71.1, 60.1, 50.0, 49.4, 37.0, 27.0, 25.9, 25.0, 22.6, 12.0.

R_f = 0.62 ((60% v/v *solvent mixture A* in CH_2Cl_2; *solvent mixture A* = 70:29:1 CH_2Cl_2/MeOH/conc. NH_3 (aq.)).

The spectroscopic data is in agreement with that reported in the literature.[8]

(1S,2R,4S,5R)-1-(3,5-di-tert-butylbenzyl)-5-ethyl-2-((S)-hydroxy(6-hydroxyquinolin-4-yl)methyl)quinuclidin-1-ium bromide (**B11•**Br)

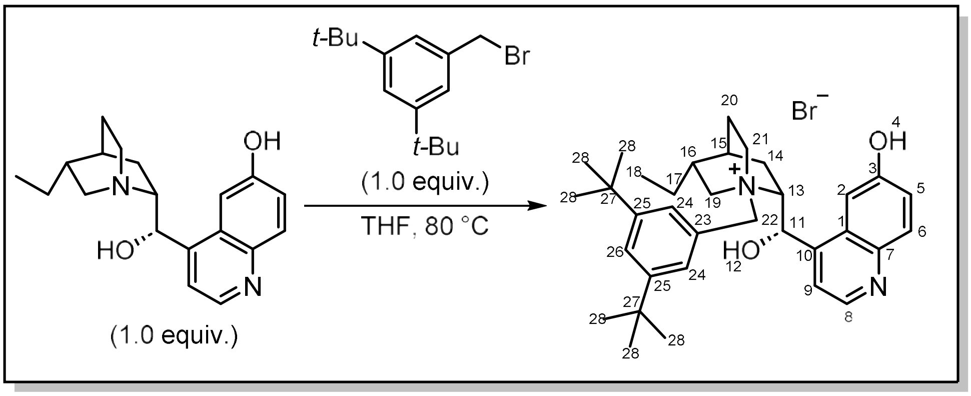

A microwave vial was charged with dihydrocupreidine (184.9 mg, 0.59 mmol, 1.0 equiv.) and 3,5-di-*tert*-butylbenzyl bromide (168.4 mg, 0.59 mmol, 1.0 equiv.). The vial was sealed, evacuated and backfilled with nitrogen thrice, before anhydrous THF (9 mL, 0.067 M) was added. The reaction mixture was heated at 80 ˚C overnight. The reaction mixture was allowed to cool to room temperature. The solid was filtered and washed with diethyl ether to give the title compound as an off-white solid (209 mg, 0.351 mmol, 59% yield).

^{1}H NMR (700 MHz, $(CD_3)_2SO$): δ 10.1 (s, 1H, H-4), 8.75 (d, *J* = 4.4 Hz, 1H, H-8), 7.95 (d, *J* = 9.2 Hz, 1H, H-6), 7.72 (d, *J* = 4.4 Hz, 1H, H-9), 7.61-7.56 (m, 4H, H-2, H-24, H-26), 7.38 (dd, *J* = 9.1, 2.4 Hz, 1H, H-5), 6.67 (d, *J* = 4.0 Hz, 1H, H-12), 6.33 (br s, 1H, H-11), 5.02 (d, *J* = 12.4 Hz, 1H, H-22a), 4.94 (d, *J* = 12.4 Hz, 1H, H-22b), 4.04-4.01 (m, 1H, H-21a), 3.95-3.92 (m, 1H, H-19a), 3.86 (t, *J* = 9.4 Hz, 1H, H-13), 3.40 (t, *J* = 11.4 Hz, 1H, H-19b), 2.87 (q, *J* = 10.3 Hz, 1H, H-21b), 2.30 (dd, *J* = 11.8, 11.3 Hz, 1H, H-14a), 1.84 (br s, 1H, H-15), 1.79-1.74 (m, 3H, H-16, H-20), 1.59-1.53 (m, 1H, H-17a), 1.51-1.46 (m, 1H, H-17b), 1.36 (s, 18H, H-28), 1.11-1.07 (m, 1H, H-14b), 0.84 (t, *J* = 7.4 Hz, 3H, H-18).

^{13}C NMR (176 MHz, $(CD_3)_2SO$): δ 156.0, 150.9, 146.5, 143.1, 142.7, 131.2, 128.2, 127.0, 125.7, 123.4, 121.9, 119.9, 104.6, 67.2, 64.7, 62.8, 56.0, 55.4, 34.7, 34.6, 31.2, 24.3, 23.8, 23.6, 20.3, 11.2.

$[\alpha]_D^{25.0}$ = +155.7 (c. 0.41, MeOH).

HRMS (+ESI): *m/z* for cation found 515.3635, $[C_{34}H_{47}N_2O_2]^+$ requires 515.3632, (δ = +0.6 ppm).

(S)-(6-((tert-butyldimethylsilyl)oxy)quinolin-4-yl)((1S,2R,4S,5R)-5-ethylquinuclidin-2-yl)methanol

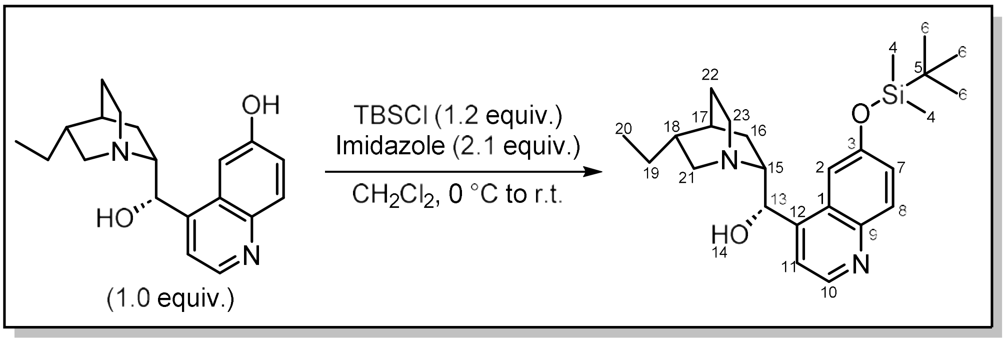

To a solution of dihydrocupreidine (779 mg, 2.49 mmol, 1.0 equiv.) in CH_2Cl_2 (15 mL, 0.17 M) was added imidazole (360 mg, 5.29 mmol, 2.1 equiv.), and TBSCl (455 mg, 3.02 mmol, 1.2 equiv.) at 0 ˚C. The reaction mixture was allowed to stir at room temperature for 4.5 h before diluting it with CH_2Cl_2. The organic layer was washed with water, dried over $MgSO_4$ and concentrated under reduced pressure. Purification of the crude residue by flash column chromatography (SiO_2, 0-35% v/v *solvent mixture A* in CH_2Cl_2, where *solvent mixture A* = 70:29:1 CH_2Cl_2/MeOH/conc. NH_3 (aq.)) afforded the title compound as a yellow solid (951 mg, 2.23 mmol, 90% yield).

^{1}H NMR (400 MHz, CDCl$_3$): δ 8.69 (d, J = 4.5 Hz, 1H, H-10), 7.94 (d, J = 9.1 Hz, 1H, H-8), 7.55 (d, J = 4.6 Hz, 1H, H-11), 7.34 (d, J = 2.5 Hz, 1H, H-2), 7.22 (dd, J = 9.1, 2.5 Hz, 1H, H-7), 5.72 (d, J = 4.0 Hz, 1H, H-13), 3.21-3.18 (m, 1H, H-21a), 3.12-3.07 (m, 1H, H-15), 2.95-2.90 (m, 2H, H-21b, H-23a), 2.81-2.73 (m, 1H, H-23b), 1.96 (dd, J = 12.7, 10.0 Hz, 1H, H-16a), 1.71 (br s, 1H, H-17), 1.54-1.49 (m, 2H, H-22), 1.46-1.38 (m, 3H, H-18, H-19), 1.14-1.06 (m, 1H, H-16b), 0.98 (s, 9H, H-6), 0.85 (t, J = 7.2 Hz, 3H, H-20), 0.22 (s, 6H, H-4).

Note: The signal of H-14 (-OH) was not observed.

^{13}C NMR (101 MHz, CDCl$_3$): δ 154.0, 148.0, 147.3, 144.4, 131.7, 126.7, 125.2, 118.8, 110.4, 71.3, 60.0, 51.2, 50.2, 37.2, 26.7, 26.3, 25.8, 25.1, 20.9, 18.4, 12.0, -4.2.

$[\alpha]_D^{25.0}$ = +209.8 (c. 0.55, $CHCl_3$).

R_f = 0.41 (30% v/v *solvent mixture A* in CH_2Cl_2; *solvent mixture A* = 70:29:1 CH_2Cl_2/MeOH/conc. NH_3 (aq.)).

HRMS (+ESI): *m/z* found $[M+H]^+$ 427.2775, $[C_{25}H_{39}N_2O_2Si]^+$ requires 427.2775, (δ = ±0.0 ppm).

(S)-((1S,2R,4S,5R)-5-ethylquinuclidin-2-yl)(6-hydroxyquinolin-4-yl)methyl benzoate

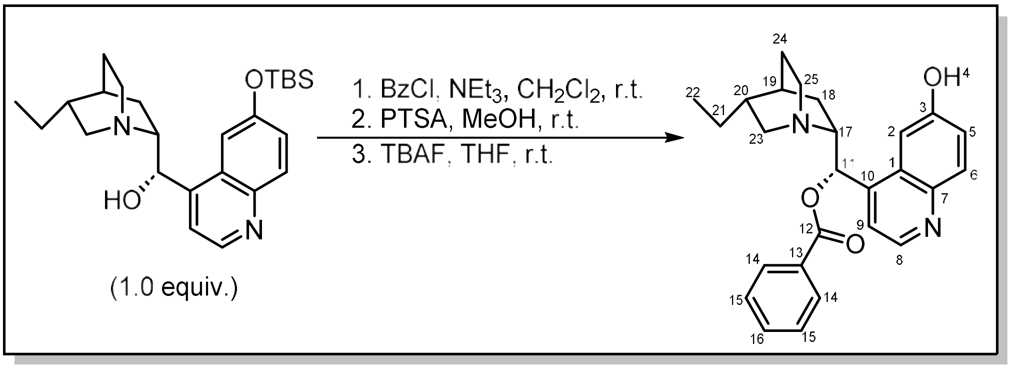

To a solution of (*S*)-(6-((*tert*-butyldimethylsilyl)oxy)quinolin-4-yl)((1*S*,2*R*,4*S*,5*R*)-5-ethylquinuclidin-2-yl)methanol (423 mg, 0.991 mmol, 1.0 equiv.) in anhydrous CH_2Cl_2 (6 mL, 0.17 M) was added triethylamine (0.3 mL, 2.14 mmol, 2.2 equiv.) and benzoyl chloride (0.15 mL, 1.27 mmol, 1.3 equiv.) at room temperature. The reaction mixture was allowed to stir overnight at room temperature and diluted with CH_2Cl_2. The organic layer was washed with aqueous saturated $NaHCO_3$, brine and dried over Na_2SO_4. The crude mixture was concentrated under reduced pressure and taken forward to the next step without further purification.

The crude residue was dissolved in MeOH (6 mL, 0.17 M) and *p*-toluenesulfonic acid (509 mg, 2.96 mmol, 3.0 equiv.) was added at room temperature. The reaction mixture was allowed to stir at room temperature overnight but TLC showed incomplete reaction. The solvent was removed under reduced pressure and the crude residue was reconstituted in EtOAc. The organic layer was washed with aqueous saturated $NaHCO_3$, and the aqueous layer was extracted with EtOAc. The combined organic layers were washed with brine and dried over Na_2SO_4. The solvent was removed under reduced pressure and the crude residue was reconstituted in THF. TBAF (0.5 mL, 0.5 mmol, 1 M in THF) was added to the reaction mixture and stirred at room temperature for 1 h. TLC analysis showed complete reaction and the reaction mixture was concentrated under reduced pressure. Purification of the crude residue by flash column chromatography (SiO_2, 0-25% v/v *solvent mixture A* in CH_2Cl_2, where *solvent mixture A* = 70:29:1 CH_2Cl_2/MeOH/conc. NH_3 (aq.)) afforded the title compound as an off-white solid (265 mg, 0.636 mmol, 64% yield over three steps).

^{1}H NMR (700 MHz, $CDCl_3$): δ 8.65 (d, *J* = 4.6 Hz, 1H, H-8), 8.05 (d, *J* = 7.1 Hz, 2H, H-14), 7.90 (d, *J* = 9.0 Hz, 1H, H-6), 7.64 (d, *J* = 2.4 Hz, 1H, H-2), 7.56 (t, *J* = 7.5 Hz, 1H, H-16), 7.42 (t, *J* = 7.9 Hz, 2H, H-15), 7.39 (d, *J* = 4.6 Hz, 1H, H-9), 7.18 (dd, *J* = 8.9, 1.8 Hz, 1H, H-5), 6.85 (d, *J* = 4.7 Hz, 1H, H-11), 3.43 (dd, *J* = 8.8, 6.0 Hz, 1H, H-17), 3.06-3.03 (m, 1H, H-23a), 2.90-2.88 (m, 2H, H-23b, H-25a), 2.77-2.73 (m, 1H, H-25b), 2.01 (dd, *J* = 11.2, 10.6

Hz, 1H, H-18a), 1.76 (br s, 1H, H-19), 1.58-1.42 (m, 6H, H-18b, H-20, H-21, H-24), 0.88 (t, *J* = 7.1 Hz, 3H, H-22).

Note: The signal of H-4 (-OH) was not observed.

^{13}C NMR (176 MHz, CDCl$_3$): *δ* 165.4, 156.5, 146.5, 143.9, 143.1, 133.5, 131.5, 129.84, 129.79, 128.7, 127.3, 122.9, 118.5, 105.8, 73.9, 58.8, 50.7, 49.9, 37.2, 26.7, 26.1, 25.4, 22.6, 12.0.

R$_f$ = 0.43 (30% v/v *solvent mixture A* in CH_2Cl_2; *solvent mixture A* = 70:29:1 CH_2Cl_2/MeOH/conc. NH_3 (aq.)).

$[\alpha]_D^{25.0}$ = -23.2 (c. 0.61, $CHCl_3$).

HRMS (+ESI): *m/z* found $[M+H]^+$ 417.2180, $[C_{26}H_{29}N_2O_3]^+$ requires 417.2173, (*δ* = +1.7 ppm).

(1S,2R,4S,5R)-2-((S)-(benzoyloxy)(6-hydroxyquinolin-4-yl)methyl)-1-(3,5-di-tert-butylbenzyl)-5-ethylquinuclidin-1-ium bromide (**B12•**Br)

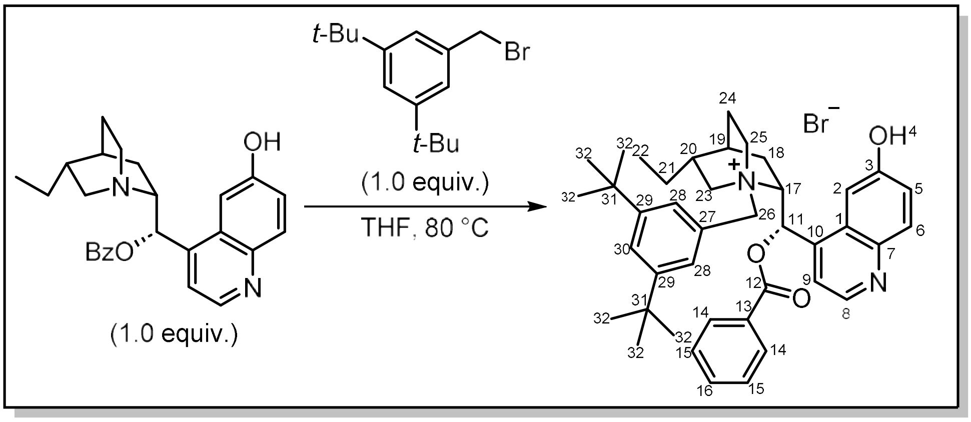

A microwave vial was charged with (*S*)-((1*S*,2*R*,4*S*,5*R*)-5-ethylquinuclidin-2-yl)(6-hydroxyquinolin-4-yl)methyl benzoate (121 mg, 0.291 mmol, 1.0 equiv.) and 3,5-di-*tert*-butylbenzyl bromide (83 mg, 0.293 mmol, 1.0 equiv.). The vial was sealed, evacuated and backfilled with nitrogen thrice, before anhydrous THF (4.5 mL, 0.065 M) was added. The reaction mixture was heated at 80 ˚C overnight. The reaction mixture was allowed to cool to room temperature. The solid was filtered and washed with diethyl ether to give the title compound as a white solid (145 mg, 0.208 mmol, 71% yield).

^{1}H NMR (700 MHz, CDCl$_3$): *δ* 9.62 (s, 1H, H-4), 8.64 (d, *J* = 4.7 Hz, 1H, H-8), 8.46 (d, *J* = 2.5 Hz, 1H, H-2), 8.25 (dd, *J* = 8.2, 1.0 Hz, 2H, H-14), 8.02 (d, *J* = 9.0 Hz, 1H, H-6), 7.80 (tt, *J* = 7.6, 1.2 Hz, 1H, H-16), 7.66 (t, *J* = 7.9 Hz, 2H, H-15), 7.57 (d, *J* = 2.4 Hz, 1H, H-11), 7.50 (m, 3H, H-28, H-30), 7.46 (dd, *J* = 9.0, 2.4 Hz, 1H, H-5), 7.33 (d, *J* = 4.4 Hz, 1H, H-9),

6.29 (d, J = 11.9 Hz, 1H, H-26a), 5.33 (dd, J = 11.9, 11.4 Hz, 1H, H-25a), 4.87 (dd, J = 9.7, 9.2 Hz, 1H, H-17), 4.22 (d, J = 12.1 Hz, 1H, H-26b), 3.55 (t, J = 11.1 Hz, 1H, H-23a), 3.45-3.41 (m, 1H, H-23b), 2.94-2.90 (m, 1H, H-25b), 2.41 (dd, J = 12.6, 12.1 Hz, 1H, H-18a), 2.08-2.04 (m, 2H, H-19, H-24a), 1.83-1.65 (m, 4H, H-20, H-21, H-24b), 1.42-1.38 (m, 1H, H-18b), 1.30 (s, 18H, H-32), 0.91 (t, J = 7.4 Hz, 3H, H-22).

^{13}C NMR (176 MHz, $CDCl_3$): δ 163.9, 157.8, 152.3, 146.1, 143.9, 137.4, 135.0, 131.8, 129.8, 129.6, 128.45, 128.44, 125.6, 125.1, 124.7, 124.3, 117.4, 105.3, 69.6, 65.2, 63.5, 56.7, 56.0, 35.9, 35.1, 31.5, 25.3, 24.9, 24.3, 22.7, 11.2.

$[\alpha]_D^{25.0}$ = -12.3 (c. 0.47, $CHCl_3$).

HRMS (+ESI): *m/z* for cation found 619.3889, $[C_{41}H_{51}N_2O_3]^+$ requires 619.3894, (δ = -0.8 ppm).

(R)-((1S,2S,4S,5R)-5-ethylquinuclidin-2-yl)(6-methoxyquinolin-4-yl)methanamine

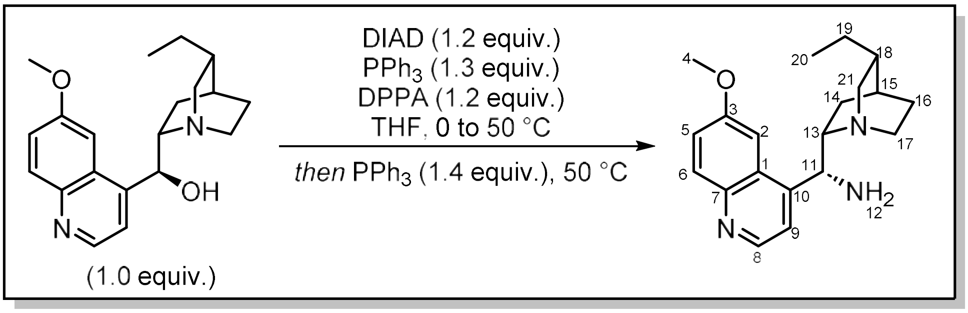

To a solution of 9-*epi*-dihydroquinine (8.00 g, 24.5 mmol, 1.0 equiv.) and triphenylphosphine (8.35 g, 31.8 mmol, 1.3 equiv.) in anhydrous THF (180 mL, 0.14 M) at 0 ˚C, was added diisopropyl azodicarboxylate (5.8 mL, 29.4 mmol, 1.2 equiv.) dropwise. The reaction mixture was allowed to stir at 0 ˚C for 10 min before the dropwise addition of a solution of diphenyl phosphoryl azide (6.3 mL, 29.4 mmol, 1.2 equiv.) in THF (80 mL, 0.37 M). The reaction mixture was warmed to room temperature and stirred at this temperature overnight. The reaction mixture was heated to 50 ˚C for 2 h before the addition of triphenylphosphine (9.00 g, 34.3 mmol, 1.4 equiv.). The reaction mixture was stirred at 50 ˚C for an additional 2 h and cooled to room temperature. Distilled water (8.9 mL) was added and the reaction mixture was stirred for 2 h. The solvent was removed under reduced pressure and the crude residue was reconstituted in CH_2Cl_2. Aqueous 3 M HCl was added and the phases were separated. The organic layer was washed with aqueous 3 M HCl and combined aqueous layers were washed with CH_2Cl_2. The aqueous layer was basified with concentrated aqueous ammonia solution (35%) and extracted with CH_2Cl_2. The combined organic layers were dried over $MgSO_4$ and concentrated under reduced pressure. The crude residue was purified by flash

column chromatography (SiO_2, 0-30% v/v *solvent mixture A* in CH_2Cl_2, where *solvent mixture A* = 70:29:1 CH_2Cl_2/MeOH/conc. NH_3 (aq.)) to afford the title compound as a yellow solid (5.49 g, 16.9 mmol, 69% yield).

^{1}H NMR (400 MHz, CDCl$_3$): δ 8.73 (d, J = 4.6 Hz, 1H, H-8), 8.00 (d, J = 9.2 Hz, 1H, H-6), 7.55 (d, J = 2.0 Hz, 1H, H-2), 7.36 (dd, J = 9.1, 2.5 Hz, 1H, H-5), 7.33 (d, J = 4.6 Hz, 1H, H-9), 4.03 (s, 3H, H-4), 3.59 (br s, 1H, H-17a), 3.25-3.19 (m, 1H, H-13), 3.15 (dd, J = 13.4, 10.6 Hz, 1H, H-21a), 2.76-2.72 (m, 1H, H-17b), 2.52 (d, J = 13.0 Hz, 1H, H-21b), 1.93 (br s, 1H, H-15), 1.89-1.79 (m, 2H, H-14a, H-16a), 1.69-1.64 (m, 1H, H-14b), 1.62-1.52 (m, 2H, H, H-16b, H-18), 1.39-1.31 (m, 2H, H-19a, H-19b), 0.86 (t, J = 7.4 Hz, 3H, H-20).

Note: The signals of H-11 (-CH) and H-12 (-NH$_2$) were not observed.

^{13}C NMR (176 MHz, CDCl$_3$): δ 158.5, 148.4, 147.7, 144.9, 132.0, 127.3, 122.1, 118.0, 101.3, 60.4, 57.7, 56.6, 52.3, 42.7, 36.9, 27.7, 27.4, 25.4, 12.1.

Note: The signal of C-14 was not observed.

$[\alpha]_D^{25.0}$ = -56.0 (c. 0.99, $CHCl_3$).

R_f = 0.42 (30% v/v *solvent mixture A* in CH_2Cl_2; *solvent mixture A* = 70:29:1 CH_2Cl_2/MeOH/conc. NH_3 (aq.)).

HRMS (+ESI): *m/z* found $[M+H]^+$ 326.2226, $[C_{20}H_{28}N_3O]^+$ requires 326.2227, (δ = -0.3 ppm).

1-(3,5-bis(trifluoromethyl)phenyl)-3-((R)-((1S,2S,4S,5R)-5-ethylquinuclidin-2-yl)(6-methoxyquinolin-4-yl)methyl)urea

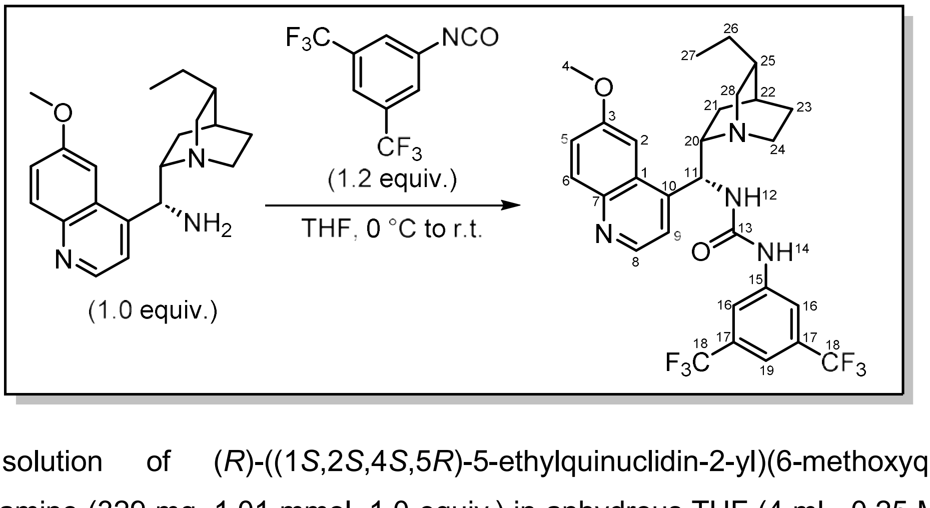

To a solution of (*R*)-((1*S*,2*S*,4*S*,5*R*)-5-ethylquinuclidin-2-yl)(6-methoxyquinolin-4-yl)methanamine (329 mg, 1.01 mmol, 1.0 equiv.) in anhydrous THF (4 mL, 0.25 M) at 0 °C was added 3,5-bis(trifluoromethyl)phenyl isocyanate (0.21 mL, 1.20 mmol, 1.2 equiv.). The reaction mixture was allowed to warm to room temperature overnight and concentrated under reduced pressure. The crude residue was purified by flash column chromatography

(SiO$_2$, 0-40% v/v *solvent mixture A* in CH_2Cl_2, where *solvent mixture A* = 70:29:1 CH_2Cl_2/MeOH/conc. NH_3 (aq.)) to afford the title compound as a pale yellow solid (444 mg, 0.765 mmol, 76% yield).

^{1}H NMR (700 MHz, CDCl$_3$): *δ* 8.56 (br s, 1H, H-14), 8.37 (d, *J* = 4.6 Hz, 1H, H-8), 7.88 (s, 2H, H-16), 7.52 (d, *J* = 9.1 Hz, 1H, H-6), 7.47-7.46 (m, 2H, H-2, H-19), 7.14 (d, *J* = 4.6 Hz, 1H, H-9), 7.09 (dd, *J* = 9.1, 2.6 Hz, 1H, H-5), 5.73 (t, *J* = 9.7 Hz, 1H, H-11), 5.57 (d, *J* = 7.8 Hz, 1H, H-12), 3.90 (s, 3H, H-4), 3.27-3.23 (m, 1H, H-20), 3.05 (dd, *J* = 13.2, 9.0 Hz, 1H, H-28a), 2.85-2.81 (m, 1H, H-24a), 2.50-2.46 (m, 1H, H-24b), 2.41 (d, *J* = 13.3 Hz, 1H, H-28b), 2.00-1.97 (m, 1H, H-21a), 1.83 (br s, 1H, H-22), 1.78-1.74 (m, 1H, H-23a), 1.66 (dd, *J* = 12.7, 6.9 Hz, 1H, H-21b), 1.46-1.38 (m, 4H, H-23b, H-25, H-26a, H-26b), 0.92 (t, *J* = 7.1 Hz, 3H, H-27).

^{13}C NMR (176 MHz, CDCl$_3$): *δ* 158.6, 155.0, 146.8, 145.9, 144.0, 141.1, 132.4 (q, J_{C-F} = 33.2 Hz),130.0, 128.5, 123.3 (q, J_{C-F} = 272.4 Hz), 122.9, 119.0, 118.0, 115.7, 101.6, 57.81, 57.76, 55.9, 50.4, 41.7, 37.5, 28.3, 28.0, 25.33, 25.27, 12.3.

^{19}F NMR (376 MHz, CDCl$_3$): *δ* -63.1.

$[\alpha]_D^{25.0}$ = -25.6 (c. 0.73, $CHCl_3$).

R_f = 0.31 (30% v/v *solvent mixture A* in CH_2Cl_2; *solvent mixture A* = 70:29:1 CH_2Cl_2/MeOH/conc. NH_3 (aq.)).

HRMS (+ESI): *m/z* found $[M+H]^+$ 581.2329, $[C_{29}H_{31}N_4O_2F_6]^+$ requires 581.2346, (*δ* = -2.9 ppm).

(1S,2S,4S,5R)-2-((R)-(3-(3,5-bis(trifluoromethyl)phenyl)ureido)(6-methoxyquinolin-4-yl)methyl)-1-(3,5-di-tert-butylbenzyl)-5-ethylquinuclidin-1-ium bromide (**C4**•Br)

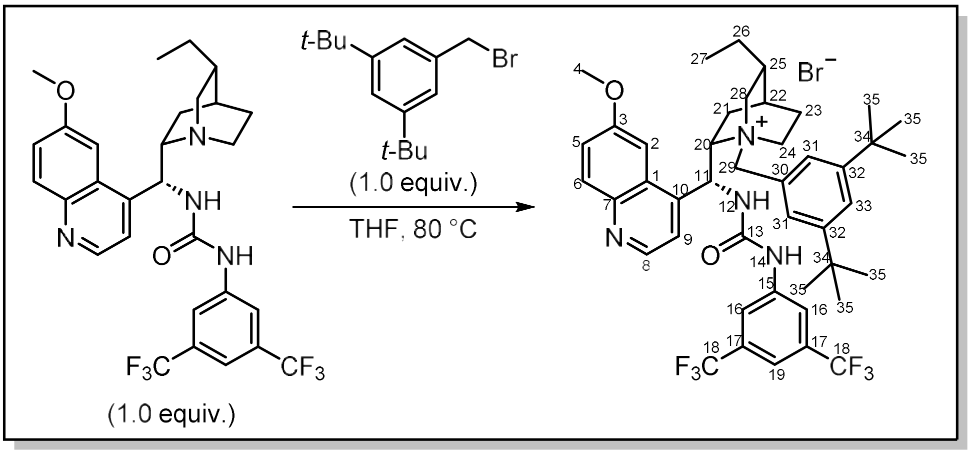

A microwave vial was charged with 1-(3,5-bis(trifluoromethyl)phenyl)-3-((*R*)-((1*S*,2*S*,4*S*,5*R*)-5-ethylquinuclidin-2-yl)(6-methoxyquinolin-4-yl)methyl)urea (170 mg, 0.293 mmol, 1.0 equiv.) and 3,5-di-*tert*-butylbenzyl bromide (87 mg, 0.307 mmol, 1.05 equiv.). The vial was sealed, evacuated and backfilled with nitrogen thrice, before anhydrous THF (4.5 mL, 0.067 M) was added. The reaction mixture was heated at 80 °C overnight. The reaction mixture was allowed to cool to room temperature and concentrated under reduced pressure. Purification of the crude residue by flash column chromatography (SiO_2, 0-25% v/v *solvent mixture A* in CH_2Cl_2, where *solvent mixture A* = 70:29:1 CH_2Cl_2/MeOH/conc. NH_3 (aq.)) afforded the title compound as a yellow solid (201 mg, 0.233 mmol, 79% yield).

^{1}H NMR (700 MHz, $CDCl_3$): δ 10.2 (s, 1H, H-14), 9.43 (d, *J* = 10.1 Hz, 1H, H-12), 8.84 (d, *J* = 4.6 Hz, 1H, H-8), 8.16 (s, 2H, H-16), 8.11 (d, *J* = 9.2 Hz, 1H, H-6), 7.93 (d, *J* = 4.5 Hz, 1H, H-9), 7.55 (t, *J* = 1.6 Hz, 1H, H-33), 7.46-7.44 (m, 2H, H-5, H-19), 7.34 (d, *J* = 2.6 Hz, 1H, H-2), 7.18 (d, *J* = 1.7 Hz, 2H, H-31), 6.94 (d, *J* = 10.1 Hz, 1H, H-11), 5.45 (d, *J* = 12.7 Hz, 1H, H-29a), 4.56 (d, *J* = 12.6 Hz, 1H, H-29b), 4.11-4.06 (m, 1H, H-24a), 3.97 (s, 3H, H-4), 3.72-3.66 (m, 2H, H-20, H-28a), 3.16-3.12 (m, 1H, H-24b), 2.99-2.94 (m, 1H, H-23a), 2.86-2.83 (m, 1H, H-28b), 2.81-2.78 (m, 1H, H-21a), 2.25-2.24 (m, 1H, H-22), 1.92-1.89 (m, 1H, H-25), 1.88-1.83 (m, 1H, H-21b), 1.81-1.76 (m, 1H, H-23b), 1.37-1.31 (m, 2H, H-26a, H-26b), 1.21 (s, 18H, H-35), 0.83 (t, *J* = 7.4 Hz, 3H, H-27).

^{13}C NMR (176 MHz, $CDCl_3$): δ 158.9, 155.8, 152.9, 148.4, 144.8, 141.1, 140.5, 133.1, 132.1 (q, $J_{C\text{-}F}$ = 33.0 Hz), 127.3, 126.1, 125.5, 125.0, 123.4 (q, $J_{C\text{-}F}$ = 273.2 Hz), 121.02, 121.00, 118.2, 115.6, 101.5, 68.4, 66.2, 65.0, 56.1, 51.5, 47.7, 36.5, 35.0, 31.3, 26.7, 25.2, 24.3, 23.3, 11.4.

^{19}F NMR (376 MHz, $CDCl_3$): δ -63.9.

$[\alpha]_D^{25.0}$ = +11.3 (c. 0.46, $CHCl_3$).

R_f = 0.50 (30% v/v *solvent mixture A* in CH_2Cl_2; *solvent mixture A* = 70:29:1 CH_2Cl_2/MeOH/conc. NH_3 (aq.)).

HRMS (+ESI): *m/z* for cation found 783.4086, $[C_{44}H_{53}N_4O_2F_6]^+$ requires 783.4067, (δ = +2.4 ppm).

1-(3,5-bis(trifluoromethyl)phenyl)-3-((R)-((1S,2S,4S,5R)-5-ethylquinuclidin-2-yl)(6-methoxyquinolin-4-yl)methyl)thiourea

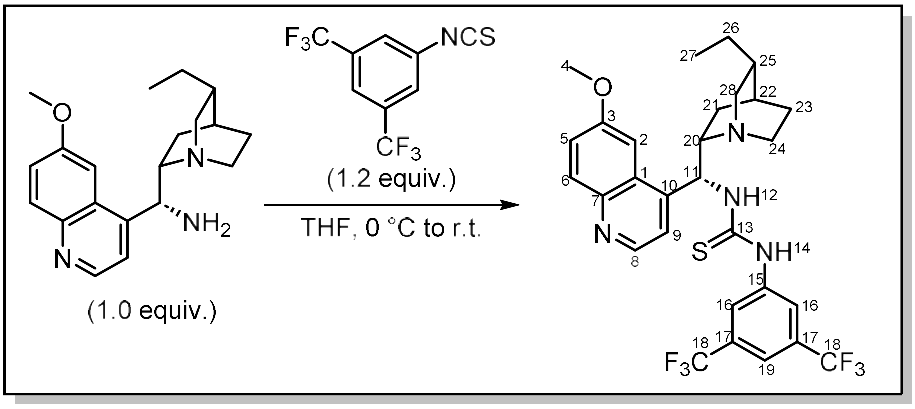

To a solution of (*R*)-((1*S*,2*S*,4*S*,5*R*)-5-ethylquinuclidin-2-yl)(6-methoxyquinolin-4-yl)methanamine (324 mg, 0.996 mmol, 1.0 equiv.) in anhydrous THF (4 mL, 0.25 M) at 0 ˚C was added 3,5-bis(trifluoromethyl)phenyl isothiocyanate (0.22 mL, 1.20 mmol, 1.2 equiv.). The reaction mixture was allowed to warm to room temperature overnight and concentrated under reduced pressure. The crude residue was purified by flash column chromatography (SiO_2, 0-35% v/v *solvent mixture A* in CH_2Cl_2, where *solvent mixture A* = 70:29:1 CH_2Cl_2/MeOH/conc. NH_3 (aq.)) to afford the title compound as a pale yellow solid (425 mg, 0.712 mmol, 72% yield).

^{1}H NMR (700 MHz, $CDCl_3$): δ 9.26 (br s, 1H, H-12), 8.40 (s, 1H, H-8), 7.83 (s, 2H, H-16), 7.77 (s, 1H, H-2), 7.63 (d, J = 8.3 Hz, 1H, H-6), 7.59 (s, 1H, H-19), 7.18 (d, J = 7.6 Hz, 1H, H-5), 7.15 (d, J = 3.7 Hz, 1H, H-9), 6.60 (br s, 1H, H-11), 3.97 (s, 3H, H-4), 3.44-3.41 (m, 1H, H-20), 3.05 (dd, J = 13.2, 9.4 Hz, 1H, H-28a), 2.99-2.93 (m, 1H, H-24a), 2.53-2.49 (m, 1H, H-24b), 2.40 (d, J = 13.1 Hz, 1H, H-28b), 2.07-2.01 (m, 1H, H-21a), 1.96-1.90 (m, 1H, H-21b), 1.85-1.81 (m, 2H, H-22, H-23a), 1.49-1.39 (m, 4H, H-23b, H-25, H-26a, H-26b), 0.92 (t, J = 7.1 Hz, 3H, H-27).

Note: The signal of H-14 (-NH) was not observed.

^{13}C NMR (176 MHz, $CDCl_3$): δ 181.2, 158.6, 146.8, 144.8, 144.2, 140.1, 132.6 (q, $J_{C\text{-}F}$ = 33.1 Hz), 130.3, 128.6, 123.04, 122.99 (q, $J_{C\text{-}F}$ = 272.6 Hz), 122.7, 119.0, 118.5, 102.6, 58.6, 57.9, 56.6, 54.4, 42.2, 37.5, 28.0, 27.8, 25.4, 24.3, 12.3.

^{19}F NMR (376 MHz, $CDCl_3$): δ -64.1.

$[\alpha]_D^{25.0}$ = +44.6 (c. 0.53, $CHCl_3$).

R_f = 0.25 (30% v/v *solvent mixture A* in CH_2Cl_2; *solvent mixture A* = 70:29:1 CH_2Cl_2/MeOH/conc. NH_3 (aq.)).

HRMS (+ESI): *m/z* found $[M+H]^+$ 597.2112, $[C_{29}H_{31}N_4OF_6S]^+$ requires 597.2117, (δ = -0.8 ppm).

(1S,2S,4S,5R)-2-((R)-(3-(3,5-bis(trifluoromethyl)phenyl)thioureido)(6-methoxyquinolin-4-yl)methyl)-1-(3,5-di-tert-butylbenzyl)-5-ethylquinuclidin-1-ium bromide (**C5•**Br)

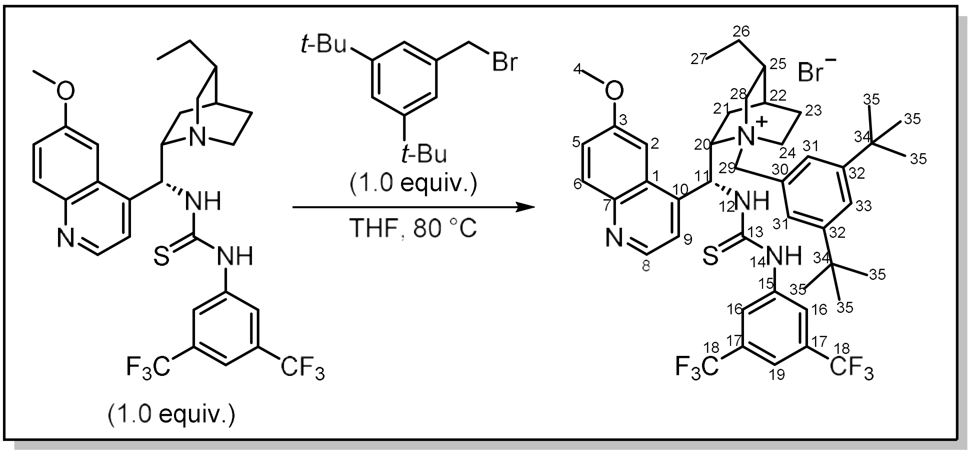

A microwave vial was charged with 1-(3,5-bis(trifluoromethyl)phenyl)-3-((*R*)-((1*S*,2*S*,4*S*,5*R*)-5-ethylquinuclidin-2-yl)(6-methoxyquinolin-4-yl)methyl)thiourea (176 mg, 0.295 mmol, 1.0 equiv.) and 3,5-di-*tert*-butylbenzyl bromide (86 mg, 0.304 mmol, 1.03 equiv.). The vial was sealed, evacuated and backfilled with nitrogen thrice, before anhydrous THF (4.5 mL, 0.067 M) was added. The reaction mixture was heated at 80 ˚C overnight. The reaction mixture was allowed to cool to room temperature and concentrated under reduced pressure. Purification of the crude residue by flash column chromatography (SiO_2, 0-30% v/v *solvent mixture A* in CH_2Cl_2, where *solvent mixture A* = 70:29:1 CH_2Cl_2/MeOH/conc. NH_3 (aq.)) afforded the title compound as a yellow solid (114 mg, 0.130 mmol, 44% yield).

^{1}H NMR (700 MHz, $CDCl_3$): δ 11.5 (br s, 1H, H-14), 10.8 (br s, 1H, H-12), 8.89 (d, J = 4.6 Hz, 1H, H-8), 8.38 (s, 2H, H-16), 8.15-8.13 (m, 2H, H-6, H-9), 7.91 (d, J = 9.9 Hz, 1H, H-11), 7.61 (br s, 1H, H-19), 7.60 (t, J = 1.7 Hz, 1H, H-33), 7.48-7.46 (m, 2H, H-2, H-5), 7.28 (d, J = 1.7 Hz, 2H, H-31), 6.02 (d, J = 12.5 Hz, 1H, H-29a), 4.47 (d, J = 12.3 Hz, 1H, H-29b), 4.26-4.22 (m, 1H, H-24a), 3.99 (s, 3H, H-4), 3.71-3.65 (m, 2H, H-20, H-28a), 3.10-3.06 (m, 2H, H-21a, H-24b), 3.04-2.98 (m, 1H, H-23a), 2.75-2.72 (m, 1H, H-28b), 2.30-2.28 (m, 1H, H-22), 1.94-1.90 (m, 1H, H-21b), 1.89-1.85 (m, 1H, H-25), 1.84-1.79 (m, 1H, H-23b), 1.36-1.31 (m, 2H, H-26a, H-26b), 1.29 (s, 18H, H-35), 0.83 (t, J = 7.3 Hz, 3H, H-27).

^{13}C NMR (176 MHz, $CDCl_3$): δ 181.9, 158.9, 153.0, 148.6, 144.8, 140.8, 140.5, 133.1, 131.7 (q, J_{C-F} = 33.5 Hz), 127.5, 126.0, 125.6, 125.3, 123.32 (q, J_{C-F} = 272.4 Hz), 123.29, 121.5,

120.8, 118.3, 102.0, 69.9, 66.8, 64.6, 56.2, 51.9, 50.8, 36.4, 35.1, 31.4, 26.5, 25.1, 24.2, 23.2, 11.3.

^{19}F NMR (376 MHz, $CDCl_3$): δ -62.9.

$[\alpha]_D^{25.0}$ = +80.4 (c. 0.61, $CHCl_3$).

R_f = 0.36 (30% v/v *solvent mixture A* in CH_2Cl_2; *solvent mixture A* = 70:29:1 CH_2Cl_2/MeOH/conc. NH_3 (aq.)).

HRMS (+ESI): *m/z* for cation found 799.3831, $[C_{44}H_{53}N_4OF_6S]^+$ requires 799.3839, (δ = -1.0 ppm).

3-((3,5-bis(trifluoromethyl)phenyl)amino)-4-methoxycyclobut-3-ene-1,2-dione

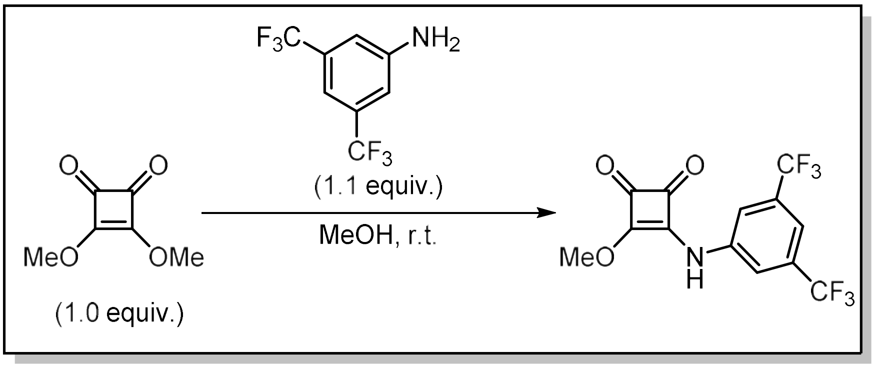

Prepared according to the protocol reported by Rawal and co-workers with modifications.[9] To a solution of 3,4-dimethoxy-3-cyclobutene-1,2-dione (1.05 g, 7.39 mmol, 1.0 equiv.) in anhydrous MeOH (10 mL, 0.74 M) was added 3,5-bis(trifluoromethyl)aniline (1.25 mL, 8.00 mmol, 1.1 equiv.). The reaction mixture was stirred at room temperature for 68 h and the white precipitate was filtered off to give the title compound (2.34 g, 6.90 mmol, 93% yield), which was used in the next step without further purifications.

3-((3,5-bis(trifluoromethyl)phenyl)amino)-4-(((R)-((1S,2S,4S,5R)-5-ethylquinuclidin-2-yl)(6-methoxyquinolin-4-yl)methyl)amino)cyclobut-3-ene-1,2-dione

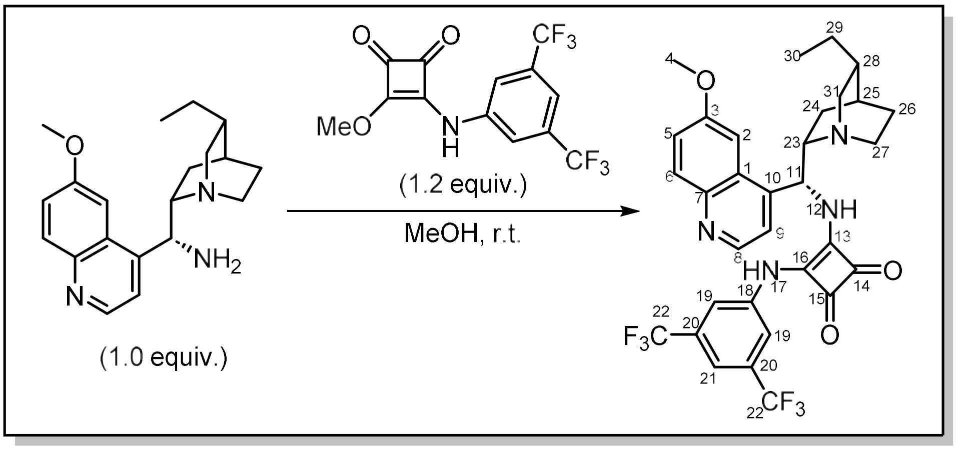

To a solution of (*R*)-((1*S*,2*S*,4*S*,5*R*)-5-ethylquinuclidin-2-yl)(6-methoxyquinolin-4-yl)methanamine (324 mg, 0.996 mmol, 1.0 equiv.) in anhydrous MeOH (5 mL, 0.2 M) was added 3-((3,5-bis(trifluoromethyl)phenyl)amino)-4-methoxycyclobut-3-ene-1,2-dione (409 mg, 1.21 mmol, 1.2 equiv.) at room temperature. The reaction mixture was allowed to stir at room temperature for 64 h, and concentrated under reduced pressure. Purification of the crude residue by flash column chromatography (SiO_2, 0-30% v/v *solvent mixture A* in CH_2Cl_2, where *solvent mixture A* = 70:29:1 CH_2Cl_2/MeOH/conc. NH_3 (aq.)) afforded the title compound as a yellow solid (426 mg, 0.673 mmol, 68% yield).

^{1}H NMR (700 MHz, CD_3OD): δ 8.74 (d, J = 4.7 Hz, 1H, H-8), 7.97-7.95 (m, 3H, H-6, H-19), 7.77 (d, J = 2.6 Hz, 1H, H-2), 7.64 (d, J = 4.7 Hz, 1H, H-9), 7.55 (s, 1H, H-21), 7.43 (dd, J = 9.2, 2.6 Hz, 1H, H-5), 6.39 (d, J = 10.6 Hz, 1H, H-11), 3.98 (s, 3H, H-4), 3.70-3.66 (m, 1H, H-23), 3.01-2.98 (m, 2H, H-27a, H-31a), 2.55-2.51 (m, 1H, H-27b), 2.44-2.42 (m, 1H, H-31b), 2.18-2.15 (m, 1H, H-24a), 1.89-1.85 (m, 2H, H-25, H-26a), 1.57-1.46 (m, 5H, H-24b, H-26b, H-28, H-29a, H-29b), 0.92 (t, J = 7.3 Hz, 3H, H-30).

Note: The signals of H-12 (-NH) and H-17 (-NH) were not observed.

^{13}C NMR (176 MHz, CD_3OD): δ 186.3, 182.2, 170.1, 164.7, 160.4, 148.6, 146.2, 145.6, 142.1, 133.8 (q, $J_{C\text{-}F}$ = 33.4 Hz), 131.7, 129.2, 124.6 (q, $J_{C\text{-}F}$ = 272.1 Hz), 124.2, 120.1, 119.6, 116.9, 101.6, 60.1, 58.3, 56.6, 56.1, 42.8, 38.2, 28.8, 28.5, 27.0, 26.6, 12.4.

^{19}F NMR (376 MHz, CD_3OD): δ -64.7.

$[\alpha]_D^{25.0}$ = +3.4 (c. 0.64, MeOH).

R_f = 0.47 (30% v/v *solvent mixture A* in CH_2Cl_2; *solvent mixture A* = 70:29:1 CH_2Cl_2/MeOH/conc. NH_3 (aq.)).

HRMS (+ESI): *m/z* found $[M+H]^+$ 633.2312, $[C_{32}H_{31}N_4O_3F_6]^+$ requires 633.2295, (δ = +2.7 ppm).

(1S,2S,4S,5R)-2-((R)-((2-((3,5-bis(trifluoromethyl)phenyl)amino)-3,4-dioxocyclobut-1-en-1-yl)amino)(6-methoxyquinolin-4-yl)methyl)-1-(3,5-di-tert-butylbenzyl)-5-ethylquinuclidin-1-ium bromide (**C6•**Br)

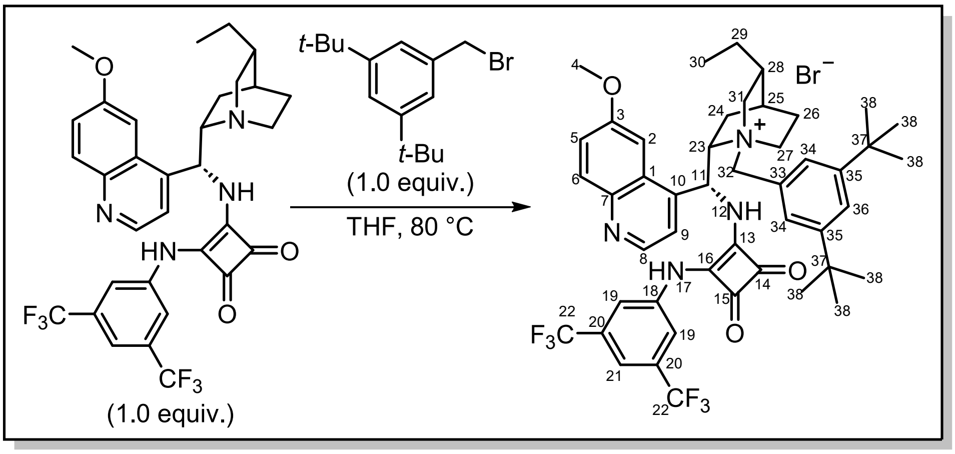

A microwave vial was charged with 3-((3,5-bis(trifluoromethyl)phenyl)amino)-4-(((*R*)-((1*S*,2*S*,4*S*,5*R*)-5-ethylquinuclidin-2-yl)(6-methoxyquinolin-4-yl)methyl)amino)cyclobut-3-ene-1,2-dione (183 mg, 0.289 mmol, 1.0 equiv.) and 3,5-di-*tert*-butylbenzyl bromide (86 mg, 0.304 mmol, 1.05 equiv.). The vial was sealed, evacuated and backfilled with nitrogen thrice, before anhydrous THF (4.5 mL, 0.067 M) was added. The reaction mixture was heated at 80 ˚C overnight. The reaction mixture was cooled to room temperature and concentrated under reduced pressure. Purification of the crude residue by flash column chromatography (SiO_2, 0-30% v/v *solvent mixture A* in CH_2Cl_2, where *solvent mixture A* = 70:29:1 CH_2Cl_2/MeOH/conc. NH_3 (aq.)) afforded the title compound as a red solid (129 mg, 0.141 mmol, 49% yield).

^{1}H NMR (700 MHz, $CDCl_3$): δ 11.5 (br s, 1H, H-17), 10.3 (br s, 1H, H-12), 8.81 (d, J = 4.6 Hz, 1H, H-8), 8.26 (s, 2H, H-19), 8.07 (d, J = 9.2 Hz, 1H, H-6), 7.95 (d, J = 4.4 Hz, 1H, H-9), 7.55 (t, J = 1.6 Hz, 1H, H-36), 7.51 (br s, 1H, H-21), 7.44-7.37 (m, 3H, H-2, H-5, H-11), 7.12 (s, 2H, H-34), 5.28 (d, J = 12.5 Hz, 1H, H-32a), 4.72 (d, J = 12.8 Hz, 1H, H-32b), 3.92-3.88 (m, 1H, H-27a), 3.86 (s, 3H, H-4), 3.81-3.79 (m, 1H, H-23), 3.63 (dd, J = 12.6, 10.4 Hz, 1H, H-31a), 3.31-3.27 (m, 2H, H-24a, H-27b), 2.89-2.85 (m, 1H, H-26a), 2.75-2.71 (m, 1H, H-31b), 2.36 (br s, 1H, H-25), 2.14-2.10 (m, 1H, H-24b), 1.93-1.87 (m, 2H, H-26b, H-28), 1.39-1.33 (m, 2H, H-29a, H-29b), 1.20 (s, 18H, H-38), 0.83 (t, J = 7.3 Hz, 3H, H-30).

^{13}C NMR (176 MHz, $CDCl_3$): δ 184.9, 180.3, 167.6, 167.3, 159.4, 153.1, 148.2, 145.0, 140.3, 133.0, 132.7 (q, $J_{C\text{-}F}$ = 33.5 Hz), 127.3, 125.7, 125.5, 124.8, 123.2 (q, $J_{C\text{-}F}$ = 272.6 Hz), 121.7, 120.6, 119.0, 116.8, 100.7, 69.1, 66.7, 64.4, 56.2, 51.5, 36.5, 35.0, 31.3, 26.1, 25.0, 24.5, 22.2, 11.2.

^{19}F NMR (376 MHz, $CDCl_3$): δ -63.9.

[α]$_D^{25.0}$ = +70.8 (c. 0.21, $CHCl_3$).

R_f = 0.58 (30% v/v *solvent mixture A* in CH_2Cl_2; *solvent mixture A* = 70:29:1 CH_2Cl_2/MeOH/conc. NH_3 (aq.)).

HRMS (+ESI): *m/z* for cation found 835.4027, $[C_{47}H_{53}N_4O_3F_6]^+$ requires 835.4016, (δ = +1.3 ppm).

1-cyclohexyl-3-((R)-((1S,2S,4S,5R)-5-ethylquinuclidin-2-yl)(6-methoxyquinolin-4-yl)methyl)urea

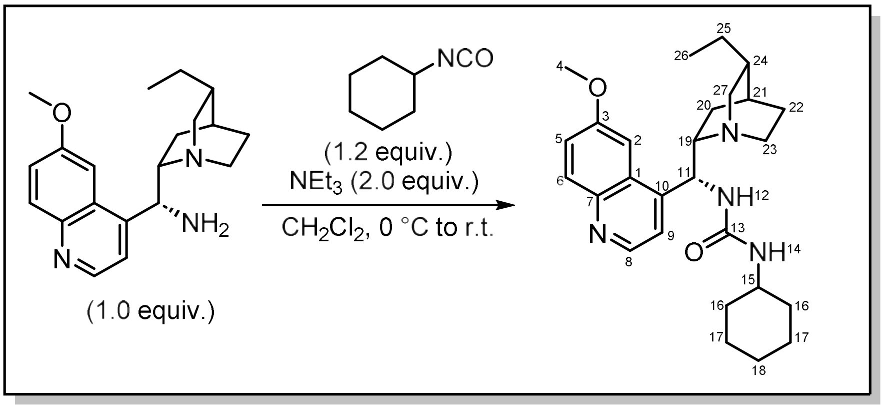

To a solution of (*R*)-((1*S*,2*S*,4*S*,5*R*)-5-ethylquinuclidin-2-yl)(6-methoxyquinolin-4-yl)methanamine (651 mg, 2.00 mmol, 1.0 equiv.) and triethylamine (0.56 mL, 4.00 mmol, 2.0 equiv.) in anhydrous CH_2Cl_2 (20 mL, 0.1 M) at 0 ˚C was added cyclohexyl isocyanate (0.31 mL, 2.40 mmol, 1.2 equiv.). The reaction mixture was allowed to warm to room temperature overnight and quenched with aqueous saturated $NaHCO_3$. The phases were separated and the aqueous layer was extracted with CH_2Cl_2. The combined organic layers were dried over $MgSO_4$ and the solvent removed under reduced pressure. The crude residue was purified by flash column chromatography (SiO_2, 0-25% v/v *solvent mixture A* in CH_2Cl_2, where *solvent mixture A* = 70:29:1 CH_2Cl_2/MeOH/conc. NH_3 (aq.)) to afford the title compound as a white solid (823 mg, 1.83 mmol, 91% yield).

^{1}H NMR (700 MHz, $CDCl_3$): δ 8.58 (d, *J* = 4.6 Hz, 1H, H-8), 7.89 (d, *J* = 9.1 Hz, 1H, H-6), 7.67 (br s, 1H, H-2), 7.28-7.25 (m, 2H, H-5, H-9), 5.77 (br s, 1H, H-11), 4.79 (br s, 1H, H-12), 4.43 (br s, 1H, H-14), 3.94 (s, 3H, H-4), 3.48-3.41 (m, 1H, H-15), 3.30-3.26 (m, 1H, H-19), 3.05 (dd, *J* = 13.1, 9.4 Hz, 1H, H-27a), 2.90 (br s, 1H, H-23a), 2.50-2.47 (m, 1H, H-23b), 2.42 (d, *J* = 13.2 Hz, 1H, H-27b), 1.92-1.89 (m, 1H, H-20a), 1.85 (d, *J* = 10.8 Hz, 1H, H-16a), 1.82-1.75 (m, 3H, H-16b, H-21, H-22a), 1.64-1.60 (m, 3H, H-17a, H-17b, H-20b), 1.57-1.53 (m, 1H, H-18a), 1.44-1.38 (m, 4H, H-22b, H-24, H-25a, H-25b), 1.33-1.23 (m, 2H, H-17c, H-17d), 1.09-0.94 (m, 3H, H-16c, H-16d, H-18b), 0.90 (t, *J* = 7.1 Hz, 3H, H-26).

^{13}C NMR (176 MHz, CDCl$_3$): δ 158.2, 157.1, 147.6, 146.0, 144.8, 131.4, 128.5, 122.1, 118.8, 101.9, 58.4, 57.7, 55.9, 50.3, 49.5, 41.8, 37.4, 33.91, 33.85, 28.2, 27.8, 25.6, 25.5, 24.99, 24.98, 12.3.

$[\alpha]_D^{25.0}$ = +18.4 (c. 0.93, CHCl$_3$).

R$_f$ = 0.40 (30% v/v *solvent mixture A* in CH$_2$Cl$_2$; *solvent mixture A* = 70:29:1 CH$_2$Cl$_2$/MeOH/conc. NH$_3$ (aq.)).

HRMS (+ESI): *m/z* found [M+H]$^+$ 451.3065, [C$_{27}$H$_{39}$N$_4$O$_2$]$^+$ requires 451.3068, (δ = -0.7 ppm).

(1S,2S,4S,5R)-2-((R)-(3-cyclohexylureido)(6-methoxyquinolin-4-yl)methyl)-5-ethyl-1-((3,3'',5,5''-tetra-tert-butyl-[1,1':3',1''-terphenyl]-5'-yl)methyl)quinuclidin-1-ium bromide (**C7**•Br)

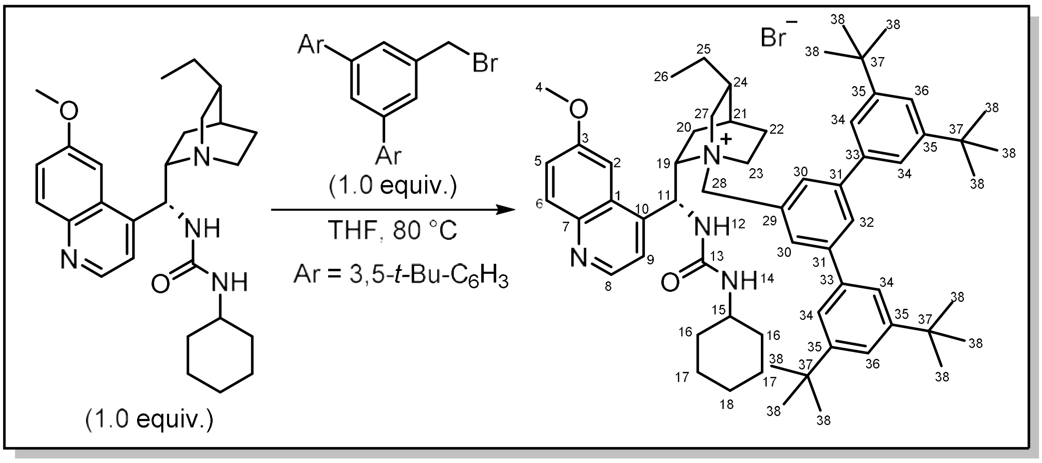

A microwave vial was charged with 1-cyclohexyl-3-((*R*)-((1*S*,2*S*,4*S*,5*R*)-5-ethylquinuclidin-2-yl)(6-methoxyquinolin-4-yl)methyl)urea (451 mg, 1.00 mmol, 1.0 equiv.) and 5'-(bromomethyl)-3,3'',5,5''-tetra-tert-butyl-1,1':3',1''-terphenyl (548 mg, 1.00 mmol, 1.0 equiv.). The vial was sealed, evacuated and backfilled with nitrogen thrice, before anhydrous THF (15 mL, 0.067 M) was added. The reaction mixture was heated at 80 ˚C overnight. The reaction mixture was allowed to cool to room temperature and concentrated under reduced pressure. The crude residue was reconstituted in CH$_2$Cl$_2$ and the organic layer was washed with water twice. The organic layer was dried over MgSO$_4$ and the solvent removed under reduced pressure. Purification of the crude residue by flash column chromatography (SiO$_2$, 0-8% v/v *solvent mixture A* in CH$_2$Cl$_2$, where *solvent mixture A* = 70:29:1 CH$_2$Cl$_2$/MeOH/conc. NH$_3$ (aq.)) afforded the title compound as an orange solid (688 mg, 0.689 mmol, 69% yield).

^{1}H NMR (700 MHz, CD_3OD): δ 8.61 (d, J = 4.7 Hz, 1H, H-8), 7.88 (d, J = 9.2 Hz, 1H, H-6), 7.71 (t, J = 1.4 Hz, 1H, H-32), 7.62 (d, J = 1.2 Hz, 2H, H-30), 7.57 (d, J = 2.6 Hz, 1H, H-2), 7.47-7.45 (m, 3H, H-9, H-36), 7.40 (dd, J = 9.2, 2.6 Hz, 1H, H-5), 7.32 (d, J = 1.6 Hz, 4H, H-34), 6.79 (s, 1H, H-11), 5.26 (d, J = 13.1 Hz, 1H, H-28a), 5.00 (d, J = 13.2 Hz, 1H, H-28b), 4.20 (dd, J = 11.4, 5.1 Hz, 1H, H-19), 4.15-4.10 (m, 1H, H-23a), 4.02 (dd, J = 12.2, 11.0 Hz, 1H, H-27a), 3.99 (s, 3H, H-4), 3.67-3.63 (m, 1H, H-15), 3.53-3.49 (m, 1H, H-23b), 3.34-3.32 (m, 1H, H-27b), 2.45 (dd, J = 13.3, 10.8 Hz, 1H, H-20a), 2.28-2.24 (m, 2H, H-20b, H-22a), 2.17 (br s, 1H, H-21), 2.11-2.06 (m, 1H, H-24), 1.98 (dd, J = 14.4, 12.0 Hz, 1H, H-22b), 1.91-1.88 (m, 1H, H-16a), 1.84-1.82 (m, 1H, H-16b), 1.72-1.68 (m, 2H, H-17a, H-17b), 1.60-1.51 (m, 3H, H-18a, H-25a, H-25b), 1.34 (s, 38H, H-17c, H-17d, H-38), 1.24-1.18 (m, 3H, H-16c, H-16d, H-18b), 0.96 (t, J = 7.3 Hz, 3H, H-26).

Note: The signals of H-12 (-NH) and H-14 (-NH) were not observed.

^{13}C NMR (176 MHz, CD_3OD): δ 160.3, 159.1, 152.6, 148.1, 145.7, 145.3, 144.5, 140.8, 132.0, 131.5, 129.7, 129.3, 128.3, 123.8, 123.2, 122.5, 120.6, 102.7, 68.5, 66.1, 65.6, 56.5, 53.0, 50.4, 48.6, 37.5, 35.8, 34.9, 34.3, 31.9, 26.64, 26.57, 26.5, 26.2, 25.9, 24.2, 11.7.

$[\alpha]_D^{25.0}$ = -55.7 (c. 1.06, $CHCl_3$).

R_f = 0.36 (30% v/v *solvent mixture A* in CH_2Cl_2; *solvent mixture A* = 70:29:1 CH_2Cl_2/MeOH/conc. NH_3 (aq.)).

HRMS (+ESI): *m/z* for cation found 917.6636, $[C_{62}H_{85}N_4O_2]^+$ requires 917.6667, (δ = -3.4 ppm).

(S)-((1S,2S,4S,5R)-5-ethylquinuclidin-2-yl)(6-methoxyquinolin-4-yl)methanamine

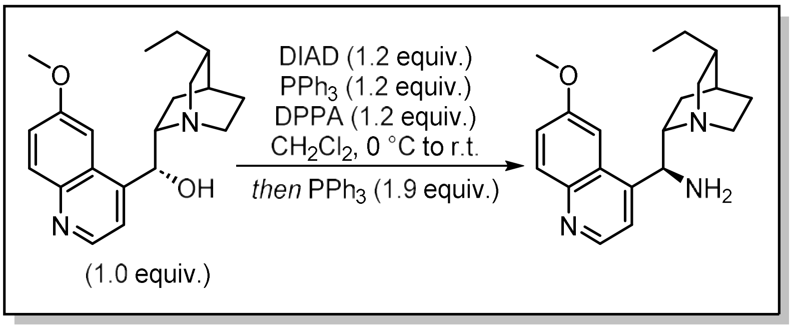

To a solution of dihydroquinine (5.04 g, 15.4 mmol, 1.0 equiv.) and triphenylphosphine (4.95 g, 18.9 mmol, 1.2 equiv.) in CH_2Cl_2 (60 mL, 0.26 M) at 0 ˚C, was added diisopropyl azodicarboxylate (3.7 mL, 18.8 mmol, 1.2 equiv.) dropwise over 10 min. The reaction mixture was allowed to stir at 0 ˚C for 10 min before the dropwise addition of diphenyl phosphoryl azide (4 mL, 18.5 mmol, 1.2 equiv.) over 20 min. The reaction mixture was warmed to room

temperature and stirred for 4 h at this temperature. Distilled water (13.5 mL) was added, followed by a dropwise addition of a solution of triphenylphosphine (7.66 g, 29.2 mmol, 1.9 equiv.) in CH_2Cl_2 (15 mL, 1.95 M) at room temperature. The reaction mixture was allowed to stir at room temperature for 17 h and quenched with aqueous 3 M HCl (24 mL). The phases were separated and the aqueous phase was washed with CH_2Cl_2 (26 mL x 2). The aqueous phase was basified with concentrated aqueous ammonia solution (35%, 6.3 mL) and extracted with CH_2Cl_2 (26 mL x 3). The combined organic layers were concentrated under reduced pressure to afford the title compound as a yellow oil (4.92 g), which was taken forward without further purifications.

1-(3,5-bis(trifluoromethyl)phenyl)-3-((S)-((1S,2S,4S,5R)-5-ethylquinuclidin-2-yl)(6-methoxyquinolin-4-yl)methyl)urea

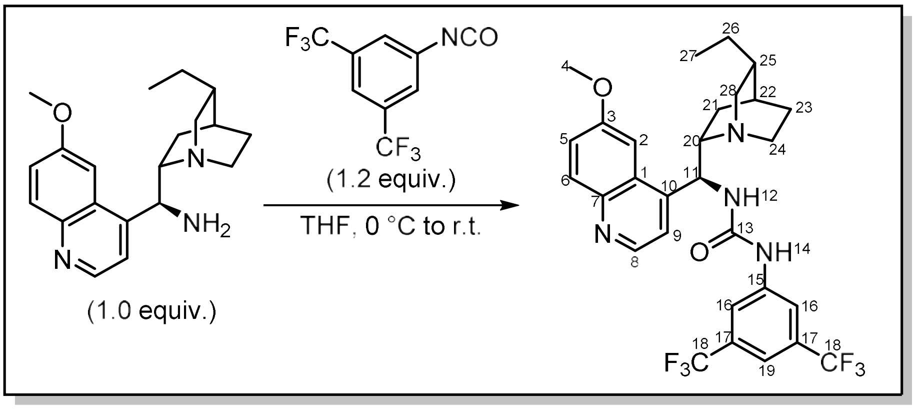

To a solution of (*S*)-((1*S*,2*S*,4*S*,5*R*)-5-ethylquinuclidin-2-yl)(6-methoxyquinolin-4-yl)methanamine (700 mg, 2.15 mmol, 1.0 equiv.) in anhydrous THF (7 mL, 0.31 M) at 0 ˚C was added 3,5-bis(trifluoromethyl)phenyl isocyanate (0.41 mL, 2.40 mmol, 1.12 equiv.). The reaction mixture was allowed to warm to room temperature overnight and concentrated under reduced pressure. The crude residue was purified by flash column chromatography (SiO_2, 0-35% v/v *solvent mixture A* in CH_2Cl_2, where *solvent mixture A* = 70:29:1 CH_2Cl_2/MeOH/conc. NH_3 (aq.)) to afford the title compound as a white solid (945 mg, 1.63 mmol, 76% yield).

^{1}H NMR (700 MHz, $CDCl_3$): δ 8.83 (d, *J* = 4.5 Hz, 1H, H-8), 8.11 (br s, 1H, H-14), 8.06 (d, *J* = 9.1 Hz, 1H, H-6), 7.76 (s, 1H, H-2), 7.68 (s, 2H, H-16), 7.43 (dd, *J* = 9.2, 2.7 Hz, 1H, H-5), 7.34 (d, *J* = 4.5 Hz, 1H, H-9), 7.29 (s, 1H, H-19), 6.24 (s, 1H, H-12), 5.68 (br s, 1H, H-11), 4.02 (s, 3H, H-4), 3.60 (br s, 1H, H-24a), 3.24 (br s, 1H, H-20), 2.94-2.85 (m, 1H, H-28a), 2.69-2.65 (m, 1H, H-24b), 1.88 (br s, 1H, H-28b), 1.72-1.68 (m, 3H, H-21a, H-22, H-23a), 1.56-1.52 (m, 1H, H-23b), 1.43-1.35 (m, 1H, H-25), 1.26-1.19 (m, 1H, H-26a), 1.16-1.10 (m, 1H, H-26b), 0.92 (dd, *J* = 12.7, 6.3 Hz, 1H, H-21b), 0.71 (t, *J* = 7.4 Hz, 3H, H-27).

^{13}C NMR (176 MHz, CDCl$_3$): *δ* 158.7, 154.8, 147.3, 145.2, 144.1, 140.6, 132.00, 131.96 (q, $J_{C\text{-}F}$ = 33.2 Hz), 128.7, 123.2 (q, $J_{C\text{-}F}$ = 272.4 Hz), 122.8, 118.3, 118.0, 115.6, 101.9, 59.8, 57.3, 56.0, 50.5, 41.8, 36.6, 27.9, 27.6, 27.0, 24.9, 12.0.

^{19}F NMR (376 MHz, CDCl$_3$): *δ* -64.2.

R$_f$ = 0.44 (30% v/v *solvent mixture A* in CH_2Cl_2; *solvent mixture A* = 70:29:1 CH_2Cl_2/MeOH/conc. NH_3 (aq.)).

The spectroscopic data is in agreement with that reported in the literature.[10]

(1S,2S,4S,5R)-2-((S)-(3-(3,5-bis(trifluoromethyl)phenyl)ureido)(6-methoxyquinolin-4-yl)methyl)-1-(3,5-di-tert-butylbenzyl)-5-ethylquinuclidin-1-ium bromide (***epi*-C4**•Br)

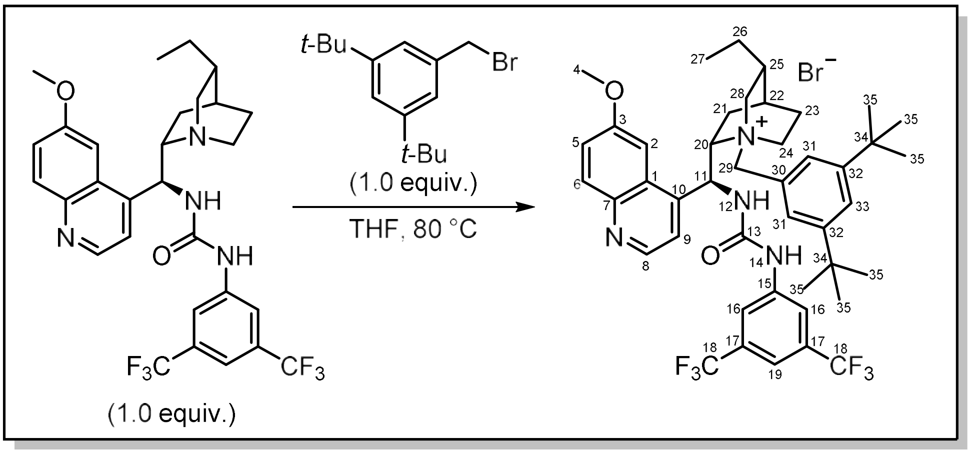

A microwave vial was charged with 1-(3,5-bis(trifluoromethyl)phenyl)-3-((*S*)-((1*S*,2*S*,4*S*,5*R*)-5-ethylquinuclidin-2-yl)(6-methoxyquinolin-4-yl)methyl)urea (361 mg, 0.62 mmol, 1.0 equiv.) and 3,5-di-*tert*-butylbenzyl bromide (182 mg, 0.64 mmol, 1.03 equiv.). The vial was sealed, evacuated and backfilled with nitrogen thrice, before anhydrous THF (9 mL, 0.067 M) was added. The reaction mixture was heated at 80 ˚C overnight. The reaction mixture was allowed to cool to room temperature and concentrated under reduced pressure. Purification of the crude residue by flash column chromatography (SiO_2, 0-10% v/v MeOH in EtOAc) afforded the title compound as a yellow solid (409 mg, 0.473 mmol, 76% yield).

^{1}H NMR (700 MHz, CD$_3$OD): *δ* 8.81 (d, *J* = 3.7 Hz, 1H, H-8), 8.11 (s, 2H, H-16), 8.01 (d, *J* = 9.1 Hz, 1H, H-6), 7.84 (d, *J* = 3.8 Hz, 1H, H-9), 7.76 (s, 1H, H-2), 7.56 (s, 1H, H-33), 7.54 (s, 1H, H-19), 7.49 (dd, *J* = 9.2, 2.5 Hz, 1H, H-5), 7.36 (s, 2H, H-31), 6.46 (d, *J* = 10.3 Hz, 1H, H-11), 5.36 (d, *J* = 12.9 Hz, 1H, H-29a), 5.07-5.04 (m, 1H, H-20), 4.78 (d, *J* = 12.6 Hz, 1H, H-29b), 4.50-4.46 (m, 1H, H-24a), 4.03 (s, 3H, H-4), 3.87 (t, *J* = 11.0 Hz, 1H, H-28a), 3.42 (dd, *J* = 11.8, 8.8 Hz, 1H, H-28b), 3.23-3.18 (m, 1H, H-24b), 2.28 (dd, *J* = 11.7, 11.1 Hz, 1H, H-21a), 2.24-2.19 (m, 1H, H-23a), 2.03-1.99 (m, 1H, H-23b), 1.94-1.88 (m, 2H, H-

22, H-25), 1.59-1.54 (m, 1H, H-26a), 1.49-1.43 (m, 1H, H-26b), 1.28 (s, 18H, H-35), 1.25-1.18 (m, 1H, H-21b), 0.89 (t, J = 7.5 Hz, 3H, H-27).

Note: The signals of H-12 (-NH) and H-14 (-NH) were not observed.

^{13}C NMR (176 MHz, CD_3OD): δ 160.7, 156.2, 153.4, 148.6, 145.7, 145.0, 142.5, 133.3, (q, $J_{C\text{-}F}$ = 33.5 Hz), 132.1, 129.01, 128.85, 128.1, 125.6, 124.7 (q, $J_{C\text{-}F}$ = 271.3 Hz), 124.3, 120.8, 119.4, 116.6, 102.3, 68.9, 68.0, 64.0, 56.4, 51.9, 50.4, 36.4, 35.8, 31.6, 28.6, 26.3, 26.1, 25.8, 11.4.

^{19}F NMR (376 MHz, CD_3OD): δ -65.6.

$[\alpha]_D^{25.0}$ = +3.9 (c. 0.44, $CHCl_3$).

R_f = 0.48 (10% v/v MeOH in EtOAc).

HRMS (+ESI): *m/z* for cation found 783.4086, $[C_{44}H_{53}N_4O_2F_6]^+$ requires 783.4067, (δ = +2.4 ppm).

1-cyclohexyl-3-((S)-((1S,2S,4S,5R)-5-ethylquinuclidin-2-yl)(6-methoxyquinolin-4-yl)methyl)urea

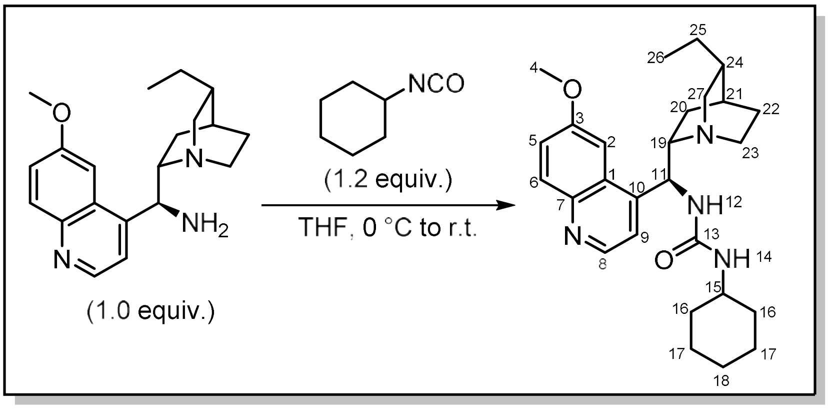

To a solution of (S)-((1S,2S,4S,5R)-5-ethylquinuclidin-2-yl)(6-methoxyquinolin-4-yl)methanamine (700 mg, 2.15 mmol, 1.0 equiv.) in anhydrous THF (7 mL, 0.31 M) at 0 ˚C was added cyclohexyl isocyanate (0.31 mL, 2.40 mmol, 1.12 equiv.). The reaction mixture was allowed to warm to room temperature overnight and concentrated under reduced pressure. The crude residue was purified by flash column chromatography (SiO_2, 0-40% v/v *solvent mixture A* in CH_2Cl_2, where *solvent mixture A* = 70:29:1 CH_2Cl_2/MeOH/conc. NH_3 (aq.)) to afford the title compound as a white solid (728 mg, 1.62 mmol, 75% yield).

^{1}H NMR (700 MHz, $CDCl_3$): δ 8.73 (d, J = 4.5 Hz, 1H, H-8), 8.01 (d, J = 9.1 Hz, 1H, H-6), 7.72 (d, J = 2.4 Hz, 1H, H-2), 7.40 (d, J = 4.4 Hz, 1H, H-9), 7.37 (dd, J = 9.2, 2.7 Hz, 1H, H-5), 5.81 (s, 1H, H-12), 5.15 (br s, 1H, H-11), 4.45 (d, J = 7.4 Hz, 1H, H-14), 3.97 (s, 3H, H-

4), 3.44-3.40 (m, 1H, H-15), 3.18-3.15 (m, 2H, H-23a, H-27a), 2.99 (br s, 1H, H-19), 2.71-2.66 (m, 1H, H-23b), 2.32 (dd, *J* = 13.6, 2.6 Hz, 1H, H-27b), 1.79 (d, *J* = 10.0 Hz, 2H, H-16a, H-16b), 1.64-1.60 (m, 2H, H-21, H-22a), 1.56-1.50 (m, 4H, H-17a, H-17b, H-18a, H-22b), 1.44-1.40 (m, 1H, H-24), 1.31-1.28 (m, 1H, H-20a), 1.26-1.16 (m, 4H, H-17c, H-17d, H-25a, H-25b), 1.08-1.03 (m, 1H, H-18b), 0.98-0.90 (m, 3H, H-16c, H-16d, H-20b), 0.77 (t, *J* = 7.4 Hz, 3H, H-26).

^{13}C NMR (176 MHz, $CDCl_3$): δ 157.9, 157.6, 147.7, 146.4, 145.0, 131.9, 128.5, 121.7, 120.0, 102.0, 60.4, 57.7, 55.8, 52.7, 49.2, 41.1, 37.3, 34.0, 33.8, 28.6, 27.5, 25.9, 25.6, 25.2, 25.0, 24.9, 12.1.

$[\alpha]_D^{25.0}$ = -52.7 (c. 0.56, $CHCl_3$).

R_f = 0.39 (30% v/v *solvent mixture A* in CH_2Cl_2; *solvent mixture A* = 70:29:1 CH_2Cl_2/MeOH/conc. NH_3 (aq.)).

HRMS (+ESI): *m/z* found $[M+H]^+$ 451.3050, $[C_{27}H_{39}N_4O_2]^+$ requires 451.3068, (δ = -4.0 ppm).

(1S,2S,4S,5R)-2-((S)-(3-cyclohexylureido)(6-methoxyquinolin-4-yl)methyl)-5-ethyl-1-((3,3'',5,5''-tetra-tert-butyl-[1,1':3',1''-terphenyl]-5'-yl)methyl)quinuclidin-1-ium bromide (***epi*-C7**•Br)

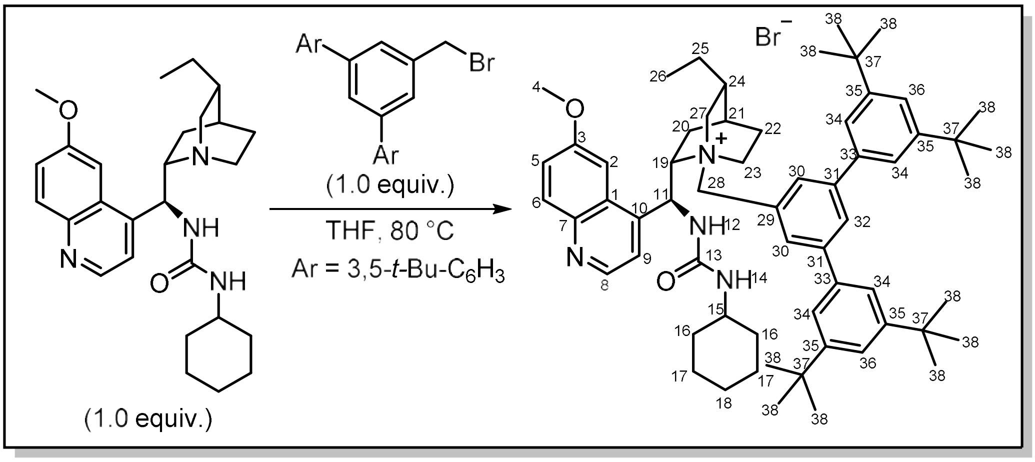

A microwave vial was charged with 1-cyclohexyl-3-((*S*)-((1*S*,2*S*,4*S*,5*R*)-5-ethylquinuclidin-2-yl)(6-methoxyquinolin-4-yl)methyl)urea (273 mg, 0.61 mmol, 1.0 equiv.) and 5'-(bromomethyl)-3,3'',5,5''-tetra-tert-butyl-1,1':3',1''-terphenyl (338 mg, 0.62 mmol, 1.01 equiv.). The vial was sealed, evacuated and backfilled with nitrogen thrice, before anhydrous THF (9 mL, 0.067 M) was added. The reaction mixture was heated at 80 ˚C overnight. The reaction mixture was allowed to cool to room temperature and concentrated under reduced pressure. Purification of the crude residue by flash column chromatography (SiO_2, 0-10%

v/v MeOH in EtOAc) afforded the title compound as a white solid (493 mg, 0.493 mmol, 81% yield).

^{1}H NMR (700 MHz, CD_3OD): δ 8.78 (d, J = 4.6 Hz, 1H, H-8), 8.01 (d, J = 9.2 Hz, 1H, H-6), 7.85 (s, 1H, H-32), 7.76 (s, 2H, H-30), 7.74 (br s, 1H, H-2), 7.72 (d, J = 5.0 Hz, 1H, H-9), 7.55 (t, J = 1.7 Hz, 2H, H-36), 7.52-7.49 (m, 5H, H-5, H-34), 6.38 (d, J = 10.7 Hz, 1H, H-11), 5.43 (d, J = 13.7 Hz, 1H, H-28a), 4.95-4.91 (m, 2H, H-19, H-28b), 4.72 (t, J = 12.2 Hz, 1H, H-23a), 4.05 (s, 3H, H-4), 4.01 (dd, J = 12.2, 10.7 Hz, 1H, H-27a), 3.55-3.51 (m, 1H, H-15), 3.48-3.40 (m, 2H, H-23b, H-27b), 2.27 (dd, J = 12.6, 11.9 Hz, 1H, H-20a), 2.22-2.18 (m, 1H, H-22a), 2.04 (dd, J = 11.5, 10.0 Hz, 1H, H-22b), 1.98-1.93 (m, 1H, H-24), 1.93-1.87 (m, 1H, H-21), 1.70-1.68 (m, 1H, H-16a), 1.62-1.53 (m, 3H, H-16b, H-17a, H-25a), 1.47-1.38 (m, 38H, H-18a, H-25b, H-38), 1.37-1.32 (m, 1H, H-17b), 1.24-1.19 (m, 2H, H-17c, H-20b), 1.04-0.95 (m, 4H, H-16c, H-16d, H-17d, H-18b), 0.90 (t, J = 7.4 Hz, 3H, H-26).

Note: The signals of H-12 (-NH) and H-14 (-NH) were not observed.

^{13}C NMR (176 MHz, CD_3OD): δ 160.7, 158.2, 152.8, 148.6, 145.8, 145.7, 145.6, 141.0, 132.0, 131.9, 130.01, 129.55, 128.9, 124.3, 123.3, 122.9, 120.3, 102.5, 69.4, 67.2, 64.5, 56.5, 52.0, 50.9, 50.1, 36.4, 35.9, 34.6, 34.3, 32.0, 28.6, 26.4, 26.3, 26.2, 25.81, 25.75, 25.7, 11.5.

$[\alpha]_D^{25.0}$ = -42.6 (c. 0.59, $CHCl_3$).

R_f = 0.61 (10% v/v MeOH in EtOAc).

HRMS (+ESI): *m/z* for cation found 917.6636, $[C_{62}H_{85}N_4O_2]^+$ requires 917.6667, (δ = -3.4 ppm).

N-((R)-((1S,2S,4S,5R)-5-ethylquinuclidin-2-yl)(6-methoxyquinolin-4-yl)methyl)acetamide

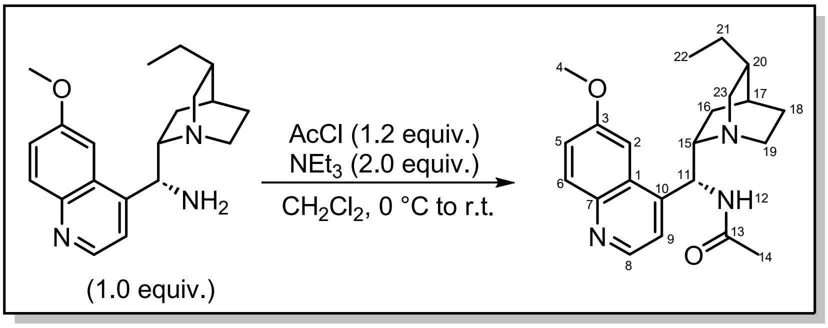

To a solution of (*R*)-((1*S*,2*S*,4*S*,5*R*)-5-ethylquinuclidin-2-yl)(6-methoxyquinolin-4-yl)methanamine (651 mg, 2.00 mmol, 1.0 equiv.) in CH_2Cl_2 (20 mL, 0.1 M), was added acetyl chloride (0.39 mL, 2.40 mmol, 1.2 equiv.) and NEt_3 (0.56 mL, 4.00 mmol, 2.0 equiv.) at 0 °C. The reaction mixture was allowed to stir at room temperature overnight and was quenched

with aqueous saturated $NaHCO_3$ solution. The phases were separated and the aqueous phase was extracted with CH_2Cl_2. The combined organic layers were dried over $MgSO_4$ and the solvent removed under reduced pressure. Purification of the crude residue by flash column chromatography (SiO_2, 0-25% v/v *solvent mixture A* in CH_2Cl_2, where *solvent mixture A* = 70:29:1 CH_2Cl_2/MeOH/conc. NH_3 (aq.)) afforded the title compound as a light yellow solid (408 mg, 1.11 mmol, 56% yield).

^{1}H NMR (700 MHz, $CDCl_3$): δ 8.68 (d, J = 4.5 Hz, 1H, H-8), 7.95 (d, J = 9.1 Hz, 1H, H-6), 7.61 (d, J = 2.6 Hz, 1H, H-2), 7.34-7.32 (m, 2H, H-5, H-9), 5.96 (t, J = 10.1 Hz, 1H, H-11), 5.86 (br s, 1H, H-12), 3.96 (s, 3H, H-4), 3.40-3.36 (m, 1H, H-15), 3.10 (dd, J = 13.4, 9.2 Hz, 1H, H-23a), 2.88-2.84 (m, 1H, H-19a), 2.54-2.50 (m, 1H, H-19b), 2.47 (d, J = 13.5 Hz, 1H, H-23b), 1.99-1.95 (m, 1H, H-16a), 1.94 (s, 3H, H-14), 1.82 (br s, 1H, H-17), 1.76-1.71 (m, 1H, H-18a), 1.51-1.41 (m, 5H, H-16b, H-18b, H-20, H-21a, H-21b), 0.93 (t, J = 7.2 Hz, 3H, H-22).

^{13}C NMR (176 MHz, $CDCl_3$): δ 169.8, 158.3, 147.7, 145.0, 144.5, 131.6, 128.5, 122.3, 119.3, 101.7, 57.8, 57.7, 56.0, 49.6, 41.7, 37.4, 28.2, 27.9, 25.4, 25.2, 23.5, 12.3.

$[\alpha]_D^{25.0}$ = -44.9 (c. 0.70, $CHCl_3$).

R_f = 0.44 (30% v/v *solvent mixture A* in CH_2Cl_2; *solvent mixture A* = 70:29:1 CH_2Cl_2/MeOH/conc. NH_3 (aq.)).

HRMS (+ESI): *m/z* found $[M+H]^+$ 368.2316, $[C_{22}H_{30}N_3O_2]^+$ requires 368.2333, (δ = -4.6 ppm).

(1S,2S,4S,5R)-2-((R)-acetamido(6-methoxyquinolin-4-yl)methyl)-5-ethyl-1-((3,3'',5,5''-tetra-tert-butyl-[1,1':3',1''-terphenyl]-5'-yl)methyl)quinuclidin-1-ium bromide (**C8**•Br)

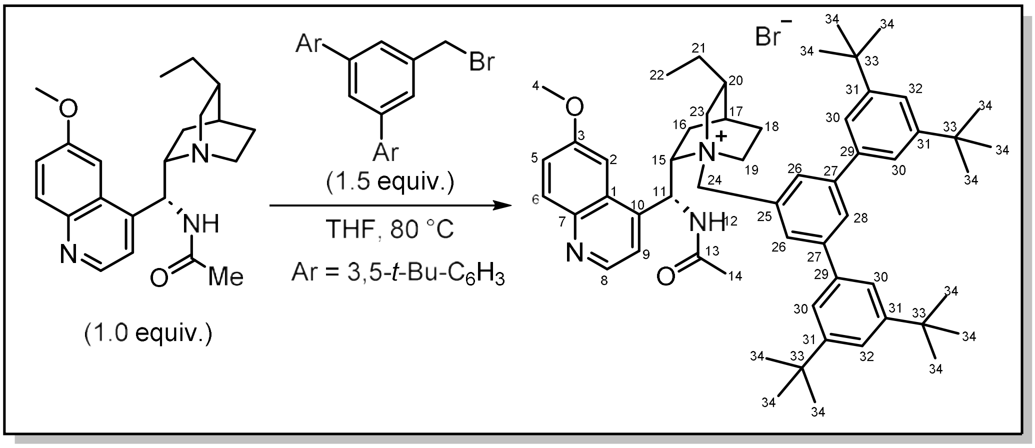

A microwave vial was charged with *N*-((*R*)-((1*S*,2*S*,4*S*,5*R*)-5-ethylquinuclidin-2-yl)(6-methoxyquinolin-4-yl)methyl)acetamide (367 mg, 1.00 mmol, 1.0 equiv.) and 5'-(bromomethyl)-3,3'',5,5''-tetra-tert-butyl-1,1':3',1''-terphenyl (822 mg, 1.50 mmol, 1.5 equiv.). The vial was sealed, evacuated and backfilled with nitrogen thrice, before anhydrous THF (15 mL, 0.067 M) was added. The reaction mixture was heated at 80 ˚C overnight. The reaction mixture was allowed to cool to room temperature and concentrated under reduced pressure. Purification of the crude residue by flash column chromatography (SiO_2, 0-8% v/v MeOH in CH_2Cl_2) afforded the title compound as a light yellow solid (241 mg, 0.263 mmol, 26% yield).

^{1}H NMR (700 MHz, $CDCl_3$): *δ* 10.3 (d, *J* = 6.7 Hz, 1H, H-12), 8.85 (d, *J* = 4.5 Hz, 1H, H-8), 8.49 (d, *J* = 3.1 Hz, 1H, H-9), 8.09 (d, *J* = 9.2 Hz, 1H, H-6), 7.88 (t, *J* = 1.4 Hz, 1H, H-28), 7.542-7.537 (m, 4H, H-26, H-32), 7.42-7.40 (m, 5H, H-5, H-30), 7.35 (br s, 1H, H-2), 7.02 (br d, *J* = 6.5 Hz, 1H, H-11), 5.58 (d, *J* = 10.6 Hz, 1H, H-24a), 4.48 (d, *J* = 12.3 Hz, 1H, H-24b), 4.04-3.93 (m, 5H, H-4, H-15, H-19a), 3.74 (dd, *J* = 12.5, 10.9 Hz, 1H, H-23a), 3.30-3.27 (m, 1H, H-16a), 3.19-3.15 (m, 1H, H-19b), 3.09-3.08 (m, 1H, H-23b), 2.99-2.95 (m, 1H, H-18a), 2.38 (s, 3H, H-14), 2.23-2.21 (m, 1H, H-17), 1.88-1.81 (m, 2H, H-16b, H-20), 1.73-1.70 (m, 1H, H-18b), 1.39 (s, 36H, H-34), 1.37-1.33 (m, 2H, H-21a, H-21b), 0.84 (t, *J* = 7.3 Hz, 3H, H-22).

^{13}C NMR (176 MHz, $CDCl_3$): *δ* 173.1, 158.7, 152.0, 148.5, 144.9, 144.1, 141.0, 139.3, 132.6, 130.8, 129.8, 127.0, 126.0, 122.8, 122.6, 121.9, 120.8, 101.7, 69.9, 65.7, 64.6, 56.2, 50.5, 47.2, 36.6, 35.2, 31.6, 26.5, 24.8, 24.1, 23.8, 23.1, 11.5.

$[\alpha]_D^{25.0}$ = -60.9 (c. 0.94, $CHCl_3$).

R_f = 0.39 (30% v/v *solvent mixture A* in CH_2Cl_2; *solvent mixture A* = 70:29:1 CH_2Cl_2/MeOH/conc. NH_3 (aq.)).

HRMS (+ESI): *m/z* for cation found 834.5909, $[C_{57}H_{76}N_3O_2]^+$ requires 834.5932, (*δ* = -2.8 ppm).

(R)-[1,1'-binaphthalene]-2,2'-diyl bis(trifluoromethanesulfonate)

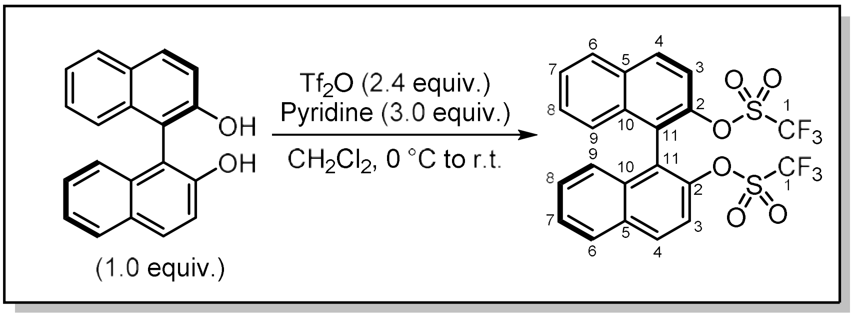

Prepared according to the protocol reported by Hayashi and co-workers with modifications.[11] To a solution of (*R*)-(+)-1,1'-bi(2-naphthol) (5.73 g, 20.0 mmol. 1.0 equiv.) and pyridine (4.8 mL, 59.2 mmol, 3.0 equiv.) in anhydrous CH_2Cl_2 (40 mL, 0.5 M) was added trifluoromethanesulfonic anhydride (8 mL, 47.6 mmol, 2.4 equiv.) dropwise at 0 ˚C over 20 min. The reaction mixture was allowed to stir at 0 ˚C for 2.5 h and concentrated under reduced pressure. The residual brown oil was diluted with EtOAc and the organic layer was washed with aqueous 1 M HCl, aqueous saturated $NaHCO_3$ and brine. The organic layer was dried over Na_2SO_4 and the solvent removed under reduced pressure. Purification of the crude residue by flash column chromatography (SiO_2, 100% CH_2Cl_2) afforded the title compound as a white solid (11.0 g, 20.0 mmol, quant.).

^{1}H NMR (700 MHz, $CDCl_3$): *δ* 8.14 (d, *J* = 9.0 Hz, 2H, H-4), 8.01 (d, *J* = 8.4 Hz, 2H, H-6), 7.62 (d, *J* = 9.1 Hz, 2H, H-3), 7.60-7.58 (m, 2H, H-7), 7.43-7.40 (m, 2H, H-8), 7.26 (d, *J* = 8.5 Hz, 2H, H-9).

^{13}C NMR (176 MHz, $CDCl_3$): *δ* 145.5, 133.3, 132.5, 132.1, 128.5, 128.1, 127.5, 126.9, 123.6, 119.5, 118.3 (q, $J_{C\text{-}F}$ = 320.6 Hz).

^{19}F NMR (376 MHz, $CDCl_3$): *δ* -75.5.

The spectroscopic data is in agreement with that reported in the literature.[11]

(R)-2'-(diphenylphosphoryl)-[1,1'-binaphthalen]-2-yl trifluoromethanesulfonate

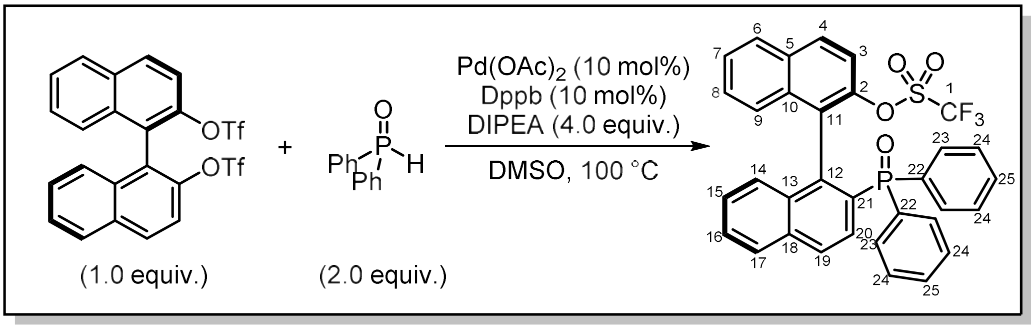

Prepared according to the protocol reported by Hayashi and co-workers with modifications.[11] A 250 mL round-bottomed flask was charged with (*R*)-[1,1'-binaphthalene]-2,2'-diyl bis(trifluoromethanesulfonate) (2.75 g, 5.0 mmol, 1.0 equiv.), diphenylphosphine oxide (2.02 g, 10.0 mmol, 2.0 equiv.), 1.4-bis(diphenylphosphino)butane (213 mg, 0.5 mmol, 10 mol%) and $Pd(OAc)_2$ (112 mg, 0.5 mmol, 10 mol%). The flask was evacuated and back-filled with nitrogen thrice before the addition of DIPEA (3.5 mL, 20.0 mmol, 4.0 equiv.) and anhydrous DMSO (22 mL, 0.23 M). The reaction mixture was stirred at 100 ˚C for 16 h and cooled to room temperature. The reaction mixture was diluted with EtOAc and the organic layer was washed with water thrice. The combined aqueous layers were extracted with EtOAc. The combined organic layers were washed with brine, dried over $MgSO_4$, and concentrated under reduced pressure. Purification of the crude residue by flash column chromatography (SiO_2, 0-50% v/v EtOAc in petroleum ether) afforded the title compound as a yellow solid (2.34 g, 3.88 mmol, 78% yield).

^{1}H NMR (700 MHz, $CDCl_3$): δ 8.01 (d, J = 8.2 Hz, 1H, H-19), 7.94 (d, J = 8.0 Hz, 1H, H-17), 7.91 (d, J = 9.2 Hz, 1H, H-4), 7.84 (d, J = 8.0 Hz, 1H, H-6), 7.65 (dd, J = 11.3, 8.9 Hz, 1H, H-20), 7.58 (dd, J = 7.7, 7.3 Hz, 1H, H-16), 7.49 (dd, J = 11.8, 7.9 Hz, 2H, H-23a), 7.44-7.42 (m, 3H, H-7, H-23b), 7.40-7.37 (m, 2H, H-25a, H-25b), 7.35-7.31 (m, 2H, H-3, H-15), 7.27-7.25 (m, 4H, H-24a, H-24b), 7.18-7.15 (m, 2H, H-8, H-14), 7.00 (d, J = 8.4 Hz, 1H, H-9).

^{13}C NMR (176 MHz, $CDCl_3$): δ 145.7, 137.3 (d, J_{C-P} = 6.8 Hz), 134.6, 134.0, 133.2 (d, J_{C-P} = 11.1 Hz), 133.1 (d, J_{C-P} = 104.2 Hz), 132.6 (d, J_{C-P} = 104.7 Hz), 132.0-131.9 (m), 131.6, 131.1, 130.2 (d, J_{C-P} = 100.4 Hz), 128.8 (d, J_{C-P} = 12.1 Hz), 128.7 (d, J_{C-P} = 12.1 Hz), 128.5, 128.3-128.0 (m), 127.4, 127.3, 127.1, 126.7, 119.0, 118.2 (q, J_{C-F} = 319.0 Hz).

^{19}F NMR (376 MHz, $CDCl_3$): δ -75.0.

^{31}P NMR (162 MHz, $CDCl_3$): δ 28.2.

R_f = 0.34 (50% v/v EtOAc in petroleum ether).

The spectroscopic data is in agreement with that reported in the literature.[11]

(R)-(2'-hydroxy-[1,1'-binaphthalen]-2-yl)diphenylphosphine oxide

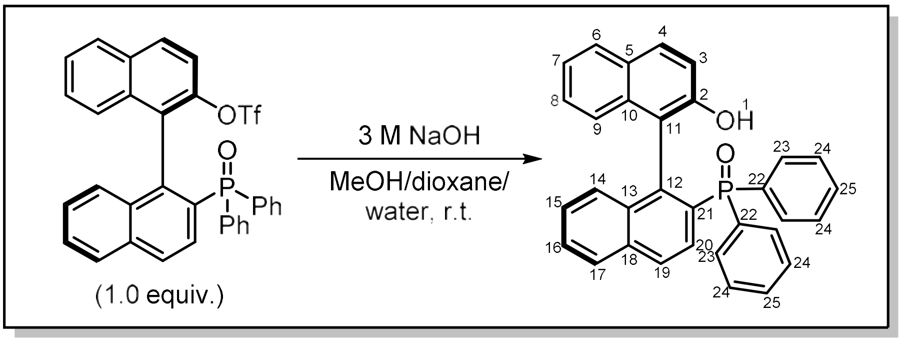

Prepared according to the protocol reported by Hayashi and co-workers with modifications.[11] To a solution of (*R*)-2'-(diphenylphosphoryl)-[1,1'-binaphthalen]-2-yl trifluoromethanesulfonate (2.34 g, 3.88 mmol, 1.0 equiv.) in dioxane (11.5 mL) and MeOH (5.4 mL) was added aqueous 3 M NaOH (5.5 mL, 16.5 mmol, 4.3 equiv.) at room temperature. The reaction mixture was allowed to stir at room temperature for 15 h and was acidified to pH 1 with concentrated HCl. The phases were separated and the aqueous phase was extracted with EtOAc. The combined organic layers were dried over $MgSO_4$ and concentrated under reduced pressure. Purification of the crude residue by flash column chromatography (SiO_2, 50% v/v EtOAc in petroleum ether) afforded the title compound as a pink solid (1.60 g, 3.40 mmol, 88% yield).

^{1}H NMR (700 MHz, $CDCl_3$): δ 8.99 (br s, 1H, H-1), 7.93-7.89 (m, 4H, H-17, H-19, H-23a), 7.62 (d, J = 8.6 Hz, 1H, H-4), 7.59 (t, J = 7.3 Hz, 1H, H-25a), 7.54-7.49 (m, 4H, H-6, H-16, H-24a), 7.40 (dd, J = 10.3, 10.0 Hz, 1H, H-20), 7.36 (d, J = 8.6 Hz, 1H, H-3), 7.25-7.20 (m, 3H, H-15, H-23b), 7.13-7.09 (m, 2H, H-7, H-14), 6.92 (t, J = 7.3 Hz, 1H, H-8), 6.82 (t, J = 7.3 Hz, 1H, H-25b), 6.72-6.70 (m, 2H, H-24b), 6.44 (d, J = 8.3 Hz, 1H, H-9).

^{13}C NMR (176 MHz, $CDCl_3$): δ 153.8, 141.6 (d, $J_{C\text{-}P}$ = 7.8 Hz), 135.3, 133.7 (d, $J_{C\text{-}P}$ = 11.0 Hz), 133.6, 132.3 (d, $J_{C\text{-}P}$ = 8.9 Hz), 132.2, 131.0 (d, $J_{C\text{-}P}$ = 105.0 Hz), 130.8 (d, $J_{C\text{-}P}$ = 104.3 Hz), 130.5, 130.2, 129.8 (d, $J_{C\text{-}P}$ = 10.0 Hz), 129.2, 129.0 (d, $J_{C\text{-}P}$ = 105.2 Hz), 128.8 (d, $J_{C\text{-}P}$ = 11.6 Hz), 128.4, 128.2 (d, $J_{C\text{-}P}$ = 12.4 Hz), 128.0, 127.8, 127.7 (d, $J_{C\text{-}P}$ = 13.1 Hz), 127.6, 127.4 (d, $J_{C\text{-}P}$ = 12.3 Hz), 127.3, 126.0, 125.5, 123.4, 123.32, 123.28.

^{31}P NMR (162 MHz, $CDCl_3$): δ 30.8.

R_f = 0.34 (50% v/v EtOAc in petroleum ether).

The spectroscopic data is in agreement with that reported in the literature.[11]

(R)-2'-(diphenylphosphaneyl)-[1,1'-binaphthalen]-2-ol

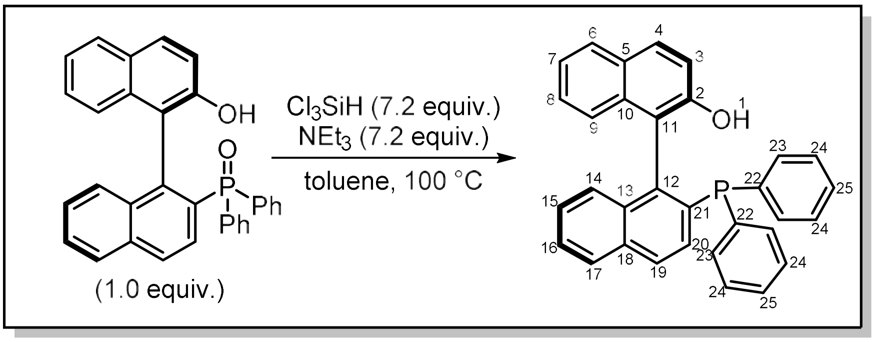

Prepared according to the protocol reported by Hayashi and co-workers with modifications.[11] To a solution of (*R*)-(2'-hydroxy-[1,1'-binaphthalen]-2-yl)diphenylphosphine oxide (1.60 g, 3.40 mmol, 1.0 equiv.) and triethylamine (3.4 mL, 24.4 mmol, 7.2 equiv.) in toluene (34 mL, 0.1 M) was added trichlorosilane (2.5 mL, 24.4 mmol, 7.2 equiv.) dropwise at 0 ˚C. The reaction mixture was warmed to room temperature, refluxed for 16 h and cooled to room temperature. The reaction mixture was diluted with diethyl ether and quenched with aqueous saturated $NaHCO_3$. The resulting suspension was filtered over Celite® and washed with diethyl ether. The filtrate was dried over $MgSO_4$ and concentrated under reduced pressure. Purification of the crude residue by flash column chromatography (SiO_2, 25% v/v EtOAc in petroleum ether) afforded the title compound as a white solid (0.906 g, 1.99 mmol, 59% yield).

^{1}H NMR (700 MHz, $CDCl_3$): δ 7.94 (d, *J* = 8.6 Hz, 1H, H-19), 7.92 (d, *J* = 8.8 Hz, 2H, H-4, H-17), 7.82 (d, *J* = 8.1 Hz, 1H, H-6), 7.53-7.51 (m, 1H, H-16), 7.48 (dd, *J* = 8.6, 2.6 Hz, 1H, H-20), 7.34-7.30 (m, 3H, H-24a, H-25a), 7.29-7.27 (m, 4H, H-14, H-15, H-23a), 7.26 (d, *J* = 2.2 Hz, 1H, H-3), 7.25-7.22 (m, 1H, H-7), 7.21-7.19 (m, 1H, H-25b), 7.17-7.14 (m, 2H, H-24b), 7.09-7.06 (m, 2H, H-23b), 7.04-7.01 (m, 1H, H-8), 6.77 (d, *J* = 8.4 Hz, 1H, H-9), 4.60 (s, 1H, H-1).

^{13}C NMR (176 MHz, $CDCl_3$): δ 151.2, 138.7 (d, $J_{C\text{-}P}$ = 15.8 Hz), 138.5 (d, $J_{C\text{-}P}$ = 34.3 Hz), 137.4 (d, $J_{C\text{-}P}$ = 12.8 Hz), 136.8 (d, $J_{C\text{-}P}$ = 12.6 Hz), 134.1, 133.9 (d, $J_{C\text{-}P}$ = 1.5 Hz), 133.8 (d, $J_{C\text{-}P}$ = 20.1 Hz), 133.7 (d, $J_{C\text{-}P}$ = 19.8 Hz), 133.4 (d, $J_{C\text{-}P}$ = 6.5 Hz), 130.4, 130.3, 129.3, 128.9, 128.8, 128.7 (d, $J_{C\text{-}P}$ = 6.6 Hz), 128.5, 128.30 (d, $J_{C\text{-}P}$ = 7.1 Hz), 128.28, 128.0, 127.4, 127.3, 126.5, 126.4 (d, $J_{C\text{-}P}$ = 2.3 Hz), 125.0, 123.4, 118.4 (d, $J_{C\text{-}P}$ = 8.7 Hz), 117.6.

^{31}P NMR (162 MHz, $CDCl_3$): δ -13.6.

R_f = 0.64 (25% v/v EtOAc in petroleum ether).

The spectroscopic data is in agreement with that reported in the literature.[11]

(R)-(3,5-di-tert-butylbenzyl)(2'-hydroxy-[1,1'-binaphthalen]-2-yl)diphenylphosphonium bromide (**D1•**Br)

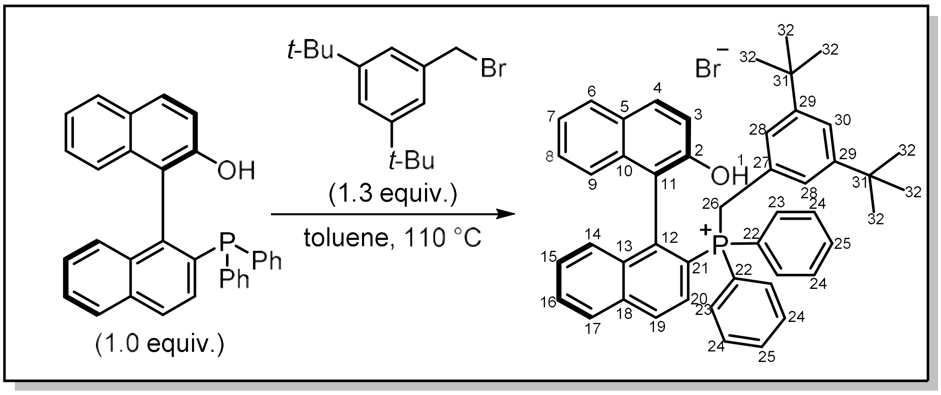

Prepared according to the protocol reported by Maruoka and co-workers.[12] A microwave vial was charged with (*R*)-2'-(diphenylphosphaneyl)-[1,1'-binaphthalen]-2-ol (131 mg, 0.288 mmol, 1.0 equiv.) and 3,5-di-*tert*-butylbenzyl bromide (106 mg, 0.374 mmol, 1.3 equiv.). The vial was evacuated and back-filled with nitrogen thrice before anhydrous toluene (6 mL, 0.05 M) was added. The reaction mixture was heated at 110 ˚C for 16 h and cooled to room temperature. The volatiles were removed under reduced pressure and the crude residue was purified by flash column chromatography (SiO_2, 0-10% v/v MeOH in CH_2Cl_2) to afford the title compound as a white solid (181 g, 0.245 mmol, 85% yield).

^{1}H NMR (700 MHz, $CDCl_3$): δ 10.0 (s, 1H, H-1), 7.93 (d, J = 8.8 Hz, 1H, H-3), 7.88 (d, J = 8.2 Hz, 1H, H-17), 7.74 (dd, J = 8.8, 2.7 Hz, 1H, H-19), 7.64-7.47 (m, 6H, H-4, H-6, H-16, H-23a, H-25a), 7.50-7.47 (m, 2H, H-24a), 7.35-7.31 (m, 2H, H-14, H-15), 7.24-7.20 (m, 4H, H-30, H-23b, H-25b), 7.15 (t, J = 7.6 Hz, 1H, H-7), 7.06-7.02 (m, 3H, H-8, H-24b), 6.89 (t, J = 9.7 Hz, 1H, H-20), 6.65 (d, J = 8.4 Hz, 1H, H-9), 6.62 (br s, 2H, H-28), 4.96 (t, J = 14.8 Hz, 1H, H-26a), 3.75 (t, J = 13.8 Hz, 1H, H-26b), 0.98 (s, 18H, H-32).

^{13}C NMR (176 MHz, $CDCl_3$): δ 155.0, 151.5 (d, $J_{C\text{-}P}$ = 3.1 Hz), 146.8 (d, $J_{C\text{-}P}$ = 9.2 Hz), 135.6 (d, $J_{C\text{-}P}$ = 1.9 Hz), 134.1 (d, $J_{C\text{-}P}$ = 2.6 Hz), 134.0 (d, $J_{C\text{-}P}$ = 12.0 Hz), 133.9, 133.7 (d, $J_{C\text{-}P}$ = 9.2 Hz), 133.1 (d, $J_{C\text{-}P}$ = 2.4 Hz), 132.5 (d, $J_{C\text{-}P}$ = 9.7 Hz), 131.6, 130.3, 129.8 (d, $J_{C\text{-}P}$ = 12.5 Hz), 128.7 (d, $J_{C\text{-}P}$ = 12.2 Hz), 128.4 (d, $J_{C\text{-}P}$ = 10.6 Hz), 128.35, 128.28, 128.07, 128.04, 127.97 (d, $J_{C\text{-}P}$ = 12.3 Hz), 127.96, 126.7, 125.9 (d, $J_{C\text{-}P}$ = 5.6 Hz), 125.8 (d, $J_{C\text{-}P}$ = 8.6 Hz), 123.9, 122.9, 122.3 (d, $J_{C\text{-}P}$ = 3.9 Hz), 120.7 (d, $J_{C\text{-}P}$ = 86.4 Hz), 120.3, 119.2 (d, $J_{C\text{-}P}$ = 84.3 Hz), 114.8 (d, $J_{C\text{-}P}$ = 5.1 Hz), 114.6 (d, $J_{C\text{-}P}$ = 87.7 Hz), 34.7, 31.2, 30.4 (d, $J_{C\text{-}P}$ = 46.0 Hz).

^{31}P NMR (162 MHz, $CDCl_3$): δ 23.2.

$[\alpha]_D^{25.0}$ = +39.8 (c. 0.67, $CHCl_3$).

R_f = 0.45 (10% v/v MeOH in CH_2Cl_2)

HRMS (+ESI): *m/z* for cation found 657.3304, $[C_{47}H_{46}PO]^+$ requires 657.3281, (δ = +3.5 ppm).

5 Synthesis and Characterisation of Chiral Rh(II,II) Tetracarboxylate Dimers

The syntheses of compounds Rh_2(**A1**)$_2$•(**B1**)$_2$,[4] Rh_2(**A1**)$_2$•(**B2**)$_2$,[6] Rh_2(**A1**)$_2$•(**B6**)$_2$,[7] Rh_2(**A1**)$_2$•(**B7**)$_2$,[7] Rh_2(**A1**)$_2$•(**B8**)$_2$,[7] Rh_2(**A1**)$_2$•(**B9**)$_2$,[7] Rh_2(**A1**)$_2$•(**B10**)$_2$,[4] Rh_2(**A1**)$_2$•(**C1**)$_2$,[7] and Rh_2(**A1**)$_2$•(**C2**)$_2$[4] have been previously reported.

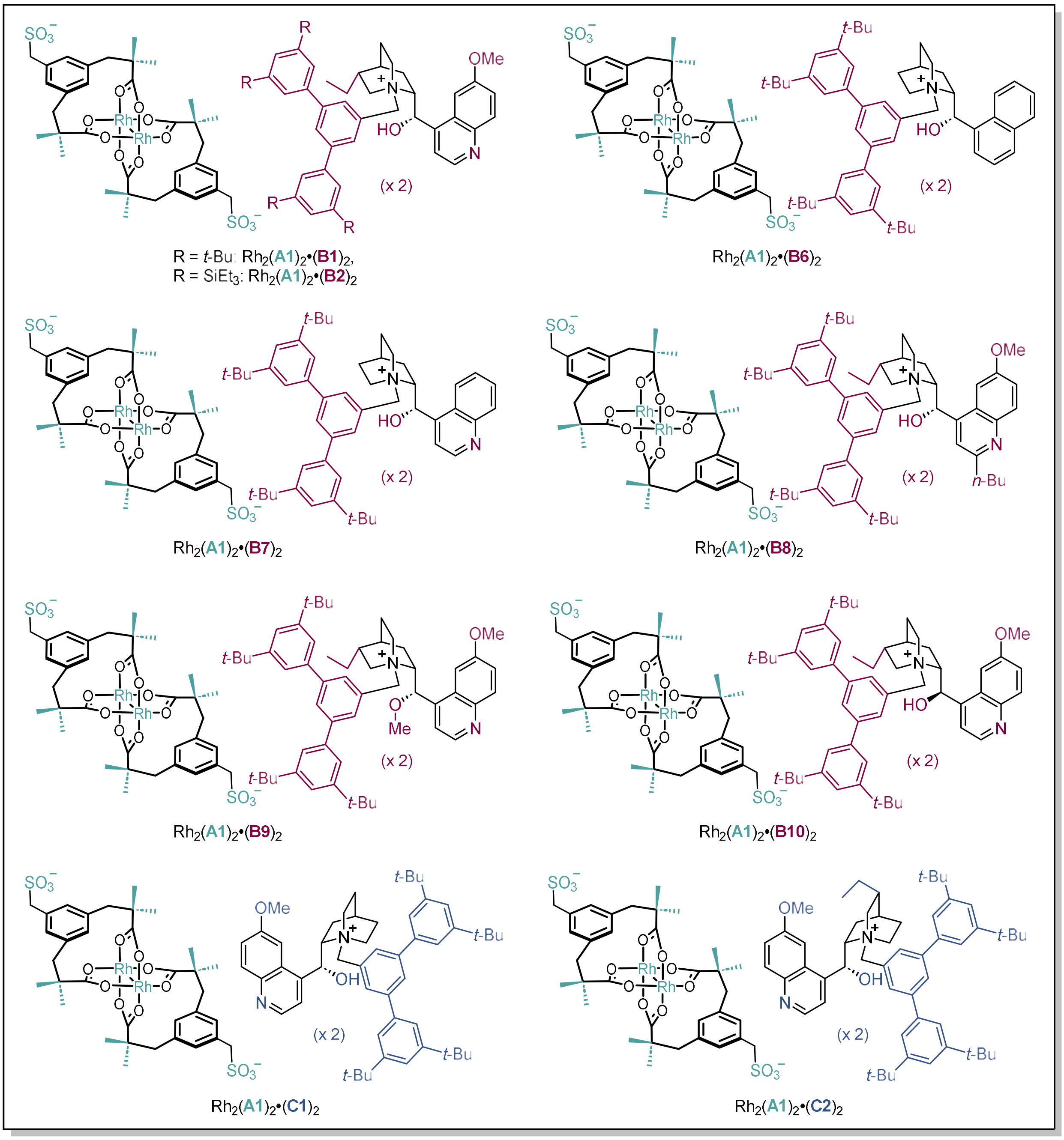

Bis[rhodium (1S,2R,4S,5R)-1-(3,5-di-tert-butylbenzyl)-5-ethyl-2-((S)-hydroxy(6-methoxyquinolin-4-yl)methyl)quinuclidin-1-ium (3,5-bis(2-carboxy-2-methylpropyl)phenyl)methanesulfonate)] (Rh_2(**A1**)$_2$•(**B3**)$_2$)

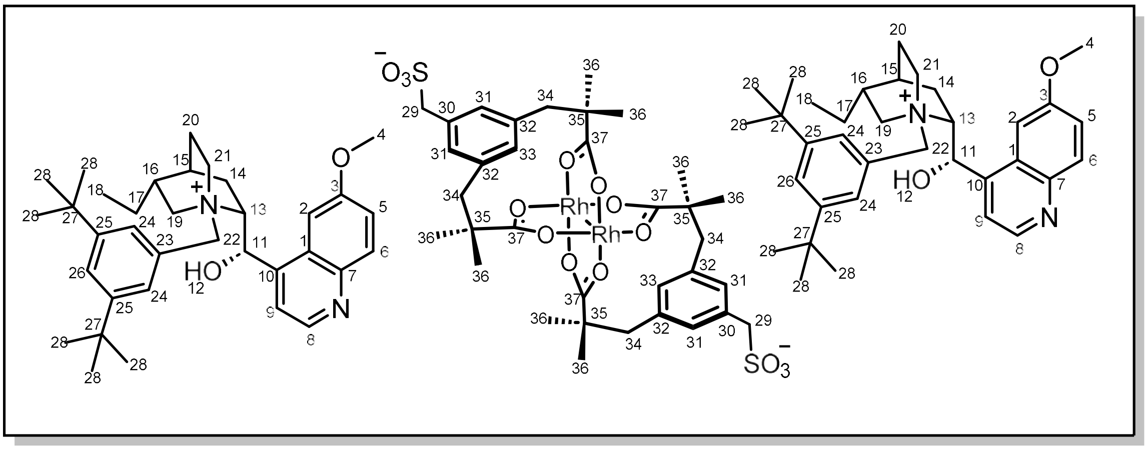

A sinter funnel was loosely packed with a pad of Amberlite® IRC120 H hydrogen form beads (approximately 6 cm column height) and equilibrated by flushing through with three column volumes of MeOH. Following this, a solution of Rh_2(**A1**)$_2$•(***n*-Bu$_4$N**)$_2$ (28.6 mg, 20 µmol, 1.0 equiv.) in MeOH (4 mL) was loaded onto the pad and eluted with light suction. The eluent was recycled through the pad a further four times. Following this, the eluent was evaporated to dryness, reconstituted in water (10 mL) and basified with aqueous 10% NaOH solution (10 drops). A 3:1 mixture of $CHCl_3$/*i*-PrOH (20 mL) was added, followed by **B3**•Br (22.0 mg, 36 µmol, 1.8 equiv.). The biphasic mixture was stirred rapidly overnight, during which the blue colour of the aqueous phase gradually fades and the organic layer gradually turns red. The layers were separated and the aqueous phase was extracted with a 3:1 mixture of $CHCl_3$/*i*-PrOH (3 x 20 mL). The combined organic layers were dried over $MgSO_4$ and the solvent removed under reduced pressure. The catalyst was subsequently desolvated at 90 ˚C under vacuum overnight to afford the title compound as a brown solid (32.2 mg, 16.1 µmol, 45% yield).

To obtain the pyridine solvate of the catalyst, a portion of the desolvated catalyst was fully dissolved in pyridine to give a bright pink solution. The excess pyridine was removed under a gentle stream of nitrogen and the oily residue was dried thoroughly under vacuum to obtain a free-flowing pink powder, which is ready for use.

^{1}H NMR (700 MHz, C_5D_5N): δ 9.08 (d, J = 4.5 Hz, 2H, H-8), 8.70 (br s, 2H, H-12), 8.37 (d, J = 9.1 Hz, 2H, H-6), 8.07 (d, J = 4.5 Hz, 2H, H-9), 7.99 (d, J = 1.6 Hz, 4H, H-24), 7.88 (d, J = 2.5 Hz, 2H, H-2), 7.73 (t, J = 1.6 Hz, 2H, H-26), 7.55 (dd, J = 9.2, 2.6 Hz, 2H, H-5), 7.14 (t, J = 1.7 Hz, 2H, H-33), 7.07-7.05 (m, 6H, H, H-11, H-31), 5.83 (d, J = 12.3 Hz, 2H, H-22a), 5.37 (d, J = 12.0 Hz, 2H, H-22b), 4.54-4.49 (m, 4H, H-19a, H-21a), 4.32 (d, J = 13.0 Hz, 2H,

H-29a), 4.27 (d, J = 13.0 Hz, 2H, H-29b), 4.18 (dd, J = 10.1, 9.1 Hz, 2H, H-13), 4.01 (s, 6H, H-4), 3.72 (dd, J = 11.3, 10.6 Hz, 2H, H-19b), 3.31-3.27 (m, 2H, H-21b), 2.62 (dd, J = 11.9, 10.9 Hz, 2H, H-14a), 2.44 (d, J = 12.5 Hz, 4H, H-34a), 2.33 (d, J = 12.4 Hz, 4H, H-34b), 1.67 (br s, 2H, H-15), 1.57-1.55 (m, 4H, H-17a, H-17b), 1.48-1.43 (m, 2H, H-16), 1.42 (s, 36H, H-28), 1.35-1.28 (m, 4H, H-20a, H-20b), 1.09-1.05 (m, 2H, H-14b), 1.02 (s, 12H, H-36a), 1.00 (s, 12H, H-36b), 0.80 (t, J = 7.1 Hz, 6H, H-18).

^{13}C NMR (176 MHz, C_5D_5N): δ 197.2, 159.1, 152.5, 148.5, 145.7, 145.0, 138.6, 134.7, 133.0, 131.2, 130.1, 129.8, 128.6, 127.3, 124.5, 122.1, 121.8, 103.6, 69.0, 66.1, 64.5, 59.4, 57.2, 56.64, 56.57, 47.3, 46.3, 36.4, 35.6, 32.0, 30.4, 26.5, 25.7, 25.0, 21.8, 12.0.

$[\alpha]_D^{25.0}$ = +26.0 (c. 0.10, C_5H_5N).

HRMS (-ESI): *m/z* for monoanion found $[M+H]^-$ 945.0171, $[C_{34}H_{43}O_{14}Rh_2S_2]^-$ requires 945.0210, (δ = -4.1 ppm).

HRMS (+ESI): *m/z* for cation found 529.3796, $[C_{35}H_{49}N_2O_2]^+$ requires 529.3789, (δ = +1.3 ppm).

Bis[rhodium (1S,2R,4S,5R)-1-(3,5-bis(trifluoromethyl)benzyl)-5-ethyl-2-((S)-hydroxy(6-methoxyquinolin-4-yl)methyl)quinuclidin-1-ium (3,5-bis(2-carboxy-2-methylpropyl)phenyl)methanesulfonate)] (Rh_2(**A1**)$_2$•(**B4**)$_2$)

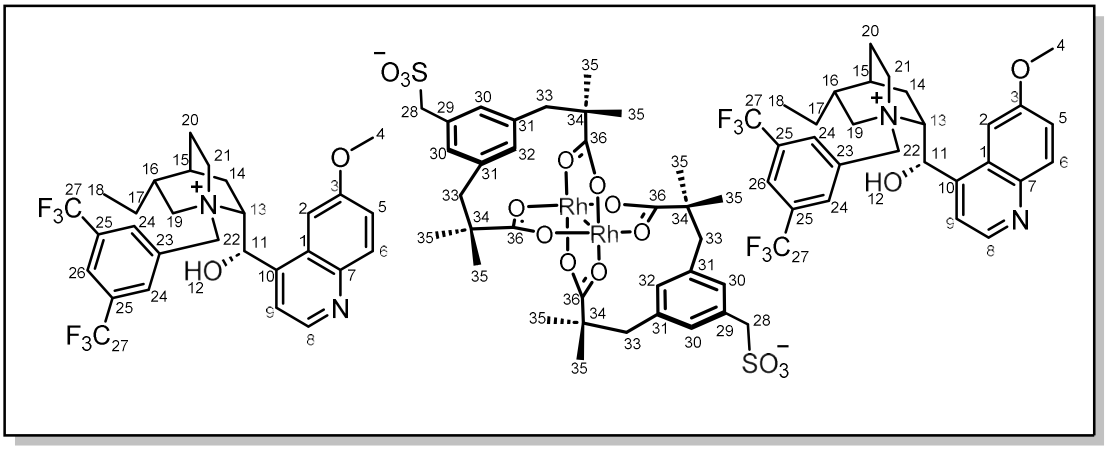

A sinter funnel was loosely packed with a pad of Amberlite® IRC120 H hydrogen form beads (approximately 6 cm column height) and equilibrated by flushing through with three column volumes of MeOH. Following this, a solution of Rh_2(**A1**)$_2$•(***n*-Bu$_4$N**)$_2$ (28.6 mg, 20 µmol, 1.0 equiv.) in MeOH (4 mL) was loaded onto the pad and eluted with light suction. The eluent was recycled through the pad a further four times. Following this, the eluent was evaporated to dryness, reconstituted in water (10 mL) and basified with aqueous 10% NaOH solution (10 drops). A 3:1 mixture of $CHCl_3$/*i*-PrOH (20 mL) was added, followed by **B4**•Br (22.8 mg, 36

μmol, 1.8 equiv.). The biphasic mixture was stirred rapidly overnight, during which the blue colour of the aqueous phase gradually fades and a red solid precipitated out. The red solid was filtered off and washed with water (10 mL x 3), $CHCl_3$ (10 mL x 3) and diethyl ether (10 mL x 2). The red solid was dissolved in MeOH and the solvent removed under reduced pressure. The catalyst was subsequently desolvated at 90 ˚C under vacuum overnight to afford the title compound as a grey solid (30.4 mg, 14.8 μmol, 41% yield).

To obtain the pyridine solvate of the catalyst, a portion of the desolvated catalyst was fully dissolved in pyridine to give a bright pink solution. The excess pyridine was removed under a gentle stream of nitrogen and the oily residue was dried thoroughly under vacuum to obtain a free-flowing pink powder, which is ready for use.

^{1}H NMR (700 MHz, C_5D_5N): δ 9.07 (d, J = 4.4 Hz, 2H, H-8), 8.91 (s, 4H, H-24), 8.71 (d, J = 2.8 Hz, 2H, H-12), 8.36 (d, J = 9.1 Hz, 2H, H-6), 8.16 (br s, 2H, H-26), 8.13 (d, J = 4.4 Hz, 2H, H-9), 8.07 (d, J = 2.1 Hz, 2H, H-2), 7.56 (dd, J = 9.2, 2.5 Hz, 2H, H-5), 7.14 (s, 2H, H-32), 7.05 (s, 4H, H-30), 7.03 (br s, 2H, H-11), 5.83-5.78 (m, 4H, H-22a, H-22b), 4.65 (dd, J = 11.2, 11.0 Hz, 2H, H-21a), 4.58 (dd, J = 9.8, 9.5 Hz, 2H, H-19a), 4.33-4.27 (m, 6H, H-13, H-28a, H-28b), 4.11 (s, 6H, H-4), 3.68 (t, J = 11.1 Hz, 2H, H-19b), 3.25-3.21 (m, 2H, H-21b), 2.60 (t, J = 11.4 Hz, 2H, H-14a), 2.46 (d, J = 12.3 Hz, 4H, H-33a), 2.31 (d, J = 12.4 Hz, 4H, H-33b), 1.69 (br s, 2H, H-15), 1.66-1.61 (m, 2H, H-16), 1.56-1.46 (m, 8H, H-17a, H-17b, H-20a, H-20b), 1.08-1.05 (m, 2H, H-14b), 1.03 (s, 12H, H-35a), 1.00 (s, 12H, H-35b), 0.79 (t, J = 7.4 Hz, 6H, H-18).

^{13}C NMR (176 MHz, C_5D_5N): δ 197.2, 159.3, 148.4, 145.7, 144.6, 138.7, 135.7, 134.5, 133.0, 132.52, 132.46 (q, $J_{C\text{-}F}$ = 33.3 Hz), 131.2, 130.2, 127.5, 124.7, 124.2 (q, $J_{C\text{-}F}$ = 272.8 Hz), 122.3, 122.0, 103.9, 69.2, 66.9, 61.9, 59.3, 57.3, 57.1, 56.8, 47.3, 46.3, 36.4, 26.44, 26.42, 25.5, 25.0, 24.7, 22.0, 12.0.

^{19}F NMR (376 MHz, C_5D_5N): δ -61.9.

$[\alpha]_D^{25.0}$ = +52.5 (c. 0.08, C_5H_5N).

HRMS (-ESI): *m/z* for monoanion found $[M+H]^-$ 945.0171, $[C_{34}H_{43}O_{14}Rh_2S_2]^-$ requires 945.0210, (δ = -4.1 ppm).

HRMS (+ESI): *m/z* for cation found 553.2289, $[C_{29}H_{31}F_6N_2O_2]^+$ requires 553.2284, (δ = +0.9 ppm).

Bis[rhodium (1S,2R,4S,5R)-1-(3,5-dibromobenzyl)-5-ethyl-2-((S)-hydroxy(6-methoxyquinolin-4-yl)methyl)quinuclidin-1-ium (3,5-bis(2-carboxy-2-methylpropyl)phenyl)methanesulfonate)] (Rh_2(**A1**)$_2$•(**B5**)$_2$)

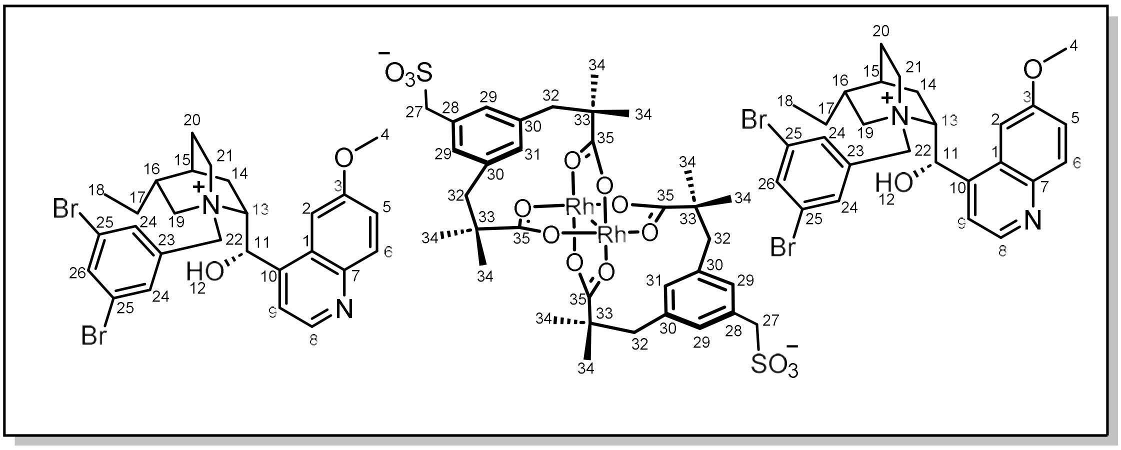

A sinter funnel was loosely packed with a pad of Amberlite® IRC120 H hydrogen form beads (approximately 6 cm column height) and equilibrated by flushing through with three column volumes of MeOH. Following this, a solution of Rh_2(**A1**)$_2$•(***n*-Bu$_4$N**)$_2$ (28.6 mg, 20 µmol, 1.0 equiv.) in MeOH (4 mL) was loaded onto the pad and eluted with light suction. The eluent was recycled through the pad a further four times. Following this, the eluent was evaporated to dryness, reconstituted in water (10 mL) and basified with aqueous 10% NaOH solution (10 drops). A 3:1 mixture of $CHCl_3$/*i*-PrOH (20 mL) was added, followed by **B5**•Br (23.6 mg, 36 µmol, 1.8 equiv.). The biphasic mixture was stirred rapidly overnight, during which the blue colour of the aqueous phase gradually fades and a red solid precipitated out. The red solid was filtered off and washed with water (10 mL x 3), $CHCl_3$ (10 mL x 3), diethyl ether (10 mL x 2) and MeOH (10 mL x 2). The catalyst was subsequently desolvated at 90 ˚C under vacuum overnight to afford the title compound as a purple solid (31.2 mg, 14.9 µmol, 41% yield).

To obtain the pyridine solvate of the catalyst, a portion of the desolvated catalyst was fully dissolved in pyridine to give a bright pink solution. The excess pyridine was removed under a gentle stream of nitrogen and the oily residue was dried thoroughly under vacuum to obtain a free-flowing pink powder, which is ready for use.

^{1}H NMR (700 MHz, C_5D_5N): *δ* 9.07 (d, *J* = 4.4 Hz, 2H, H-8), 8.66 (d, *J* = 3.0 Hz, 2H, H-12), 8.38 (s, 4H, H-24), 8.36 (d, *J* = 9.2 Hz, 2H, H-6), 8.11 (d, *J* = 4.3 Hz, 2H, H-9), 7.98 (d, *J* = 2.1 Hz, 2H, H-2), 7.86 (t, *J* = 1.6 Hz, 2H, H-26), 7.55 (dd, *J* = 9.1, 2.4 Hz, 2H, H-5), 7.16 (br s, 2H, H-31), 7.08 (s, 4H, H-29), 7.00 (br s, 2H, H-11), 5.57 (d, *J* = 12.5 Hz, 2H, H-22a), 5.47 (d, *J* = 12.4 Hz, 2H, H-22b), 4.45-4.41 (m, 4H, H-19a, H-21a), 4.32 (d, *J* = 13.0 Hz, 2H, H-27a), 4.27 (d, *J* = 12.9 Hz, 2H, H-27b), 4.22 (dd, *J* = 9.2, 9.0 Hz, 2H, H-13), 4.07 (s, 6H, H-

4), 3.61 (t, J = 11.1 Hz, 2H, H-19b), 3.12-3.08 (m, 2H, H-21b), 2.57 (dd, J = 11.9, 11.5 Hz, 2H, H-14a), 2.52 (d, J = 12.4 Hz, 4H, H-32a), 2.38 (d, J = 12.4 Hz, 4H, H-32b), 1.69 (br s, 2H, H-15), 1.61-1.57 (m, 2H, H-16), 1.54-1.45 (m, 8H, H-17a, H-17b, H-20a, H-20b), 1.04-1.02 (m, 26H, H, H14b, H-34a, H-34b), 0.78 (t, J = 7.4 Hz, 6H, H-18).

^{13}C NMR (176 MHz, C_5D_5N): δ 197.2, 159.3, 148.4, 145.7, 144.7, 138.7, 136.6, 136.5, 134.6, 133.3, 133.0, 131.2, 130.2, 127.4, 124.2, 122.3, 121.9, 103.6, 69.1, 66.7, 62.1, 59.3, 57.3, 57.2, 56.7, 47.4, 46.3, 36.4, 26.5, 25.4, 24.9, 24.7, 21.9, 12.0.

$[\alpha]_D^{25.0}$ = +57.3 (c. 0.07, C_5H_5N).

HRMS (-ESI): *m/z* for monoanion found $[M+H]^-$ 945.0171, $[C_{34}H_{43}O_{14}Rh_2S_2]^-$ requires 945.0210, (δ = -4.1 ppm).

HRMS (+ESI): *m/z* for cation found 575.0749, $[C_{27}H_{31}Br_2N_2O_2]^+$ requires 575.0727, (δ = +3.8 ppm).

6 Synthesis of Reagents for Rhodium-Catalysed Nitrene Transfer

The syntheses of compounds **Hfbs-NH$_2$** (**2a**), **Pfps-NH$_2$** (**2b**), **Tfes-NH$_2$, Tces-NH$_2$, C_6F_5IO, $C_6F_5I(OTFA)_2$** have been previously reported.[4, 6-7, 13]

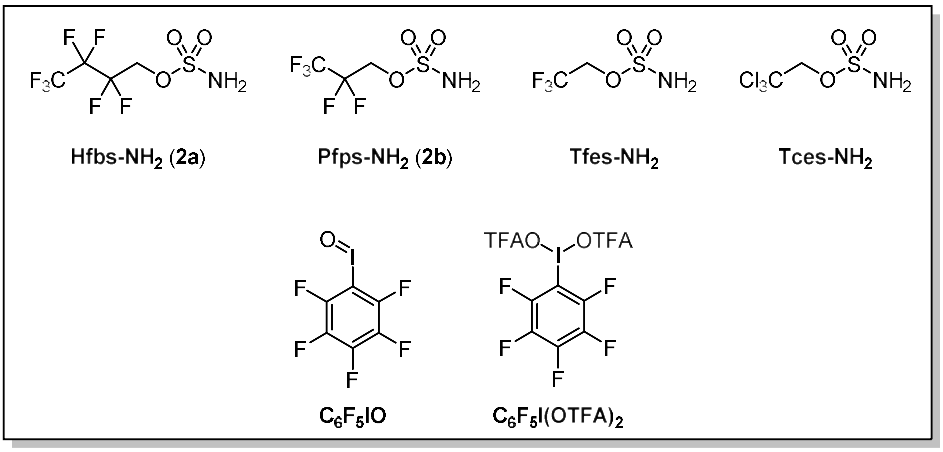

Note: We have found it very important to thoroughly remove excess trifluoroacetic acid when preparing $C_6F_5I(OTFA)_2$ since any excess trifluoroacetic acid will adversely affect the reaction outcome.

7 Synthesis and Characterisation of Substrates (Aziridination)

7.1 Synthesis of Substrates – Propargyl Alcohols

General Procedure 1 (GP 1):

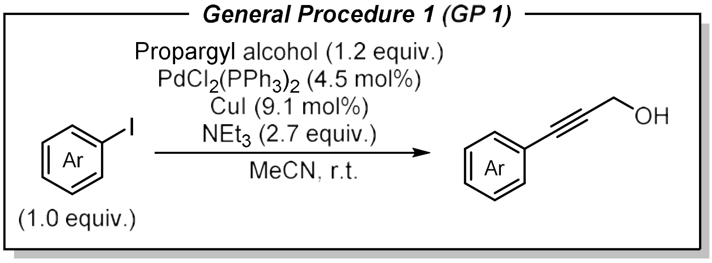

To a solution of aryl iodide (5.0 mmol, 1.0 equiv.) in MeCN (20 mL, 0.25 M) was added $PdCl_2(PPh_3)_2$ (157.9 mg, 0.23 mmol, 4.5 mol%), CuI (86.7 mg, 0.46 mmol, 9.1 mol%) and Et_3N (1.9 mL, 13.5 mmol, 2.7 equiv.) at room temperature under a nitrogen atmosphere. The reaction mixture was allowed to stir at room temperature for 20 min before the addition of propargyl alcohol (0.35 mL, 6.0 mmol, 1.2 equiv.). The resultant reaction mixture was stirred at room temperature overnight (~16 h) and the solvent was removed under reduced pressure. Purification of the crude residue by flash column chromatography afforded the corresponding propargyl alcohol product.

3-phenylprop-2-yn-1-ol

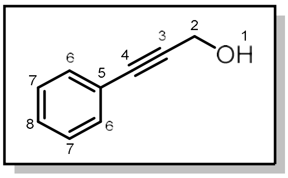

Prepared according to **GP 1** with iodobenzene (2.3 mL, 20 mmol, 1.0 equiv.). Purification by flash column chromatography (SiO_2, 0-25% v/v acetone in petroleum ether) afforded the title compound as a red oil (2.64 g, 20 mmol, quant.).

^{1}H NMR (400 MHz, $CDCl_3$): *δ* 7.46-7.42 (m, 2H, H-6), 7.33-7.30 (m, 3H, H-7, H-8), 4.50 (d, *J* = 6.0 Hz, 2H, H-2), 1.82 (t, *J* = 6.1 Hz, 1H, H-1).

^{13}C NMR (101 MHz, $CDCl_3$): *δ* 131.8, 128.6, 128.5, 122.7, 87.3, 85.9, 51.8.

R_f = 0.57 (30% v/v acetone in petroleum ether)

The spectroscopic data is in agreement with that reported in the literature.[6]

3-(2-isopropylphenyl)prop-2-yn-1-ol

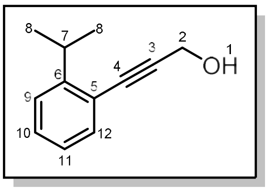

Prepared according to **GP 1** with 1-iodo-2-isopropylbenzene (0.80 mL, 5.0 mmol, 1.0 equiv.). Purification by flash column chromatography (SiO_2, 0-15% v/v EtOAc in petroleum ether) afforded the title compound as a red oil (483 mg, 2.77 mmol, 55% yield).

^{1}H NMR (400 MHz, $CDCl_3$): δ 7.42 (d, J = 8.1 Hz, 1H, H-12), 7.32-7.26 (m, 2H, H-9, H10), 7.15-7.11 (m, 1H, H-11), 4.54 (d, J = 5.9 Hz, 2H, H-2), 3.47 (sept, J = 6.9 Hz, 1H, H-7), 1.81 (t, J = 6.0 Hz, 1H, H-1), 1.26 (d, J = 6.9 Hz, 6H, H-8).

^{13}C NMR (101 MHz, $CDCl_3$): δ 150.7, 132.7, 129.0, 125.6, 125.1, 121.4, 90.8, 84.6, 51.9, 31.6, 23.3.

R_f = 0.41 (20% v/v EtOAc in petroleum ether).

The spectroscopic data is in agreement with that reported in the literature.[14]

3-(2-fluorophenyl)prop-2-yn-1-ol

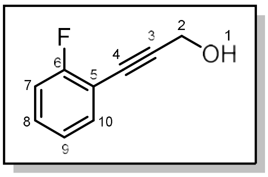

Prepared according to **GP 1** with 2-fluoroiodobenzene (0.58 mL, 5.0 mmol, 1.0 equiv.). Purification by flash column chromatography (SiO_2, 0-25% v/v EtOAc in petroleum ether) afforded the title compound as a red oil (751 mg, 5.0 mmol, quant.).

^{1}H NMR (400 MHz, $CDCl_3$): δ 7.45-7.41 (m, 1H, H-10), 7.33-7.27 (m, 1H, H, H-8), 7.11-7.04 (m, 2H, H-7, H-9), 4.53 (s, 2H, H-2) 1.98 (s, 1H, H-1).

^{13}C NMR (101 MHz, $CDCl_3$): δ 162.9 (d, $J_{C\text{-}F}$ = 251.0 Hz), 133.8 (d, $J_{C\text{-}F}$ = 1.3 Hz), 130.4 (d, $J_{C\text{-}F}$ = 8.0 Hz), 124.1 (d, $J_{C\text{-}F}$ = 3.7 Hz), 115.6 (d, $J_{C\text{-}F}$ = 20.5 Hz), 111.2 (d, $J_{C\text{-}F}$ = 15.4 Hz), 92.6 (d, $J_{C\text{-}F}$ = 3.4 Hz), 79.2 (d, $J_{C\text{-}F}$ = 1.0 Hz), 51.8.

^{19}F NMR (471 MHz, $CDCl_3$): δ -110.2.

R_f = 0.47 (30% v/v EtOAc in petroleum ether).

The spectroscopic data is in agreement with that reported in the literature.[15]

tert-butyl (3-iodophenyl)carbamate

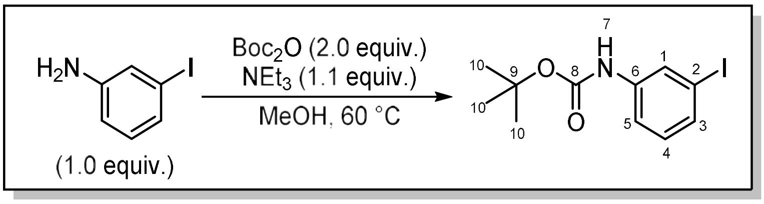

Prepared according to procedure reported by Costello and Ferreira with modifications.[16] To a solution of 3-iodoaniline (0.72 mL, 6.0 mmol, 1.0 equiv.) in MeOH (10 mL, 0.6 M) was added triethylamine (0.92 mL, 6.6 mmol, 1.1 equiv.) and Boc_2O (2.62 g, 12.0 mmol, 2.0 equiv.) at room temperature. The reaction mixture was stirred at 60 ˚C overnight and cooled to room temperature. The volatiles were removed under reduced pressure and the crude residue was dissolved in EtOAc (20 mL). The organic layer was washed with water, brine and dried over $MgSO_4$. The solvent was removed under reduced pressure and the crude residue was purified by flash column chromatography (SiO_2, 0-10% v/v EtOAc in petroleum ether) to afford the title compound as a white solid (1.72 g, 5.39 mmol, 90% yield).

^{1}H NMR (700 MHz, $CDCl_3$): δ 7.83 (s, 1H, H-1), 7.37-7.35 (m, 1H, H-3), 7.26 (d, *J* = 7.4 Hz, 1H, H-5), 6.99 (t, *J* = 8.0 Hz, 1H, H-4), 6.42 (br s, 1H, H-7), 1.51 (s, 9H, H-10).

^{13}C NMR (176 MHz, $CDCl_3$): δ 152.5, 139.7, 132.2, 130.5, 127.3, 117.7, 94.4, 81.1, 28.4.

R_f = 0.47 (10% v/v EtOAc in petroleum ether).

The spectroscopic data is in agreement with that reported in the literature.[17]

tert-butyl (3-(3-hydroxyprop-1-yn-1-yl)phenyl)carbamate

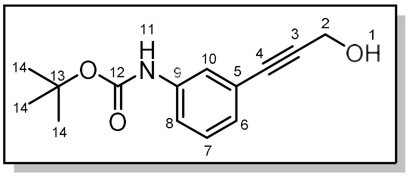

Prepared according to **GP 1** with *tert*-butyl (3-iodophenyl)carbamate (1.60 g, 5.0 mmol, 1.0 equiv.). Purification by flash column chromatography (SiO_2, 0-25% v/v EtOAc in petroleum ether) afforded the title compound as an orange oil (1.24 g, 5.01 mmol, quant.).

^{1}H NMR (400 MHz, $CDCl_3$): δ 7.48 (s, 1H, H-10), 7.30 (d, *J* = 8.1 Hz, 1H, H-8), 7.20 (dd, *J* = 8.1, 7.6 Hz, 1H, H-7), 7.08 (dd, *J* = 7.6, 1.1 Hz, 1H, H-6), 6.59 (s, 1H, H-11), 4.46 (d, *J* = 5.8 Hz, 2H, H-2), 2.07 (t, *J* = 5.8 Hz, 1H, H-1), 1.51 (s, 9H, H-14).

^{13}C NMR (101 MHz, $CDCl_3$): δ 152.8, 138.5, 129.1, 126.4, 123.3, 121.7, 118.9, 87.4, 85.5, 81.0, 51.7, 28.4.

R_f = 0.31 (30% v/v EtOAc in petroleum ether).

HRMS (+ESI): *m/z* found $[M-C_5H_8O_2]^+$ 148.0763, $[C_9H_{10}NO]^+$ requires 148.0757, (δ = +4.1 ppm).

3-(3-(tert-butyl)phenyl)prop-2-yn-1-ol

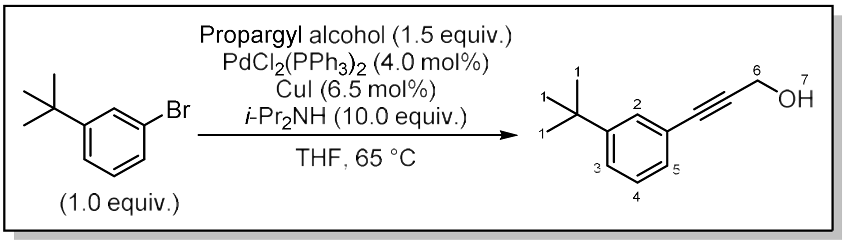

An oven-dried 100 mL round bottom flask equipped with a Teflon-coated magnetic stir bar was charged with 1-bromo-3-(*tert*-butyl)benzene (0.855 mL, 5.00 mmol, 1.00 equiv.), $(Ph_3P)_2PdCl_2$ (140 mg, 0.200 mmol, 4.00 mol%) and freshly prepared copper iodide (61.9 mg, 0.325 mmol, 6.50 mol%). The flask was sealed and evacuated and backfilled with N_2 three times, following which THF (12 mL) and *i*-Pr_2NH (7.1 mL, 50.3 mmol, 10.1 equiv.) were added. The reaction mixture was stirred for 15 min following which propargyl alcohol (0.433 mL, 7.50 mmol, 1.50 equiv.) was added and the reaction was heated at 65 °C for 18 h. The reaction was then allowed to cool to room temperature, was diluted with DCM (100 mL) and the organic layer was successively washed with NH_4Cl (sat. aq., 3 x 50 mL), $NaHCO_3$ (sat. aq., 3 x 50 mL) and brine (50 mL). The organic layer was then dried over $MgSO_4$, filtered, and concentrated under reduced pressure. Purification of the crude residue by flash column chromatography (SiO_2, 0-12% v/v EtOAc in petroleum ether) afforded the title compound as a yellow oil (280 mg, 1.49 mmol, 30% yield).

^{1}H NMR (500 MHz, $CDCl_3$): δ 7.50 (br s, 1H, H-2), 7.40–7.36 (m, 1H, H-3), 7.28–7.25 (m, 2H, H-4, H-5), 4.52 (d, *J* = 6.1 Hz, 2H, H-6), 1.75 (t, *J* = 6.2 Hz, 1H, H-7), 1.32 (s, 9H, H-1)

^{13}C NMR (126 MHz, $CDCl_3$): δ 151.4, 128.95, 128.89, 128.2, 125.9, 122.2, 86.7, 86.4, 51.9, 34.8, 31.3.

R_f = 0.50 (30% v/v EtOAc in petroleum ether).

HRMS: *This compound did not ionise.*

3-(3-bromophenyl)prop-2-yn-1-ol

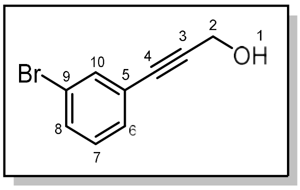

Prepared according to **GP 1** with 3-bromoiodobenzene (0.64 mL, 5.0 mmol, 1.0 equiv.). Purification by flash column chromatography (SiO_2, 0-20% v/v EtOAc in petroleum ether) afforded the title compound as a brown oil (1.06 g, 5.0 mmol, quant.).

^{1}H NMR (400 MHz, $CDCl_3$): δ 7.58 (t, J = 1.7 Hz, 1H, H-10), 7.45 (d, J = 8.1 Hz, 1H, H-8), 7.35 (d, J = 7.7 Hz, 1H, H-6), 7.17 (t, J = 7.8 Hz, 1H, H-7), 4.49 (d, J = 6.2 Hz, 2H, H-2), 1.84 (t, J = 6.1 Hz, 1H, H-1).

^{13}C NMR (101 MHz, $CDCl_3$): δ 134.6, 131.8, 130.4, 129.9, 124.6, 122.2, 88.7, 84.3, 51.7.

R_f = 0.47 (30% v/v EtOAc in petroleum ether).

The spectroscopic data is in agreement with that reported in the literature.[18]

ethyl 3-(3-hydroxyprop-1-yn-1-yl)benzoate

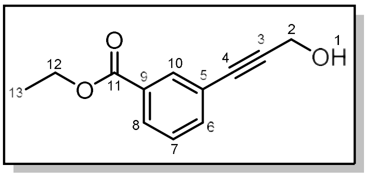

Prepared according to **GP 1** with ethyl 3-iodobenzoate (1.7 mL, 10 mmol, 1.0 equiv.). Purification by flash column chromatography (SiO_2, 0-40% v/v EtOAc in petroleum ether) afforded the title compound as a brown, amorphous solid (1.93 g, 9.45 mmol, 95% yield).

^{1}H NMR (400 MHz, $CDCl_3$): δ 8.10 (t, J = 1.5 Hz, 1H, H-10), 7.99 (dt, J = 7.9, 1.4 Hz, 1H, H-8), 7.59 (dt, J = 7.7, 1.4 Hz, 1H, H-6), 7.38 (t, J = 7.8 Hz, 1H, H-7), 4.51 (d, J = 6.1 Hz, 2H, H-2), 4.37 (q, J = 7.2 Hz, 2H, H-12), 1.91 (t, J = 6.0 Hz, 1H, H-1), 1.39 (t, J = 7.1 Hz, 3H, H-13).

^{13}C NMR (101 MHz, $CDCl_3$): δ 166.0, 135.8, 132.9, 130.9, 129.6, 128.6, 123.1, 88.3, 84.8, 61.4, 51.7, 14.4.

R_f = 0.37 (30% v/v EtOAc in petroleum ether).

The spectroscopic data is in agreement with that reported in the literature.[19]

3-(3,4-dichlorophenyl)prop-2-yn-1-ol

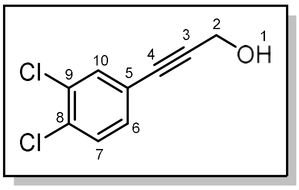

Prepared according to **GP 1** with 3,4-dichloroiodobenzene (1.57 g, 5.75 mmol, 1.0 equiv.). Purification by flash column chromatography (SiO_2, 0-20% v/v EtOAc in petroleum ether) afforded the title compound as an orange solid (1.12 g, 5.57 mmol, 97% yield).

^{1}H NMR (700 MHz, $CDCl_3$): δ 7.51 (s, 1H, H-10), 7.38 (d, J = 8.2 Hz, 1H, H-7), 7.25 (d, J = 8.2 Hz, 1H, H-6), 4.48 (d, J = 5.9 Hz, 2H, H-2), 1.80 (t, J = 6.2 Hz, 1H, H-1).

^{13}C NMR (176 MHz, $CDCl_3$): δ 133.4, 133.1, 132.7, 130.9, 130.5, 122.6, 89.3, 83.6, 51.6.

R_f = 0.40 (30% v/v EtOAc in petroleum ether).

The spectroscopic data is in agreement with that reported in the literature.[19]

3-(4-(tert-butyl)phenyl)prop-2-yn-1-ol

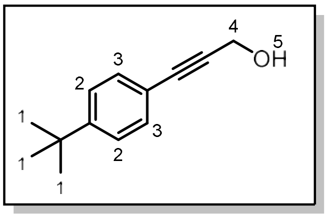

Prepared according to **GP 1** with 1-(*tert*-butyl)-4-iodobenzene (0.89 mL, 5.0 mmol, 1.0 equiv.). Purification by flash column chromatography (SiO_2, 0-10% v/v EtOAc in petroleum ether) afforded the title compound as a yellow solid (909 mg, 4.83 mmol, 97% yield).

^{1}H NMR (400 MHz, $CDCl_3$): δ 7.38 (d, J = 8.7 Hz, 2H, H-3), 7.33 (d, J = 8.7 Hz, 2H, H-2), 4.49 (d, J = 6.2 Hz, 2 H, H-4), 1.69 (t, J = 6.2 Hz, 1 H, H-5), 1.31 (s, 9 H, H-1).

^{13}C NMR (101 MHz, $CDCl_3$): δ 151.9, 131.6, 125.5, 119.6, 86.6, 86.0, 51.9, 34.9, 31.3.

R_f = 0.29 (20% v/v EtOAc in petroleum ether).

The spectroscopic data is in agreement with that reported in the literature.[20]

3-(4-(trifluoromethoxy)phenyl)prop-2-yn-1-ol

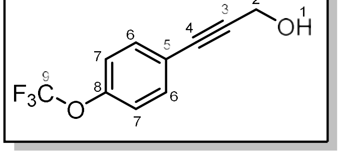

Prepared according to **GP 1** with 4-(trifluoromethoxy)iodobenzene (0.78 mL, 5.0 mmol, 1.0 equiv.). Purification by flash column chromatography (SiO_2, 0-20% v/v EtOAc in petroleum ether) afforded the title compound as an orange oil (1.05 g, 4.86 mmol, 97% yield).

^{1}H NMR (700 MHz, $CDCl_3$): δ 7.45 (d, *J* = 8.5 Hz, 2H, H-6), 7.16 (d, *J* = 8.4 Hz, 2H, H-7), 4.50 (d, *J* = 6.0 Hz, 2H, H-2), 1.83 (t, *J* = 6.2 Hz, 1H, H-1).

^{13}C NMR (176 MHz, $CDCl_3$): δ 149.2, 133.4, 121.4, 121.0, 120.5 (q, $J_{C\text{-}F}$ = 258.3 Hz), 88.2, 84.5, 51.7.

^{19}F NMR (376 MHz, $CDCl_3$): δ -57.8.

R_f = 0.29 (20% v/v EtOAc in petroleum ether).

The spectroscopic data is in agreement with that reported in the literature.[21]

3-(4-chlorophenyl)prop-2-yn-1-ol

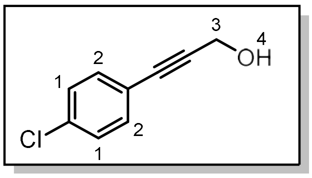

Prepared according to **GP 1** with 1-chloro-4-iodobenzene (1.19 g, 5.0 mmol, 1.0 equiv.). Purification by flash column chromatography (SiO_2, 0-25% v/v EtOAc in petroleum ether) afforded the title compound as a yellow solid (813 mg, 4.87 mmol, 98% yield).

^{1}H NMR (400 MHz, $CDCl_3$): δ 7.36 (d, *J* = 8.5 Hz, 2H, H-2), 7.29 (d, *J* = 8.4 Hz, 2H, H-1), 4.49 (d, *J* = 6.2 Hz, 2H, H-3), 1.72 (t, *J* = 6.1 Hz, 1H, H-4).

^{13}C NMR (101 MHz, $CDCl_3$): δ 134.7, 133.1, 128.8, 121.2, 88.3, 84.8, 51.8.

R_f = 0.22 (20% v/v EtOAc in petroleum ether).

The spectroscopic data is in agreement with that reported in the literature.[15]

3-(4-(trifluoromethyl)phenyl)prop-2-yn-1-ol

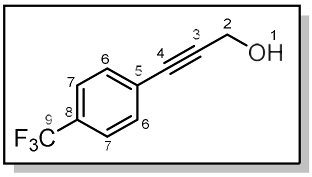

Prepared according to **GP 1** with 4-iodobenzotrifluoride (0.73 mL, 5.0 mmol, 1.0 equiv.). Purification by flash column chromatography (SiO_2, 0-20% v/v EtOAc in petroleum ether) afforded the title compound as a brown solid (955 mg, 4.77 mmol, 95% yield).

^{1}H NMR (700 MHz, $CDCl_3$): δ 7.57 (d, *J* = 8.2 Hz, 2H, H-7), 7.53 (d, *J* = 8.2 Hz, 2H, H-6), 4.52 (d, *J* = 6.1 Hz, 2H, H-2), 1.78 (t, *J* = 6.2 Hz, 1H, H-1).

^{13}C NMR (176 MHz, $CDCl_3$): δ 132.1, 130.4 (q, $J_{C\text{-}F}$ = 32.7 Hz), 126.5, 125.4 (q, $J_{C\text{-}F}$ = 3.5 Hz), 124.0 (q, $J_{C\text{-}F}$ = 273.0 Hz), 89.7, 84.5, 51.7.

^{19}F NMR (376 MHz, $CDCl_3$): δ -63.9.

R_f = 0.41 (30% v/v EtOAc in petroleum ether).

The spectroscopic data is in agreement with that reported in the literature.[19]

3-(naphthalen-1-yl)prop-2-yn-1-ol

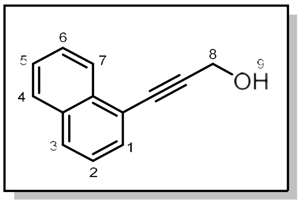

Prepared according to **GP 1** with 1-iodonaphthalene (0.73 mL, 5.0 mmol, 1.0 equiv.). Purification by flash column chromatography (SiO_2, 0-12.5% v/v EtOAc in petroleum ether) afforded the title compound as a light brown solid (879 mg, 4.82 mmol, 96% yield).

^{1}H NMR (500 MHz, $CDCl_3$): δ 8.33 (d, J = 8.9 Hz, 1 H, H-7), 7.85 (t, J = 7.5 Hz, 2 H, H-3, H-4), 7.68 (dd, J = 7.2, 1.3 Hz, 1 H, H-1), 7.57 (ddd, J = 8.3, 6.8, 1.4 Hz, 1 H, H-6), 7.52 (ddd, J = 8.2, 6.9, 1.4 Hz, 1 H, H-5), 7.42 (dd, J = 8.4, 7.2 Hz, 1 H, H-2), 4.66 (d, J = 6.2 Hz, 2 H, H-8), 1.83 (t, J = 6.3 Hz, 1 H, H-9).

^{13}C NMR (126 MHz, $CDCl_3$): δ 133.4, 133.3, 130.8, 129.2, 128.4, 127.0, 126.6, 126.2, 125.3, 120.3, 92.2, 84.0, 52.0.

R_f = 0.23 (20% v/v EtOAc in petroleum ether).

The spectroscopic data is in agreement with that reported in the literature.[22]

5-iodo-1H-indole-3-carbaldehyde

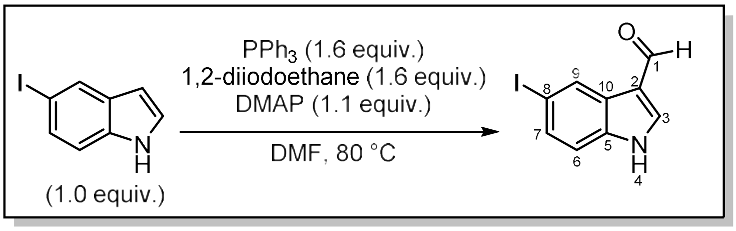

Prepared according to the protocol reported by Xiao and co-workers with slight modifications.[23] A 250 mL round-bottomed flask was charged with 5-iodo-1H-indole (2.30 g, 9.46 mmol, 1.0 equiv.), triphenyl phosphine (4.03 g, 15.4 mmol, 1.6 equiv.), 1,2-diiodoethane (4.31 g, 15.3 mmol, 1.6 equiv.) and DMAP (1.28 g, 10.5 mmol, 1.1 equiv.) under air. At room temperature, DMF (20 mL, 0.47 M) was carefully added to the flask due to the release of ethylene upon mixing of the reagents with the solvent. The reaction mixture was stirred at 80 ˚C for 2 h, followed by the addition of distilled water (50 mL). The reaction mixture was stirred at 80 ˚C for an additional 2 h and cooled to room temperature. Aqueous brine (50 mL) was added to the reaction mixture and the aqueous phase was extracted with

EtOAc. The combined organic layers were dried over $MgSO_4$ and concentrated under reduced pressure. Purification of the crude residue by flash column chromatography (SiO_2, 0-25% v/v acetone in petroleum ether) afforded the title compound as a white solid (2.20 g, 8.12 mmol, 86% yield).

^{1}H NMR (700 MHz, $(CD_3)_2SO$): δ 12.3 (s, 1H, H-4), 9.92 (s, 1H, H-1), 8.43 (d, J = 1.6 Hz, 1H, H-9), 8.29 (s, 1H, H-3), 7.54 (dd, J = 8.5, 1.7 Hz, 1H, H-7), 7.37 (d, J = 8.4 Hz, 1H, H-6).

^{13}C NMR (176 MHz, $(CD_3)_2SO$): δ 185.1, 138.9, 136.1, 131.5, 129.1, 126.6, 117.1, 114.9, 86.5.

R_f = 0.39 (30% v/v acetone in petroleum ether).

The spectroscopic data is in agreement with that reported in the literature.[23]

5-iodo-1-tosyl-1H-indole-3-carbaldehyde

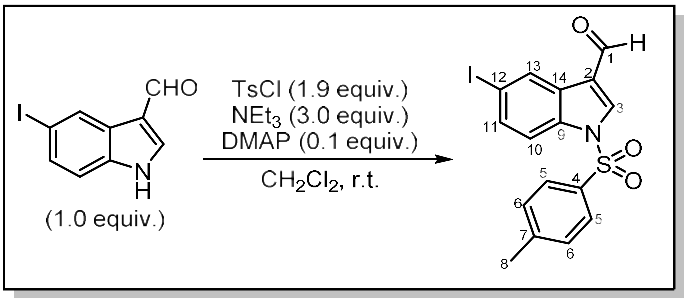

To a solution of 5-iodo-1*H*-indole-3-carbaldehyde (2.20 g, 8.12 mmol, 1.0 equiv.) and DMAP (106 mg, 0.87 mmol, 0.1 equiv.) in anhydrous THF (46 mL, 0.18 M) was added triethylamine (3.4 mL, 24.3 mmol, 3.0 equiv.). The reaction mixture was cooled to 0 ˚C and tosylchloride (2.87 g, 15.1 mmol, 1.9 equiv.) was added in one portion. The reaction mixture was allowed to warm to room temperature overnight. The solvent was removed under reduced pressure. MeOH (50 mL) was added and the reaction mixture was heated to reflux for 10 min. The solvent was removed under reduced pressure and the crude residue was suspended in acetone. The solid was filtered and washed with acetone. The filtrate was concentrated under reduced pressure and the crude residue was suspended in acetone, filtered and washed with acetone. The combined filtered solids were dissolved in $CHCl_3$. The organic layer was washed with water, dried over $MgSO_4$ and concentrated under reduced pressure to afford the title compound as a white solid (2.92 g, 6.87 mmol, 85% yield).

^{1}H NMR (700 MHz, $CDCl_3$): δ 10.0 (s, 1H, H-1), 8.63 (d, J = 1.2 Hz, 1H, H-13), 8.17 (s, 1H, H-3), 7.82 (d, J = 8.4 Hz, 2H, H-5), 7.71-7.68 (m, 2H, H-10, H-11), 7.31 (d, J = 8.4 Hz, 2H, H-6), 2.39 (s, 3H, H-8).

^{13}C NMR (126 MHz, CDCl$_3$): δ 185.1, 146.6, 136.5, 135.1, 134.6, 134.2, 131.6, 130.6, 128.4, 127.4, 121.4, 115.1, 89.8, 21.8.

HRMS (+ESI): *m/z* found [M+H]$^+$ 425.9670, [$C_{16}H_{13}NIO_3S$]$^+$ requires 425.9655, (δ = +3.5 ppm).

methyl 5-iodo-1-tosyl-1H-indole-3-carboxylate

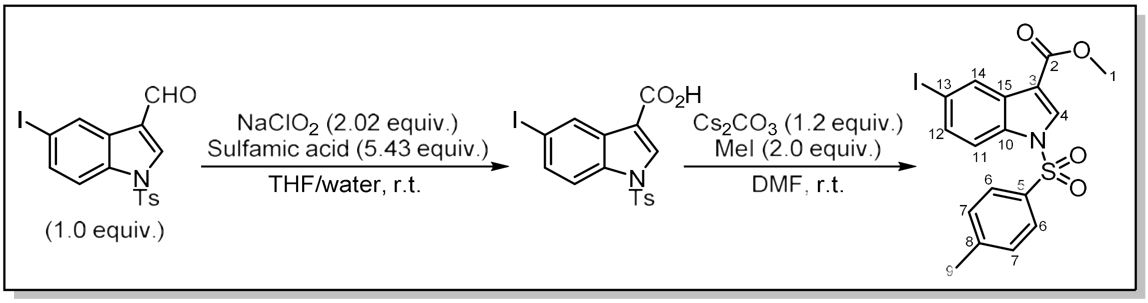

To a solution of 5-iodo-1-tosyl-1*H*-indole-3-carbaldehyde (1.00 g, 2.35 mmol, 1.0 equiv.) in THF (16 mL, 0.15 M) was added a solution of sodium chlorite (430 mg, 4.75 mmol, 2.02 equiv.) and sulfamic acid (1.24 g, 12.8 mmol, 5.43 equiv.) in water (15 mL). The reaction mixture was stirred at room temperature for 1 h and basified with aqueous saturated $NaHCO_3$ to pH 8. The volatiles were removed under reduced pressure and the resultant suspension was diluted with EtOAc and acidified with 6 M HCl to pH 2. The phases were separated and the aqueous phase was extracted with EtOAc. The combined organic layers were dried over $MgSO_4$ and concentrated under reduced pressure to give a white solid (1.10 g), which was dissolved in anhydrous DMF (12 mL). Cs_2CO_3 (975 mg, 2.99 mmol, 1.2 equiv.) was added to the reaction mixture and allowed to stir at room temperature for 10 min. MeI (0.31 mL, 4.99 mmol, 2.0 equiv.) was added to the reaction mixture, which was stirred at room temperature overnight. The reaction mixture was quenched with brine (20 mL) and the aqueous phase was extracted with EtOAc. The combined organic layers were washed with 10% w/v aqueous LiCl, brine, dried over $MgSO_4$ and concentrated under reduced pressure. Purification of the crude residue by flash column chromatography (SiO_2, 0-30% v/v EtOAc in petroleum ether) afforded the title compound as a white solid (830 mg, 1.82 mmol, 78% yield over two steps).

^{1}H NMR (700 MHz, CDCl$_3$): δ 8.48 (d, J = 1.5 Hz, 1H, H-14), 8.21 (s, 1H, H-4), 7.79 (d, J = 8.4 Hz, 2H, H-6), 7.72 (d, J = 8.6 Hz, 1H, H-11), 7.64 (dd, J = 8.8, 1.7 Hz, 1H, H-12), 7.28 (d, J = 8.1 Hz, 2H, H-7), 3.92 (s, 3H, H-1), 2.37 (s, 3H, H-9).

^{13}C NMR (126 MHz, CDCl$_3$): δ 163.8, 146.3, 134.5, 134.2, 134.1, 132.7, 131.2, 130.4, 129.9, 127.3, 115.2, 112.8, 89.2, 51.9, 21.8.

R_f = 0.69 (30% v/v EtOAc in petroleum ether).

HRMS (+ESI): *m/z* found $[M+H]^+$ 455.9760, $[C_{17}H_{15}NIO_4S]^+$ requires 455.9761, (δ = -0.2 ppm).

methyl 5-(3-hydroxyprop-1-yn-1-yl)-1-tosyl-1H-indole-3-carboxylate

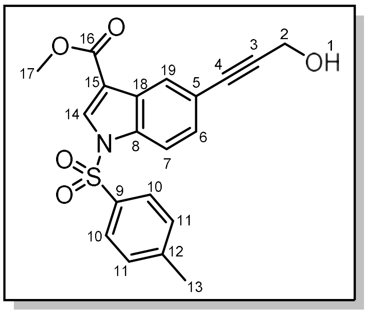

Prepared according to **GP 1** with methyl 5-iodo-1-tosyl-1*H*-indole-3-carboxylate (913 mg, 2.01 mmol, 1.0 equiv.). Purification by flash column chromatography (SiO_2, 0-35% v/v acetone in petroleum ether) afforded the title compound as a yellow solid (706 mg, 1.84 mmol, 92% yield).

^{1}H NMR (400 MHz, $CDCl_3$): δ 8.27 (s, 1H, H-14), 8.21 (d, *J* = 1.0 Hz, 1H, H-19), 7.89 (d, *J* = 8.7 Hz, 1H, H-7), 7.81 (d, *J* = 8.4 Hz, 2H, H-10), 7.41 (dd, *J* = 8.7, 1.7 Hz, 1H, H-6), 7.27 (d, *J* = 8.5 Hz, 2H, H-11), 4.50 (d, *J* = 6.0 Hz, 2H, H-2), 3.92 (s, 3H, H-17), 2.37 (s, 3H, H-13), 1.74 (t, *J* = 6.1 Hz, 1H, H-1).

^{13}C NMR (101 MHz, $CDCl_3$): δ 163.9, 146.2, 134.6, 134.5, 133.0, 130.4, 128.9, 127.8, 127.3, 125.9, 118.9, 113.53, 113.48, 87.1, 85.7, 51.9, 51.8, 21.8.

R_f = 0.36 (30% v/v acetone in petroleum ether).

HRMS (+ESI): *m/z* found $[M+H]^+$ 384.0913, $[C_{20}H_{18}NO_5S]^+$ requires 384.0900, (δ = +3.4 ppm).

4-(4-(tert-butyl)phenyl)but-3-yn-2-ol

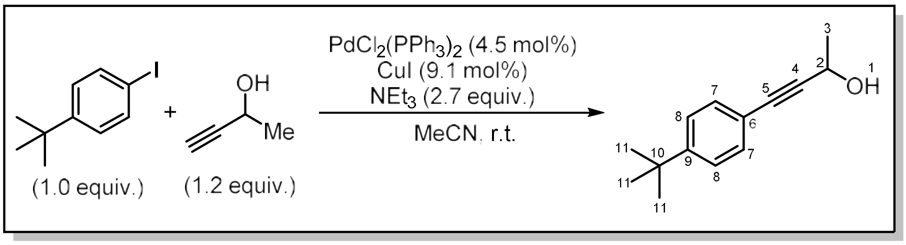

To a solution of 1-(*tert*-butyl)-4-iodobenzene (1.30 g, 5.0 mmol, 1.0 equiv.) in MeCN (20 mL, 0.25 M) was added $PdCl_2(PPh_3)_2$ (157.9 mg, 0.23 mmol, 4.5 mol%), CuI (86.7 mg, 0.46 mmol, 9.1 mol%) and Et_3N (1.9 mL, 13.5 mmol, 2.7 equiv.) at room temperature under a nitrogen atmosphere. The reaction mixture was allowed to stir at room temperature for 20 min before the addition of 3-butyn-2-ol (0.47 mL, 6.0 mmol, 1.2 equiv.). The resultant reaction

mixture was stirred at room temperature overnight (~16 h) and the solvent was removed under reduced pressure. Purification of the crude residue by flash column chromatography (SiO_2, 0-14% v/v EtOAc in petroleum ether) afforded the title compound as an off white solid (1.00 g, 4.96 mmol, 99% yield).

^{1}H NMR (700 MHz, $CDCl_3$): *δ* 7.36 (d, *J* = 8.2 Hz, 2H, H-7), 7.32 (d, *J* = 8.2 Hz, 2H, H-8), 4.76-4.73 (m, 1H, H-2), 1.92 (d, *J* = 5.3 Hz, 1H, H-1), 1.55 (d, *J* = 6.7 Hz, 3H, H-3), 1.31 (s, 9H, H-11).

^{13}C NMR (126 MHz, $CDCl_3$): *δ* 151.8, 131.5, 125.4, 119.7, 90.4, 84.3, 59.1, 34.9, 31.3, 24.6.

R_f = 0.35 (15% v/v EtOAc in petroleum ether).

The spectroscopic data is in agreement with that reported in the literature.[24]

1-(4-(tert-butyl)phenyl)-4-methylpent-1-yn-3-ol

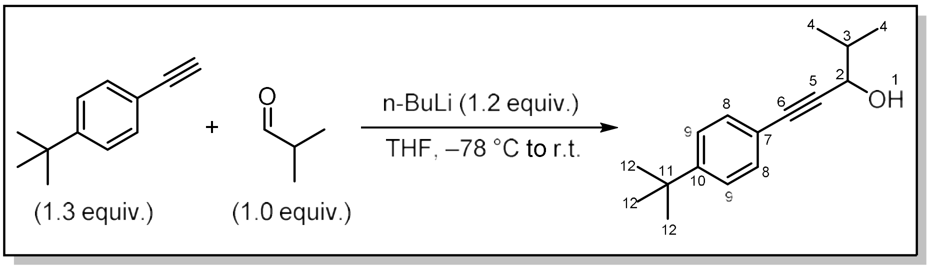

To a solution of 4-(*tert*)-butylphenylacetylene (1.45 mL, 8.02 mmol, 1.3 equiv.) in anhydrous THF (16 mL) at -78 ˚C was added *n*-BuLi (4.5 mL, 1.6 M in hexanes, 7.20 mmol, 1.2 equiv.) dropwise. The reaction mixture was allowed to stir at -78 ˚C for 30 min before the dropwise addition of isobutyraldehyde (0.55 mL, 6.00 mmol, 1.0 equiv.) at -78 ˚C. The reaction mixture was allowed to warm to room temperature overnight and was quenched with aqueous saturated NH_4Cl. The volatiles were removed under reduced pressure and the aqueous phase was extracted with diethyl ether. The combined organic layers were dried over Na_2SO_4, and the solvent removed under reduced pressure. Purification of the crude residue by flash column chromatography (SiO_2, 0-10% v/v EtOAc in petroleum ether) afforded the title compound as a yellow solid (1.38 g, 5.99 mmol, quant.).

^{1}H NMR (400 MHz, $CDCl_3$): *δ* 7.38 (d, *J* = 8.6 Hz, 2H, H-8), 7.33 (d, *J* = 8.7 Hz, 2H, H-9), 4.39 (t, *J* = 5.7 Hz, 1H, H-2), 2.01-1.93 (m, 1H, H-3), 1.88 (d, *J* = 5.8 Hz, 1H, H-1), 1.31 (s, 9H, H-12), 1.08 (d, *J* = 8.9 Hz, 3H, H-4a), 1.05 (d, *J* = 8.9 Hz, 3H, H-4b).

^{13}C NMR (101 MHz, $CDCl_3$): *δ* 151.8, 131.6, 125.4, 119.8, 88.3, 85.8, 68.6, 34.90, 34.89, 31.3, 18.3, 17.7.

R_f = 0.32 (10% v/v EtOAc in petroleum ether).

HRMS (+ESI): *m/z* found $[M-OH]^+$ 213.1641, $[C_{16}H_{21}]^+$ requires 213.1638, (δ = +1.4 ppm).

7.2 Synthesis of Substrates – α,β-Unsaturated Esters

ethyl (Z)-3-iodoacrylate

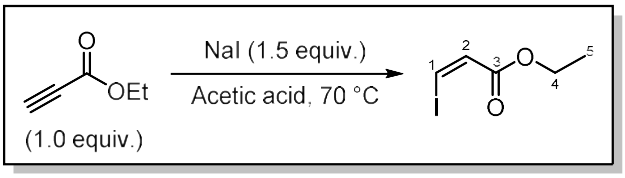

Prepared according to the protocol reported by Schoenebeck and co-workers.[25] A stirred suspension of sodium iodide (previously dried at 100 ˚C for 12 h under vacuum, 22.5 g, 150 mmol, 1.5 equiv.) in acetic acid (100 mL) was heated to 70 ˚C until all the solid material dissolved. Ethyl propiolate (10 mL, 100 mmol, 1.0 equiv.) was added to the reaction mixture at 70 ˚C and the resultant reaction mixture was stirred at 70 ˚C for 19 h. The reaction mixture was cooled to room temperature and diethyl ether (100 mL) and water (100 mL) were added. The organic layer was separated and the aqueous layer was extracted with diethyl ether (3 x 50 mL). The pH of the combined organic layers was adjusted to 7 with 3 M aqueous KOH and the organic layers were washed with 10% aqueous $Na_2S_2O_3$ (30 mL), brine (30 mL) and dried over $MgSO_4$. Removal of the solvent under reduced pressure afforded the title compound as a yellow oil (21.9 g, 96.9 mmol, 96% yield).

^{1}H NMR (700 MHz, CDCl$_3$): δ 7.43 (d, *J* = 8.9 Hz, 1H, H-1), 6.88 (d, *J* = 8.7 Hz, 1H, H-2), 4.24 (q, *J* = 7.2 Hz, 2H, H-4), 1.31 (t, *J* = 7.1 Hz, 3H, H-5).

^{13}C NMR (176 MHz, CDCl$_3$): δ 164.7, 130.0, 94.8, 60.9, 14.3.

The spectroscopic data is in agreement with that reported in the literature.[25]

(Z)-3-iodoprop-2-en-1-ol

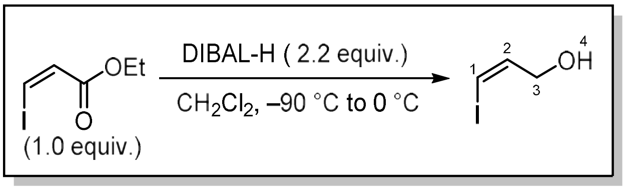

Prepared according to the protocol reported by Schoenebeck and co-workers.[25] To a solution of ethyl (*Z*)-3-iodoacrylate (3.45 g, 15.3 mmol, 1.0 equiv.) in CH_2Cl_2 (18 mL, 0.85 M)

was added DIBAL-H (33 mL, 33.0 mmol, 1.0 M in hexane, 2.2 equiv.) dropwise at -90 ˚C. The reaction mixture was warmed to 0 ˚C and stirred at this temperature for 1 h. The reaction mixture was quenched with the dropwise addition aqueous 1 M HCl (20 mL). The phases were separated and the aqueous phase was extracted with diethyl ether. The combined organic layers were dried over Na_2SO_4 and concentrated under reduced pressure. The crude residue was filtered over a silica plug and eluted with diethyl ether to afford the title compound as a yellow oil (2.41 g, 13.1 mmol, 86% yield)

^{1}H NMR (500 MHz, $CDCl_3$): δ 6.49 (dt, J = 7.7, 5.7 Hz, 1H, H-2), 6.36 (dt, J = 7.7, 1.5 Hz, 1H, H-1), 4.24 (dd, J = 5.8, 1.5 Hz, 2H, H-3), 1.78 (s, 1H, H-4).

^{13}C NMR (126 MHz, $CDCl_3$): δ 140.1, 82.8, 65.7.

The spectroscopic data is in agreement with that reported in the literature.[25]

General Procedure 2 (GP 2):

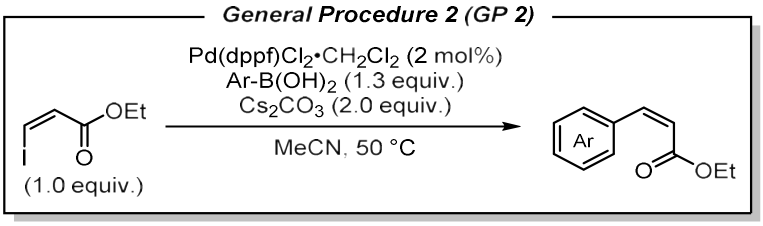

An oven-dried round-bottomed flask was charged with cesium carbonate (3.26 g, 10.0 mmol, 2.0 equiv.), aryl boronic acid (6.5 mmol, 1.3 equiv.) and Pd(dppf)Cl_2•CH_2Cl_2 (81.2 mg, 0.10 mmol, 2 mol%) under a nitrogen atmosphere. A solution of ethyl (*Z*)-3-iodoacrylate (1.13 g, 5.0 mmol, 1.0 equiv.) in anhydrous MeCN (33 mL, 0.15 M) was added to the reaction mixture. The resultant reaction mixture was stirred at 50 ˚C overnight (~16 h) and allowed to cool to room temperature. The reaction mixture was diluted with EtOAc, filtered over Celite® and eluted with EtOAc. The filtrate was concentrated under reduced pressure. Purification of the crude residue by flash column chromatography afforded the corresponding α,β-unsaturated ester product.

ethyl (Z)-3-(2-methoxyphenyl)acrylate

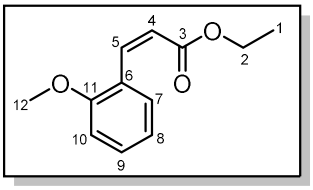

Prepared according to **GP 2** with ethyl (*Z*)-3-iodoacrylate (1.15 g, 5.09 mmol, 1.0 equiv.) and 2-methoxyphenyl boronic acid (996 mg, 6.55 mmol, 1.3 equiv.). Purification by flash column chromatography (SiO_2, 0-12.5% v/v diethyl ether in petroleum ether) afforded the title compound as a colourless oil (683 mg, 3.31 mmol, 65% yield).

^{1}H NMR (400 MHz, $CDCl_3$): δ 7.54 (dd, *J* = 7.6, 1.4 Hz, 1H, H-7), 7.33-7.29 (m, 1H, H-9), 7.17 (d, *J* = 12.5 Hz, 1H, H-5), 6.93 (t, *J* = 7.6 Hz, 1H, H-8), 6.88 (d, *J* = 8.4 Hz, 1H, H-10), 5.97 (d, *J* = 12.5 Hz, 1H, H-4), 4.14 (q, *J* = 7.2 Hz, 2H, H-2), 3.84 (s, 3H, H-12), 1.20 (t, *J* = 7.3 Hz, 3H, H-1).

^{13}C NMR (101 MHz, $CDCl_3$): δ 166.5, 157.2, 139.1, 130.8, 130.5, 124.3, 120.2, 120.0, 110.4, 60.2, 55.6, 14.2.

R_f = 0.47 (20% v/v diethyl ether in petroleum ether).

The spectroscopic data is in agreement with that reported in the literature.[26]

ethyl (Z)-3-(naphthalen-2-yl)acrylate

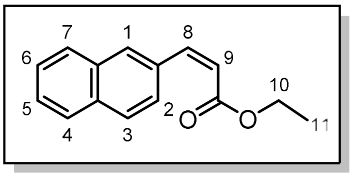

Prepared according to **GP 2** with ethyl (*Z*)-3-iodoacrylate (1.13 g, 5.00 mmol, 1.0 equiv.) and 2-naphthylboronic acid (1.12 g, 6.50 mmol, 1.3 equiv.). Purification by flash column chromatography (SiO_2, 0-2% v/v EtOAc in petroleum ether) afforded the title compound as a white solid (601 mg, 2.66 mmol, 53% yield, 91% purity) with an unknown impurity. This compound was taken forward without further purification.

^{1}H NMR (500 MHz, $CDCl_3$): δ 8.04 (s, 1H, H-1), 7.86–7.78 (m, 3H, H-3, H-4, H-7), 7.73 (dd, *J* = 8.6, 1.7 Hz, 1H, H-2), 7.51-7.46 (m, 2H, H-5, H-6), 7.10 (d, *J* = 12.6 Hz, 1H, H-8), 6.03 (d, *J* = 12.6 Hz, 1H, H-9), 4.21 (q, *J* = 7.1 Hz, 2H, H-10), 1.25 (t, *J* = 7.2 Hz, 3H, H-11).

^{13}C NMR (126 MHz, $CDCl_3$): δ 166.5, 143.1, 133.6, 133.1, 132.6, 130.0, 128.6, 127.7, 127.6, 127.1, 126.9, 126.3, 120.2, 60.5, 14.3.

R_f = 0.38 (5% v/v EtOAc in petroleum ether).

The spectroscopic data is in agreement with that reported in the literature.[27]

ethyl (Z)-3-(thiophen-3-yl)acrylate

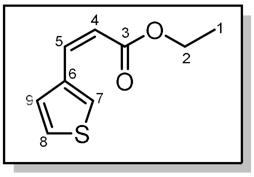

Prepared according to **GP 2** with ethyl (*Z*)-3-iodoacrylate (1.22 g, 5.40 mmol, 1.0 equiv.) and 3-thienylboronic acid (843.6 mg, 6.60 mmol, 1.2 equiv.). Purification by flash column chromatography (SiO_2, 0-1% v/v EtOAc in petroleum ether) afforded the title compound as a pale-yellow oil (666 mg, 3.65 mmol, 68% yield).

^{1}H NMR (400 MHz, $CDCl_3$): δ 8.07 (d, J = 2.9 Hz, 1H, H-7), 7.54 (dd, J = 5.1, 1.0 Hz, 1H, H-8), 7.27-7.25 (m, 1H, H-9), 6.87 (d, J = 12.8 Hz, 1H, H-5), 5.83 (d, J = 12.6 Hz, 1H, H-4), 4.21 (q, J = 7.1 Hz, 2H, H-2), 1.30 (t, J = 7.2 Hz, 3H, H-1).

^{13}C NMR (101 MHz, $CDCl_3$): δ 166.4, 136.7, 136.3, 130.4, 130.3, 125.0, 117.1, 60.3, 14.4.

R_f = 0.68 (10% v/v EtOAc in petroleum ether).

The spectroscopic data is in agreement with that reported in the literature.[27]

(Z)-3-(3-methoxyphenyl)prop-2-en-1-ol

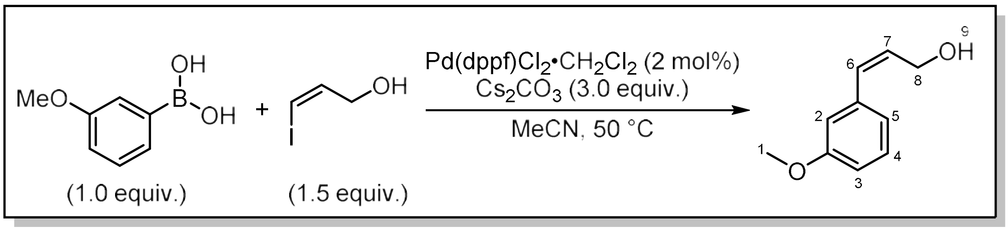

An oven-dried 100 mL round bottom flask equipped with a Teflon-coated magnetic stir bar was charged with caesium carbonate (3.26 g, 10.0 mmol, 2.00 equiv.), (3-methoxyphenyl)boronic acid (0.760 g, 5.00 mmol, 1.00 equiv.) and $Pd(dppf)_2Cl_2{\bullet}CH_2Cl_2$ (81.7 mg, 0.100 mmol, 2.00 mol%). The flask was sealed and evacuated and backfilled with N_2 three times. (*Z*)-3-iodoprop-2-en-1-ol (1.38 g, 7.50 mmol, 1.50 equiv.) dissolved in dry MeCN (33 mL) was added and the reaction mixture was heated at 50 °C for 22 h. After cooling to room temperature, the reaction was mixture was diluted with EtOAc and filtered over Celite®, eluting with further EtOAc. The filtrate was collected, the solvent was removed under reduced pressure and purification of the crude residue by flash column chromatography (SiO_2, 0-25% v/v EtOAc in Petrol) afforded the title compound as a light-yellow oil (0.233 g, 1.42 mmol, 28%).

^{1}H NMR (400 MHz, $CDCl_3$): δ 7.26 (t, J = 8.2 Hz, 1H, H-4), 6.85–6.78 (m, 2H, H-3, H-5), 6.78–6.72 (m, 1H, H-2), 6.55 (dt, J = 11.7, 1.9 Hz, 1H, H-6), 5.87 (dt, J = 11.7, 6.4 Hz, 1H, H-7), 4.44 (t, J = 5.1 Hz, 2H, H-8), 3.81 (s, 3H, H-1), 1.50 (t, J = 5.4 Hz, 1H, H-9).

^{13}C NMR (126 MHz, CDCl$_3$): δ 159.6, 138.0, 131.6, 131.1, 129.4, 121.4, 114.5, 112.9, 59.9, 55.4.

R$_f$ = 0.32 (30% v/v EtOAc in petroleum ether).

The spectroscopic data is in agreement with that reported in the literature.[28]

7.3 Synthesis of Substrates – N-H Allylic Carbamates

General Procedure 3 (GP 3):

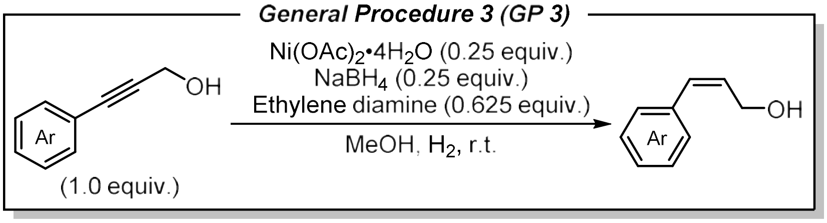

An oven-dried round-bottomed flask was charged with Ni(OAc)$_2$•4H$_2$O (311 mg, 1.25 mmol, 0.25 equiv.) and anhydrous MeOH (13 mL, 0.1 M). The reaction mixture was evacuated until the solvent began to boil and backfilled with a balloon of hydrogen gas. This process was repeated twice following which the reaction mixture was left stirring under an atmosphere of hydrogen gas. The reaction mixture was cooled to 0 ˚C followed by the addition of NaBH$_4$ (47.3 mg, 1.25 mmol, 0.25 equiv.) in one portion. The resultant black mixture was allowed to warm to room temperature over 15 min. Ethylenediamine (0.21 mL, 3.13 mmol, 0.63 equiv.) was added, followed by a solution of the propargyl alcohol (5.0 mmol, 1.0 equiv.) in MeOH (10 mL, 0.5 M). The reaction mixture was allowed to stir at room temperature and the reaction progress was monitored by TLC analysis. Upon reaction completion, the reaction mixture was filtered through Celite® and eluted with EtOAc. The solvent was removed under reduced pressure and the crude residue was passed through a short silica plug and eluted with EtOAc. The resultant crude residue was taken forward to the next step without further purification.

General Procedure 4 (GP 4):

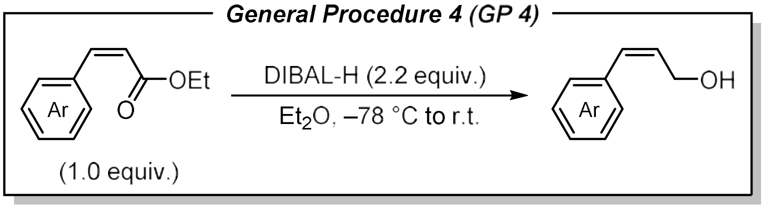

An oven-dried round-bottomed flask was charged with the α,β-unsaturated ester (3.0 mmol, 1.0 equiv.) and anhydrous diethyl ether (20 mL, 0.15 M). The reaction mixture was cooled to -78 ˚C and DIBAL-H (6.6 mL, 1 M in hexanes, 6.6 mmol, 2.2 equiv.) was added dropwise to

the reaction mixture over 5 min. The reaction mixture was allowed to stir at -78 ˚C for an additional 3 h and was gradually warmed to room temperature. The reaction mixture was allowed to stir at room temperature for 1 h and cooled to 0 ˚C. The reaction mixture was quenched with saturated aqueous NH_4Cl (7 mL) at 0 ˚C and stirred at room temperature overnight. The crude reaction mixture was filtered over Celite® and eluted with diethyl ether. The filtrate was dried over $MgSO_4$ and the solvent removed under reduced pressure. The resultant crude residue was taken forward to the next step without further purification.

General Procedure 5 (GP 5):

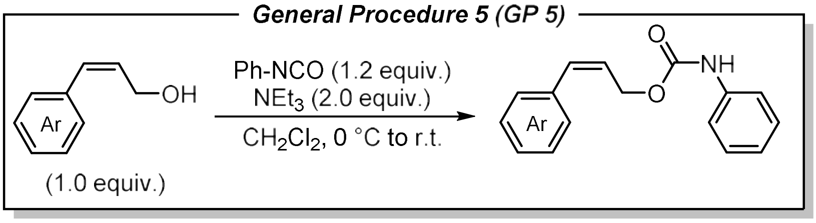

To a solution of allylic alcohol (5.0 mmol, 1.0 equiv.) in CH_2Cl_2 (25 mL, 0.2 M) was added triethylamine (1.4 mL, 10.0 mmol, 2.0 equiv.) under a nitrogen atmosphere. The reaction mixture was cooled to 0 ˚C and phenyl isocyanate (0.66 mL, 6.0 mmol, 1.2 equiv.) was added dropwise. The reaction mixture was warmed to room temperature and the reaction progress was monitored by TLC analysis. Upon reaction completion, the crude residue was directly purified by flash column chromatography to afford the corresponding allylic carbamate product.

(Z)-3-phenylallyl phenylcarbamate (**S1**)

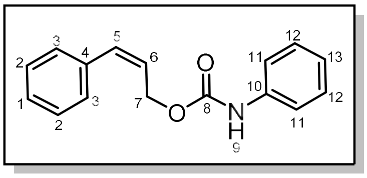

Prepared according to **GP 3**, followed by **GP 5** with 3-phenylprop-2-yn-1-ol (2.64 g, 20.0 mmol, 1.0 equiv.). Purification by flash column chromatography (SiO_2, 0-15% v/v EtOAc in petroleum ether) afforded the title compound as a white solid (4.71 g, 18.6 mmol, 93% yield over two steps).

^{1}H NMR (400 MHz, CDCl$_3$): δ 7.40-7.35 (m, 4H, H-2, H-11), 7.33-7.29 (m, 3H, H-1, H-12), 7.27-7.25 (m, 2H, H-3), 7.09-7.05 (m, 1H, H-13), 6.70 (d, J = 11.7 Hz, 1H, H-5), 6.66 (br s, 1H, H-9), 5.87 (dt, J = 11.7, 6.6 Hz, 1H, H-6), 4.95 (dd, J = 6.6, 1.6 Hz, 2H, H-7).

^{13}C NMR (101 MHz, CDCl$_3$): δ 153.4, 137.9, 136.1, 133.2, 129.2, 128.9, 128.6, 127.7, 126.1, 123.7, 118.8, 62.3.

R_f = 0.33 (10% v/v EtOAc in petroleum ether).

HRMS (+ESI): *m/z* found $[M+Na]^+$ 276.0987, $[C_{16}H_{15}NO_2Na]^+$ requires 276.0995, (δ = -2.9 ppm).

(Z)-3-(2-isopropylphenyl)allyl phenylcarbamate

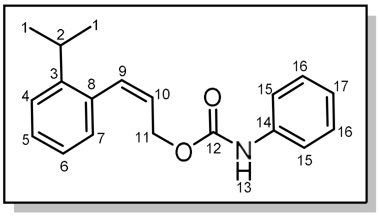

Prepared according to **GP 3**, followed by **GP 5** with 3-(2-isopropylphenyl)prop-2-yn-1-ol (483 mg, 2.77 mmol, 1.0 equiv.). Purification by flash column chromatography (SiO_2, 0-7.5% v/v EtOAc in petroleum ether) afforded the title compound as a white solid (711 mg, 2.41 mmol, 87% yield over two steps).

^{1}H NMR (500 MHz, $CDCl_3$): δ 7.37 (d, *J* = 8.1 Hz, 2H, H-15), 7.32-7.29 (m, 4H, H-4, H-5, H-16), 7.19-7.16 (m, 1H, H-6), 7.10 (d, *J* = 7.5 Hz, 1H, H-7), 7.06 (tt, *J* = 7.4, 1.2 Hz, H, H-17), 6.89 (d, *J* = 11.6 Hz, 1 H, H-9), 6.60 (br s, 1H, H-13), 5.93 (dt, *J* = 11.4, 6.8 Hz, 1H, H-10), 4.77 (dd, *J* = 6.8, 1.4 Hz, 2H, H-11), 3.11 (sept, *J* = 6.9 Hz, 1H, H-2), 1.22 (d, *J* = 6.9 Hz, 6H, H-1).

^{13}C NMR (126 MHz, $CDCl_3$): δ 153.4, 146.9, 138.0, 134.2, 133.0, 129.5, 129.2, 128.3, 126.4, 125.6, 125.1, 123.6, 118.7, 62.2, 30.1, 23.4.

R_f = 0.69 (20% v/v EtOAc in petroleum ether).

HRMS (+ESI): *m/z* found $[M+Na]^+$ 318.1472, $[C_{19}H_{21}NO_2Na]^+$ requires 318.1464, (δ = +2.5 ppm).

(Z)-3-(2-methoxyphenyl)allyl phenylcarbamate

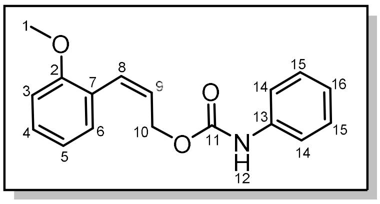

Prepared according to **GP 4**, followed by **GP 5** with ethyl (*Z*)-3-(2-methoxyphenyl)acrylate (683 mg, 3.3 mmol, 1.0 equiv.). Purification by flash column chromatography (SiO_2, 0-12% v/v EtOAc in petroleum ether) afforded the title compound as a pale orange solid (854 mg, 3.01 mmol, 91% yield over two steps).

^{1}H NMR (400 MHz, $CDCl_3$): δ 7.38 (d, *J* = 8.1 Hz, 2H, H-14), 7.32-7.26 (m, 3H, H-4, H-15), 7.17 (dd, *J* = 7.5, 1.3 Hz, 1H, H-6), 7.06 (t, *J* = 7.3 Hz, 1H, H-16), 6.96 (t, *J* = 7.5 Hz, 1H, H-5), 6.90 (d, *J* = 8.3 Hz, 1H, H-3), 6.82 (d, *J* = 11.6 Hz, 1H, H-8), 6.64 (br s, 1H, H-12), 5.91 (dt, *J* = 11.7, 6.8 Hz, 1H, H-9), 4.87 (dd, *J* = 6.8, 1.4 Hz, 2H, H-10), 3.85 (s, 3H, H-1).

^{13}C NMR (101 MHz, $CDCl_3$): δ 157.1, 153.5, 138.0, 130.2, 129.3, 129.18, 129.17, 125.8, 125.0, 123.6, 120.4, 118.8, 110.6, 62.6, 55.6.

R_f = 0.42 (20% v/v EtOAc in petroleum ether).

HRMS (+ESI): *m/z* found $[M+Na]^+$ 306.1099, $[C_{17}H_{17}NO_3Na]^+$ requires 306.1101, (δ = -0.7 ppm).

(Z)-3-(2-fluorophenyl)allyl phenylcarbamate

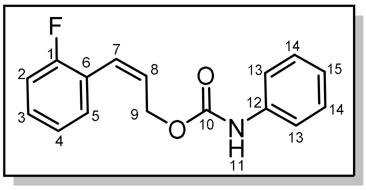

Prepared according to **GP 3**, followed by **GP 5** with 3-(2-fluorophenyl)prop-2-yn-1-ol (751 mg, 5.0 mmol, 1.0 equiv.). Purification by flash column chromatography (SiO_2, 0-10% v/v EtOAc in petroleum ether) afforded the title compound as a white solid (1.14 g, 4.20 mmol, 84% yield over two steps).

^{1}H NMR (400 MHz, $CDCl_3$): δ 7.38 (d, J = 8.0 Hz, 2H, H-13), 7.33-7.27 (m, 3H, H-3, H-14), 7.26-7.22 (m, 1H, H-5), 7.16-7.12 (m, 1H, H-4), 7.10-7.05 (m, 2H, H-2, H-15), 6.71 (d, J = 11.7 Hz, 1H, H-7), 6.63 (br s, 1H, H-11), 5.98 (dt, J = 11.8, 6.6 Hz, 1H, H-8), 4.86 (d, J = 6.6 Hz, 2H, H-9).

^{13}C NMR (101 MHz, $CDCl_3$): δ 160.2 (d, J_{C-F} = 248.9 Hz), 153.4, 137.9, 130.6 (d, J_{C-F} = 3.2 Hz), 129.7 (d, J_{C-F} = 8.2 Hz), 129.2, 128.1 (d, J_{C-F} = 1.1 Hz), 125.8 (d, J_{C-F} = 3.6 Hz), 124.0 (d, J_{C-F} = 3.8 Hz), 123.8 (d, J_{C-F} = 14.7 Hz), 123.7, 118.8, 115.7, (d, J_{C-F} = 21.9 Hz), 62.3.

^{19}F NMR (376 MHz, $CDCl_3$): δ -116.1.

R_f = 0.56 (20% v/v EtOAc in petroleum ether).

HRMS (+ESI): *m/z* found $[M+Na]^+$ 294.0910, $[C_{16}H_{14}FNO_2Na]^+$ requires 294.0901, (δ = +3.1 ppm).

(Z)-3-(3-(tert-butyl)phenyl)allyl phenylcarbamate

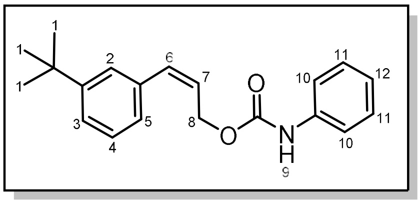

Prepared according to **GP 3**, followed by **GP 5** with 3-(3-(*tert*-butyl)phenyl)prop-2-yn-1-ol (260 mg, 1.38 mmol, 1.0 equiv.). Purification by flash column chromatography (SiO_2, 0-10% v/v EtOAc in petroleum ether) afforded the title compound as a colourless oil (249 mg, 0.804 mmol, 58% yield over two steps).

^{1}H NMR (400 MHz, CDCl$_3$): δ 7.38 (d, J = 8.1 Hz, 2H, H-10), 7.35-7.26 (m, 5H, H-2, H-3, H-4, H-11), 7.09-7.05 (m, 2H, H-5, H-12), 6.72 (d, J = 11.6 Hz, 1H, H-6), 6.63 (br s, 1 H, H-9), 5.86 (dt, J = 11.7, 6.6 Hz, 1H, H-7), 4.96 (dd, J = 6.6, 1.6 Hz, 2H, H-8), 1.34 (s, 9 H, H-1).

^{13}C NMR (101 MHz, CDCl$_3$): δ 151.4, 137.9, 135.8, 133.9, 129.2, 128.3, 126.0, 125.9, 125.7, 124.8, 123.7, 118.9, 62.4, 34.8, 31.5.

R_f = 0.61 (20% v/v EtOAc in petroleum ether).

HRMS (+ESI): *m/z* found $[M+H]^+$ 310.1808, $[C_{20}H_{24}NO_2]^+$ requires 310.1802, (δ = +1.9 ppm).

(Z)-3-(3-methoxyphenyl)allyl phenylcarbamate

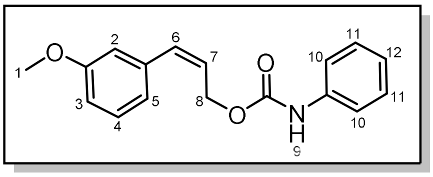

Prepared according to **GP 5** with (*Z*)-3-(3-methoxyphenyl)prop-2-en-1-ol (221 mg, 1.35 mmol, 1.0 equiv.). Purification by flash column chromatography (SiO_2, 0-12% v/v EtOAc in petroleum ether) afforded the title compound as a colourless oil (310 mg, 1.09 mmol, 81% yield).

^{1}H NMR (400 MHz, CDCl$_3$): δ 7.38 (d, J = 7.9 Hz, 2H, H-10), 7.34-7.26 (m, 3H, H-4, H-11), 7.07 (tt, J = 7.5, 1.2 Hz, 1H, H-12), 6.84 (dd, J = 7.9, 2.3 Hz, 2H, H-3, H-5), 6.80-6.79 (m, 1H, H-2), 6.67 (dt, J = 11.7, 1.8 Hz, 1H, H-6), 6.64 (s, 1H, H-9), 5.87 (dt, J = 11.7, 6.7 Hz, 1H, H-7), 4.95 (dd, J = 6.7, 1.7 Hz, 2H, H-8), 3.82 (s, 3H, H-1).

^{13}C NMR (101 MHz, CDCl$_3$): δ 159.7, 153.4, 137.9, 137.5, 133.2, 129.6, 129.2, 126.4, 123.7, 121.4, 118.8, 114.5, 113.3, 62.3, 55.4.

R_f = 0.56 (20% v/v EtOAc in petroleum ether).

HRMS (+ESI): *m/z* found $[M+H]^+$ 284.1289 , $[C_{17}H_{18}NO_3]^+$ requires 284.1281, (δ = +2.8 ppm).

(Z)-3-(3-bromophenyl)allyl phenylcarbamate

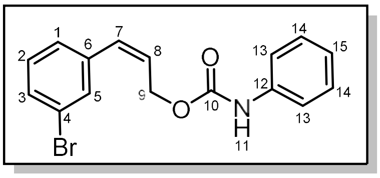

Prepared according to **GP 3**, followed by **GP 5** with 3-(3-bromophenyl)prop-2-yn-1-ol (1.06 g, 5.0 mmol, 1.0 equiv.). Purification by flash column chromatography (SiO_2, 0-10% v/v EtOAc in petroleum ether) afforded the title compound as a light yellow solid (1.34 g, 4.03 mmol, 81% yield over two steps).

^{1}H NMR (500 MHz, CDCl$_3$): δ 7.43-7.41 (m, 1H, H-3), 7.40-7.38 (m, 3H, H-5, H-13), 7.33-7.30 (m, 2H, H-14), 7.24 (t, J = 7.8 Hz, 1H, H-2), 7.18 (d, J = 7.8 Hz, 1H, H-1), 7.08 (tt, J = 7.4, 1.2 Hz, 1H, H-15), 6.64 (br s, 1H, H-11), 6.61 (d, J = 11.8 Hz, 1H, H-7), 5.91 (dt, J = 11.7, 6.8 Hz, 1H, H-8), 4.91 (dd, J = 6.6, 1.5 Hz, 2H, H-9).

^{13}C NMR (126 MHz, CDCl$_3$): δ 153.3, 138.2, 137.8, 131.8, 131.7, 130.7, 130.1, 129.2, 127.6, 127.4, 123.7, 122.7, 118.8, 61.9.

R$_f$ = 0.25 (10% v/v EtOAc in petroleum ether).

HRMS (+ESI): *m/z* found [M+Na]$^+$ 354.0098, [$C_{16}H_{14}NBrO_2Na$]$^+$ requires 354.0100, (δ = -0.6 ppm).

ethyl (Z)-3-(3-((phenylcarbamoyl)oxy)prop-1-en-1-yl)benzoate

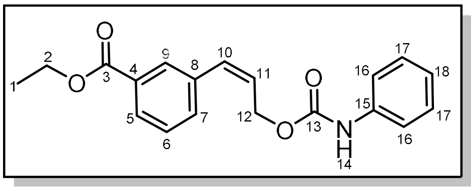

Prepared according to **GP 3**, followed by **GP 5** with ethyl 3-(3-hydroxyprop-1-yn-1-yl)benzoate (1.05 g, 5.1 mmol, 1.0 equiv.). Purification by flash column chromatography (SiO$_2$, 0-20% v/v EtOAc in petroleum ether) afforded the title compound as a pale yellow solid (1.30 g, 4.00 mmol, 78% yield over two steps).

^{1}H NMR (500 MHz, CDCl$_3$): δ 7.99-7.95 (m, 1H, H-5), 7.94 (br s, 1H, H-9), 7.45-7.42 (m, 2H, H-6, H-7), 7.39 (d, J = 7.9 Hz, 2H, H-16), 7.32-7.29 (m, 2H, H-17), 7.07 (tt, J = 7.4, 1.2 Hz, 1H, H-18), 6.72 (d, J = 11.7 Hz, 1H, H-10), 6.72 (br s, 1H, H-14), 5.93 (dt, J = 11.7, 6.7 Hz, 1H, H-11), 4.92 (dd, J = 6.6, 1.4 Hz, 2H, H-12), 4.38 (q, J = 7.2 Hz, 2H, H-2), 1.39 (t, J = 7.1 Hz, 3H, H-1).

^{13}C NMR (126 MHz, CDCl$_3$): δ 166.5, 153.4, 137.9, 136.4, 133.0, 132.4, 130.9, 130.0, 129.2, 128.7, 128.6, 127.3, 123.7, 118.8, 61.9, 61.3, 14.4.

R$_f$ = 0.56 (30% v/v EtOAc in petroleum ether).

HRMS (+ESI): *m/z* found [M+Na]$^+$ 348.1210, [$C_{19}H_{19}NO_4Na$]$^+$ requires 348.1206, (δ = +1.1 ppm).

(Z)-3-(3,4-dichlorophenyl)allyl phenylcarbamate

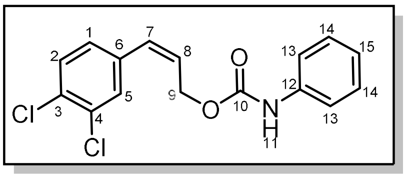

Prepared according to **GP 3**, followed by **GP 5** with 3-(3,4-dichlorophenyl)prop-2-yn-1-ol (1.12 g, 5.57 mmol, 1.0 equiv.). Purification by flash column chromatography (SiO_2, 0-12.5% v/v EtOAc in petroleum ether) afforded the title compound as an off white solid (1.58 g, 4.90 mmol, 88% yield over two steps).

^{1}H NMR (700 MHz, $CDCl_3$): δ 7.43 (d, *J* = 8.2 Hz, 1H, H-2), 7.38 (d, *J* = 6.3 Hz, 2H, H-13), 7.34 (d, *J* = 1.3 Hz, 1H, H-5), 7.31 (t, *J* = 7.9 Hz, 2H, H-14), 7.10-7.07 (m, 2H, H-1, H-15), 6.65 (br s, 1H, H-11), 6.57 (d, *J* = 11.7 Hz, 1H, H-7), 5.93 (dt, *J* = 11.8, 6.5 Hz, 1H, H-8), 4.89 (d, *J* = 6.6 Hz, 2H, H-9).

^{13}C NMR (176 MHz, $CDCl_3$): δ 153.2, 137.7, 136.1, 132.7, 131.8, 130.8, 130.6, 130.5, 129.2, 128.11, 128.09, 123.8, 118.8, 61.7.

R_f = 0.55 (20% v/v EtOAc in petroleum ether).

HRMS (+ESI): *m/z* found $[M+Na]^+$ 344.0228, $[C_{16}H_{13}NCl_2O_2Na]^+$ requires 344.0216, (δ = +3.5 ppm).

(Z)-3-(4-(tert-butyl)phenyl)allyl phenylcarbamate

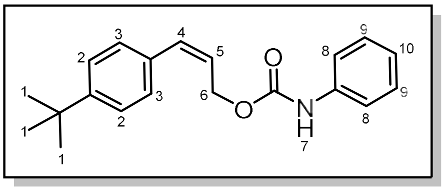

Prepared according to **GP 3**, followed by **GP 5** with 3-(4-(*tert*-butyl)phenyl)prop-2-yn-1-ol (894 mg, 4.75 mmol, 1.0 equiv.). Purification by flash column chromatography (SiO_2, 0-15% v/v EtOAc in petroleum ether) afforded the title compound as a white solid (1.32 g, 4.27 mmol, 90% yield over two steps).

^{1}H NMR (500 MHz, $CDCl_3$): δ 7.40-7.38 (m, 4H, H-2, H-8), 7.33-7.29 (m, 2H, H-9), 7.20 (dt, *J* = 8.2, 2.3 Hz, 2H, H-3), 7.07 (tt, *J* = 7.5, 1.2 Hz, 1H, H-10,), 6.67 (d, *J* = 11.7 Hz, 1H, H-4), 6.63 (br s, 1H, H-7), 5.83 (dt, *J* = 11.7, 6.7 Hz, 1H, H-5), 4.97 (dd, *J* = 6.6, 1.7 Hz, 2H, H-6), 1.33 (s, 9H, H-1).

^{13}C NMR (126 MHz, $CDCl_3$): δ 153.5, 150.8, 137.9, 133.3, 133.1, 129.2, 128.7, 125.5, 125.4, 123.6, 118.8, 62.4, 34.8, 31.4.

R_f = 0.55 (15% v/v EtOAc in petroleum ether).

HRMS (+ESI): *m/z* found $[M+H]^+$ 310.1810, $[C_{20}H_{24}NO_2]^+$ requires 310.1802, (δ = +2.6 ppm).

(Z)-3-(4-(trifluoromethoxy)phenyl)allyl phenylcarbamate

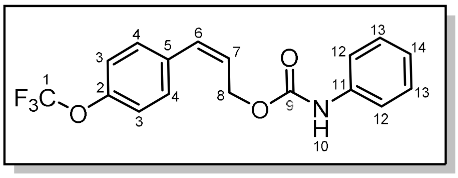

Prepared according to **GP 3**, followed by **GP 5** with 3-(4-(trifluoromethoxy)phenyl)prop-2-yn-1-ol (1.05 g, 4.86 mmol, 1.0 equiv.). Purification by flash column chromatography (SiO_2, 0-10% v/v EtOAc in petroleum ether) afforded the title compound as a white solid (1.27 g, 3.77 mmol, 77% yield over two steps).

^{1}H NMR (700 MHz, CDCl$_3$): *δ* 7.38 (d, *J* = 7.2 Hz, 2H, H-12), 7,33-7.30 (m, 2H, H-13), 7.28 (d, *J* = 8.5 Hz, 2H, H-4), 7.21 (d, *J* = 8.2 Hz, 2H, H-3), 7.08 (t, *J* = 7.4 Hz, 1H, H-14), 6.66 (d, *J* = 11.6 Hz, 1H, H-6), 6.65 (br s, 1H, H-10), 5.91 (dt, *J* = 11.7, 6.7 Hz, 1H, H-7), 4.91 (dd, *J* = 6.7, 1.3 Hz, 2H, H-8).

^{13}C NMR (176 MHz, CDCl$_3$): *δ* 153.3, 148.6, 137.8, 134.8, 131.8, 130.3, 129.2, 127.1, 123.8, 121.0, 120.6 (q, $J_{C\text{-}F}$ = 257.0 Hz), 118.8, 62.0.

^{19}F NMR (376 MHz, CDCl$_3$): *δ* -58.8.

R_f = 0.34 (10% v/v EtOAc in petroleum ether).

HRMS (+ESI): *m/z* found [M+Na]$^+$ 360.0829, $[C_{17}H_{14}NF_3O_3Na]^+$ requires 360.0818, (*δ* = +3.1 ppm).

(Z)-3-(4-chlorophenyl)allyl phenylcarbamate

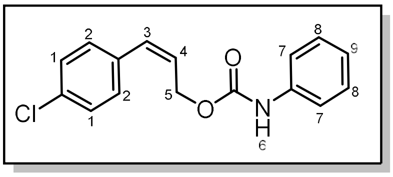

Prepared according to **GP 3**, followed by **GP 5** with 3-(4-chlorophenyl)prop-2-yn-1-ol (791 mg, 4.75 mmol, 1.0 equiv.). Purification by flash column chromatography (SiO_2, 0-10% v/v EtOAc in petroleum ether, *then* 30-50% v/v CH_2Cl_2 in petroleum ether) afforded the title compound as a white solid (854 mg, 2.97 mmol, 62% yield over two steps).

^{1}H NMR (700 MHz, CDCl$_3$): *δ* 7.38 (d, *J* = 8.6 Hz, 2H, H-7), 7.34-7.30 (m, 4H, H-1, H-8), 7.18 (d, *J* = 8.4 Hz, 2H, H-2), 7.08 (t, *J* = 7.4 Hz, 1H, H-9), 6.63 (br d, *J* = 11.8 Hz, 2H, H-3, H-6), 5.89 (dt, *J* = 11.6, 6.6 Hz, 1H, H-4), 4.90 (dd, *J* = 6.6, 1.7 Hz, 2H, H-5).

^{13}C NMR (176 MHz, CDCl$_3$): *δ* 153.3, 137.8, 134.6, 133.6, 132.0, 130.2, 129.2, 128.8, 126.8, 123.8, 118.8, 62.0.

R_f = 0.49 (15% v/v EtOAc in petroleum ether), 0.59 (80% v/v CH_2Cl_2 in petroleum ether).

HRMS (+ESI): *m/z* found $[M+H]^+$ 288.0794, $[C_{16}H_{15}ClNO_2]^+$ requires 288.0786, (δ = +2.8 ppm).

(Z)-3-(4-(trifluoromethyl)phenyl)allyl phenylcarbamate

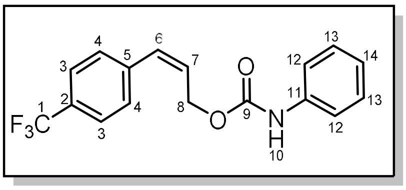

Prepared according to **GP 3**, followed by **GP 5** with 3-(4-(trifluoromethyl)phenyl)prop-2-yn-1-ol (955 mg, 4.78 mmol, 1.0 equiv.). Purification by flash column chromatography (SiO_2, 0-10% v/v EtOAc in petroleum ether, *then* 30-50% v/v CH_2Cl_2 in petroleum ether) afforded the title compound as a white solid (871 mg, 2.71 mmol, 57% yield over two steps).

^{1}H NMR (700 MHz, $CDCl_3$): δ 7.62 (d, J = 8.0 Hz, 2H, H-3), 7.41-7.36 (m, 4H, H-4, H-12), 7.31 (t, J = 7.7 Hz, 2H, H-13), 7.08 (t, J = 7.4 Hz, 1H, H-14), 6.71 (d, J = 11.8 Hz, 1H, H-6), 6.65 (br s, 1H, H-10), 5.98 (dt, J = 11.7, 6.6 Hz, 1H, H-7), 4.92 (d, J = 6.6 Hz, 2H, H-8).

^{13}C NMR (176 MHz, $CDCl_3$): δ 153.3, 139.6, 137.8, 131.8, 129.7 (q, J_{C-F} = 32.4 Hz), 129.3, 129.1, 128.4, 125.5, (q, J_{C-F} = 3.5 Hz), 124.2 (q, J_{C-F} = 272.8 Hz), 123.8, 118.8, 61.9.

^{19}F NMR (376 MHz, $CDCl_3$): δ -63.6.

R_f = 0.54 (20% v/v EtOAc in petroleum ether), 0.56 (80% v/v CH_2Cl_2 in petroleum ether).

HRMS (+ESI): *m/z* found $[M+H]^+$ 322.1058, $[C_{17}H_{15}F_3NO_2]^+$ requires 322.1049, (δ = +2.8 ppm).

(Z)-3-(naphthalen-1-yl)allyl phenylcarbamate

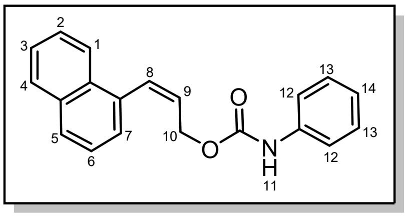

Prepared according to **GP 3**, followed by **GP 5** with 3-(naphthalen-1-yl)prop-2-yn-1-ol (869 mg, 4.77 mmol, 1.0 equiv.). Purification by flash column chromatography (SiO_2, 0-6% v/v EtOAc in petroleum ether) afforded the title compound as a white solid (1.20 g, 3.97 mmol, 83% yield over two steps).

^{1}H NMR (400 MHz, $CDCl_3$): δ 7.98-7.96 (m, 1H, H-1), 7.89-7.86 (m, 1H, H-4), 7.82 (d, J = 8.5 Hz, 1H, H-5), 7.55-7.45 (m, 2H, H-2, H-3), 7.47 (t, J = 7.7 Hz, 1H, H-6), 7.37-7.27 (m, 5 H, H-7, H-12, H-13), 7.22 (d, J = 11.4 Hz, 1H, H-8), 7.06 (t, J = 7.2 Hz, 1H, H-14), 6.57 (br s, 1H, H-11), 6.15 (dt, J = 11.6, 6.7 Hz, 1H, H-9), 4.82 (d, J = 6.7 Hz, 2H, H-10).

^{13}C NMR (101 MHz, $CDCl_3$): δ 153.4, 137.9, 133.6, 133.2, 131.8, 131.7, 129.2, 128.6, 128.4, 127.7, 126.7, 126.4, 126.1, 125.4, 124.8, 123.6, 118.8, 62.4.

R_f = 0.53 (15% v/v EtOAc in petroleum ether).

HRMS (+ESI): *m/z* found $[M+H]^+$ 304.1334, $[C_{20}H_{18}NO_2]^+$ requires 304.1332, (δ = +0.7 ppm).

(Z)-3-(naphthalen-2-yl)allyl phenylcarbamate

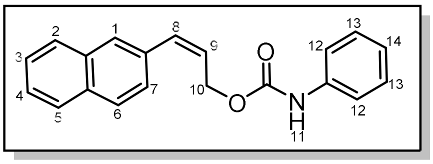

Prepared according to **GP 4**, followed by **GP 5** with ethyl (*Z*)-3-(naphthalen-2-yl)acrylate (579 mg, 2.56 mmol, 1.0 equiv.). Purification by flash column chromatography (SiO_2, 0-8% v/v EtOAc in petroleum ether) afforded the title compound as an off white solid (682 mg, 2.25 mmol, 88% yield over two steps).

^{1}H NMR (500 MHz, $CDCl_3$): δ 7.86-7.81 (m, 3H, H-2, H-5, H-6), 7.69 (s, 1H, H-1), 7.52-7.46 (m, 2H, H-3, H-4), 7.40-7.38 (m, 3H, H-7, H-12, H-12), 7.33-7.29 (m, 2H, H-13, H-13), 7.08 (tt, *J* = 7.3, 1.2 Hz, 1H, H-14), 6.84 (d, *J* = 11.7 Hz, 1H, H-8), 6.66 (br s, 1H, H-11), 5.96 (dt, *J* = 11.7, 6.6 Hz, 1H, H-9), 5.05 (dd, *J* = 6.6, 1.7 Hz, 2H, H-10).

^{13}C NMR (126 MHz, $CDCl_3$): δ 153.5, 137.9, 133.7, 133.3, 133.2, 132.7, 129.2, 128.3, 128.2, 128.0, 127.8, 126.8, 126.52, 126.49, 126.4, 123.7, 118.8, 62.3.

R_f = 0.52 (15% v/v EtOAc in petroleum ether).

HRMS (+ESI): *m/z* found $[M+H]^+$ 304.1339, $[C_{20}H_{18}NO_2]^+$ requires 304.1332, (δ = +2.3 ppm).

(Z)-3-(thiophen-3-yl)allyl phenylcarbamate

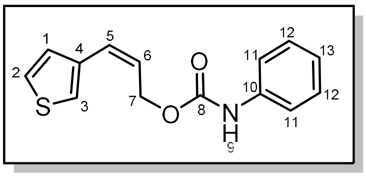

Prepared according to **GP 4**, followed by **GP 5** with ethyl (*Z*)-3-(thiophen-3-yl)acrylate (672 mg, 3.67 mmol, 1.0 equiv.). Purification by flash column chromatography (SiO_2, 0-10% v/v EtOAc in petroleum ether) afforded the title compound as an off white solid (864 mg, 3.33 mmol, 91% yield over two steps).

^{1}H NMR (400 MHz, CDCl$_3$): *δ* 7.39 (d, *J* = 7.9 Hz, 2H, H-11), 7.33-7.29 (m, 3H, H-2, H-12), 7.21 (d, *J* = 2.8 Hz, 1H, H-3), 7.10-7.05 (m, 2H, H-1, H-13), 6.66 (br s, 1H, H-9), 6.62 (d, *J* = 11.6 Hz, 1H, H-5), 5.81 (dt, *J* = 11.6, 6.6 Hz, 1H, H-6), 4.97 (dd, *J* = 6.6, 1.5 Hz, 2H, H-7).

^{13}C NMR (101 MHz, CDCl$_3$): *δ* 153.5, 137.9, 137.4, 129.2, 128.4, 127.1, 125.9, 125.1, 124.4, 123.7, 118.8, 62.5.

R$_f$ = 0.48 (20% v/v EtOAc in petroleum ether).

HRMS (+ESI): *m/z* found [M+Na]$^+$ 282.0565, [$C_{14}H_{13}NO_2SNa$]$^+$ requires 282.0559, (*δ* = +2.1 ppm).

(Z)-4-(4-(tert-butyl)phenyl)but-3-en-2-yl phenylcarbamate

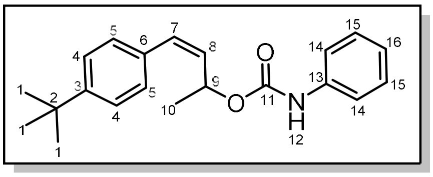

Prepared according to **GP 3**, followed by **GP 5** with 4-(4-(*tert*-butyl)phenyl)but-3-yn-2-ol (785 mg, 3.84 mmol, 1.0 equiv.). Purification by flash column chromatography (SiO$_2$, 0-5% v/v EtOAc in petroleum ether) afforded the title compound as a pale yellow, viscous oil (971 mg, 3.00 mmol, 78% yield over two steps).

^{1}H NMR (400 MHz, CDCl$_3$): *δ* 7.39-7.37 (m, 4H, H-4, H-14), 7.32-7.26 (m, 4H, H-5, H-15), 7.05 (t, *J* = 7.3 Hz, 1H, H-16), 6.58 (br s, 1H, H-12), 6.53 (d, *J* = 11.8 Hz, 1H, H-7), 5.92-5.85 (m, 1H, H-9), 5.67 (dd, *J* = 11.5, 9.0 Hz, 1H, H-8), 1.47 (d, *J* = 6.3 Hz, 3H, H-10), 1.33 (s, 9H, H-1).

^{13}C NMR (101 MHz, CDCl$_3$): *δ* 152.9, 150.6, 138.1, 133.4, 131.0, 130.7, 129.2, 128.6, 125.5, 123.5, 118.8, 69.1, 34.7, 31.4, 21.4.

R$_f$ = 0.44 (10% v/v EtOAc in petroleum ether).

HRMS (+ESI): *m/z* found [M+Na]$^+$ 346.1786, [$C_{21}H_{25}NO_2Na$]$^+$ requires 346.1777, (*δ* = +2.6 ppm).

(Z)-1-(4-(tert-butyl)phenyl)-4-methylpent-1-en-3-yl phenylcarbamate

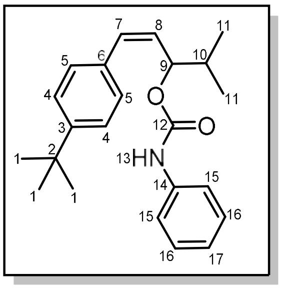

Prepared according to **GP 3**, followed by **GP 5** with 1-(4-(*tert*-butyl)phenyl)-4-methylpent-1-yn-3-ol (1.38 g, 5.99 mmol, 1.0 equiv.). Purification by flash column chromatography (SiO_2, 0-5% v/v EtOAc in petroleum ether) afforded the title compound as a pale yellow, viscous oil (1.78 g, 5.06 mmol, 85% yield over two steps).

^{1}H NMR (400 MHz, $CDCl_3$): δ 7.40-7.34 (m, 6H, H-4, H-5, H-15), 7.30 (t, J = 7.7 Hz, 2H, H-16), 7.05 (t, J = 7.3 Hz, 1H, H-17), 6.60 (d, J = 11.7 Hz, 1H, H-7), 6.59 (br s, 1H, H-13), 5.70 (dd, J = 9.5, 5.8 Hz, 1H, H-9), 5.62 (dd, J = 11.6, 9.4 Hz, 1H, H-8), 2.01 (sext, J = 6.7 Hz, 1H, H-10), 1.32 (s, 9H, H-1), 0.97 (d, J = 6.8 Hz, 3H, H-11a), 0.96 (d, J = 6.8 Hz, 3H, H-11b).

^{13}C NMR (101 MHz, $CDCl_3$): δ 153.2, 150.3, 138.2, 133.7, 132.6, 129.1, 128.6, 128.2, 125.5, 123.4, 118.8, 76.3, 34.7, 33.1, 31.4, 18.3, 18.0.

R_f = 0.46 (10% v/v EtOAc in petroleum ether).

HRMS (+ESI): *m/z* found $[M+Na]^+$ 374.2096, $[C_{23}H_{29}NO_2Na]^+$ requires 374.2090, (δ = +1.6 ppm).

2-phenylallyl phenylcarbamate

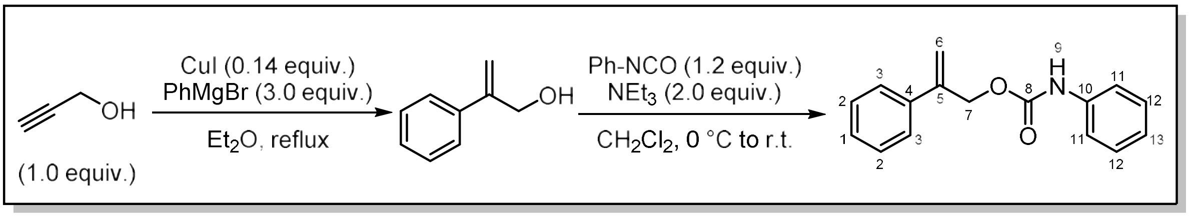

To a suspension of CuI (360 mg, 1.89 mmol, 0.14 equiv.) in Et_2O (20 mL) was added phenylmagnesium bromide (12.5 mL, 37.5 mmol, 3.0 M in Et_2O, 2.7 equiv.) dropwise at room temperature. The reaction mixture was allowed to stir at room temperature for 30 min. Propargyl alcohol (0.8 mL, 13.9 mmol, 1.0 equiv.) in Et_2O (11 mL, 1.26 M) was added dropwise at room temperature. The reaction mixture was refluxed overnight and cooled to 0 ˚C. The reaction mixture was quenched with the dropwise addition of aqueous, saturated NH_4Cl (25 mL). The reaction mixture was warmed to room temperature and the organic layer was separated. The aqueous layer was extracted with Et_2O and the combined organic layers were dried over $MgSO_4$. The solvent was removed under reduced pressure and the crude residue was subjected to **GP 5**. Purification by flash column chromatography (SiO_2, 0-20%

v/v EtOAc in petroleum ether) afforded the title compound as a pale yellow solid (1.79 g, 7.07 mmol, 51% yield over two steps).

^{1}H NMR (400 MHz, $CDCl_3$): *δ* 7.49-7.46 (m, 2H, H-3), 7.40-7.34 (m, 4H, H-2, H-11), 7.33-7.28 (m, 3H, H-1, H-12), 7.09-7.05 (m, 1H, H-13), 6.65 (s, 1H, H-9), 5.59 (s, 1H, H-6a), 5.42 (d, *J* = 0.8 Hz, 1H, H-6b), 5.10 (s, 2H, H-7).

^{13}C NMR (101 MHz, $CDCl_3$): *δ* 153.3, 142.8, 138.1, 137.8, 129.2, 128.7, 128.3, 126.2, 123.7, 118.8, 115.6, 66.7.

R_f = 0.27 (10% v/v EtOAc in petroleum ether).

HRMS (+ESI): *m/z* found $[M+Na]^+$ 276.0988, $[C_{16}H_{15}NO_2Na]^+$ requires 276.0995, (*δ* = -2.5 ppm).

3-methylbut-2-en-1-yl phenylcarbamate

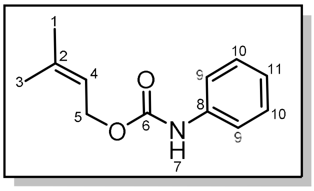

Prepared according to **GP 5** with 3-methylbut-2-en-1-ol (1.0 mL, 10.0 mmol, 1.0 equiv.). Purification by flash column chromatography (SiO_2, 0-10% v/v EtOAc in petroleum ether) afforded the title compound as an off white solid (2.01 g, 9.79 mmol, 98% yield).

^{1}H NMR (400 MHz, $CDCl_3$): *δ* 7.37 (d, *J* = 8.0 Hz, 2H, H-9), 7.30 (t, *J* = 7.9 Hz, 2H, H-10), 7.05 (t, *J* = 7.4 Hz, 1H, H-11), 6.62 (br s, 1H, H-7), 5.40 (t, *J* = 7.3 Hz, 1H, H-4), 4.67 (d, *J* = 7.3 Hz, 2H, H-5), 1.78 (s, 3H, H-1), 1.75 (s, 3H, H-3).

^{13}C NMR (101 MHz, $CDCl_3$): *δ* 153.7, 139.5, 138.1, 129.2, 123.5, 118.83, 118.76, 62.1, 25.9, 18.2.

R_f = 0.40 (10% v/v EtOAc in petroleum ether).

The spectroscopic data is in agreement with that reported in the literature.[29]

(Z)-hex-2-en-1-yl phenylcarbamate

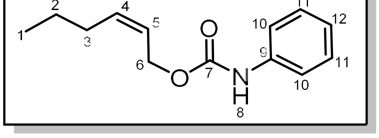

Prepared according to **GP 5** with (*Z*)-hex-2-en-1-ol (1.2 mL, 10.0 mmol, 1.0 equiv.). Purification by flash column chromatography (SiO_2, 0-10% v/v EtOAc in petroleum ether) afforded the title compound as a yellow oil (1.85 g, 8.44 mmol, 84% yield).

^{1}H NMR (400 MHz, CDCl$_3$): δ 7.38 (d, J = 7.9 Hz, 2H, H-10), 7.32-7.28 (m, 2H, H-11), 7.06 (tt, J = 7.3, 1.2 Hz, 1H, H-12), 6.60 (br s, 1H, H-8), 5.72-5.65 (m, 1H, H-4), 5.63-5.56 (m, 1H, H-5), 4.72 (d, J = 6.6 Hz, 2H, H-6), 2.12 (q, J = 7.3 Hz, 2H, H-3), 1.42 (sext, J = 7.4 Hz, 2H, H-2), 0.93 (t, J = 7.4 Hz, 3H, H-1).

^{13}C NMR (101 MHz, CDCl$_3$): δ 153.6, 138.0, 135.5, 129.2, 123.7, 123.6, 118.8, 61.2, 29.7, 22.7, 13.8.

R_f = 0.40 (10% v/v EtOAc in petroleum ether).

HRMS: *This compound did not ionise.*

cinnamyl phenylcarbamate

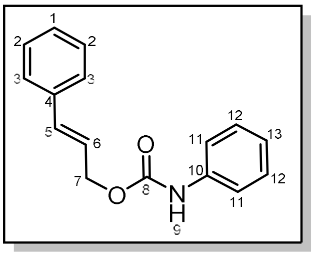

Prepared according to **GP 5** with cinnamyl alcohol (1.59 g, 11.8 mmol, 1.0 equiv.). Purification by flash column chromatography (SiO_2, 30-50% v/v CH_2Cl_2 in petroleum ether) afforded the title compound as a white solid (2.47 g, 9.75 mmol, 83% yield).

^{1}H NMR (400 MHz, CDCl$_3$): δ 7.41-7.39 (m, 4H, H-3, H-11), 7.35-7.29 (m, 4H, H-2, H-12), 7.27-7.25 (m, 1H, H-1), 7.08 (t, J = 7.3 Hz, 1H, H-13), 6.70 (d, J = 15.9 Hz, 1H, H-5), 6.68 (s, 1H, H-9), 6.34 (dt, J = 15.9, 6.4 Hz, 1H, H-6), 4.83 (dd, J = 6.4, 1.0 Hz, 2H, H-7).

^{13}C NMR (101 MHz, CDCl$_3$): δ 153.4, 137.9, 136.3, 134.4, 129.2, 128.8, 128.2, 126.8, 123.7, 123.5, 118.9, 65.9.

The spectroscopic data is in agreement with that reported in the literature.[30]

7.4 Synthesis of Substrates – N-Me Allylic Carbamates

General Procedure 6A (GP 6A):

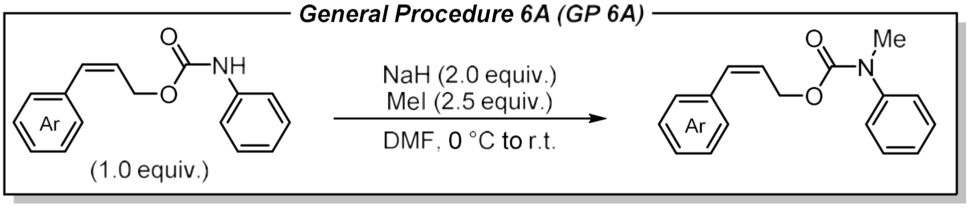

To a solution of carbamate (2.0 mmol, 1.0 equiv.) in anhydrous DMF (8 mL, 0.25 M) was added NaH (160 mg, 60% dispersion in mineral oil, 4.0 mmol, 2.0 equiv.) at 0 ˚C under a nitrogen atmosphere. The reaction mixture was allowed to stir at 0 ˚C for 30 min before methyl iodide (0.31 mL, 5.0 mmol, 2.5 equiv.) was added dropwise. The reaction mixture was allowed to

slowly warm to room temperature overnight (~16 h). The reaction mixture was quenched with water and the aqueous phase was extracted with diethyl ether. The combined organic layers were washed with 10% w/v aqueous LiCl, brine and dried over $MgSO_4$. The solvent was removed under reduced pressure and the crude residue was purified by flash column chromatography to afford the corresponding carbamate product.

General Procedure 6B (GP 6B):

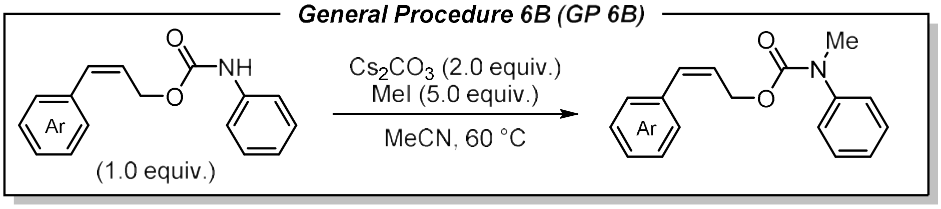

An oven-dried microwave vial was charged with carbamate (2.0 mmol, 1.0 equiv.) and cesium carbonate (1.3 g, 4.0 mmol, 2.0 equiv.). The vial was evacuated and back-filled with nitrogen thrice before the addition of anhydrous MeCN (13 mL, 0.15 M) and methyl iodide (0.62 mL, 10.0 mmol, 5.0 equiv.). The reaction mixture was stirred at 60 ˚C overnight (~17 h) and allowed to cool to room temperature. The solvent was removed under reduced pressure and the crude reaction mixture was diluted with water and $CHCl_3$. The layers were separated and the aqueous layer was extracted with $CHCl_3$. The combined organic layers were dried over $MgSO_4$ and concentrated under reduced pressure. Purification of the crude residue by flash column chromatography afforded the corresponding carbamate product.

(Z)-3-phenylallyl methyl(phenyl)carbamate (**1a**)

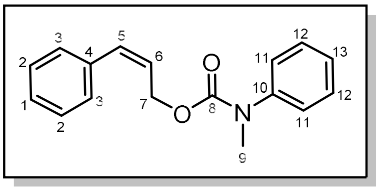

Prepared according to **GP 6A** with (*Z*)-3-phenylallyl phenylcarbamate (2.60 g, 10.3 mmol, 1.0 equiv.). Purification by flash column chromatography (SiO_2, 0-15% v/v EtOAc in petroleum ether) afforded the title compound as a colourless oil (2.75 g, 10.3 mmol, quant.).

^{1}H NMR (400 MHz, $CDCl_3$): *δ* 7.37-7.31 (m, 4H, H-2, H-12), 7.28-7.20 (m, 6H, H-1, H-3, H-11, H-13), 6.62 (d, *J* = 11.8 Hz, 1H, H-5), 5.82 (dt, *J* = 11.7, 6.4 Hz, 1H, H-6), 4.89 (d, *J* = 6.6 Hz, 2H, H-7), 3.32 (s, 3H, H-9).

^{13}C NMR (101 MHz, $CDCl_3$): *δ* 155.6, 143.4, 136.2, 132.7, 129.0, 128.9, 128.4, 127.5, 126.7, 126.2, 125.9, 62.8, 37.9.

R_f = 0.53 (20% v/v EtOAc in petroleum ether).

HRMS (+ESI): *m/z* found $[M+Na]^+$ 290.1148, $[C_{17}H_{17}NO_2Na]^+$ requires 290.1151, (δ = -1.0 ppm).

(Z)-3-(2-isopropylphenyl)allyl methyl(phenyl)carbamate (**1b**)

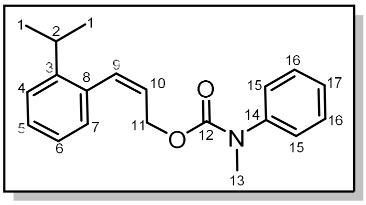

Prepared according to **GP 6B** with (*Z*)-3-(2-isopropylphenyl)allyl phenylcarbamate (445 mg, 1.51 mmol, 1.0 equiv.). Purification by flash column chromatography (SiO_2, 0-15% v/v EtOAc in petroleum ether) afforded the title compound as a colourless oil (435 mg, 1.41 mmol, 93% yield).

^{1}H NMR (400 MHz, $CDCl_3$): δ 7.37-7.33 (m, 2H, H-16), 7.29-7.20 (m, 5H, H-4, H-5, H-15, H-17), 7.14-7.10 (m, 1H, H-6), 7.04 (d, *J* = 7.4 Hz, 1H, H-7), 6.75 (d, *J* = 11.5 Hz, 1H, H-9), 5.89 (dt, *J* = 11.2, 6.7 Hz, 1H, H-10), 4.70 (d, *J* = 6.7 Hz, 2H, H-11), 3.30 (s, 3H, H-13), 3.08 (sept, *J* = 6.9 Hz, 1H, H-2), 1.19 (d, *J* = 6.8 Hz, 6H, H-1).

^{13}C NMR (101 MHz, $CDCl_3$): δ 155.7, 146.8, 143.4, 134.3, 132.6, 129.5, 128.9, 128.1, 126.9, 126.1, 125.8, 125.5, 125.0, 62.6, 37.8, 30.0, 23.4.

R_f = 0.56 (20% v/v EtOAc in petroleum ether).

HRMS (+ESI): *m/z* found $[M+Na]^+$ 332.1629, $[C_{20}H_{23}NO_2Na]^+$ requires 332.1621, (δ = +2.4 ppm).

(Z)-3-(2-methoxyphenyl)allyl methyl(phenyl)carbamate (**1c**)

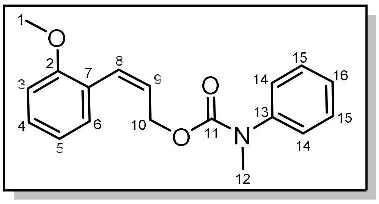

Prepared according to **GP 6B** with (*Z*)-3-(2-methoxyphenyl)allyl phenylcarbamate (605 mg, 2.14 mmol, 1.0 equiv.). Purification by flash column chromatography (SiO_2, 0-17% v/v EtOAc in petroleum ether) afforded the title compound as a pale-yellow oil (612 mg, 2.06 mmol, 96% yield).

^{1}H NMR (400 MHz, $CDCl_3$): δ 7.37-7.33 (m, 2H, H-15), 7.28-7.19 (m, 4H, H-4, H-14, H-16), 7.11 (d, *J* = 7.2 Hz, 1H, H-6), 6.92-6.86 (m, 2H, H-3, H-5), 6.75 (d, *J* = 11.7 Hz, 1H, H-8), 5.86 (dt, *J* = 11.6, 6.6 Hz, 1H, H-9), 4.81 (d, *J* = 6.6 Hz, 2H, H-10), 3.82 (s, 3H, H-1), 3.31 (s, 3 H, H-12).

^{13}C NMR (101 MHz, CDCl$_3$): δ 157.1, 155.7, 143.4, 130.2, 129.1, 128.9, 128.4, 126.5, 126.1, 125.9, 125.1, 120.4, 110.5, 63.1, 55.5, 37.8.

R_f = 0.40 (20% v/v EtOAc in petroleum ether).

HRMS (+ESI): *m/z* found [M+Na]$^+$ 320.1263, [$C_{18}H_{19}NO_3Na$]$^+$ requires 320.1257, (δ = +1.9 ppm).

(Z)-3-(2-fluorophenyl)allyl methyl(phenyl)carbamate (**1d**)

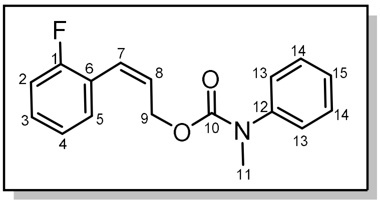

Prepared according to **GP 6B** with (*Z*)-3-(2-fluorophenyl)allyl phenylcarbamate (536 mg, 1.98 mmol, 1.0 equiv.). Purification by flash column chromatography (SiO_2, 0-15% v/v EtOAc in petroleum ether) afforded the title compound as a colourless oil (543 mg, 1.90 mmol, 96% yield).

^{1}H NMR (400 MHz, CDCl$_3$): δ 7.35 (t, *J* = 7.7 Hz, 2H, H-14), 7.27-7.17 (m, 5H, H-3, H-5, H-13, H-15), 7.10-7.02 (m, 2H, H-2, H-4), 6.65 (d, *J* = 11.6 Hz, 1H, H-7), 5.93 (dt, *J* = 11.4, 6.2 Hz, 1H, H-8), 4.81 (d, *J* = 6.2 Hz, 2H, H-9), 3.31 (s, 3H, H-11).

^{13}C NMR (101 MHz, CDCl$_3$): δ 160.2 (d, $J_{C\text{-}F}$ = 246.9 Hz), 155.5, 143.3, 130.6 (d, $J_{C\text{-}F}$ = 3.2 Hz), 129.5 (d, $J_{C\text{-}F}$ = 8.3 Hz), 129.0, 128.7 (d, $J_{C\text{-}F}$ = 1.0 Hz), 126.2, 125.9, 125.2 (d, $J_{C\text{-}F}$ = 3.9 Hz), 124.0 (d, $J_{C\text{-}F}$ = 14.4 Hz), 123.9 (d, $J_{C\text{-}F}$ = 3.7 Hz), 115.6, (d, $J_{C\text{-}F}$ = 22.0 Hz), 62.8 (d, $J_{C\text{-}F}$ = 2.5 Hz), 37.8.

^{19}F NMR (376 MHz, CDCl$_3$): δ -115.3.

R_f = 0.54 (20% v/v EtOAc in petroleum ether).

HRMS (+ESI): *m/z* found [M+Na]$^+$ 308.1063, [$C_{17}H_{16}FNO_2Na$]$^+$ requires 308.1057, (δ = +1.9 ppm).

prop-2-yn-1-yl methyl(phenyl)carbamate

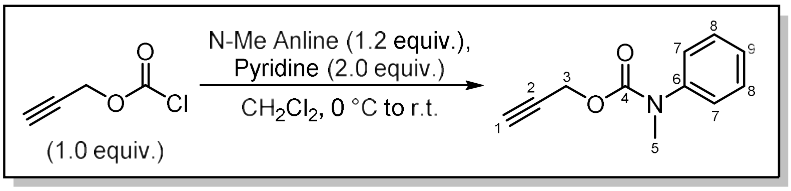

To a solution of N-methylaniline (2.6 mL, 24.0 mmol, 1.2 equiv.) and pyridine (3.2 mL, 40.0 mmol, 2.0 equiv.) in anhydrous CH_2Cl_2 (100 mL, 0.2 M) was added propargyl chloroformate (1.95 mL, 20.0 mmol, 1.0 equiv.) dropwise at 0 ˚C. The reaction mixture was allowed to warm to room temperature and stirred at this temperature for 20 h. The reaction mixture was added to ice water (150 mL) and the two layers separated. The aqueous phase was extracted with CH_2Cl_2 and the combined organic layers were washed with brine and dried over $MgSO_4$. The solvent was removed under reduced pressure and the crude residue was purified by flash column chromatography (SiO_2, 0-20% v/v EtOAc in petroleum ether) to give the title compound as a brown oil (3.19 g, 16.7 mmol, 84% yield).

^{1}H NMR (400 MHz, CDCl$_3$): δ 7.38-7.34 (m, 2H, H-8), 7.26-7.21 (m, 3H, H-7, H-9), 4.71 (br s, 2H, H-3), 3.33 (s, 3H, H-5), 2.44 (t, *J* = 2.1 Hz, 1H, H-1).

^{13}C NMR (101 MHz, CDCl$_3$): δ 154.8, 143.0, 129.0, 126.5, 125.9, 78.5, 74.6, 53.2, 38.0.

R_f = 0.35 (10% v/v EtOAc in petroleum ether).

HRMS (+ESI): *m/z* found $[M+H]^+$ 190.0860, $[C_{11}H_{12}NO_2]^+$ requires 190.0863, (δ = -1.6 ppm).

3-(o-tolyl)prop-2-yn-1-yl methyl(phenyl)carbamate

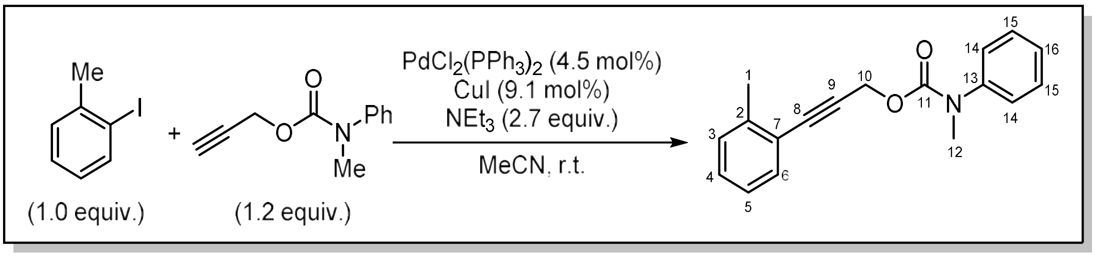

To a solution of 2-iodotoluene (0.38 mL, 3.0 mmol, 1.0 equiv.) in MeCN (12 mL, 0.25 M) was added $PdCl_2(PPh_3)_2$ (94.8 mg, 0.14 mmol, 4.5 mol%), CuI (52.0 mg, 0.27 mmol, 9.1 mol%) and Et_3N (1.1 mL, 8.1 mmol, 2.7 equiv.) at room temperature under a nitrogen atmosphere. The reaction mixture was allowed to stir at room temperature for 20 min before the addition of prop-2-yn-1-yl methyl(phenyl)carbamate (681.2 mg, 3.6 mmol, 1.2 equiv.) in MeCN (2.4 mL, 1.5 M). The resultant reaction mixture was stirred at room temperature overnight (~16 h) and the solvent was removed under reduced pressure. Purification of the crude residue

by flash column chromatography (SiO_2, 0-10% v/v EtOAc in petroleum ether) afforded the title compound as a yellow oil (236 mg, 0.84 mmol, 28% yield).

^{1}H NMR (400 MHz, $CDCl_3$): δ 7.42-7.35 (m, 3H, H-6, H-15), 7.28 (d, J = 7.7 Hz, 2H, H-14), 7.25-7.18 (m, 3H, H-3, H-4, H-16), 7.13 (td, J = 7.3, 1.4 Hz, 1H, H-5), 4.99 (s, 2H, H-10), 3.36 (s, 3H, H-12), 2.42 (s, 3H, H-1).

^{13}C NMR (101 MHz, $CDCl_3$): δ 155.0, 143.2, 140.7, 132.2, 129.5, 129.0, 128.7, 126.4, 125.9, 125.6, 122.3, 87.7, 85.2, 54.2, 38.0, 20.7.

R_f = 0.38 (10% v/v EtOAc in petroleum ether).

HRMS (+ESI): *m/z* found $[M+H]^+$ 280.1340, $[C_{18}H_{18}NO_2]^+$ requires 280.1332, (δ = +2.9 ppm).

(Z)-3-(o-tolyl)allyl methyl(phenyl)carbamate (**1e**)

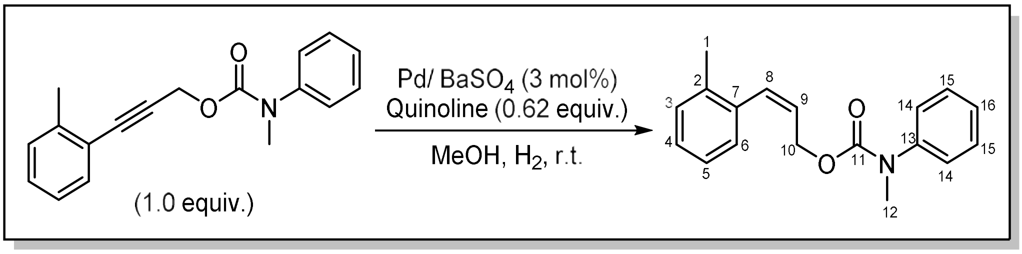

An oven-dried microwave vial was charged with 5% Pd/$BaSO_4$ (54.2 mg, 0.03 mmol, 3 mol%) and anhydrous MeOH (2 mL). The reaction mixture was evacuated until the solvent began to boil and backfilled with a balloon of hydrogen gas. This process was repeated twice following which the reaction mixture was left stirring under an atmosphere of hydrogen gas for 30 min. Quinoline (0.07 mL, 0.53 mmol, 0.62 equiv.) and a solution of 3-(*o*-tolyl)prop-2-yn-1-yl methyl(phenyl)carbamate (226 mg, 0.81 mmol, 1.0 equiv.) in anhydrous MeOH (3 mL, 0.27 M) were added sequentially to the reaction mixture. The resultant reaction mixture was allowed to stir at room temperature for 45 h, filtered through Celite and eluted with EtOAc. The solvent was removed under reduced pressure and the crude residue was purified via flash column chromatography (SiO_2, 0-10% v/v diethyl ether in petroleum ether) to afford the title compound as a colourless oil (115 mg, 0.41 mmol, 50% yield)

^{1}H NMR (400 MHz, $CDCl_3$): δ 7.38-7.33 (m, 2H, H-15), 7.26-7.20 (m, 3H, H-14, H-16), 7.18-7.11 (m, 3H, H-3, H-4, H-5), 7.07 (d, J = 7.3 Hz, 1H, H-6), 6.68 (d, J = 11.5 Hz, 1H, H-8), 5.87 (dt, J = 11.4, 6.8 Hz, 1H, H-9), 4.74 (dd, J = 6.6, 1.0 Hz, 2H, H-10), 3.31 (s, 3H, H-12), 2.25 (s, 3H, H-1).

^{13}C NMR (101 MHz, $CDCl_3$): δ 155.7, 143.4, 136.4, 135.3, 132.2, 130.0, 129.1, 128.9, 127.8, 126.6, 126.2, 125.9, 125.7, 62.7, 37.9, 20.0.

R_f = 0.29 (20% v/v diethyl ether in petroleum ether).

HRMS (+ESI): *m/z* found $[M+H]^+$ 304.1314, $[C_{18}H_{19}NO_2Na]^+$ requires 304.1308, (δ = +2.0 ppm).

tert-butyl (Z)-methyl(3-(3-((methyl(phenyl)carbamoyl)oxy)prop-1-en-1-yl)phenyl)carbamate (**1f**)

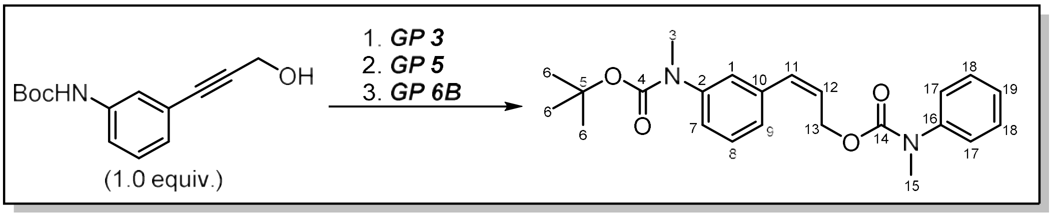

Prepared according to **GP 3**, followed by **GP 5** with *tert*-butyl (3-(3-hydroxyprop-1-yn-1-yl)phenyl)carbamate (1.27 g, 5.15 mmol, 1.0 equiv.). Purification by flash column chromatography (SiO_2, 0-20% v/v EtOAc in petroleum ether) afforded the intermediate N-H carbamate as a light yellow solid (926 mg), which was subjected to **GP 6B** with slight modifications.

An oven-dried microwave vial was charged with (*Z*)-3-(3-((*tert*-butoxycarbonyl)amino)phenyl)allyl phenylcarbamate (926 mg, 1.0 equiv.) and cesium carbonate (1.78 g, 5.46 mmol, 2.18 equiv.). The vial was evacuated and back-filled with nitrogen thrice before the addition of anhydrous MeCN (14 mL) and methyl iodide (1 mL, 16.0 mmol, 6.39 equiv.). The reaction mixture was stirred at 60 ˚C overnight (~17 h) and allowed to cool to room temperature. The solvent was removed under reduced pressure and the crude reaction mixture was diluted with water and $CHCl_3$. The layers were separated and the aqueous layer was extracted with $CHCl_3$. The combined organic layers were dried over $MgSO_4$ and concentrated under reduced pressure. Purification by flash column chromatography (SiO_2, 0-10% v/v acetone in petroleum ether) afforded the title compound as a colourless oil (539 mg, 1.36 mmol, 26% yield over three steps).

^{1}H NMR (700 MHz, $CDCl_3$): δ 7.34 (t, *J* = 7.7 Hz, 2H, H-18), 7.28-7.20 (m, 4H, H-8, H-17, H-19), 7.14 (d, *J* = 7.8 Hz, 1H, H-7), 7.06 (s, 1H, H-1), 7.00 (d, *J* = 7.2 Hz, 1H, H-9), 6.59 (d, *J* = 11.5 Hz, 1H, H-11), 5.85-5.81 (m, 1H, H-12), 4.86 (d, *J* = 5.7 Hz, 2H, H-13), 3.32 (s, 3H, H-15), 3.22 (s, 3H, H-3), 1.45 (s, 9H, H-6).

^{13}C NMR (176 MHz, $CDCl_3$): δ 155.6, 154.8, 144.0, 143.3, 136.8, 132.4, 129.0, 128.6, 127.1, 126.3, 125.9, 125.8, 124.9, 80.5, 62.6, 37.9, 37.4, 28.5.

R_f = 0.50 (20% v/v acetone in petroleum ether).

HRMS (+ESI): *m/z* found $[M+Na]^+$ 419.1953, $[C_{23}H_{28}N_2O_4Na]^+$ requires 419.1941, (δ = +2.9 ppm).

(Z)-3-(3-(tert-butyl)phenyl)allyl methyl(phenyl)carbamate (**1g**)

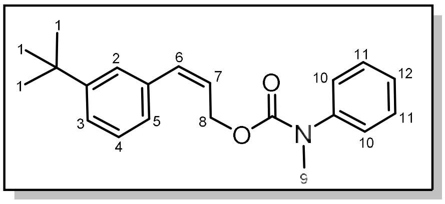

Prepared according to **GP 6A** with (*Z*)-3-(3-(*tert*-butyl)phenyl)allyl phenylcarbamate (232 mg, 0.749 mmol, 1.0 equiv.). Purification by flash column chromatography (SiO_2, 0-10% v/v EtOAc in petroleum ether) afforded the title compound as a yellow oil (226 mg, 0.697 mmol, 93% yield).

^{1}H NMR (700 MHz, $CDCl_3$): δ 7.35 (t, *J* = 8.0 Hz, 2H, H-11), 7.30 (d, *J* = 7.9 Hz, 1H, H-3), 7.28-7.20 (m, 5H, H-2, H-4, H-10, H-12), 7.03 (d, *J* = 7.4 Hz, 1H, H-5), 6.64 (d, *J* = 11.5 Hz, 1H, H-6), 5.82 (br s, 1H, H-7), 4.89 (d, *J* = 6.8 Hz, 2H, H-8), 3.32 (s, 3H, H-9), 1.30 (s, 9H, H-1).

^{13}C NMR (176 MHz, $CDCl_3$): δ 155.7, 151.3, 143.4, 135.9, 133.3, 129.0, 128.2, 126.3, 126.2, 126.00, 125.97, 125.9, 124.6, 62.9, 37.9, 34.7, 31.4.

R_f = 0.53 (15% v/v EtOAc in petroleum ether).

HRMS (+ESI): *m/z* found $[M+H]^+$ 324.1968, $[C_{21}H_{26}NO_2]^+$ requires 324.1958, (δ = +3.1 ppm).

(Z)-3-(3-methoxyphenyl)allyl methyl(phenyl)carbamate (**1h**)

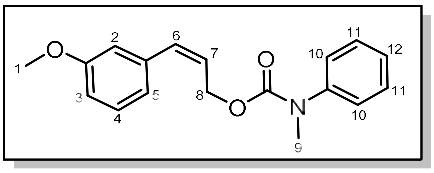

Prepared according to **GP 6A** with (*Z*)-3-(3-methoxyphenyl)allyl phenylcarbamate (282 mg, 0.995 mmol, 1.0 equiv.). Purification by flash column chromatography (SiO_2, 0-15% v/v EtOAc in petroleum ether) afforded the title compound as a light yellow oil (267 mg, 0.903 mmol, 91% yield).

^{1}H NMR (400 MHz, $CDCl_3$): δ 7.37-7.32 (m, 2H, H-11), 7.26-7.19 (m, 4H, H-4, H-10, H-12), 6.82-6.78 (m, 2H, H-3, H-5), 6.74 (br s, 1H, H-2), 6.59 (d, *J* = 11.9 Hz, 1H, H-6), 5.82 (dt, *J* = 12.2, 6.4 Hz, 1H, H-7), 4.88 (d, *J* = 6.5 Hz, 2H, H-8), 3.76 (s, 3H, H-1), 3.32 (s, 3H, H-9).

^{13}C NMR (101 MHz, $CDCl_3$): δ 159.6, 155.6, 143.4, 137.6, 132.7, 129.5, 129.0, 127.0, 126.2, 125.9, 121.4, 114.2, 113.3, 62.8, 55.3, 37.9.

R_f = 0.33 (15% v/v EtOAc in petroleum ether).

HRMS (+ESI): *m/z* found $[M+H]^+$ 298.1449, $[C_{18}H_{20}NO_3]^+$ requires 298.1438, (δ = +3.7 ppm).

(Z)-3-(3-bromophenyl)allyl methyl(phenyl)carbamate (**1i**)

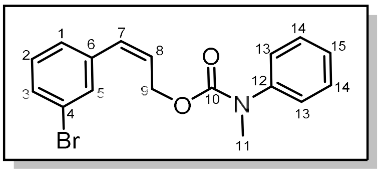

Prepared according to **GP 6A** with (*Z*)-3-(3-bromophenyl)allyl phenylcarbamate (683 mg, 2.06 mmol, 1.0 equiv.). Purification by flash column chromatography (SiO_2, 0-20% v/v diethyl ether in petroleum ether) afforded the title compound as a colourless oil (507 mg, 1.46 mmol, 71% yield).

^{1}H NMR (400 MHz, $CDCl_3$): δ 7.40-7.34 (m, 4H, H-3, H-5, H-14), 7.26-7.17 (m, 4H, H-2, H-13, H-15), 7.13 (d, *J* = 7.7 Hz, 1H, H-1), 6.54 (d, *J* = 11.7 Hz, 1H, H-7), 5.86 (dt, *J* = 11.6, 6.4 Hz, 1H, H-8), 4.84 (d, *J* = 6.3 Hz, 2H, H-9), 3.32 (s, 3H, H-11).

^{13}C NMR (101 MHz, $CDCl_3$): δ 155.5, 143.3, 138.3, 131.7, 131.4, 130.5, 130.0, 129.0, 128.2, 127.4, 126.3, 125.9, 122.6, 62.3, 38.0.

R_f = 0.42 (20% v/v diethyl ether in petroleum ether).

HRMS (+ESI): *m/z* found $[M+Na]^+$ 368.0268, $[C_{17}H_{16}NBrO_2Na]^+$ requires 368.0257, (δ = +3.0 ppm).

ethyl (Z)-3-(3-((methyl(phenyl)carbamoyl)oxy)prop-1-en-1-yl)benzoate (**1j**)

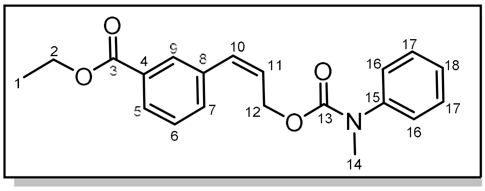

Prepared according to **GP 6A** with ethyl (*Z*)-3-(3-((phenylcarbamoyl)oxy)prop-1-en-1-yl)benzoate (674 mg, 2.07 mmol, 1.0 equiv.). Purification by flash column chromatography (SiO_2, 0-12% v/v EtOAc in petroleum ether) afforded the title compound as a pale-yellow oil (226.1 mg, 0.666 mmol, 32% yield).

^{1}H NMR (400 MHz, $CDCl_3$): δ 7.95-7.93 (m, 1H, H-5), 7.89 (br s, 1H, H-9), 7.42-7.33 (m, 4H, H-6, H-7, H-17), 7.26-7.19 (m, 3H, H-16, H-18), 6.64 (d, *J* = 11.8 Hz, 1H, H-10), 5.88 (dt, *J* = 11.4, 6.4 Hz, 1H, H-11), 4.87 (dd, *J* = 6.5, 1.1 Hz, 2H, H-12), 4.38 (q, *J* = 7.2 Hz, 2H, H-2), 3.32 (s, 3H, H-14), 1.39 (t, *J* = 7.2 Hz, 3H, H-1).

^{13}C NMR (101 MHz, $CDCl_3$): δ 166.5, 155.5, 143.3, 136.5, 133.0, 131.8, 130.8, 129.9, 129.0, 128.6, 128.5, 127.9, 126.3, 125.9, 62.5, 61.2, 37.9, 14.5.

R_f = 0.36 (20% v/v EtOAc in petroleum ether).

HRMS (+ESI): *m/z* found $[M+Na]^+$ 362.1366, $[C_{20}H_{21}NO_4Na]^+$ requires 362.1363, (δ = 0.8 ppm).

(Z)-3-(3,4-dichlorophenyl)allyl methyl(phenyl)carbamate (**1k**)

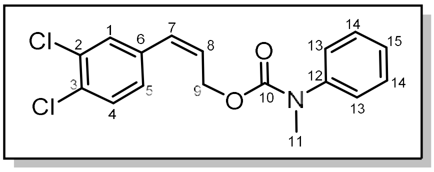

Prepared according to **GP 6B** with (Z)-3-(3,4-dichlorophenyl)allyl phenylcarbamate (659.3 mg, 2.05 mmol, 1.0 equiv.). Purification by flash column chromatography (SiO_2, 0-10% v/v EtOAc in petroleum ether) afforded the title compound as a colourless oil (603 mg, 1.79 mmol, 87% yield.).

^{1}H NMR (700 MHz, $CDCl_3$): δ 7.41-7.34 (m, 3H, H-4, H-14), 7.29 (s, 1H, H-1), 7.24-7.22 (m, 3H, H-13, H-15), 7.03 (d, J = 7.5 Hz, 1H, H-5), 6.50 (d, J = 11.6 Hz, 1H, H-7), 5.90-5.86 (m, 1H, H-8), 4.81 (d, J = 3.8 Hz, 2H, H-9), 3.31 (s, 3H, H-11).

^{13}C NMR (176 MHz, $CDCl_3$): δ 155.5, 143.2, 136.2, 132.6, 131.6, 130.60, 130.57, 130.4, 129.1, 128.6, 128.1, 126.4, 125.9, 62.1, 38.0.

R_f = 0.53 (20% v/v EtOAc in petroleum ether).

HRMS (+ESI): *m/z* found $[M+Na]^+$ 358.0388, $[C_{17}H_{15}NCl_2O_2Na]^+$ requires 358.0372, (δ = +4.5 ppm).

(Z)-3-(4-(tert-butyl)phenyl)allyl methyl(phenyl)carbamate (**1l**)

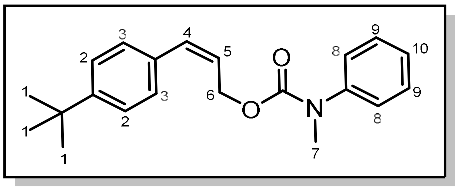

Prepared according to **GP 6B** with (*Z*)-3-(4-(*tert*-butyl)phenyl)allyl phenylcarbamate (439 mg, 1.42 mmol, 1.0 equiv.). Purification by flash column chromatography (SiO_2, 0-8% v/v EtOAc in petroleum ether) afforded the title compound as a colourless oil (448 mg, 1.28 mmol, 98% yield).

^{1}H NMR (500 MHz, $CDCl_3$): δ 7.36-7.33 (m, 4H, H-2, H-9), 7.26-7.20 (m, 3H, H-8, H-10), 7.15 (d, J = 8.4 Hz, 2H, H-3), 6.58 (d, J = 11.8 Hz, 1H, H-4), 5.77 (dt, J = 11.8, 6.0 Hz, 1H, H-5), 4.90 (d, J = 6.0 Hz, 2H, H-6), 3.32 (s, 3H, H-7), 1.32 (s, 9H, H-1).

^{13}C NMR (126 MHz, $CDCl_3$): δ 155.7, 150.6, 143.4, 133.5, 132.5, 129.0, 128.7, 126.2, 126.1, 125.9, 125.4, 62.9, 37.9, 34.7, 31.4.

R_f = 0.38 (10% v/v EtOAc in petroleum ether).

HRMS (+ESI): *m/z* found $[M+H]^+$ 324.1964, $[C_{21}H_{26}NO_2]^+$ requires 324.1958, (δ = +1.9 ppm).

(Z)-3-(4-(trifluoromethoxy)phenyl)allyl methyl(phenyl)carbamate (**1m**)

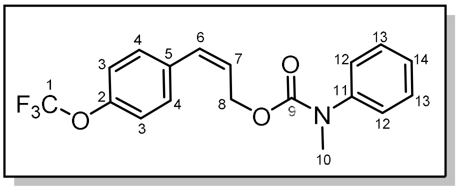

Prepared according to **GP 6B** with (*Z*)-3-(4-(trifluoromethoxy)phenyl)allyl phenylcarbamate (855 mg, 2.53 mmol, 1.0 equiv.). Purification by flash column chromatography (SiO_2, 0-10% v/v EtOAc in petroleum ether) afforded the title compound as a pale yellow oil (842 mg, 2.40 mmol, 95% yield).

^{1}H NMR (700 MHz, CDCl$_3$): δ 7.37-7.34 (m, 2H, H-13), 7.24-7.22 (m, 5H, H-4, H-12, H-14), 7.16 (d, *J* = 8.2 Hz, 2H, H-3), 6.58 (d, *J* = 11.6 Hz, 1H, H-6), 5.88-5.84 (m, 1H, H-7), 4.84 (d, *J* = 5.1 Hz, 2H, H-8), 3.32 (s, 3H, H-10).

^{13}C NMR (176 MHz, CDCl$_3$): δ 155.5, 148.5, 143.3, 135.0, 131.4, 130.3, 129.0, 127.6, 126.4, 126.0, 120.9, 120.6 (q, J_{C-F} = 256.5 Hz), 62.4, 37.9.

^{19}F NMR (376 MHz, CDCl$_3$): δ -58.8.

R_f = 0.38 (10% v/v EtOAc in petroleum ether).

HRMS (+ESI): *m/z* found $[M+Na]^+$ 374.0991, $[C_{18}H_{16}NF_3O_3Na]^+$ requires 374.0974, (δ = +4.5 ppm).

(Z)-3-(4-chlorophenyl)allyl methyl(phenyl)carbamate (**1n**)

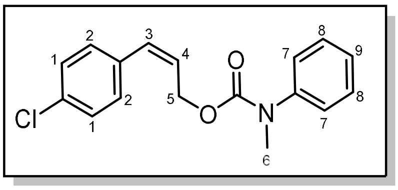

Prepared according to **GP 6B** with (*Z*)-3-(4-chlorophenyl)allyl phenylcarbamate (575 mg, 2.00 mmol, 1.0 equiv.). Purification by flash column chromatography (SiO_2, 0-12% v/v EtOAc in petroleum ether) afforded the title compound as a colourless oil (567 mg, 1.88 mmol, 94% yield).

^{1}H NMR (500 MHz, CDCl$_3$): δ 7.37-7.33 (m, 2H, H-8), 7.28 (d, *J* = 8.6 Hz, 2H, H-1), 7.24-7.21 (m, 3H, H-7, H-9), 7.13 (d, *J* = 8.5 Hz, 2H, H-2), 6.55 (d, *J* = 11.6 Hz, 1H, H-3), 5.83 (dt, *J* = 11.7, 6.9 Hz, 1H, H-4), 4.83 (d, *J* = 6.2 Hz, 2H, H-5), 3.31 (s, 3H, H-6).

^{13}C NMR (126 MHz, CDCl$_3$): δ 155.5, 143.3, 134.7, 133.4, 131.6, 130.2, 129.0, 128.6, 127.4, 126.3, 125.9, 62.4, 37.9.

R_f = 0.48 (15% v/v EtOAc in petroleum ether).

HRMS (+ESI): *m/z* found $[M+Na]^+$ 324.0759, $[C_{17}H_{16}ClNNaO_2]^+$ requires 324.0762, (δ = −0.9 ppm).

(Z)-3-(4-(trifluoromethyl)phenyl)allyl methyl(phenyl)carbamate (**1o**)

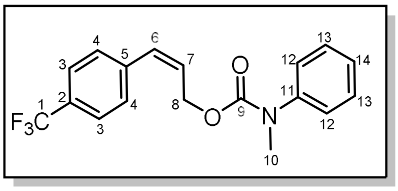

Prepared according to **GP 6B** with slight modifications. To an oven-dried microwave vial containing cesium carbonate (489 mg, 1.5 mmol, 2.0 equiv.) and methyl iodide (0.47 mL, 7.5 mmol, 10.0 equiv.) in MeCN (7.5 mL) was added a solution of (*Z*)-3-(4-(trifluoromethyl)phenyl)allyl phenylcarbamate (235 mg, 0.73 mmol, 1.0 equiv.) in MeCN (7.5 mL) at room temperature. The reaction mixture was heated to 60 ˚C immediately and allowed to stir at this temperature for 17 h. The reaction mixture was cooled to room temperature and the solvent was removed under reduced pressure. The crude reaction mixture was diluted with water and $CHCl_3$. The layers were separated and the aqueous layer was extracted with $CHCl_3$. The combined organic layers were dried over $MgSO_4$ and concentrated under reduced pressure. Purification of the crude residue by flash column chromatography (SiO_2, 0-6% v/v EtOAc in petroleum ether) afforded the title compound as a pale-yellow oil (166 mg, 0.5 mmol, 68% yield).

^{1}H NMR (700 MHz, $CDCl_3$): δ 7.57 (d, *J* = 8.0 Hz, 2H, H-3), 7.37-7.34 (m, 2H, H-13), 7.31 (d, *J* = 7.9 Hz, 2H, H-4), 7.25-7.22 (m, 3H, H-12, H-14), 6.63 (d, *J* = 11.5 Hz, 1H, H-6), 5.94-5.92 (m, 1H, H-7), 4.84 (d, *J* = 6.1 Hz, 2H, H-8), 3.31 (s, 3H, H-10).

^{13}C NMR (176 MHz, $CDCl_3$): δ 155.5, 143.3, 139.8, 131.4, 129.5 (q, J_{C-F} = 32.4 Hz), 129.1, 129.0, 128.9, 126.4, 126.0, 125.4 (q, J_{C-F} = 3.6 Hz), 124.2 (q, J_{C-F} = 272.1 Hz), 62.3, 38.0.

^{19}F NMR (471 MHz, $CDCl_3$): δ -62.6.

R_f = 0.52 (20% v/v EtOAc in petroleum ether).

HRMS (+ESI): *m/z* found $[M+Na]^+$ 358.1032, $[C_{18}H_{16}F_3NO_2Na]^+$ requires 358.1025, (δ = +2.0 ppm).

(Z)-3-(naphthalen-1-yl)allyl methyl(phenyl)carbamate (**1p**)

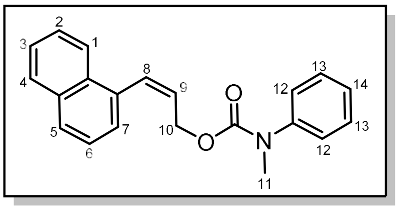

Prepared according to **GP 6B** with (*Z*)-3-(naphthalen-1-yl)allyl phenylcarbamate (607 mg, 2.00 mmol, 1.0 equiv.). Purification by flash column chromatography (SiO_2, 0-8% v/v EtOAc in petroleum ether) afforded the title compound as a colourless oil (634 mg, 2.00 mmol, quant.).

^{1}H NMR (500 MHz, $CDCl_3$): *δ* 7.96-7.93 (m, 1H, H-1), 7.87-7.84 (m, 1H, H-4), 7.79 (d, *J* = 8.3 Hz, 1H, H-5), 7.51-7.47 (m, 2H, H-2, H-3), 7.42 (t, *J* = 7.7 Hz, 1H, H-6), 7.38-7.32 (m, 2H, H-13), 7.27 (d, *J* = 6.8 Hz, 1H, H-7), 7.25-7.20 (m, 3H, H-12, H-14), 7.15 (d, *J* = 11.8 Hz, 1H, H-8), 6.10 (dt, *J* = 11.5, 6.2 Hz, 1H, H-9), 4.76 (d, *J* = 6.4 Hz, 2H, H-10), 3.29 (s, 3H, H-11).

^{13}C NMR (126 MHz, $CDCl_3$): *δ* 155.6, 143.4, 133.6, 133.2, 131.7, 131.2, 129.0, 128.6, 128.3, 128.2, 126.8, 126.24, 126.17, 126.0, 125.9, 125.4, 124.9, 62.9, 37.9.

R_f = 0.49 (15% v/v EtOAc in petroleum ether).

HRMS (+ESI): *m/z* found $[M+H]^+$ 318.1495, $[C_{21}H_{20}NO_2]^+$ requires 318.1489, (*δ* = +1.9 ppm).

(Z)-3-(naphthalen-2-yl)allyl methyl(phenyl)carbamate (**1q**)

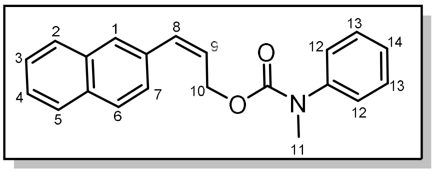

Prepared according to **GP 6B** with (*Z*)-3-(naphthalen-2-yl)allyl phenylcarbamate (461 mg, 1.52 mmol, 1.0 equiv.). Purification by flash column chromatography (SiO_2, 0-8% v/v EtOAc in petroleum ether) afforded the title compound as a colourless oil (482 mg, 1.52 mmol, quant.).

^{1}H NMR (500 MHz, $CDCl_3$): *δ* 7.82-7.75 (m, 3H, H-2, H-5, H-6), 7.64 (s, 1H, H-1), 7.49-7.46 (m, 2H, H-3, H-4), 7.38-7.34 (m, 3H, H-7, H-13), 7.27-7.22 (m, 3H, H-12, H-14), 6.78 (d, *J* = 11.7 Hz, 1H, H-8), 5.93 (dt, *J* = 11.5, 6.4 Hz, 1H, H-9), 4.98 (d, *J* = 6.2 Hz, 2H, H-10), 3.33 (s, 3H, H-11).

^{13}C NMR (126 MHz, $CDCl_3$): *δ* 155.6, 143.4, 133.8, 133.3, 132.9, 132.7, 129.0, 128.3, 128.0, 127.9, 127.7, 127.1, 126.9, 126.4, 126.3, 126.2, 125.9, 62.8, 37.9.

R_f = 0.48 (15% v/v EtOAc in petroleum ether).

HRMS (+ESI): *m/z* found $[M+H]^+$ 318.1492, $[C_{21}H_{20}NO_2]^+$ requires 318.1489, (δ = +0.9 ppm).

(Z)-3-(thiophen-3-yl)allyl methyl(phenyl)carbamate (**1r**)

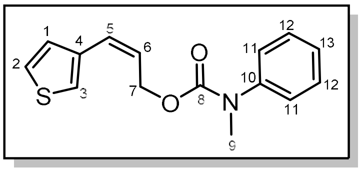

Prepared according to **GP 6B** with (*Z*)-3-(thiophen-3-yl)allyl phenylcarbamate (359 mg, 1.38 mmol, 1.0 equiv.). Purification by flash column chromatography (SiO_2, 0-10% v/v EtOAc in petroleum ether) afforded the title compound as a colourless oil (377 mg, 1.38 mmol, quant.).

^{1}H NMR (400 MHz, $CDCl_3$): δ 7.35 (t, *J* = 7.8 Hz, 2H, H-12), 7.29-7.20 (m, 4H, H-1, H-11, H-13), 7.14 (br s, 1H, H-3), 7.04 (d, *J* = 4.8 Hz, 1H, H-2), 6.55 (d, *J* = 11.7 Hz, 1H, H-5), 5.77 (dt, *J* = 11.5, 6.4 Hz, 1H, H-6), 4.90 (d, *J* = 6.4 Hz, 2H, H-7), 3.33 (s, 3H, H-9).

^{13}C NMR (101 MHz, $CDCl_3$): δ 155.6, 143.3, 137.5, 129.0, 128.5, 126.7, 126.3, 125.9, 125.7, 125.6, 124.2, 62.9, 37.9.

R_f = 0.46 (20% v/v EtOAc in petroleum ether).

HRMS (+ESI): *m/z* found $[M+Na]^+$ 296.0723, $[C_{15}H_{15}NO_2SNa]^+$ requires 296.0716, (δ = +2.4 ppm).

methyl (Z)-5-(3-((methyl(phenyl)carbamoyl)oxy)prop-1-en-1-yl)-1-tosyl-1H-indole-3-carboxylate (**1s**)

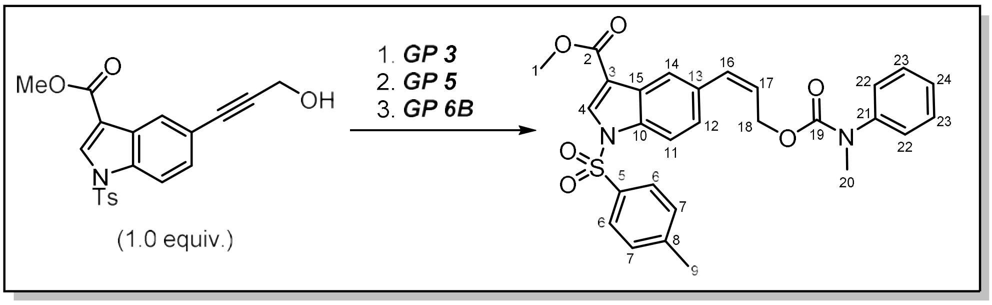

Prepared according to **GP 3**, followed by **GP 5** with methyl 5-(3-hydroxyprop-1-yn-1-yl)-1-tosyl-1H-indole-3-carboxylate (610 mg, 1.59 mmol, 1.0 equiv.). Purification by flash column chromatography (SiO_2, 0-25% v/v acetone in petroleum ether) afforded the title compound as an off white solid (790 mg), which was subjected to **GP 6B** with slight modifications.

An oven-dried microwave vial was charged with methyl (*Z*)-5-(3-((phenylcarbamoyl)oxy)prop-1-en-1-yl)-1-tosyl-1H-indole-3-carboxylate (517 mg, 1.02

mmol, 1.0 equiv.) and cesium carbonate (698 mg, 2.14 mmol, 2.1 equiv.). The vial was evacuated and back-filled with nitrogen thrice before the addition of anhydrous MeCN (7 mL) and methyl iodide (0.37 mL, 6.00 mmol, 5.9 equiv.). The reaction mixture was stirred at 60 ˚C overnight (~17 h) and allowed to cool to room temperature. The solvent was removed under reduced pressure and the crude reaction mixture was diluted with water and $CHCl_3$. The layers were separated and the aqueous layer was extracted with $CHCl_3$. The combined organic layers were dried over $MgSO_4$ and concentrated under reduced pressure. Purification by flash column chromatography (SiO_2, 0-16% v/v acetone in petroleum ether) afforded the title compound as a white foam (324 mg, 0.625 mmol, 39% yield over three steps).

^{1}H NMR (700 MHz, $CDCl_3$): δ 8.26 (s, 1H, H-4), 7.97 (s, 1H, H-14), 7.89 (d, J = 8.8 Hz, 1H, H-11), 7.82 (d, J = 8.6 Hz, 2H, H-6), 7.34 (t, J = 7.9 Hz, 2H, H-23), 7.28 (d, J = 8.3 Hz, 2H, H-7), 7.24-7.19 (m, 4H, H-12, H-22, H-24), 6.69 (d, J = 11.2 Hz, 1H, H-16), 5.83 (br s, 1H, H-17), 4.89 (d, J = 6.2 Hz, 2H, H-18), 3.91 (s, 3H, H-1), 3.31 (s, 3H, H-20), 2.36 (s, 3H, H-9).

^{13}C NMR (176 MHz, $CDCl_3$): δ 164.1, 155.6, 146.0, 143.3, 134.7, 134.0, 132.8, 132.6, 132.3, 130.4, 129.0, 128.0, 127.3, 126.9, 126.4, 126.2, 125.9, 122.4, 113.6, 113.3, 62.7, 51.8, 37.9, 21.8.

R_f = 0.57 (30% v/v acetone in petroleum ether).

HRMS (+ESI): m/z found $[M+Na]^+$ 541.1407, $[C_{28}H_{26}N_2O_6SNa]^+$ requires 541.1404, (δ = +0.6 ppm).

(Z)-4-(4-(tert-butyl)phenyl)but-3-en-2-yl methyl(phenyl)carbamate ((***rac***)-**1t**))

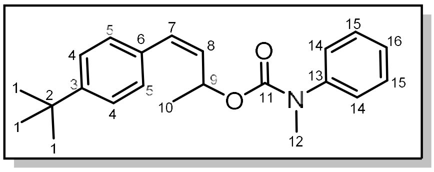

Prepared according to **GP 6B** with (*Z*)-4-(4-(*tert*-butyl)phenyl)but-3-en-2-yl phenylcarbamate (485.2 mg, 1.50 mmol, 1.0 equiv.). Purification by flash column chromatography (SiO_2, 0-4% v/v EtOAc in petroleum ether) afforded the title compound as a viscous oil (426 mg, 1.26 mmol, 84% yield.).

^{1}H NMR (400 MHz, $CDCl_3$): δ 7.38-7.32 (m, 4H, H-4, H-15), 7.27-7.18 (m, 5H, H-5, H-14, H-16), 6.45 (d, J = 11.8 Hz, 1H, H-7), 5.87-5.83 (m, 1H, H-9), 5.61-5.56 (m, 1H, H-8), 3.29 (s, 3H, H-12), 1.39 (d, J = 6.0 Hz, 3H, H-10), 1.32 (s, 9H, H-1).

^{13}C NMR (101 MHz, $CDCl_3$): δ 155.1, 150.4, 143.6, 133.6, 131.3, 130.4, 128.8, 128.6, 125.9, 125.8, 125.5, 69.5, 37.7, 34.7, 31.4, 21.3.

R_f = 0.44 (10% v/v EtOAc in petroleum ether).

HRMS (+ESI): *m/z* found $[M+Na]^+$ 360.1940, $[C_{22}H_{27}NO_2Na]^+$ requires 360.1934, (δ = +1.7 ppm).

(Z)-1-(4-(tert-butyl)phenyl)-4-methylpent-1-en-3-yl methyl(phenyl)carbamate ((***rac***)-**1u**))

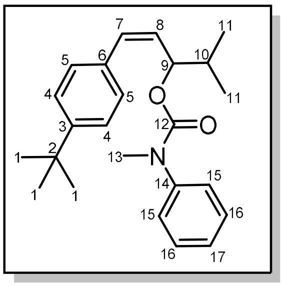

Prepared according to **GP 6B** with (*Z*)-1-(4-(*tert*-butyl)phenyl)-4-methylpent-1-en-3-yl phenylcarbamate (784 mg, 2.23 mmol, 1.0 equiv.). Purification by flash column chromatography (SiO_2, 0-5% v/v EtOAc in petroleum ether) afforded the title compound as a colourless oil (622 mg, 1.70 mmol, 76% yield).

^{1}H NMR (700 MHz, $CDCl_3$): δ 7.36-7.34 (m, 6H, H-4, H-5, H-16), 7.25 (d, *J* = 7.6 Hz, 2H, H-15), 7.21 (t, *J* = 7.4 Hz, 1H, H-17), 6.54 (d, *J* = 11.8 Hz, 1H, H-7), 5.63-5.61 (m, 1H, H-9), 5.55-5.51 (m, 1H, H-8), 3.30 (s, 3H, H-13), 1.90 (br s, 1H, H-10), 1.32 (s, 9H, H-1), 0.86-0.83 (m, 6H, H-11a, H-11b).

^{13}C NMR (176 MHz, $CDCl_3$): δ 155.4, 150.1, 143.6, 133.9, 131.9, 128.9, 128.8, 128.6, 126.0, 125.4, 76.8, 37.8, 34.7, 33.1, 31.4, 18.4, 17.7.

R_f = 0.50 (10% v/v EtOAc in petroleum ether).

HRMS (+ESI): *m/z* found $[M+Na]^+$ 388.2256, $[C_{24}H_{31}NO_2Na]^+$ requires 388.2247, (δ = +2.3 ppm).

2-phenylallyl methyl(phenyl)carbamate (**15a**)

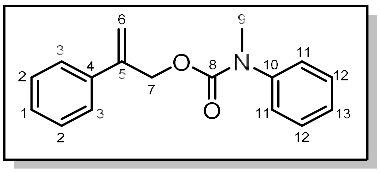

Prepared according to **GP 6A** with 2-phenylallyl phenylcarbamate (866 mg, 3.42 mmol, 1.0 equiv.). Purification by flash column chromatography (SiO_2, 0-15% v/v EtOAc in petroleum ether) afforded the title compound as a yellow oil (825 mg, 3.09 mmol, 90% yield).

^{1}H NMR (400 MHz, $CDCl_3$): δ 7.40-7.26 (m, 7H, H-1, H-2, H-3, H-12), 7.21-7.14 (m, 3H, H-11, H-13), 5.46 (s, 1H, H-6a), 5.25 (s, 1H, H-6b), 5.02 (s, 2H, H-7), 3.29 (s, 3H, H-9).

^{13}C NMR (101 MHz, $CDCl_3$): δ 155.4, 143.2, 143.1, 138.4, 128.9, 128.5, 128.0, 126.3, 126.2, 125.9, 114.6, 67.1, 37.8.

R_f = 0.27 (10% v/v EtOAc in petroleum ether).

HRMS (+ESI): *m/z* found $[M+H]^+$ 268.1327, $[C_{17}H_{18}NO_2]^+$ requires 268.1332, (δ = -1.9 ppm).

3-methylbut-2-en-1-yl methyl(phenyl)carbamate (**15b**)

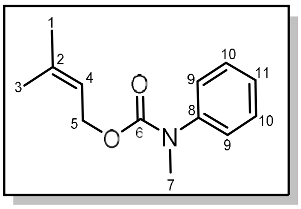

Prepared according to **GP 6A** with 3-methylbut-2-en-1-yl phenylcarbamate (1.03 g, 5.00 mmol, 1.0 equiv.). Purification by flash column chromatography (SiO_2, 0-12.5% v/v EtOAc in petroleum ether) afforded the title compound as a pale-yellow oil (1.06 g, 4.83 mmol, 97% yield).

^{1}H NMR (400 MHz, $CDCl_3$): δ 7.36-7.32 (m, 2H, H-10), 7.24 (d, J = 7.8 Hz, 2H, H-9), 7.22-7.17 (m, 1H, H-11), 5.33 (t, J = 6.5 Hz, 1H, H-4), 4.61 (d, J = 7.0 Hz, 2H, H-5), 3.30 (s, 3H, H-7), 1.73 (s, 3H, H-1), 1.69 (s, 3H, H-3).

^{13}C NMR (101 MHz, $CDCl_3$): δ 155.9, 143.6, 138.2, 128.9, 126.0, 125.8, 119.5, 62.8, 37.8, 25.9, 18.2.

R_f = 0.39 (10% v/v EtOAc in petroleum ether).

The spectroscopic data is in agreement with that reported in the literature.[31]

(Z)-hex-2-en-1-yl methyl(phenyl)carbamate (**15c**)

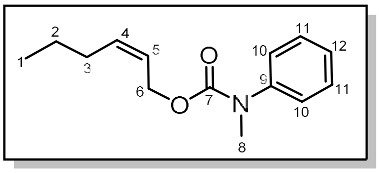

Prepared according to **GP 6A** with (*Z*)-hex-2-en-1-yl phenylcarbamate (681 mg, 3.11 mmol, 1.0 equiv.). Purification by flash column chromatography (SiO_2, 0-10% v/v EtOAc in petroleum ether) afforded the title compound as a colourless oil (700 mg, 3.00 mmol, 96% yield).

^{1}H NMR (400 MHz, $CDCl_3$): δ 7.36-7.32 (m, 2H, H-11), 7.26-7.18 (m, 3H, H-10, H-12), 5.63-5.50 (m, 2H, H-4, H-5), 4.67 (d, J = 6.1 Hz, 2H, H-6), 3.31 (s, 3H, H-8), 2.07 (q, J = 7.1 Hz, 2H, H-3), 1.38 (sext, J = 7.4 Hz, 2H, H-2), 0.89 (t, J = 7.3 Hz, 3H, H-1).

^{13}C NMR (101 MHz, $CDCl_3$): δ 155.8, 143.5, 134.8, 128.9, 126.1, 125.8, 124.3, 61.8, 37.8, 29.7, 22.7, 13.8.

R_f = 0.46 (10% v/v EtOAc in petroleum ether).

HRMS (+ESI): *m/z* found $[M+H]^+$ 234.1484, $[C_{14}H_{20}NO_2]^+$ requires 234.1489, (δ = -2.1 ppm).

cinnamyl methyl(phenyl)carbamate (**S2**)

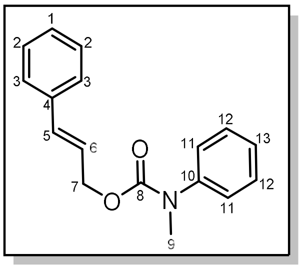

Prepared according to **GP 6A** with cinnamyl phenylcarbamate (261 mg, 1.03 mmol, 1.0 equiv.). Purification by flash column chromatography (SiO_2, 0-20% v/v Et_2O in petroleum ether) afforded the title compound as a colourless oil (255 mg, 0.954 mmol, 93% yield).

^{1}H NMR (400 MHz, $CDCl_3$): δ 7.39-7.35 (m, 4H, H-2, H-12), 7.33-7.29 (m, 3H, H-1, H-3), 7.27-7.21 (m, 3H, H-11, H-13), 6.58 (d, J = 15.6 Hz, 1H, H-5), 6.29 (dt, J = 15.8, 5.9 Hz, 1H, H-6), 4.78 (d, J = 5.9 Hz, 2H, H-7), 3.34 (s, 3H, H-9).

^{13}C NMR (101 MHz, $CDCl_3$): δ 155.6, 143.4, 136.5, 133.4, 129.0, 128.7, 128.0, 126.7, 126.3, 125.9, 124.0, 66.3, 37.9.

The spectroscopic data is in agreement with that reported in the literature.[32]

7.5 Synthesis of Substrate – N-Me Homoallylic Amide

N-methyl-N-phenylpent-4-ynamide

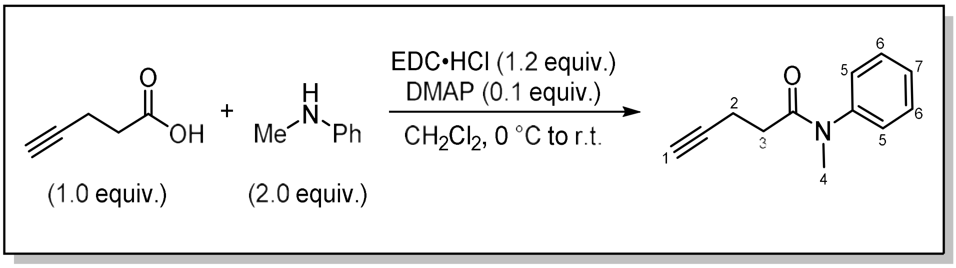

To a solution of 4-pentynoic acid (1.96 g, 20.0 mmol, 1.0 equiv.) in CH_2Cl_2 (85 mL, 0.24 M) at 0 ˚C was added 1-ethyl-3-(3-(dimethylamino) propyl)-carbodiimide hydrochloride (4.60 g, 24.0 mmol, 1.2 equiv.) and DMAP (244 mg, 2.0 mmol, 0.1 equiv.). N-methyl aniline (4.3 mL, 40.0 mmol, 2.0 equiv.) was subsequently added to the reaction mixture at 0 ˚C and the reaction mixture was allowed to warm to room temperature overnight. The reaction mixture was washed with aqueous 1 M HCl solution and brine. The organic layer was dried over $MgSO_4$ and concentrated under reduced pressure. Purification by flash column chromatography (SiO_2, 0-20% v/v acetone in petroleum ether) afforded the title compound as an orange oil (3.60 g, 19.2 mmol, 96% yield).

^{1}H NMR (400 MHz, $CDCl_3$): δ 7.44-7.40 (m, 2H, H-6), 7.36-7.32 (m, 1H, H-7), 7.19-7.16 (m, 2H, H-5), 3.26 (s, 3H, H-4), 2.49-2.44 (m, 2H, H-2), 2.29 (t, J = 7.4 Hz, 2H, H-3), 1.89 (t, J = 2.6 Hz, 1H, H-1).

^{13}C NMR (101 MHz, $CDCl_3$): δ 171.0, 143.8, 130.0, 128.1, 127.5, 83.6, 68.6, 37.5, 33.3, 14.7.

R_f = 0.29 (20% v/v acetone in petroleum ether).

The spectroscopic data is in agreement with that reported in the literature.[33]

N-methyl-N,5-diphenylpent-4-ynamide

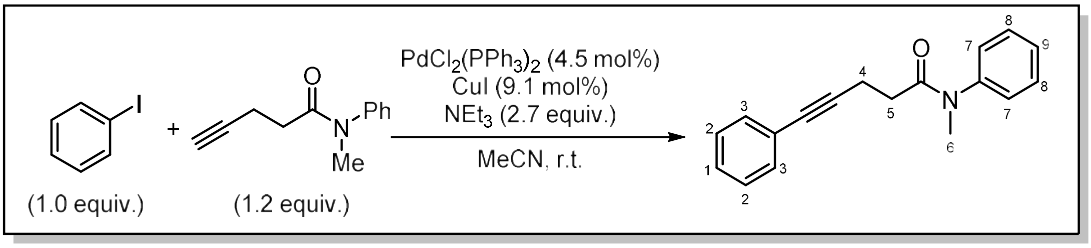

To a solution of iodobenzene (0.56 mL, 5.0 mmol, 1.0 equiv.) in MeCN (20 mL, 0.25 M) was added $PdCl_2(PPh_3)_2$ (158 mg, 0.23 mmol, 4.5 mol%), CuI (86.7 mg, 0.46 mmol, 9.1 mol%) and Et_3N (1.9 mL, 13.5 mmol, 2.7 equiv.) at room temperature under a nitrogen atmosphere. The reaction mixture was allowed to stir at room temperature for 20 min before the addition of *N*-methyl-*N*-phenylpent-4-ynamide (1.12 g, 6.0 mmol, 1.2 equiv.) in MeCN (4.0 mL, 1.5 M). The resultant reaction mixture was stirred at room temperature overnight (~16 h) and the solvent was removed under reduced pressure. Purification of the crude residue by flash column chromatography (SiO_2, 0-20% v/v acetone in petroleum ether) afforded the title compound as a yellow solid (1.32 g, 5.0 mmol, quant.).

^{1}H NMR (400 MHz, $CDCl_3$): δ 7.44-7.41 (m, 2H, H-8), 7.37-7.33 (m, 3H, H-3, H-9), 7.26-7.24 (m, 3H, H-1, H-2), 7.22-7.20 (m, 2H, H-7), 3.29 (s, 3H, H-6), 2.71 (t, J = 7.6 Hz, 2H, H-4), 2.39 (t, J = 7.5 Hz, 2H, H-5).

^{13}C NMR (101 MHz, $CDCl_3$): δ 171.3, 143.9, 131.7, 130.0, 128.3, 128.1, 127.8, 127.5, 123.8, 89.2, 80.9, 37.5, 33.5, 15.9.

R_f = 0.31 (20% v/v acetone in hexane).

HRMS (+ESI): *m/z* found $[M+H]^+$ 264.1387, $[C_{18}H_{18}NO]^+$ requires 264.1383, (δ = +1.5 ppm).

(Z)-N-methyl-N,5-diphenylpent-4-enamide

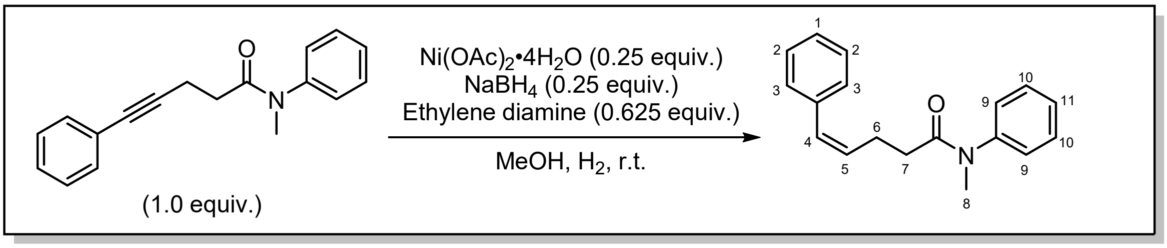

An oven-dried round-bottomed flask was charged with $Ni(OAc)_2 \cdot 4H_2O$ (249 mg, 1.00 mmol, 0.25 equiv.) and anhydrous MeOH (10.4 mL, 0.1 M). The reaction mixture was evacuated until the solvent began to boil and backfilled with a balloon of hydrogen gas. This process was repeated twice following which the reaction mixture was left stirring under an atmosphere of hydrogen gas. The reaction mixture was cooled to 0 ˚C followed by the addition of $NaBH_4$ (37.8 mg, 1.00 mmol, 0.25 equiv.) in one portion. The resultant black mixture was allowed to warm to room temperature over 15 min. Ethylenediamine (0.16 mL, 2.50 mmol, 0.63 equiv.) was added, followed by a solution of the *N*-methyl-*N*,5-diphenylpent-4-ynamide (1.05 g, 4.0 mmol, 1.0 equiv.) in MeOH (8 mL, 0.5 M). The reaction mixture was allowed to stir at room temperature and the reaction progress was monitored by ^{1}H NMR analysis. Upon reaction completion, the reaction mixture was filtered through a short silica plug and eluted with EtOAc. The solvent was removed under reduced pressure and the crude residue was purified by flash column chromatography (SiO_2, 0-15% v/v acetone in petroleum ether) afforded the title compound as a yellow oil (1.06 g, 4.0 mmol, quant.).

^{1}H NMR (700 MHz, $CDCl_3$): δ 7.40-7.38 (m, 2H, H-10), 7.34-7.29 (m, 3H, H-2, H-11), 7.22-7.20 (m, 3H, H-1, H-3), 7.14 (d, *J* = 7.7 Hz, 2H, H-9), 6.37 (d, *J* = 11.6 Hz, 1H, H-4), 5.54 (dt, *J* = 11.6, 7.4 Hz, 1H, H-5), 3.26 (s, 3H, H-8), 2.61 (dt, *J* = 7.4, 7.1 Hz, 2H, H-6), 2.21 (t, *J* = 7.6 Hz, 2H, H-7).

^{13}C NMR (176 MHz, $CDCl_3$): δ 172.4, 144.2, 137.4, 131.3, 129.9, 129.7, 128.8, 128.3, 127.9, 127.5, 126.7, 37.5, 34.4, 24.8.

R_f = 0.30 (20% v/v acetone in hexane).

HRMS (+ESI): *m/z* found $[M+H]^+$ 266.1551, $[C_{18}H_{20}NO]^+$ requires 266.1539, (δ = +4.5 ppm).

8 Characterisation of Enantioenriched Products from Aziridination

General Procedure 7 (GP 7):

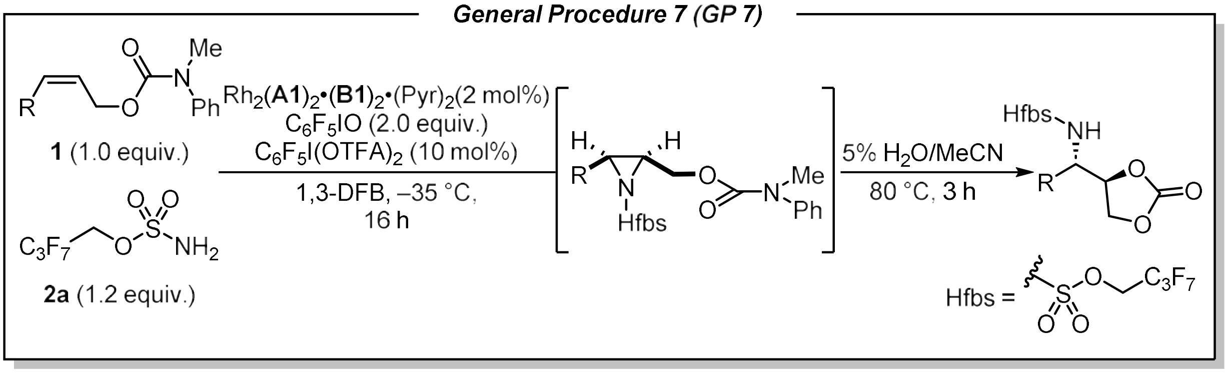

Under an atmosphere of air, a 4.0 mL crimp-top vial was charged with the allylic carbamate substrate **1** (0.1 mmol, 1.0 equiv.) and Rh_2(**A1**)$_2$•(**B1**)$_2$•(Pyr)$_2$ (5.4 mg, 2 μmol, 2 mol%). A solution of 2,2,3,3,4,4,4-heptafluorobutyl sulfamate **2a** (33.5 mg, 0.12 mmol, 1.2 equiv.) in 1,3-difluorobenzene (1 mL, 0.1 M) was added and the vial was cooled to -35 ˚C over 10 min. Pentafluoroiodosobenzene (C_6F_5IO) (62.0 mg, 0.2 mmol, 2.0 equiv.) and perfluorophenyl-λ^3-iodanediyl bis(2,2,2-trifluoroacetate) ($C_6F_5I(OTFA)_2$) (5.2 mg, 0.01 mmol, 10 mol%) were subsequently added together in a single portion at -35 ˚C. The vial was sealed and the reaction mixture stirred at -35 ˚C overnight (~16 h). Saturated aqueous thiourea (1 mL) and $CHCl_3$ (1 mL) were added and the biphasic mixture was stirred vigorously for 5 min at -35 ˚C. The mixture was allowed to warm to room temperature and stirred for a further 15 min. The layers were separated and the organic layer was passed through a short pad of $MgSO_4$. The aqueous layer was extracted with $CHCl_3$ (6 x 1 mL) with each organic layer passed through a short pad of $MgSO_4$.The combined organic layers were concentrated under reduced pressure, reconstituted in 5% H_2O/MeCN (3.5 mL) and transferred to a crimp-top vial. The vial was sealed and heated at 80 ˚C for 3 h. The reaction mixture was concentrated under a gentle stream of nitrogen and the crude residue was purified by flash column chromatography to afford the corresponding carbonate product.

The racemic reactions were prepared according to **GP 7** with the following procedural modifications: $Rh_2(esp)_2$ (2 mol%) was used with 1,3-DFB (0.5 mL, 0.2 M) in the absence of $C_6F_5I(OTFA)_2$.

2,2,3,3,4,4,4-heptafluorobutyl ((S)-((R)-2-oxo-1,3-dioxolan-4-yl)(phenyl)methyl)sulfamate (**4a**)

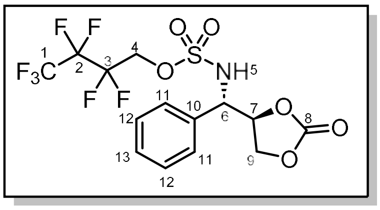

Prepared according to **GP 7** with catalyst Rh_2(**A1**)$_2$•(**B5**)$_2$•(Pyr)$_2$ (2.3 mg, 1 μmol, 1 mol%) and (*Z*)-3-phenylallyl methyl(phenyl)carbamate **1a** (26.8 mg, 0.100 mmol, 1.0 equiv.). Purification by flash column chromatography (SiO_2, 0-62% v/v diethyl ether/CH_2Cl_2 (1:1) in hexane) afforded the title compound as colourless flakes (38.1 mg, 0.0837 mmol, 84% yield, 91% *ee*).

^{1}H NMR (700 MHz, CD_3CN): δ 7.46-7.39 (m, 5H, H-11, H-12, H-13), 7.20 (br s, 1H, H-5), 5.01-4.98 (m, 1H, H-7), 4.73 (d, J = 5.1 Hz, 1H, H-6), 4.47-4.42 (m, 2H, H-4a, H-9a), 4.25 (dd, J = 8.9, 6.9 Hz, 1H, H-9b), 4.20 (q, J = 12.6 Hz, 1H, H-4b).

^{13}C NMR (176 MHz, CD_3CN): δ 155.4, 137.0, 130.1, 130.0, 128.4, 118.4 (qt, $J_{C\text{-}F}$ = 286.8, 33.4 Hz), 114.4 (tt, $J_{C\text{-}F}$ = 256.4, 31.3 Hz), 111.2-107.6 (m), 78.9, 67.7, 65.0 (t, $J_{C\text{-}F}$ = 27.0 Hz), 60.8.

^{19}F NMR (376 MHz, CD_3CN): δ -82.7 (t, J = 9.3 Hz), -122.1 - -122.3 (m), -129.0 - -129.1 (m).

R_f = 0.25 (60% v/v diethyl ether/ CH_2Cl_2 (1:1) in hexane).

$[\alpha]_D^{25.0}$ = +30.2 (c. 2.11, $CHCl_3$).

HRMS (-ESI): *m/z* found $[M-H]^-$ 454.0189, $[C_{14}H_{11}F_7NO_6S]^-$ requires 454.0201, (δ = -2.6 ppm).

Chiral SFC Analysis: CHIRALPAK IC (CO_2:MeOH, 95:5, 1.25 mL min^{-1}, 40 ˚C), t_R = 5.6 (major), 6.0 (minor) minutes.

2,2,3,3,4,4,4-heptafluorobutyl ((R)-((S)-2-oxo-1,3-dioxolan-4-yl)(phenyl)methyl)sulfamate (***ent*-4a**)

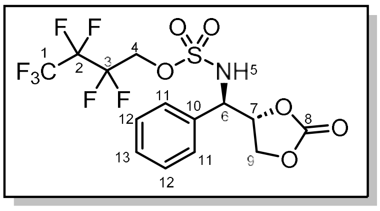

Prepared according to **GP 7** with catalyst Rh_2(**A1**)$_2$•(**C1**)$_2$•(Pyr)$_2$ (5.3 mg, 2 μmol, 2 mol%) (*Z*)-3-phenylallyl methyl(phenyl)carbamate **1a** (26.7 mg, 0.0999 mmol, 1.0 equiv.). Purification by flash column chromatography (SiO_2, 0-62% v/v diethyl ether/CH_2Cl_2 (1:1) in hexane) afforded the title compound as colourless flakes (30.4 mg, 0.0669 mmol, 67% yield, 88% *ee*).

[α]$_D^{25.0}$ = -31.2 (c. 2.03, $CHCl_3$).

Chiral SFC Analysis: CHIRALPAK IC (CO_2:MeOH, 95:5, 1.25 mL min^{-1}, 40 ˚C), t_R = 5.5 (minor), 5.9 (major) minutes.

2,2,3,3,4,4,4-heptafluorobutyl ((S)-(2-isopropylphenyl)((R)-2-oxo-1,3-dioxolan-4-yl)methyl)sulfamate (**4b**)

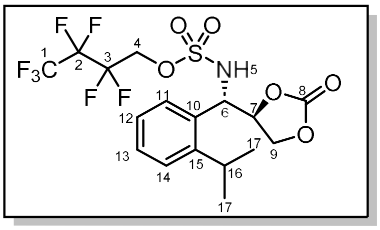

Prepared according to **GP 7** at -25 ˚C with (*Z*)-3-(2-isopropylphenyl)allyl methyl(phenyl)carbamate **1b** (30.5 mg, 0.0986 mmol, 1.0 equiv.). Purification by flash column chromatography (SiO_2, 0-5% acetone in $CHCl_3$, *then* 0-60% v/v diethyl ether/CH_2Cl_2 (1:1) in hexane) afforded the title compound as a pale-yellow oil (19.2 mg, 0.0386 mmol, 39% yield, 92% *ee*).

^{1}H NMR (500 MHz, $CDCl_3$): δ 7.41-7.35 (m, 3H, H-11, H-13, H-14), 7.27-7.24 (m, 1H, H-12), 6.49 (br s, 1H, H-5), 5.07 (d, *J* = 3.0 Hz, 1H, H-6), 4.95-4.91 (m, 1H, H-7), 4.58 (t, *J* = 8.7 Hz, 1H, H-9a), 4.45 (dd, *J* = 8.9, 7.0 Hz, 1H, H-9b), 4.28 (q, *J* = 12.7 Hz, 1H, H-4a), 3.94 (q, *J* = 12.7 Hz, 1H, H-4b), 3.18-3.10 (m, 1H, H-16), 1.28 (d, *J* = 9.4 Hz, 3H, H-17a), 1.27 (d, *J* = 9.5 Hz, 3H, H-17b).

^{13}C NMR (126 MHz, $CDCl_3$): δ 154.8, 146.1, 132.3, 129.8, 127.0, 126.58, 126.56, 117.4 (qt, $J_{C\text{-}F}$ = 287.8, 32.4 Hz), 112.9 (tt, $J_{C\text{-}F}$ = 259.0, 32.4 Hz), 110.3-105.8 (m), 78.7, 67.1, 64.4 (t, $J_{C\text{-}F}$ = 27.1 Hz), 54.6, 28.9, 24.1, 23.9.

^{19}F NMR (376 MHz, $CDCl_3$): δ -81.9 (t, *J* = 9.3 Hz), -121.86 - -121.94 (m), -128.57 - -128.65 (m).

R_f = 0.40 (10% v/v acetone in $CHCl_3$), 0.28 (60% v/v diethyl ether/ CH_2Cl_2 (1:1) in hexane).

[α]$_D^{25.0}$ = +17.4 (c. 1.20, $CHCl_3$).

HRMS (-ESI): *m/z* found $[M-H]^-$ 496.0670, $[C_{17}H_{17}F_7NO_6S]^-$ requires 496.0670, (δ = ±0.0 ppm).

Chiral SFC Analysis: CHIRALPAK IK (CO_2:MeOH, 97:3, 2.50 mL min^{-1}, 40 ˚C), t_R = 4.9 (minor), 5.5 (major) minutes.

2,2,3,3,4,4,4-heptafluorobutyl ((S)-(2-methoxyphenyl)((R)-2-oxo-1,3-dioxolan-4-yl)methyl)sulfamate (**4c**)

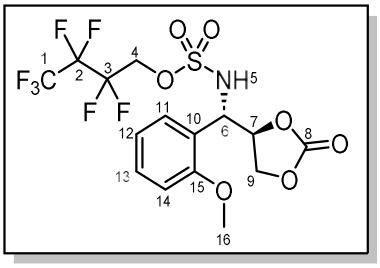

Prepared according to **GP 7** with (*Z*)-3-(2-methoxyphenyl)allyl methyl(phenyl)carbamate **1c** (31.8 mg, 0.107 mmol, 1.0 equiv.). Purification by flash column chromatography (SiO_2, 0-62% v/v diethyl ether/CH_2Cl_2 (1:1) in hexane) afforded the title compound as a pale-yellow oil (37 mg, 0.0762 mmol, 71% yield, 91% *ee*).

^{1}H NMR (700 MHz, CDCl$_3$): δ 7.37 (td, J = 7.9, 1.6 Hz, 1H, H-13), 7.30 (dd, J = 7.6, 1.5 Hz, 1H, H-11), 7.00 (td, J = 7.5, 0.8 Hz, 1H, H-12), 6.95 (d, J = 8.2 Hz, 1H, H-14), 6.65 (d, J = 8.5 Hz, 1H, H-5), 5.08-5.04 (m, 1H, H-7), 4.85 (t, J = 6.6 Hz, 1H, H-6), 4.40-4.35 (m, 2H, H-4a, H-9a), 4.29 (dd, J = 8.9, 6.7 Hz, 1H, H-9b), 4.21 (q, J = 12.6 Hz, 1H, H-4b), 3.89 (s, 3H, H-16).

^{13}C NMR (176 MHz, CDCl$_3$): δ 156.5, 154.8, 131.1, 129.4, 122.2, 121.7, 117.5 (qt, J_{C-F} = 288.3, 33.3 Hz), 113.1 (tt, J_{C-F} = 258.9, 31.4 Hz), 111.5, 110.2-106.6 (m), 77.2, 66.9, 64.4 (t, J_{C-F} = 27.4 Hz), 57.7, 55.8.

^{19}F NMR (471 MHz, CDCl$_3$): δ -80.9 (t, J = 9.2 Hz), -120.9 - -121.0 (m), -127.6 - -127.7 (m).

R_f = 0.27 (60% v/v diethyl ether/ CH_2Cl_2 (1:1) in hexane).

$[\alpha]_D^{25.0}$ = +28.1 (c. 2.68, $CHCl_3$).

HRMS (-ESI): *m/z* found $[M-H]^-$ 484.0300, $[C_{15}H_{13}F_7NO_7S]^-$ requires 484.0306, (δ = -1.2 ppm).

Chiral SFC Analysis: CHIRALPAK IK (CO_2:MeOH, 96:4, 2.50 mL min^{-1}, 40 ˚C), t_R = 4.4 (minor), 5.2 (major) minutes.

2,2,3,3,4,4,4-heptafluorobutyl ((S)-(2-fluorophenyl)((R)-2-oxo-1,3-dioxolan-4-yl)methyl)sulfamate (**4d**)

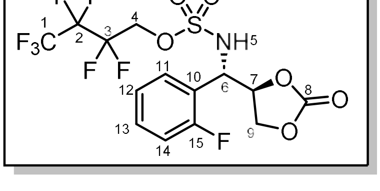

Prepared according to **GP 7** with (*Z*)-3-(2-fluorophenyl)allyl methyl(phenyl)carbamate **1d** (28.2 mg, 0.0988 mmol, 1.0 equiv.). Purification by flash column chromatography (SiO_2, 0-65% v/v diethyl ether/CH_2Cl_2 (1:1) in hexane) afforded the title compound as a pale yellow oil (23.5 mg, 0.0497 mmol, 50% yield, 90% *ee*).

^{1}H NMR (700 MHz, CDCl$_3$): δ 7.47 (td, J = 7.6, 1.5 Hz, 1 H, H-11), 7,41-7.38 (m, 1 H, H-13), 7.21 (td, J = 7.7, 1.0 Hz, 1 H, H-12), 7.15-7.12 (m, 1 H, H-14), 6.72 (br s, 1 H, H-5), 5.02-4.98 (m, 2 H, H-6, H-7), 4.59 (t, J = 8.7 Hz, 1 H, H-9a), 4.50 (dd, J = 9.0, 6.6 Hz, 1 H, H-9b), 4.40 (q, J = 12.8 Hz, 1 H, H-4a), 4.24 (q, J = 12.8 Hz, 1 H, H-4b).

^{13}C NMR (176 MHz, CDCl$_3$): δ 159.7 (d, $J_{C\text{-}F}$ = 247.0 Hz), 154.8, 131.4 (d, $J_{C\text{-}F}$ = 8.5 Hz), 128.8 (d, $J_{C\text{-}F}$ = 2.4 Hz), 125.4 (d, $J_{C\text{-}F}$ = 3.1 Hz), 122.5 (d, $J_{C\text{-}F}$ = 13.1 Hz), 117.4 (qt, $J_{C\text{-}F}$ = 287.8, 33.1 Hz), 116.2 (d, $J_{C\text{-}F}$ = 22.1 Hz), 113.0 (tt, $J_{C\text{-}F}$ = 258.4, 31.2 Hz), 110.2-106.5 (m), 77.8, 66.9, 64.5 (t, $J_{C\text{-}F}$ = 27.3 Hz), 54.2.

^{19}F NMR (376 MHz, CDCl$_3$): δ -81.9 (t, J = 9.2 Hz), -118.8, -121.8 - -122.0 (m), -128.57 - -128.64 (m).

R$_f$ = 0.28 (60% v/v diethyl ether/ CH_2Cl_2 (1:1) in hexane).

$[\alpha]_D^{25.0}$ = +29.1 (c. 2.05, $CHCl_3$).

HRMS (-ESI): *m/z* found [M−H]$^-$ 472.0093, $[C_{14}H_{10}F_8NO_6S]^-$ requires 472.0107, (δ = -3.0 ppm).

Chiral SFC Analysis: CHIRALPAK IC (CO_2:MeOH, 98:2, 2.50 mL min^{-1}, 40 ˚C), t$_R$ = 6.6 (major), 7.4 (minor) minutes.

2,2,3,3,4,4,4-heptafluorobutyl ((S)-((R)-2-oxo-1,3-dioxolan-4-yl)(o-tolyl)methyl)sulfamate (**4e**)

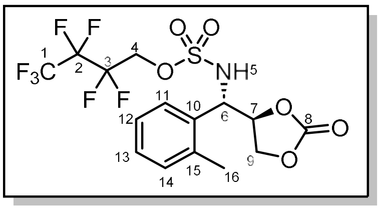

Prepared according to **GP 7** with (*Z*)-3-(*o*-tolyl)allyl methyl(phenyl)carbamate **1e** (29.1 mg, 0.103 mmol, 1.0 equiv.). Purification by flash column chromatography (SiO_2, 0-62% v/v diethyl ether/CH_2Cl_2 (1:1) in hexane) afforded the title compound as a pale-yellow oil (14.2 mg, 0.0303 mmol, 29% yield, 92% *ee*).

^{1}H NMR (700 MHz, CDCl$_3$): δ 7.42 (d, J = 7.3 Hz, 1H, H-11), 7.30-7.24 (m, 3H, H-12, H-13, H-14), 6.50 (br s, 1H, H-5), 5.00-4.93 (m, 2H, H-6, H-7), 4.56 (t, J = 8.6 Hz, 1H, H-9a), 4.42 (dd, J = 8.4, 7.2 Hz, 1H, H-9b), 4.27 (q, J = 12.8 Hz, 1H, H-4a), 3.93 (q, J = 12.5 Hz, 1H, H-4b), 2.42 (s, 3H, H-16).

^{13}C NMR (176 MHz, CDCl$_3$): δ 154.8, 135.6, 133.6, 131.5, 129.5, 127.4, 126.7, 117.4 (qt, $J_{C\text{-}F}$ = 287.6, 33.3 Hz), 112.9 (tt, $J_{C\text{-}F}$ = 259.0, 31.3 Hz), 110.2-106.5 (m), 78.0, 67.1, 64.4 (t, $J_{C\text{-}F}$ = 27.1 Hz), 55.5, 19.5.

^{19}F NMR (376 MHz, $CDCl_3$): δ -81.9 (t, J = 9.2 Hz), -121.8 - -122.0 (m), -128.6 - -128.7 (m).

R_f = 0.27 (60% v/v diethyl ether/ CH_2Cl_2 (1:1) in hexane).

$[\alpha]_D^{25.0}$ = +16.8 (c. 1.48, $CHCl_3$).

HRMS (-ESI): *m/z* found $[M{-}H]^-$ 468.0352, $[C_{15}H_{13}F_7NO_6S]^-$ requires 468.0357, (δ = -1.1 ppm).

Chiral SFC Analysis: CHIRALPAK IK (CO_2:MeOH, 96:4, 2.50 mL min^{-1}, 40 ˚C), t_R = 3.8 (minor), 4.3 (major) minutes.

2,2,3,3,4,4,4-heptafluorobutyl ((S)-(3-((tert-butoxycarbonyl)(methyl)amino)phenyl)((R)-2-oxo-1,3-dioxolan-4-yl)methyl)sulfamate (**4f**)

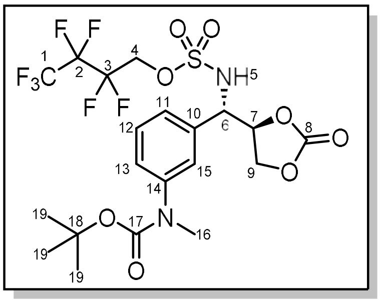

Prepared according to **GP 7** with (*Z*)-3-(3-((*tert*-butoxycarbonyl)(methyl)amino)phenyl)allylmethyl(phenyl)carbamate **1f** (42.1 mg, 0.106 mmol, 1.0 equiv.). Purification by flash column chromatography (SiO_2, 0-10% v/v acetone in $CHCl_3$) afforded the title compound as a colourless oil (28.4 mg, 0.0486 mmol, 46% yield, 80% *ee*).

^{1}H NMR (700 MHz, CD_3CN): δ 7.41 (t, J = 7.9 Hz, 1H, H-12), 7.33 (t, J = 1.8 Hz, 1H, H-15), 7.31-7.29 (m, 1H, H-13), 7.20 (d, J = 7.7 Hz, 1H, H-11), 7.20 (br s, 1H, H-5), 4.98-4.95 (m, 1H, H-7), 4.73 (d, J = 5.0 Hz, 1H, H-6), 4.47 (q, J = 12.7 Hz, 1H, H-4a), 4.45 (t, J = 8.7 Hz, 1H, H-9a), 4.29 (q, J = 12.8 Hz, 1H, H-4b), 4.26 (dd, J = 8.9, 6.8 Hz, 1H, H-9b), 3.21 (s, 3H, H-16), 1.43 (s, 9H, H-19).

^{13}C NMR (176 MHz, CD_3CN): δ 155.3, 155.2, 145.7, 137.5, 130.3, 126.6, 125.6, 125.0, 118.4 (qt, $J_{C\text{-}F}$ = 287.1, 33.6 Hz), 114.4 (tt, $J_{C\text{-}F}$ = 257.7, 30.8 Hz), 111.2-107.8 (m), 81.1, 78.9, 67.6, 65.1 (t, $J_{C\text{-}F}$ = 27.2 Hz), 60.5, 37.6, 28.4.

^{19}F NMR (376 MHz, CD_3CN): δ -82.7 (t, J = 9.4 Hz), -122.1 - -122.2 (m), -128.9 - -129.0 (m).

R_f = 0.44 (10% v/v acetone in $CHCl_3$).

$[\alpha]_D^{25.0}$ = +27.0 (c. 1.89, $CHCl_3$).

HRMS (-ESI): *m/z* found $[M{-}H]^-$ 583.0984, $[C_{20}H_{22}F_7N_2O_8S]^-$ requires 583.0991, (δ = -1.2 ppm).

Chiral SFC Analysis: CHIRALPAK IK (CO_2:MeOH, 96:4, 2.50 mL min^{-1}, 40 ˚C), t_R = 4.7 (minor), 5.1 (major) minutes.

2,2,3,3,4,4,4-heptafluorobutyl ((S)-(3-(tert-butyl)phenyl)((R)-2-oxo-1,3-dioxolan-4-yl)methyl)sulfamate (**4g**)

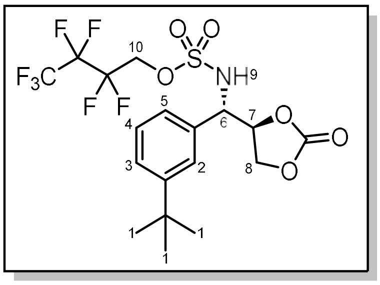

Prepared according to **GP 7** with (*Z*)-3-(3-(*tert*-butyl)phenyl)allyl methyl(phenyl)carbamate **1g** (32.3 mg, 0.10 mmol, 1.0 equiv.). Purification by flash column chromatography (SiO_2, 0-5% v/v acetone in $CHCl_3$) afforded the title compound as a white foam (32.9 mg, 0.0643 mmol, 64% yield, 93% *ee*).

^{1}H NMR (700 MHz, $CDCl_3$): δ 7.43 (ddd, *J* = 7.9, 2.0, 1.1 Hz, 1H, H-3), 7.39 (t, *J* = 1.9 Hz, 1H, H-2), 7.35 (t, *J* = 7.7 Hz, 1H, H-4), 7.22 (dt, *J* = 7.7, 1.3 Hz, 1H, H-5), 6.59 (br s, 1H, H-9), 5.02-4.99 (m, 1H, H-7), 4.59 (d, *J* = 1.9 Hz, 1H, H-6), 4.56 (t, *J* = 8.6 Hz, 1H, H-8a), 4.48 (dd, *J* = 8.9, 6.9 Hz, 1H, H-8b), 4.27 (q, *J* = 12.5 Hz, 1H, H-10a), 3.95 (q, *J* = 12.9 Hz, 1H, H-10b), 1.31 (s, 9H, H-1).

^{13}C NMR (176 MHz, $CDCl_3$): δ 154.9, 153.0, 135.0, 129.4, 126.8, 124.4, 124.2, 117.4 (qt, J_{C-F} = 287.7, 34.0 Hz), 112.9 (tt, J_{C-F} = 258.1, 30.9 Hz), 110.6-105.8 (m), 78.5, 67.1, 64.3 (t, J_{C-F} = 27.0 Hz), 60.4, 35.0, 31.3.

^{19}F NMR (376 MHz, $CDCl_3$): δ -81.9 (t, *J* = 9.8 Hz), -121.8 - -121.9 (m), -128.5 - -128.6 (m).

R_f = 0.35 (5% v/v acetone in $CHCl_3$).

$[\alpha]_D^{25.0}$ = +42.3 (c. 2.00, $CHCl_3$).

HRMS (–ESI): *m/z* found [M−H]$^-$ 510.0817, $[C_{18}H_{19}F_7NO_6S]^-$ requires 510.0827, (δ = −2.0 ppm).

Chiral SFC Analysis: CHIRALPAK IK (CO_2:MeOH, 97:3, 2.50 mL min^{-1}, 40 ˚C), t_R = 4.4 (minor), 5.2 (major) minutes.

2,2,3,3,4,4,4-heptafluorobutyl ((S)-(3-methoxyphenyl)((R)-2-oxo-1,3-dioxolan-4-yl)methyl)sulfamate (**4h**)

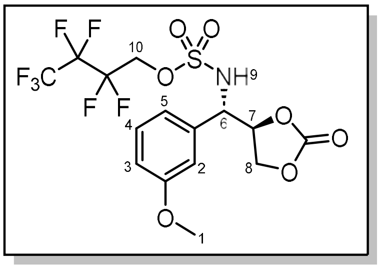

Prepared according to **GP 7** with (*Z*)-3-(3-methoxyphenyl)allyl methyl(phenyl)carbamate **1h** (29.7 mg, 0.10 mmol, 1.0 equiv.). Purification by flash column chromatography (SiO_2, 0-60% v/v diethyl ether/CH_2Cl_2 (1:1) in hexane) afforded the title compound as a white foam (27.8 mg, 0.0573 mmol, 57% yield, 92% *ee*).

^{1}H NMR (700 MHz, $CDCl_3$): *δ* 7.32 (t, *J* = 8.2 Hz, 1H, H-4), 6.96 (d, *J* = 7.7 Hz, 1H, H-5), 6.94-6.92 (m, 2H, H-2, H-3), 6.53 (br s, 1H, H-9), 5.00-4.98 (m, 1H, H-7), 4.57-4.54 (m, 2H, H-6, H-8a), 4.46 (dd, *J* = 9.1, 6.9 Hz, 1H, H-8b), 4.33 (q, *J* = 12.9 Hz, 1H, H-10a), 4.07 (q, *J* = 13.0 Hz, 1H, H-10b), 3.79 (s, 3H, H-1).

^{13}C NMR (176 MHz, $CDCl_3$): *δ* 160.5, 154.9, 136.7, 130.8, 119.4, 117.5 (qt, J_{C-F} = 287.7, 34.1 Hz), 115.0, 113.2, 113.0 (tt, J_{C-F} = 258.6, 32.0 Hz), 110.3-106.7 (m), 78.3, 67.1, 64.4 (t, J_{C-F} = 27.0 Hz), 60.1, 55.5.

^{19}F NMR (376 MHz, $CDCl_3$): *δ* -81.9 (t, *J* = 9.8 Hz), -121.7 - -121.9 (m), -128.5 - -128.7 (m).

R_f = 0.20 (60% v/v diethyl ether/ CH_2Cl_2 (1:1) in hexane).

$[\alpha]_D^{25.0}$ = +45.7 (c. 1.40, $CHCl_3$).

HRMS (–ESI): *m/z* found $[M-H]^-$ 484.0299, $[C_{15}H_{13}F_7NO_7S]^-$ requires 484.0306, (δ = −1.4 ppm).

Chiral SFC Analysis: CHIRALPAK IK (CO_2:MeOH, 96:4, 2.50 mL min^{-1}, 40 ˚C), t_R = 4.7 (minor), 5.3 (major) minutes.

2,2,3,3,4,4,4-heptafluorobutyl ((S)-(3-bromophenyl)((R)-2-oxo-1,3-dioxolan-4-yl)methyl)sulfamate (**4i**)

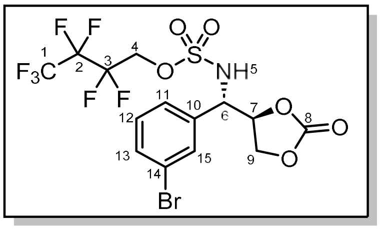

Prepared according to **GP 7** with catalyst Rh_2(**A1**)$_2$•(**B1**)$_2$•(Pyr)$_2$ (2.7 mg, 1 μmol, 1 mol%) and (*Z*)-3-(3-bromophenyl)allyl methyl(phenyl)carbamate **1i** (34.6 mg, 0.100 mmol, 1.0 equiv.). Purification by flash column chromatography (SiO_2, 0-62% v/v diethyl ether/CH_2Cl_2 (1:1) in hexane) afforded the title compound as a pale-yellow oil (30.8 mg, 0.0577 mmol, 58% yield, 87% *ee*).

^{1}H NMR (700 MHz, CD_3CN): *δ* 7.61 (t, *J* = 1.8 Hz, 1H, H-15), 7.58-7.57 (m, 1H, H-13), 7.40 (d, *J* = 7.8 Hz, 1H, H-11), 7.37 (t, *J* = 7.8 Hz, 1H, H-12), 7.20 (br s, 1H, H-5), 4.99-4.96 (m,

1H, H-7), 4.73 (d, J = 4.7 Hz, 1H, H-6), 4.53-4.47 (m, 2H, H-4a, H-9a), 4.31-4.26 (m, 2H, H-4b, H-9b).

^{13}C NMR (176 MHz, CD_3CN): δ 155.2, 139.6, 133.0, 132.0, 131.4, 127.4, 123.4, 118.4 (qt, J_{C-F} = 287.0, 33.3 Hz), 114.4 (tt, J_{C-F} = 257.2, 31.2 Hz), 111.2-107.5 (m), 78.7, 67.6, 65.0 (t, J_{C-F} = 27.1 Hz), 60.1.

^{19}F NMR (376 MHz, CD_3CN): δ -81.8 (t, J = 9.5 Hz), -121.2 - -121.3 (m), -128.0 - -128.1 (m).

R_f = 0.25 (60% v/v diethyl ether/ CH_2Cl_2 (1:1) in hexane).

$[\alpha]_D^{25.0}$ = +30.8 (c. 2.07, $CHCl_3$).

HRMS (-ESI): *m/z* found $[M-H]^-$ 531.9296, $[C_{14}H_{10}BrF_7NO_6S]^-$ requires 531.9306, (δ = -1.9 ppm).

Chiral SFC Analysis: CHIRALPAK IJ (CO_2:MeOH, 95:5, 2.50 mL min^{-1}, 40 ˚C), t_R = 4.0 (major), 4.4 (minor) minutes.

ethyl 3-((S)-(((2,2,3,3,4,4,4-heptafluorobutoxy)sulfonyl)amino)((R)-2-oxo-1,3-dioxolan-4-yl)methyl)benzoate (**4j**)

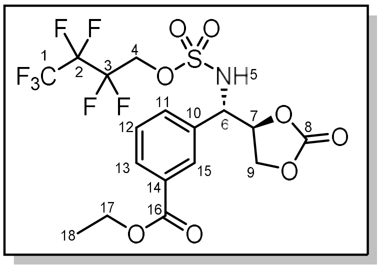

Prepared according to **GP 7** with ethyl (*Z*)-3-(3-((methyl(phenyl)carbamoyl)oxy)prop-1-en-1-yl)benzoate **1j** (34.6 mg, 0.102 mmol, 1.0 equiv.). Purification by flash column chromatography (SiO_2, 0-70% v/v diethyl ether/CH_2Cl_2 (1:1) in hexane) afforded the title compound as a pale yellow oil (32.7 mg, 0.0620 mmol, 61% yield, 88% *ee*).

^{1}H NMR (700 MHz, $CDCl_3$): δ 8.16 (s, 1H, H-15), 8.05 (d, J = 7.9 Hz, 1H, H-13), 7.61 (d, J = 7.7 Hz, 1H, H-11), 7.47 (t, J = 7.7 Hz, 1H, H-12), 7.22 (br s, 1H, H-5), 5.08-5.05 (m, 1H, H-7), 4.73 (d, J = 3.5 Hz, 1H, H-6), 4.58 (t, J = 8.5 Hz, 1H, H-9a), 4.50 (t, J = 7.9 Hz, 1H, H-9b), 4.39-4.34 (m, 3H, H-4a, H-17), 4.15 (q, J = 12.8 Hz, 1H, H-4b), 1.37 (t, J = 7.1 Hz, 3H, H-18).

^{13}C NMR (176 MHz, $CDCl_3$): δ 166.3, 154.9, 136.1, 132.1, 131.6, 130.5, 129.6, 128.4, 117.4 (qt, J_{C-F} = 286.8, 34.4 Hz), 113.0 (tt, J_{C-F} = 259.4, 30.9 Hz), 110.1-106.5 (m), 78.3, 67.0, 64.3 (t, J_{C-F} = 27.3 Hz), 61.8, 59.8, 14.3.

^{19}F NMR (376 MHz, $CDCl_3$): δ -81.9 (t, J = 9.1 Hz), -121.8 - -122.0 (m), -128.6 - -128.7 (m).

R_f = 0.16 (60% v/v diethyl ether/ CH_2Cl_2 (1:1) in hexane).

$[\alpha]_D^{25.0}$ = +24.9 (c. 2.71, $CHCl_3$).

HRMS (-ESI): *m/z* found $[M-H]^-$ 526.0407, $[C_{17}H_{15}F_7NO_8S]^-$ requires 526.0412, (δ = -1.0 ppm).

Chiral SFC Analysis: CHIRALPAK IC (CO_2:MeOH, 96:4, 2.50 mL min^{-1}, 40 ˚C), t_R = 4.7 (major), 5.9 (minor) minutes.

2,2,3,3,4,4,4-heptafluorobutyl ((S)-(3,4-dichlorophenyl)((R)-2-oxo-1,3-dioxolan-4-yl)methyl)sulfamate (**4k**)

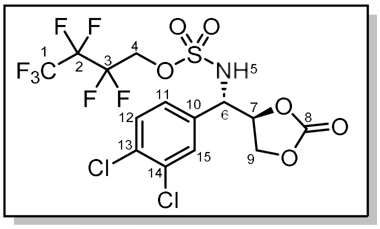

Prepared according to **GP 7** with (*Z*)-3-(3,4-dichlorophenyl)allyl methyl(phenyl)carbamate **1k** (33.8 mg, 0.101 mmol, 1.0 equiv.). Purification by flash column chromatography (SiO_2, 0-70% v/v diethyl ether/CH_2Cl_2 (1:1) in hexane) afforded the title compound as a colourless oil (21.5 mg, 0.0410 mmol, 41% yield, 90% *ee*).

^{1}H NMR (700 MHz, CD_3CN): δ 7.60 (d, *J* = 5.2 Hz, 1H, H-12), 7.59 (s, 1H, H-15), 7.35 (dd, *J* = 8.4, 2.0 Hz, 1H, H-11), 7.23 (br s, 1H, H-5), 4.98-4.95 (m, 1H, H-7), 4.74 (d, *J* = 4.5 Hz, 1H, H-6), 4.54-4.48 (m, 2H, H-4a, H-9a), 4.33 (q, *J* = 12.8 Hz, 1H, H-4b), 4.27 (dd, *J* = 9.0, 6.8 Hz, 1H, H-9b).

^{13}C NMR (176 MHz, CD_3CN): δ 155.2, 137.9, 133.54, 133.48, 132.2, 130.6, 128.5, 118.4 (qt, $J_{C\text{-}F}$ = 287.0, 34.1 Hz), 114.4 (tt, $J_{C\text{-}F}$ = 258.7, 32.7 Hz), 111.2-107.7 (m), 78.6, 67.6, 65.1 (t, $J_{C\text{-}F}$ = 26.9 Hz), 59.6.

^{19}F NMR (376 MHz, CD_3CN): δ -82.7 (t, *J* = 9.5 Hz), -122.2 - -122.3 (m), -129.0 - -129.1 (m).

R_f = 0.52 (80% v/v diethyl ether/ CH_2Cl_2 (1:1) in hexane).

$[\alpha]_D^{25.0}$ = +35.2 (c. 1.43, $CHCl_3$).

HRMS (-ESI): *m/z* found $[M-H]^-$ 521.9418, $[C_{14}H_9F_7NCl_2O_6S]^-$ requires 521.9421, (δ = -0.6 ppm).

Chiral SFC Analysis: CHIRALPAK IG (CO_2:MeOH, 95:5, 2.50 mL min^{-1}, 40 ˚C), t_R = 6.7 (minor), 7.3 (major) minutes.

2,2,3,3,4,4,4-heptafluorobutyl ((S)-(4-(tert-butyl)phenyl)((R)-2-oxo-1,3-dioxolan-4-yl)methyl)sulfamate (**4l**)

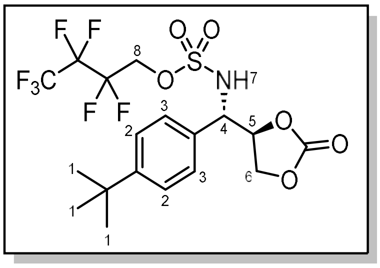

Prepared according to **GP 7** with (*Z*)-3-(4-(*tert*-butyl)phenyl)allyl methyl(phenyl)carbamate **1l** (32.3 mg, 0.10 mmol, 1.0 equiv.). Purification by flash column chromatography (SiO_2, 0-60% v/v diethyl ether/CH_2Cl_2 (1:1) in hexane) afforded the title compound as a white foam (31.9 mg, 0.0624 mmol, 62% yield, 97% *ee*).

^{1}H NMR (700 MHz, $CDCl_3$): δ 7.43 (d, J = 8.6 Hz, 2H, H-2), 7.34 (d, J = 8.4 Hz, 2H, H-3), 5.02-4.99 (m, 1H, H-5), 4.82 (br s, 1H, H-7), 4.58 (d, J = 4.0 Hz, 1H, H-4), 4.55 (t, J = 8.7 Hz, 1H, H-6a), 4.46 (dd, J = 8.9, 6.9 Hz, 1H, H-6b), 4.26 (q, J = 12.5 Hz, 1H, H-8a), 3.95 (q, J = 12.5 Hz, 1H, H-8b), 1.30 (s, 9H, H-1).

^{13}C NMR (176 MHz, $CDCl_3$): δ 155.0, 153.1, 132.3, 127.2, 126.5, 117.4 (qt, $J_{C\text{-}F}$ = 286.8, 33.6 Hz), 113.0 (tt, $J_{C\text{-}F}$ = 258.8, 31.2 Hz), 110.3-106.3 (m), 78.4, 67.2, 64.3 (t, $J_{C\text{-}F}$ = 27.3 Hz), 59.9, 34.8, 31.2.

^{19}F NMR (376 MHz, $CDCl_3$): δ -81.9 (t, J = 9.8 Hz), -121.8 - -121.9 (m), -128.5 - -128.6 (m).

R_f = 0.28 (60% v/v diethyl ether/ CH_2Cl_2 (1:1) in hexane).

$[\alpha]_D^{25.0}$ = +52.2 (c. 1.40, $CHCl_3$).

HRMS (+ESI): *m/z* found $[M+Na]^+$ 534.0790, $[C_{18}H_{20}F_7NNaO_6S]^+$ requires 534.0792, (δ = -0.4 ppm).

Chiral SFC Analysis: CHIRALPAK IK (CO_2:MeOH, 97:3, 2.50 mL min^{-1}, 40 ˚C), t_R = 6.1 (minor), 6.7 (major) minutes.

2,2,3,3,4,4,4-heptafluorobutyl ((S)-((R)-2-oxo-1,3-dioxolan-4-yl)(4-(trifluoromethoxy)phenyl)methyl)sulfamate (**4m**)

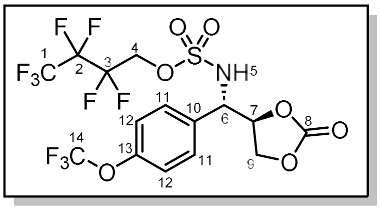

Prepared according to **GP 7** with (*Z*)-3-(4-(trifluoromethoxy)phenyl)allyl methyl(phenyl)carbamate **1m** (37.3 mg, 0.106 mmol, 1.0 equiv.). Purification by flash column chromatography (SiO_2, 0-60% v/v diethyl ether/CH_2Cl_2 (1:1) in hexane) afforded the title compound as a pale yellow oil (26.1 mg, 0.0484 mmol, 46% yield, 92% *ee*).

^{1}H NMR (700 MHz, CD_3CN): δ 7.52 (d, J = 8.5 Hz, 2H, H-11), 7.36 (d, J = 8.4 Hz, 2H, H-12), 7.27 (br s, 1H, H-5), 4.99-4.96 (m, 1H, H-7), 4.78 (d, J = 4.5 Hz, 1H, H-6), 4.50-4.44 (m, 2H, H-4a, H-9a), 4.27 (dd, J = 8.8, 6.9 Hz, 1H, H-9b), 4.24 (q, J = 12.9 Hz, 1H, H-4b).

^{13}C NMR (176 MHz, CD_3CN): δ 155.3, 150.3, 136.4, 130.4, 122.6, 121.4 (q, $J_{C\text{-}F}$ = 255.6 Hz), 118.4 (qt, $J_{C\text{-}F}$ = 286.9, 33.9 Hz), 114.3 (tt, $J_{C\text{-}F}$ = 257.2, 30.9 Hz), 111.2-107.5 (m), 78.8, 67.6, 65.0 (t, $J_{C\text{-}F}$ = 26.9 Hz), 59.9.

^{19}F NMR (376 MHz, CD_3CN): δ -59.8, -82.8 (t, J = 9.4 Hz), -122.3 - -122.4 (m), -129.1 - -129.2 (m).

R_f = 0.52 (80% v/v diethyl ether/ CH_2Cl_2 (1:1) in hexane).

$[\alpha]_D^{25.0}$ = +29.0 (c. 1.77, $CHCl_3$).

HRMS (-ESI): *m/z* found $[M-H]^-$ 538.0017, $[C_{15}H_{10}F_{10}NO_7S]^-$ requires 538.0024, (δ = -1.3 ppm).

Chiral SFC Analysis: CHIRALPAK IJ (CO_2:MeOH, 97:3, 1.25 mL min^{-1}, 40 ˚C), t_R = 6.7 (major), 7.6 (minor) minutes.

2,2,3,3,4,4,4-heptafluorobutyl ((S)-(4-chlorophenyl)((R)-2-oxo-1,3-dioxolan-4-yl)methyl)sulfamate (**4n**)

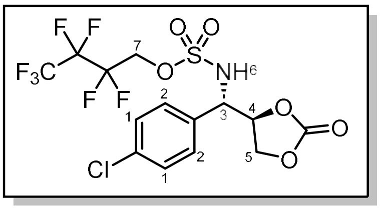

Prepared according to **GP 7** with (*Z*)-3-(4-chlorophenyl)allyl methyl(phenyl)carbamate **1n** (30.2 mg, 0.10 mmol, 1.0 equiv.). Purification by flash column chromatography (SiO_2, 0-60% v/v diethyl ether/CH_2Cl_2 (1:1) in hexane) afforded the title compound as a white foam (27.3 mg, 0.0557 mmol, 56% yield, 90% *ee*).

^{1}H NMR (700 MHz, $CDCl_3$): δ 7.39 (d, J = 8.6 Hz, 2H, H-1), 7.35 (d, J = 8.7 Hz, 2H, H-2), 6.75 (br s, 1H, H-6), 5.00-4.97 (m, 1H, H-4), 4.61-4.59 (m, 2H, H-3, H-5a), 4.54-4.48 (m, 1H, H-5b), 4.37 (q, J = 12.8 Hz, 1H, H-7a), 4.14 (q, J = 12.6 Hz, 1H, H-7b).

^{13}C NMR (176 MHz, $CDCl_3$): δ 154.9, 135.9, 133.9, 129.8, 128.7, 117.4 (qt, $J_{C\text{-}F}$ = 286.7, 32.6 Hz), 113.0 (tt, $J_{C\text{-}F}$ = 258.1, 30.9 Hz), 110.2-106.5 (m), 78.4, 67.1, 64.4 (t, $J_{C\text{-}F}$ = 27.0 Hz), 59.4.

^{19}F NMR (376 MHz, $CDCl_3$): δ -81.9 (t, J = 9.8 Hz), -121.6 - -121.9 (m), -128.4 - -128.7 (m).

R_f = 0.23 (60% v/v diethyl ether/ CH_2Cl_2 (1:1) in hexane).

$[\alpha]_D^{25.0}$ = +51.5 (c. 1.50, $CHCl_3$).

HRMS (–ESI): *m/z* found $[M-H]^-$ 487.9816, $[C_{14}H_{10}ClF_7NO_6S]^-$ requires 487.9811, (δ = +1.0 ppm).

Chiral SFC Analysis: CHIRALPAK IJ (CO_2:MeOH, 97:3, 2.50 mL min^{-1}, 40 ˚C), t_R = 6.4 (major), 7.6 (minor) minutes.

2,2,3,3,4,4,4-heptafluorobutyl ((S)-((R)-2-oxo-1,3-dioxolan-4-yl)(4-(trifluoromethyl)phenyl)methyl)sulfamate (**4o**)

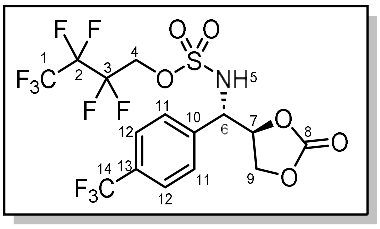

Prepared according to **GP 7** with (*Z*)-3-(4-(trifluoromethyl)phenyl)allyl methyl(phenyl)carbamate **1o** (33.2 mg, 0.0990 mmol, 1.0 equiv.). Purification by flash column chromatography (SiO_2, 0-70% v/v diethyl ether/CH_2Cl_2 (1:1) in hexane) afforded the title compound as a pale-yellow oil (22.1 mg, 0.0422 mmol, 43% yield, 95% *ee*).

^{1}H NMR (700 MHz, $CDCl_3$): δ 7.68 (d, *J* = 8.1 Hz, 2H, H-12), 7.56 (d, *J* = 8.1 Hz, 2H, H-11), 6.90 (br s, 1H, H-5), 5.03-5.01 (m, 1H, H-7), 4.71 (d, *J* = 2.7 Hz, 1H, H-6), 4.67 (t, *J* = 8.8 Hz, 1H, H-9a), 4.56 (dd, *J* = 8.9, 6.8 Hz, 1H, H-9b), 4.37 (q, *J* = 12.5 Hz, 1H, H-4a), 4.15 (q, *J* = 12.5 Hz, 1H, H-4b).

^{13}C NMR (176 MHz, $CDCl_3$): δ 154.9, 139.4, 132.0 (q, $J_{C\text{-}F}$ = 33.4 Hz). 127.8, 126.5 (q, $J_{C\text{-}F}$ = 3.5 Hz), 123.7 (q, $J_{C\text{-}F}$ = 272.2 Hz), 117.3 (qt, $J_{C\text{-}F}$ = 288.6, 32.8 Hz), 112.9 (tt, $J_{C\text{-}F}$ = 257.9, 32.8 Hz), 110.1-106.5 (m), 78.3, 67.1, 64.4 (t, $J_{C\text{-}F}$ = 27.5 Hz), 59.5.

^{19}F NMR (376 MHz, $CDCl_3$): δ -64.2, -82.0 (t, *J* = 9.3 Hz), -121.8 - -121.9 (m), -128.6 - -128.7 (m).

R_f = 0.21 (60% v/v diethyl ether/ CH_2Cl_2 (1:1) in hexane).

$[\alpha]_D^{25.0}$ = +21.8 (c. 1.65, $CHCl_3$).

HRMS (-ESI): *m/z* found $[M-H]^-$ 522.0064, $[C_{15}H_{10}F_{10}NO_6S]^-$ requires 522.0075, (δ = -2.1 ppm).

Chiral SFC Analysis: CHIRALPAK IJ (CO_2:MeOH, 97:3, 2.50 mL min^{-1}, 40 ˚C), t_R = 3.8 (major), 4.4 (minor) minutes.

2,2,3,3,4,4,4-heptafluorobutyl ((S)-naphthalen-1-yl((R)-2-oxo-1,3-dioxolan-4-yl)methyl)sulfamate (**4p**)

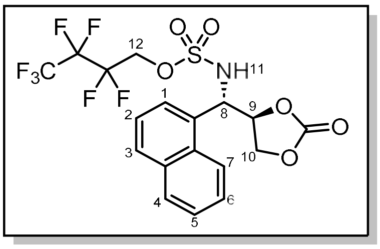

Prepared according to **GP 7** with (*Z*)-3-(naphthalen-1-yl)allyl methyl(phenyl)carbamate **1p** (31.7 mg, 0.10 mmol, 1.0 equiv.). Purification by flash column chromatography (SiO_2, 0-60% v/v diethyl ether/CH_2Cl_2 (1:1) in hexane) afforded the title compound as a white foam (27.9 mg, 0.0552 mmol, 55% yield, 92% *ee*).

^{1}H NMR (700 MHz, CDCl$_3$): δ 8.00 (d, J = 8.6 Hz, 1H, H-7), 7.94 (d, J = 8.3 Hz, 1H, H-4), 7.91 (d, J = 8.2 Hz, 1H, H-3), 7.65 (d, J = 7.3 Hz, 1H, H-1), 7.62 (t, J = 7.7 Hz, 1H, H-6), 7.57 (t, J = 7.5 Hz, 1H, H-5), 7.48 (t, J = 7.6 Hz, 1H, H-2), 6.74 (br s, 1H, H-11), 5.54 (d, J = 3.5 Hz, 1H, H-8), 5.19-5.17 (m, 1H, H-9), 4.58 (t, J = 8.7 Hz, 1H, H-10a), 4.50 (t, J = 7.9 Hz, 1H, H-10b), 4.22 (q, J = 12.8 Hz, 1H, H-12a), 4.06 (q, J = 12.6 Hz, 1H, H-12b).

^{13}C NMR (176 MHz, CDCl$_3$): δ 154.9, 134.2, 130.7, 130.3, 130.1, 129.7, 127.7, 126.6, 125.5, 125.4, 121.5, 117.3 (qt, $J_{C\text{-}F}$ = 286.8, 33.5 Hz), 112.8 (tt, $J_{C\text{-}F}$ = 258.8, 31.2 Hz), 110.0-106.4 (m), 78.1, 67.2, 64.4 (t, $J_{C\text{-}F}$ = 27.3 Hz), 55.5.

^{19}F NMR (376 MHz, CDCl$_3$): δ -81.0 (t, J = 9.7 Hz), -121.0 - -121.1 (m), -127.7 - -127.8 (m).

R_f = 0.21 (60% v/v diethyl ether/ CH_2Cl_2 (1:1) in hexane).

$[\alpha]_D^{25.0}$ = +8.80 (c. 1.40, $CHCl_3$).

HRMS (–ESI): *m/z* found $[M-H]^-$ 504.0338, $[C_{18}H_{13}F_7NO_6S]^-$ requires 504.0357, (δ = -3.8 ppm).

Chiral SFC Analysis: CHIRALPAK IJ (CO_2:MeOH, 94:6, 2.50 mL min^{-1}, 40 ˚C), t_R = 3.9 (major), 4.8 (minor) minutes.

2,2,3,3,4,4,4-heptafluorobutyl ((S)-naphthalen-2-yl((R)-2-oxo-1,3-dioxolan-4-yl)methyl)sulfamate (**4q**)

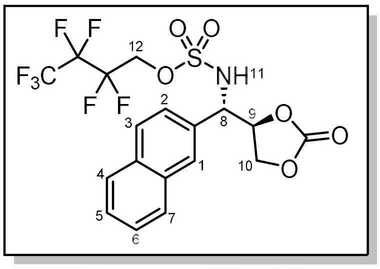

Prepared according to **GP 7** with (*Z*)-3-(naphthalen-2-yl)allyl methyl(phenyl)carbamate **1q** (31.7 mg, 0.10 mmol, 1.0 equiv.). Purification by flash column chromatography (SiO_2, 0-60% v/v diethyl ether/CH_2Cl_2 (1:1) in hexane) afforded the title compound as an off-white foam (10.0 mg, 0.0198 mmol, 20% yield, 84% *ee*).

^{1}H NMR (700 MHz, $CDCl_3$): δ 7.90 (d, J = 8.6 Hz, 1H, H-3), 7.87 (s, 1H, H-1), 7.85 (d, J = 8.0 Hz, 1H, H-4), 7.83 (d, J = 7.5 Hz, 1H, H-7), 7.56-7.52 (m, 2H, H-5, H-6), 7.48 (d, J = 8.4 Hz, 1H, H-2), 6.33 (br s, 1H, H-11), 5.11-5.08 (m, 1H, H-9), 4.78 (d, J = 3.8 Hz, 1H, H-8), 4.56 (t, J = 8.7 Hz, 1H, H-10a), 4.51 (dd, J = 8.9, 7.0 Hz, 1H, H-10b), 4.34 (q, J = 12.6 Hz, 1H, H-12a), 4.11 (q, J = 12.6 Hz, 1H, H-12b).

^{13}C NMR (176 MHz, $CDCl_3$): δ 154.5, 133.6, 133.3, 132.3, 129.9, 128.2, 127.9, 127.4, 127.3, 127.2, 123.9, 117.3 (qt, J = 287.5, 32.7 Hz), 113.0 (tt, J = 258.8, 31.6 Hz), 110.2-106.4 (m), 78.1, 67.0, 64.4 (t, J = 27.3 Hz), 60.4.

^{19}F NMR (376 MHz, $CDCl_3$): δ -80.9 (t, J = 9.6 Hz), -120.7 - -120.8 (m), -127.6 - -127.7 (m)

R_f = 0.24 (60% v/v diethyl ether/ CH_2Cl_2 (1:1) in hexane).

$[\alpha]_D^{25.0}$ = +48.5 (c. 0.46, $CHCl_3$).

HRMS (–ESI): *m/z* found $[M-H]^-$ 504.0347, $[C_{18}H_{13}F_7NO_6S]^-$ requires 504.0357, (δ = -2.0 ppm).

Chiral SFC Analysis: CHIRALPAK IJ (CO_2:MeOH, 94:6, 2.50 mL min^{-1}, 40 ˚C), t_R = 5.6 (major), 8.0 (minor) minutes.

2,2,3,3,4,4,4-heptafluorobutyl ((S)-((R)-2-oxo-1,3-dioxolan-4-yl)(thiophen-3-yl)methyl)sulfamate (**4r**)

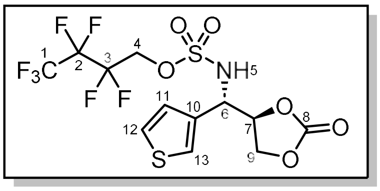

Prepared according to **GP 7** with (*Z*)-3-(thiophen-3-yl)allyl methyl(phenyl)carbamate **1r** (27.4 mg, 0.100 mmol, 1.0 equiv.). Purification by flash column chromatography (SiO_2, 0-65% v/v diethyl ether/CH_2Cl_2 (1:1) in hexane *then* 0-8% acetone in $CHCl_3$) afforded the title compound as a pale yellow oil (21.6 mg, 0.0468 mmol, 47% yield, 92% *ee*).

^{1}H NMR (700 MHz, $CDCl_3$): δ 7.43-7.41 (m, 2H, H-12, H-13), 7.18 (d, J = 5.0 Hz, 1H, H-11), 6.49 (br s, 1H, H-5), 5.05-5.02 (m, 1H, H-7), 4.76 (d, J = 2.7 Hz, 1H, H-6), 4.60 (t, J = 8.7 Hz, 1H, H-9a), 4.51 (dd, J = 8.8, 6.7 Hz, 1H, H-9b), 4.38 (q, J = 12.7 Hz, 1H, H-4a), 4.08 (q, J = 12.7 Hz, 1H, H-4b).

^{13}C NMR (176 MHz, $CDCl_3$): δ 155.0, 135.9, 128.1, 126.1, 124.7, 117.4 (qt, J_{C-F} = 287.6, 33.2 Hz), 113.1 (tt, J_{C-F} = 259.1, 31.9 Hz), 110.2-106.6 (m), 78.1, 67.0, 64.4 (t, J_{C-F} = 27.1 Hz), 55.6.

^{19}F NMR (376 MHz, $CDCl_3$): δ -81.8 (t, J = 9.2 Hz), -121.6 - -121.8 (m), -128.4 - -128.5 (m).

R_f = 0.25 (60% v/v diethyl ether/ CH_2Cl_2 (1:1) in hexane), 0.38 (10% v/v acetone in $CHCl_3$).

$[\alpha]_D^{25.0}$ = +31.6 (c. 1.74, $CHCl_3$).

HRMS (-ESI): *m/z* found $[M-H]^-$ 459.9759, $[C_{12}H_9F_7NO_6S_2]^-$ requires 459.9765, (δ = -1.3 ppm).

Chiral SFC Analysis: CHIRALPAK IK (CO_2:MeOH, 97:3, 2.50 mL min^{-1}, 40 ˚C), t_R = 5.9 (minor), 6.5 (major) minutes.

methyl 5-((S)-(((2,2,3,3,4,4,4-heptafluorobutoxy)sulfonyl)amino)((R)-2-oxo-1,3-dioxolan-4-yl)methyl)-1-tosyl-1H-indole-3-carboxylate (**4s**)

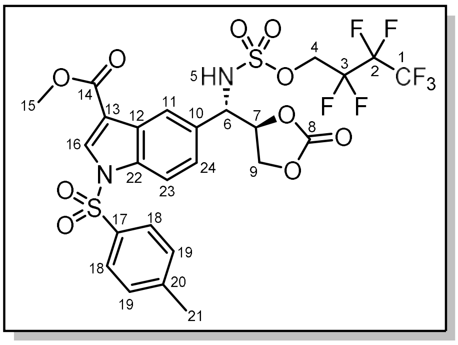

Prepared according to **GP 7** with methyl (*Z*)-5-(3-((methyl(phenyl)carbamoyl)oxy)prop-1-en-1-yl)-1-tosyl-1*H*-indole-3-carboxylate **1s** (54.2 mg, 0.105 mmol, 1.0 equiv.). Purification by flash column chromatography (SiO_2, 0-80% v/v diethyl ether/CH_2Cl_2 (1:1) in hexane) afforded the title compound as a white solid (29.7 mg, 0.0420 mmol, 40% yield, 91% *ee*).

^{1}H NMR (700 MHz, $CDCl_3$): δ 8.27 (s, 1H, H-16), 8.18 (d, *J* = 1.6 Hz, 1H, H-11), 7.98 (d, *J* = 8.6 Hz, 1H, H-23), 7.81 (d, *J* = 8.5 Hz, 2H, H-18), 7.40 (dd, *J* = 8.7, 1.7 Hz, 1H, H-24), 7.28 (d, *J* = 8.2 Hz, 2H, H-19), 6.99 (d, *J* = 8.8 Hz, 1H, H-5), 5.07-5.04 (m, 1H, H-7), 4.76 (dd, *J* = 8.8, 4.3 Hz, 1H, H-6), 4.52 (t, *J* = 8.7 Hz, 1H, H-9a), 4.46 (dd, *J* = 8.9, 7.0 Hz, 1H, H-9b), 4.32 (q, *J* = 12.9 Hz, 1H, H-4a), 4.10 (q, *J* = 12.9 Hz, 1H, H-4b), 3.87 (s, 3H, H-15), 2.36 (s, 3H, H-21).

^{13}C NMR (176 MHz, $CDCl_3$): δ 163.9, 154.9, 146.4, 135.0, 134.4, 133.2, 131.7, 130.4, 128.5, 127.3, 124.5, 121.0, 117.3 (qt, J_{C-F} = 287.4, 33.3 Hz), 114.4, 113.0, 112.9 (tt, J_{C-F} = 258.3, 31.7 Hz), 110.0-106.4 (m), 78.5, 67.1, 64.3 (t, J_{C-F} = 27.2 Hz), 60.2, 51.9, 21.7.

^{19}F NMR (376 MHz, $CDCl_3$): δ -82.0 (t, *J* = 8.8 Hz), -121.2 - -122.0 (m), -128.7 - -128.8 (m).

R_f = 0.43 (80% v/v diethyl ether/ CH_2Cl_2 (1:1) in hexane).

$[\alpha]_D^{25.0}$ = +34.1 (c. 1.82, $CHCl_3$).

HRMS (-ESI): *m/z* found $[M-H]^-$ 705.0432, $[C_{25}H_{20}F_7N_2O_{10}S_2]^-$ requires 705.0453, (δ = -3.0 ppm).

Chiral SFC Analysis: CHIRALPAK IC (CO_2:MeOH, 85:15, 2.50 mL min^{-1}, 40 ˚C), t_R = 4.3 (minor), 14.9 (major) minutes.

Kinetic Resolution of (*rac*)-1t

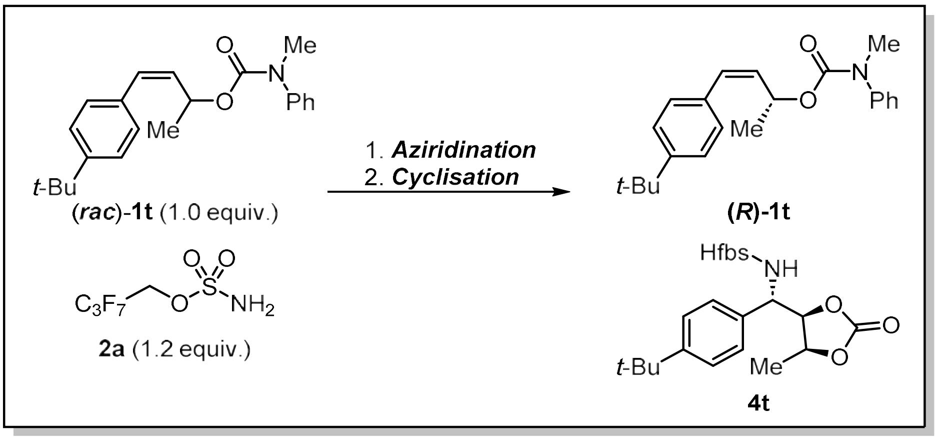

Prepared according to **GP 7** with (*Z*)-4-(4-(*tert*-butyl)phenyl)but-3-en-2-yl methyl(phenyl)carbamate **(*rac*)-1t** (33.9 mg, 0.100 mmol, 1.0 equiv.). Purification by flash column chromatography (SiO_2, 0-60% v/v diethyl ether/CH_2Cl_2 (1:1) in hexane) recovered (*R*,*Z*)-4-(4-(*tert*-butyl)phenyl)but-3-en-2-yl methyl(phenyl)carbamate **(*R*)-1t** (13.5 mg, 0.0400 mmol, 40% yield, 94% *ee*) and afforded the carbonate product **4t** as a colourless oil (19.2 mg, 0.0365 mmol, 37% yield, 97% *ee*).

(R,Z)-4-(4-(tert-butyl)phenyl)but-3-en-2-yl methyl(phenyl)carbamate ((***R***)-**1t**)

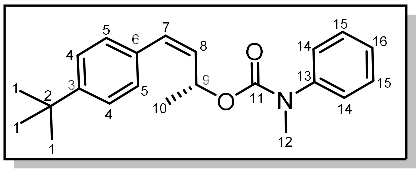

R_f = 0.88 (60% v/v diethyl ether/ CH_2Cl_2 (1:1) in hexane).

$[\alpha]_D^{25.0}$ = -130.8 (c. 0.72, $CHCl_3$).

Chiral SFC Analysis: CHIRALPAK IJ (CO_2:MeOH, 94:6, 2.50 mL min^{-1}, 40 ˚C), t_R = 3.4 (major), 5.7 (minor) minutes.

2,2,3,3,4,4,4-heptafluorobutyl ((S)-(4-(tert-butyl)phenyl)((4R,5S)-5-methyl-2-oxo-1,3-dioxolan-4-yl)methyl)sulfamate (**4t**)

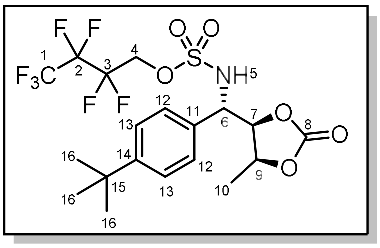

^{1}H NMR (500 MHz, CDCl$_3$): δ 7.43 (d, J = 8.5 Hz, 2H, H-13), 7.33 (d, J = 8.5 Hz, 2H, H-12), 6.21 (br s, 1H, H-5), 5.00 (dd, J = 7.6, 5.9 Hz, 1H, H-7), 4.89-4.83 (m, 1H, H-9), 4.70 (d, J = 5.6 Hz, 1H, H-6), 4.20 (q, J = 12.7 Hz, 1H, H-4a), 3.87 (q, J = 12.7 Hz, 1H, H-4b), 1.51 (d, J = 6.8 Hz, 3H, H-10), 1.30 (s, 9H, H-16).

^{13}C NMR (126 MHz, CDCl$_3$): δ 154.1, 153.2, 132.9, 127.3, 126.6, 117.4 (qt, $J_{C\text{-}F}$ = 286.9, 32.4 Hz), 112.9 (tt, $J_{C\text{-}F}$ = 259.1, 32.4 Hz), 110.8-105.6 (m), 80.4, 76.2, 64.2 (t, $J_{C\text{-}F}$ = 27.3 Hz), 57.2, 34.8, 31.2, 14.7.

^{19}F NMR (376 MHz, CDCl$_3$): δ -81.8 (t, J = 9.2 Hz), -121.8 - -121.9 (m), -128.5 - -128.6 (m).

R_f = 0.36 (60% v/v diethyl ether/ CH_2Cl_2 (1:1) in hexane).

$[\alpha]_D^{25.0}$ = +34.9 (c. 1.28, $CHCl_3$).

HRMS (+ESI): *m/z* found $[M+Na]^+$ 543.1389, $[C_{19}H_{26}N_2F_7O_6S]^+$ requires 543.1394, (δ = -0.9 ppm).

Chiral SFC Analysis: CHIRALPAK IC (CO_2:MeOH, 97:3, 2.50 mL min^{-1}, 40 ˚C), t_R = 6.0 (minor), 7.1 (major) minutes.

Note: The relative stereochemistry for this compound was tentatively assigned on the basis of ^{1}H-^{1}H NOESY experiments (see below). The NOESY shows strong nOes between H^{10} and H^6, as well as between H^{10} and H^9. The interaction between H^{10} and H^9 would be expected for both possible diastereomers but the similarly strong interaction between H^{10} and H^6 seems more consistent with the syn diastereomer shown, where the methyl group and the benzylic carbon are syn on the cyclic carbonate. In the possible anti diastereomer, H^{10} and H^6 would probably be more distant. In addition to this, and in contrast to the strong nOes mentioned above, there is no nOe observed between H^{10} and H^7. In a possible anti diastereomer, this nOe is expected to be strongly visible but there is no evidence of it in this compound. On the basis of the rationale above, we propose that the syn diastereomer is obtained for the cyclic carbonate, in the absence of other characterisation data to prove otherwise. For the absolute stereochemistry we tentatively assign in analogy with the rest of the compounds in the scope.

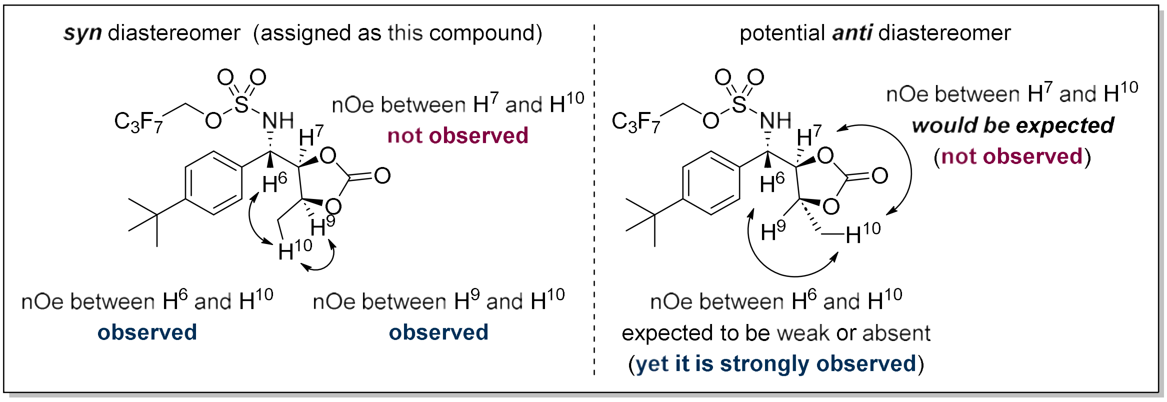

Kinetic Resolution of (*rac*)-1u

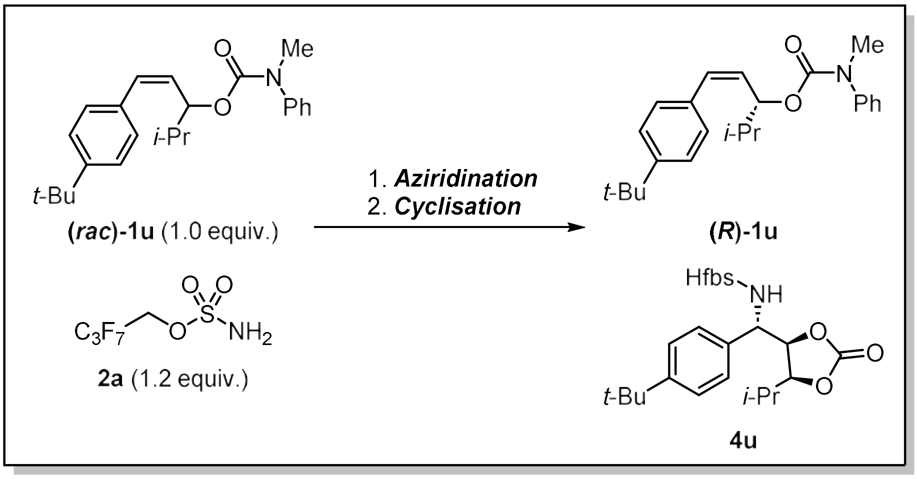

Prepared according to **GP 7** with (*Z*)-1-(4-(*tert*-butyl)phenyl)-4-methylpent-1-en-3-yl methyl(phenyl)carbamate **(*rac*)-1u** (36.6 mg, 0.100 mmol, 1.0 equiv.), 2,2,3,3,4,4,4-heptafluorobutyl sulfamate (41.9 mg, 0.15 mmol, 1.5 equiv.) and Rh_2(**A1**)$_2$•(**B1**)$_2$(Pyr)$_2$ (8.1 mg, 3 µmol, 3 mol%) at -10 ˚C. Purification by flash column chromatography (SiO_2, 0-3% v/v acetone in $CHCl_3$) recovered (*R*,*Z*)-1-(4-(*tert*-butyl)phenyl)-4-methylpent-1-en-3-yl methyl(phenyl)carbamate **(*R*)-1u** (16.3 mg, 0.0446 mmol, 45% yield, 57% *ee*) and afforded the carbonate product **4u** as a colourless oil (10.5 mg, 0.0190 mmol, 19% yield, 91% *ee*).

(R,Z)-1-(4-(tert-butyl)phenyl)-4-methylpent-1-en-3-yl methyl(phenyl)carbamate (**(*R*)-1u**)

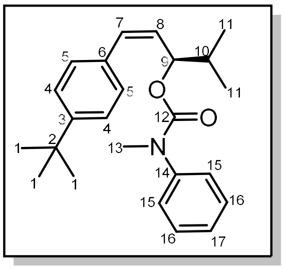

R_f = 0.93 (5% v/v acetone in $CHCl_3$).

$[\alpha]_D^{25.0}$ = -80.5 (c. 0.44, $CHCl_3$).

Chiral SFC Analysis: CHIRALPAK IC (CO_2:MeOH, 97:3, 1.25 mL min^{-1}, 40 ˚C), t_R = 11.7 (minor), 12.8 (major) minutes.

2,2,3,3,4,4,4-heptafluorobutyl ((S)-(4-(tert-butyl)phenyl)((4R,5S)-5-isopropyl-2-oxo-1,3-dioxolan-4-yl)methyl)sulfamate (**4u**)

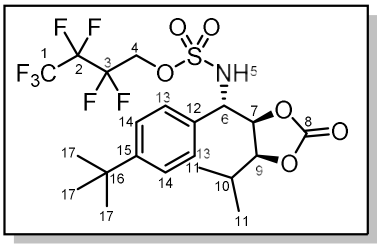

^{1}H NMR (700 MHz, $CDCl_3$): δ 7.43 (d, J = 8.4 Hz, 2H, H-14), 7.35 (d, J = 8.4 Hz, 2H, H-13), 6.05 (br s, 1H, H-5), 4.90 (dd, J = 7.2, 2.4 Hz, 1H, H-7), 4.82 (br s, 1H, H-6), 4.36 (dd, J = 9.6, 7.2 Hz, 1H, H-9), 4.21 (q, J = 12.7 Hz, 1H, H-4a), 3.86 (q, J = 12.7 Hz, 1H, H-4b), 2.48-2.43 (m, 1H, H-10), 1.30 (s, 9H, H-17), 1.19 (d, J = 6.6 Hz, 3H, H-11a), 1.00 (d, J = 6.7 Hz, 3H, H-11b).

^{13}C NMR (176 MHz, $CDCl_3$): δ 154.4, 153.0, 134.2, 126.9, 126.6, 85.4, 81.2, 64.2 (t, $J_{C\text{-}F}$ = 27.0 Hz), 56.7, 34.8, 31.2, 26.9, 19.4, 18.8.

^{19}F NMR (376 MHz, $CDCl_3$): δ -81.8 (t, J = 9.2 Hz), -121.8 - -121.9 (m), -128.5 - -128.6 (m).

R_f = 0.54 (5% v/v acetone in $CHCl_3$).

$[\alpha]_D^{25.0}$ = +39.6 (c. 0.45, $CHCl_3$).

HRMS (+ESI): *m/z* found $[M+NH_4]^+$ 571.1706, $[C_{21}H_{30}N_2F_7O_6S]^+$ requires 571.1707, (δ = -0.2 ppm).

Chiral SFC Analysis: CHIRALPAK IK (CO_2:MeOH, 97:3, 1.25 mL min^{-1}, 40 ˚C), t_R = 6.5 (minor), 8.2 (major) minutes.

Note: The diastereomer above has been tentatively assigned in analogy with the earlier methyl-substituted analogue.

2,2,3,3,4,4,4-heptafluorobutyl ((S)-((S)-5-oxotetrahydrofuran-2-yl)(phenyl)methyl)sulfamate (**9**)

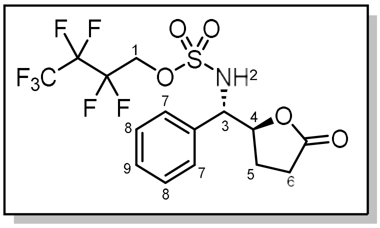

Prepared according to **GP 7** with (*Z*)-*N*-methyl-*N*,5-diphenylpent-4-enamide (26.5 mg, 0.100 mmol, 1.0 equiv.). Purification by flash column chromatography (SiO_2, 60% v/v diethyl ether/CH_2Cl_2 (1:1) in hexane, *then* 10% v/v acetone in $CHCl_3$) afforded the title compound as colourless oil (30.6 mg, 0.0675 mmol, 68% yield, 71% *ee*).

^{1}H NMR (700 MHz, $CDCl_3$): δ 7.41-7.35 (m, 5 H, H-7, H-8, H-9), 6.32 (d, J = 8.2 Hz, 0.84 H, H-2a)*, 6.13 (d, J = 8.0 Hz, 0.14 H, H-2b)*, 4.78-4.74 (m, 1 H, H-4), 4.54-4.52 (m, 1 H,

H-3), 4.29-4.22 (m, 1 H, H-1a), 4.08-4.00 (m, 1 H, H-1b), 2.65-2.54 (m, 2 H, H-6), 2.33-2.22 (m, 2 H, H-5).

* These signals were observed as two signals probably due to the rotamers. ^{1}H-^{1}H COSY experiment showed these signals are same position.

^{13}C NMR (176 MHz, $CDCl_3$)*: δ (177.0, 176.7), (136.9, 136.7), (129.36-129.32 (m), 129.2)†, (127.44, 127.38), 117.4 (qt, $J_{C\text{-}F}$ = 287.4, 33.3 Hz), 113.0 (tt, $J_{C\text{-}F}$ = 258.5, 31.2 Hz), 110.0-106.8 (m), (82.1, 81.7), 64.4-64.0 (m), (61.85, 61.76), 28.4, (25.1, 25.0).

* Most signals were observed as two signals probably due to the rotamers. ^{1}H-^{13}C HSQC and HMBC experiments clearly showed the signals in parentheses are the same position.

† ^{1}H-^{13}C HSQC and HMBC experiments showed this signal corresponds to two ^{13}C environments.

^{19}F NMR (376 MHz, $CDCl_3$): δ -80.8 - -80.9 (m), -120.7 - -120.9 (m), -127.56 - -127.64 (m).

R_f = 0.25 (60% v/v diethyl ether/ CH_2Cl_2 (1:1) in hexane), 0.30 (10% v/v acetone in $CHCl_3$).

$[\alpha]_D^{25.0}$ = +31.5 (c. 0.79, $CHCl_3$).

HRMS (-ESI): *m/z* found $[M+Cl]^-$ 488.0178, $[C_{15}H_{14}ClF_7NO_5S]^-$ requires 488.0175, (δ = +0.6 ppm).

Chiral SFC Analysis: CHIRALPAK IC (CO_2:MeOH, 95:5, 1.25 mL min^{-1}, 40 °C), t_R = 5.9 (major), 7.8 (minor) minutes.

2,2,3,3,4,4,4-heptafluorobutyl ((2-oxo-4-phenyl-1,3-dioxolan-4-yl)methyl)sulfamate (**16a**)

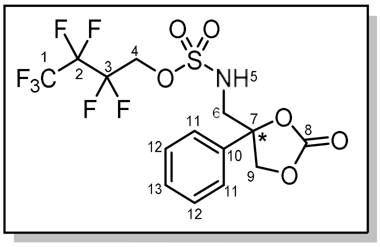

Prepared according to **GP 7** with catalyst Rh_2(**A1**)$_2$•(**B3**)$_2$•(Pyr)$_2$ (2.2 mg, 1 μmol, 1 mol%) and 2-phenylallyl methyl(phenyl)carbamate **15a** (26.5 mg, 0.0991 mmol, 1.0 equiv.). Purification by flash column chromatography (SiO_2, 0-60% v/v diethyl ether/CH_2Cl_2 (1:1) in hexane) afforded the title compound as a yellow oil (26.6 mg, 0.0584 mmol, 59% yield, 5% *ee*).

^{1}H NMR (700 MHz, $CDCl_3$): δ 7.49-7.46 (m, 2H, H-12), 7.45-7.42 (m, 1H, H-13), 7.34 (d, *J* = 8.4 Hz, 2H, H-11), 6.44 (br s, 1H, H-5), 4.99 (dd, *J* = 8.8, 2.6 Hz, 1H, H-9a), 4.58 (d, *J* = 8.9 Hz, 1H, H-9b), 4.52 (q, *J* = 12.9 Hz, 1H, H-4a), 4.46 (q, *J* = 12.9 Hz, 1H, H-4b), 3.68 (d, *J* = 14.9 Hz, 1H, H-6a), 3.62 (d, *J* = 14.9 Hz, 1H, H-6b).

^{13}C NMR (176 MHz, $CDCl_3$): δ 154.6, 137.5, 129.7, 129.6, 124.2, 117.5 (qt, $J_{C\text{-}F}$ = 287.0, 33.3 Hz), 113.3 (tt, $J_{C\text{-}F}$ = 258.2, 31.0 Hz), 110.4-106.7 (m), 85.3, 72.7, 64.6 (t, $J_{C\text{-}F}$ = 27.2 Hz), 50.8.

^{19}F NMR (376 MHz, $CDCl_3$): δ -81.7 (t, J = 9.2 Hz), -121.6 - -121.7 (m), -128.27 - -128.33 (m).

R_f = 0.44 (60% v/v diethyl ether/ CH_2Cl_2 (1:1) in hexane).

HRMS (-ESI): *m/z* found $[M-H]^-$ 454.0193, $[C_{14}H_{11}F_7NO_6S]^-$ requires 454.0201, (δ = -1.8 ppm).

Chiral SFC Analysis: CHIRALPAK IJ (CO_2:MeOH, 97:3, 2.50 mL min^{-1}, 40 ˚C), t_R = 2.7 (major), 2.9 (minor) minutes.

2,2,3,3,4,4,4-heptafluorobutyl (2-(2-oxo-1,3-dioxolan-4-yl)propan-2-yl)sulfamate (**16b**)

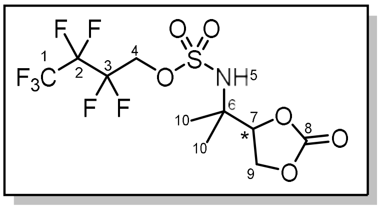

Prepared according to **GP 7** with catalyst Rh_2(**A1**)$_2$•(**B3**)$_2$•(Pyr)$_2$ (2.2 mg, 1 µmol, 1 mol%) and 3-methylbut-2-en-1-yl methyl(phenyl)carbamate **15b** (23.2 mg, 0.106 mmol, 1.0 equiv.). Purification by flash column chromatography (SiO_2, 0-62% v/v diethyl ether/CH_2Cl_2 (1:1) in hexane) afforded the title compound as white flakes (1.7 mg, 4.17 µmol, 4% yield, 27% *ee*).

^{1}H NMR (500 MHz, $CDCl_3$): δ 5.36 (d, J = 5.3 Hz, 1H, H-5), 4.58 (t, J = 13.1 Hz, 2H, H-4), 4.48 (dd, J = 9.8, 3.0 Hz, 1H, H-7), 3.51-3.46 (m, 1H, H-9a), 3.39-3.34 (m, 1H, H-9b), 1.57 (s, 3H, H-10a)*, 1.44 (s, 3 H, H-10b).

*Partially obscured by water peak.

^{13}C NMR (126 MHz, $CDCl_3$): δ 152.9, 83.1, 82.5, 64.6 (t, $J_{C\text{-}F}$ = 26.4 Hz), 43.7, 26.9, 21.1.

^{19}F NMR (376 MHz, $CDCl_3$): δ -81.7 (t, J = 9.3 Hz), -121.45 - -121.54 (m), -128.2 - -128.3 (m).

R_f = 0.24 (60% v/v diethyl ether/ CH_2Cl_2 (1:1) in hexane).

HRMS (-ESI): *m/z* found $[M-H]^-$ 406.0192, $[C_{10}H_{11}F_7NO_6S]^-$ requires 406.0201, (δ = -2.2 ppm).

Chiral GC Analysis: CP-Chirasil-Dex CB column (165 ˚C Isothermal, 1.44 mL min^{-1}), t_R = 45.5 (major), 46.8 (minor) minutes.

2,2,3,3,4,4,4-heptafluorobutyl (1-(2-oxo-1,3-dioxolan-4-yl)butyl)sulfamate (**16c**)

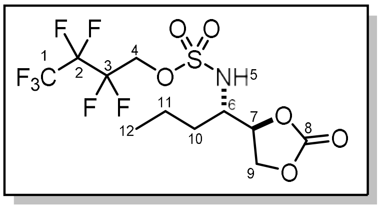

Prepared according to **GP 7** with catalyst Rh_2(**A1**)$_2$•(**B3**)$_2$•(Pyr)$_2$ (2.2 mg, 1 μmol, 1 mol%) and (*Z*)-hex-2-en-1-yl methyl(phenyl)carbamate **15c** (23.8 mg, 0.102 mmol, 1.0 equiv.). Purification by flash column chromatography (SiO_2, 0-60% v/v diethyl ether/CH_2Cl_2 (1:1) in hexane, *then* 0-5% v/v acetone in $CHCl_3$) afforded the title compound as a pale-yellow oil (12.4 mg, 0.0294 mmol, 29% yield, 28% *ee* – determined from *N*-benzylated adduct: see the next compound).

^{1}H NMR (500 MHz, $CDCl_3$): δ 5.74 (br s, 1H, H-5), 4.83-4.80 (m, 1H, H-7), 4.61-4.51 (m, 4H, H-4a, H-4b, H-9a, H-9b), 3.59 (t, J = 7.0 Hz, 1H, H-6), 1.80-1.68 (m, 2H, H-10), 1.52-1.44 (m, 2H, H-11), 0.98 (t, J = 7.3 Hz, 3H, H-12).

^{13}C NMR (126 MHz, $CDCl_3$): δ 155.0, 117.5 (qt, $J_{C\text{-}F}$ = 286.8, 33.6 Hz), 113.4 (tt, $J_{C\text{-}F}$ = 258.7, 31.4 Hz), 110.9-106.1 (m), 76.7, 66.7, 64.4 (t, $J_{C\text{-}F}$ = 27.6 Hz), 56.4, 34.1, 19.0, 13.7.

^{19}F NMR (376 MHz, $CDCl_3$): δ -81.7 (t, J = 9.2 Hz), -121.5 - -121.6 (m), -128.3 - -128.4 (m).

R_f = 0.24 (60% v/v diethyl ether/ CH_2Cl_2 (1:1) in hexane).

HRMS (-ESI): *m/z* found [M−H]$^-$ 420.0350, $[C_{11}H_{13}F_7NO_6S]^-$ requires 420.0357, (δ = -1.7 ppm).

2,2,3,3,4,4,4-heptafluorobutyl (4-bromobenzyl)(1-(2-oxo-1,3-dioxolan-4-yl)butyl)sulfamate

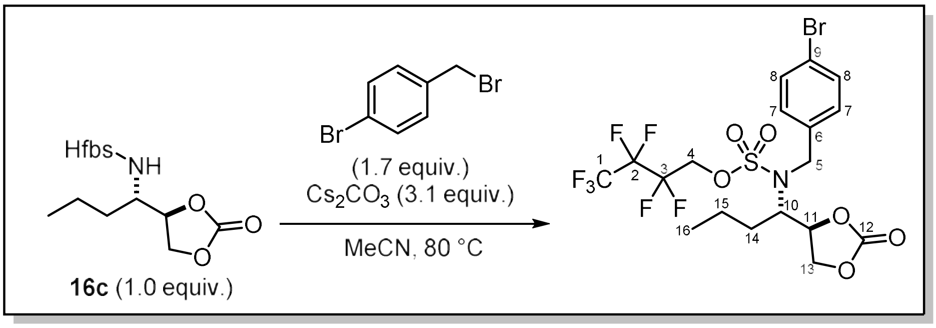

A microwave vial was charged with 2,2,3,3,4,4,4-heptafluorobutyl (1-(2-oxo-1,3-dioxolan-4-yl)butyl)sulfamate **16c** (12.4 mg, 0.0294 mmol, 1.0 equiv.), 4-bromobenzyl bromide (12.3 mg, 0.0492 mmol, 1.7 equiv.) and cesium carbonate (29.5 mg, 0.0905 mmol, 3.1 equiv.). The vial was evacuated and backfilled with nitrogen thrice before the addition of anhydrous MeCN (2 mL). The vial was stirred at 80 ˚C for 15 h and cooled to room temperature. The solvent was removed under a gentle stream of nitrogen and the crude residue was purified

by flash column chromatography (SiO_2, 0-20% v/v acetone in hexane) to give the title compound as a pale-yellow oil (7.4 mg, 0.0125 mmol, 43% yield, 28% *ee*).

^{1}H NMR (500 MHz, $CDCl_3$): δ 7.52 (d, J = 8.5 Hz, 2H, H-8), 7.27 (d, J = 8.6 Hz, 2H, H-7), 4.69 (q, J = 7.7 Hz, 1H, H-11), 4.59-4.52 (m, 3H, H-4a, H-4b, H-5a), 4.43-4.37 (m, 2H, H-5b, H-13a), 4.14 (dd, J = 9.0, 7.8 Hz, 1H, H-13b), 3.73-3.69 (m, 1H, H-10), 1.80-1.73 (m, 1H, H-14a), 1.50-1.43 (m, 1H, H-15a), 1.35-1.28 (m, 1H, H-14b), 1.25-1.19 (m, 1H, H-15b), 0.87 (t, J = 7.3 Hz, 3H, H-16).

^{13}C NMR (126 MHz, $CDCl_3$): δ 153.5, 133.9, 132.3, 130.6, 123.1, 117.5 (qt, $J_{C\text{-}F}$ = 287.3, 34.3 Hz), 113.3 (tt, $J_{C\text{-}F}$ = 255.9, 30.4 Hz), 109.1-106.0 (m), 76.8, 66.7, 64.3 (t, $J_{C\text{-}F}$ = 27.5 Hz), 61.7, 51.9, 30.0, 19.3, 13.7.

^{19}F NMR (376 MHz, $CDCl_3$): δ -81.7 (t, J = 9.2 Hz), -121.5 - -121.6 (m), -128.2 - -128.3 (m).

R_f = 0.26 (20% v/v acetone in hexane).

HRMS (+ESI): *m/z* found $[M+NH_4]^+$ 607.0342, $[C_{18}H_{23}BrF_7N_2O_6S]^+$ requires 607.0343, (δ = -0.2 ppm).

Chiral SFC Analysis: CHIRALPAK IC (CO_2:MeOH, 97:3, 2.50 mL min^{-1}, 40 ˚C), t_R = 5.1 (minor), 5.5 (major) minutes.

2,2,3,3,4,4,4-heptafluorobutyl ((2-oxo-1,3-dioxolan-4-yl)(phenyl)methyl)sulfamate (**S3**)

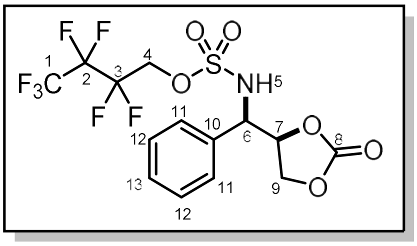

Prepared according to **GP 7** with $Rh_2(\mathbf{A1})_2{\bullet}(\mathbf{B3})_2{\bullet}(Pyr)_2$ (2.2 mg, 1 μmol, 1 mol%) and (*E*)-3-phenylallyl methyl(phenyl)carbamate **S2** (26.8 mg, 0.100 mmol, 1.0 equiv.). Purification by flash column chromatography (SiO_2, 0-14% v/v diethyl ether in CH_2Cl_2) afforded the title compound as a yellow oil (9.2 mg, 0.0202 mmol, 20% yield, 19% *ee*).

^{1}H NMR (700 MHz, CD_3CN): δ 7.47-7.41 (m, 5H, H-11, H-12, H-13), 7.08 (br s, 1H, H-5), 5.08 (dt, J = 8.5, 5.6 Hz, 1H, H-7), 4.76 (d, J = 5.6 Hz, 1H, H-6), 4.55 (dd, J = 9.2, 8.5 Hz, 1H, H-9a), 4.42 (q, J = 12.6 Hz, 1H, H-4a), 4.25 (dd, J = 9.2, 5.7 Hz, 1H, H-9b), 4.21 (q, J = 12.5 Hz, 1H, H-4b).

^{13}C NMR (176 MHz, CD_3CN): δ 155.3, 135.8, 130.1, 130.0, 128.9, 78.0, 67.6, 65.0 (t, $J_{C\text{-}F}$ = 27.0 Hz), 60.7.

^{19}F NMR (376 MHz, CD_3CN): δ -81.8 (t, J = 9.3 Hz), -121.2 - -121.3 (m), -128.0 - -128.1 (m).

HRMS (-ESI): *m/z* found $[M-H]^-$ 454.0194, $[C_{14}H_{11}F_7NO_6S]^-$ requires 454.0201, (δ = -1.5 ppm).

Chiral SFC Analysis: CHIRALPAK IC (CO_2:MeOH, 96:4, 2.50 mL min^{-1}, 40 ˚C), t_R = 3.7 (major), 10.4 (minor) minutes.

9 Isolation and Synthetic Elaboration of Aziridines

2,2,3,3,4,4,4-heptafluorobutyl (2S,3S)-2-(((methyl(phenyl)carbamoyl)oxy)methyl)-3-phenylaziridine-1-sulfonate (**3a**)

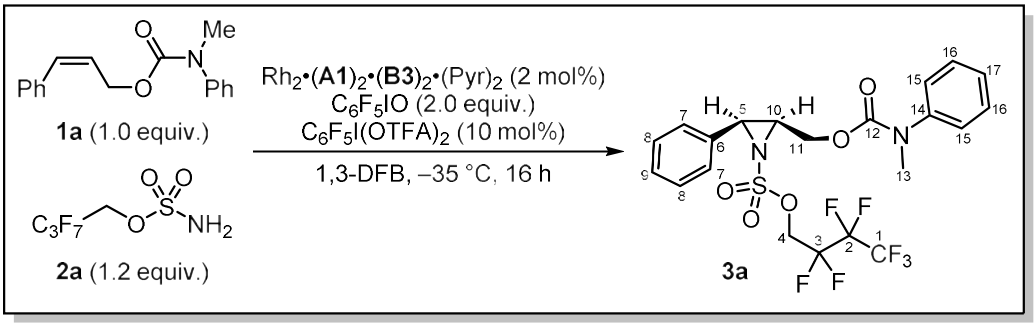

Under an atmosphere of air, four 4.0 mL crimp-top vials were each charged with the Rh_2(**A1**)$_2$•(**B3**)$_2$•(Pyr)$_2$ (2.2 mg, 1 μmol, 1 mol%). A stock solution of (*Z*)-3-phenylallyl methyl(phenyl)carbamate **1a** (110 mg, 0.411 mmol, 1.0 equiv.) and 2,2,3,3,4,4,4-heptafluorobutyl sulfamate **2a** (137 mg, 0.491 mmol, 1.2 equiv.) in 1,3-difluorobenzene (4.1 mL, 0.1 M) was prepared. The stock solution (1 mL) was added to each vial and the vials were cooled to -35 ˚C over 10 min. Pentafluoroiodosobenzene (C_6F_5IO) (62.0 mg, 0.2 mmol, 2.0 equiv.) and perfluorophenyl-λ^3-iodanediyl bis(2,2,2-trifluoroacetate) ($C_6F_5I(OTFA)_2$) (5.2 mg, 0.01 mmol, 10 mol%) were subsequently added together in a single portion to each vial at -35 ˚C. The vials were sealed and the reaction mixtures stirred at -35 ˚C overnight (~16 h). Saturated aqueous thiourea (1 mL) and $CHCl_3$ (1 mL) were added to each vial and the biphasic mixtures were stirred vigorously for 5 min at -35 ˚C. The mixtures were allowed to warm to room temperature and stirred for a further 15 min. The layers were separated and the organic layers were passed through a short pad of $MgSO_4$. The aqueous layers were extracted with $CHCl_3$ (6 x 1 mL) with each organic layer passed through a short pad of $MgSO_4$.The combined organic layers were concentrated under a gentle stream of nitrogen and the crude residue was purified by flash column chromatography (SiO_2, 0-12% v/v acetone in hexane) to afford the title compound as a white solid (209 mg, 0.384 mmol, 93% yield, 89% *ee*).

^{1}H NMR (700 MHz, CD_3CN): δ 7.39-7.35 (m, 7H, H-7, H-8, H-9, H-16), 7.25-7.23 (m, 3H, H-15, H-17), 4.99-4.95 (m, 2H, H-4a, H-4b), 4.23 (d, J = 7.4 Hz, 1H, H-5), 3.97 (dd, J = 12.2, 7.2 Hz, 1H, H-11a), 3.91 (br s, 1H, H-11b), 3.45 (br s, 1H, H-10), 3.21 (s, 3H, H-13).

^{13}C NMR (176 MHz, CD_3CN): δ 155.6, 144.2, 132.1, 129.8, 129.63, 129.60, 128.3, 127.2, 126.8, 118.5 (qt, $J_{C\text{-}F}$ = 286.3, 33.1 Hz), 114.4 (tt, $J_{C\text{-}F}$ = 257.7, 31.9 Hz), 111.3-107.7 (m), 67.2 (t, $J_{C\text{-}F}$ = 27.3 Hz), 62.1, 47.1, 46.7, 38.2.

^{19}F NMR (376 MHz, CD_3CN): δ -82.6 (t, J = 9.5 Hz), -122.0 - -122.1 (m), -128.65 - -128.73 (m).

R_f = 0.32 (20% v/v acetone in hexane).

$[\alpha]_D^{25.0}$ = +49.9 (c. 1.09, $CHCl_3$).

HRMS (+ESI): *m/z* found $[M+H]^+$ 545.0980, $[C_{21}H_{20}N_2F_7O_5S]^+$ requires 545.0976, (δ = +0.7 ppm).

Chiral SFC Analysis: CHIRALPAK IJ (CO_2:MeOH, 98:2, 1.25 mL min^{-1}, 40 ˚C), t_R = 7.3 (major), 8.1 (minor) minutes.

2,2,3,3,4,4,4-heptafluorobutyl 2-(4-chlorophenyl)-3-(((methyl(phenyl)carbamoyl)oxy)methyl)aziridine-1-sulfonate

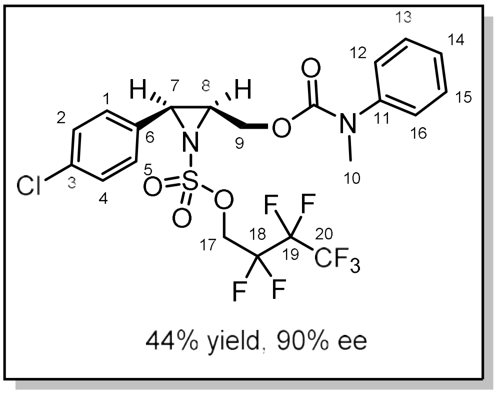

Prepared according to the procedure above with *(Z)*-3-(4-chlorophenyl)allyl methyl(phenyl)carbamate (30.2 mg, 0.1 mmol, 1.0 equiv.). Analysis of the crude ^{1}H-NMR showed the yield of aziridine to be 68% by comparison with internal standard. Purification by flash column chromatography (SiO_2, 0-15% v/v acetone in $CHCl_3$) afforded the title compound as a colourless oil (25.4 mg, 0.044 mmol, 44% yield, 90% *ee*).

^{1}H NMR (400 MHz, $CDCl_3$): δ 7.49 – 7.29 (m, 4H), 7.28 – 7.13 (m, 5H), 4.76 (t, J = 13.1 Hz, 2H), 4.08 – 3.98 (m, 2H), 3.91 (s, 1H), 3.40 (s, 1H), 3.27 (s, 3H).

R_f = 0.25 (20% v/v acetone in hexanes).

Chiral SFC Analysis: CHIRALPAK IJ (CO_2:MeOH, 98:2, 2.50 mL min^{-1}, 40 ˚C), t_R = 5.5 (minor), 7.4 (major) minutes.

Full characterisation could not be obtained due to decomposition of the sample.

2,2,3,3,4,4,4-heptafluorobutyl 2-(((methyl(phenyl)carbamoyl)oxy)methyl)-3-(4-(trifluoromethyl)phenyl)aziridine-1-sulfonate

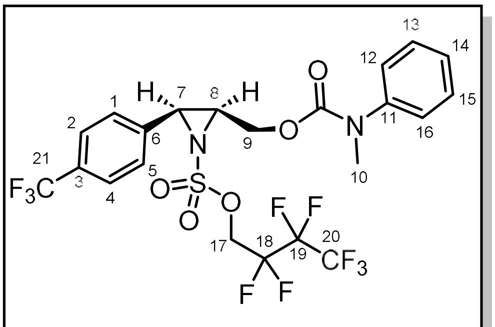

26% yield, 94% ee

Prepared according to the procedure above with *(Z)*-3-(4-(trifluoromethyl)phenyl)allyl methyl(phenyl)carbamate (33.5 mg, 0.1 mmol, 1.0 equiv.). Analysis of the crude ^{1}H-NMR showed the yield of aziridine to be 60% by comparison with internal standard. Purification by flash column chromatography (SiO_2, 0-15% v/v acetone in $CHCl_3$) afforded the title compound as a colourless oil (15.9 mg, 0.026 mmol, 26% yield, 94% *ee*).

^{1}H NMR (400 MHz, CD_2Cl_2): δ 7.64 (d, *J* = 7.9 Hz, 2H), 7.47 (d, *J* = 7.9 Hz, 2H), 7.37 (t, *J* = 7.6 Hz, 2H), 7.29 – 7.19 (m, 3H), 4.84 (t, *J* = 13.1 Hz, 2H), 4.17 (d, *J* = 7.5 Hz, 1H), 4.06 – 3.98 (m, 1H), 3.93 (s, 1H), 3.49 (s, 1H), 3.25 (s, 3H).

R_f = 0.50 (30% v/v acetone in hexanes).

Chiral SFC Analysis: CHIRALPAK IJ (CO_2:MeOH, 99:1, 2.50 mL min^{-1}, 40 ˚C), t_R = 3.5 (minor), 5.6 (major) minutes.

Full characterisation could not be obtained due to decomposition of the sample.

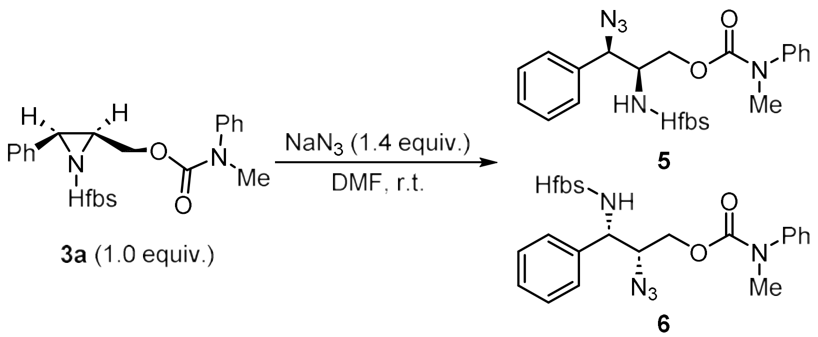

A 4.0 mL crimp-top vial was charged with aziridine **3a** (61.8 mg, 0.114 mmol, 1.0 equiv.) and NaN_3 (10.6 mg, 0.163 mmol, 1.4 equiv.). The vial was evacuated and back-filled thrice with nitrogen before the addition of DMF (1 mL, 0.11 M). The reaction mixture was allowed to stir at room temperature overnight and diluted with water. The aqueous phase was extracted with EtOAc and the combined organic layers were washed with 10% w/v aqueous LiCl and brine. The organic layers were dried over $MgSO_4$ and concentrated under reduced pressure. Purification by flash column chromatography (SiO_2, 0-5% v/v acetone in $CHCl_3$) afforded **5** (49.2 mg, 0.0837 mmol, 73% yield, 89% *ee*) as a colourless oil and **6** (12.4 mg, 0.0211 mmol, 19% yield, 89% *ee*) as a colourless oil.

2,2,3,3,4,4,4-heptafluorobutyl ((1R,2S)-1-azido-3-((methyl(phenyl)carbamoyl)oxy)-1-phenylpropan-2-yl)sulfamate (**5**)

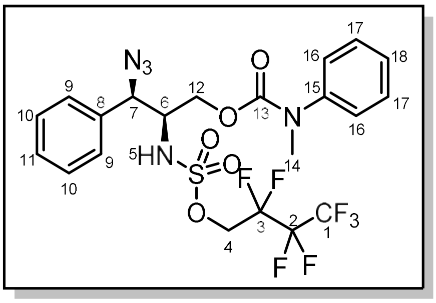

^{1}H NMR (700 MHz, CD_3CN): δ 7.44-7.40 (m, 5H, H-10, H-11, H-17), 7.34-7.21 (m, 5H, H-9, H-16, H-18), 6.51 (br s, 1H, H-5), 4.67-4.49 (m, 3H, H-4a, H-4b, H-7), 4.25 (dd, J = 11.8, 4.4 Hz, 1H, H-12a), 3.83 (br s, 1H, H-6), 3.71 (br s, 1H, H-12b), 3.28 (s, 3H, H-14).

^{13}C NMR (176 MHz, CD_3CN): δ 155.6, 144.4, 136.7, 130.1, 130.0, 128.7, 127.4, 127.1, 118.5 (qt, $J_{C\text{-}F}$ = 287.3, 33.0 Hz), 114.7 (tt, $J_{C\text{-}F}$ = 256.7, 30.6 Hz), 111.4-107.8 (m), 66.4, 65.1 (t, $J_{C\text{-}F}$ = 26.8 Hz), 65.0, 59.3, 38.2.

^{19}F NMR (376 MHz, CD_3CN): δ -82.6 (t, J = 9.5 Hz), -121.9 - -122.1 (m), -128.8 - -128.9 (m).

R_f = 0.65 (5% v/v acetone in $CHCl_3$).

$[\alpha]_D^{25.0}$ = -29.8 (c. 3.28, $CHCl_3$).

HRMS (-ESI): *m/z* found $[M\text{-}H]^-$ 586.0995, $[C_{21}H_{19}N_5F_7O_5S]^-$ requires 586.1001, (δ = -1.0 ppm).

Chiral SFC Analysis: CHIRALPAK IK (CO_2:MeOH, 96:4, 2.50 mL min^{-1}, 40 °C), t_R = 4.0 (minor), 4.7 (major) minutes.

2,2,3,3,4,4,4-heptafluorobutyl ((1S,2R)-2-azido-3-((methyl(phenyl)carbamoyl)oxy)-1-phenylpropyl)sulfamate (**6**)

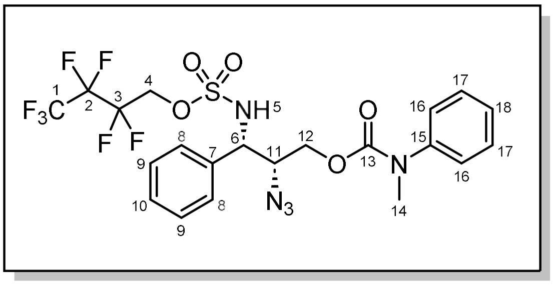

^{1}H NMR (700 MHz, CD_3CN): δ 7.43-7.35 (m, 5H, H-9, H-10, H-17), 7.32-7.21 (m, 3H, H-16, H-18), 7.20 (br s, 2H, H-8), 6.95 (br s, 1H, H-5), 4.41-4.36 (m, 2H, H-4a, H-6), 4.24 (dd, J = 11.8, 3.2 Hz, 1H, H-12a), 4.15 (q, J = 12.8 Hz, 1H, H-4b), 3.96 (br s, 1H, H-11), 3.72 (br s, 1H, H-12b), 3.25 (s, 3H, H-14).

^{13}C NMR (176 MHz, CD_3CN): δ 155.4,144.3, 138.6, 130.0, 129.9, 129.7, 128.2, 127.5, 127.2, 118.4 (qt, $J_{C\text{-}F}$ = 287.1, 33.3 Hz), 114.4 (tt, $J_{C\text{-}F}$ = 257.3, 31.5 Hz), 65.2, 65.1, 64.8 (t, $J_{C\text{-}F}$ = 27.0 Hz), 60.3, 38.3.

^{19}F NMR (376 MHz, CD_3CN): δ -82.7 (t, J = 9.4 Hz), -122.2 - -122.3 (m), -129.0 - -129.1 (m).

R_f = 0.50 (5% v/v acetone in $CHCl_3$).

$[\alpha]_D^{25.0}$ = -0.9 (c. 0.83, $CHCl_3$).

HRMS (-ESI): *m/z* found $[M-H]^-$ 586.0996, $[C_{21}H_{19}N_5F_7O_5S]^-$ requires 586.1001, (δ = -0.9 ppm).

Chiral SFC Analysis: CHIRALPAK IJ (CO_2:MeOH, 97:3, 2.50 mL min^{-1}, 40 ˚C), t_R = 5.5 (major), 6.1 (minor) minutes.

2,2,3,3,4,4,4-heptafluorobutyl (S)-(1-((methyl(phenyl)carbamoyl)oxy)-3-phenylpropan-2-yl)sulfamate (**7**)

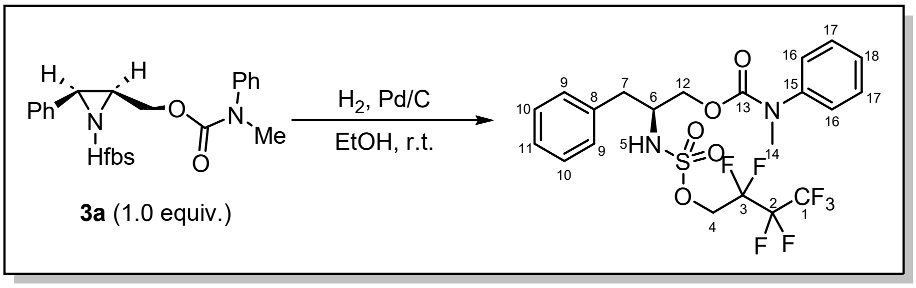

A microwave vial was charged with aziridine **3a** (38.1 mg, 0.0700 mmol, 1.0 equiv.), Pd/C (5.1 mg, 10 wt.% loading Pd) and EtOH (3 mL). The vial was evacuated and back-filled with hydrogen gas and the reaction mixture was allowed to stir at room temperature overnight. The reaction mixture was filtered over Celite® and eluted with EtOAc. The filtrate was concentrated under reduced pressure and the crude residue was purified via flash column chromatography (SiO_2, 0-16% v/v acetone in hexane) to afford the title compound as a white solid (34.8 mg, 0.0637 mmol, 91% yield, 89% *ee*).

^{1}H NMR (700 MHz, CD_3CN): δ 7.42-7.39 (m, 2H, H-17), 7.35-7.31 (m, 4H, H-10, H-16), 7.27-7.24 (m, 2H, H-11, H-18), 7.21 (m, 2H, H-9), 6.28 (br s, 1H, H-5), 4.30 (q, J = 13.3 Hz, 1H, H-4a), 4.21 (dd, J = 11.4, 3.8 Hz, 1H, H-12a), 4.04-3.98 (m, 2H, H-4b, H-12b), 3.83 (br s, 1H, H-6), 3.30 (s, 3H, H-14), 2.85 (br s, 1H, H-7a), 2.76 (br s, 1H, H-7b).

^{13}C NMR (176 MHz, CD_3CN): δ 155.9, 144.5, 138.3, 130.4, 129.9, 129.6, 127.9, 127.2, 126.9, 118.5 (qt, $J_{C\text{-}F}$ = 286.8, 33.0 Hz), 114.6 (tt, $J_{C\text{-}F}$ = 256.8, 30.9 Hz), 111.3-107.7 (m), 66.8, 64.6 (t, $J_{C\text{-}F}$ = 27.1 Hz), 57.0, 38.2, 38.1.

^{19}F NMR (376 MHz, CD_3CN): δ -82.7 (t, J = 9.5 Hz), -122.0 - -122.1 (m), -128.7 - -128.8 (m).

R_f = 0.32 (20% v/v acetone in hexane).

$[\alpha]_D^{25.0}$ = +1.4 (c. 2.32, $CHCl_3$).

HRMS (-ESI): *m/z* found $[M+Cl]^-$ 581.0740, $[C_{21}H_{21}N_2F_7O_5SCl]^-$ requires 581.0753, (δ = -2.2 ppm).

Chiral SFC Analysis: CHIRALPAK IC (CO_2:MeOH, 97:3, 2.50 mL min^{-1}, 40 ˚C), t_R = 4.7 (major), 8.2 (minor) minutes.

2,2,3,3,4,4,4-heptafluorobutyl ((1S,2R)-3-chloro-2-((methyl(phenyl)carbamoyl)oxy)-1-phenylpropyl)sulfamate (**8**)

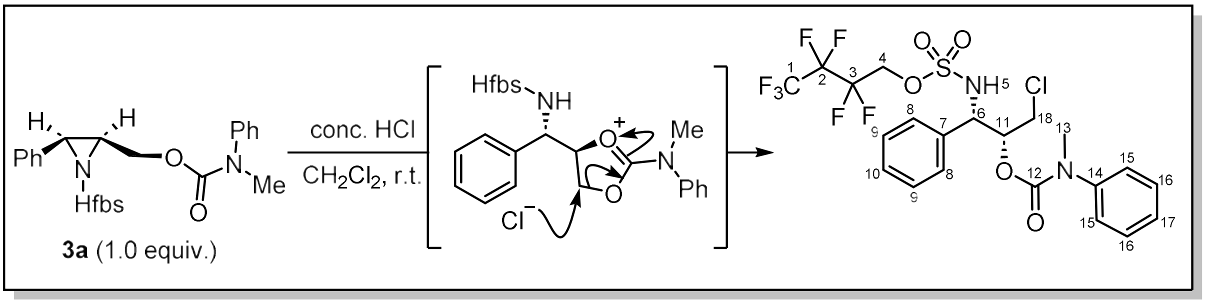

To a solution of aziridine **3a** (61.9 mg, 0.114 mmol, 1.0 equiv.) in CH_2Cl_2 (1.5 mL, 0.08 M) was added concentrated HCl (0.1 mL). The reaction mixture was allowed to stir at room temperature overnight and diluted with water (2 mL). The layers were separated and the organic layer was passed through a short pad of $MgSO_4$. The aqueous layer was extracted with CH_2Cl_2 (4 x 1 mL) with each organic layer passed through a short pad of $MgSO_4$.The combined organic layers were concentrated under reduced pressure and the crude residue was purified by flash column chromatography (SiO_2, 0-2% v/v acetone in $CHCl_3$, *then* 0-30% v/v diethyl ether/CH_2Cl_2 (1:1) in hexane) to afford the title compound as a colourless oil (29.0 mg, 0.0499 mmol, 44% yield, 89% *ee*).

^{1}H NMR (500 MHz, CD_3CN): δ 7.43-7.38 (m, 7H, H-8, H-9, H-10, H-16), 7.30-7.25 (m, 3H, H-15, H-17), 6.88 (br s, 1H, H-5), 5.26-5.22 (m, 1H, H-11), 4.74 (br s, 1H, H-6), 4.29 (q, *J* = 12.9 Hz, 1H, H-4a), 4.05 (q, *J* = 12.2 Hz, 1H, H-4b), 3.73 (d, *J* = 10.7 Hz, 1H, H-18a), 3.42 (br s 1H, H-18b), 3.31 (s, 3H, H-13).

^{13}C NMR (176 MHz, CD_3CN): δ 155.4, 144.1, 137.9, 129.95, 129.90, 129.8, 128.4, 127.3, 126.9, 118.4 (qt, $J_{C\text{-}F}$ = 286.8, 33.5 Hz), 114.3 (tt, $J_{C\text{-}F}$ = 256.9, 31.1 Hz), 111.1-107.5 (m), 76.3, 64.7 (t, $J_{C\text{-}F}$ = 27.3 Hz), 60.7, 44.7, 38.4.

^{19}F NMR (471 MHz, CD_3CN): δ -81.8 (t, *J* = 9.4 Hz), -121.26 - -121.33 (m), -128.1 - -128.2 (m).

R_f = 0.55 (5% v/v acetone in $CHCl_3$), 0.59 (60% v/v Et_2O/CH_2Cl_2 (1:1) in hexane).

$[\alpha]_D^{25.0}$ = +13.3 (c. 1.41, $CHCl_3$).

HRMS (+ESI): *m/z* found $[M+H]^+$ 581.0742, $[C_{21}H_{21}ClN_2F_7O_5S]^+$ requires 581.0742, (δ = ±0.0 ppm).

Chiral SFC Analysis: CHIRALPAK IJ (CO_2:MeOH, 97:3, 2.50 mL min^{-1}, 40 ˚C), t_R = 4.2 (major), 9.4 (minor) minutes.

10 Investigation of Impact of Chiral Cation Structure on Selectivity

All reactions shown in Scheme 3A and 3C in the manuscript were carried out according to **GP 7** using the allylic carbamate substrate **1a**.

Table S6: Summary of yields and ee values for **4a** using various chiral cations (to accompany Scheme 3A.)

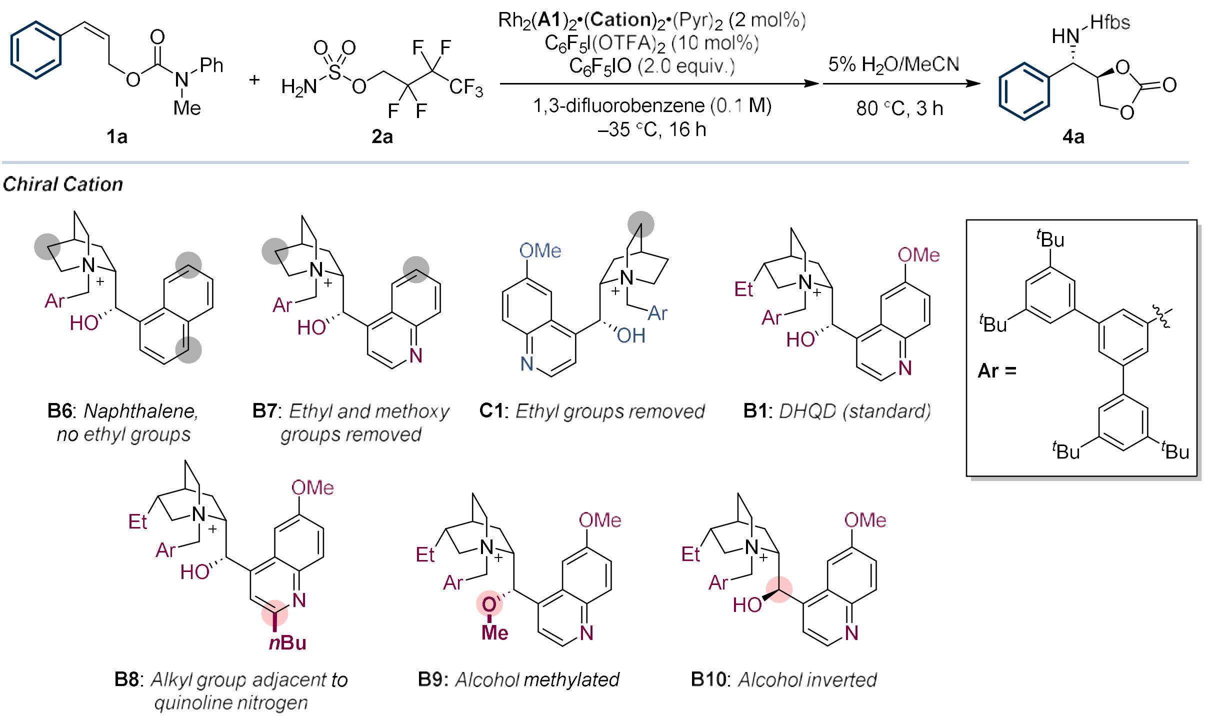

Entry	Chiral Cation	Yield **4a**/ %	*ee* **4a**/ %[a]
1	**B6**	55	23
2	**B7**	68	67
3	**C1**	67	-88
4	**B1**	65	91
5	**B8**	65	89
6	**B9**	69	95
7	**B10**	67	-39

Reactions performed on 0.10 mmol scale with respect to allylic carbamate **1a**. Yields and *ee* values correspond to isolated values. [a] *ee* determined by chiral SFC analysis.

11 Additional Optimization Data of C-H Amination

Table S7: Effect of sulfamate ester **2** and temperature on the C-H amination of phenethyl carbamate **S4**

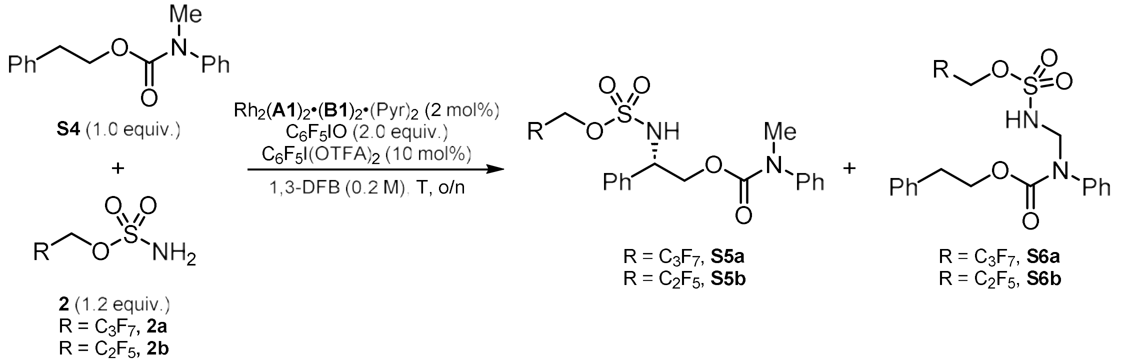

Entry	**2**	Product	Byproduct	T/ ˚C	Product / %[a]	*ee* / %[b]	Byproduct/ %[a]
1	**2a**	**S5a**	**S6a**	-25	45	93	10
2	**2b**	**S5b**	**S6b**	-25	54	93	7
3	**2b**	**S5b**	**S6b**	-35	63	93	10

Reactions performed on 0.10 mmol scale with respect to *N*-Me carbamate substrate **S4**. [a] Yields determined by ^{1}H NMR with reference to 1,3,5-trimethoxybenzene as the internal standard. [b] *ee* determined by chiral SFC analysis.

Table S8: Optimization of reaction conditions for the enantioselective C-H amination of phenethyl carbamates.

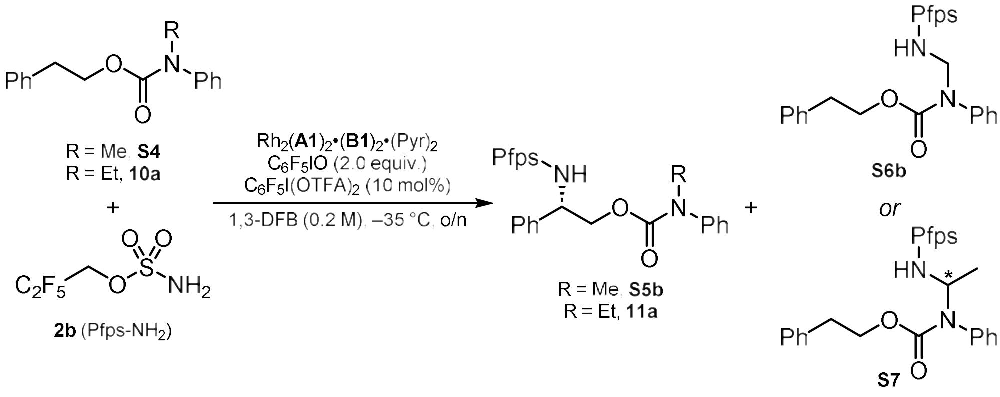

Entry	Substrate	Product	Rh$_2$(**A1**)$_2$•(**B1**)$_2$•(Pyr)$_2$ /mol%	**2b**/ equiv.	Product / %[a]	*ee* / %[b]	Remarks
1	**S4**	**S5b**	1	1.2	63	93	**S6b** detected in 10%
2	**S4**	**S5b**	2	1.2	67	95	**S6b** detected in 16%
3	**S4**	**S5b**	2	1.5	76	95	**S6b** detected in 7%
4	**10a**	**11a**	2	1.5	85	96	**S7** detected in 15%, 38% *ee*

Reactions performed on 0.10 mmol scale with respect to carbamate substrate. [a] Yields determined by ^{1}H NMR with reference to 1,3,5-trimethoxybenzene as the internal standard. [b] *ee* determined by chiral SFC analysis.

12 Unsuccessful C-H Amination Substrates

Attempted C-H amination on an aliphatic chain resulted only in *N*-Me amination.

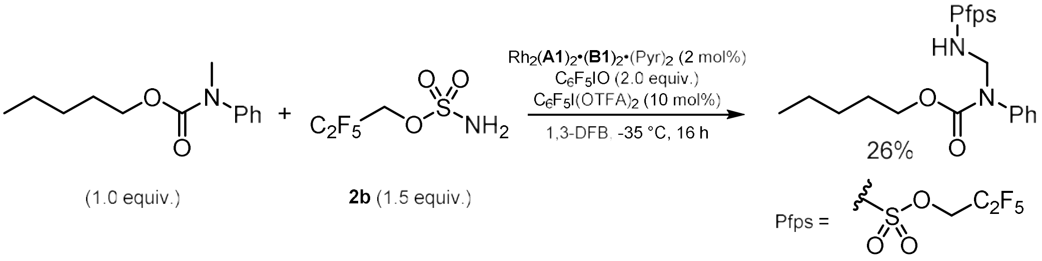

pentyl phenylcarbamate

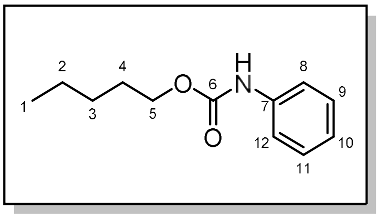

Prepared according to **GP 5** with pentan-1-ol (882 mg, 10.0 mmol, 1.0 equiv.). Purification by recrystallisation from hexanes afforded the title compound as white needles (1128 mg, 5.44 mmol, 54% yield).

^{1}H NMR (700 MHz, CDCl$_3$): δ 7.39 (d, *J* = 7.9 Hz, 2H, H-8,12), 7.33 – 7.27 (m, 2H, H-9,11), 7.06 (tt, *J* = 7.4, 1.2 Hz, 1H, H-10), 6.65 (s, 1H, NH), 4.16 (t, *J* = 6.7 Hz, 2H, H-5), 1.68 (tt, *J* = 9.7, 5.8 Hz, 2H, H-4), 1.41 – 1.32 (m, 4H, H-2,3), 0.93 – 0.91 (m, 3H, H-1).

^{13}C NMR (176 MHz, CDCl$_3$): δ 153.7, 138.0, 129.2, 129.0, 128.9, 123.3, 118.6, 77.2, 77.1, 76.9, 65.4, 28.7, 28.0, 22.4, 14.0.

HRMS: *This compound did not ionise.*

pentyl methyl(phenyl)carbamate

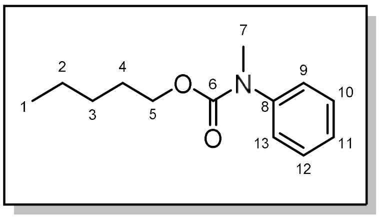

Prepared according to **GP 6B** with pentyl phenylcarbamate (622 mg, 3.0 mmol, 1.0 equiv.). Purification by flash column chromatography (SiO$_2$, 0-20% v/v EtOAc in hexanes) afforded the title compound as a colourless oil (476 mg, 2.15 mmol, 72% yield).

^{1}H NMR (700 MHz, CDCl$_3$): δ 7.37 – 7.31 (m, 2H, H-9,13), 7.24 (d, *J* = 7.8 Hz, 2H, H-10,12), 7.22 – 7.18 (m, 1H, H-11), 4.10 (t, *J* = 6.7 Hz, 2H, H5), 3.30 (s, 3H, H-7), 1.63 – 1.55 (m, 2H, H-4), 1.34 – 1.20 (m, 4H, H-2,3), 0.87 (t, *J* = 6.9 Hz, 3H, H-1).

^{13}C NMR (176 MHz, CDCl$_3$): δ 155.8, 143.4, 128.8, 125.9, 125.7, 77.2, 77.1, 76.9, 65.9, 37.6, 28.6, 28.1, 22.3, 14.0.

R_f = 0.49 (30% v/v EtOAc in hexanes).

HRMS: *This compound did not ionise.*

2,2,3,3,3-pentafluoropropyl ((((pentyloxy)carbonyl)(phenyl)amino)methyl)sulfamate

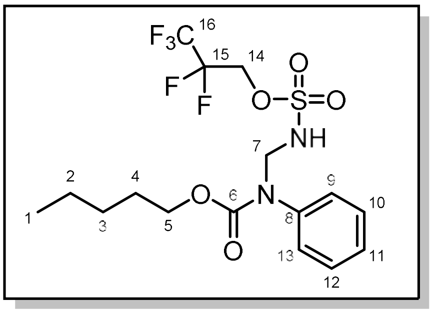

Prepared according to **GP10** with pentyl methyl(phenyl)carbamate (22.1 mg, 0.1 mmol, 1.0 equiv.). Purification by flash column chromatography (SiO_2, 0-15% v/v acetone in hexanes) afforded the title compound as a colourless oil (11.8 mg, 0.026 mmol, 26% yield).

^{1}H NMR (700 MHz, $CDCl_3$): δ 7.38 (t, *J* = 7.8 Hz, 2H, H-10,12), 7.32 – 7.28 (m, 1H, H-11), 7.23 (d, *J* = 7.8 Hz, 2H, H-9,13), 6.27 (s, 1H, NH), 4.97 (d, *J* = 6.5 Hz, 2H, H-7), 4.42 (t, *J* = 12.6 Hz, 2H, H-15), 4.11 (t, *J* = 6.7 Hz, 2H, H-5), 1.63 – 1.55 (m, 2H, H-4), 1.34 – 1.15 (m, 4H, H-2,3), 0.85 (t, *J* = 7.1 Hz, 3H, H-1).

^{13}C NMR (176 MHz, $CDCl_3$): δ 156.1, 140.4, 129.2, 127.6, 126.9, 118.2 (qt, J_{C-F} = 287, 33.6 Hz), 111.4 (tq, J_{C-F} = 258, 38.4 Hz), 66.8, 64.1 (t, J = 27.9 Hz), 60.1, 28.3, 27.9, 22.2, 13.9.

^{19}F NMR (376 MHz, $CDCl_3$): δ -84.51, -124.47.

R_f = 0.17 (20% v/v EtOAc in hexanes).

HRMS (-ESI): *m/z* found $[M-H]^-$ 447.1014, $[C_{16}H_{20}F_5N_2O_5S]^-$ requires 447.1019, (*δ* = -0.91 ppm).

Attempted C-H amination using a morpholine-derived carbamate resulted in no amination at the desired benzylic position and only amination on the morpholine was detected.

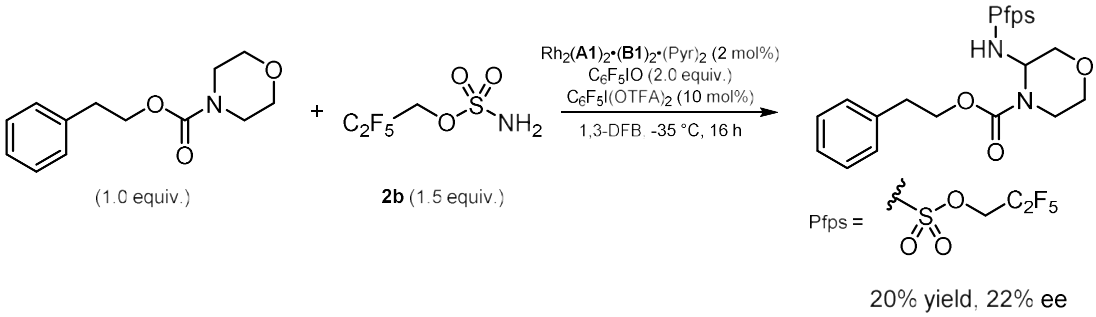

phenethyl morpholine-4-carboxylate

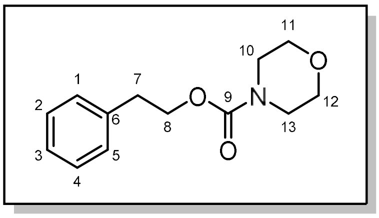

To a solution of *N,N'*-Carbonyldiimidazole (324 mg, 2.0 mmol, 1.0 equiv.) in anhydrous CH_2Cl_2 (5.0 mL) at 0°C , phenylethyl alcohol (244 mg, 2.0 mmol, 1.0 equiv.) was added as a solution in anhydrous CH_2Cl_2 (5.0 mL) and the resulting mixture was stirred at room temperature for 15 min. Morpholine (0.17 mL, 2.0 mmol, 1.0 equiv.) was added and the reaction was left to stir for 16 h, upon which the mixture was diluted with water and extracted with CH_2Cl_2. The combined organic layers were washed with aqueous HCl (1 M) and dried over $MgSO_4$. Purification by flash column chromatography (SiO_2, 0-20% v/v acetone in hexanes) afforded the title compound as a colourless oil (131 mg, 0.55 mmol, 28% yield).

^{1}H NMR (700 MHz, $CDCl_3$): δ 7.30 (t, *J* = 7.5 Hz, 2H, H-2,4), 7.25 – 7.18 (m, 3H, H-1,3,5), 4.32 (t, *J* = 7.0 Hz, 2H, H-7), 3.71 – 3.54 (m, 4H, H-10,13), 3.50 – 3.35 (m, 4H, H-11,12), 2.95 (t, *J* = 7.0 Hz, 2H, H-8).

^{13}C NMR (176 MHz, $CDCl_3$): δ 155.3, 138.0, 128.9, 128.5, 126.5, 77.2, 77.1, 76.9, 66.6, 66.0, 44.0 (d, *J* = 52.5 Hz), 35.6.

R_f = 0.31 (20% v/v acetone in hexanes).

HRMS: *This compound did not ionise.*

phenethyl 3-(((2,2,3,3,3-pentafluoropropoxy)sulfonyl)amino)morpholine-4-carboxylate

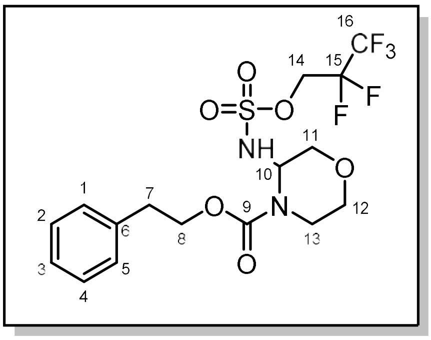

Prepared according to **GP10** with phenethyl morpholine-4-carboxylate (23.5 mg, 0.1 mmol, 1.0 equiv.). Purification by flash column chromatography (SiO_2, 0-5% v/v acetone in $CHCl_3$) afforded the title compound as a colourless oil (9.1 mg, 0.020 mmol, 20% yield, 22% *ee*).

^{1}H NMR (700 MHz, CD_3CN): δ 7.31 (t, J = 7.5 Hz, 2H, H-2,4), 7.28 – 7.20 (m, 3H, H-1,3,5), 7.00 (s, 1H, NH), 5.48 (s, 1H, H-10), 4.58 (t, J = 12.8 Hz, 2H, H-14), 4.26 (s, 2H, H-7), 3.91 – 3.77 (m, 2H, H-13a, H-11a), 3.69 (s, 1H, H-13b), 3.56 (d, J = 12.0 Hz, 1H, H-11b), 3.44 (s, 1H, H12-a), 3.32 – 3.15 (m, 1H, H12-b), 2.93 (t, J = 6.8 Hz, 2H, H-8).

^{13}C NMR (176 MHz, CD_3CN): δ 154.4, 138.4, 129.0, 128.4, 126.4, 118.3 (qt, $J_{C\text{-}F}$ = 287, 34.7 Hz), 111.9 (tq, $J_{C\text{-}F}$ = 226, 38.3 Hz), 69.6, 66.3 (d, J = 56.7 Hz), 64.0 (t, J = 28.0 Hz), 61.4 (d, J = 76.1 Hz), 39.0 (d, J = 133 Hz), 34.8.

^{19}F NMR (376 MHz, CD_3CN): δ -85.24, -125.05.

R_f = 0.21 (5% v/v acetone in $CHCl_3$).

HRMS (+ESI): *m/z* found $[M+H]^+$ 463.0962, $[C_{16}H_{20}F_5N_2O_6S]^+$ requires 463.0957, (*δ* = +1.07 ppm).

Chiral SFC Analysis: CHIRALPAK IJ (CO_2:MeOH, 98:2, 2.50 mL min^{-1}, 40 ˚C), t_R = 7.3 (major), 8.1 (minor) minutes.

13 Synthesis and Characterisation of Substrates (C-H Amination)

General Procedure 8 (GP 8):

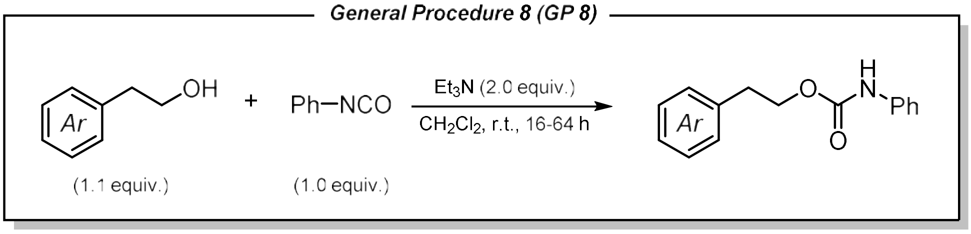

To a solution of an alcohol substrate (2.20 mmol, 1.1 equiv.) and Et_3N (0.56 mL, 4.00 mmol, 2.0 equiv.) in dry CH_2Cl_2 (15 mL), phenyl isocyanate (0.22 mL, 2.02 mmol, 1.0 equiv.) was added dropwise at room temperature under an inert atmosphere of nitrogen. The reaction mixture was stirred at room temperature overnight (>16 h). After that, all the volatiles were removed under reduced pressure. Purification by flash column chromatography afforded the N-H carbamate product.

phenethyl phenylcarbamate

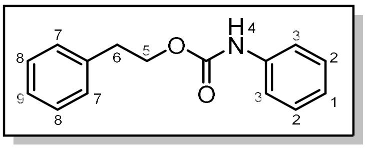

Prepared according to **GP 8** with 2-phenylethan-1-ol (0.263 mL, 2.20 mmol, 1.1 equiv). Purification by flash column chromatography (SiO_2, 20% v/v EtOAc in hexane) afforded the title compound as a white solid (467 mg, 1.94 mmol, 96% yield).

^{1}H NMR (700 MHz, $CDCl_3$): δ 7.36-7.29 (m, 6 H, H-2, H-3, H-8), 7.26-7.23 (m, 3 H, H-7, H-9), 7.08-7.05 (m, 1 H, H-1), 6.58 (br s, 1 H, H-4), 4.40 (t, J = 7.0 Hz, 2 H, H-5), 3.00 (t, J = 7.0 Hz, 2 H, H-6).

^{13}C NMR (176 MHz, $CDCl_3$): δ 153.5, 137.9*, 129.2, 129.0, 128.7, 126.7, 123.6, 118.8, 65.7, 35.5.

* ^{1}H-^{13}C HSQC and HMBC experiments showed this signal corresponds to two ^{13}C environments.

R_f = 0.51 (20% v/v EtOAc in hexane).

HRMS (+ESI): *m/z* found $[M+Na]^+$ 264.1002, $[C_{15}H_{15}NO_2Na]^+$ requires 264.0995, (δ = +2.7 ppm).

The spectroscopic data is in agreement with that reported in the literature.[34]

4-methoxyphenethyl phenylcarbamate

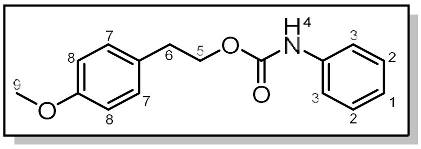

Prepared according to **GP 8** with 2-(4-methoxyphenyl)ethan-1-ol (304 mg, 2.20 mmol, 1.1 equiv.). Purification by flash column chromatography (SiO_2, 20% v/v EtOAc in hexane) afforded the title compound as a white solid (503 mg, 1.85 mmol, 92% yield).

^{1}H NMR (700 MHz, $CDCl_3$): δ 7.36-7.29 (m, 4 H, H-2, H-3), 7.18-7.16 (m, 2 H, H-7), 7.07-7.05 (m, 1 H, H-1), 6.87-6.85 (m, 2 H, H-8), 6.56 (br s, 1 H, H-4), 4.35 (t, J = 7.0 Hz, 2 H, H-5), 3.80 (s, 3 H, H-9), 2.94 (t, J = 7.0 Hz, 2 H, H-6).

^{13}C NMR (176 MHz, $CDCl_3$): δ 158.5, 153.6, 138.0, 130.0, 129.9, 129.2, 123.6, 118.8, 114.1, 66.0, 55.4, 34.7.

R_f = 0.43 (20% v/v EtOAc in hexane).

HRMS (+ESI): *m/z* found $[M+Na]^+$ 294.1090, $[C_{16}H_{17}NO_3Na]^+$ requires 294.1101, (δ = -3.7 ppm).

The spectroscopic data is in agreement with that reported in the literature.[35]

4-(tert-butyl)phenethyl phenylcarbamate

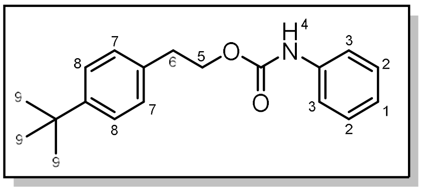

Prepared according to **GP 8** with 2-(4-(*tert*-butyl)phenyl)ethan-1-ol (329 mg, 2.20 mmol, 1.1 equiv.). Purification by flash column chromatography (SiO_2, 15% v/v EtOAc in hexane) afforded the title compound as a white solid (583 mg, 1.96 mmol, 97% yield).

^{1}H NMR (700 MHz, $CDCl_3$): δ 7.36-7.33 (m, 4 H, H-3, H-8), 7.31-7.29 (m, 2 H, H-2), 7.20-7.18 (m, 2 H, H-7), 7.07-7.05 (m, 1 H, H-1), 6.56 (br s, 1 H, H-4), 4.39 (t, J = 7.0 Hz, 2 H, H-5), 2.97 (t, J = 7.0 Hz, 2 H, H-6), 1.32 (s, 9 H, H-9).

^{13}C NMR (176 MHz, $CDCl_3$): δ 153.6, 149.6, 138.0, 134.8, 129.2, 128.7, 125.6, 123.6, 118.8, 65.8, 35.0, 34.6, 31.5.

R_f = 0.63 (20% v/v EtOAc in hexane).

HRMS (+ESI): *m/z* found $[M+Na]^+$ 320.1617, $[C_{19}H_{23}NO_2Na]^+$ requires 320.1621, (δ = -1.2 ppm).

4-methylphenethyl phenylcarbamate

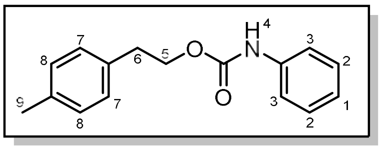

Prepared according to **GP 8** with 2-(*p*-tolyl)ethan-1-ol (0.306 mL, 2.20 mmol, 1.1 equiv.). Purification by flash column chromatography (SiO_2, 15% v/v EtOAc in hexane) afforded the title compound as a white solid (473 mg, 1.85 mmol, 92% yield).

^{1}H NMR (700 MHz, $CDCl_3$): δ 7.36-7.29 (m, 4 H, H-2, H-3), 7.15-7.13 (m, 4 H, H-7, H-8), 7.08-7.05 (m, 1 H, H-1), 6.58 (br s, 1 H, H-4), 4.38 (t, *J* = 7.1 Hz, 2 H, H-5), 2.96 (t, *J* = 7.1 Hz, 2 H, H-6), 2.34 (s, 3 H, H-9).

^{13}C NMR (176 MHz, $CDCl_3$): δ 153.6, 138.0, 136.3, 134.8, 129.4, 129.2, 128.9, 123.6, 118.7, 65.9, 35.1, 21.2.

R_f = 0.56 (20% v/v EtOAc in hexane).

HRMS (+ESI): *m/z* found $[M+Na]^+$ 278.1150, $[C_{16}H_{17}NO_2Na]^+$ requires 278.1151, (δ = -0.4 ppm).

4-bromophenethyl phenylcarbamate

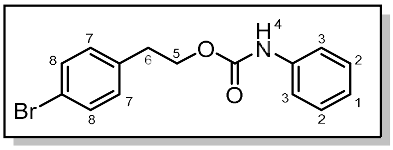

Prepared according to **GP 8** with 2-(4-bromophenyl)ethan-1-ol (0.308 mL, 2.20 mmol, 1.1 equiv.). Purification by flash column chromatography (SiO_2, 15% v/v EtOAc in hexane) afforded the title compound as a white solid (574 mg, 1.79 mmol, 89% yield).

^{1}H NMR (700 MHz, $CDCl_3$): δ 7.45-7.43 (m, 2 H, H-8), 7.35-7.29 (m, 4 H, H-2, H-3), 7.13-7.11 (m, 2 H, H-7), 7.08-7.06 (m, 1 H, H-1), 6.55 (br s, 1 H, H-4), 4.37 (t, *J* = 6.9 Hz, 2 H, H-5), 2.95 (t, *J* = 6.9 Hz, 2 H, H-6).

^{13}C NMR (176 MHz, $CDCl_3$): δ 153.4, 137.8, 136.9, 131.8, 130.8, 129.2, 123.7, 120.6, 118.8, 65.3, 35.0.

R_f = 0.29 (10% v/v EtOAc in hexane).

HRMS (+ESI): *m/z* found $[M+Na]^+$ 342.0090, $[C_{15}H_{14}BrNO_2Na]^+$ requires 342.0100, (δ = -2.9 ppm).

4-chlorophenethyl phenylcarbamate

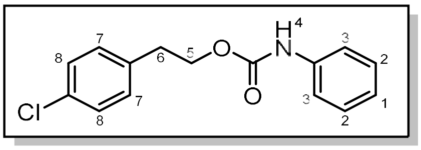

Prepared according to **GP 8** with 2-(4-chlorophenyl)ethan-1-ol (0.298 mL, 2.20 mmol, 1.1 equiv.). Purification by flash column chromatography (SiO_2, 15% v/v EtOAc in hexane) afforded the title compound as a white solid (443 mg, 1.61 mmol, 80% yield).

^{1}H NMR (700 MHz, $CDCl_3$): *δ* 7.35-7.28 (m, 6 H, H-2, H-3, H-8), 7.19-7.17 (m, 2 H, H-7), 7.08-7.06 (m, 1 H, H-1), 6.55 (br s, 1 H, H-4), 4.37 (t, *J* = 6.9 Hz, 2 H, H-5), 2.97 (t, *J* = 6.9 Hz, 2 H, H-6).

^{13}C NMR (176 MHz, $CDCl_3$): *δ* 153.4, 137.8, 136.4, 132.6, 130.4, 129.2, 128.8, 123.7, 118.8, 65.4, 34.9.

R_f = 0.44 (20% v/v EtOAc in hexane).

HRMS (+ESI): *m/z* found $[M+Na]^+$ 298.0598, $[C_{15}H_{14}ClNO_2Na]^+$ requires 298.0605, (*δ* = -2.3 ppm).

4-(trifluoromethyl)phenethyl phenylcarbamate

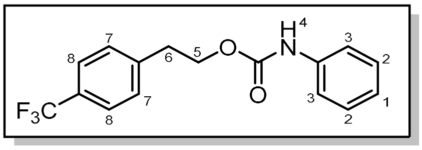

Prepared according to **GP 8** with 2-(4-(trifluoromethyl)phenyl)ethan-1-ol (0.340 mL, 2.24 mmol, 1.1 equiv.). Purification by flash column chromatography (SiO_2, 15% v/v EtOAc in hexane) afforded the title compound as a white solid (596 mg, 1.93 mmol, 96% yield).

^{1}H NMR (700 MHz, $CDCl_3$): *δ* 7.58 (d, *J* = 7.9 Hz, 2 H, H-8), 7.37-7.29 (m, 6 H, H-2, H-3, H-7), 7.07 (t, *J* = 7.4 Hz, 1 H, H-1), 6.55 (br s, 1 H, H-4), 4.42 (t, *J* = 6.7 Hz, 2 H, H-5), 3.06 (t, *J* = 6.7 Hz, 2 H, H-6).

^{13}C NMR (176 MHz, $CDCl_3$): *δ* 153.4, 142.1, 137.8, 129.4, 129.23, 129.17 (q, J_{C-F} = 32.3 Hz), 125.6 (q, J_{C-F} = 3.6 Hz), 124.4 (q, J_{C-F} = 272 Hz), 123.8, 118.8, 65.1, 35.4.

^{19}F NMR (376 MHz, $CDCl_3$): *δ* -62.4.

R_f = 0.42 (20% v/v EtOAc in hexane).

HRMS (–ESI): *m/z* found $[M-H]^-$ 308.0896, $[C_{16}H_{13}F_3NO_2]^-$ requires 308.0904, (*δ* = -2.6 ppm).

3-methoxyphenethyl phenylcarbamate

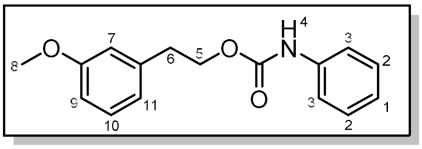

Prepared according to **GP 8** with 2-(3-methoxyphenyl)ethan-1-ol (0.307 mL, 2.20 mmol, 1.1 equiv.). Purification by flash column chromatography (SiO_2, 15% v/v EtOAc in hexane) afforded the title compound as a white solid (501 mg, 1.85 mmol, 92% yield).

^{1}H NMR (700 MHz, $CDCl_3$): *δ* 7.36-7.29 (m, 4 H, H-2, H-3), 7.25-7.23 (m, 1 H, H-10), 7.07-7.05 (m, 1 H, H-1), 6.84 (d, *J* = 7.7 Hz, 1 H, H-11), 6.80-6.79 (m, 2 H, H-7, H-9), 6.58 (br s, 1 H, H-4), 4.40 (t, *J* = 6.9 Hz, 2 H, H-5), 3.80 (s, 3 H, H-8), 2.98 (t, *J* = 6.9 Hz, 2 H, H-6).

^{13}C NMR (176 MHz, $CDCl_3$): *δ* 159.9, 153.5, 139.5, 137.9, 129.7, 129.2, 123.6, 121.4, 118.8, 114.9, 112.0, 65.7, 55.3, 35.6.

R_f = 0.46 (20% v/v EtOAc in hexane).

HRMS (+ESI): *m/z* found $[M+Na]^+$ 294.1105, $[C_{16}H_{17}NO_3Na]^+$ requires 294.1101, (*δ* = +1.4 ppm).

3-methylphenethyl phenylcarbamate

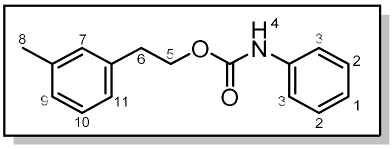

Prepared according to **GP 8** with 2-(*m*-tolyl)ethan-1-ol (0.299 mL, 2.20 mmol, 1.1 equiv.). Purification by flash column chromatography (SiO_2, 15% v/v EtOAc in hexane) afforded the title compound as a pale-yellow solid (456 mg, 1.78 mmol, 88% yield).

^{1}H NMR (700 MHz, $CDCl_3$): *δ* 7.37-7.29 (m, 4 H, H-2, H-3), 7.23-7.21 (m, 1 H, H-10), 7.08-7.05 (m, 4 H, H-1, H-7, H-9, H-11), 6.59 (br s, 1 H, H-4), 4.39 (t, *J* = 7.0 Hz, 2 H, H-5), 2.96 (t, *J* = 7.0 Hz, 2 H, H-6), 2.35 (s, 3 H, H-8).

^{13}C NMR (176 MHz, $CDCl_3$): *δ* 153.6, 138.3, 138.0, 137.8, 129.8, 129.2, 128.6, 127.5, 126.0, 123.6, 118.8, 65.8, 35.5, 21.5 ppm;

R_f = 0.53 (20% v/v EtOAc in hexane);

HRMS (+ESI): *m/z* found $[M+Na]^+$ 278.1149, $[C_{16}H_{17}NO_2Na]^+$ requires 278.1151, (*δ* = -0.7 ppm).

3-chlorophenethyl phenylcarbamate

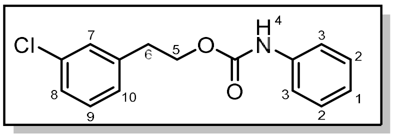

Prepared according to **GP 8** with 2-(3-chlorophenyl)ethan-1-ol (0.292 mL, 2.20 mmol, 1.1 equiv.). Purification by flash column chromatography (SiO_2, 20% v/v EtOAc in hexane) afforded the title compound as a white solid (537 mg, 1.95 mmol, 96% yield).

^{1}H NMR (700 MHz, $CDCl_3$): *δ* 7.36-7.29 (m, 4 H, H-2, H-3), 7.25-7.22 (m, 3 H, H-7, H-8, H-9), 7.14-7.12 (m, 1 H, H-10), 7.08-7.06 (m, 1 H, H-1), 6.59 (br s, 1 H, H-4), 4.38 (t, *J* = 6.8 Hz, 2 H, H-5), 2.97 (t, *J* = 6.8 Hz, 2 H, H-6).

^{13}C NMR (176 MHz, $CDCl_3$): *δ* 153.4, 140.0, 137.8, 134.4, 129.9, 129.2*, 127.2, 127.0, 123.7, 118.8, 65.3, 35.2.

* ^{1}H-^{13}C HSQC and HMBC experiments showed this signal corresponds to two ^{13}C environments.

R_f = 0.40 (20% v/v EtOAc in hexane).

HRMS (+ESI): *m/z* found $[M+Na]^+$ 298.0609, $[C_{15}H_{14}ClNO_2Na]^+$ requires 298.0605, (*δ* = +1.3 ppm).

3-bromophenethyl phenylcarbamate

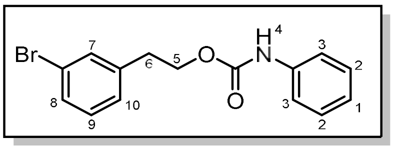

Prepared according to **GP 8** with 2-(3-bromophenyl)ethan-1-ol (0.299 mL, 2.20 mmol, 1.1 equiv.). Purification by flash column chromatography (SiO_2, 15% v/v EtOAc in hexane) afforded the title compound as a white solid (641 mg, 2.00 mmol, 99% yield).

^{1}H NMR (700 MHz, $CDCl_3$): *δ* 7.41-7.29 (m, 6 H, H-2, H-3, H-7, H-8), 7.20-7.17 (m, 2 H, H-9, H-10), 7.08-7.06 (m, 1 H, H-1), 6.60 (br s, 1 H, H-4), 4.38 (t, *J* = 6.8 Hz, 2 H, H-5), 2.96 (t, *J* = 6.8 Hz, 2 H, H-6).

^{13}C NMR (176 MHz, $CDCl_3$): *δ* 153.4, 140.3, 137.8, 132.1, 130.2, 129.9, 129.2, 127.7, 123.7, 122.7, 118.8, 65.3, 35.1.

R_f = 0.37 (20% v/v EtOAc in hexane).

HRMS (+ESI): *m/z* found $[M+Na]^+$ 342.0100, $[C_{15}H_{14}BrNO_2Na]^+$ requires 342.0100, (δ = ±0.0 ppm).

3-(trifluoromethyl)phenethyl phenylcarbamate

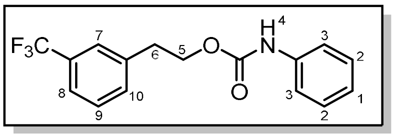

Prepared according to **GP 8** with 2-(3-(trifluoromethyl)phenyl)ethan-1-ol (0.332 mL, 2.20 mmol, 1.1 equiv.). Purification by flash column chromatography (SiO_2, 20% v/v EtOAc in hexane) afforded the title compound as a white solid (569 mg, 1.84 mmol, 91% yield).

^{1}H NMR (700 MHz, CDCl$_3$): δ 7.52-7.51 (m, 2 H, H-7, H-8), 7.45-7.43 (m, 2 H, H-9, H-10), 7.36-7.29 (m, 4 H, H-2, H-3), 7.08-7.06 (m, 1 H, H-1), 6.56 (br s, 1 H, H-4), 4.41 (t, J = 6.8 Hz, 2 H, H-5), 3.06 (t, J = 6.8 Hz, 2 H, H-4).

^{13}C NMR (176 MHz, CDCl$_3$): δ 153.4, 138.9, 137.8, 132.4, 131.0 (q, *J* = 32.2 Hz), 129.2, 129.1, 125.8 (q, *J* = 3.4 Hz), 124.3 (q, *J* = 272 Hz), 123.8, 123.7 (q, *J* = 3.7 Hz), 118.8, 65.2, 35.3.

^{19}F NMR (376 MHz, CDCl$_3$): δ -62.6.

R_f = 0.41 (20% v/v EtOAc in hexane).

HRMS (+ESI): *m/z* found $[M+K]^+$ 348.0600, $[C_{16}H_{14}F_3NO_2K]^+$ requires 348.0608, (δ = -2.3 ppm).

2-methylphenethyl phenylcarbamate

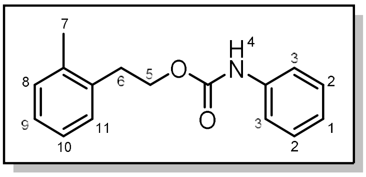

Prepared according to **GP 8** with 2-(o-tolyl)ethan-1-ol (0.295 mL, 2.20 mmol, 1.1 equiv.). Purification by flash column chromatography (SiO_2, 10% v/v EtOAc in hexane) afforded the title compound as a white solid (519 mg, 2.03 mmol, quant.).

^{1}H NMR (700 MHz, CDCl$_3$): δ 7.37-7.30 (m, 4 H, H-2, H-3), 7.20-7.15 (m, 4 H, H-8, H-9, H-10, H-11), 7.08-7.06 (m, 1 H, H-1), 6.58 (br s, 1 H, H-4), 4.36 (t, *J* = 7.3 Hz, 2 H, H-5), 3.01 (t, *J* = 7.3 Hz, 2 H, H-6), 2.37 (s, 3 H, H-7).

^{13}C NMR (176 MHz, CDCl$_3$): δ 153.6, 137.9, 136.7, 135.9, 130.5, 129.6, 129.2, 126.9, 126.2, 123.6, 118.8, 64.8, 32.9, 19.5.

R_f = 0.36 (10% v/v EtOAc in hexane).

HRMS (+ESI): *m/z* found $[M+Na]^+$ 278.1148, $[C_{16}H_{17}NO_2Na]^+$ requires 278.1151, (δ = -1.1 ppm).

2-chlorophenethyl phenylcarbamate

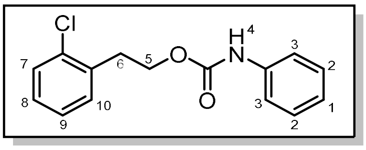

Prepared according to **GP 8** with 2-(2-chlorophenyl)ethan-1-ol (0.290 mL, 2.20 mmol, 1.1 equiv.). Purification by flash column chromatography (SiO_2, 15% v/v EtOAc in hexane) afforded the title compound as a white solid (484 mg, 1.76 mmol, 87% yield).

^{1}H NMR (700 MHz, $CDCl_3$): δ 7.38-7.28 (m, 6 H, H-2, H-3, H-7, H-10), 7.23-7.18 (m, 2 H, H-8, H-9), 7.08-7.05 (m, 1 H, H-1), 6.56 (br s, 1 H, H-4), 4.42 (t, *J* = 6.9 Hz, 2 H, H-5), 3.14 (t, *J* = 6.9 Hz, 2 H, H-6).

^{13}C NMR (176 MHz, $CDCl_3$): δ 153.5, 137.9, 135.6, 134.5, 131.2, 129.8, 129.2, 128.3, 127.0, 123.6, 118.8, 64.1, 33.3.

R_f = 0.50 (20% v/v EtOAc in hexane).

HRMS (+ESI): *m/z* found $[M+Na]^+$ 298.0598, $[C_{15}H_{14}ClNO_2Na]^+$ requires 298.0605, (δ = -2.3 ppm).

2-bromophenethyl phenylcarbamate

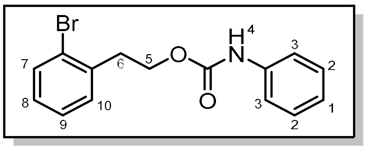

Prepared according to **GP 8** with 2-(2-bromophenyl)ethan-1-ol (0.298 mL, 2.20 mmol, 1.1 equiv.). Purification by flash column chromatography (SiO_2, 15% v/v EtOAc in hexane) afforded the title compound as a white solid (611 mg, 1.91 mmol, 95% yield).

^{1}H NMR (700 MHz, $CDCl_3$): δ 7.57 (dd, *J* = 7.9, 1.0 Hz, 1 H, H-7), 7.36-7.25 (m, 6 H, H-2, H-3, H-9, H-10), 7.13-7.10 (m, 1 H, H-8), 7.08-7.06 (m, 1 H, H-1), 6.57 (br s, 1 H, H-4), 4.42 (t, *J* = 6.9 Hz, 2 H, H-5), 3.15 (t, *J* = 6.9 Hz, 2 H, H-6).

^{13}C NMR (176 MHz, $CDCl_3$): δ 153.4, 137.9, 137.3, 133.1, 131.2, 129.2, 128.6, 127.7, 124.9, 123.7, 118.8, 64.2, 35.8.

R_f = 0.43 (20% v/v EtOAc in hexane).

HRMS (+ESI): *m/z* found $[M+Na]^+$ 342.0100, $[C_{15}H_{14}BrNO_2Na]^+$ requires 342.0100, (δ = ±0.0 ppm).

2-(thiophen-2-yl)ethyl phenylcarbamate

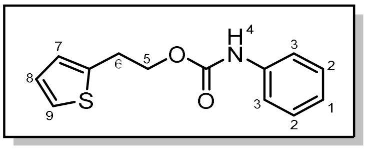

Prepared according to **GP 8** with 2-(thiophen-2-yl)ethan-1-ol (0.245 mL, 2.20 mmol, 1.1 equiv.). Purification by flash column chromatography (SiO_2, 15% v/v EtOAc in hexane) afforded the title compound as a white solid (488 mg, 1.97 mmol, 98% yield).

^{1}H NMR (700 MHz, CDCl$_3$): δ 7.37-7.30 (m, 4 H, H-2, H-3), 7.18 (dd, J = 5.1, 1.2 Hz, 1 H, H-9), 7.08-7.06 (m, 1 H, H-1), 6.96 (dd, J = 5.1, 3.5 Hz, 1 H, H-8), 6.90-6.89 (m, 1 H, H-7), 6.63 (br s, 1 H, H-4), 4.40 (t, J = 6.7 Hz, 2 H, H-5), 3.22 (td, J = 6.7, 0.5 Hz, 2 H, H-6).

^{13}C NMR (176 MHz, CDCl$_3$): δ 153.4, 140.1, 137.9, 129.2, 127.1, 125.7, 124.2, 123.7, 118.8, 65.5, 29.8.

R_f = 0.48 (20% v/v EtOAc in hexane);

HRMS (+ESI): *m/z* found $[M+Na]^+$ 270.0562, $[C_{13}H_{13}NO_2SNa]^+$ requires 270.0559, (δ = +1.1 ppm).

The spectroscopic data is in agreement with that reported in the literature.[36]

2-(thiophen-3-yl)ethyl phenylcarbamate

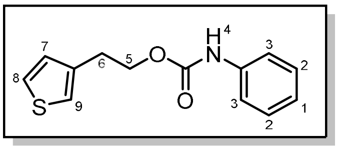

Prepared according to **GP 8** with 2-(thiophen-3-yl)ethan-1-ol (0.247 mL, 2.20 mmol, 1.1 equiv.). Purification by flash column chromatography (SiO_2, 20% v/v EtOAc in hexane) afforded the title compound as a white solid (457 mg, 1.85 mmol, 92% yield).

^{1}H NMR (700 MHz, CDCl$_3$): δ 7.37-7.28 (m, 5 H, H-2, H-3, H-8), 7.08-7.06 (m, 2 H, H-1, H-9), 7.00 (dd, J = 4.9, 1.1 Hz, 1 H, H-7), 6.59 (br s, 1 H, H-4), 4.40 (t, J = 6.9 Hz, 2 H, H-5), 3.03 (t, J = 6.9 Hz, 2 H, H-6).

^{13}C NMR (176 MHz, CDCl$_3$): δ 153.5, 138.1, 137.9, 129.2, 128.3, 125.9, 123.6, 121.7, 118.8, 65.1, 30.0.

R_f = 0.46 (20% v/v EtOAc in hexane).

HRMS (+ESI): *m/z* found $[M+Na]^+$ 270.0568, $[C_{13}H_{13}NO_2SNa]^+$ requires 270.0559, (δ = +3.3 ppm).

2,3-dihydro-1H-inden-2-yl phenylcarbamate

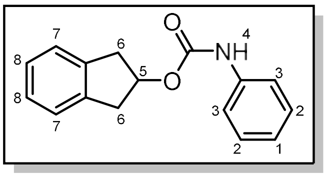

Prepared according to **GP 8** with indan-2-ol (1.10 g, 8.22 mmol, 1.1 equiv.). Purification by flash column chromatography (SiO_2, 10% v/v EtOAc in hexane) afforded the title compound as a white solid (1.62 g, 6.41 mmol, 85% yield).

^{1}H NMR (500 MHz, $CDCl_3$): *δ* 7.36-7.20 (m, 8 H, H-2, H-3, H-7, H-8, overlapped with the signal of residual chloroform), 7.07-7.04 (m, 1 H, H-1), 6.55 (br s, 1 H, H-4), 5.61-5.58 (m, 1 H, H-5), 3.35 (dd, *J* = 17.0, 6.1 Hz, 2 H, H-6a), 3.11 (dd, *J* = 17.0, 2.5 Hz, 2 H, H-6b).

^{13}C NMR (126 MHz, $CDCl_3$): *δ* 153.4, 140.6, 138.0, 129.2, 127.0, 124.9, 123.5, 118.6, 76.4, 39.9.

R_f = 0.57 (20% v/v EtOAc in hexane).

HRMS (+ESI): *m/z* found $[M+Na]^+$ 276.1008, $[C_{16}H_{15}NO_2Na]^+$ requires 276.0995, (*δ* = +4.7 ppm).

1-phenylpropan-2-yl phenylcarbamate

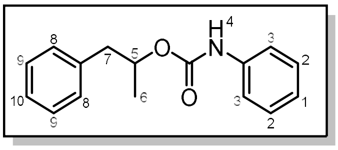

Prepared according to **GP 8** with 1-phenylpropan-2-ol (0.348 mL, 2.20 mmol, 1.1 equiv.). Purification by flash column chromatography (SiO_2, 20% v/v EtOAc in hexane) afforded the title compound as a white solid (422 mg, 1.65 mmol, 82% yield).

^{1}H NMR (700 MHz, $CDCl_3$): *δ* 7.36-7.29 (m, 6 H, H-2, H-3, H-9), 7.23-7.22 (m, 3 H, H-8, H-10), 7.07-7.04 (m, 1 H, H-1), 6.52 (br s, 1 H, H-4), 5.14 (sext, *J* = 6.3 Hz, 1 H, H-5), 3.01 (dd, *J* = 13.7, 6.3 Hz, 1 H, H-7a), 2.82 (dd, *J* = 13.7, 6.3 Hz, 1 H, H-7b), 1.28 (d, *J* = 6.3 Hz, 3 H, H-6).

^{13}C NMR (176 MHz, $CDCl_3$): *δ* 153.2, 138.1, 137.6, 129.6, 129.2, 128.5, 126.7, 123.5, 118.8, 72.6, 42.5, 19.8.

R_f = 0.55 (20% v/v EtOAc in hexane).

HRMS (+ESI): *m/z* found $[M+Na]^+$ 278.1156, $[C_{16}H_{17}NO_2Na]^+$ requires 278.1151, (*δ* = +1.8 ppm).

phenethyl methyl(phenyl)carbamate (**S4**)

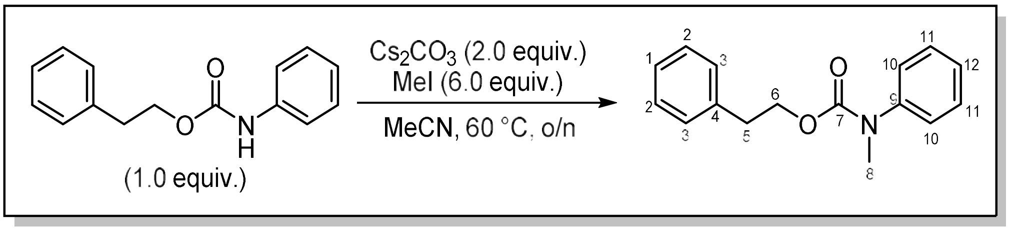

An oven-dried microwave vial was charged with phenethyl phenylcarbamate (506 mg, 2.1 mmol, 1.0 equiv.) and cesium carbonate (1.37 g, 4.2 mmol, 2.0 equiv.). The vial was evacuated and back-filled with nitrogen thrice before the addition of anhydrous MeCN (14 mL, 0.15 M) and methyl iodide (0.78 mL, 12.6 mmol, 6.0 equiv.). The reaction mixture was stirred at 60 ˚C overnight (~17 h) and allowed to cool to room temperature. The solvent was removed under reduced pressure and the crude reaction mixture was diluted with water and $CHCl_3$. The layers were separated and the aqueous layer was extracted with $CHCl_3$. The combined organic layers were dried over $MgSO_4$ and concentrated under reduced pressure. Purification of the crude residue by flash column chromatography (SiO_2, 0-10% v/v EtOAc in petroleum ether) afforded the title compound as a colourless oil (431 mg, 1.79 mmol, 85% yield).

^{1}H NMR (400 MHz, $CDCl_3$): 7.33 (t, *J* = 7.6 Hz, 2H, H-11), 7.28-7.20 (m, 4H, H-1, H-2, H-12), 7.17-7.13 (m, 4H, H-3, H-10), 4.31 (t, *J* = 6.8 Hz, 2H, H-6), 3.28 (s, 3H, H-8), 2.90 (t, *J* = 6.3 Hz, 2H, H-5).

^{13}C NMR (101 MHz, $CDCl_3$): 155.7, 143.3, 138.2, 129.2, 128.9, 128.5, 126.5, 126.2, 126.0, 66.4, 37.8, 35.5.

HRMS (+ESI): *m/z* found $[M+H]^+$ 256.1323, $[C_{16}H_{18}NO_2]^+$ requires 256.1332, (δ = -3.5 ppm).

General Procedure 9 (GP 9):

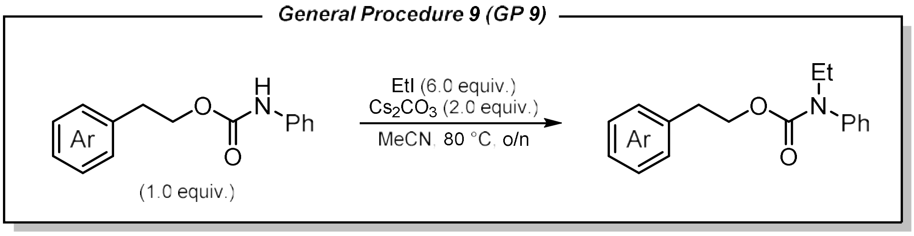

A crimp-top vial was charged with the N-H carbamate substrate (1.0 equiv.) and Cs_2CO_3 (2.0 equiv.). The vial was sealed and evacuated and backfilled with nitrogen three times, following which dry MeCN (0.125 M) and ethyl iodide (6.0 equiv.) were added. The reaction mixture was then heated at 80 °C overnight (~16 h). The reaction mixture was allowed to cool to room

temperature and water was added to the mixture. The volatiles were removed under reduced pressure and the solution was diluted with ethyl acetate and water. The aqueous layer was extracted thrice with ethyl acetate and the combined organic layers were washed with brine, dried over $MgSO_4$, filtered and concentrated under reduced pressure. Purification by flash column chromatography afforded the N-Et carbamate products.

phenethyl ethyl(phenyl)carbamate (**10a**)

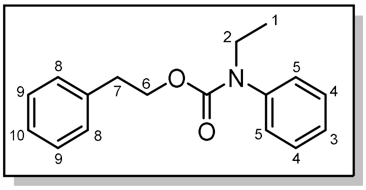

Prepared according to **GP 9** with phenethyl phenylcarbamate (493 mg, 2.04 mmol, 1.0 equiv.). Purification by flash column chromatography (SiO_2, 10% v/v EtOAc in hexane) afforded the title compound as a colourless oil (533 mg, 1.98 mmol, 97% yield).

^{1}H NMR (700 MHz, CD_3CN): δ 7.36-7.34 (m, 2 H, H-4), 7.27-7.25 (m, 3 H, H-3, H-9), 7.22-7.14 (m, 5 H, H-5, H-8, H-10), 4.22 (t, J = 6.4 Hz, 2 H, H-6), 3.63 (q, J = 7.1 Hz, 2 H, H-2), 2.86-2.84 (m, 2 H, H-7), 1.05 (t, J = 7.1 Hz, 3 H, H-1).

^{13}C NMR (176 MHz, CD_3CN): δ 155.8, 143.0, 139.7, 130.0, 129.8, 129.3, 128.5, 127.5, 127.3, 66.8, 46.0, 36.0, 13.9.

R_f = 0.49 (20% v/v EtOAc in hexane).

HRMS (+ESI): *m/z* found $[M+H]^+$ 270.1489, $[C_{17}H_{20}NO_2]^+$ requires 270.1489, (δ = ±0.0 ppm).

4-methoxyphenethyl ethyl(phenyl)carbamate (**10b**)

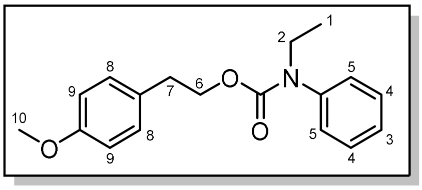

Prepared according to **GP 9** with 4-methoxyphenethyl phenylcarbamate (407 mg, 1.50 mmol, 1.0 equiv.). Purification by flash column chromatography (SiO_2, 15-20% v/v EtOAc in hexane) afforded the title compound as a pale-yellow oil (452 mg, 1.51 mmol, quant.).

^{1}H NMR (700 MHz, CD_3CN): δ 7.37-7.34 (m, 2 H, H-4), 7.28-7.25 (m, 1 H, H-3), 7.16-7.15 (br d, J = 7.6 Hz, 2 H, H-5), 7.04-7.03 (m, 2 H, H-8), 6.81-6.80 (br d, J = 8.5 Hz, 2 H, H-9), 4.17 (t, J = 6.4 Hz, 2 H, H-6), 3.75 (s, 3 H, H-10), 3.63 (q, J = 7.1 Hz, 2 H, H-2), 2.78-2.77 (m, 2 H, H-7), 1.05 (t, J = 7.1 Hz, 3 H, H-1).

^{13}C NMR (176 MHz, CD_3CN): δ 159.3, 155.9, 143.0, 131.5, 131.0, 129.9, 128.5, 127.5, 114.6, 67.0, 55.8, 46.0, 35.1, 13.9.

R_f = 0.43 (20% v/v EtOAc in hexane).

HRMS (+ESI): *m/z* found $[M+Na]^+$ 322.1408, $[C_{18}H_{21}NO_3Na]^+$ requires 322.1414, (δ = -1.9 ppm).

4-(tert-butyl)phenethyl ethyl(phenyl)carbamate (**10c**)

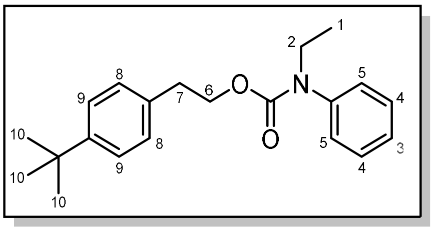

Prepared according to **GP 9** with 4-(*tert*-butyl)phenethyl phenylcarbamate (449 mg, 1.51 mmol, 1.0 equiv.). Purification by flash column chromatography (SiO_2, 15% v/v EtOAc in hexane) afforded the title compound as a pale-yellow oil (489 mg, 1.50 mmol, 99% yield).

^{1}H NMR (700 MHz, CD_3CN): δ 7.36-7.33 (m, 2 H, H-4), 7.31-7.30 (m, 2 H, H-9), 7.27-7.24 (m, 1 H, H-3), 7.13 (br d, J = 7.5 Hz, 2 H, H-5), 7.06 (br s, 2 H, H-8), 4.20 (t, J = 6.4 Hz, 2 H, H-6), 3.63 (q, J = 7.1 Hz, 2 H, H-2), 2.82-2.80 (m, 2 H, H-7), 1.29 (s, 9 H, H-10), 1.04 (t, J = 7.1, 3 H, H-1).

^{13}C NMR (176 MHz, CD_3CN): δ 155.8, 150.1, 143.0, 136.5, 129.8, 129.7, 128.5, 127.5, 126.2, 66.8, 46.0, 35.4, 35.0, 31.6, 13.9.

R_f = 0.49 (20% v/v EtOAc in hexane).

HRMS (+ESI): *m/z* found $[M+H]^+$ 326.2111, $[C_{21}H_{28}NO_2]^+$ requires 326.2115, (δ = -1.2 ppm).

4-methylphenethyl ethyl(phenyl)carbamate (**10d**)

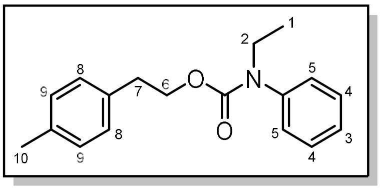

Prepared according to **GP 9** with 4-methylphenethyl phenylcarbamate (384 mg, 1.50 mmol, 1.0 equiv.). Purification by flash column chromatography (SiO_2, 15% v/v EtOAc in hexane) afforded the title compound as a pale-yellow oil (414 mg, 1.46 mmol, 97% yield).

^{1}H NMR (700 MHz, CD_3CN): δ 7.37-7.34 (m, 2 H, H-4), 7.27-7.25 (m, 1 H, H-3), 7.15 (br d, J = 7.6 Hz, 2 H, H-5), 7.08-7.02 (m, 4 H, H-8, H-9), 4.19 (t, J = 6.5 Hz, 2 H, H-6), 3.63 (q, J = 7.1 Hz, 2 H, H-2), 2.80 (br s, 2 H, H-7), 2.29 (s, 3 H, H-10), 1.05 (t, J = 7.1 Hz, 3 H, H-1).

^{13}C NMR (176 MHz, CD_3CN): δ 155.9, 143.0, 136.8, 136.5, 129.93, 129.89, 129.8, 128.5, 127.4, 66.9, 46.0, 35.6, 21.0, 13.9.

R_f = 0.50 (20% v/v EtOAc in hexane).

HRMS (+ESI): *m/z* found $[M+Na]^+$ 306.1460, $[C_{18}H_{21}NO_2Na]^+$ requires 306.1464, (δ = -1.3 ppm).

4-bromophenethyl ethyl(phenyl)carbamate (**10e**)

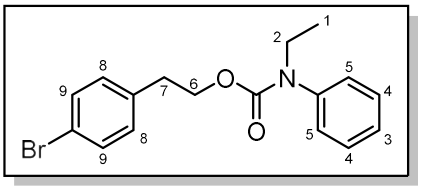

Prepared according to **GP 9** with 4-bromophenethyl phenylcarbamate (481 mg, 1.50 mmol, 1.0 equiv.). Purification by flash column chromatography (SiO_2, 15% v/v EtOAc in hexane) afforded the title compound as a white solid (516 mg, 1.48 mmol, 99% yield).

^{1}H NMR (700 MHz, CD_3CN): δ 7.40 (br d, *J* = 8.0 Hz, 2 H, H-8), 7.37-3.34 (m, 2 H, H-4), 7.28-7.25 (m, 1 H, H-3), 7.13 (br d, *J* = 7.6 Hz, 2 H, H-5), 7.04 (br s, 2 H, H-9), 4.20 (t, *J* = 6.1 Hz, 2 H, H-6), 3.61 (q, *J* = 7.1 Hz, 2 H, H-2), 2.81 (br s, 2 H, H-7), 1.04 (t, *J* = 7.1 Hz, 3 H, H-1).

^{13}C NMR (176 MHz, CD_3CN): δ 155.8, 142.9, 139.1, 132.2, 132.1, 129.9, 128.5, 127.5, 120.5, 66.4, 46.0, 35.3, 13.9.

R_f = 0.45 (20% v/v EtOAc in hexane).

HRMS (+ESI): *m/z* found $[M+H]^+$ 348.0597, $[C_{17}H_{19}BrNO_2]^+$ requires 348.0594, (δ = +0.9 ppm).

4-chlorophenethyl ethyl(phenyl)carbamate (**10f**)

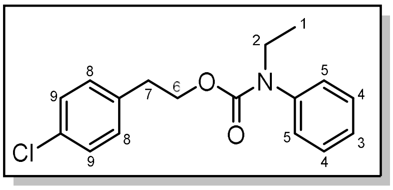

Prepared according to **GP 9** with 4-chlorophenethyl phenylcarbamate (338 mg, 1.23 mmol, 1.0 equiv.). Purification by flash column chromatography (SiO_2, 15% v/v EtOAc in hexane) afforded the title compound as a white solid (367 mg, 1.21 mmol, 98% yield).

^{1}H NMR (700 MHz, CD_3CN): δ 7.37-7.34 (m, 2 H, H-4), 7.28-7.25 (m, 3 H, H-3, H-9), 7.14-7.09 (m, 4 H, H-5, H-8), 4.20 (t, *J* = 6.1 Hz, 2 H, H-6), 3.62 (q, *J* = 7.1 Hz, 2 H, H-2), 2.82 (br s, 2 H, H-7), 1.04 (t, *J* = 7.1 Hz, 3 H, H-1).

^{13}C NMR (176 MHz, CD_3CN): δ 155.8, 142.9, 138.7, 132.5, 131.7, 129.9, 129.2, 128.5, 127.5, 66.4, 46.0, 35.2, 13.9.

R_f = 0.44 (20% v/v EtOAc in hexane).

HRMS (+ESI): *m/z* found $[M+H]^+$ 304.1104, $[C_{17}H_{19}ClNO_2]^+$ requires 304.1099, (δ = +1.6 ppm).

4-(trifluoromethyl)phenethyl ethyl(phenyl)carbamate (**10g**)

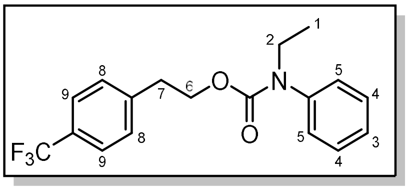

Prepared according to **GP 9** with 4-(trifluoromethyl)phenethyl phenylcarbamate (465 mg, 1.50 mmol, 1.0 equiv.). Purification by flash column chromatography (SiO_2, 15% v/v EtOAc in hexane) afforded the title compound as a white solid (491 mg, 1.46 mmol, 97% yield).

^{1}H NMR (700 MHz, CD_3CN): *δ* 7.56 (br d, *J* = 7.6 Hz, 2 H, H-9), 7.35-7.25 (m, 5 H, H-3, H-4, H-8), 7.11 (br d, *J* = 7.4 Hz, 2 H, H-5), 4.26 (t, *J* = 6.0 Hz, 2 H, H-6), 3.61 (q, *J* = 7.1 Hz, 2 H, H-2), 2.93 (br s, 2 H, H-7), 1.03 (t, *J* = 7.1 Hz, 3 H, H-1).

^{13}C NMR (176 MHz, CD_3CN): *δ* 155.7, 144.6, 142.9, 130.7, 129.9, 128.9 (q, $J_{C\text{-}F}$ = 32.3 Hz), 128.5, 127.5, 126.0 (q, $J_{C\text{-}F}$ = 3.6 Hz), 125.6 (q, $J_{C\text{-}F}$ = 272 Hz), 66.2, 46.0, 35.7, 13.9.

^{19}F NMR (376 MHz, CD_3CN): *δ* -62.9.

R_f = 0.38 (20% v/v EtOAc in hexane).

HRMS (+ESI): *m/z* found $[M+H]^+$ 338.1362, $[C_{18}H_{19}F_3NO_2]^+$ requires 338.1362, (*δ* = ±0.0 ppm).

3-methoxyphenethyl ethyl(phenyl)carbamate (**10h**)

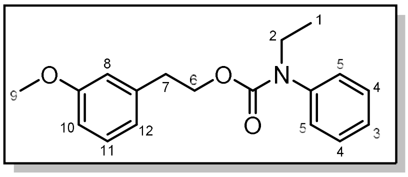

Prepared according to **GP 9** with 3-methoxyphenethyl phenylcarbamate (340 mg, 1.25 mmol, 1.0 equiv.). Purification by flash column chromatography (SiO_2, 20% v/v EtOAc in hexane) afforded the title compound as a pale-yellow oil (370 mg, 1.24 mmol, 99% yield).

^{1}H NMR (700 MHz, CD_3CN): *δ* 7.35-7.32 (m, 2 H, H-4), 7.26-7.23 (m, 1 H, H-3), 7.19-7.13 (m, 3 H, H-5, H-11), 6.78-6.72 (m, 3 H, H-8, H-10, H-12), 4.23 (t, *J* = 6.5 Hz, 2 H, H-6), 3.75 (s, 3 H, H-9), 3.63 (q, *J* = 7.1 Hz, 2 H, H-2), 2.83-2.82 (m, 2 H, H-7), 1.05 (t, *J* = 7.1 Hz, 3 H, H-1).

^{13}C NMR (176 MHz, CD_3CN): *δ* 160.7, 155.8, 143.0, 141.3, 130.3, 129.8, 128.4, 127.4, 122.3, 115.6, 112.6, 66.7, 55.8, 46.0, 36.0, 13.9.

R_f = 0.36 (20% v/v EtOAc in hexane).

HRMS (+ESI): *m/z* found $[M+Na]^+$ 322.1422, $[C_{18}H_{21}NO_3Na]^+$ requires 322.1414, (*δ* = +2.5 ppm).

3-methylphenethyl ethyl(phenyl)carbamate (**10i**)

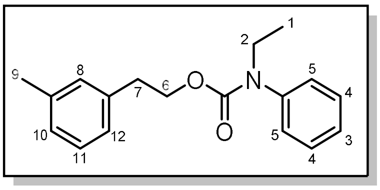

Prepared according to **GP 9** with 3-methylphenethyl phenylcarbamate (383 mg, 1.50 mmol, 1.0 equiv.). Purification by flash column chromatography (SiO_2, 15% v/v EtOAc in hexane) afforded the title compound as a pale-yellow oil (424 mg, 1.50 mmol, quant.).

^{1}H NMR (700 MHz, CD_3CN): *δ* 7.36-7.33 (m, 2 H, H-4), 7.27-7.24 (m, 1 H, H-3), 7.15-7.13 (m, 3 H, H-5, H-11), 7.03-7.02 (m, 1 H, H-10), 6.97-6.92 (m, 2 H, H-8, H-12), 4.21 (t, *J* = 6.5 Hz, 2 H, H-6), 3.63 (q, *J* = 7.1 Hz, 2 H, H-2), 2.81 (br s, 2 H, H-7), 2.28 (s, 3 H, H-9), 1.05 (t, *J* = 7.1 Hz, 3 H, H-1).

^{13}C NMR (176 MHz, CD_3CN): *δ* 155.8, 143.0, 139.6, 138.9, 130.7, 129.8, 129.2, 128.5, 127.9, 127.4, 127.0, 66.9, 46.0, 35.9, 21.4, 13.9.

R_f = 0.53 (20% v/v EtOAc in hexane).

HRMS (+ESI): *m/z* found $[M+H]^+$ 284.1643, $[C_{18}H_{22}NO_2]^+$ requires 284.1645, (*δ* = -0.7 ppm).

3-chlorophenethyl ethyl(phenyl)carbamate (**10j**)

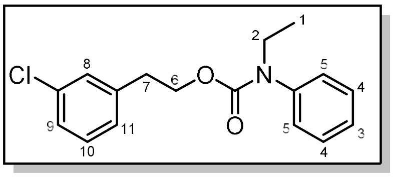

Prepared according to **GP 9** with 3-chlorophenethyl phenylcarbamate (333 mg, 1.21 mmol, 1.0 equiv.). Purification by flash column chromatography (SiO_2, 20% v/v EtOAc in hexane) afforded the title compound as a colourless oil (357 mg, 1.18 mmol, 98% yield).

^{1}H NMR (700 MHz, CD_3CN): *δ* 7.37-7.33 (m, 2 H, H-4), 7.27-7.22 (m, 3 H, H-3, H-9, H-10), 7.17-7.07 (m, 4 H, H-5, H-8, H-11), 4.23 (t, *J* = 6.1 Hz, 2 H, H-6), 3.62 (q, *J* = 7.1 Hz, 2 H, H-2), 2.84 (br s, 2 H, H-7), 1.04 (t, *J* = 7.1 Hz, 3 H, H-1).

^{13}C NMR (176 MHz, CD_3CN): *δ* 155.7, 142.9, 142.3, 134.4, 130.9, 130.0, 129.9, 128.6, 128.5, 127.5, 127.3, 66.3, 46.0, 35.6, 13.9.

R_f = 0.43 (20% v/v EtOAc in hexane).

HRMS (+ESI): *m/z* found $[M+H]^+$ 304.1103, $[C_{17}H_{19}ClNO_2]^+$ requires 304.1099, (*δ* = +1.3 ppm).

3-bromophenethyl ethyl(phenyl)carbamate (**10k**)

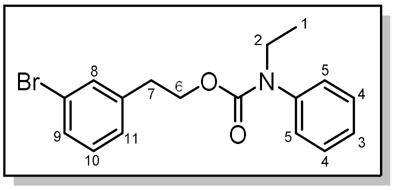

Prepared according to **GP 9** with 3-bromophenethyl phenylcarbamate (490 mg, 1.53 mmol, 1.0 equiv.). Purification by flash column chromatography (SiO_2, 20% v/v EtOAc in hexane) afforded the title compound as a pale-yellow oil (520 mg, 1.49 mmol, 97% yield).

^{1}H NMR (700 MHz, CD_3CN): δ 7.38-7.34 (m, 4 H, H-4, H-8, H-9), 7.27-7.25 (m, 1 H, H-3), 7.19-7.13 (m, 4 H, H-5, H-10, H-11), 4.22 (t, J = 6.1 Hz, 2 H, H-6), 3.62 (q, J = 7.1 Hz, 2 H, H-2), 2.84 (br s, 2 H, H-7), 1.04 (t, J = 7.1 Hz, 3 H, H-1).

^{13}C NMR (176 MHz, CD_3CN): δ 155.7, 142.9, 142.6, 132.9, 131.2, 130.3, 129.9, 129.0, 128.5, 127.5, 122.7, 66.3, 46.0, 35.6, 13.9.

R_f = 0.43 (20% v/v EtOAc in hexane).

HRMS (+ESI): *m/z* found $[M+H]^+$ 348.0605, $[C_{17}H_{19}BrNO_2]^+$ requires 348.0594, (δ = +3.2 ppm).

3-(trifluoromethyl)phenethyl ethyl(phenyl)carbamate (**10l**)

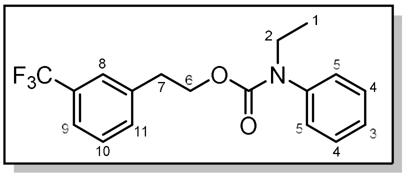

Prepared according to **GP 9** with 3-(trifluoromethyl)phenethyl phenylcarbamate (377 mg, 1.22 mmol, 1.0 equiv.). Purification by flash column chromatography (SiO_2, 20% v/v EtOAc in hexane) afforded the title compound as a pale-yellow oil (398 mg, 1.18 mmol, 97% yield).

^{1}H NMR (700 MHz, CD_3CN): δ 7.54-7.32 (m, 6 H, H-4, H-8, H-9, H-10, H-11), 7.26-7.24 (m, 1 H, H-3), 7.12 (br d, J = 7.4 Hz, 2 H, H-5), 4.27 (t, J = 6.1 Hz, 2 H, H-6), 3.61 (q, J = 7.1 Hz, 2 H, H-2), 2.94 (br s, 2 H, H-7), 1.02 (t, J = 7.1 Hz, 3 H, H-1).

^{13}C NMR (176 MHz, CD_3CN): δ 155.7, 142.9, 141.2, 134.0, 130.8 (q, J = 31.6 Hz), 130.1, 129.8, 128.4, 127.5, 126.6 (q, J = 3.5 Hz), 125.5 (q, J = 272 Hz), 124.1 (q, J = 3.8 Hz), 66.3, 46.0, 35.7, 13.8.

^{19}F NMR (376 MHz, CD_3CN): δ -63.0.

R_f = 0.43 (20% v/v EtOAc in hexane).

HRMS (+ESI): *m/z* found $[M+H]^+$ 338.1376, $[C_{18}H_{19}F_3NO_2]^+$ requires 338.1362, (δ = +4.1 ppm).

2-methylphenethyl ethyl(phenyl)carbamate (**10m**)

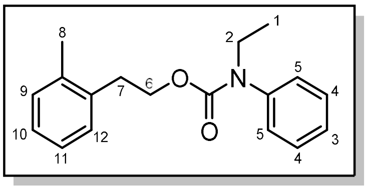

Prepared according to **GP 9** with 2-methylphenethyl phenylcarbamate (383 mg, 1.50 mmol, 1.0 equiv.). Purification by flash column chromatography (SiO_2, 15% v/v EtOAc in hexane) afforded the title compound as a pale-yellow oil (427 mg, 1.50 mmol, quant.).

^{1}H NMR (700 MHz, CD_3CN): *δ* 7.37-7.34 (m, 2 H, H-4), 7.27-7.25 (m, 1 H, H-3), 7.16-7.07 (m, 6 H, H-5, H-9, H-10, H-11, H-12), 4.21 (t, *J* = 6.6 Hz, 2 H, H-6), 3.63 (q, *J* = 7.1 Hz, 2 H, H-2), 2.89-2.87 (m, 2 H, H-7), 2.23 (br s, 3 H, H-8), 1.05 (t, *J* = 7.1 Hz, 3 H, H-1).

^{13}C NMR (176 MHz, CD_3CN): *δ* 155.9, 143.0, 137.53, 137.52, 131.1, 130.7, 129.9, 128.5, 127.5*, 126.8, 66.0, 46.0, 33.1, 19.4, 13.9.

* ^{1}H-^{13}C HSQC and HMBC experiments showed this signal corresponds to two ^{13}C environments.

R_f = 0.53 (20% v/v EtOAc in hexane).

HRMS (+ESI): *m/z* Found $[M+H]^+$ 284.1644, $[C_{18}H_{22}NO_2]^+$ requires 284.1645, (*δ* = -0.4 ppm).

2-chlorophenethyl ethyl(phenyl)carbamate (**10n**)

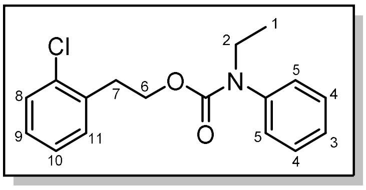

Prepared according to **GP 9** with 2-chlorophenethyl phenylcarbamate (339 mg, 1.23 mmol, 1.0 equiv.). Purification by flash column chromatography (SiO_2, 15% v/v EtOAc in hexane) afforded the title compound as a pale-yellow oil (372 mg, 1.22 mmol, 99% yield).

^{1}H NMR (700 MHz, CD_3CN): *δ* 7.38-7.33 (m, 3 H, H-4, H-8), 7.26-7.21 (m, 6 H, H-3, H-5, H-9, H-10, H-11), 4.27 (t, *J* = 6.1 Hz, 2 H, H-6), 3.62 (q, *J* = 7.1 Hz, 2 H, H-2), 3.02 (br s, 2 H, H-7), 1.04 (t, *J* = 7.1 Hz, 3 H, H-1).

^{13}C NMR (176 MHz, CD_3CN): *δ* 155.8, 142.9, 137.0, 134.7, 132.6, 130.3, 129.9, 129.2, 128.5, 128.0, 127.5, 65.0, 46.0, 33.7, 13.9.

R_f = 0.55 (20% v/v EtOAc in hexane).

HRMS (+ESI): *m/z* found $[M+H]^+$ 304.1108, $[C_{17}H_{19}ClNO_2]^+$ requires 304.1099, (*δ* = +3.0 ppm).

2-bromophenethyl ethyl(phenyl)carbamate (**10o**)

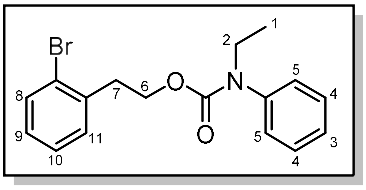

Prepared according to **GP 9** with 2-bromophenethyl phenylcarbamate (485 mg, 1.51 mmol, 1.0 equiv.). Purification by flash column chromatography (SiO_2, 20% v/v EtOAc in hexane) afforded the title compound as a pale-yellow oil (522 mg, 1.50 mmol, 99% yield).

^{1}H NMR (700 MHz, CD_3CN): *δ* 7.56 (d, *J* = 7.9 Hz, 1 H, H-8), 7.36-7.33 (m, 2 H, H-4), 7.27-7.24 (m, 2 H, H-3, H-10), 7.15-7.12 (m, 4 H, H-5, H-9, H-11), 4.26 (t, *J* = 6.0 Hz, 2 H, H-6), 3.62 (q, *J* = 7.1 Hz, 2 H, H-2), 3.02 (br s, 2 H, H-7), 1.04 (t, *J* = 7.1 Hz, 3 H, H-1).

^{13}C NMR (176 MHz, CD_3CN): *δ* 155.8, 142.9, 138.7, 133.6, 132.6, 129.9, 129.4, 128.6, 128.5, 127.5, 125.1, 65.1, 46.0, 36.1, 13.9.

R_f = 0.47 (20% v/v EtOAc in hexane).

HRMS (+ESI): *m/z* found $[M+H]^+$ 348.0604, $[C_{17}H_{19}BrNO_2]^+$ requires 348.0594, (*δ* = +2.9 ppm).

2-(thiophen-2-yl)ethyl ethyl(phenyl)carbamate (**10p**)

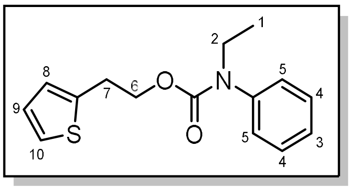

Prepared according to **GP 9** with 2-(thiophen-2-yl)ethyl phenylcarbamate (312 mg, 1.26 mmol, 1.0 equiv.). Purification by flash column chromatography (SiO_2, 20% v/v EtOAc in hexane) afforded the title compound as a pale-yellow oil (335 mg, 1.22 mmol, 97% yield).

^{1}H NMR (700 MHz, CD_3CN): *δ* 7.37-7.34 (m, 2 H, H-4), 7.27-7.24 (m, 1 H, H-3), 7.21-7.17 (m, 3 H, H-5, H-10), 6.93-6.92 (m, 1 H, H-9), 6.79 (br s, 1 H, H-8), 4.23 (t, *J* = 6.2 Hz, 2 H, H-6), 3.66 (q, *J* = 7.1 Hz, 2 H, H-2), 3.08 (br s, 2 H, H-7), 1.07 (t, *J* = 7.1 Hz, 3 H, H-1).

^{13}C NMR (176 MHz, CD_3CN): *δ* 155.8, 143.0, 141.6, 129.9, 128.5, 127.9, 127.5, 126.7, 125.0, 66.5, 46.1, 30.1, 13.9.

R_f = 0.48 (20% v/v EtOAc in hexane).

HRMS (+ESI): *m/z* found $[M+Na]^+$ 298.0883, $[C_{15}H_{17}NO_2SNa]^+$ requires 298.0872, (*δ* = +3.7 ppm).

2-(thiophen-3-yl)ethyl ethyl(phenyl)carbamate (**10q**)

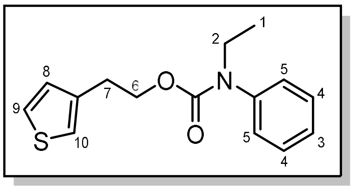

Prepared according to **GP 9** with 2-(thiophen-3-yl)ethyl phenylcarbamate (303 mg, 1.23 mmol, 1.0 equiv.). Purification by flash column chromatography (SiO_2, 15% v/v EtOAc in hexane) afforded the title compound as a pale-yellow oil (332 mg, 1.21 mmol, 98% yield).

^{1}H NMR (700 MHz, CD_3CN): *δ* 7.37-7.34 (m, 2 H, H-4), 7.30-7.25 (m, 2 H, H-3, H-9), 7.16 (br d, *J* = 7.6 Hz, 2 H, H-5), 7.00 (br s, 1 H, H-10), 6.89 (br s, 1 H, H-8), 4.22 (t, *J* = 6.4 Hz, 2 H, H-6), 3.64 (q, *J* = 7.1 Hz, 2 H, H-2), 2.89-2.87 (m, 2 H, H-7), 1.06 (t, *J* = 7.1 Hz, 3 H, H-1).

^{13}C NMR (176 MHz, CD_3CN): *δ* 155.9, 143.0, 139.8, 129.9, 129.6, 128.5, 127.5, 126.4, 122.6, 66.1, 46.0, 30.4, 13.9.

R_f = 0.45 (20% v/v EtOAc in hexane).

HRMS (+ESI): *m/z* found $[M+Na]^+$ 298.0885, $[C_{15}H_{17}NO_2SNa]^+$ requires 298.0872, (*δ* = +4.4 ppm).

2,3-dihydro-1H-inden-2-yl ethyl(phenyl)carbamate (**10r**)

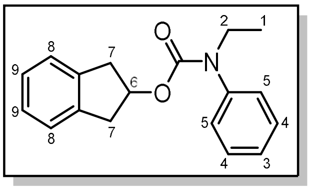

Prepared according to **GP 9** with 2,3-dihydro-1*H*-inden-2-yl phenylcarbamate (518 mg, 2,04 mmol, 1.0 equiv.). Purification by flash column chromatography (SiO_2, 10% v/v EtOAc in hexane) afforded the title compound as a white solid (567 mg, 2.02 mmol, 99% yield).

^{1}H NMR (500 MHz, CD_3CN): *δ* 7.30 (t, *J* = 7.7 Hz, 2 H, H-4), 7.21-7.13 (m, 7 H, H-3, H-5, H-8, H-9), 5.46-5.42 (m, 1 H, H-6), 3.63 (q, *J* = 7.1 Hz, 2 H, H-2), 3.27 (dd, *J* = 17.0, 6.3 Hz, 2 H, H-7a), 2.93 (br d, *J* = 17.0 Hz, 2 H, H-7b), 1.05 (t, *J* = 7.1 Hz, 3 H, H-1).

^{13}C NMR (126 MHz, CD_3CN): *δ* 155.8, 143.1, 141.8, 129.7, 128.1, 127.5, 127.2, 125.5, 77.3, 46.0, 40.3, 13.9.

R_f = 0.54 (20% v/v EtOAc in hexane).

HRMS (+ESI): *m/z* found $[M+Na]^+$ 304.1317, $[C_{18}H_{19}NO_2Na]^+$ requires 304.1308, (*δ* = +3.0 ppm).

1-phenylpropan-2-yl ethyl(phenyl)carbamate (**(*rac*)-10s**)

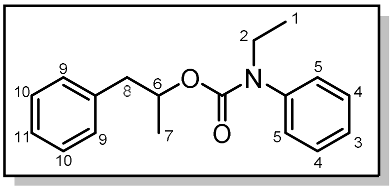

Prepared according to **GP 9** with 1-phenylpropan-2-yl phenylcarbamate (306 mg, 1.20 mmol, 1.0 equiv.). Purification by flash column chromatography (SiO_2, 15% v/v EtOAc in hexane) afforded the title compound as a pale-yellow oil (333 mg, 1.18 mmol, 98% yield).

^{1}H NMR (700 MHz, CD_3CN): δ 7.38-7.35 (m, 2 H, H-4), 7.28-7.20 (m, 4 H, H-3, H-10, H-11), 7.12-7.10 (m, 4 H, H-5, H-9), 5.00-4.96 (m, 1 H, H-6), 3.64-3.59 (m, 2 H, H-2), 2.80-2.74 (m, 2 H, H-8), 1.16 (br d, J = 6.0 Hz, 3 H, H-7), 1.03 (t, J = 7.1 Hz, 3 H, H-1).

^{13}C NMR (176 MHz, CD_3CN): δ 155.5, 143.1, 139.2, 130.5, 129.8, 129.1, 128.5, 127.4, 127.2, 73.4, 45.9, 42.7, 20.2, 13.9.

R_f = 0.56 (20% v/v EtOAc in hexane).

HRMS (+ESI): *m/z* found $[M+H]^+$ 284.1648, $[C_{18}H_{22}NO_2]^+$ requires 284.1645, (δ = +1.1 ppm).

14 Characterisation of Enantioenriched Products from C-H Amination

General Procedure 10 (GP 10):

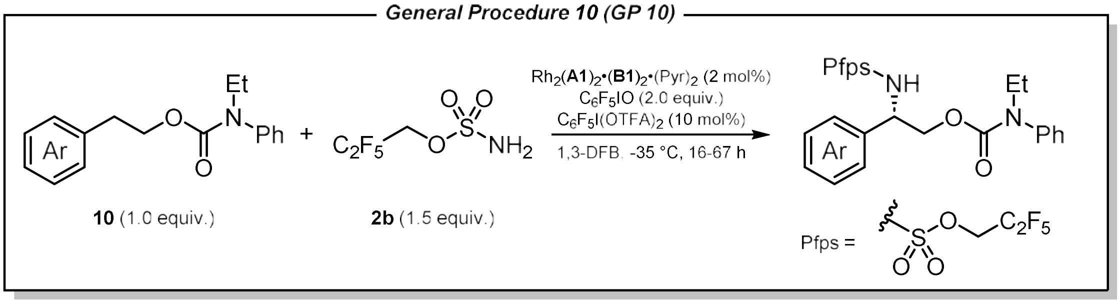

Under an atmosphere of air, a 4.0 mL crimp-top vial was charged with a carbamate substrate **10** (0.10 mmol, 1.0 equiv.) and Rh_2(**A1**)$_2$•(**B1**)$_2$•(Pyr)$_2$ (5.4 mg, 2.0 μmol, 2 mol%). A solution of 2,2,3,3,3-pentafluoropropyl sulfamate **2b** (34.4 mg, 0.15 mmol, 1.5 equiv.) in 1,3-difluorobenzene (0.5 mL, 0.2 M) was added and the vial was cooled to -35 ˚C over 10 min. Pentafluoroiodosobenzene (C_6F_5IO) (62.0 mg, 0.20 mmol, 2.0 equiv.) and perfluorophenyl-λ^3-iodanediyl bis(2,2,2-trifluoroacetate) ($C_6F_5I(OTFA)_2$) (5.2 mg, 0.010 mmol, 10 mol%) were subsequently added together in a single portion at -35 ˚C. The vial was sealed and the reaction mixture stirred at -35 ˚C. After the reaction, saturated aqueous thiourea (1 mL) and $CHCl_3$ (1.5 mL) were added and the biphasic mixture was stirred vigorously for 5 min at -35 ˚C. The mixture was allowed to warm to room temperature and stirred for a further 15 min. The layers were separated and the organic layer was passed through a short pad of $MgSO_4$. The aqueous

layer was extracted with $CHCl_3$ (4 x 1 mL) with each organic layer passed through a short pad of $MgSO_4$.The combined organic layers were concentrated under reduced pressure and the crude residue was purified by flash column chromatography to afford the corresponding product.

The racemic reactions were prepared according to **GP 10** with the following procedural modifications: $Rh_2(esp)_2$ (4 mol%) was used with 1,3-DFB (0.5 mL, 0.2 M) in the absence of $C_6F_5I(OTFA)_2$. Unfortunately, the reaction with $Rh_2(esp)_2$ often led to poor reactivity for the desired benzylic aminated products and complicated our efforts to obtain racemates. Therefore, in order to determine the *ee* values, the antipodes for all benzylic aminated products were prepared according to **GP 10** with catalyst Rh_2(**A1**)$_2$•(**C2**)$_2$•(Pyr)$_2$ (2 mol%).

2,2,3,3,3-pentafluoropropyl (S)-(2-((ethyl(phenyl)carbamoyl)oxy)-1-phenylethyl)sulfamate (**11a**)

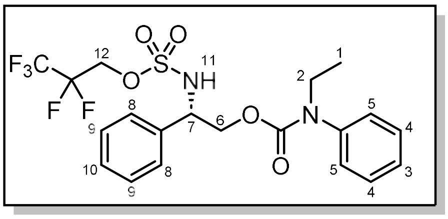

Prepared according to **GP 10** with phenethyl ethyl(phenyl)carbamate **10a** (26.9 mg, 0.10 mmol, 1.0 equiv.) overnight. Purification by flash column chromatography (SiO_2, 2% v/v acetone in $CHCl_3$) afforded the title compound as a colourless oil (42.2 mg, 0.0850 mmol, 85% yield, 96% *ee*).

^{1}H NMR (700 MHz, CD_3CN): δ 7.37-7.27 (m, 8 H, H-3, H-4, H-8, H-9, H-10), 7.16-7.15 (m, 2 H, H-5), 6.87 (br s, 1 H, H-11), 4.72 (br s, 1 H, H-7), 4.37 (q, J = 12.1 Hz, 1 H, H-12a), 4.26-4.20 (m, 3 H, H-6, H-12b), 3.66 (q, J = 7.1 Hz, 2 H, H-2), 1.06 (t, J = 7.1 Hz, 3 H, H-1).

^{13}C NMR (176 MHz, CD_3CN): δ 155.7, 142.5, 138.6, 130.0, 129.7, 129.3, 128.4, 128.1, 127.7, 119.2 (qt, J_{C-F} = 286, 34.5 Hz), 112.7 (tq, J_{C-F} = 256, 38.1 Hz), 67.8, 64.7 (t, J_{C-F} = 28.5 Hz), 58.9, 46.2, 13.8.

^{19}F NMR (376 MHz, CD_3CN): δ -84.4, -124.1 (m).

R_f = 0.46 (4% v/v acetone in $CHCl_3$).

$[\alpha]_D^{25.0}$ = +33.1 (c. 0.83, $CHCl_3$).

HRMS (+ESI): *m/z* found $[M+H]^+$ 497.1169, $[C_{20}H_{22}F_5N_2O_5S]^+$ requires 497.1164, (δ = +1.0 ppm).

Chiral SFC Analysis: CHIRALPAK IK (CO_2:MeOH, 95:5, 2.50 mL min^{-1}, 40℃) t_R = 3.3 (major), 4.0 (minor) minutes.

2,2,3,3,3-pentafluoropropyl (R)-(2-((ethyl(phenyl)carbamoyl)oxy)-1-phenylethyl)sulfamate (***ent*-11a**)

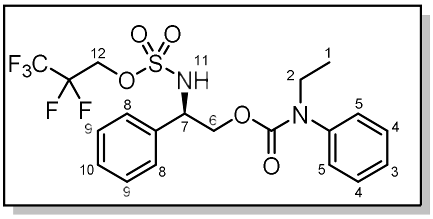

Prepared according to **GP 10** with **10a** (26.9 mg, 0.10 mmol, 1.0 equiv.) and Rh_2(**A1**)$_2$•(**C2**)$_2$•(Pyr)$_2$ (5.4 mg, 2.0 μmol, 2.0 mol%) overnight. Purification by flash column chromatography (SiO_2, 2% v/v acetone in $CHCl_3$) afforded the title compound as a colourless oil (31.7 mg, 0.0639 mmol, 64% yield, 93% *ee*).

$[\alpha]_D^{25.0}$ = -27.2 (c. 0.88, $CHCl_3$).

Chiral SFC Analysis: CHIRALPAK IK (CO_2:MeOH, 95:5, 2.50 mL min^{-1}, 40℃) t_R = 3.3 (minor), 4.0 (major) minutes.

2,2,3,3,3-pentafluoropropyl (S)-(2-((ethyl(phenyl)carbamoyl)oxy)-1-(4-methoxyphenyl) ethyl)sulfamate (**11b**)

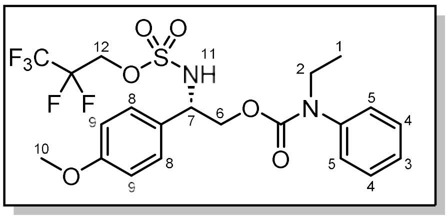

Prepared according to **GP 10** with 4-methoxyphenethyl ethyl(phenyl)carbamate **10b** (29.9 mg, 0.10 mmol, 1.0 equiv.) overnight. Purification by flash column chromatography (SiO_2, 3% v/v acetone in $CHCl_3$) afforded the title compound as a colourless oil (42.7 mg, 0.0811 mmol, 81% yield, 93% *ee*).

^{1}H NMR (700 MHz, CD_3CN): *δ* 7.38-7.35 (m, 2 H, H-4), 7.29-7.16 (m, 5 H, H-3, H-5, H-8), 6.91-6.89 (m, 2 H, H-9), 6.77 (br s, 1 H, H-11), 4.66 (br s, 1 H, H-7), 4.35 (q, *J* = 12.5 Hz, 1 H, H-12a), 4.23-4.18 (m, 3 H, H-6, H-12b), 3.78 (s, 3 H, H-10), 3.66 (q, *J* = 7.1 Hz, 2 H, H-2), 1.06 (t, *J* = 7.1 Hz, 3 H, H-1).

^{13}C NMR (176 MHz, CD_3CN): *δ* 160.7, 155.7, 142.6, 130.5, 130.0, 129.5, 128.4, 127.7, 119.2 (qt, $J_{C\text{-}F}$ = 286, 34.5 Hz), 115.0, 112.7 (tq, $J_{C\text{-}F}$ = 255, 38.1 Hz), 67.8, 64.6 (t, $J_{C\text{-}F}$ = 28.1 Hz), 58.4, 56.0, 46.2, 13.8.

^{19}F NMR (376 MHz, CD_3CN): *δ* -84.4, -124.1 (m).

R_f = 0.34 (4% v/v acetone in $CHCl_3$).

$[\alpha]_D^{25.0}$ = +37.4 (c. 0.79, $CHCl_3$).

HRMS (+ESI): *m/z* found $[M+H]^+$ 527.1274, $[C_{21}H_{24}F_5N_2O_6S]^+$ requires 527.1270, (*δ* = +0.8 ppm).

Chiral SFC Analysis: CHIRALPAK IJ (CO_2:MeOH, 94:6, 2.50 mL min^{-1}, 40℃) t_R = 3.5 (major), 4.4 (minor) minutes.

2,2,3,3,3-pentafluoropropyl (R)-(2-((ethyl(phenyl)carbamoyl)oxy)-1-(4-methoxyphenyl) ethyl)sulfamate (***ent*-11b**)

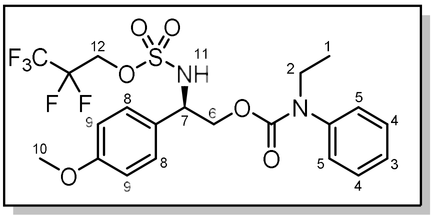

Prepared according to **GP 10** with **10b** (29.9 mg, 0.10 mmol, 1.0 equiv.) and Rh_2(**A1**)$_2$•(**C2**)$_2$•(Pyr)$_2$ (5.4 mg, 2.0 μmol, 2.0 mol%) overnight. Purification by flash column chromatography (SiO_2, 2% v/v acetone in $CHCl_3$) afforded the title compound as a colourless oil (42.4 mg, 0.0805 mmol, 81% yield, 89% *ee*).

[α]$_D^{25.0}$ = -30.3 (c. 0.59, $CHCl_3$).

Chiral SFC Analysis: CHIRALPAK IJ (CO_2:MeOH, 94:6, 2.50 mL min^{-1}, 40℃) t_R = 3.5 (minor), 4.3 (major) minutes.

2,2,3,3,3-pentafluoropropyl (S)-(1-(4-(tert-butyl)phenyl)-2-((ethyl(phenyl)carbamoyl)oxy) ethyl)sulfamate (**11c**)

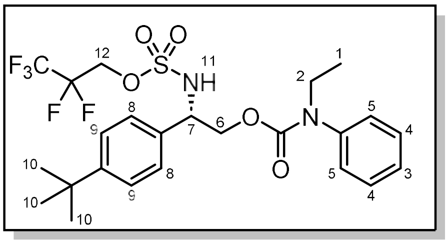

Prepared according to **GP 10** with 4-(*tert*-butyl)phenethyl ethyl(phenyl)carbamate **10c** (32.5 mg, 0.10 mmol, 1.0 equiv.) overnight. Purification by flash column chromatography (SiO_2, 2% v/v acetone in $CHCl_3$) afforded the title compound as a colourless oil (45.9 mg, 0.0831 mmol, 83% yield, 95% *ee*).

^{1}H NMR (700 MHz, CD_3CN): *δ* 7.42-7.34 (m, 4 H, H-4, H-9), 7.29-7.11 (m, 5 H, H-3, H-5, H-8), 6.83 (br s, 1 H, H-11), 4.69 (br s, 1 H, H-7), 4.35-4.13 (m, 4 H, H-6, H-12), 3.65 (q, *J* = 7.1 Hz, 2 H, H-2), 1.31 (s, 9 H, H-10), 1.05 (t, *J* = 7.1 Hz, 3 H, H-1).

^{13}C NMR (176 MHz, CD_3CN): *δ* 155.7, 152.5, 142.5, 135.6, 130.0, 128.4, 127.9, 127.7, 126.6, 119.2 (qt, $J_{C\text{-}F}$ = 286, 34.6 Hz), 112.6 (tq, $J_{C\text{-}F}$ = 255, 38.1 Hz), 67.7, 64.6 (t, $J_{C\text{-}F}$ = 27.7 Hz), 58.6, 46.3, 35.2, 31.5, 13.8.

^{19}F NMR (376 MHz, CD_3CN): *δ* -84.4, -124.2 (m).

R_f = 0.53 (4% v/v acetone in $CHCl_3$).

[α]$_D^{25.0}$ = +27.3 (c. 1.24, $CHCl_3$).

HRMS (+ESI): *m/z* found $[M+H]^+$ 553.1798, $[C_{24}H_{30}F_5N_2O_5S]^+$ requires 553.1790, (δ = +1.4 ppm).

Chiral SFC Analysis: CHIRALPAK IJ (CO_2:MeOH, 94:6, 2.50 mL min^{-1}, 40℃) t_R = 2.4 (major), 2.8 (minor) minutes.

2,2,3,3,3-pentafluoropropyl (R)-(1-(4-(tert-butyl)phenyl)-2-((ethyl(phenyl)carbamoyl)oxy) ethyl)sulfamate (***ent*-11c**)

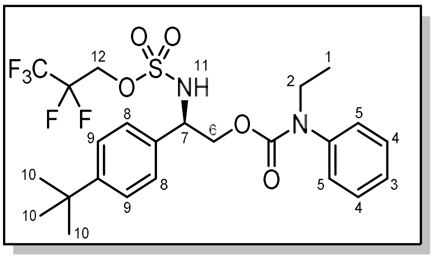

Prepared according to **GP 10** with **10c** (32.5 mg, 0.10 mmol, 1.0 equiv.) and Rh_2(**A1**)$_2$•(**C2**)$_2$•(Pyr)$_2$ (5.4 mg, 2.0 μmol, 2.0 mol%) overnight. Purification by flash column chromatography (SiO_2, 2% v/v acetone in $CHCl_3$) afforded the title compound as a colourless oil (40.5 mg, 0.0733 mmol, 73% yield, 91% *ee*).

$[\alpha]_D^{25.0}$ = -26.9 (c. 0.97, $CHCl_3$).

Chiral SFC Analysis: CHIRALPAK IJ (CO_2:MeOH, 94:6, 2.50 mL min^{-1}, 40℃) t_R = 2.4 (minor), 2.8 (major) minutes.

2,2,3,3,3-pentafluoropropyl (S)-(2-((ethyl(phenyl)carbamoyl)oxy)-1-(p-tolyl)ethyl)sulfamate (**11d**)

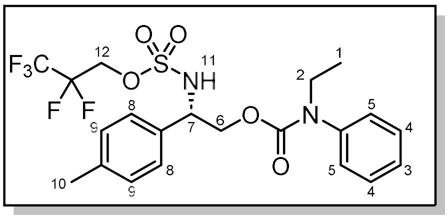

Prepared according to **GP 10** with 4-methylphenethyl ethyl(phenyl)carbamate **10d** (28.3 mg, 0.10 mmol, 1.0 equiv.) overnight. Purification by flash column chromatography (SiO_2, 2% v/v acetone in $CHCl_3$, *then* 20% v/v EtOAc in hexane) afforded the title compound as a pale-yellow oil (33.8 mg, 0.0662 mmol, 66% yield, 95% *ee*).

^{1}H NMR (700 MHz, CD_3CN): δ 7.38-7.35 (m, 2 H, H-4), 7.29-7.15 (m, 7 H, H-3, H-5, H-8, H-9), 6.80 (br s, 1 H, H-11), 4.67 (br s, 1 H, H-7), 4.38-4.18 (m, 4 H, H-6, H-12), 3.66 (q, *J* = 7.1 Hz, 2 H, H-2), 2.33 (s, 3 H, H-10), 1.06 (t, *J* = 7.1 Hz, 3 H, H-1).

^{13}C NMR (176 MHz, CD_3CN): δ 155.7, 142.6, 139.3, 135.6, 130.3, 130.0, 128.4, 128.1, 127.7, 119.2 (qt, $J_{C\text{-}F}$ = 286, 34.5 Hz), 112.7 (tq, $J_{C\text{-}F}$ = 255, 38.3 Hz), 67.8, 64.7 (t, $J_{C\text{-}F}$ = 27.8 Hz), 58.7, 46.2, 21.1, 13.8.

^{19}F NMR (376 MHz, CD_3CN): *δ* -84.4, -124.1 (m).

R_f = 0.48 (4% v/v acetone in $CHCl_3$), 0.30 (20% v/v EtOAc in hexane).

$[\alpha]_D^{25.0}$ = +30.6 (c. 0.93, $CHCl_3$).

HRMS (+ESI): *m/z* found $[M+H]^+$ 511.1328, $[C_{21}H_{24}F_5N_2O_5S]^+$ requires 511.1321, (*δ* = +1.4 ppm).

Chiral SFC Analysis: CHIRALPAK IJ (CO_2:MeOH, 94:6, 2.50 mL min^{-1}, 40℃) t_R = 2.6 (major), 3.1 (minor) minutes.

2,2,3,3,3-pentafluoropropyl (R)-(2-((ethyl(phenyl)carbamoyl)oxy)-1-(p-tolyl)ethyl)sulfamate (***ent*-11d**)

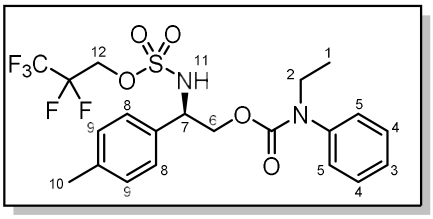

Prepared according to **GP 10** with **10d** (28.3 mg, 0.10 mmol, 1.0 equiv.) and Rh_2(**A1**)$_2$•(**C2**)$_2$•(Pyr)$_2$ (5.4 mg, 2.0 μmol, 2.0 mol%) overnight. Purification by flash column chromatography (SiO_2, 2% v/v acetone in $CHCl_3$, *then* 20% v/v EtOAc in hexane) afforded the title compound as a colourless oil (24.9 mg, 0.0487 mmol, 49% yield, 93% *ee*).

$[\alpha]_D^{25.0}$ = -31.7 (c. 0.70, $CHCl_3$).

Chiral SFC Analysis: CHIRALPAK IJ (CO_2:MeOH, 94:6, 2.50 mL min^{-1}, 40℃) t_R = 2.6 (minor), 3.1 (major) minutes.

2,2,3,3,3-pentafluoropropyl (S)-(1-(4-bromophenyl)-2-((ethyl(phenyl)carbamoyl)oxy) ethyl)sulfamate (**11e**)

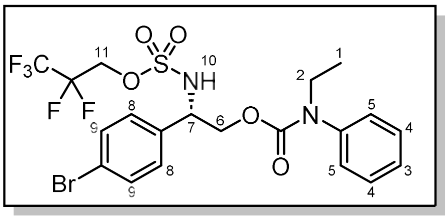

Prepared according to **GP 10** with 4-bromophenethyl ethyl(phenyl)carbamate **10e** (34.8 mg, 0.10 mmol, 1.0 equiv.) for 66 h. Purification by flash column chromatography (SiO_2, 2% v/v acetone in $CHCl_3$) afforded the title compound as a white solid (29.2 mg, 0.0508 mmol, 51% yield, 88% *ee*).

^{1}H NMR (700 MHz, CD_3CN): *δ* 7.50 (br d, *J* = 6.1 Hz, 2 H, H-9), 7.38-7.35 (m, 2 H, H-4), 7.30-7.14 (m, 5 H, H-3, H-5, H-8), 6.87 (br s, 1 H, H-10), 4.70 (br s, 1 H, H-7), 4.45-4.24 (m, 4 H, H-6, H-11), 3.64 (q, *J* = 7.2 Hz, 2 H, H-2), 1.05 (t, *J* = 7.1 Hz, 3 H, H-1).

^{13}C NMR (176 MHz, CD_3CN): δ 155.5, 142.4, 138.0, 132.6, 130.2, 130.0, 128.4, 127.8, 122.7, 119.2 (qt, J_{C-F} = 286, 34.3 Hz), 112.7 (tq, J_{C-F} = 256, 37.7 Hz), 67.5, 64.7 (t, J_{C-F} =28.1 Hz), 58.2, 46.2, 13.8.

^{19}F NMR (376 MHz, CD_3CN): δ -84.4, -124.1 (m).

R_f = 0.41 (4% v/v acetone in $CHCl_3$).

$[\alpha]_D^{25.0}$ = +32.2 (c. 0.73, $CHCl_3$).

HRMS (+ESI): *m/z* found $[M+H]^+$ 575.0278, $[C_{20}H_{21}BrF_5N_2O_5S]^+$ requires 575.0269, (δ = +1.6 ppm).

Chiral SFC Analysis: CHIRALPAK IC (CO_2:MeOH, 95:5, 2.50 mL min^{-1}, 40℃) t_R = 3.6 (major), 4.5 (minor) minutes.

2,2,3,3,3-pentafluoropropyl (R)-(1-(4-bromophenyl)-2-((ethyl(phenyl)carbamoyl)oxy) ethyl)sulfamate (***ent*-11e**)

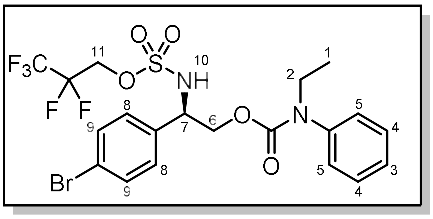

Prepared according to **GP 10** with **10e** (34.8 mg, 0.100 mmol, 1.0 equiv.) and Rh_2(**A1**)$_2$•(**C2**)$_2$•(Pyr)$_2$ (5.4 mg, 2.0 μmol, 2.0 mol%) for 67 h. Purification by flash column chromatography (SiO_2, 2% v/v acetone in $CHCl_3$) afforded the title compound as a colourless oil (19.6 mg, 0.0341 mmol, 34% yield, 86% *ee*).

$[\alpha]_D^{25.0}$ = -31.9 (c. 0.59, $CHCl_3$).

Chiral SFC Analysis: CHIRALPAK IC (CO_2:MeOH, 95:5, 2.50 mL min^{-1}, 40℃) t_R = 3.6 (minor), 4.5 (major) minutes.

2,2,3,3,3-pentafluoropropyl (S)-(1-(4-chlorophenyl)-2-((ethyl(phenyl)carbamoyl)oxy) ethyl)sulfamate (**11f**)

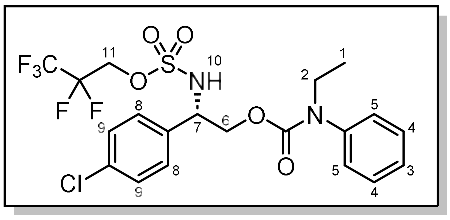

Prepared according to **GP 10** with 4-chlorophenethyl ethyl(phenyl)carbamate **10f** (30.4 mg, 0.10 mmol, 1.0 equiv.) for 66 h. Purification by flash column chromatography (SiO_2, 2% v/v acetone in $CHCl_3$) afforded the title compound as a pale-yellow oil (29.0 mg, 0.0546 mmol, 55% yield, 90% *ee*).

^{1}H NMR (700 MHz, CD_3CN): *δ* 7.38-7.25 (m, 9 H, H-3, H-4, H-5, H-8, H-9), 6.87 (br s, 1 H, H-10), 4.71 (br s, 1 H, H-7), 4.45-4.24 (m, 4 H, H-6, H-11), 3.64 (q, *J* = 7.2 Hz, 2 H, H-2), 1.05 (t, *J* = 7.2 Hz, 3 H, H-1).

^{13}C NMR (176 MHz, CD_3CN): *δ* 155.5, 142.5, 137.5, 134.6, 130.0, 129.9, 129.6, 128.4, 127.8, 119.2 (qt, $J_{C\text{-}F}$ = 286, 34.6 Hz), 112.7 (tq, $J_{C\text{-}F}$ = 256, 38.3 Hz), 67.6, 64.7 (t, $J_{C\text{-}F}$ = 27.8 Hz), 58.1, 46.2, 13.8.

^{19}F NMR (376 MHz, CD_3CN): *δ* -84.4, -124.1 (m).

R_f = 0.48 (4% v/v acetone in $CHCl_3$).

$[\alpha]_D^{25.0}$ = +34.0 (c. 0.65, $CHCl_3$).

HRMS (+ESI): *m/z* found $[M+H]^+$ 531.0777, $[C_{20}H_{21}ClF_5N_2O_5S]^+$ requires 531.0774, (*δ* = +0.6 ppm).

Chiral SFC Analysis: CHIRALPAK IJ (CO_2:MeOH, 94:6, 2.50 mL min^{-1}, 40℃) t_R = 3.5 (major), 4.3 (minor) minutes.

2,2,3,3,3-pentafluoropropyl (R)-(1-(4-chlorophenyl)-2-((ethyl(phenyl)carbamoyl)oxy) ethyl)sulfamate (***ent*-11f**)

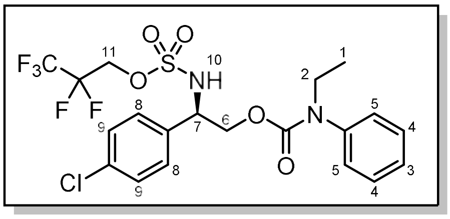

Prepared according to **GP 10** with **10f** (30.4 mg, 0.10 mmol, 1.0 equiv.) and $Rh_2(\textbf{A1})_2 \cdot (\textbf{C2})_2 \cdot (Pyr)_2$ (5.4 mg, 2.0 μmol, 2.0 mol%) for 44 h. Purification by flash column chromatography (SiO_2, 2% v/v acetone in $CHCl_3$) afforded the title compound as a pale-yellow oil (21.0 mg, 0.0396 mmol, 40% yield, 87% *ee*).

$[\alpha]_D^{25.0}$ = -33.7 (c. 0.59, $CHCl_3$).

Chiral SFC Analysis: CHIRALPAK IJ (CO_2:MeOH, 94:6, 2.50 mL min^{-1}, 40℃) t_R = 3.6 (minor), 4.3 (major) minutes.

2,2,3,3,3-pentafluoropropyl (S)-(2-((ethyl(phenyl)carbamoyl)oxy)-1-(4-(trifluoromethyl) phenyl)ethyl)sulfamate (**11g**)

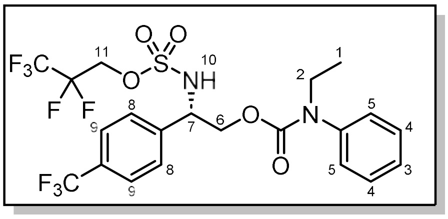

Prepared according to **GP 10** with 4-(trifluoromethyl)phenethyl ethyl(phenyl)carbamate **10g** (33.7 mg, 0.10 mmol, 1.0 equiv.) for 66 h. Purification by flash column chromatography (SiO_2, 2% v/v acetone in $CHCl_3$) afforded the title compound as a colourless oil (18.6 mg, 0.0330 mmol, 33% yield, 89% *ee*).

^{1}H NMR (700 MHz, CD_3CN): δ 7.65-7.27 (m, 7 H, H-3, H-4, H-8, H-9), 7.13-6.98 (m, 3 H, H-5, H-10), 4.81 (br s, 1 H, H-7), 4.46 (m, 4 H, H-6, H-11), 3.64 (q, J = 7.1 Hz, 2 H, H-2), 1.04 (t, J = 7.1 Hz, 3 H, H-1).

^{13}C NMR (176 MHz, CD_3CN): δ 155.5, 143.2, 142.4, 130.7 (q, $J_{C\text{-}F}$ = 32.2 Hz), 130.0, 128.9, 128.4, 127.8, 126.5 (q, $J_{C\text{-}F}$ = 3.4 Hz), 125.3 (q, $J_{C\text{-}F}$ = 272 Hz), 119.2 (qt, $J_{C\text{-}F}$ = 286, 34.5 Hz), 112.6 (tq, $J_{C\text{-}F}$ = 255, 38.3 Hz), 67.5, 64.8 (t, $J_{C\text{-}F}$ = 27.9 Hz), 58.3, 46.2, 13.7.

^{19}F NMR (376 MHz, CD_3CN): δ -63.2, -84.4, -124.2(m).

R_f = 0.40 (4% v/v acetone in $CHCl_3$).

$[\alpha]_D^{25.0}$ = +24.7 (c. 1.24, $CHCl_3$).

HRMS (+ESI): *m/z* found $[M+H]^+$ 565.1043, $[C_{21}H_{21}F_8N_2O_5S]^+$ requires 565.1038, (δ = +0.9 ppm).

Chiral SFC Analysis: CHIRALPAK IJ (CO_2:MeOH, 94:6, 2.50 mL min^{-1}, 40℃) t_R = 2.0 (major), 2.2 (minor) minutes.

2,2,3,3,3-pentafluoropropyl (R)-(2-((ethyl(phenyl)carbamoyl)oxy)-1-(4-(trifluoromethyl) phenyl)ethyl)sulfamate (***ent*-11g**)

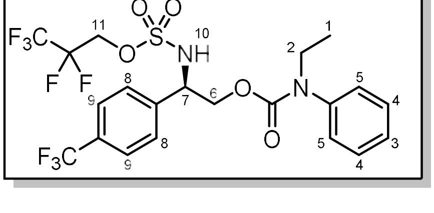

Prepared according to **GP 10** with **10g** (33.7 mg, 0.10 mmol, 1.0 equiv.) and Rh_2(**A1**)$_2$•(**C2**)$_2$•(Pyr)$_2$ (5.4 mg, 2.0 μmol, 2.0 mol%) for 44 h. Purification by flash column chromatography (SiO_2, 2% v/v acetone in $CHCl_3$) afforded the title compound as a pale-yellow oil (10.6 mg, 0.0188 mmol, 19% yield, 87% *ee*).

$[\alpha]_D^{25.0}$ = -28.8 (c. 0.59, $CHCl_3$).

Chiral SFC Analysis: CHIRALPAK IJ (CO_2:MeOH, 94:6, 2.50 mL min^{-1}, 40℃) t_R = 2.0 (minor), 2.2 (major) minutes.

2,2,3,3,3-pentafluoropropyl (S)-(2-((ethyl(phenyl)carbamoyl)oxy)-1-(3-methoxyphenyl) ethyl)sulfamate (**11h**)

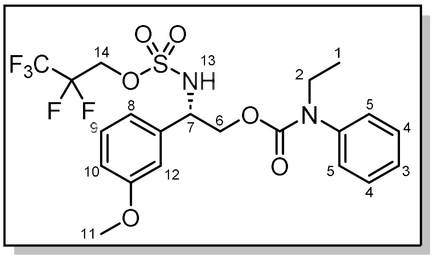

Prepared according to **GP 10** with 3-methoxyphenethyl ethyl(phenyl)carbamate **10h** (29.9 mg, 0.10 mmol, 1.0 equiv.) overnight. Purification by flash column chromatography (SiO_2, 2% v/v acetone in $CHCl_3$) afforded the title compound as a colourless oil (27.7 mg, 0.0526 mmol, 53% yield, 97% *ee*).

^{1}H NMR (700 MHz, CD_3CN): δ 7.36-7.34 (m, 2 H, H-4), 7.30-7.26 (m, 2 H, H-3, H-9), 7.15 (br d, J = 5.0 Hz, 2 H, H-5), 6.90-6.84 (m, 4 H, H-8, H-10, H-12, H-13), 4.69 (br s, 1 H, H-7), 4.40-4.21 (m, 4 H, H-6, H-14), 3.78 (s, 3 H, H-11), 3.66 (q, J = 7.1 Hz, 2 H, H-2), 1.06 (t, J = 7.1 Hz, 3 H, H-1).

^{13}C NMR (176 MHz, CD_3CN): δ 161.0, 155.7, 142.6, 140.1, 130.9, 129.9, 128.4, 127.7, 120.2, 119.2 (qt, $J_{C\text{-}F}$ = 286, 34.8 Hz), 114.8, 113.7, 112.7 (tq, $J_{C\text{-}F}$ = 255, 38.1 Hz), 67.8, 64.7 (t, $J_{C\text{-}F}$ = 27.8 Hz), 58.8, 56.0, 46.3, 13.8.

^{19}F NMR (376 MHz, CD_3CN): δ -84.4, -124.2 (m).

R_f = 0.36 (4% v/v acetone in $CHCl_3$).

$[\alpha]_D^{25.0}$ = +29.2 (c. 0.59, $CHCl_3$).

HRMS (+ESI): *m/z* found $[M+H]^+$ 527.1277, $[C_{21}H_{24}F_5N_2O_6S]^+$ requires 527.1270, (δ = +1.3 ppm).

Chiral SFC Analysis: CHIRALPAK IJ (CO_2:MeOH, 94:6, 2.50 mL min^{-1}, 40℃) t_R = 3.0 (major), 3.8 (minor) minutes.

2,2,3,3,3-pentafluoropropyl (R)-(2-((ethyl(phenyl)carbamoyl)oxy)-1-(3-methoxyphenyl) ethyl)sulfamate (***ent*-11h**)

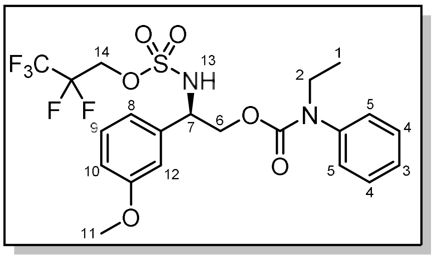

Prepared according to **GP 10** with **10h** (29.9 mg, 0.10 mmol, 1.0 equiv.) and Rh_2(**A1**)$_2$•(**C2**)$_2$•(Pyr)$_2$ (5.4 mg, 2.0 μmol, 2.0 mol%) overnight. Purification by flash column chromatography (SiO_2, 2.5% v/v acetone in $CHCl_3$) afforded the title compound as a pale-yellow oil (16.3 mg, 0.0310 mmol, 31% yield, 92% *ee*).

$[\alpha]_D^{25.0}$ = -26.6 (c. 1.09, $CHCl_3$).

Chiral SFC Analysis: CHIRALPAK IJ (CO_2:MeOH, 94:6, 2.50 mL min^{-1}, 40℃) t_R = 2.9 (minor), 3.7 (major) minutes.

2,2,3,3,3-pentafluoropropyl (S)-(2-((ethyl(phenyl)carbamoyl)oxy)-1-(m-tolyl)ethyl)sulfamate (**11i**)

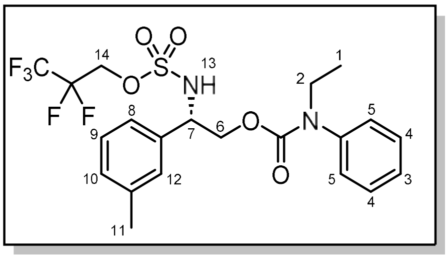

Prepared according to **GP 10** with 3-methylphenethyl ethyl(phenyl)carbamate **10i** (28.3 mg, 0.10 mmol, 1.0 equiv.) overnight. Purification by flash column chromatography (SiO_2, 2% v/v acetone in $CHCl_3$, *then* 20% v/v EtOAc in hexane) afforded the title compound as a pale-yellow oil (33.9 mg, 0.0664 mmol, 66% yield, 94% *ee*).

^{1}H NMR (700 MHz, CD_3CN): δ 7.36 (t, J = 7.7 Hz, 2 H, H-4), 7.28-7.09 (m, 7 H, H-3, H-5, H-8, H-9, H-10, H-12), 6.83 (br s, 1 H, H-13), 4.67 (br s, 1 H, H-7), 4.39-4.19 (m, 4 H, H-6, H-14), 3.66 (q, J = 7.1 Hz, 2 H, H-2), 2.32 (s, 3 H, H-11), 1.06 (t, J = 7.1 Hz, 3 H, H-1).

^{13}C NMR (176 MHz, CD_3CN): δ 155.7, 142.6, 139.5, 138.5, 129.98, 129.95, 129.6, 128.8, 128.4, 127.7, 125.1, 119.2 (qt, $J_{C\text{-}F}$ = 286, 34.5 Hz), 112.7 (tq, $J_{C\text{-}F}$ = 255, 38.7 Hz), 67.9, 64.7 (t, $J_{C\text{-}F}$ = 27.8 Hz), 58.9, 46.3, 21.4, 13.8.

^{19}F NMR (376 MHz, CD_3CN): δ -84.4, -124.2 (m).

R_f = 0.45 (4% v/v acetone in $CHCl_3$), 0.26 (20% v/v EtOAc in hexane).

$[\alpha]_D^{25.0}$ = +26.6 (c. 0.77, $CHCl_3$).

HRMS (+ESI): *m/z* found $[M+H]^+$ 511.1324, $[C_{21}H_{24}F_5N_2O_5S]^+$ requires 511.1321, (δ = +0.6 ppm).

Chiral SFC Analysis: CHIRALPAK IJ (CO_2:MeOH, 94:6, 2.50 mL min^{-1}, 40℃) t_R = 2.6 (major), 3.0 (minor) minutes.

2,2,3,3,3-pentafluoropropyl (R)-(2-((ethyl(phenyl)carbamoyl)oxy)-1-(m-tolyl)ethyl)sulfamate (***ent*-11i**)

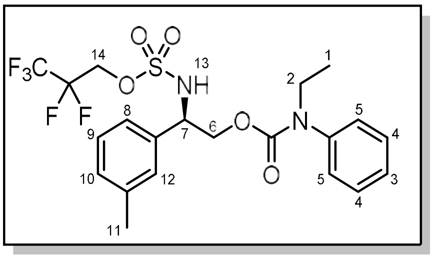

Prepared according to **GP 10** with **10i** (28.3 mg, 0.10 mmol, 1.0 equiv.) and Rh_2(**A1**)$_2$•(**C2**)$_2$•(Pyr)$_2$ (5.4 mg, 2.0 μmol, 2.0 mol%) overnight. Purification by flash column chromatography (SiO_2, 2% v/v acetone in $CHCl_3$, *then* 20% v/v EtOAc in hexane) afforded the title compound as a pale-yellow oil (18.3 mg, 0.0358 mmol, 36% yield, 86% *ee*).

$[\alpha]_D^{25.0}$ = -23.7 (c. 1.13, $CHCl_3$).

Chiral SFC Analysis: CHIRALPAK IJ (CO_2:MeOH, 94:6, 2.50 mL min^{-1}, 40℃) t_R = 2.6 (minor), 3.0 (major) minutes.

2,2,3,3,3-pentafluoropropyl (S)-(1-(3-chlorophenyl)-2-((ethyl(phenyl)carbamoyl)oxy) ethyl)sulfamate (**11j**)

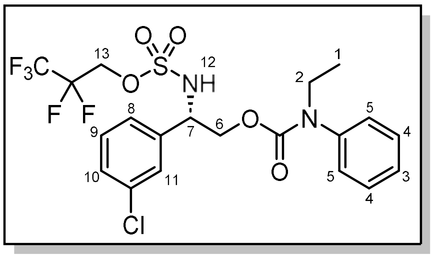

Prepared according to **GP 10** with 3-chlorophenethyl ethyl(phenyl)carbamate **10j** (30.4 mg, 0.10 mmol, 1.0 equiv.) for 44 h. Purification by flash column chromatography (SiO_2, 2% v/v acetone in $CHCl_3$) afforded the title compound as a colourless oil (21.2 mg, 0.0399 mmol, 40% yield, 92% *ee*).

^{1}H NMR (700 MHz, CD_3CN): *δ* 7.37-7.14 (m, 9 H, H-3, H-4, H-5, H-8, H-9, H-10, H-11), 6.90 (br s, 1 H, H-12), 4.72 (br s, 1 H, H-7), 4.47-4.26 (m, 4 H, H-6, H-13), 3.65 (q, *J* = 7.2 Hz, 2 H, H-2), 1.05 (t, *J* = 7.2 Hz, 3 H, H-1).

^{13}C NMR (176 MHz, CD_3CN): *δ* 155.5, 142.4, 141.0, 134.9, 131.3, 130.0, 129.3, 128.4, 128.2, 127.8, 126.7, 119.2 (qt, $J_{C\text{-}F}$ = 286, 34.6 Hz), 112.7 (tq, $J_{C\text{-}F}$ = 255, 38.1 Hz), 67.6, 64.8 (t, $J_{C\text{-}F}$ = 27.9 Hz), 58.3, 46.3, 13.8.

^{19}F NMR (376 MHz, CD_3CN): *δ* -84.4, -124.2 (m).

R_f = 0.40 (4% v/v acetone in $CHCl_3$).

$[\alpha]_D^{25.0}$ = +37.8 (c. 0.46, $CHCl_3$).

HRMS (+ESI): *m/z* found $[M+H]^+$ 531.0776, $[C_{20}H_{21}ClF_5N_2O_5S]^+$ requires 531.0774, (δ = +0.4 ppm).

Chiral SFC Analysis: CHIRALPAK IJ (CO_2:MeOH, 94:6, 2.50 mL min^{-1}, 40℃) t_R = 3.4 (major), 4.1 (minor) minutes.

2,2,3,3,3-pentafluoropropyl (R)-(1-(3-chlorophenyl)-2-((ethyl(phenyl)carbamoyl)oxy) ethyl)sulfamate (***ent*-11j**)

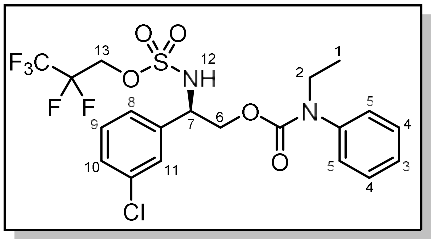

Prepared according to **GP 10** with **10j** (30.4 mg, 0.100 mmol, 1.0 equiv.) and Rh_2(**A1**)$_2$•(**C2**)$_2$•(Pyr)$_2$ (5.4 mg, 2.0 μmol, 2.0 mol%) for 43 h. Purification by flash column chromatography (SiO_2, 2% v/v acetone in $CHCl_3$) afforded the title compound as a pale-yellow oil (12.8 mg, 0.0241 mmol, 24% yield, 86% *ee*).

$[\alpha]_D^{25.0}$ = -31.4 (c. 0.72, $CHCl_3$).

Chiral SFC Analysis: CHIRALPAK IJ (CO_2:MeOH, 94:6, 2.50 mL min^{-1}, 40℃) t_R = 3.4 (minor), 4.1 (major) minutes.

2,2,3,3,3-pentafluoropropyl (S)-(1-(3-bromophenyl)-2-((ethyl(phenyl)carbamoyl)oxy) ethyl)sulfamate (**11k**)

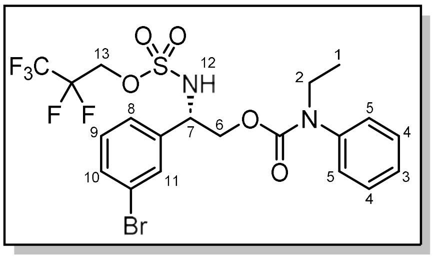

Prepared according to **GP 10** with 3-bromophenethyl ethyl(phenyl)carbamate **10k** (34.8 mg, 0.10 mmol, 1.0 equiv.) for 66 h. Purification by flash column chromatography (SiO_2, 2% v/v acetone in $CHCl_3$) afforded the title compound as a colourless oil (21.6 mg, 0.0375 mmol, 38% yield, 93% *ee*).

^{1}H NMR (700 MHz, CD_3CN): δ 7.51-7.14 (m, 9 H, H-3, H-4, H-5, H-8, H-9, H-10, H-11), 6.87 (br s, 1 H, H-12), 4.71 (br s, 1 H, H-7), 4.47-4.26 (m, 4 H, H-6, H-13), 3.65 (q, *J* = 7.2 Hz, 2 H, H-2), 1.05 (t, *J* = 7.2 Hz, 3 H, H-1).

^{13}C NMR (176 MHz, CD_3CN): δ 155.5, 142.4, 141.2, 132.3, 131.6, 131.1, 130.0, 128.4, 127.8, 127.1, 123.1, 119.2 (qt, J_{C-F} = 286, 34.5 Hz), 112.7 (tq, J_{C-F} = 255, 38.2 Hz), 67.6, 64.8 (t, J_{C-F} = 27.9 Hz), 58.2, 46.3, 13.8.

^{19}F NMR (376 MHz, CD_3CN): δ -84.4, -124.2 (m).

R_f = 0.49 (4% v/v acetone in $CHCl_3$).

$[\alpha]_D^{25.0}$ = +35.8 (c. 0.50, $CHCl_3$).

HRMS (+ESI): *m/z* found $[M+H]^+$ 575.0282, $[C_{20}H_{21}BrF_5N_2O_5S]^+$ requires 575.0269, (δ = +2.3 ppm).

Chiral SFC Analysis: CHIRALPAK IJ (CO_2:MeOH, 94:6, 2.50 mL min^{-1}, 40℃) t_R = 3.9 (major), 4.8 (minor) minutes.

2,2,3,3,3-pentafluoropropyl (R)-(1-(3-bromophenyl)-2-((ethyl(phenyl)carbamoyl)oxy) ethyl)sulfamate (***ent*-11k**)

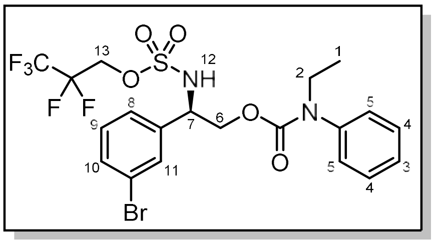

Prepared according to **GP 10** with **10k** (34.8 mg, 0.10 mmol, 1.0 equiv.) and Rh_2(**A1**)$_2$•(**C2**)$_2$•(Pyr)$_2$ (5.4 mg, 2.0 μmol, 2.0 mol%) for 43 h. Purification by flash column chromatography (SiO_2, 2% v/v acetone in $CHCl_3$) afforded the title compound as a pale-yellow oil (12.1 mg, 0.0210 mmol, 21% yield, 86% *ee*).

$[\alpha]_D^{25.0}$ = -31.4 (c. 0.64, $CHCl_3$).

Chiral SFC Analysis: CHIRALPAK IJ (CO_2:MeOH, 94:6, 2.50 mL min^{-1}, 40℃) t_R = 4.0 (minor), 4.8 (major) minutes.

2,2,3,3,3-pentafluoropropyl (S)-(2-((ethyl(phenyl)carbamoyl)oxy)-1-(3-(trifluoromethyl) phenyl)ethyl)sulfamate (**11l**)

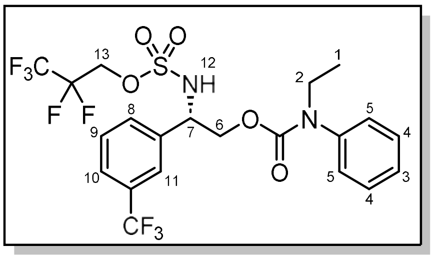

Prepared according to **GP 10** with 3-(trifluoromethyl)phenethyl ethyl(phenyl)carbamate **10l** (33.7 mg, 0.10 mmol, 1.0 equiv.) for 66 h. Purification by flash column chromatography (SiO_2, 2% v/v acetone in $CHCl_3$) afforded the title compound as a pale-yellow oil (11.6 mg, 0.0206 mmol, 21% yield, 91% *ee*).

^{1}H NMR (700 MHz, CD_3CN): δ 7.66-7.55 (m, 4 H, H-8, H-9, H-10, H-11), 7.36-7.33 (m, 2 H, H-4), 7.28-7.26 (m, 1 H, H-3), 7.13-6.95 (m, 3 H, H-5, H-12), 4.82 (br s, 1 H, H-7), 4.47-4.30 (m, 4 H, H-6, H-13), 3.64 (q, *J* = 7.1 Hz, 2 H, H-2), 1.04 (t, *J* = 7.1 Hz, 3 H, H-1).

^{13}C NMR (176 MHz, CD_3CN): δ 155.5, 142.4, 140.0, 132.2, 131.2 (q, J_{C-F} = 32.2 Hz), 130.6, 130.0, 128.3, 127.8, 126.1 (q, J_{C-F} = 3.6 Hz), 125.2 (q, J_{C-F} = 272 Hz), 124.9 (q, J_{C-F} = 3.7 Hz), 119.2 (qt, J_{C-F} = 285, 34.5 Hz), 112.6 (tq, J_{C-F} = 255, 38.1 Hz), 67.5, 64.8 (t, J_{C-F} = 27.8 Hz), 58.3, 46.3, 13.7.

^{19}F NMR (376 MHz, CD_3CN): δ -63.2, -84.5, -124.3 (m).

R_f = 0.49 (4% v/v acetone in $CHCl_3$).

$[\alpha]_D^{25.0}$ = +30.3 (c. 0.73, $CHCl_3$).

HRMS (+ESI): *m/z* found $[M+H]^+$ 565.1050, $[C_{21}H_{21}F_8N_2O_5S]^+$ requires 565.1038, (δ = +2.1 ppm).

Chiral SFC Analysis: CHIRALPAK IJ (CO_2:MeOH, 94:6, 2.50 mL min^{-1}, 40℃) t_R = 2.0 (major), 2.3 (minor) minutes.

2,2,3,3,3-pentafluoropropyl (R)-(2-((ethyl(phenyl)carbamoyl)oxy)-1-(3-(trifluoromethyl) phenyl)ethyl)sulfamate (***ent*-11l**)

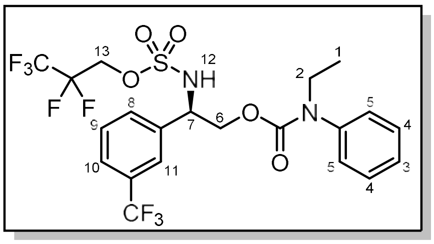

Prepared according to **GP 10** with **10l** (33.7 mg, 0.10 mmol, 1.0 equiv.) and Rh_2(**A1**)$_2$•(**C2**)$_2$•(Pyr)$_2$ (5.4 mg, 2.0 μmol, 2.0 mol%) for 67 h. Purification by flash column chromatography (SiO_2, 2% v/v acetone in $CHCl_3$) afforded the title compound as a pale-yellow oil (13.5 mg, 0.0239 mmol, 24% yield, 81% *ee*).

$[\alpha]_D^{25.0}$ = -9.84 (c. 0.85, $CHCl_3$).

Chiral SFC Analysis: CHIRALPAK IJ (CO_2:MeOH, 94:6, 2.50 mL min^{-1}, 40℃) t_R = 2.0 (minor), 2.3 (major) minutes.

2,2,3,3,3-pentafluoropropyl (S)-(2-((ethyl(phenyl)carbamoyl)oxy)-1-(o-tolyl)ethyl)sulfamate (**11m**)

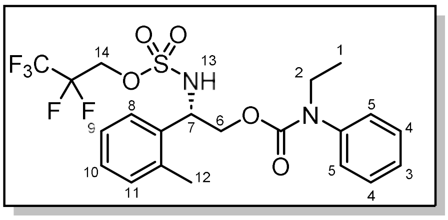

Prepared according to **GP 10** with 2-methylphenethyl ethyl(phenyl)carbamate **10m** (28.3 mg, 0.10 mmol, 1.0 equiv.) overnight. Purification by flash column chromatography (SiO_2, 2% v/v acetone in $CHCl_3$, *then* 20% v/v EtOAc in hexane) afforded the title compound as a pale-yellow oil (37.0 mg, 0.0725 mmol, 73% yield, 96% *ee*).

^{1}H NMR (700 MHz, CD_3CN): δ 7.37-7.15 (m, 9 H, H-3, H-4, H-5, H-8, H-9, H-10, H-11), 6.86 (br s, 1 H, H-13), 5.02 (br s, 1 H, H-7), 4.35-4.11 (m, 4 H, H-6, H-14), 3.66 (q, J = 7.1 Hz, 2 H, H-2), 2.32 (br s, 3 H, H-12), 1.07 (t, J = 7.1 Hz, 3 H, H-1).

^{13}C NMR (176 MHz, CD_3CN): δ 155.7, 142.6, 136.85, 136.77, 131.6, 130.0, 129.2, 128.4, 128.7, 127.43, 127.42, 119.2 (qt, $J_{C\text{-}F}$ = 286, 34.3 Hz), 112.6 (tq, $J_{C\text{-}F}$ = 255, 38.7 Hz), 67.1, 64.6 (t, $J_{C\text{-}F}$ = 28.4 Hz), 54.8, 46.3, 19.2, 13.8.

^{19}F NMR (376 MHz, CD_3CN): δ -84.4, -124.2 (m).

R_f = 0.48 (4% v/v acetone in $CHCl_3$), 0.23 (20% v/v EtOAc in hexane).

$[\alpha]_D^{25.0}$ = +26.9 (c. 0.62, $CHCl_3$).

HRMS (+ESI): *m/z* found $[M+H]^+$ 511.1319, $[C_{21}H_{24}F_5N_2O_5S]^+$ requires 511.1321, (δ = -0.4 ppm).

Chiral SFC Analysis: CHIRALPAK IJ (CO_2:MeOH, 94:6, 2.50 mL min^{-1}, 40℃) t_R = 2.5 (major), 3.0 (minor) minutes.

2,2,3,3,3-pentafluoropropyl (R)-(2-((ethyl(phenyl)carbamoyl)oxy)-1-(o-tolyl)ethyl)sulfamate (***ent*-11m**)

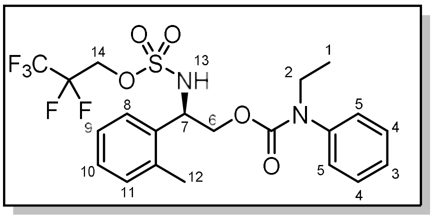

Prepared according to **GP 10** with **10m** (28.3 mg, 0.10 mmol, 1.0 equiv.) and Rh_2(**A1**)$_2$•(**C2**)$_2$•(Pyr)$_2$ (5.4 mg, 2.0 μmol, 2.0 mol%) overnight. Purification by flash column chromatography (SiO_2, 2% v/v acetone in $CHCl_3$, *then* 20% v/v EtOAc in hexane) afforded the title compound as a pale-yellow oil (23.0 mg, 0.0450 mmol, 45% yield, 93% *ee*).

[α]$_D^{25.0}$ = -24.7 (c. 0.61, $CHCl_3$).

Chiral SFC Analysis: CHIRALPAK IJ (CO_2:MeOH, 94:6, 2.50 mL min^{-1}, 40℃) t_R = 2.5 (minor), 3.0 (major) minutes.

2,2,3,3,3-pentafluoropropyl (S)-(1-(2-chlorophenyl)-2-((ethyl(phenyl)carbamoyl)oxy) ethyl)sulfamate (**11n**)

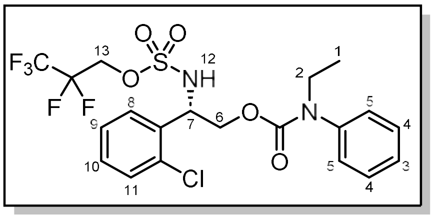

Prepared according to **GP 10** with 2-chlorophenethyl ethyl(phenyl)carbamate **10n** (30.4 mg, 0.10 mmol, 1.0 equiv.) for 66 h. Purification by flash column chromatography (SiO_2, 2% v/v acetone in $CHCl_3$) afforded the title compound as a colourless oil (18.2 mg, 0.0343 mmol, 34% yield, 85% *ee*).

^{1}H NMR (700 MHz, CD_3CN): δ 7.43-7.27 (m, 7 H, H-4, H-5, H-8, H-9, H-10, H-11), 7.15-7.00 (m, 3 H, H-3, H-12), 5.22 (br s, 1 H, H-7), 4.46-4.28 (m, 4 H, H-6, H-13), 3.65 (q, *J* = 7.1 Hz, 2 H, H-2), 1.05 (t, *J* = 7.1 Hz, 3 H, H-1).

^{13}C NMR (176 MHz, CD_3CN): δ 155.6, 142.5, 136.0, 133.4, 130.8, 130.6, 130.0, 129.8, 128.5, 128.4, 127.8, 119.2 (qt, $J_{C\text{-}F}$ = 286, 34.7 Hz), 112.6 (tq, $J_{C\text{-}F}$ = 256, 38.1 Hz), 66.5, 64.8 (t, $J_{C\text{-}F}$ = 28.0 Hz), 55.4, 46.3, 13.8.

^{19}F NMR (376 MHz, CD_3CN): δ -84.4, -124.2 (m).

R_f = 0.43 (4% v/v acetone in $CHCl_3$).

[α]$_D^{25.0}$ = +29.3 (c. 0.96, $CHCl_3$).

HRMS (+ESI): *m/z* found $[M+H]^+$ 531.0779, $[C_{20}H_{21}ClF_5N_2O_5S]^+$ requires 531.0774, (δ = +0.9 ppm).

Chiral SFC Analysis: CHIRALPAK IJ (CO_2:MeOH, 94:6, 2.50 mL min^{-1}, 40℃) t_R = 2.9 (major), 3.8 (minor) minutes.

2,2,3,3,3-pentafluoropropyl (R)-(1-(2-chlorophenyl)-2-((ethyl(phenyl)carbamoyl)oxy) ethyl)sulfamate (***ent*-11n**)

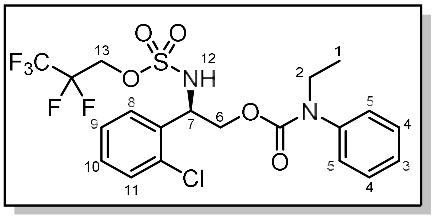

Prepared according to **GP 10** with **10n** (30.4 mg, 0.10 mmol, 1.0 equiv.) and Rh_2(**A1**)$_2$•(**C2**)$_2$•$(Pyr)_2$ (5.4 mg, 2.0 μmol, 2.0 mol%) for 67 h. Purification by flash column chromatography (SiO_2, 2% v/v acetone in $CHCl_3$) afforded the title compound as a pale-yellow oil (10.0 mg, 0.0188 mmol, 19% yield, 85% *ee*).

$[\alpha]_D^{25.0}$ = -27.4 (c. 0.59, $CHCl_3$).

Chiral SFC Analysis: CHIRALPAK IJ (CO_2:MeOH, 94:6, 2.50 mL min^{-1}, 40℃) t_R = 2.9 (minor), 3.8 (major) minutes.

2,2,3,3,3-pentafluoropropyl (S)-(1-(2-bromophenyl)-2-((ethyl(phenyl)carbamoyl)oxy) ethyl)sulfamate (**11o**)

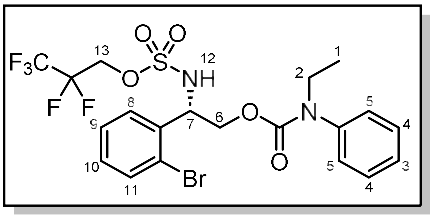

Prepared according to **GP 10** with 2-bromophenethyl ethyl(phenyl)carbamate **10o** (34.8 mg, 0.10 mmol, 1.0 equiv.) overnight. Purification by flash column chromatography (SiO_2, 2% v/v acetone in $CHCl_3$) afforded the title compound as a colourless oil (15.5 mg, 0.0270 mmol, 27% yield, 82% *ee*).

^{1}H NMR (700 MHz, CD_3CN): δ 7.61 (br d, *J* = 8.0 Hz, 1 H, H-11), 7.37-7.01 (m, 9 H, H-3, H-4, H-5, H-8, H-9, H-10, H-12), 5.21 (br s, 1 H, H-7), 4.44-4.27 (m, 4 H, H-6, H-13), 3.65 (q, *J* = 7.1 Hz, 2 H, H-2), 1.05 (t, *J* = 7.1 Hz, 3 H, H-1).

^{13}C NMR (176 MHz, CD_3CN): δ 155.6, 142.5, 137.7, 134.0, 131.1, 130.0, 129.9, 129.1, 128.4, 127.8, 123.6, 119.2 (qt, $J_{C\text{-}F}$ = 286, 34.8 Hz), 112.6 (tq, $J_{C\text{-}F}$ = 255, 37.9 Hz), 66.6, 64.8 (t, $J_{C\text{-}F}$ = 28.0 Hz), 57.8, 46.3, 13.8.

^{19}F NMR (376 MHz, CD_3CN): δ -84.4, -124.1 (m).

R_f = 0.41 (4% v/v acetone in $CHCl_3$).

$[\alpha]_D^{25.0}$ = +21.2 (c. 0.58, $CHCl_3$).

HRMS (+ESI): *m/z* found $[M+H]^+$ 575.0274, $[C_{20}H_{21}BrF_5N_2O_5S]^+$ requires 575.0269, (δ = +0.9 ppm).

Chiral SFC Analysis: CHIRALPAK IJ (CO_2:MeOH, 94:6, 2.50 mL min^{-1}, 40℃) t_R = 3.3 (major), 4.4 (minor) minutes.

2,2,3,3,3-pentafluoropropyl (R)-(1-(2-bromophenyl)-2-((ethyl(phenyl)carbamoyl)oxy) ethyl)sulfamate (***ent*-11o**)

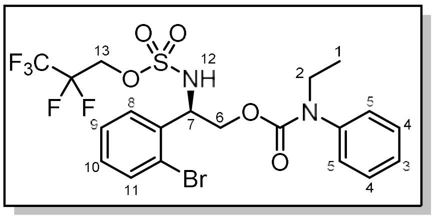

Prepared according to **GP 10** with **10o** (34.8 mg, 0.10 mmol, 1.0 equiv.) and Rh_2(**A1**)$_2$•(**C2**)$_2$•(Pyr)$_2$ (5.4 mg, 2.0 μmol, 2.0 mol%) for 67 h. Purification by flash column chromatography (SiO_2, 2% v/v acetone in $CHCl_3$) afforded the title compound as a pale-yellow oil (8.0 mg, 0.0139 mmol, 14% yield, 83% *ee*).

$[\alpha]_D^{25.0}$ = -27.1 (c. 0.52, $CHCl_3$).

Chiral SFC Analysis: CHIRALPAK IJ (CO_2:MeOH, 94:6, 2.50 mL min^{-1}, 40℃) t_R = 3.3 (minor), 4.3 (major) minutes.

2,2,3,3,3-pentafluoropropyl (S)-(2-((ethyl(phenyl)carbamoyl)oxy)-1-(thiophen-2-yl)ethyl)sulfamate (**11p**)

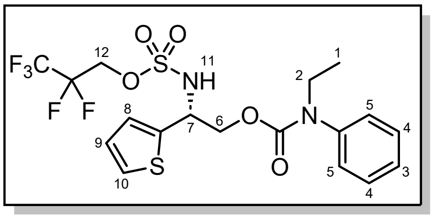

Prepared according to **GP 10** with 2-(thiophen-2-yl)ethyl ethyl(phenyl)carbamate **10p** (27.5 mg, 0.10 mmol, 1.0 equiv.) overnight. Purification by flash column chromatography (SiO_2, 2% v/v acetone in $CHCl_3$) afforded the title compound as a pale-yellow oil (20.6 mg, 0.0410 mmol, 41% yield, 98% *ee*).

^{1}H NMR (700 MHz, CD_3CN): δ 7.39-7.35 (m, 3 H, H-4, H-10), 7.29-7.26 (m, 1 H, H-3), 7.19 (br d, J = 5.7 Hz, 2 H, H-5), 7.04-7.00 (m, 2 H, H-8, H-9), 6.85 (br s, 1 H, H-11), 5.03 (br s, 1 H, H-7), 4.47-4.27 (m, 4 H, H-6, H-12), 3.68 (q, J = 7.1 Hz, 2 H, H-2), 1.08 (t, J = 7.1 Hz, 3 H, H-1).

^{13}C NMR (176 MHz, CD_3CN): δ 155.6, 142.6, 141.0, 130.0, 128.4, 128.1, 127.7, 127.5, 127.0, 119.2 (qt, $J_{C\text{-}F}$ = 286, 34.8 Hz), 112.7 (tq, $J_{C\text{-}F}$ = 255, 38.1 Hz), 67.7, 64.8 (t, $J_{C\text{-}F}$ = 27.9 Hz), 54.4, 46.3, 13.8.

^{19}F NMR (376 MHz, CD_3CN): δ -84.3, -124.1 (m).

R_f = 0.42 (4% v/v acetone in $CHCl_3$).

[α]$_D$$^{25.0}$ = +28.2 (c. 0.59, $CHCl_3$).

HRMS (+ESI): *m/z* found $[M+H]^+$ 503.0738, $[C_{18}H_{20}F_5N_2O_5S_2]^+$ requires 503.0728, (*δ* = +2.0 ppm).

Chiral SFC Analysis: CHIRALPAK IJ (CO_2:MeOH, 94:6, 2.50 mL min^{-1}, 40℃) t_R = 3.0 (major), 3.7 (minor) minutes.

2,2,3,3,3-pentafluoropropyl (R)-(2-((ethyl(phenyl)carbamoyl)oxy)-1-(thiophen-2-yl)ethyl)sulfamate (***ent*-11p**)

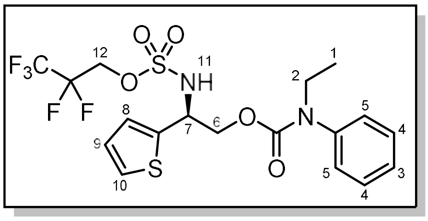

Prepared according to **GP 10** with **10p** (27.5 mg, 0.100 mmol, 1.0 equiv.) and Rh_2(**A1**)$_2$•(**C2**)$_2$•(Pyr)$_2$ (5.4 mg, 2.0 µmol, 2.0 mol%) overnight. Purification by flash column chromatography (SiO_2, 2% v/v acetone in $CHCl_3$) afforded the title compound as a pale-yellow oil (20.6 mg, 0.0410 mmol, 41% yield, 89% *ee*).

[α]$_D$$^{25.0}$ = -22.4 (c. 0.67, $CHCl_3$).

Chiral SFC Analysis: CHIRALPAK IJ (CO_2:MeOH, 94:6, 2.50 mL min^{-1}, 40℃) t_R = 3.0 (minor), 3.7 (major) minutes.

2,2,3,3,3-pentafluoropropyl (S)-(2-((ethyl(phenyl)carbamoyl)oxy)-1-(thiophen-3-yl)ethyl)sulfamate (**11q**)

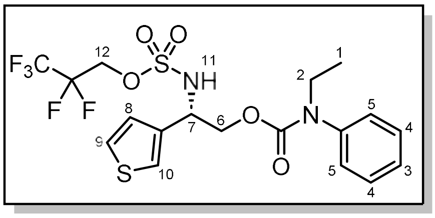

Prepared according to **GP 10** with 2-(thiophen-3-yl)ethyl ethyl(phenyl)carbamate **10q** (27.5 mg, 0.10 mmol, 1.0 equiv.) overnight. Purification by flash column chromatography (SiO_2, 2% v/v acetone in $CHCl_3$) afforded the title compound as a pale-yellow oil (21.3 mg, 0.0424 mmol, 42% yield, 94% *ee*).

^{1}H NMR (700 MHz, CD_3CN): *δ* 7.40-7.26 (m, 5 H, H-3, H-4, H-9, H-10), 7.17 (br d, *J* = 6.8 Hz, 2 H, H-5), 7.02 (br s, 1 H, H-8), 6.75 (br s, 1 H, H-11), 4.84 (br s, 1 H, H-7), 4.43-4.22 (m, 4 H, H-6, H-12), 3.66 (q, *J* = 7.1 Hz, 2 H, H-2), 1.06 (t, *J* = 7.1 Hz, 3 H, H-1).

^{13}C NMR (176 MHz, CD_3CN): *δ* 155.6, 142.6, 139.3, 130.0, 128.4, 127.8, 127.7, 127.3, 124.5, 119.2 (qt, J_{C-F} = 285, 34.6 Hz), 112.7 (tq, J_{C-F} = 256, 38.2 Hz), 67.4, 64.7 (t, J_{C-F} = 27.9 Hz), 54.6, 46.2, 13.8.

^{19}F NMR (376 MHz, CD_3CN): δ -84.4, -124.1 (m).

R_f = 0.38 (4% v/v acetone in $CHCl_3$).

$[\alpha]_D^{25.0}$ = +28.3 (c. 0.77, $CHCl_3$).

HRMS (+ESI): *m/z* found $[M+H]^+$ 503.0739, $[C_{18}H_{20}F_5N_2O_5S_2]^+$ requires 503.0728, (δ = +2.0 ppm).

Chiral SFC Analysis: CHIRALPAK IJ (CO_2:MeOH, 94:6, 2.50 mL min^{-1}, 40℃) t_R = 3.2 (major), 3.9 (minor) minutes.

2,2,3,3,3-pentafluoropropyl (R)-(2-((ethyl(phenyl)carbamoyl)oxy)-1-(thiophen-3-yl)ethyl)sulfamate (***ent*-11q**)

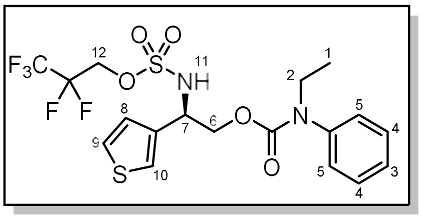

Prepared according to **GP 10** with **10q** (27.5 mg, 0.10 mmol, 1.0 equiv.) and $Rh_2(\mathbf{A1})_2\bullet(\mathbf{C2})_2\bullet(Pyr)_2$ (5.4 mg, 2.0 μmol, 2.0 mol%) overnight. Purification by flash column chromatography (SiO_2, 2% v/v acetone in $CHCl_3$) afforded the title compound as a pale-yellow oil (17.8 mg, 0.0354 mmol, 35% yield, 90% *ee*).

$[\alpha]_D^{25.0}$ = -28.7 (c. 1.00, $CHCl_3$).

Chiral SFC Analysis: CHIRALPAK IJ (CO_2:MeOH, 94:6, 2.50 mL min^{-1}, 40℃) t_R = 3.2 (minor), 3.9 (major) minutes.

2,2,3,3,3-pentafluoropropyl ((1S,2S)-2-((ethyl(phenyl)carbamoyl)oxy)-2,3-dihydro-1H-inden-1-yl)sulfamate (**11r**)

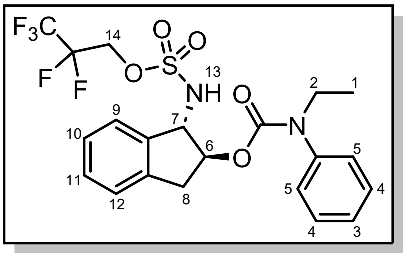

Prepared according to **GP 10** with 2,3-dihydro-1*H*-inden-2-yl ethyl(phenyl)carbamate **10r** (28.1 mg, 0.10 mmol, 1.0 equiv.) overnight. Purification by flash column chromatography (SiO_2, 3% v/v acetone in $CHCl_3$) afforded the title compound as a colourless oil (39.7 mg, 0.0781 mmol, 78% yield, 72% *ee*).

^{1}H NMR (500 MHz, CD_3CN): δ 7.39-7.23 (m, 9 H, H-3, H-4, H-5, H-9, H-10, H-11, H-12), 6.64 (br s, 1 H, H-13), 5.34 (dt, *J* = 7.1, 5.5 Hz, 1 H, H-6), 4.88 (br s, 1 H, H-7), 4.66 (t, *J* = 12.9 Hz, 2 H, H-14), 3.75-3.64 (m, 2 H, H-2), 3.42 (dd, *J* = 16.5, 7.1 Hz, 1 H, H-8a), 2.85 (br d, *J* = 11.9 Hz, 1 H, H-8b), 1.10 (t, *J* = 7.1 Hz, 3 H, H-1).

^{13}C NMR (126 MHz, CD_3CN): δ 155.5, 142.7, 140.7, 139.2, 130.2, 129.9, 128.4, 128.2, 127.5, 126.0, 125.6, 119.4 (qt, J_{C-F} = 286, 34.6 Hz), 113.0 (tq, J_{C-F} = 255, 38.1 Hz), 81.6, 65.1 (t, J_{C-F} = 27.7 Hz), 64.9, 46.2, 37.2, 13.9.

^{19}F NMR (376 MHz, CD_3CN): δ -84.2, -123.9 (m).

R_f = 0.29 (4% v/v acetone in $CHCl_3$).

$[\alpha]_D^{25.0}$ = +51.7 (c. 0.99, $CHCl_3$).

HRMS (+ESI): *m/z* found $[M+H]^+$ 509.1168, $[C_{21}H_{22}F_5N_2O_5S]^+$ requires 509.1164, (δ = +0.8 ppm).

Chiral SFC Analysis: CHIRALPAK IJ (CO_2:MeOH, 94:6, 2.50 mL min^{-1}, 40 ℃) t_R = 3.2 (major), 3.5 (minor) minutes.

2,2,3,3,3-pentafluoropropyl ((1R,2R)-2-((ethyl(phenyl)carbamoyl)oxy)-2,3-dihydro-1H-inden-1-yl)sulfamate (***ent*-11r**)

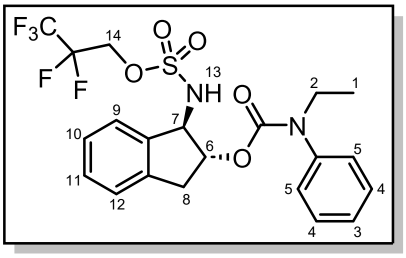

Prepared according to **GP 10** with **10r** (28.1 mg, 0.10 mmol, 1.0 equiv.) and Rh_2(**A1**)$_2$•(**C2**)$_2$•(Pyr)$_2$ (5.4 mg, 2.0 μmol, 2.0 mol%) overnight. Purification by flash column chromatography (SiO_2, 3% v/v acetone in $CHCl_3$) afforded the title compound as a colourless oil (43.3 mg, 0.0852 mmol, 85% yield, 71% *ee*).

$[\alpha]_D^{25.0}$ = -50.7 (c. 0.89, $CHCl_3$).

Chiral SFC Analysis: CHIRALPAK IJ (CO_2:MeOH, 94:6, 2.50 mL min^{-1}, 40 ℃) t_R = 3.2 (minor), 3.5 (major) minutes.

2,2,3,3,4,4,4-heptafluorobutyl (S)-(2-((methyl(phenyl)carbamoyl)oxy)-1-phenylethyl)sulfamate (**S5a**)

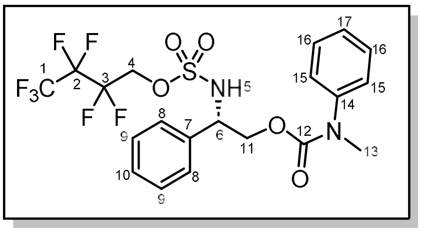

Prepared according to **GP 10** with phenethyl methyl(phenyl)carbamate **S4** (26.8 mg, 0.105 mmol, 1.0 equiv.), 2,2,3,3,4,4,4-heptafluorobutyl sulfamate **2a** (33.5 mg, 0.12 mmol, 1.2 equiv.) and Rh_2(**A1**)$_2$•(**B1**)$_2$•(Pyr)$_2$ (2.7 mg, 1.0 μmol, 1.0 mol%) overnight. Purification by flash column chromatography (SiO_2, 0-7% v/v acetone in $CHCl_3$) afforded the title compound as a white solid (13.8 mg, 0.0259 mmol, 25% yield, 92% *ee*, 95% pure with **S6a** as an inseparable impurity).

^{1}H NMR (700 MHz, CD_3CN): δ 7.38-7.32 (m, 7H, H-8, H-9, H-10, H-16), 7.25-7.21 (m, 3H, H-15, H-17), 6.88 (br s, 1H, H-5), 4.73 (br s, 1H, H-6), 4.41 (q, J = 13.1 Hz, 1H, H-4a), 4.30-4.25 (m, 3H, H-4b, H-11a, H-11b), 3.23 (s, 3H, H-13).

^{13}C NMR (176 MHz, CD_3CN): δ 155.9, 144.2, 138.6, 129.9, 129.7, 129.3, 128.1, 127.2, 126.9, 118.4 (qt, $J_{C\text{-}F}$ = 286.8, 33.2 Hz), 114.5 (tt, $J_{C\text{-}F}$ = 257.6, 31.6 Hz), 111.9-107.6 (m), 68.0, 64.9 (t, $J_{C\text{-}F}$ = 27.2 Hz), 58.9, 38.2.

^{19}F NMR (376 MHz, CD_3CN): δ -82.7 (t, J = 9.5 Hz), -122.1 - -122.2 (m), -128.9 - -129.0 (m).

R_f = 0.48 (10% v/v acetone in $CHCl_3$).

HRMS (-ESI): *m/z* found $[M-H]^-$ 531.0830, $[C_{20}H_{18}F_7N_2O_5S]^-$ requires 531.0830, (δ = 0.0 ppm).

Chiral SFC Analysis: CHIRALPAK IJ (CO_2:MeOH, 94:6, 2.50 mL min^{-1}, 40 ˚C), t_R = 2.8 (minor), 3.1 (major) minutes.

2,2,3,3,4,4,4-heptafluorobutyl (R)-(2-((methyl(phenyl)carbamoyl)oxy)-1-phenylethyl)sulfamate (***ent*-S5a**)

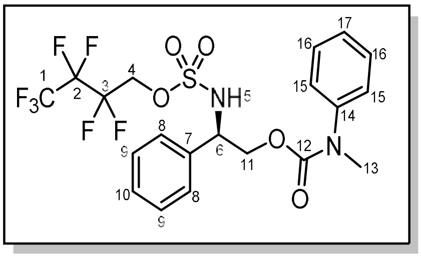

Prepared according to **GP 10** with phenethyl methyl(phenyl)carbamate **S4** (27.8 mg, 0.109 mmol, 1.0 equiv.) and Rh_2(**A1**)$_2$•(**C2**)$_2$•(Pyr)$_2$ (2.7 mg, 1.0 µmol, 1.0 mol%) overnight. Purification by flash column chromatography (SiO_2, 0-7% v/v acetone in $CHCl_3$) afforded the title compound as a white solid (13.0 mg, 0.0244 mmol, 22% yield, 85% *ee*, 95% pure with **S6a** as an inseparable impurity).

Chiral SFC Analysis: CHIRALPAK IJ (CO_2:MeOH, 94:6, 2.50 mL min^{-1}, 40 ˚C), t_R = 2.8 (major), 3.1 (minor) minutes.

2,2,3,3,4,4,4-heptafluorobutyl (((phenethoxycarbonyl)(phenyl)amino)methyl)sulfamate (**S6a**)

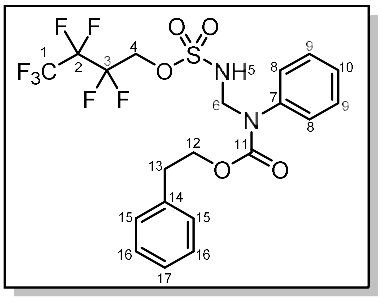

Prepared according to **racemic** version of **GP 10** with phenethyl methyl(phenyl)carbamate **S4** (26.8 mg, 0.105 mmol, 1.0 equiv.) and 2,2,3,3,4,4,4-heptafluorobutyl sulfamate **2a** (33.5 mg, 0.12 mmol, 1.2 equiv.) overnight. Purification by flash column chromatography (SiO_2, 0-7% v/v acetone in $CHCl_3$) afforded the title compound as a white solid (7.8 mg, 0.0146 mmol, 14% yield).

^{1}H NMR (700 MHz, CD_3CN): *δ* 7.38 (t, *J* = 7.9 Hz, 2H, H-9), 7.33-7.30 (m, 1H, H-10), 7.27-7.23 (m, 2H, H-16), 7.22-7.19 (m, 3H, H-8, H-17), 7.11 (br s, 2 H, H-15), 6.81 (br s, 1H, H-5), 4.95 (s, 2H, H-6), 4.54 (t, *J* = 13.6 Hz, 2H, H-4), 4.28-4.24 (m, 2H, H-12), 2.90-2.80 (m, 2H, H-13).

^{13}C NMR (176 MHz, CD_3CN): *δ* 155.9, 141.4, 139.3, 130.02, 129.98, 129.4, 128.7, 128.3, 127.4, 67.6, 65.1 (t, $J_{C\text{-}F}$ = 27.1 Hz), 59.9, 35.7.

^{19}F NMR (376 MHz, $CDCl_3$): *δ* -81.7 (t, *J* = 9.2 Hz), -121.5 - -121.6 (m), -128.25 - -128.33 (m).

R_f = 0.54 (10% v/v acetone in $CHCl_3$).

HRMS (-ESI): *m/z* found $[M-H]^-$ 531.0834, $[C_{20}H_{18}F_7N_2O_5S]^-$ requires 531.0830, (*δ* = +0.8 ppm).

2,2,3,3,3-pentafluoropropyl (1-((phenethoxycarbonyl)(phenyl)amino)ethyl)sulfamate (**S7**)

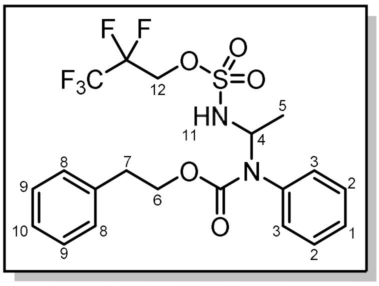

Prepared according to **racemic** version of **GP 10** with phenethyl ethyl(phenyl)carbamate **10a** (53.8 mg, 0.20 mmol, 1.0 equiv.) and $Rh_2(esp)_2$ (5 mol%) overnight. Purification by flash column chromatography (SiO_2, 2% v/v acetone in $CHCl_3$) afforded the title compound as a pale-yellow oil (40.4 mg, 0.0814 mmol, 41% yield).

^{1}H NMR (700 MHz, CD_3CN): δ 7.42-7.37 (m, 3 H, H-1, H-2), 7.24-7.17 (m, 5 H, H-3, H-9, H-10), 7.04 (br s, 2 H, H-8), 6.69 (br s, 1 H, H-11), 5.76-5.73 (m, 1 H, H-4), 4.67-4.58 (m, 2 H, H-12), 4.20 (br s, 2 H, H-6), 2.79 (br s, 2 H, H-7), 1.37 (d, J = 6.7 Hz, 3 H, H-5).

^{13}C NMR (176 MHz, CD_3CN): δ 155.5, 139.4, 138.6, 130.7, 130.01, 129.96, 129.3, 129.0, 127.3, 119.4 (qt, J_{C-F} = 285, 34.6 Hz), 112.9 (tq, J_{C-F} = 255, 38.2 Hz), 67.3, 65.7 65.1 (t, J_{C-F} = 27.7 Hz), 35.8, 20.6.

^{19}F NMR (376 MHz, CD_3CN): δ -84.2, -124.1 (m).

R_f = 0.56 (4% v/v acetone in $CHCl_3$).

HRMS (+ESI): *m/z* found $[M+Na]^+$ 519.0991, $[C_{20}H_{21}F_5N_2O_5SNa]^+$ requires 519.0984, (δ = +1.3 ppm).

Chiral reaction using $Rh_2(\textbf{A1})_2$•$(\textbf{B1})_2$•$(Pyr)_2$

Prepared according to **GP 10** with phenethyl ethyl(phenyl)carbamate **10a** (26.9 mg, 0.10 mmol, 1.0 equiv.) overnight. Purification by flash column chromatography (SiO_2, 2% v/v acetone in $CHCl_3$) afforded the title compound as pale-yellow oil (3.1 mg, 0.00624 mmol, 6% yield, 38% *ee*).

Chiral SFC Analysis: CHIRALPAK IC (CO_2:MeOH, 97:3, 2.50 mL min^{-1}, 40℃) t_R = 3.2 (minor), 3.5 (major) minutes.

Kinetic Resolution of (*rac*)-10s

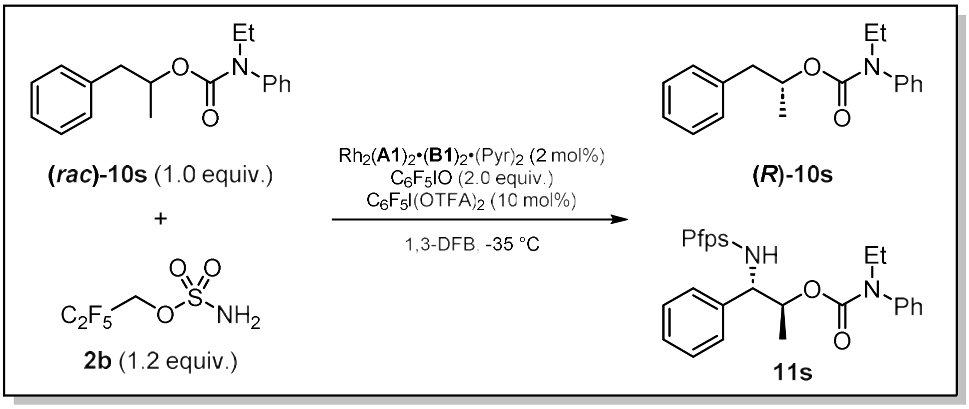

Prepared according to **GP 10** with 1-phenylpropan-2-yl ethyl(phenyl)carbamate **(*rac*)-10s** (28.3 mg, 0.10 mmol, 1.0 equiv.) overnight. Purification by flash column chromatography (SiO_2, 2% v/v acetone in $CHCl_3$, *then* 10% v/v EtOAc in hexane) recovered (*R*)-1-phenylpropan-2-yl ethyl(phenyl)carbamate **(*R*)-10s** (13.2 mg, 0.0466 mmol, 47% yield, 38% *ee*) and afforded the benzylic aminated product **11s** as a colourless oil (13.4 mg, 0.0262 mmol, 26% yield, 99% *ee*).

(R)-1-phenylpropan-2-yl ethyl(phenyl)carbamate (**(*R*)-10s**)

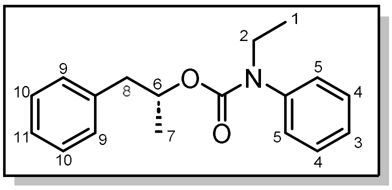

R_f = 0.68 (4% v/v acetone in $CHCl_3$), 0.26 (10% EtOAc in hexane)

$[\alpha]_D^{25.0}$ = -13.9 (c. 0.88, $CHCl_3$).

Chiral SFC Analysis: CHIRALPAK IJ (CO_2:MeOH, 94:6, 2.50 mL min^{-1}, 40 ˚C), t_R = 2.9 (major), 3.3 (minor) minutes.

2,2,3,3,3-pentafluoropropyl ((1S,2S)-2-((ethyl(phenyl)carbamoyl)oxy)-1-phenylpropyl)sulfamate (**11s**)

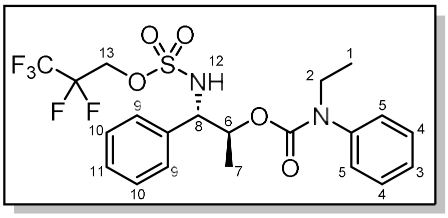

^{1}H NMR (700 MHz, CD_3CN): δ 7.41-7.21 (m, 10 H, H-3, H-4, H-5, H-9, H-10, H-11), 6.82 (br s, 1 H, H-12), 5.04 (p, J = 6.5 Hz, 1 H, H-6), 4.45 (br s, 1 H, H-8), 4.27 (q, J = 12.6 Hz, 1 H, H-13a), 4.05 (q, J = 12.6 Hz, 1 H, H-13b), 3.75-3.65 (m, 2 H, H-2), 1.10-1.08 (m, 6 H, H-1, H-7).

^{13}C NMR (176 MHz, CD_3CN): δ 155.6, 142.7, 139.1, 130.0, 129.7, 129.3, 128.5, 128.4, 127.7, 119.1 (qt, $J_{C\text{-}F}$ = 286, 34.7 Hz), 112.6 (tq, $J_{C\text{-}F}$ = 255, 38.2 Hz), 74.1, 64.5 (t, $J_{C\text{-}F}$ = 28.0 Hz), 63.8, 46.1, 17.7, 13.9.

^{19}F NMR (376 MHz, CD_3CN): δ -84.5, -124.3 (m).

R_f = 0.42 (4% v/v acetone in $CHCl_3$).

$[\alpha]_D^{25.0}$ = +24.4 (c. 0.78, $CHCl_3$).

HRMS (+ESI): *m/z* found $[M+H]^+$ 511.1324, $[C_{21}H_{24}F_5N_2O_5S]^+$ requires 511.1321, (δ = +0.6 ppm).

Chiral SFC Analysis: CHIRALPAK IJ (CO_2:MeOH, 94:6, 2.50 mL min^{-1}, 40℃) t_R = 2.0 (major), 2.5 (minor) minutes.

2,2,3,3,3-pentafluoropropyl ((1R,2R)-2-((ethyl(phenyl)carbamoyl)oxy)-1-phenylpropyl)sulfamate (***ent*-11s**)

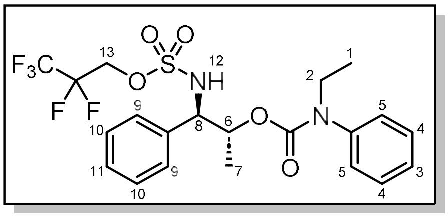

Prepared according to **GP 10** with 1-phenylpropan-2-yl ethyl(phenyl)carbamate **(*rac*)-10s** (84.9 mg, 0.30 mmol, 1.0 equiv.) and Rh_2(**A1**)$_2$•(**C2**)$_2$•(Pyr)$_2$ (16.2 mg, 6.0 µmol, 2.0 mol%) overnight. Purification by flash column chromatography (SiO_2, 2% v/v acetone in $CHCl_3$) afforded the benzylic aminated product ***ent*-11s** as a pale-yellow oil (9.7 mg, 0.0190 mmol, 6% yield, 97% *ee*).

$[\alpha]_D^{25.0}$ = -30.2 (c. 0.62, $CHCl_3$).

Chiral SFC Analysis: CHIRALPAK IJ (CO_2:MeOH, 94:6, 2.50 mL min^{-1}, 40℃) t_R = 1.9 (minor), 2.5 (minor) minutes.

Note: The relative stereochemistry of ***11s*** *was assigned by derivatising* ***11s*** *to the cyclised compound* ***S8*** *(see below). The absolute stereochemistry of* ***11s*** *was tentatively assigned in analogy to the other C-H amination products obtained.*

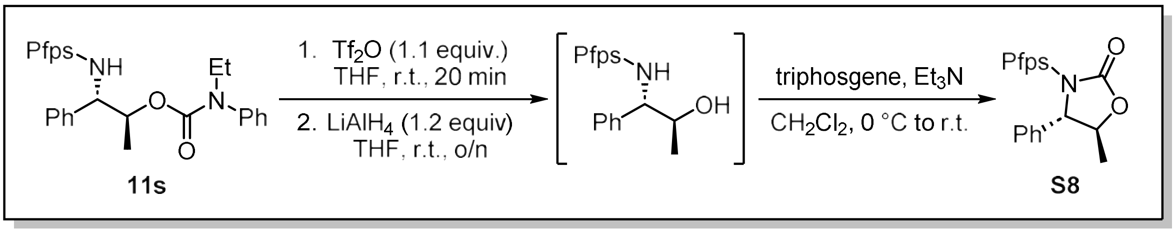

Prepared according to the modified protocol reported by Barbe and Charette.[37] A crimp-top vial was charged with **11s** (51.9 mg, 0.116 mmol, 99% *ee*, 1.0 equiv.). The vial was sealed and evacuated and backfilled with nitrogen three times, following which dry THF (10 mL) and trifluoromethanesulfonic anhydride (0.022 mL, 0.128 mmol, 1.1 equiv.) were added at room temperature. The reaction mixture was stirred for 20 min, and $LiAlH_4$ (0.058 mL, 2.4 M in THF, 0.139 mmol, 1.2 equiv.) was added to the mixture. The resulting mixture was stirred overnight at room temperature and quenched with sodium sulfate decahydrate until effervescence had subsided. The resulting mixture was diluted with EtOAc and filtered through Celite®, eluting with copious amounts of EtOAc and $CHCl_3$. The filtrate was collected and the solvent removed under reduced pressure. Purification by flash column chromatography (SiO_2, 30% v/v EtOAc in hexane) afforded the alcohol intermediate with some impurities as a colourless oil (25.0 mg) and was taken forward to the next step without further purifications.

A crimp-top vial was charged with the alcohol intermediate and sealed. The vial was evacuated and backfilled with nitrogen three times, following which anhydrous CH_2Cl_2 (5.0 mL) and Et_3N (0.024 mL, 0.172 mmol) were added at room temperature. After the solution was cooled to 0 ˚C, triphosgene (22.5 mg, 0.0757 mmol) in dry CH_2Cl_2 (5 mL) was added to the mixture and the reaction mixture was warmed to room temperature and stirred overnight. The reaction mixture was quenched with aqueous, saturated $NaHCO_3$ solution and diluted with CH_2Cl_2 and water. The aqueous layer was extracted thrice with CH_2Cl_2, and the combined organic layers were dried over Na_2SO_4, filtered and concentrated. Purification by flash column chromatography (SiO_2, 20% v/v EtOAc in hexane) afforded the cyclised compound **S8** as a colourless oil (20.6 mg, 0.0529 mmol, 46% yield over two steps).

2,2,3,3,3-pentafluoropropyl (4S,5S)-5-methyl-2-oxo-4-phenyloxazolidine-3-sulfonate (**S8**)

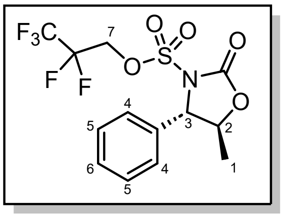

^{1}H NMR (700 MHz, CD_3CN): *δ* 7.48-7.42 (m, 5 H, H-4, H-5, H-6), 5.03 (d, *J* = 5.7 Hz, 1 H, H-3), 4.86-4.78 (m, 2 H, H-7), 4.67 (p, *J* = 6.2 Hz, 1 H, H-2), 1.51 (d, *J* = 6.2 Hz, 3 H, H-1).

^{13}C NMR (176 MHz, CD$_3$CN): δ 152.3, 137.9, 130.5, 130.2, 128.1, 119.1 (qt, $J_{C\text{-}F}$ = 286, 34.4 Hz), 112.3 (tq, $J_{C\text{-}F}$ = 256, 38.4 Hz), 81.7, 69.1, 67.9 (t, $J_{C\text{-}F}$ = 27.9 Hz), 19.3.

^{19}F NMR (376 MHz, CD$_3$CN): δ -84.3, -124.3 (m).

R_f = 0.38 (20% v/v EtOAc in hexane).

$[a]_D^{25.0}$ = +20.6 (c. 0.67, CHCl$_3$).

HRMS (+ESI): *m/z* found $[M+NH_4]^+$ 407.0699, $[C_{13}H_{16}F_5N_2O_5S]^+$ requires 407.0695, (δ = +1.0 ppm).

Note: The relative stereochemistry for this compound was tentatively assigned on the basis of ^{1}H-^{1}H NOESY experiments. Strong nOe correlations were observed between H^1 and H^3. In contrast, nOe correlations were not observed between H^1 and the aromatic protons on the phenyl ring. Therefore, we propose that the methyl substituent is anti to the phenyl ring.

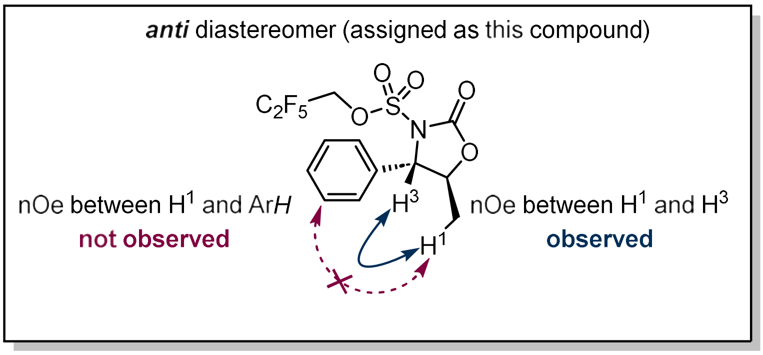

C-H amination of 10a using Rh$_2$(A1)$_2$•(B9)$_2$•(Pyr)$_2$

This reaction was carried out according to **GP 10** using the phenethyl carbamate substrate **10a**. The purification method of the product was the same as that of compound **11a**.

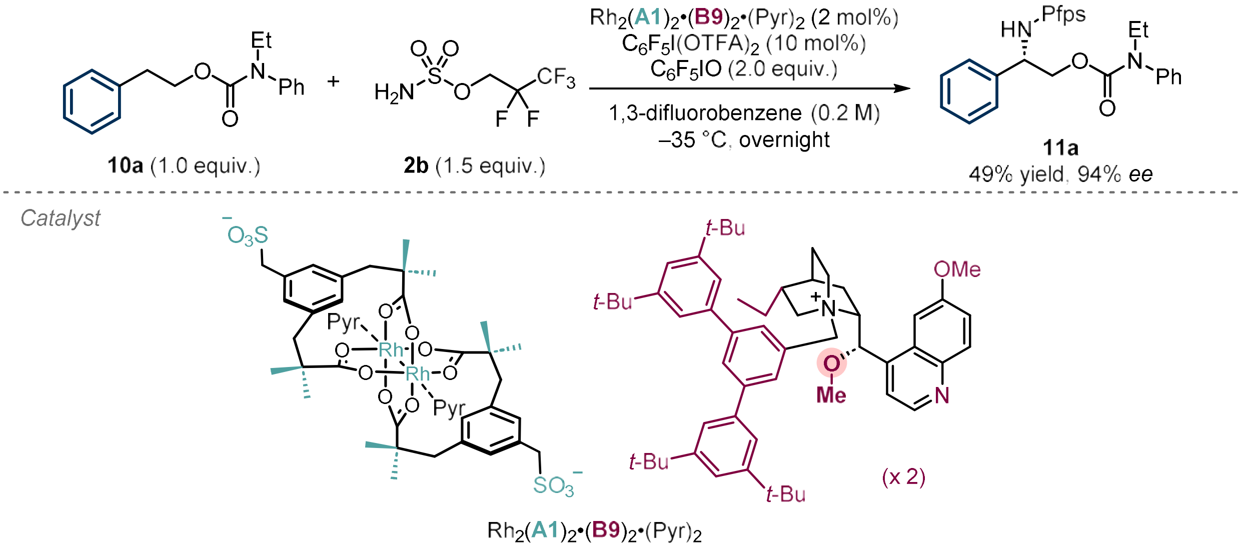

15 Synthetic Elaboration of C-H Amination Product

2,2,3,3,3-pentafluoropropyl (S)-(2-hydroxy-1-phenylethyl)sulfamate (**12**)

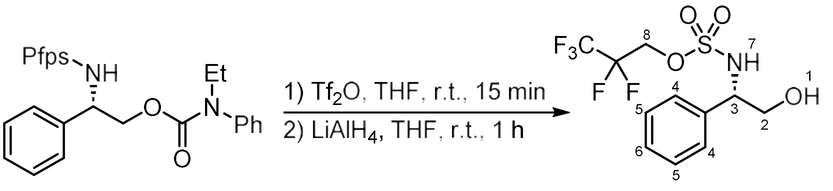

11a **12**

Prepared according to the modified protocol reported by Barbe and Charette.[37] A crimp-top vial was charged with **11a** (83.8 mg, 0.169 mmol, 96% *ee*, 1.0 equiv.). The vial was sealed and evacuated and backfilled with nitrogen three times, following which dry THF (5.0 mL) and trifluoromethanesulfonic anhydride (0.031 mL, 0.186 mmol, 1.1 equiv.) were added at room temperature. The reaction mixture was stirred for 15 min, and $LiAlH_4$ (0.085 mL, 2,4 M in THF, 0.203 mmol, 1.2 equiv.) was added to the mixture. The resulting mixture was stirred for 1 h at room temperature and quenched with sodium sulfate decahydrate until effervescence had subsided. The resulting mixture was diluted with EtOAc and filtered through Celite®, eluting with copious amounts of EtOAc and $CHCl_3$. The filtrate was collected and the solvent removed under reduced pressure. Purification by flash column chromatography (SiO_2, 20% v/v acetone in $CHCl_3$) afforded title compound as a white solid (42.9 mg, 0.123 mmol, 73% yield, 96% *ee*).

^{1}H NMR (700 MHz, CD_3CN): δ 7.40-7.36 (m, 4 H, H-4, H-5), 7.34-7.31 (m, 1 H, H-6), 4.57-4.49 (m, 2 H, H-3, H-8a), 4.40-4.34 (m, 1 H, H-8b), 3.72 (dd, J = 11.5, 4.9 Hz, 1 H, H-2a), 3.64 (dd, J = 11.5, 7.7 Hz, 1 H, H-2b).

Note: The signals of H-1 (-OH) and H-7 (-NH) were not clearly observed.

^{13}C NMR (176 MHz, CD_3CN): δ 139.9, 129.6, 129.0, 128.0, 119.3 (qt, $J_{C\text{-}F}$ = 286, 34.6 Hz), 112.8 (tq, $J_{C\text{-}F}$ = 255, 38.1 Hz), 65.8, 64.7 (t, $J_{C\text{-}F}$ = 28.0 Hz), 61.8.

^{19}F NMR (376 MHz, CD_3CN): δ -84.4, -124.2 (m).

R_f = 0.41 (20% v/v acetone in $CHCl_3$).

$[\alpha]_D^{25.0}$ = +38.3 (c. 0.79, $CHCl_3$).

HRMS (-ESI): *m/z* found $[M–H]^-$ 348.0332, $[C_{11}H_{11}F_5NO_4S]^-$ requires 348.0334, (δ = -0.6 ppm).

Chiral SFC Analysis: CHIRALPAK IK (CO_2:MeOH, 94:6, 2.50 mL min^{-1}, 40℃) t_R = 2.5 (minor), 3.0 (major) minutes.

2,2,3,3,3-pentafluoropropyl (R)-(2-hydroxy-1-phenylethyl)sulfamate (***ent*-12**)

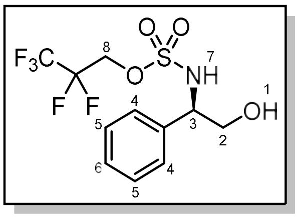

Prepared according to the above procedure with ***ent*-11a** (59.8 mg, 0.120 mmol, 93% *ee*, 1.0 equiv.). Purification by flash column chromatography (SiO_2, 20% v/v acetone in $CHCl_3$) afforded the title compound as a white solid (31.9 mg, 0.0913 mmol, 76% yield, 93% *ee*).

$[\alpha]_D^{25.0}$ = -45.4 (c. 0.55, $CHCl_3$).

Chiral SFC Analysis: CHIRALPAK IK (CO_2:MeOH, 94:6, 2.50 mL min^{-1}, 40 ˚C), t_R = 2.6 (major), 3.0 (minor) minutes.

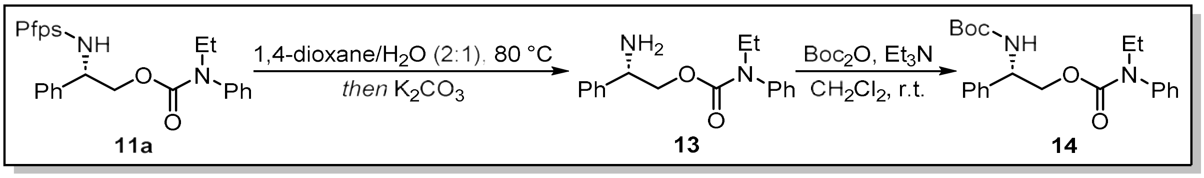

Prepared according to the protocol reported by Phipps and co-workers.[38] A crimp-top vial was charged with **11a** (71.4 mg, 0.144 mmol, 96% *ee*, 1.0 equiv.). The vial was sealed and evacuated and backfilled with nitrogen three times, following which 1,4-dioxane (1.5 mL) and water (0.75 mL) were added. The reaction mixture was heated at 80 ˚C for 20 h. After cooling down to room temperature, potassium carbonate (207 mg, 1.5 mmol) was added to the reaction mixture. The resulting mixture was stirred for 1 h at room temperature and the mixture was diluted with water and EtOAc. The aqueous layer was extracted thrice with EtOAc and the combined organic layers were dried over $MgSO_4$, filtered and concentrated. Purification by flash column chromatography (SiO_2, 20% v/v acetone in $CHCl_3$) afforded **13** as a pale-yellow oil (38.3 mg, 0.135 mmol, 94% yield).

A crimp-top vial was charged with **13** (38.3 mg, 0.135 mmol, 1.0 equiv.). The vial was sealed and evacuated and backfilled with nitrogen three times, following which dry CH_2Cl_2 (1.0 mL), triethylamine (0.028 mL, 0.203 mmol, 1.5 equiv.) and di-*tert*-butyl decarbonate (0.037 mL, 0.162 mmol, 1.2 equiv.) were added at room temperature. The reaction mixture was stirred for 18 h and the reaction mixture was diluted with water and EtOAc. The aqueous layer was extracted thrice with EtOAc and the combined organic layers were dried over $MgSO_4$, filtered and concentrated. Purification by flash column chromatography (SiO_2, 20% v/v EtOAc in hexane) afforded **14** as a white solid (46.1 mg, 0.120 mmol, 89% yield, 96% *ee*).

(S)-2-amino-2-phenylethyl ethyl(phenyl)carbamate (**13**)

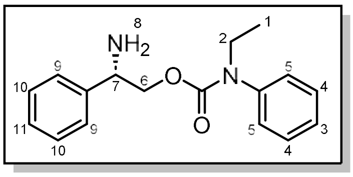

^{1}H NMR (700 MHz, CD_3CN): δ 7.38-7.35 (m, 2 H, H-4), 7.31-7.24 (m, 6 H, H-3, H-9, H-10, H-11), 7.17 (br d, J = 7.7 Hz, 2 H, H-5), 4.12-4.01 (m, 3 H, H-6, H-7), 3.64 (q, J = 7.1 Hz, 2 H, H-2), 1.84 (br s, 2 H, H-8), 1.06 (t, J = 7.1 Hz, 3 H, H-1).

^{13}C NMR (176 MHz, CD_3CN): δ 155.7, 144.0, 142.9, 129.9, 129.2, 128.5, 128.1, 127.9, 127.5, 71.5, 55.5, 46.0, 13.9.

R_f = 0.27 (20% v/v acetone in $CHCl_3$).

$[\alpha]_D^{25.0}$ = +28.8 (c. 1.04, $CHCl_3$).

HRMS (+ESI): *m/z* found $[M+H]^+$ 285.1601, $[C_{17}H_{21}N_2O_2]^+$ requires 285.1598, (δ = +1.1 ppm).

(S)-2-((tert-butoxycarbonyl)amino)-2-phenylethyl ethyl(phenyl)carbamate (**14**)

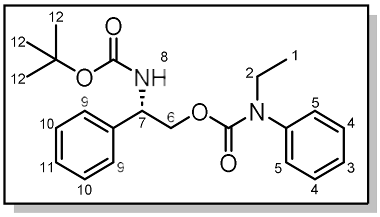

^{1}H NMR (700 MHz, CD_3CN): δ 7.38-7.35 (m, 2 H, H-4), 7.32 (br t, J = 7.3 Hz, 2 H, H-10), 7.29-7.24 (m, 4 H, H-3, H-9, H-11), 7.17 (br d, J = 7.1 Hz, 2 H, H-5), 5.87-5.53 (m, 1 H, H-5), 4.83 (br s, 1 H, H-7), 4.21-4.15 (m, 2 H, H-6), 3.65 (q, J = 7.1 Hz, 2 H, H-2), 1.40-1.28 (m, 9 H, H-12), 1.06 (t, J = 7.1 Hz, 3 H, H-1).

^{13}C NMR (176 MHz, CD_3CN): δ 156.2, 155.8, 142.8, 141.0, 129.9, 129.4, 128.5, 128.4, 127.7, 127.6, 79.7, 68.3, 55.1, 46.2, 28.6, 13.9.

R_f = 0.31 (20% v/v EtOAc in hexane).

$[\alpha]_D^{25.0}$ = +21.2 (c. 0.87, $CHCl_3$).

HRMS (+ESI): *m/z* found $[M+Na]^+$ 407.1947, $[C_{22}H_{28}N_2O_4Na]^+$ requires 407.1941, (δ = +1.5 ppm).

Chiral SFC Analysis: CHIRALPAK IJ (CO_2:MeOH, 94:6, 2.50 mL min^{-1}, 40℃) t_R = 3.0 (major), 4.7 (minor) minutes.

(R)-2-((tert-butoxycarbonyl)amino)-2-phenylethyl ethyl(phenyl)carbamate (***ent*-14**)

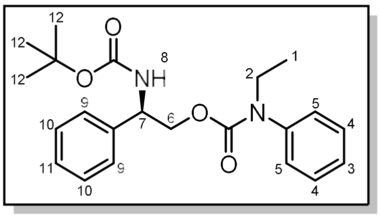

Prepared according to the above procedure with ***ent*-11a** (60.6 mg, 0.122 mmol, 93% *ee*, 1.0 equiv.). Purification by flash column chromatography (SiO_2, 20% v/v EtOAc in hexane) afforded the title compound as a white solid (25.5 mg, 0.119 mmol, 54% yield over two steps, 93% *ee*).

$[\alpha]_D^{25.0}$ = -21.0 (c. 0.75, $CHCl_3$).

Chiral SFC Analysis: CHIRALPAK IJ (CO_2:MeOH, 94:6, 2.50 mL min^{-1}, 40 ˚C), t_R = 3.0 (minor), 4.7 (major) minutes.

16 Determination of Absolute Stereochemistry

(4S,5R)-5-(hydroxymethyl)-4-phenyloxazolidin-2-one

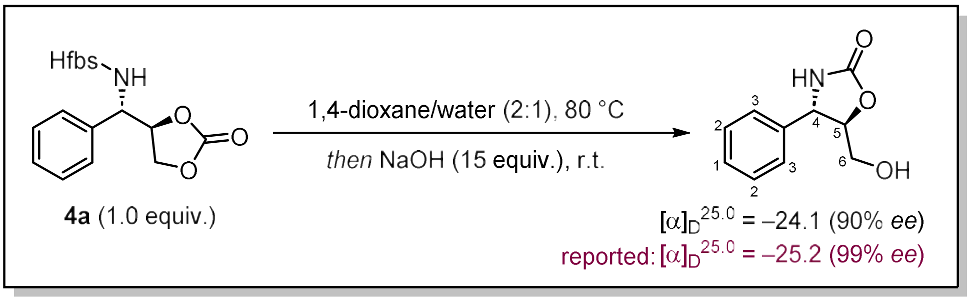

Prepared according to the protocol reported by Phipps and co-workers.[38] Under an atmosphere of air, a 4.0 mL crimp-top vial was charged with 2,2,3,3,4,4,4-heptafluorobutyl ((*S*)-((*R*)-2-oxo-1,3-dioxolan-4-yl)(phenyl)methyl)sulfamate **4a** (38.2 mg, 83.4 µmol, 1.00 equiv., 90% *ee*) dissolved in 1,4-dioxane/water (2:1, 2.25 mL). The vial was then capped and heated at 80 °C for 20 h. After cooling down to room temperature, the solvent was removed *in vacuo* and the residue was transferred with MeOH (2.50 mL) into a 10 mL microwave vial. Freshly ground NaOH (50.3 mg, 1.26 mmol, 15.0 equiv.) was added. The reaction was stirred at 23 °C for 16 h. Then, the mixture was concentrated under reduced pressure, diluted with EtOAc (20 mL) and washed with H_2O (4.0 mL). The aqueous layer was extracted with EtOAc (3 x 20 mL) and the combined organic phases were dried over $MgSO_4$, filtered, and concentrated under reduced pressure. Purification by flash column chromatography (SiO_2, 0-80% v/v EtOAc in petroleum ether) afforded the title compound as a white solid (5.30 mg, 27.0 µmol, 33% yield, 90% *ee*).

^{1}H NMR (700 MHz, CD_3OD): δ 7.42-7.37 (m, 4H, H-2, H-3), 7.34 (tt, J = 6.6, 1.4 Hz, 1H, H-1), 4.81 (d, J = 6.3 Hz, 1H, H-4), 4.34 (dt, J = 6.3, 4.0 Hz, 1H, H-5), 3.83 (dd, J = 12.6, 3.7 Hz, 1H, H-6a), 3.71 (dd, J = 12.6, 4.2 Hz, 1H, H-6b).

Note: N-H and O-H signals were not observed.

^{13}C NMR (176 MHz, CD_3OD): δ 161.6, 141.9, 130.1, 129.5, 127.3, 86.7, 62.6, 59.0.

R_f = 0.12 (40% v/v EtOAc in petroleum ether).

$[\alpha]_D^{25.0}$ = -24.1 (c. 0.35, MeOH); [reported[39] $[\alpha]_D^{25.0}$ = -25.2 (c. 0.51, MeOH) for 99% *ee*].

Chiral SFC Analysis: CHIRALPAK IK (CO_2:MeOH, 90:10, 2.50 mL min^{-1}, 40 ˚C), t_R = 9.5 (major), 11.6 (minor) minutes.

The spectroscopic data is in agreement with that reported in the literature.[39]

All remaining enantioenriched oxyamination products were tentatively assigned the same absolute stereochemistry by analogy with the result above.

General Procedure 11 (GP 11):

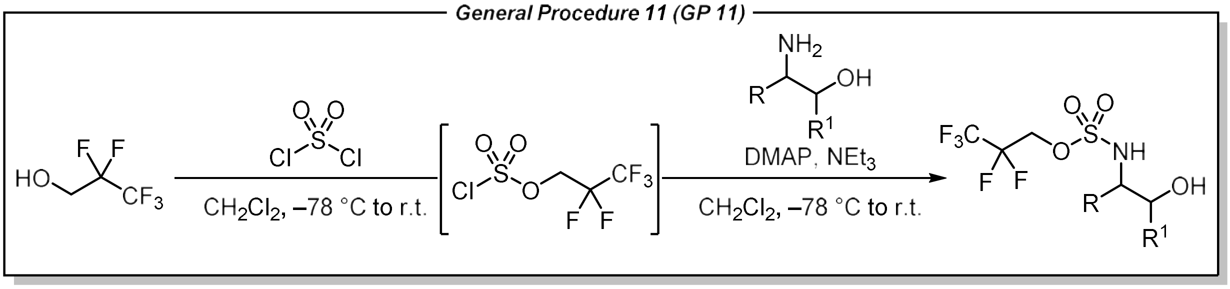

To a solution of 2,2,3,3,3-pentafluoro-1-propanol (0.448 mL, 5.0 mmol, 1.0 equiv.) and pyridine (0.403 mL, 5.0 mL, 1 equiv.) in dry CH_2Cl_2 (20 mL), was added dropwise sulfuryl chloride in CH_2Cl_2 (1 M solution, 5.50 mL, 5.5 mmol, 1.1 equiv.) at -78 °C under an inert atmosphere of nitrogen. The reaction mixture was allowed to warm to room temperature and stirred overnight. Reaction completion was determined via ^{1}H NMR spectroscopy and the corresponding sulfurochloridate intermediate was used directly in the next step.

To a solution of the amino alcohol (5.0 mmol, 1.0 equiv.), Et_3N (2.09 mL, 15.0 mmol, 3 equiv.) and DMAP (611 mg, 5.0 mmol, 1 equiv.) in dry CH_2Cl_2 (20 mL), was added dropwise the sulfurochloridate solution at -78 ˚C under an inert atmosphere of nitrogen. The reaction mixture was allowed to warm to room temperature and stirred overnight. Aqueous saturated $NaHCO_3$ solution (40 mL) was subsequently added to the reaction mixture at 0 °C. The aqueous layer was extracted thrice with CH_2Cl_2 and the combined organic layers were dried over $MgSO_4$,

filtered and concentrated. Purification by flash column chromatography afforded the corresponding product.

2,2,3,3,3-pentafluoropropyl (S)-(2-hydroxy-1-phenylethyl)sulfamate (**12**)

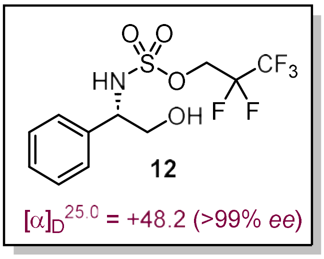

Prepared according to **GP 11** with (*S*)-(+)-2-phenylglycinol (693 mg, 5.1 mmol, 1.0 equiv.). Purification by flash column chromatography (SiO_2, 15-20% v/v acetone in $CHCl_3$, *then* 40% v/v EtOAc in hexane) afforded the title compound as a white solid (88.1 mg, 0.252 mmol, 5% yield).

[a]$_D^{25.0}$ = +21.2 (c. 0.87, $CHCl_3$).

Chiral SFC Analysis: CHIRALPAK IK (CO_2:MeOH, 94:6, 2.50 mL min^{-1}, 40 ℃) t_R = 3.0 minutes.

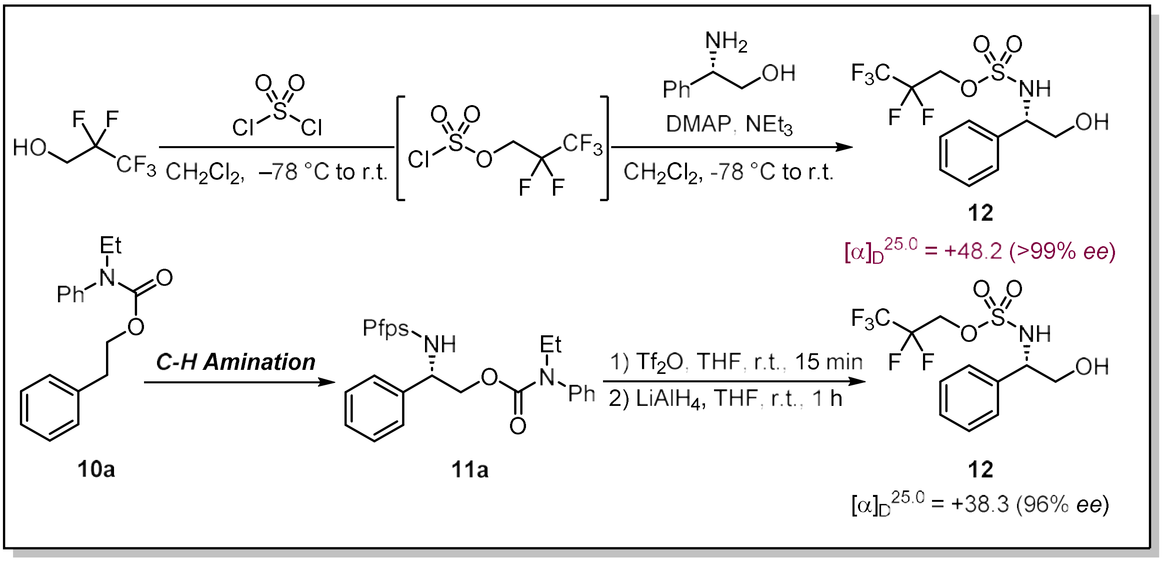

An authentic, enantiopure sample of **12** was prepared from (*S*)-(+)-2-phenylglycinol. The optical rotation of **12** was positive for the products obtained via both synthetic routes. Furthermore, the major enantiomeric peak observed in the chiral SFC analysis of **12** was consistent for both products. Therefore, we tentatively assign the (*S*)-configuration to **11a** when catalyst Rh_2(**A1**)$_2$•(**B1**)$_2$•(Pyr)$_2$ was used in the C-H amination of **10a**.

All remaining enantioenriched C-H amination products were tentatively assigned the same absolute stereochemistry by analogy with the result above.

2,2,3,3,3-pentafluoropropyl ((1S,2S)-2-hydroxy-2,3-dihydro-1H-inden-1-yl)sulfamate (**S9**)

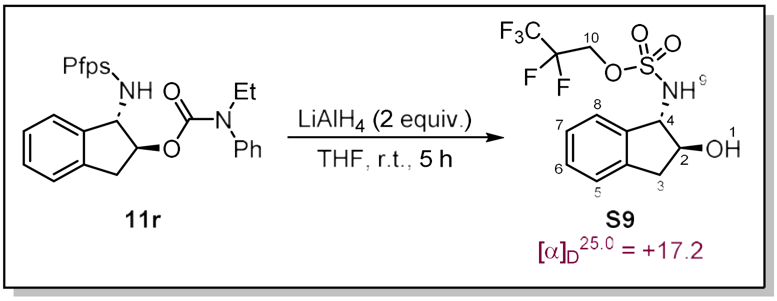

A crimp-top vial was charged with **11r** (14.8 mg, 0.0291 mmol, 72% *ee*, 1.0 equiv.). The vial was sealed and evacuated and backfilled with nitrogen three times, following which dry THF (5.0 mL) and $LiAlH_4$ (0.024 mL, 2.4 M in THF, 0.0582 mmol, 2 equiv.) were added at room temperature. The resulting mixture was stirred for 5 h at room temperature and quenched with sodium sulfate decahydrate until effervescence had subsided. The resulting mixture was diluted with EtOAc and filtered through Celite®, eluting with copious amounts of EtOAc and $CHCl_3$. The filtrate was collected and the solvent removed under reduced pressure. Purification by flash column chromatography (SiO_2, 20% v/v acetone in $CHCl_3$) afforded the title compound as a white solid (3.9 mg, 0.0108 mmol, 37% yield, 71% *ee*).

^{1}H NMR (500 MHz, CD_3CN): δ 7.37-7.34 (m, 1 H, H-8), 7.30-7.22 (m, 3 H, H-5, H-6, H-7), 6.52 (br s, 1 H, H-9), 4.80-4.64 (m, 3 H, H-4, H-10), 4.34 (q, J = 6.8 Hz, 1 H, H-2), 3.62 (br s, 1 H, H-1), 3.21 (dd, J = 15.7, 7.0 Hz, 1 H, H-3a), 2.76 (dd, J = 15.7, 7.0 Hz, 1 H, H-3b).

^{13}C NMR (126 MHz, CD_3CN): δ 140.8, 140.1, 129.7, 128.1, 126.0, 125.3, 119.4* (qt, $J_{C\text{-}F}$ = 285, 34.6 Hz), 113.0* (tq, $J_{C\text{-}F}$ = 255, 38.2 Hz), 79.4, 67.0, 65.0 (t, $J_{C\text{-}F}$ = 27.8 Hz), 39.1.

* These signals which coupled with fluorine atoms could not be observed clearly. These coupling constants were confirmed by using the opposite enantiomer ***ent*-S9** which is the authentic sample (see below).

^{19}F NMR (376 MHz, CD_3CN): δ -84.3, -124.0 (m).

R_f = 0.57 (20% v/v acetone in $CHCl_3$).

$[a]_D^{25.0}$ = +17.2 (c. 0.33, $CHCl_3$).

HRMS (-ESI): *m/z* found $[M–H]^-$ 360.0330, $[C_{12}H_{11}F_5NO_4S]^-$ requires 360.0334, (δ = -1.1 ppm).

Chiral SFC Analysis: CHIRALPAK IC (CO_2:MeOH, 95:5, 2.50 mL min^{-1}, 40℃) t_R = 2.8 (major), 3.1 (minor) minutes.

2,2,3,3,3-pentafluoropropyl ((1R,2R)-2-hydroxy-2,3-dihydro-1H-inden-1-yl)sulfamate (**ent-S9**)

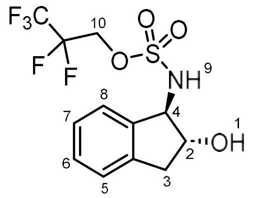

***ent*-S9**

$[\alpha]_D^{25.0}$ = -19.9 (>99% *ee*)

Prepared according to **GP 11** with (*1R*, *2R*)-1-amino-2-indanol (749 mg, 5.02 mmol, 1.0 equiv.). Purification by flash column chromatography (SiO_2, 30% v/v EtOAc in hexane, *then* 10% v/v acetone in $CHCl_3$) afforded the title compound as a white solid (195 mg, 0.540 mmol, 11% yield).

R_f = 0.26 (30% v/v EtOAc in hexane), 0.39 (10% v/v acetone in $CHCl_3$).

$[a]_D^{25.0}$ = -19.9 (c. 0.49, $CHCl_3$).

HRMS (-ESI): *m/z* found $[M–H]^-$ 360.0324, $[C_{12}H_{11}F_5NO_4S]^-$ requires 360.0334, (δ = -2.8 ppm).

Chiral SFC Analysis: CHIRALPAK IC (CO_2:MeOH, 95:5, 2.50 mL min^{-1}, 40℃) t_R = 3.1 minutes.

2,2,3,3,3-pentafluoropropyl ((1S,2R)-2-hydroxy-2,3-dihydro-1H-inden-1-yl)sulfamate (**S10**)

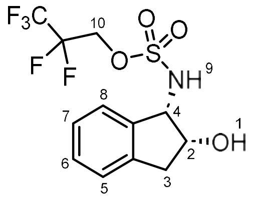

S10

Prepared according to **GP 11** with (*1S*, *2R*)-1-amino-2-indanol (746 mg, 5.00 mmol, 1.0 equiv.). Purification by flash column chromatography (SiO_2, 40% v/v EtOAc in hexane, *then* 10% v/v acetone in $CHCl_3$) afforded the title compound with impurities as a brown solid (126 mg, 0.348 mmol, 7% yield, 95% purity).

^{1}H NMR (700 MHz, CD_3CN): δ 7.39-7.38 (m, 1 H, H-8), 7.29-7.25 (m, 3 H, H-5, H-6, H-7), 6.46 (br s, 1 H, H-9), 4.86 (d, *J* = 4.9 Hz, 1 H, H-4), 4.79-4.73 (m, 1 H, H-10a), 4.68-4.63 (m, 1 H, H-10b, overlapped with impurities), 4.57-4.55 (m, 1 H, H-2), 3.39 (d, *J* = 4.1 Hz, 1 H, H-1), 3.10 (dd, *J* = 16.4, 4.9 Hz, 1 H, H-3a), 2.88 (dd, *J* = 16.4, 1.5 Hz, 1 H, H-3b).

^{13}C NMR (176 MHz, CD_3CN): δ 141.4, 140.6, 129.4, 127.9, 126.3, 125.3, 119.4 (qt, J_{C-F} = 286, 34.6 Hz), 113.0 (tq, J_{C-F} = 255, 38.1 Hz), 73.2, 64.9 (t, J_{C-F} = 27.7 Hz), 62.9, 40.2.

^{19}F NMR (376 MHz, CD_3CN): δ -84.3, -124.1 (m)

R_f = 0.51 (40% v/v EtOAc in hexane), 0.26 (10% v/v acetone in $CHCl_3$).

HRMS (-ESI): *m/z* found $[M–H]^-$ 360.0321, $[C_{12}H_{11}F_5NO_4S]^-$ requires 360.0334, (δ = -3.6 ppm).

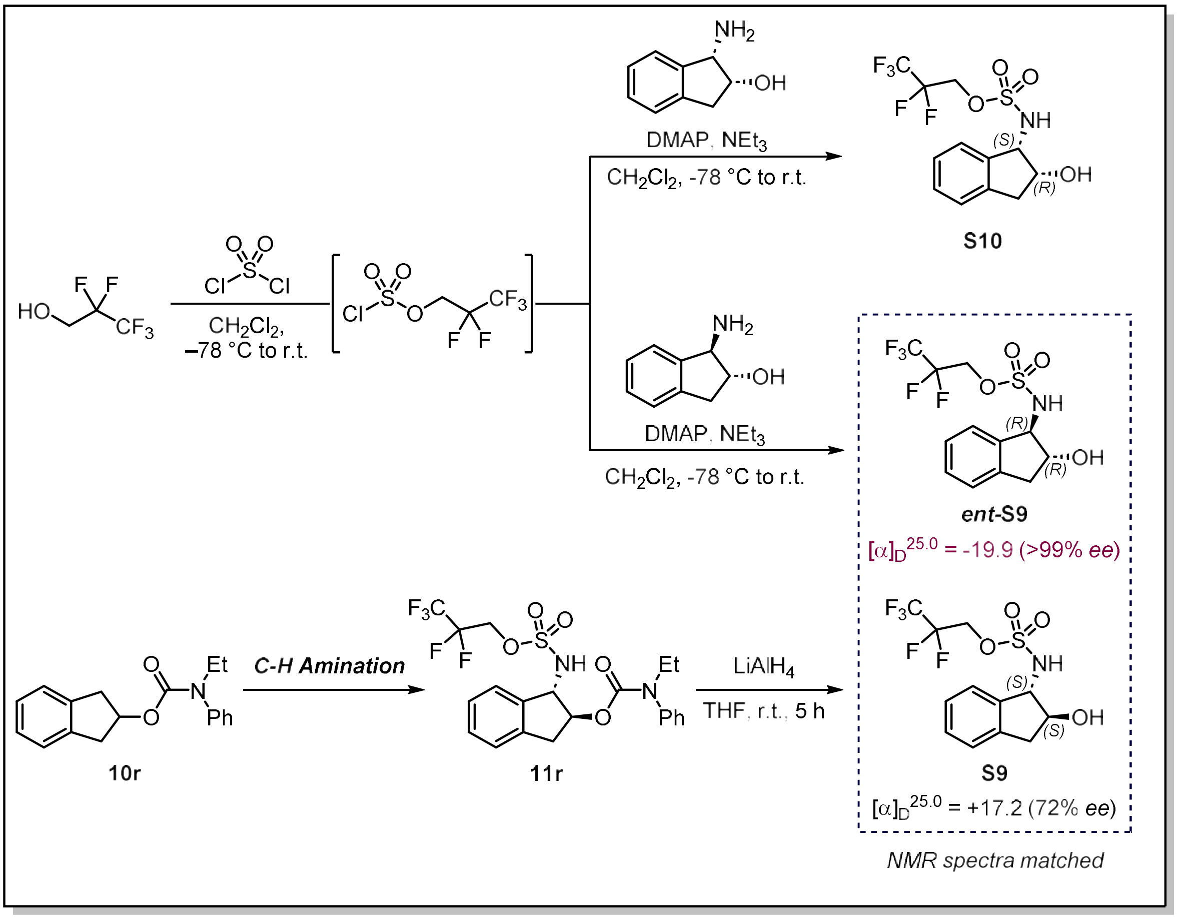

Authentic, enantiopure samples of ***ent*-S9** and **S10** were prepared from the corresponding 1-amino-2-indanols. The NMR spectrum of **S9**, which was derived from the C-H amination product **11r**, matched that of ***ent*-S9** (**Figure S1**). Furthermore, the NMR spectrum of **S9** did not match that of **S10**. These results demonstrate that **11r** is the *anti* diastereomer and not the *syn* diastereomer. Lastly, based on optical rotation and chiral SFC analysis comparisons of **S9** and ***ent*-S9**, we assign the (1*S*, 2*S*)-configuration to **11r** when catalyst Rh_2(**A1**)$_2$•(**B1**)$_2$•(Pyr)$_2$ was used in the C-H amination of **10r**.

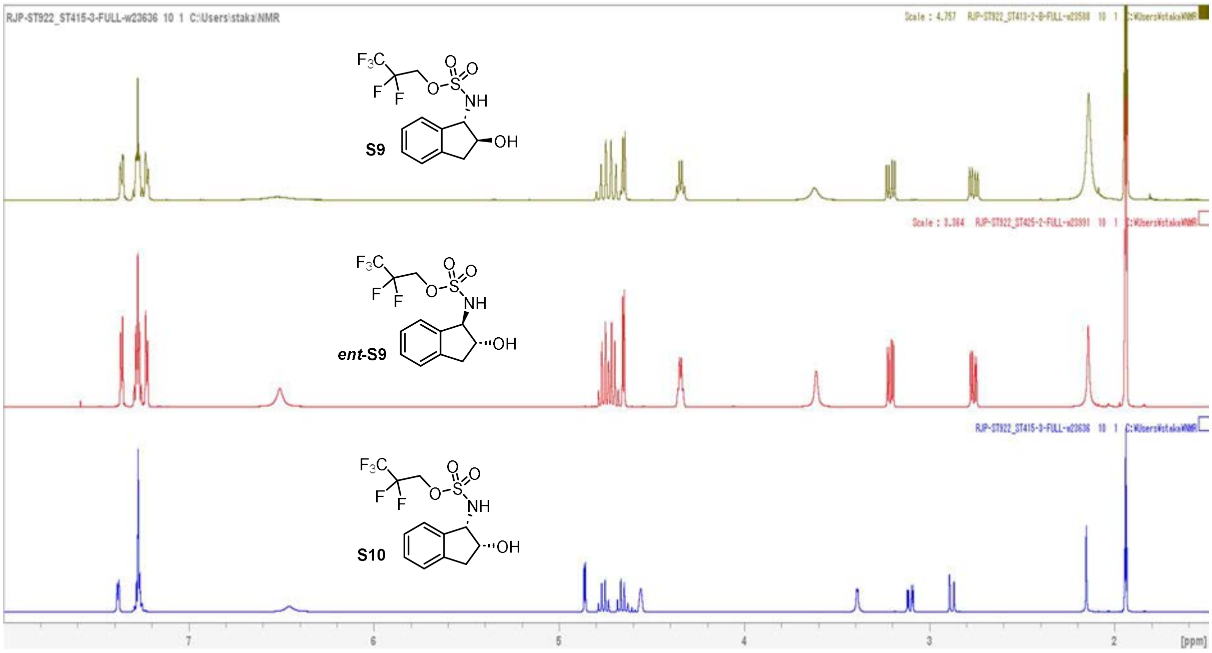

Figure S1: Comparison of ^{1}H NMR spectra in CD_3CN for compounds **S9**, ***ent*-S9**, and **S10**. Top: compound **S9**, middle: compound ***ent*-S9**, bottom: compound **S10**.

17 Rapid Catalyst Generation with SFC-MS Analysis

A sinter funnel was loosely packed with a pad of Amberlite® IRC120 H hydrogen form beads (approximately 6 cm column height) and equilibrated by flushing through with three column volumes of MeOH. Following this, a solution of Rh_2(**A1**)$_2$•(***n*-Bu$_4$N**)$_2$ (28.6 mg, 20 µmol) in MeOH (4 mL) was loaded onto the pad and eluted with light suction. The eluent was recycled through the pad a further four times. Following this, the eluent was evaporated to dryness and a stock solution of the catalyst in MeOH (2 mL, 10 mM) was prepared.

To a vial containing the chiral cation bromide (4 µmol, 8 mol%) was added MeOH (0.3 mL) and the catalyst stock solution (0.1 mL, 1 µmol, 2 mol%). The reaction mixture was stirred for 3 h under an atmosphere of air to effect the salt metathesis. Following this, the reaction mixture was concentrated under a gentle stream of nitrogen and residual MeOH was removed under vacuum.

A stock solution containing the allylic carbamate substrate (0.73 mmol, 1.0 equiv.) and 2,2,3,3,4,4,4-heptafluorobutyl sulfamate **2a** (243 mg, 0.87 mmol, 1.2 equiv.) in 1,3-difluorobenzene (2.9 mL, 0.25 M) was prepared. To the vial containing the catalyst was added 1,3-difluorobenzene (0.3 mL) and the substrate stock solution (0.2 mL, 0.05 mmol with respect to the allylic carbamate substrate), bringing the final concentration to 0.1 M. The vial was cooled to -35 ˚C over 10 min. C_6F_5IO (31.0 mg, 0.1 mmol, 2.0 equiv.) and $C_6F_5I(OTFA)_2$ (2.6 mg, 5 µmol, 10 mol%) were subsequently added together in a single portion at -35 ˚C. The vial was sealed and the reaction mixture stirred at -35 ˚C overnight (~16 h). Saturated aqueous thiourea (1 mL) and $CHCl_3$ (1 mL) were added and the biphasic mixture was stirred vigorously for 5 min at -35 ˚C. The mixture was allowed to warm to room temperature and stirred for a further 15 min. The layers were separated and the organic layer was passed through a short pad of $MgSO_4$. The aqueous layer was extracted with $CHCl_3$ (6 x 1 mL) with each organic layer passed through a short pad of $MgSO_4$.The combined organic layers were concentrated, reconstituted in 5% H_2O/MeCN (3.5 mL) and transferred to a crimp-top vial. The vial was sealed and heated at 80 ˚C for 3 h. The reaction mixture was concentrated under a gentle stream of nitrogen and the crude residue was passed through a short pipette of silica, eluting with 20% v/v diethyl ether in CH_2Cl_2. The crude mixture was concentrated under a gentle stream of nitrogen. NMR yields were determined from the crude mixture using 1,2-dimethoxyethane as an internal standard.

Having obtained the NMR yields for the reaction, the crude residue was concentrated under a gentle stream of nitrogen and reconstituted in CH_2Cl_2 (1.5 mL). An aliquot (0.02 mL) was withdrawn and concentrated under a gentle stream of nitrogen. The residue was reconstituted

in 5% v/v *i*-PrOH in hexane (1.5 mL), and a 0.15 mL aliquot was removed and diluted to a final volume of 1.5 mL. From this sample, a 0.15 mL aliquot was removed and diluted to a final volume of 1.5 mL. This final sample was subsequently submitted to SFC/SIM-MS analysis for *ee* determination.

*Catalyst screen with allylic carbamate **1a***

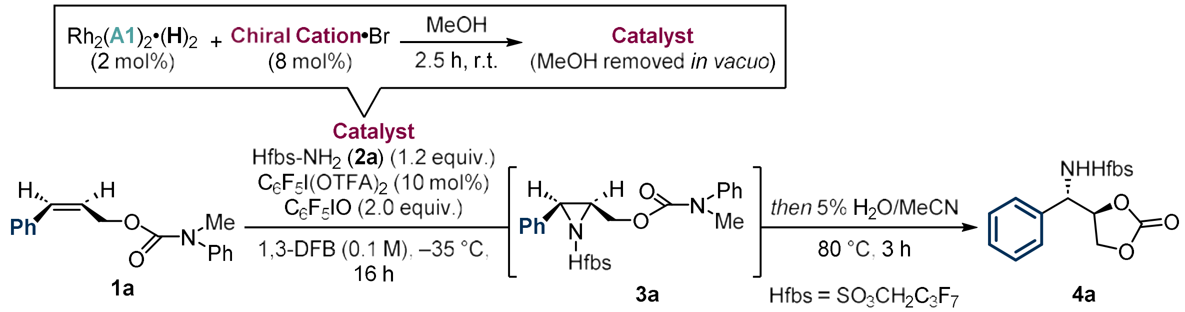

SFC Details: Enantiomer separation was carried out on the SFC instrument, using a CHIRALPAK IC stationary phase and an isocratic CO_2:MeOH (95:5) mobile phase at 40 ˚C with a flow rate of 1.25 mL/min for 9 min. A MeOH/H_2O/formic acid (90/10/1 (v/v/v)) mixture was introduced by the second pump post-column prior to entering the QDa detector. For mass spec detection, positive mode selected ion monitoring [SIM, *m/z* 478, $(M+Na)^+$, cone voltage 15 V, sampling frequency 20 Hz] was used. The SIM traces were smoothed (20 iterations, using a window of 2) and then integrated.

Table S9: Evaluation of chiral cation with substrate **1a**.

Entry	Chiral cation•Br	Unreacted 1a/ %[a]	4a/ %[a]	*ee*/ %[b]
1	**B3**•Br	-	27	+87
2	**B11**•Br	14	34	+20
3	**B12**•Br	9	31	-3
4	**C3**•Br	3	32	-65
5	**C4**•Br	20	23	+18
6	**C5**•Br	24	26	-3
7	**C6**•Br	22	26	+4
8	**C7**•Br	21	21	+22
9	***epi*-C4**•Br	20	22	-6
10	***epi*-C7**•Br	25	29	-9
11	**C8**•Br	22	33	+28
12	**D1**•Br	63	13	-2

Reactions performed on 0.05 mmol scale with respect to allylic carbamate **1a**. [a] Yields determined from crude ^1H NMR analysis with 1,2-dimethoxyethane as an internal standard. [b] *ee* was determined by chiral SFC/SIM-MS analysis.

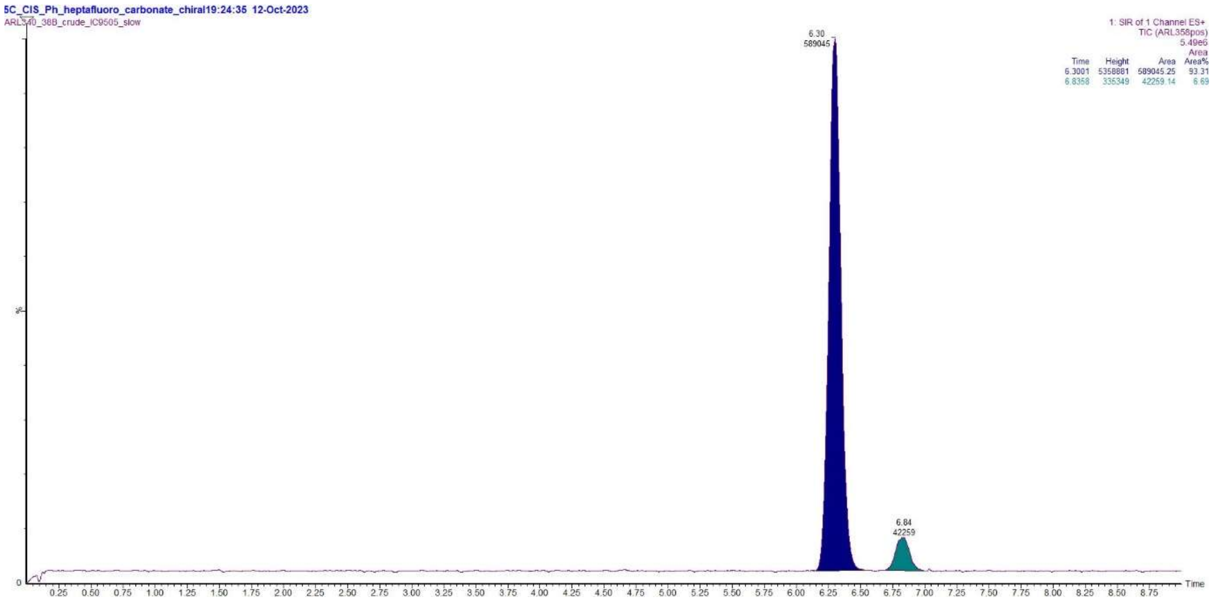

Figure S2: Representative SIM chromatogram of carbonate product **4a** (**Table S9**, Entry 1).

*Catalyst screen with allylic carbamate **15a***

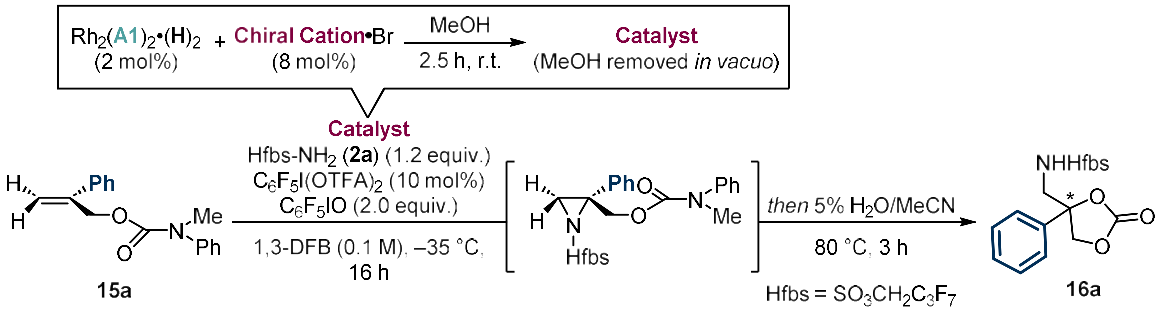

SFC Details: Enantiomer separation was carried out on the SFC instrument, using a CHIRALPAK IJ stationary phase and an isocratic CO_2:MeOH (97:3) mobile phase at 40 ˚C with a flow rate of 2.50 mL/min for 5 min. A MeOH/H_2O/formic acid (90/10/1 (v/v/v)) mixture was introduced by the second pump post-column prior to entering the QDa detector. For mass spec detection, positive mode selected ion monitoring [SIM, *m/z* 478, $(M+Na)^+$, cone voltage 15 V, sampling frequency 20 Hz] was used. The SIM traces were smoothed (20 iterations, using a window of 2) and then integrated.

Table S10: Evaluation of chiral cation with substrate **15a**.

Entry	Chiral cation•Br	Unreacted 15a/ %[a]	16a/ %[a]	*ee*/ %[b]
1	**B3**•Br	3	42	Racemic
2	**B11**•Br	-	39	+3
3	**B12**•Br	-	47	-5
4	**C3**•Br	2	42	+7
5	**C4**•Br	20	45	-11
6	**C5**•Br	-	50	-15
7	**C6**•Br	4	43	Racemic
8	**C7**•Br	3	48	+6
9	***epi*-C4**•Br	-	44	-3
10	***epi*-C7**•Br	-	47	-2
11	**C8**•Br	-	40	+15
12	**D1**•Br	-	47	+5
13	**D2**•Br	4	51	Racemic

Reactions performed on 0.05 mmol scale with respect to allylic carbamate **15a**. [a] Yields determined from crude ^{1}H NMR analysis with 1,2-dimethoxyethane as an internal standard. [b] *ee* was determined by chiral SFC/SIM-MS analysis.

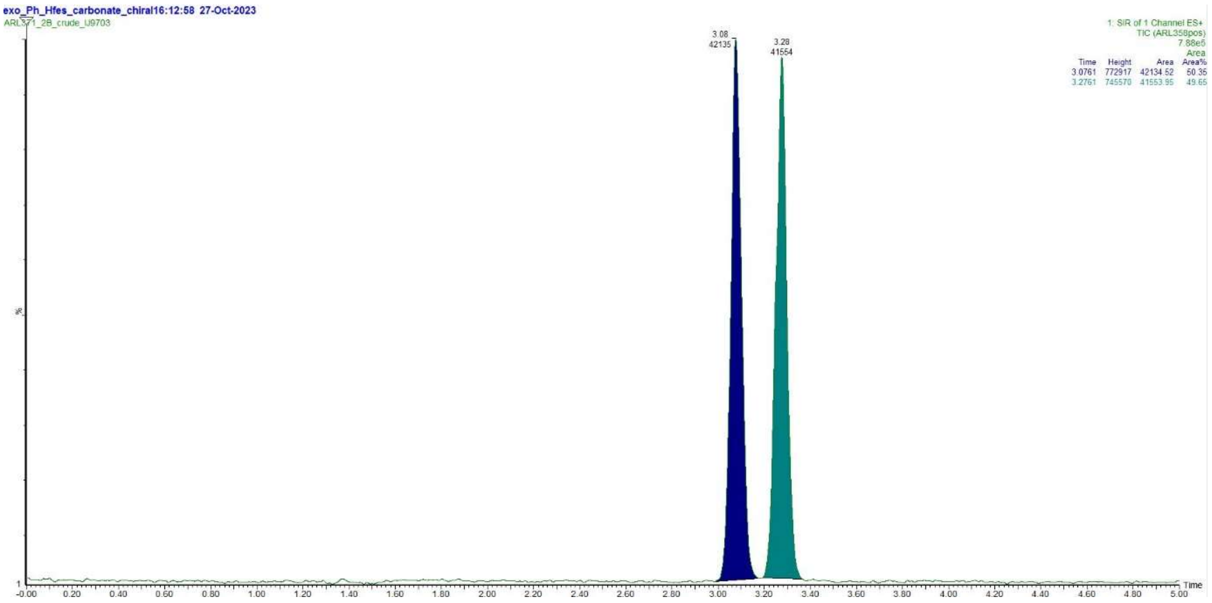

Figure S3: Representative SIM chromatogram of carbonate product **16a** (**Table S10**, Entry 1).

*Catalyst screen with allylic carbamate **15b***

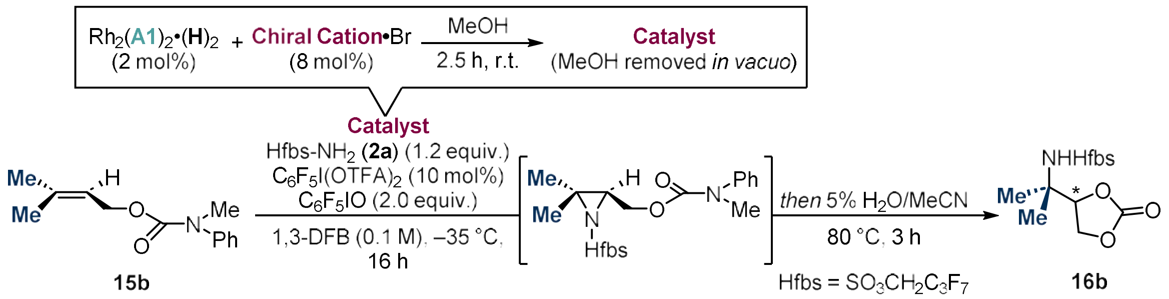

SFC Details: Enantiomer separation was carried out on the SFC instrument, using a CHIRALPAK IC stationary phase and an isocratic CO_2:MeOH (97:3) mobile phase at 40 ˚C with a flow rate of 2.50 mL/min for 5 min. A MeOH/H_2O/formic acid (90/10/1 (v/v/v)) mixture was introduced by the second pump post-column prior to entering the QDa detector. For mass spec detection, positive mode selected ion monitoring [SIM, *m/z* 430, $(M+Na)^+$, cone voltage 15 V, sampling frequency 20 Hz] was used. The SIM traces were smoothed (20 iterations, using a window of 2) and then integrated.

Table S11: Evaluation of chiral cation with substrate **16b**.

Entry	Chiral cation•Br	Unreacted 15b/ %[a]	16b/ %[a]	ee/ %[b]
1	**B3**•Br	3	18	+30
2	**B11**•Br	6	16	+8
3	**B12**•Br	6	16	+5
4	**C3**•Br	37	16	-11
5	**C4**•Br	8	13	+7
6	**C5**•Br	9	15	Racemic
7	**C6**•Br	13	10	+17
8	**C7**•Br	9	9	+23
9	***epi*-C4**•Br	4	14	-10
10	***epi*-C7**•Br	9	19	-14
11	**C8**•Br	6	13	+8
12	**D1**•Br	11	15	Racemic
13	**D2**•Br	3	8	Racemic

Reactions performed on 0.05 mmol scale with respect to allylic carbamate **15b**. [a] Yields determined from crude ^{1}H NMR analysis with 1,2-dimethoxyethane as an internal standard. [b] *ee* was determined by chiral SFC/SIM-MS analysis.

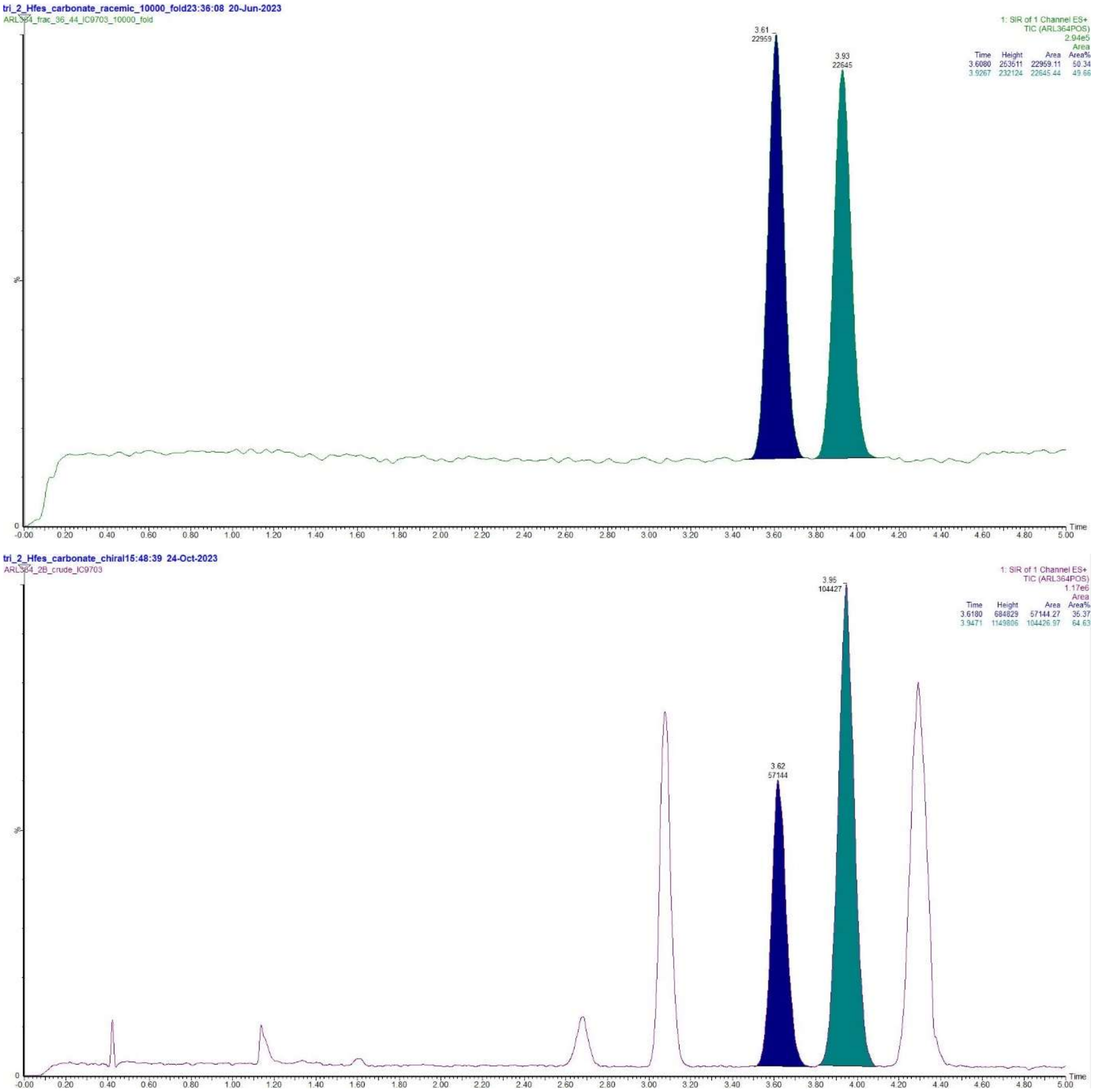

Figure S4: (Top) Racemic SIM chromatogram of **16b**. (Bottom) Representative chiral SIM chromatogram of **16b** (**Table S11**, Entry 1).

*Catalyst screen with allylic carbamate **15c***

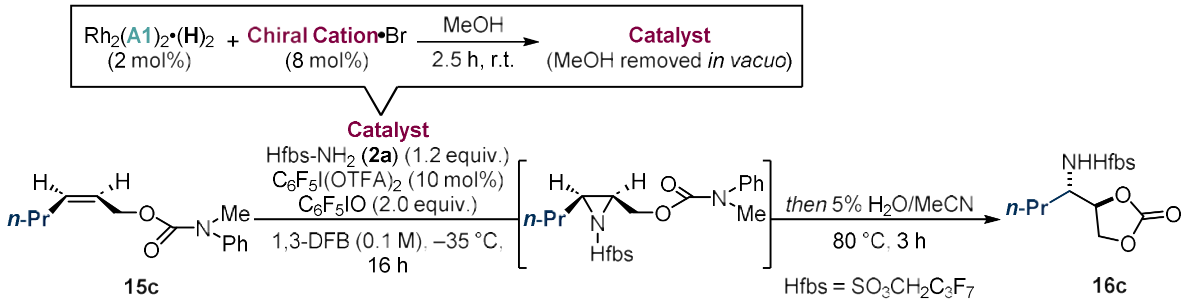

SFC Details: Enantiomer separation was carried out on the SFC instrument, using a CHIRALPAK IC stationary phase and an isocratic CO_2:MeOH (98:2) mobile phase at 40 ˚C with a flow rate of 2.50 mL/min for 8 min. A MeOH/H_2O/formic acid (90/10/1 (v/v/v)) mixture was introduced by the second pump post-column prior to entering the QDa detector. For mass spec detection, positive mode selected ion monitoring [SIM, *m/z* 444, $(M+Na)^+$, cone voltage 15 V, sampling frequency 20 Hz] was used. The SIM traces were smoothed (20 iterations, using a window of 2) and then integrated.

Table S12: Evaluation of chiral cation with substrate **16c**.

Entry	Chiral cation•Br	Unreacted 15c/ %[a]	16c/ %[a]	ee/ %[b]
1	**B3**•Br	21	37	+29
2	**B11**•Br	27	31	+6
3	**B12**•Br	29	37	+2
4	**C3**•Br	21	32	-14
5	**C4**•Br	26	20	+5
6	**C5**•Br	27	24	Racemic
7	**C6**•Br	37	19	+12
8	**C7**•Br	25	28	-5
9	***epi*-C4**•Br	23	24	+13
10	***epi*-C7**•Br	28	30	+13
11	**C8**•Br	32	17	Racemic
12	**D1**•Br	38	27	Racemic
13	**D2**•Br	23	26	+9

Reactions performed on 0.05 mmol scale with respect to allylic carbamate **15c**. [a] Yields determined from crude ^{1}H NMR analysis with 1,2-dimethoxyethane as an internal standard. [b] *ee* was determined by chiral SFC/SIM-MS analysis.

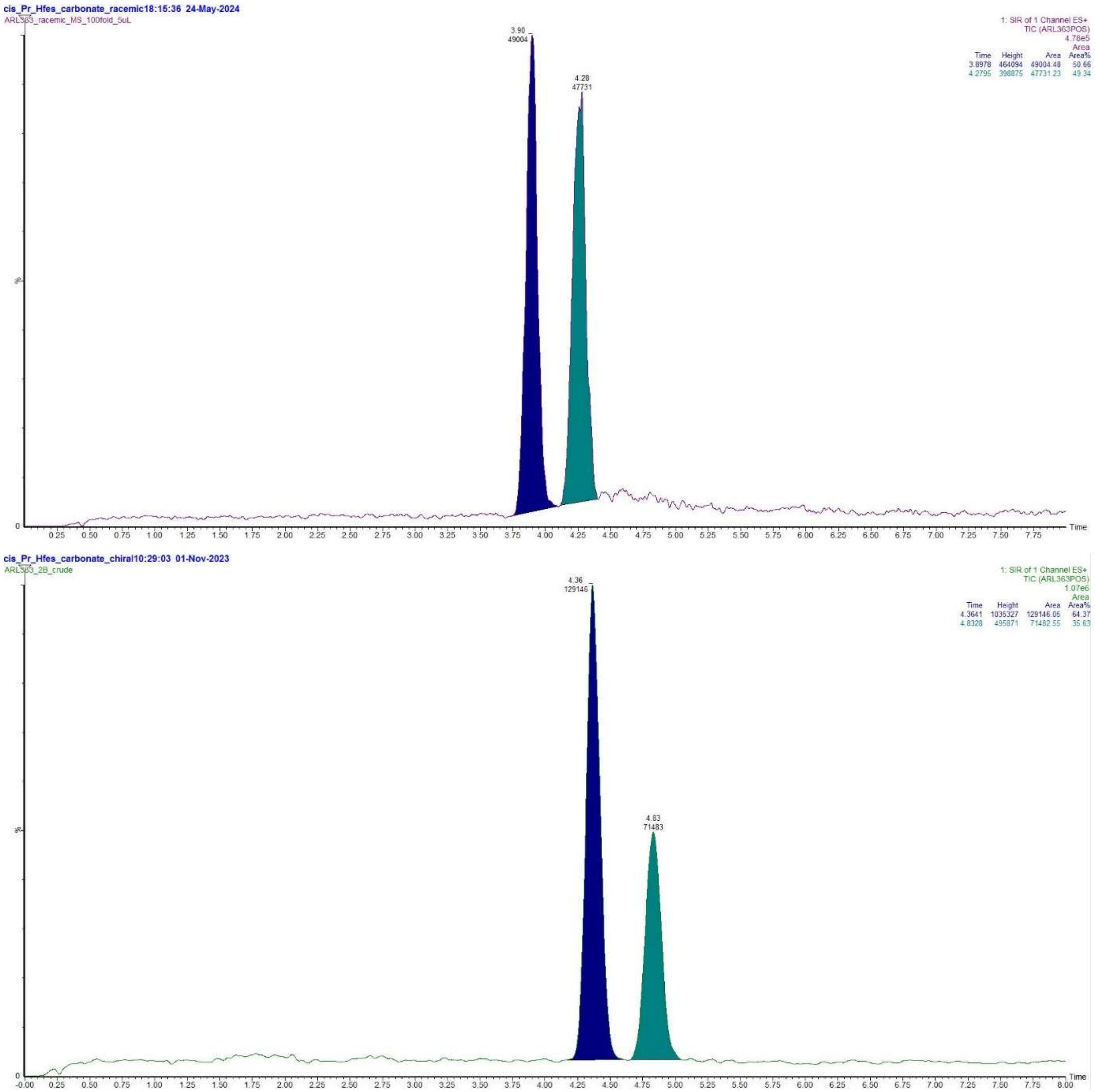

Figure S5: (Top) Racemic SIM chromatogram of **16c**. (Bottom) Representative chiral SIM chromatogram of **16c** (**Table S12**, Entry 1).

18 Chiral SFC, HPLC and GC Traces

2,2,3,3,4,4,4-heptafluorobutyl ((*S*)-((*R*)-2-oxo-1,3-dioxolan-4-yl)(phenyl)methyl)sulfamate (**4a**)

Chiral SFC Analysis CHIRALPAK IC (CO_2:MeOH, 95:5, 1.25 mL min^{-1}, 40 ˚C, 204 nm) indicated 91% *ee*, t_R = 5.6 (major), 6.0 (minor) minutes.

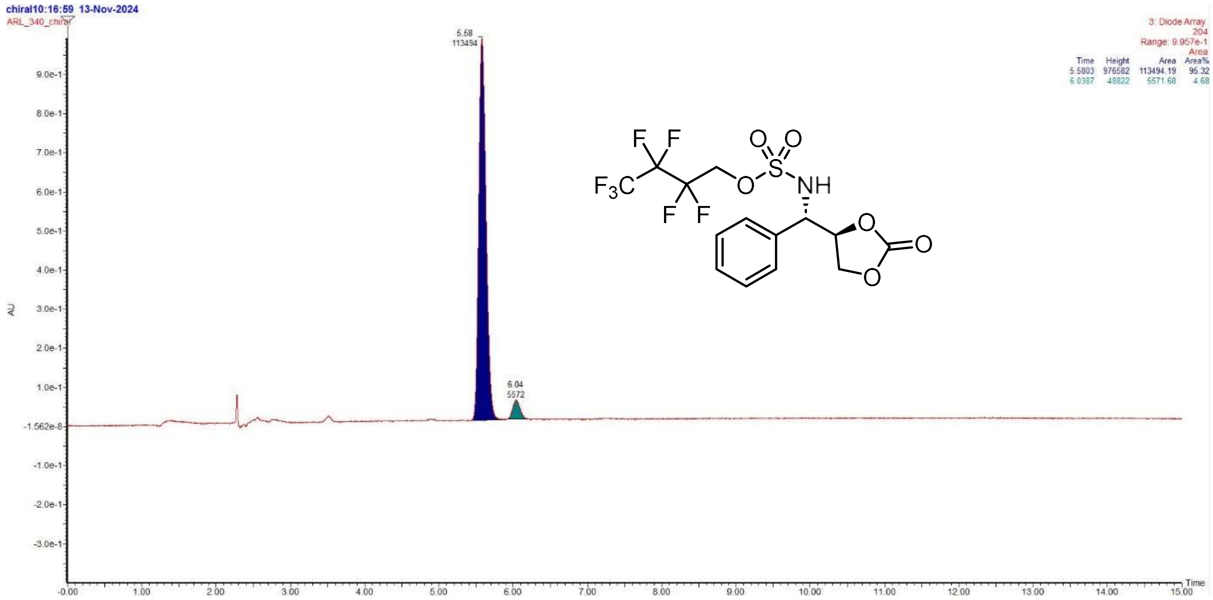

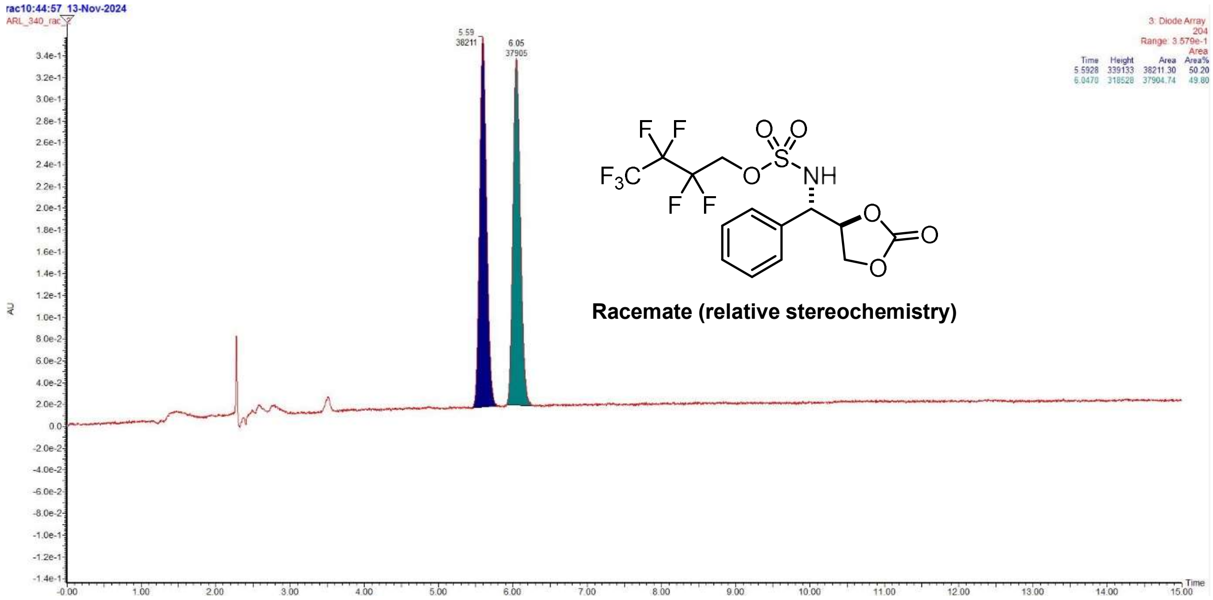

2,2,3,3,4,4,4-heptafluorobutyl ((*R*)-((*S*)-2-oxo-1,3-dioxolan-4-yl)(phenyl)methyl)sulfamate (***ent*-4a**)

Chiral SFC Analysis CHIRALPAK IC (CO_2:MeOH, 95:5, 1.25 mL min^{-1}, 40 ˚C, 204 nm) indicated 88% *ee*, t_R = 5.5 (minor), 5.9 (major) minutes.

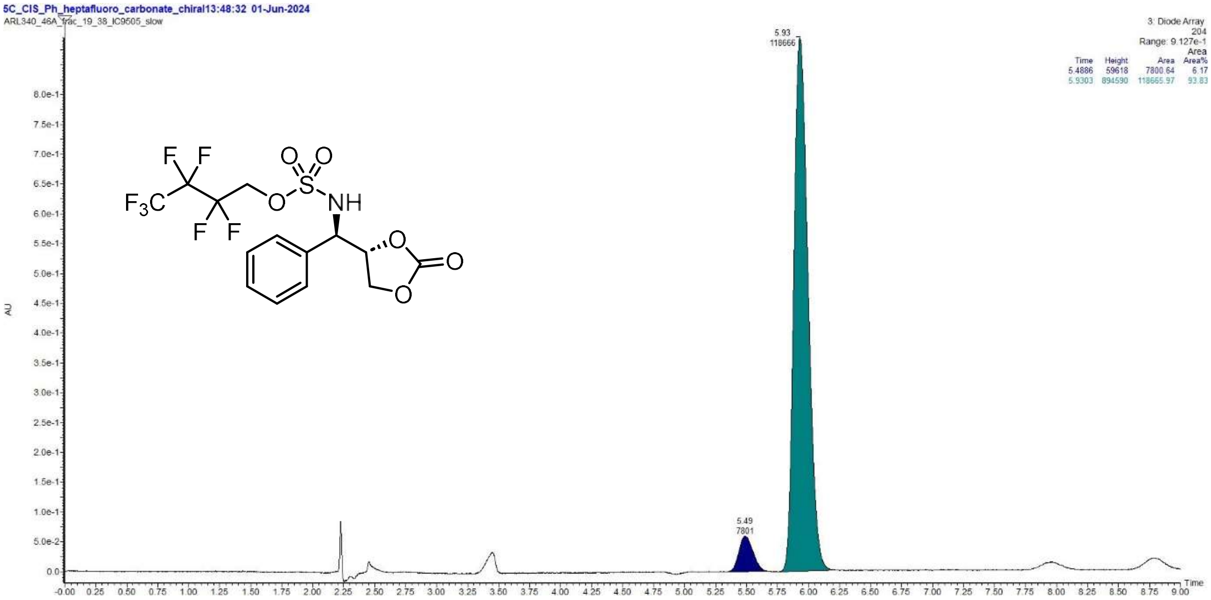

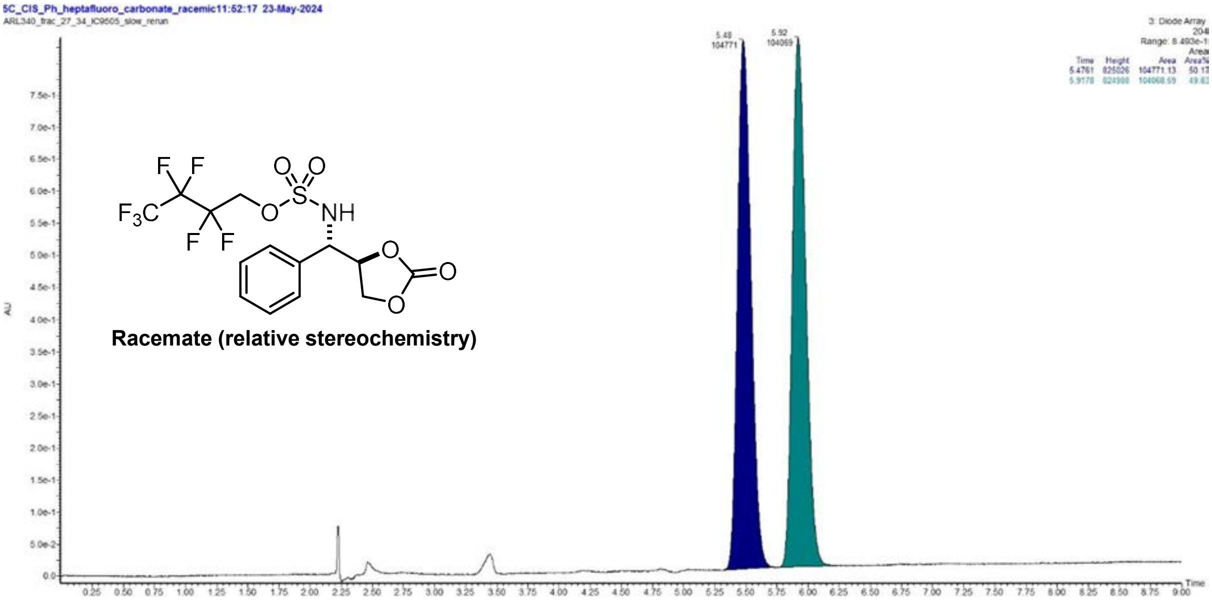

2,2,3,3,4,4,4-heptafluorobutyl ((*S*)-(2-isopropylphenyl)((*R*)-2-oxo-1,3-dioxolan-4-yl)methyl)sulfamate (**4b**)

Chiral SFC Analysis CHIRALPAK IK (CO_2:MeOH, 97:3, 2.50 mL min^{-1}, 40 ˚C, 208 nm) indicated 92% *ee*, t_R = 4.9 (minor), 5.5 (major) minutes.

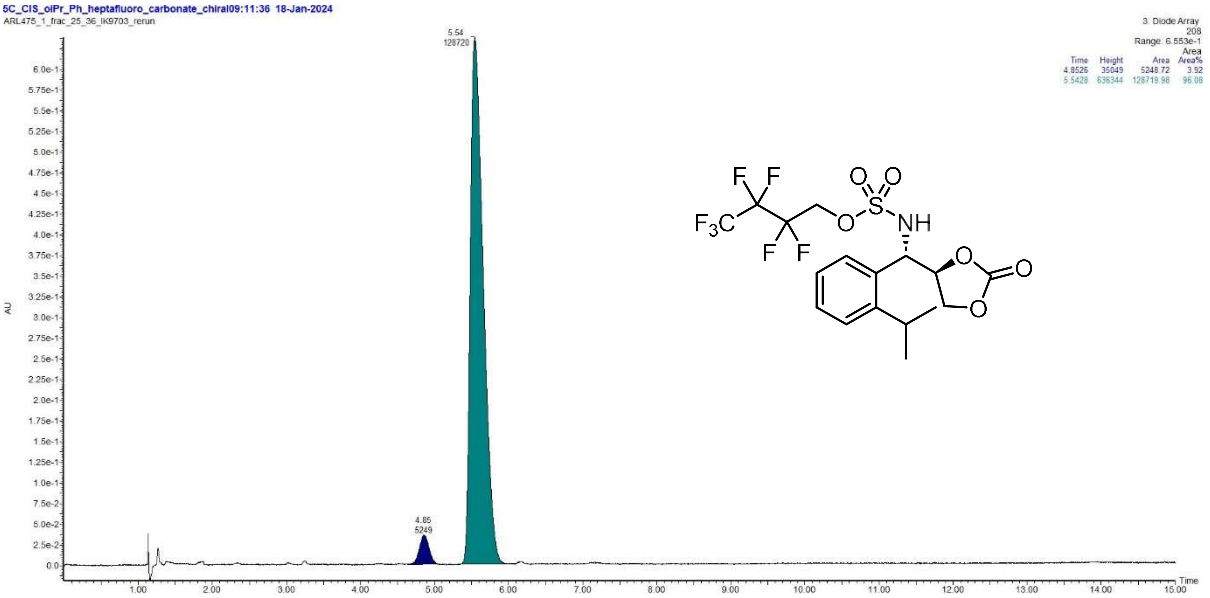

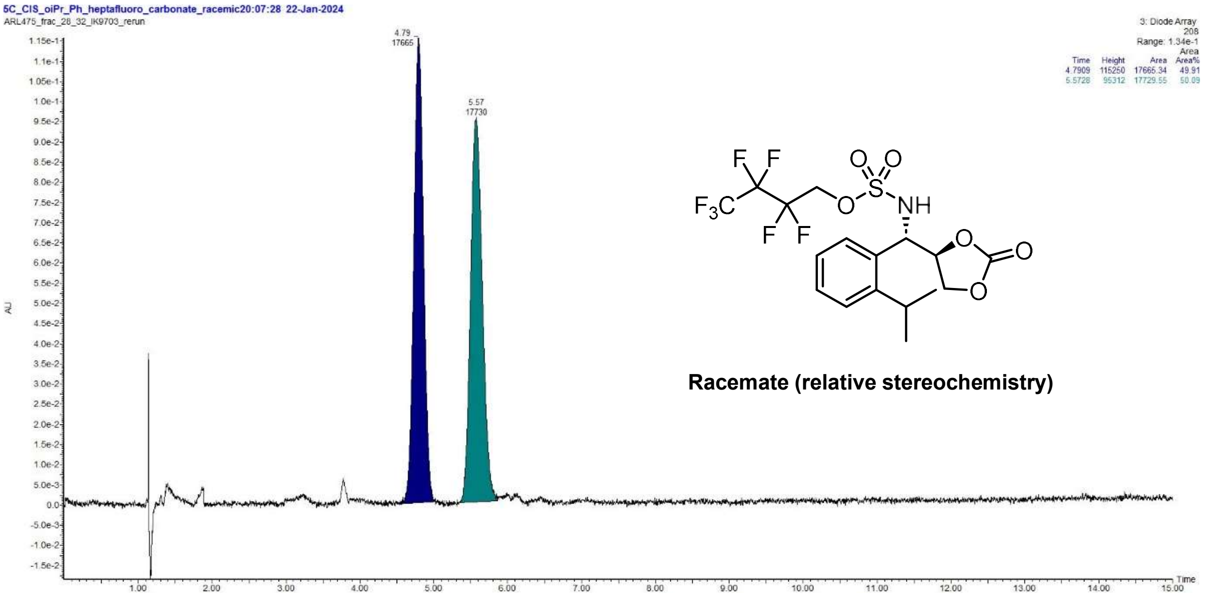

2,2,3,3,4,4,4-heptafluorobutyl ((*S*)-(2-methoxyphenyl)((*R*)-2-oxo-1,3-dioxolan-4-yl)methyl)sulfamate (**4c**)

Chiral SFC Analysis CHIRALPAK IK (CO_2:MeOH, 96:4, 2.50 mL min^{-1}, 40 ˚C, 214 nm) indicated 90% *ee*, t_R = 4.4 (minor), 5.2 (major) minutes.

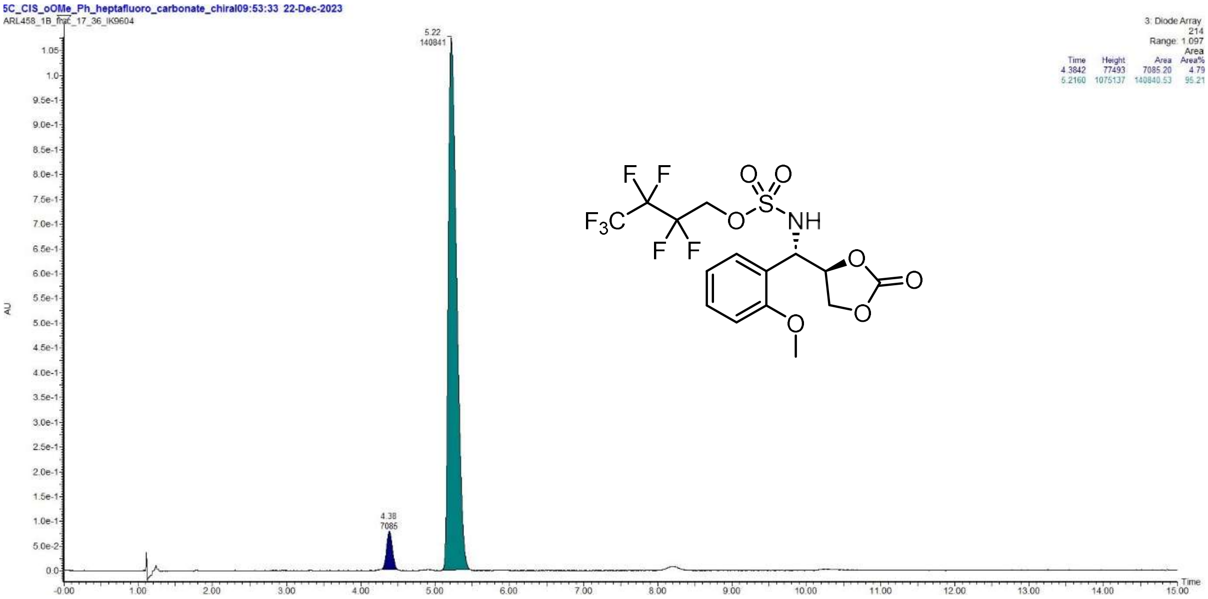

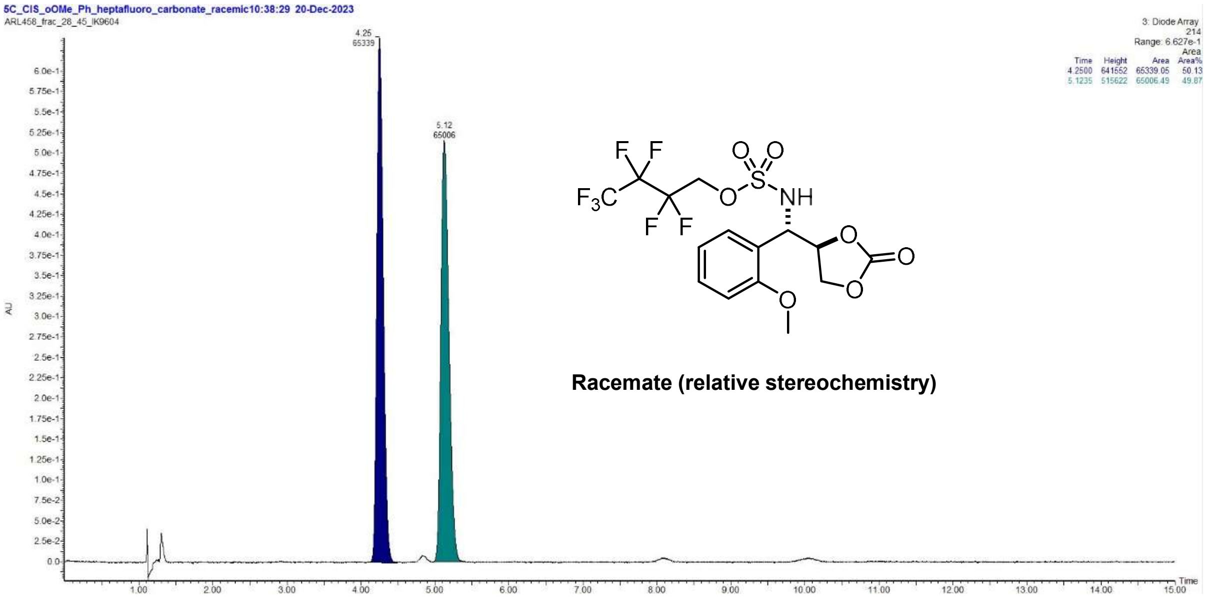

2,2,3,3,4,4,4-heptafluorobutyl ((*S*)-(2-fluorophenyl)((*R*)-2-oxo-1,3-dioxolan-4-yl)methyl)sulfamate (**4d**)

Chiral SFC Analysis CHIRALPAK IC (CO_2:MeOH, 98:2, 2.50 mL min^{-1}, 40 ˚C, 202 nm) indicated 90% ee, t_R = 6.6 (major), 7.4 (minor) minutes.

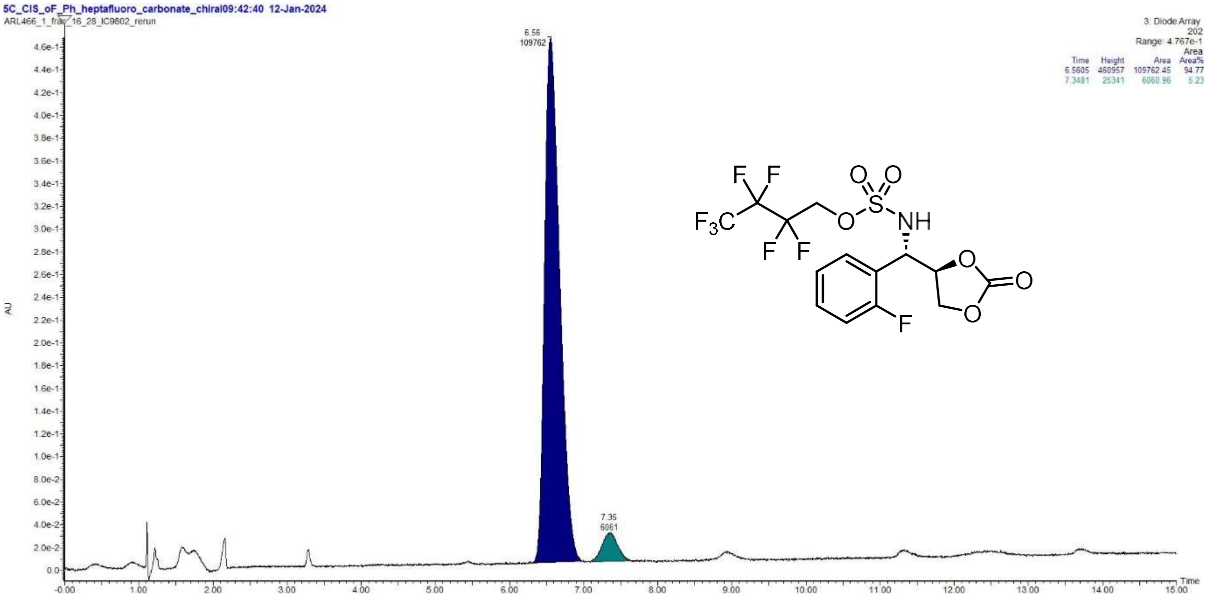

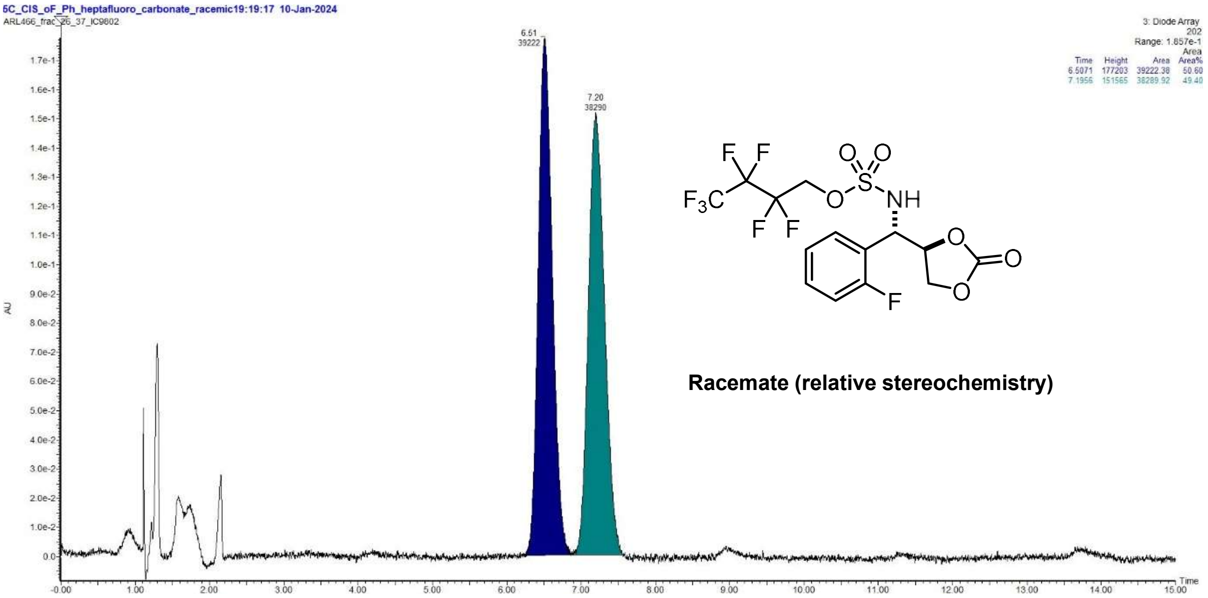

2,2,3,3,4,4,4-heptafluorobutyl ((*S*)-((*R*)-2-oxo-1,3-dioxolan-4-yl)(o-tolyl)methyl)sulfamate (**4e**)

Chiral SFC Analysis CHIRALPAK IK (CO_2:MeOH, 96:4, 2.50 mL min^{-1}, 40 ˚C, 207 nm) indicated 92% *ee*, t_R = 3.8 (minor), 4.3 (major) minutes.

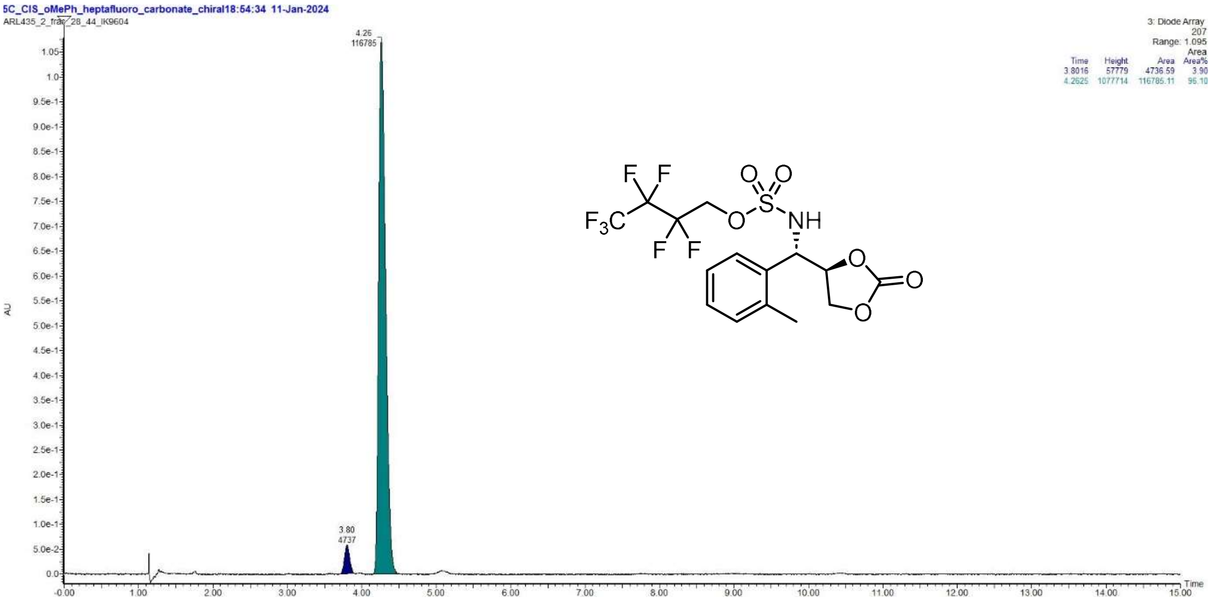

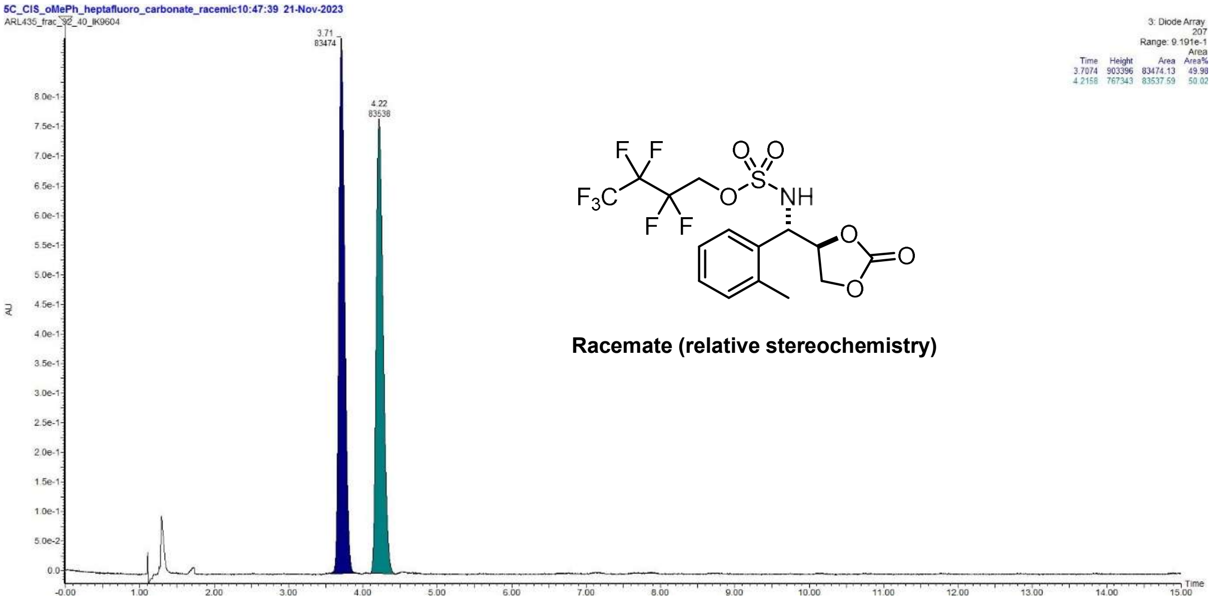

2,2,3,3,4,4,4-heptafluorobutyl ((*S*)-(3-((*tert*-butoxycarbonyl)(methyl)amino)phenyl)((*R*)-2-oxo-1,3-dioxolan-4-yl)methyl)sulfamate (**4f**)

Chiral SFC Analysis CHIRALPAK IK (CO_2:MeOH, 96:4, 2.50 mL min^{-1}, 40 ˚C, 237 nm) indicated 80% *ee*, t_R = 4.7 (minor), 5.1 (major) minutes.

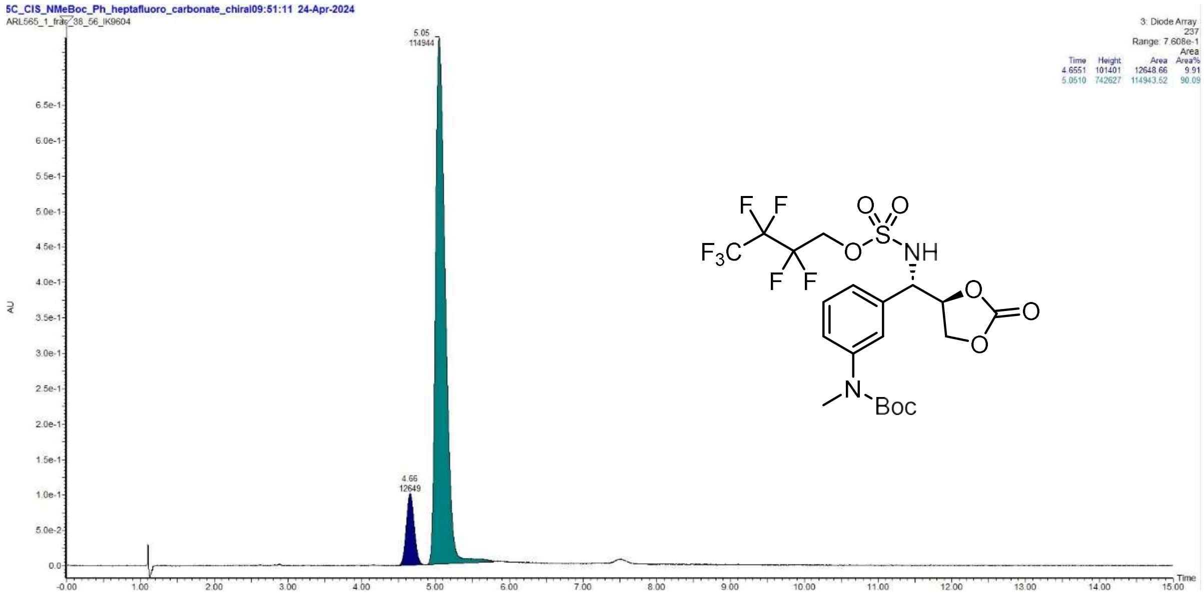

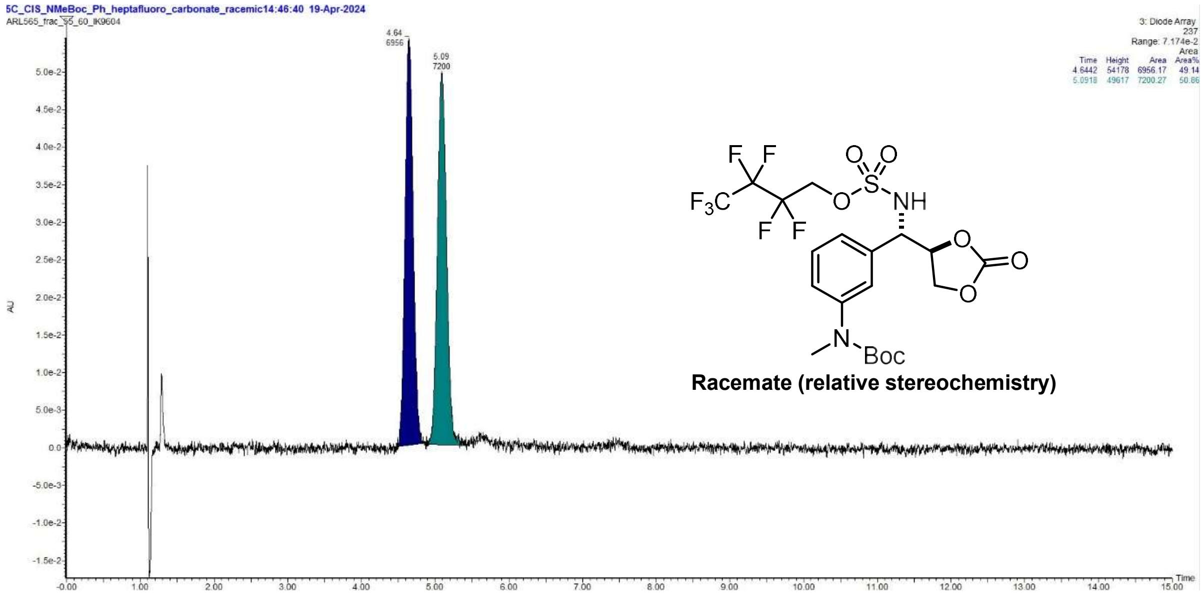

2,2,3,3,4,4,4-heptafluorobutyl ((*S*)-(3-(*tert*-butyl)phenyl)((*R*)-2-oxo-1,3-dioxolan-4-yl)methyl)sulfamate (**4g**)

Chiral SFC Analysis CHIRALPAK IK (CO_2:MeOH, 97:3, 2.50 mL min^{-1}, 40 ˚C, 208 nm) indicated 93% *ee*, t_R = 4.4 (minor), 5.2 (major) minutes.

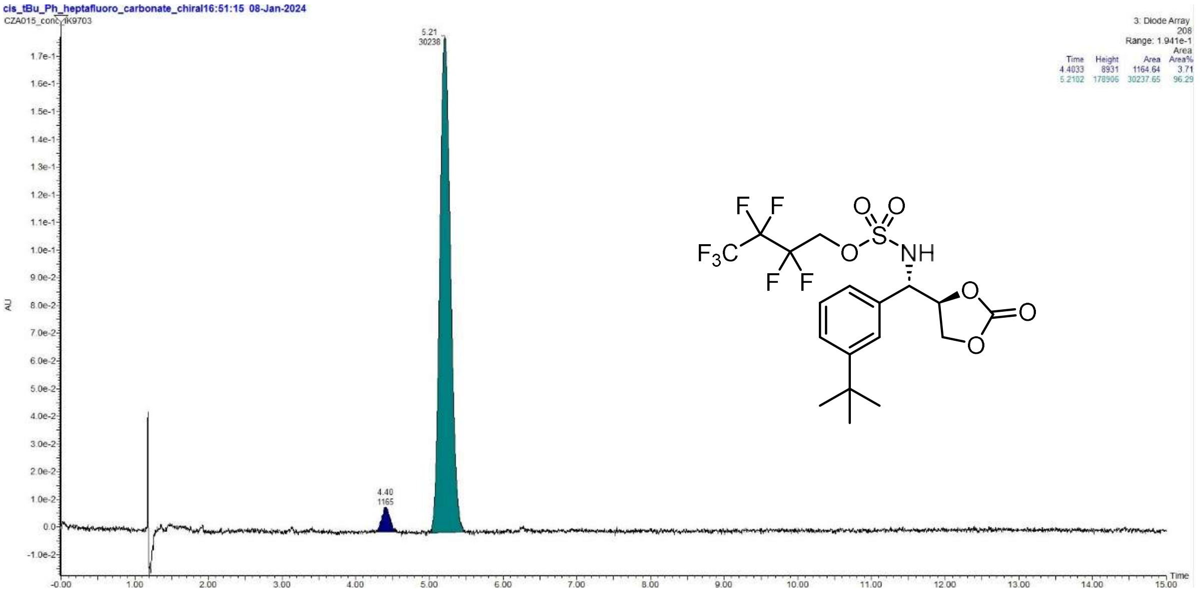

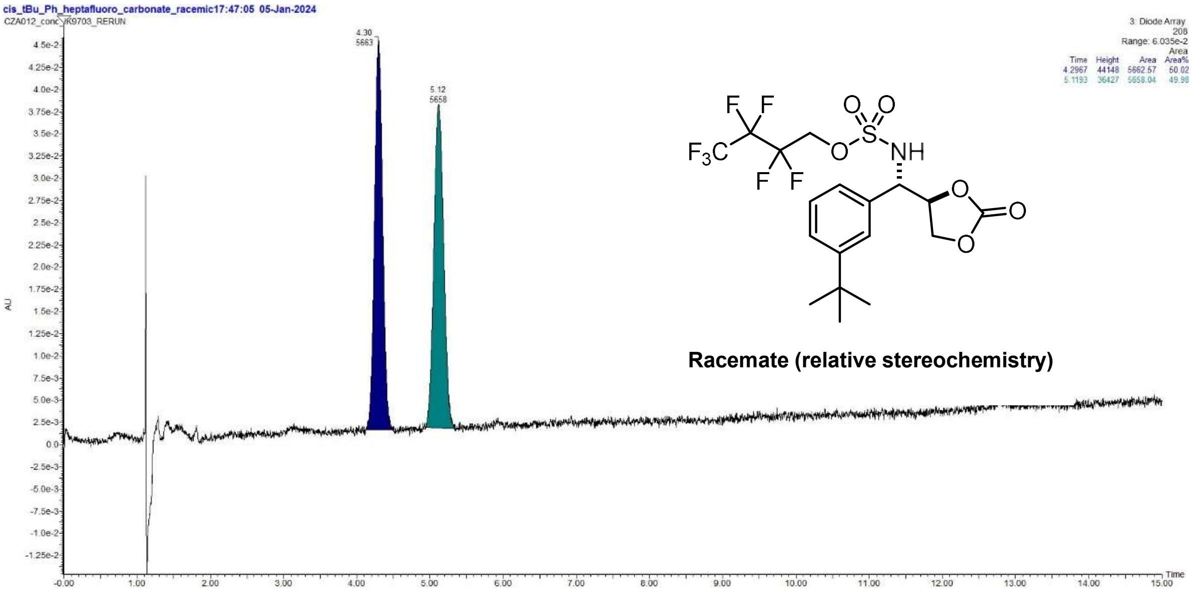

2,2,3,3,4,4,4-heptafluorobutyl ((*S*)-(3-methoxyphenyl)((*R*)-2-oxo-1,3-dioxolan-4-yl)methyl)sulfamate (**4h**)

Chiral SFC Analysis CHIRALPAK IK (CO_2:MeOH, 96:4, 2.50 mL min^{-1}, 40 ˚C, 219 nm) indicated 92% *ee*, t_R = 4.7 (minor), 5.3 (major) minutes.

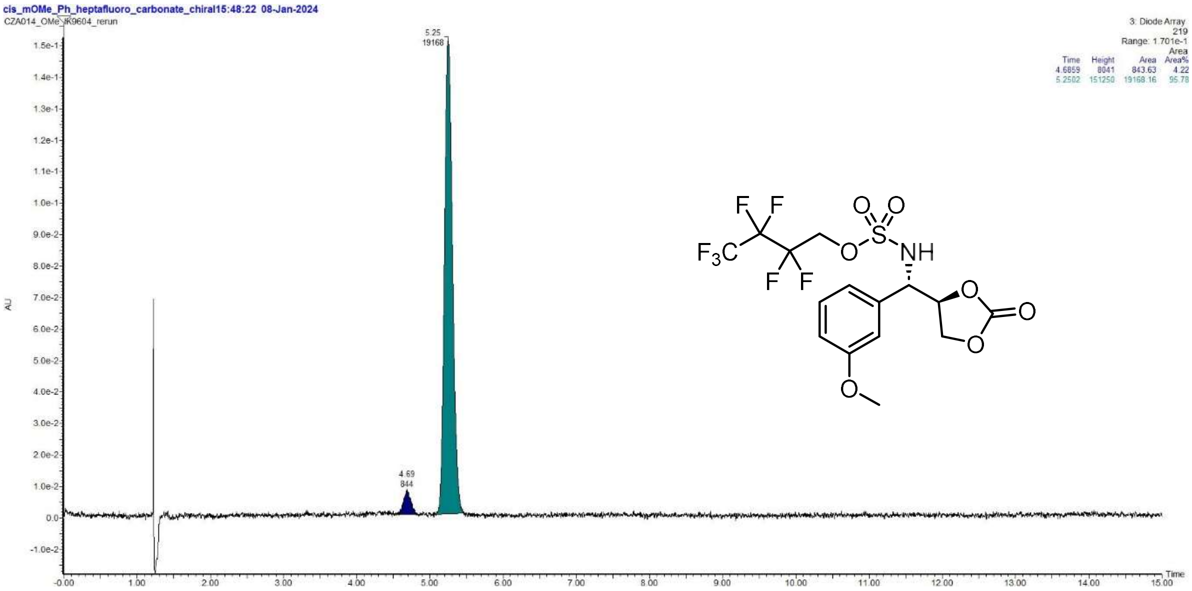

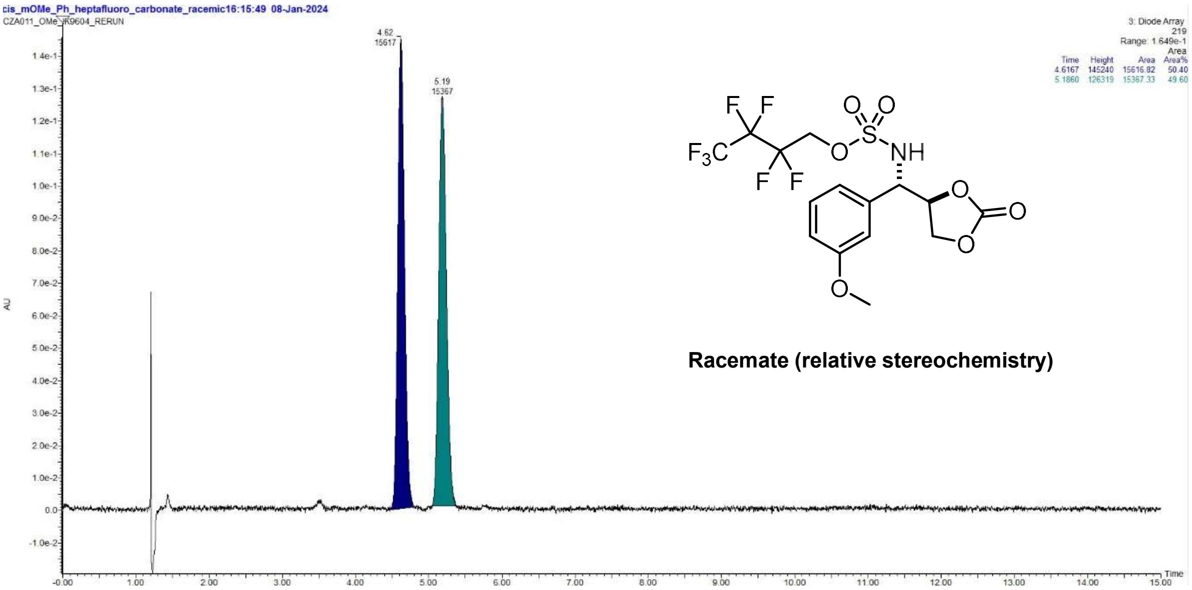

2,2,3,3,4,4,4-heptafluorobutyl ((*S*)-(3-bromophenyl)((*R*)-2-oxo-1,3-dioxolan-4-yl)methyl)sulfamate (**4i**)

Chiral SFC Analysis CHIRALPAK IJ (CO_2:MeOH, 95:5, 2.50 mL min^{-1}, 40 ˚C, 203 nm) indicated 87% *ee*, t_R = 4.0 (major), 4.4 (minor) minutes.

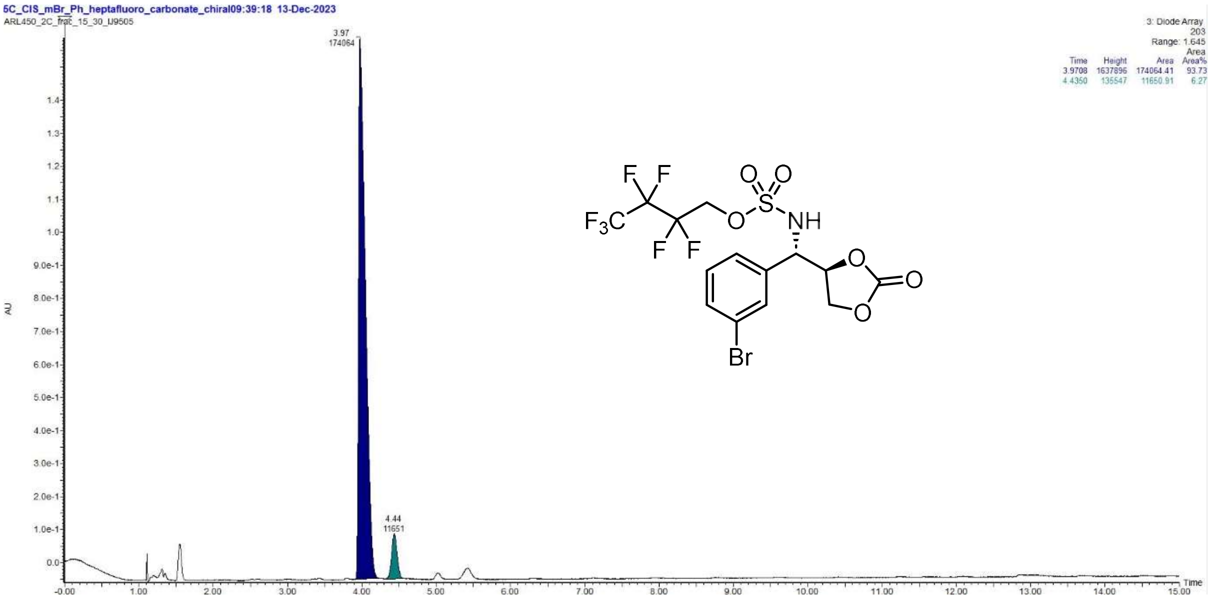

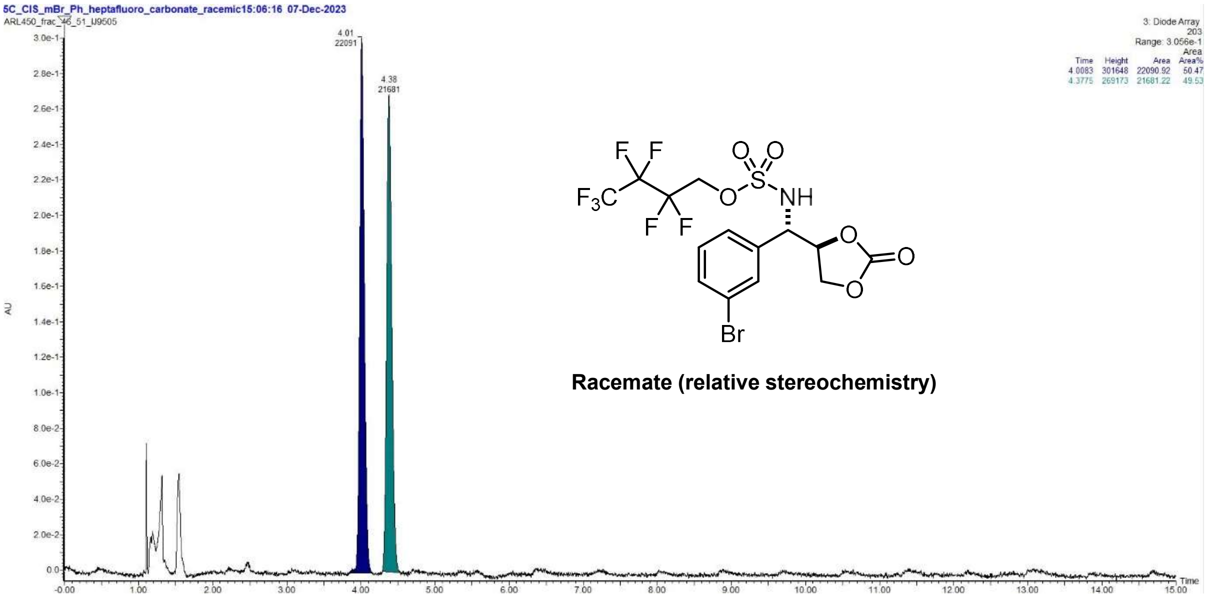

ethyl 3-((*S*)-(((2,2,3,3,4,4,4-heptafluorobutoxy)sulfonyl)amino)((*R*)-2-oxo-1,3-dioxolan-4-yl)methyl)benzoate (**4j**)

Chiral SFC Analysis CHIRALPAK IC (CO_2:MeOH, 96:4, 2.50 mL min^{-1}, 40 ˚C, 227 nm) indicated 88% *ee*, t_R = 4.7 (major), 5.9 (minor) minutes.

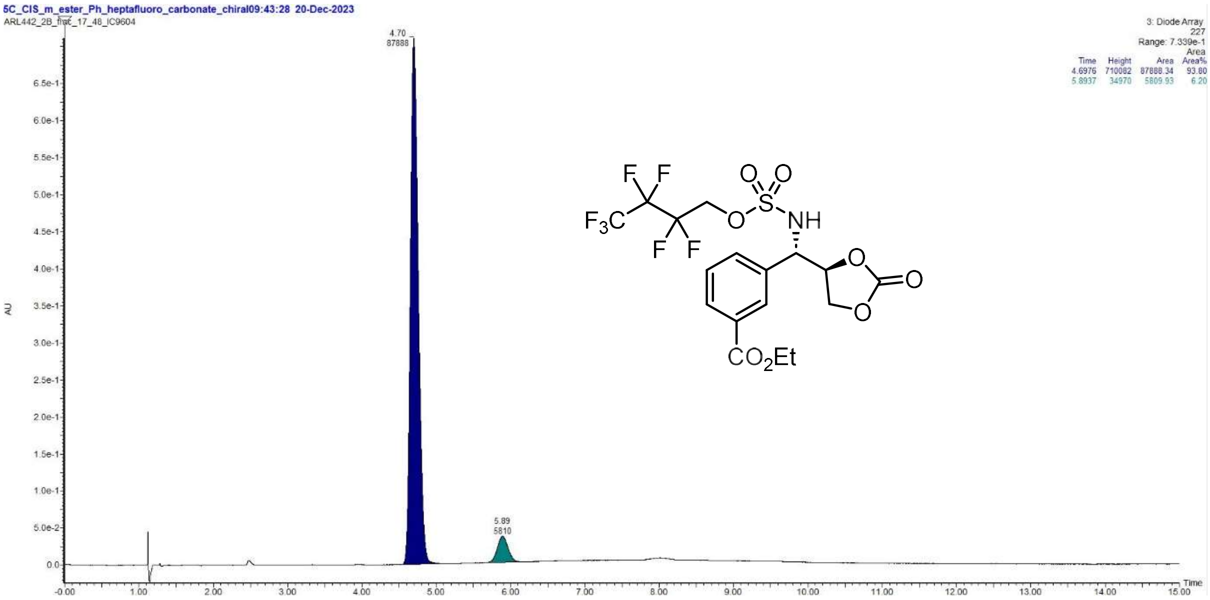

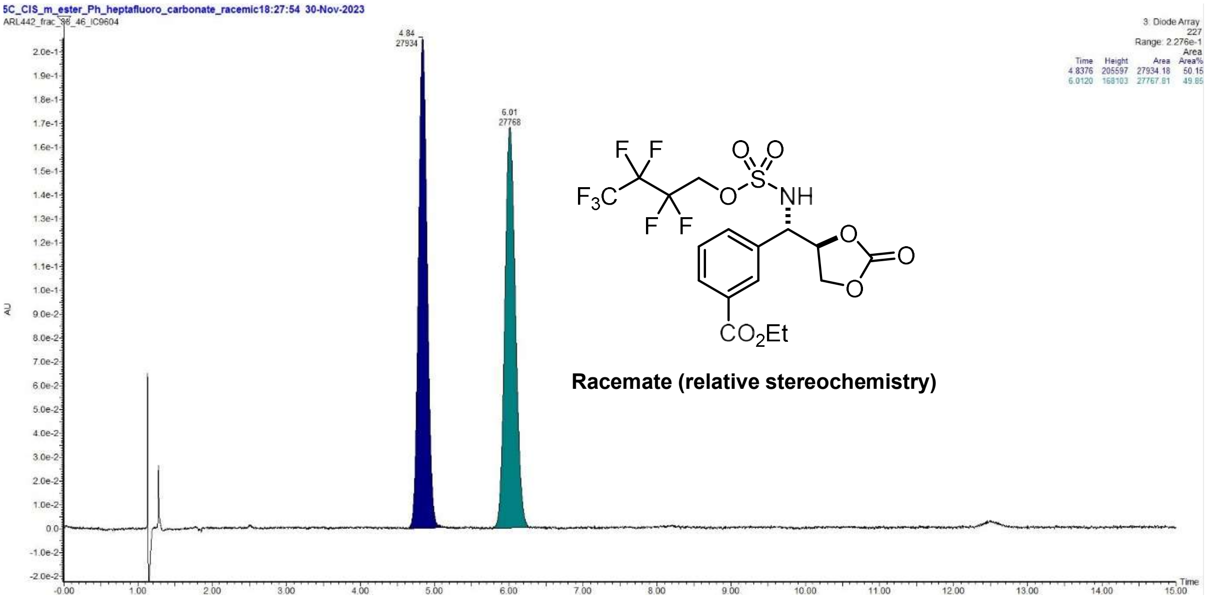

2,2,3,3,4,4,4-heptafluorobutyl ((*S*)-(3,4-dichlorophenyl)((*R*)-2-oxo-1,3-dioxolan-4-yl)methyl)sulfamate (**4k**)

Chiral SFC Analysis CHIRALPAK IG (CO_2:MeOH, 95:5, 2.50 mL min^{-1}, 40 ˚C, 201 nm) indicated 90% *ee*, t_R = 6.7 (minor), 7.3 (major) minutes.

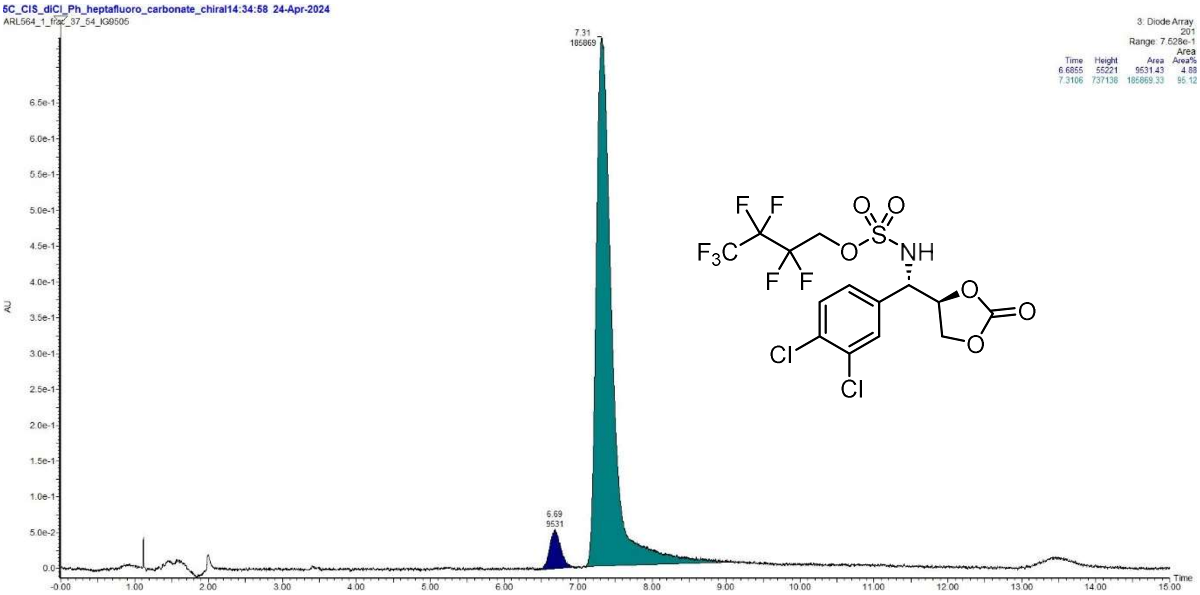

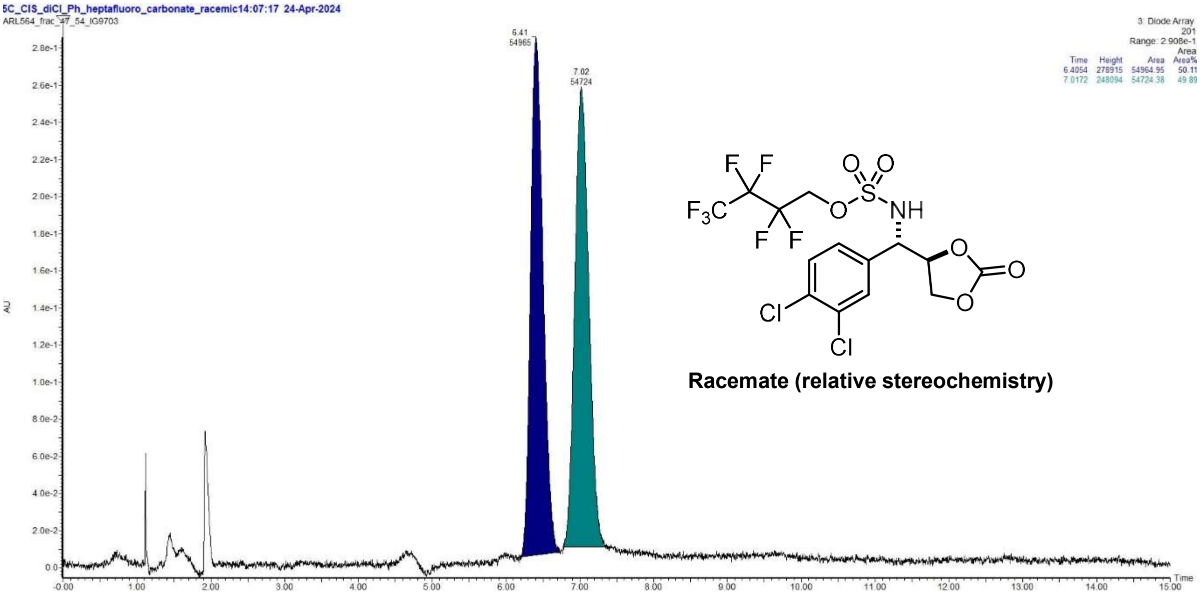

2,2,3,3,4,4,4-heptafluorobutyl ((*S*)-(4-(*tert*-butyl)phenyl)((*R*)-2-oxo-1,3-dioxolan-4-yl)methyl)sulfamate (**4l**)

Chiral SFC Analysis CHIRALPAK IK (CO_2:MeOH, 97:3, 2.50 mL min^{-1}, 40 ˚C, 215 nm) indicated 97% *ee*, t_R = 6.1 (minor), 6.7 (major) minutes.

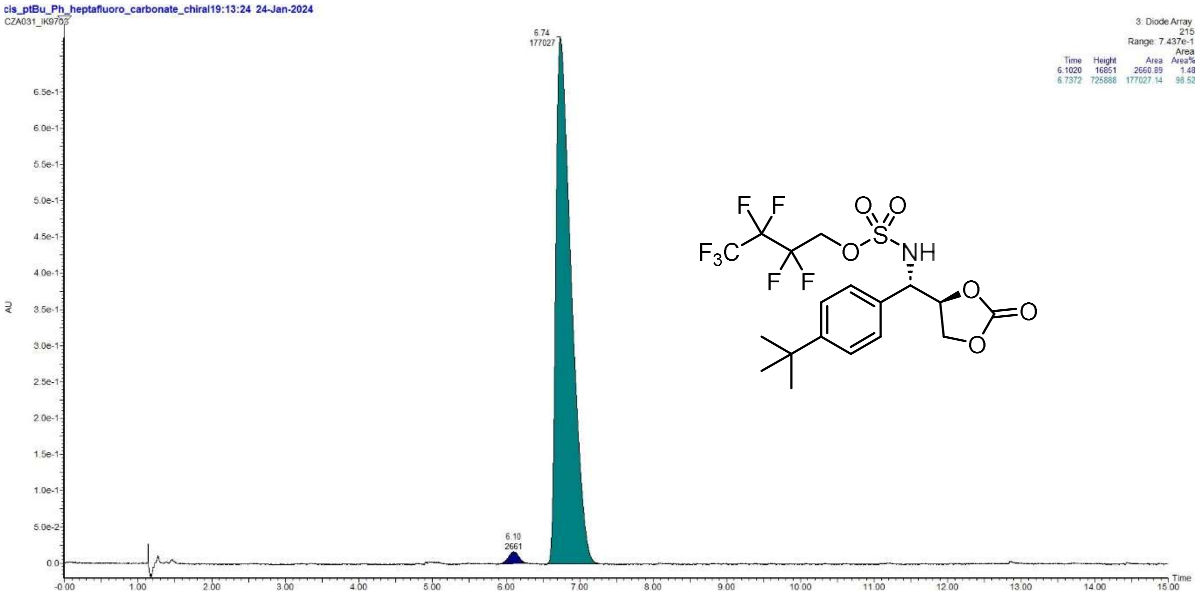

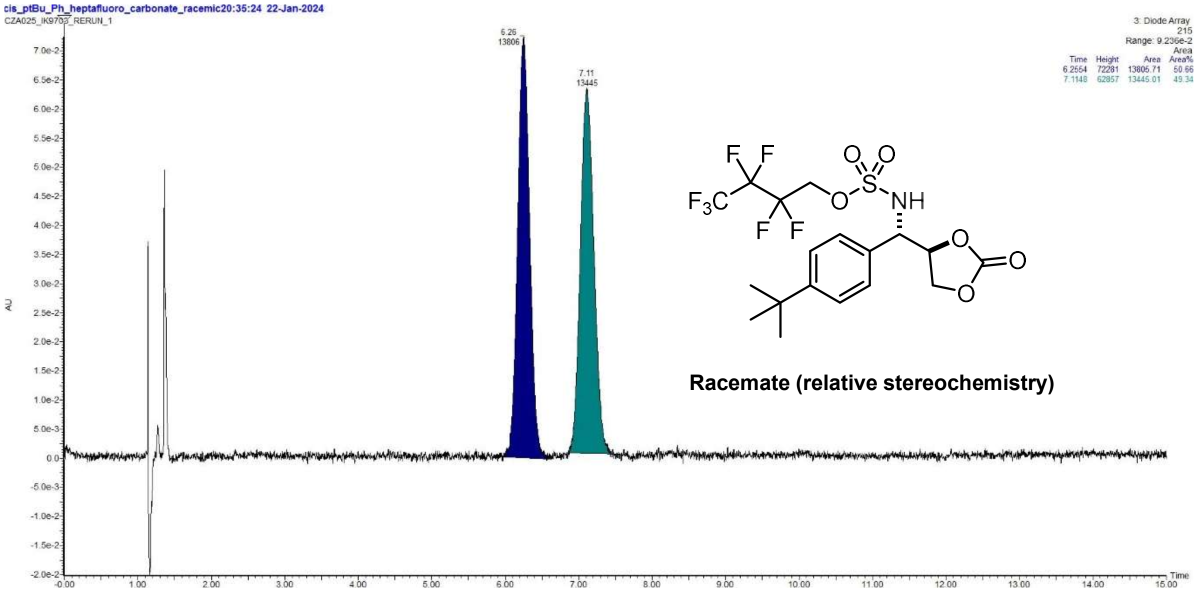

2,2,3,3,4,4,4-heptafluorobutyl ((*S*)-((*R*)-2-oxo-1,3-dioxolan-4-yl)(4-(trifluoromethoxy)phenyl)methyl)sulfamate (**4m**)

Chiral SFC Analysis CHIRALPAK IJ (CO_2:MeOH, 97:3, 2.50 mL min^{-1}, 40 ˚C, 203 nm) indicated 92% *ee*, t_R = 6.7 (major), 7.6 (minor) minutes.

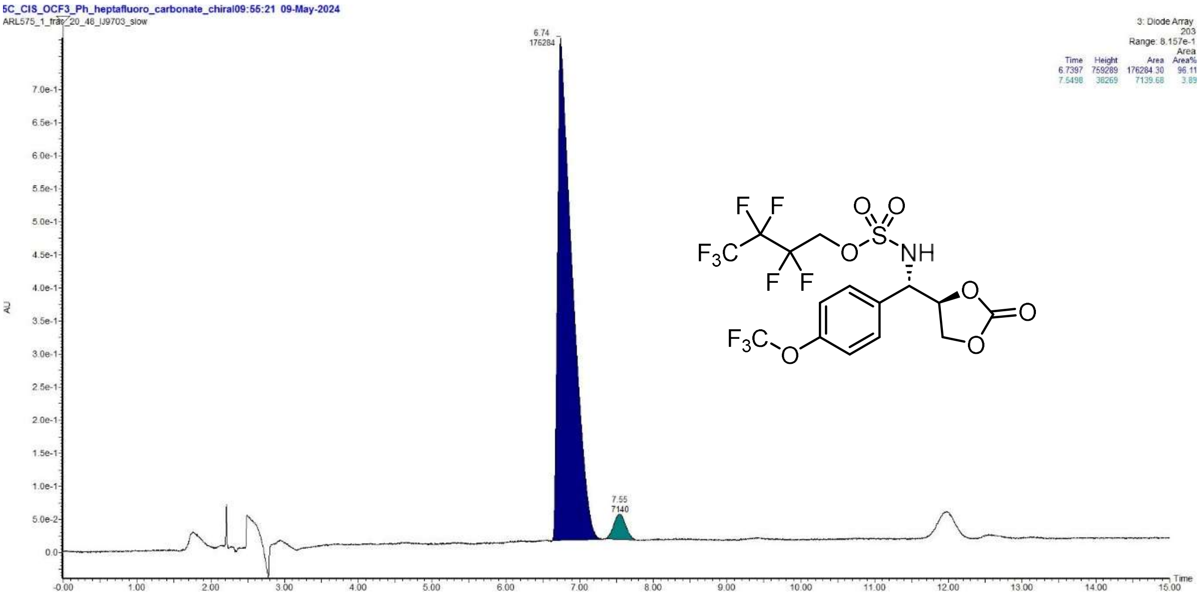

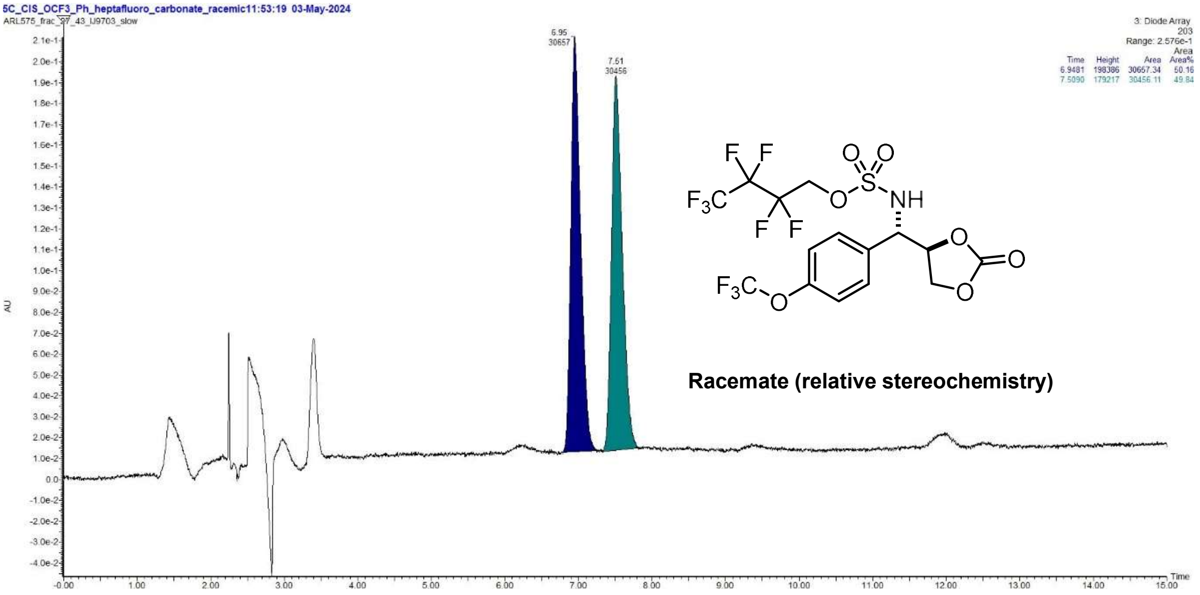

2,2,3,3,4,4,4-heptafluorobutyl ((*S*)-(4-chlorophenyl)((*R*)-2-oxo-1,3-dioxolan-4-yl)methyl)sulfamate (**4n**)

Chiral SFC Analysis CHIRALPAK IJ (CO_2:MeOH, 97:3, 2.50 mL min^{-1}, 40 ˚C, 218 nm) indicated 90% *ee*, t_R = 6.4 (major), 7.6 (minor) minutes.

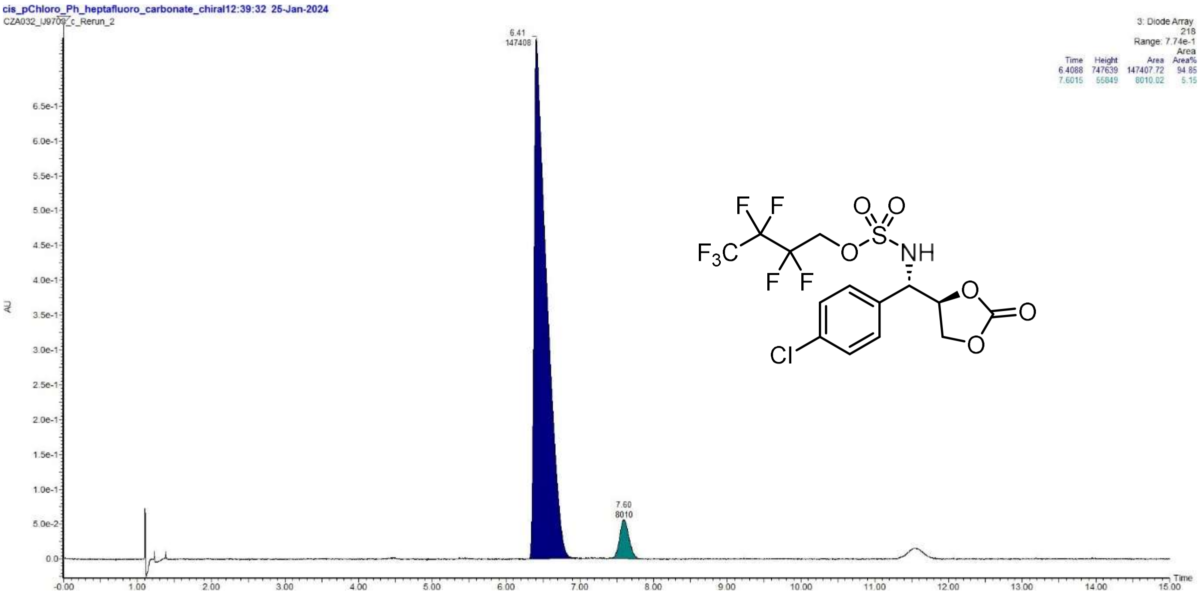

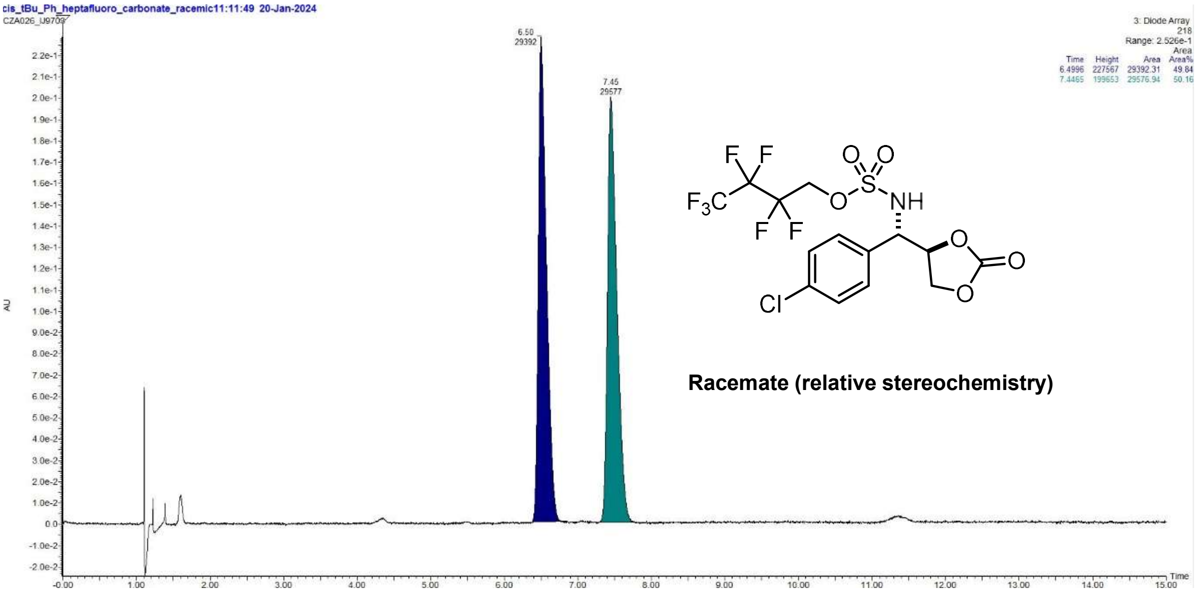

2,2,3,3,4,4,4-heptafluorobutyl ((*S*)-((*R*)-2-oxo-1,3-dioxolan-4-yl)(4-(trifluoromethyl)phenyl)methyl)sulfamate (**4o**)

Chiral SFC Analysis CHIRALPAK IJ (CO_2:MeOH, 97:3, 2.50 mL min^{-1}, 40 °C, 206 nm) indicated 95% *ee*, t_R = 3.8 (major), 4.4 (minor) minutes.

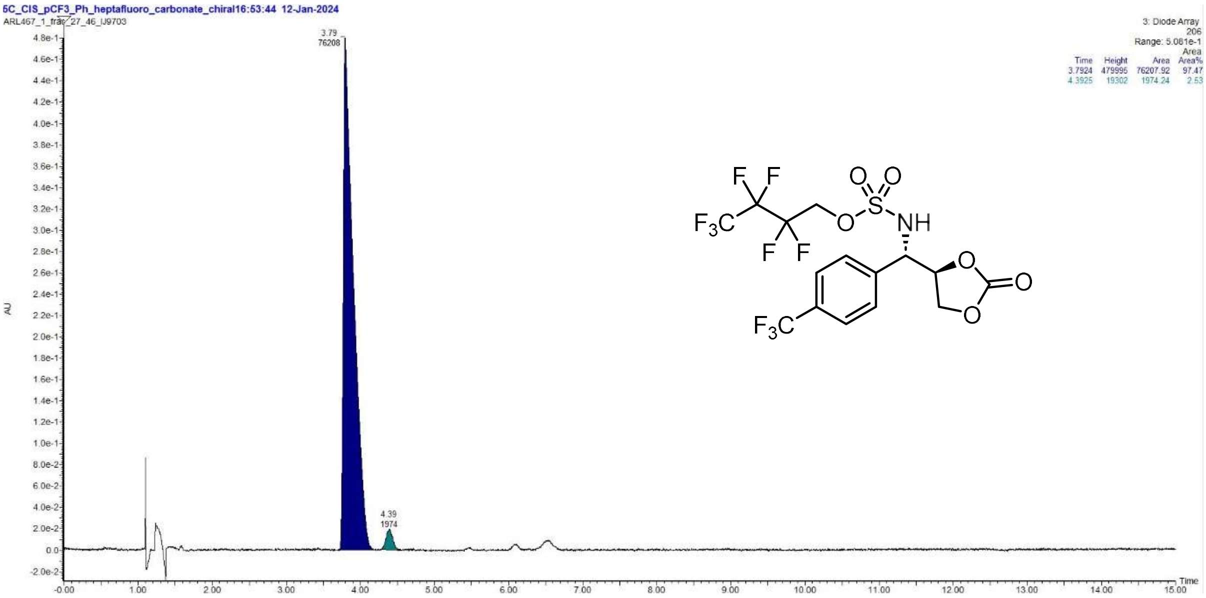

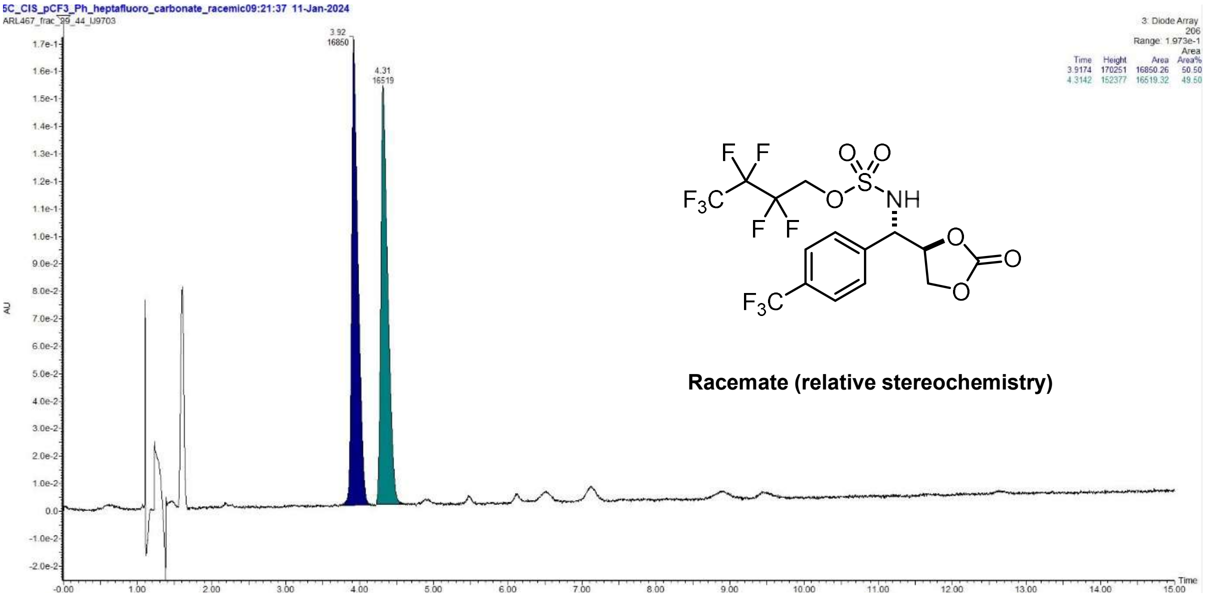

2,2,3,3,4,4,4-heptafluorobutyl ((*S*)-naphthalen-1-yl((*R*)-2-oxo-1,3-dioxolan-4-yl)methyl)sulfamate (**4p**)

Chiral SFC Analysis CHIRALPAK IJ (CO_2:MeOH, 94:6, 2.50 mL min^{-1}, 40 ˚C, 273 nm) indicated 92% *ee*, t_R = 3.9 (major), 4.8 (minor) minutes.

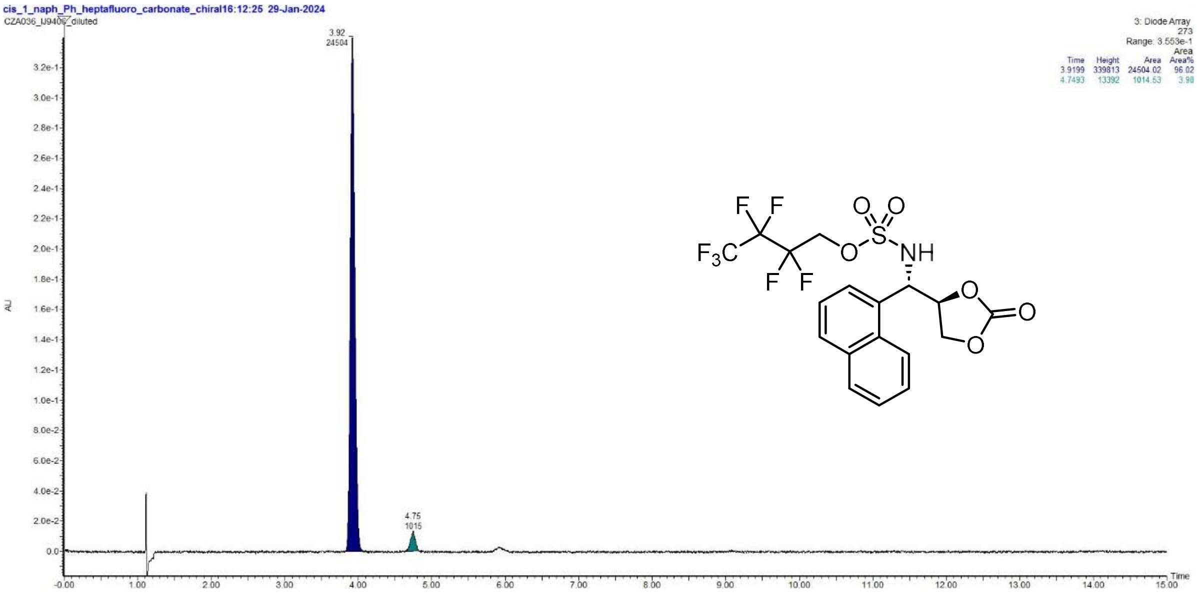

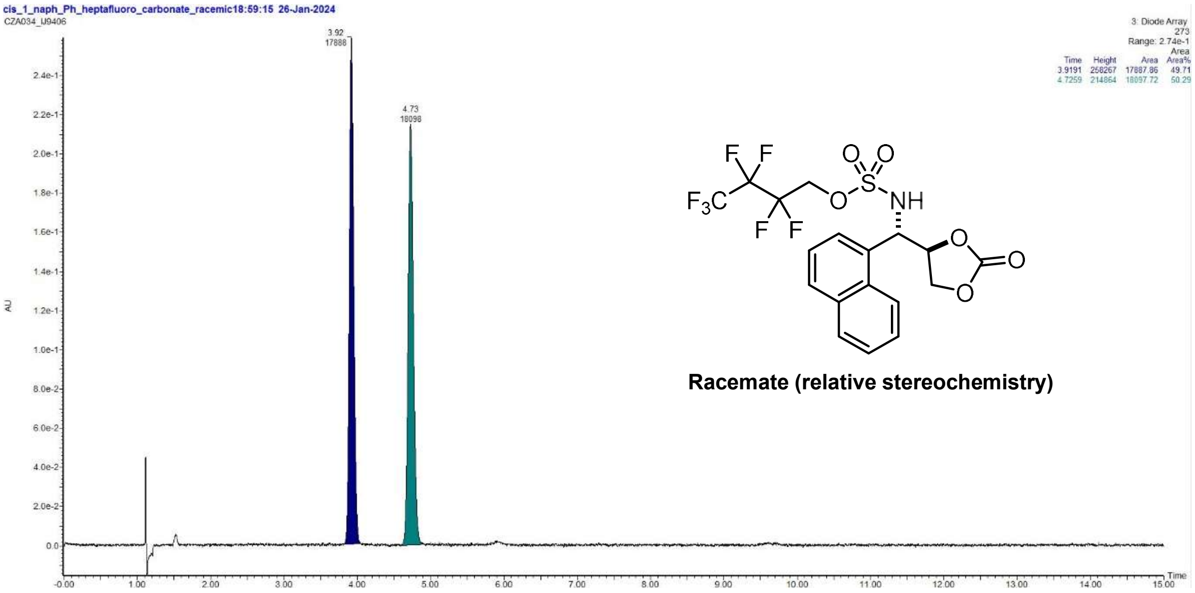

2,2,3,3,4,4,4-heptafluorobutyl ((*S*)-naphthalen-2-yl((*R*)-2-oxo-1,3-dioxolan-4-yl)methyl)sulfamate (**4q**)

Chiral SFC Analysis CHIRALPAK IJ (CO_2:MeOH, 94:6, 2.50 mL min^{-1}, 40 ˚C, 273 nm) indicated 84% *ee*, t_R = 5.6 (major), 8.0 (minor) minutes.

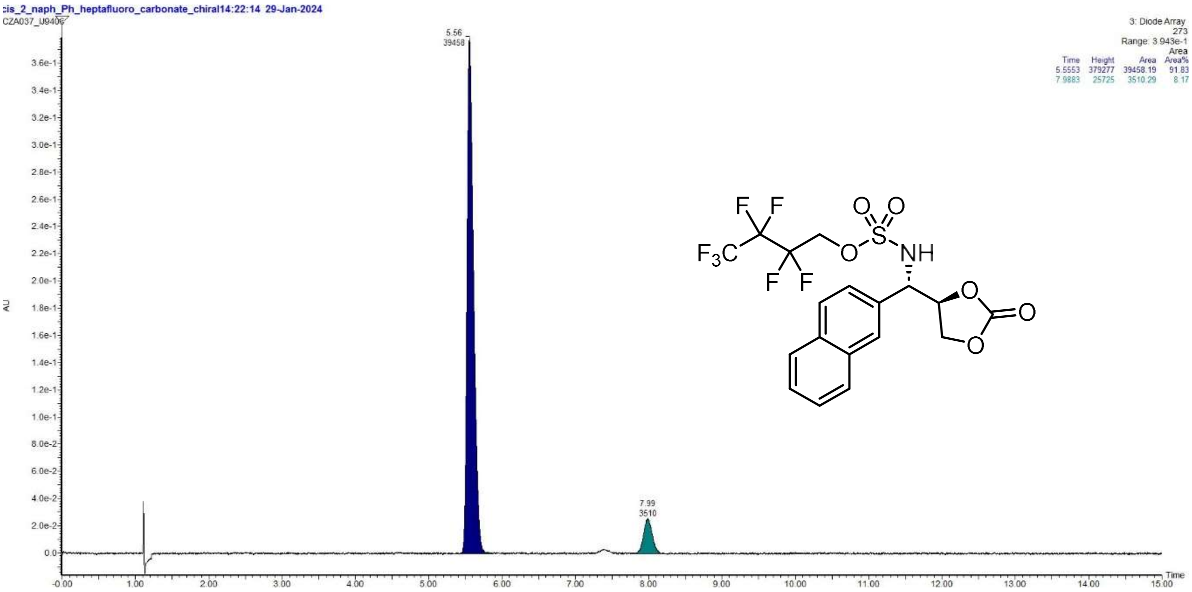

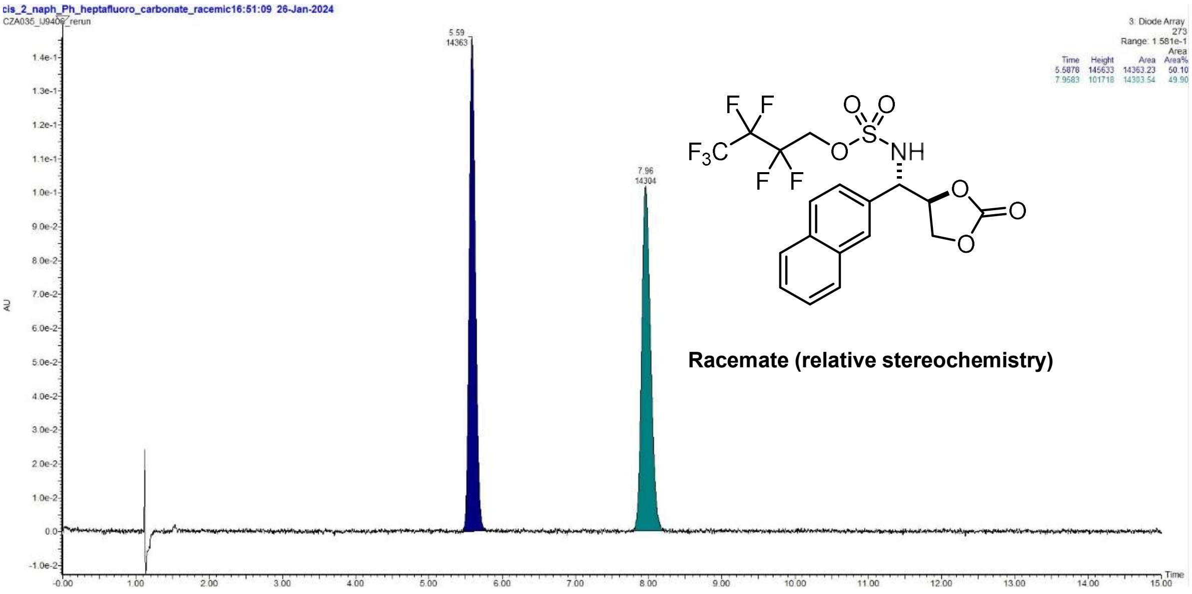

2,2,3,3,4,4,4-heptafluorobutyl ((*S*)-((*R*)-2-oxo-1,3-dioxolan-4-yl)(thiophen-3-yl)methyl)sulfamate (**4r**)

Chiral SFC Analysis CHIRALPAK IK (CO_2:MeOH, 97:3, 2.50 mL min^{-1}, 40 ˚C, 231 nm) indicated 92% *ee*, t_R = 5.9 (minor), 6.5 (major) minutes.

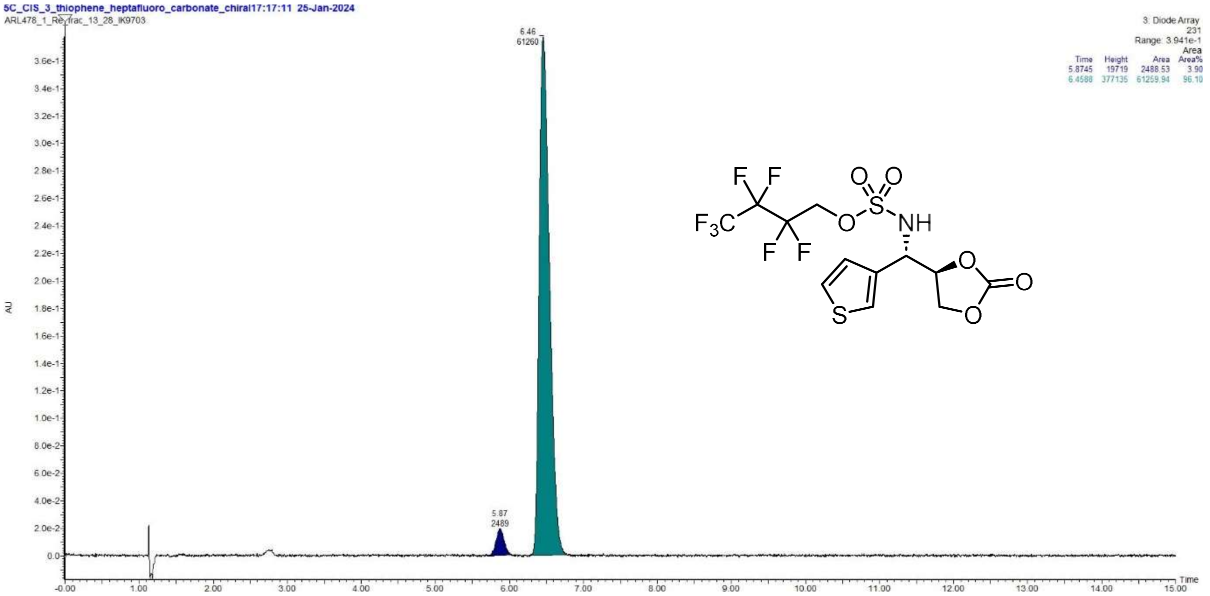

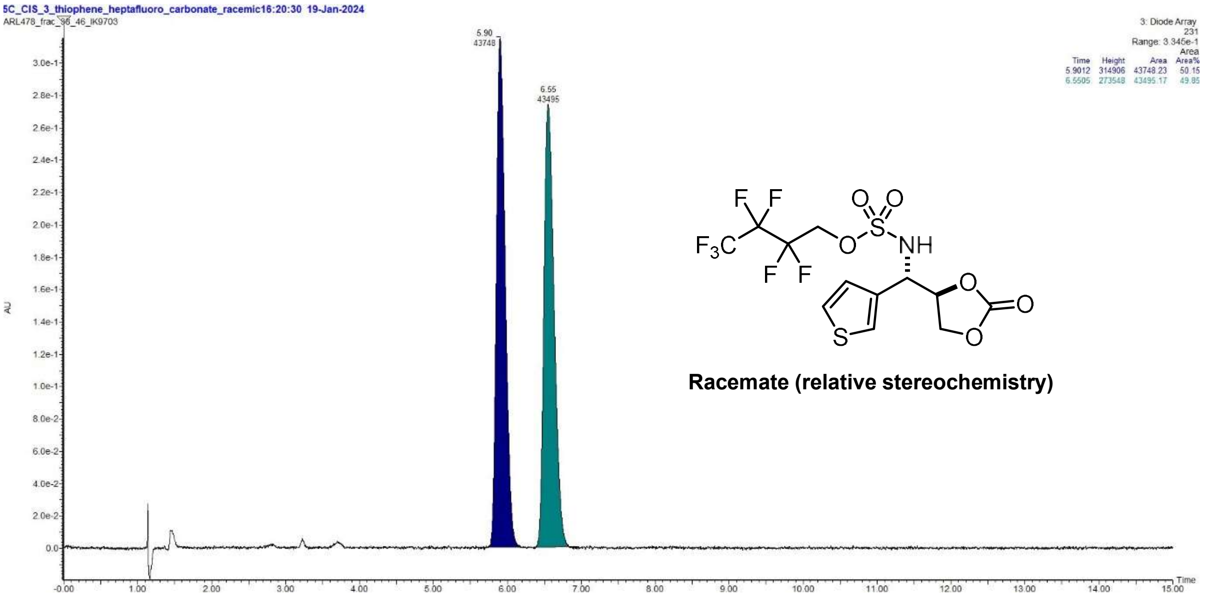

methyl 5-((*S*)-(((2,2,3,3,4,4,4-heptafluorobutoxy)sulfonyl)amino)((*R*)-2-oxo-1,3-dioxolan-4-yl)methyl)-1-tosyl-1*H*-indole-3-carboxylate (**4s**)

Chiral SFC Analysis CHIRALPAK IC (CO_2:MeOH, 85:15, 2.50 mL min^{-1}, 40 ˚C, 266 nm) indicated 91% *ee*, t_R = 4.3 (minor), 14.9 (major) minutes.

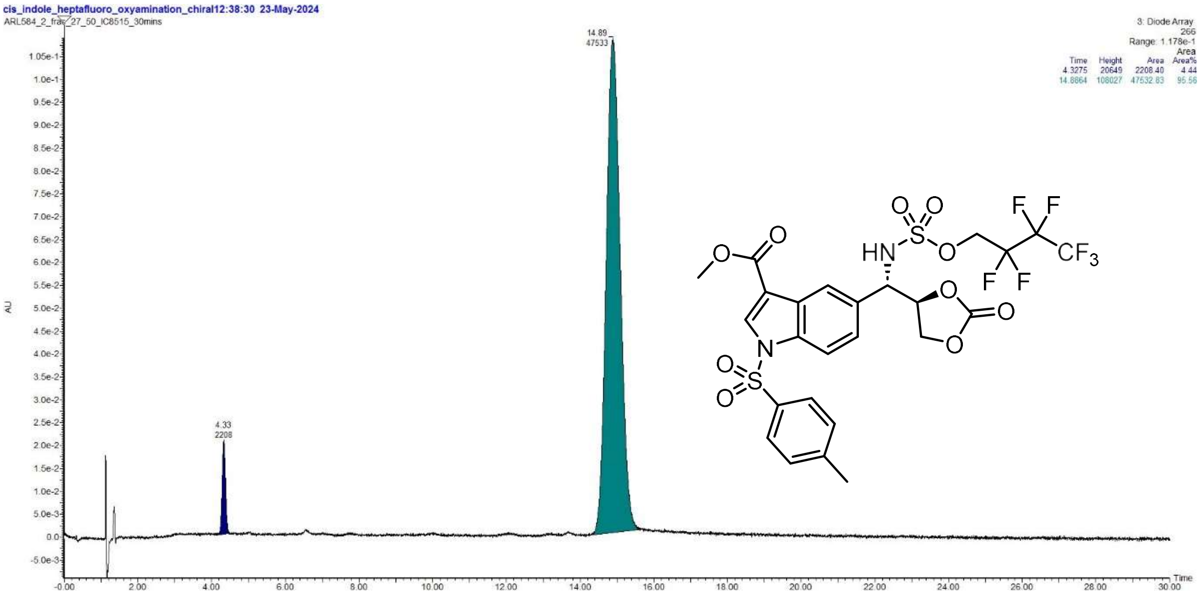

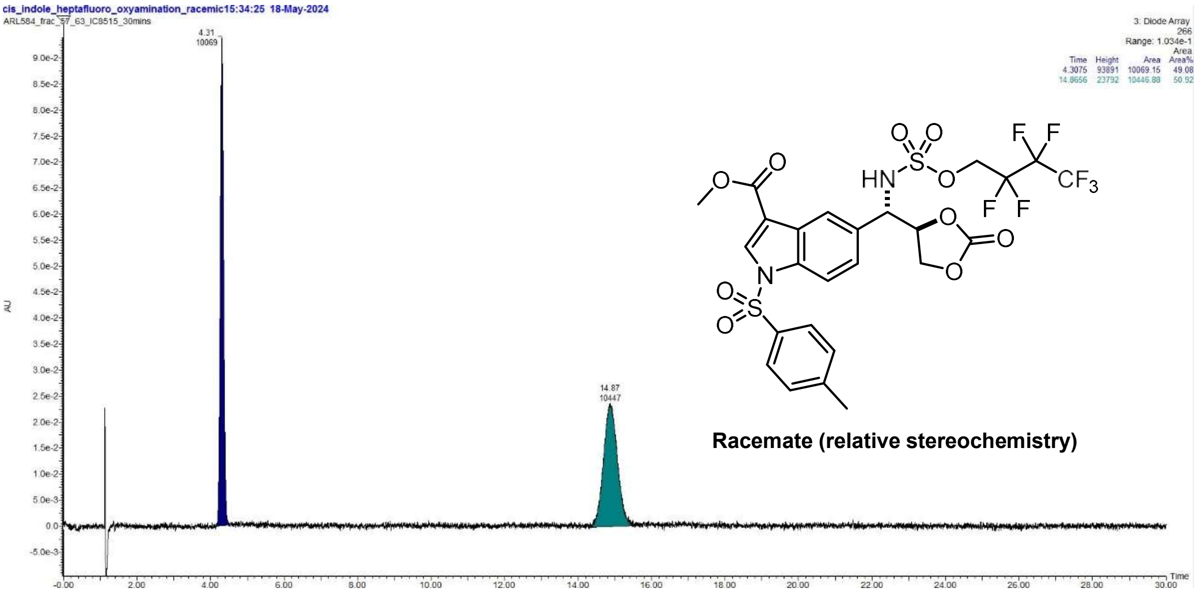

(R,Z)-4-(4-(*tert*-butyl)phenyl)but-3-en-2-yl methyl(phenyl)carbamate ((***R***)-**1t**)

Chiral SFC Analysis CHIRALPAK IJ (CO_2:MeOH, 94:6, 2.50 mL min^{-1}, 40 ˚C, 241 nm) indicated 94% *ee*, t_R = 3.4 (major), 5.7 (minor) minutes.

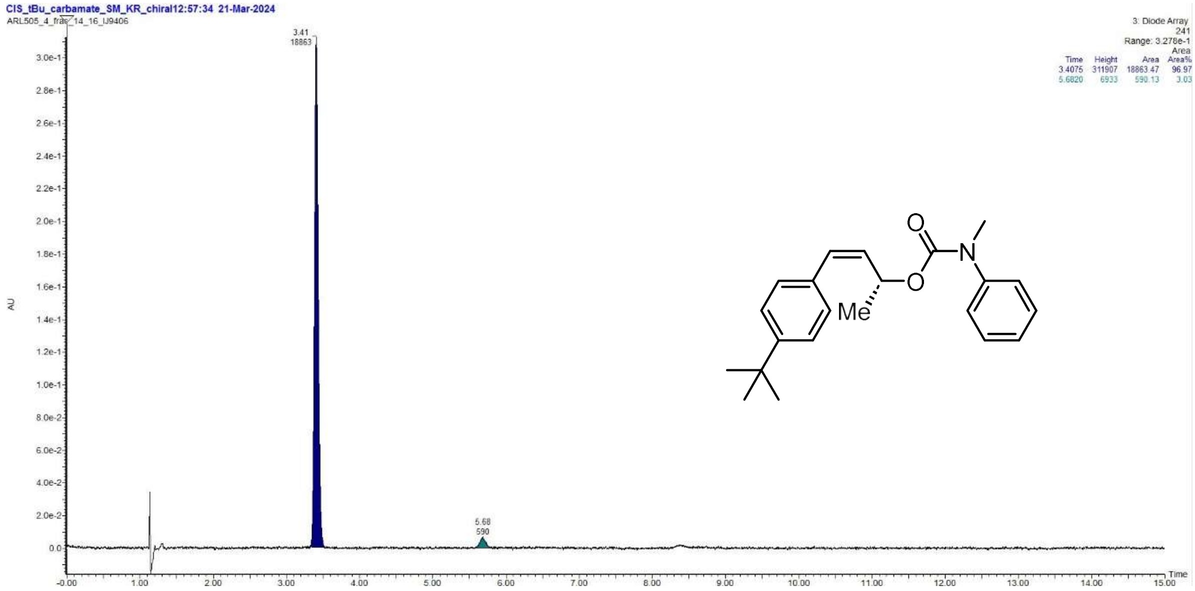

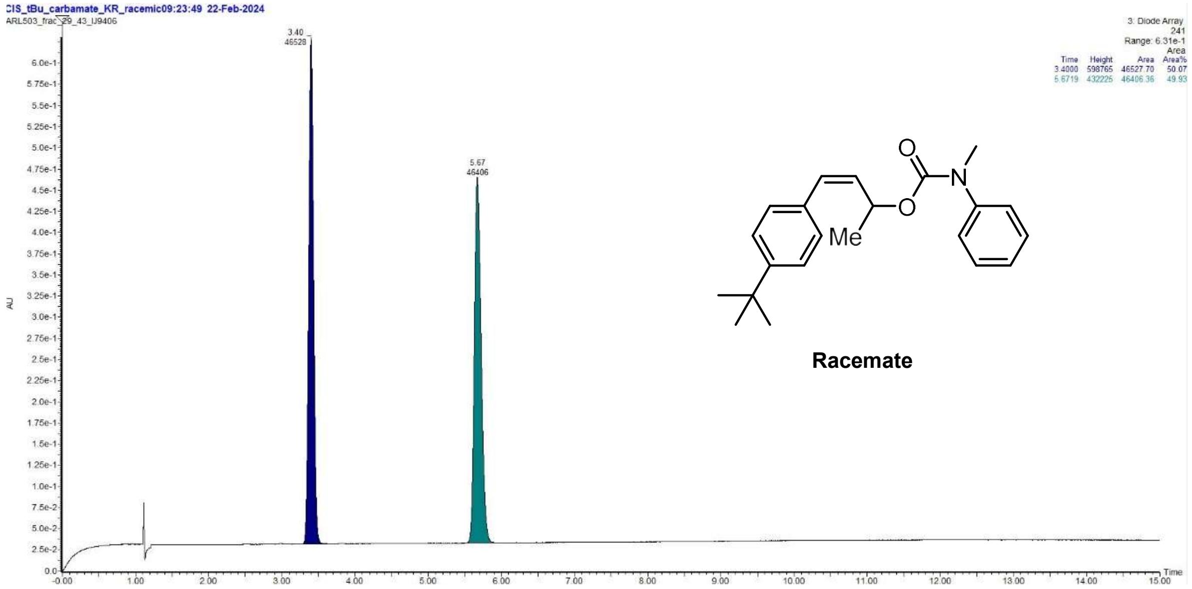

2,2,3,3,4,4,4-heptafluorobutyl ((*S*)-(4-(*tert*-butyl)phenyl)((4*R*,5S)-5-methyl-2-oxo-1,3-dioxolan-4-yl)methyl)sulfamate (**4t**)

Chiral SFC Analysis CHIRALPAK IC (CO_2:MeOH, 97:3, 2.50 mL min^{-1}, 40 ˚C, 216 nm) indicated 97% *ee*, t_R = 6.0 (minor), 7.1 (major) minutes.

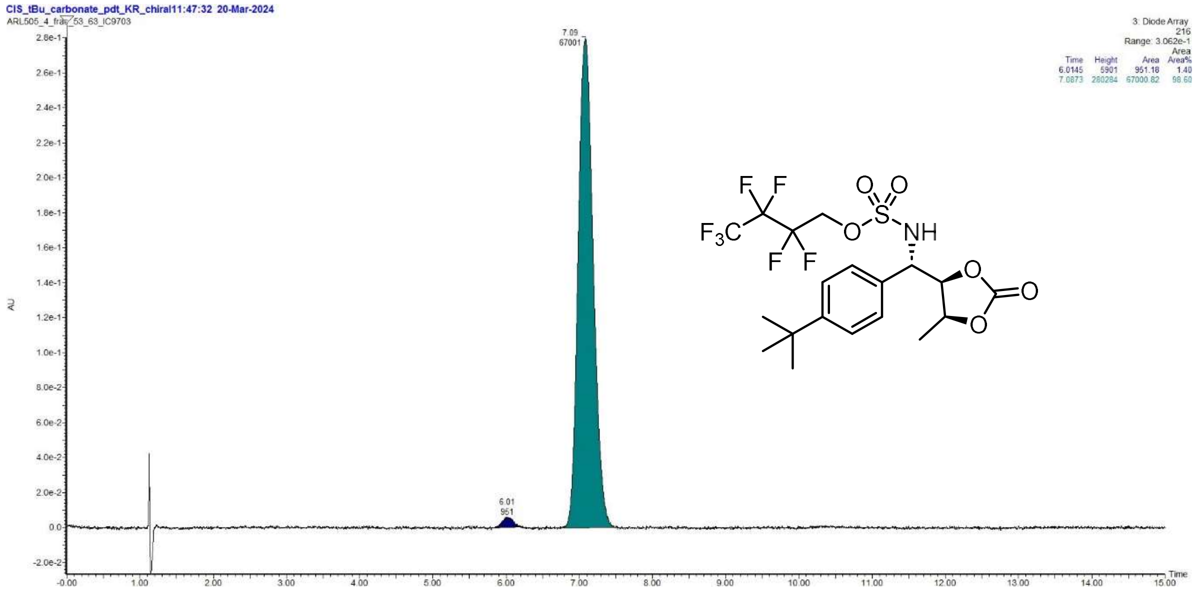

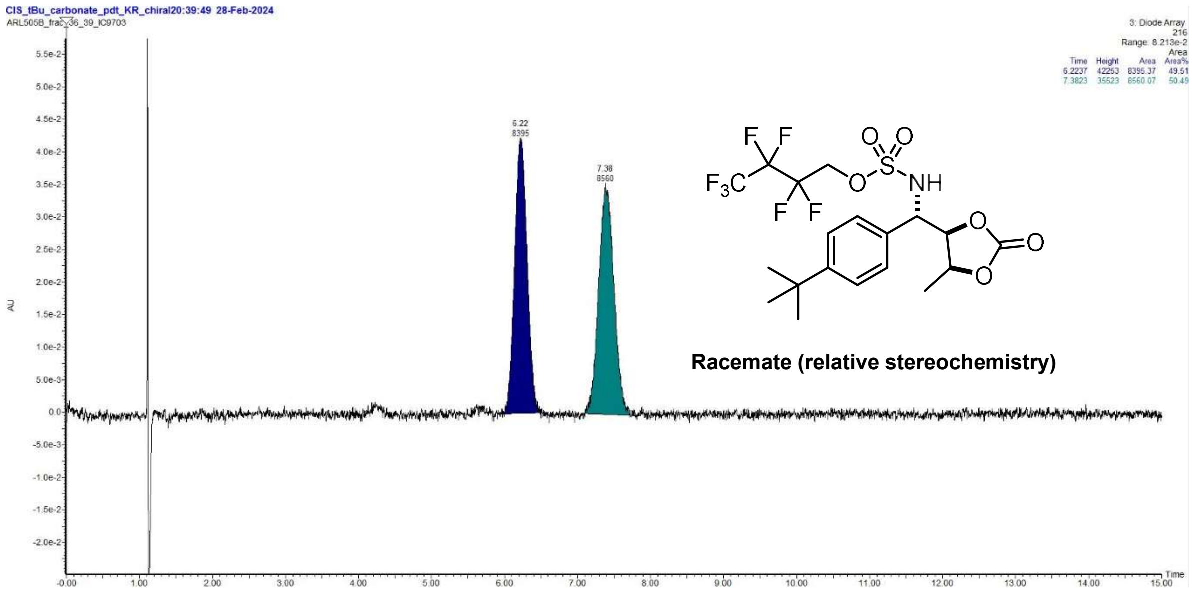

(R,Z)-1-(4-(*tert*-butyl)phenyl)-4-methylpent-1-en-3-yl methyl(phenyl)carbamate ((***R***)-**1u**)

Chiral SFC Analysis CHIRALPAK IC (CO_2:MeOH, 97:3, 1.25 mL min^{-1}, 40 ˚C, 240 nm) indicated 57% *ee*, t_R = 11.7 (minor), 12.8 (major) minutes.

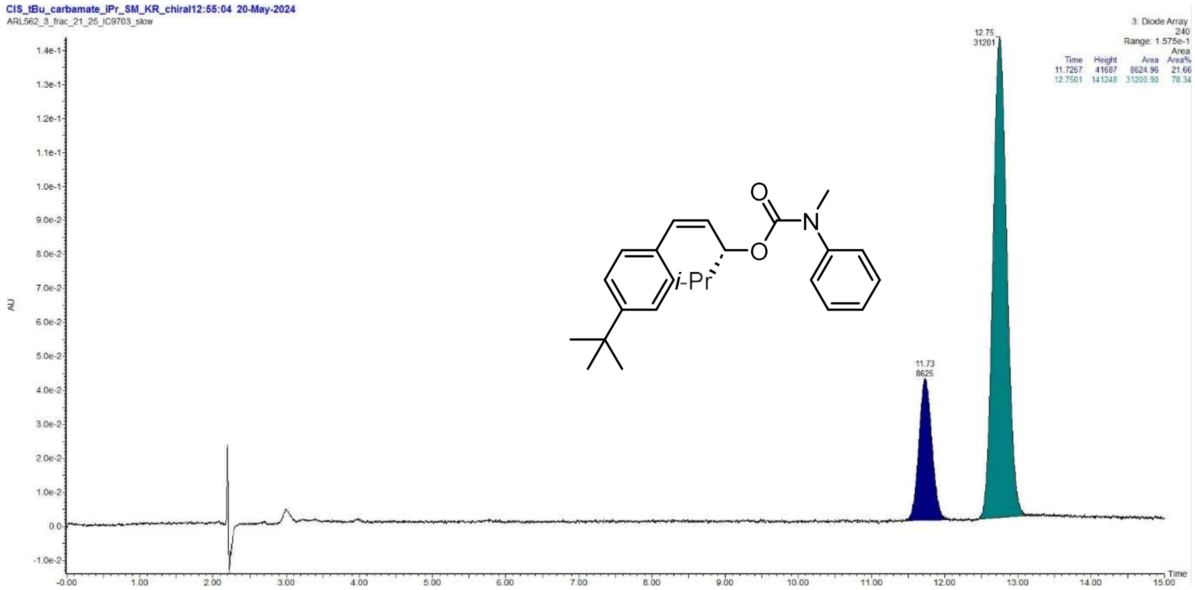

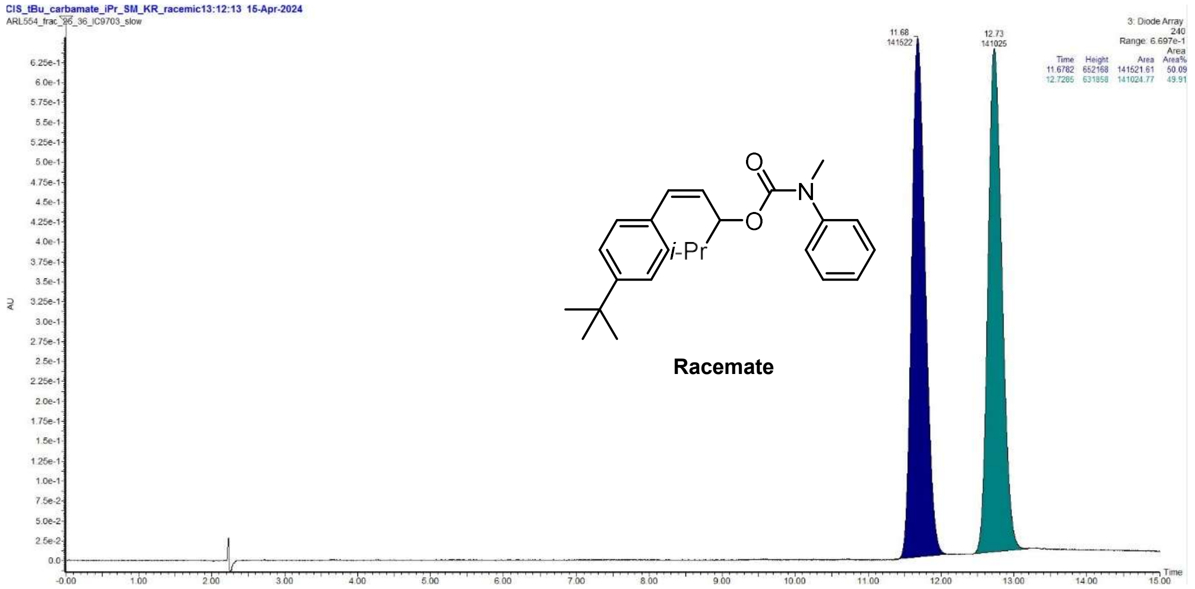

2,2,3,3,4,4,4-heptafluorobutyl ((*S*)-(4-(*tert*-butyl)phenyl)((4*R*,5S)-5-isopropyl-2-oxo-1,3-dioxolan-4-yl)methyl)sulfamate (**4u**)

Chiral SFC Analysis CHIRALPAK IK (CO_2:MeOH, 97:3, 1.25 mL min^{-1}, 40 ˚C, 216 nm) indicated 91% *ee*, t_R = 6.5 (minor), 8.2 (major) minutes.

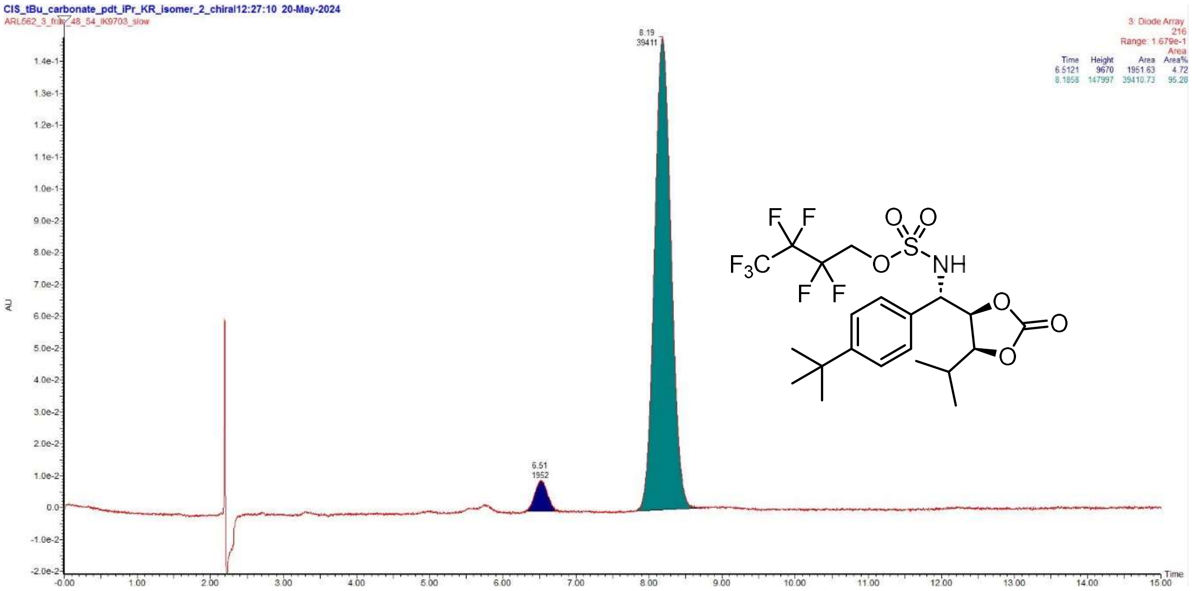

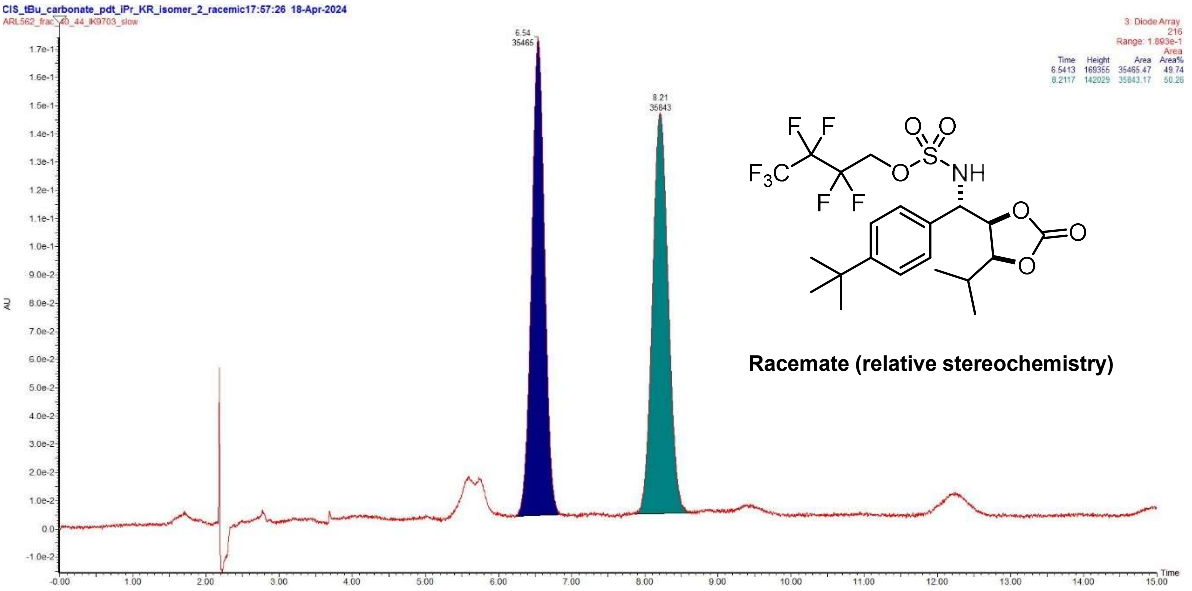

2,2,3,3,4,4,4-heptafluorobutyl ((*S*)-((*S*)-5-oxotetrahydrofuran-2-yl)(phenyl)methyl)sulfamate (**9**)

Chiral SFC Analysis CHIRALPAK IC (CO_2:MeOH, 95:5, 1.25 mL min^{-1}, 40 ˚C, 204 nm) indicated 71% *ee*, t_R =5.9 (major), 7.8 (minor) minutes.

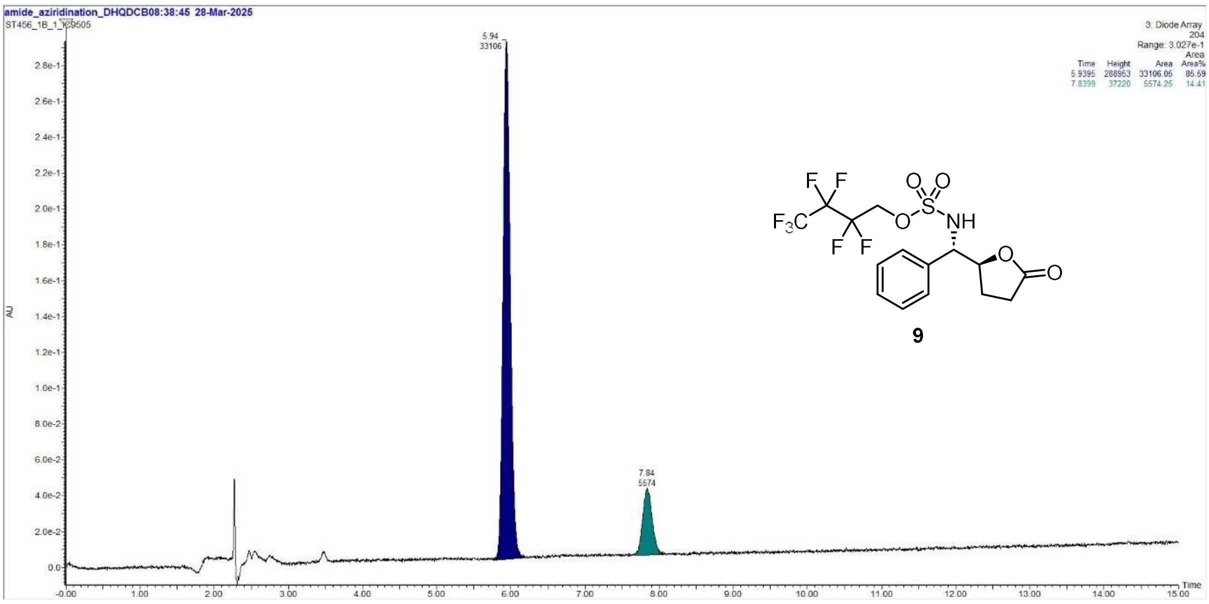

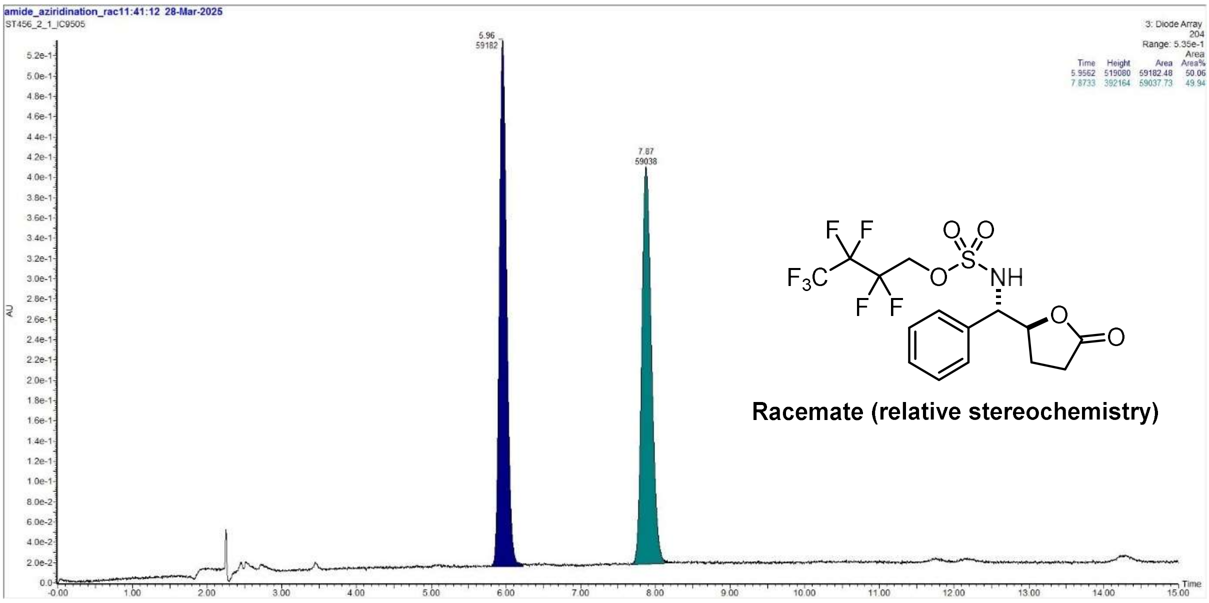

2,2,3,3,4,4,4-heptafluorobutyl ((2-oxo-4-phenyl-1,3-dioxolan-4-yl)methyl)sulfamate (**16a**)

Chiral SFC Analysis CHIRALPAK IJ (CO_2:MeOH, 97:3, 2.50 mL min^{-1}, 40 ˚C, 203 nm) indicated 5% *ee*, t_R = 2.7 (major), 2.9 (minor) minutes.

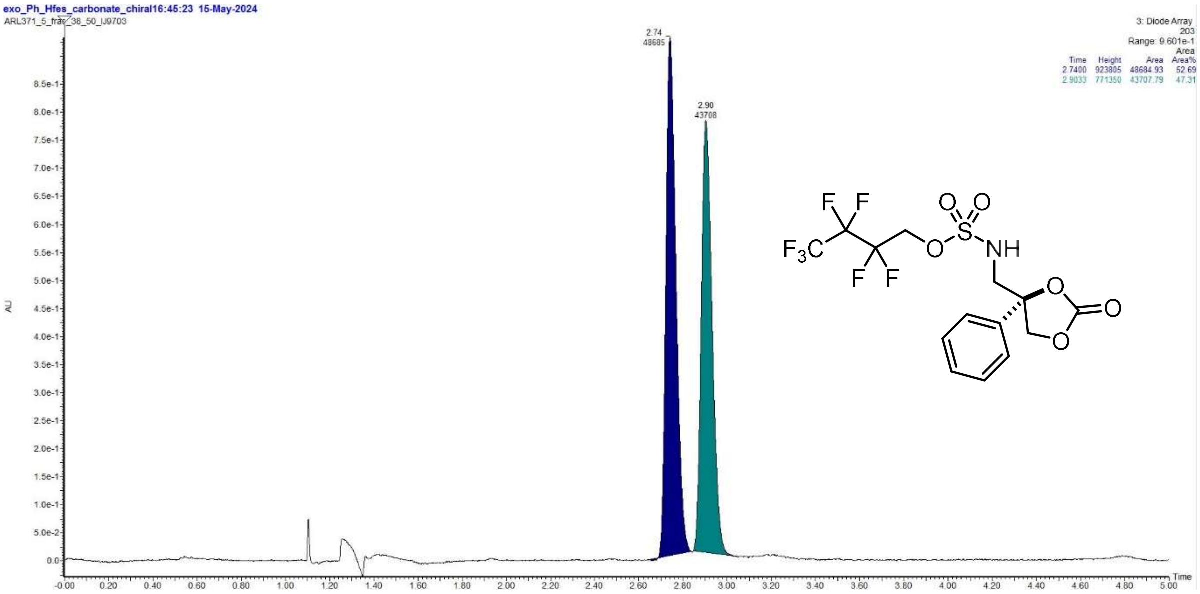

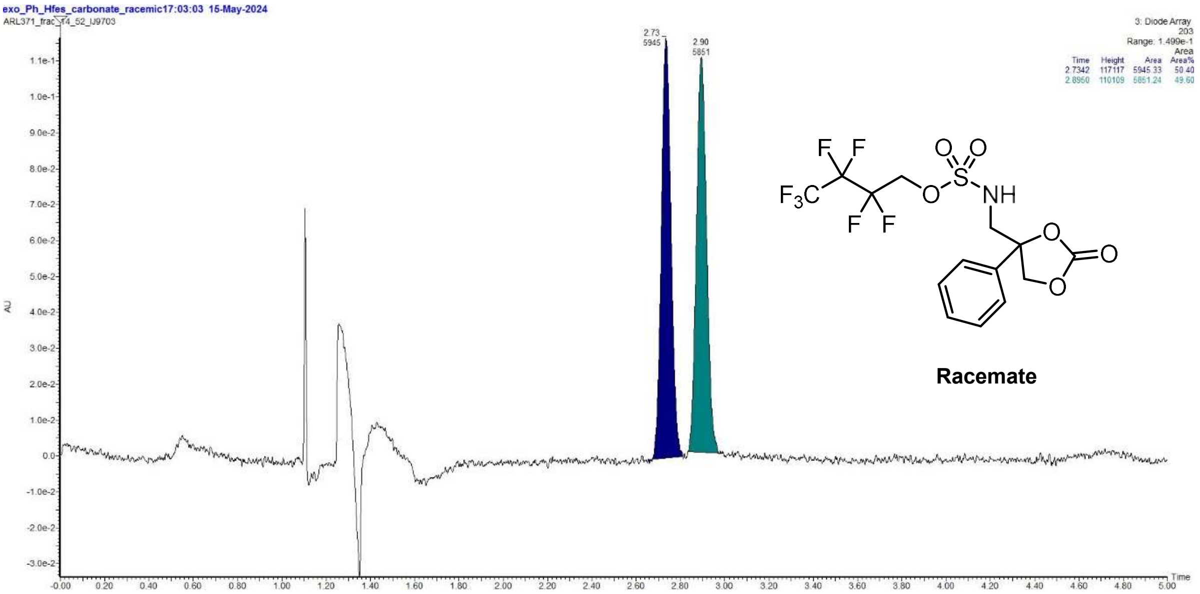

2,2,3,3,4,4,4-heptafluorobutyl (2-(2-oxo-1,3-dioxolan-4-yl)propan-2-yl)sulfamate (**16b**)

Chiral GC Analysis CP-Chirasil-Dex CB column (165 ˚C Isothermal, 1.44 mL min^{-1}) indicated 27% *ee*, t_R = 45.5 (major), 46.8 (minor) minutes.

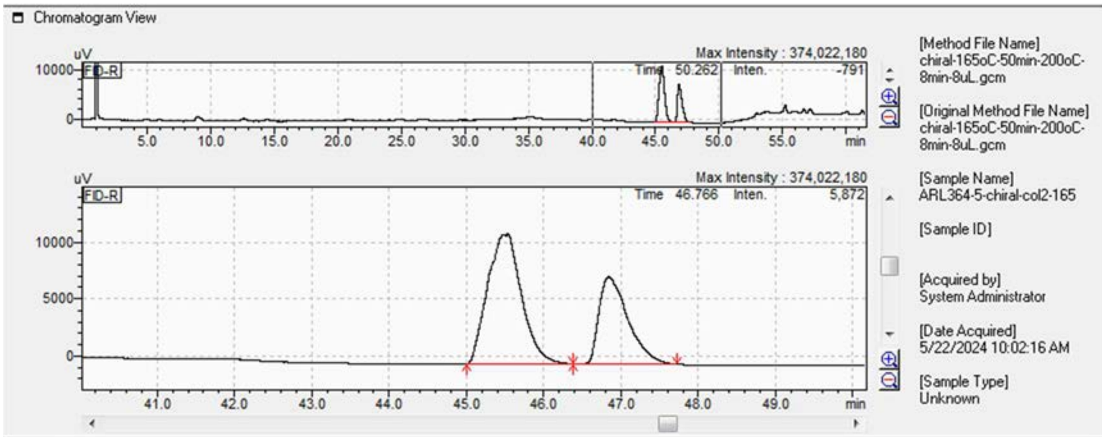

Results View - Peak Table

Peak#	Ret. Time	Area	Conc.	Area%
1	45.533	349333	63.660	63.660
2	46.840	199412	36.340	36.340
Total		548745	100.000	100.000

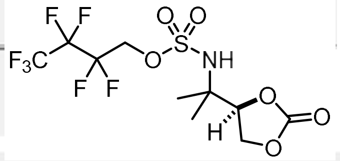

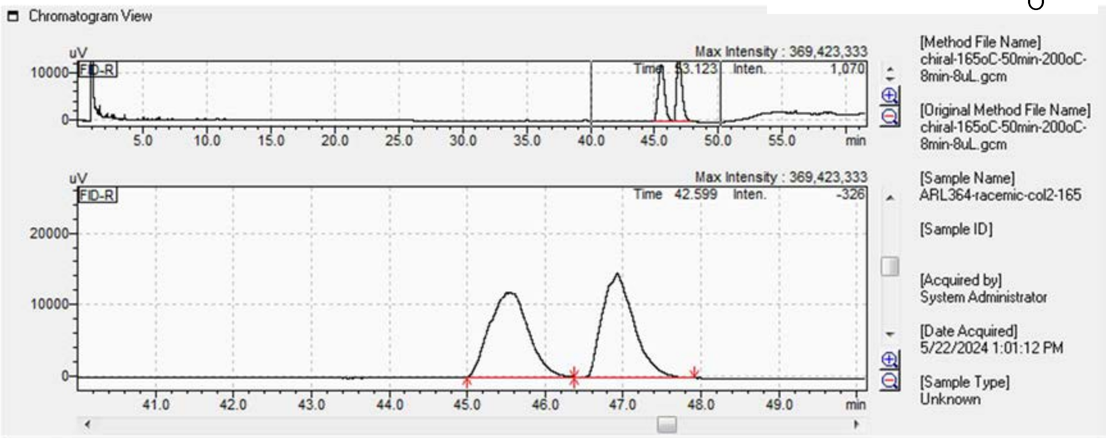

Results View - Peak Table

Peak#	Ret. Time	Area	Conc.	Area%
1	45.549	418441	50.182	50.182
2	46.926	415413	49.818	49.818
Total		833855	100.000	100.000

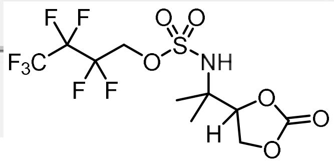

Racemate

2,2,3,3,4,4,4-heptafluorobutyl (4-bromobenzyl)(1-(2-oxo-1,3-dioxolan-4-yl)butyl)sulfamate (derived from **16c**)

Chiral SFC Analysis CHIRALPAK IC (CO_2:MeOH, 97:3, 2.50 mL min^{-1}, 40 ˚C, 223 nm) indicated 28% *ee*, t_R = 5.1 (minor), 5.5 (major) minutes.

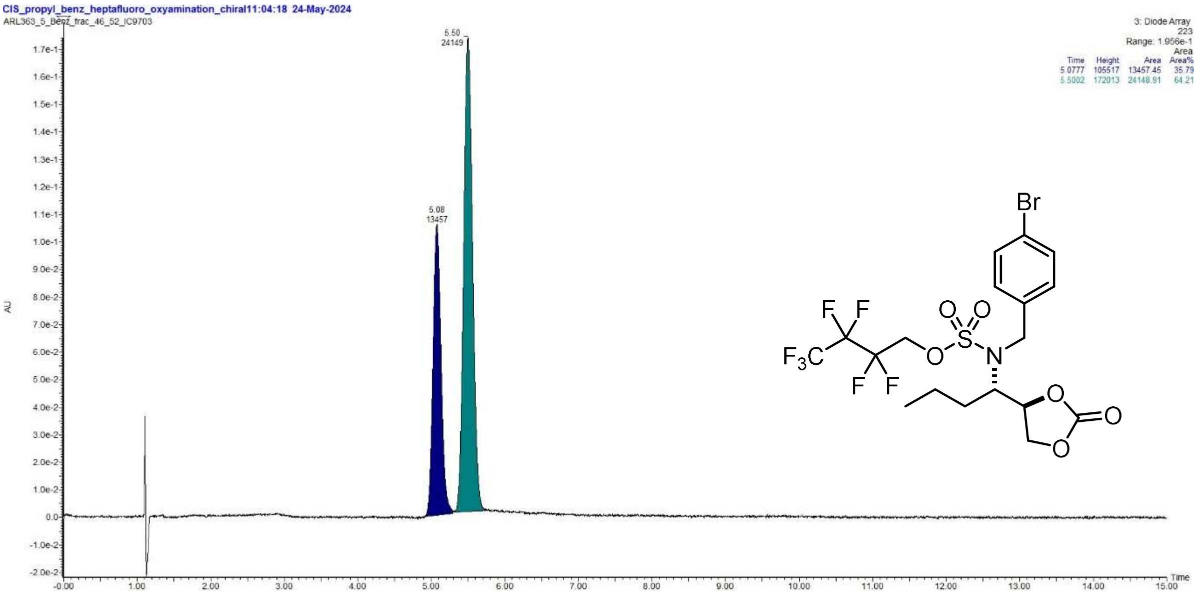

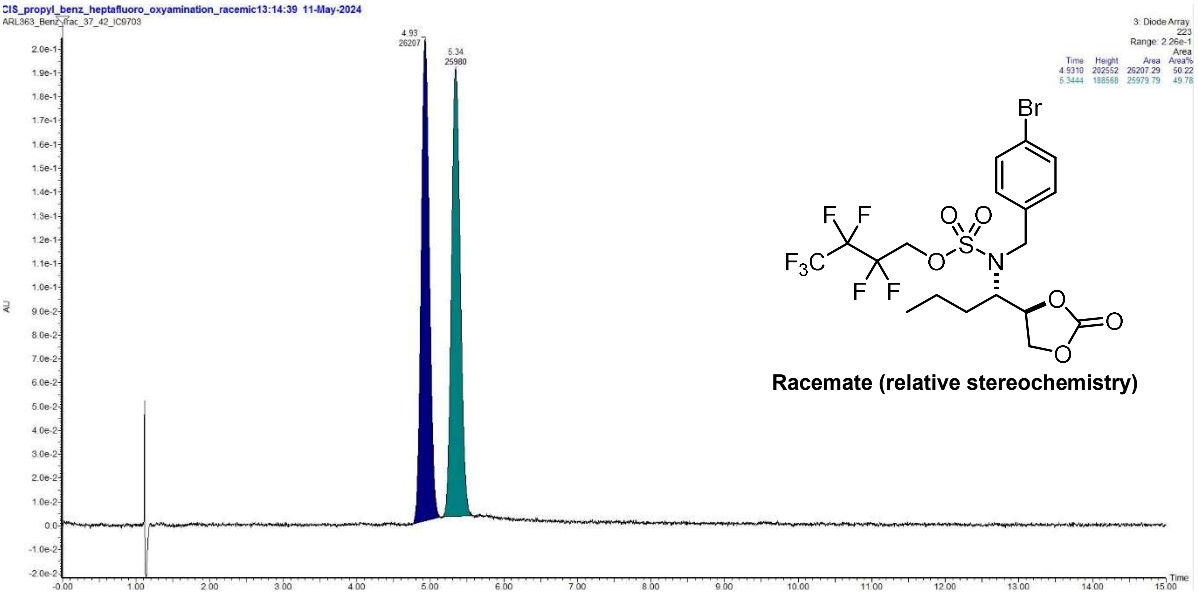

2,2,3,3,4,4,4-heptafluorobutyl ((2-oxo-1,3-dioxolan-4-yl)(phenyl)methyl)sulfamate (**S3**)

Chiral SFC Analysis CHIRALPAK IC (CO_2:MeOH, 96:4, 2.50 mL min^{-1}, 40 ˚C, 204 nm) indicated 19% *ee*, t_R = 3.7 (major), 10.4 (minor) minutes.

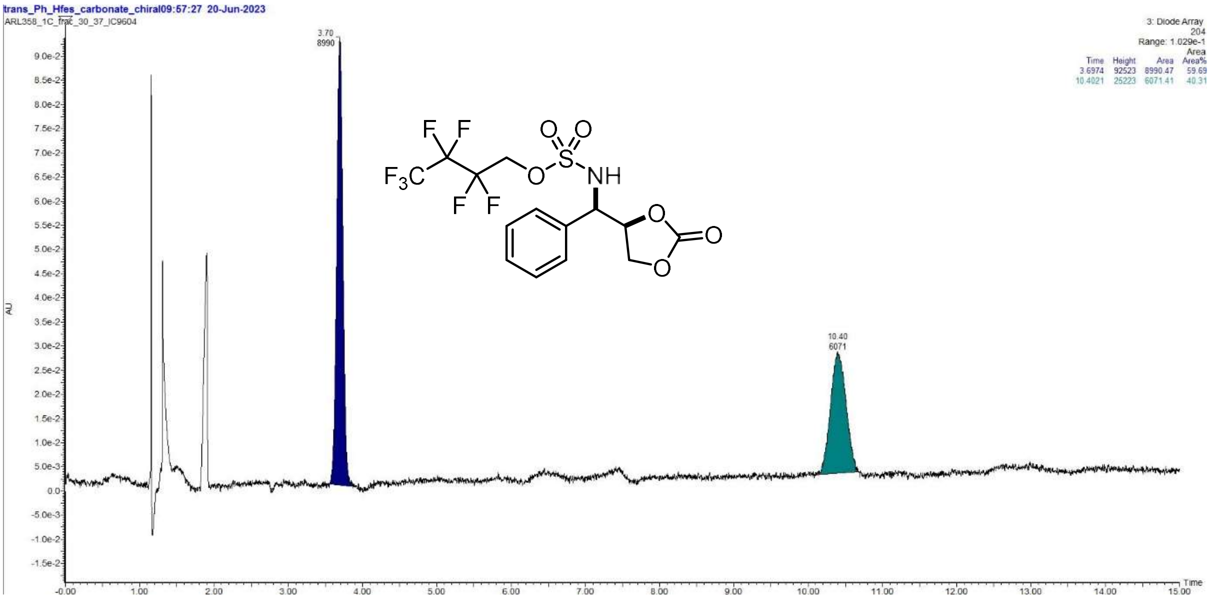

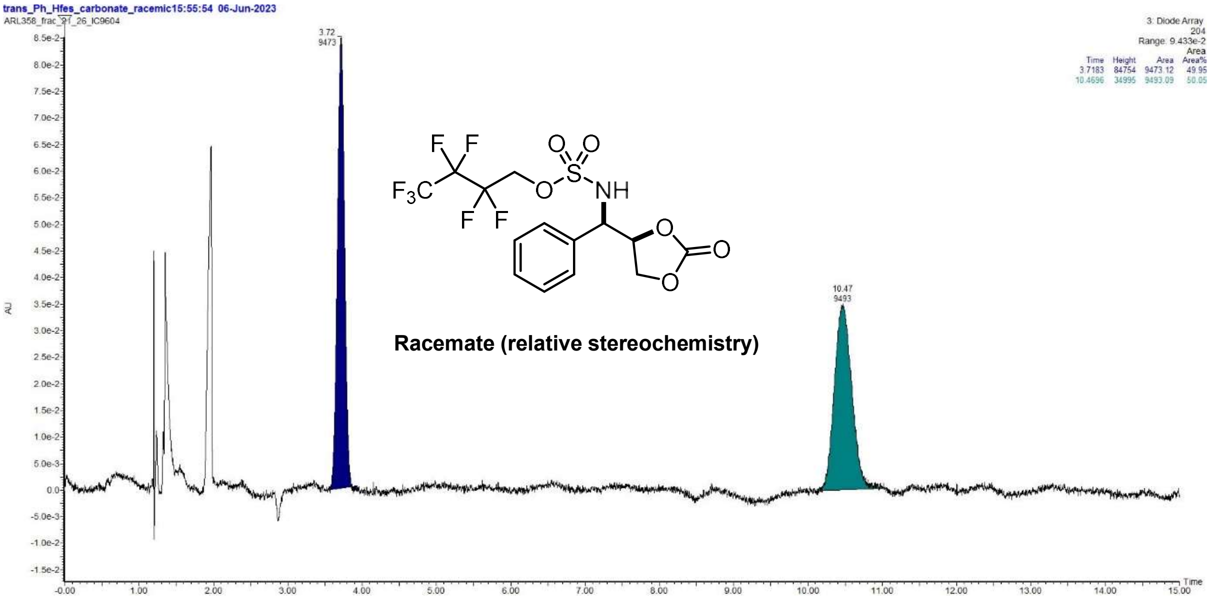

2,2,3,3,4,4,4-heptafluorobutyl (2*S*,3*S*)-2-(((methyl(phenyl)carbamoyl)oxy)methyl)-3-phenylaziridine-1-sulfonate (**3a**)

Chiral SFC Analysis CHIRALPAK IJ (CO_2:MeOH, 98:2, 1.25 mL min^{-1}, 40 ˚C, 230 nm) indicated 89% *ee*, t_R = 7.3 (major), 8.1 (minor) minutes.

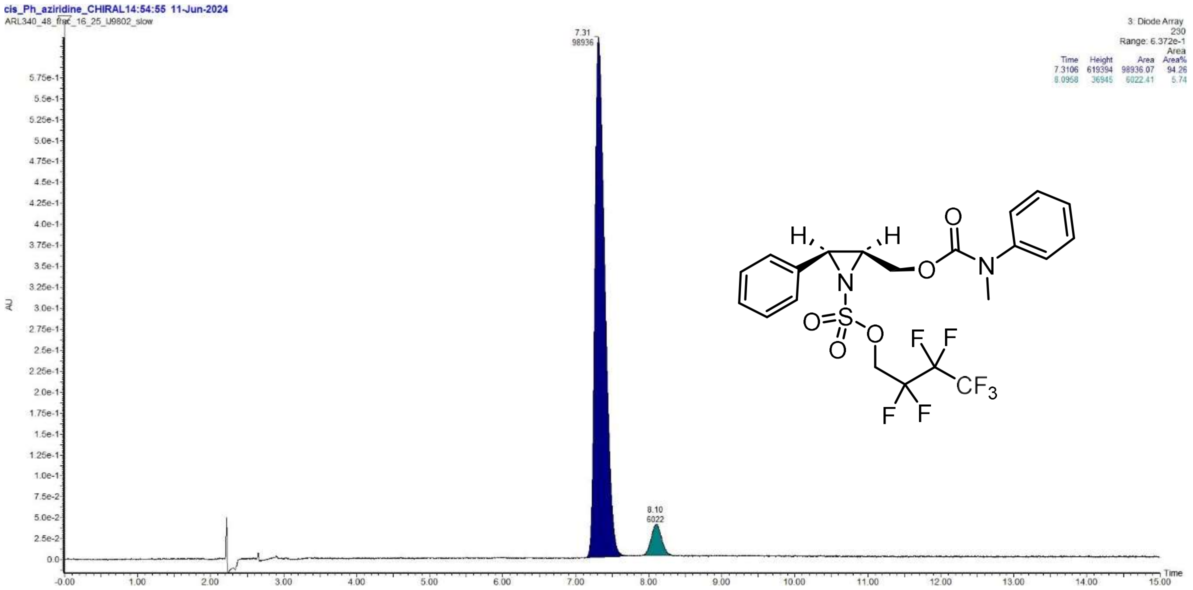

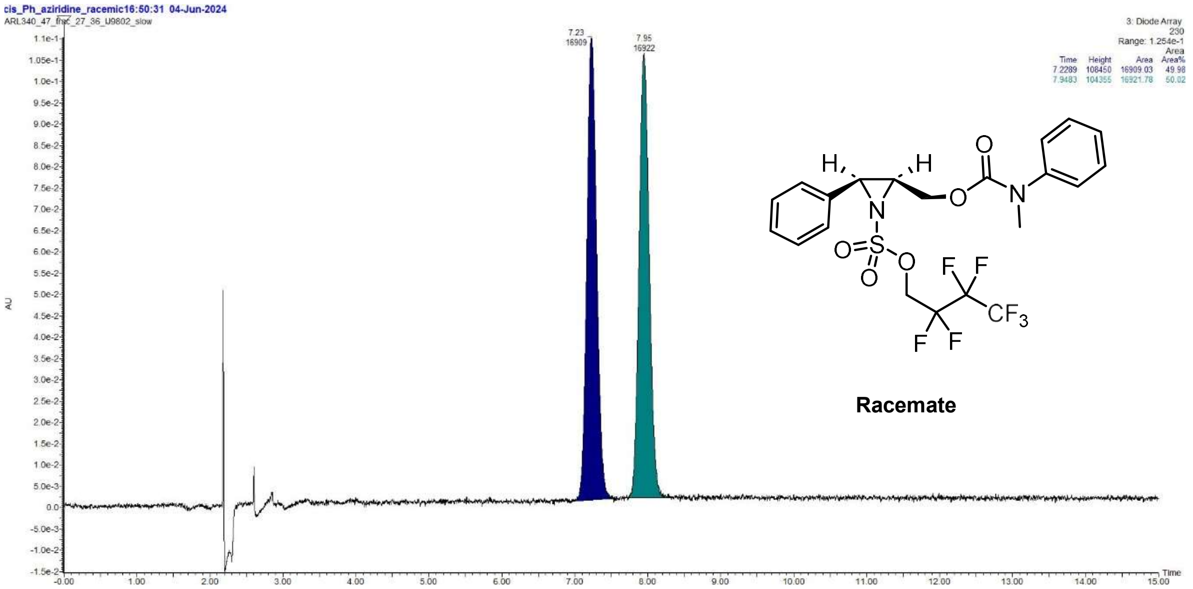

2,2,3,3,4,4,4-heptafluorobutyl 2-(4-chlorophenyl)-3-(((methyl(phenyl)carbamoyl)oxy)methyl)aziridine-1-sulfonate

Chiral SFC Analysis: CHIRALPAK IJ (CO_2:MeOH, 98:2, 2.50 mL min^{-1}, 40 ˚C, 218 nm) indicated 90% *ee*, t_R = 5.5 (minor), 7.4 (major) minutes.

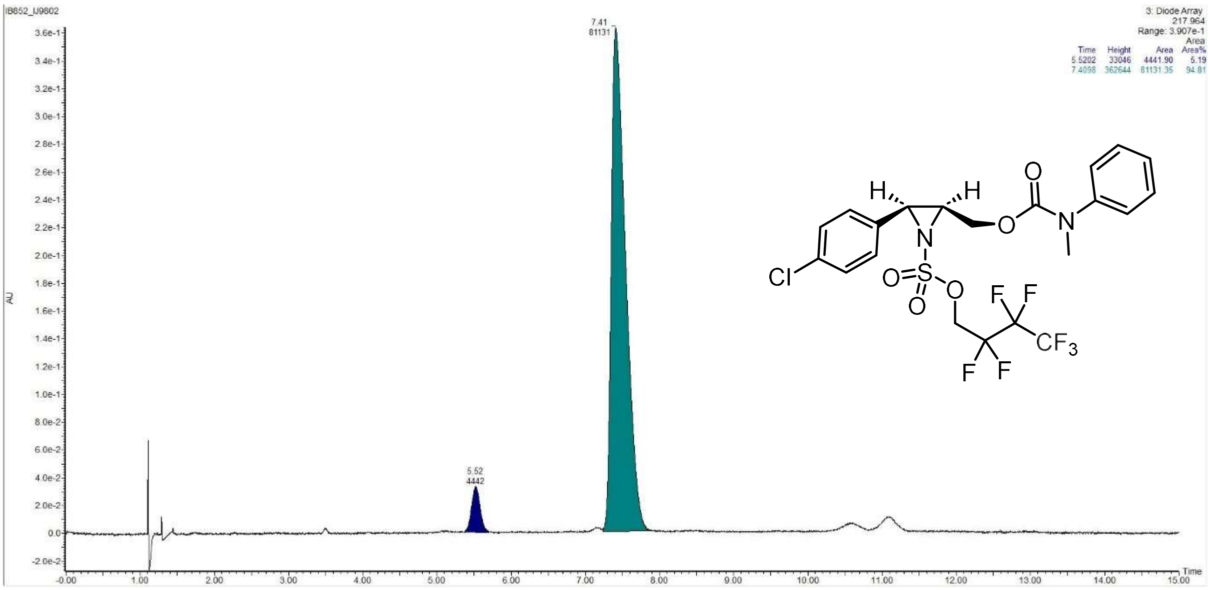

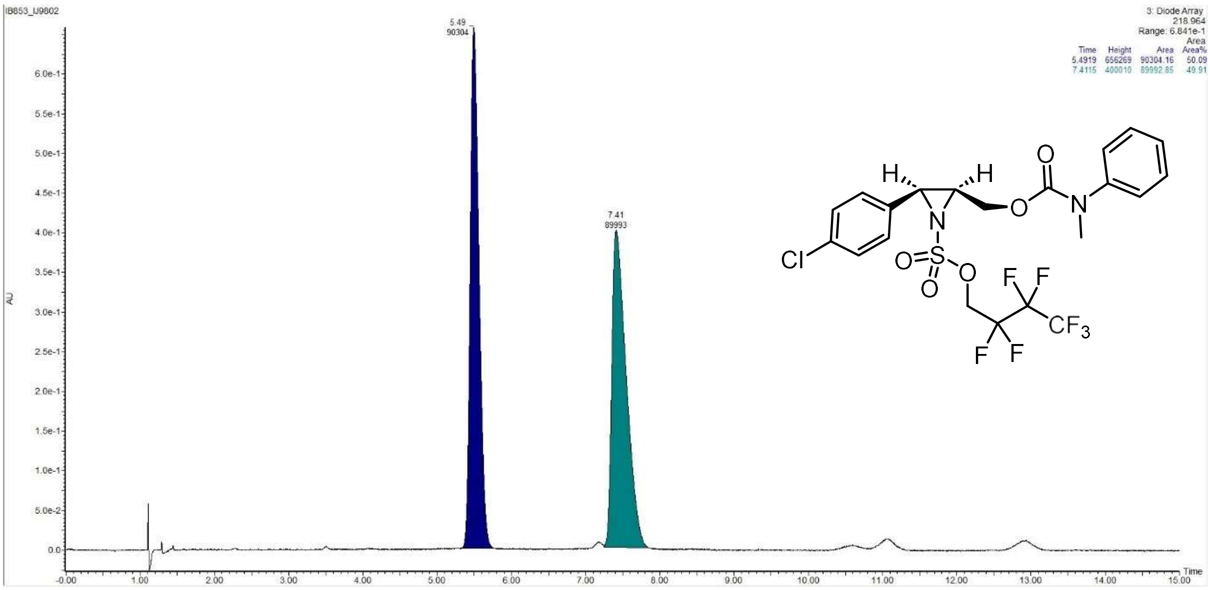

2,2,3,3,4,4,4-heptafluorobutyl 2-(((methyl(phenyl)carbamoyl)oxy)methyl)-3-(4-(trifluoromethyl)phenyl)aziridine-1-sulfonate

Chiral SFC Analysis: CHIRALPAK IJ (CO_2:MeOH, 99:1, 2.50 mL min^{-1}, 40 ˚C, 217 nm), t_R = 3.5 (minor), 5.6 (major) minutes.

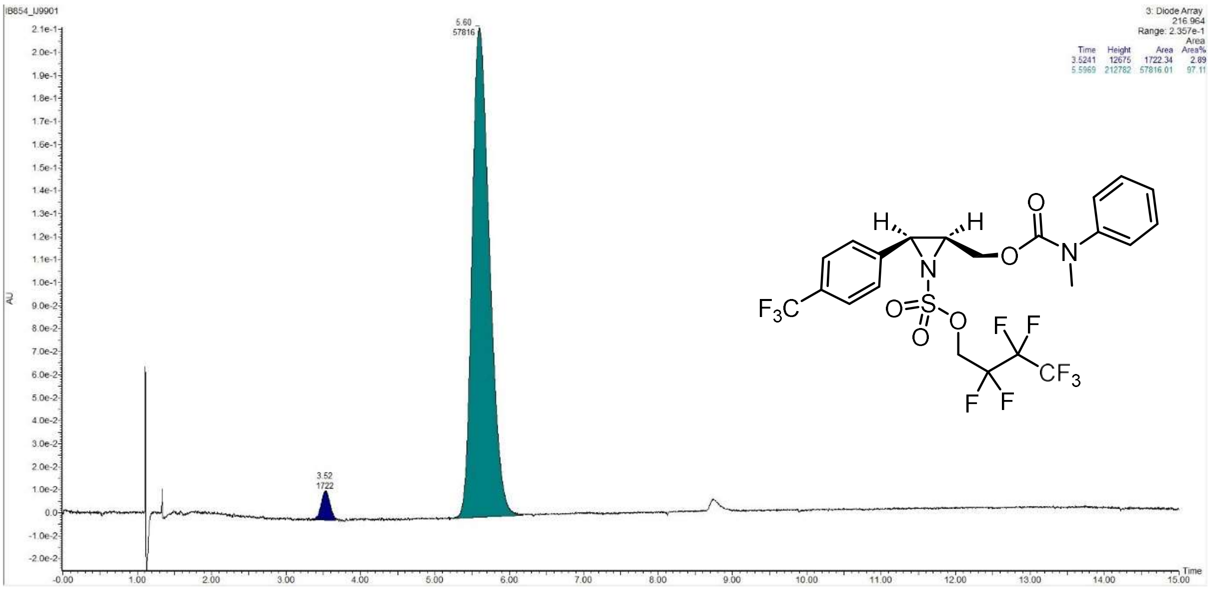

A racemic sample could not be isolated due to decomposition.

2,2,3,3,4,4,4-heptafluorobutyl ((1*R*,2*S*)-1-azido-3-((methyl(phenyl)carbamoyl)oxy)-1-phenylpropan-2-yl)sulfamate (**5**)

Chiral SFC Analysis CHIRALPAK IK (CO_2:MeOH, 96:4, 2.50 mL min^{-1}, 40 ˚C, 230 nm) indicated 89% *ee*, t_R = 4.0 (minor), 4.7 (major) minutes.

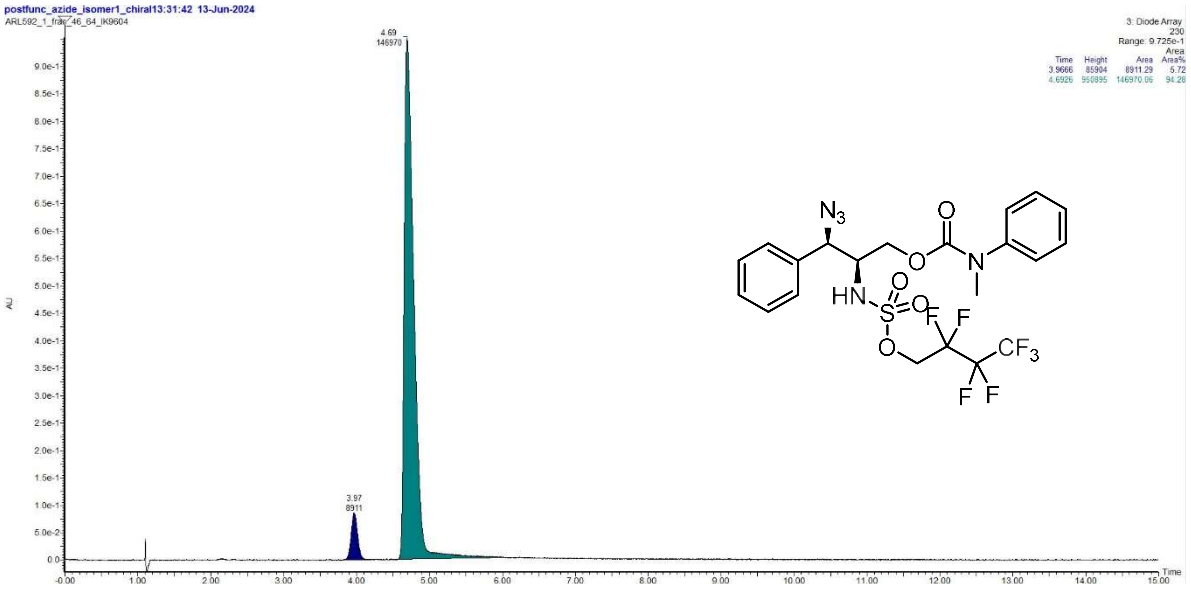

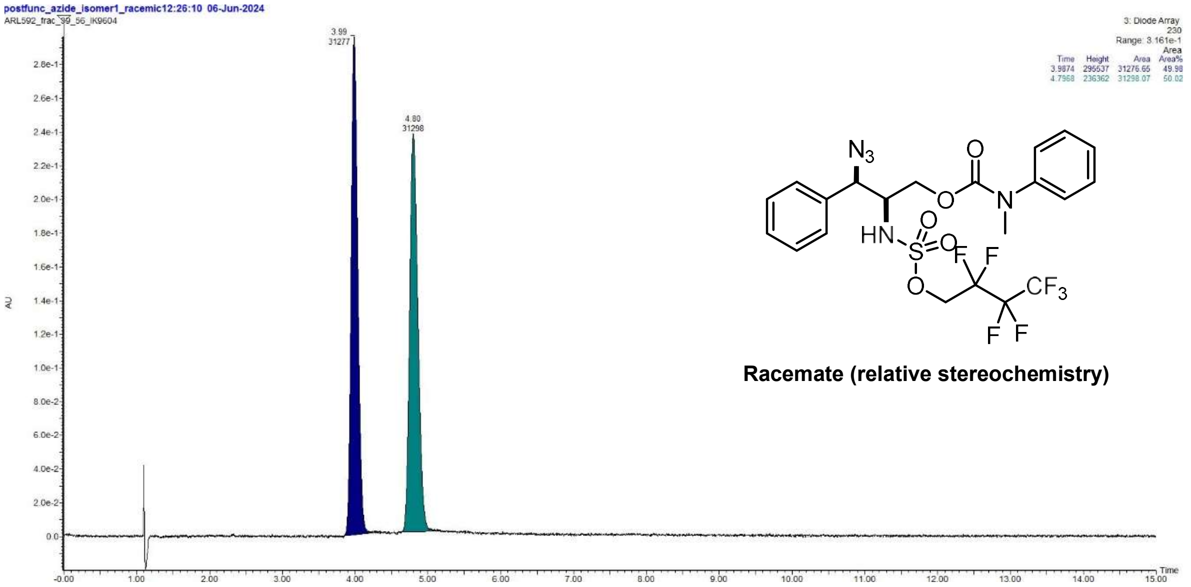

2,2,3,3,4,4,4-heptafluorobutyl ((1*S*,2*R*)-2-azido-3-((methyl(phenyl)carbamoyl)oxy)-1-phenylpropyl)sulfamate (**6**)

Chiral SFC Analysis CHIRALPAK IJ (CO_2:MeOH, 97:3, 2.50 mL min^{-1}, 40 ˚C, 225 nm) indicated 89% *ee*, t_R = 5.5 (major), 6.1 (minor) minutes.

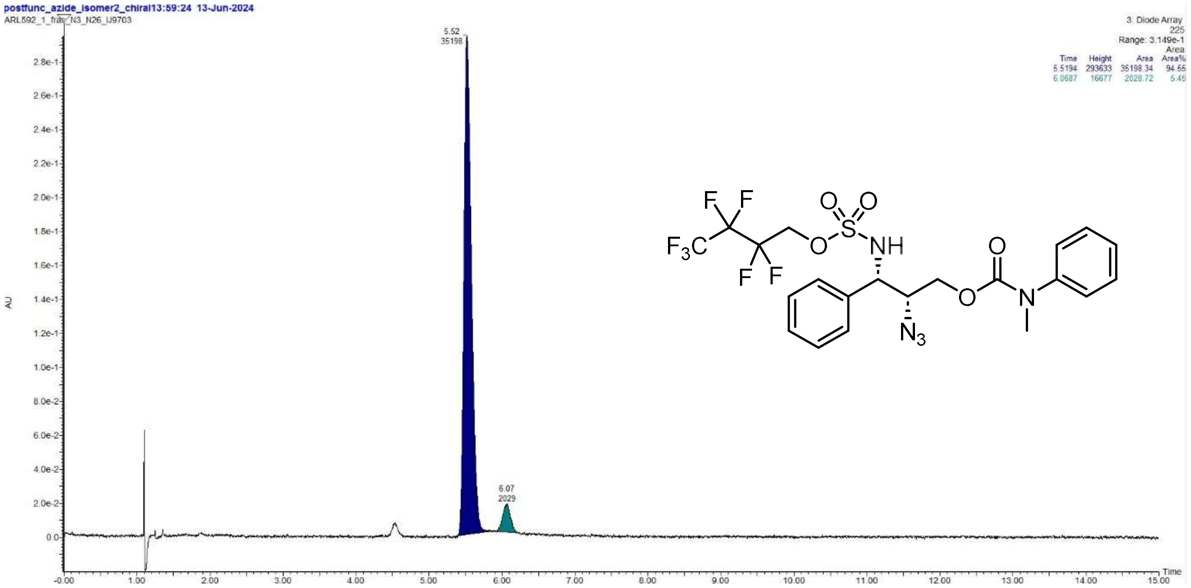

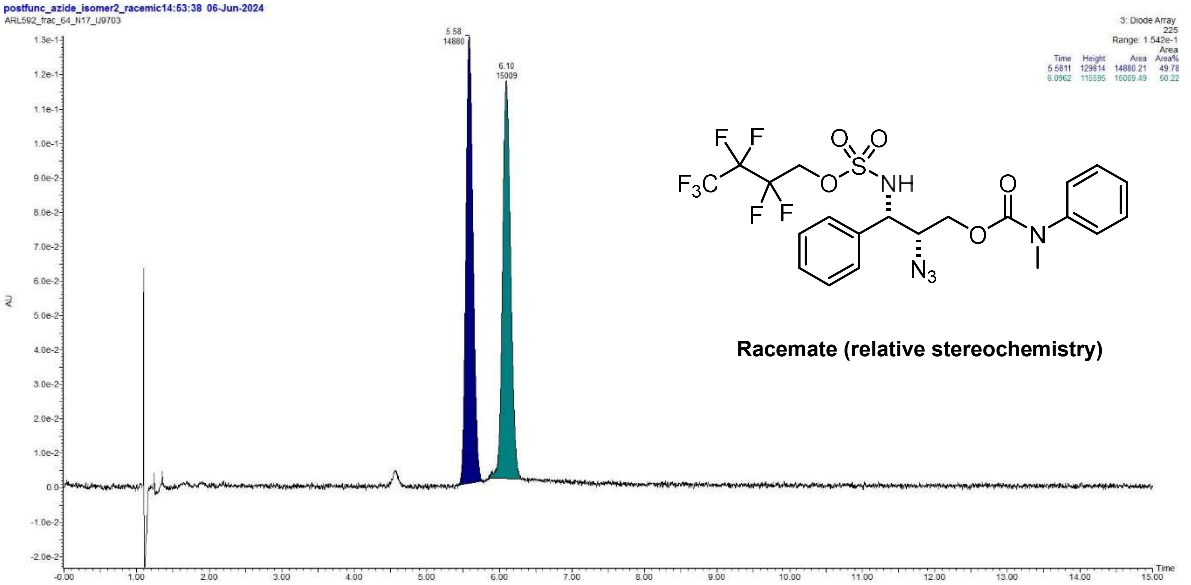

2,2,3,3,4,4,4-heptafluorobutyl (*S*)-(1-((methyl(phenyl)carbamoyl)oxy)-3-phenylpropan-2-yl)sulfamate (**7**)

Chiral SFC Analysis CHIRALPAK IC (CO_2:MeOH, 97:3, 2.50 mL min^{-1}, 40 ˚C, 228 nm) indicated 89% *ee*, t_R = 4.7 (major), 8.2 (minor) minutes.

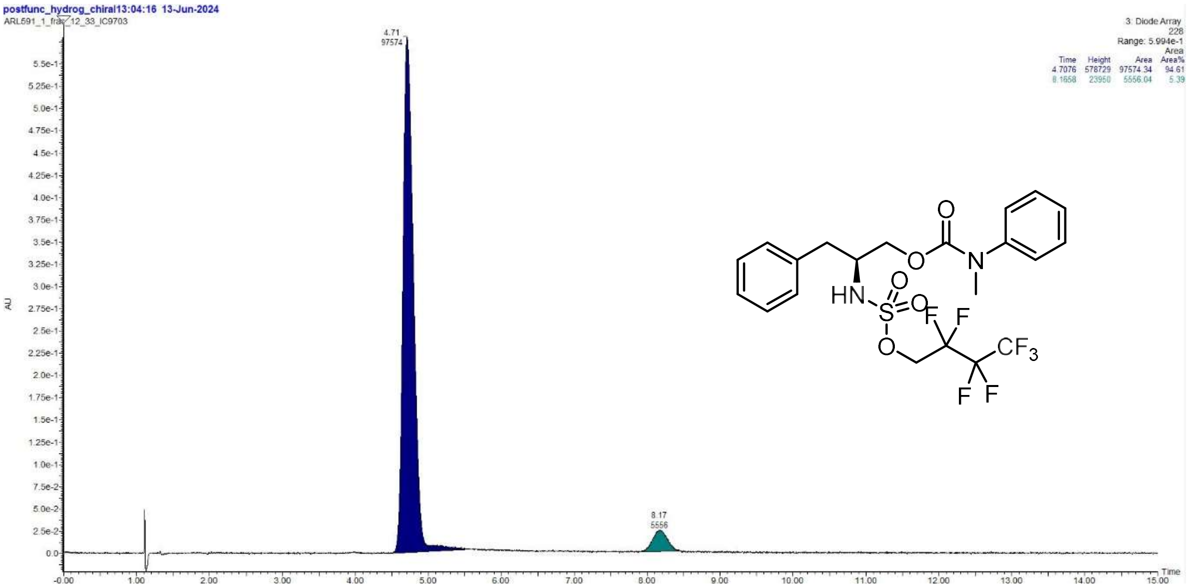

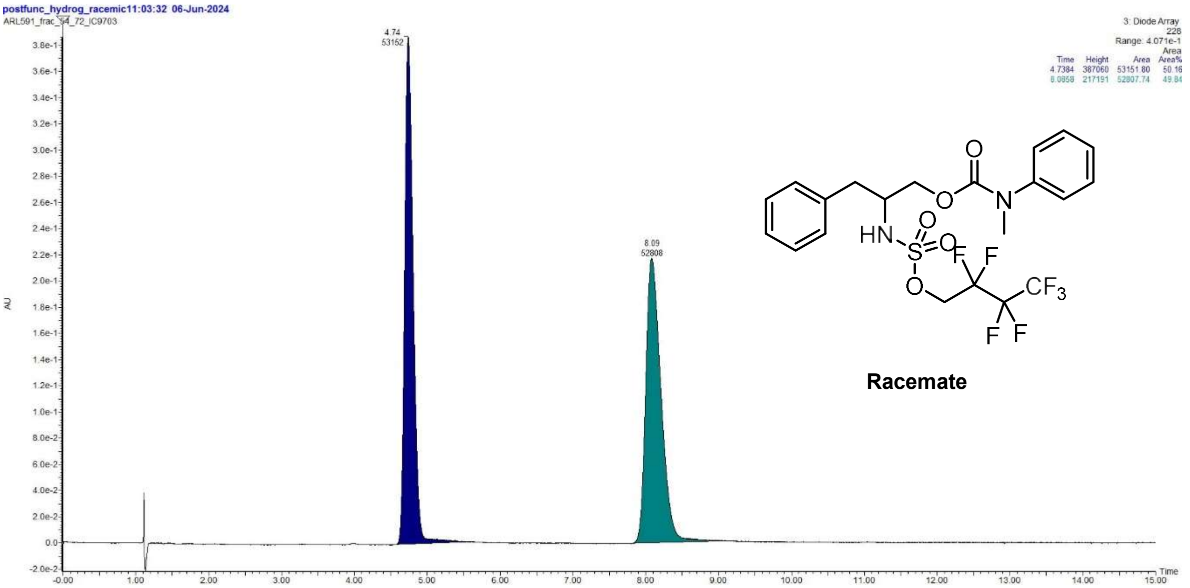

2,2,3,3,4,4,4-heptafluorobutyl ((1*S*,2*R*)-3-chloro-2-((methyl(phenyl)carbamoyl)oxy)-1-phenylpropyl)sulfamate (**8**)

Chiral SFC Analysis CHIRALPAK IJ (CO_2:MeOH, 97:3, 2.50 mL min^{-1}, 40 ˚C, 227 nm) indicated 89% *ee*, t_R = 4.2 (major), 9.4 (minor) minutes.

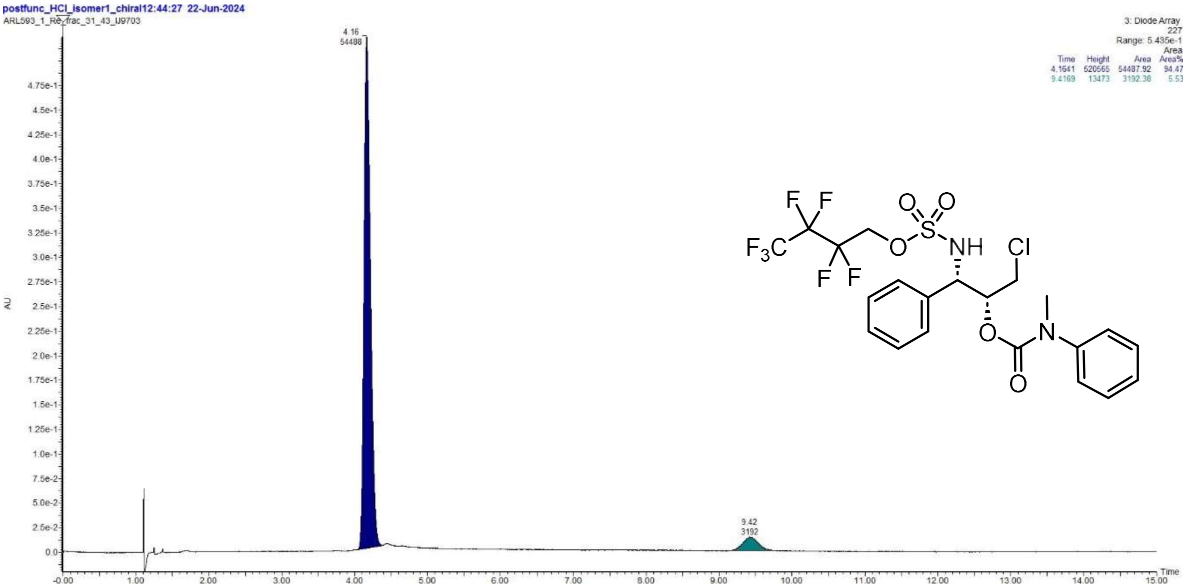

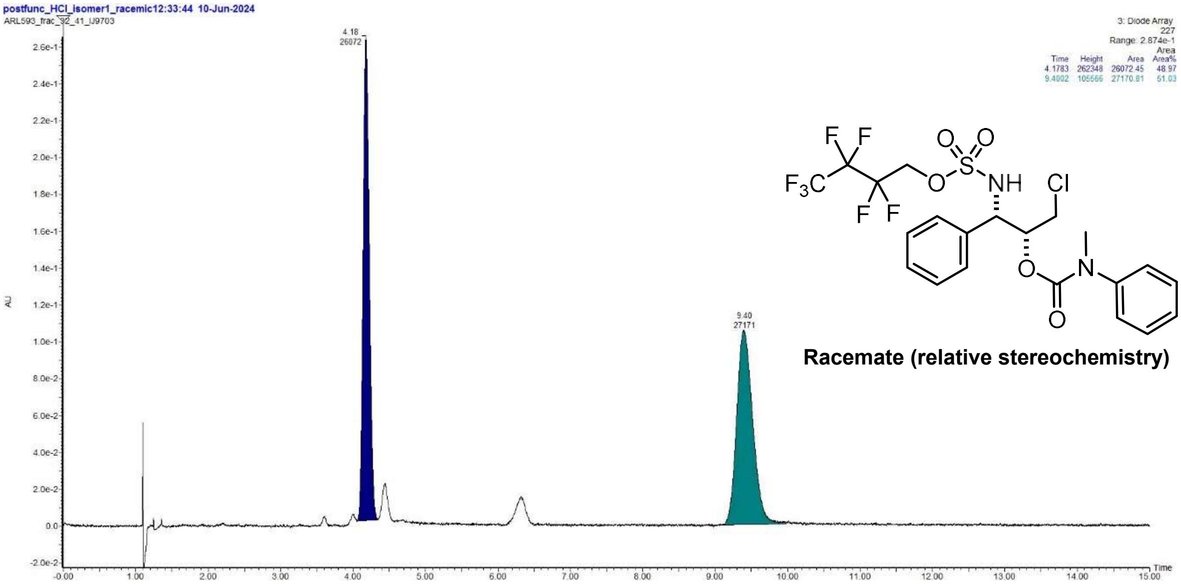

(4*S*,5*R*)-5-(hydroxymethyl)-4-phenyloxazolidin-2-one

Chiral SFC Analysis CHIRALPAK IK (CO_2:MeOH, 90:10, 2.50 mL min^{-1}, 40 ˚C, 204 nm) indicated 90% *ee*, t_R = 9.5 (major), 11.6 (minor) minutes.

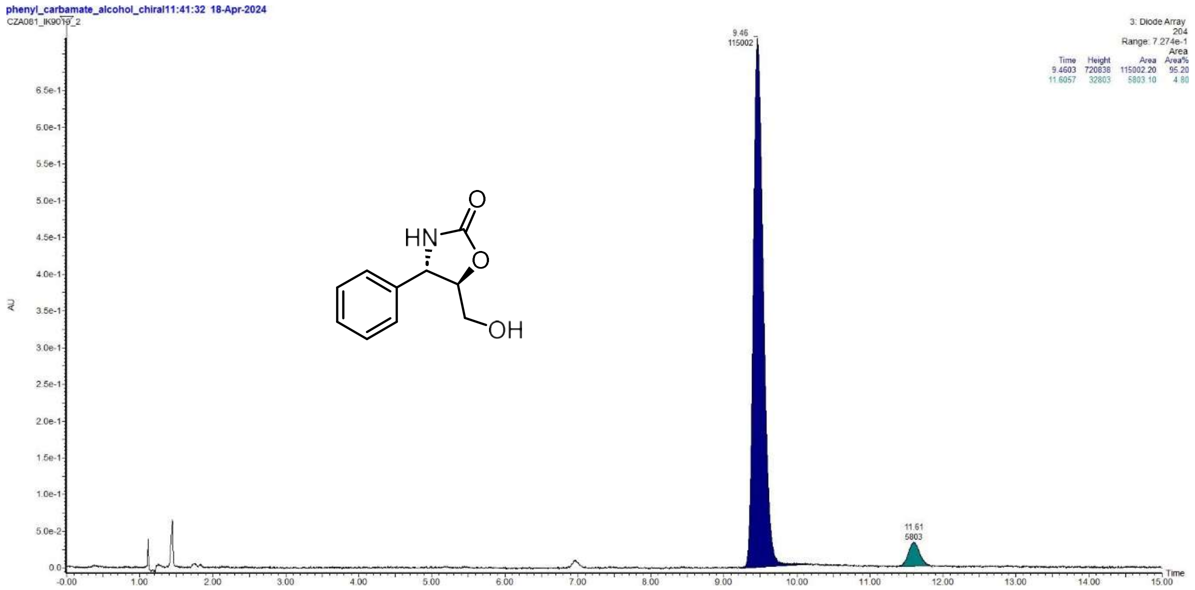

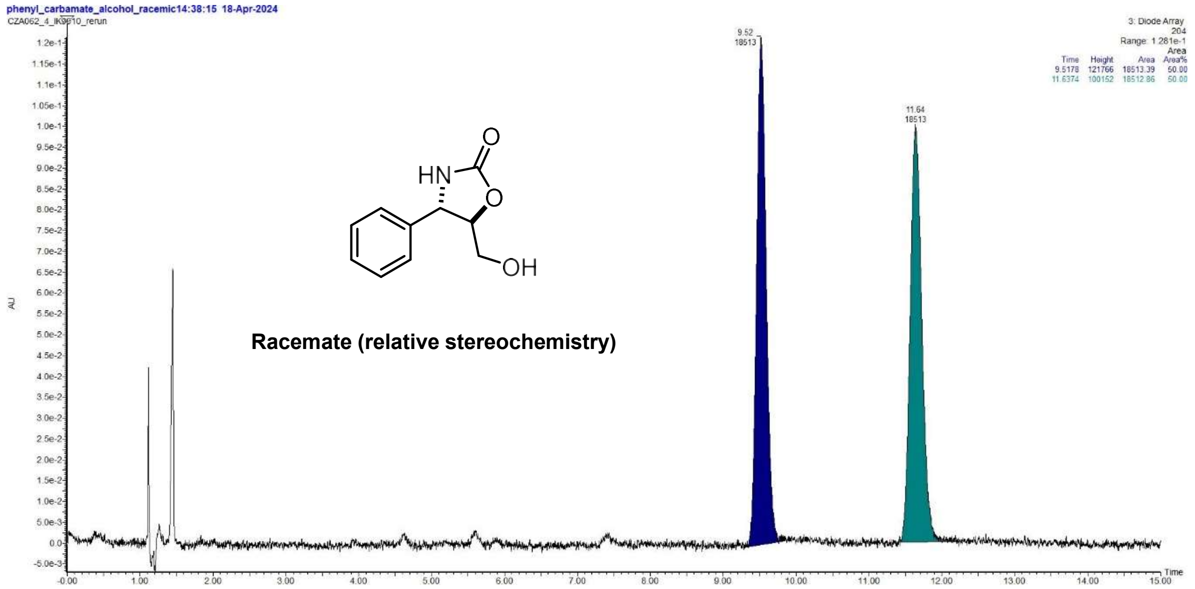

2,2,3,3,3-pentafluoropropyl (*S*)-(2-((ethyl(phenyl)carbamoyl)oxy)-1-phenylethyl)sulfamate (**11a**)

Chiral SFC Analysis CHIRALPAK IK (CO_2:MeOH, 95:5, 2.50 mL min^{-1}, 40 ˚C, 202 nm) indicated 96% *ee*, t_R = 3.3 (major), 4.0 (minor) minutes.

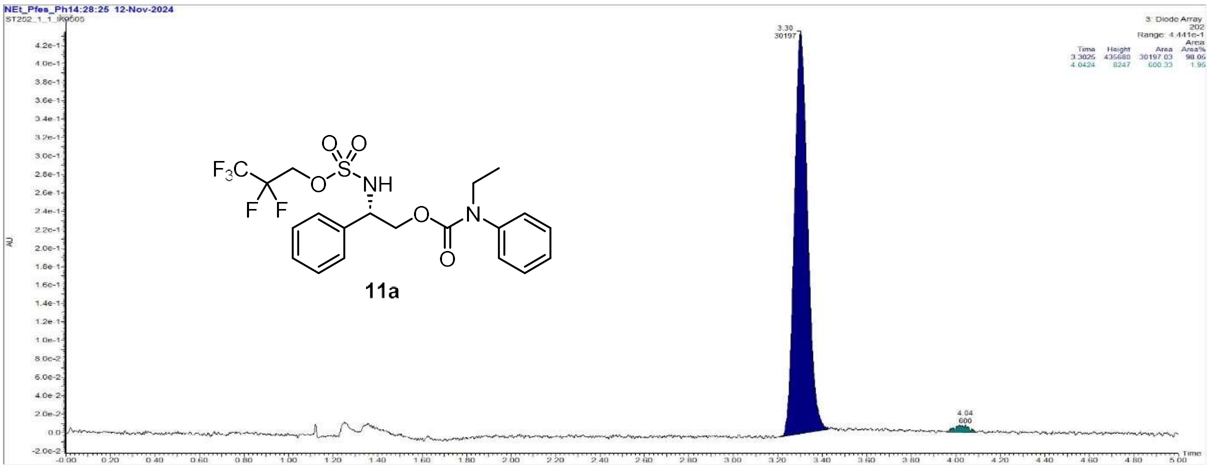

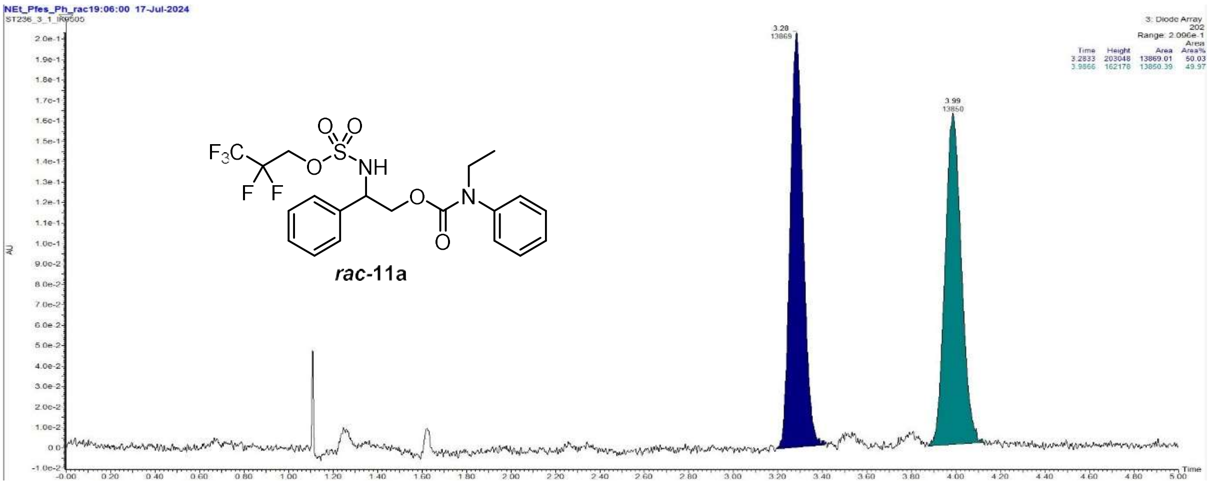

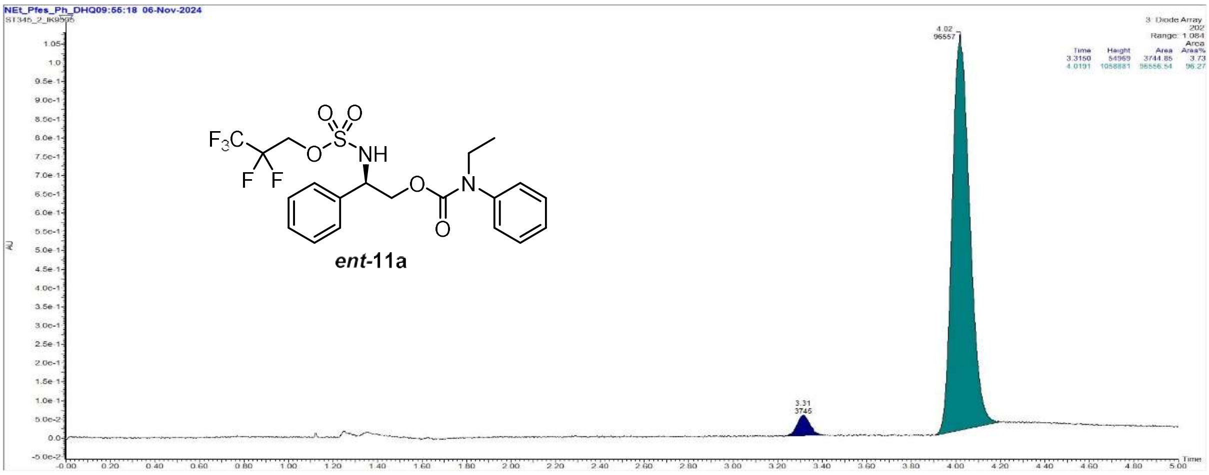

2,2,3,3,3-pentafluoropropyl (*S*)-(2-((ethyl(phenyl)carbamoyl)oxy)-1-(4-methoxyphenyl)ethyl)sulfamate (**11b**)

Chiral SFC Analysis CHIRALPAK IJ (CO_2:MeOH, 94:6, 2.50 mL min^{-1}, 40 ˚C, 224 nm) indicated 93% *ee*, t_R = 3.5 (major), 4.4 (minor) minutes.

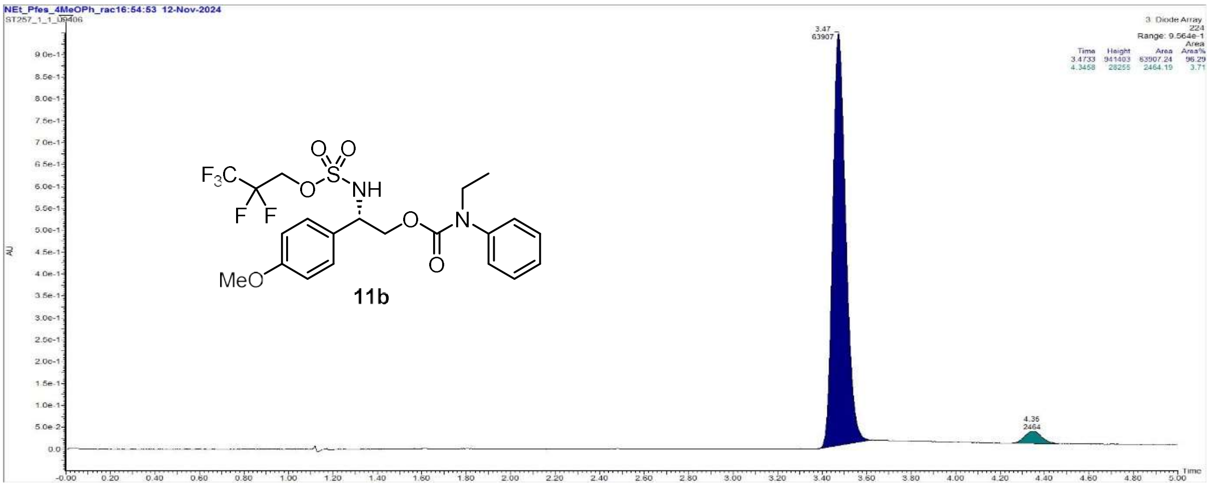

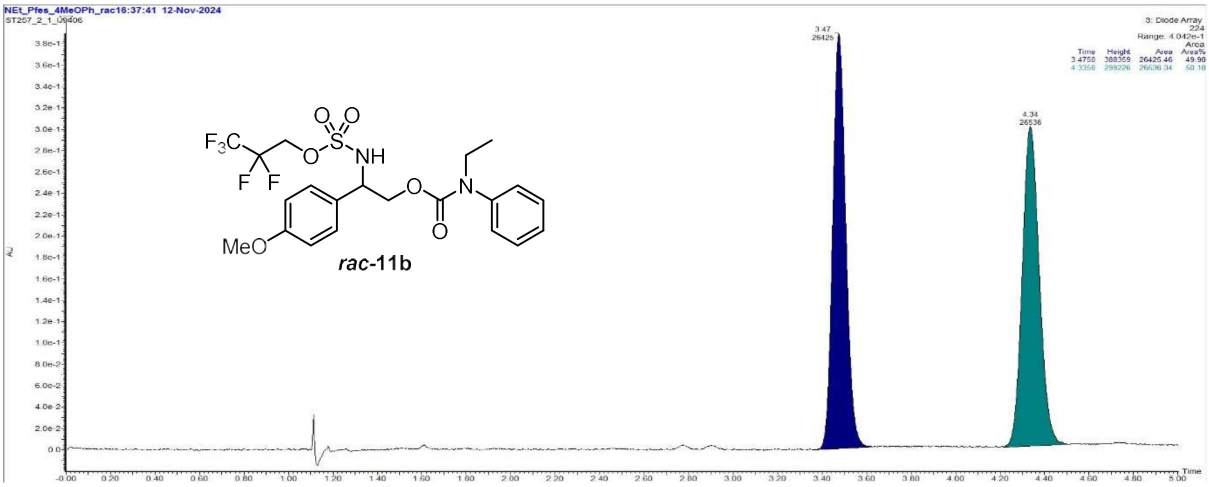

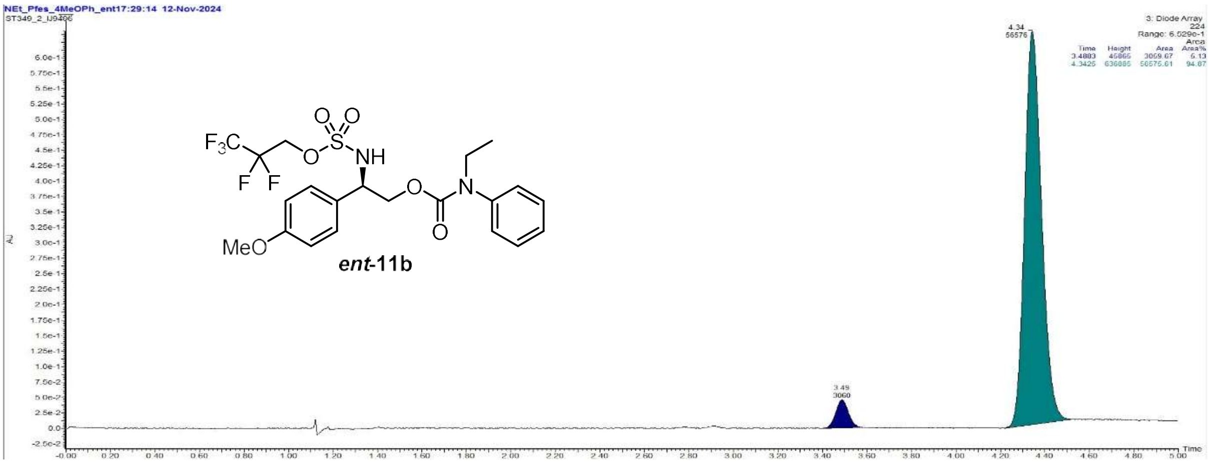

2,2,3,3,3-pentafluoropropyl (*S*)-(1-(4-(tert-butyl)phenyl)-2-((ethyl(phenyl)carbamoyl)oxy)ethyl)sulfamate (**11c**)

Chiral SFC Analysis CHIRALPAK IJ (CO_2:MeOH, 94:6, 2.50 mL min^{-1}, 40 ˚C, 202 nm) indicated 95% *ee*, t_R = 2.4 (major), 2.8 (minor) minutes.

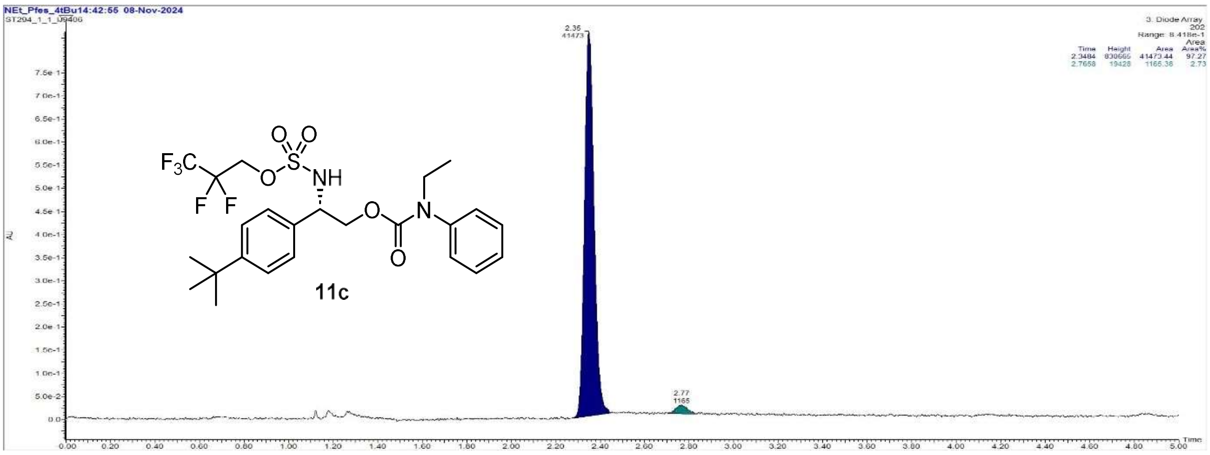

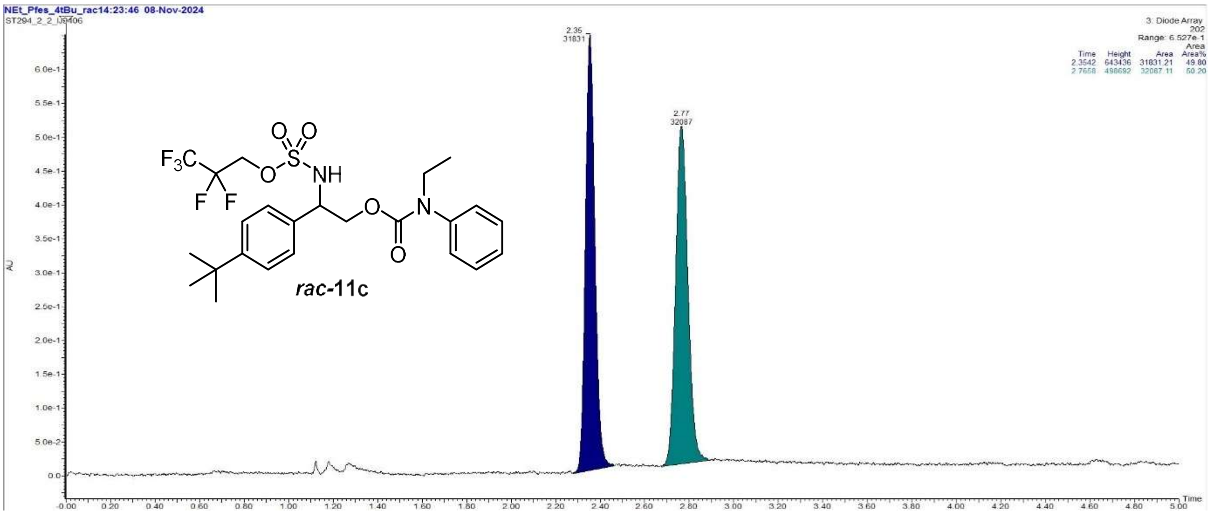

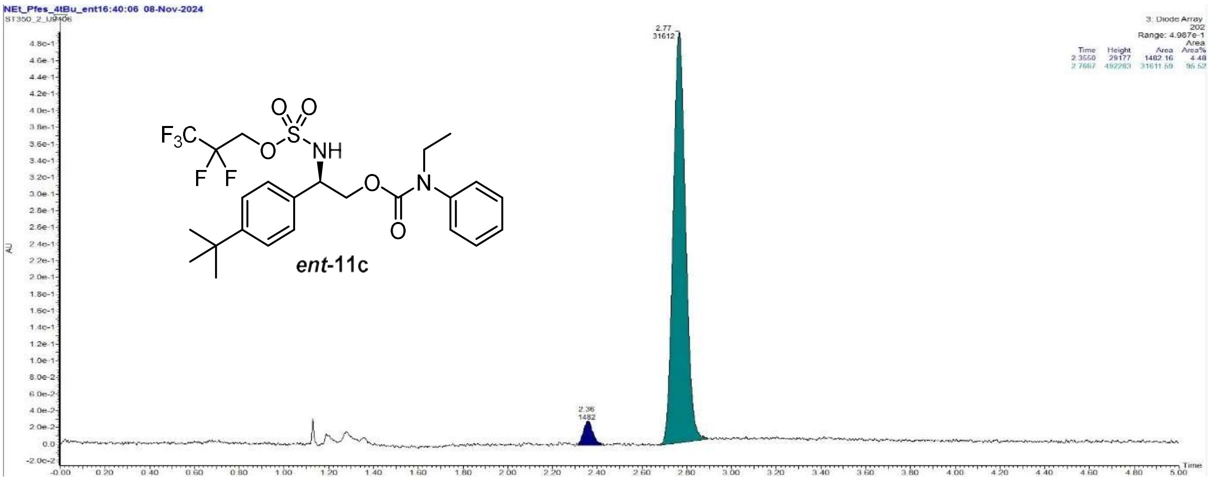

2,2,3,3,3-pentafluoropropyl (*S*)-(2-((ethyl(phenyl)carbamoyl)oxy)-1-(p-tolyl)ethyl)sulfamate (**11d**)

Chiral SFC Analysis CHIRALPAK IJ (CO_2:MeOH, 94:6, 2.50 mL min^{-1}, 40 ˚C, 202 nm) indicated 95% *ee*, t_R = 2.6 (major), 3.1 (minor) minutes.

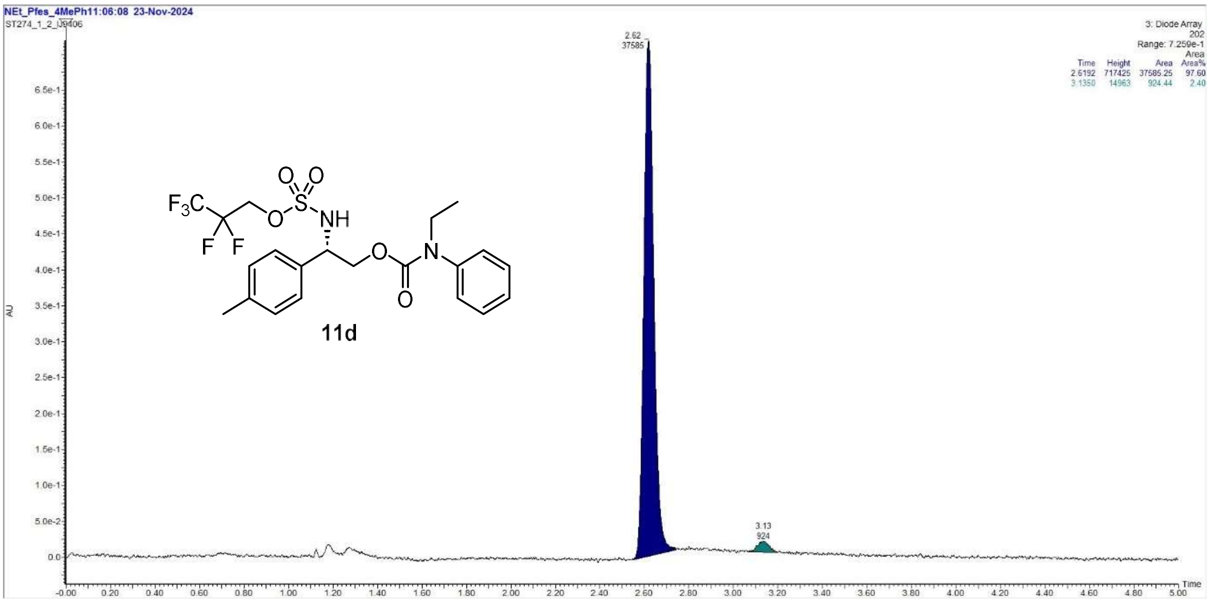

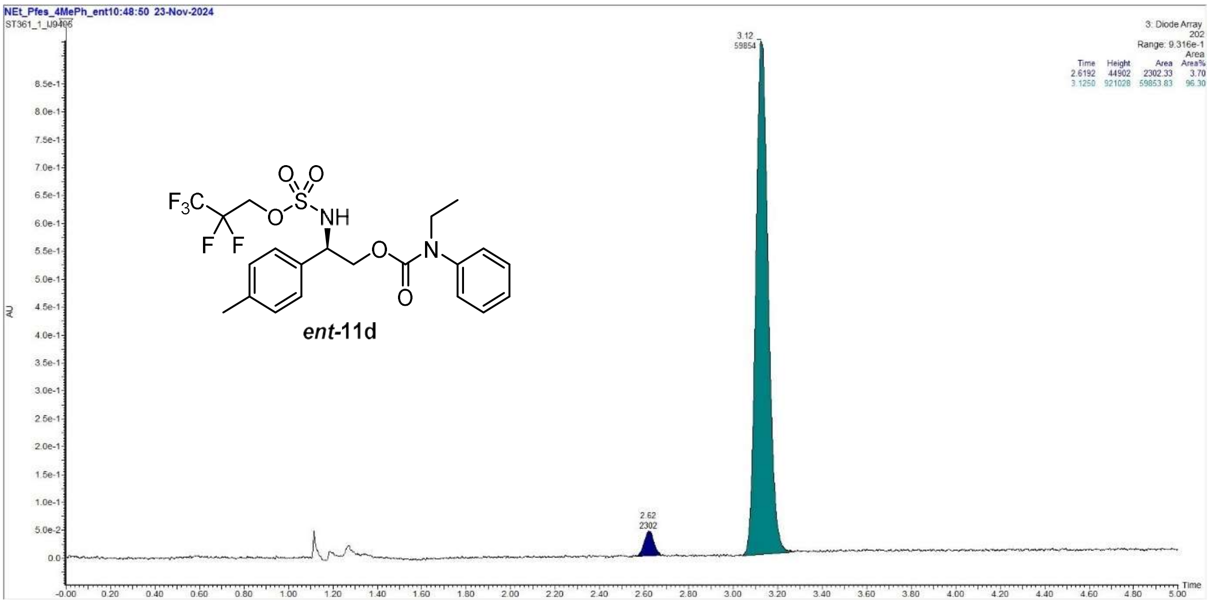

2,2,3,3,3-pentafluoropropyl (*S*)-(1-(4-bromophenyl)-2-((ethyl(phenyl)carbamoyl)oxy)ethyl)sulfamate (**11e**)

Chiral SFC Analysis CHIRALPAK IC (CO_2:MeOH, 95:5, 2.50 mL min^{-1}, 40 ˚C, 220 nm) indicated 88% *ee*, t_R = 3.6 (major), 4.4 (minor) minutes.

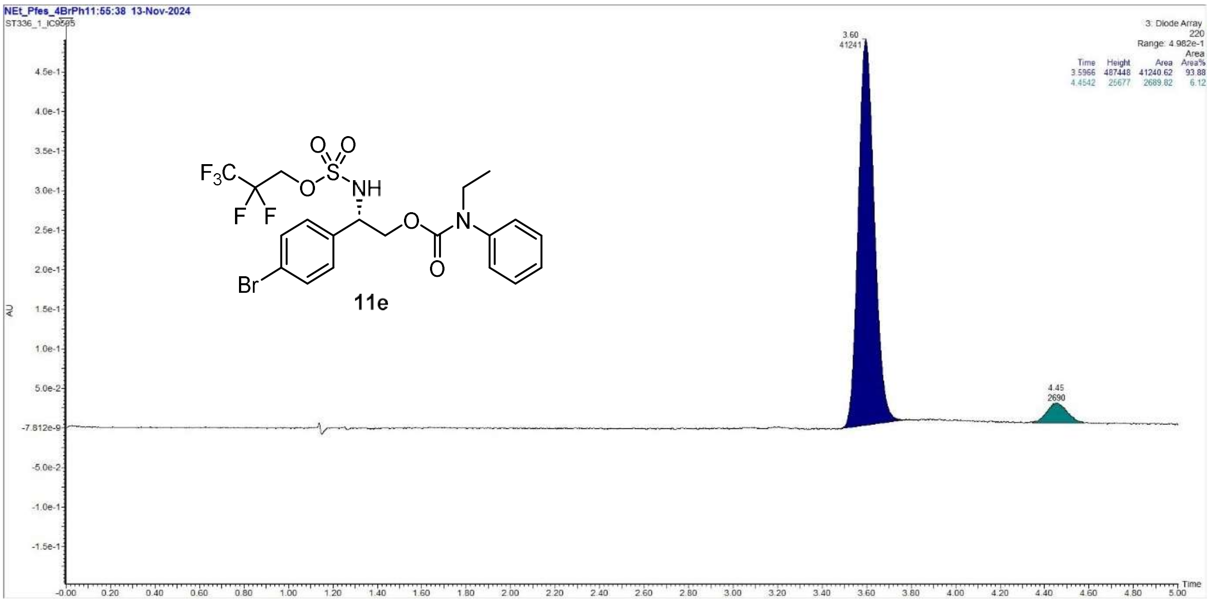

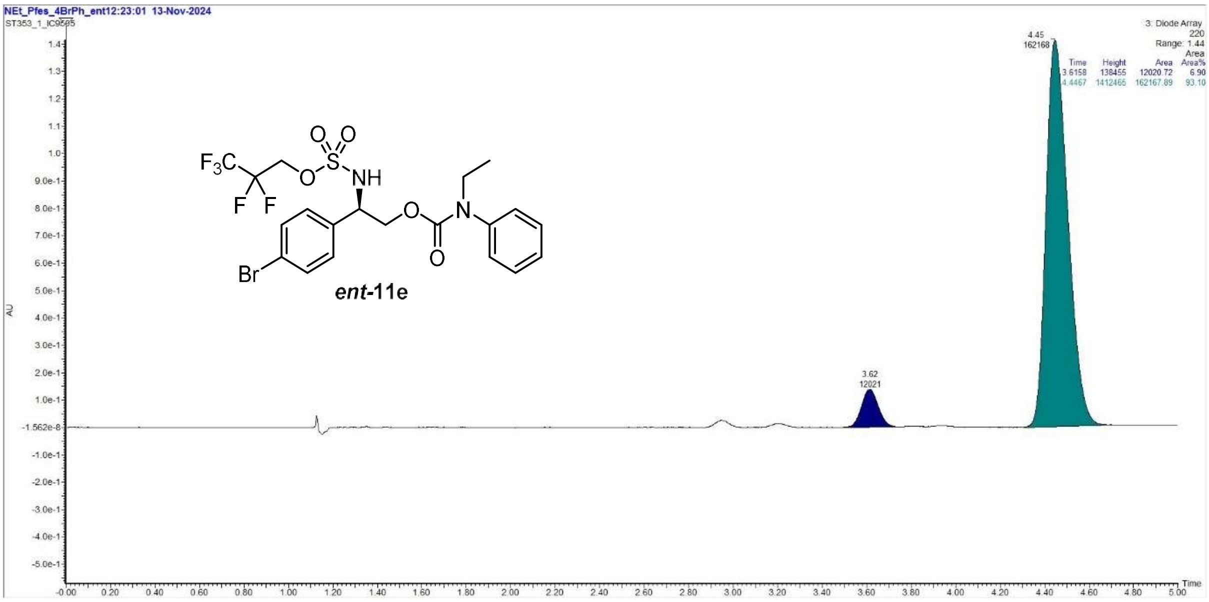

2,2,3,3,3-pentafluoropropyl (*S*)-(1-(4-chlorophenyl)-2-((ethyl(phenyl)carbamoyl)oxy)ethyl)sulfamate (**11f**)

Chiral SFC Analysis CHIRALPAK IJ (CO_2:MeOH, 94:6, 2.50 mL min^{-1}, 40 ˚C, 219 nm) indicated 90% *ee*, t_R = 3.5 (major), 4.3 (minor) minutes.

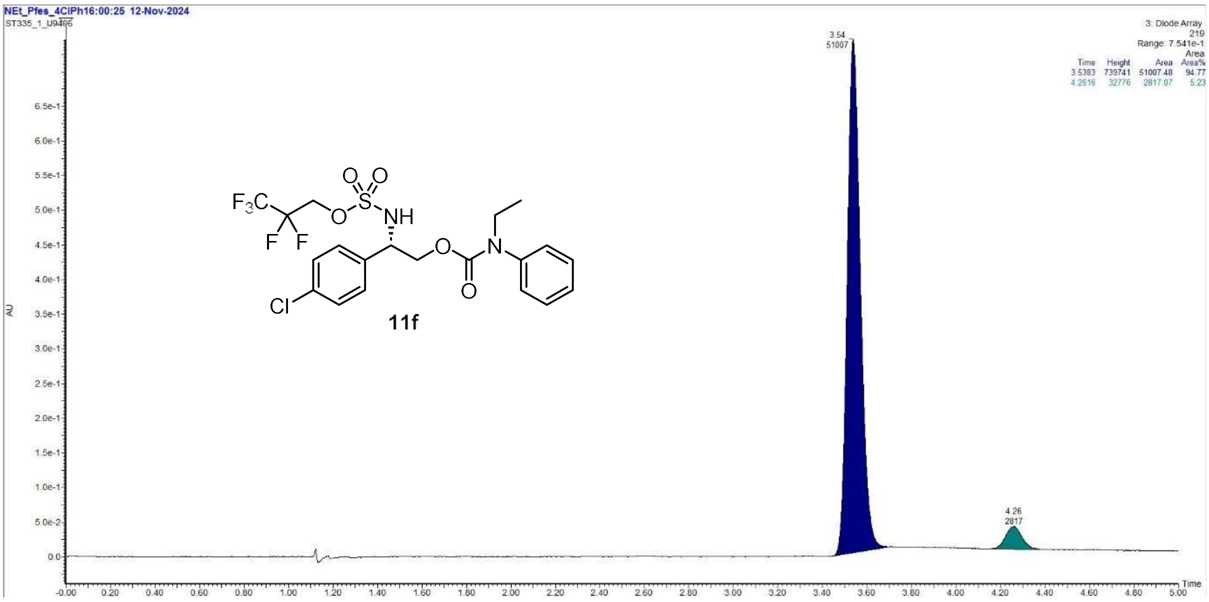

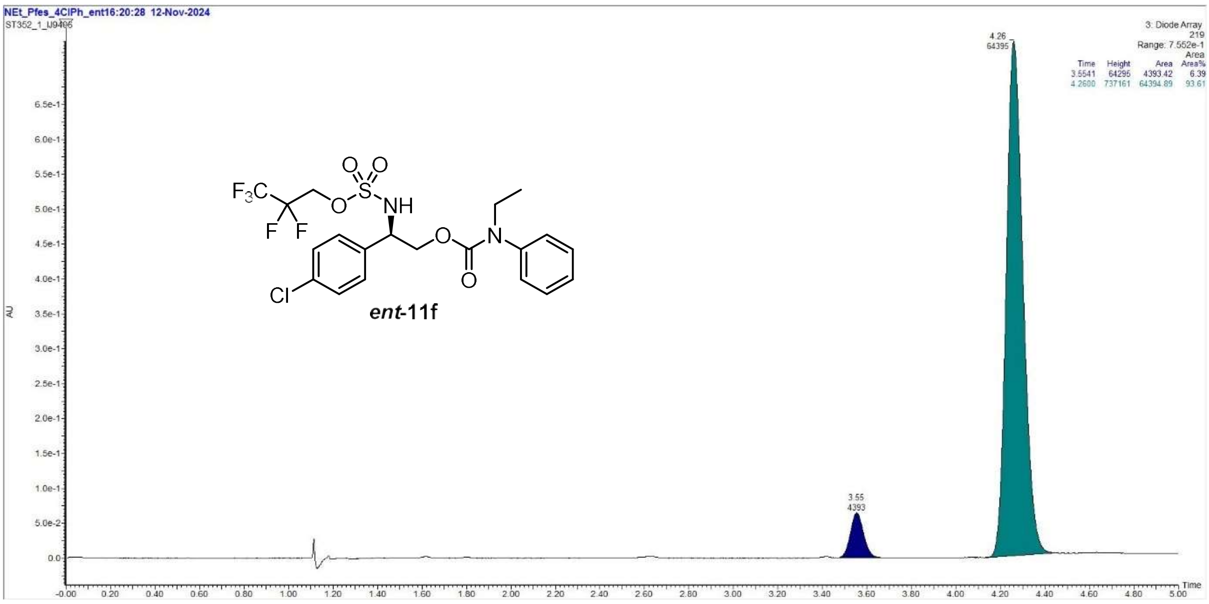

2,2,3,3,3-pentafluoropropyl (*S*)-(2-((ethyl(phenyl)carbamoyl)oxy)-1-(4-(trifluoromethyl)phenyl)ethyl)sulfamate (**11g**)

Chiral SFC Analysis CHIRALPAK IJ (CO_2:MeOH, 94:6, 2.50 mL min^{-1}, 40 ˚C, 202 nm) indicated 89% *ee*, t_R = 2.0 (major), 2.2 (minor) minutes.

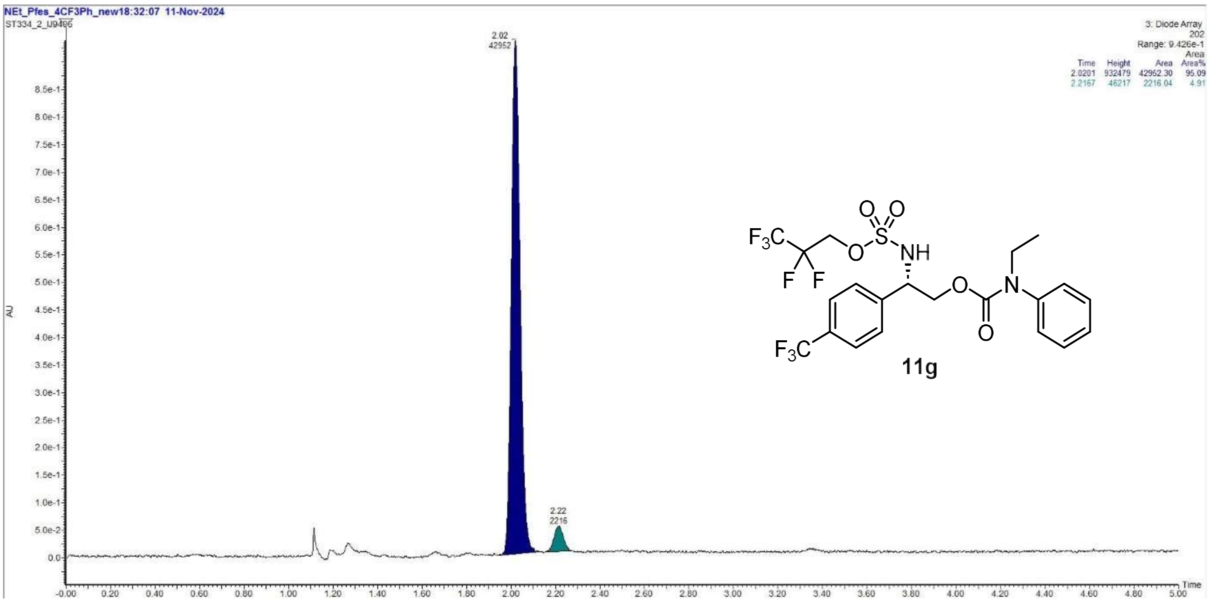

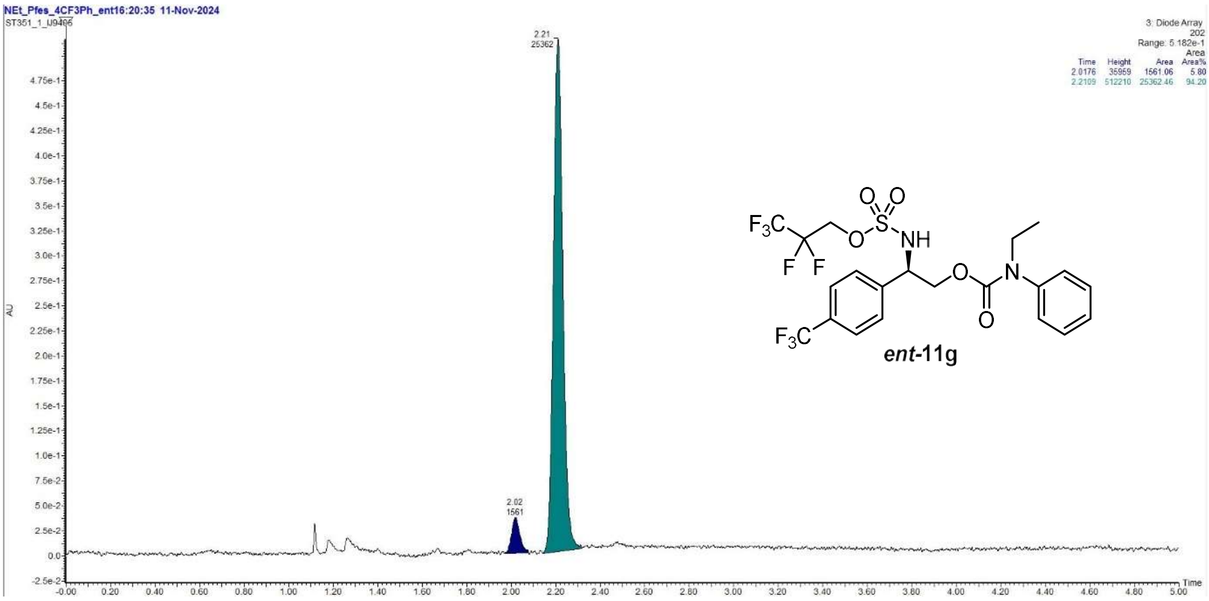

2,2,3,3,3-pentafluoropropyl (*S*)-(2-((ethyl(phenyl)carbamoyl)oxy)-1-(3-methoxyphenyl)ethyl)sulfamate (**11h**)

Chiral SFC Analysis CHIRALPAK IJ (CO_2:MeOH, 94:6, 2.50 mL min^{-1}, 40 ˚C, 202 nm) indicated 97% *ee*, t_R = 3.0 (major), 3.8 (minor) minutes.

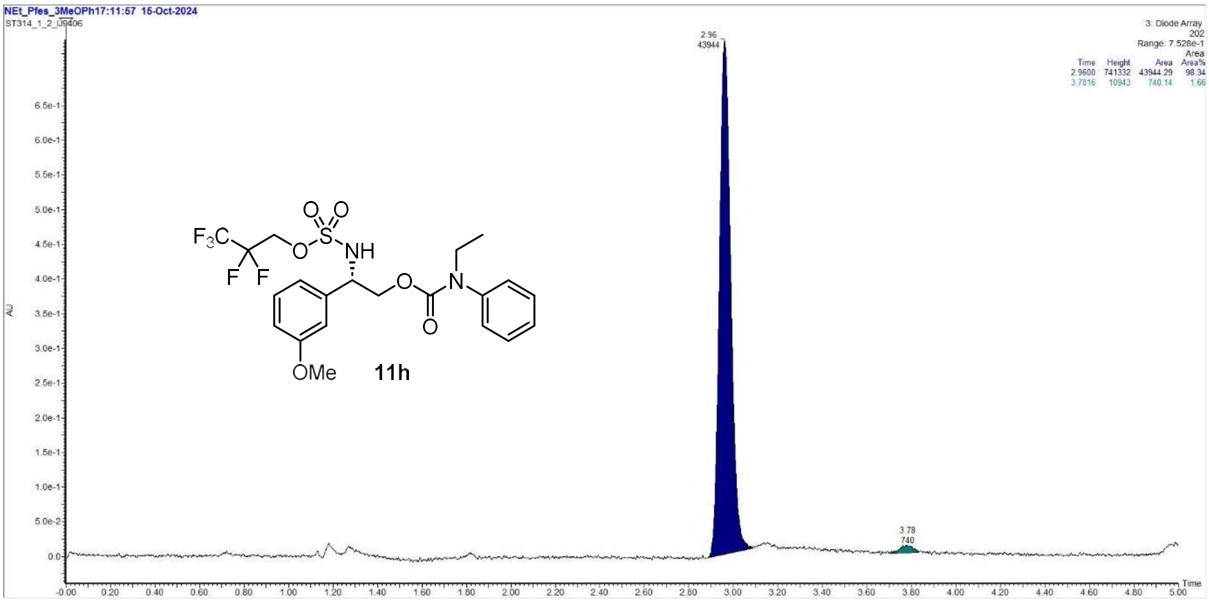

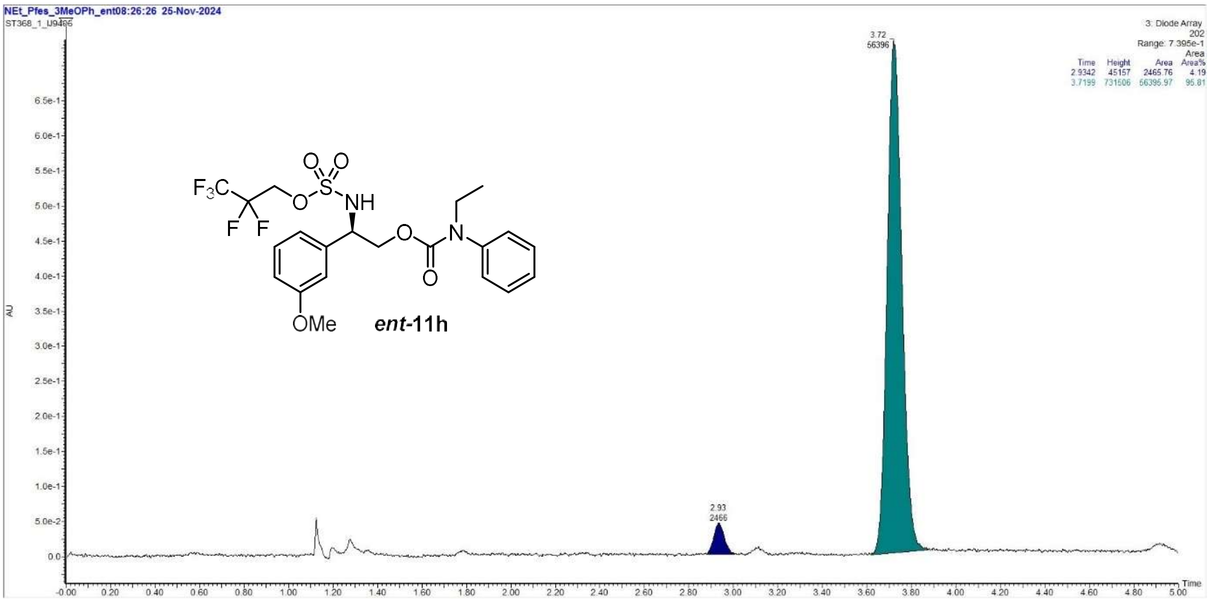

2,2,3,3,3-pentafluoropropyl (*S*)-(2-((ethyl(phenyl)carbamoyl)oxy)-1-(m-tolyl)ethyl)sulfamate (**11i**)

Chiral SFC Analysis CHIRALPAK IJ (CO_2:MeOH, 94:6, 2.50 mL min^{-1}, 40 ˚C, 202 nm) indicated 94% *ee*, t_R = 2.6 (major), 3.0 (minor) minutes.

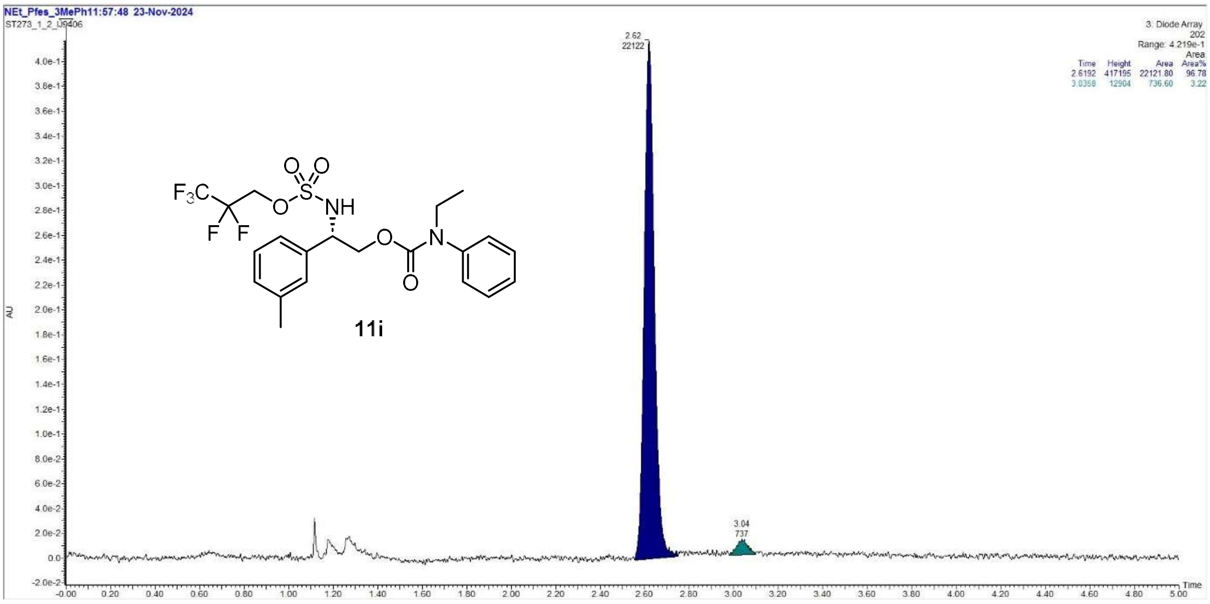

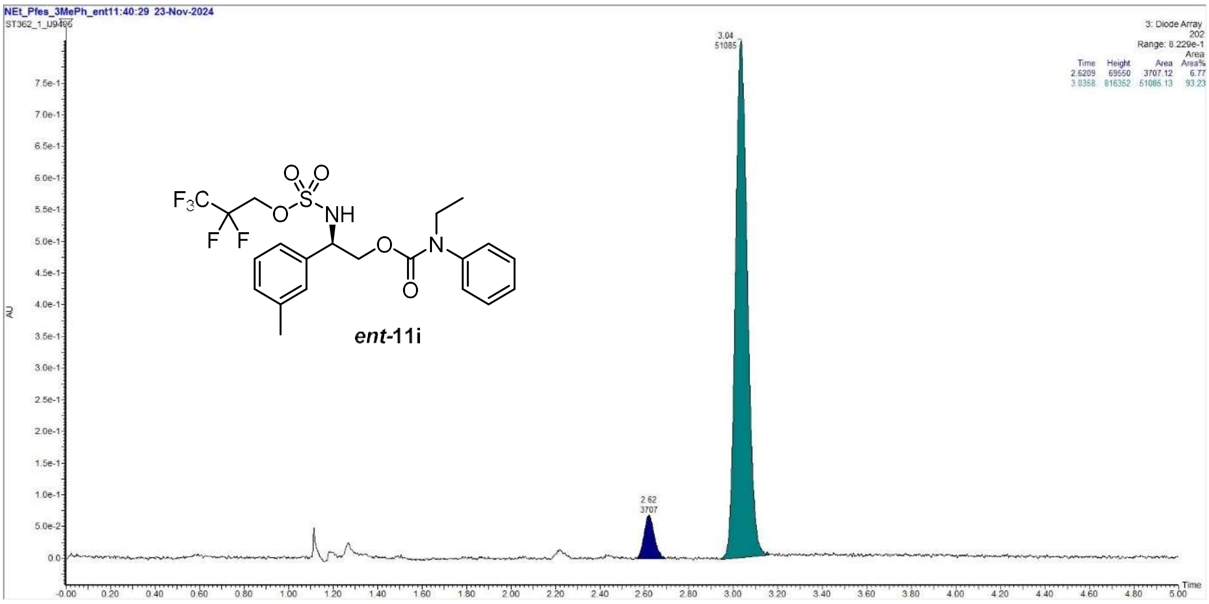

2,2,3,3,3-pentafluoropropyl (*S*)-(1-(3-chlorophenyl)-2-((ethyl(phenyl)carbamoyl)oxy)ethyl)sulfamate (**11j**)

Chiral SFC Analysis CHIRALPAK IJ (CO_2:MeOH, 94:6, 2.50 mL min^{-1}, 40 ˚C, 202 nm) indicated 92% *ee*, t_R = 3.4 (major), 4.1 (minor) minutes.

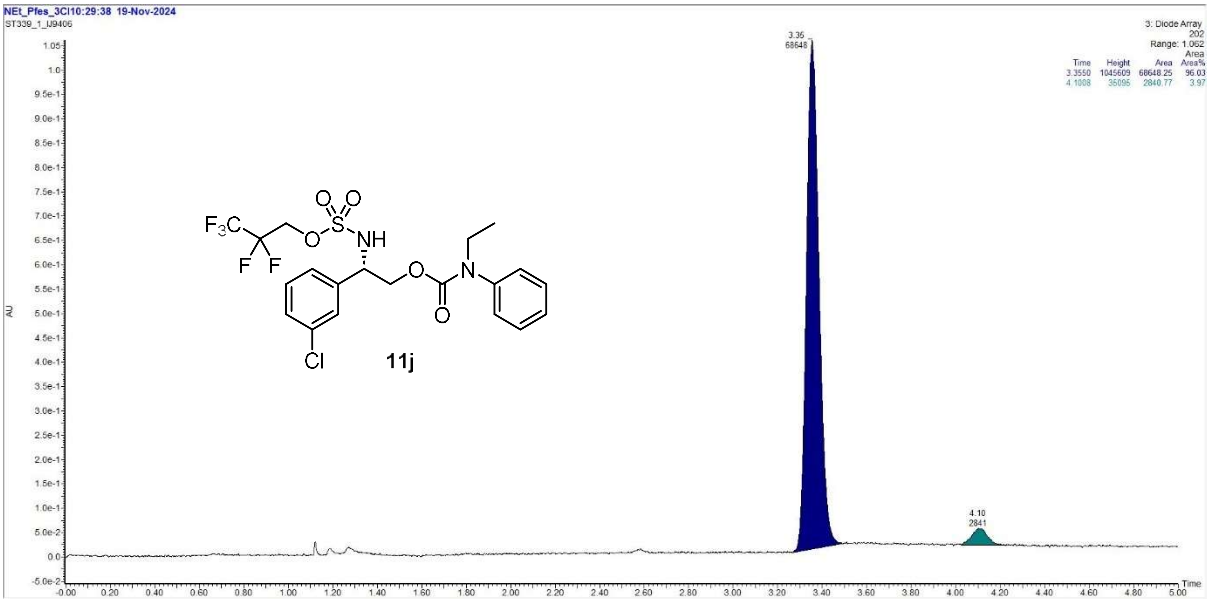

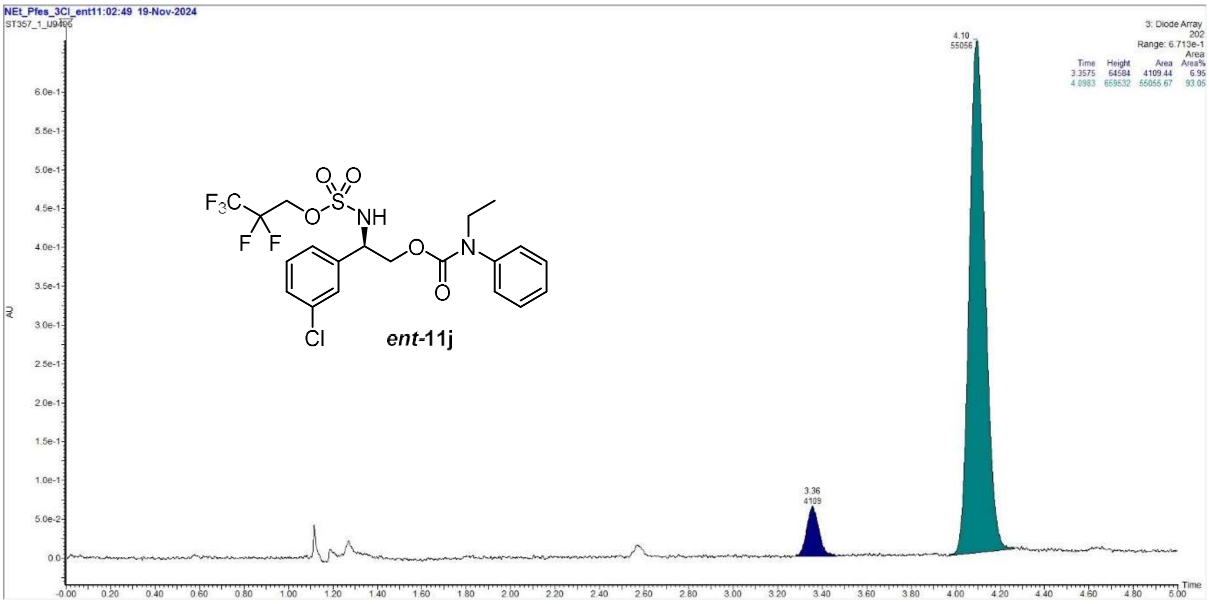

2,2,3,3,3-pentafluoropropyl (*S*)-(1-(3-bromophenyl)-2-((ethyl(phenyl)carbamoyl)oxy)ethyl)sulfamate (**11k**)

Chiral SFC Analysis CHIRALPAK IJ (CO_2:MeOH, 94:6, 2.50 mL min^{-1}, 40 ˚C, 202 nm) indicated 93% *ee*, t_R = 3.9 (major), 4.8 (minor) minutes.

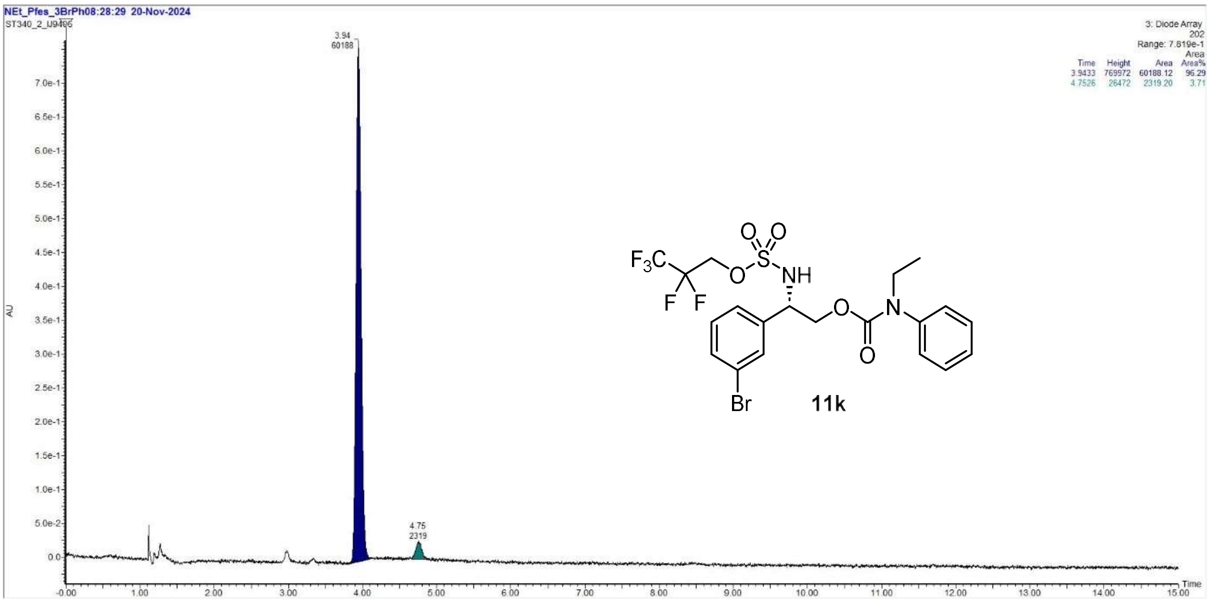

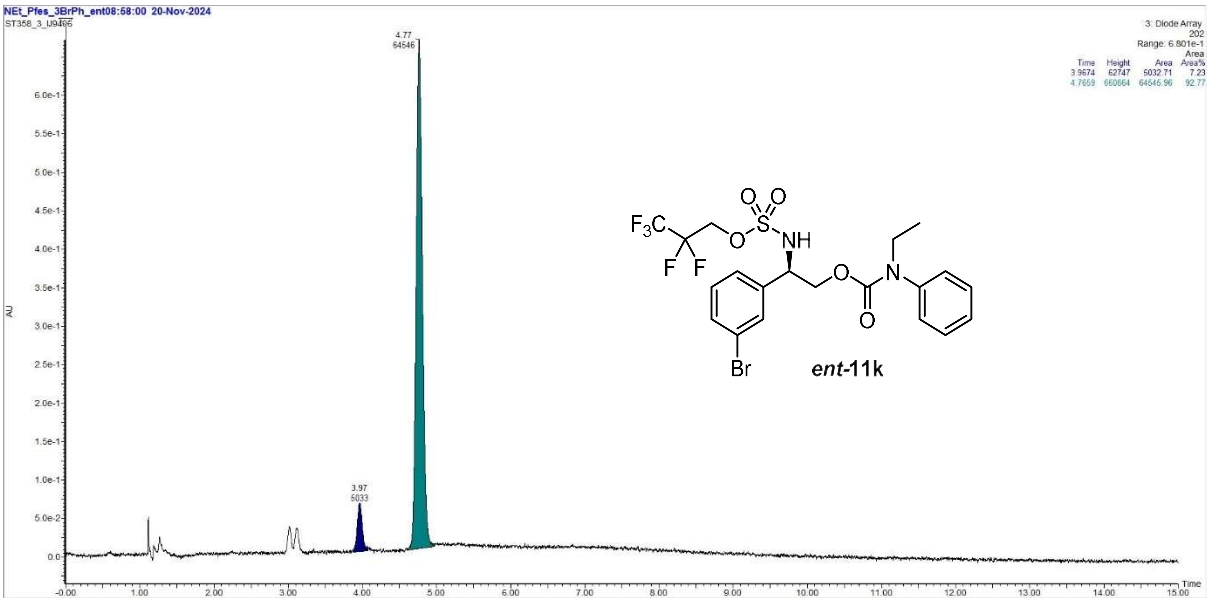

2,2,3,3,3-pentafluoropropyl (*S*)-(2-((ethyl(phenyl)carbamoyl)oxy)-1-(3-(trifluoromethyl)phenyl)ethyl)sulfamate (**11l**)

Chiral SFC Analysis CHIRALPAK IJ (CO_2:MeOH, 94:6, 2.50 mL min^{-1}, 40 ˚C, 202 nm) indicated 91% *ee*, t_R = 2.0 (major), 2.3 (minor) minutes.

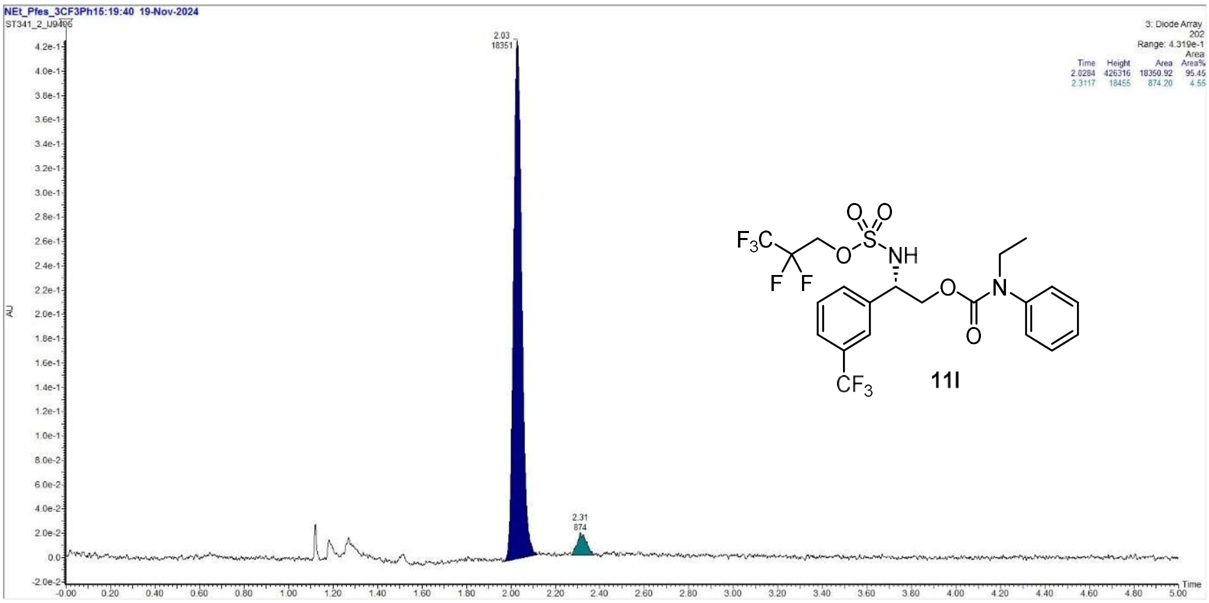

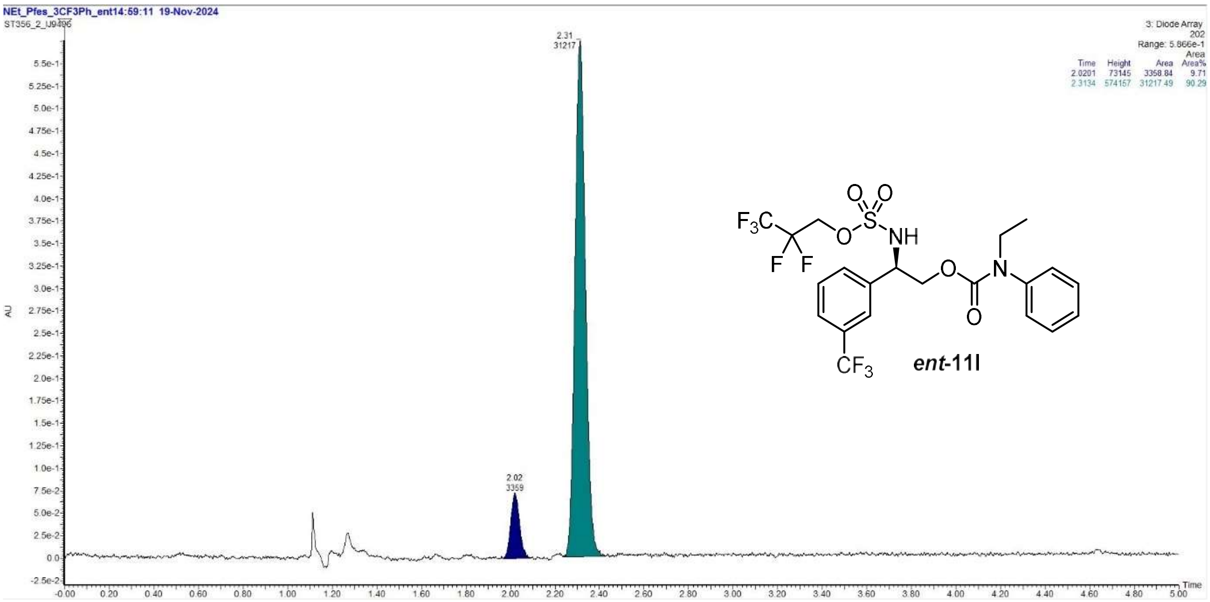

2,2,3,3,3-pentafluoropropyl (*S*)-(2-((ethyl(phenyl)carbamoyl)oxy)-1-(o-tolyl)ethyl)sulfamate (**11m**)

Chiral SFC Analysis CHIRALPAK IJ (CO_2:MeOH, 94:6, 2.50 mL min^{-1}, 40 ˚C, 202 nm) indicated 96% *ee*, t_R = 2.5 (major), 3.0 (minor) minutes.

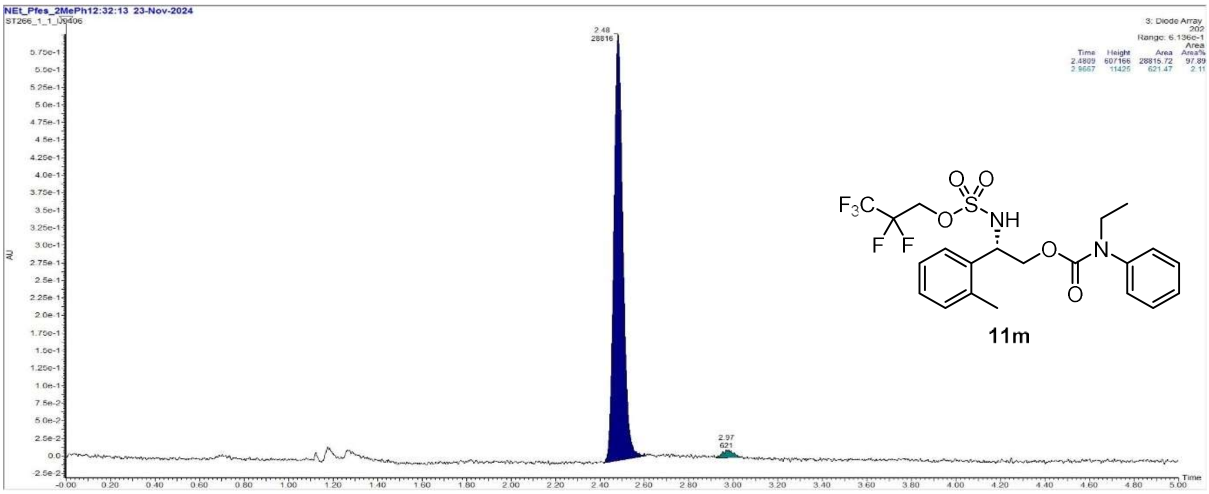

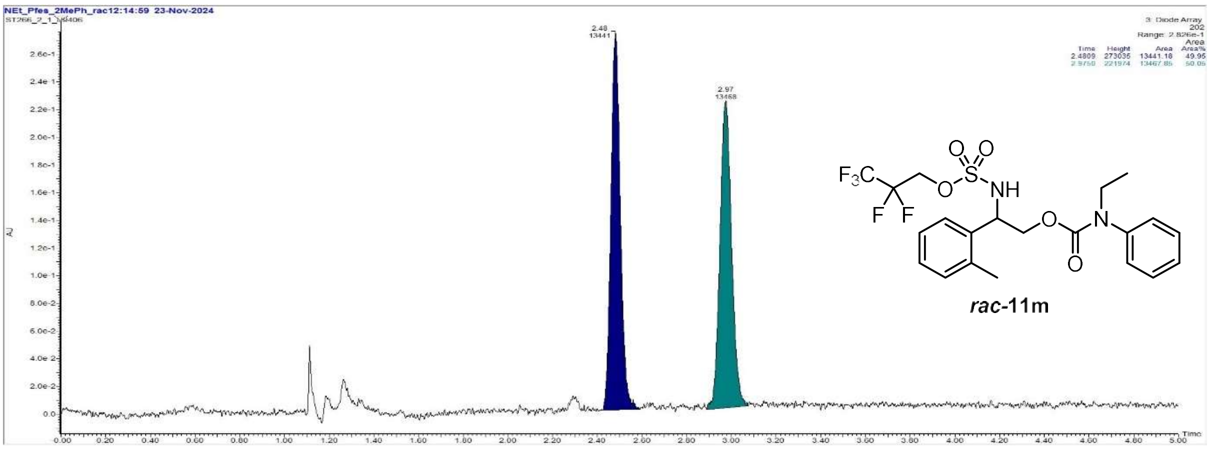

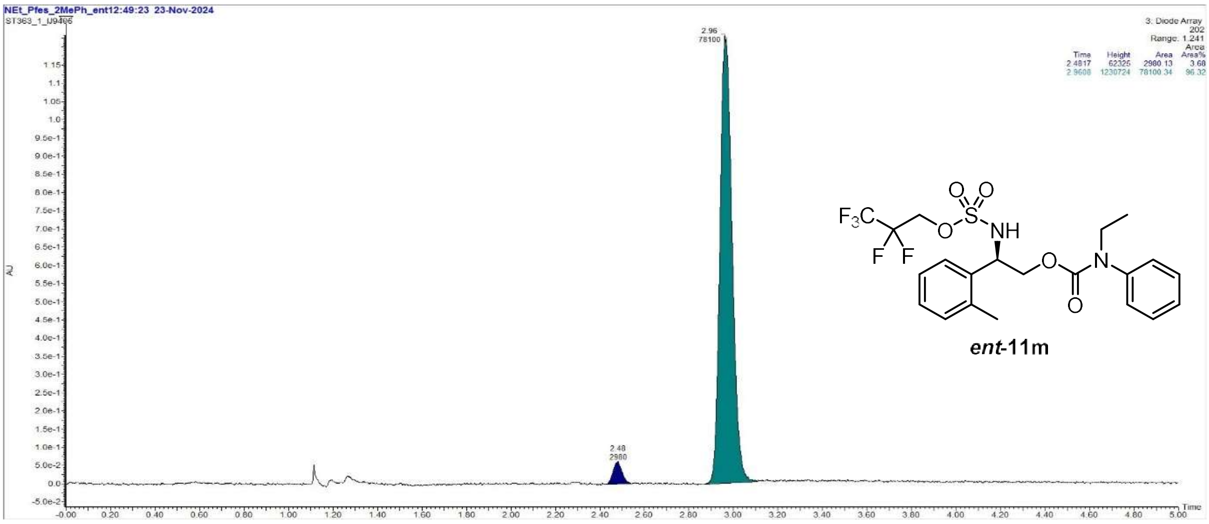

2,2,3,3,3-pentafluoropropyl (*S*)-(1-(2-chlorophenyl)-2-((ethyl(phenyl)carbamoyl)oxy)ethyl)sulfamate (**11n**)

Chiral SFC Analysis CHIRALPAK IJ (CO_2:MeOH, 94:6, 2.50 mL min^{-1}, 40 ˚C, 202 nm) indicated 85% *ee*, t_R = 2.9 (major), 3.8 (minor) minutes.

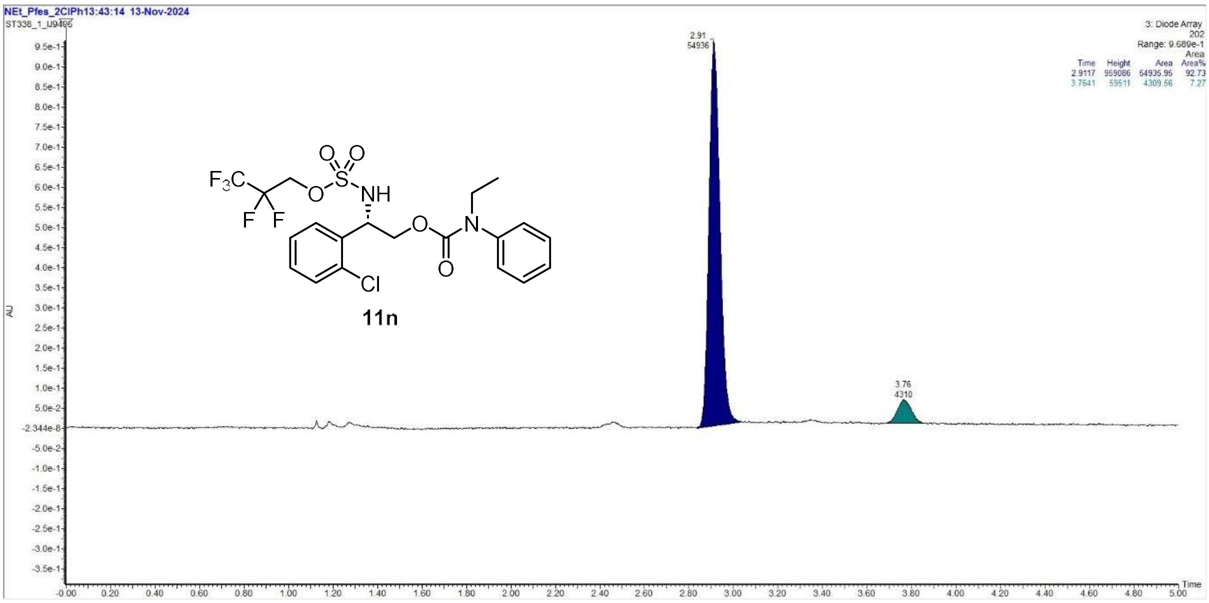

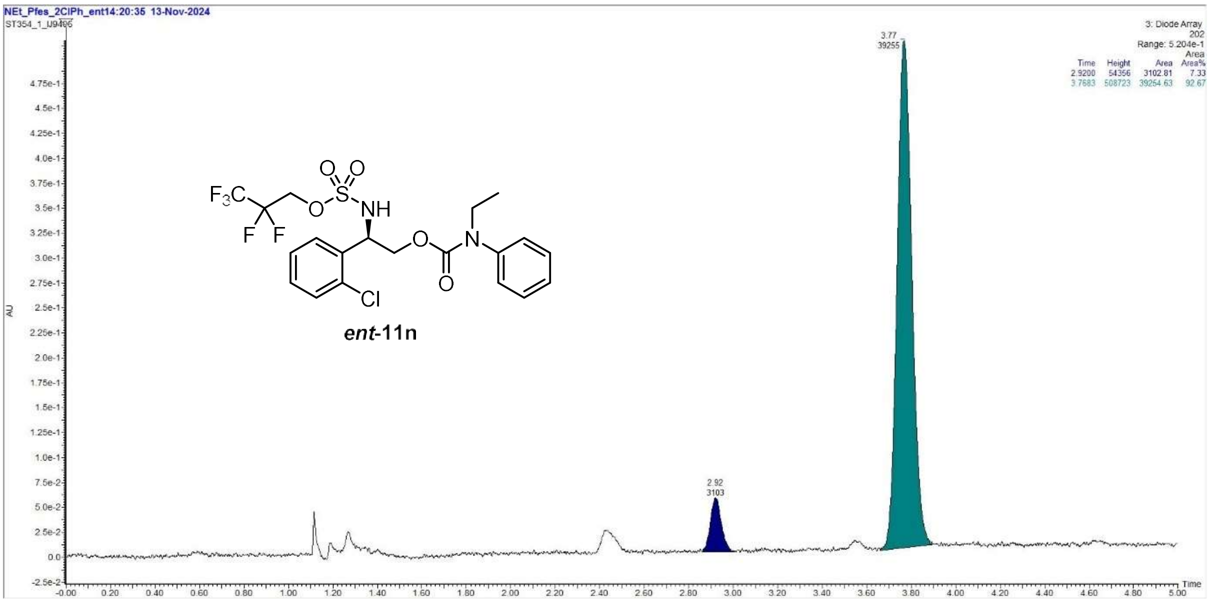

2,2,3,3,3-pentafluoropropyl (*S*)-(1-(2-bromophenyl)-2-((ethyl(phenyl)carbamoyl)oxy)ethyl)sulfamate (**11o**)

Chiral SFC Analysis CHIRALPAK IJ (CO_2:MeOH, 94:6, 2.50 mL min^{-1}, 40 ˚C, 202 nm) indicated 82% *ee*, t_R = 3.3 (major), 4.4 (minor) minutes.

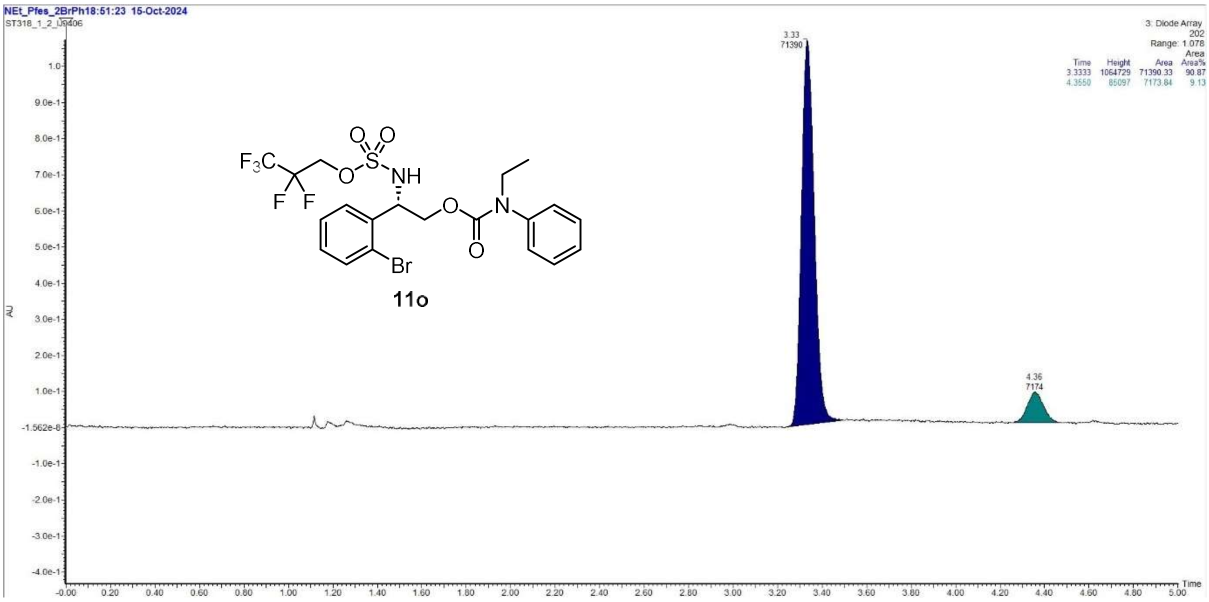

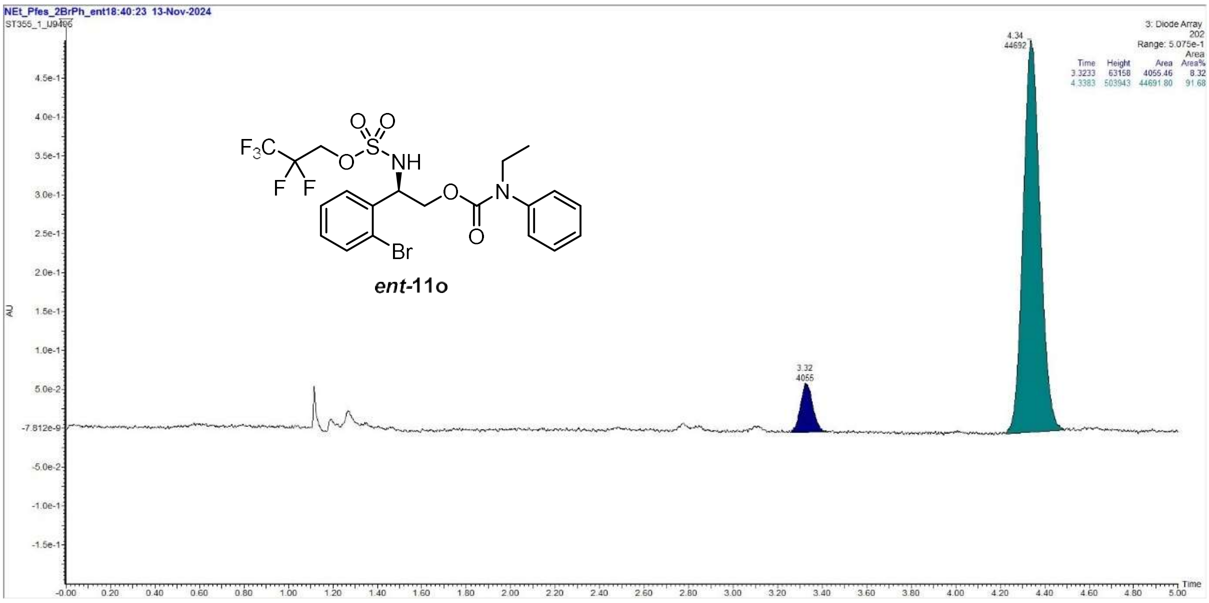

2,2,3,3,3-pentafluoropropyl (*S*)-(2-((ethyl(phenyl)carbamoyl)oxy)-1-(thiophen-2-yl)ethyl)sulfamate (**11p**)

Chiral SFC Analysis CHIRALPAK IJ (CO_2:MeOH, 94:6, 2.50 mL min^{-1}, 40 ˚C, 228 nm) indicated 98% *ee*, t_R = 3.0 (major), 3.7 (minor) minutes.

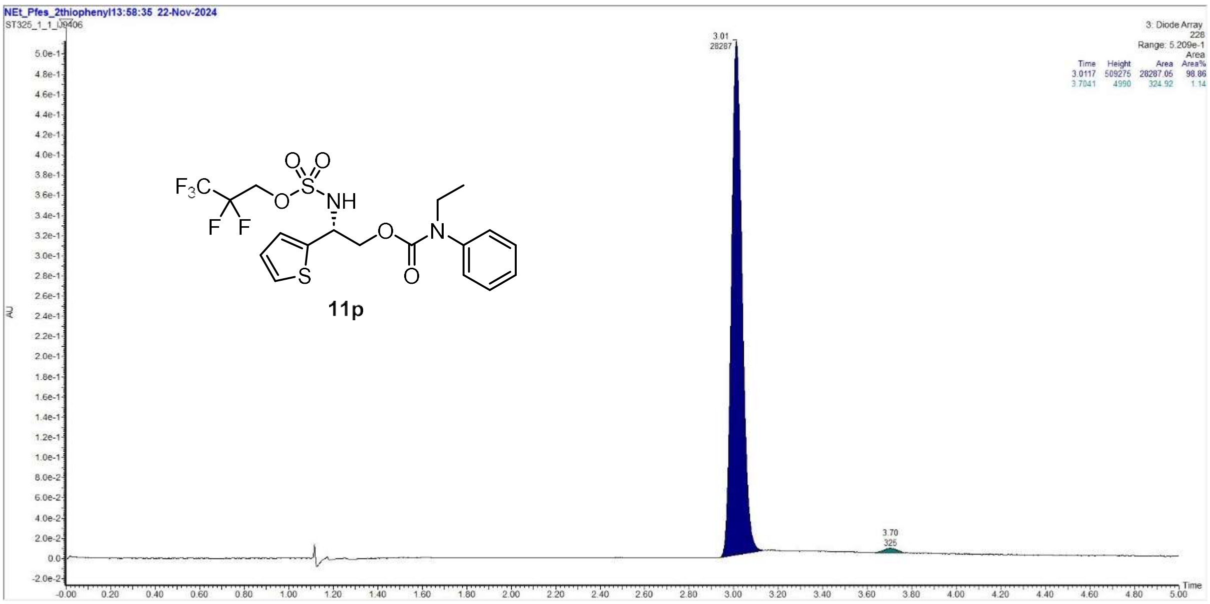

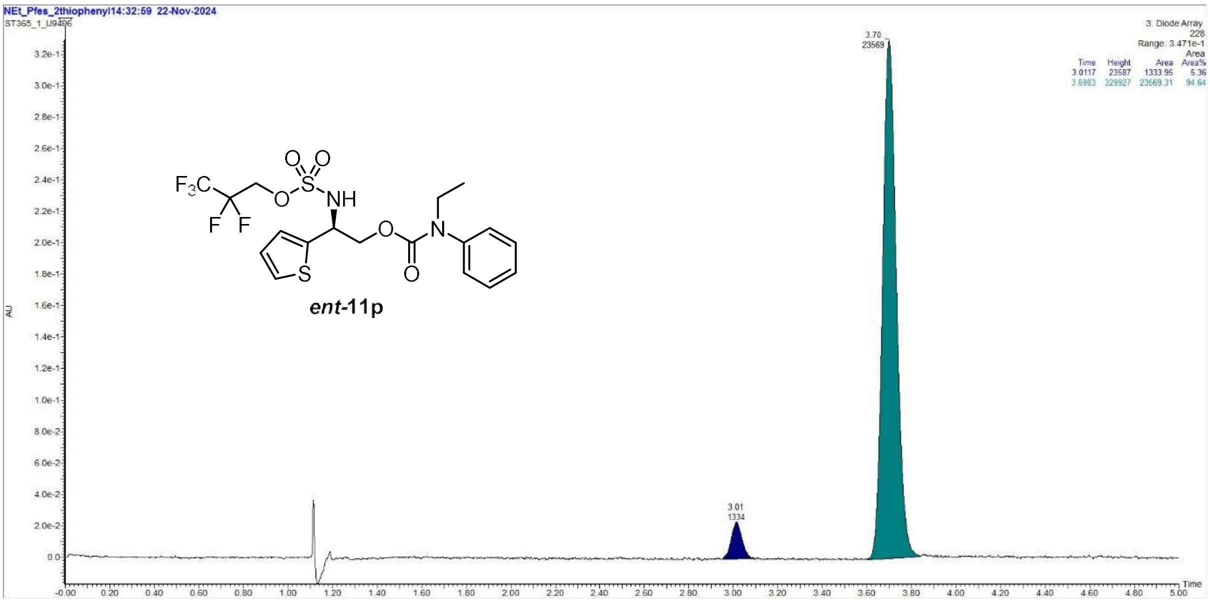

2,2,3,3,3-pentafluoropropyl (*S*)-(2-((ethyl(phenyl)carbamoyl)oxy)-1-(thiophen-3-yl)ethyl)sulfamate (**11q**)

Chiral SFC Analysis CHIRALPAK IJ (CO_2:MeOH, 94:6, 2.50 mL min^{-1}, 40 ˚C, 228 nm) indicated 94% *ee*, t_R = 3.2 (major), 3.9 (minor) minutes.

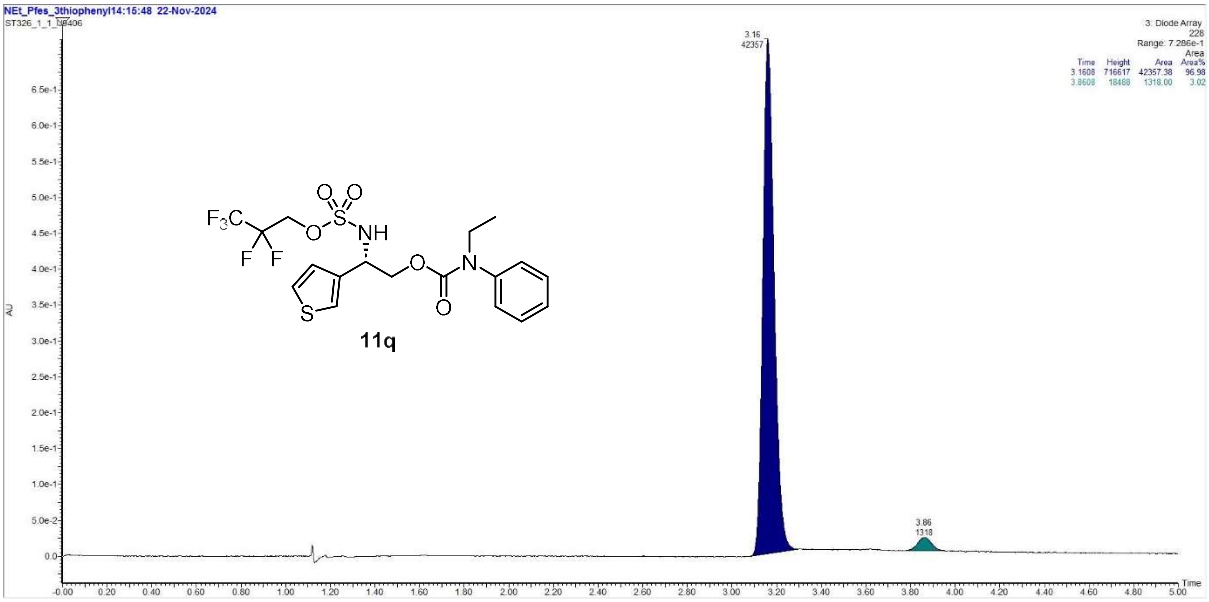

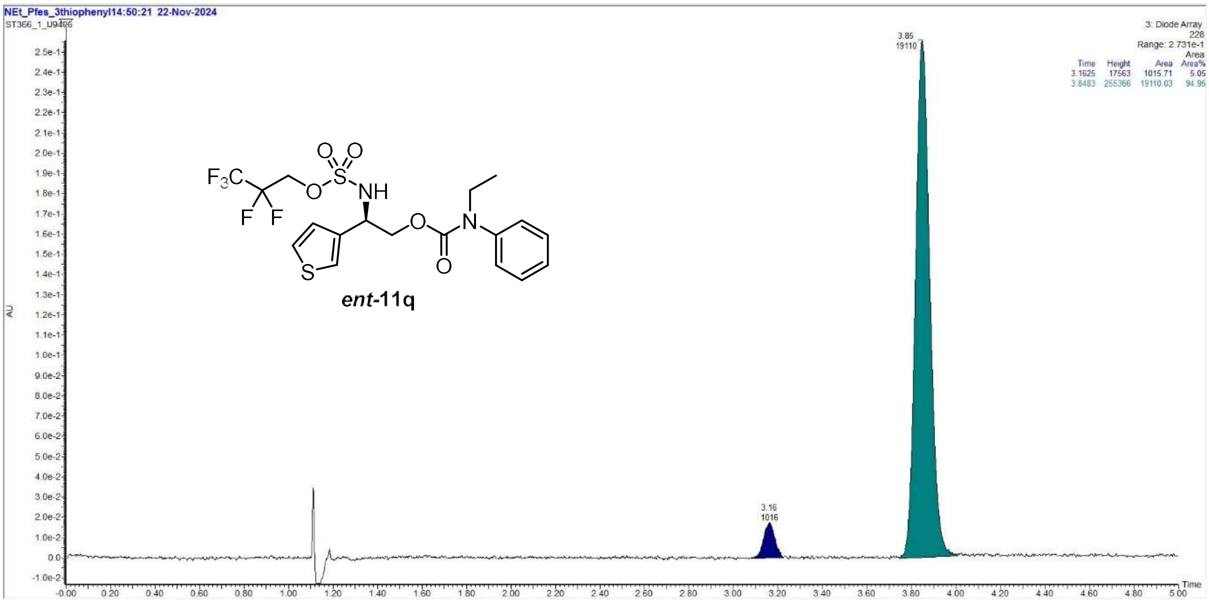

2,2,3,3,3-pentafluoropropyl ((1*S*,2*S*)-2-((ethyl(phenyl)carbamoyl)oxy)-2,3-dihydro-1*H*-inden-1-yl)sulfamate (**11r**)

Chiral SFC Analysis CHIRALPAK IJ (CO_2:MeOH, 94:6, 2.50 mL min^{-1}, 40℃, 202 nm) indicated 72% *ee*, t_R = 3.2 (major), 3.5 (minor) minutes.

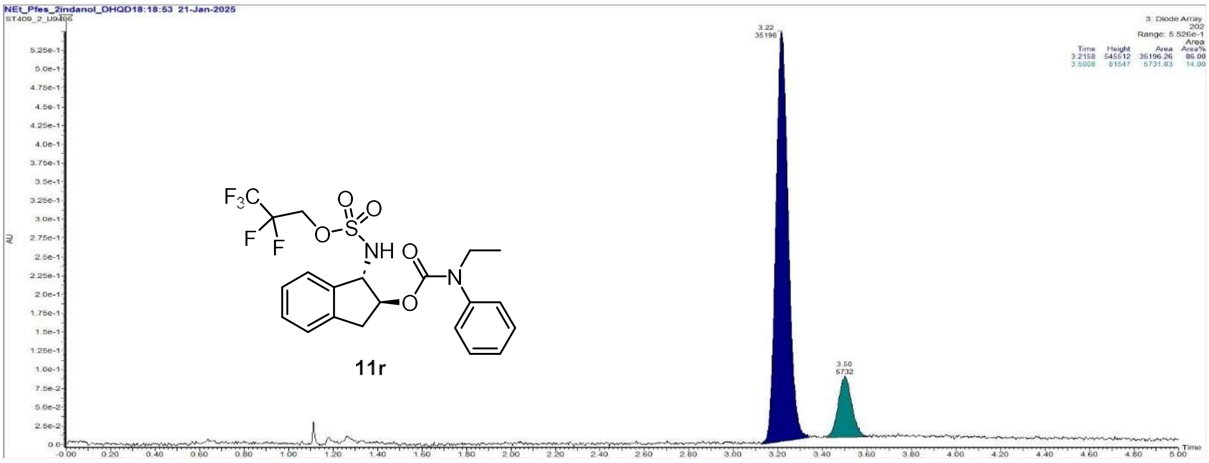

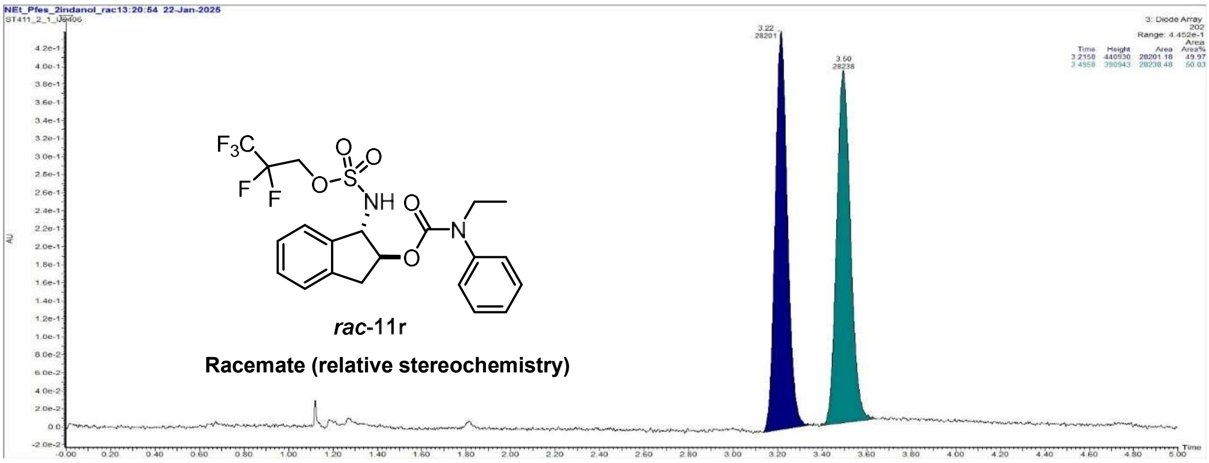

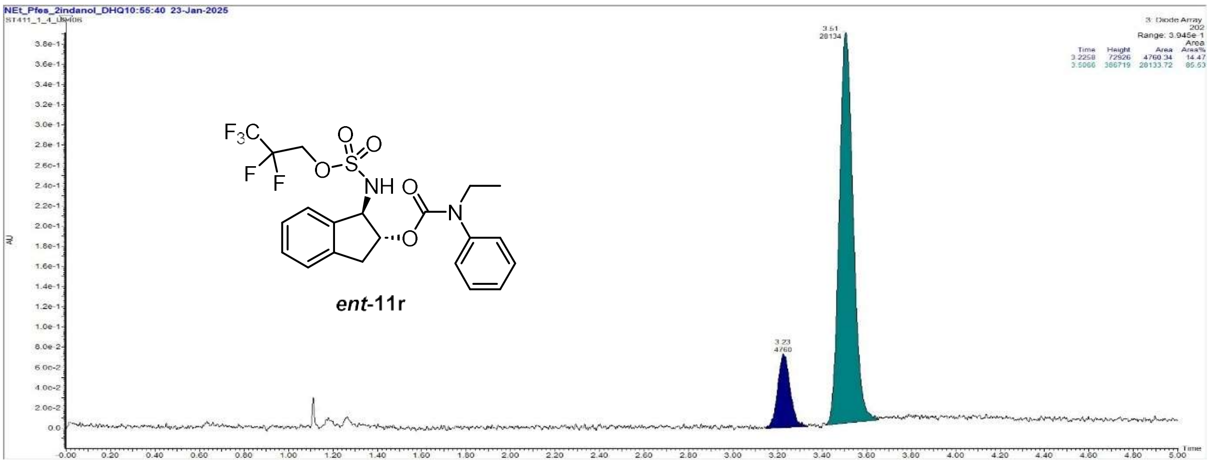

2,2,3,3,4,4,4-heptafluorobutyl (*S*)-(2-((methyl(phenyl)carbamoyl)oxy)-1-phenylethyl)sulfamate (**S5a**)

Chiral SFC Analysis CHIRALPAK IJ (CO_2:MeOH, 94:6, 2.50 mL min^{-1}, 40 ˚C, 227 nm) indicated 92% *ee*, t_R = 2.6 (N-Me aminated byproduct: **S6a**), 2.8 (minor), 3.1 (major) minutes.

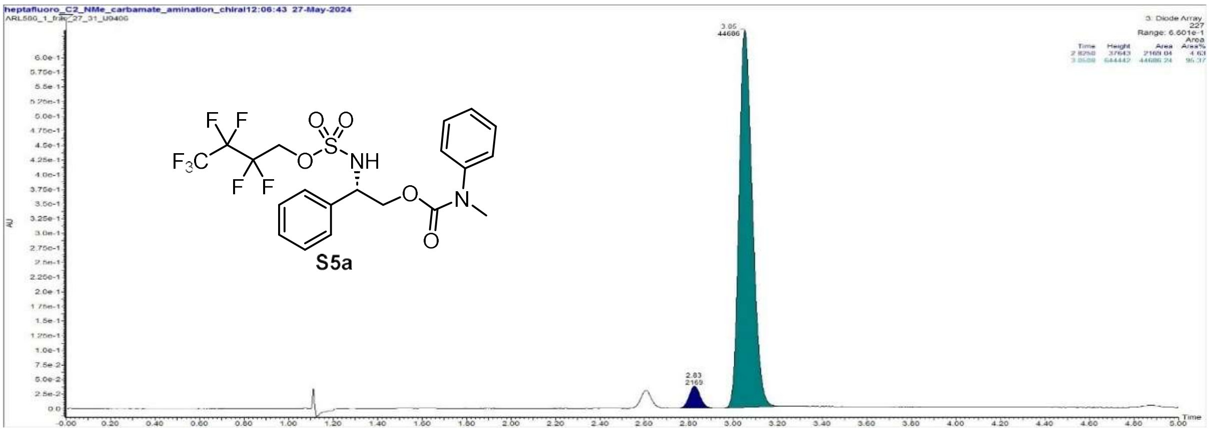

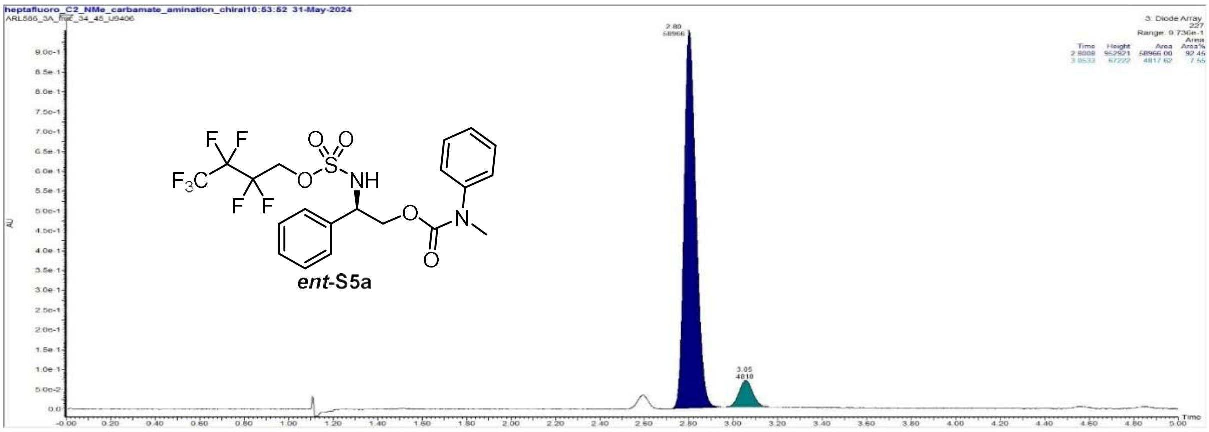

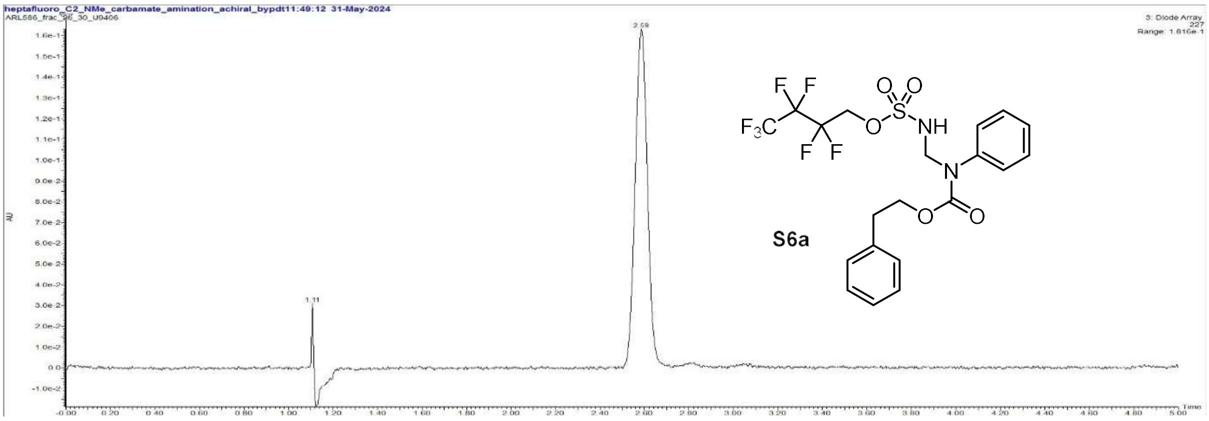

phenethyl 3-(((2,2,3,3,3-pentafluoropropoxy)sulfonyl)amino)morpholine-4-carboxylate

Chiral SFC Analysis: CHIRALPAK IJ (CO_2:MeOH, 98:2, 2.50 mL min^{-1}, 40 ˚C, 203 nm) indicated 22% *ee*, t_R = 7.3 (major), 8.1 (minor) minutes.

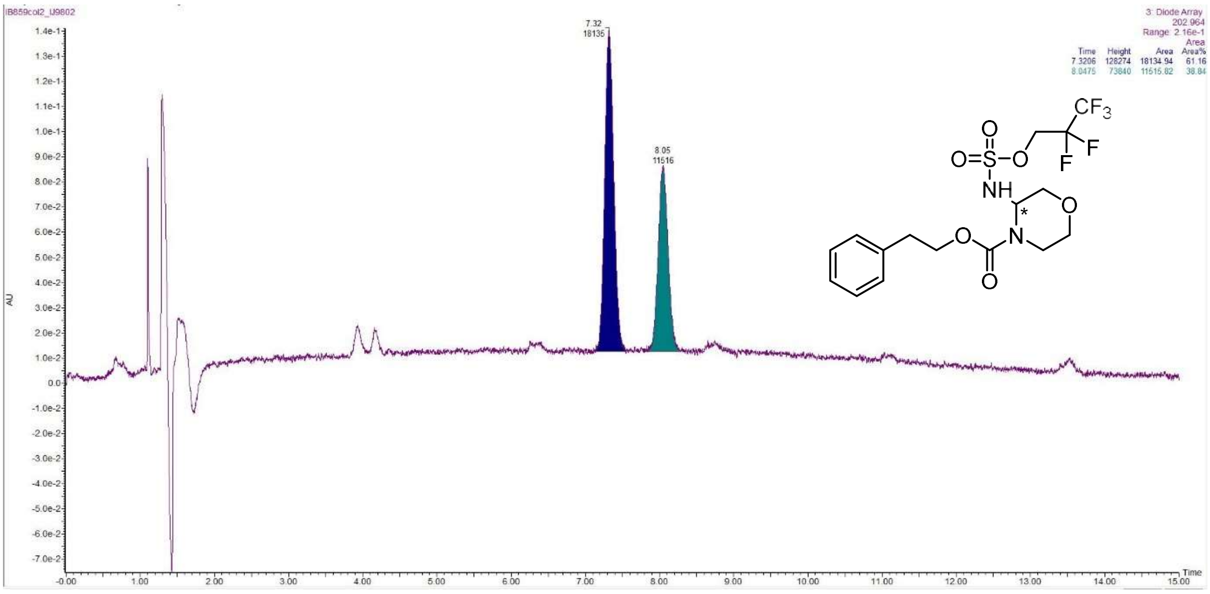

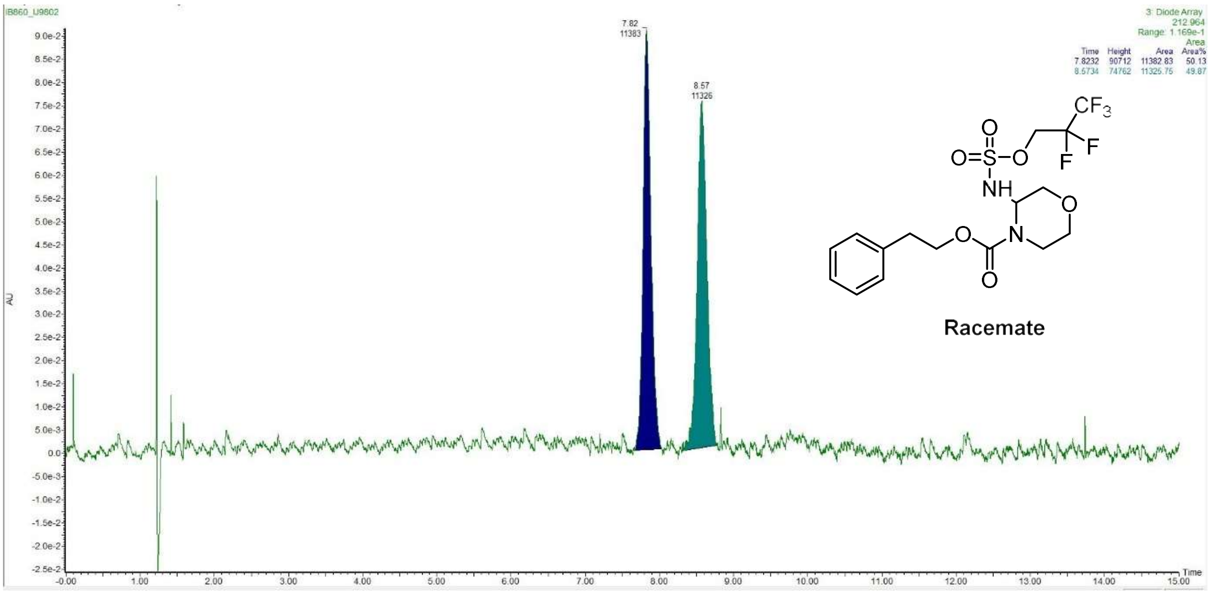

2,2,3,3,3-pentafluoropropyl (1-((phenethoxycarbonyl)(phenyl)amino)ethyl)sulfamate (**S7**)

Chiral SFC Analysis CHIRALPAK IC (CO_2:MeOH, 97:3, 2.50 mL min^{-1}, 40 ˚C, 202 nm) indicated 38% *ee*, t_R = 3.2 (minor), 3.5 (major) minutes.

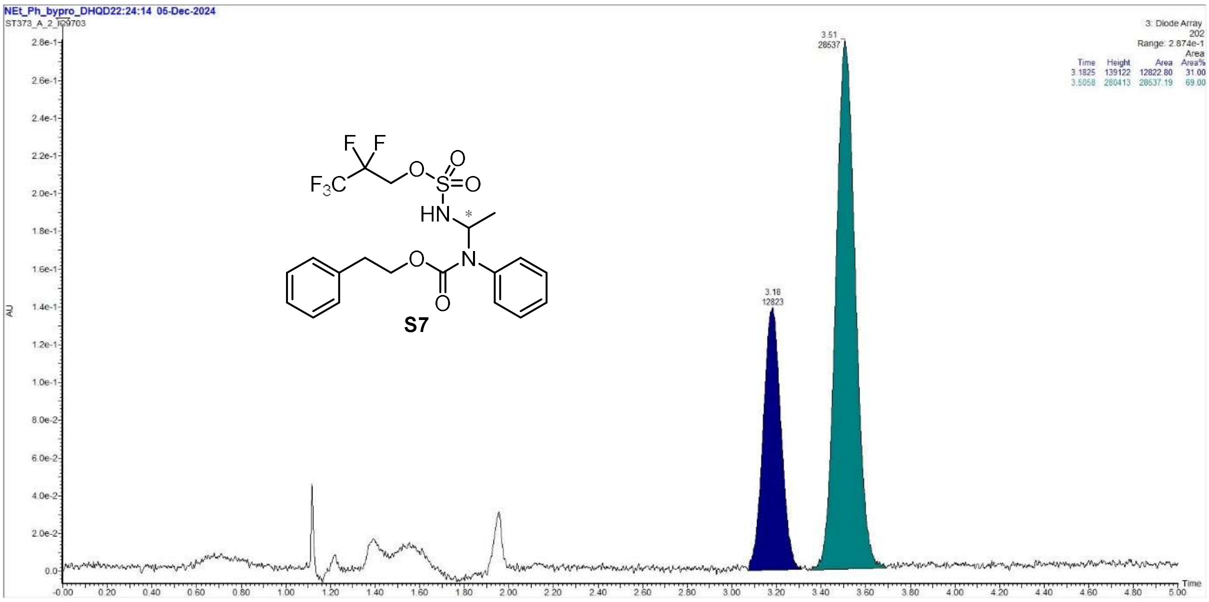

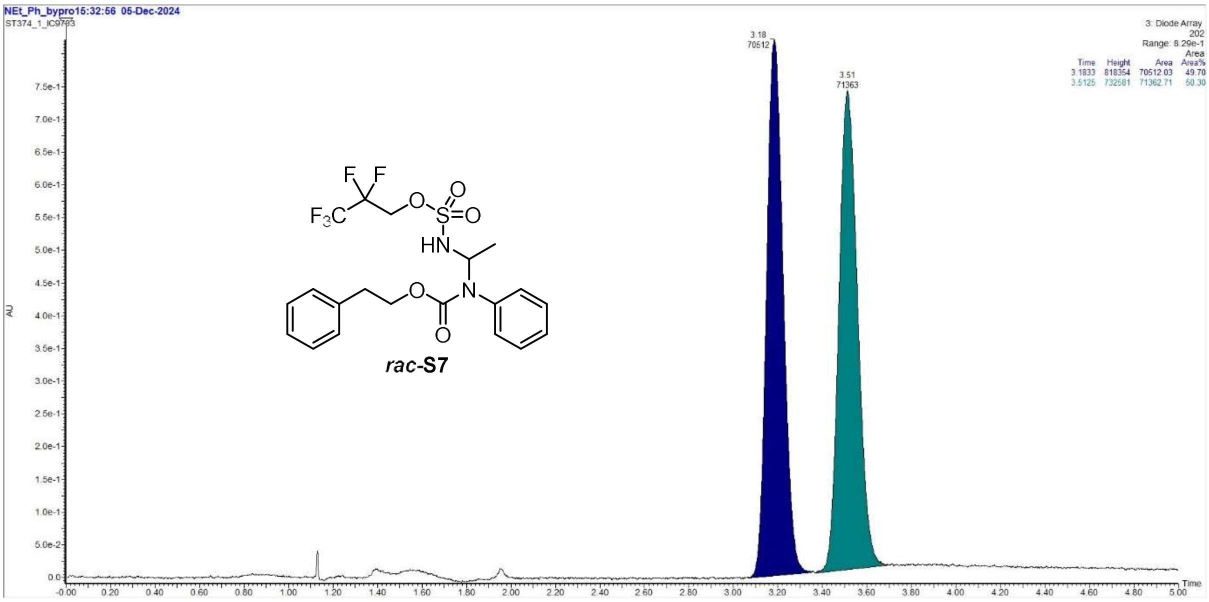

(*R*)-1-phenylpropan-2-yl ethyl(phenyl)carbamate (**(*R*)-10s**)

Chiral SFC Analysis CHIRALPAK IJ (CO_2:MeOH, 94:6, 2.50 mL min^{-1}, 40 ˚C, 202 nm) indicated 38% *ee*, t_R = 2.9 (major), 3.3 (minor) minutes.

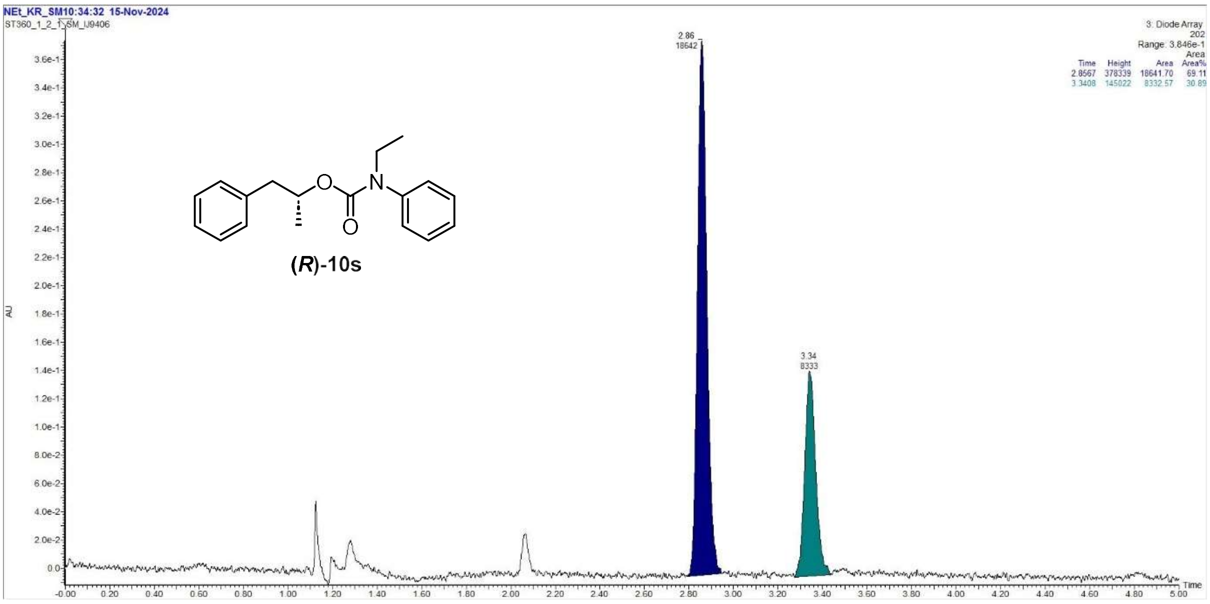

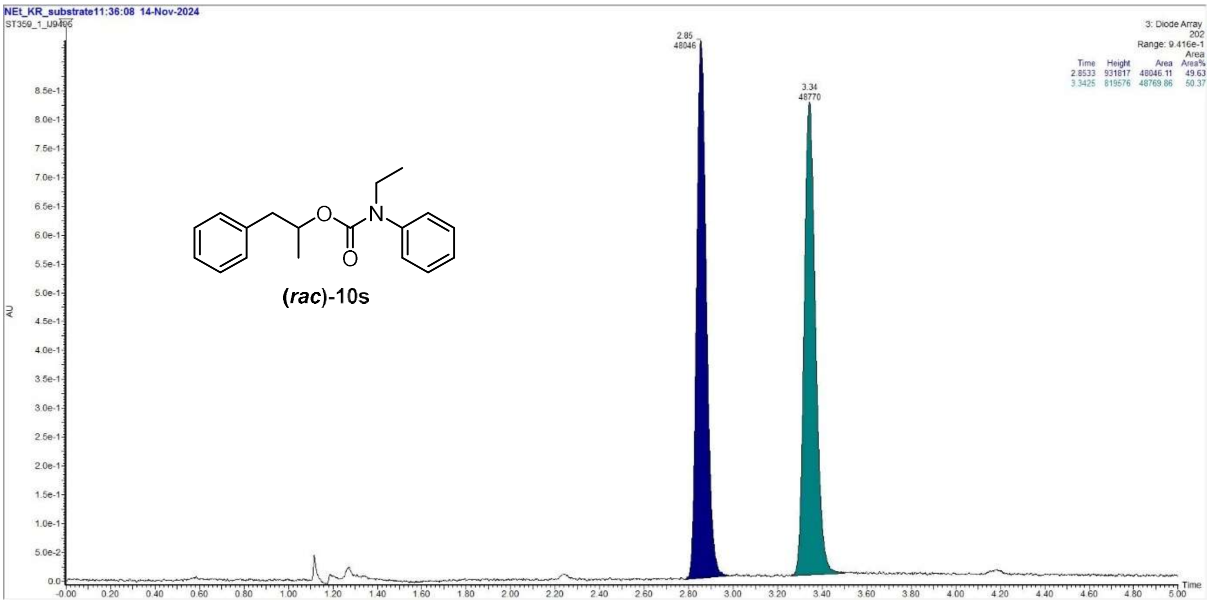

2,2,3,3,3-pentafluoropropyl ((1*S*,2*S*)-2-((ethyl(phenyl)carbamoyl)oxy)-1-phenylpropyl)sulfamate (**11s**)

Chiral SFC Analysis CHIRALPAK IJ (CO_2:MeOH, 94:6, 2.50 mL min^{-1}, 40 ˚C, 202 nm) indicated 99% *ee*, t_R = 2.0 (major), 2.5 (minor) minutes.

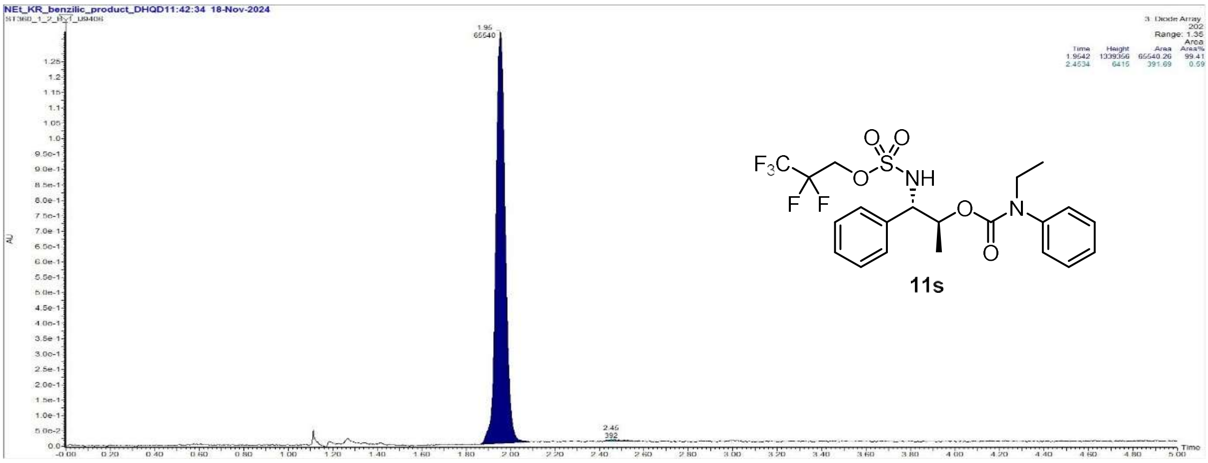

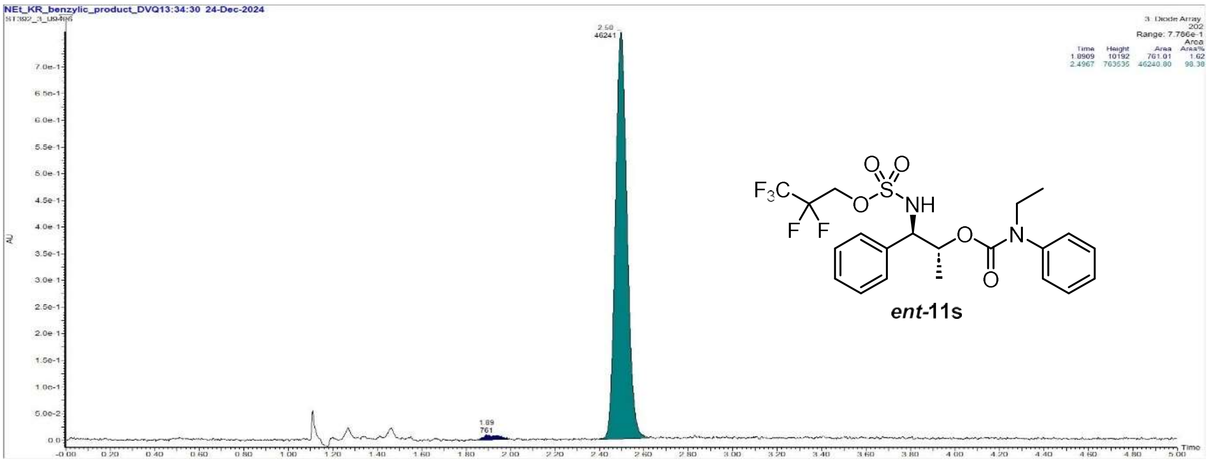

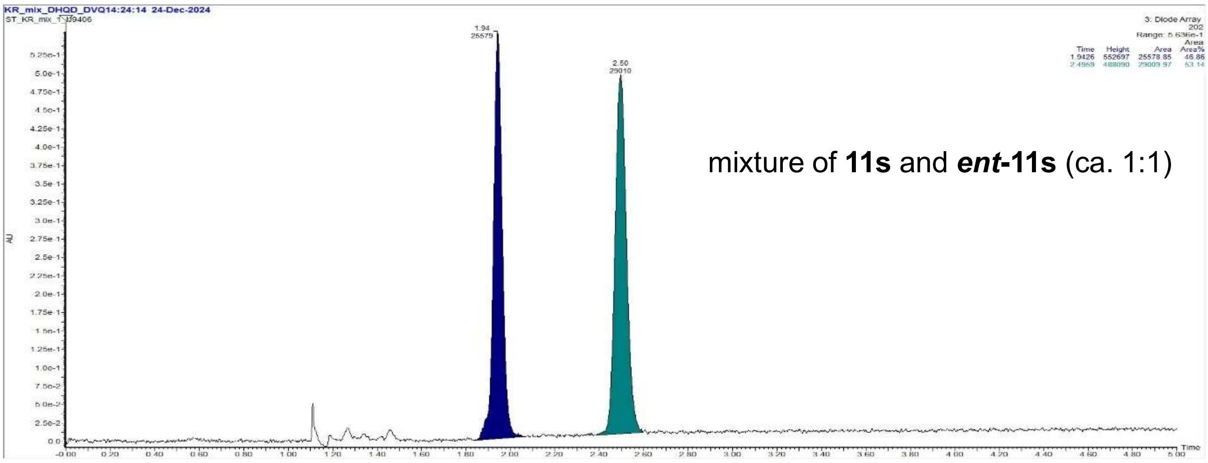

2,2,3,3,3-pentafluoropropyl (*S*)-(2-hydroxy-1-phenylethyl)sulfamate (**12**)

Chiral SFC Analysis CHIRALPAK IK (CO_2:MeOH, 94:6, 2.50 mL min^{-1}, 40 ˚C, 204 nm) indicated 96% *ee*, t_R = 2.5 (minor), 3.0 (major) minutes.

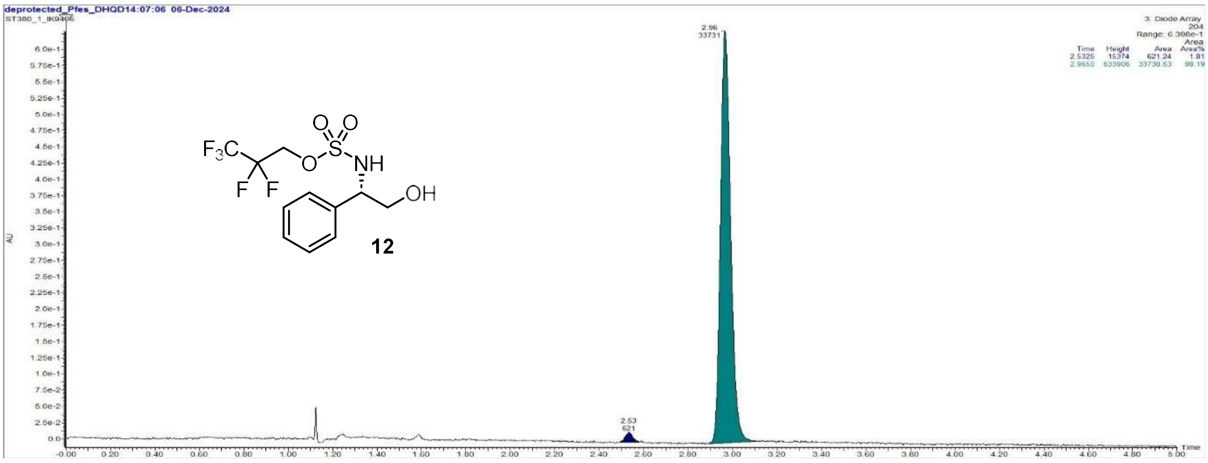

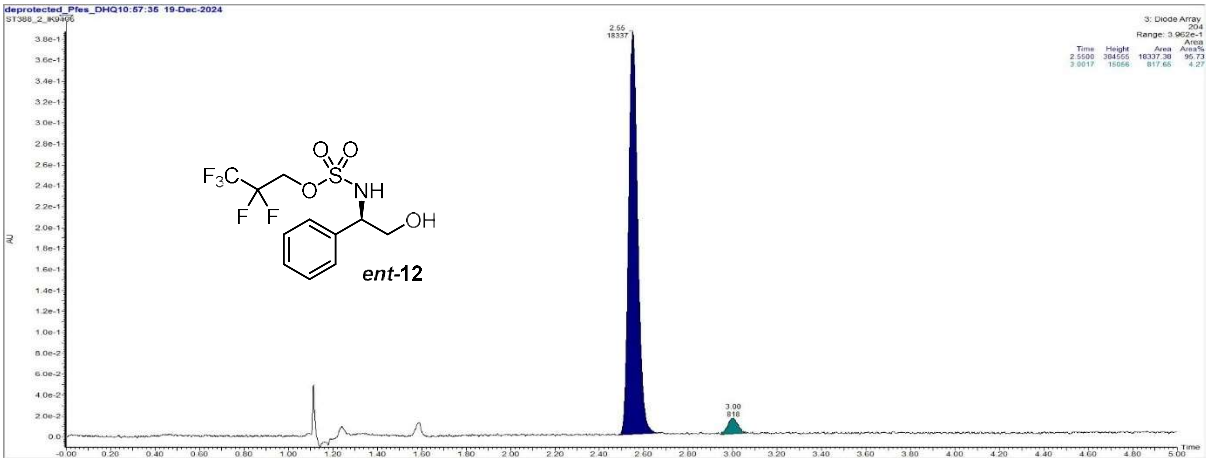

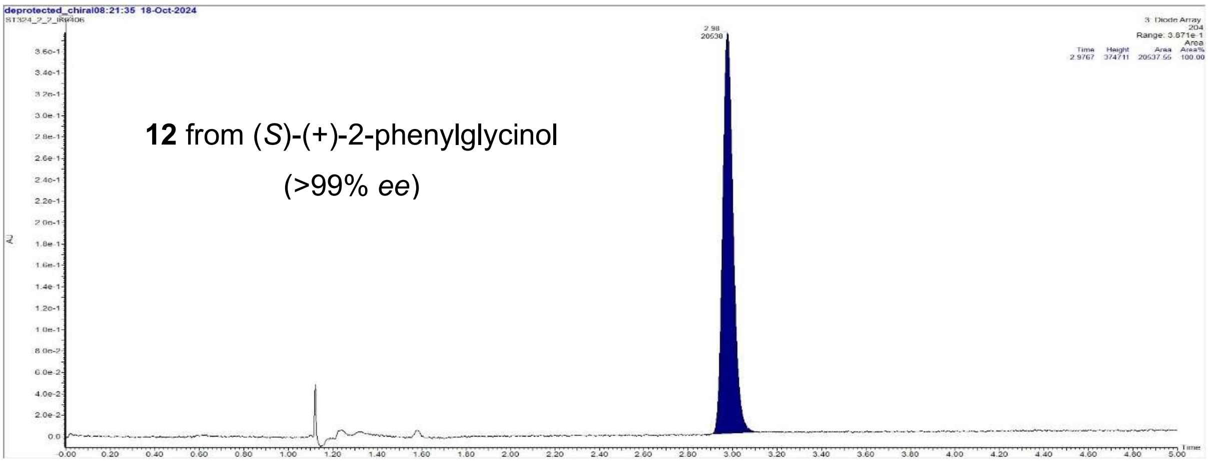

(*S*)-2-((tert-butoxycarbonyl)amino)-2-phenylethyl ethyl(phenyl)carbamate (**14**)

Chiral SFC Analysis CHIRALPAK IJ (CO_2:MeOH, 94:6, 2.50 mL min^{-1}, 40 ˚C, 202 nm) indicated 96% *ee*, t_R = 3.0 (major), 4.7 (minor) minutes.

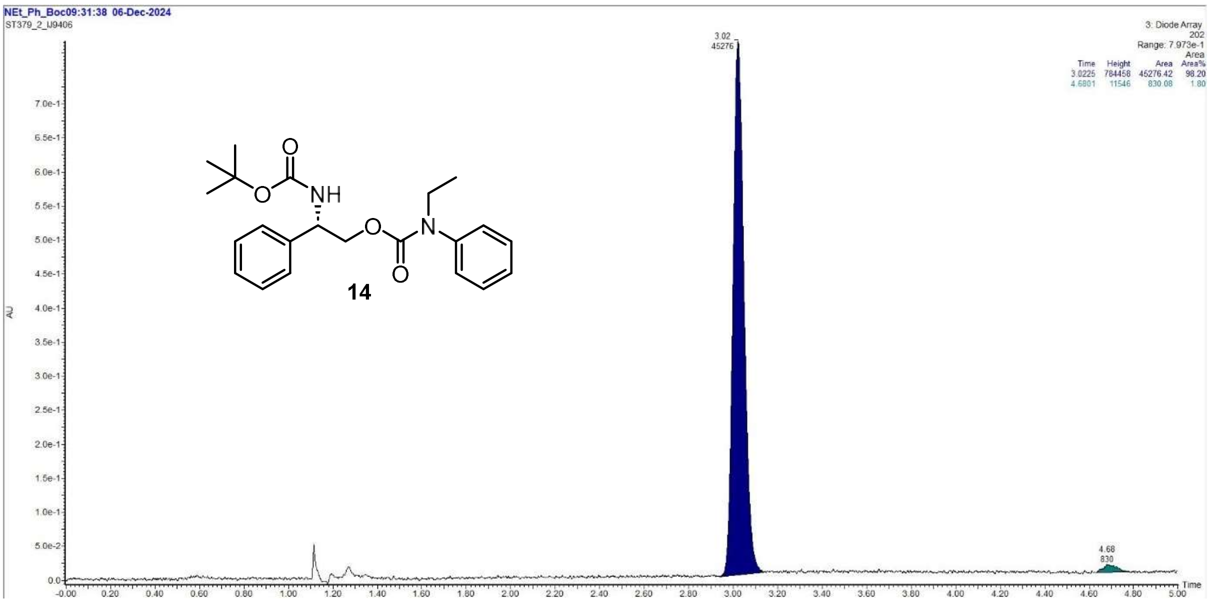

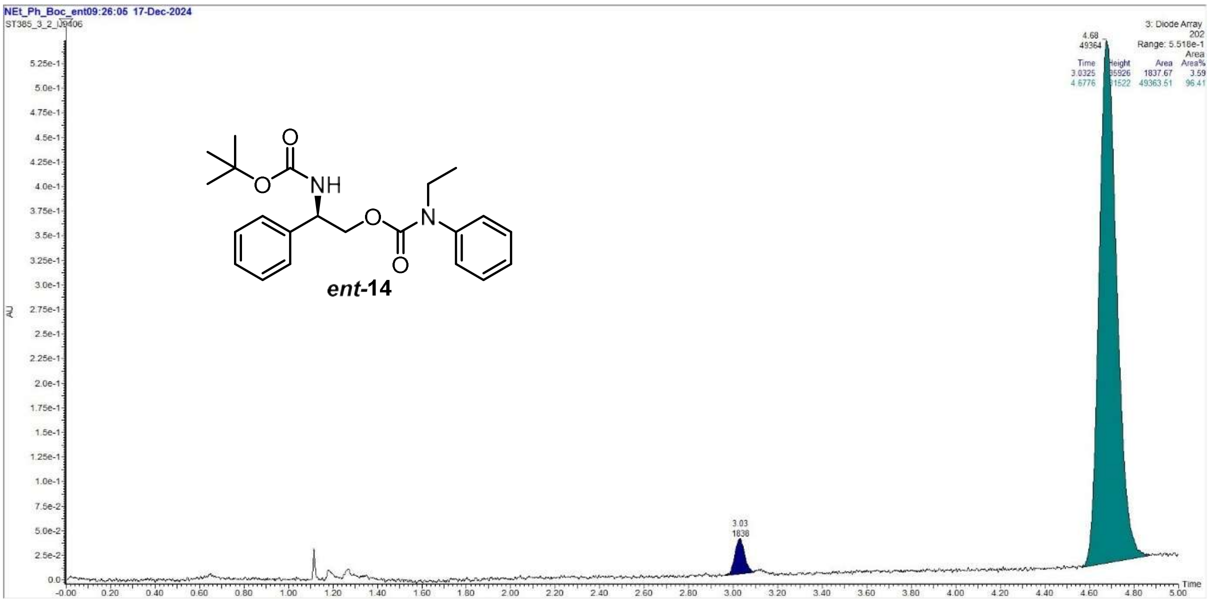

2,2,3,3,3-pentafluoropropyl ((1*S*,2*S*)-2-hydroxy-2,3-dihydro-1*H*-inden-1-yl)sulfamate (**S9**)

Chiral SFC Analysis CHIRALPAK IC (CO_2:MeOH, 95:5, 2.50 mL min^{-1}, 40 ˚C, 204 nm) indicated 71% *ee*, t_R = 2.8 (major), 3.1 (minor) minutes.

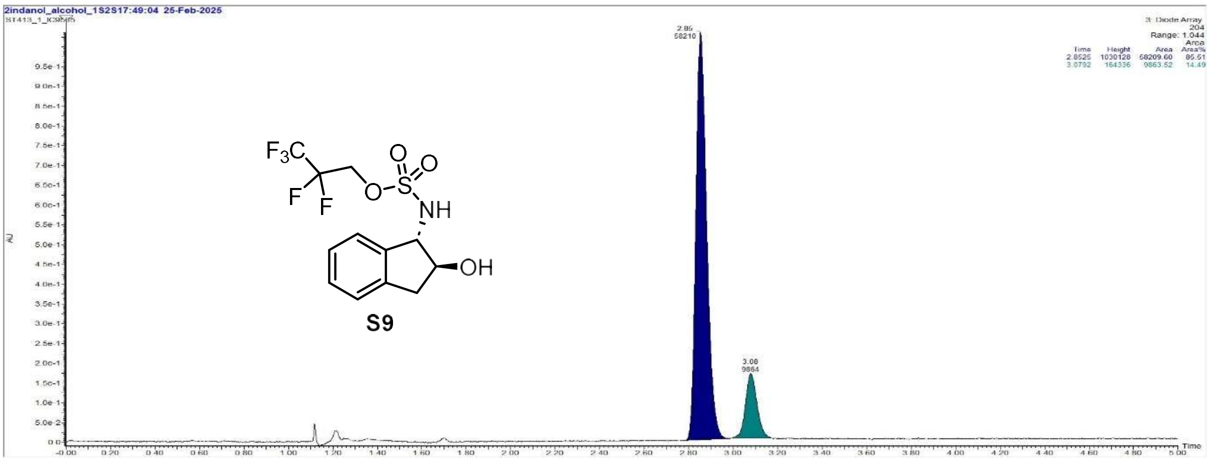

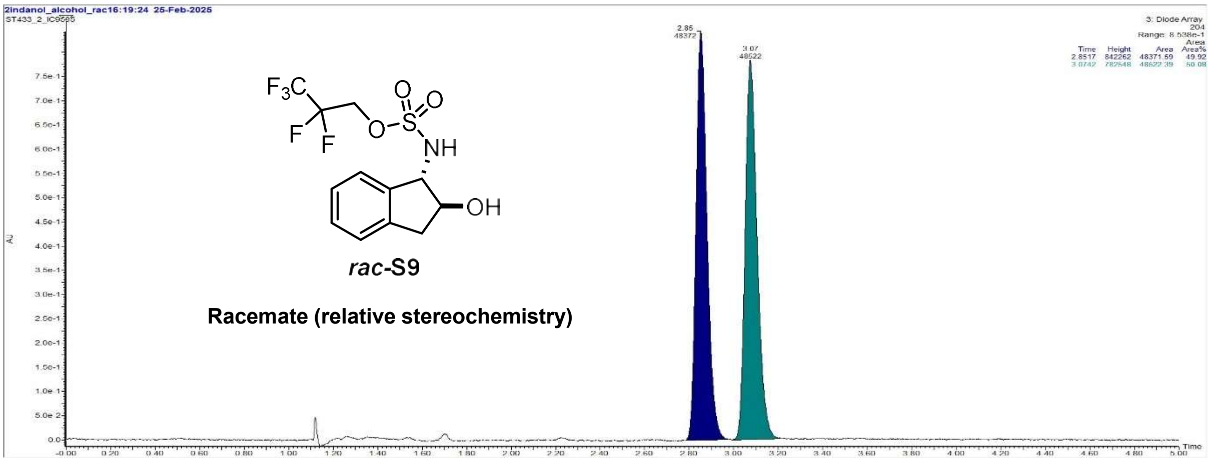

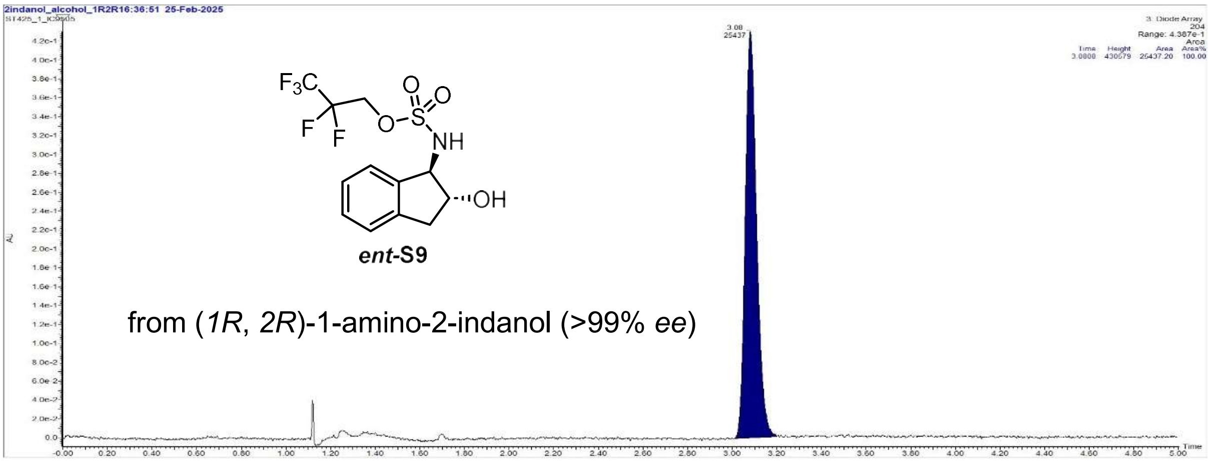

19 References

[1] G. B. Kauffman, L. Y. Fang, *Inorganic Syntheses, Vol. 22*, John Wiley & Sons, Inc, Toronto, Canada, **1983**.

[2] H. E. Gottlieb, V. Kotlyar, A. Nudelman, *J. Org. Chem.* **1997**, *62*, 7512-7515.

[3] M. Piotto, M. Bourdonneau, K. Elbayed, J.-M. Wieruszeski, G. Lippens, *Mag. Reson. Chem.* **2006**, *44*, 943-947.

[4] A. Fanourakis, B. D. Williams, K. J. Paterson, R. J. Phipps, *J. Am. Chem. Soc.* **2021**, *143*, 10070-10076.

[5] G. R. Genov, J. L. Douthwaite, A. S. K. Lahdenperä, D. C. Gibson, R. J. Phipps, *Science* **2020**, *367*, 1246-1251.

[6] A. Fanourakis, N. J. Hodson, A. R. Lit, R. J. Phipps, *J. Am. Chem. Soc.* **2023**, *145*, 7516-7527.

[7] N. J. Hodson, S. Takano, A. Fanourakis, R. J. Phipps, *J. Am. Chem. Soc.* **2024**, *146*, 22629-22641.

[8] S. E. Luderer, B. Masoudi, A. Sarkar, C. Grant, A. Jaganathan, J. E. Jackson, B. Borhan, *J. Org. Chem.* **2024**, *89*, 11921-11929.

[9] H. Konishi, T. Y. Lam, J. P. Malerich, V. H. Rawal, *Org. Lett.* **2010**, *12*, 2028-2031.

[10] M. S. Manna, S. Mukherjee, *J. Am. Chem. Soc.* **2015**, *137*, 130-133.

[11] Y. Uozumi, M. Kawatsura, T. Hayashi, *Org. Synth.* **2002**, *78*, 1.

[12] S. Shirakawa, A. Kasai, T. Tokuda, K. Maruoka, *Chem. Sci.* **2013**, *4*, 2248-2252.

[13] N. van den Heuvel, S. M. Mason, B. Q. Mercado, S. J. Miller, *J. Am. Chem. Soc.* **2023**, *145*, 12377-12385.

[14] M. Dell'Acqua, V. Pirovano, G. Confalonieri, A. Arcadi, E. Rossi, G. Abbiati, *Org. Biomol. Chem.* **2014**, *12*, 8019-8030.

[15] M. Wang, S. Jiang, X.-X. Lu, K. Zhang, Z.-Y. Yuan, R.-L. Xu, B.-T. Zhao, A.-X. Wu, *Org. Biomol. Chem.* **2023**, *21*, 5949-5952.

[16] J. P. Costello, E. M. Ferreira, *Org. Lett.* **2019**, *21*, 9934-9939.

[17] T. J. M. Byrne, M. E. Mylrea, J. D. Cuthbertson, *Org. Lett.* **2023**, *25*, 2361-2365.

[18] R. S. Kim, L. V. Dinh-Nguyen, K. W. Shimkin, D. A. Watson, *Org. Lett.* **2020**, *22*, 8106-8110.

[19] B. R. Ambler, S. Peddi, R. A. Altman, *Org. Lett.* **2015**, *17*, 2506-2509.

[20] Q. Zhang, Y. Duan, H. Guo, H. Yang, J. Zhai, T. Li, Z. Wang, X. Lu, Y. Wang, Y. Yin, *Chem. Asian J.* **2021**, *16*, 1832-1838.

[21] C. Wang, D. Abegg, D. G. Hoch, A. Adibekian, *Angew. Chem. Int. Ed.* **2016**, *55*, 2911-2915.

[22] A. Wang, M. Lu, X. Xie, Y. Liu, *Org. Lett.* **2022**, *24*, 2944-2949.

[23] Y.-R. Zhu, J.-H. Lin, J.-C. Xiao, *Synlett* **2021**, *33*, 259-263.

[24] F. Ke, C. Yu, X. Li, H. Sheng, Q. Song, *Org. Lett.* **2023**, *25*, 2733-2738.

[25] M. Mendel, L. Gnägi, U. Dabranskaya, F. Schoenebeck, *Angew. Chem. Int. Ed.* **2023**, *62*, e202211167.

[26] F. Seifert, D. Drikermann, J. Steinmetzer, Y. Zi, S. Kupfer, I. Vilotijevic, *Org. Biomol. Chem.* **2021**, *19*, 6092-6097.

[27] Z. Liu, B. Liu, X.-F. Zhao, Y.-B. Wu, X. Bi, *Eur. J. Org. Chem.* **2017**, *2017*, 928-932.

[28] C. Chen, Y. Huang, Z. Zhang, X.-Q. Dong, X. Zhang, *Chem. Commun.* **2017**, *53*, 4612-4615.

[29] X. Yi, X. Hu, *Angew. Chem. Int. Ed.* **2019**, *58*, 4700-4704.

[30] D. J. Vyas, M. Oestreich, *Chem. Commun.* **2010**, *46*, 568-570.

[31] H.-K. Kim, A. Lee, *Org. Biomol. Chem.* **2016**, *14*, 7345-7353.

[32] A. M. Fournier, C. J. Nichols, M. A. Vincent, I. H. Hillier, J. Clayden, *Chem. Eur. J.* **2012**, *18*, 16478-16490.

[33] J. Qin, Y. Li, Y. Hu, Z. Huang, W. Miao, L. Chu, *J. Am. Chem. Soc.* **2024**, *146*, 27583-27593.

[34] X. Zhu, Y. Qi, Y. Yang, D. Guo, Z. Huang, L. Zhang, Y. Wei, S. Zhou, S. Wang, *Inorg. Chem.* **2022**, *61*, 3202-3211.

[35] U. S. Ismailani, M. Munch, B. A. Mair, B. H. Rotstein, *Chem. Commun.* **2021**, *57*, 5266-5269.

[36] W. Li, M. Lv, X. Luo, Z. Wang, Q. Song, X. Yu, *RSC Adv.* **2024**, *14*, 20656-20659.

[37] G. Barbe, A. B. Charette, *J. Am. Chem. Soc.* **2008**, *130*, 18-19.

[38] K. J. Paterson, A. Dahiya, B. D. Williams, R. J. Phipps, *Angew. Chem. Int. Ed.* **2024**, *63*, e202317489.

[39] Y. Matsushima, M. Orita, *Tetrahedron Lett.* **2021**, *73*, 153095.

20 NMR Spectra

NMR Spectra for Chiral Cation Bromides:

^{1}H NMR (400 MHz, $CDCl_3$) for (1*S*,2*R*,4*S*,5*R*)-1-(3,5-dibromobenzyl)-5-ethyl-2-((*S*)-hydroxy(6-methoxyquinolin-4-yl)methyl)quinuclidin-1-ium bromide (**B5**•Br)

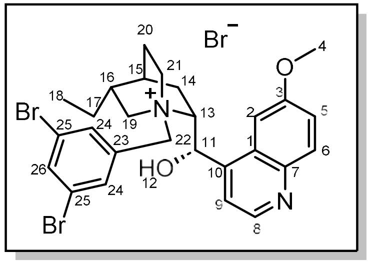

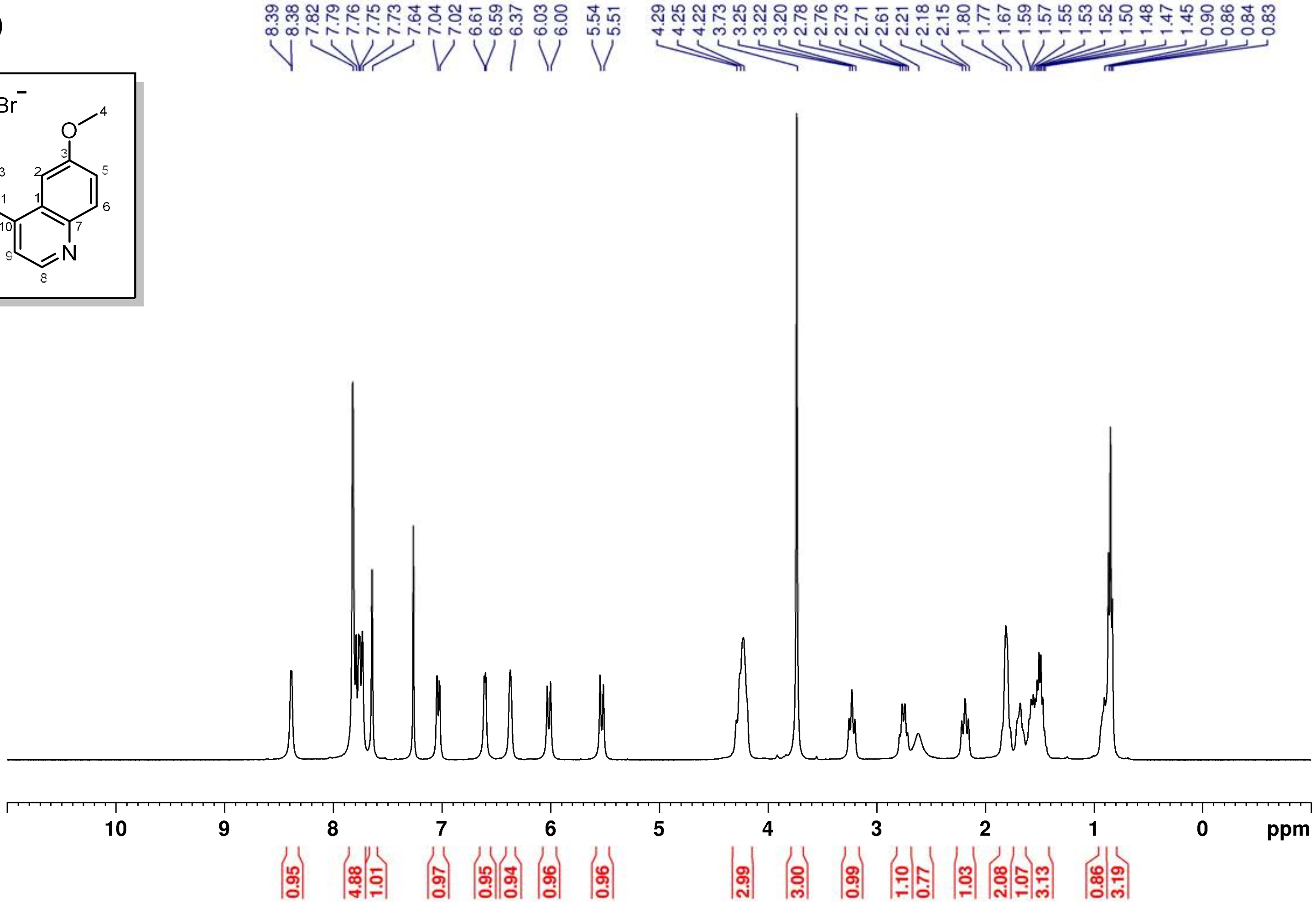

^{13}C NMR (101 MHz, CDCl$_3$) for (1*S*,2*R*,4*S*,5*R*)-1-(3,5-dibromobenzyl)-5-ethyl-2-((*S*)-hydroxy(6-methoxyquinolin-4-yl)methyl)quinuclidin-1-ium bromide (**B5**•Br)

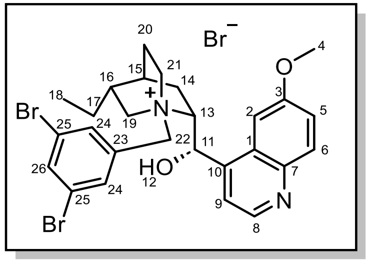

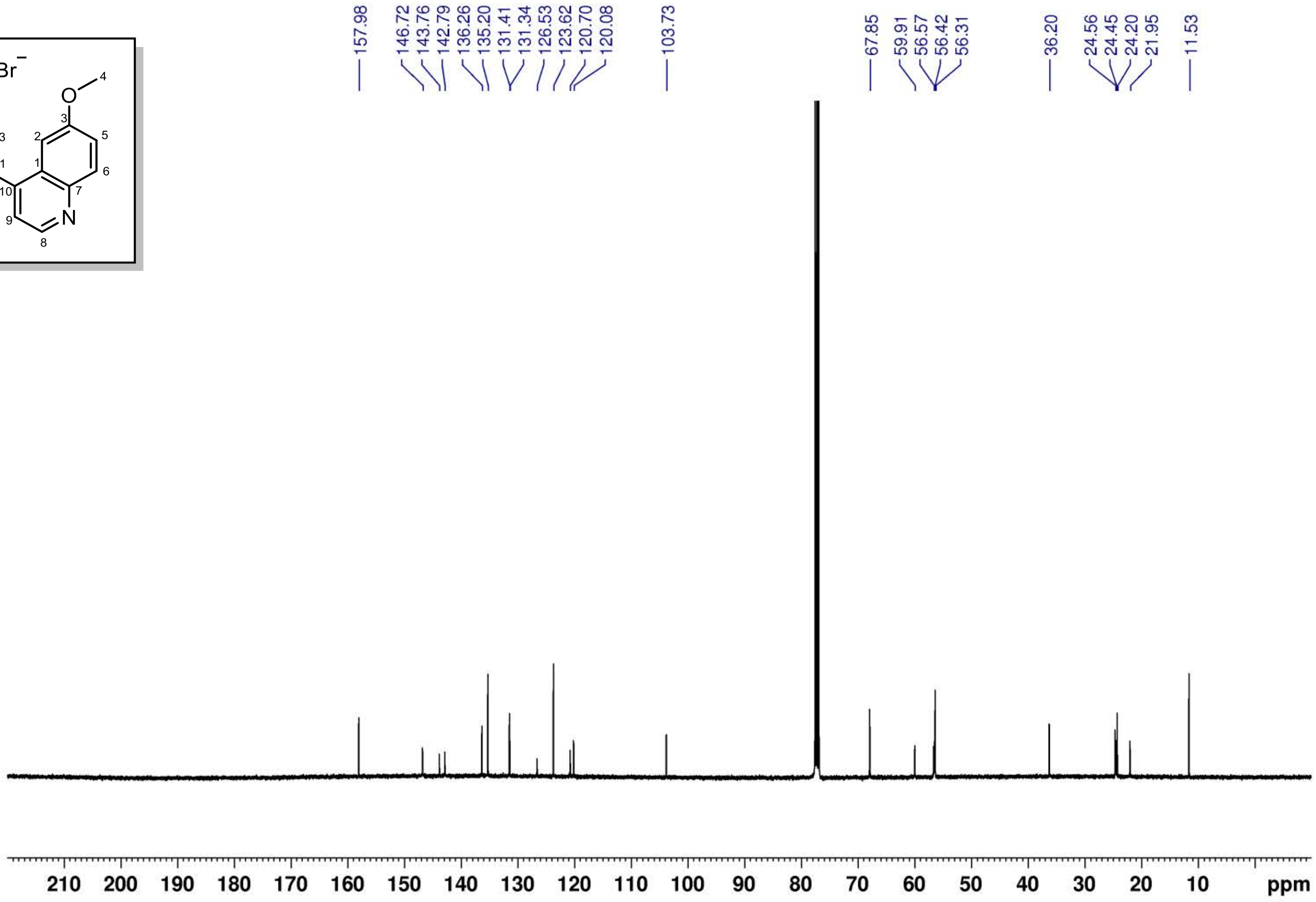

^{1}H NMR (400 MHz, $(CD_3)_2SO$) for dihydrocupreidine

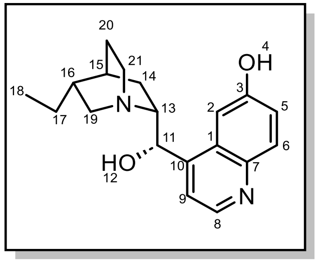

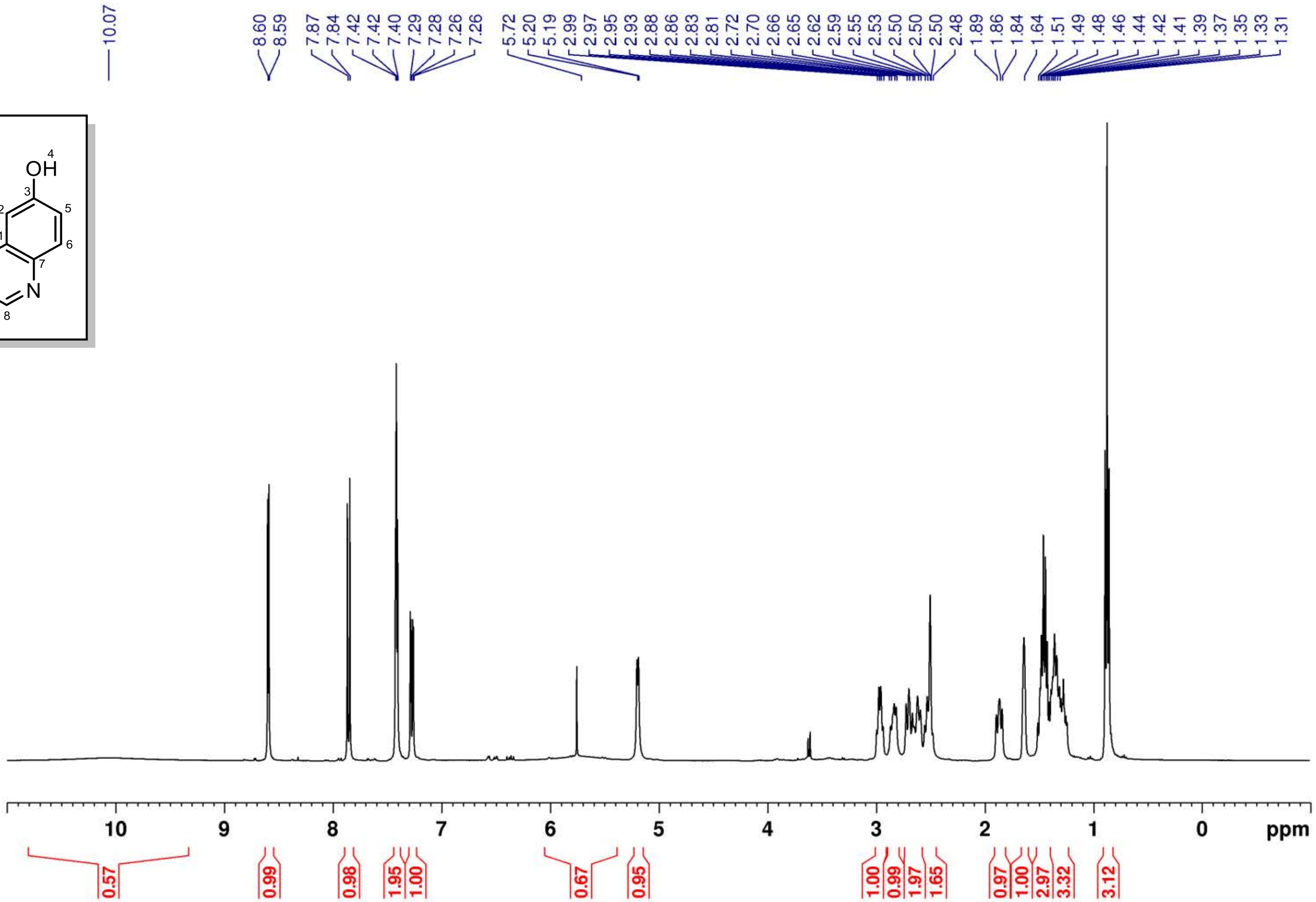

^{13}C NMR (101 MHz, $(CD_3)_2SO$) for dihydrocupreidine

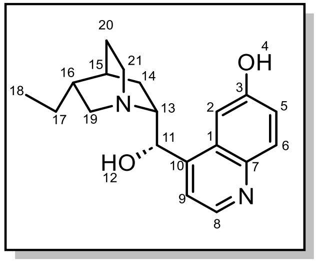

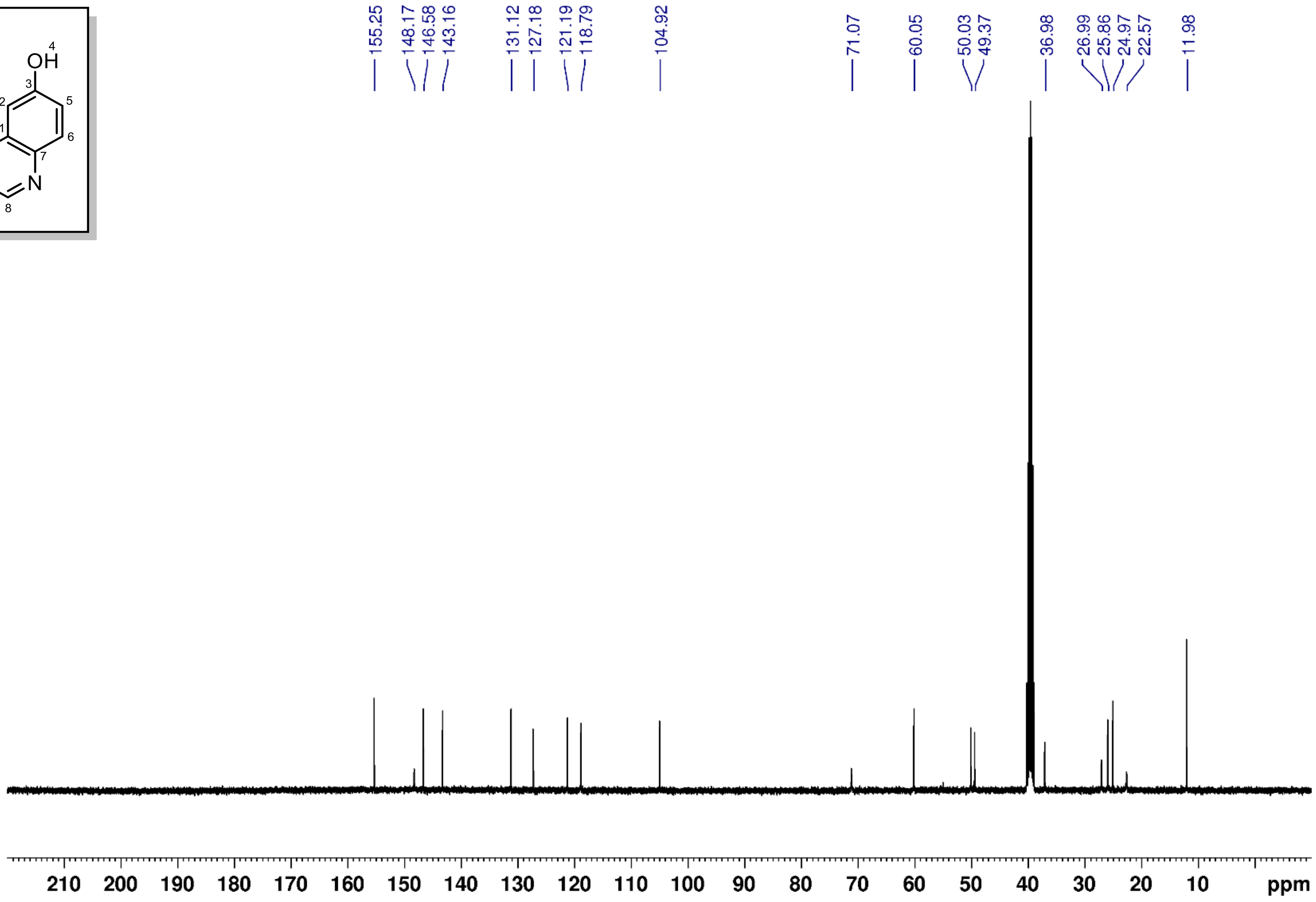

^{1}H NMR (700 MHz, $(CD_3)_2SO$) for (1*S*,2*R*,4*S*,5*R*)-1-(3,5-di-*tert*-butylbenzyl)-5-ethyl-2-((*S*)-hydroxy(6-hydroxyquinolin-4-yl)methyl)quinuclidin-1-ium bromide (**B11**•Br)

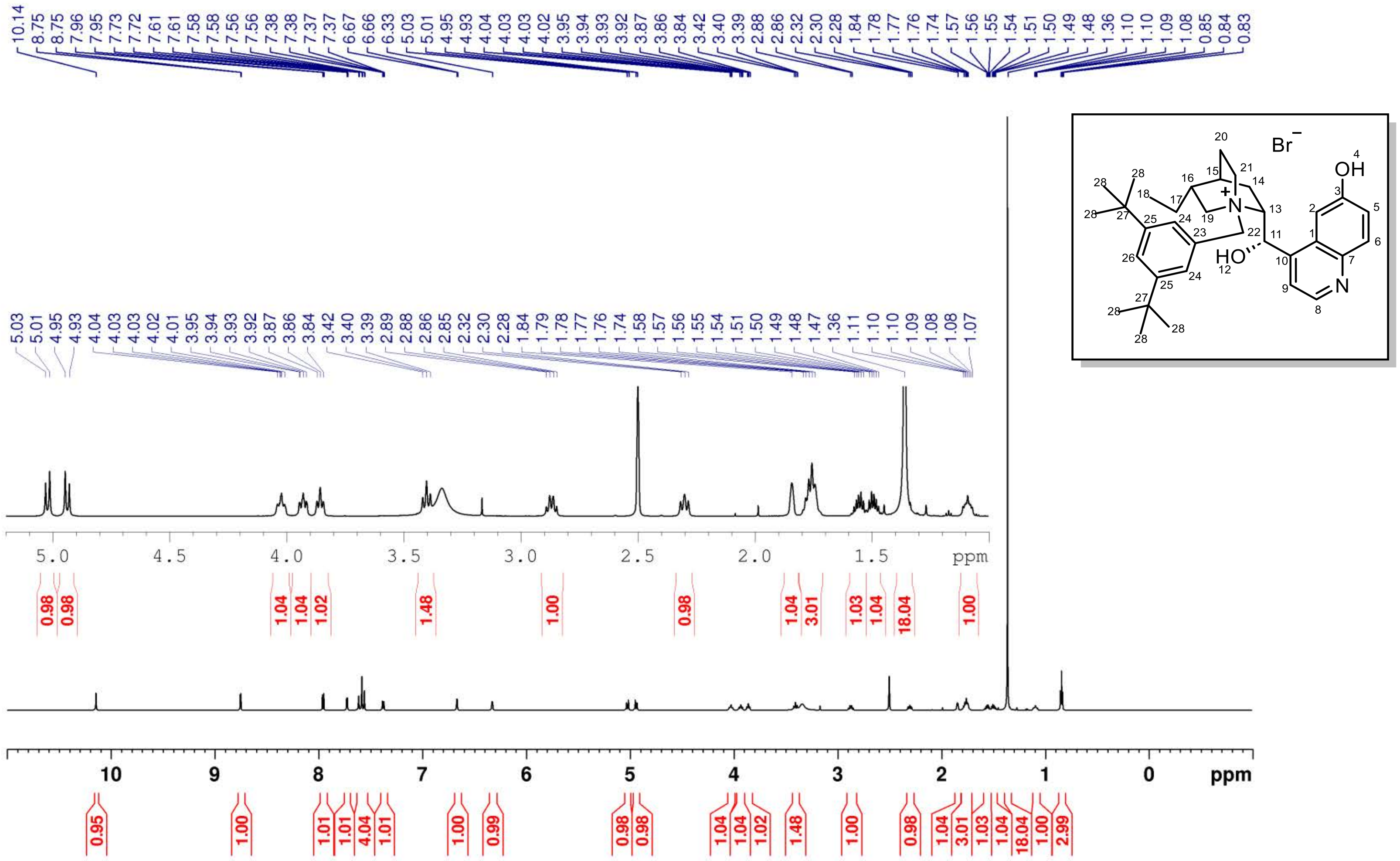

^{13}C NMR (101 MHz, $(CD_3)_2SO$) for (1*S*,2*R*,4*S*,5*R*)-1-(3,5-di-*tert*-butylbenzyl)-5-ethyl-2-((*S*)-hydroxy(6-hydroxyquinolin-4-yl)methyl)quinuclidin-1-ium bromide (**B11**•Br)

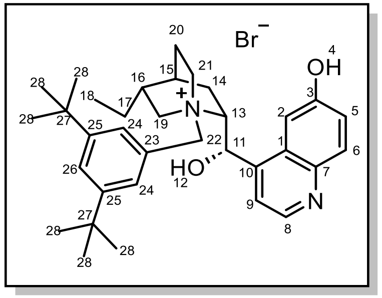

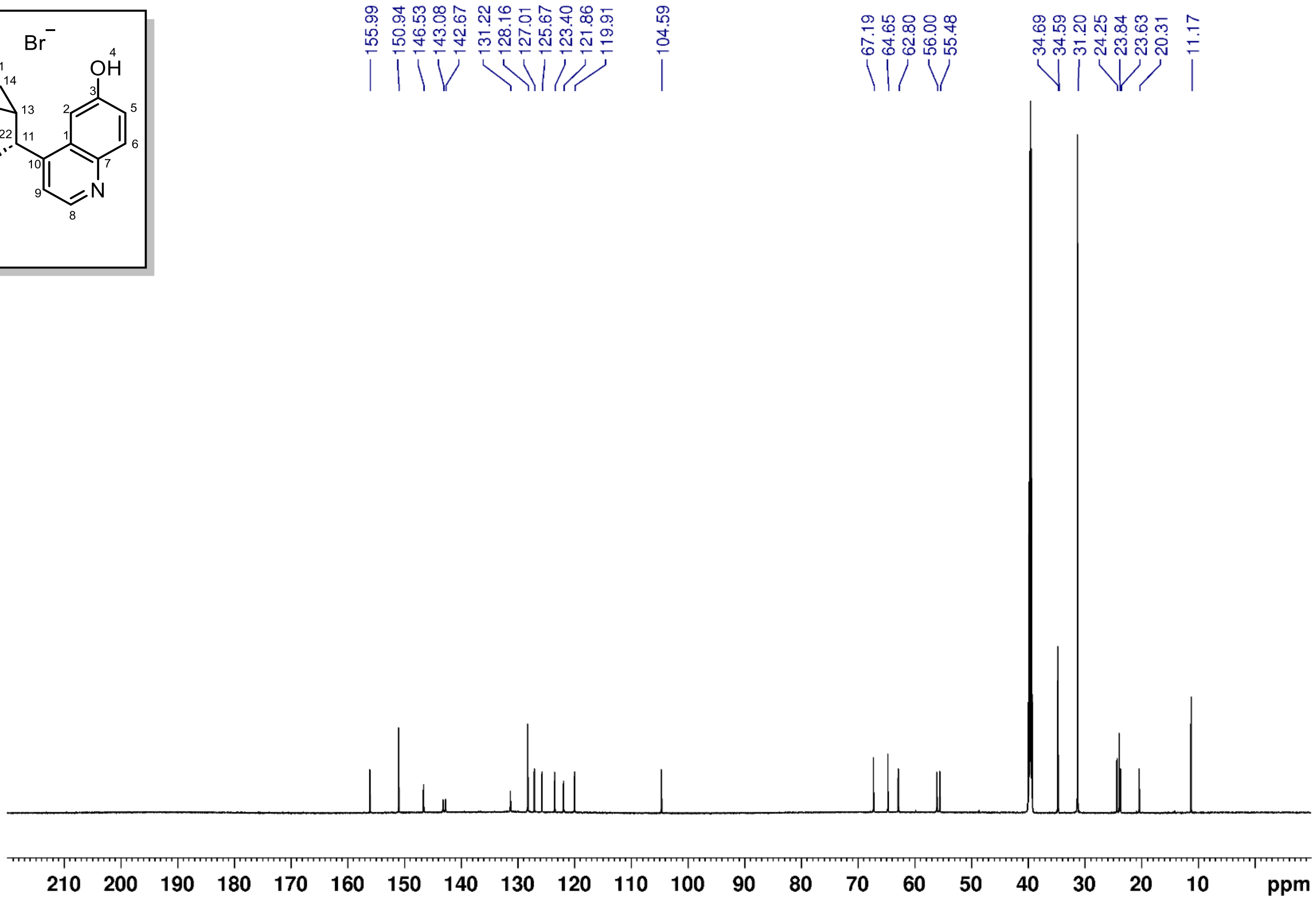

^{1}H NMR (400 MHz, $CDCl_3$) for (*S*)-(6-((*tert*-butyldimethylsilyl)oxy)quinolin-4-yl)((1*S*,2*R*,4*S*,5*R*)-5-ethylquinuclidin-2-yl)methanol

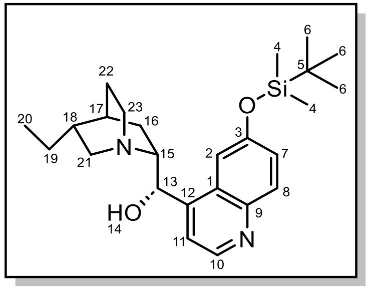

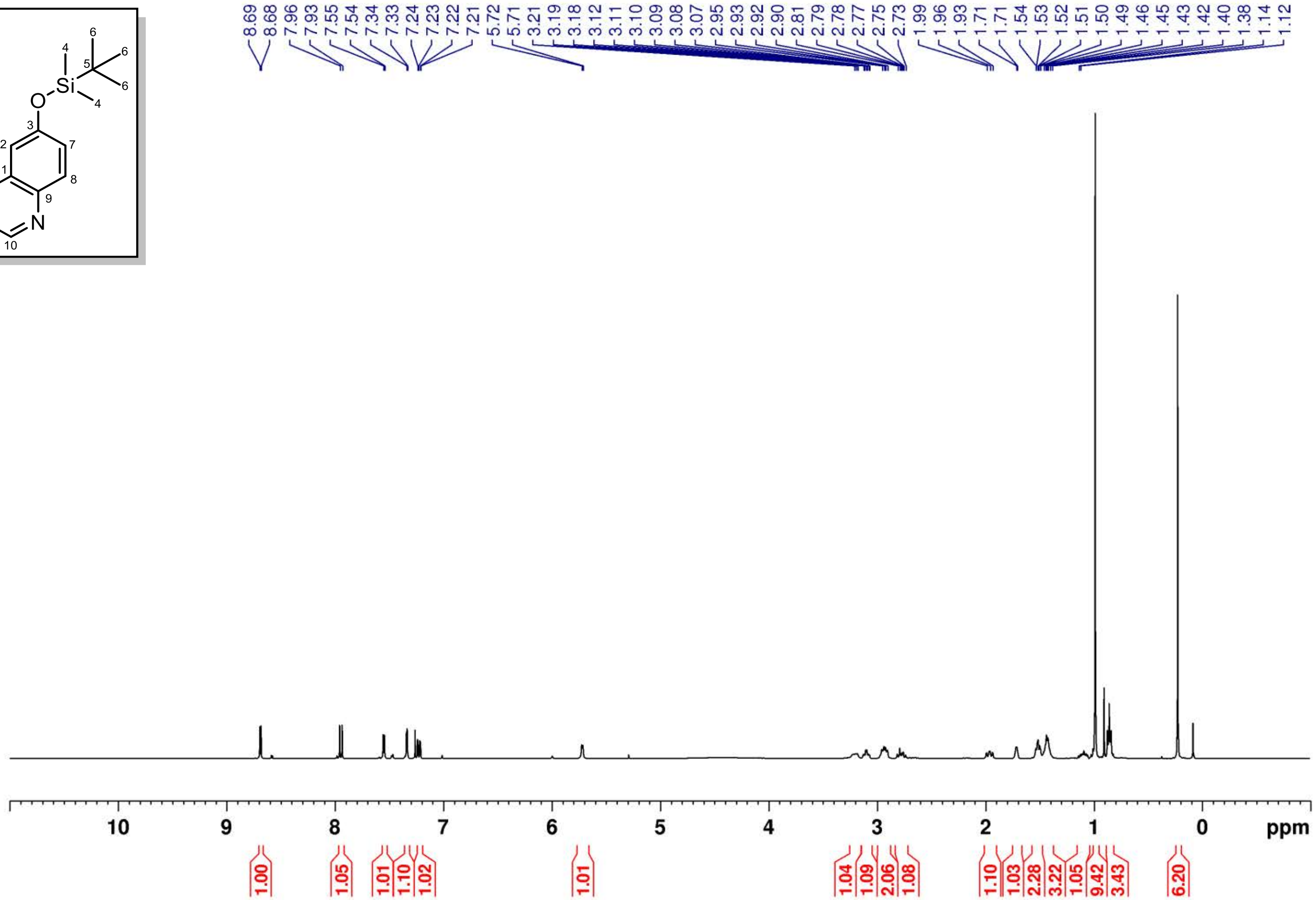

^{13}C NMR (101 MHz, CDCl$_3$) for (*S*)-(6-((*tert*-butyldimethylsilyl)oxy)quinolin-4-yl)((1*S*,2*R*,4*S*,5*R*)-5-ethylquinuclidin-2-yl)methanol

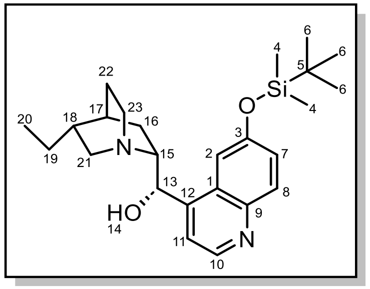

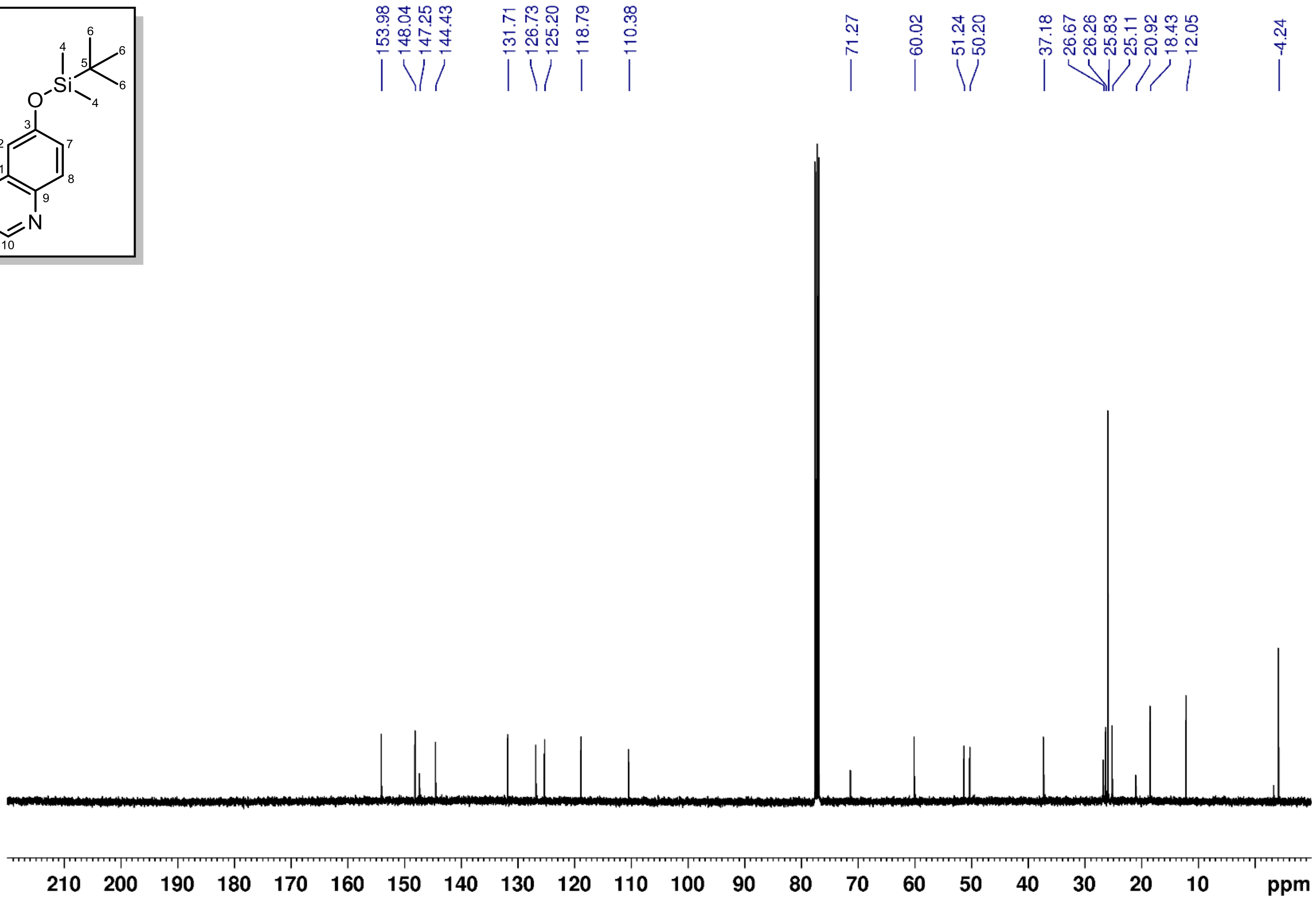

^{1}H NMR (700 MHz, CDCl$_3$) for (*S*)-((1*S*,2*R*,4*S*,5*R*)-5-ethylquinuclidin-2-yl)(6-hydroxyquinolin-4-yl)methyl benzoate

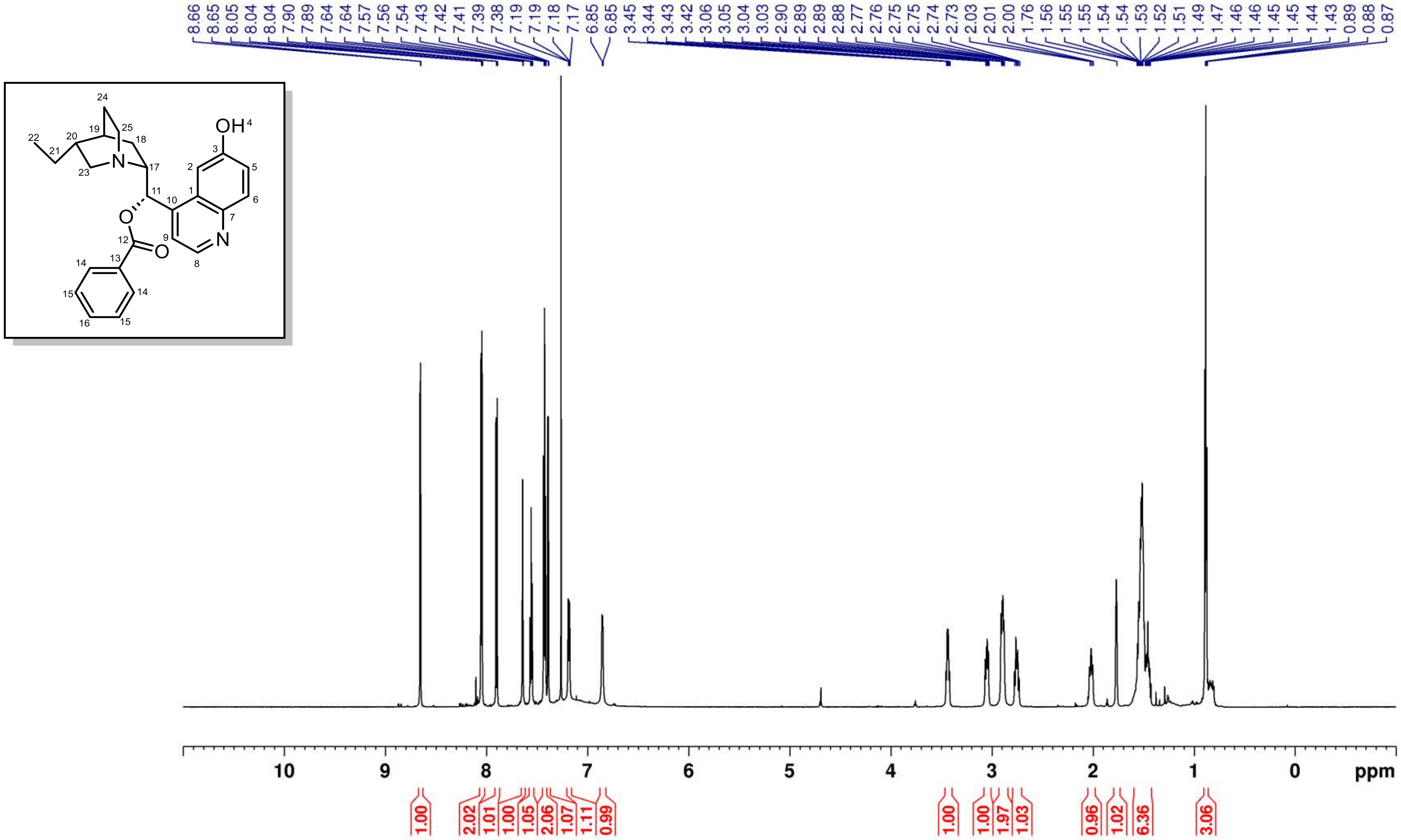

^{13}C NMR (176 MHz, CDCl$_3$) for (*S*)-((1*S*,2*R*,4*S*,5*R*)-5-ethylquinuclidin-2-yl)(6-hydroxyquinolin-4-yl)methyl benzoate

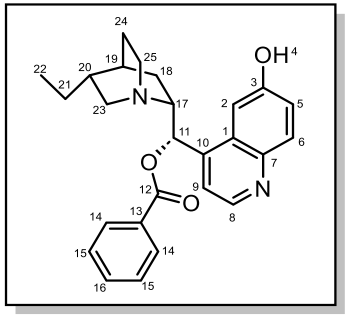

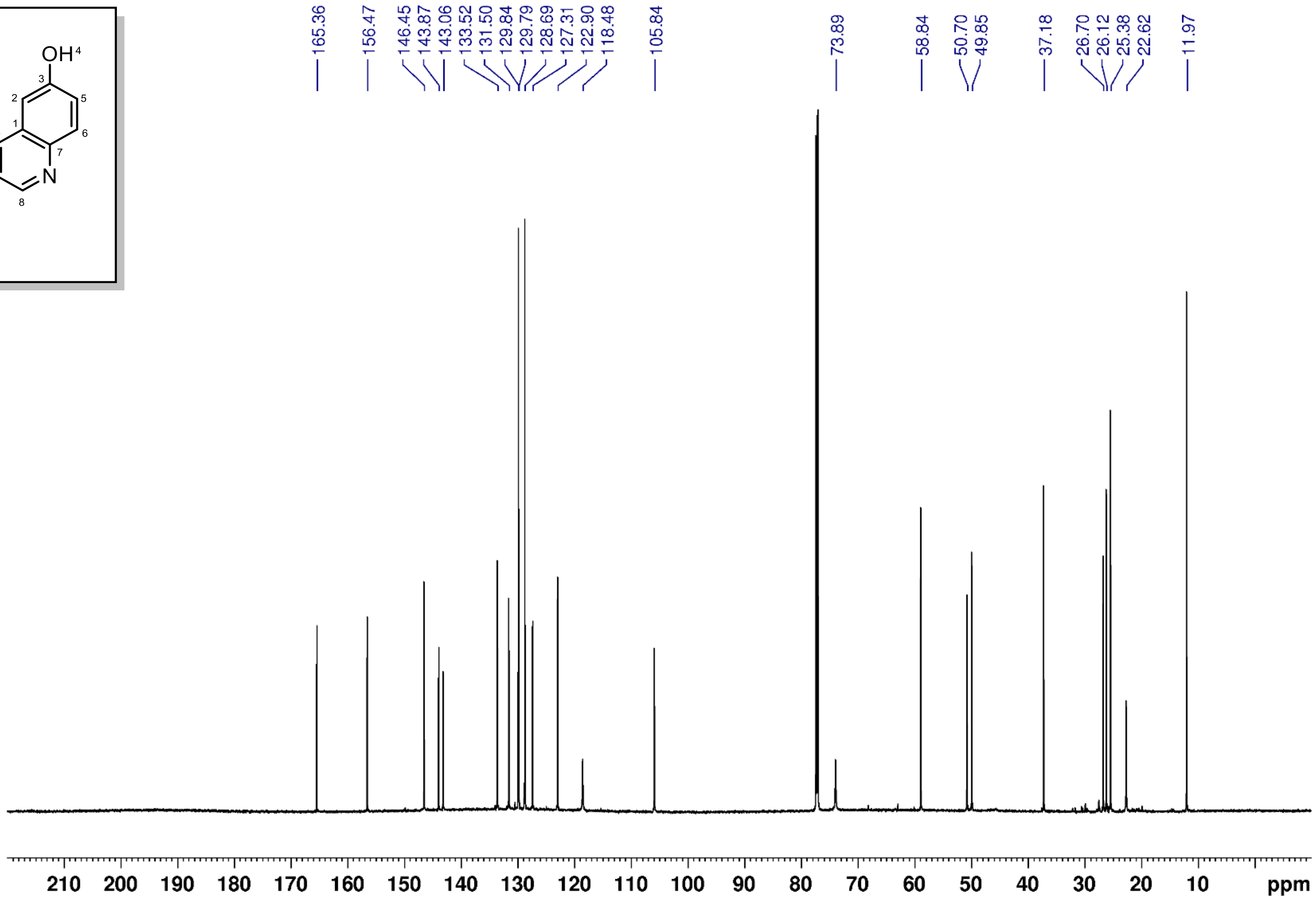

^{1}H NMR (700 MHz, $CDCl_3$) for (1*S*,2*R*,4*S*,5*R*)-2-((*S*)-(benzoyloxy)(6-hydroxyquinolin-4-yl)methyl)-1-(3,5-di-*tert*-butylbenzyl)-5-ethylquinuclidin-1-ium bromide (**B12•Br**)

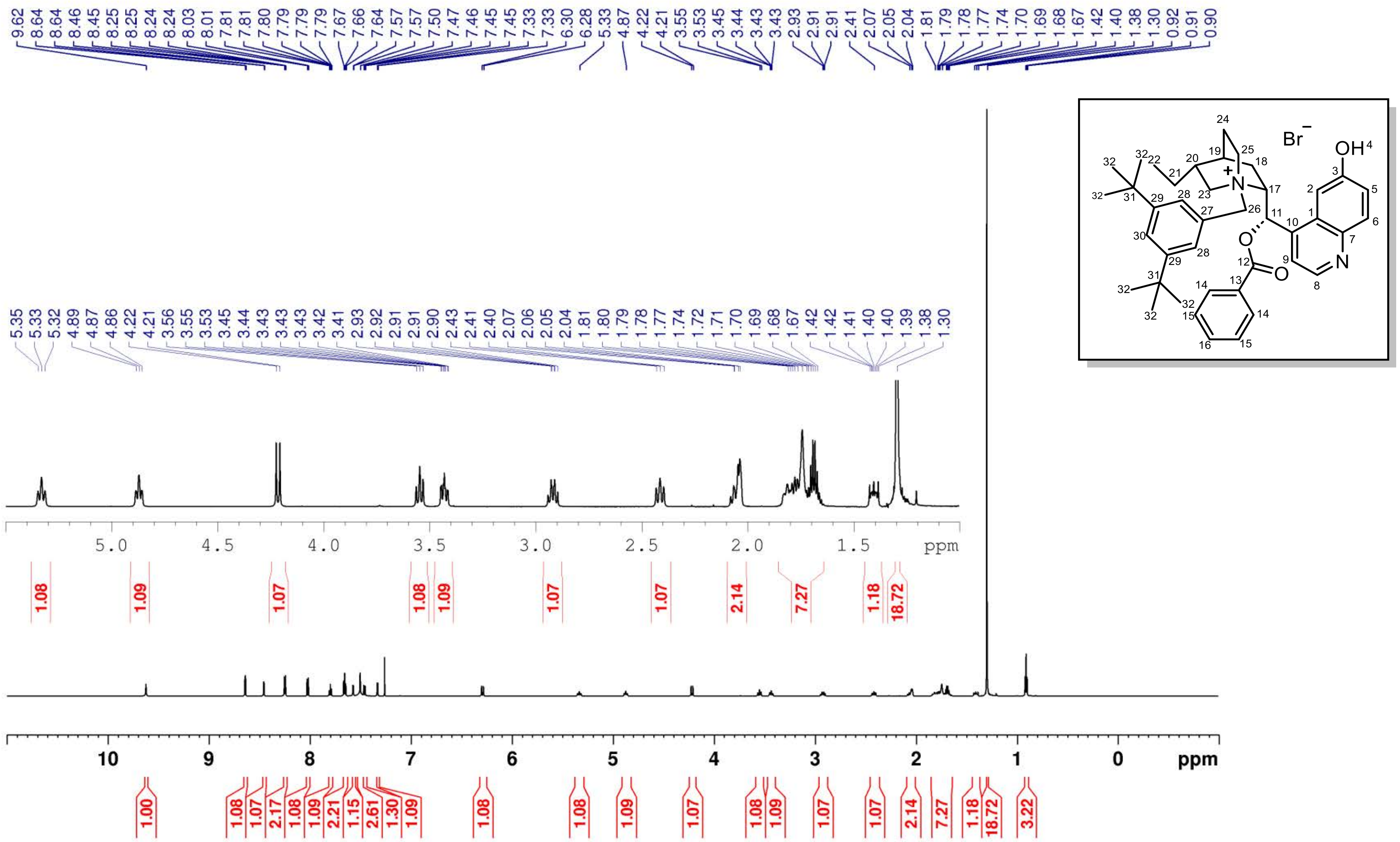

^{13}C NMR (176 MHz, CDCl$_3$) for (1*S*,2*R*,4*S*,5*R*)-2-((*S*)-(benzoyloxy)(6-hydroxyquinolin-4-yl)methyl)-1-(3,5-di-*tert*-butylbenzyl)-5-ethylquinuclidin-1-ium bromide (**B12•**Br)

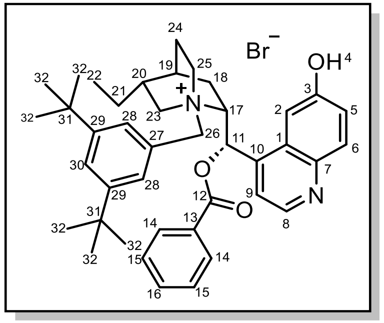

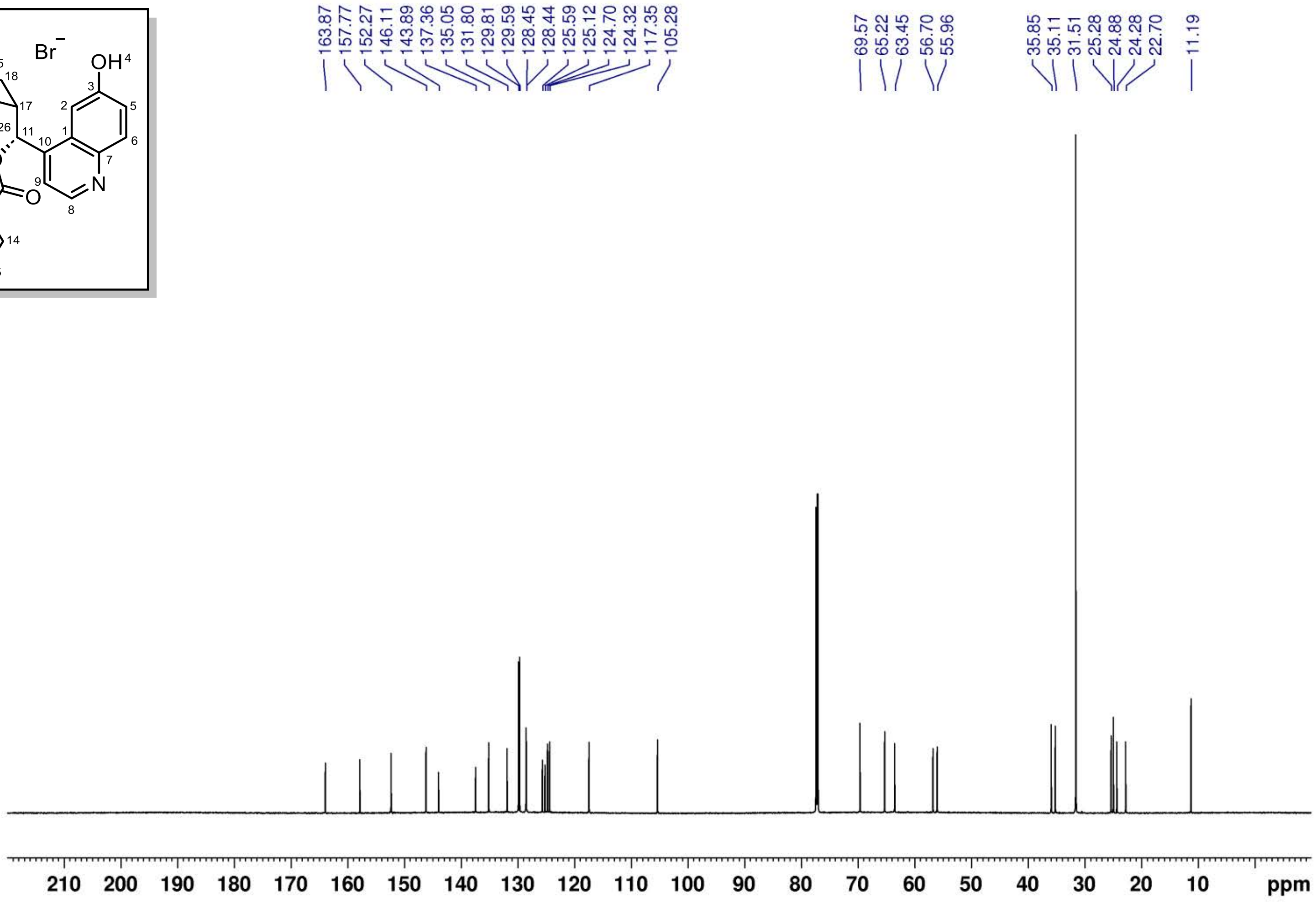

^{1}H NMR (400 MHz, CDCl$_3$) for (*R*)-((1*S*,2*S*,4*S*,5*R*)-5-ethylquinuclidin-2-yl)(6-methoxyquinolin-4-yl)methanamine

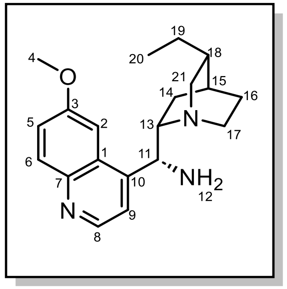

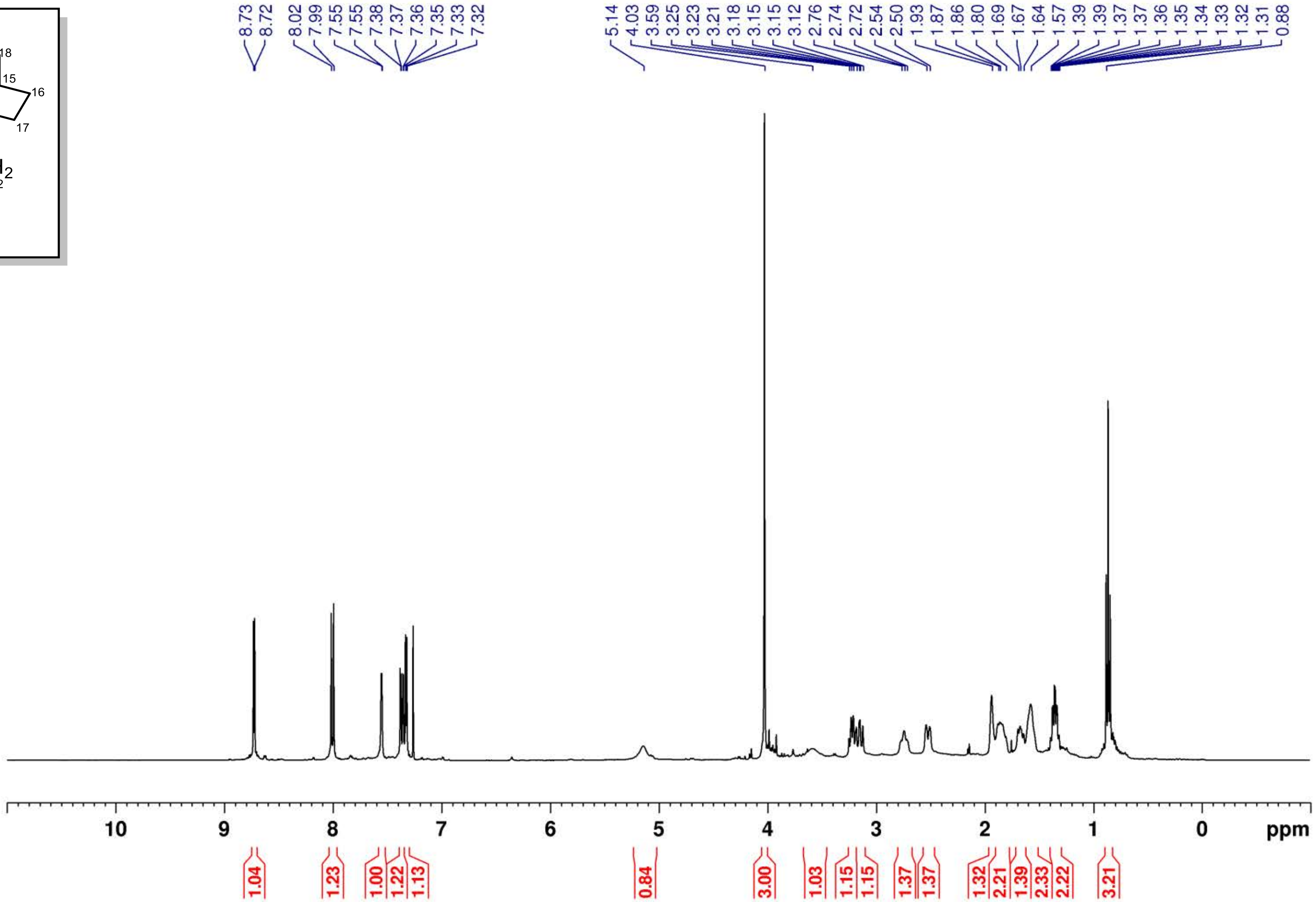

^{13}C NMR (176 MHz, $CDCl_3$) for (*R*)-((1*S*,2*S*,4*S*,5*R*)-5-ethylquinuclidin-2-yl)(6-methoxyquinolin-4-yl)methanamine

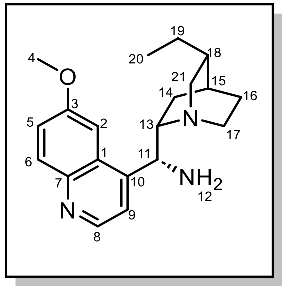

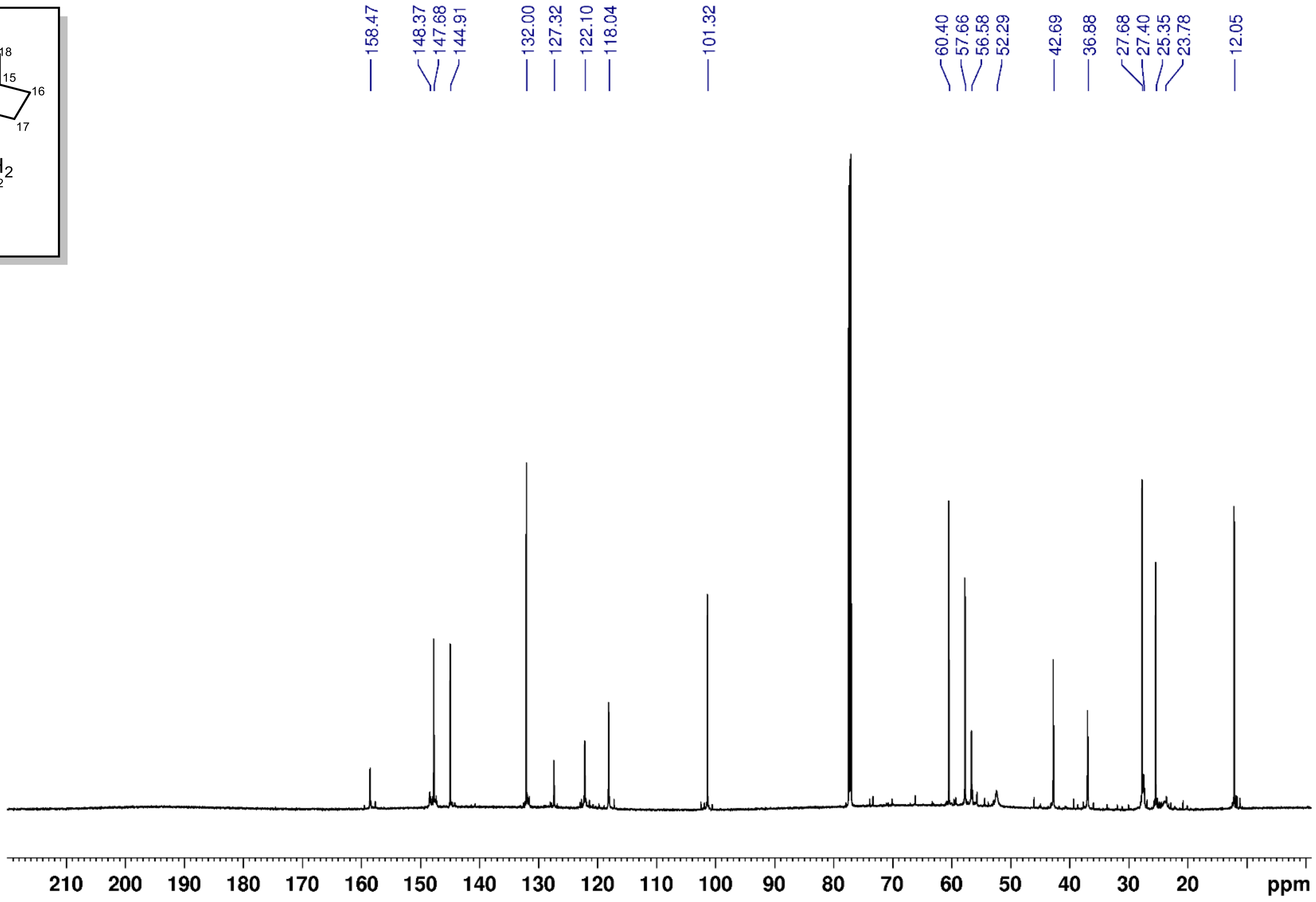

^{1}H NMR (700 MHz, $CDCl_3$) for 1-(3,5-bis(trifluoromethyl)phenyl)-3-((*R*)-((1*S*,2S,4S,5R)-5-ethylquinuclidin-2-yl)(6-methoxyquinolin-4-yl)methyl)urea

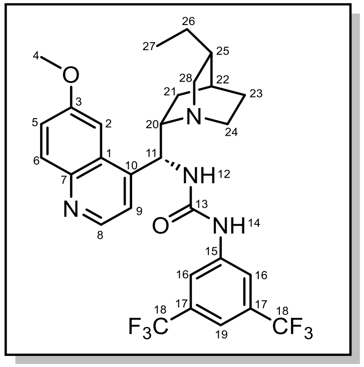

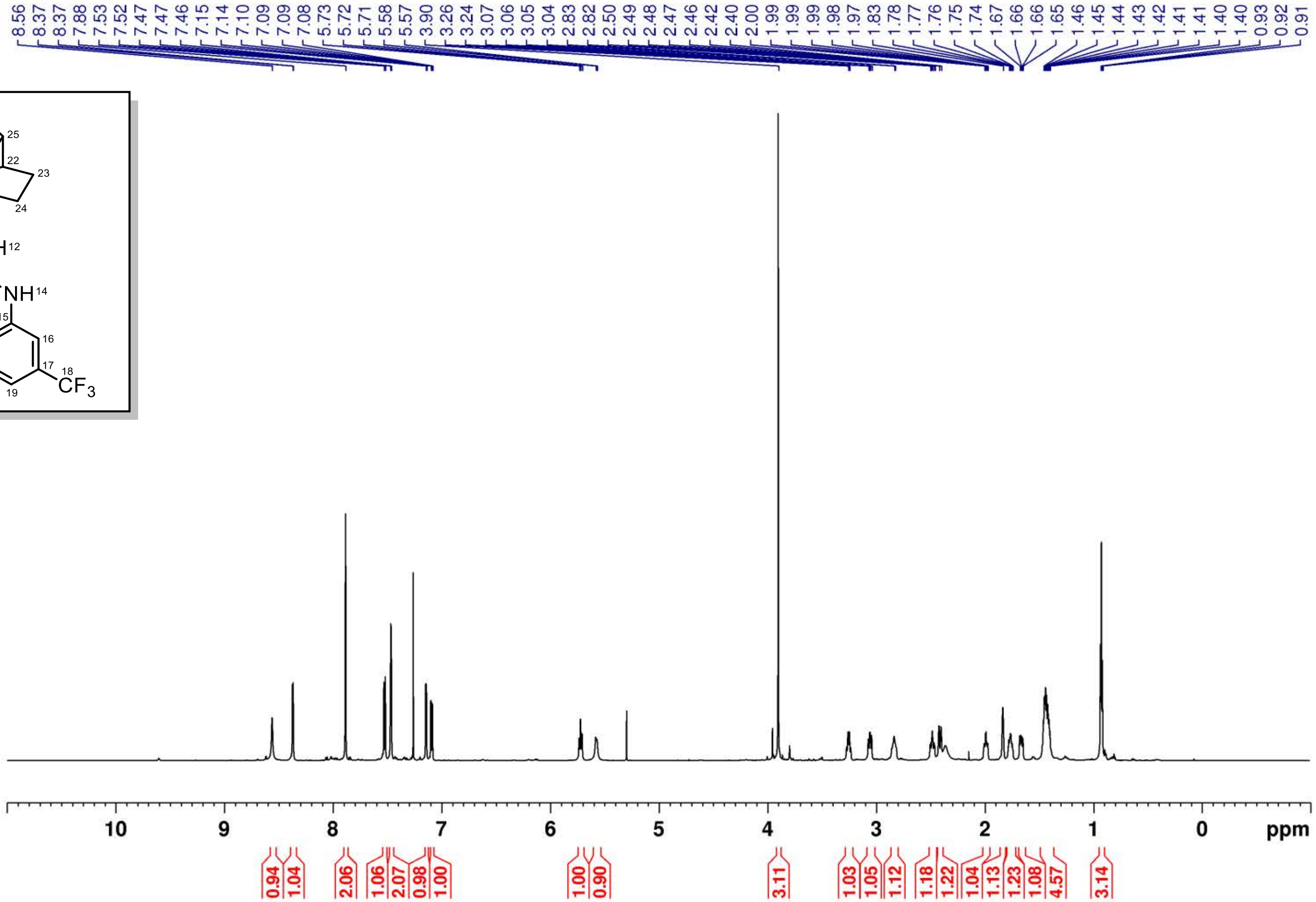

^{13}C NMR (176 MHz, CDCl$_3$) for 1-(3,5-bis(trifluoromethyl)phenyl)-3-((*R*)-((1*S*,2S,4S,5R)-5-ethylquinuclidin-2-yl)(6-methoxyquinolin-4-yl)methyl)urea

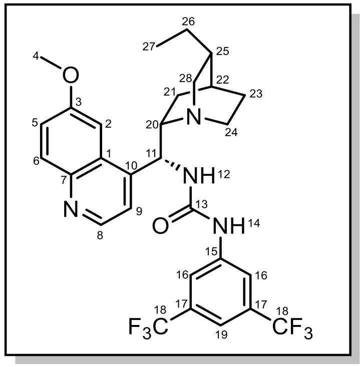

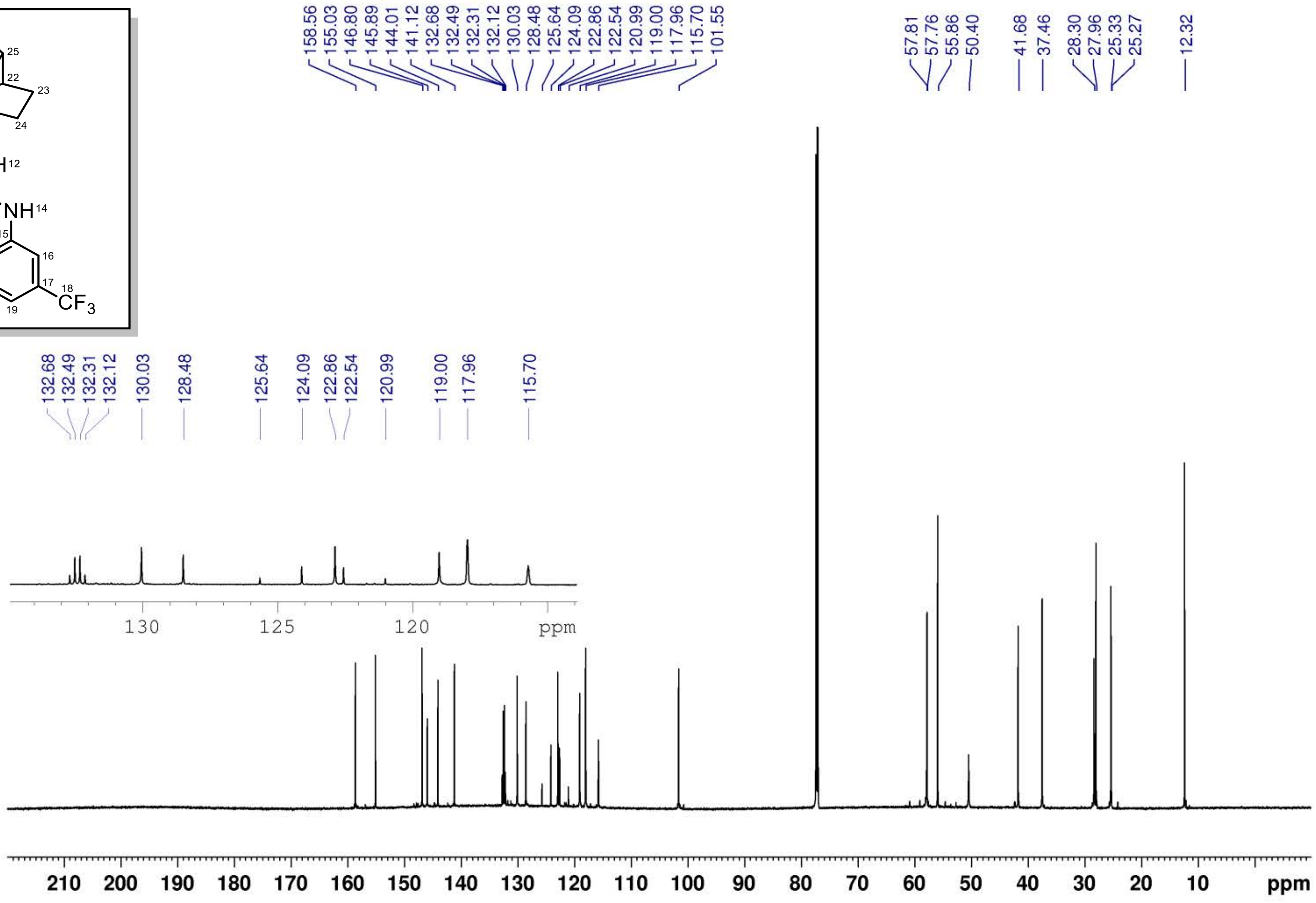

^{19}F NMR (376 MHz, $CDCl_3$) for 1-(3,5-bis(trifluoromethyl)phenyl)-3-((*R*)-((1*S*,2S,4S,5R)-5-ethylquinuclidin-2-yl)(6-methoxyquinolin-4-yl)methyl)urea

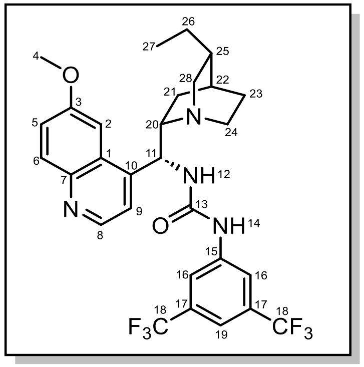

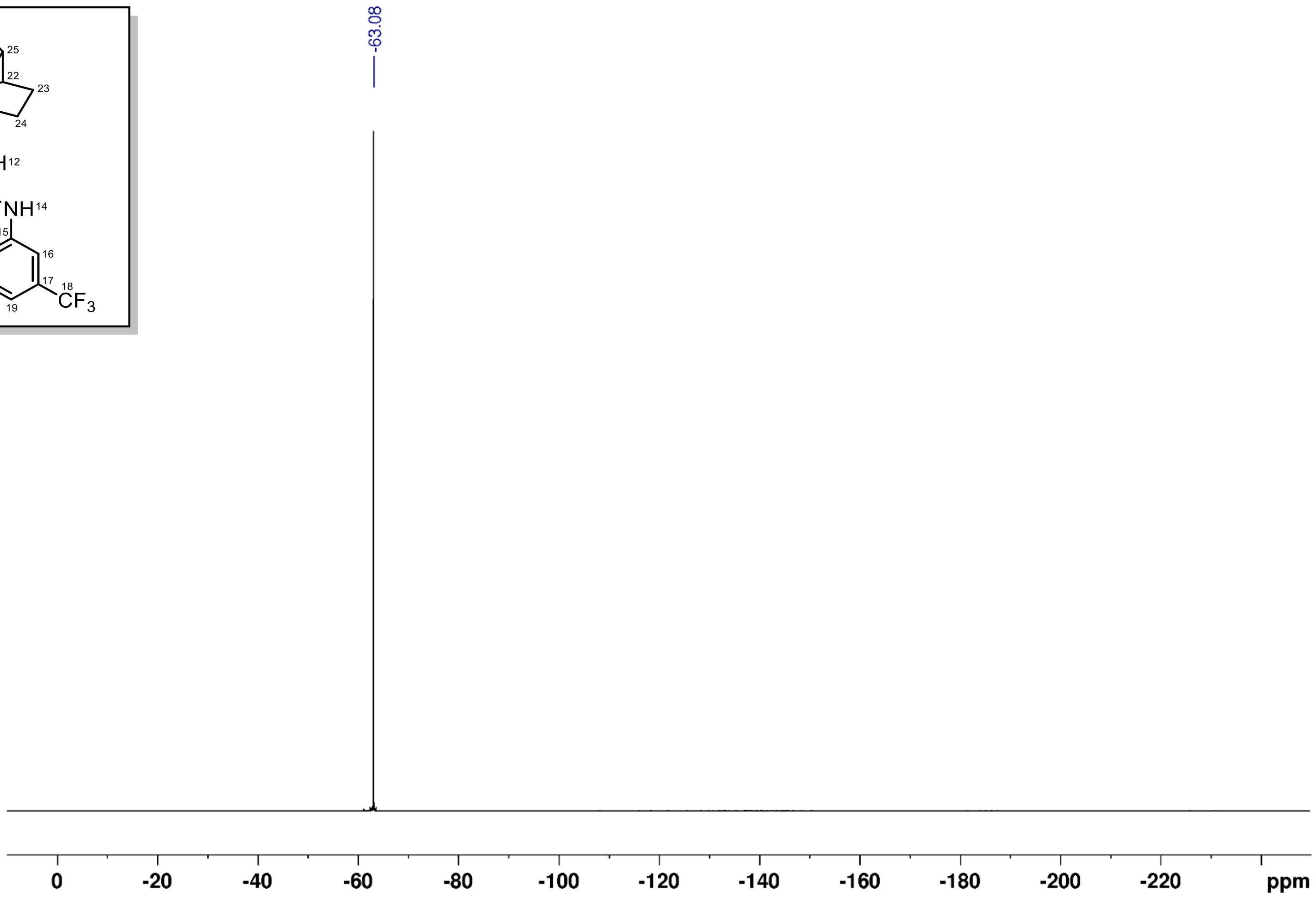

^{1}H NMR (700 MHz, $CDCl_3$) for (1*S*,2*S*,4*S*,5*R*)-2-((*R*)-(3-(3,5-bis(trifluoromethyl)phenyl)ureido)(6-methoxyquinolin-4-yl)methyl)-1-(3,5-di-*tert*-butylbenzyl)-5-ethylquinuclidin-1-ium bromide (**C4**•Br)

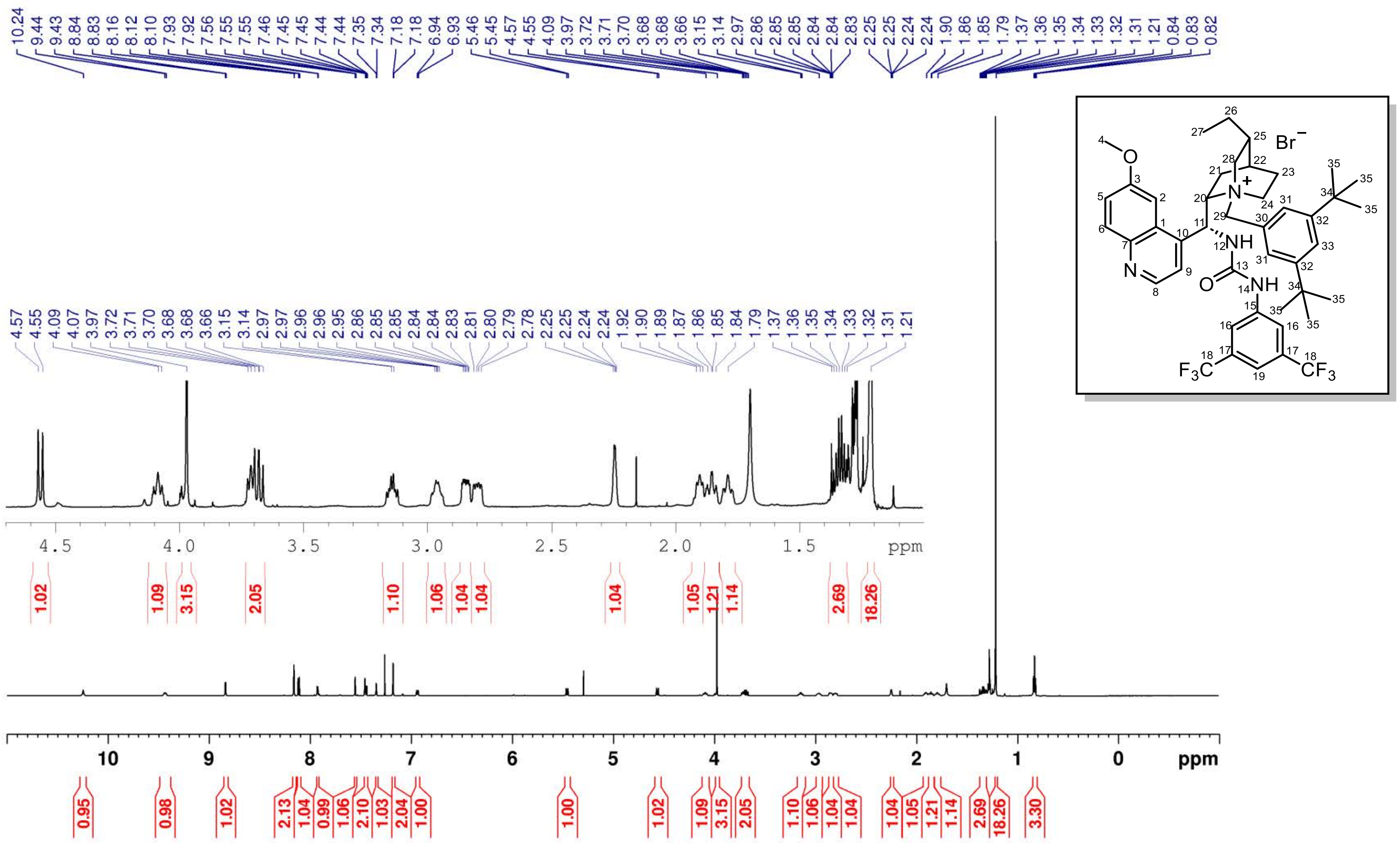

^{13}C NMR (176 MHz, CDCl$_3$) for (1*S*,2*S*,4*S*,5*R*)-2-((*R*)-(3-(3,5-bis(trifluoromethyl)phenyl)ureido)(6-methoxyquinolin-4-yl)methyl)-1-(3,5-di-*tert*-butylbenzyl)-5-ethylquinuclidin-1-ium bromide (**C4**•Br)

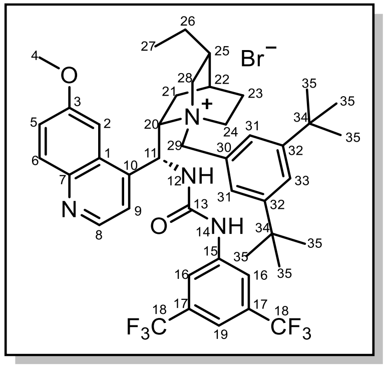

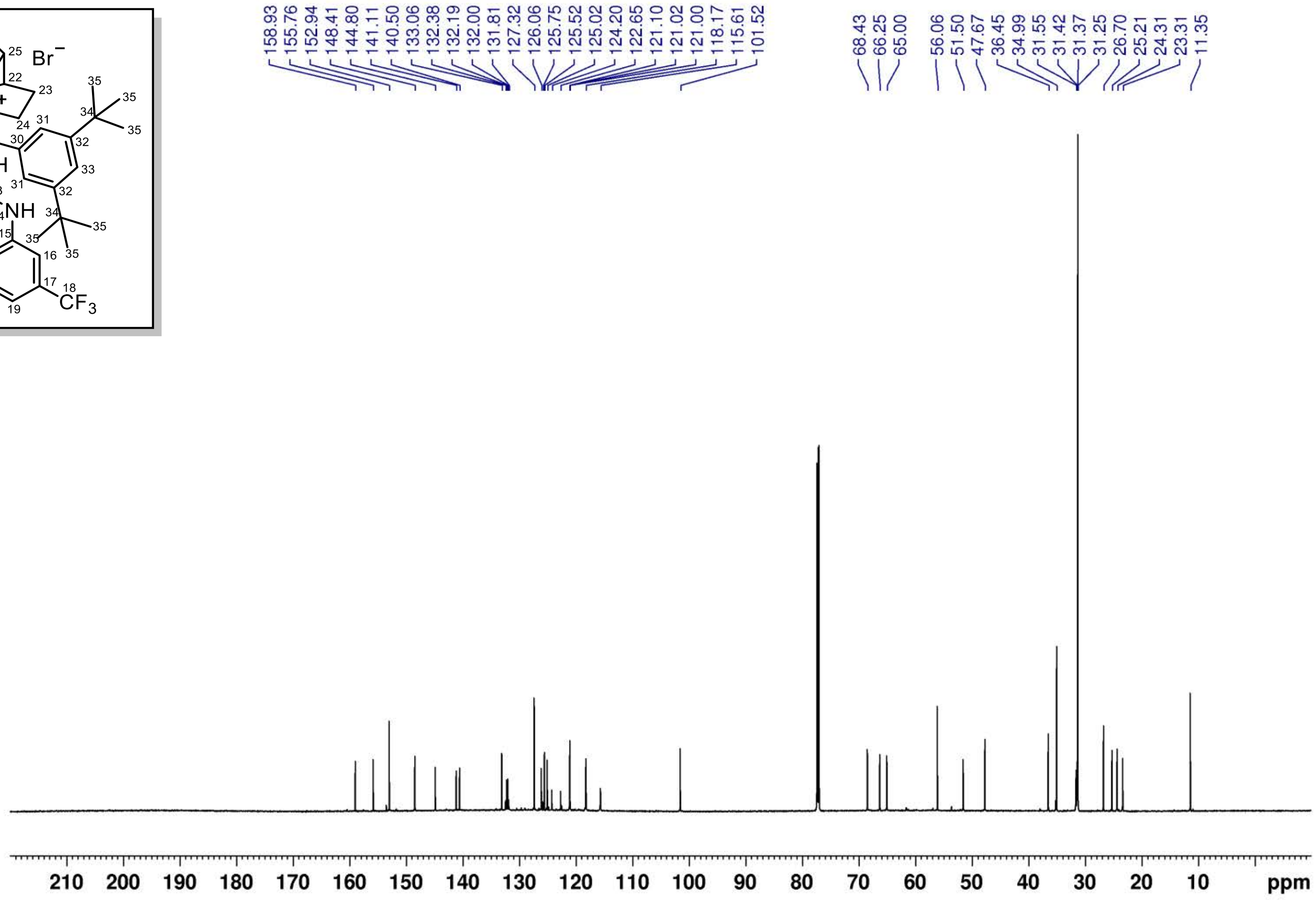

^{19}F NMR (376 MHz, $CDCl_3$) for (1*S*,2*S*,4*S*,5*R*)-2-((*R*)-(3-(3,5-bis(trifluoromethyl)phenyl)ureido)(6-methoxyquinolin-4-yl)methyl)-1-(3,5-di-*tert*-butylbenzyl)-5-ethylquinuclidin-1-ium bromide (**C4**•Br)

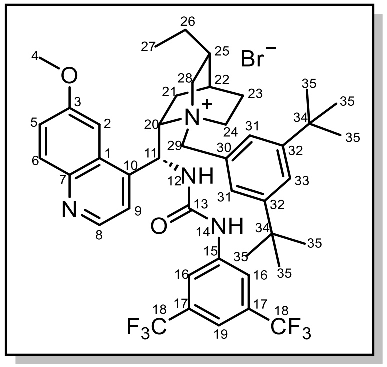

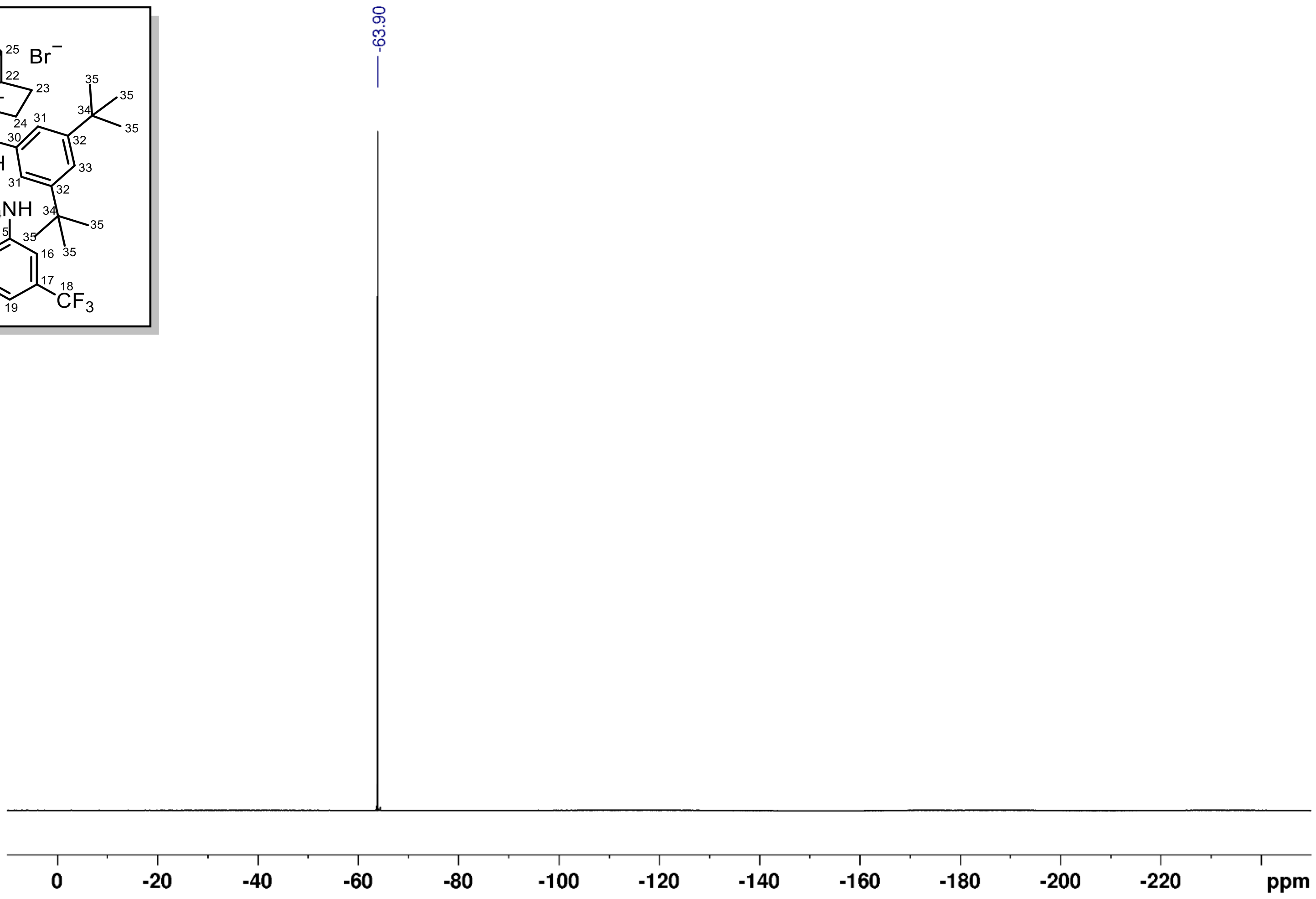

^{1}H NMR (700 MHz, CDCl3) for 1-(3,5-bis(trifluoromethyl)phenyl)-3-((*R*)-((1*S*,2*S*,4*S*,5*R*)-5-ethylquinuclidin-2-yl)(6-methoxyquinolin-4-yl)methyl)thiourea

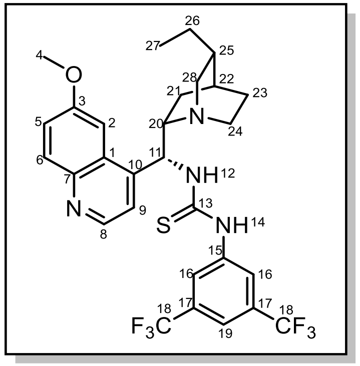

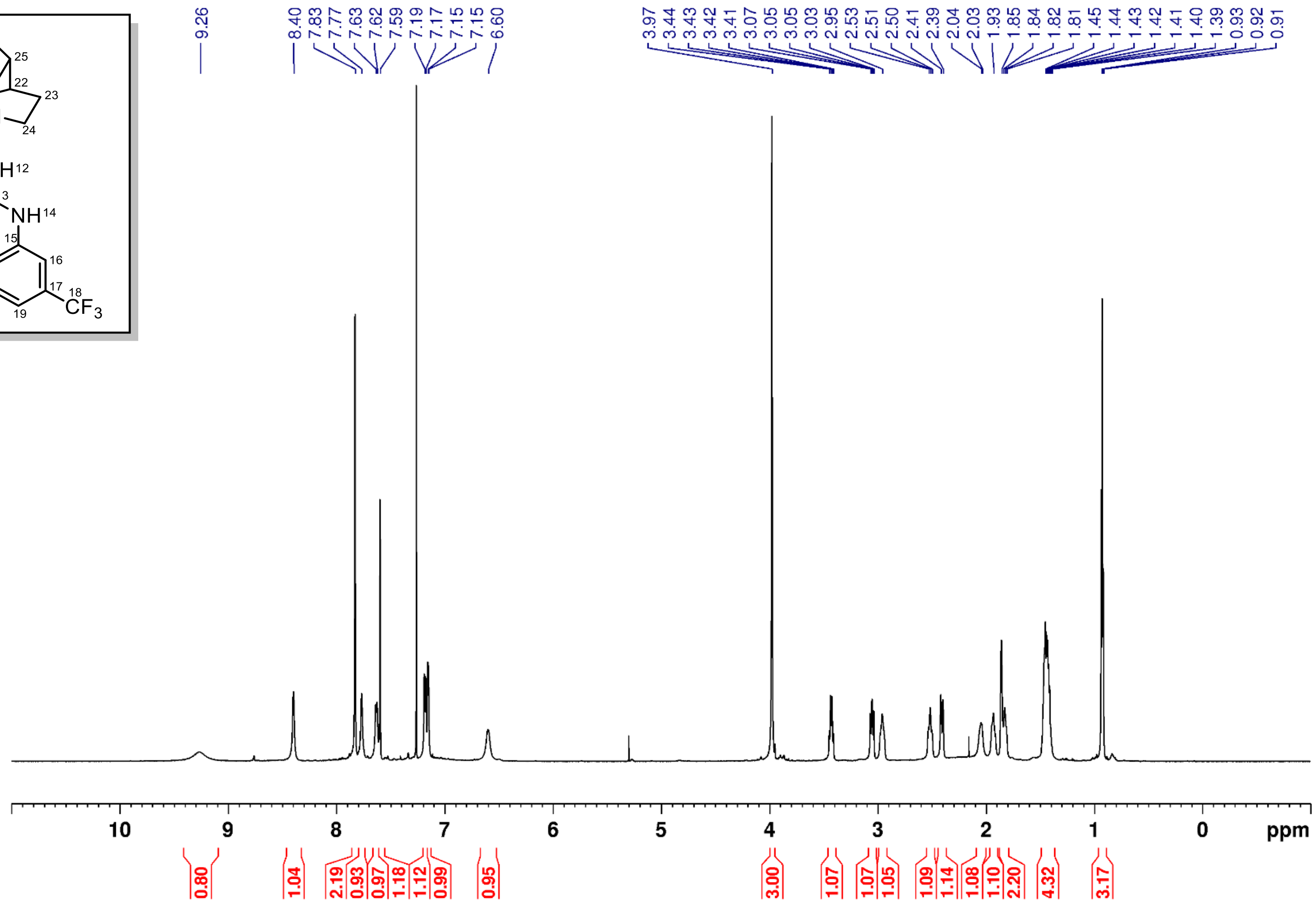

^{13}C NMR (176 MHz, CDCl$_3$) for 1-(3,5-bis(trifluoromethyl)phenyl)-3-((*R*)-((1*S*,2*S*,4*S*,5*R*)-5-ethylquinuclidin-2-yl)(6-methoxyquinolin-4-yl)methyl)thiourea

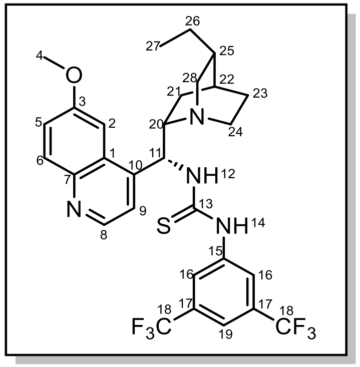

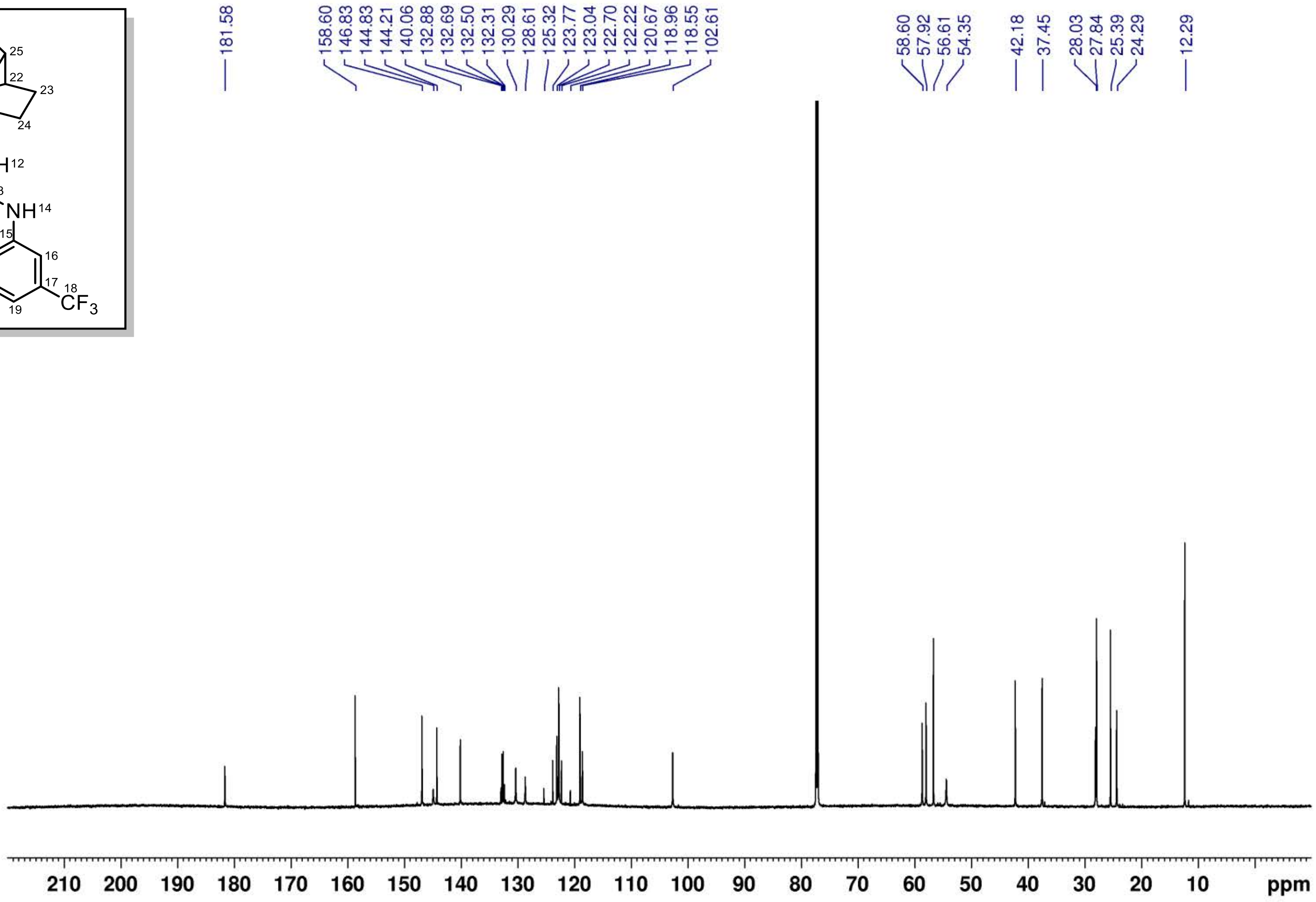

^{19}F NMR (376 MHz, $CDCl_3$) for 1-(3,5-bis(trifluoromethyl)phenyl)-3-((*R*)-((1*S*,2*S*,4*S*,5*R*)-5-ethylquinuclidin-2-yl)(6-methoxyquinolin-4-yl)methyl)thiourea

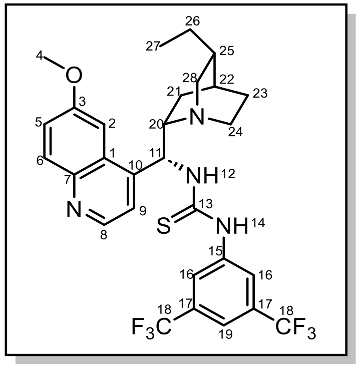

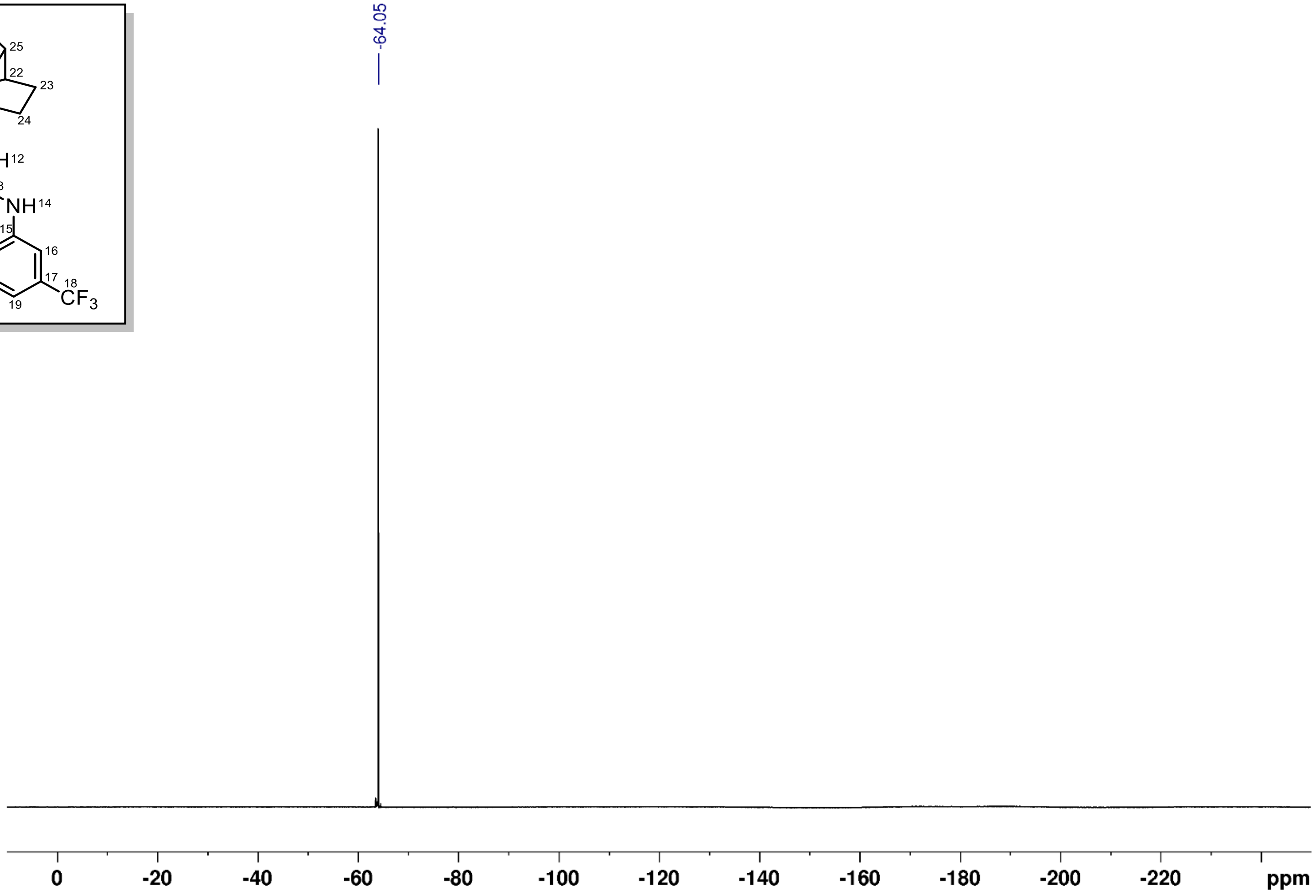

^{1}H NMR (700 MHz, $CDCl_3$) for (1*S*,2*S*,4*S*,5*R*)-2-((*R*)-(3-(3,5-bis(trifluoromethyl)phenyl)thioureido)(6-methoxyquinolin-4-yl)methyl)-1-(3,5-di-*tert*-butylbenzyl)-5-ethylquinuclidin-1-ium bromide (**C5•**Br)

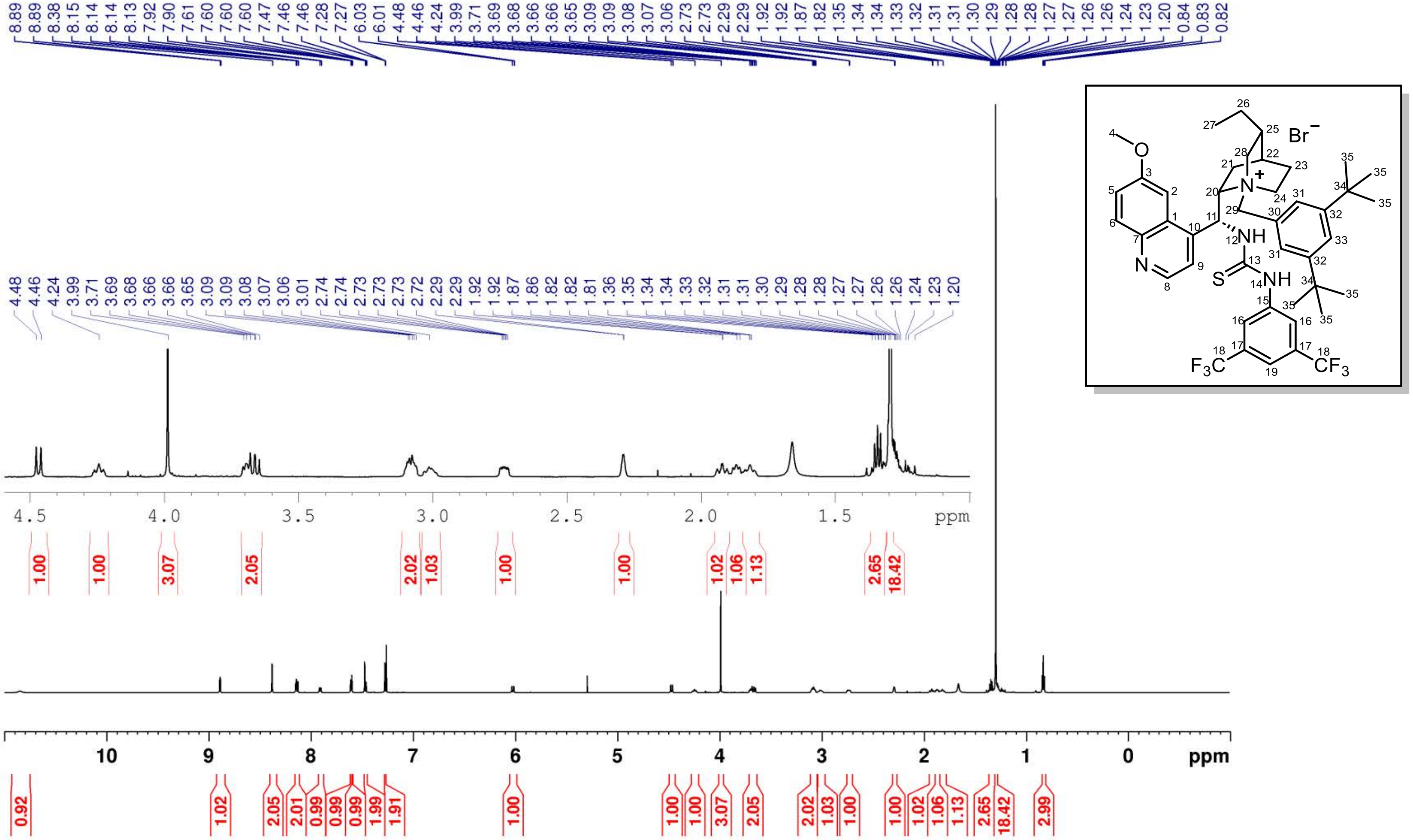

^{13}C NMR (176 MHz, CDCl$_3$) for (1*S*,2*S*,4*S*,5*R*)-2-((*R*)-(3-(3,5-bis(trifluoromethyl)phenyl)thioureido)(6-methoxyquinolin-4-yl)methyl)-1-(3,5-di-*tert*-butylbenzyl)-5-ethylquinuclidin-1-ium bromide (**C5**•Br)

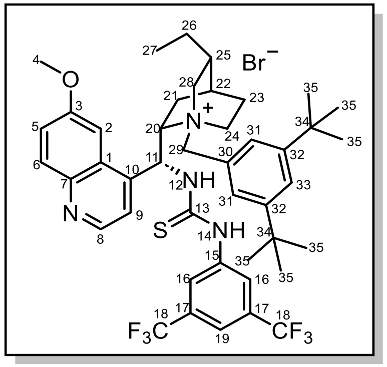

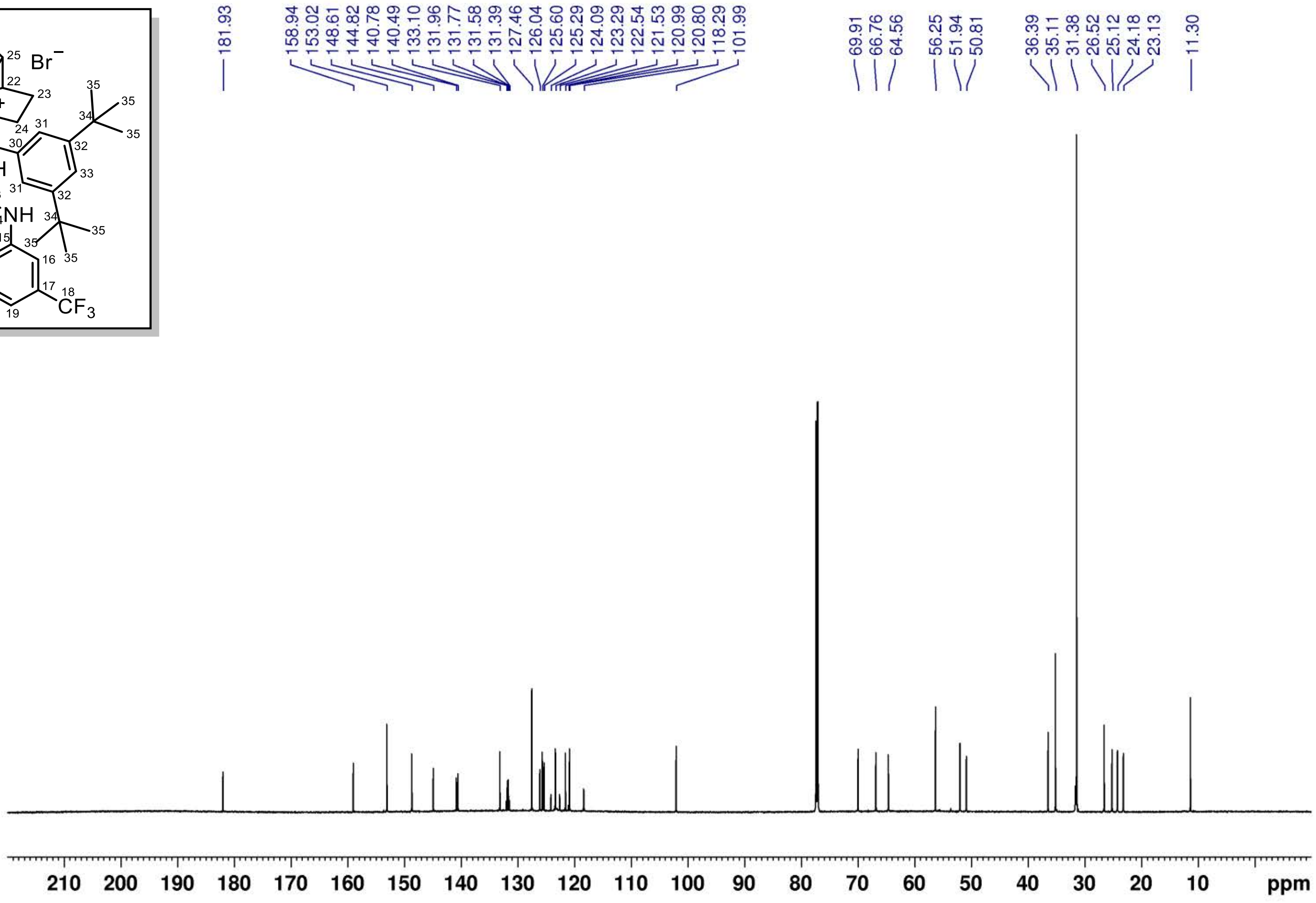

^{19}F NMR (376 MHz, CDCl$_3$) for (1*S*,2*S*,4*S*,5*R*)-2-((*R*)-(3-(3,5-bis(trifluoromethyl)phenyl)thioureido)(6-methoxyquinolin-4-yl)methyl)-1-(3,5-di-*tert*-butylbenzyl)-5-ethylquinuclidin-1-ium bromide (**C5**•Br)

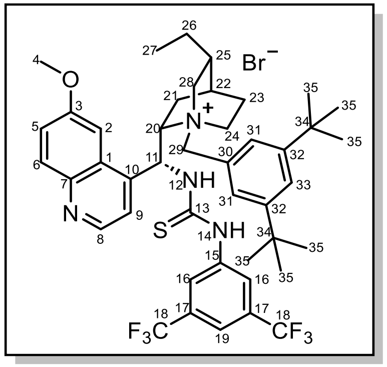

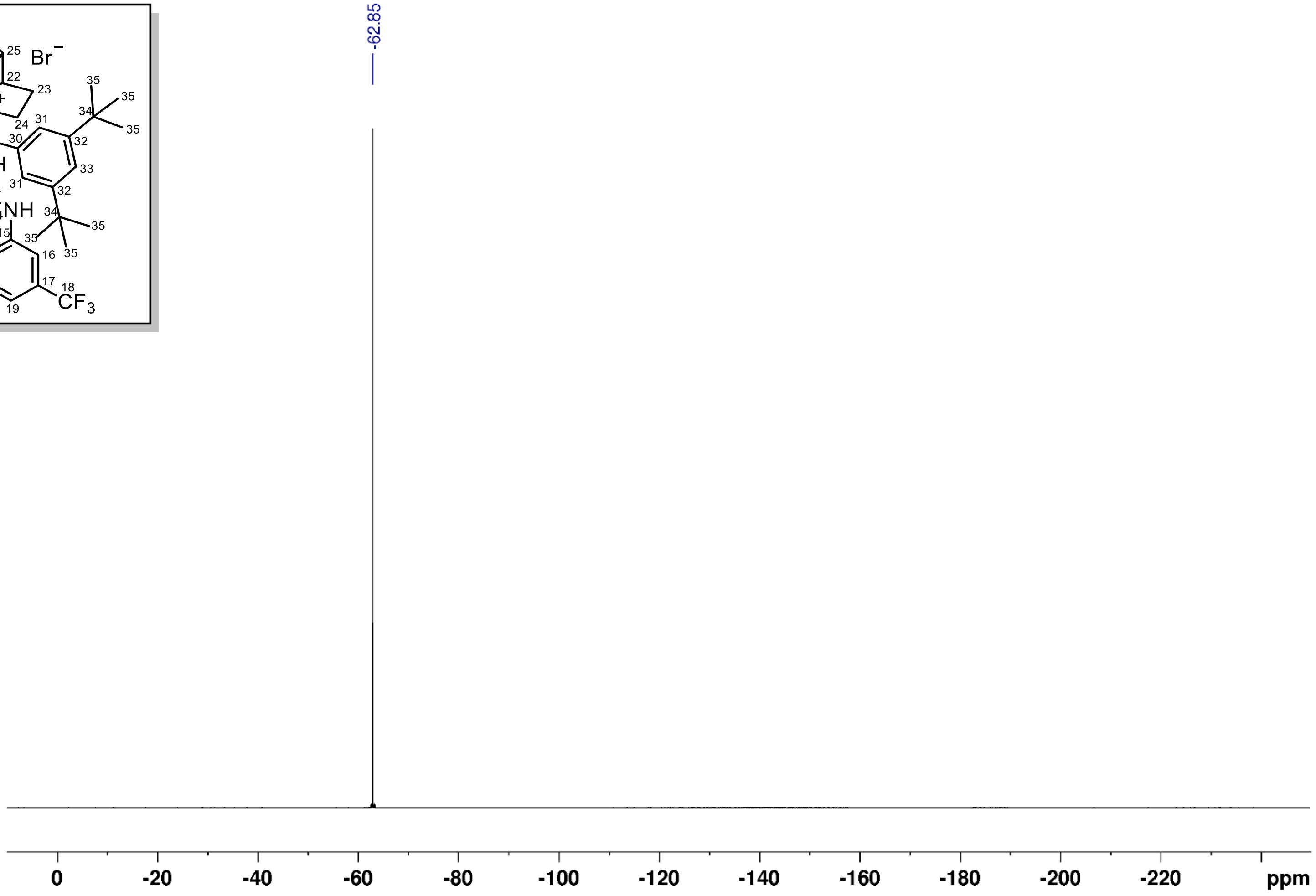

^{1}H NMR (700 MHz, CD_3OD) for 3-((3,5-bis(trifluoromethyl)phenyl)amino)-4-(((*R*)-((1*S*,2*S*,4*S*,5*R*)-5-ethylquinuclidin-2-yl)(6-methoxyquinolin-4-yl)methyl)amino)cyclobut-3-ene-1,2-dione

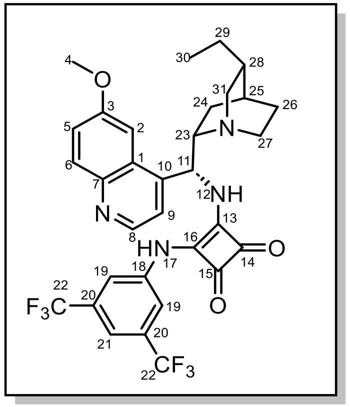

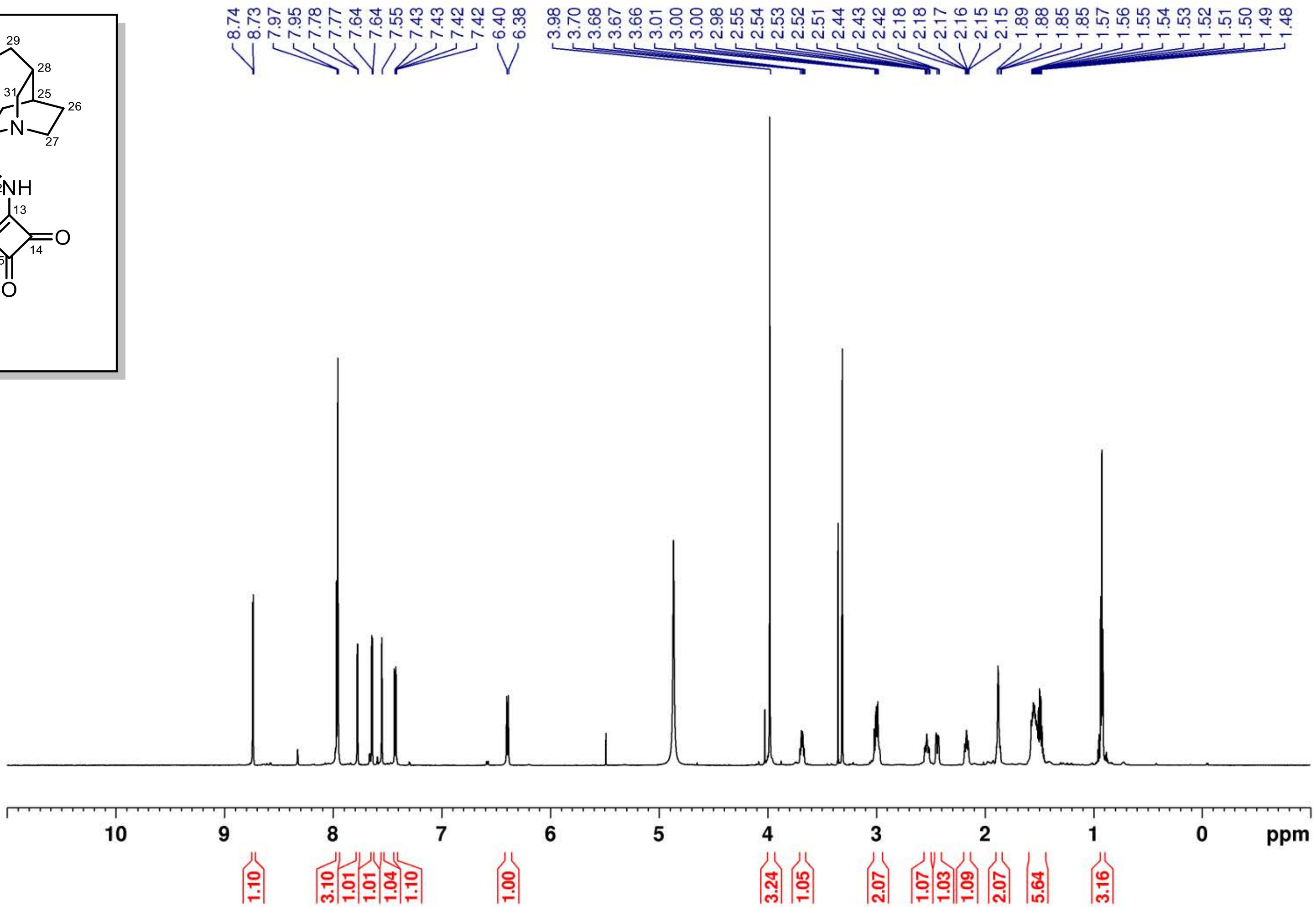

^{13}C NMR (176 MHz, CD_3OD) for 3-((3,5-bis(trifluoromethyl)phenyl)amino)-4-(((*R*)-((1*S*,2*S*,4*S*,5*R*)-5-ethylquinuclidin-2-yl)(6-methoxyquinolin-4-yl)methyl)amino)cyclobut-3-ene-1,2-dione

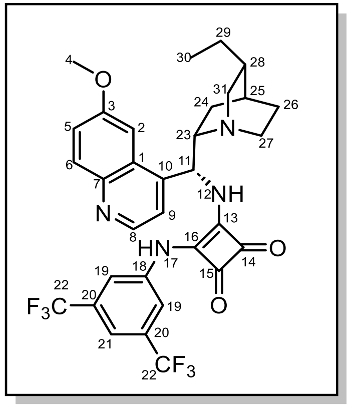

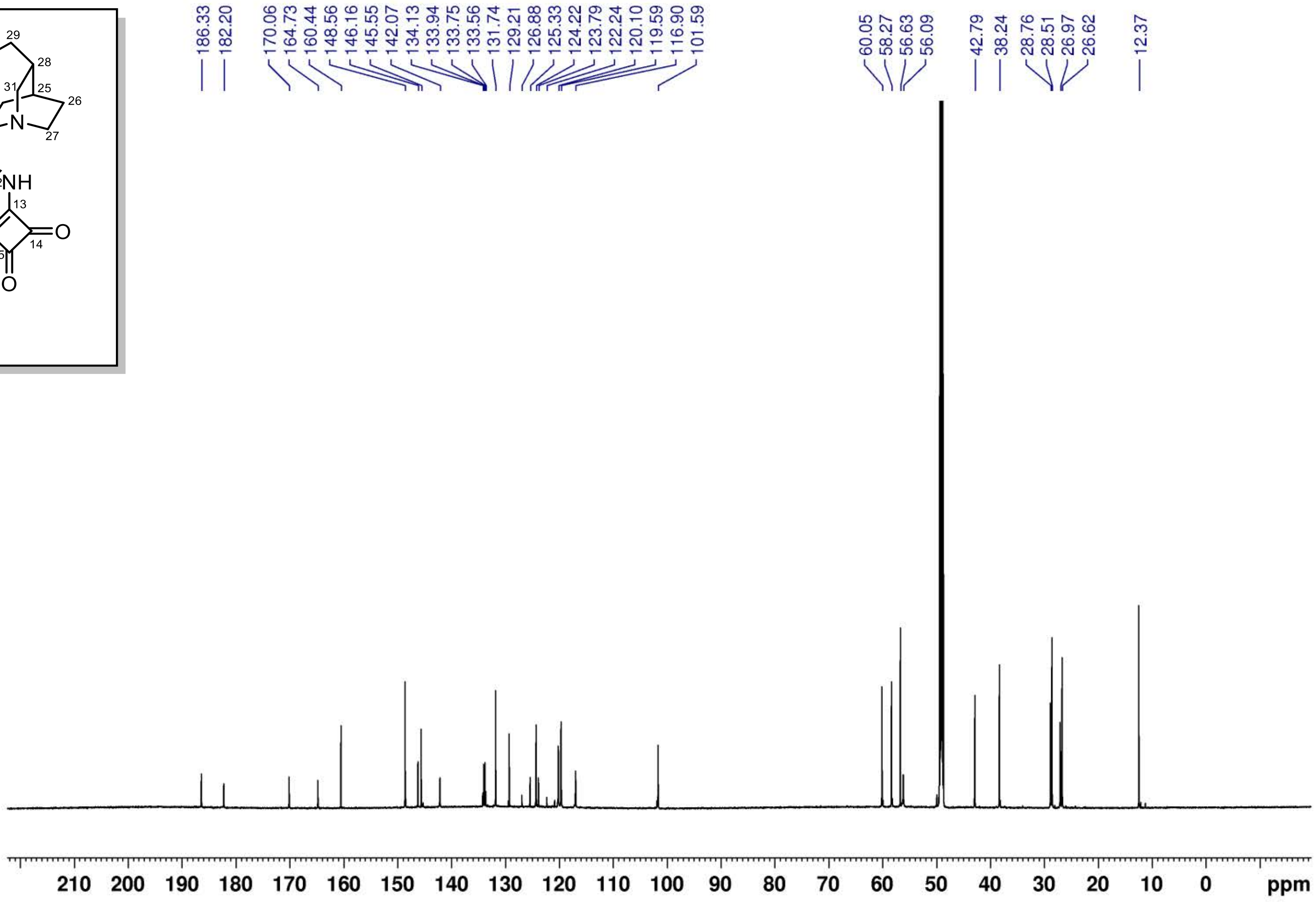

^{19}F NMR (376 MHz, CD_3OD) for 3-((3,5-bis(trifluoromethyl)phenyl)amino)-4-(((*R*)-((1*S*,2*S*,4*S*,5*R*)-5-ethylquinuclidin-2-yl)(6-methoxyquinolin-4-yl)methyl)amino)cyclobut-3-ene-1,2-dione

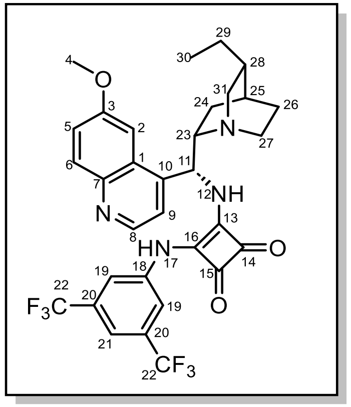

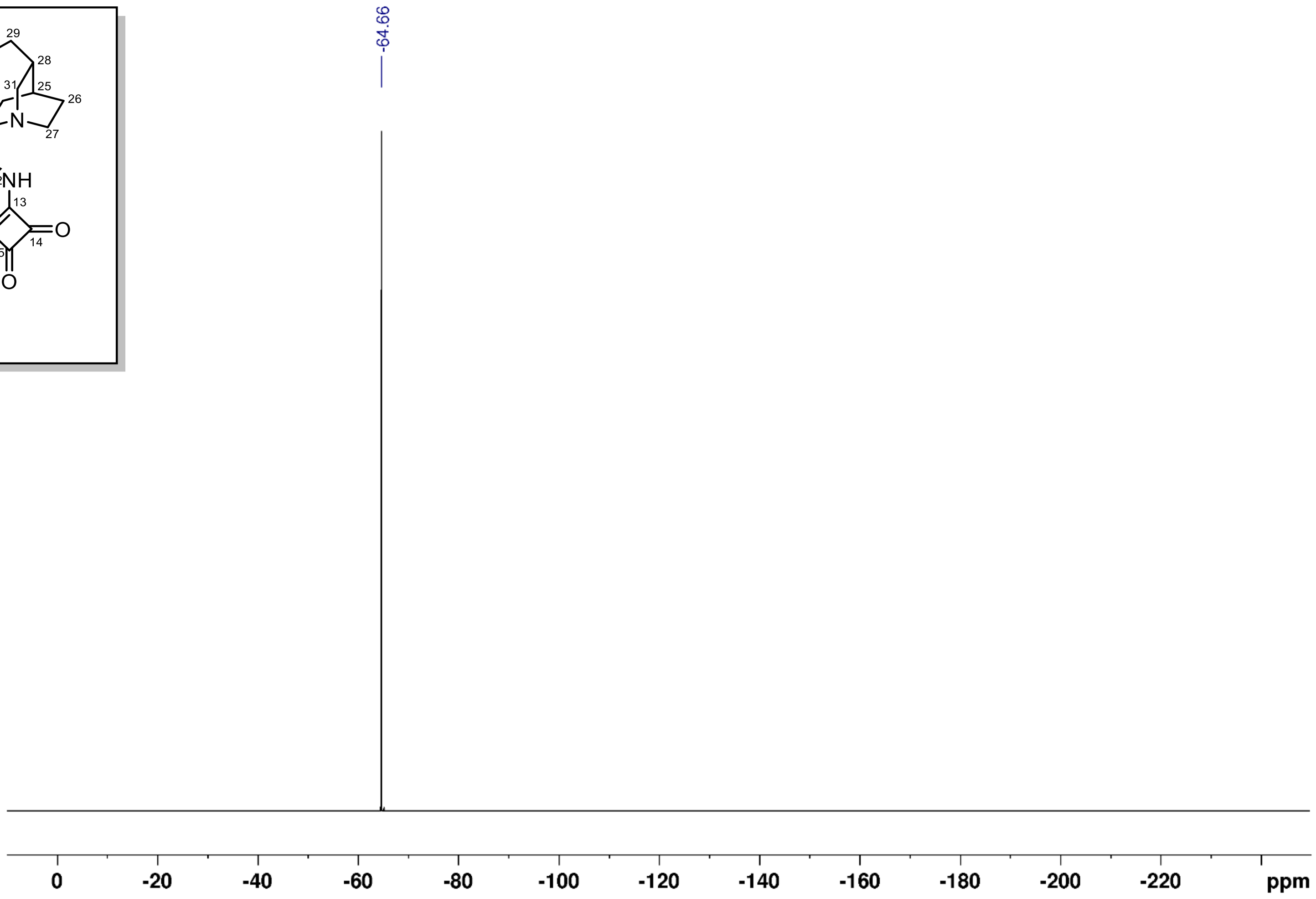

^{1}H NMR (700 MHz, $CDCl_3$) for (1*S*,2*S*,4*S*,5*R*)-2-((*R*)-((2-((3,5-bis(trifluoromethyl)phenyl)amino)-3,4-dioxocyclobut-1-en-1-yl)amino)(6-methoxyquinolin-4-yl)methyl)-1-(3,5-di-*tert*-butylbenzyl)-5-ethylquinuclidin-1-ium bromide (**C6**•Br)

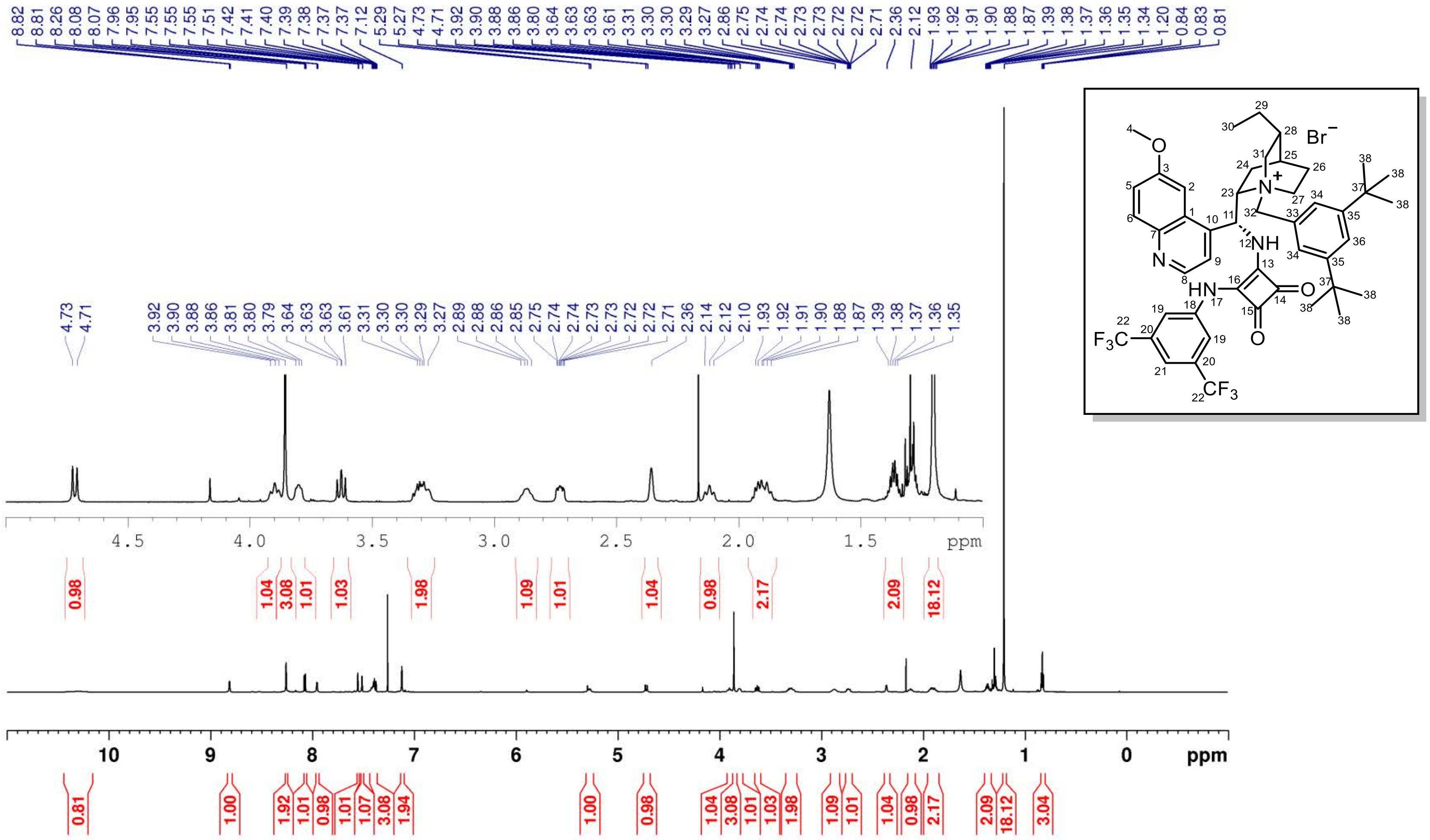

^{13}C NMR (176 MHz, CDCl$_3$) for (1*S*,2*S*,4*S*,5*R*)-2-((*R*)-((2-((3,5-bis(trifluoromethyl)phenyl)amino)-3,4-dioxocyclobut-1-en-1-yl)amino)(6-methoxyquinolin-4-yl)methyl)-1-(3,5-di-*tert*-butylbenzyl)-5-ethylquinuclidin-1-ium bromide (**C6**•Br)

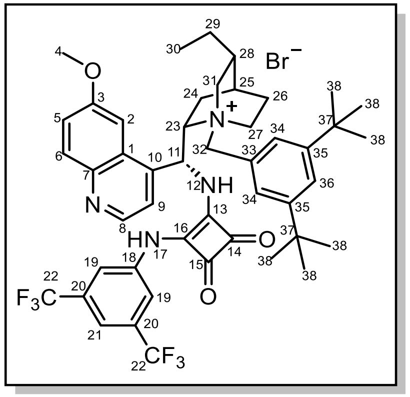

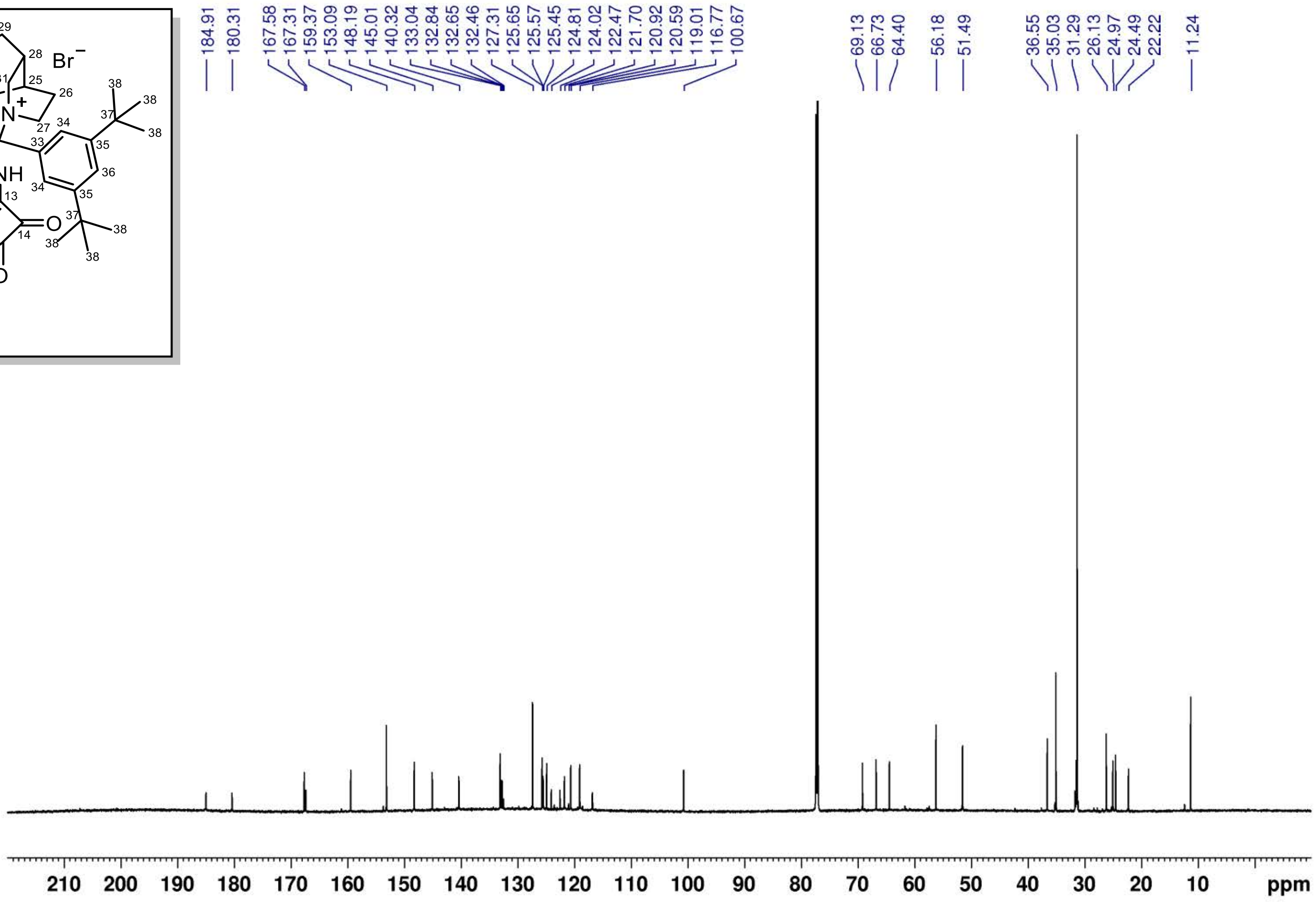

^{19}F NMR (376 MHz, $CDCl_3$) for (1*S*,2*S*,4*S*,5*R*)-2-((*R*)-((2-((3,5-bis(trifluoromethyl)phenyl)amino)-3,4-dioxocyclobut-1-en-1-yl)amino)(6-methoxyquinolin-4-yl)methyl)-1-(3,5-di-*tert*-butylbenzyl)-5-ethylquinuclidin-1-ium bromide (**C6**•Br)

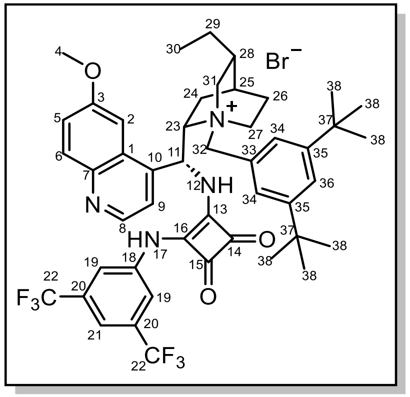

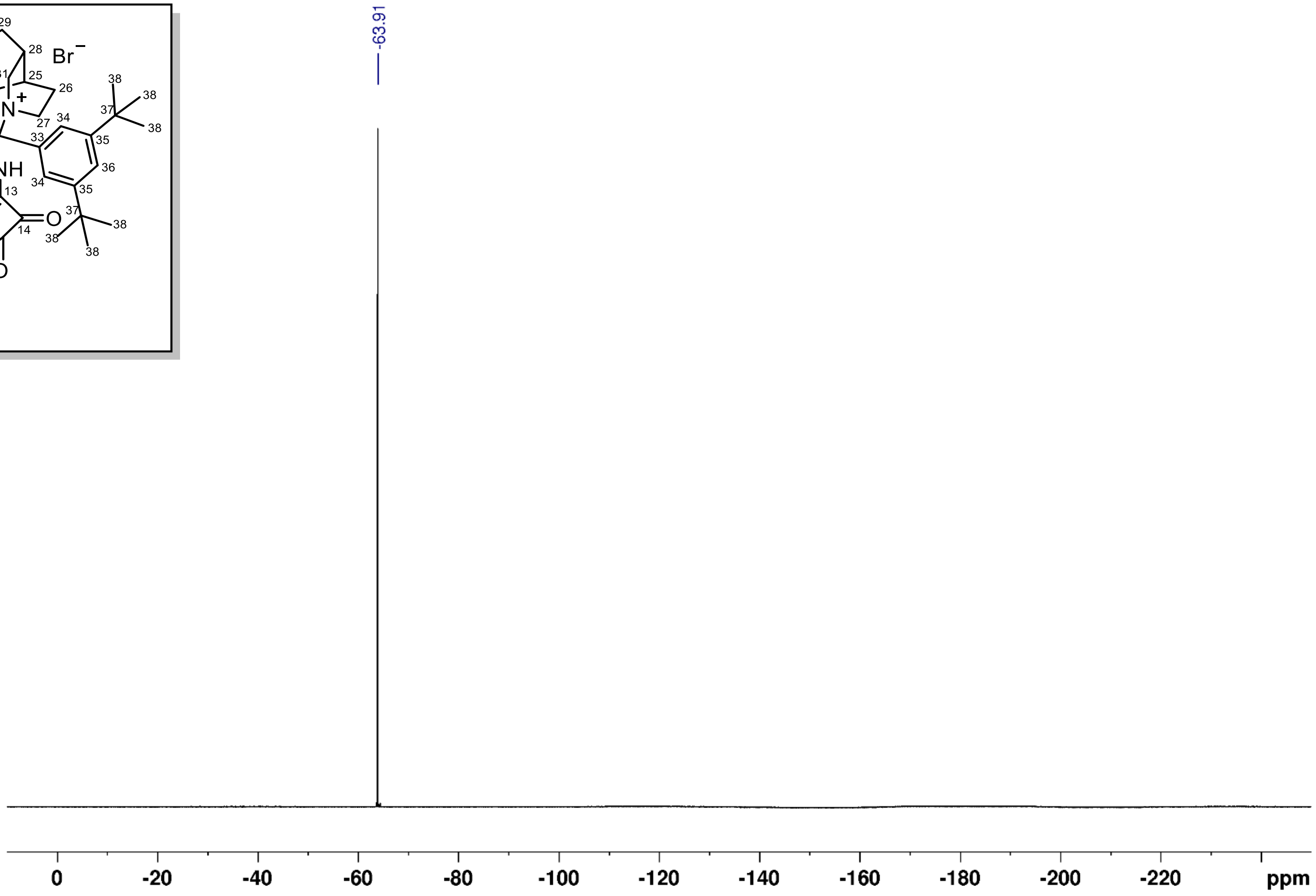

^{1}H NMR (700 MHz, CDCl$_3$) for 1-cyclohexyl-3-((*R*)-((1*S*,2*S*,4*S*,5*R*)-5-ethylquinuclidin-2-yl)(6-methoxyquinolin-4-yl)methyl)urea

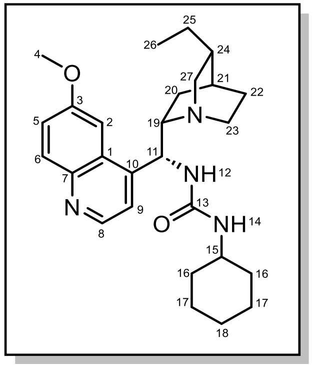

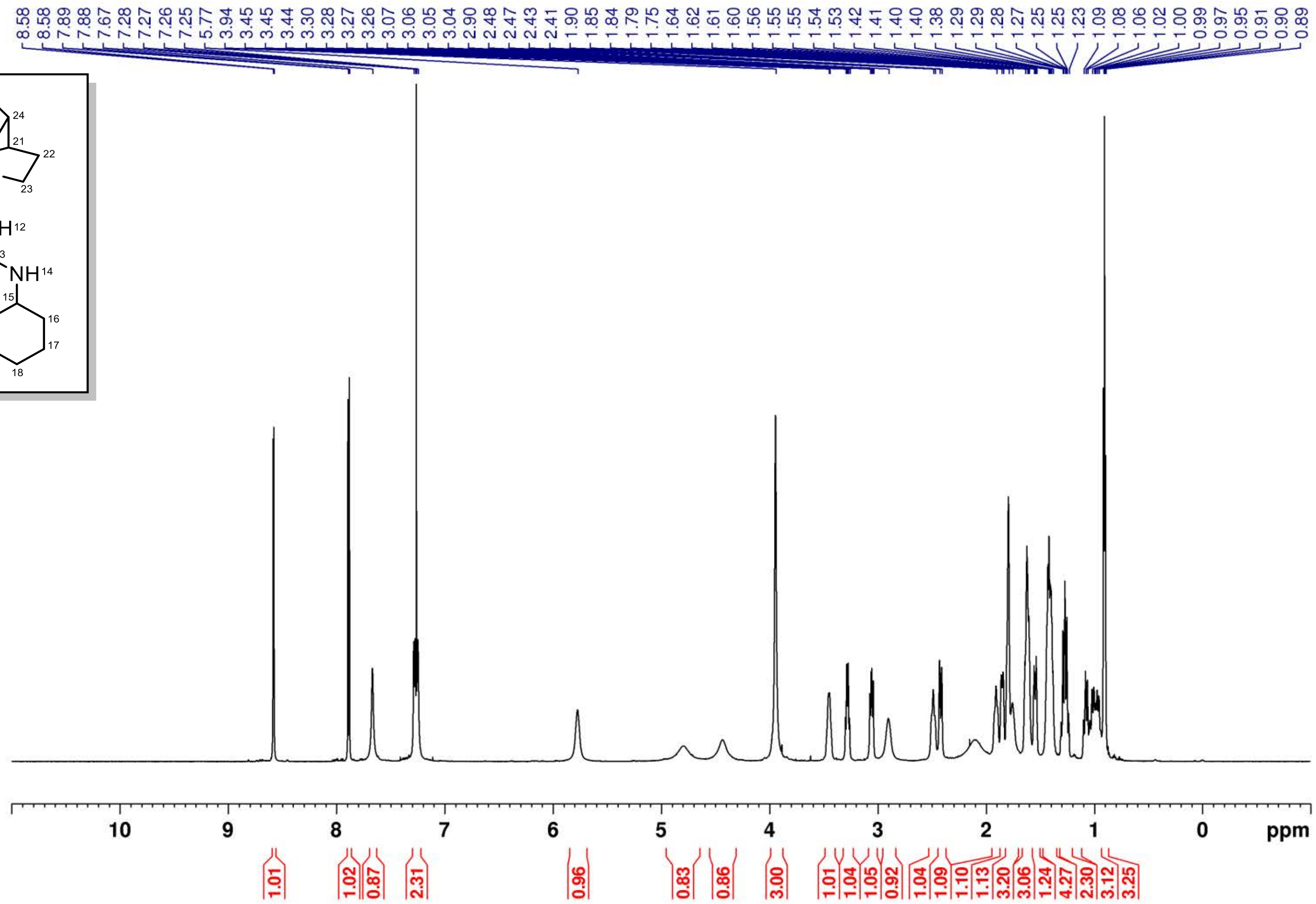

^{13}C NMR (176 MHz, $CDCl_3$) for 1-cyclohexyl-3-((*R*)-((1*S*,2*S*,4*S*,5*R*)-5-ethylquinuclidin-2-yl)(6-methoxyquinolin-4-yl)methyl)urea

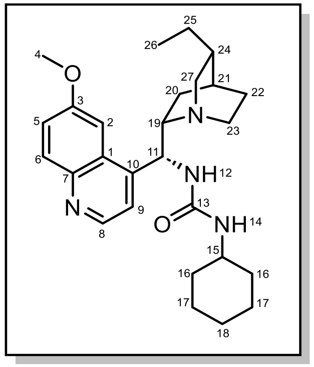

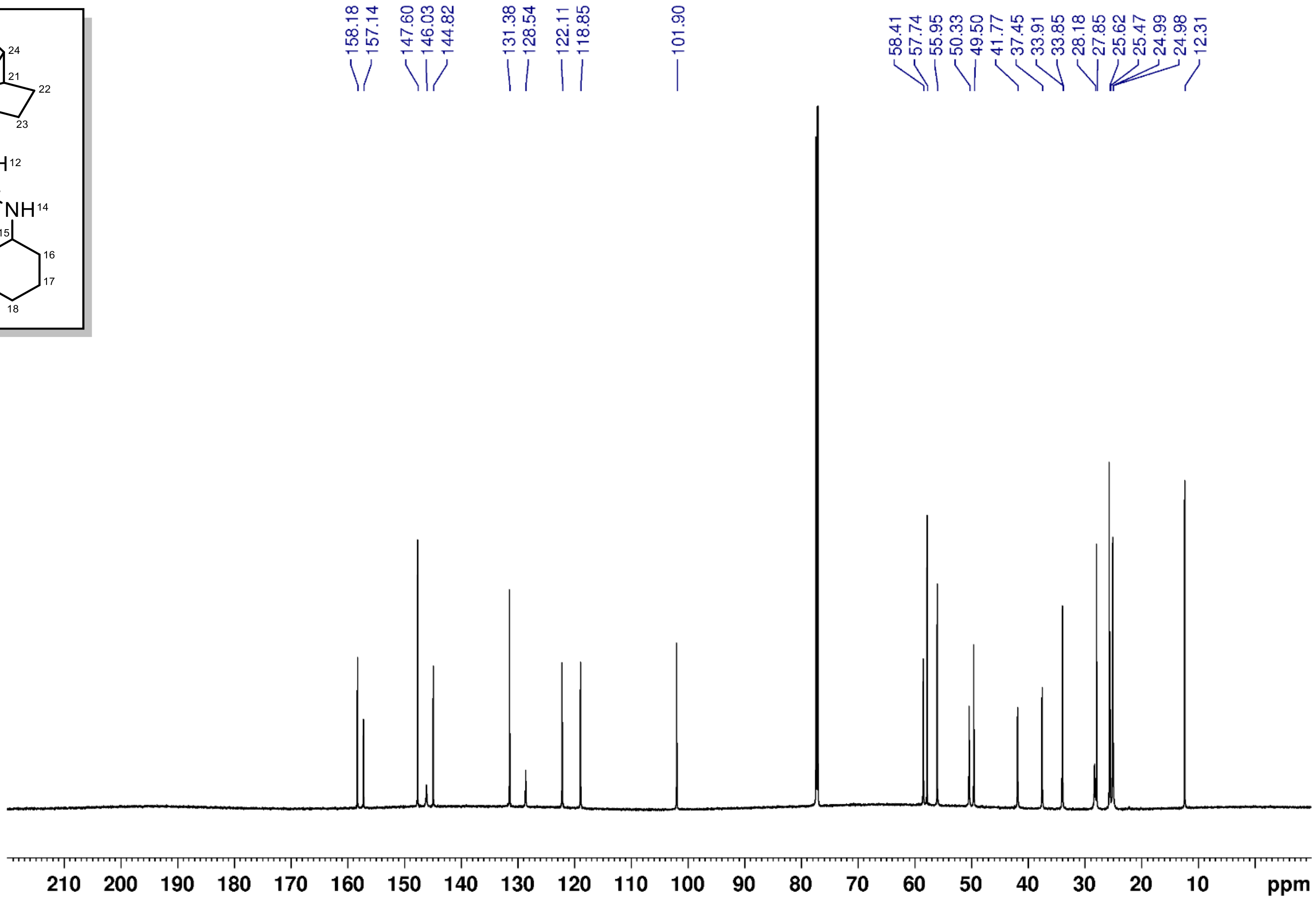

^{1}H NMR (700 MHz, CD_3OD) for (1*S*,2*S*,4*S*,5*R*)-2-((*R*)-(3-cyclohexylureido)(6-methoxyquinolin-4-yl)methyl)-5-ethyl-1-((3,3'',5,5''-tetra-*tert*-butyl-[1,1':3',1''-terphenyl]-5'-yl)methyl)quinuclidin-1-ium bromide (**C7**•Br)

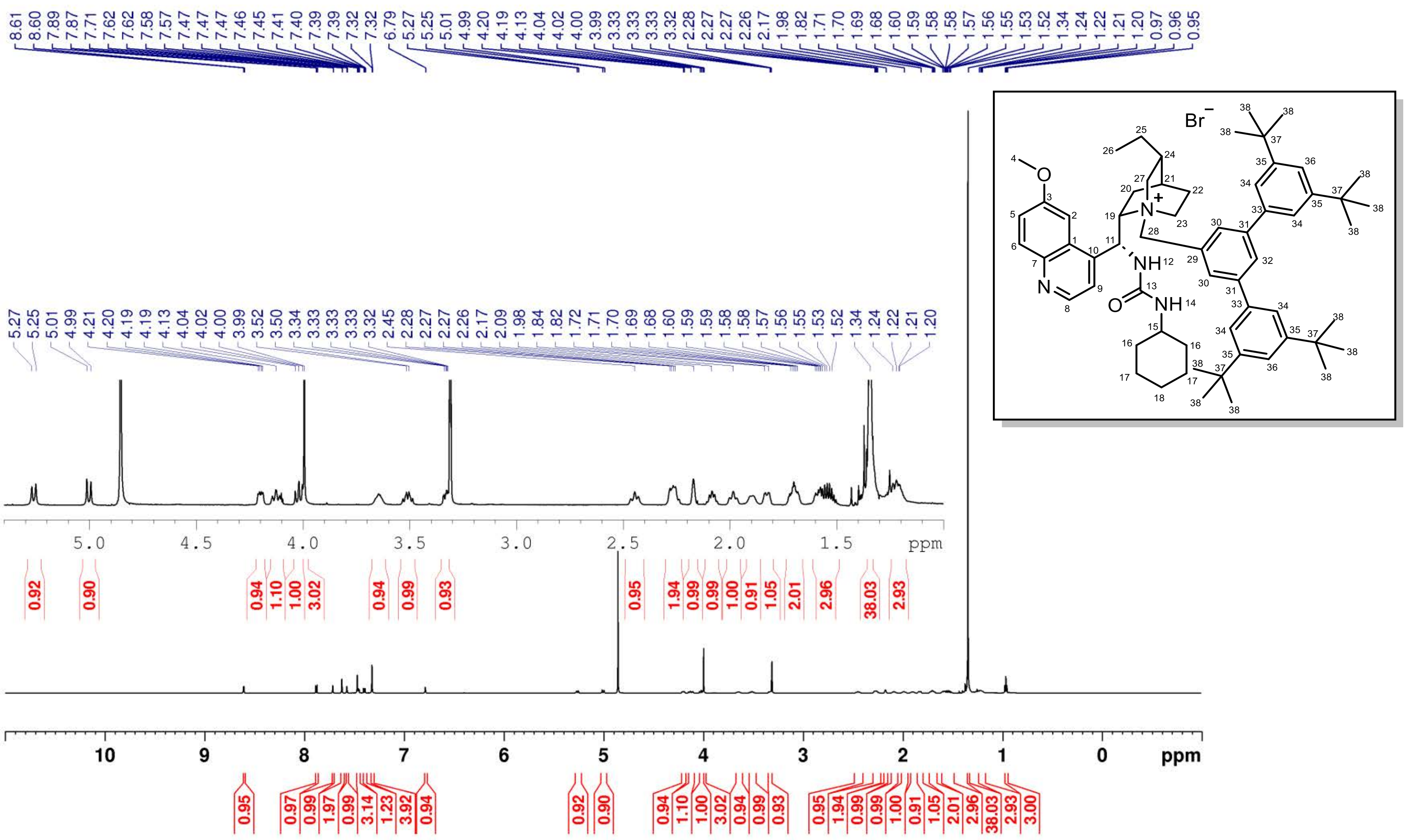

^{13}C NMR (176 MHz, CD$_3$OD) for (1*S*,2*S*,4*S*,5*R*)-2-((*R*)-(3-cyclohexylureido)(6-methoxyquinolin-4-yl)methyl)-5-ethyl-1-((3,3'',5,5''-tetra-*tert*-butyl-[1,1':3',1''-terphenyl]-5'-yl)methyl)quinuclidin-1-ium bromide (**C7**•Br)

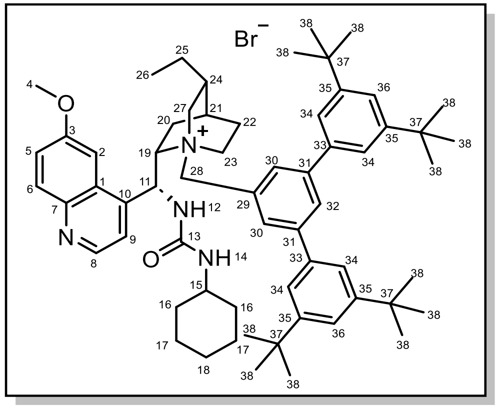

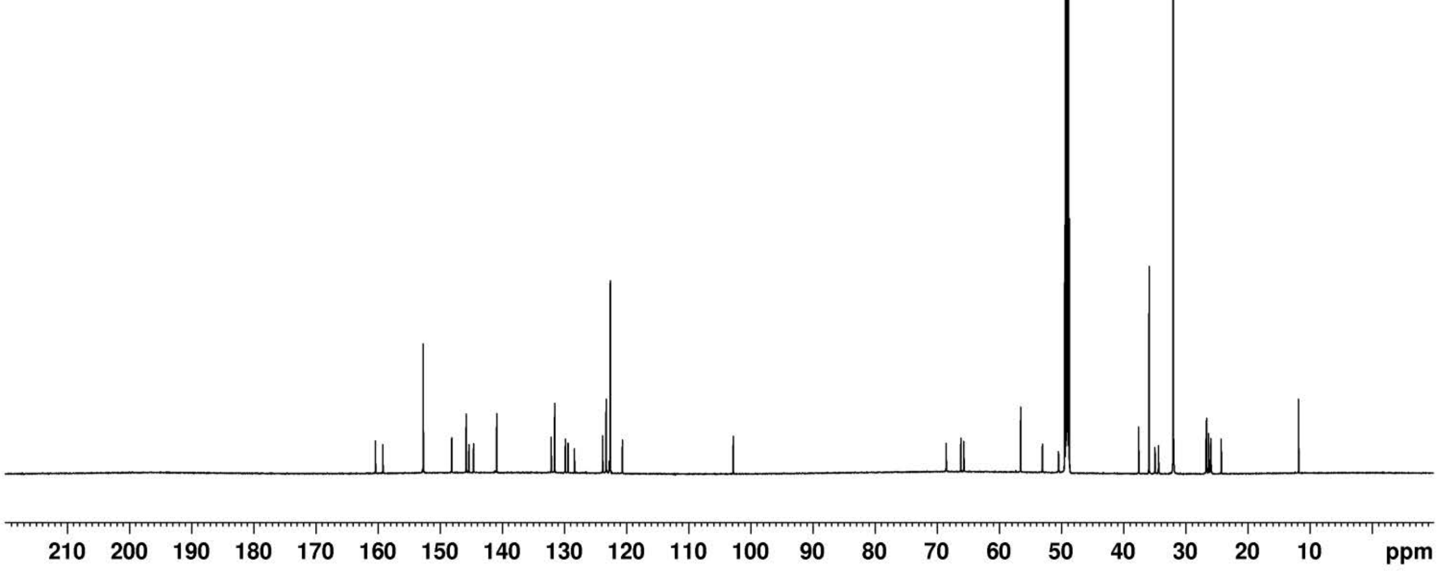

^{1}H NMR (700 MHz, $CDCl_3$) for 1-(3,5-bis(trifluoromethyl)phenyl)-3-((*S*)-((1*S*,2*S*,4*S*,5*R*)-5-ethylquinuclidin-2-yl)(6-methoxyquinolin-4-yl)methyl)urea

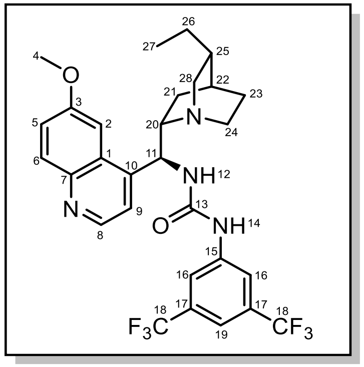

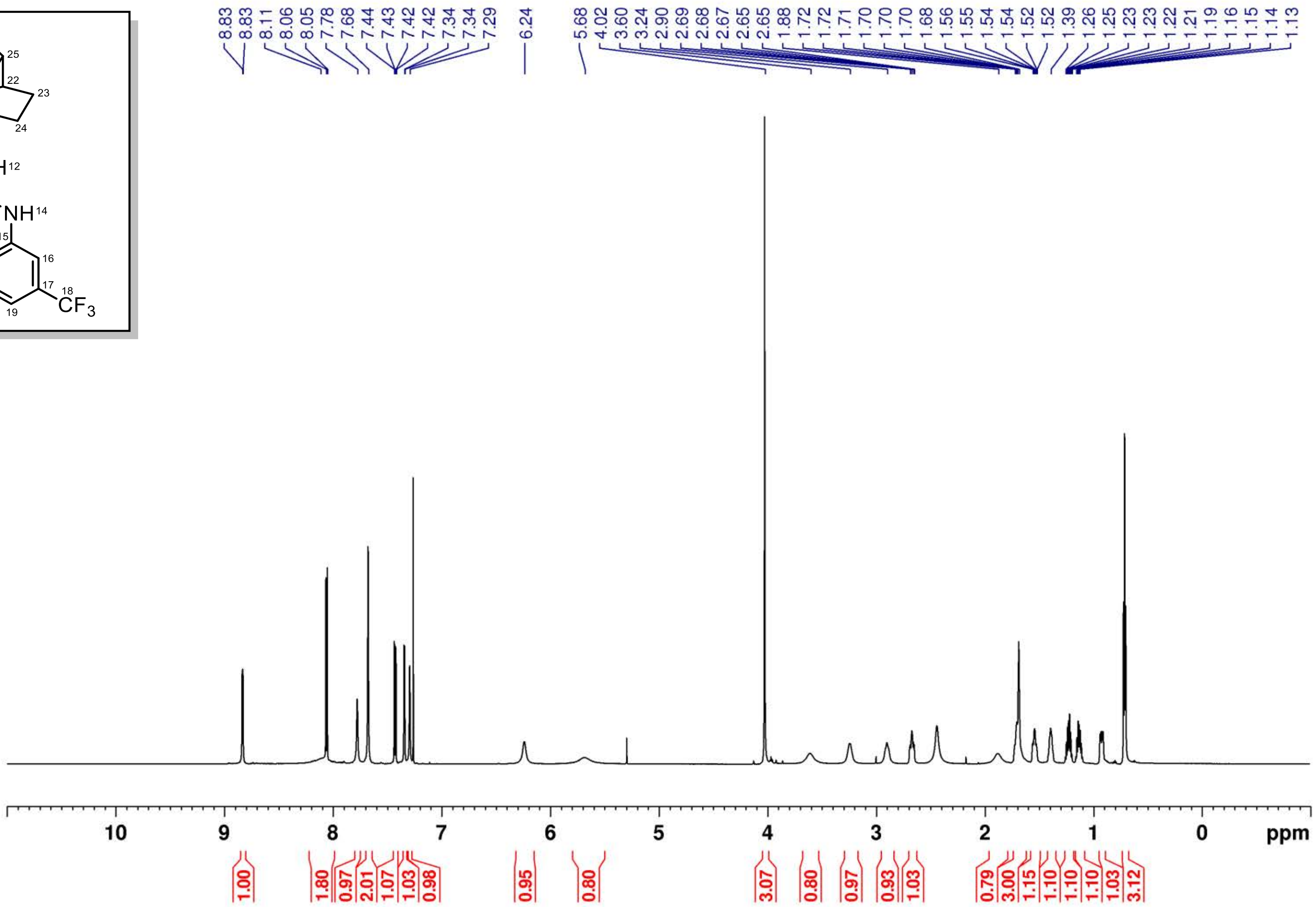

^{13}C NMR (176 MHz, CDCl$_3$) for 1-(3,5-bis(trifluoromethyl)phenyl)-3-((*S*)-((1*S*,2*S*,4*S*,5*R*)-5-ethylquinuclidin-2-yl)(6-methoxyquinolin-4-yl)methyl)urea

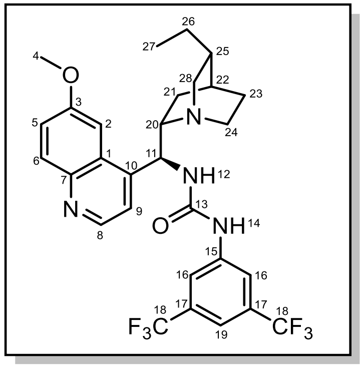

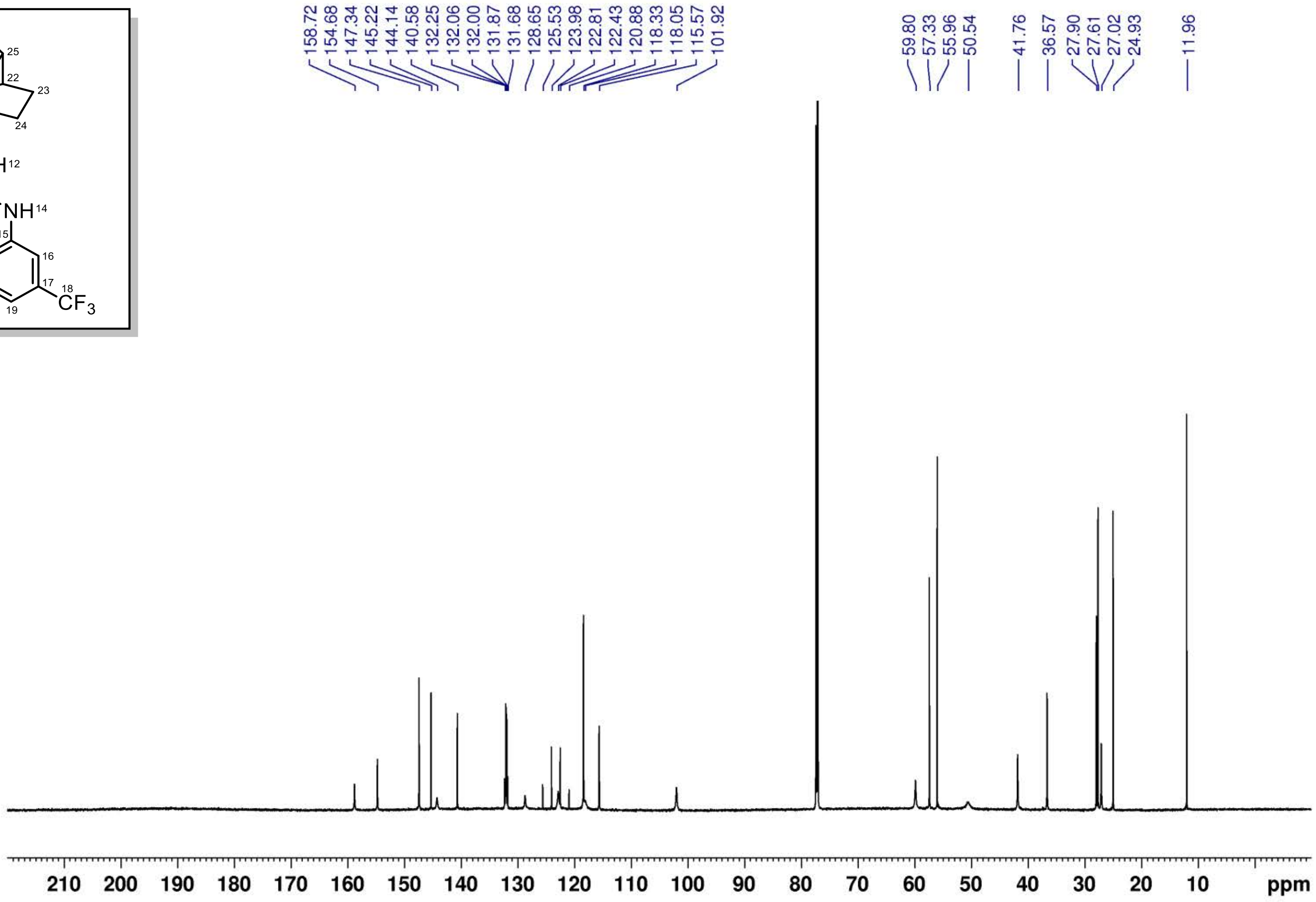

^{19}F NMR (376 MHz, $CDCl_3$) for 1-(3,5-bis(trifluoromethyl)phenyl)-3-((*S*)-((1*S*,2*S*,4*S*,5*R*)-5-ethylquinuclidin-2-yl)(6-methoxyquinolin-4-yl)methyl)urea

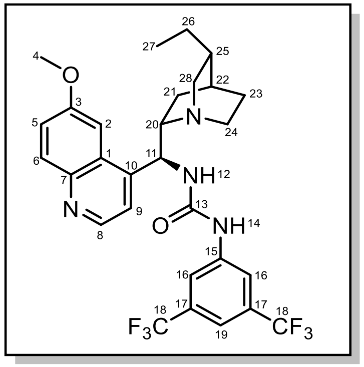

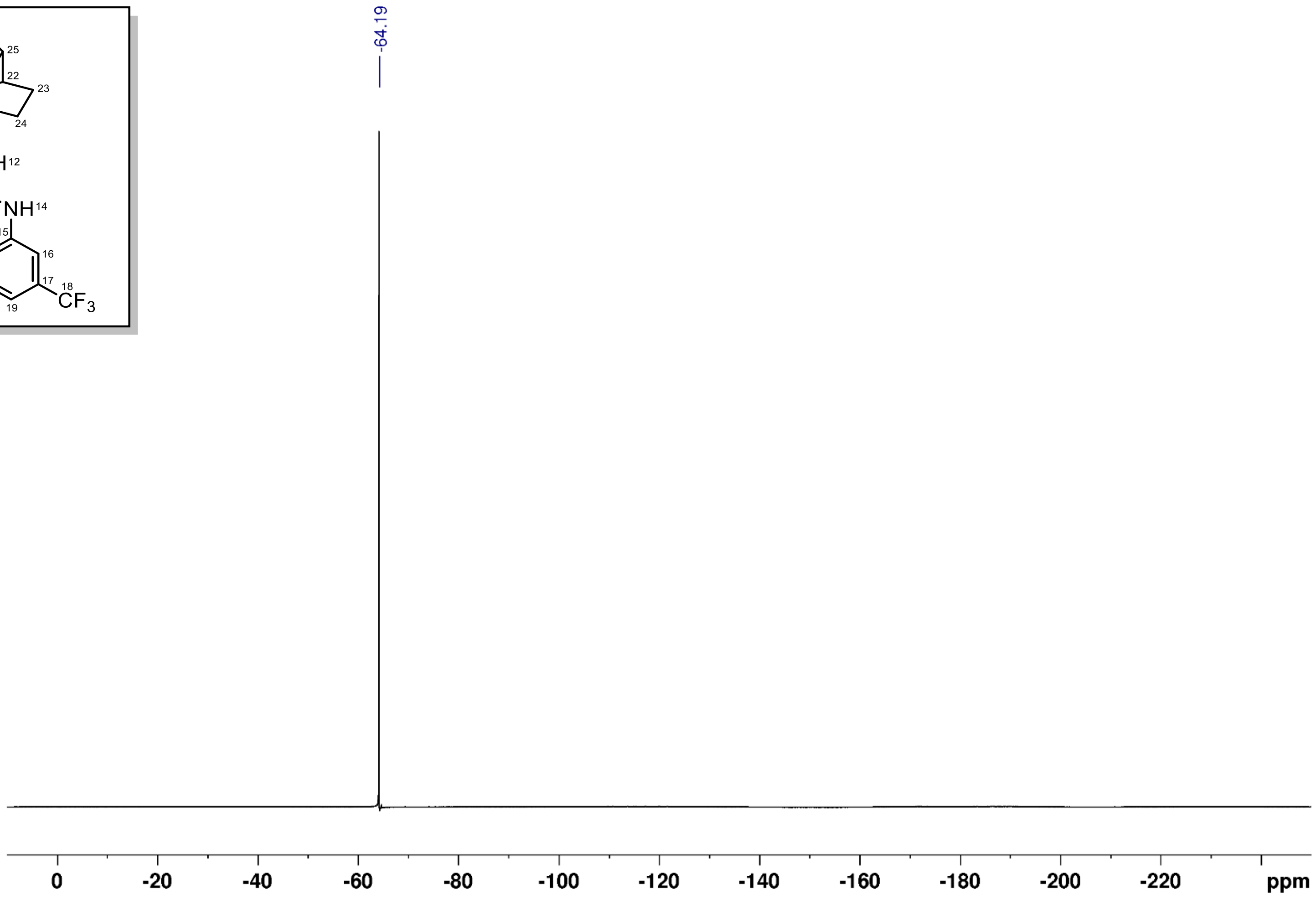

^{1}H NMR (700 MHz, CD_3OD) for (1*S*,2*S*,4*S*,5*R*)-2-((*S*)-(3-(3,5-bis(trifluoromethyl)phenyl)ureido)(6-methoxyquinolin-4-yl)methyl)-1-(3,5-di-*tert*-butylbenzyl)-5-ethylquinuclidin-1-ium bromide (***epi*-C4**•Br)

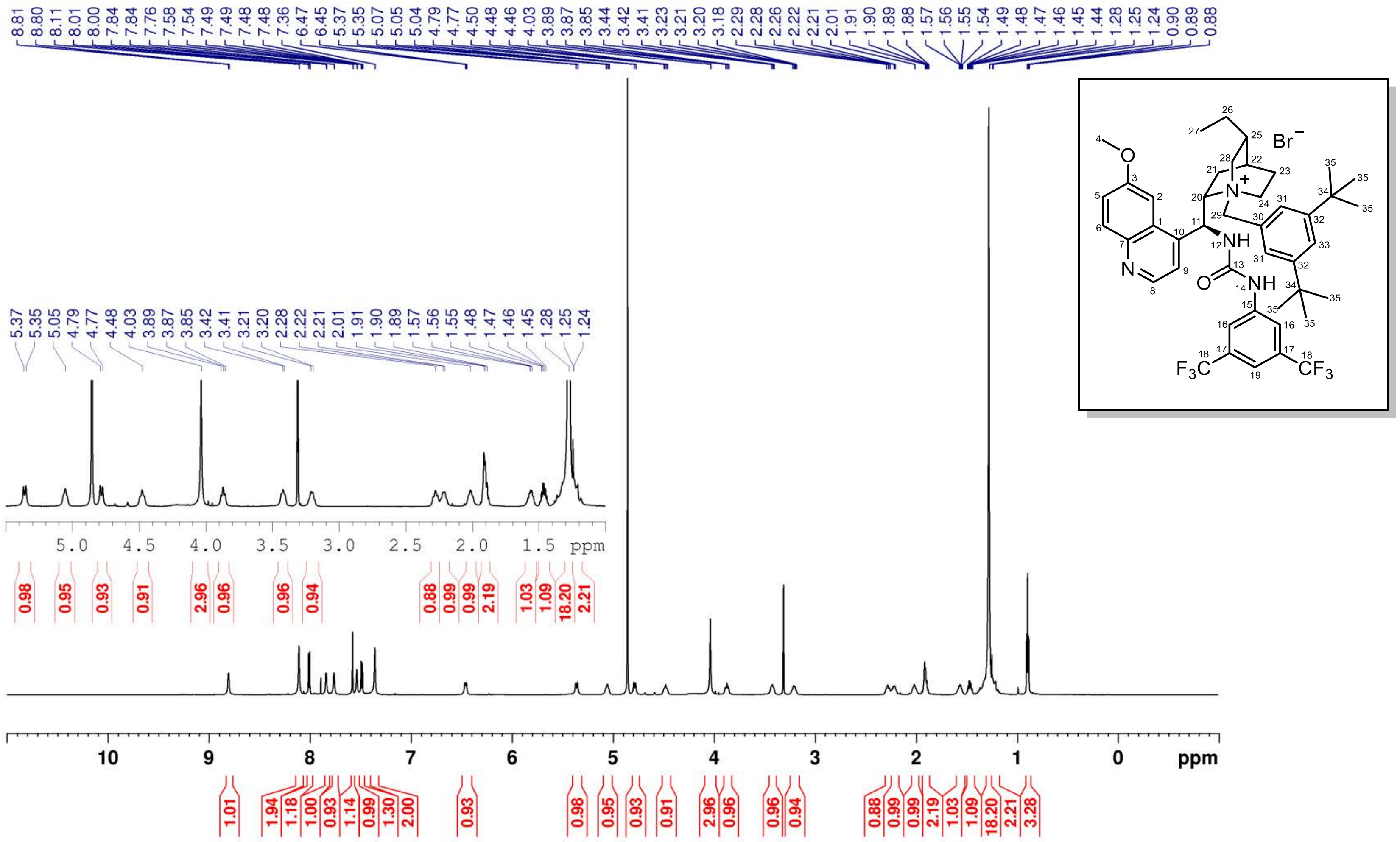

^{13}C NMR (176 MHz, CD_3OD) for (1*S*,2*S*,4*S*,5*R*)-2-((*S*)-(3-(3,5-bis(trifluoromethyl)phenyl)ureido)(6-methoxyquinolin-4-yl)methyl)-1-(3,5-di-*tert*-butylbenzyl)-5-ethylquinuclidin-1-ium bromide (***epi*-C4**•Br)

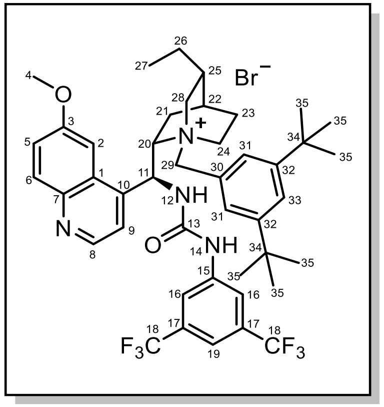

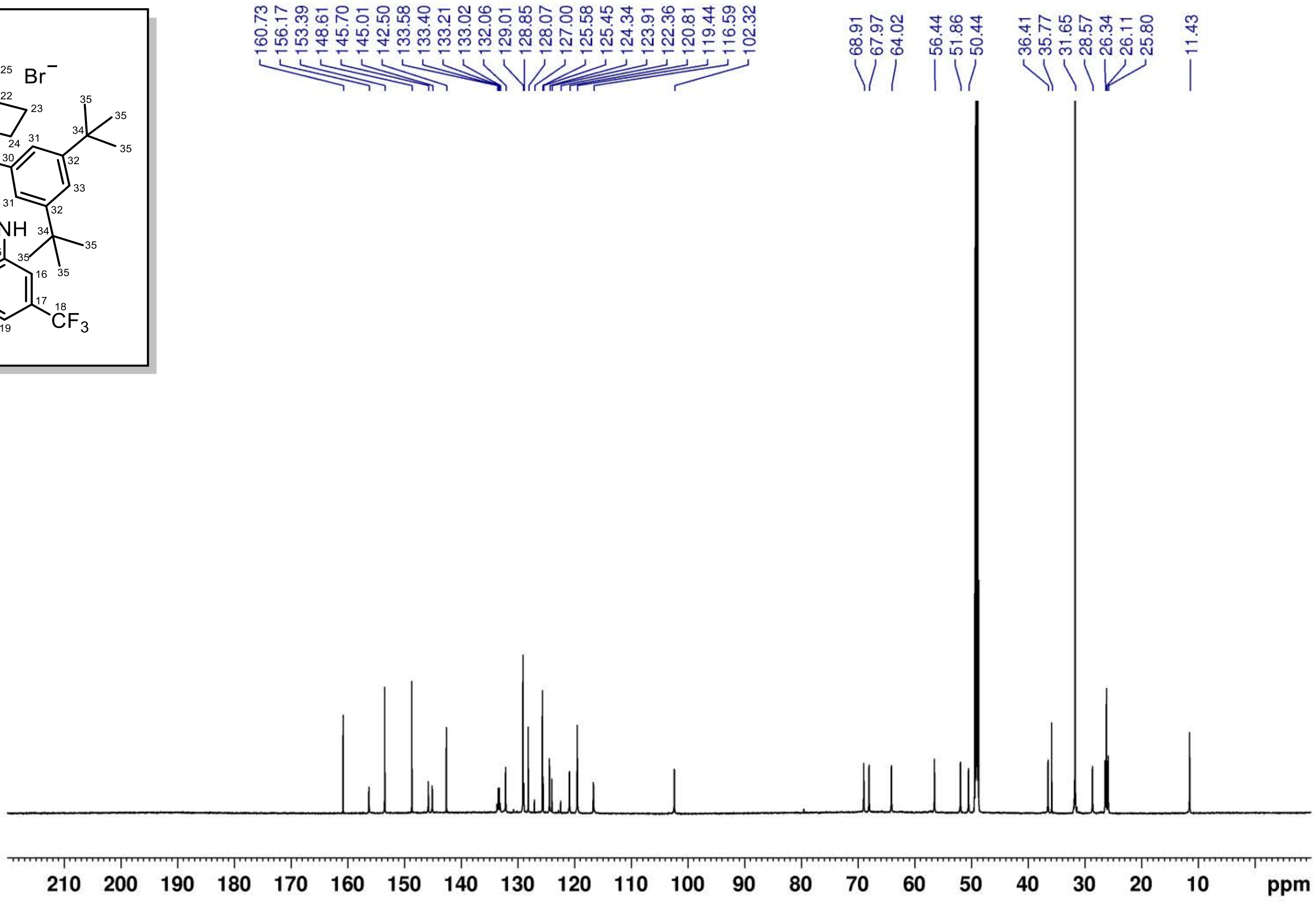

^{19}F NMR (376 MHz, CD_3OD) for (1*S*,2*S*,4*S*,5*R*)-2-((*S*)-(3-(3,5-bis(trifluoromethyl)phenyl)ureido)(6-methoxyquinolin-4-yl)methyl)-1-(3,5-di-*tert*-butylbenzyl)-5-ethylquinuclidin-1-ium bromide (***epi*-C4**•Br)

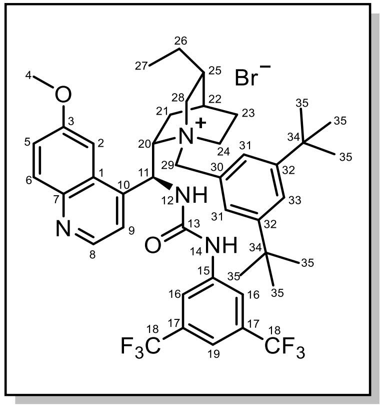

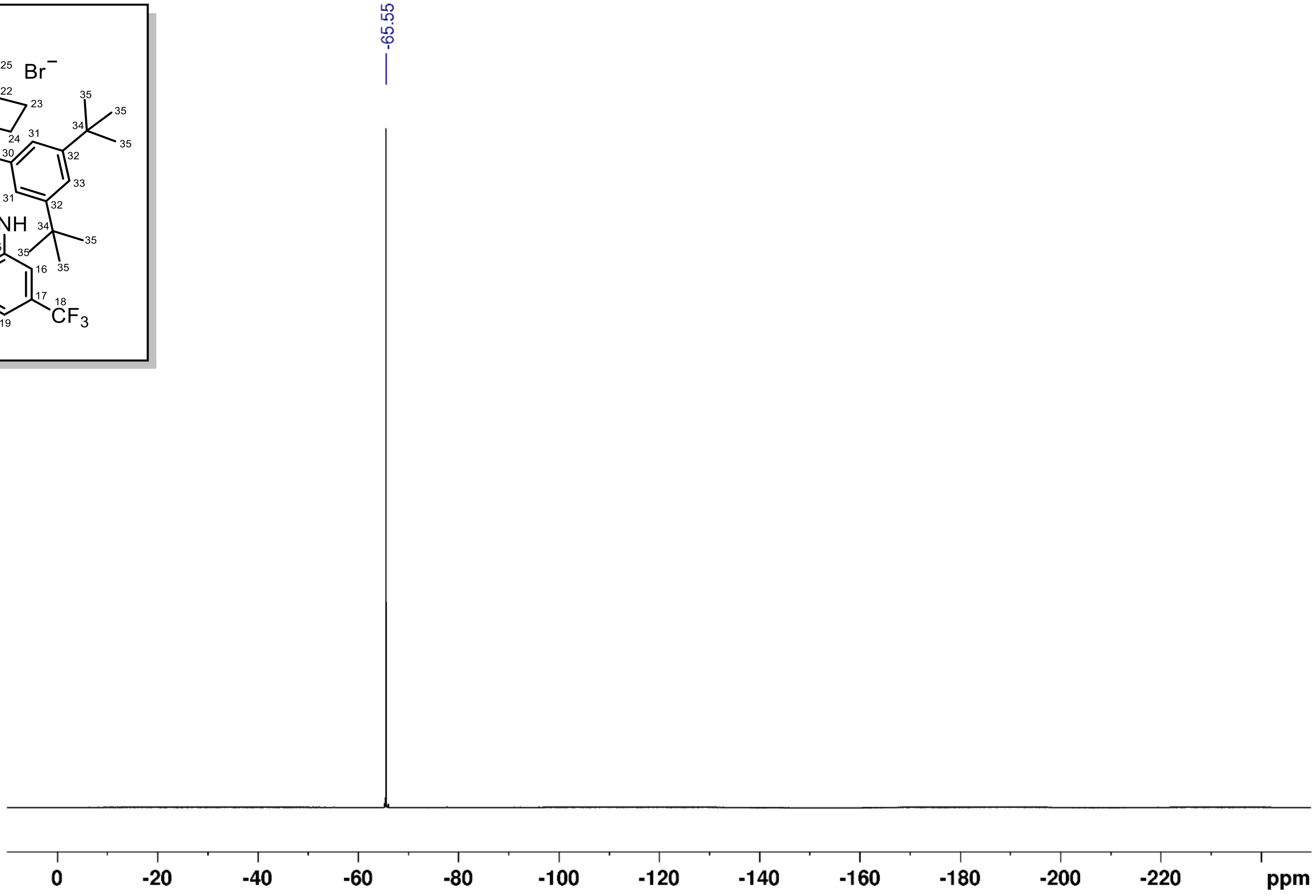

^{1}H NMR (700 MHz, $CDCl_3$) for 1-cyclohexyl-3-((*S*)-((1*S*,2*S*,4*S*,5*R*)-5-ethylquinuclidin-2-yl)(6-methoxyquinolin-4-yl)methyl)urea

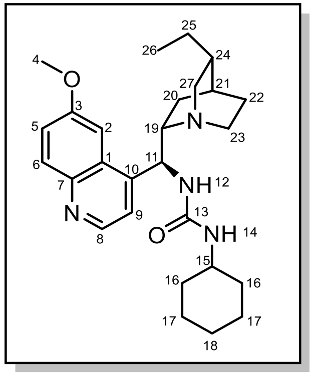

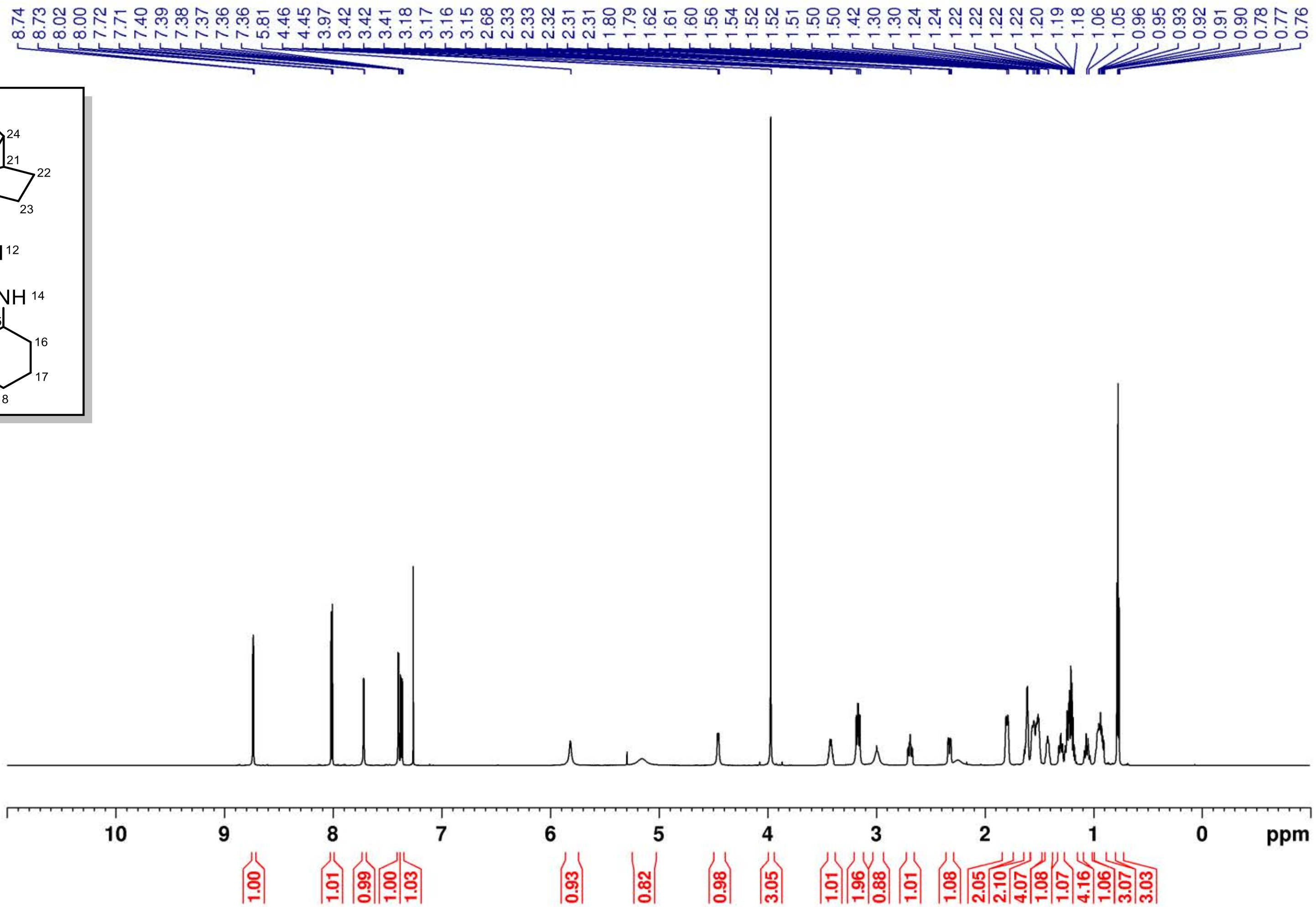

^{13}C NMR (176 MHz, CDCl$_3$) for 1-cyclohexyl-3-((*S*)-((1*S*,2*S*,4*S*,5*R*)-5-ethylquinuclidin-2-yl)(6-methoxyquinolin-4-yl)methyl)urea

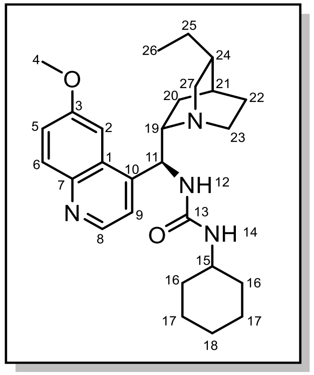

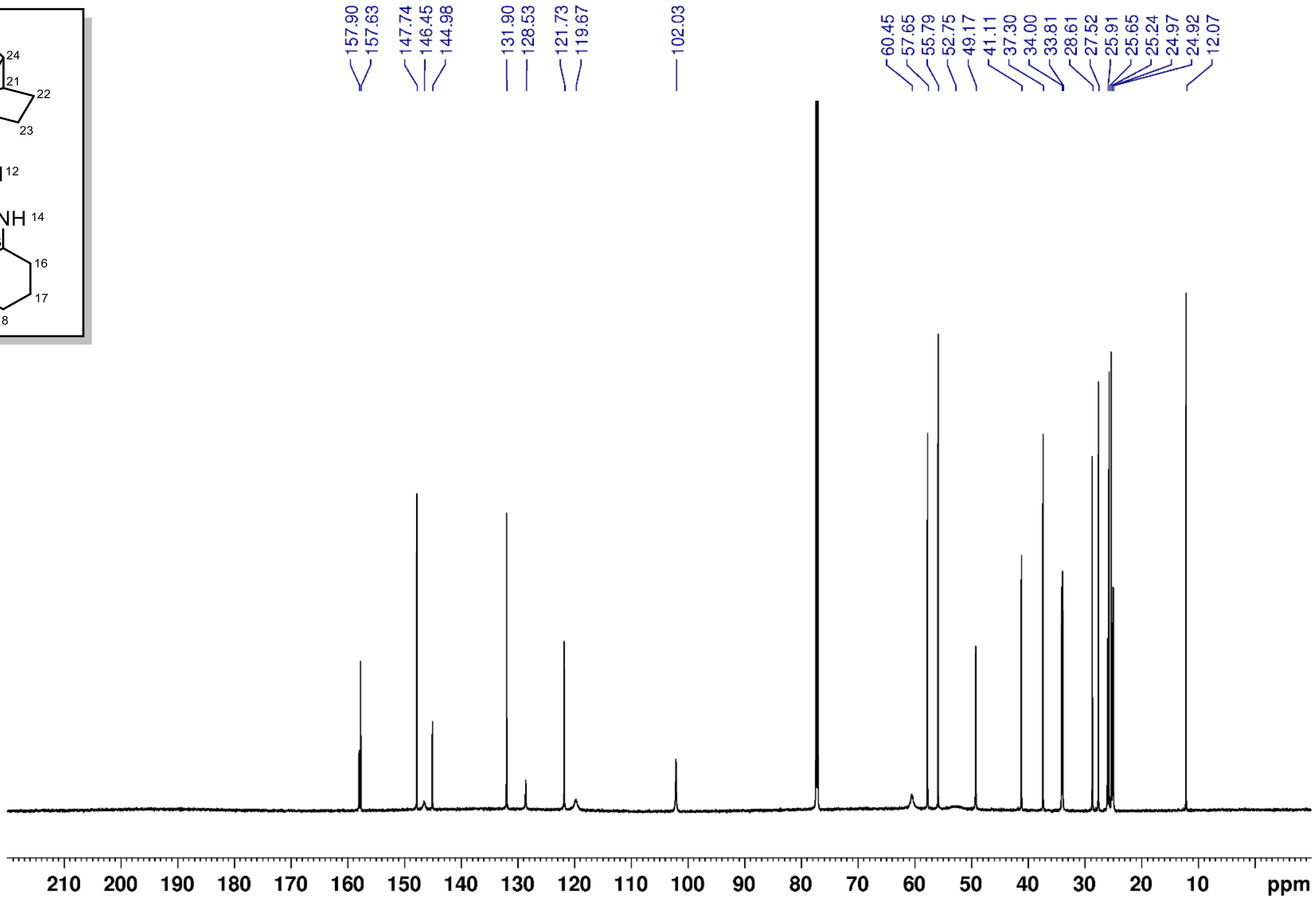

^{1}H NMR (700 MHz, CD_3OD) for (1*S*,2*S*,4*S*,5*R*)-2-((*S*)-(3-cyclohexylureido)(6-methoxyquinolin-4-yl)methyl)-5-ethyl-1-((3,3'',5,5''-tetra-*tert*-butyl-[1,1':3',1''-terphenyl]-5'-yl)methyl)quinuclidin-1-ium bromide (***epi*-C7**•Br)

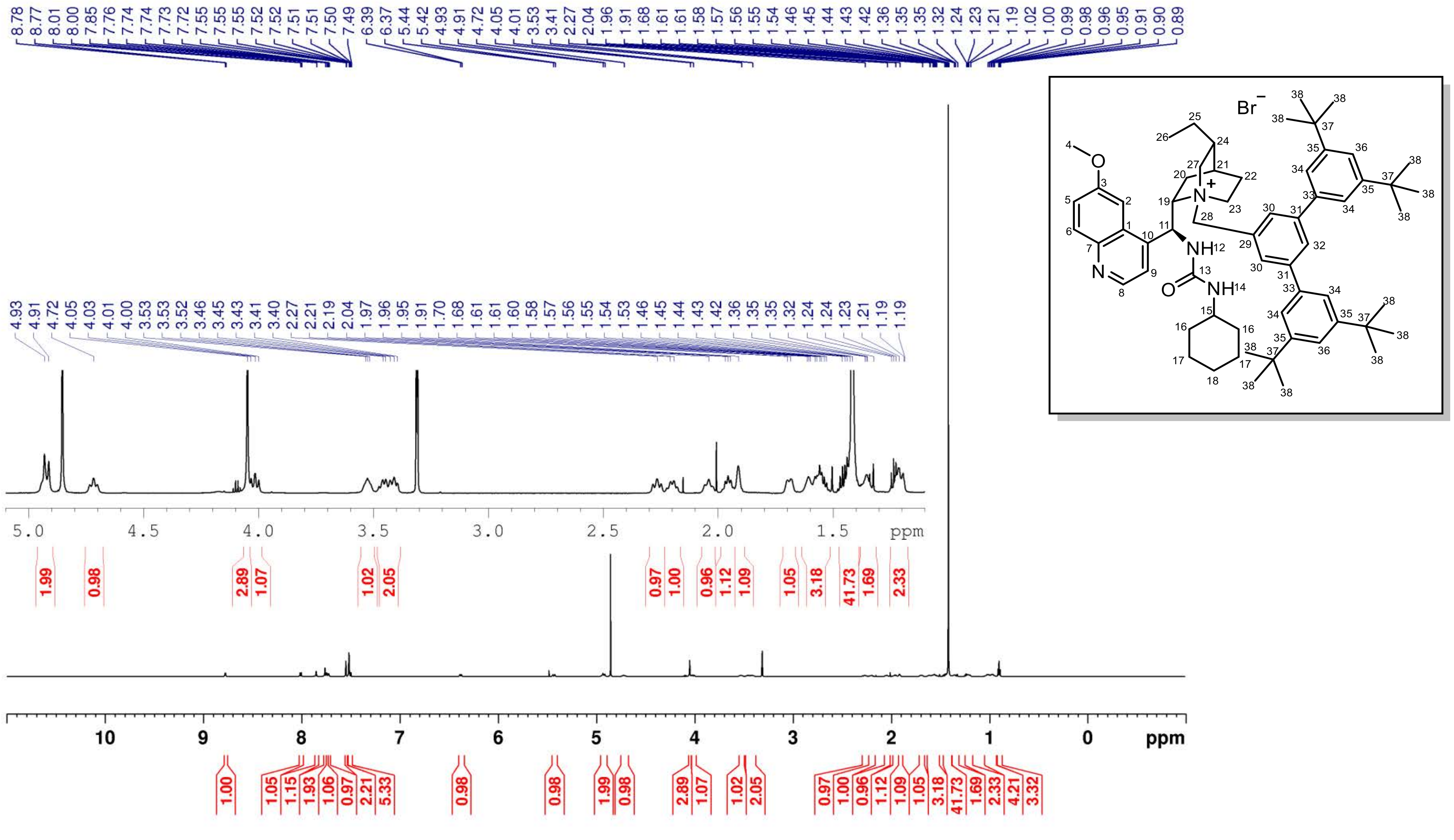

^{13}C NMR (176 MHz, CD$_3$OD) for (1*S*,2*S*,4*S*,5*R*)-2-((*S*)-(3-cyclohexylureido)(6-methoxyquinolin-4-yl)methyl)-5-ethyl-1-((3,3'',5,5''-tetra-*tert*-butyl-[1,1':3',1''-terphenyl]-5'-yl)methyl)quinuclidin-1-ium bromide (***epi*-C7**•Br)

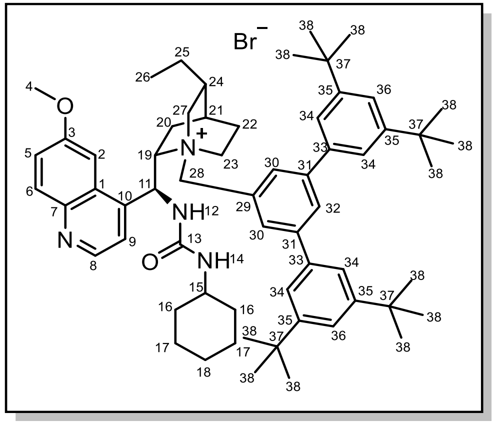

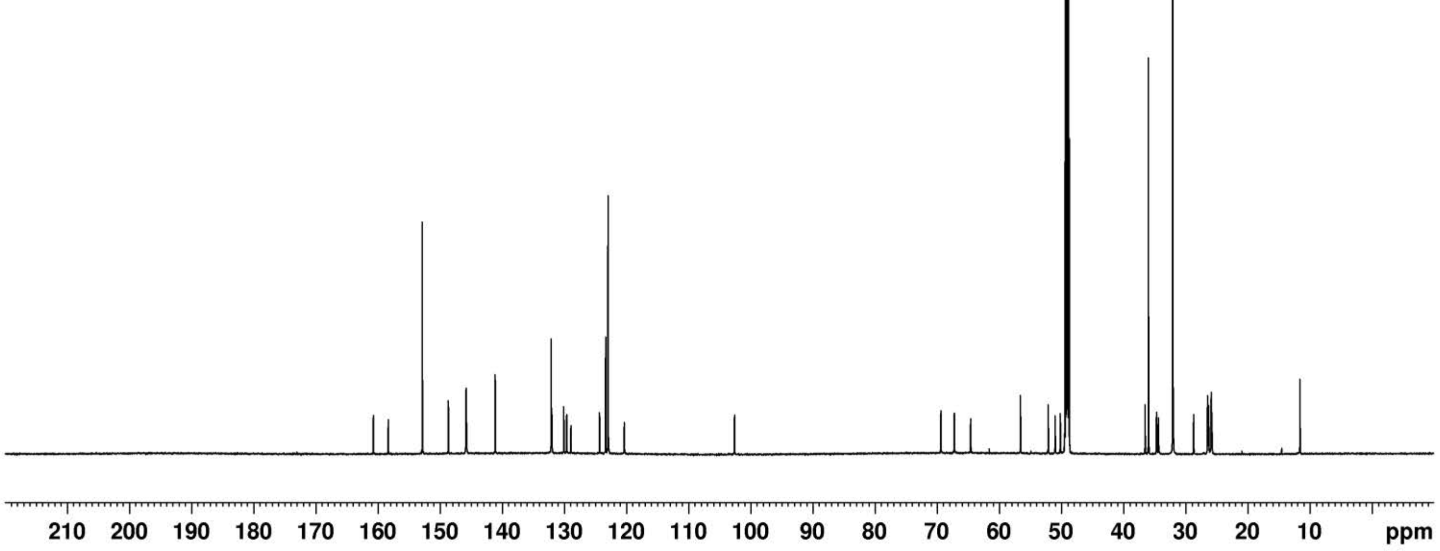

^{1}H NMR (700 MHz, CDCl$_3$) for *N*-((*R*)-((1*S*,2*S*,4*S*,5*R*)-5-ethylquinuclidin-2-yl)(6-methoxyquinolin-4-yl)methyl)acetamide

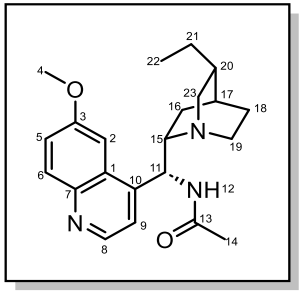

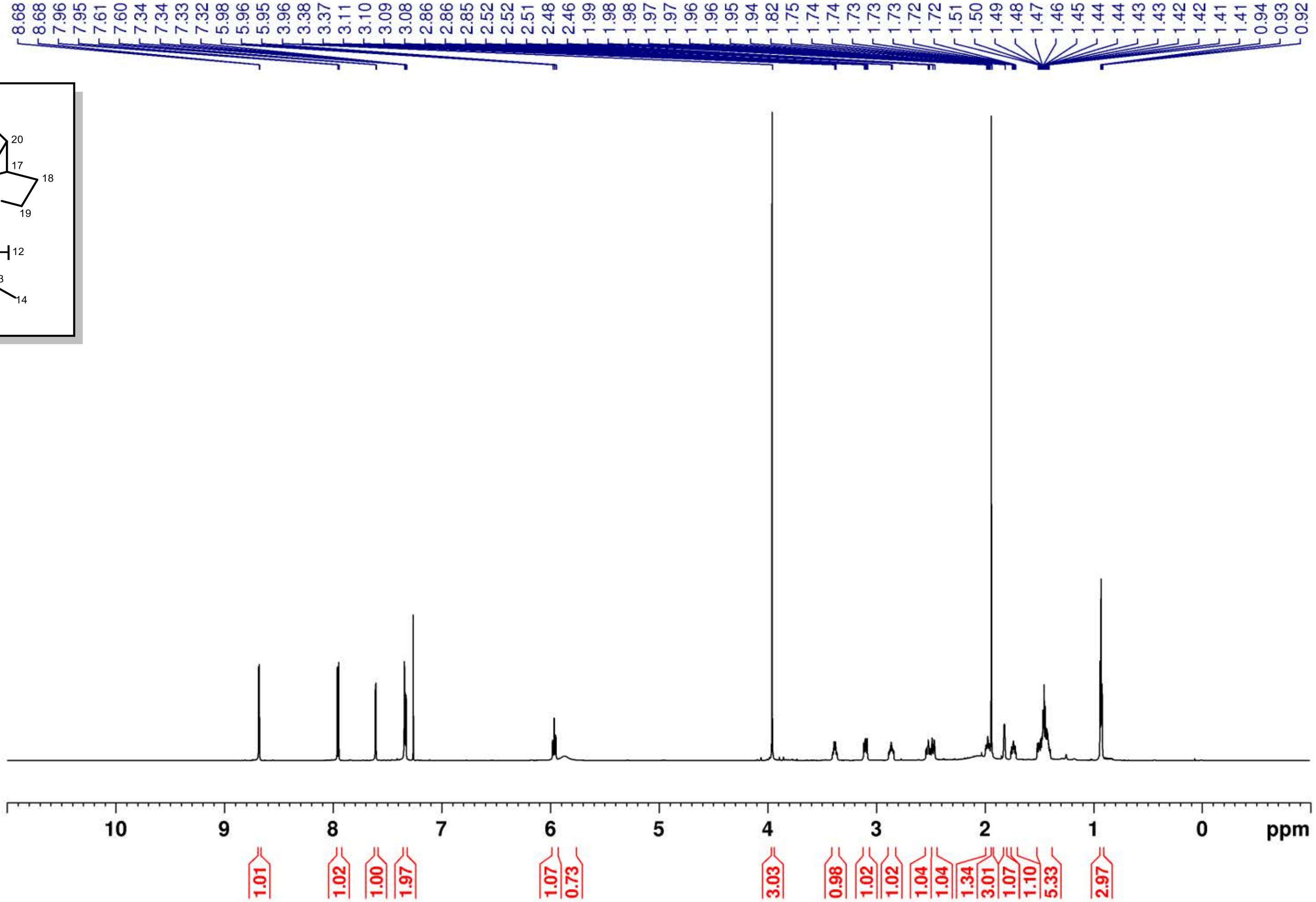

^{13}C NMR (176 MHz, CDCl$_3$) for *N*-((*R*)-((1*S*,2*S*,4*S*,5*R*)-5-ethylquinuclidin-2-yl)(6-methoxyquinolin-4-yl)methyl)acetamide

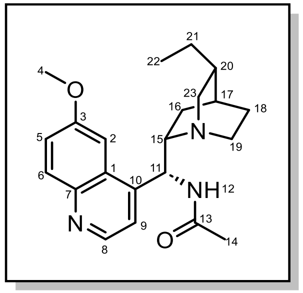

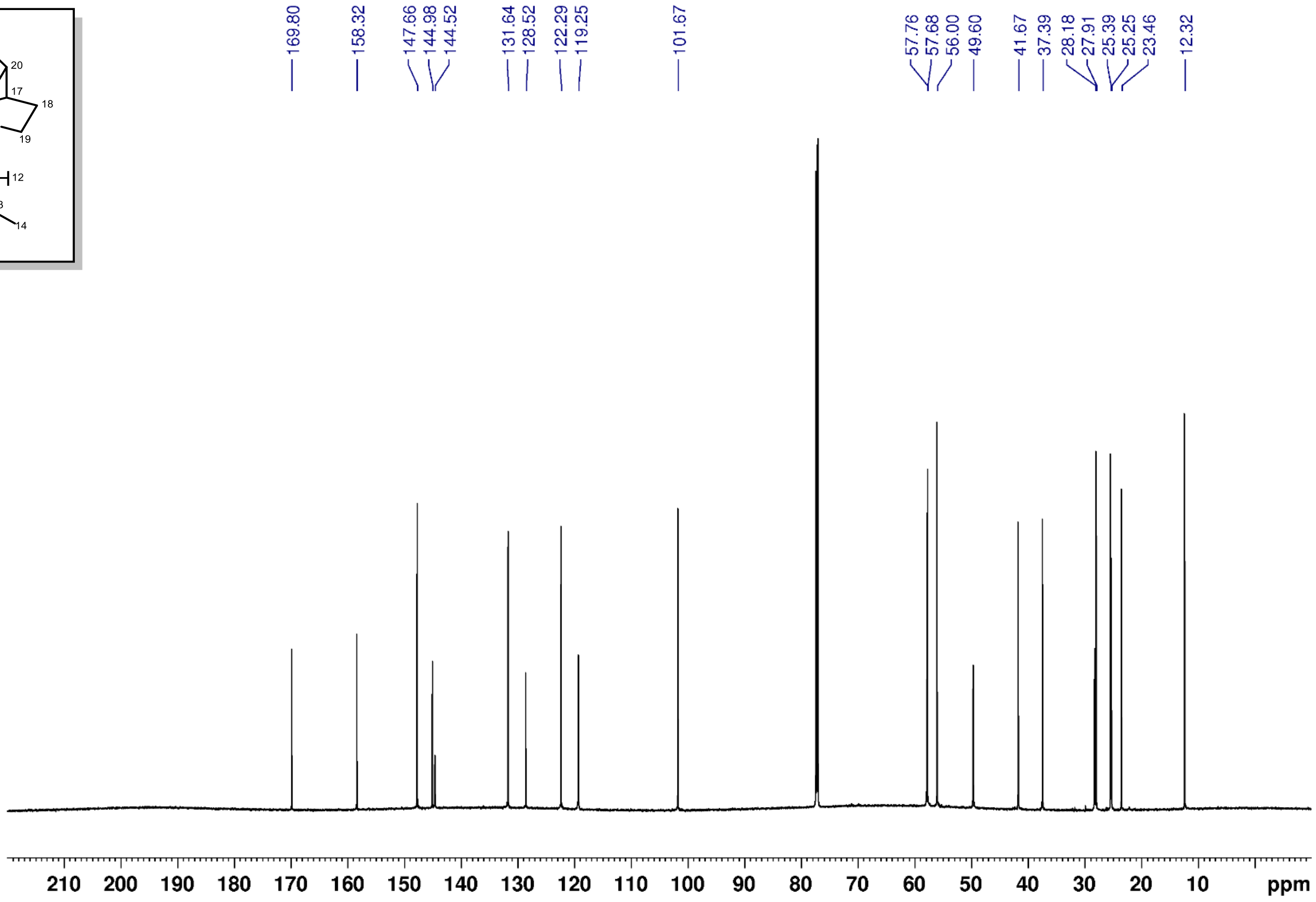

^{1}H NMR (700 MHz, CDCl$_3$) for (1*S*,2*S*,4*S*,5*R*)-2-((*R*)-acetamido(6-methoxyquinolin-4-yl)methyl)-5-ethyl-1-((3,3'',5,5''-tetra-*tert*-butyl-[1,1':3',1''-terphenyl]-5'-yl)methyl)quinuclidin-1-ium bromide (**C8**•Br)

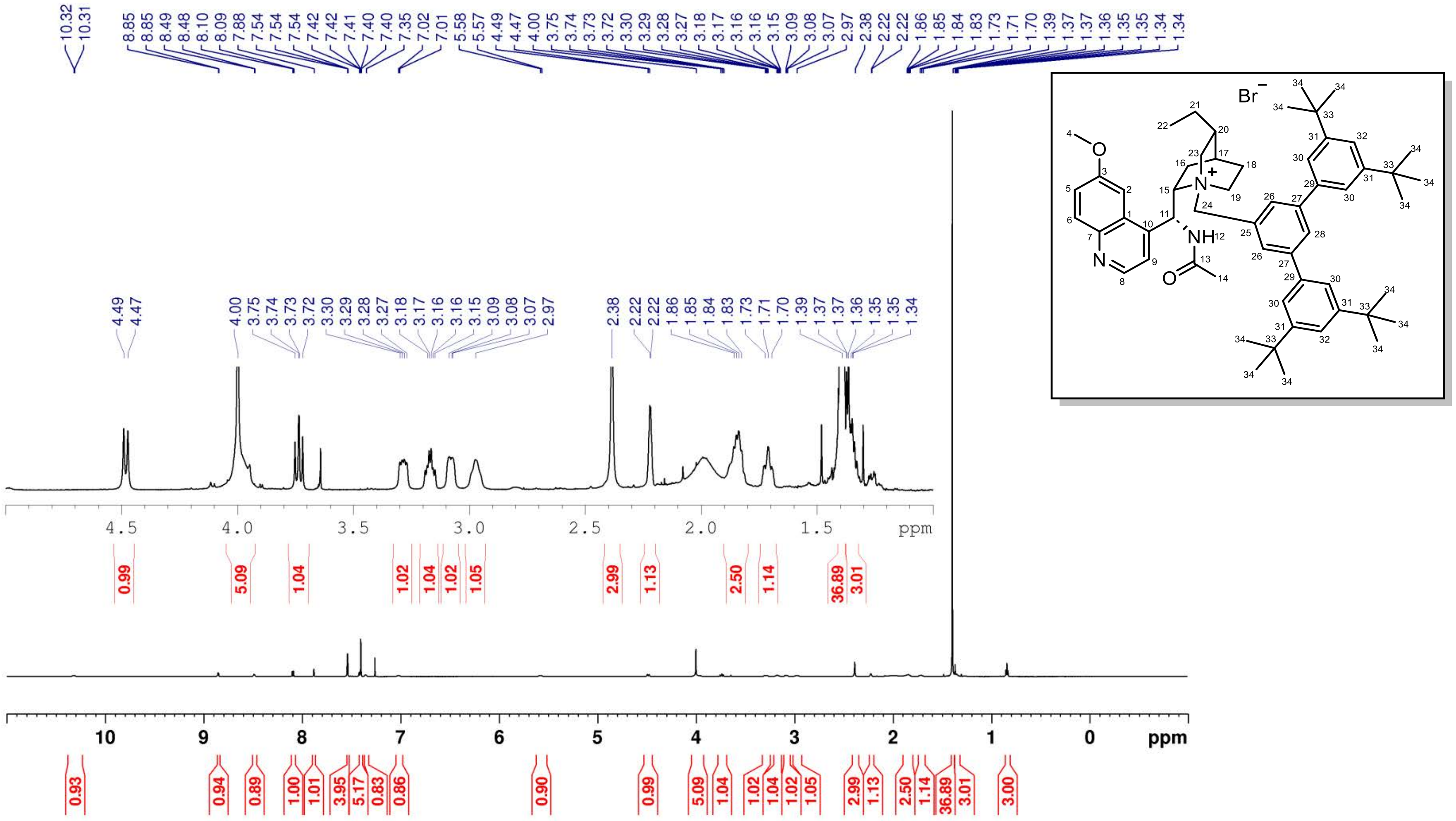

^{13}C NMR (176 MHz, $CDCl_3$) for (1*S*,2*S*,4*S*,5*R*)-2-((*R*)-acetamido(6-methoxyquinolin-4-yl)methyl)-5-ethyl-1-((3,3'',5,5''-tetra-*tert*-butyl-[1,1':3',1''-terphenyl]-5'-yl)methyl)quinuclidin-1-ium bromide (**C8**•Br)

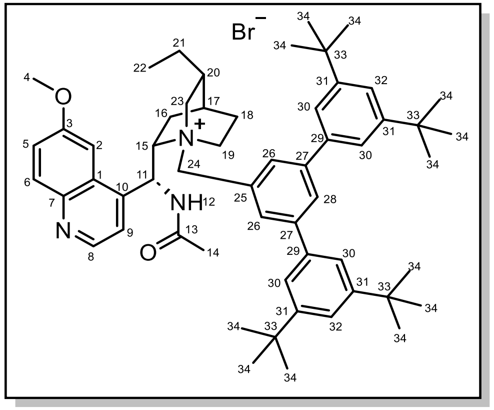

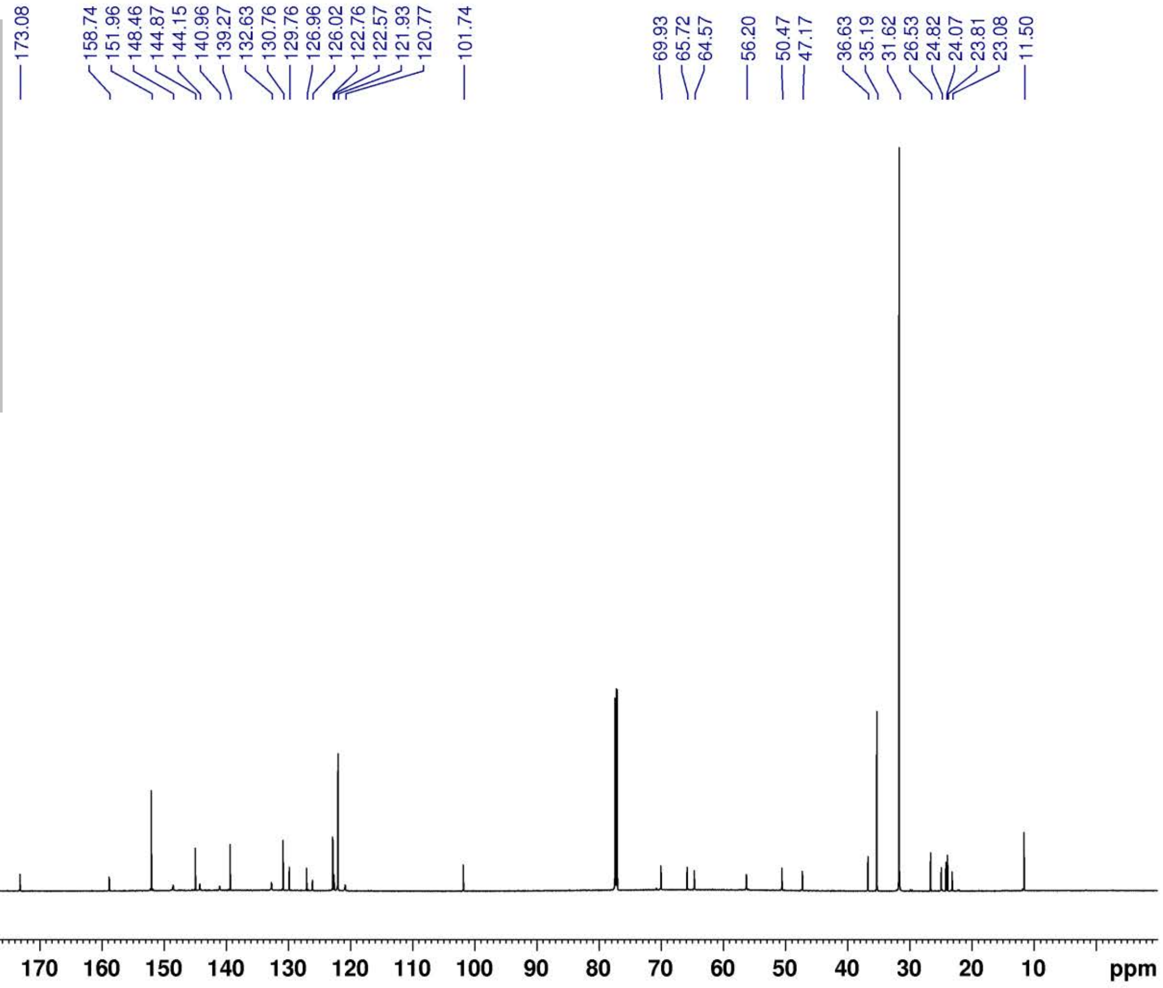

^{1}H NMR (700 MHz, CDCl$_3$) for (*R*)-[1,1'-binaphthalene]-2,2'-diyl bis(trifluoromethanesulfonate)

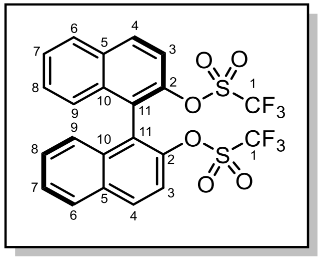

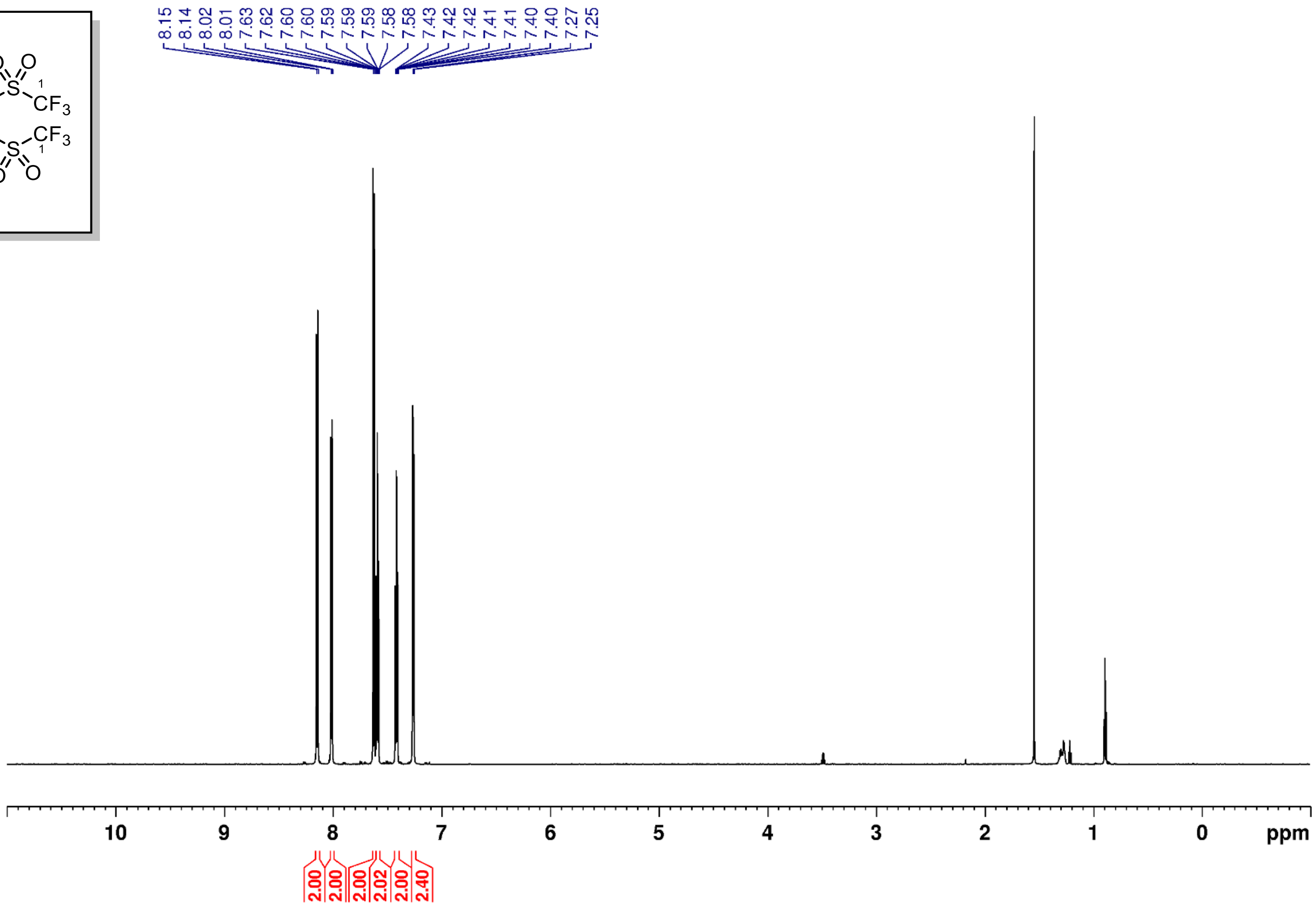

^{13}**C NMR (176 MHz, $CDCl_3$)** for (*R*)-[1,1'-binaphthalene]-2,2'-diyl bis(trifluoromethanesulfonate)

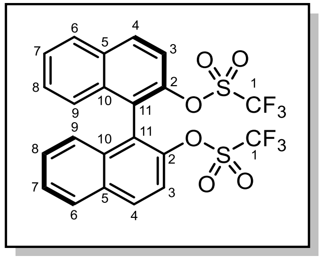

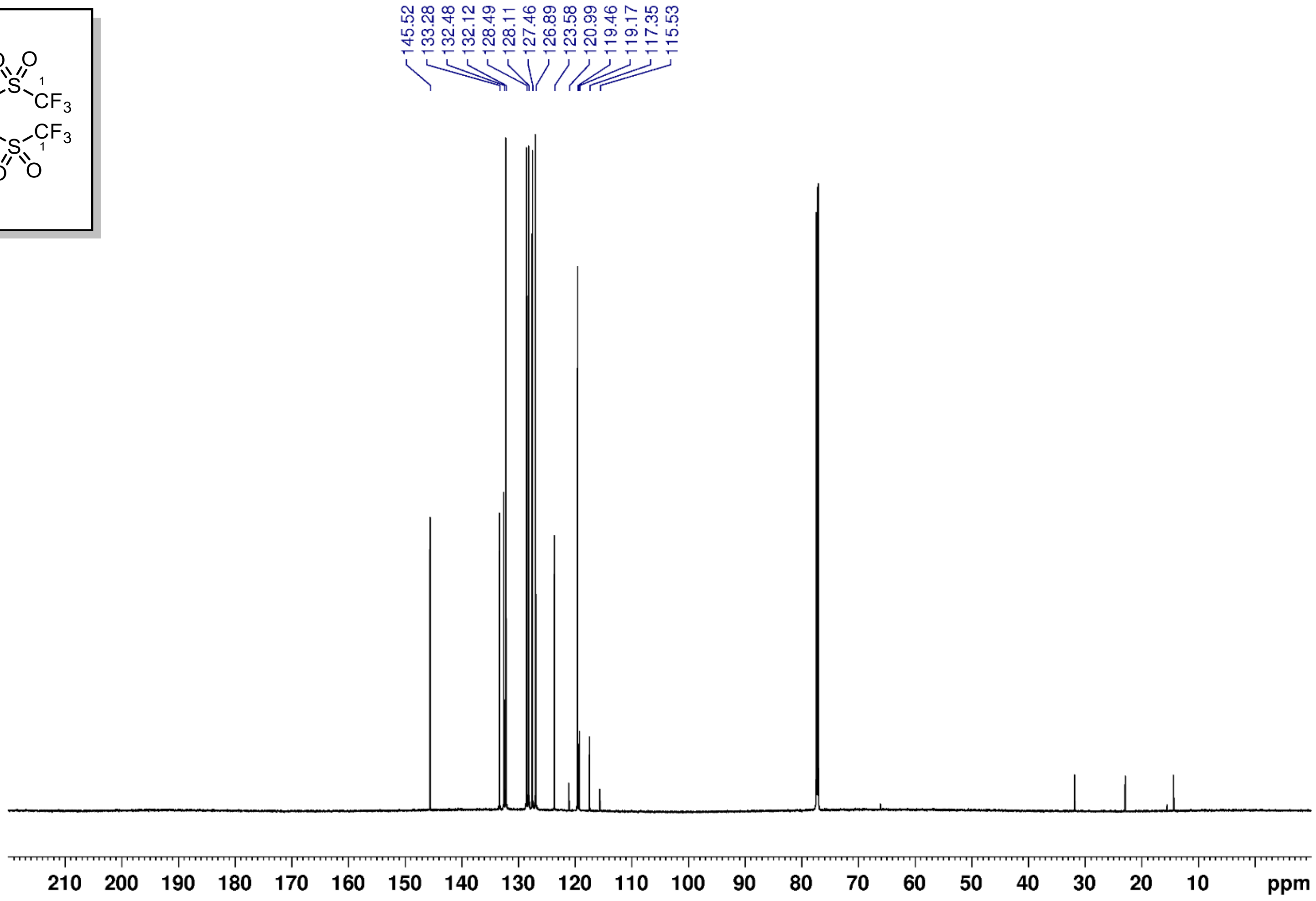

^{19}**F NMR (376 MHz, $CDCl_3$)** for (*R*)-[1,1'-binaphthalene]-2,2'-diyl bis(trifluoromethanesulfonate)

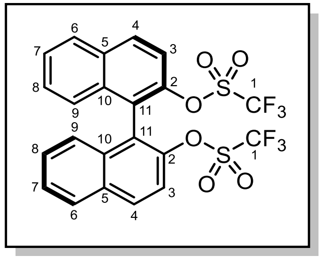

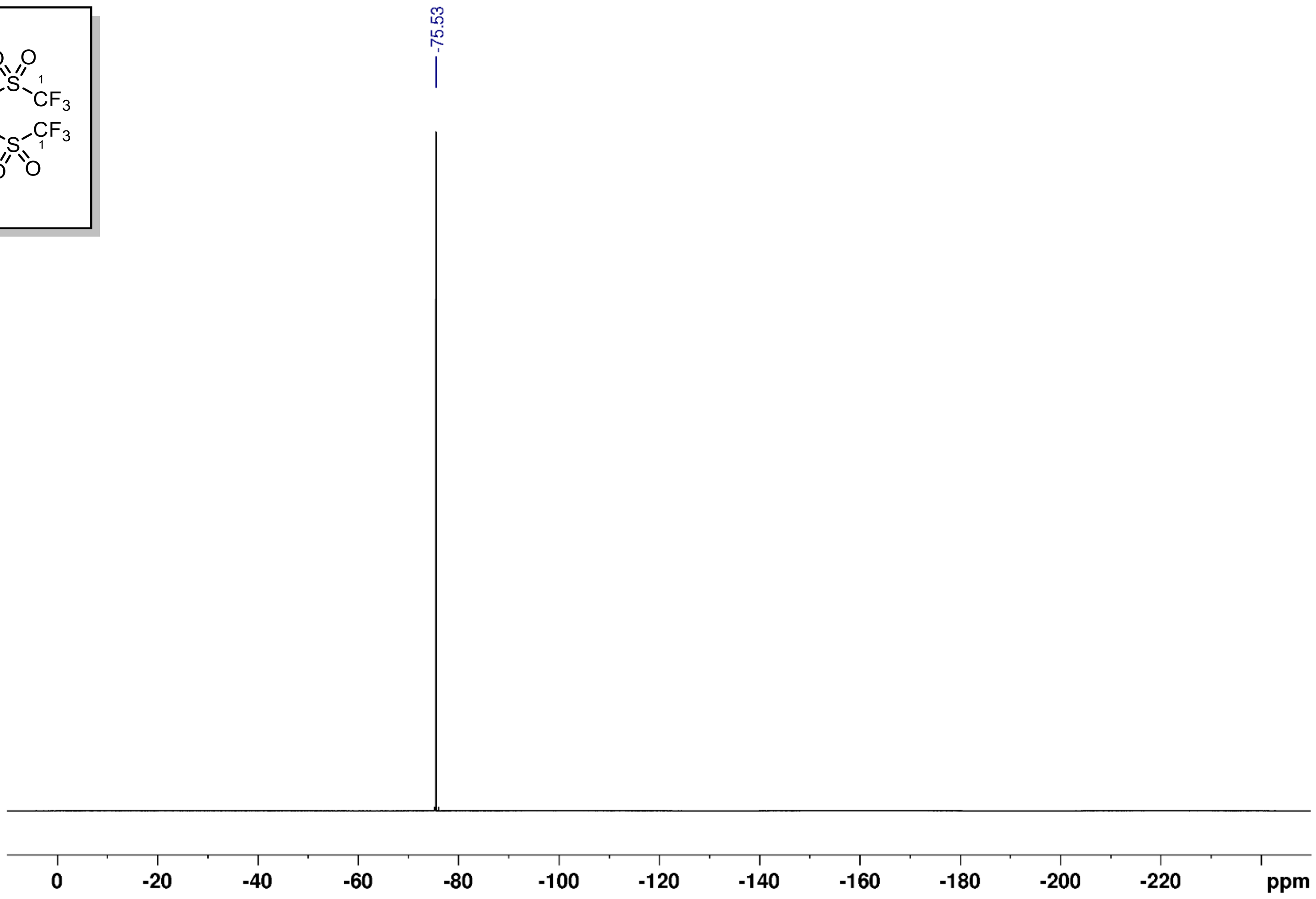

^{1}H NMR (700 MHz, $CDCl_3$) for (*R*)-2'-(diphenylphosphoryl)-[1,1'-binaphthalen]-2-yl trifluoromethanesulfonate

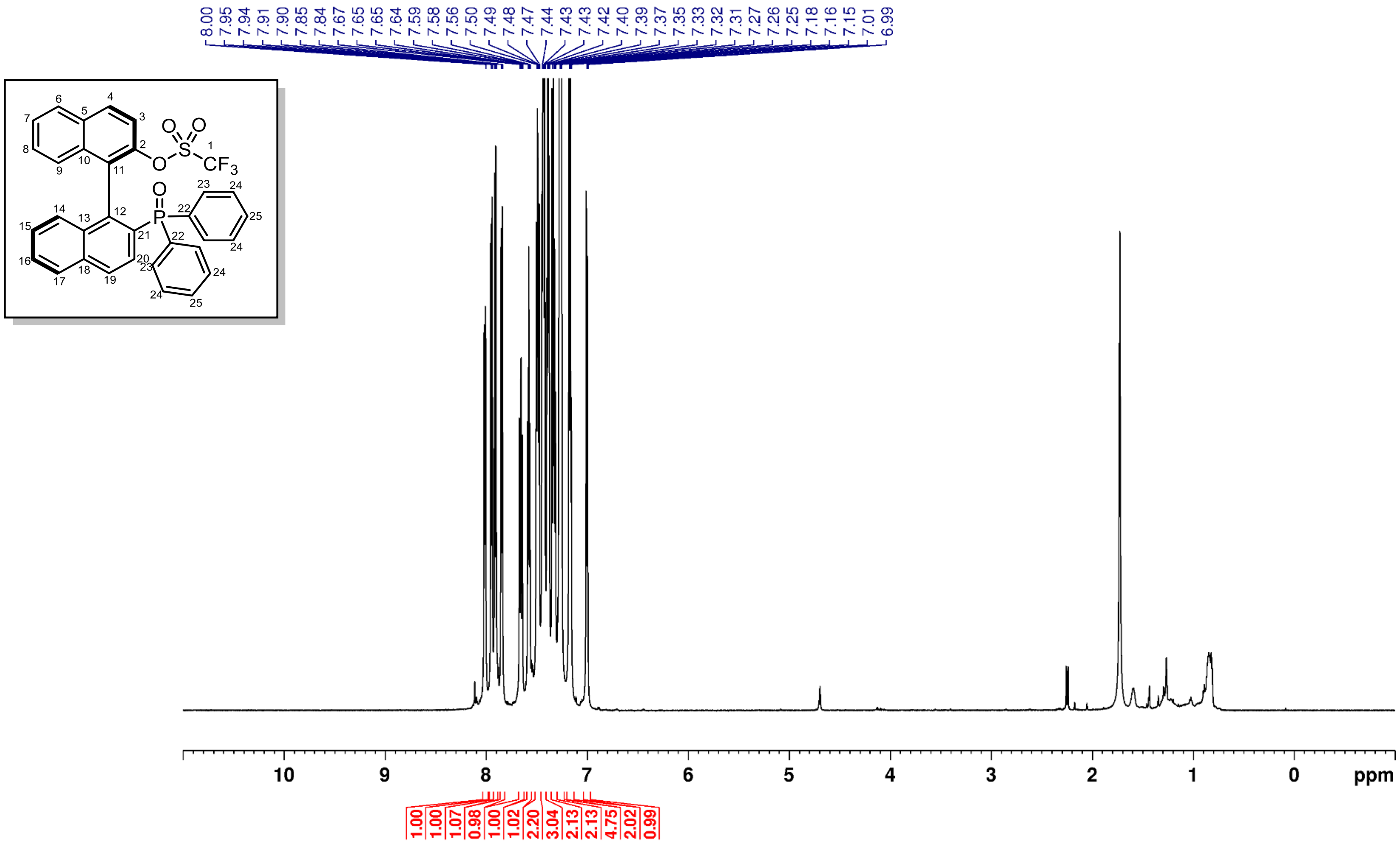

^{13}C NMR (176 MHz, CDCl$_3$) for (*R*)-2'-(diphenylphosphoryl)-[1,1'-binaphthalen]-2-yl trifluoromethanesulfonate

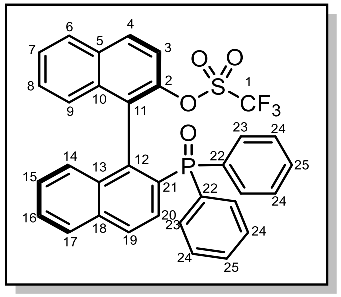

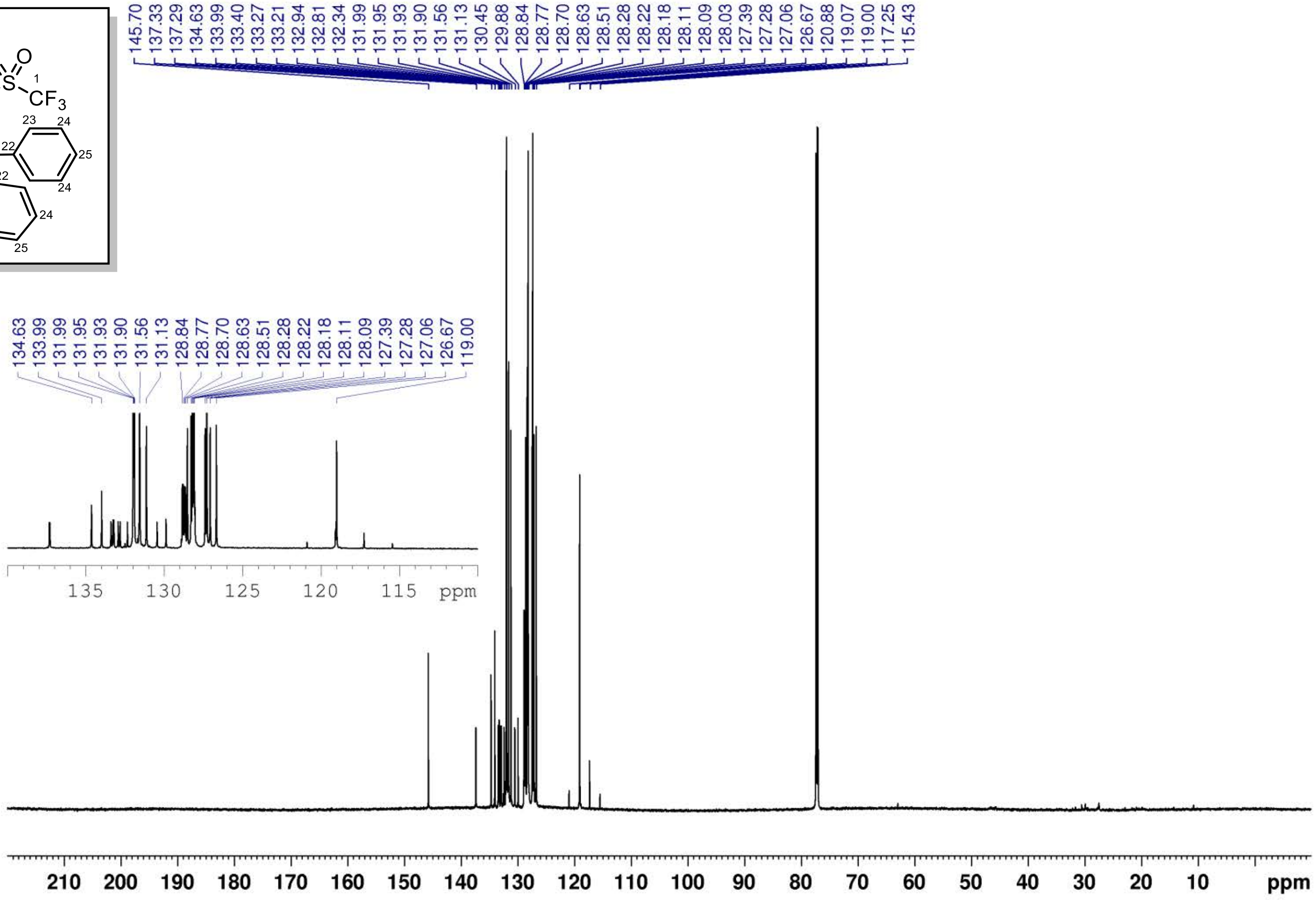

^{19}F NMR (376 MHz, $CDCl_3$) for (*R*)-2'-(diphenylphosphoryl)-[1,1'-binaphthalen]-2-yl trifluoromethanesulfonate

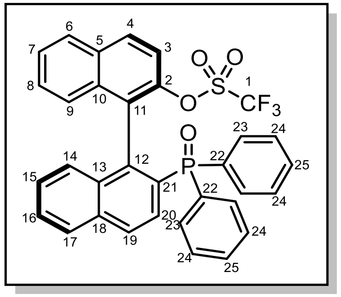

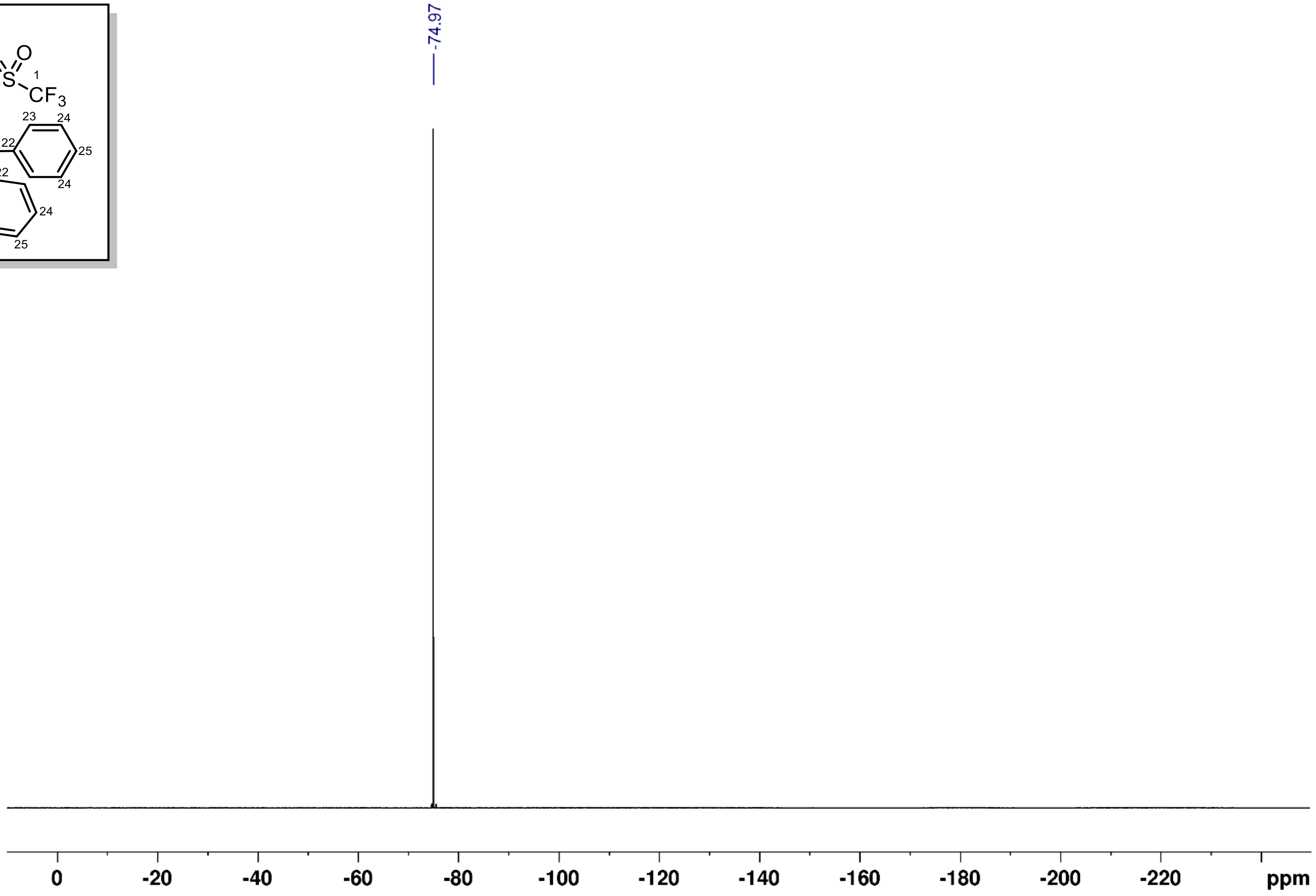

^{31}P NMR (162 MHz, CDCl$_3$) for (*R*)-2'-(diphenylphosphoryl)-[1,1'-binaphthalen]-2-yl trifluoromethanesulfonate

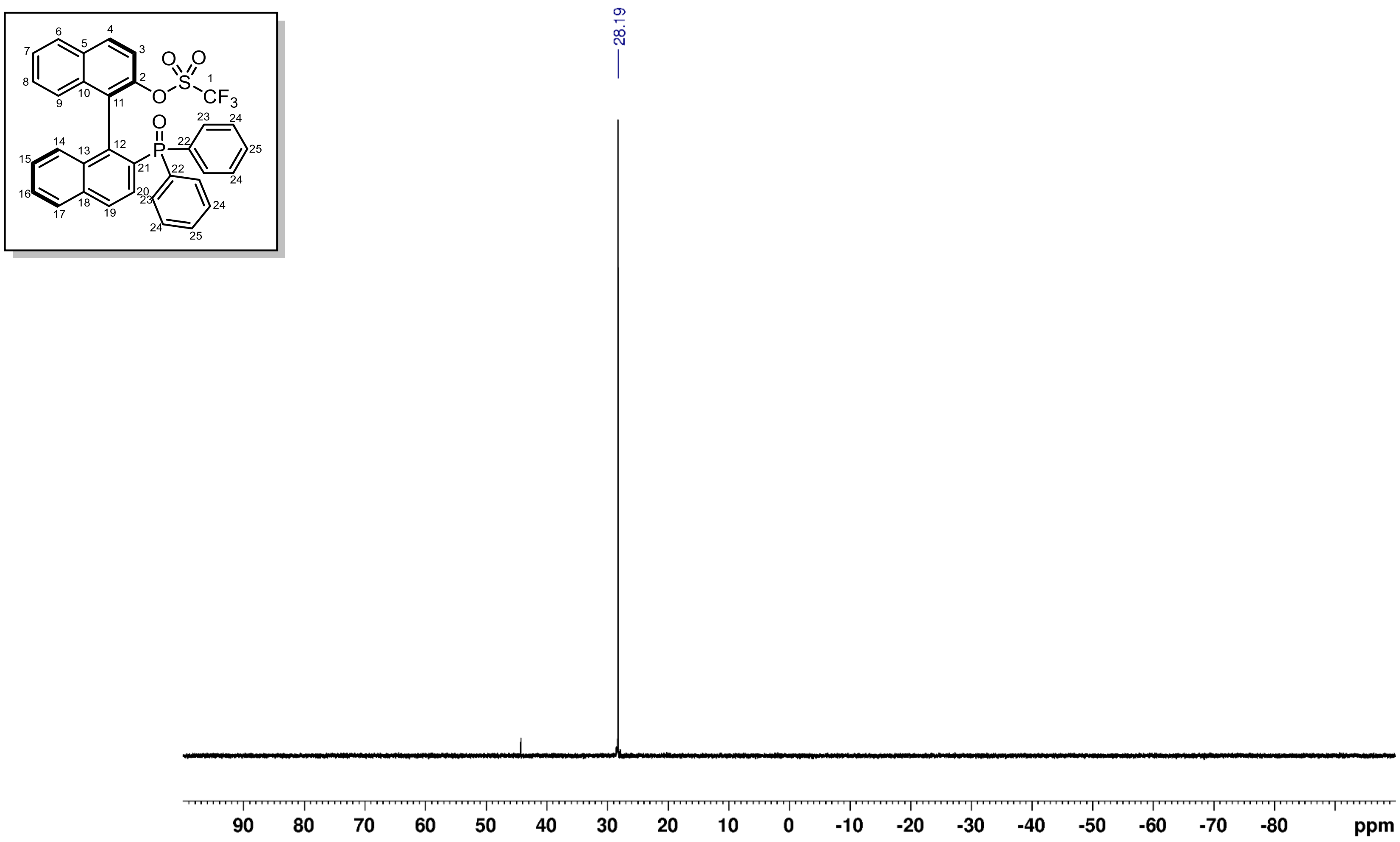

^{1}H NMR (700 MHz, CDCl$_3$) for (*R*)-(2'-hydroxy-[1,1'-binaphthalen]-2-yl)diphenylphosphine oxide

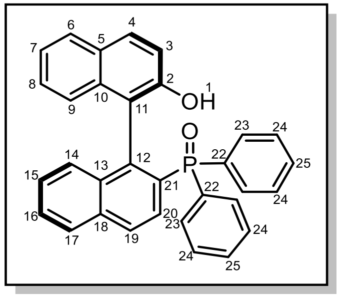

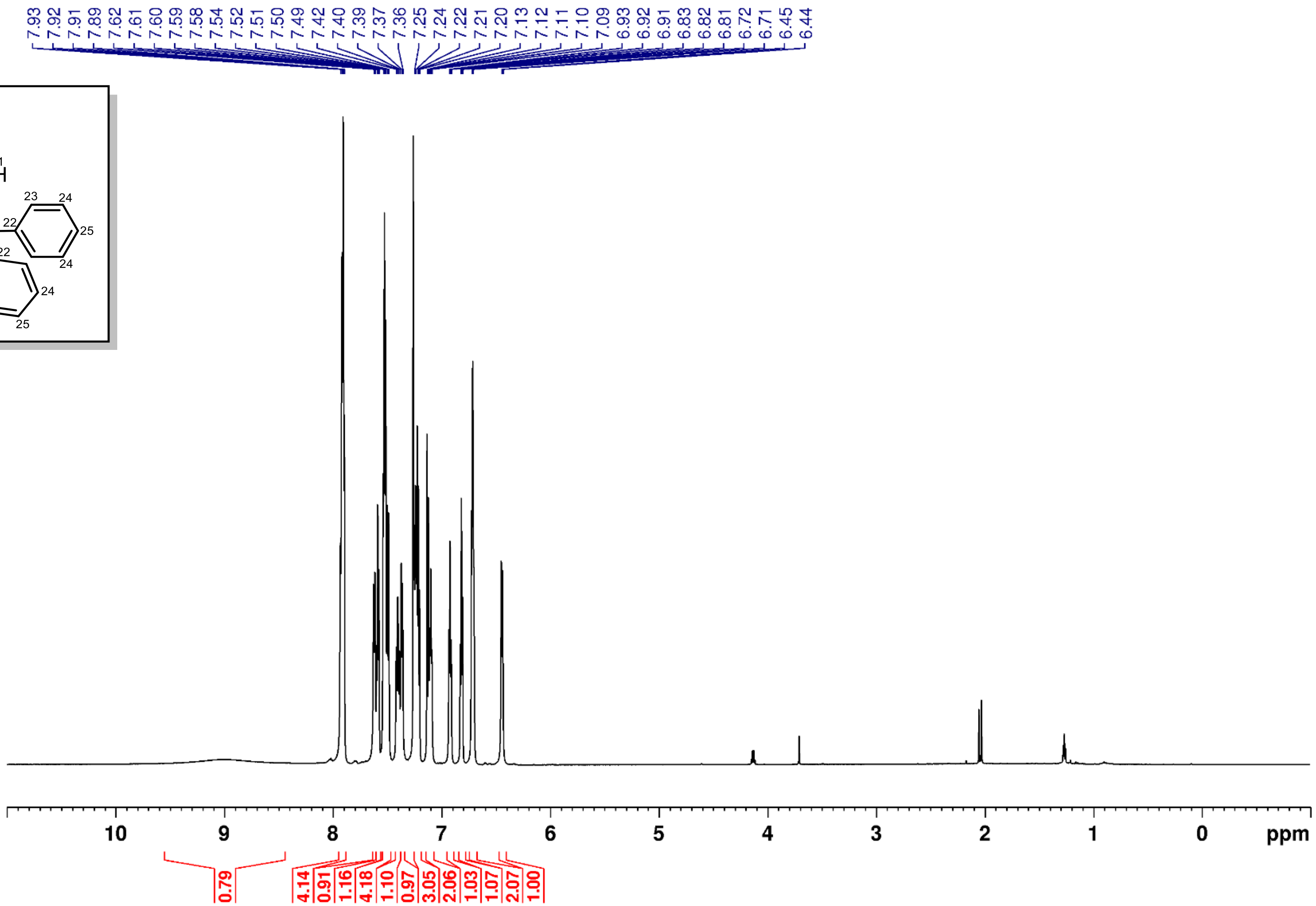

^{13}C NMR (176 MHz, $CDCl_3$) for (*R*)-(2'-hydroxy-[1,1'-binaphthalen]-2-yl)diphenylphosphine oxide

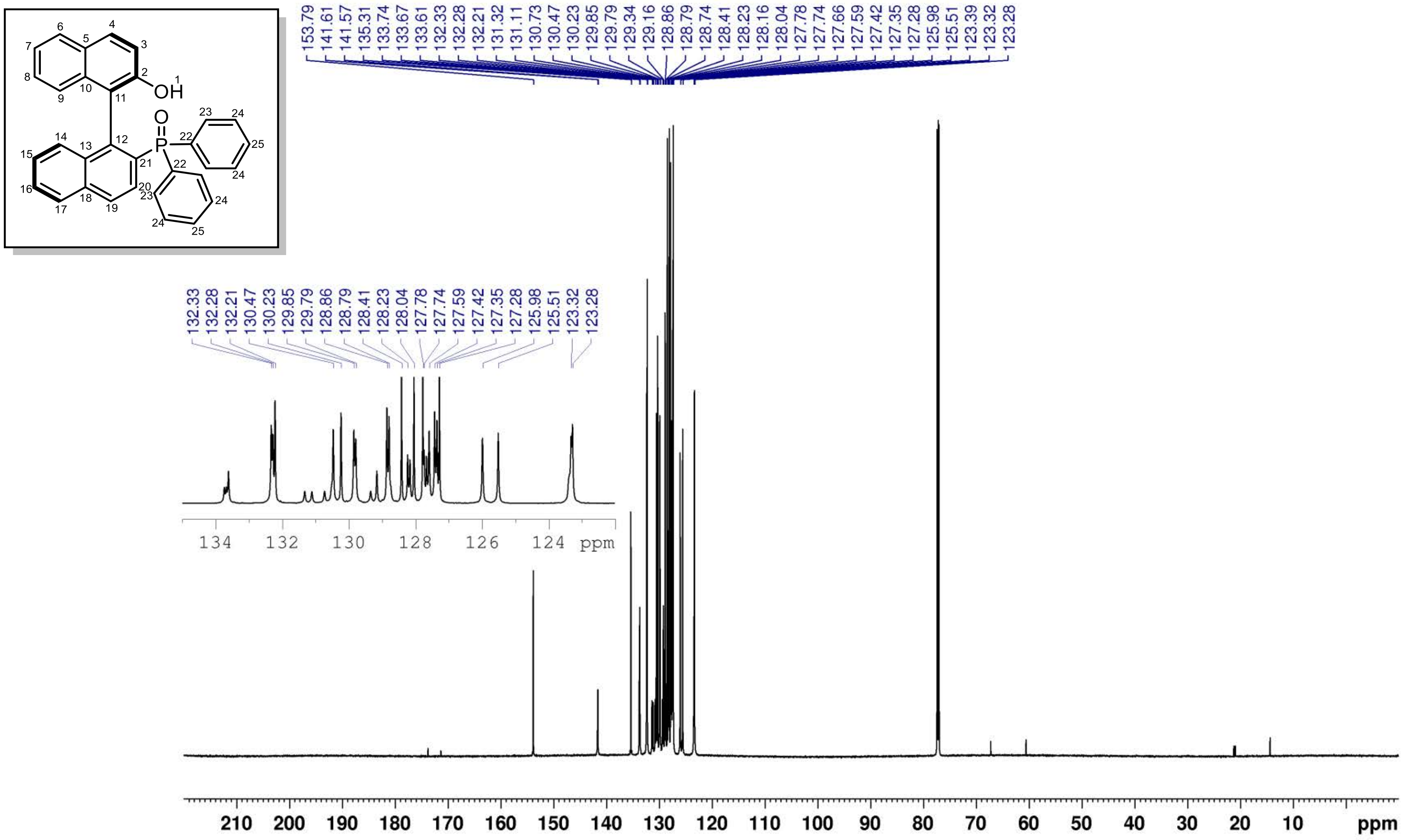

^{31}P NMR (162 MHz, CDCl$_3$) for (*R*)-(2'-hydroxy-[1,1'-binaphthalen]-2-yl)diphenylphosphine oxide

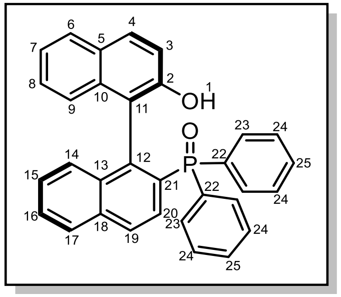

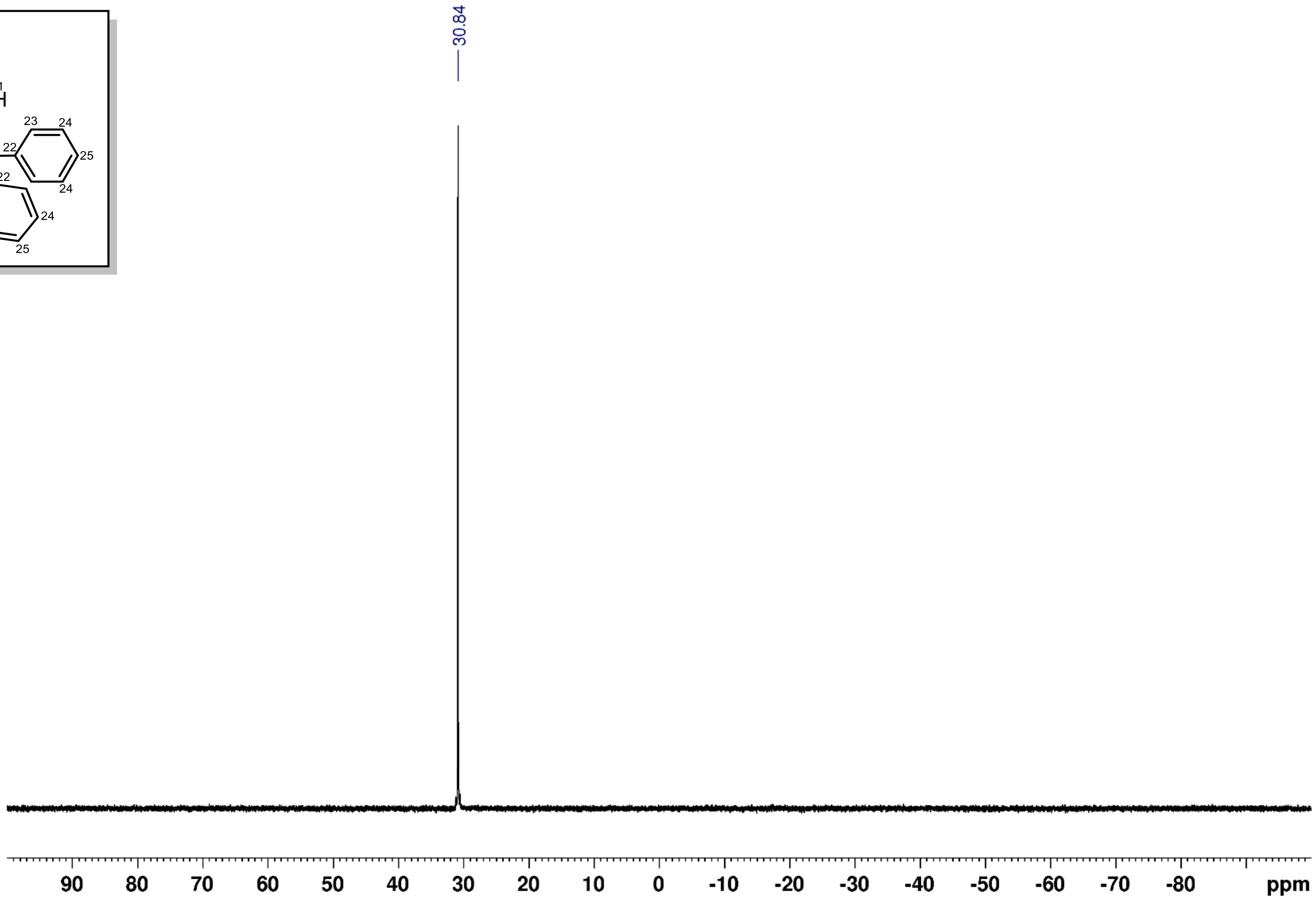

^{1}H NMR (700 MHz, CDCl$_3$) for (*R*)-2'-(diphenylphosphaneyl)-[1,1'-binaphthalen]-2-ol

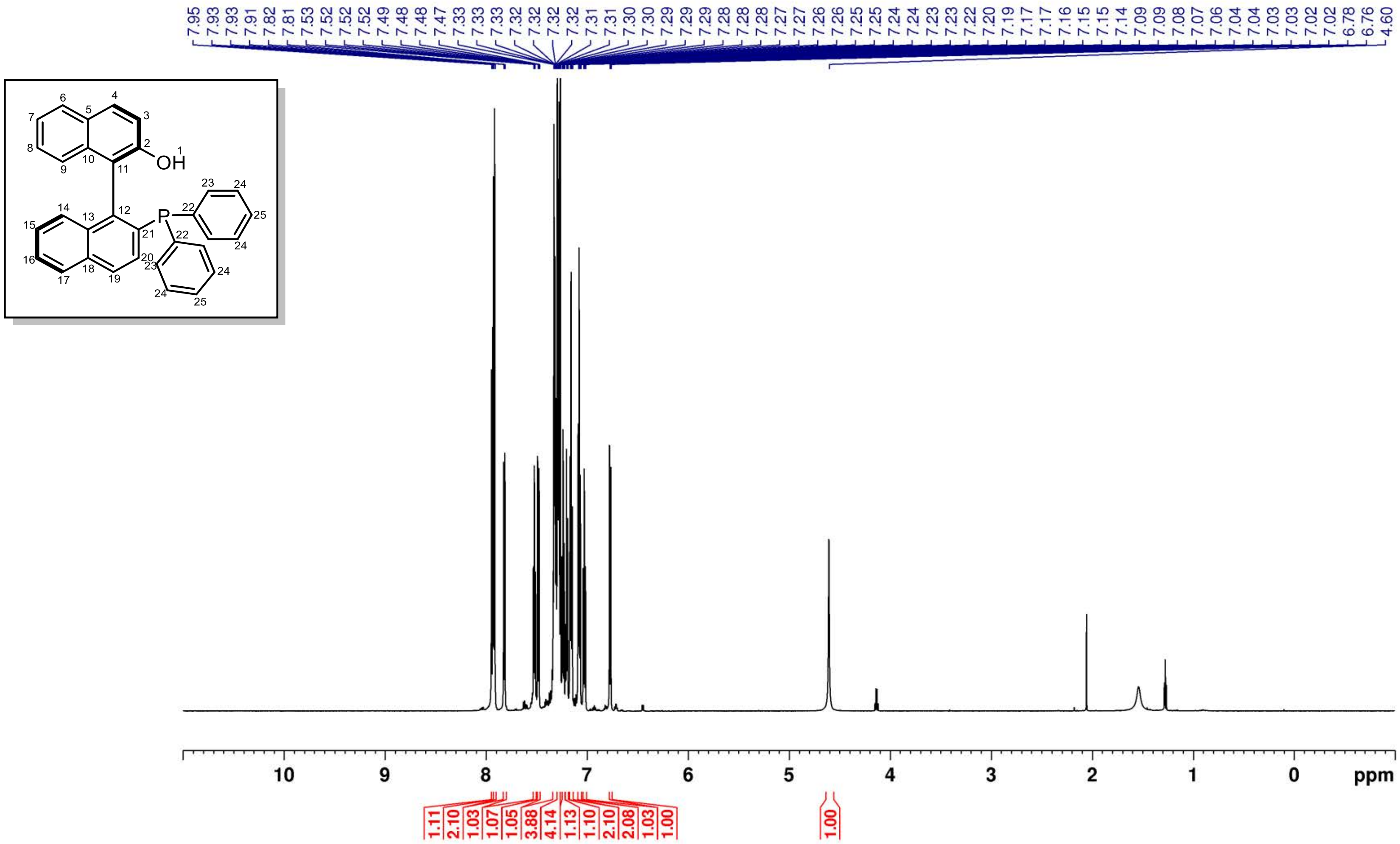

^{13}C NMR (176 MHz, $CDCl_3$) for (*R*)-2'-(diphenylphosphaneyl)-[1,1'-binaphthalen]-2-ol

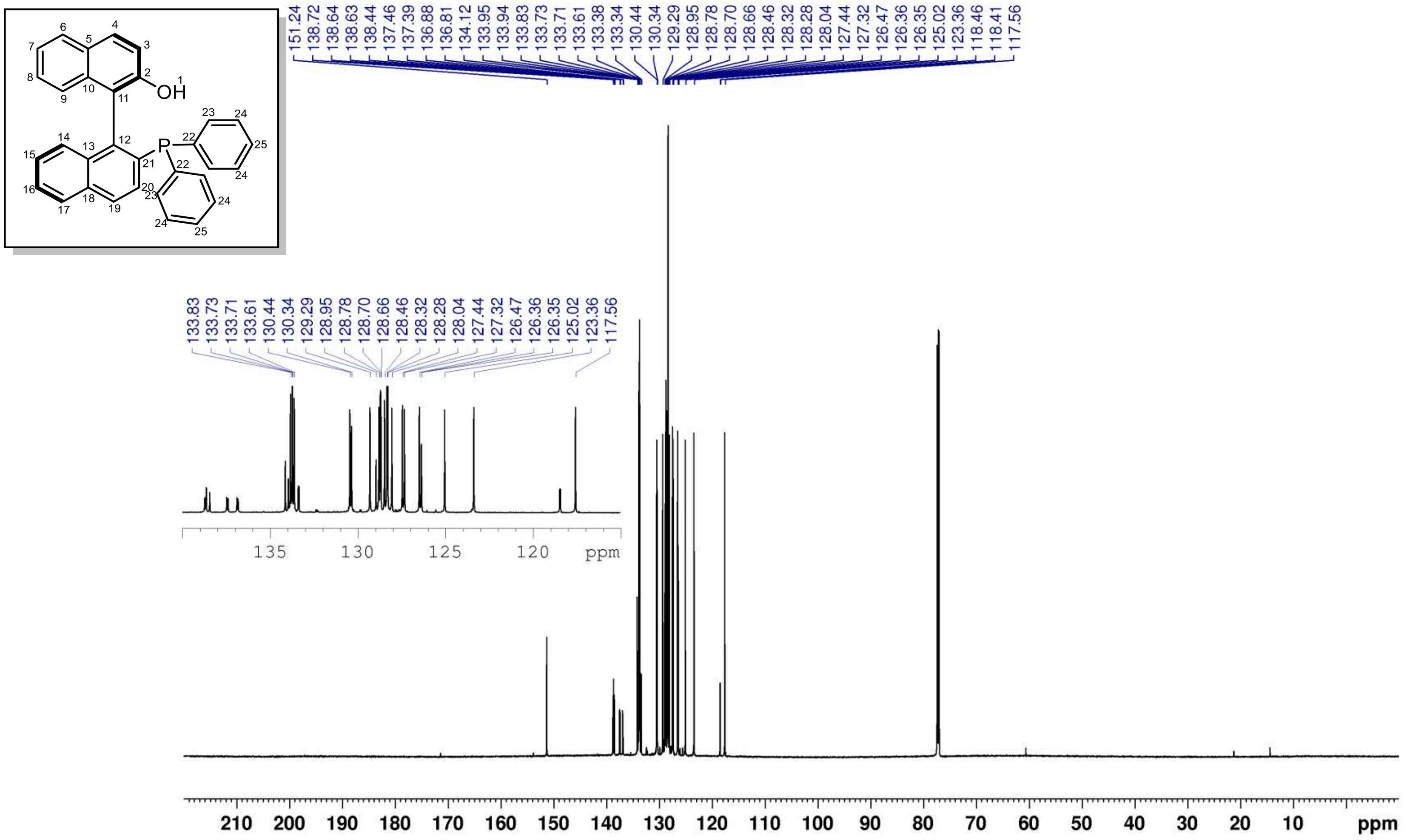

^{31}P NMR (162 MHz, $CDCl_3$) for (*R*)-2'-(diphenylphosphaneyl)-[1,1'-binaphthalen]-2-ol

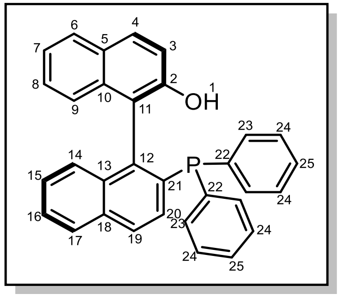

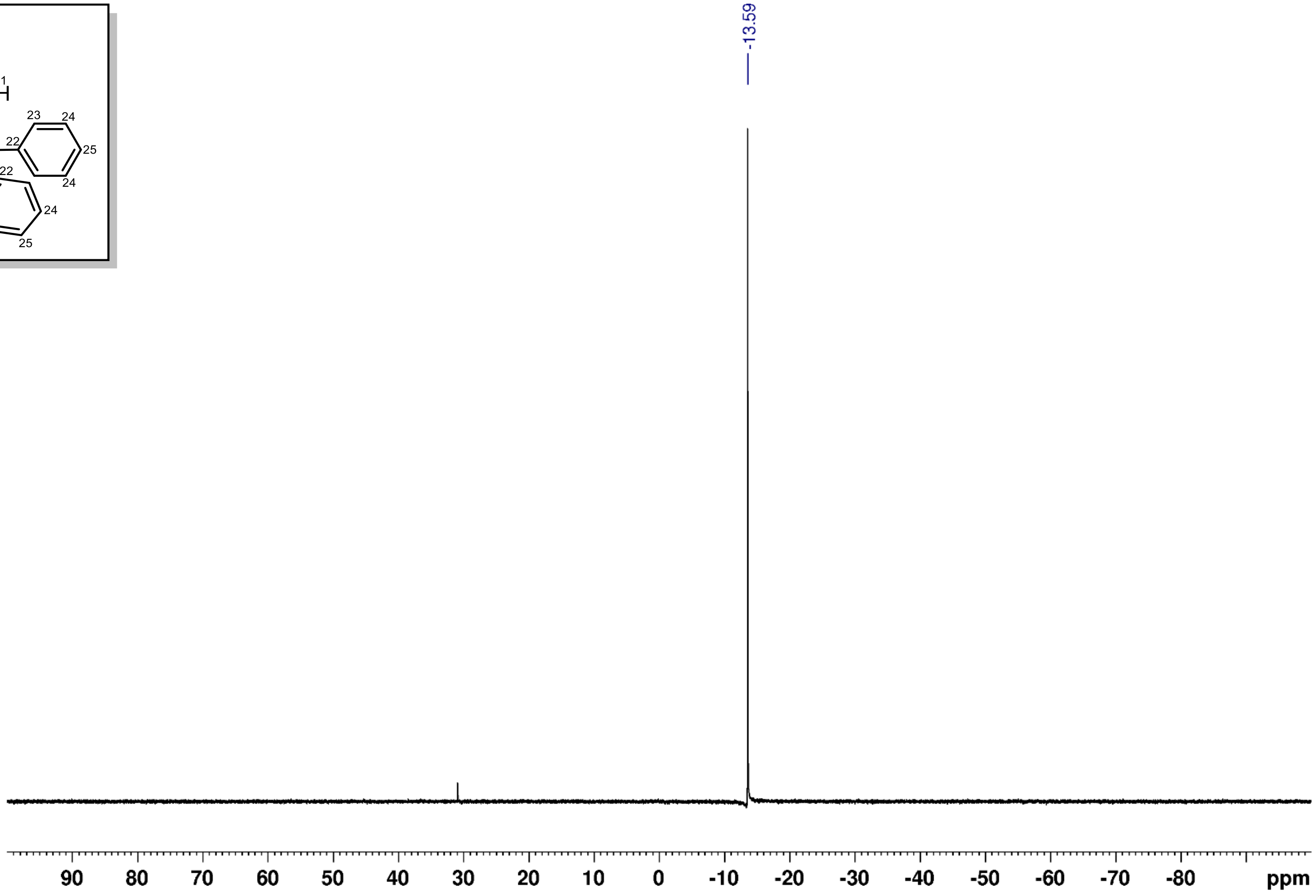

^{1}H NMR (700 MHz, CDCl$_3$) for (*R*)-(3,5-di-*tert*-butylbenzyl)(2'-hydroxy-[1,1'-binaphthalen]-2-yl)diphenylphosphonium bromide (**D1**•Br)

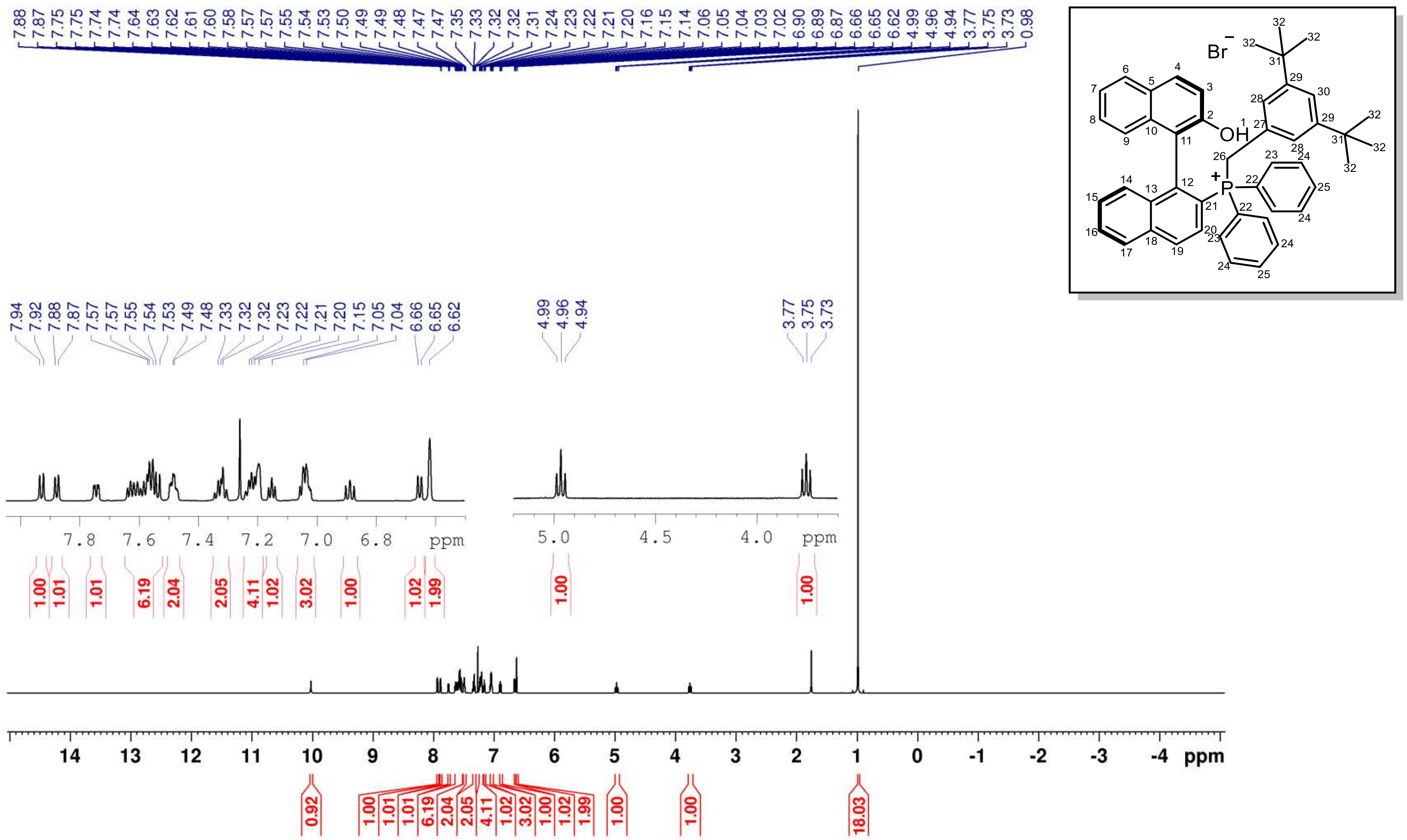

^{13}C NMR (176 MHz, CDCl$_3$) for (*R*)-(3,5-di-*tert*-butylbenzyl)(2'-hydroxy-[1,1'-binaphthalen]-2-yl)diphenylphosphonium bromide (**D1**•Br)

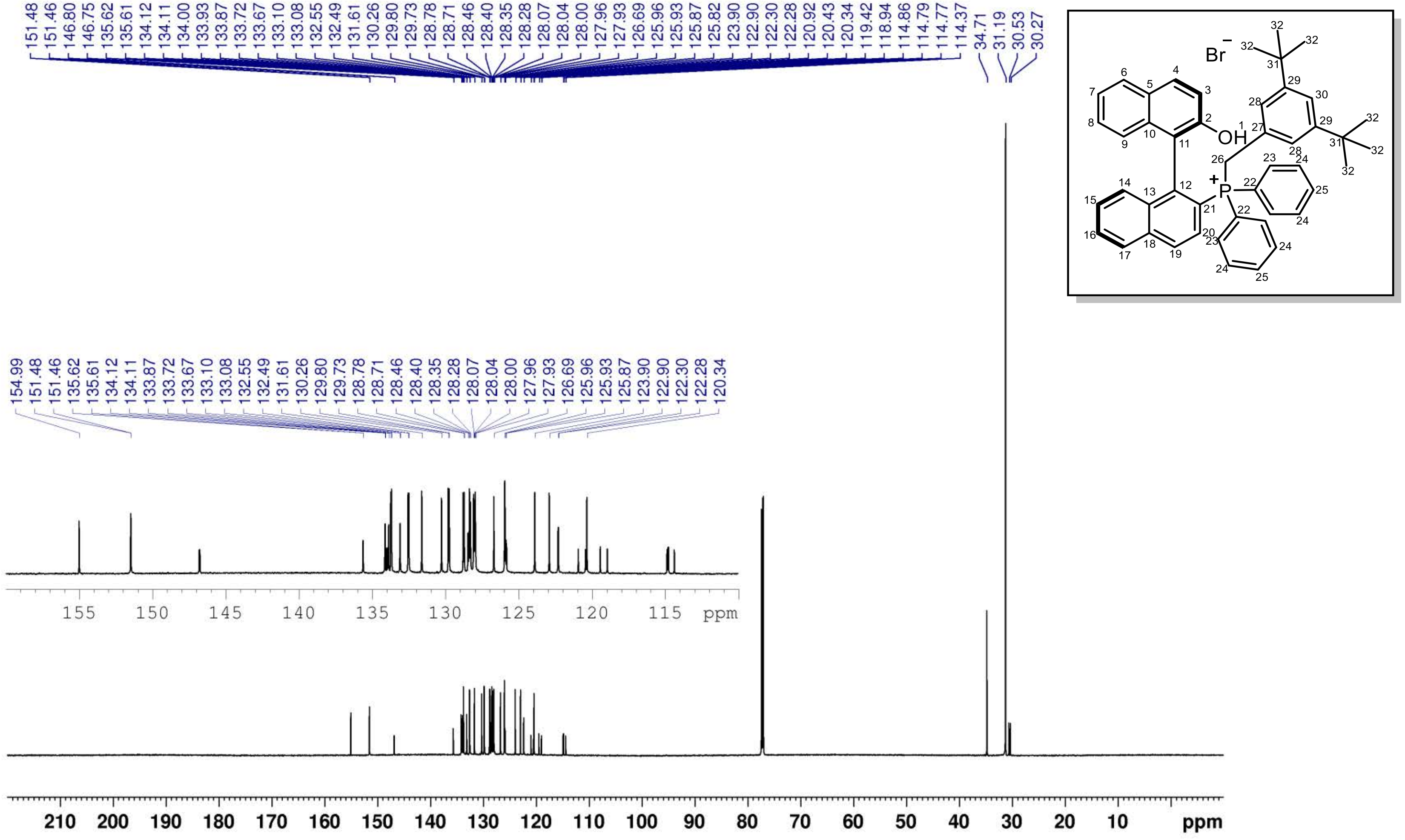

^{31}P NMR (162 MHz, CDCl$_3$) for (*R*)-(3,5-di-*tert*-butylbenzyl)(2'-hydroxy-[1,1'-binaphthalen]-2-yl)diphenylphosphonium bromide (**D1**•Br)

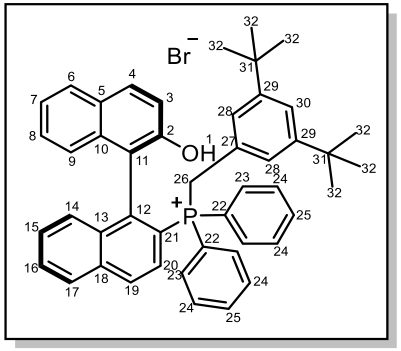

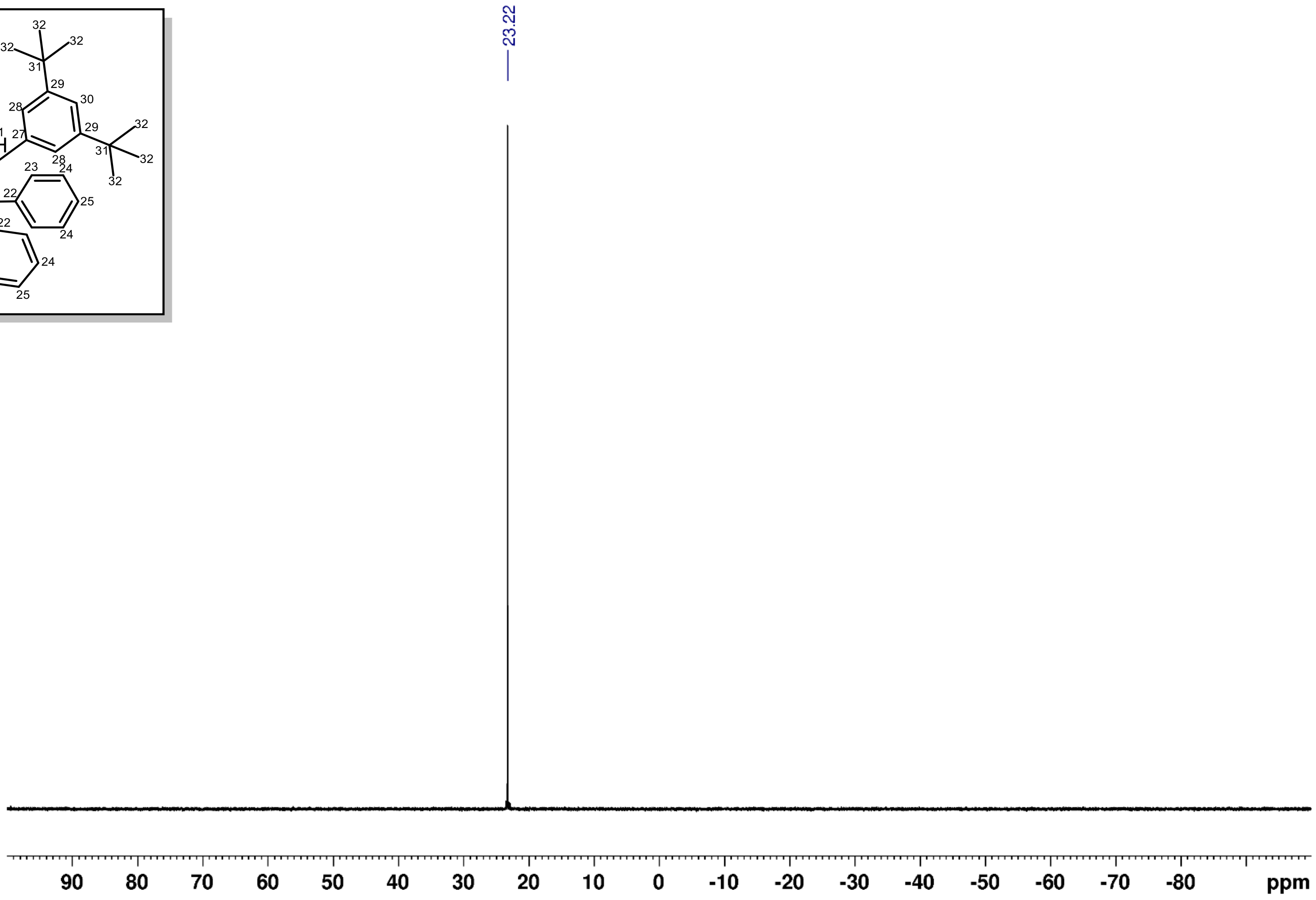

NMR Spectra for Chiral Rh(II,II) Tetracarboxylate Dimers

^{1}H NMR (700 MHz, C_5D_5N) for Bis[rhodium (1*S*,2*R*,4*S*,5*R*)-1-(3,5-di-*tert*-butylbenzyl)-5-ethyl-2-((*S*)-hydroxy(6-methoxyquinolin-4-yl)methyl)quinuclidin-1-ium (3,5-bis(2-carboxy-2-methylpropyl)phenyl)methanesulfonate)] (Rh_2(**A1**)$_2$•(**B3**)$_2$)

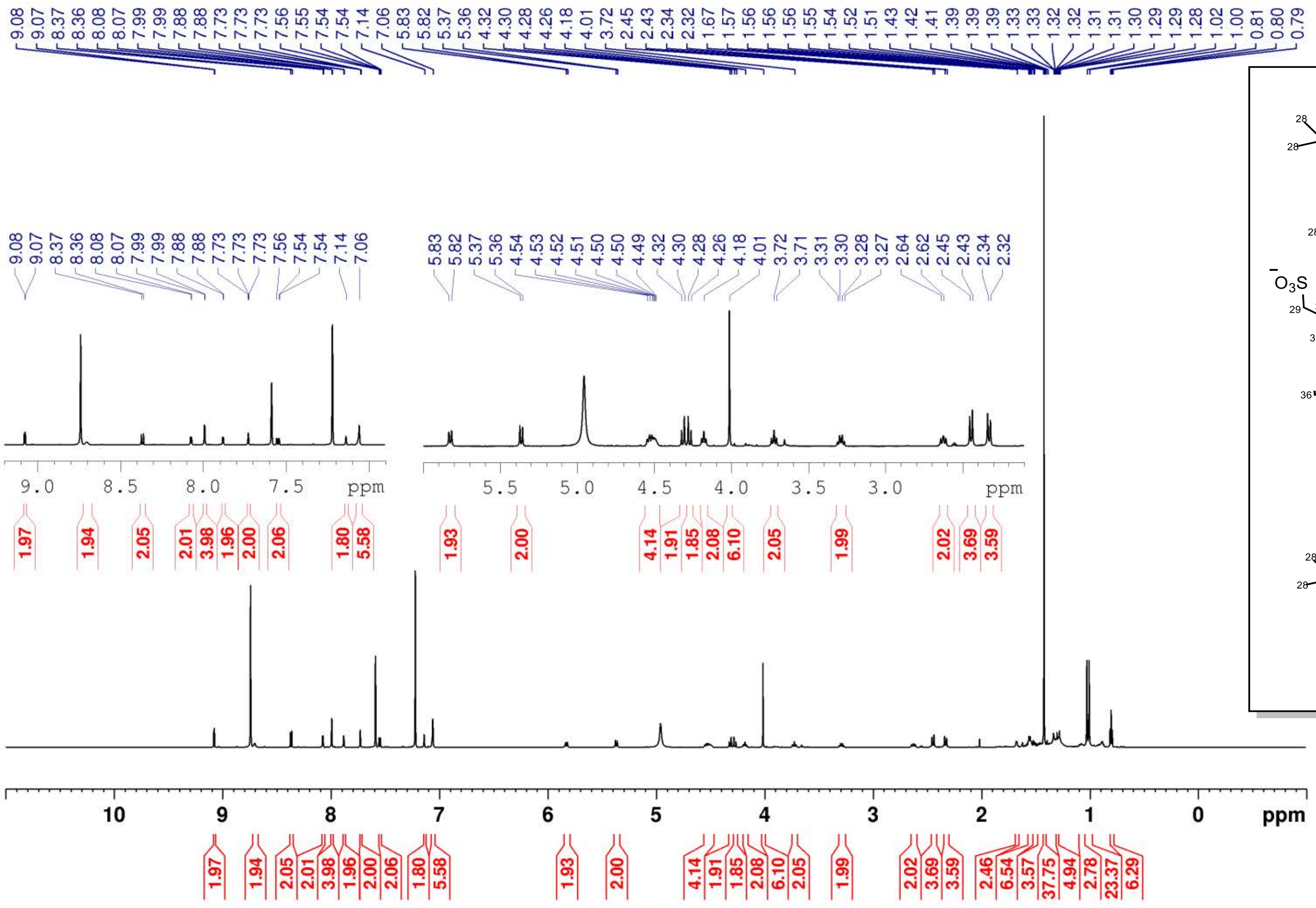

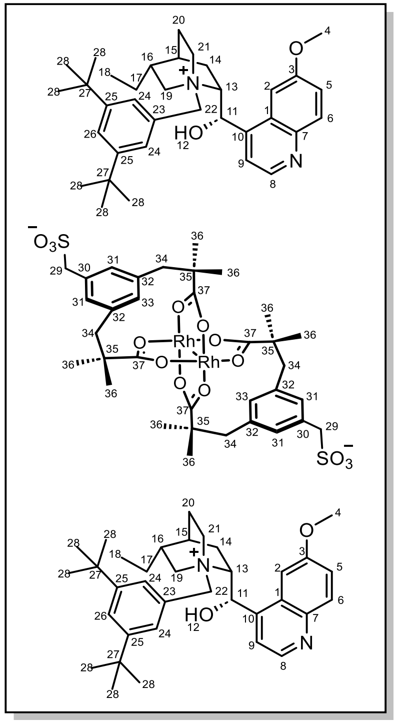

^{13}C NMR (176 MHz, C_5D_5N) for Bis[rhodium (1*S*,2*R*,4*S*,5*R*)-1-(3,5-di-*tert*-butylbenzyl)-5-ethyl-2-((*S*)-hydroxy(6-methoxyquinolin-4-yl)methyl)quinuclidin-1-ium (3,5-bis(2-carboxy-2-methylpropyl)phenyl)methanesulfonate)] (Rh_2(**A1**)$_2$•(**B3**)$_2$)

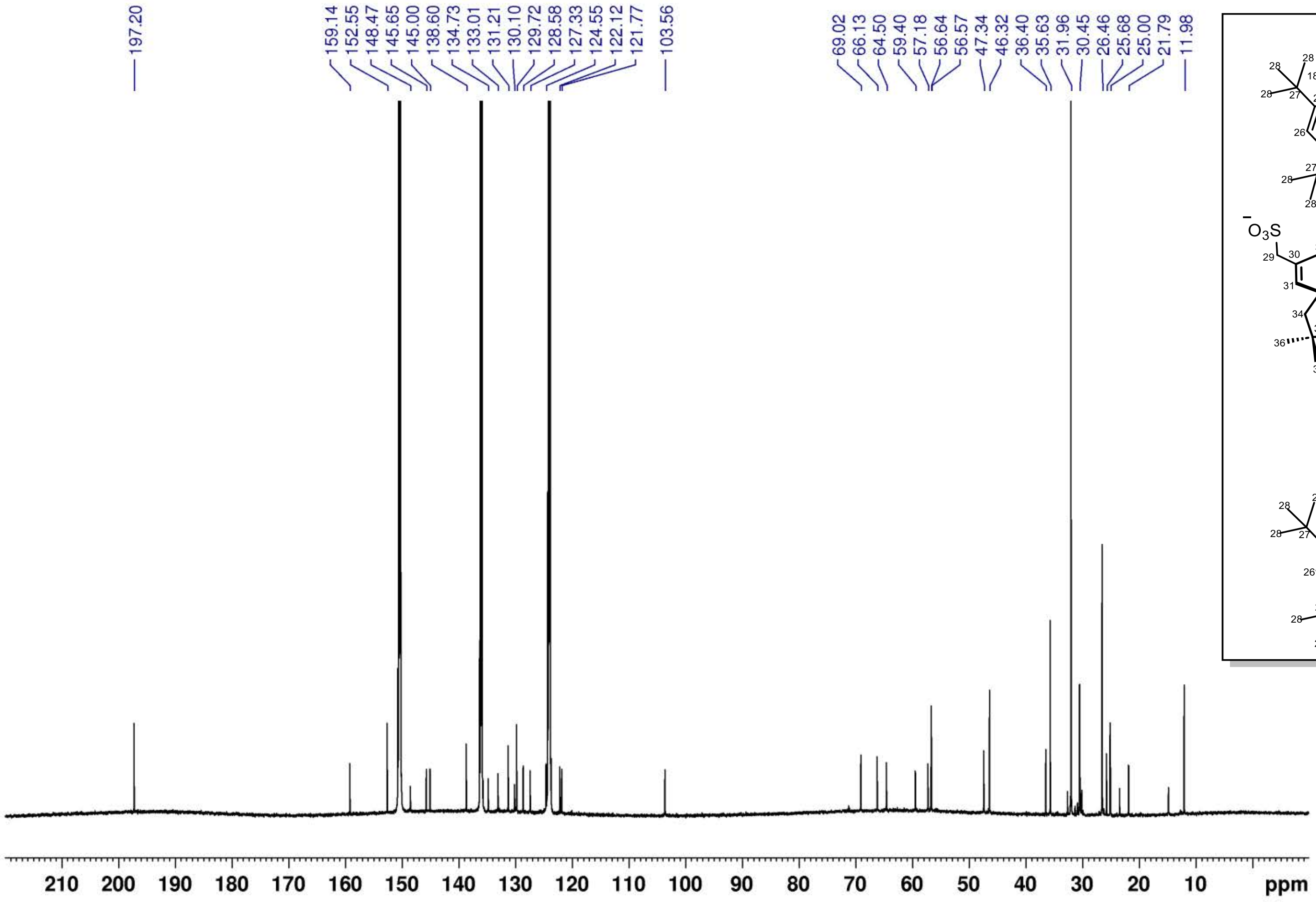

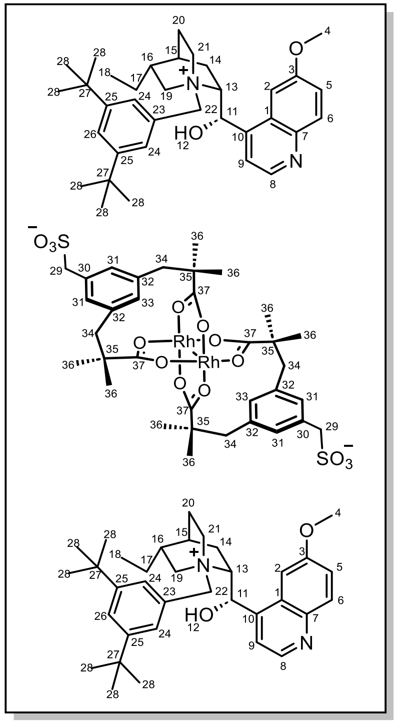

^{1}H NMR (700 MHz, C_5D_5N) for Bis[rhodium (1*S*,2*R*,4*S*,5*R*)-1-(3,5-bis(trifluoromethyl)benzyl)-5-ethyl-2-((*S*)-hydroxy(6-methoxyquinolin-4-yl)methyl)quinuclidin-1-ium (3,5-bis(2-carboxy-2-methylpropyl)phenyl)methanesulfonate)] (Rh_2(**A1**)$_2$•(**B4**)$_2$)

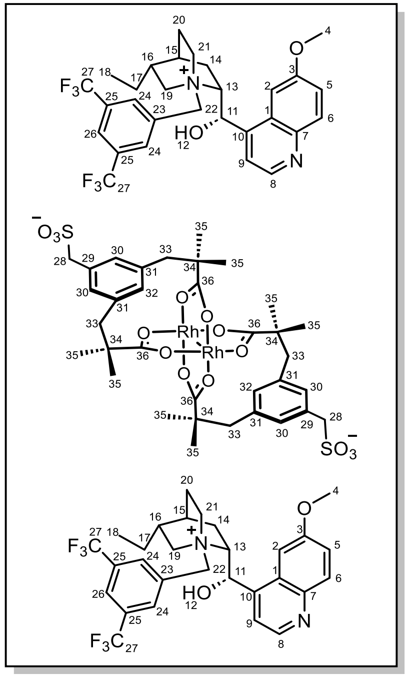

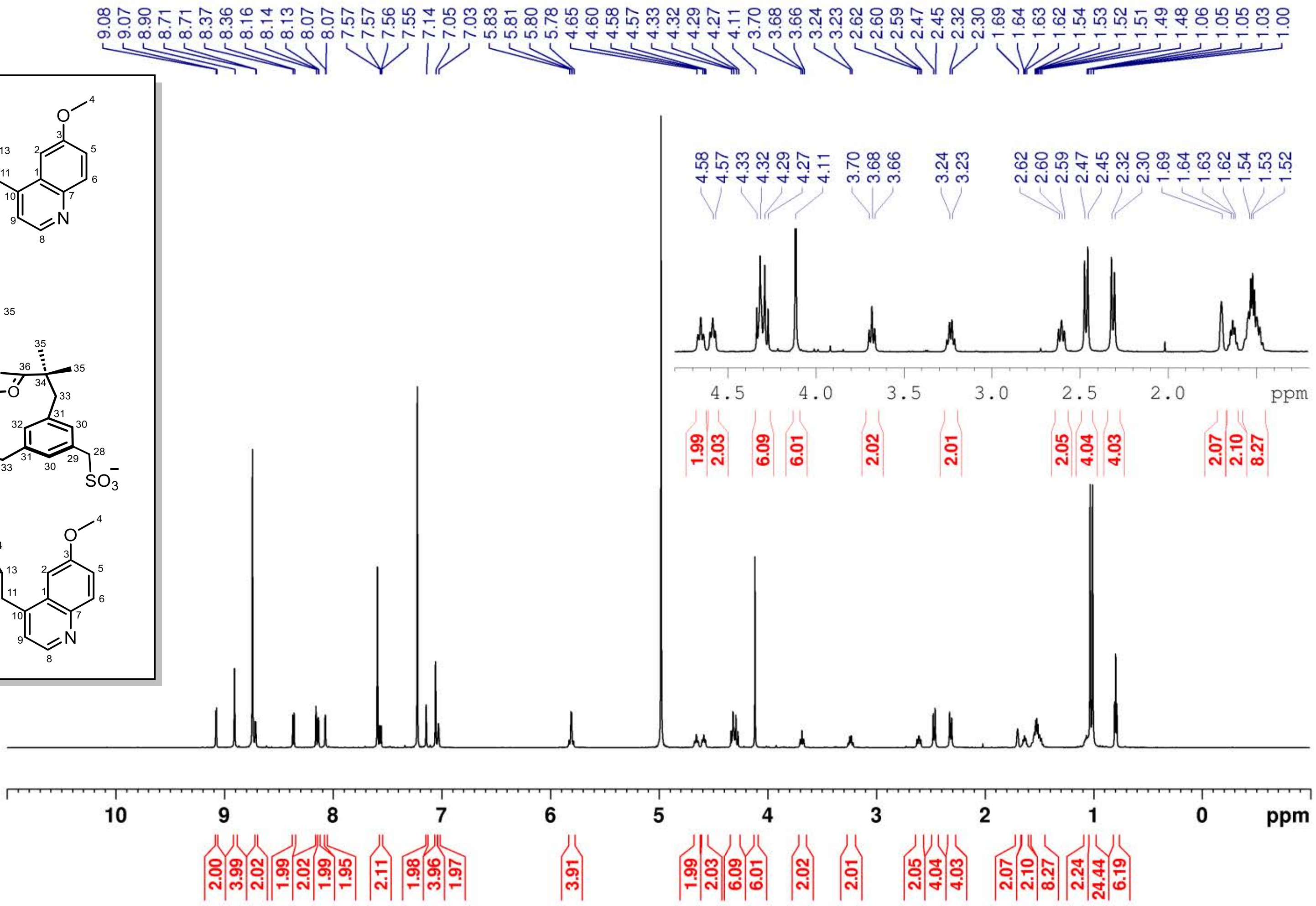

^{13}C NMR (176 MHz, C_5D_5N) for Bis[rhodium (1*S*,2*R*,4*S*,5*R*)-1-(3,5-bis(trifluoromethyl)benzyl)-5-ethyl-2-((*S*)-hydroxy(6-methoxyquinolin-4-yl)methyl)quinuclidin-1-ium (3,5-bis(2-carboxy-2-methylpropyl)phenyl)methanesulfonate)] (Rh_2(**A1**)$_2$•(**B4**)$_2$)

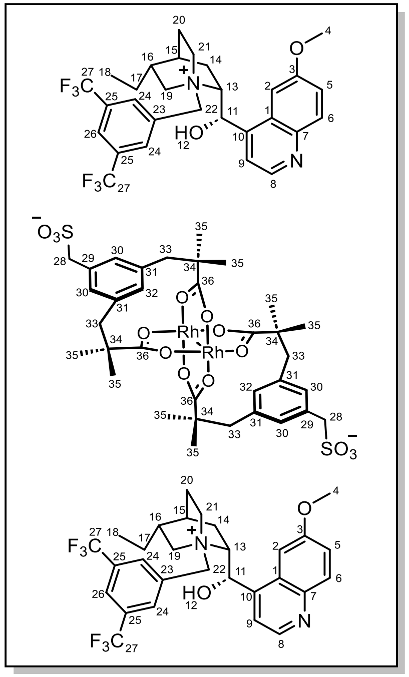

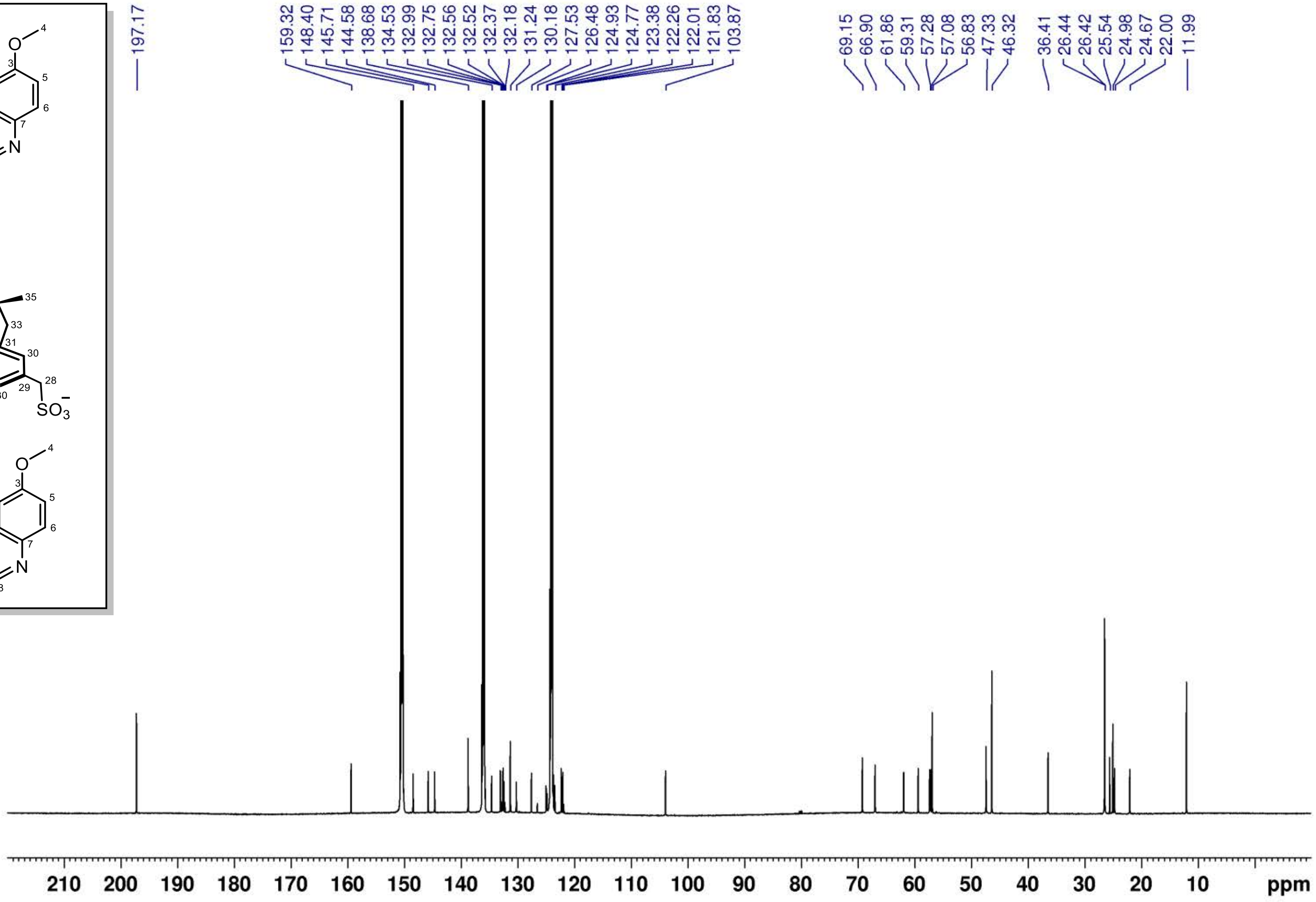

^{19}F NMR (376 MHz, C_5D_5N) for Bis[rhodium (1*S*,2*R*,4*S*,5*R*)-1-(3,5-bis(trifluoromethyl)benzyl)-5-ethyl-2-((*S*)-hydroxy(6-methoxyquinolin-4-yl)methyl)quinuclidin-1-ium (3,5-bis(2-carboxy-2-methylpropyl)phenyl)methanesulfonate)] (Rh_2(**A1**)$_2$•(**B4**)$_2$)

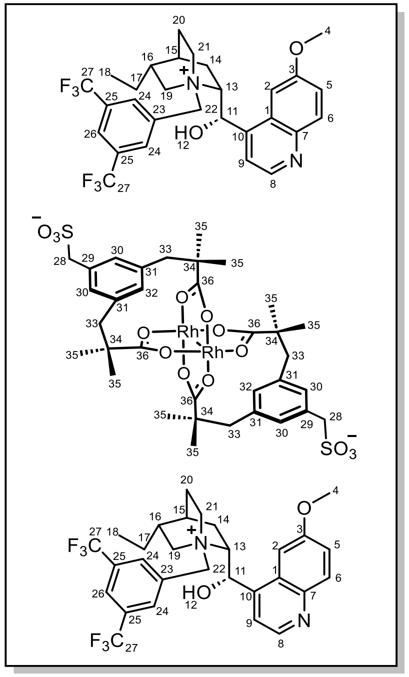

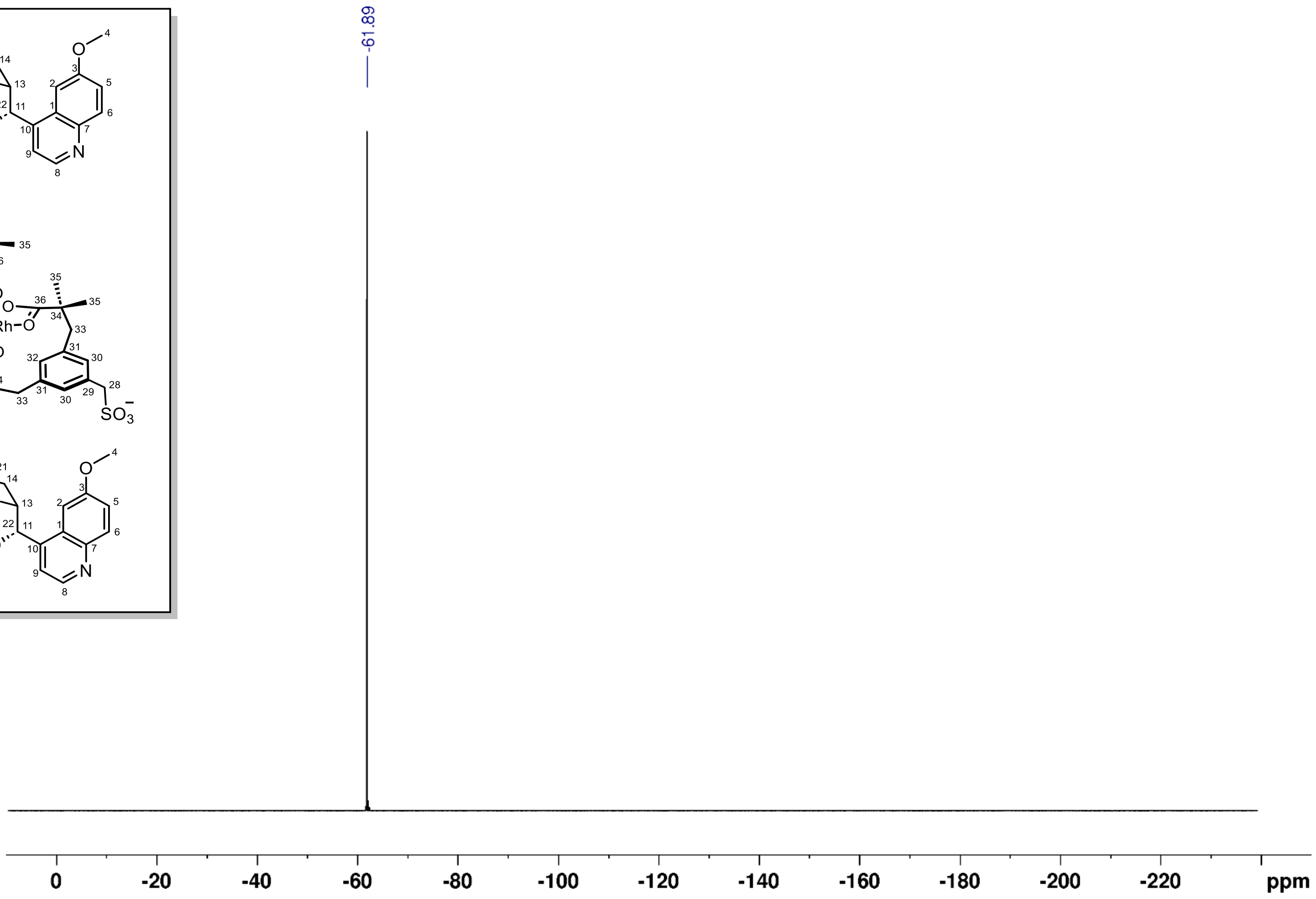

^{1}H NMR (700 MHz, C_5D_5N) for Bis[rhodium (1*S*,2*R*,4*S*,5*R*)-1-(3,5-dibromobenzyl)-5-ethyl-2-((*S*)-hydroxy(6-methoxyquinolin-4-yl)methyl)quinuclidin-1-ium (3,5-bis(2-carboxy-2-methylpropyl)phenyl)methanesulfonate)] (Rh_2(**A1**)$_2$•(**B5**)$_2$)

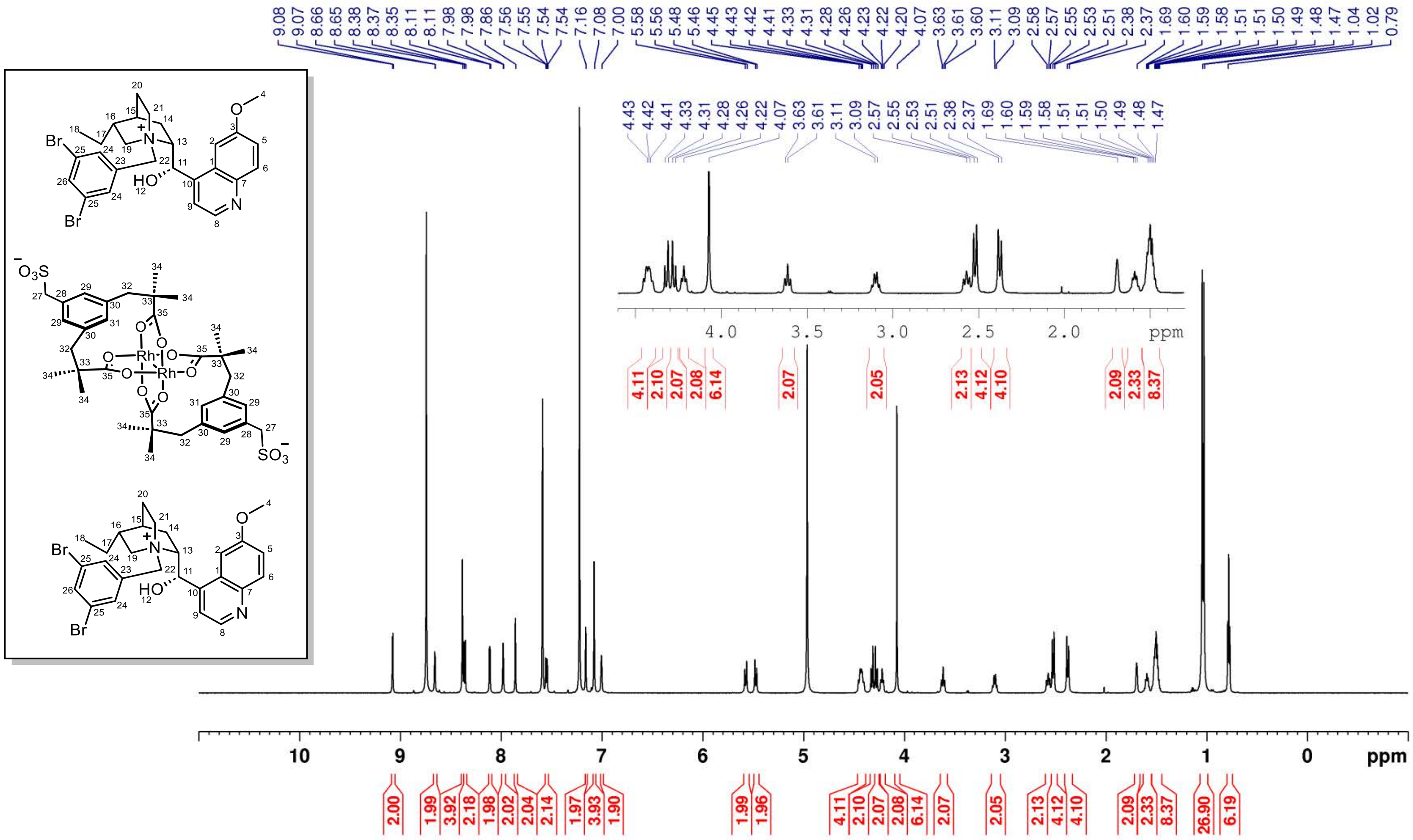

^{13}C NMR (176 MHz, C_5D_5N) for Bis[rhodium (1*S*,2*R*,4*S*,5*R*)-1-(3,5-dibromobenzyl)-5-ethyl-2-((*S*)-hydroxy(6-methoxyquinolin-4-yl)methyl)quinuclidin-1-ium (3,5-bis(2-carboxy-2-methylpropyl)phenyl)methanesulfonate)] (Rh_2(**A1**)$_2$•(**B5**)$_2$)

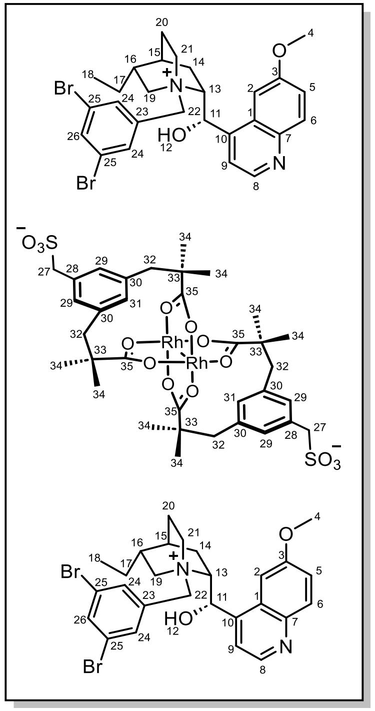

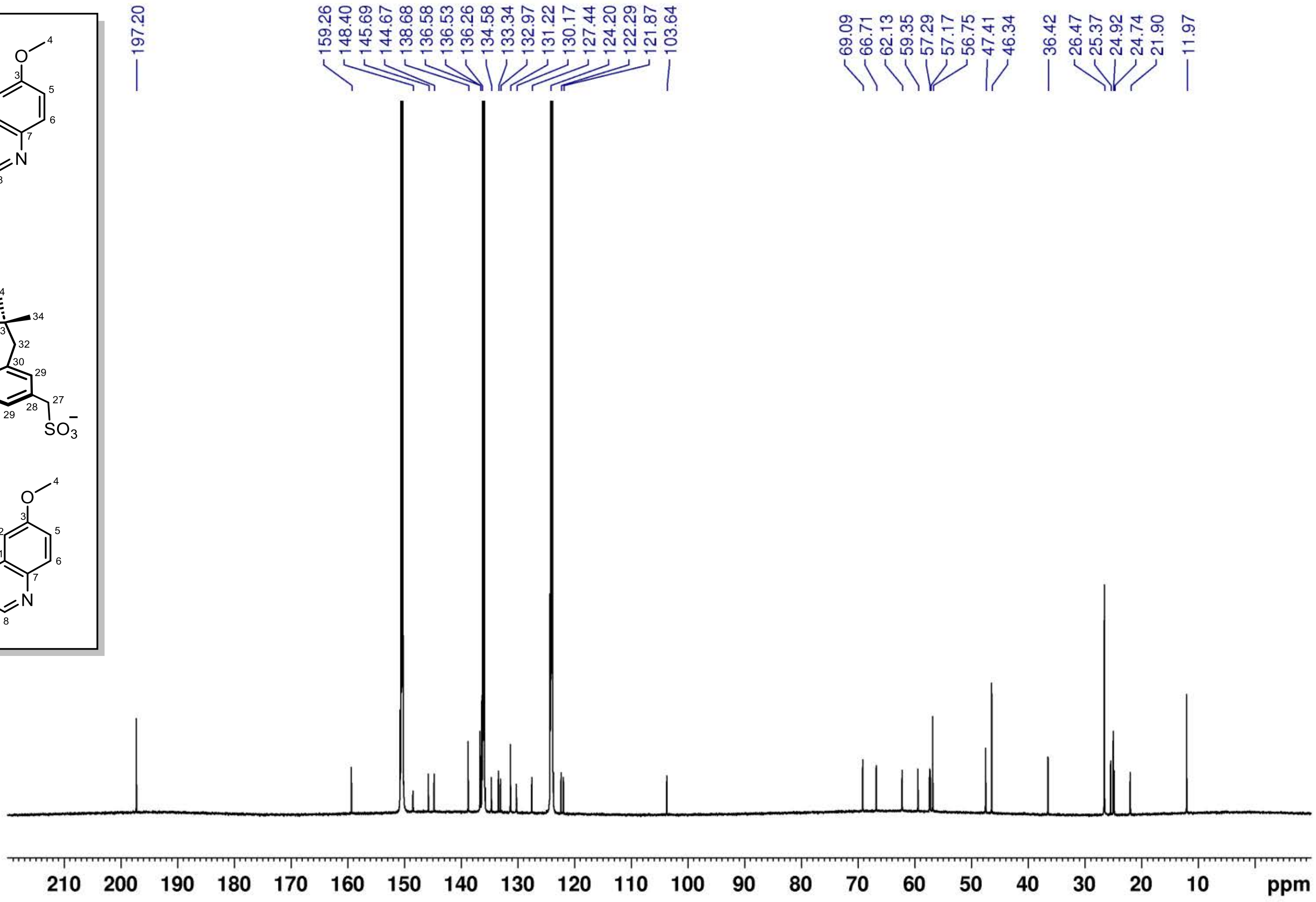

NMR Spectra for Substrates – Propargyl Alcohol:

^{1}H NMR (400 MHz, $CDCl_3$) for 3-phenylprop-2-yn-1-ol

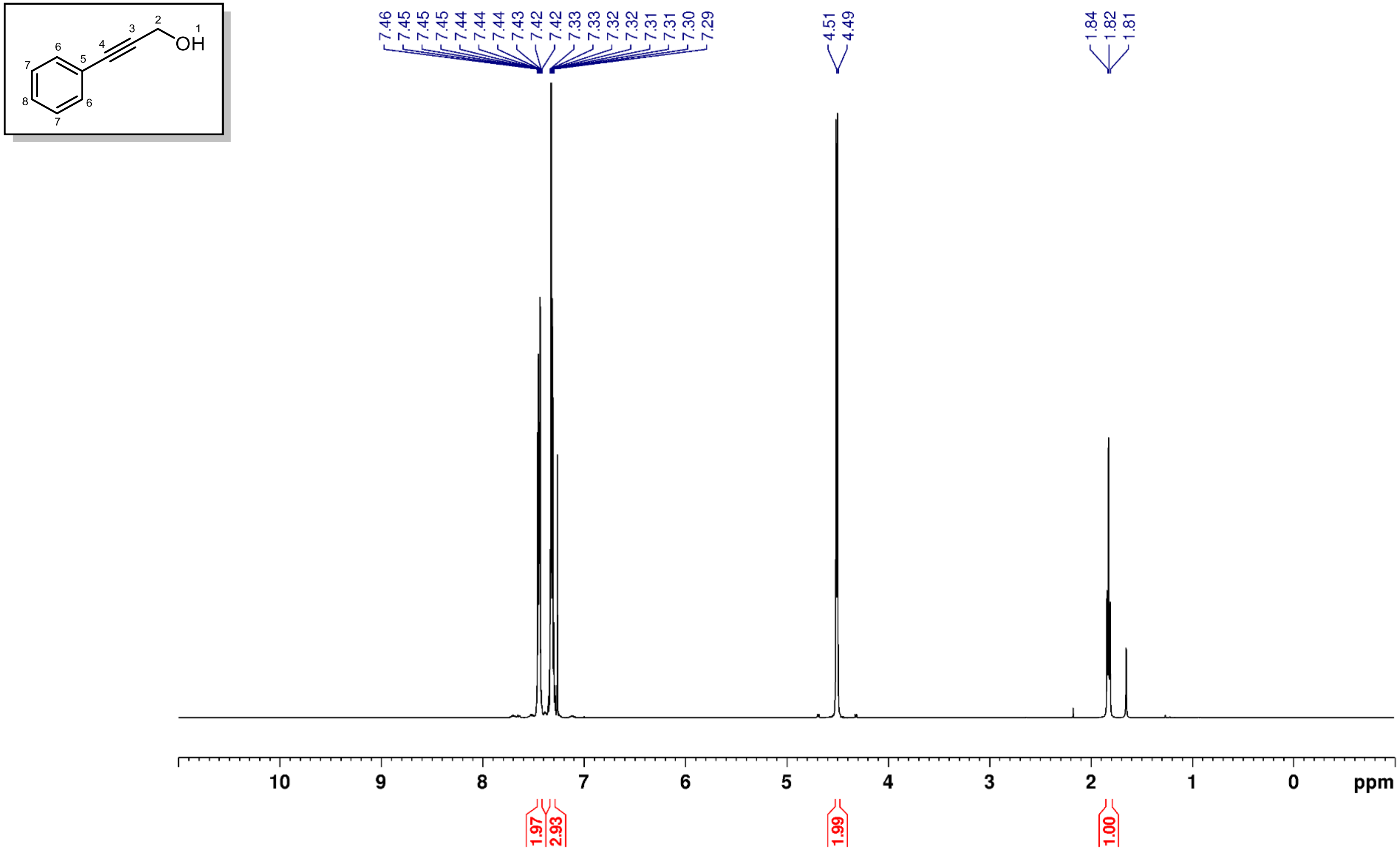

^{13}C NMR (101 MHz, CDCl$_3$) for 3-phenylprop-2-yn-1-ol

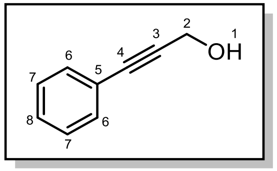

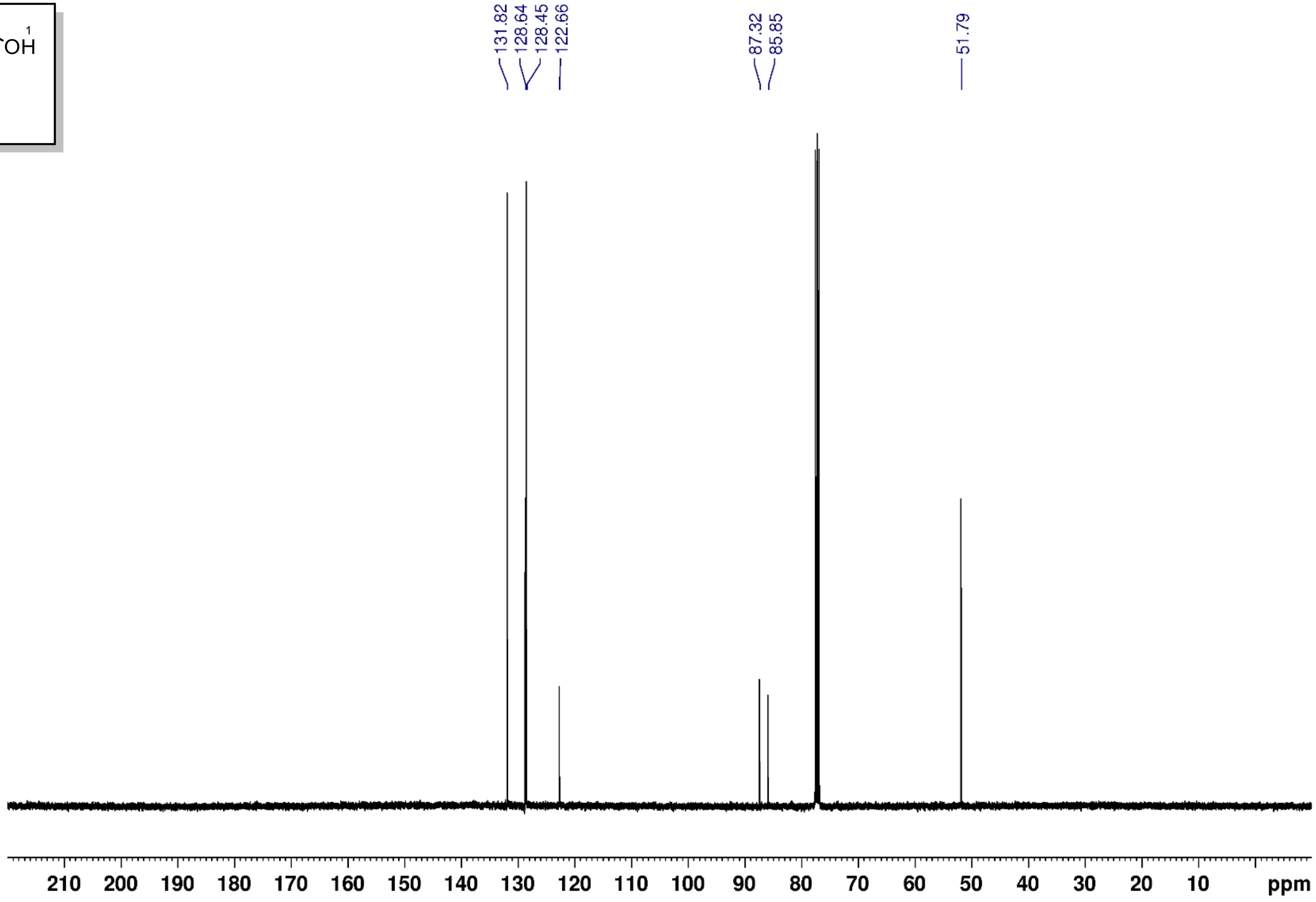

^{1}H NMR (400 MHz, $CDCl_3$) for 3-(2-isopropylphenyl)prop-2-yn-1-ol

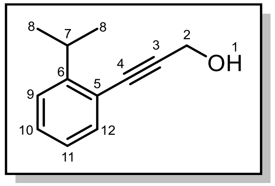

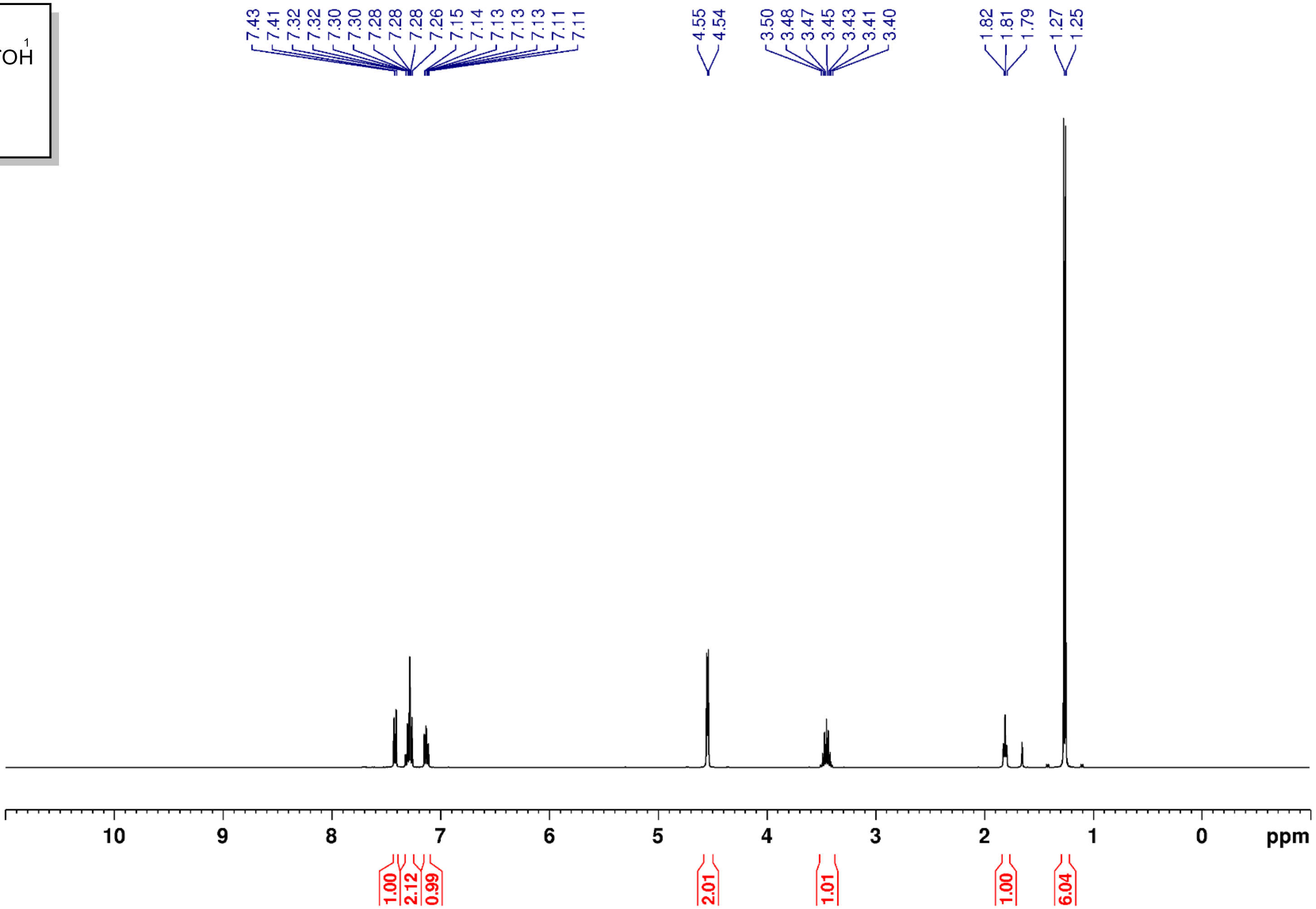

^{13}C NMR (101 MHz, CDCl$_3$) for 3-(2-isopropylphenyl)prop-2-yn-1-ol

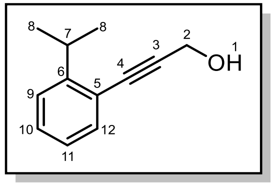

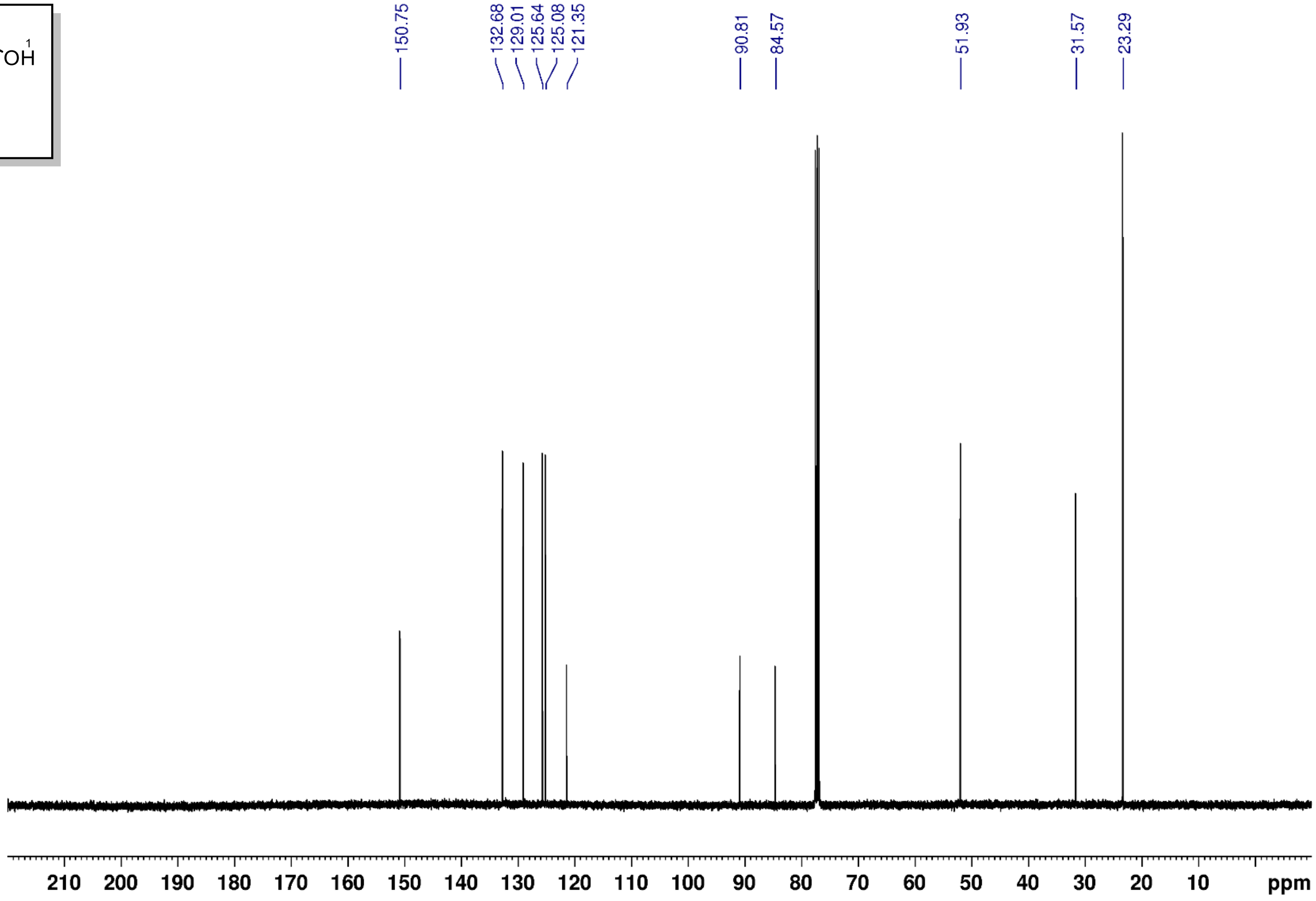

^{1}H NMR (400 MHz, $CDCl_3$) for 3-(2-fluorophenyl)prop-2-yn-1-ol

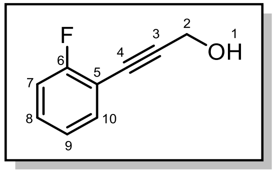

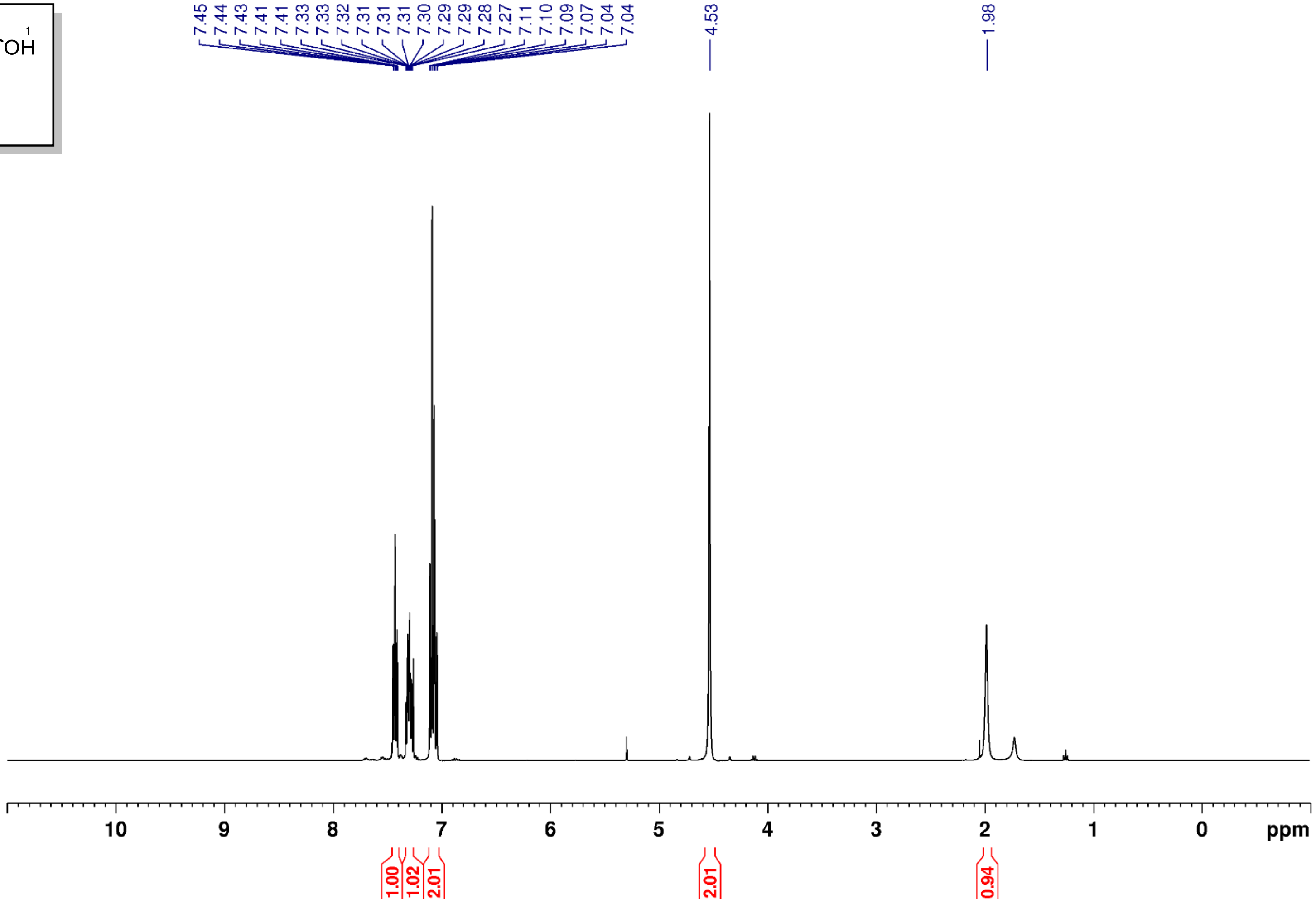

^{13}C NMR (101 MHz, $CDCl_3$) for 3-(2-fluorophenyl)prop-2-yn-1-ol

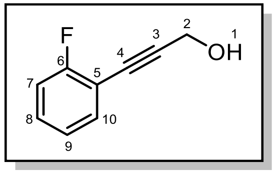

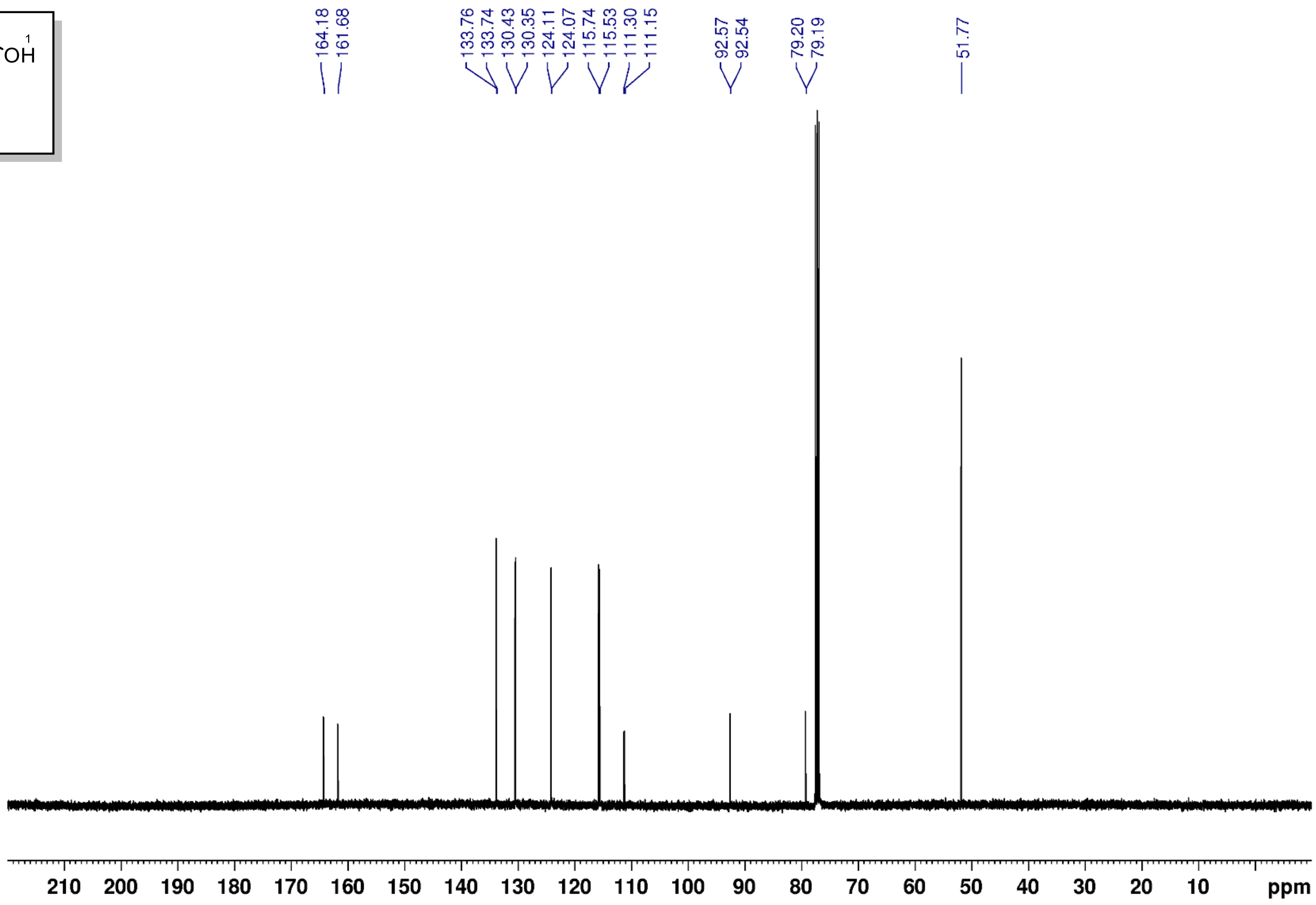

^{19}F NMR (471 MHz, $CDCl_3$) for 3-(2-fluorophenyl)prop-2-yn-1-ol

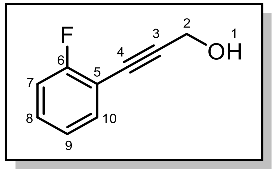

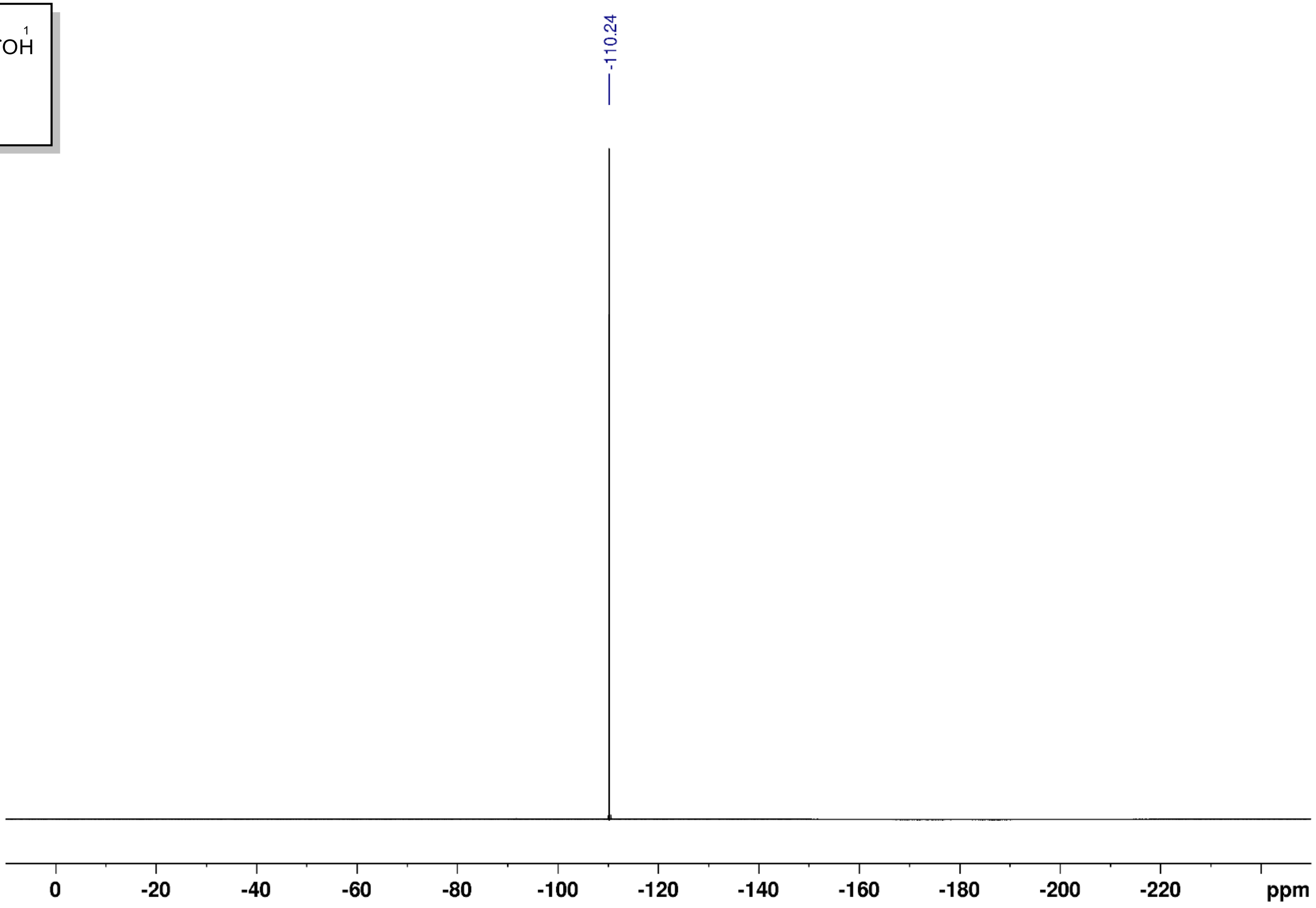

^{1}H NMR (700 MHz, CDCl$_3$) for *tert*-butyl (3-iodophenyl)carbamate

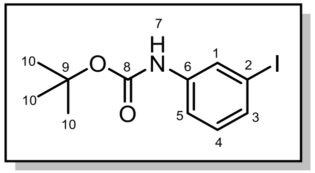

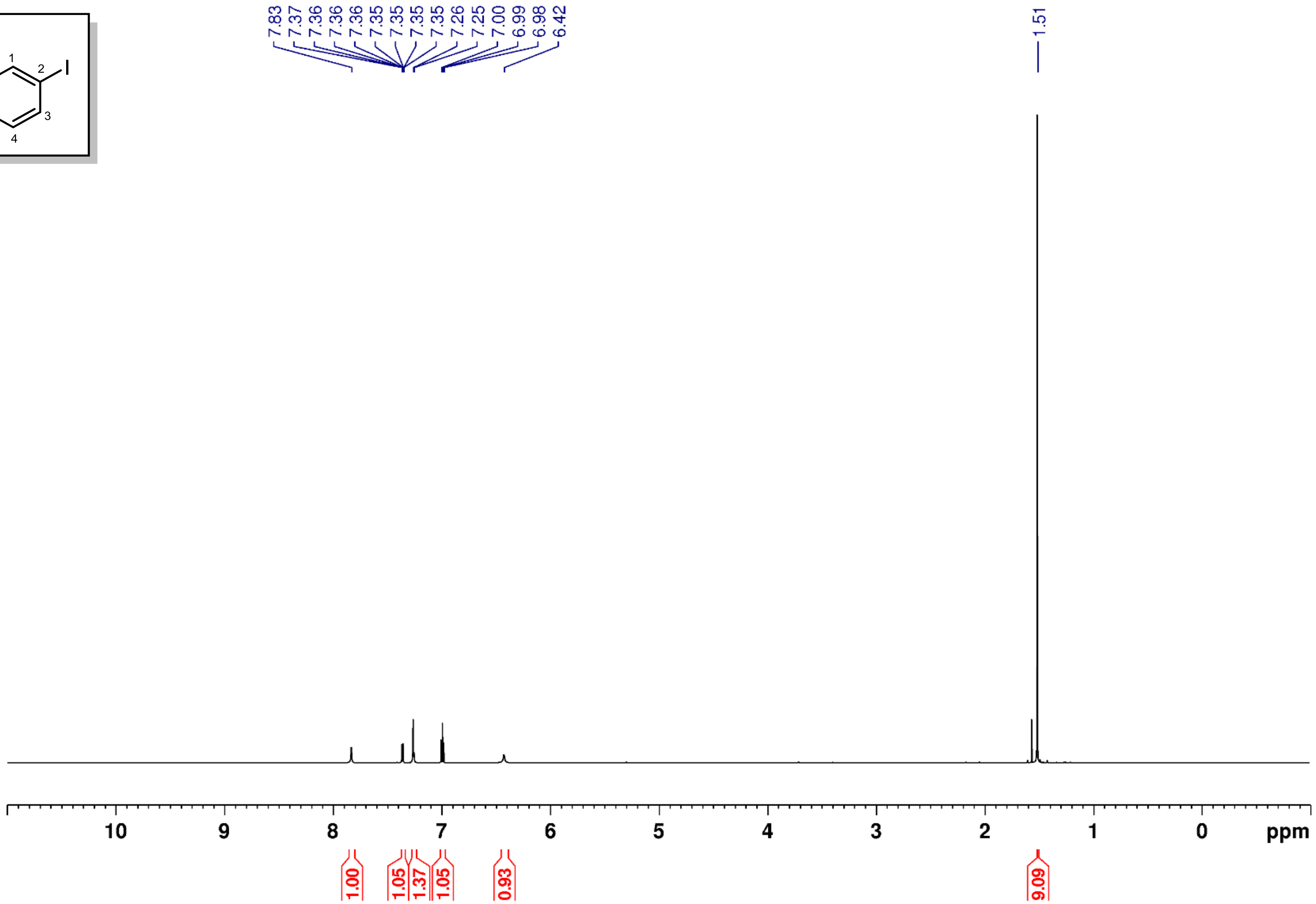

^{13}C NMR (176 MHz, $CDCl_3$) for *tert*-butyl (3-iodophenyl)carbamate

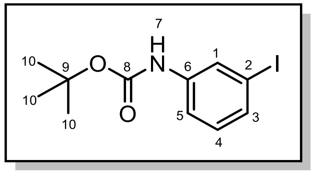

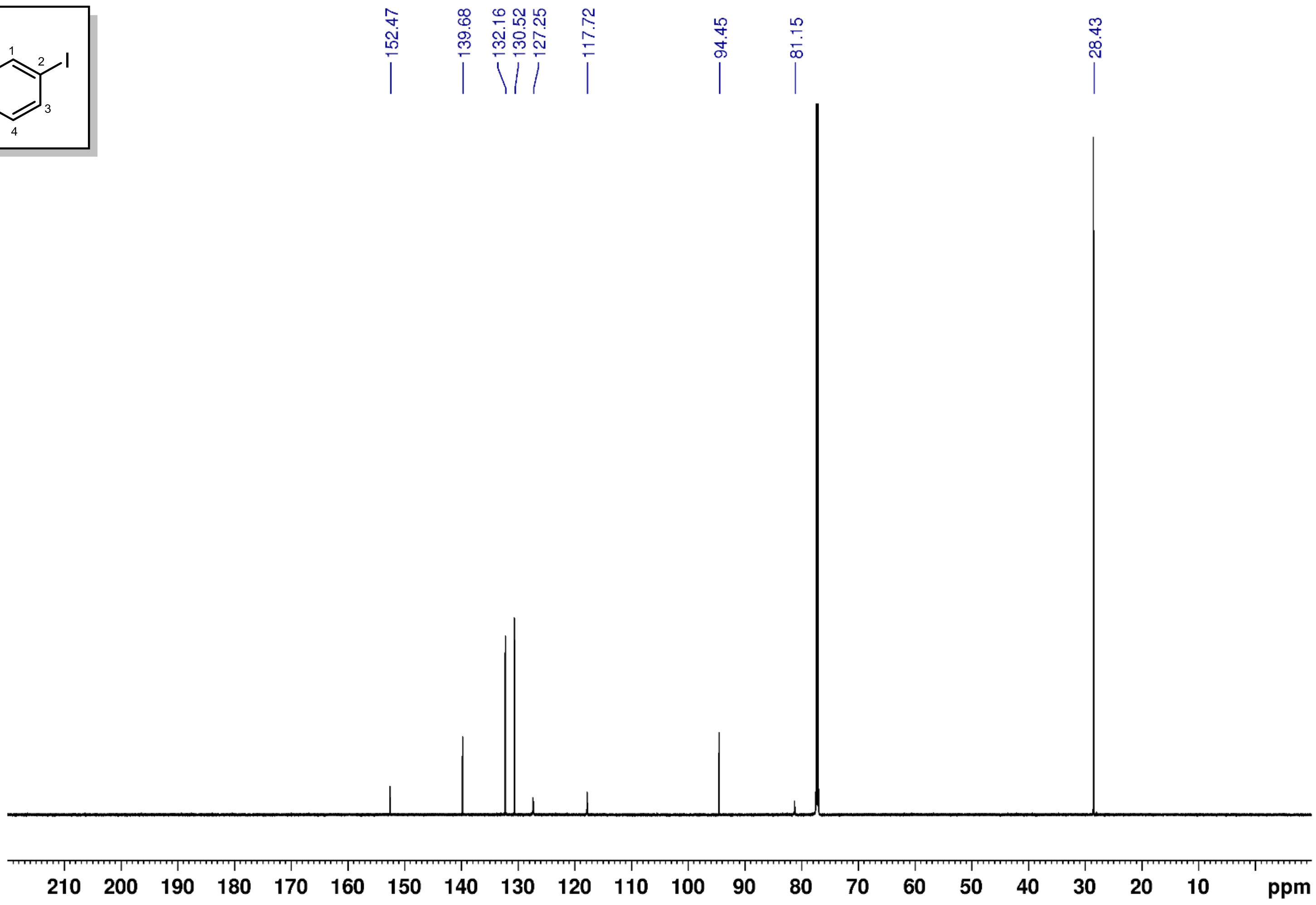

^{1}H NMR (400 MHz, CDCl$_3$) for *tert*-butyl (3-(3-hydroxyprop-1-yn-1-yl)phenyl)carbamate

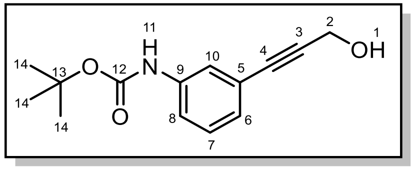

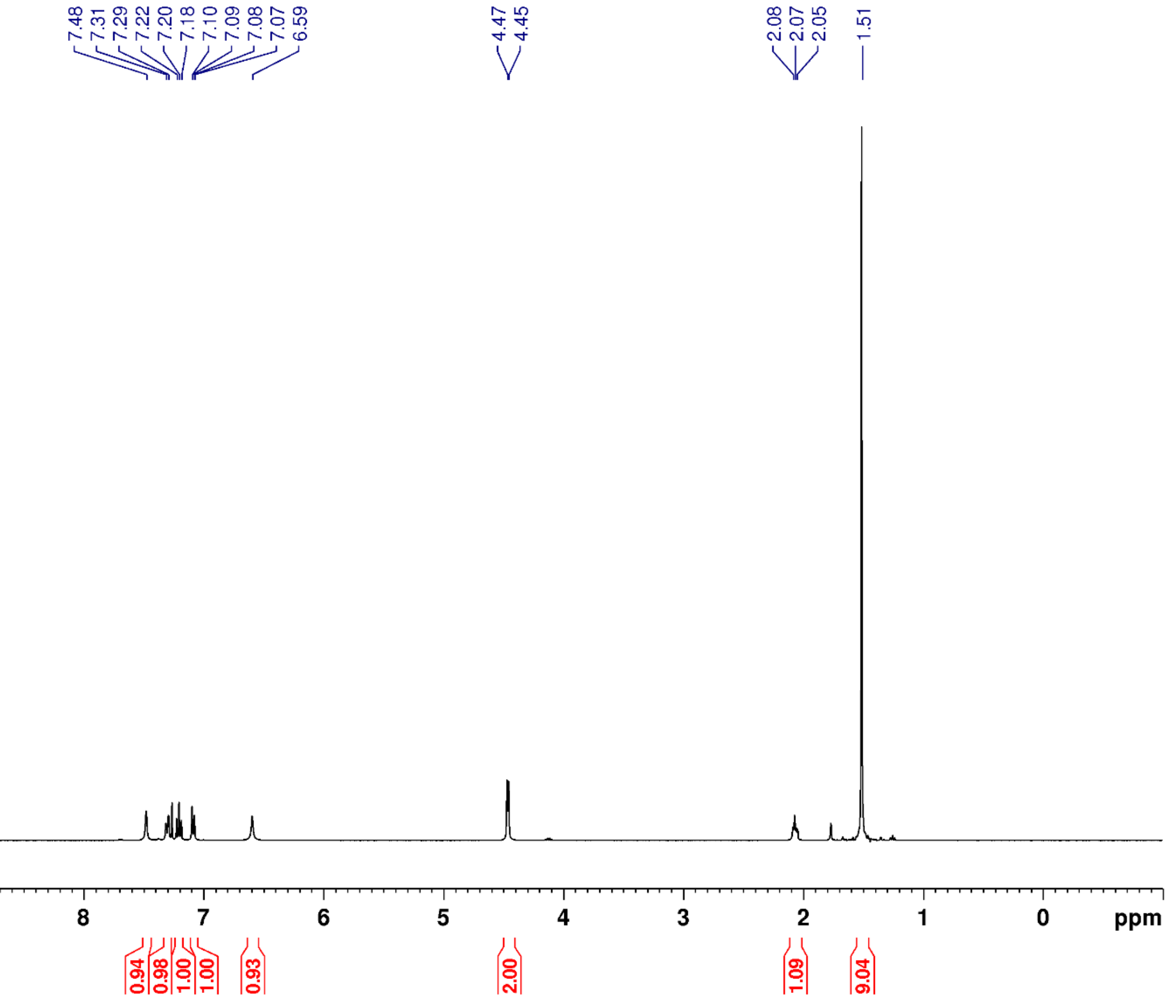

^{13}C NMR (101 MHz, CDCl$_3$) for *tert*-butyl (3-(3-hydroxyprop-1-yn-1-yl)phenyl)carbamate

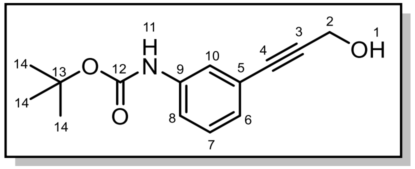

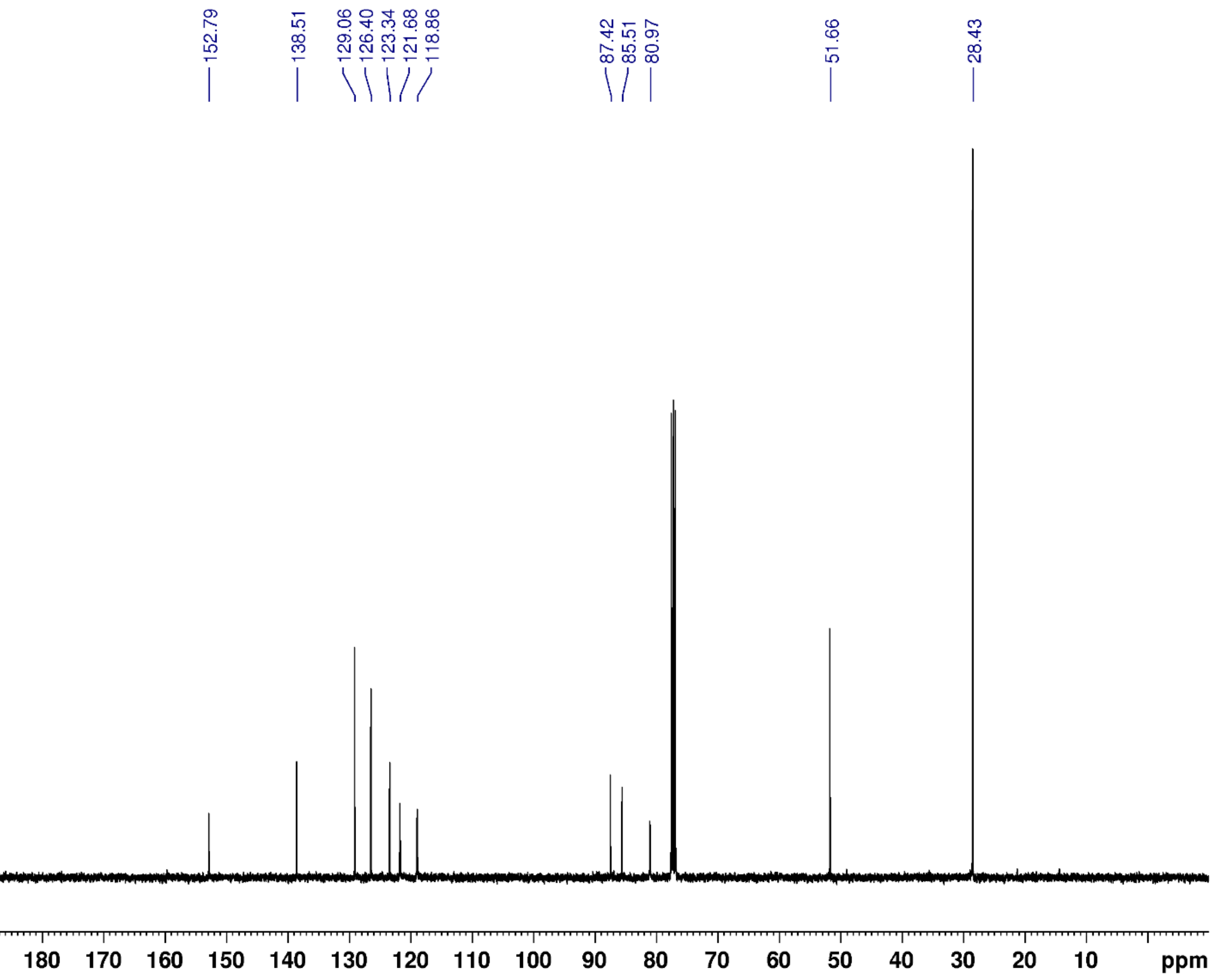

^{1}H NMR (500 MHz, CDCl$_3$) for 3-(3-(*tert*-butyl)phenyl)prop-2-yn-1-ol

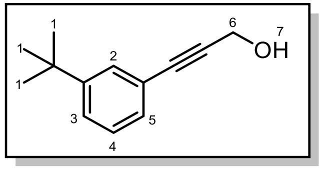

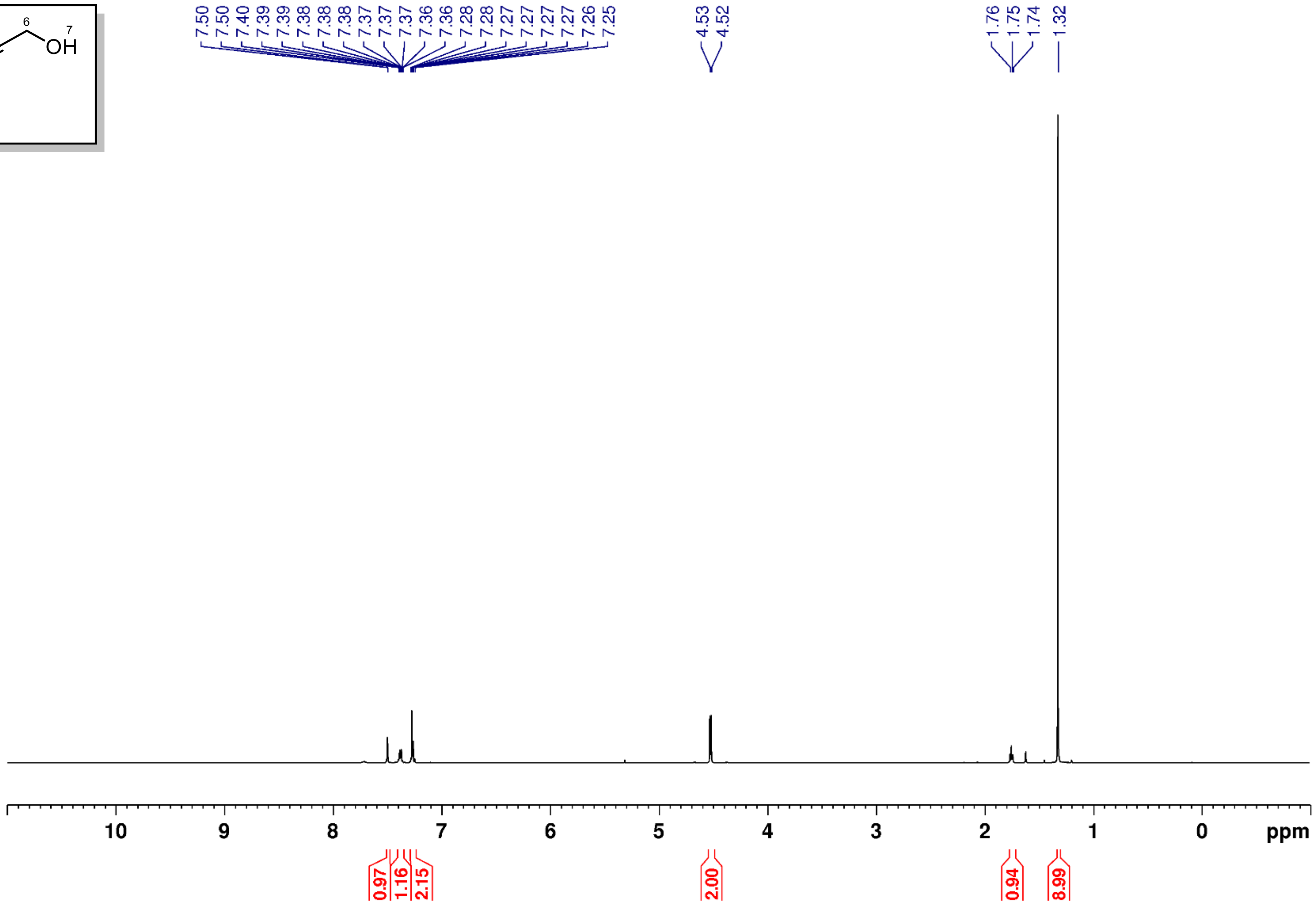

^{13}C NMR (126 MHz, $CDCl_3$) for 3-(3-(*tert*-butyl)phenyl)prop-2-yn-1-ol

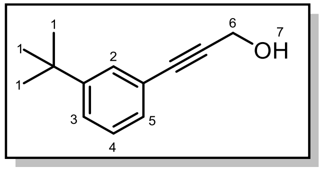

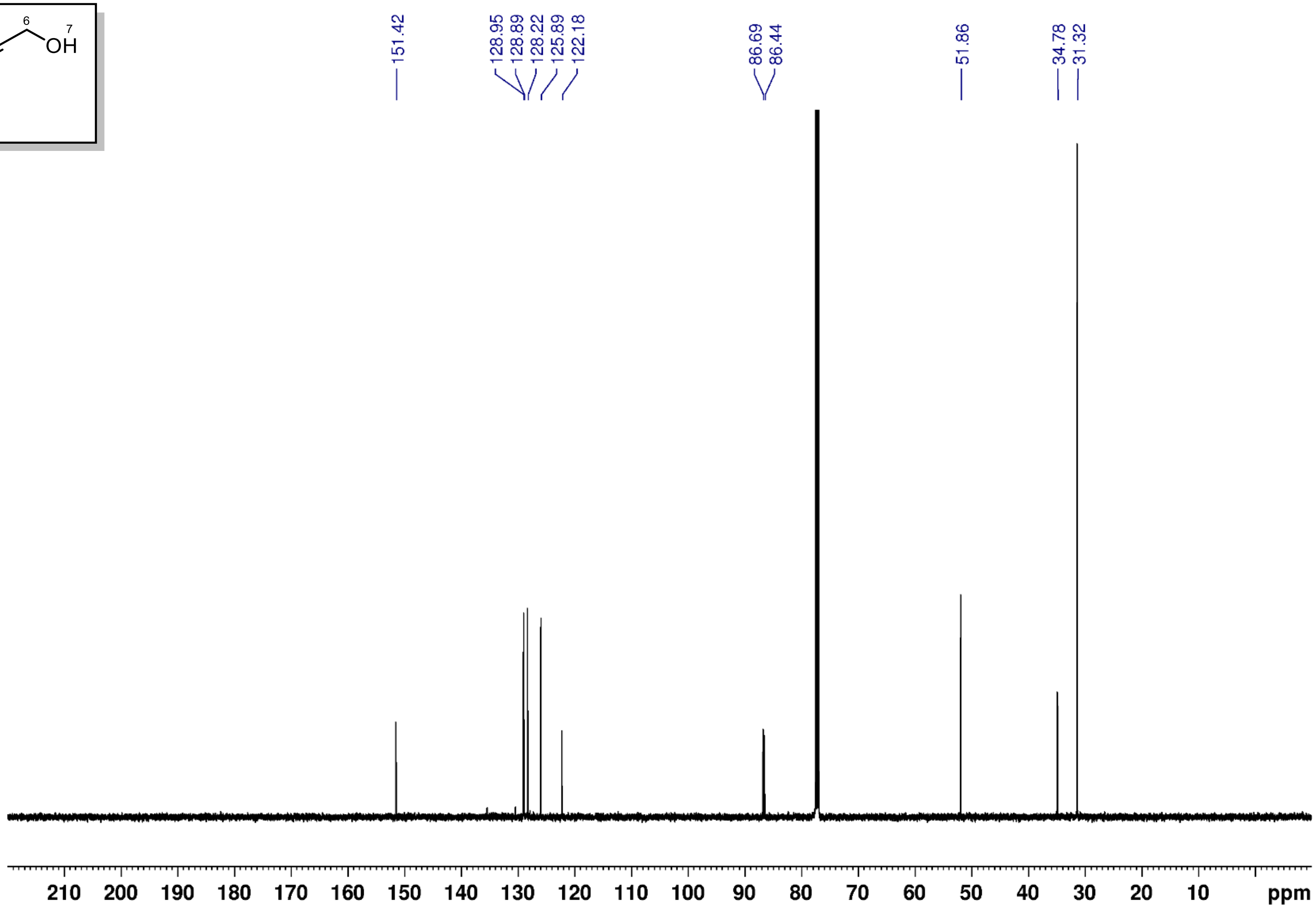

^{1}H NMR (400 MHz, $CDCl_3$) for 3-(3-bromophenyl)prop-2-yn-1-ol

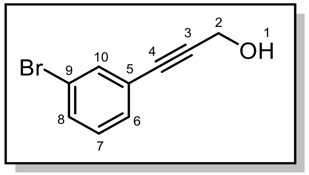

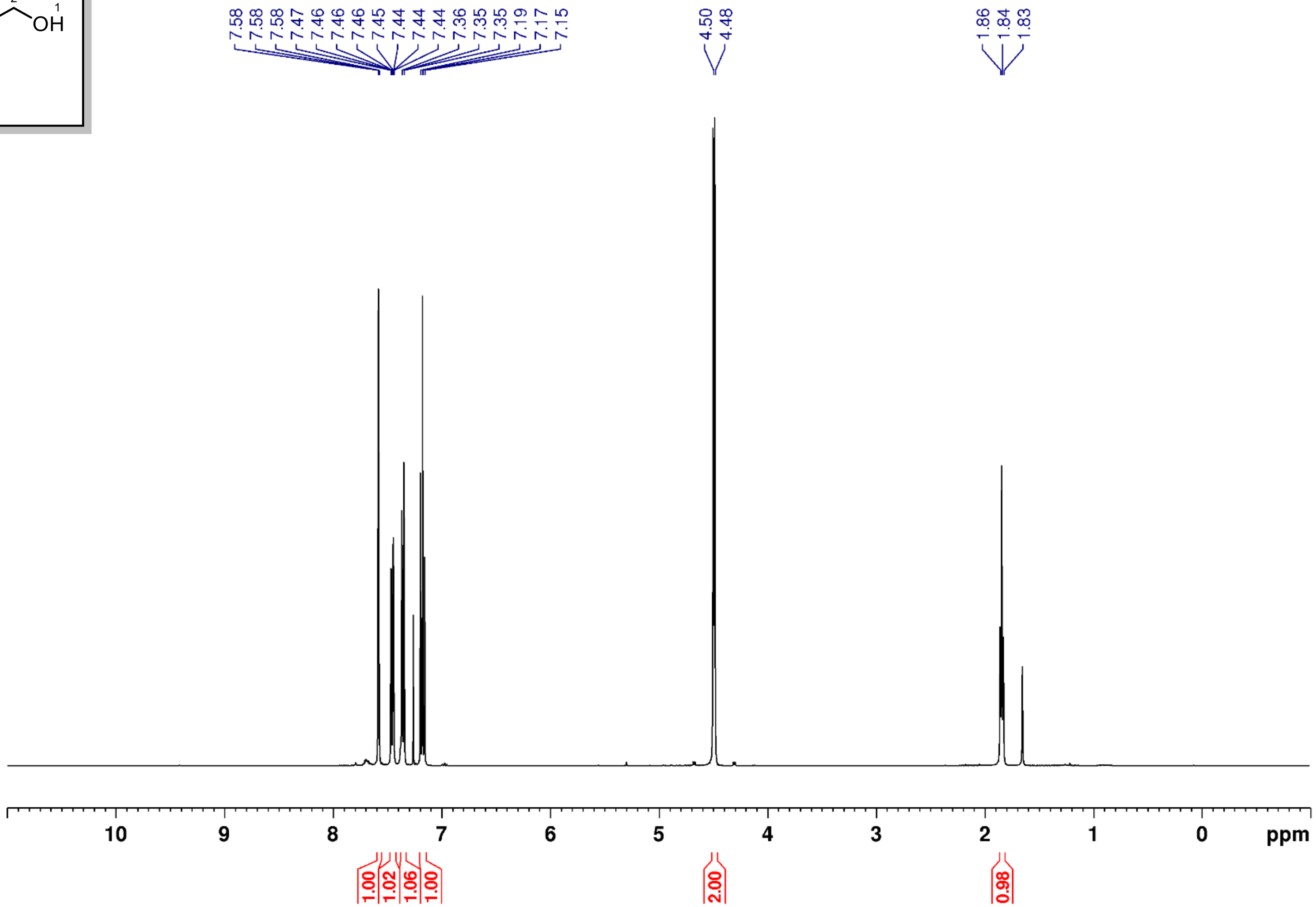

^{13}C NMR (101 MHz, $CDCl_3$) for 3-(3-bromophenyl)prop-2-yn-1-ol

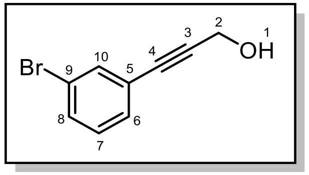

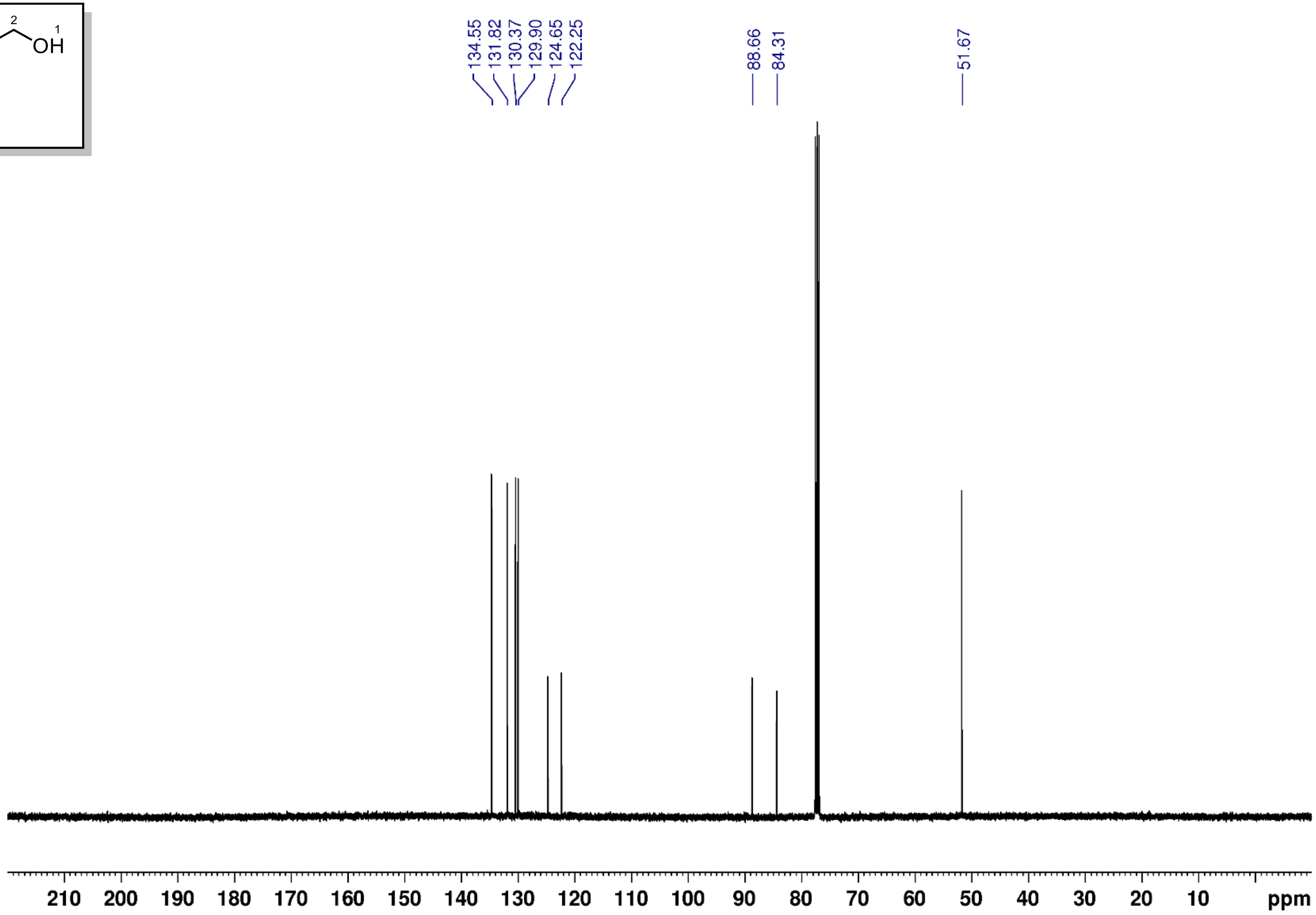

^{1}H NMR (400 MHz, CDCl$_3$) for ethyl 3-(3-hydroxyprop-1-yn-1-yl)benzoate

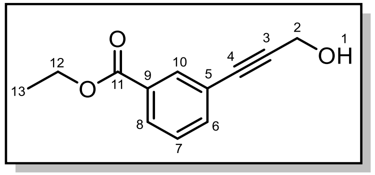

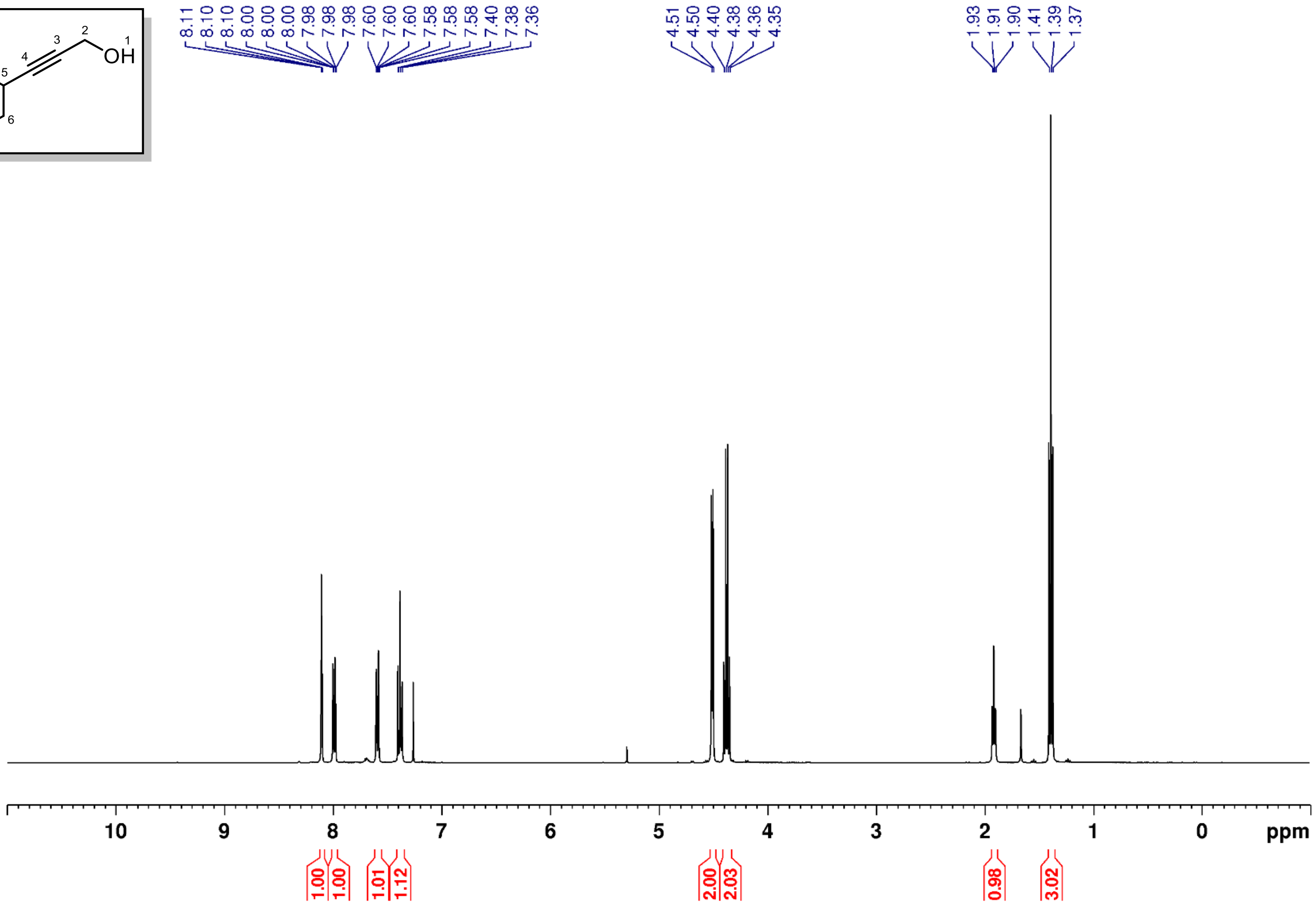

^{13}C NMR (101 MHz, CDCl$_3$) for ethyl 3-(3-hydroxyprop-1-yn-1-yl)benzoate

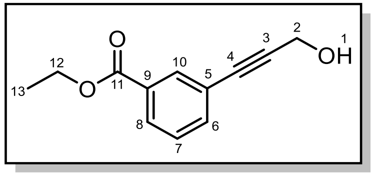

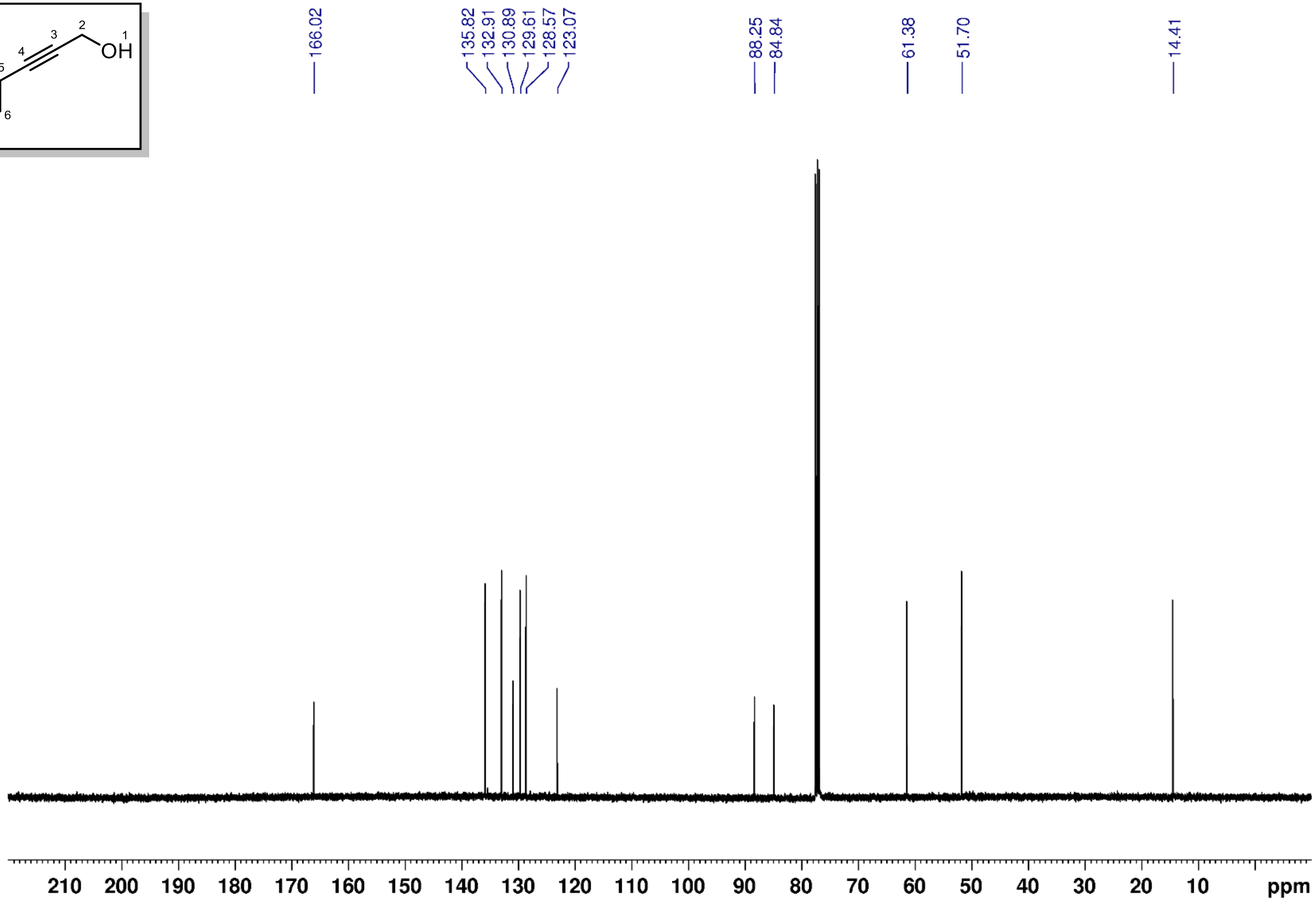

^{1}H NMR (700 MHz, $CDCl_3$) for 3-(3,4-dichlorophenyl)prop-2-yn-1-ol

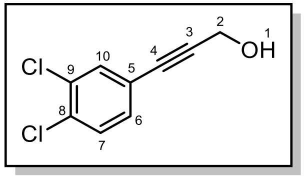

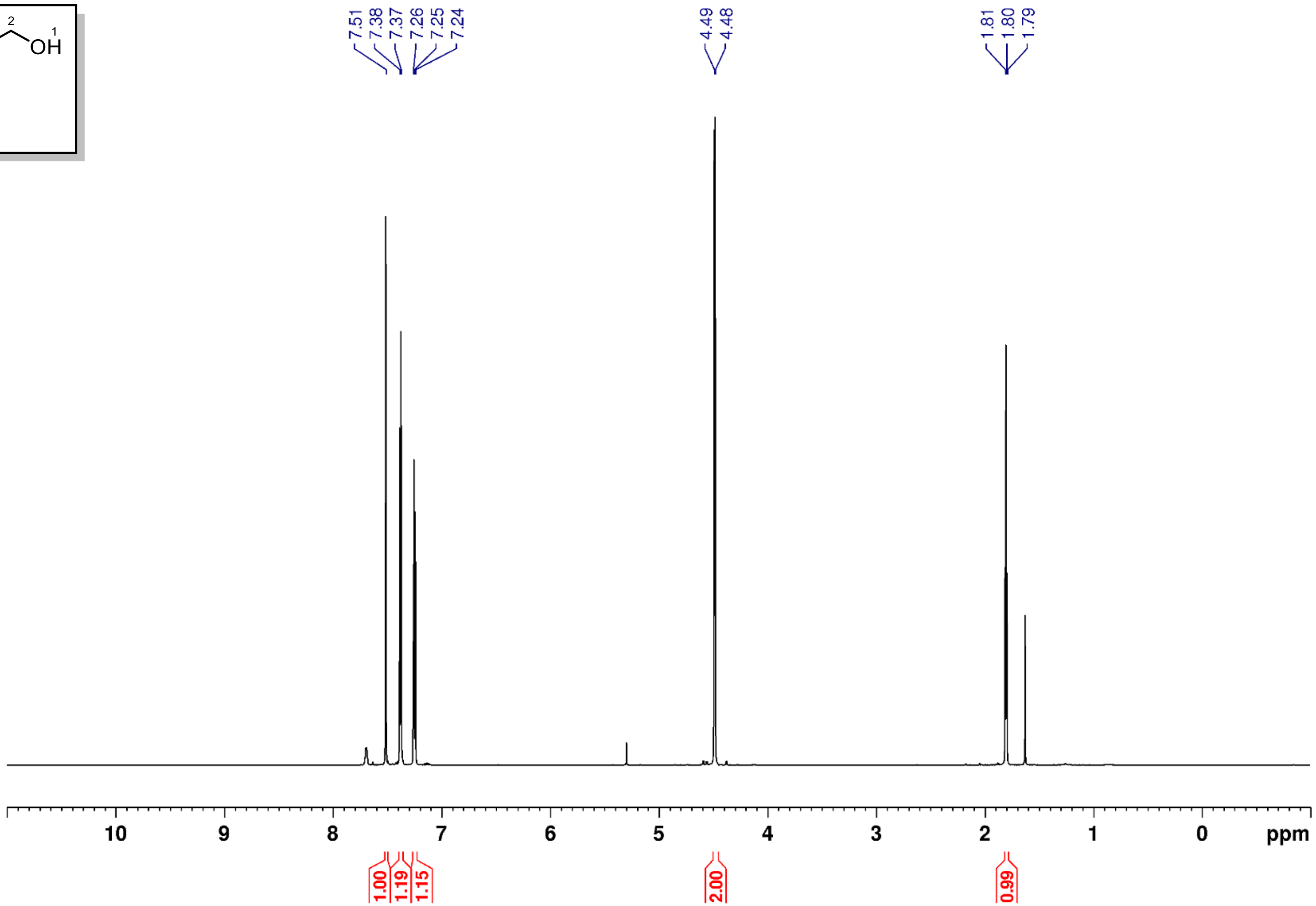

^{13}C NMR (176 MHz, CDCl$_3$) for 3-(3,4-dichlorophenyl)prop-2-yn-1-ol

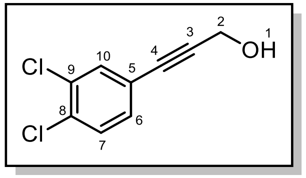

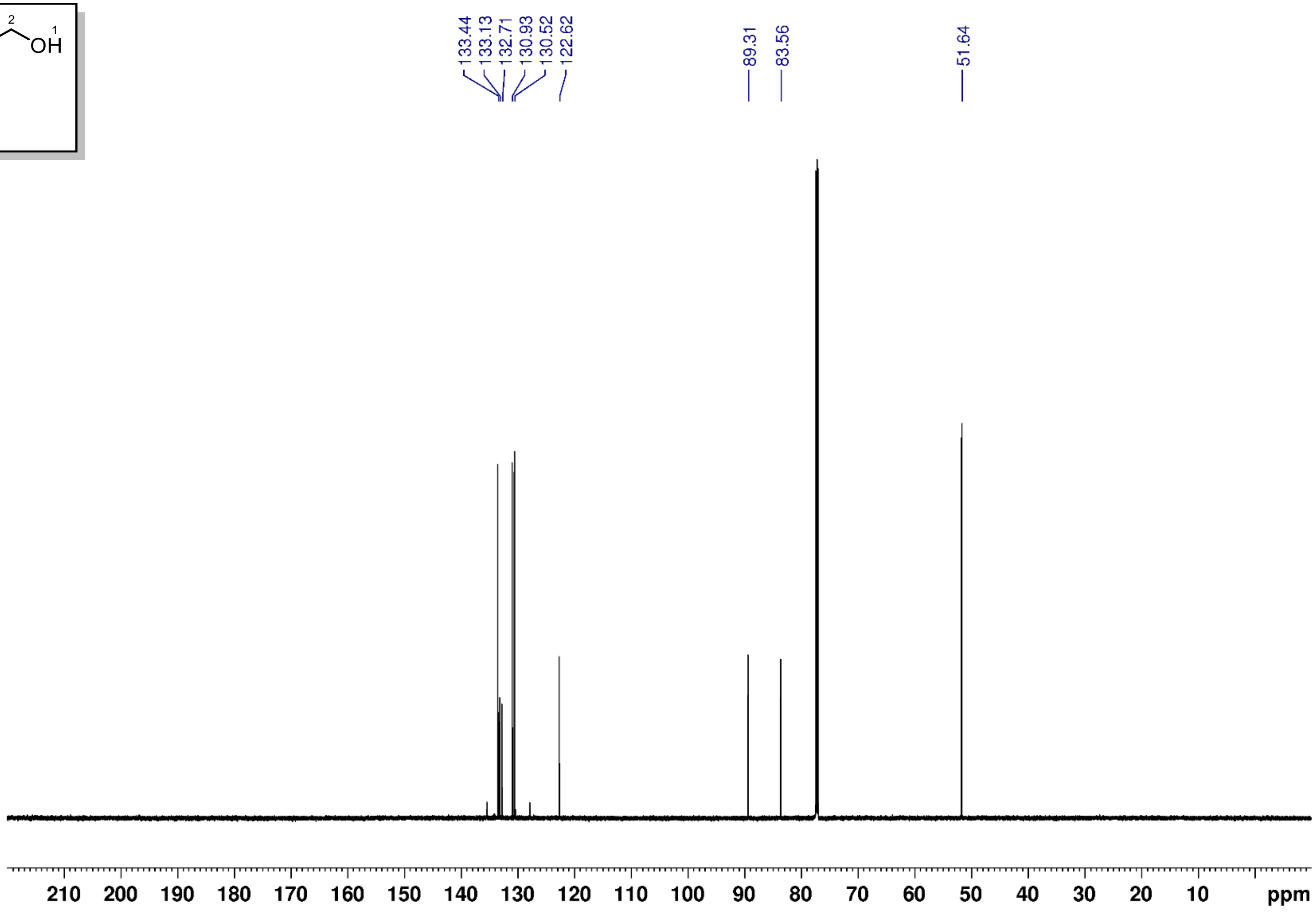

^{1}H NMR (400 MHz, $CDCl_3$) for 3-(4-(*tert*-butyl)phenyl)prop-2-yn-1-ol

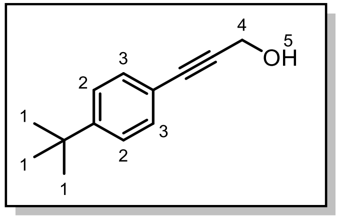

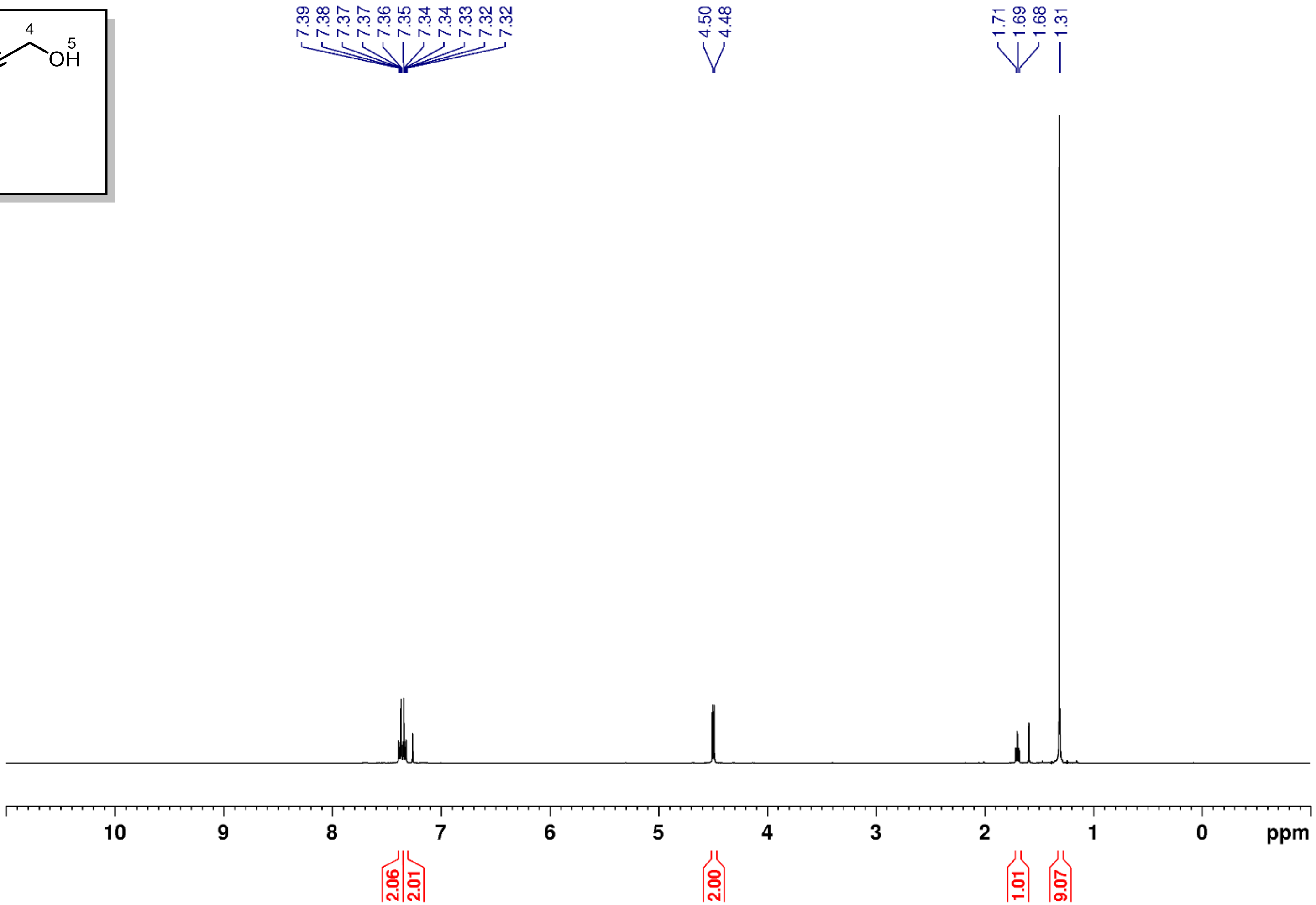

^{13}C NMR (101 MHz, $CDCl_3$) for 3-(4-(*tert*-butyl)phenyl)prop-2-yn-1-ol

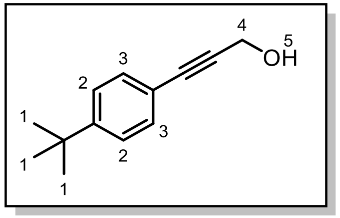

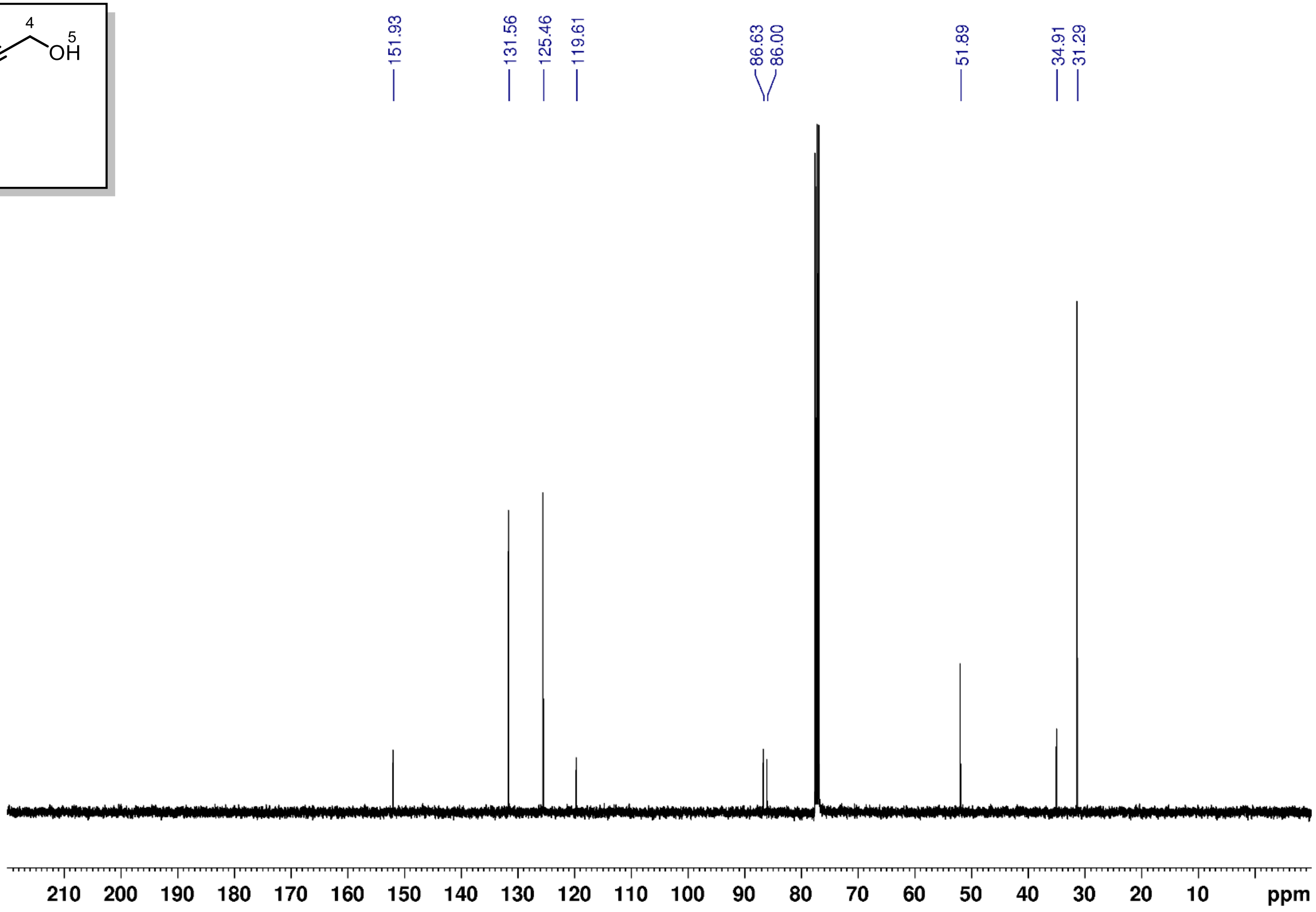

^{1}H NMR (700 MHz, $CDCl_3$) for 3-(4-(trifluoromethoxy)phenyl)prop-2-yn-1-ol

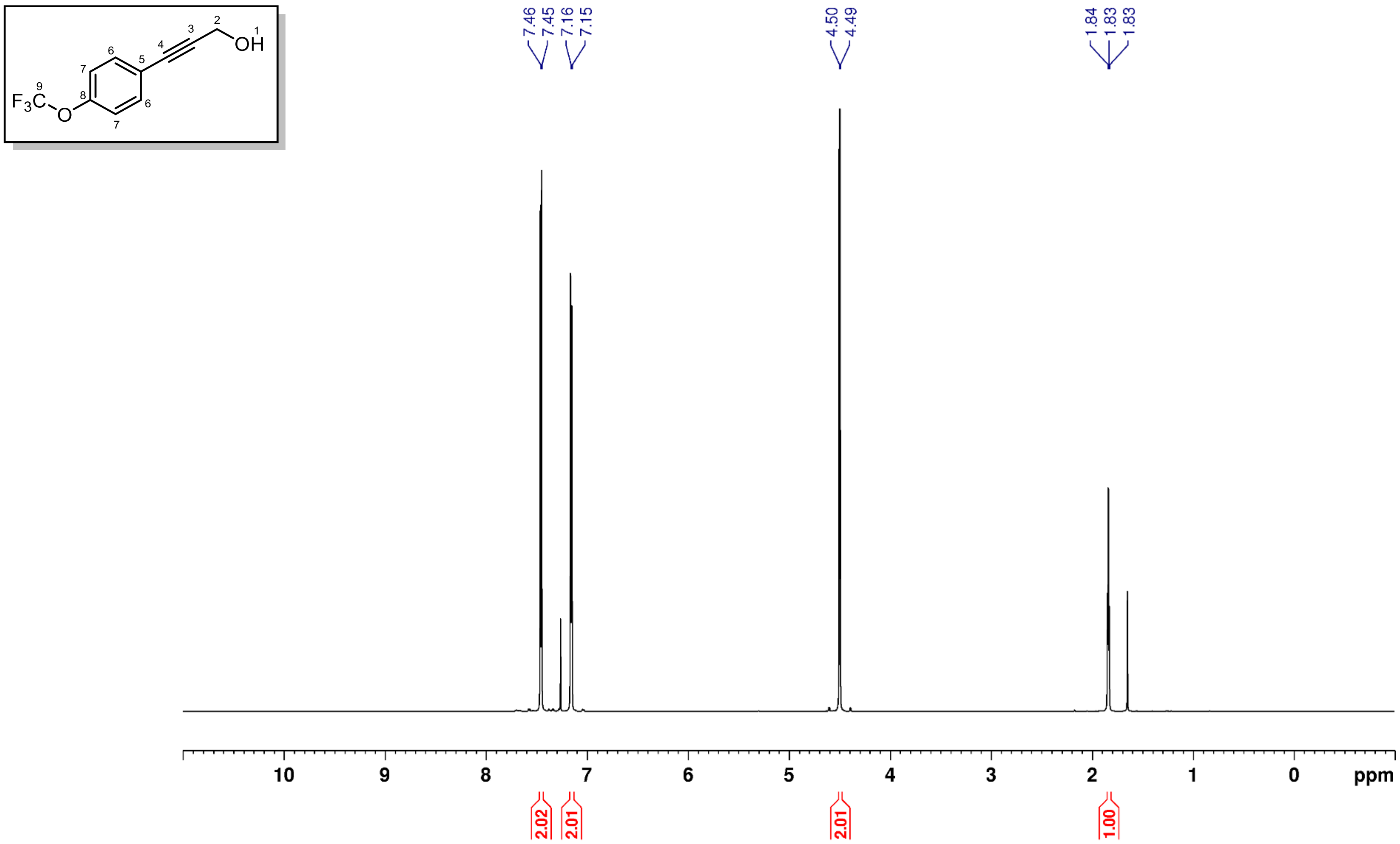

^{13}C NMR (176 MHz, CDCl$_3$) for 3-(4-(trifluoromethoxy)phenyl)prop-2-yn-1-ol

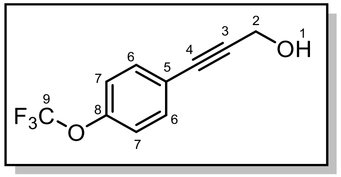

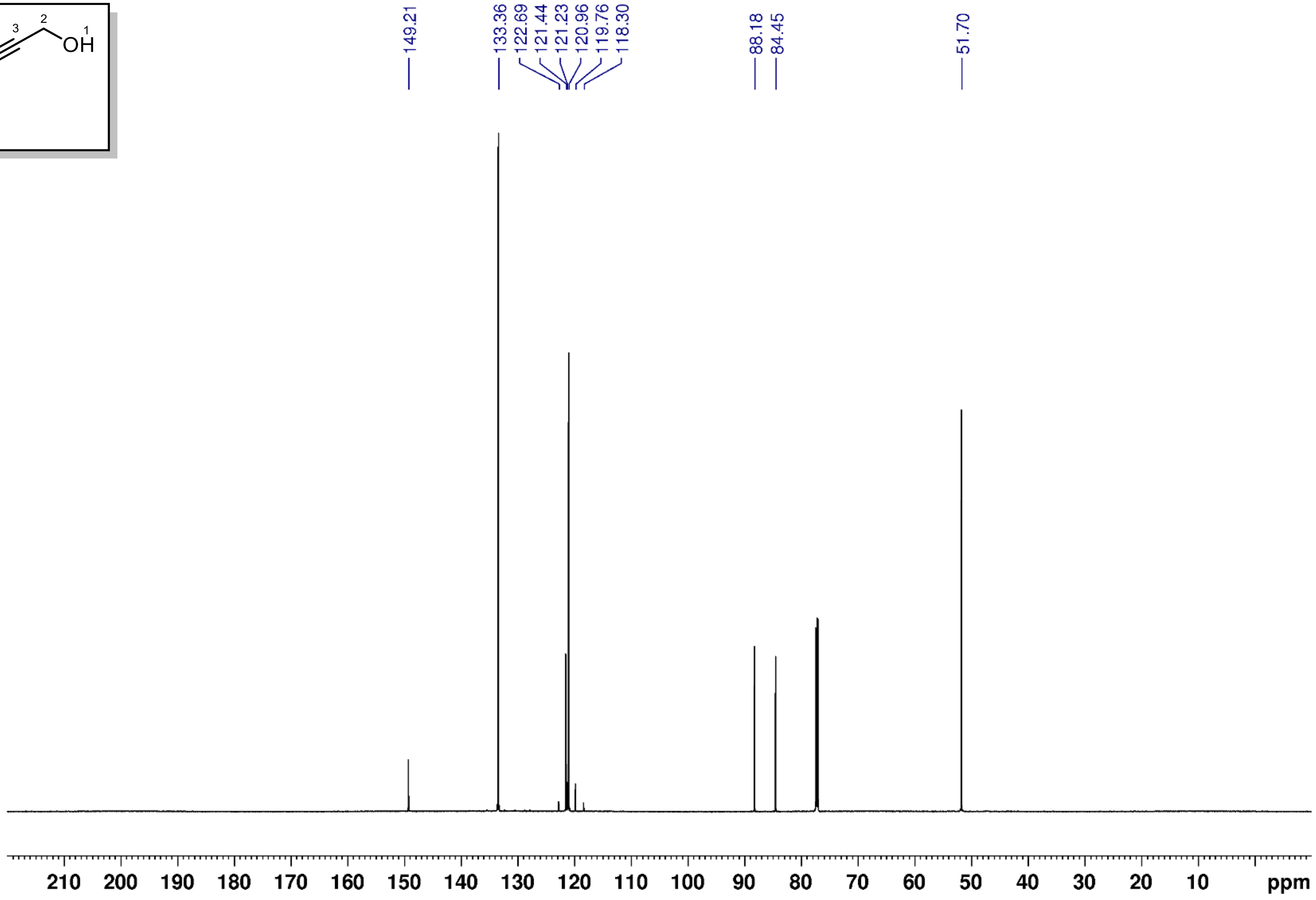

^{19}F NMR (376 MHz, CDCl$_3$) for 3-(2-fluorophenyl)prop-2-yn-1-ol

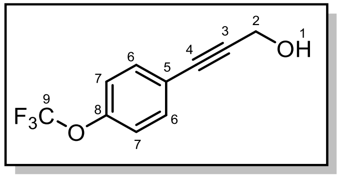

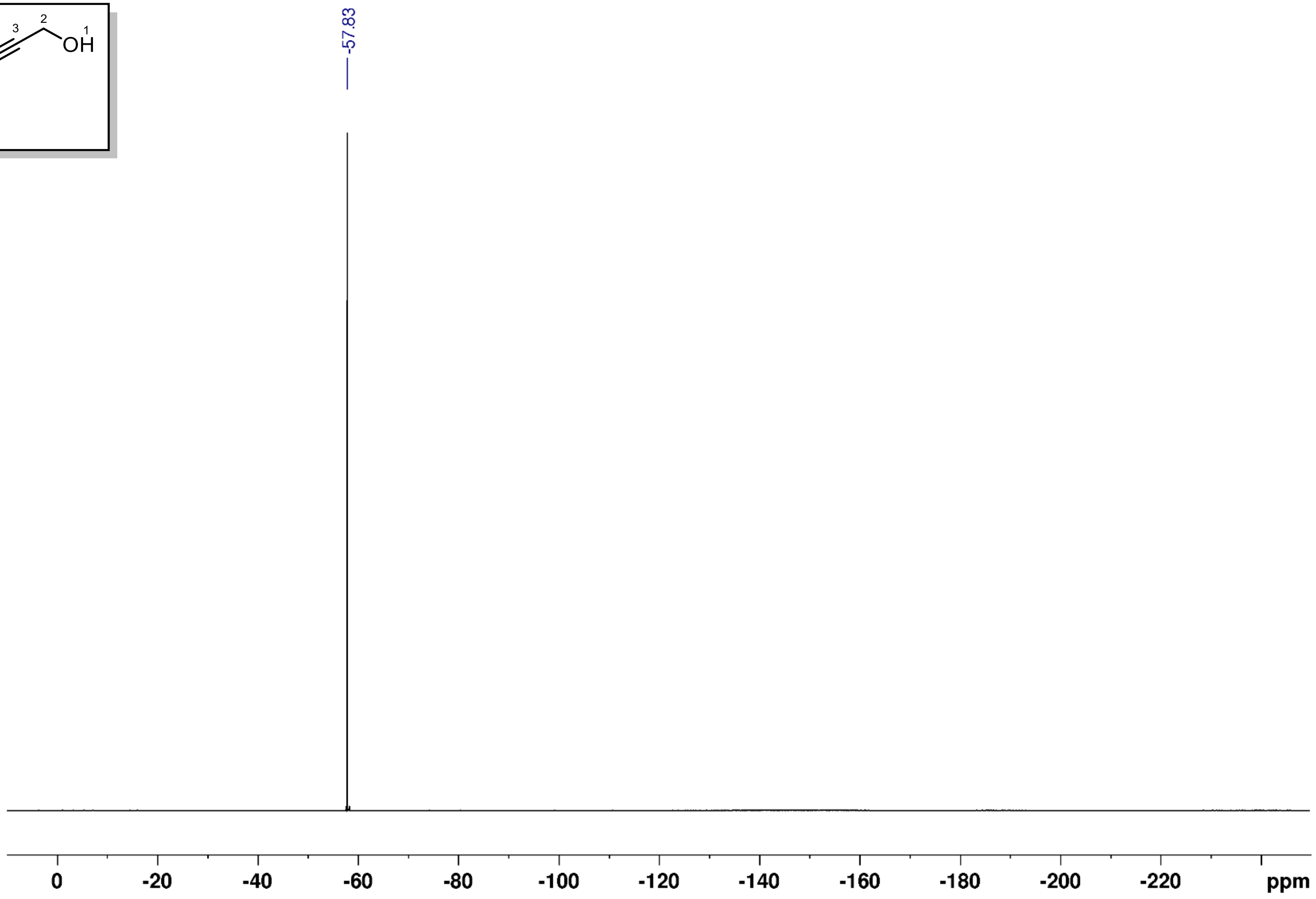

^{1}H NMR (400 MHz, CDCl$_3$) for 3-(4-chlorophenyl)prop-2-yn-1-ol

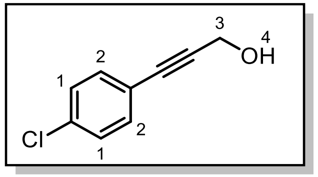

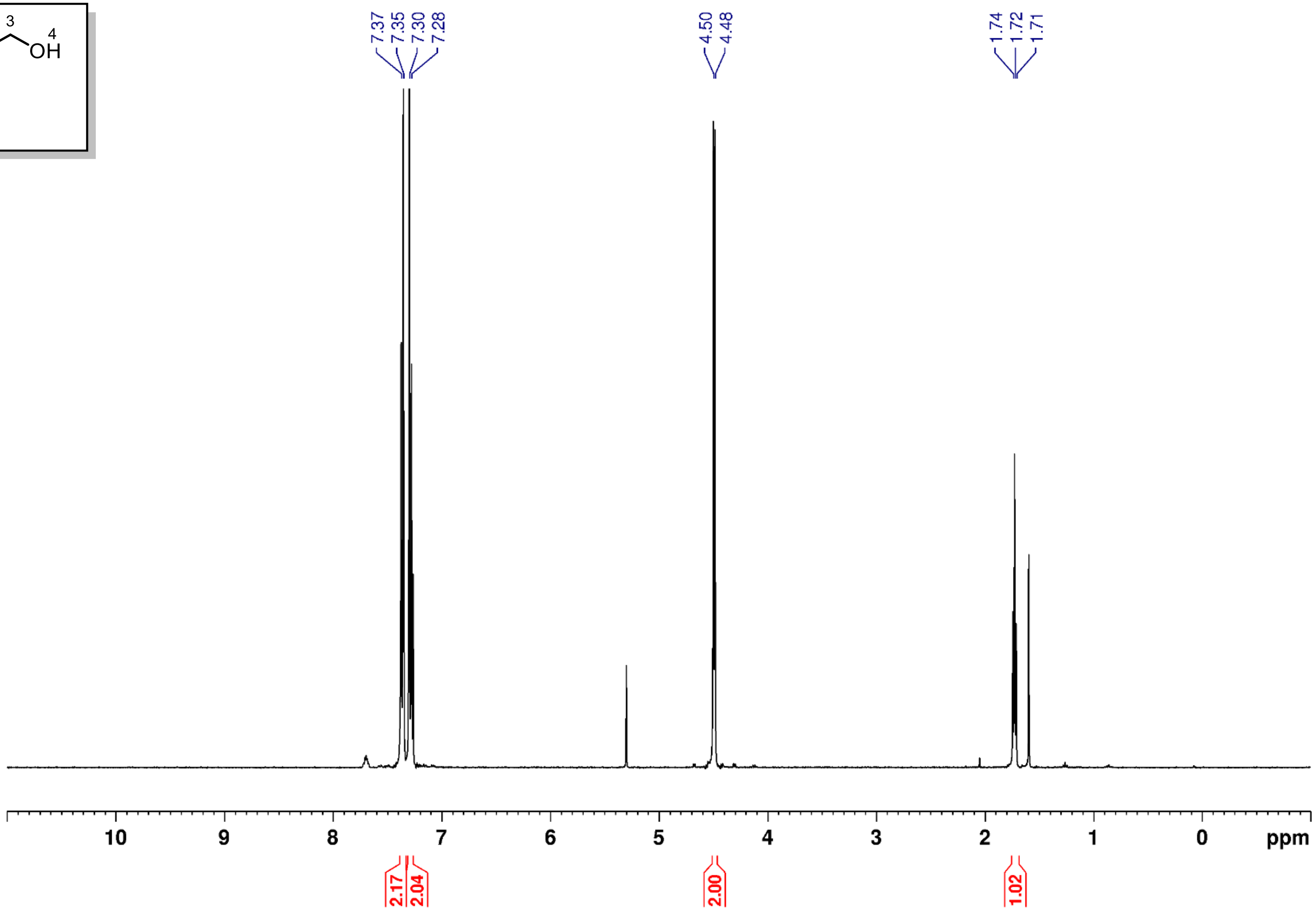

^{13}C NMR (101 MHz, CDCl$_3$) for 3-(4-chlorophenyl)prop-2-yn-1-ol

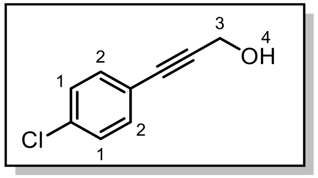

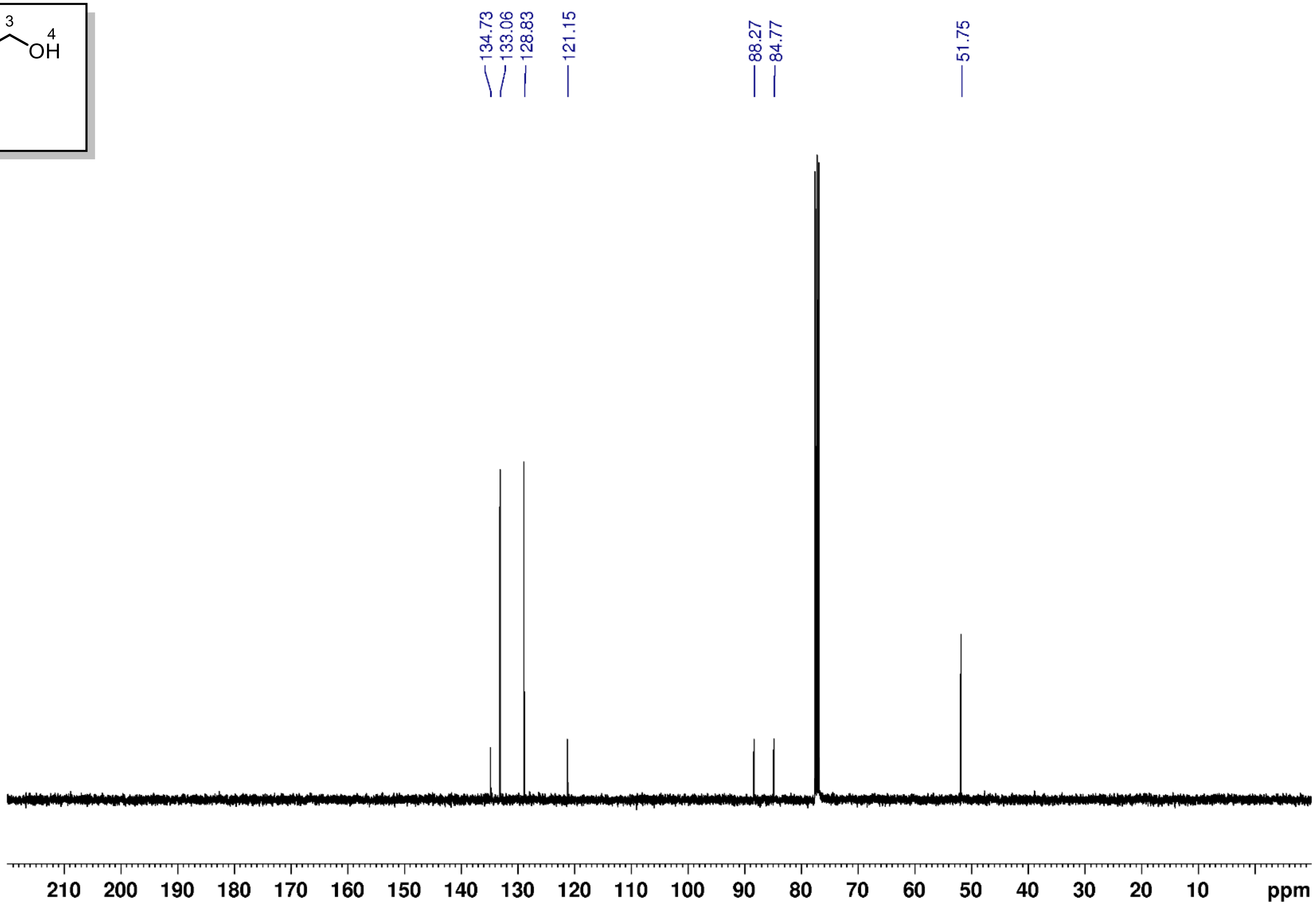

^{1}**H NMR (700 MHz, CDCl$_3$)** for 3-(4-(trifluoromethyl)phenyl)prop-2-yn-1-ol

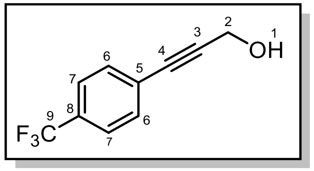

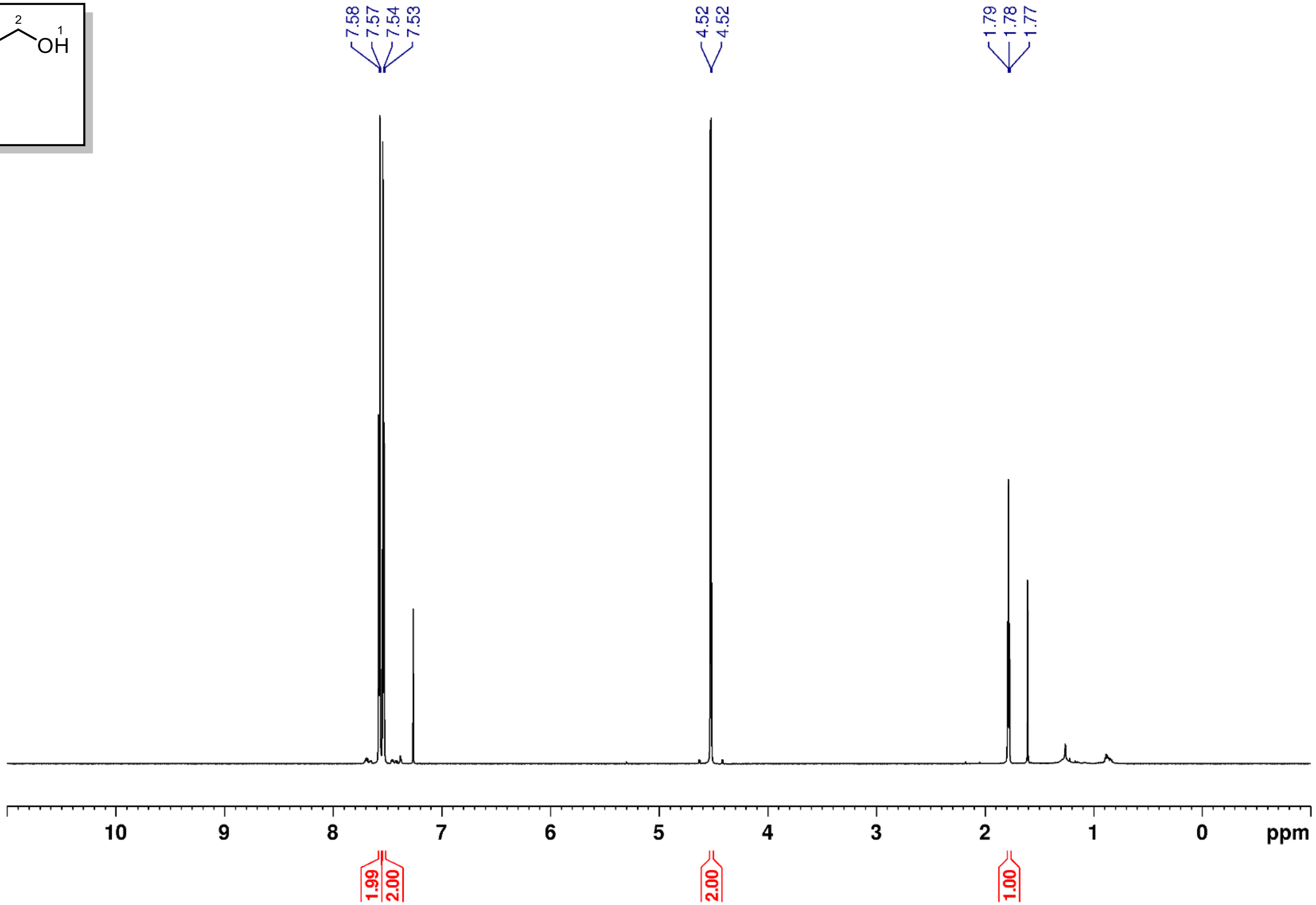

^{13}C NMR (176 MHz, $CDCl_3$) for 3-(4-(trifluoromethyl)phenyl)prop-2-yn-1-ol

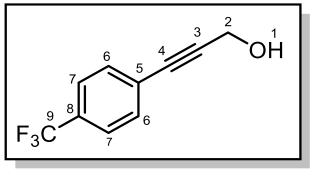

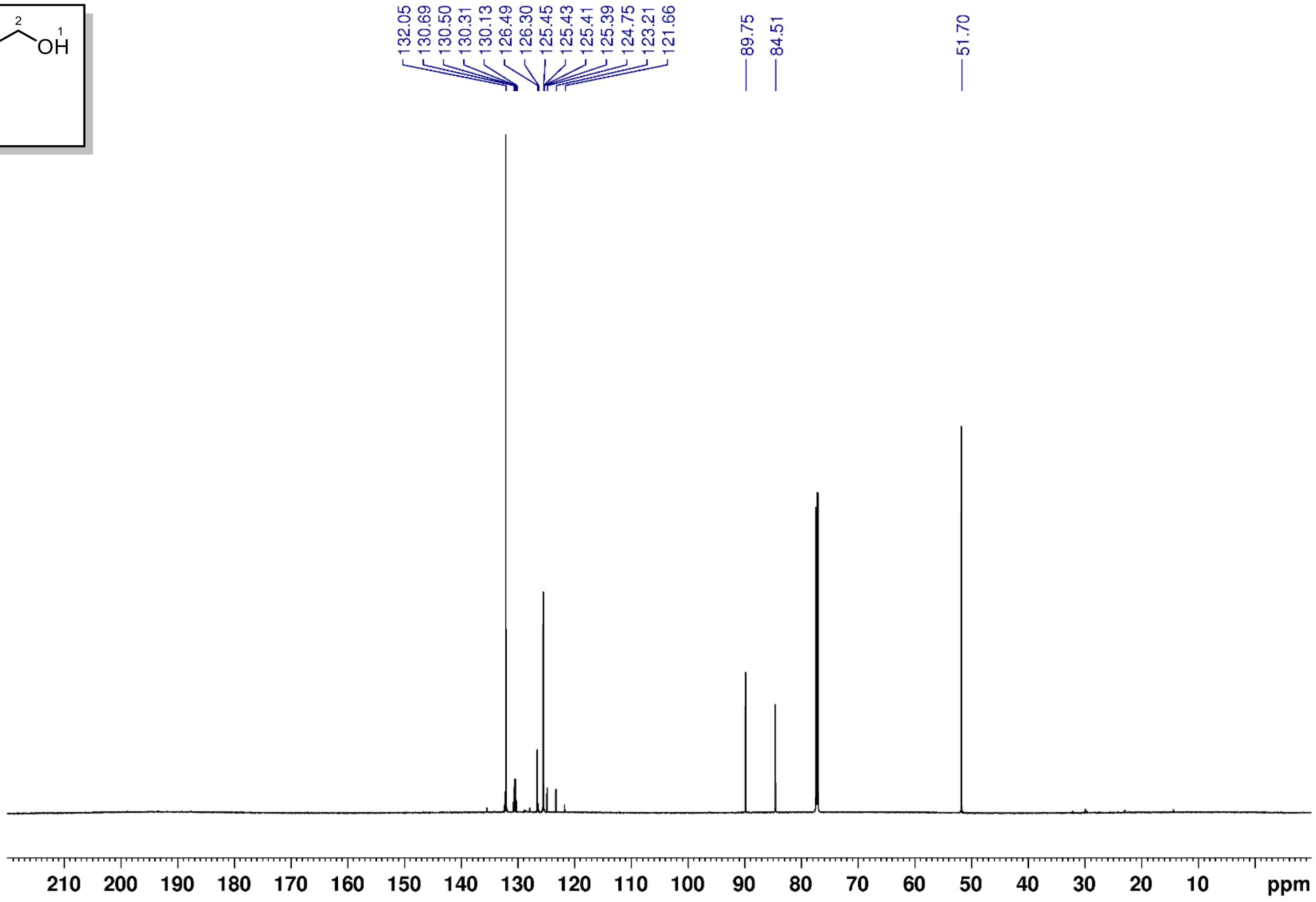

^{19}F NMR (376 MHz, CDCl$_3$) for 3-(4-(trifluoromethyl)phenyl)prop-2-yn-1-ol

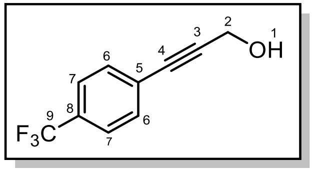

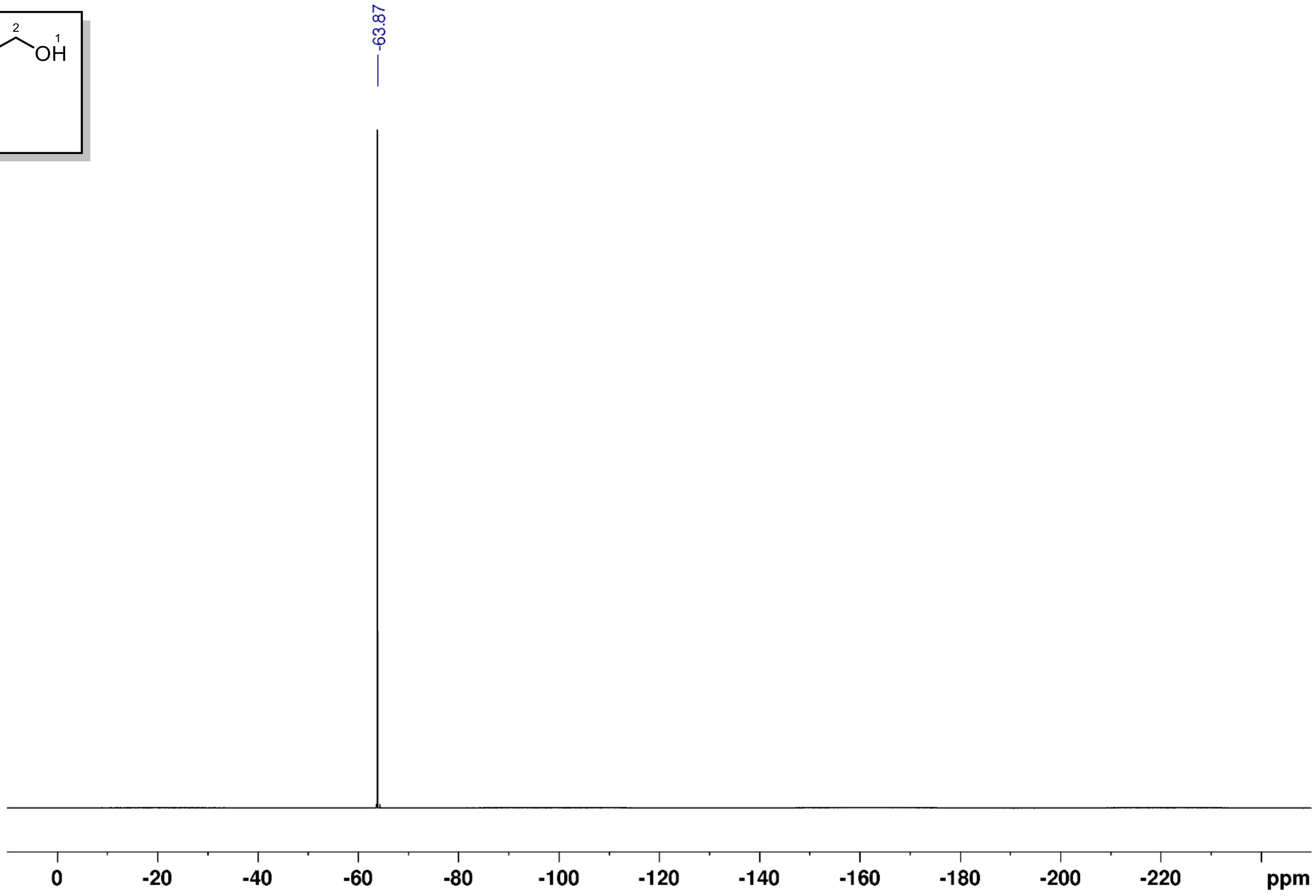

^{1}H NMR (500 MHz, CDCl$_3$) for 3-(naphthalen-1-yl)prop-2-yn-1-ol

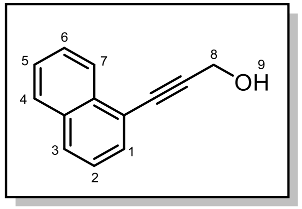

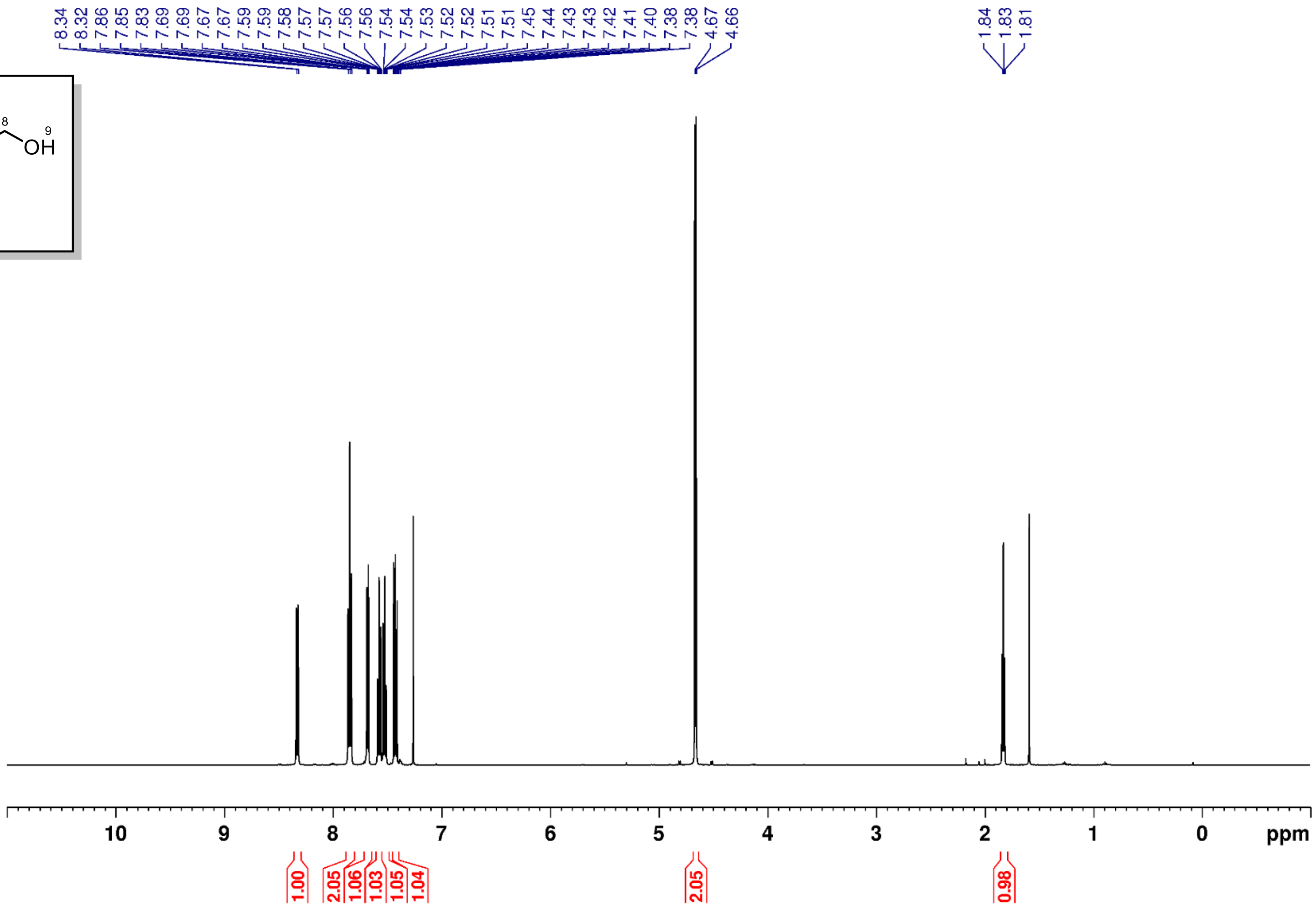

^{13}C NMR (126 MHz, $CDCl_3$) for 3-(naphthalen-1-yl)prop-2-yn-1-ol

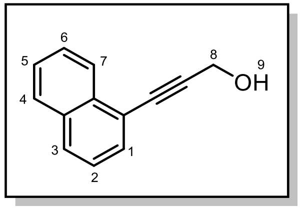

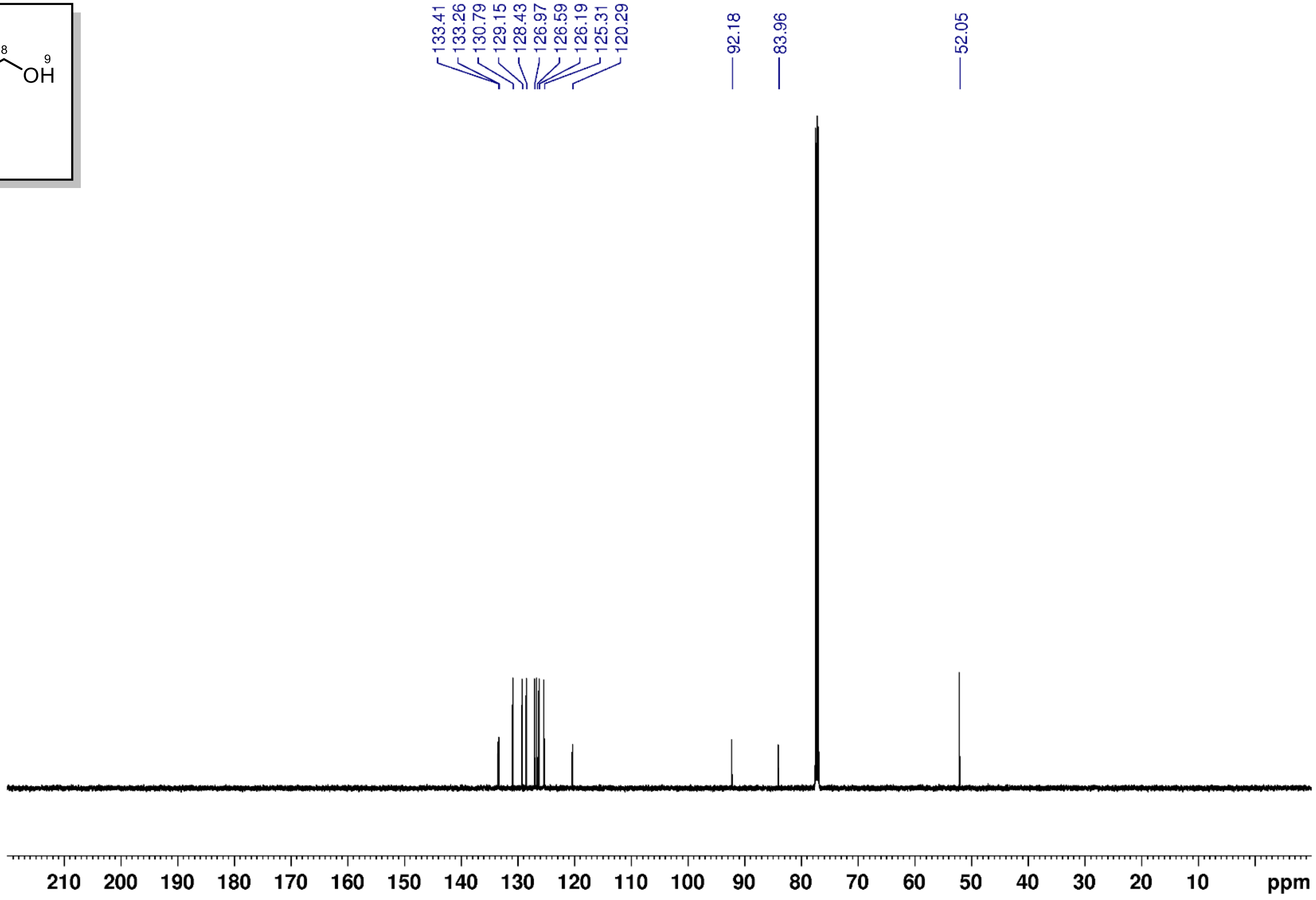

^{1}H NMR (700 MHz, $(CD_3)_2SO$) for 5-iodo-1*H*-indole-3-carbaldehyde

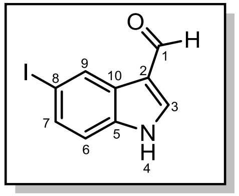

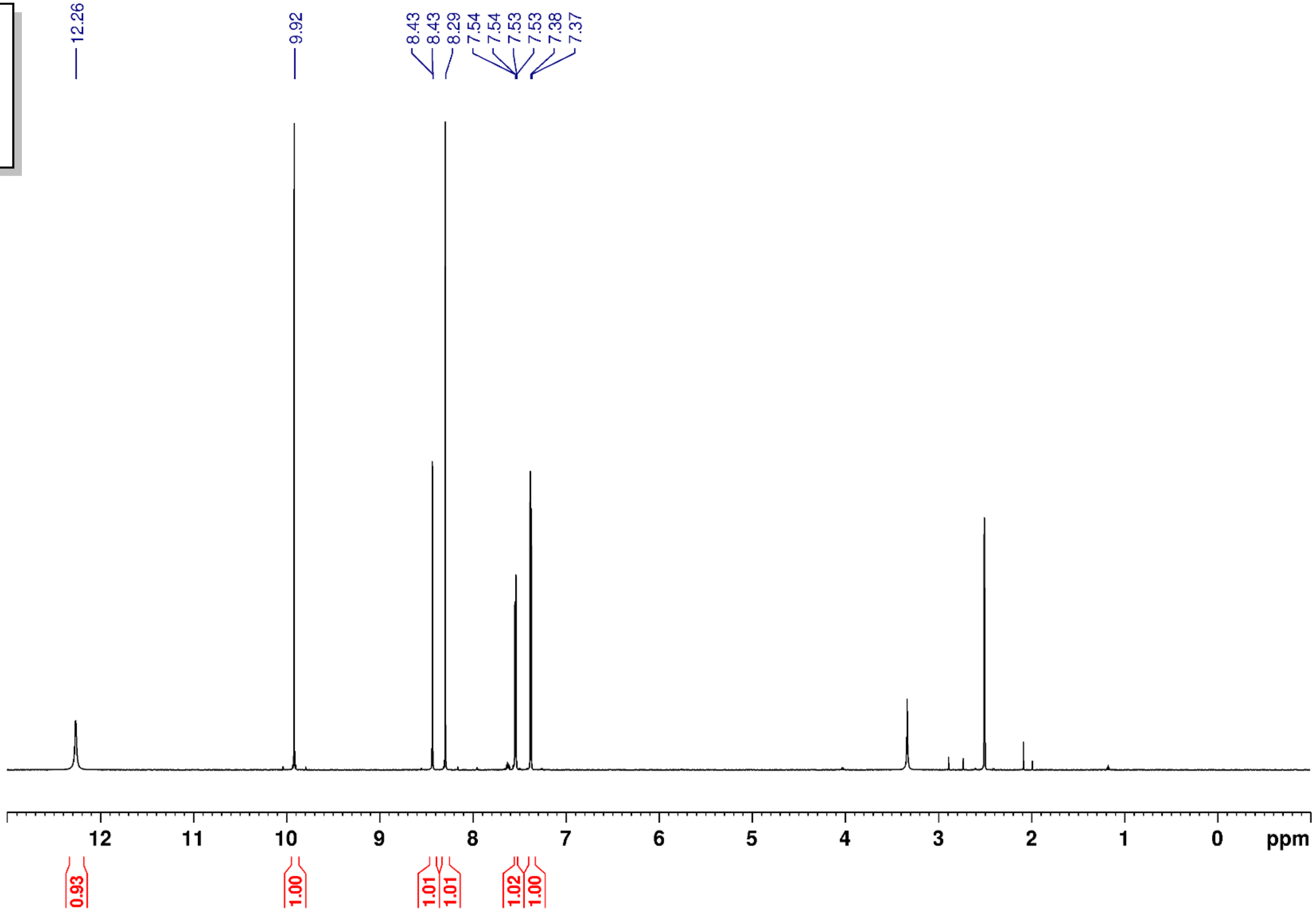

^{13}C NMR (176 MHz, $(CD_3)_2SO$) for 5-iodo-1*H*-indole-3-carbaldehyde

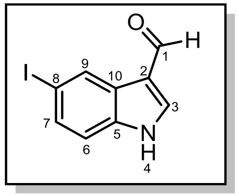

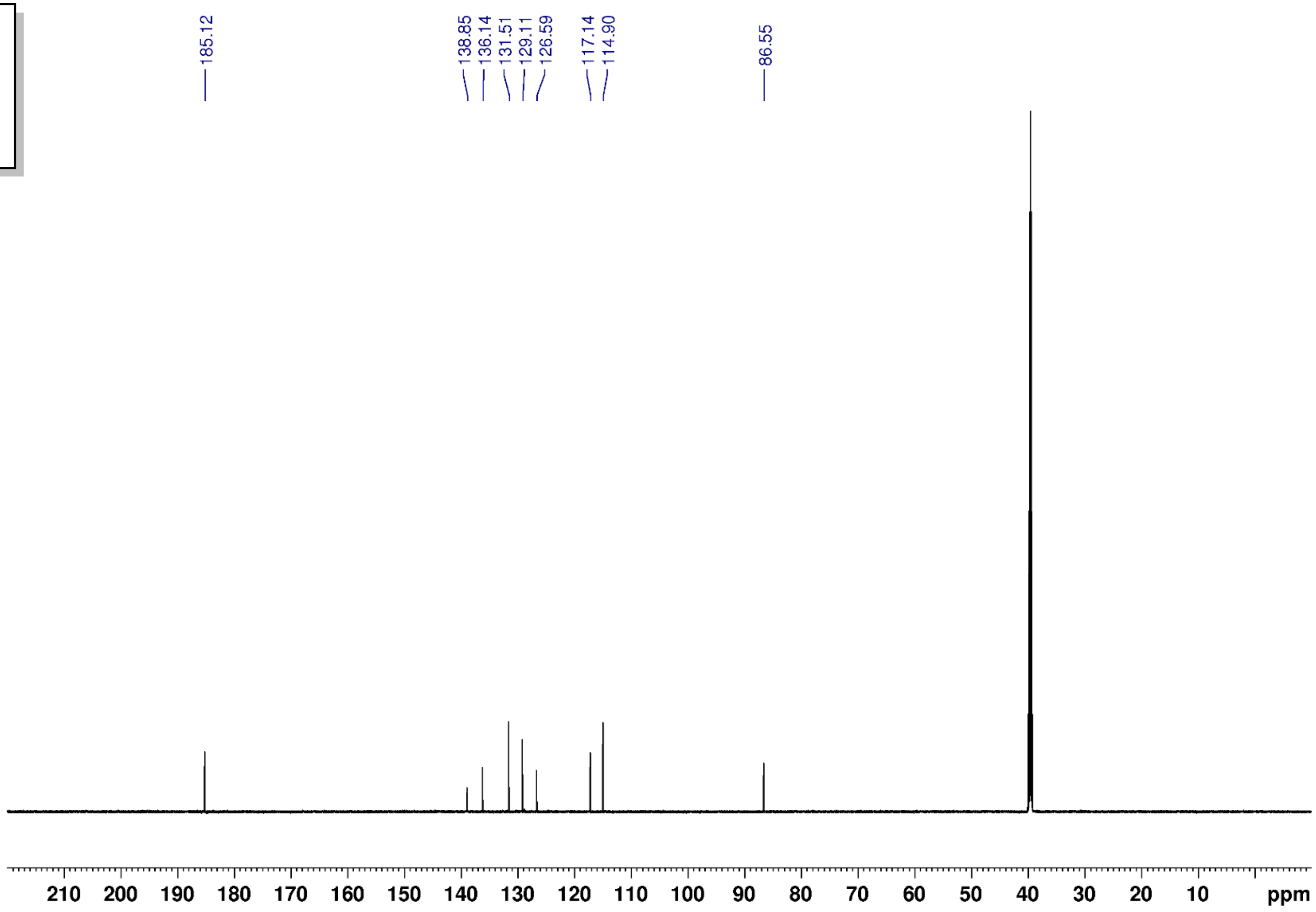

^{1}H NMR (700 MHz, $CDCl_3$) for 5-iodo-1-tosyl-1*H*-indole-3-carbaldehyde

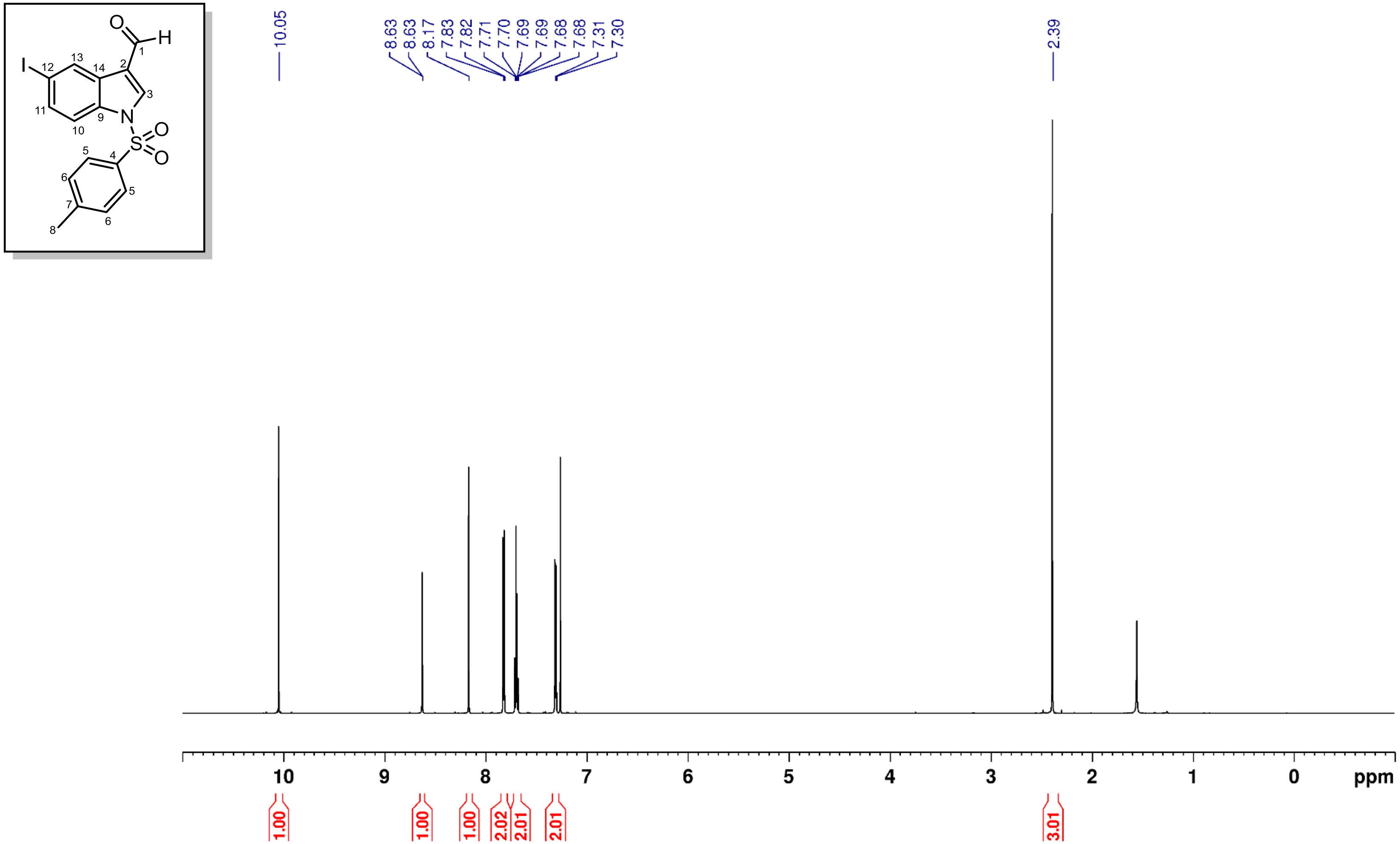

^{13}C NMR (176 MHz, $CDCl_3$) for 5-iodo-1-tosyl-1*H*-indole-3-carbaldehyde

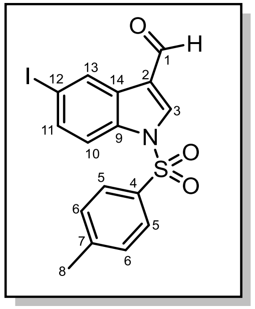

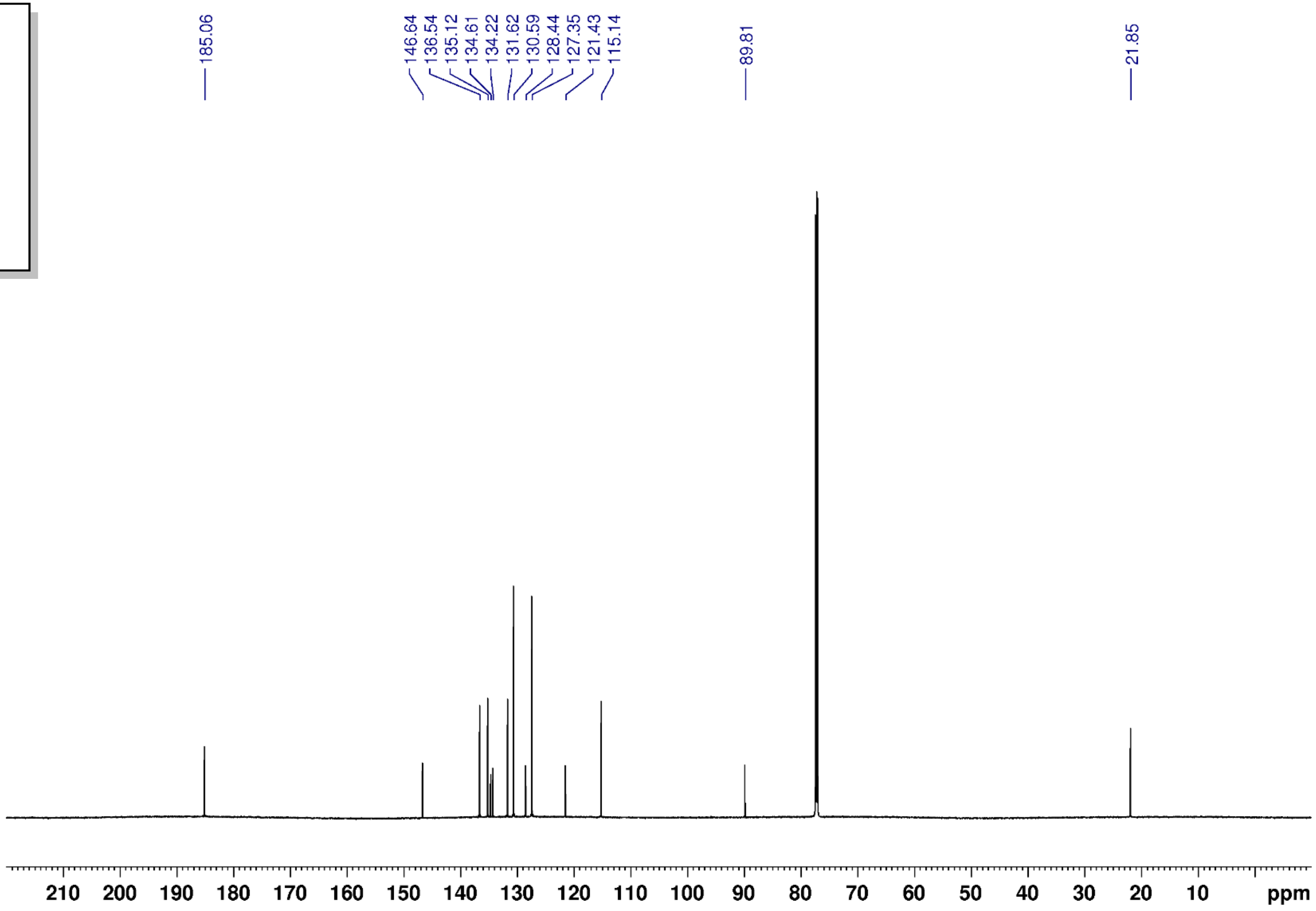

¹H NMR (700 MHz, CDCl$_3$) for methyl 5-iodo-1-tosyl-1*H*-indole-3-carboxylate

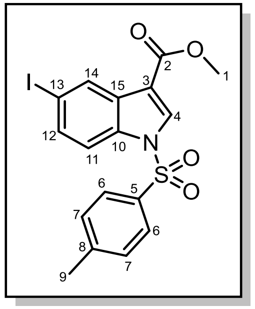

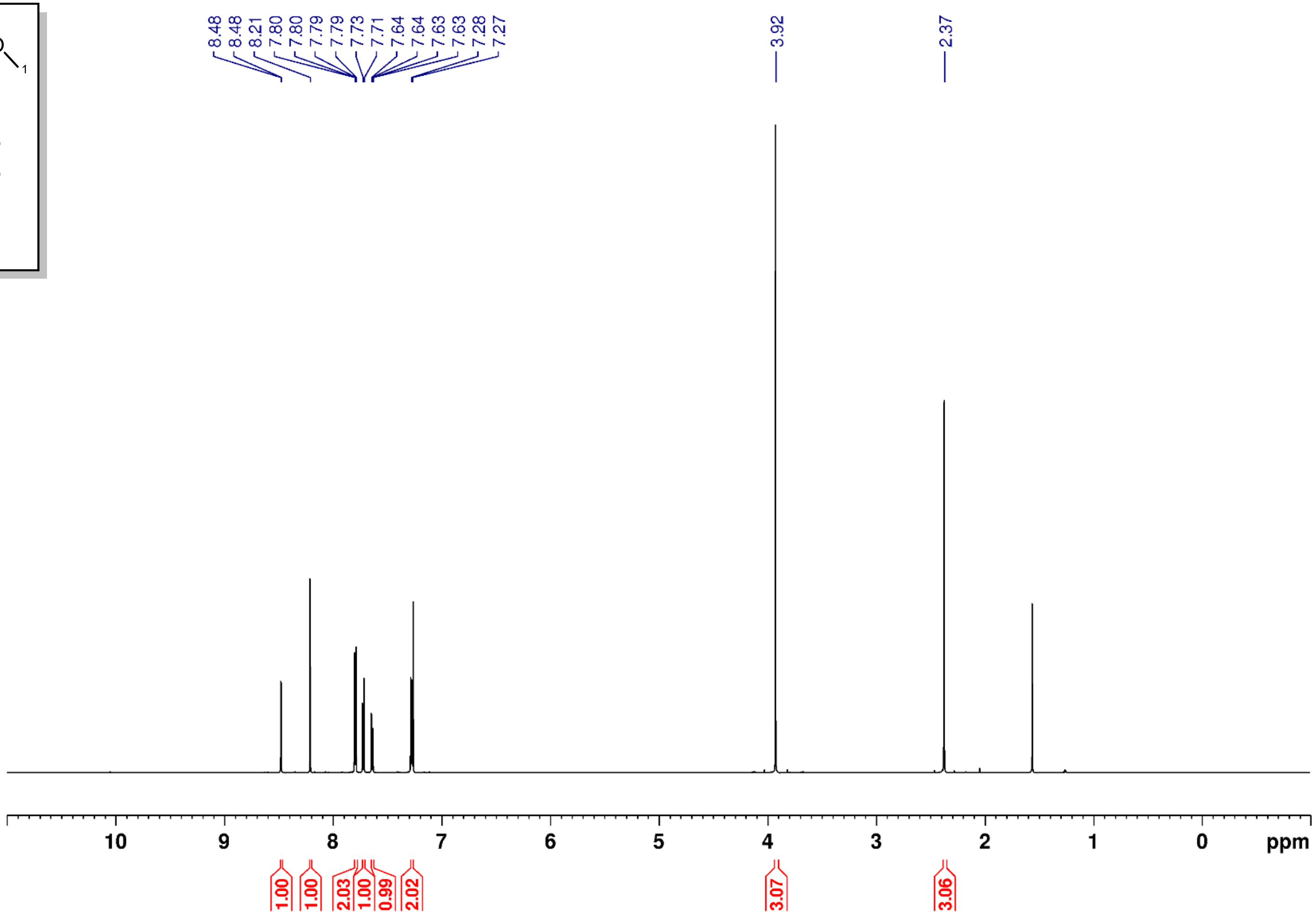

^{13}C NMR (176 MHz, $CDCl_3$) for methyl 5-iodo-1-tosyl-1*H*-indole-3-carboxylate

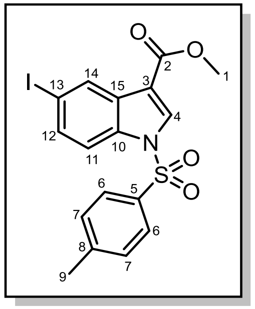

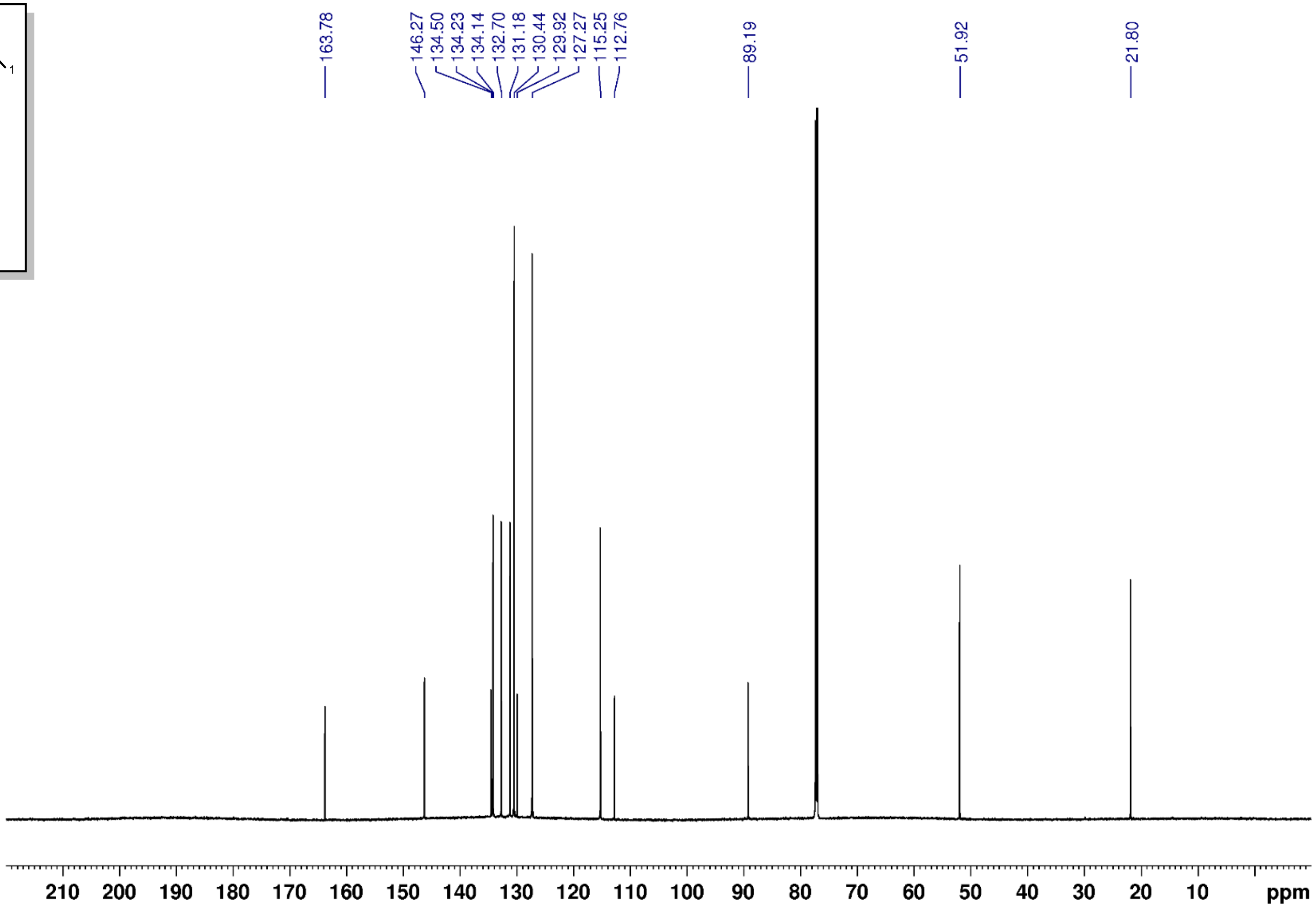

^{1}H NMR (400 MHz, CDCl$_3$) for methyl 5-(3-hydroxyprop-1-yn-1-yl)-1-tosyl-1*H*-indole-3-carboxylate

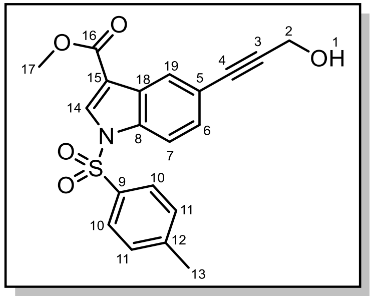

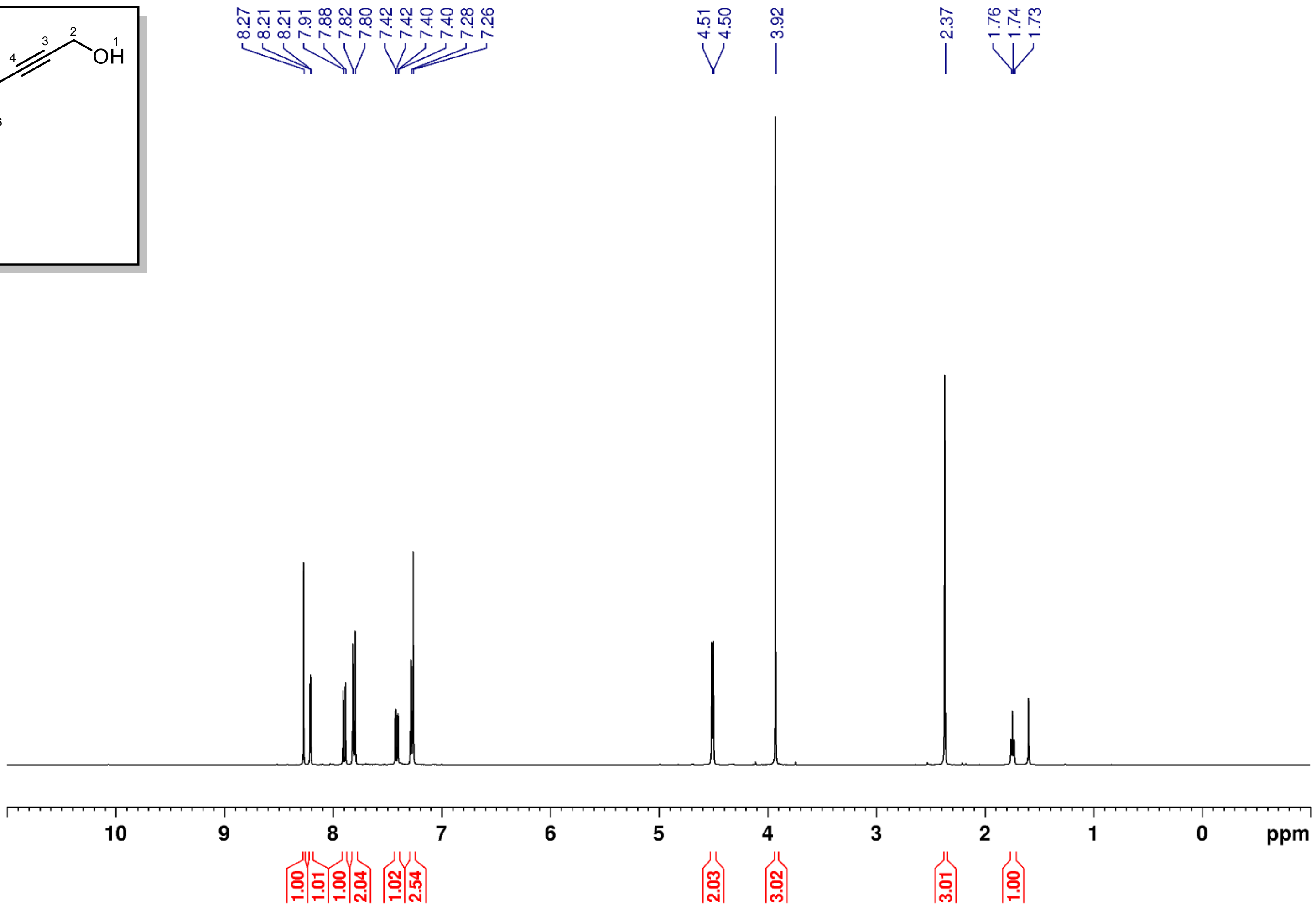

^{13}C NMR (101 MHz, $CDCl_3$) for methyl 5-(3-hydroxyprop-1-yn-1-yl)-1-tosyl-1*H*-indole-3-carboxylate

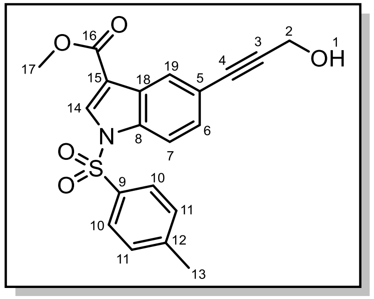

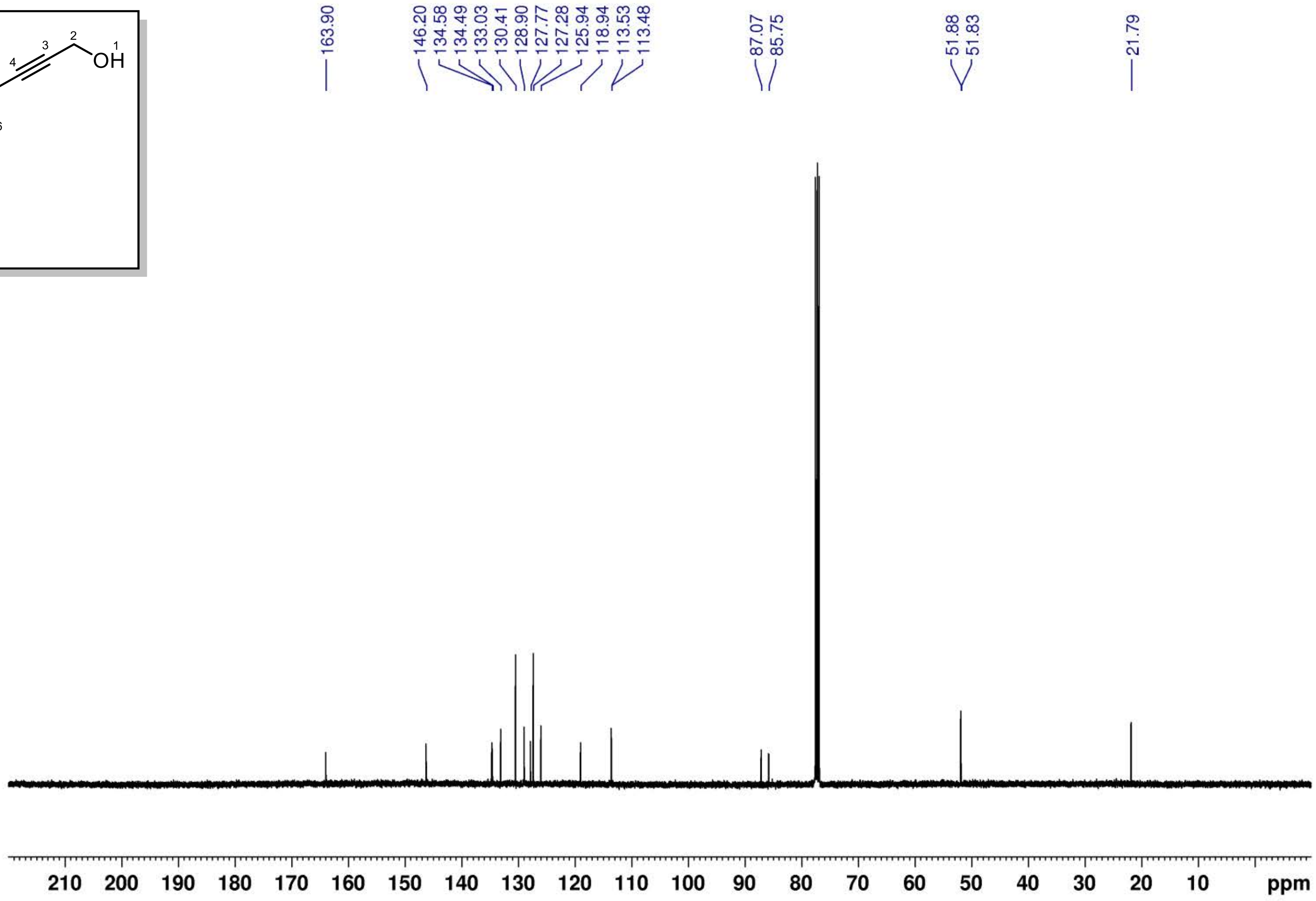

^{1}H NMR (700 MHz, CDCl$_3$) for 4-(4-(*tert*-butyl)phenyl)but-3-yn-2-ol

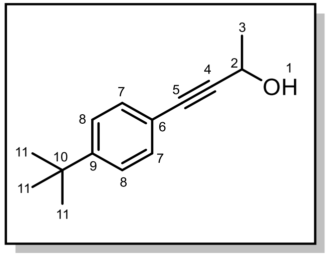

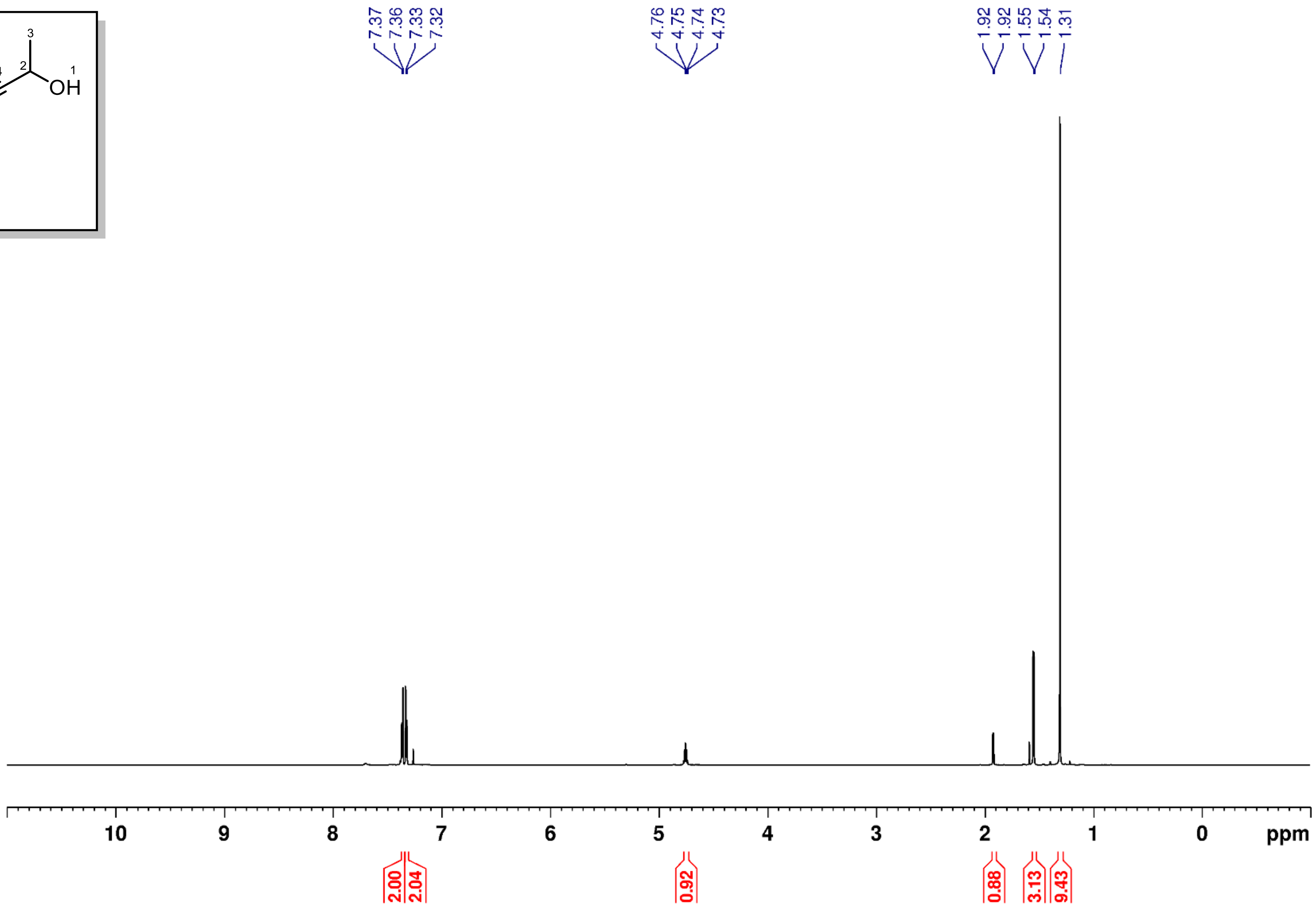

^{13}C NMR (176 MHz, $CDCl_3$) for 4-(4-(*tert*-butyl)phenyl)but-3-yn-2-ol

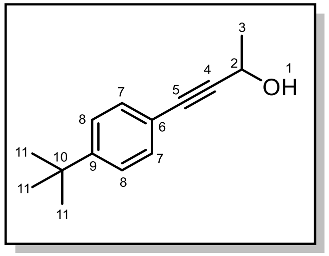

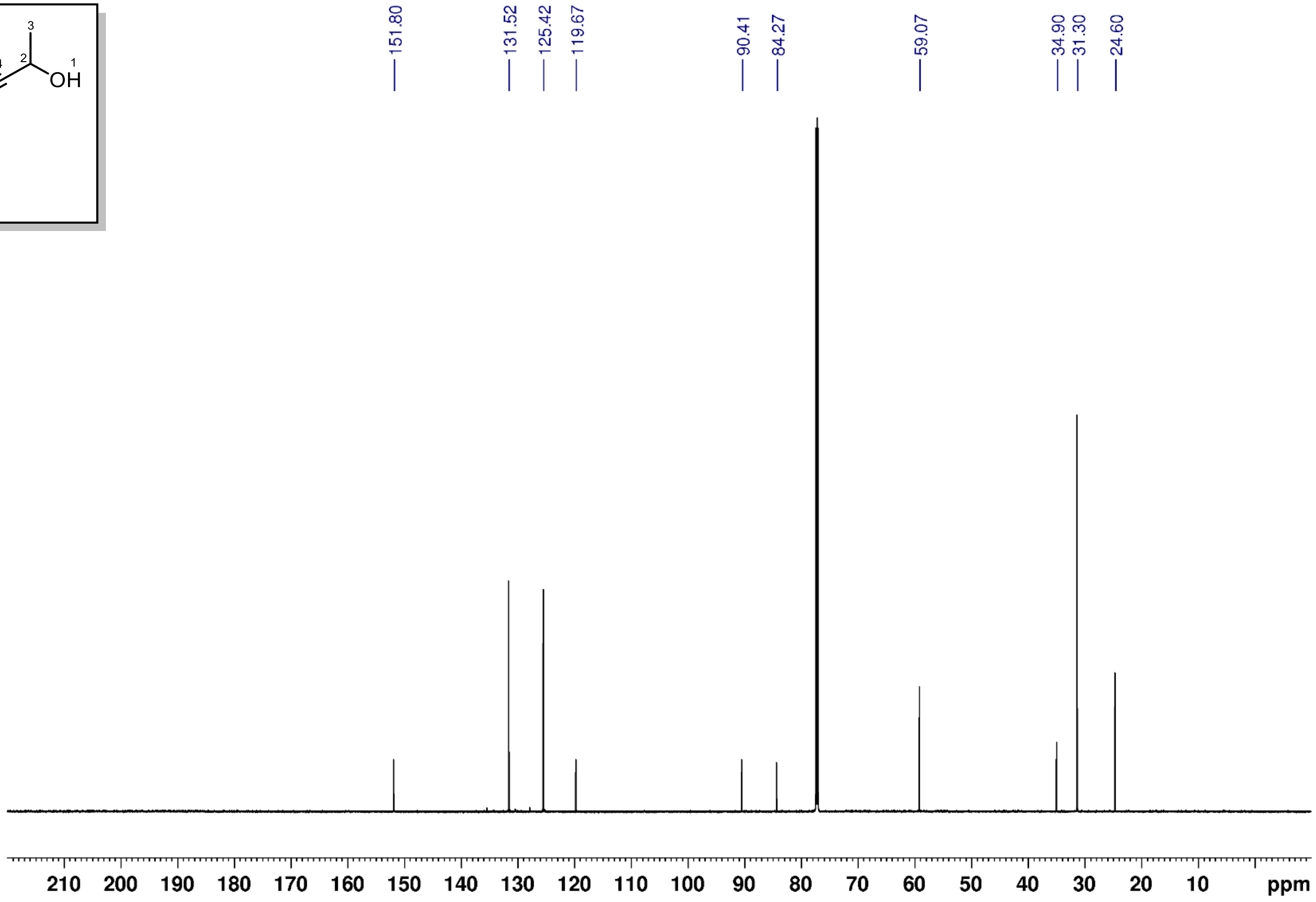

^{1}H NMR (400 MHz, $CDCl_3$) for 1-(4-(*tert*-butyl)phenyl)-4-methylpent-1-yn-3-ol

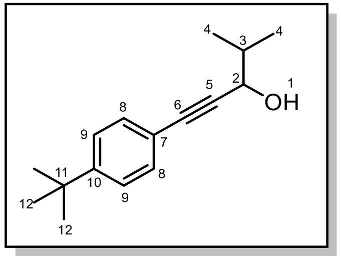

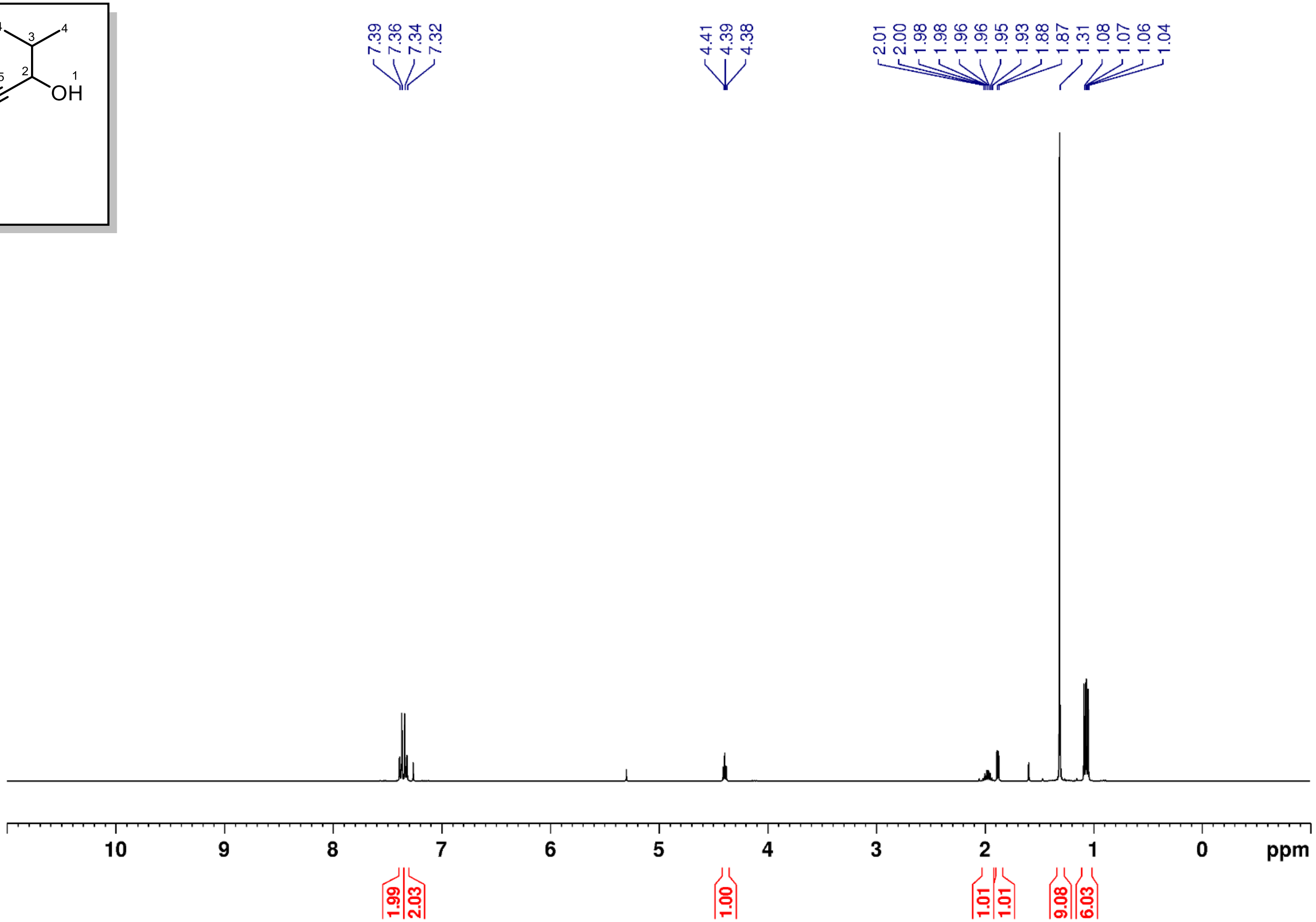

^{13}C NMR (101 MHz, CDCl$_3$) for 1-(4-(*tert*-butyl)phenyl)-4-methylpent-1-yn-3-ol

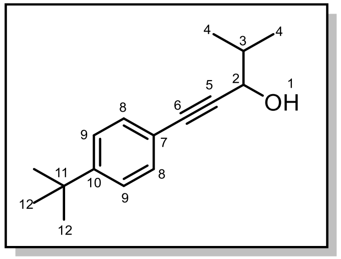

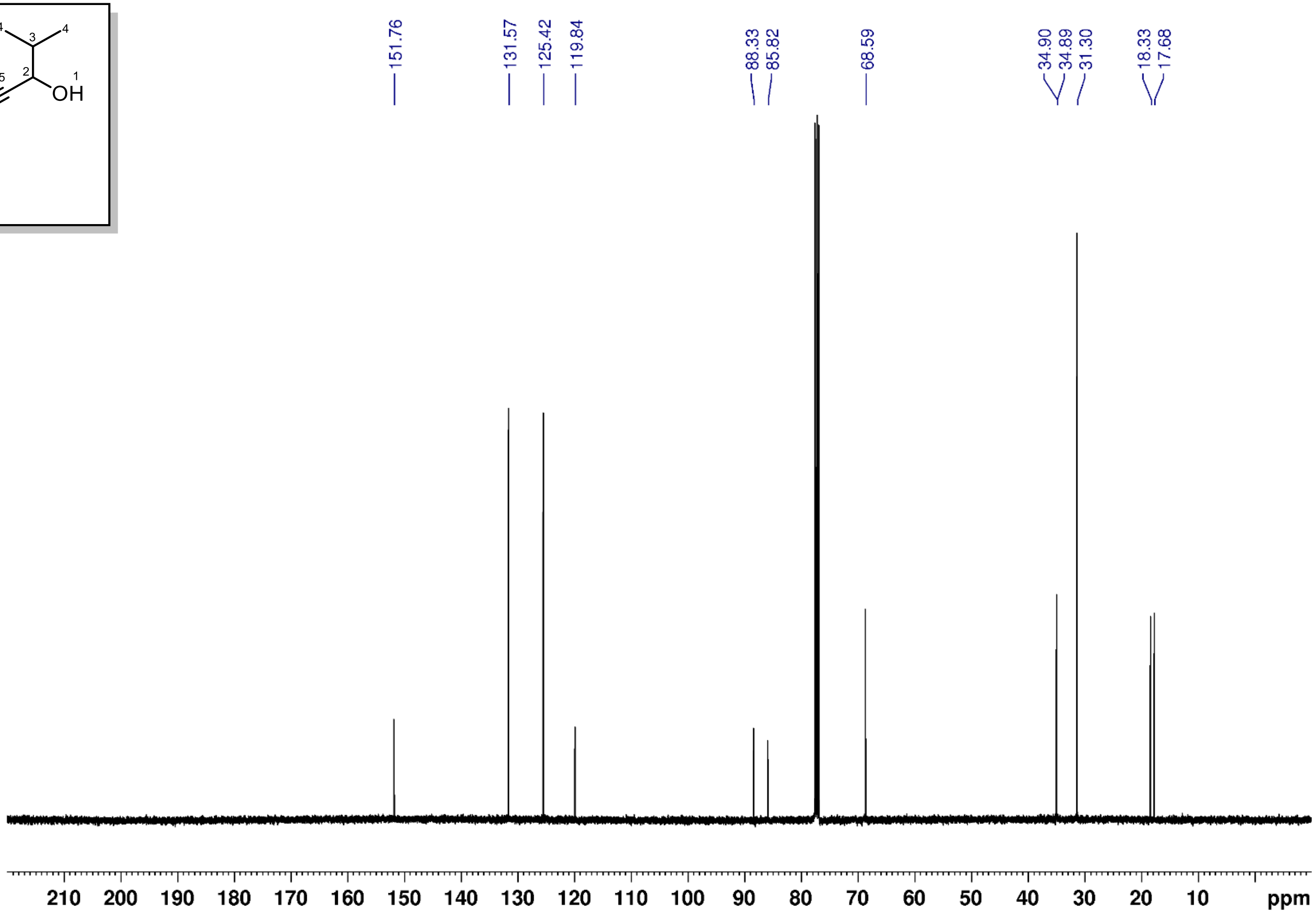

NMR Spectra for Substrates – α,β-Unsaturated Esters:

^{1}H NMR (700 MHz, CDCl$_3$) for ethyl (*Z*)-3-iodoacrylate

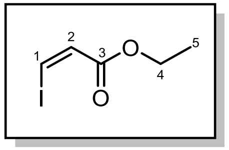

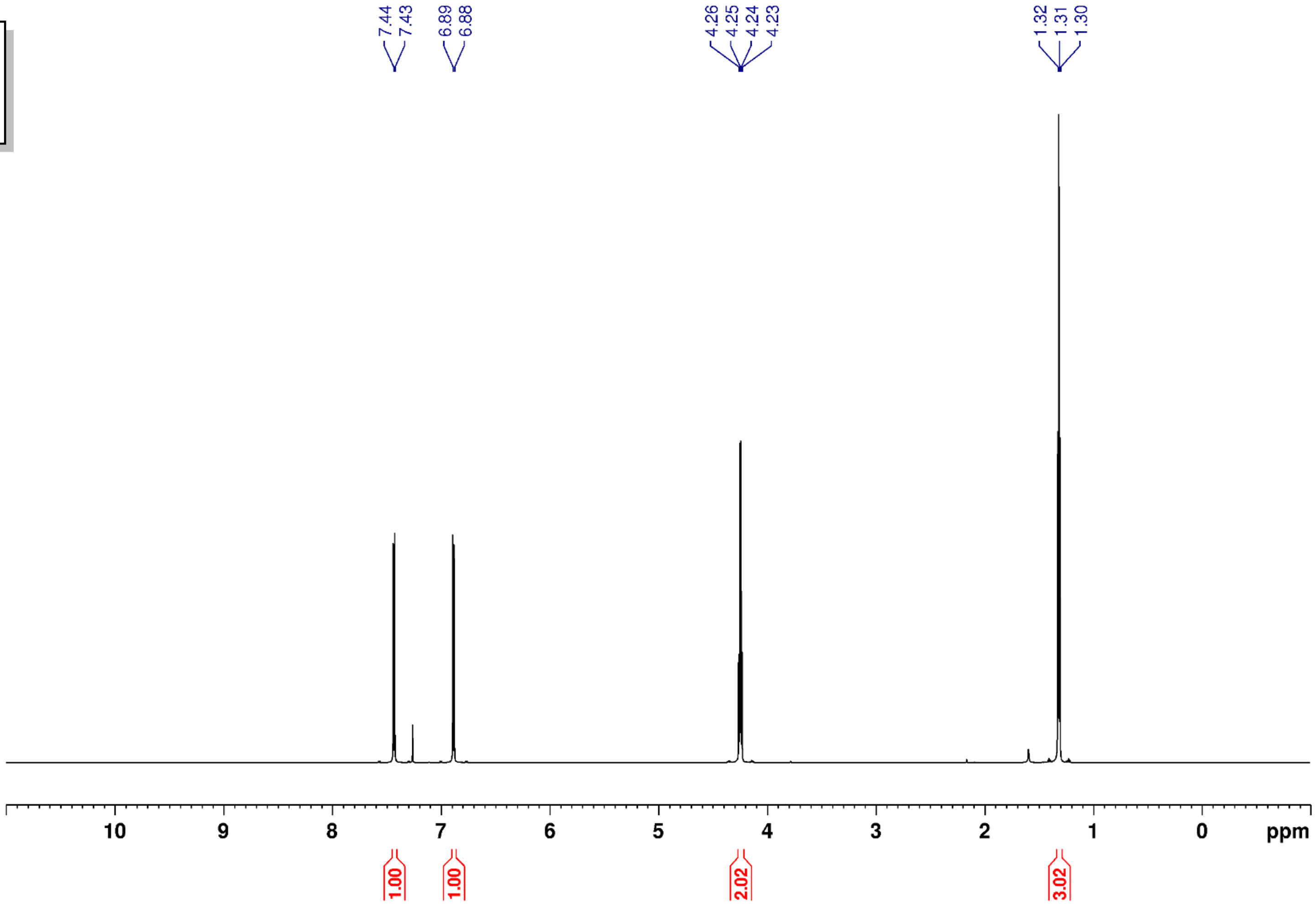

^{13}C NMR (176 MHz, CDCl$_3$) for ethyl (*Z*)-3-iodoacrylate

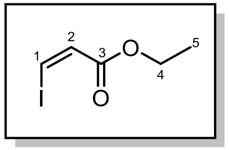

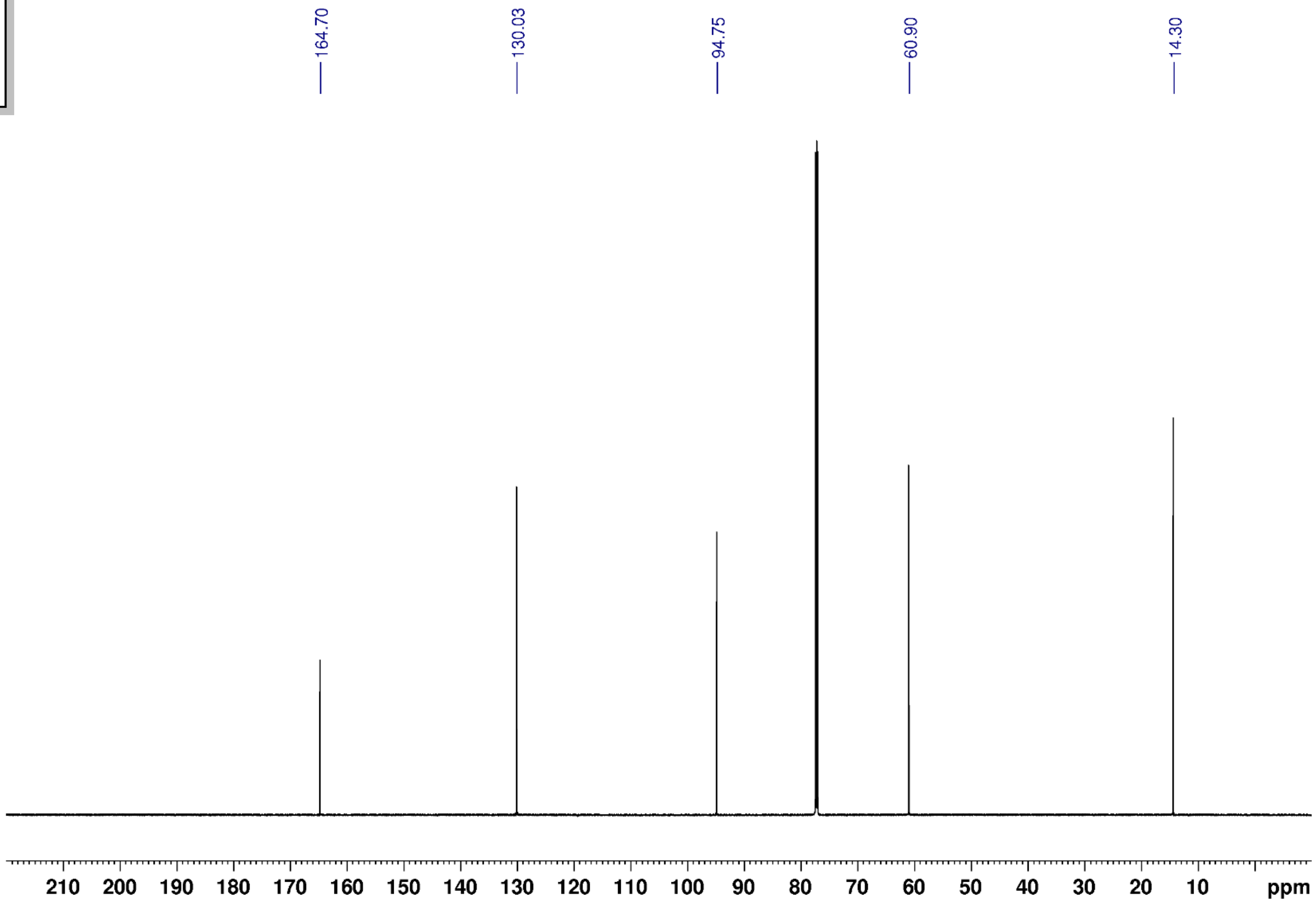

^{1}H NMR (500 MHz, CDCl$_3$) for (*Z*)-3-iodoprop-2-en-1-ol

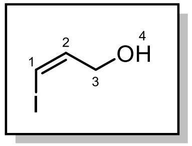

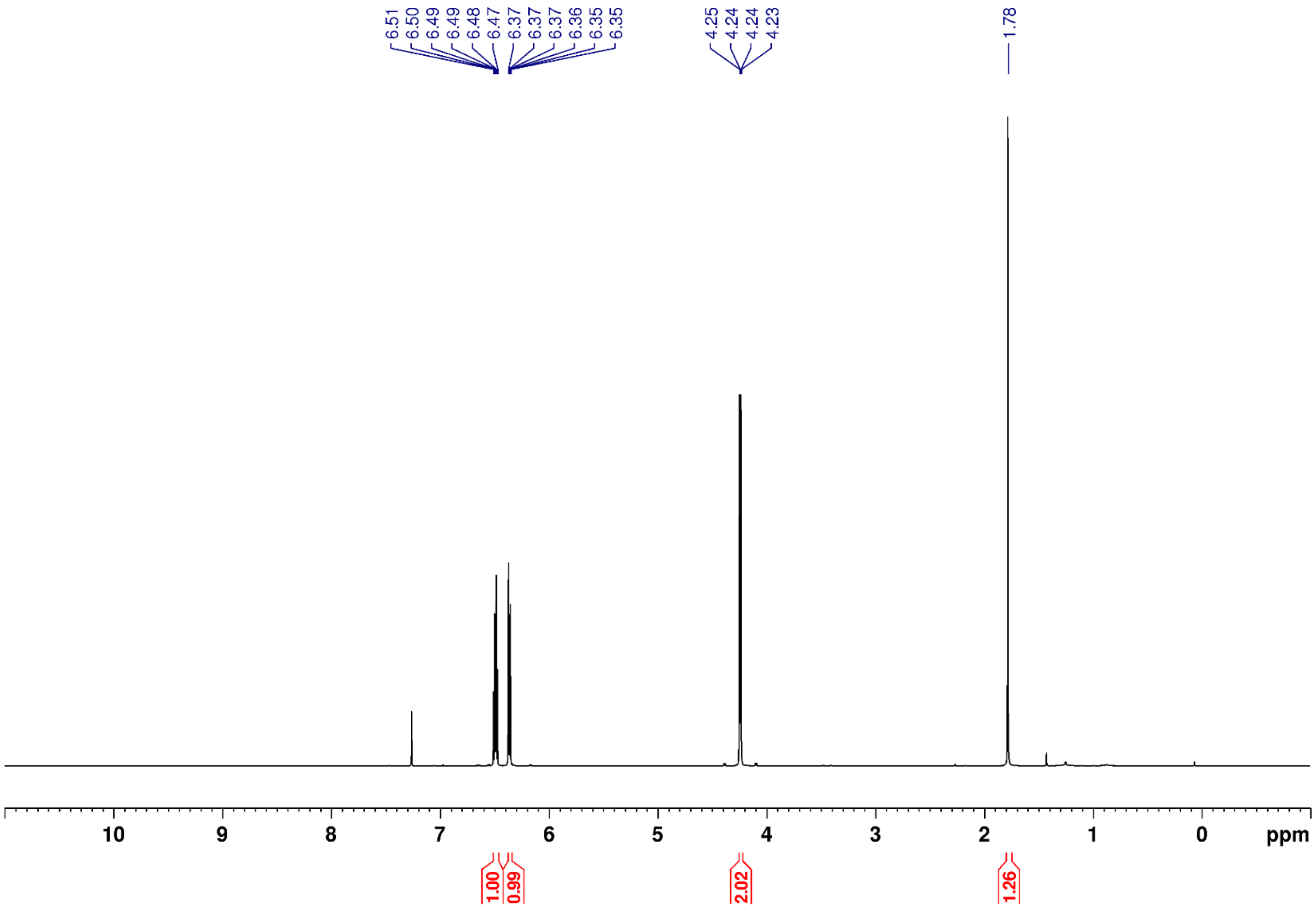

^{13}C NMR (126 MHz, $CDCl_3$) for (*Z*)-3-iodoprop-2-en-1-ol

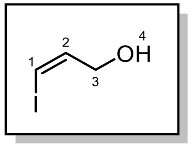

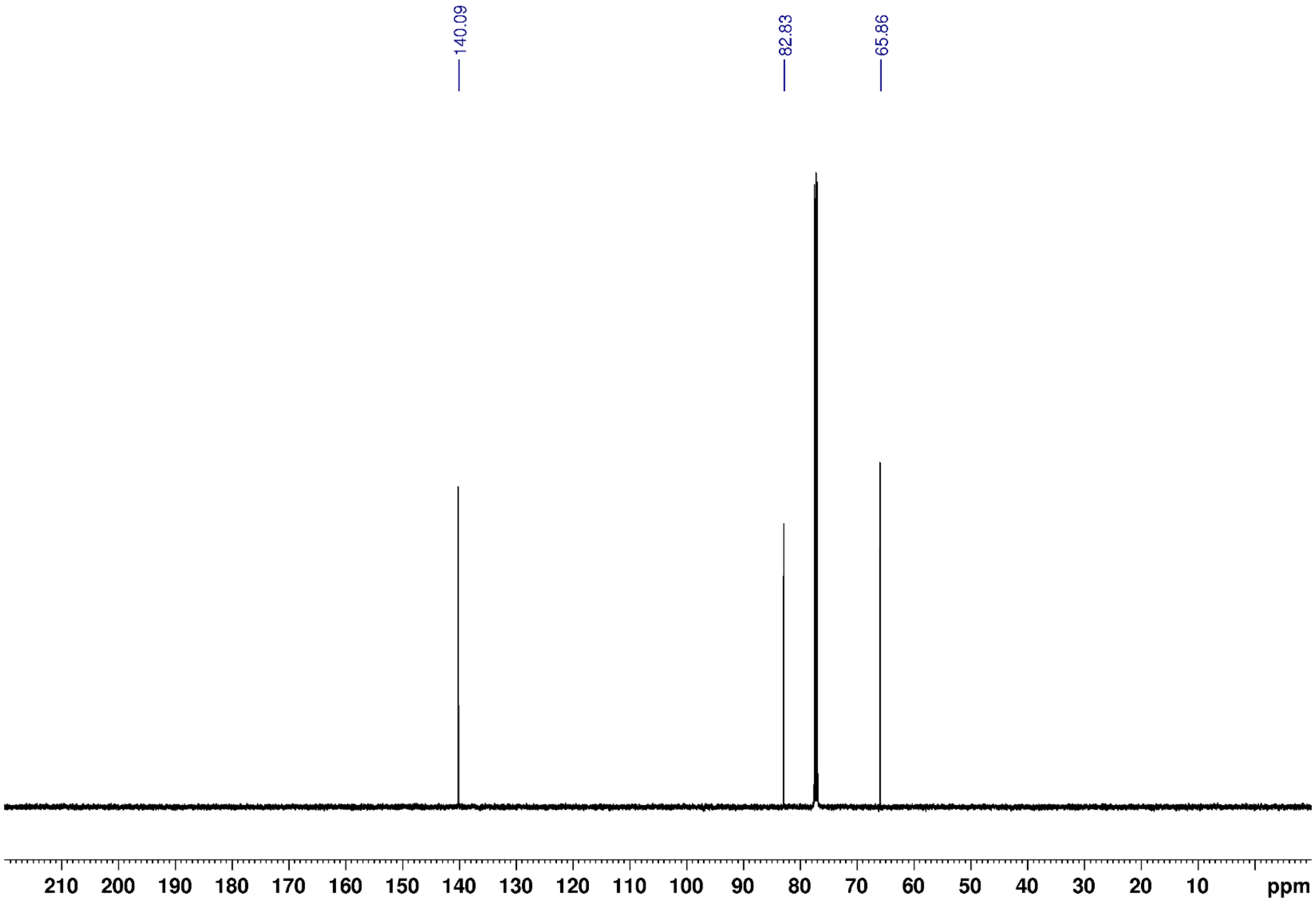

^{1}H NMR (400 MHz, CDCl$_3$) for ethyl (*Z*)-3-(2-methoxyphenyl)acrylate

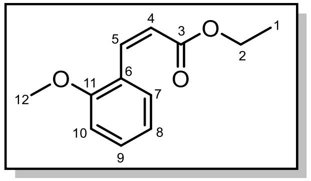

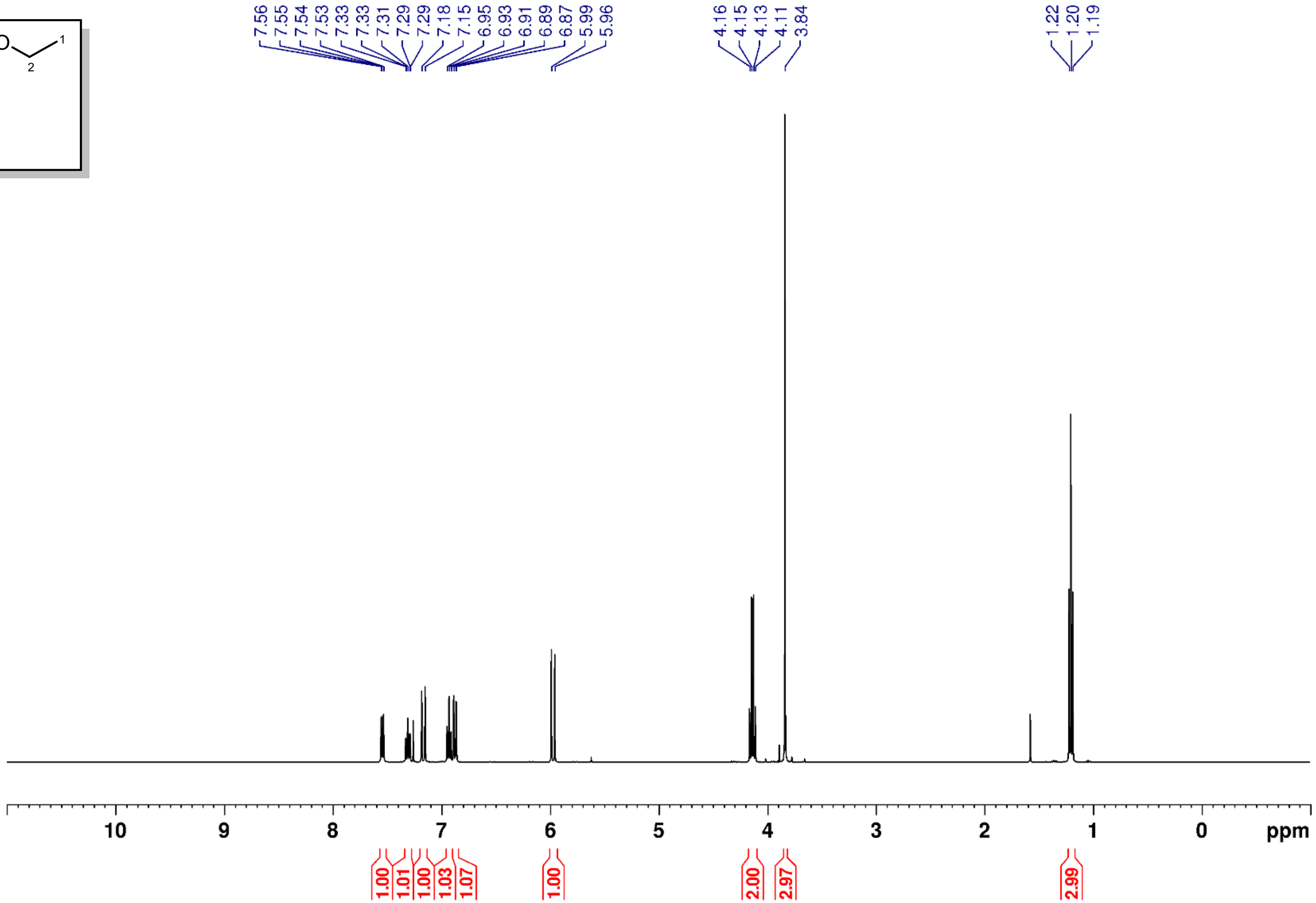

^{13}C NMR (101 MHz, $CDCl_3$) for ethyl (*Z*)-3-(2-methoxyphenyl)acrylate

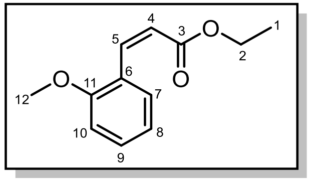

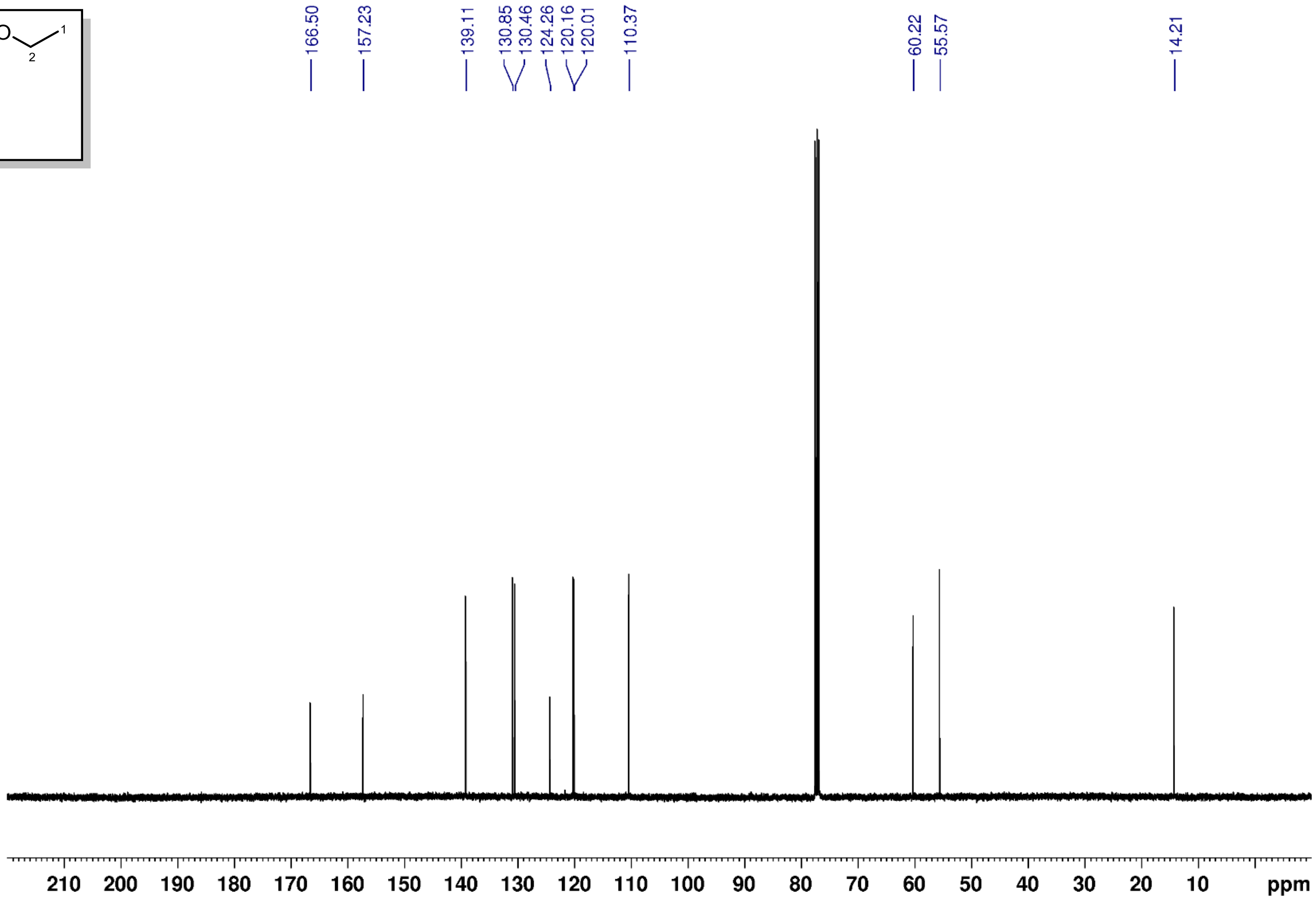

^{1}H NMR (500 MHz, $CDCl_3$) for ethyl (*Z*)-3-(naphthalen-2-yl)acrylate

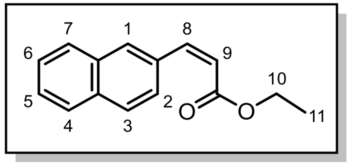

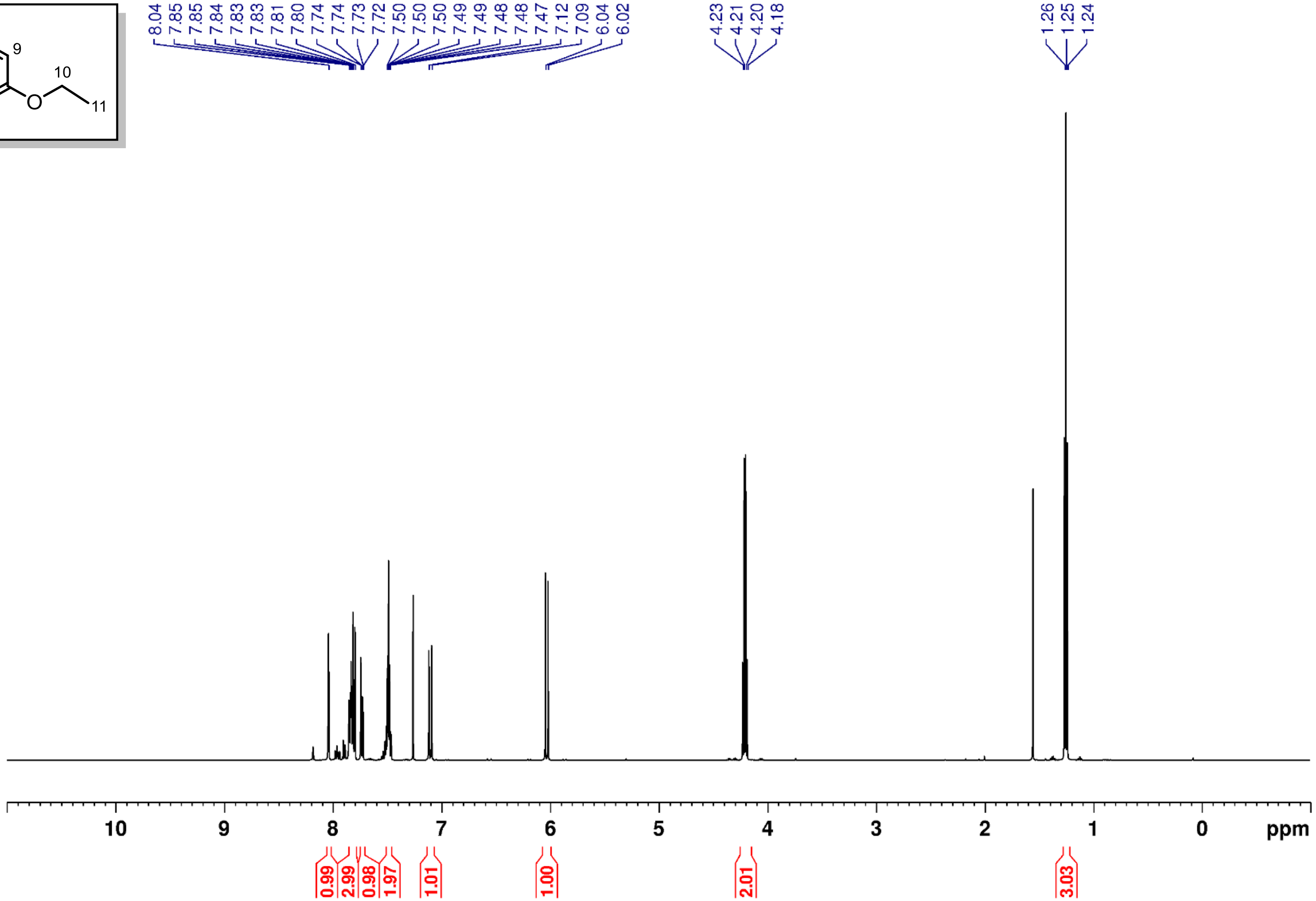

^{13}C NMR (126 MHz, $CDCl_3$) for ethyl (*Z*)-3-(naphthalen-2-yl)acrylate

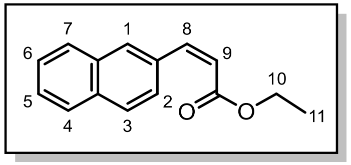

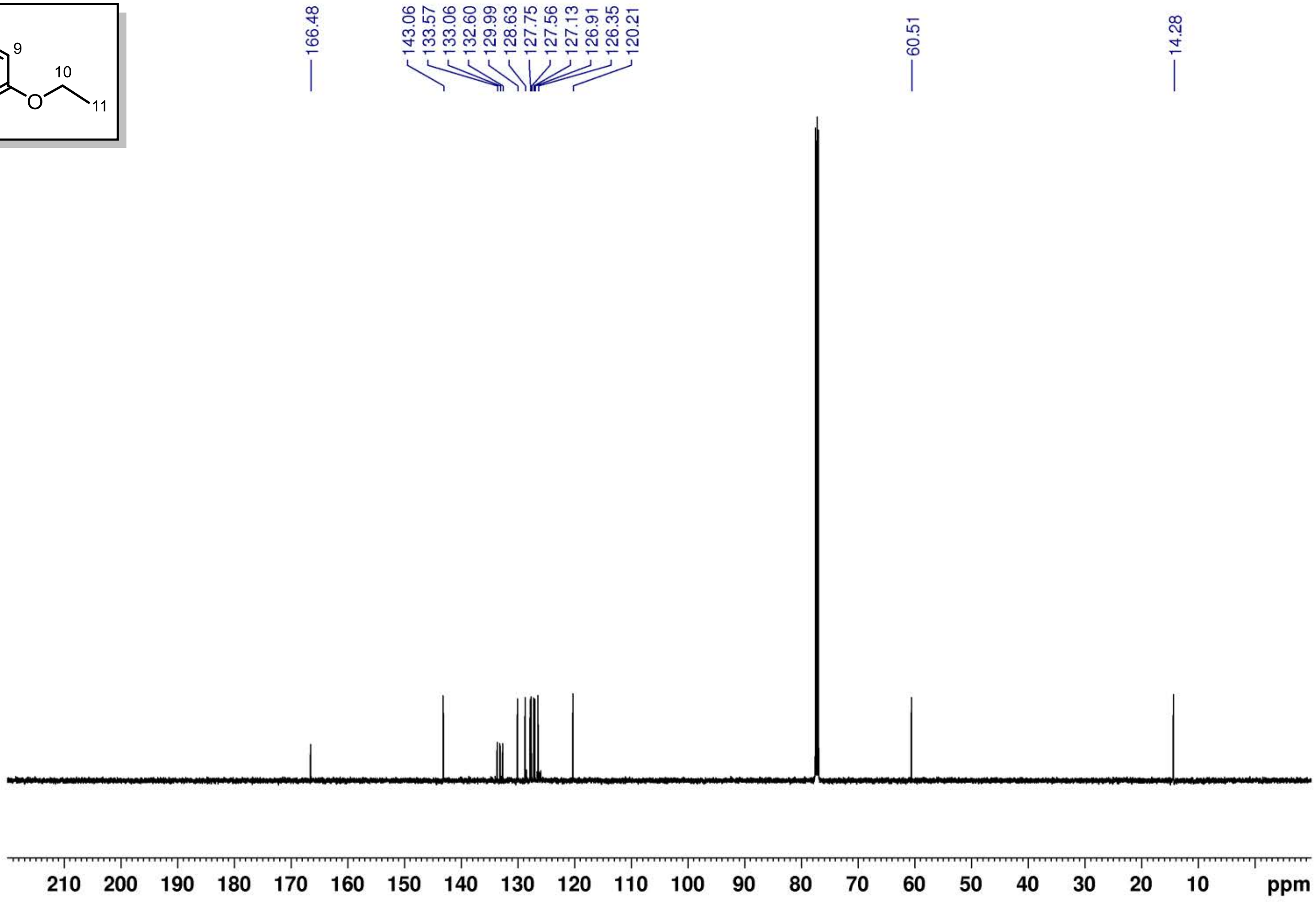

^{1}H NMR (400 MHz, $CDCl_3$) for ethyl (*Z*)-3-(thiophen-3-yl)acrylate

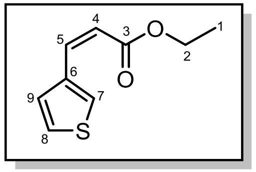

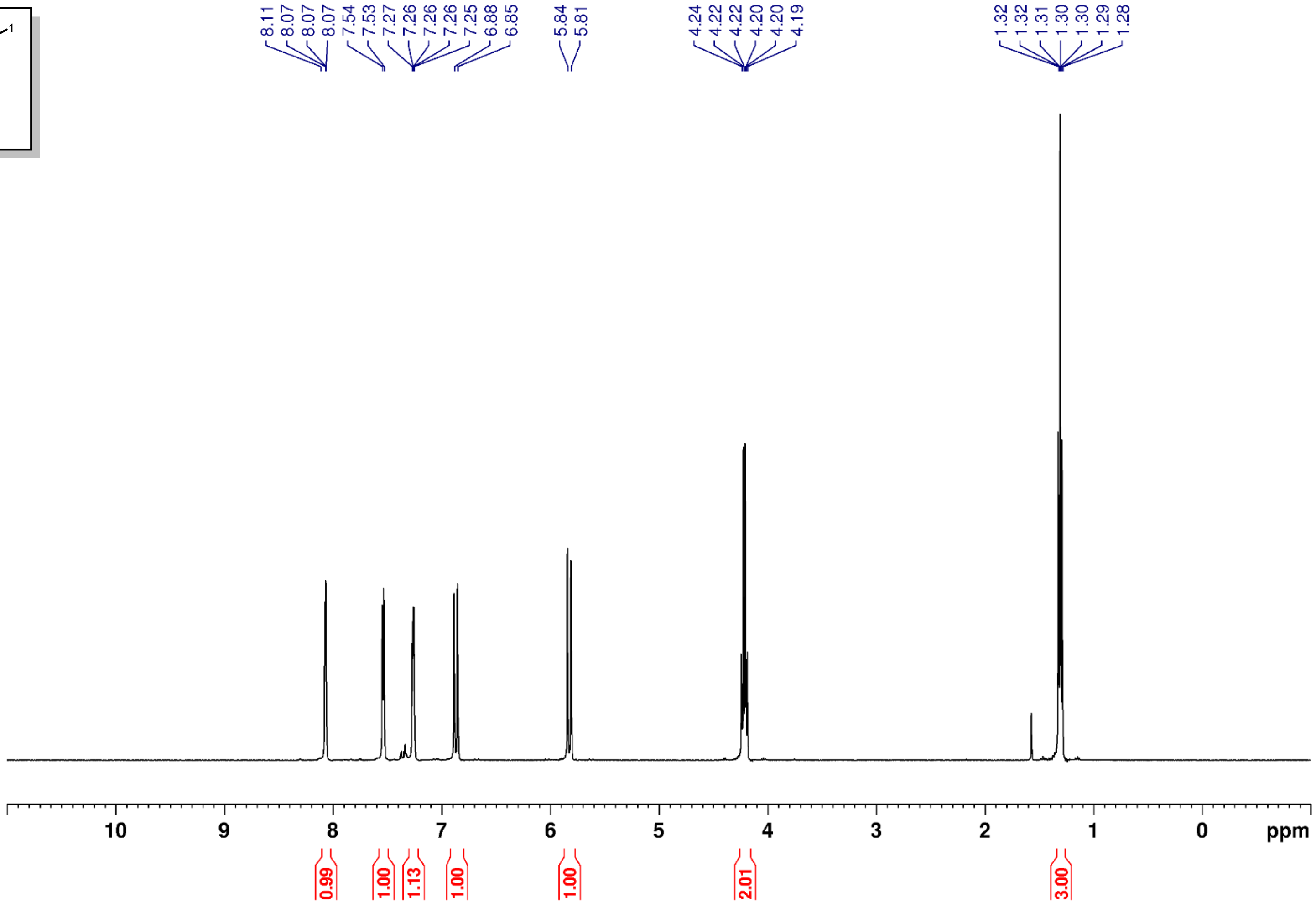

^{13}C NMR (101 MHz, CDCl$_3$) for ethyl (*Z*)-3-(thiophen-3-yl)acrylate

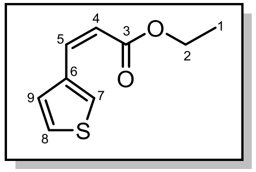

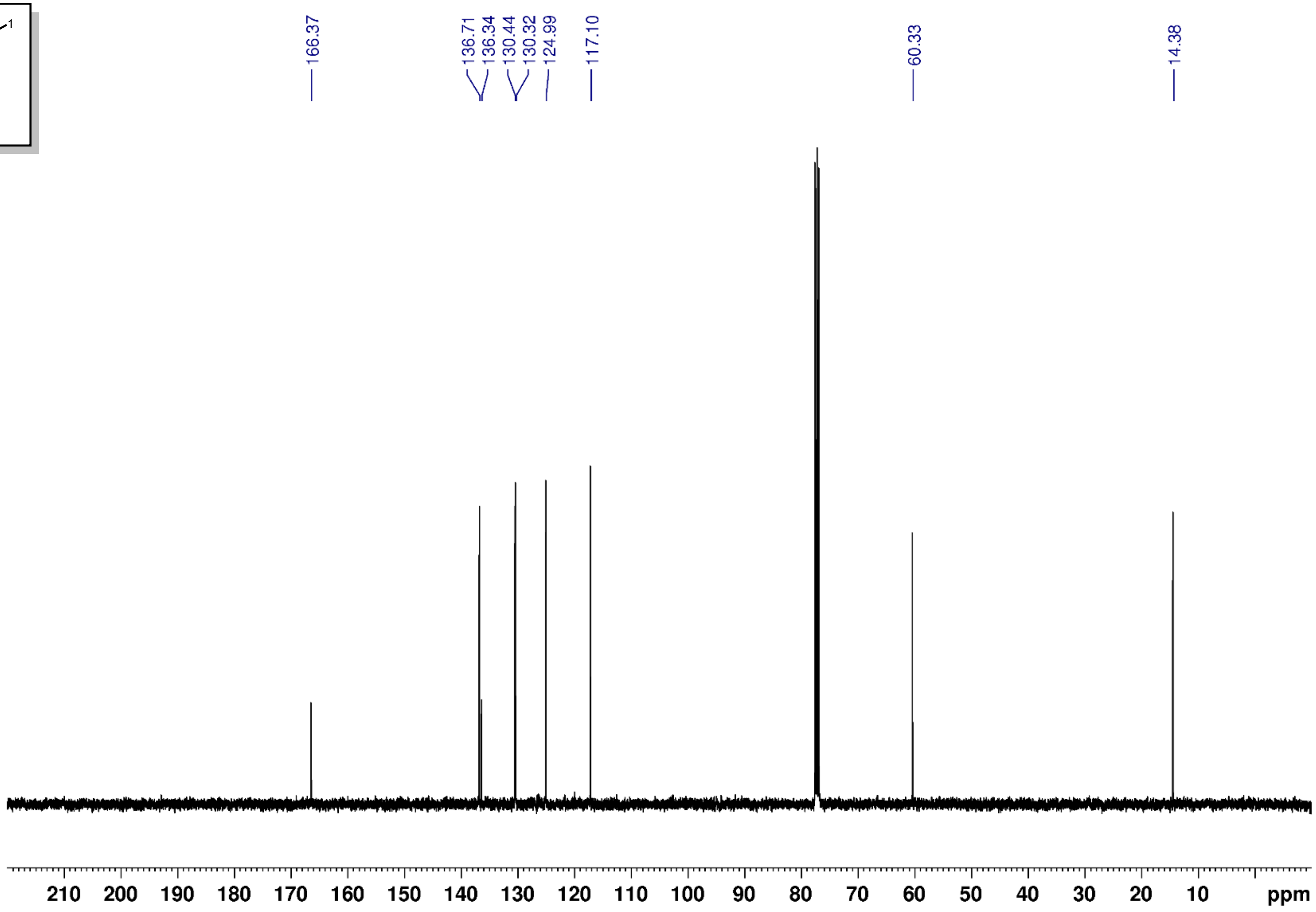

^{1}H NMR (400 MHz, CDCl$_3$) for (*Z*)-3-(3-methoxyphenyl)prop-2-en-1-ol

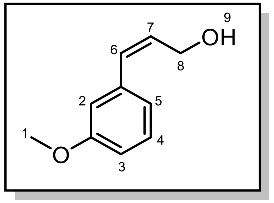

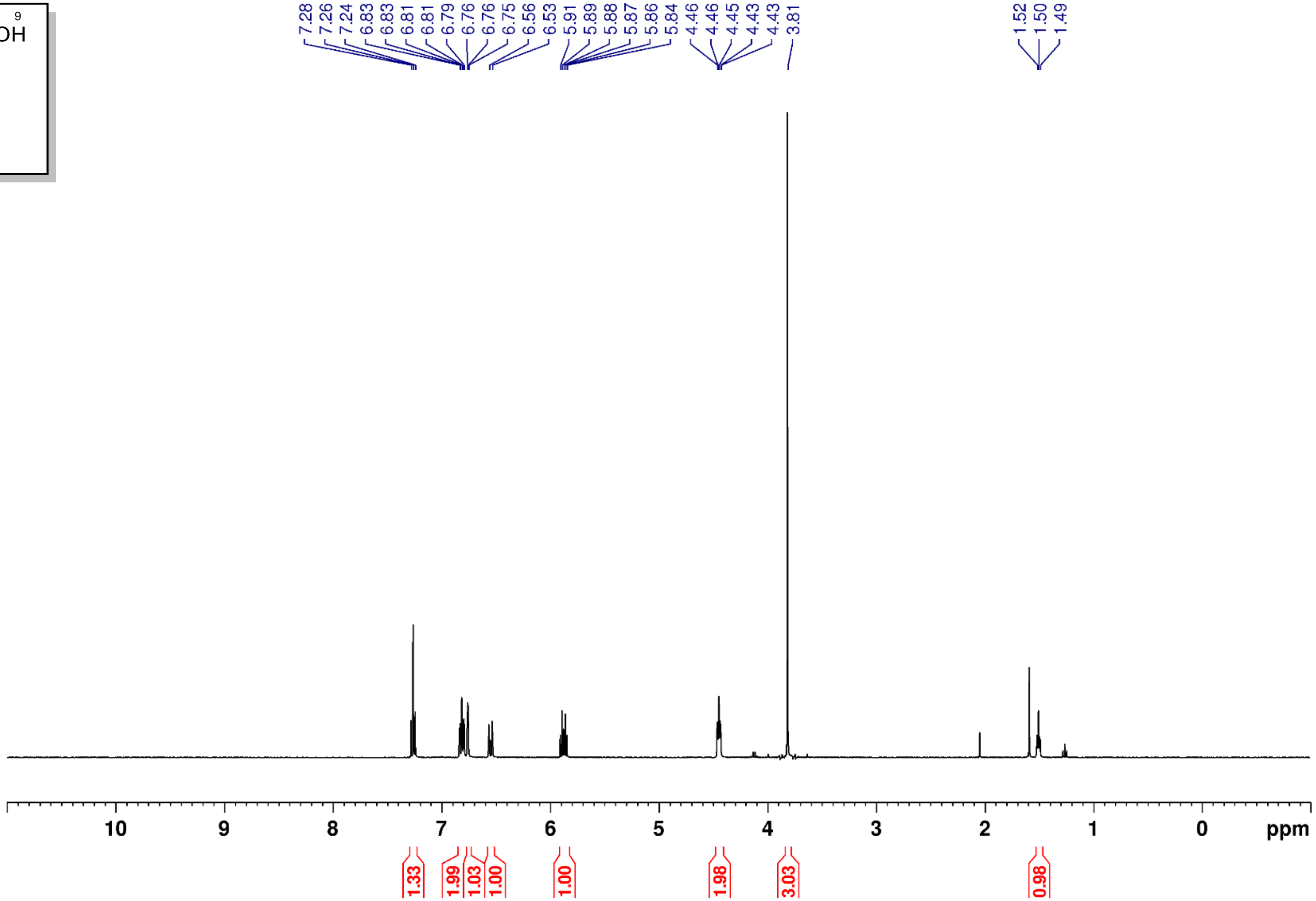

^{13}C NMR (101 MHz, CDCl$_3$) for (*Z*)-3-(3-methoxyphenyl)prop-2-en-1-ol

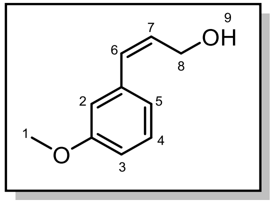

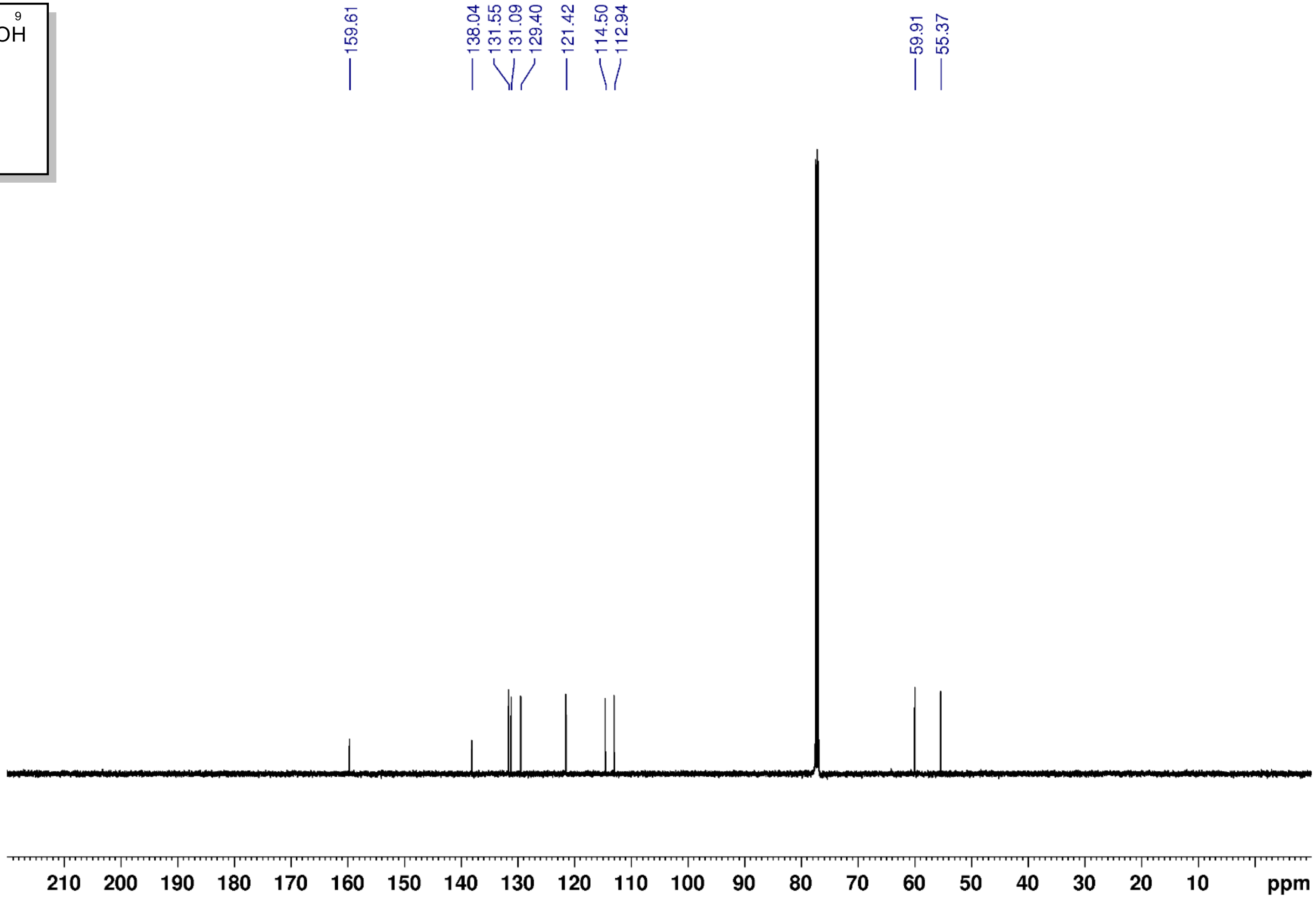

NMR Spectra for Substrates – N-H Allylic Carbamates:

^{1}H NMR (400 MHz, $CDCl_3$) for (*Z*)-3-phenylallyl phenylcarbamate (**S1**)

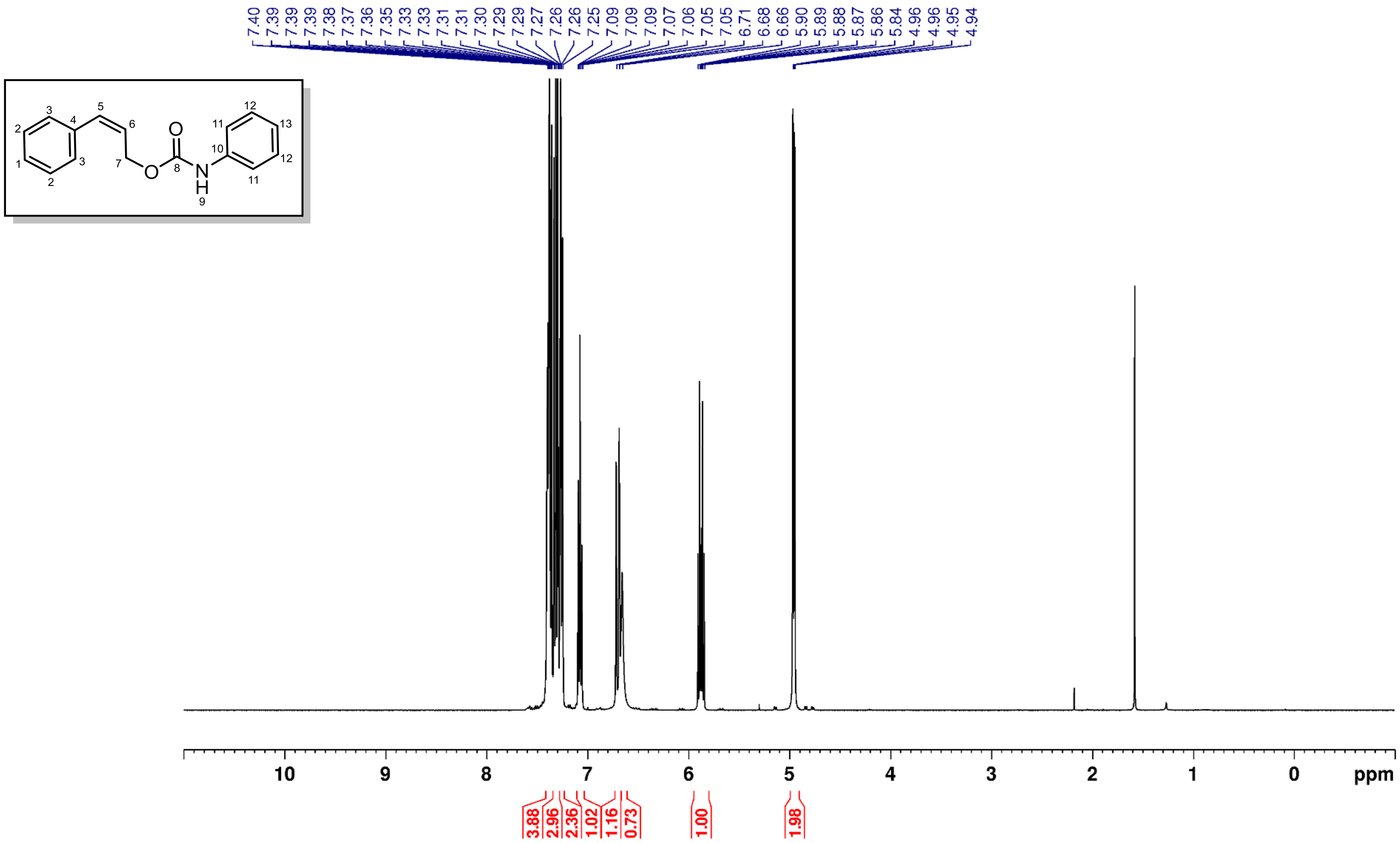

^{13}C NMR (101 MHz, $CDCl_3$) for (*Z*)-3-phenylallyl phenylcarbamate (**S1**)

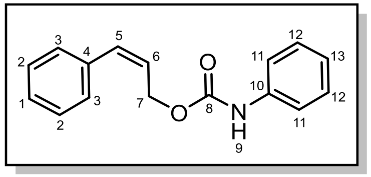

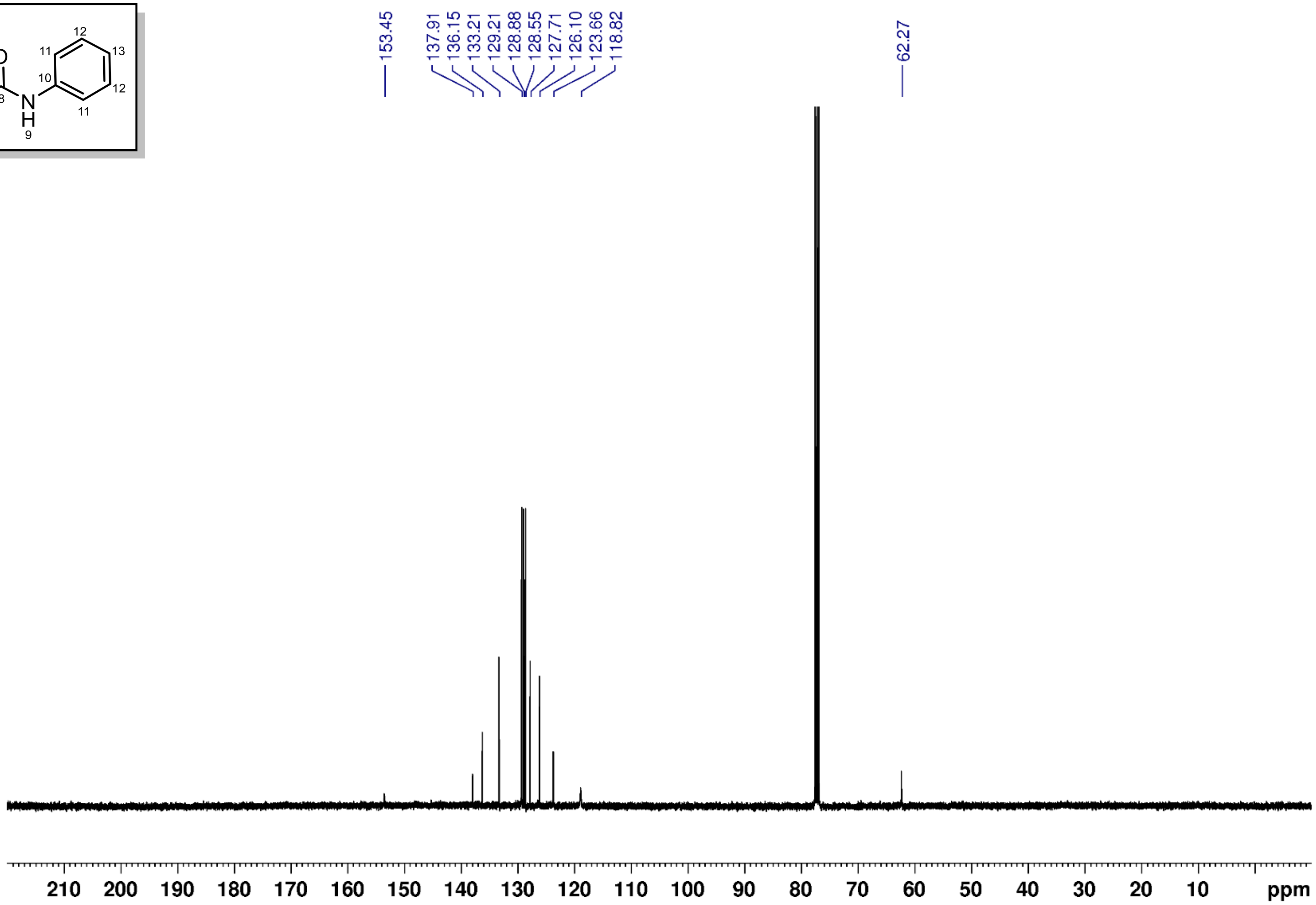

^{1}H NMR (700 MHz, $CDCl_3$) for *(Z)*-3-phenylallyl morpholine-4-carboxylate

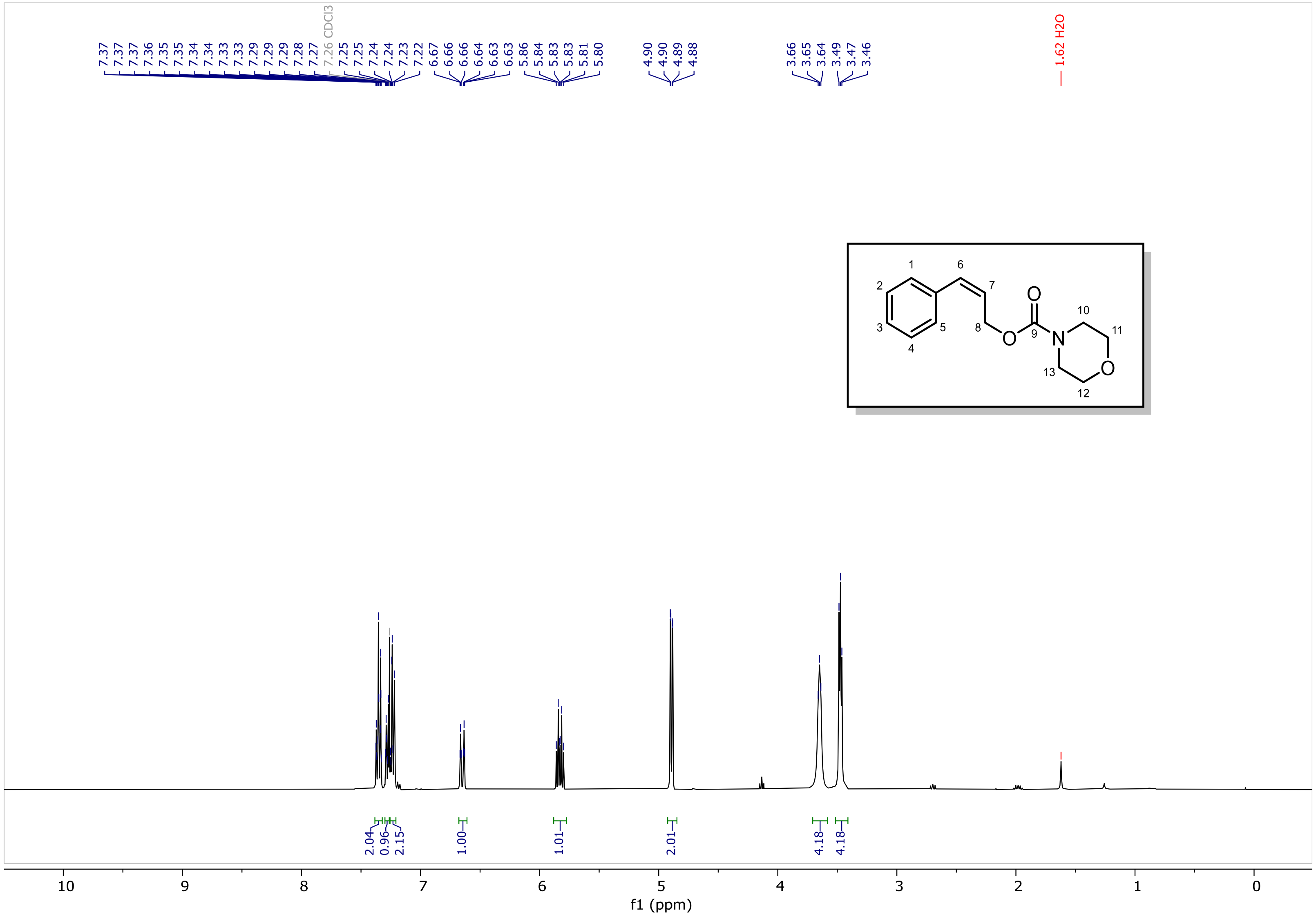

^{13}C NMR (176 MHz, CDCl$_3$) for *(Z)*-3-phenylallyl morpholine-4-carboxylate

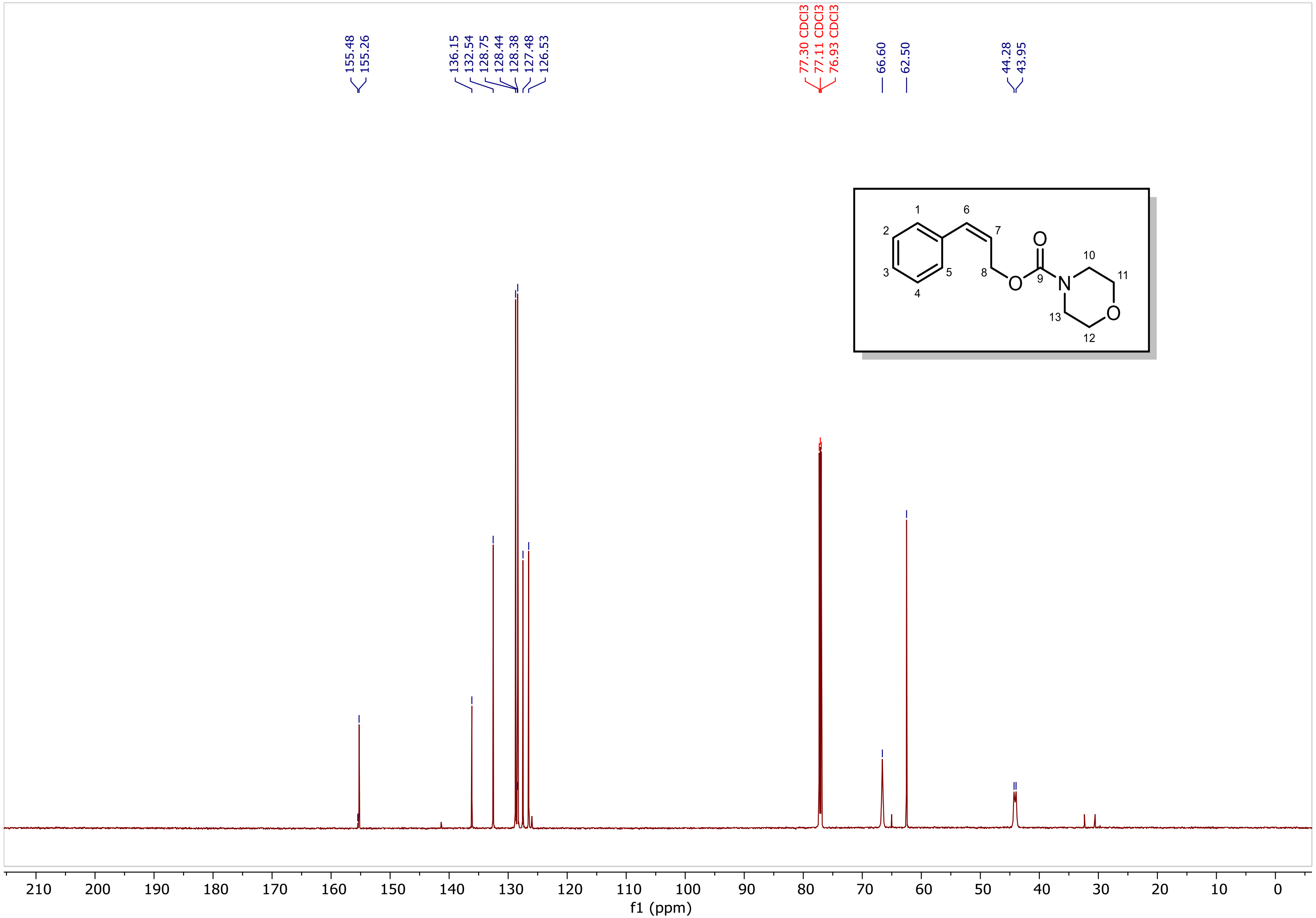

^{1}H NMR (500 MHz, $CDCl_3$) for (*Z*)-3-(2-isopropylphenyl)allyl phenylcarbamate

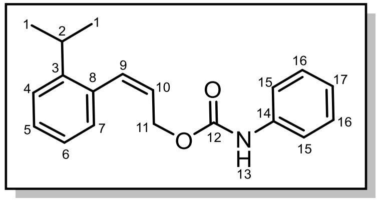

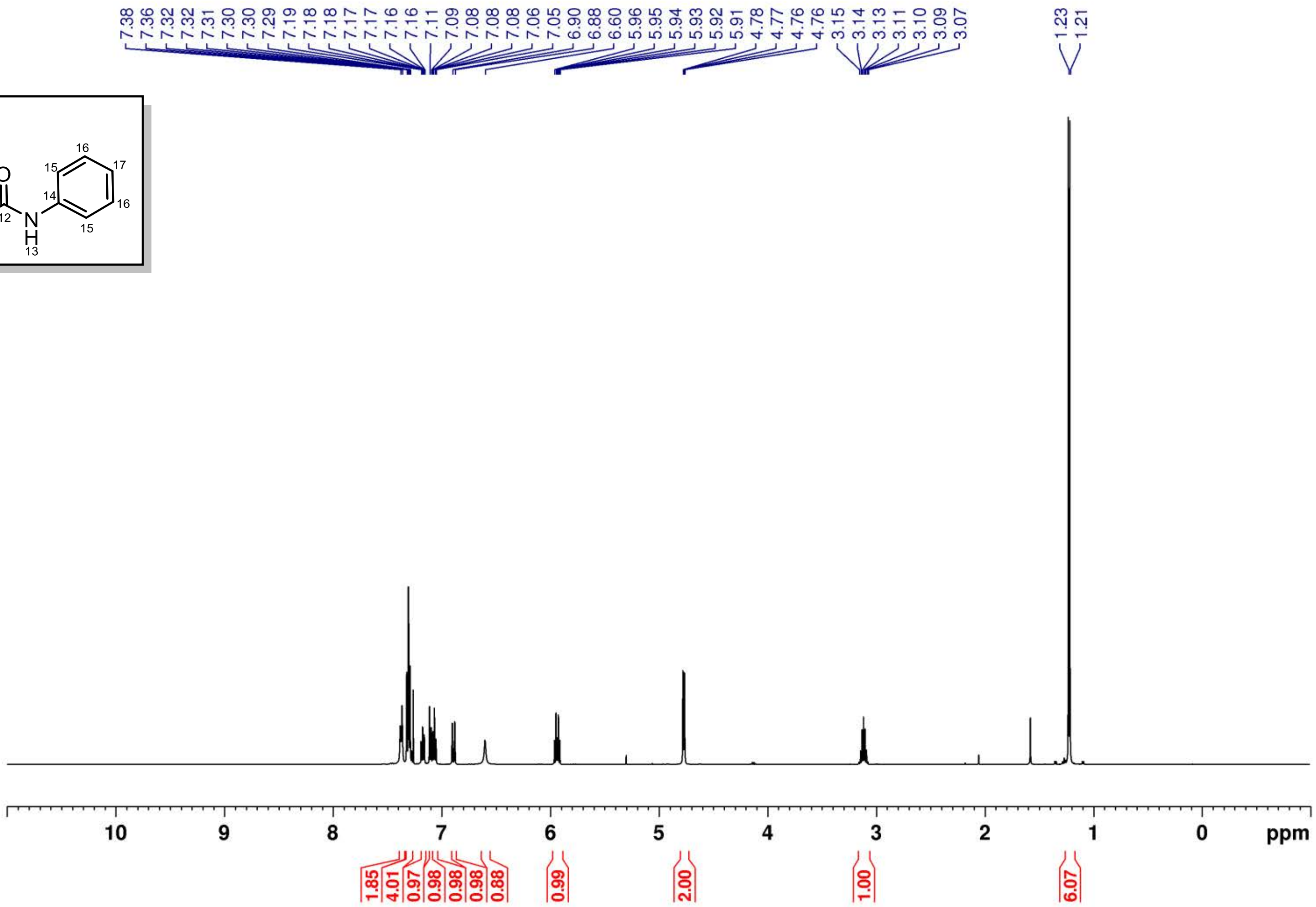

^{13}C NMR (126 MHz, $CDCl_3$) for (*Z*)-3-(2-isopropylphenyl)allyl phenylcarbamate

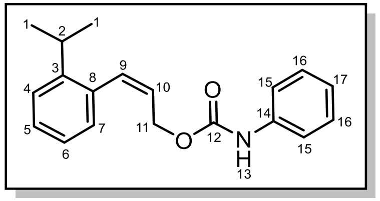

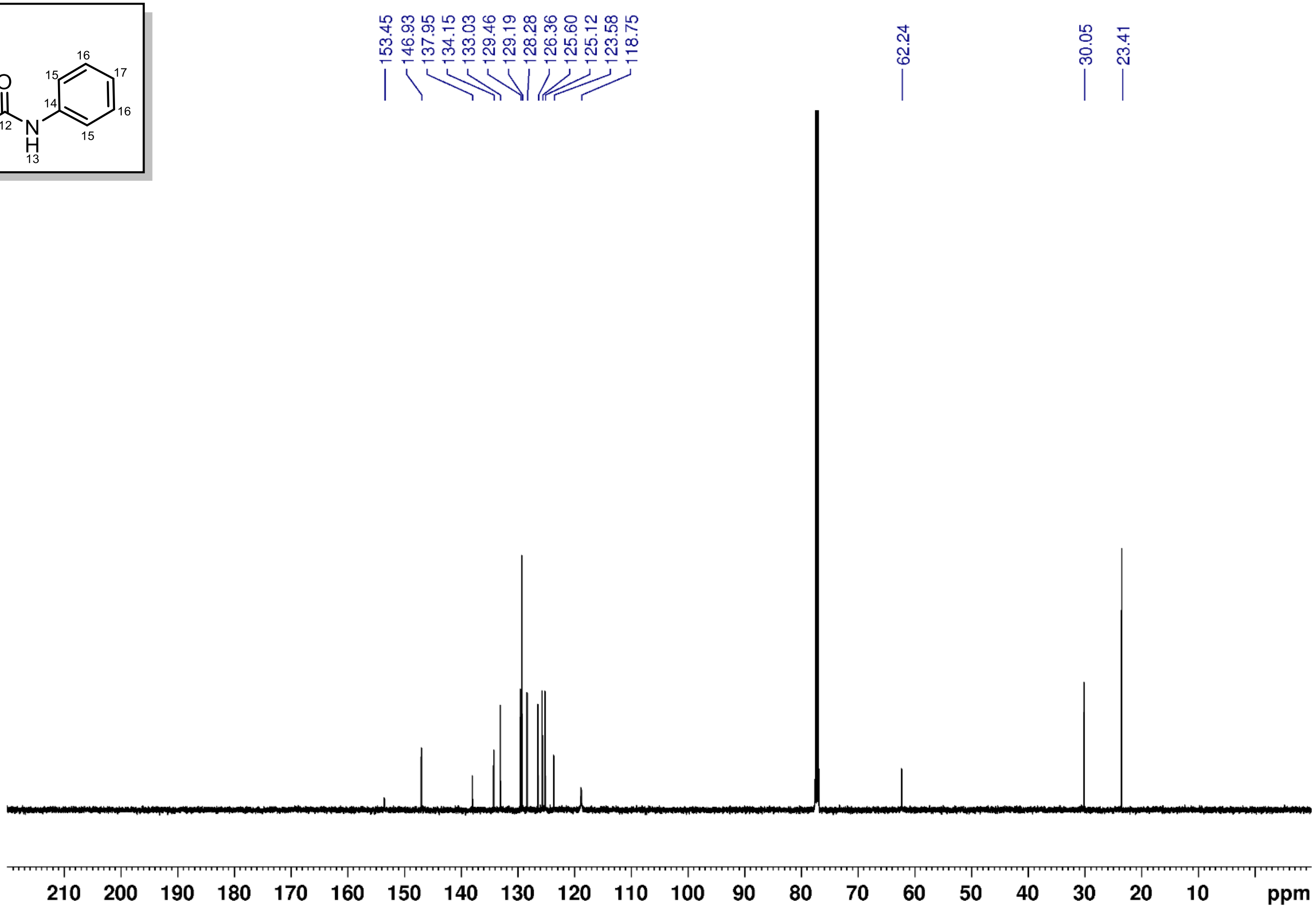

^{1}H NMR (400 MHz, CDCl$_3$) for (*Z*)-3-(2-methoxyphenyl)allyl phenylcarbamate

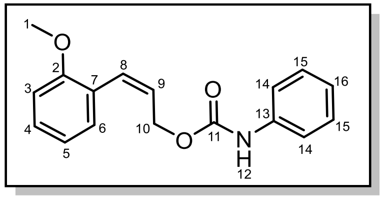

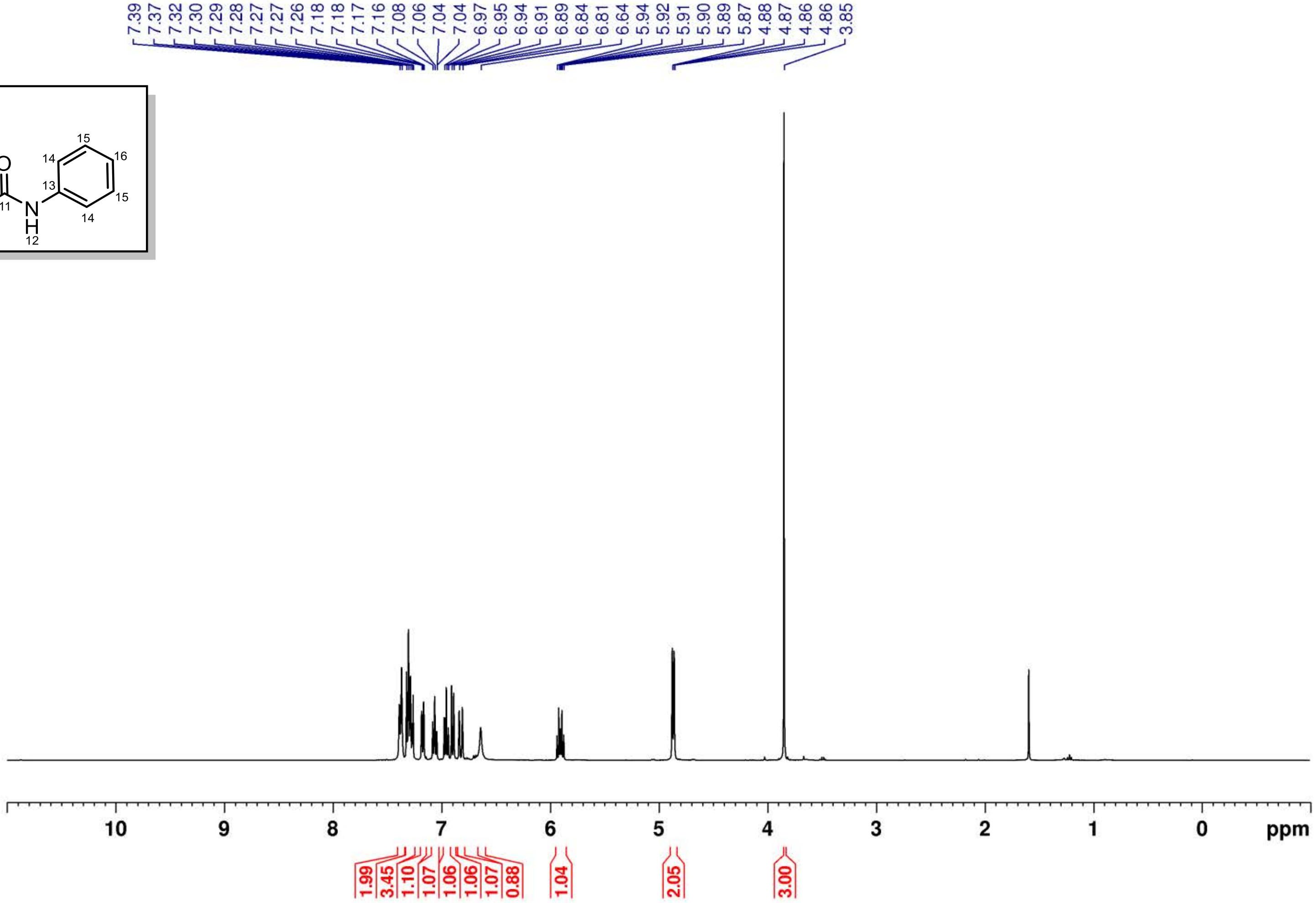

^{13}C NMR (101 MHz, CDCl$_3$) for (*Z*)-3-(2-methoxyphenyl)allyl phenylcarbamate

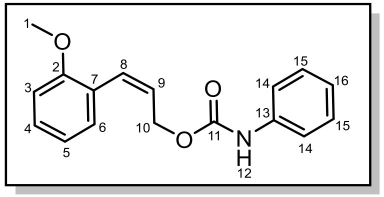

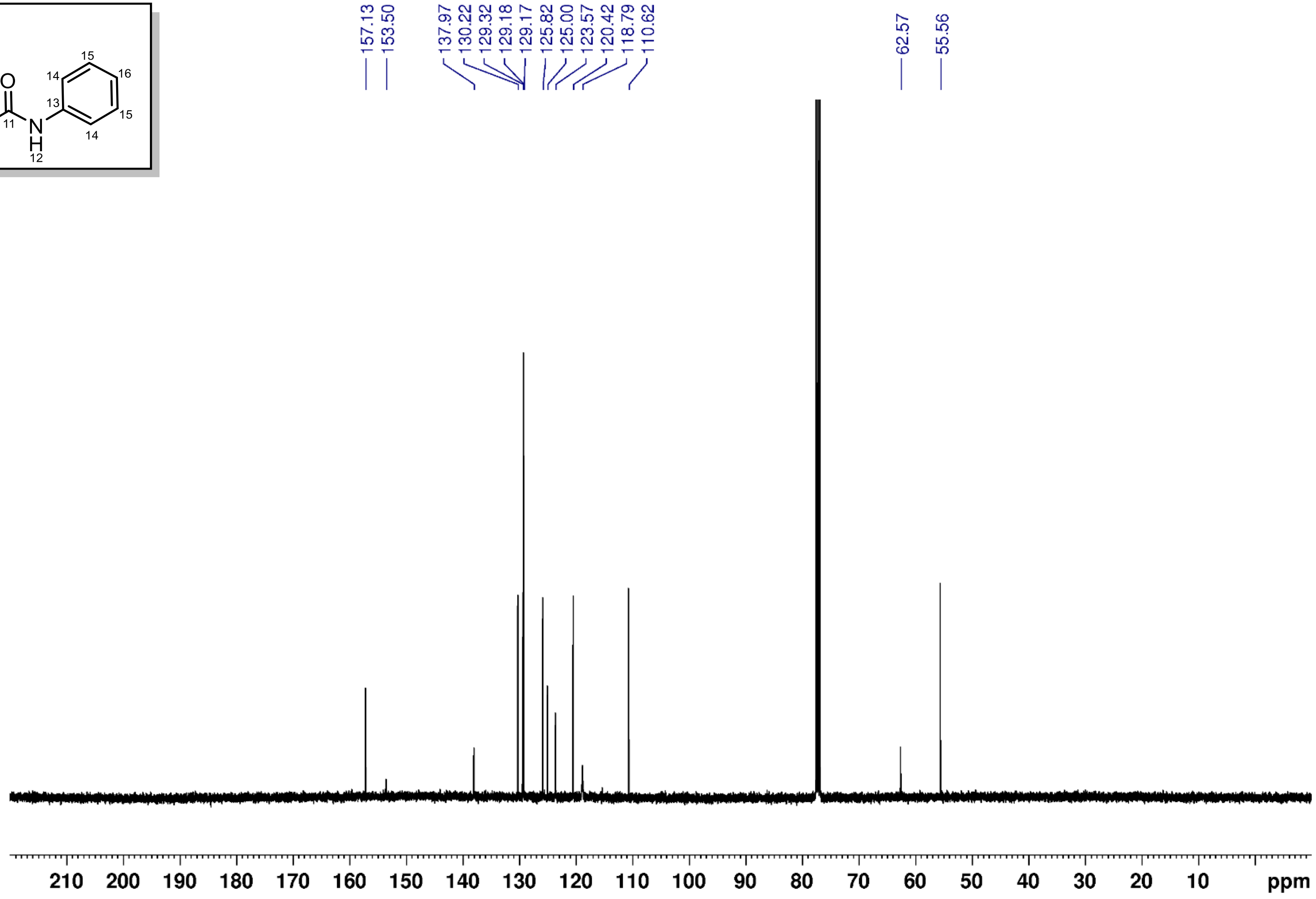

^{1}H NMR (400 MHz, $CDCl_3$) for (*Z*)-3-(2-fluorophenyl)allyl phenylcarbamate

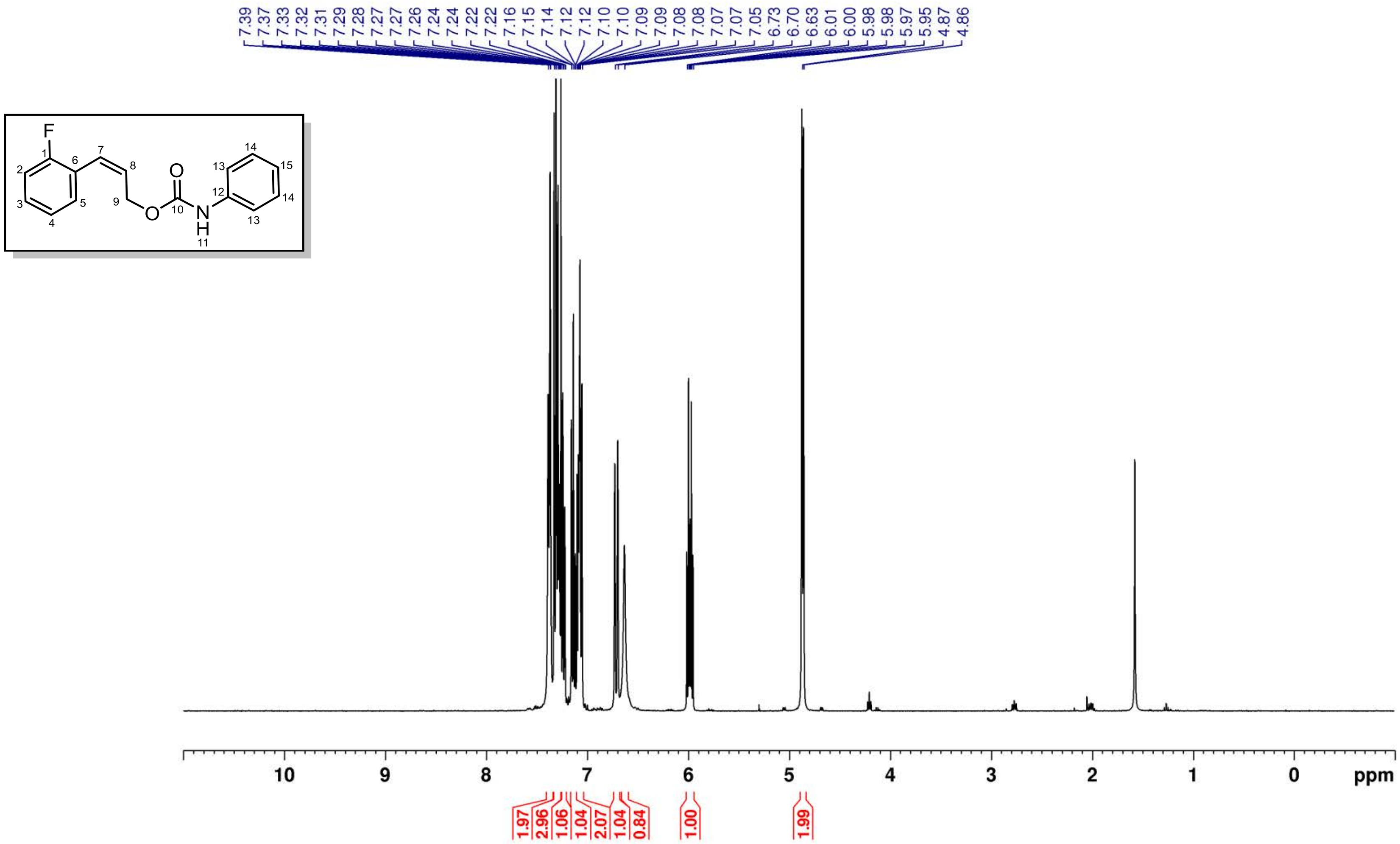

^{13}C NMR (101 MHz, CDCl$_3$) for (*Z*)-3-(2-fluorophenyl)allyl phenylcarbamate

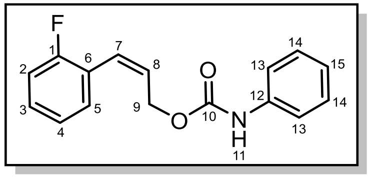

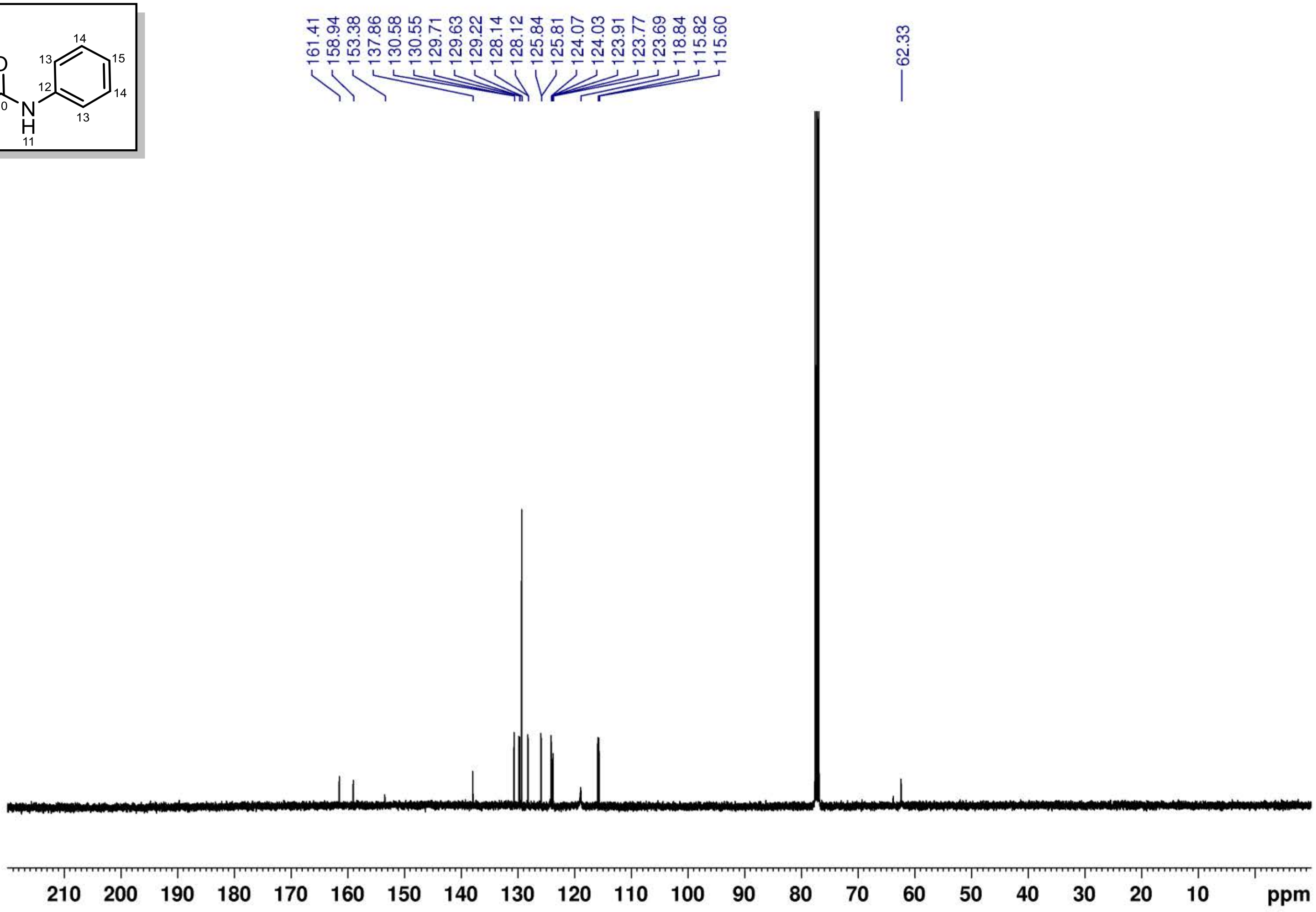

^{19}F NMR (376 MHz, CDCl$_3$) for (*Z*)-3-(2-fluorophenyl)allyl phenylcarbamate

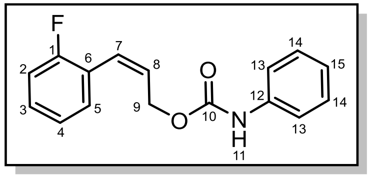

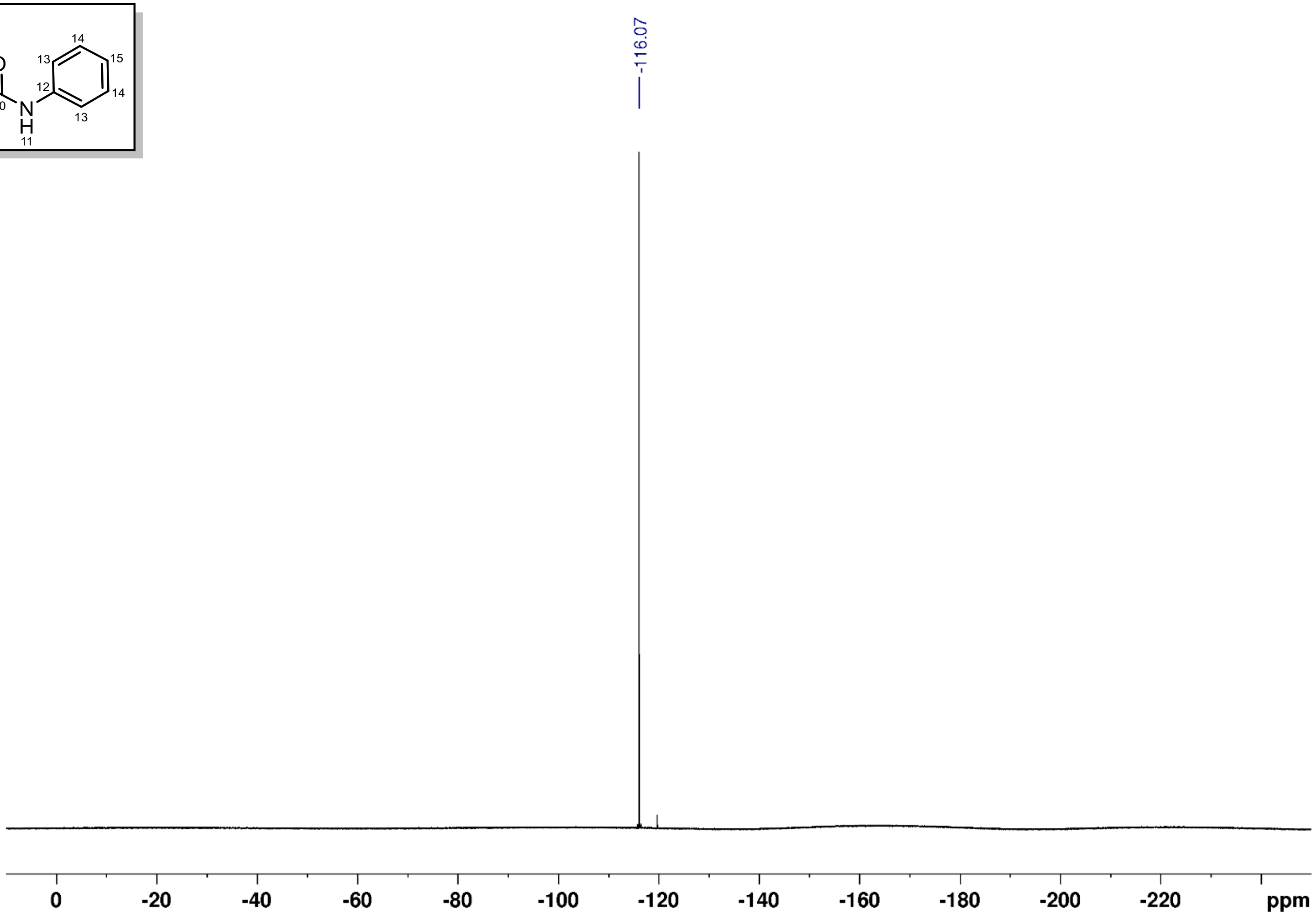

^{1}H NMR (400 MHz, CDCl$_3$) for (*Z*)-3-(3-(tert-butyl)phenyl)allyl phenylcarbamate

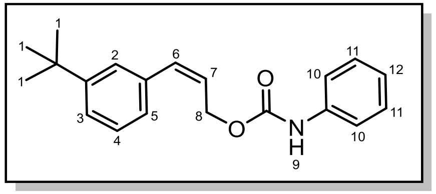

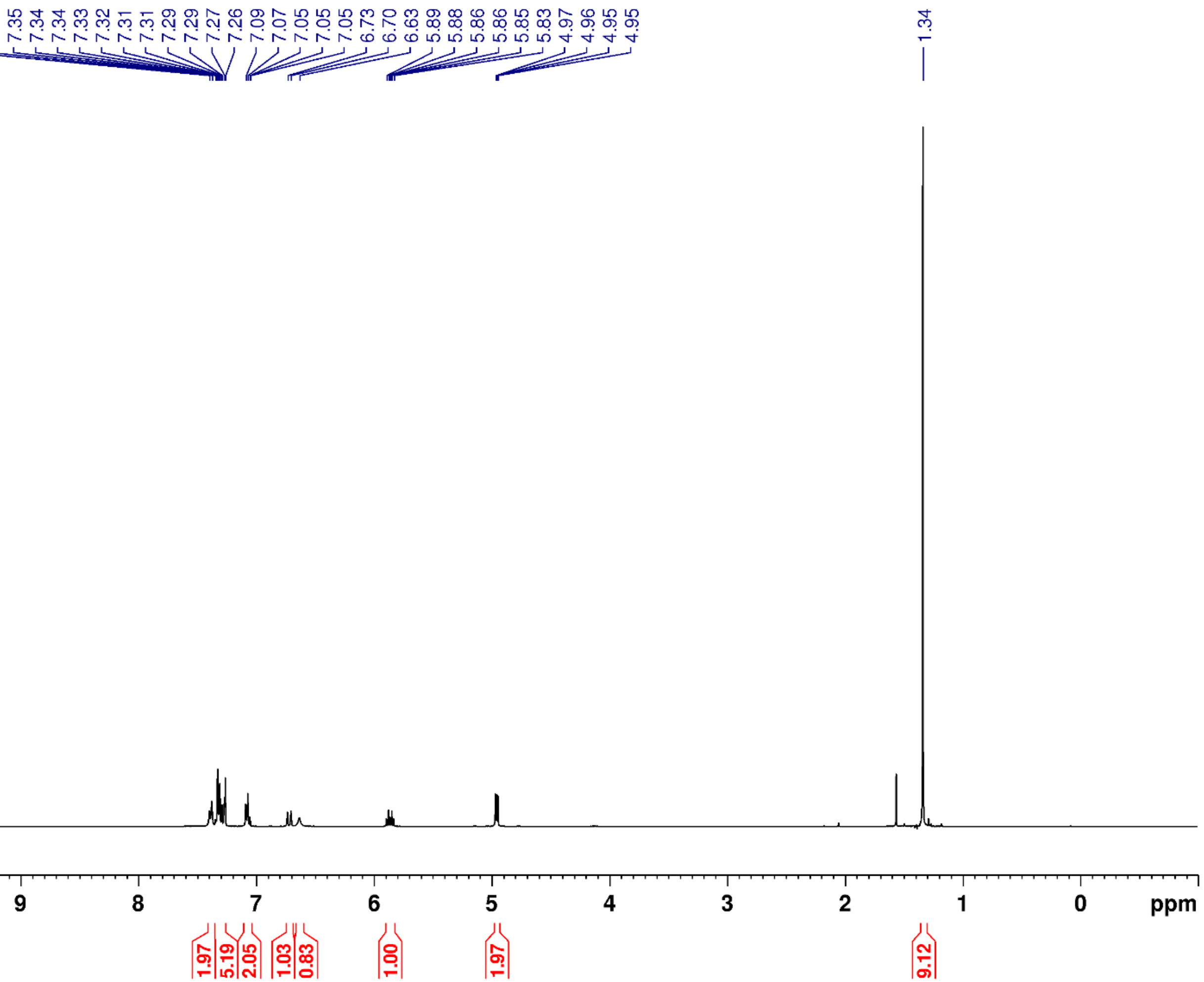

^{13}C NMR (101 MHz, CDCl$_3$) for (*Z*)-3-(3-(tert-butyl)phenyl)allyl phenylcarbamate

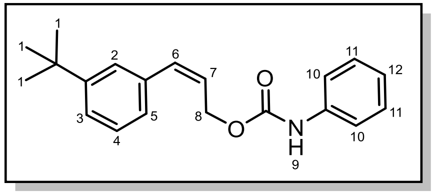

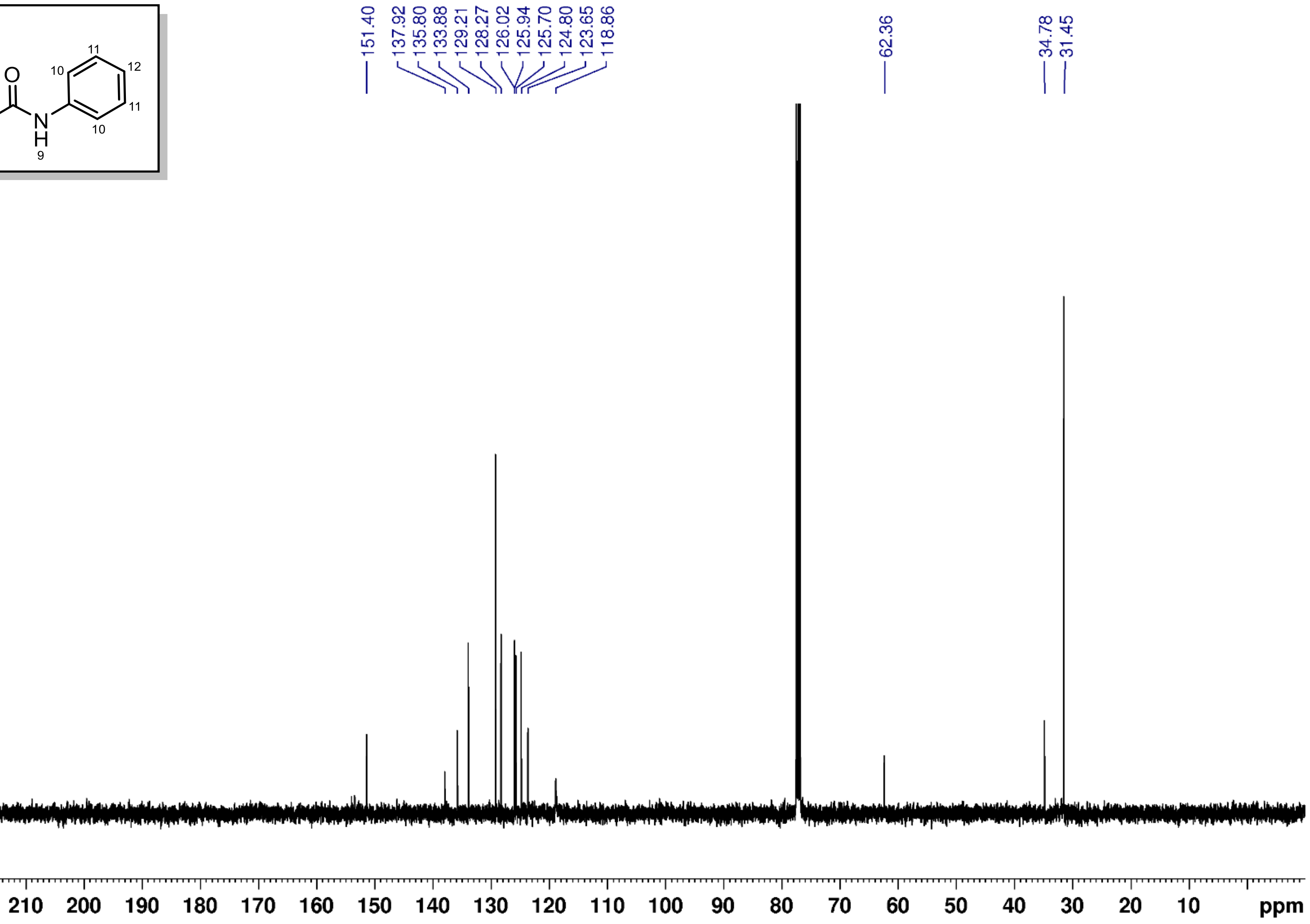

^{1}H NMR (400 MHz, CDCl$_3$) for (*Z*)-3-(3-methoxyphenyl)allyl phenylcarbamate

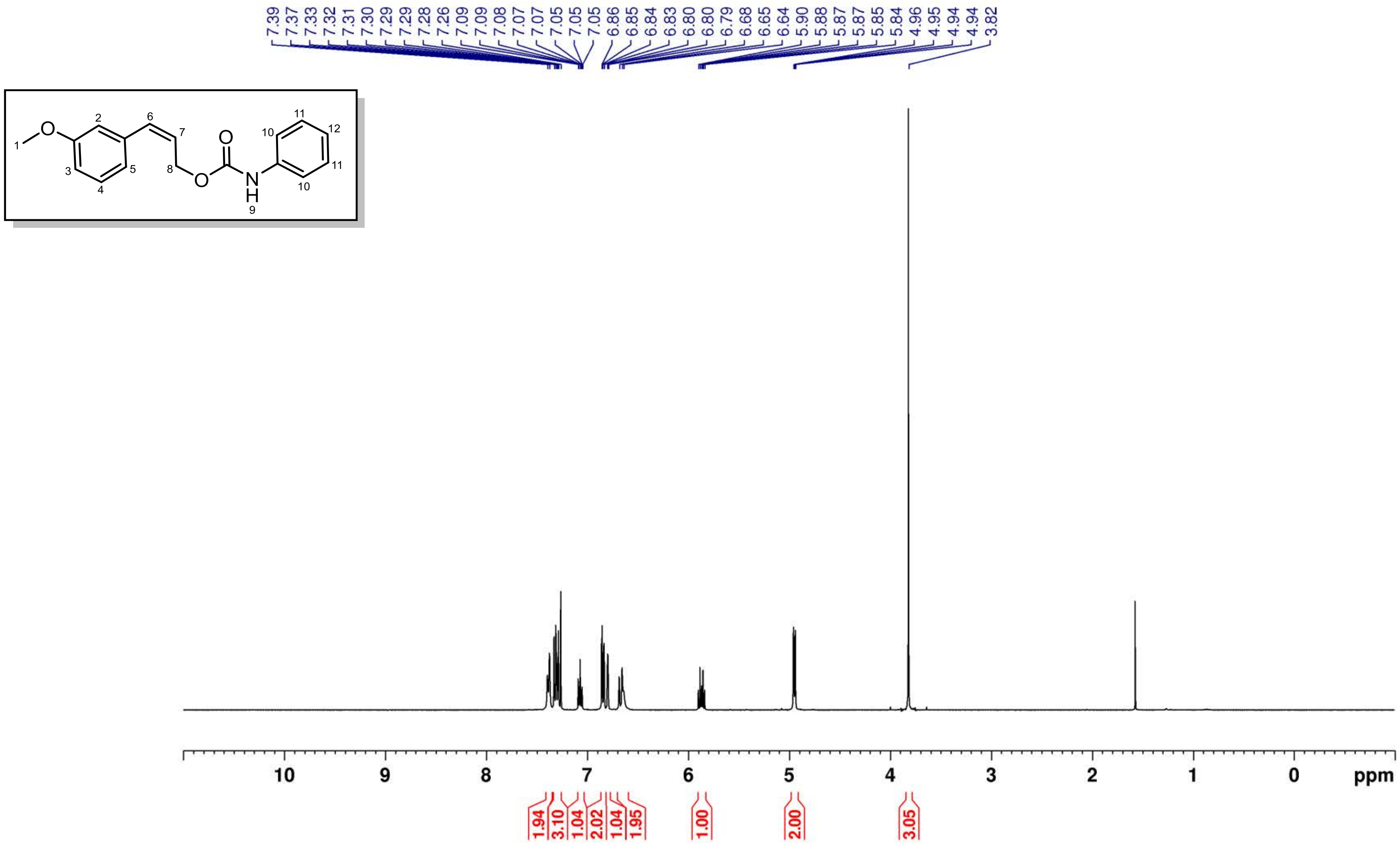

^{13}C NMR (101 MHz, CDCl$_3$) for (*Z*)-3-(3-methoxyphenyl)allyl phenylcarbamate

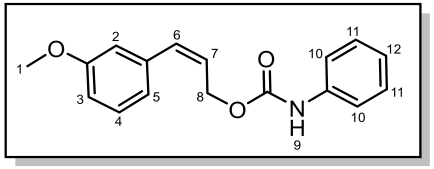

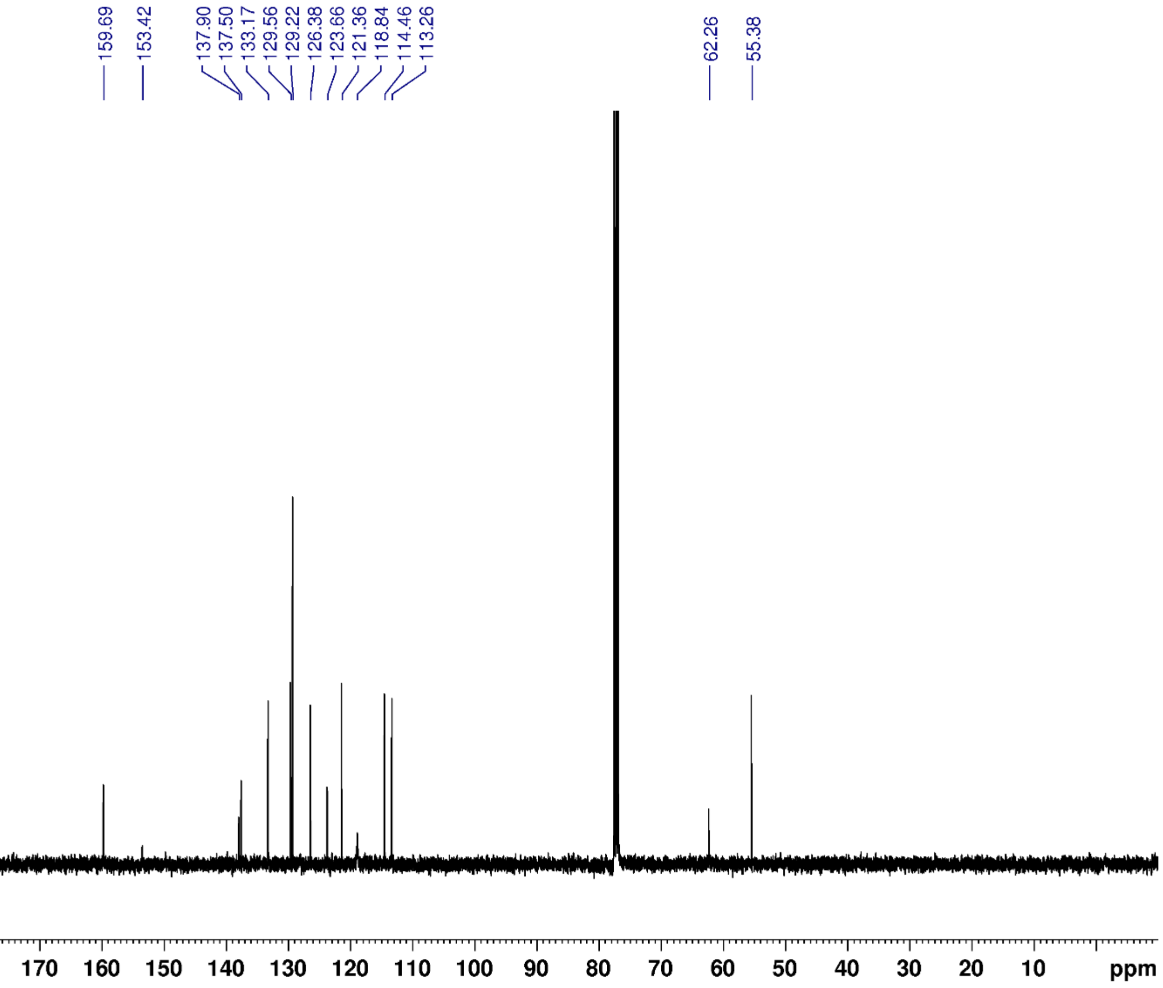

^{1}H NMR (500 MHz, CDCl$_3$) for (*Z*)-3-(3-bromophenyl)allyl phenylcarbamate

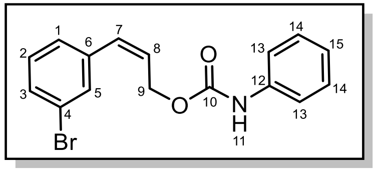

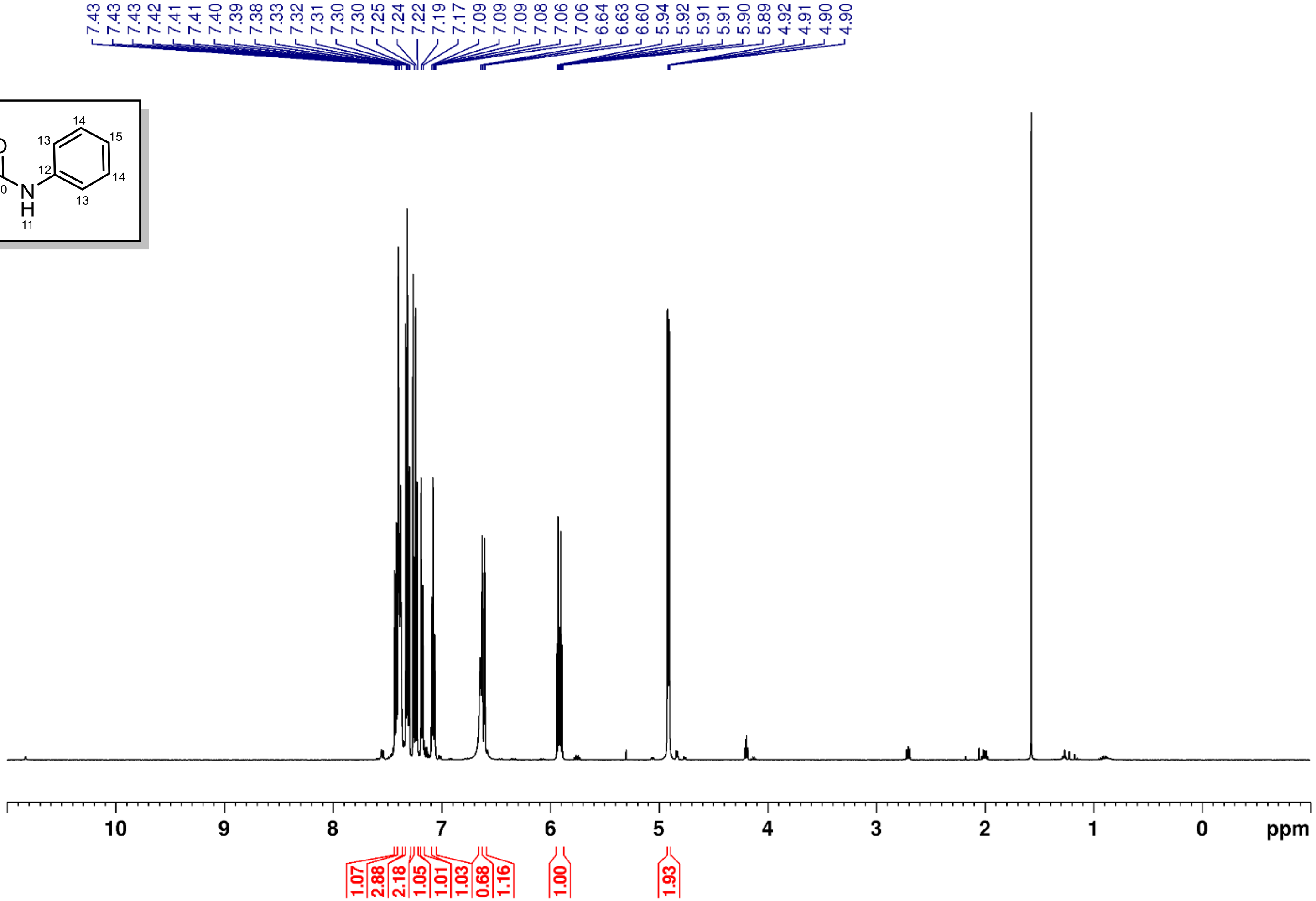

^{13}C NMR (126 MHz, $CDCl_3$) for (*Z*)-3-(3-bromophenyl)allyl phenylcarbamate

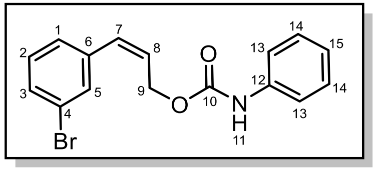

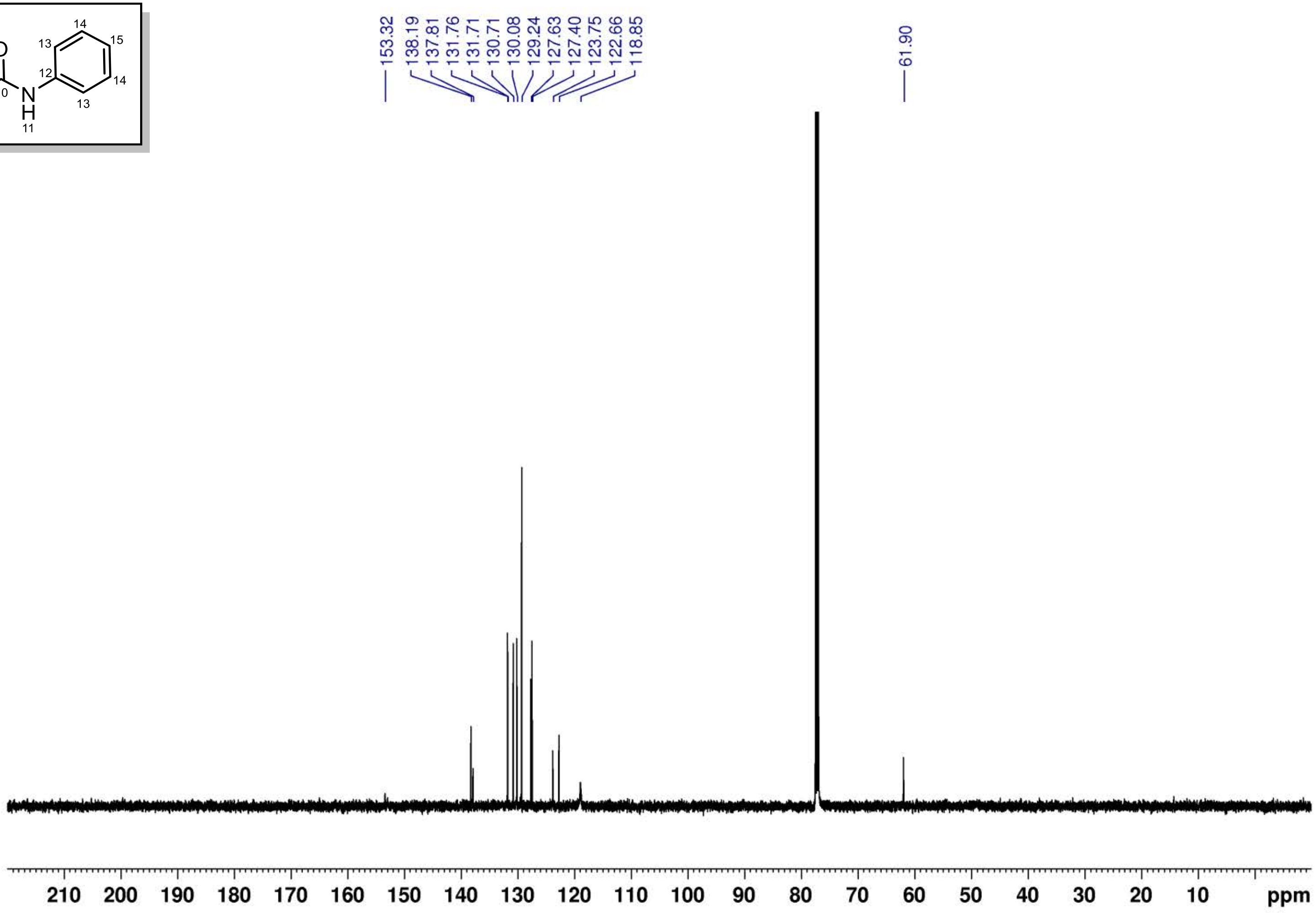

^{1}H NMR (500 MHz, CDCl$_3$) for ethyl (*Z*)-3-(3-((phenylcarbamoyl)oxy)prop-1-en-1-yl)benzoate

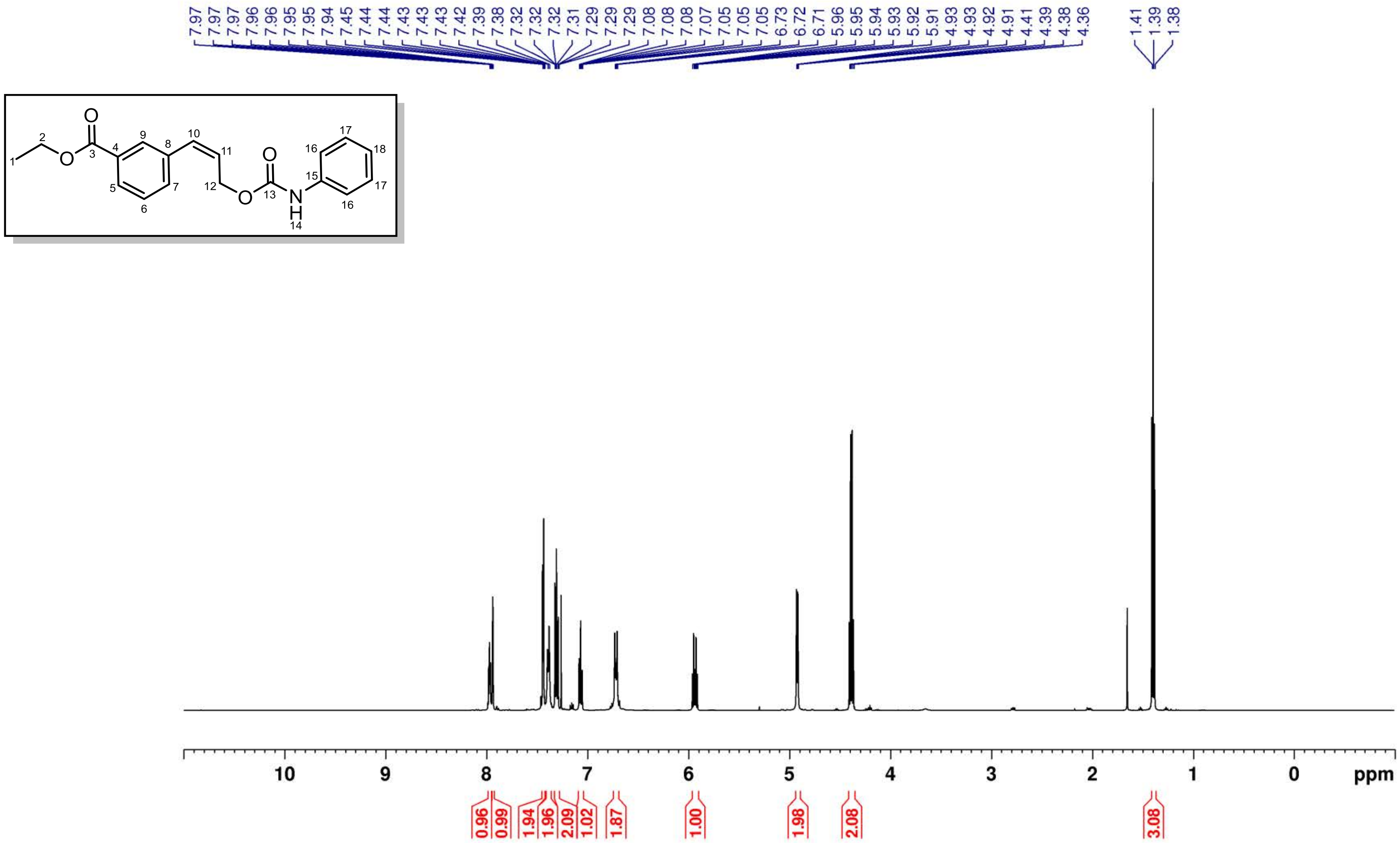

^{13}C NMR (126 MHz, CDCl$_3$) for ethyl (*Z*)-3-(3-((phenylcarbamoyl)oxy)prop-1-en-1-yl)benzoate

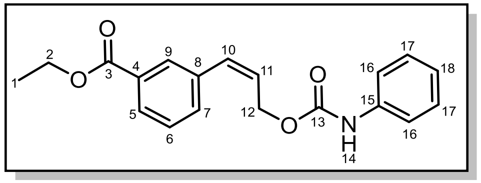

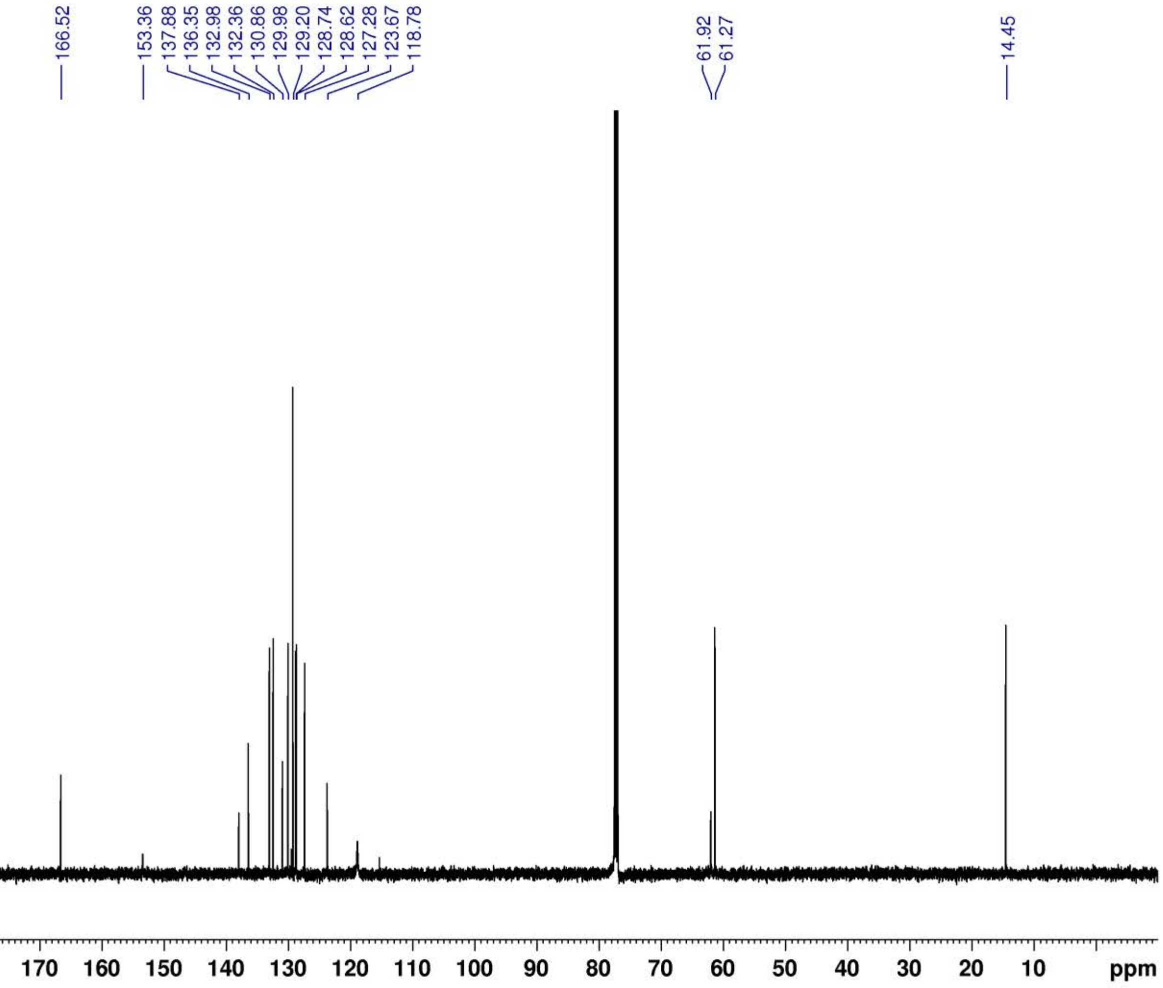

^{1}H NMR (700 MHz, CDCl$_3$) for (*Z*)-3-(3,4-dichlorophenyl)allyl phenylcarbamate

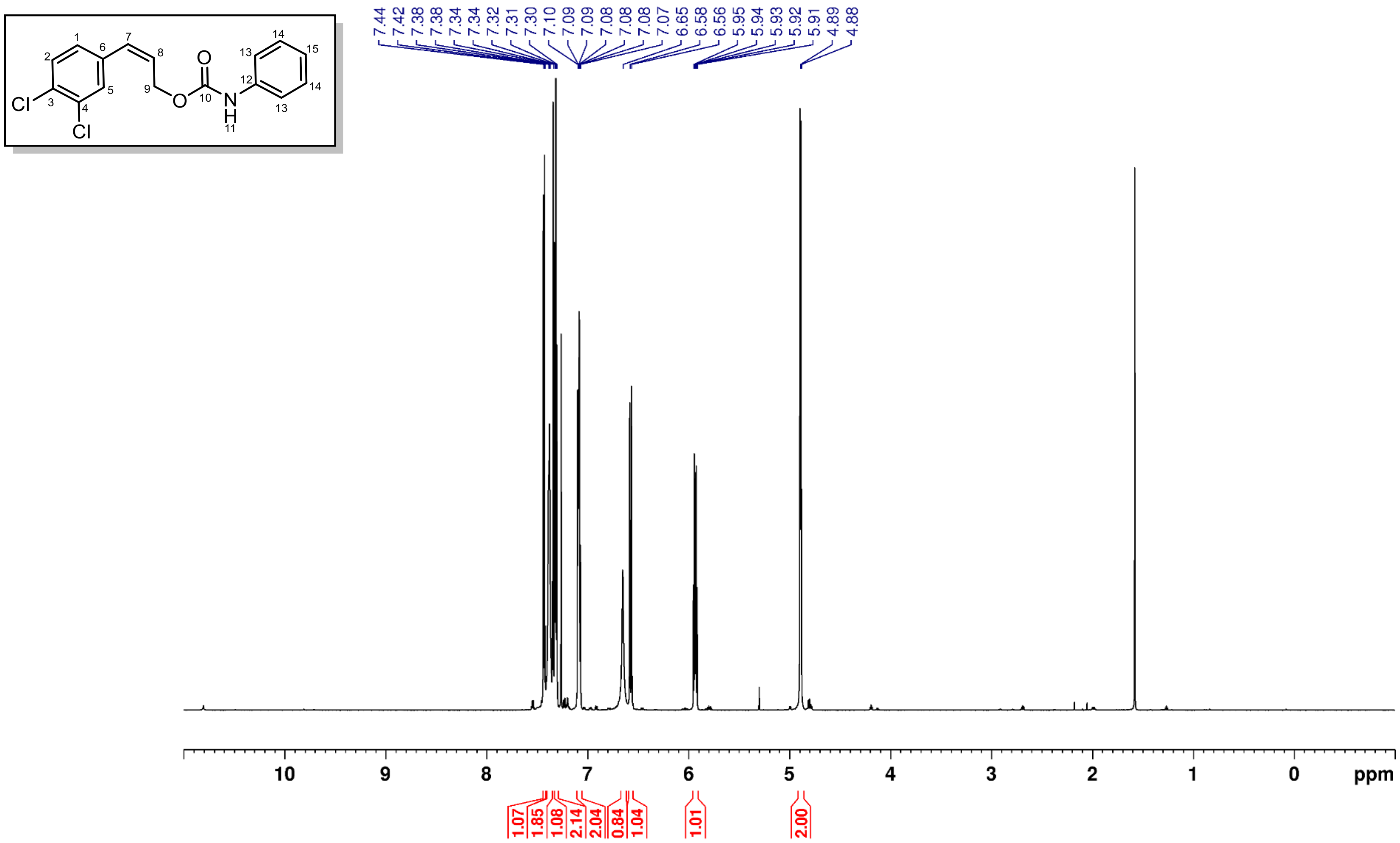

^{13}C NMR (176 MHz, CDCl$_3$) for (*Z*)-3-(3,4-dichlorophenyl)allyl phenylcarbamate

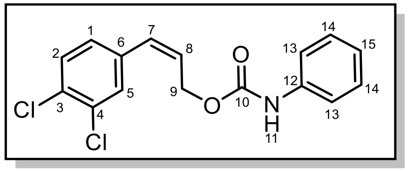

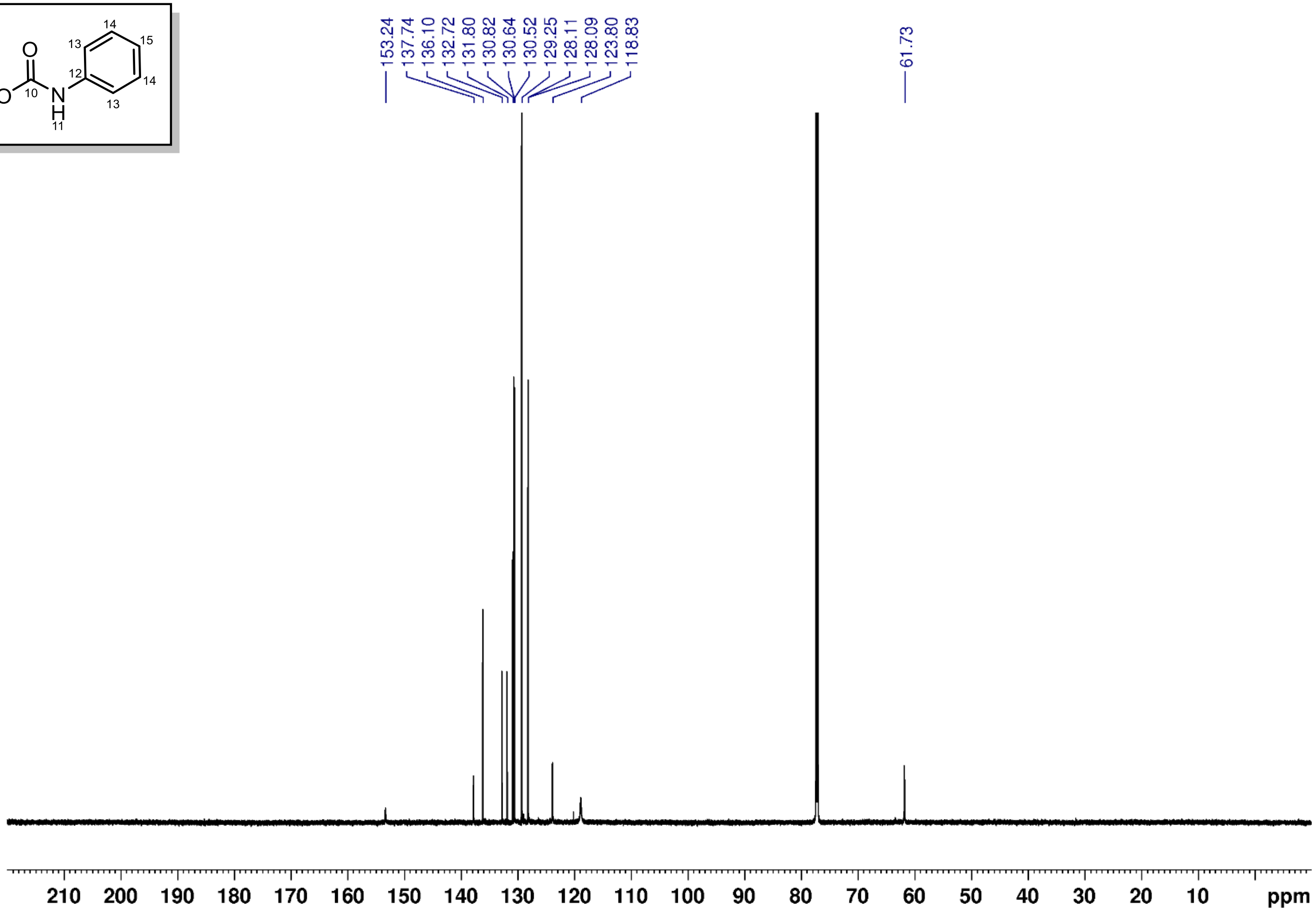

^{1}H NMR (500 MHz, CDCl$_3$) for (*Z*)-3-(4-(*tert*-butyl)phenyl)allyl phenylcarbamate

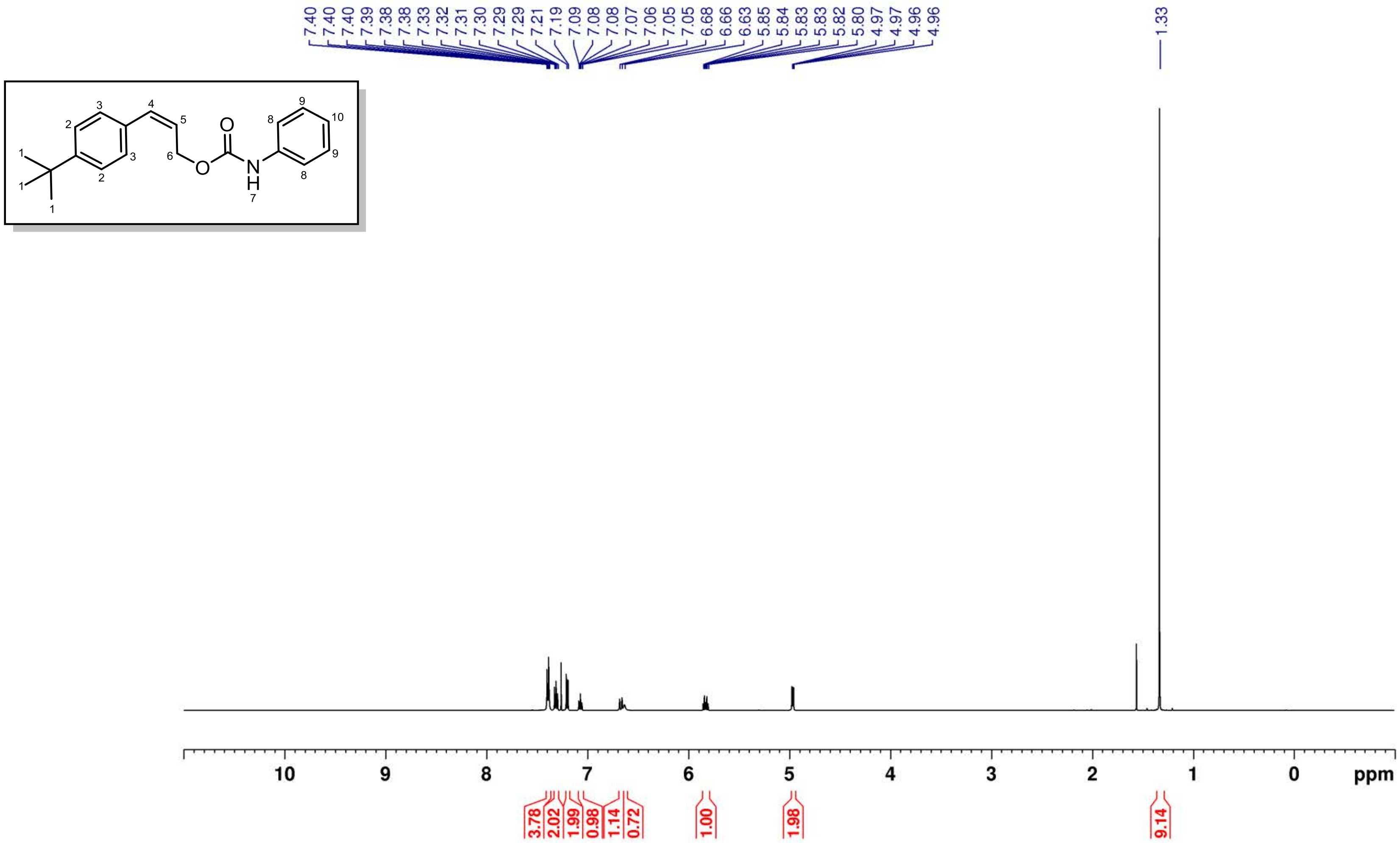

^{13}C NMR (126 MHz, CDCl$_3$) for (*Z*)-3-(4-(*tert*-butyl)phenyl)allyl phenylcarbamate

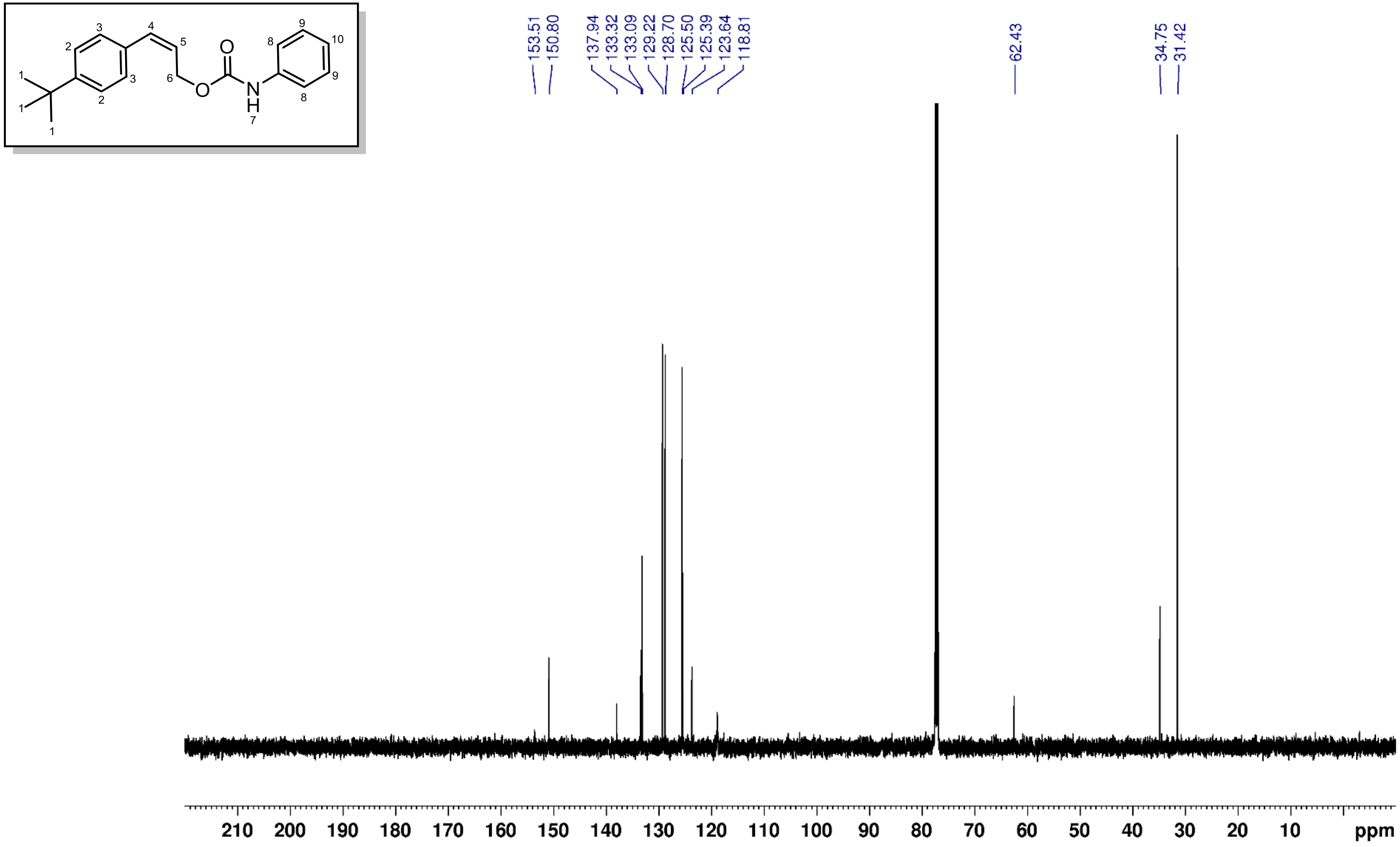

^{1}H NMR (700 MHz, CDCl$_3$) for (*Z*)-3-(4-(trifluoromethoxy)phenyl)allyl phenylcarbamate

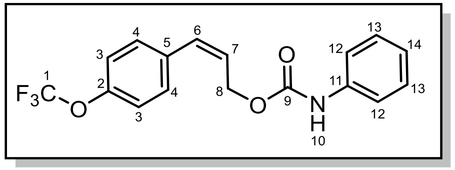

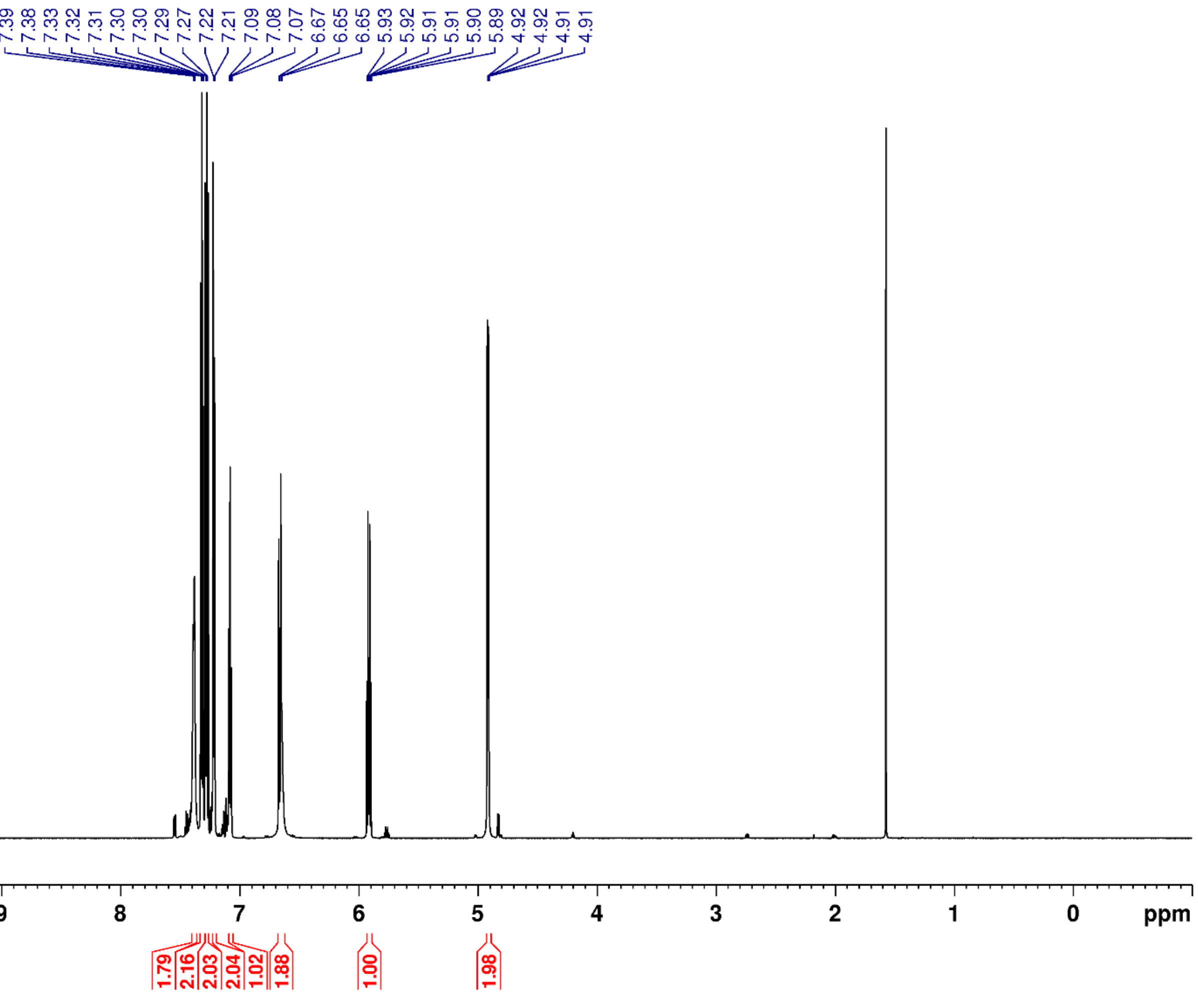

^{13}C NMR (101 MHz, $CDCl_3$) for (*Z*)-3-(4-(trifluoromethoxy)phenyl)allyl phenylcarbamate

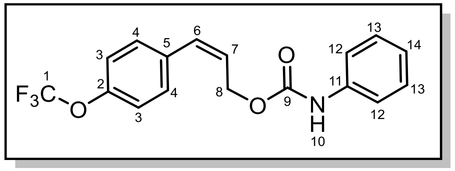

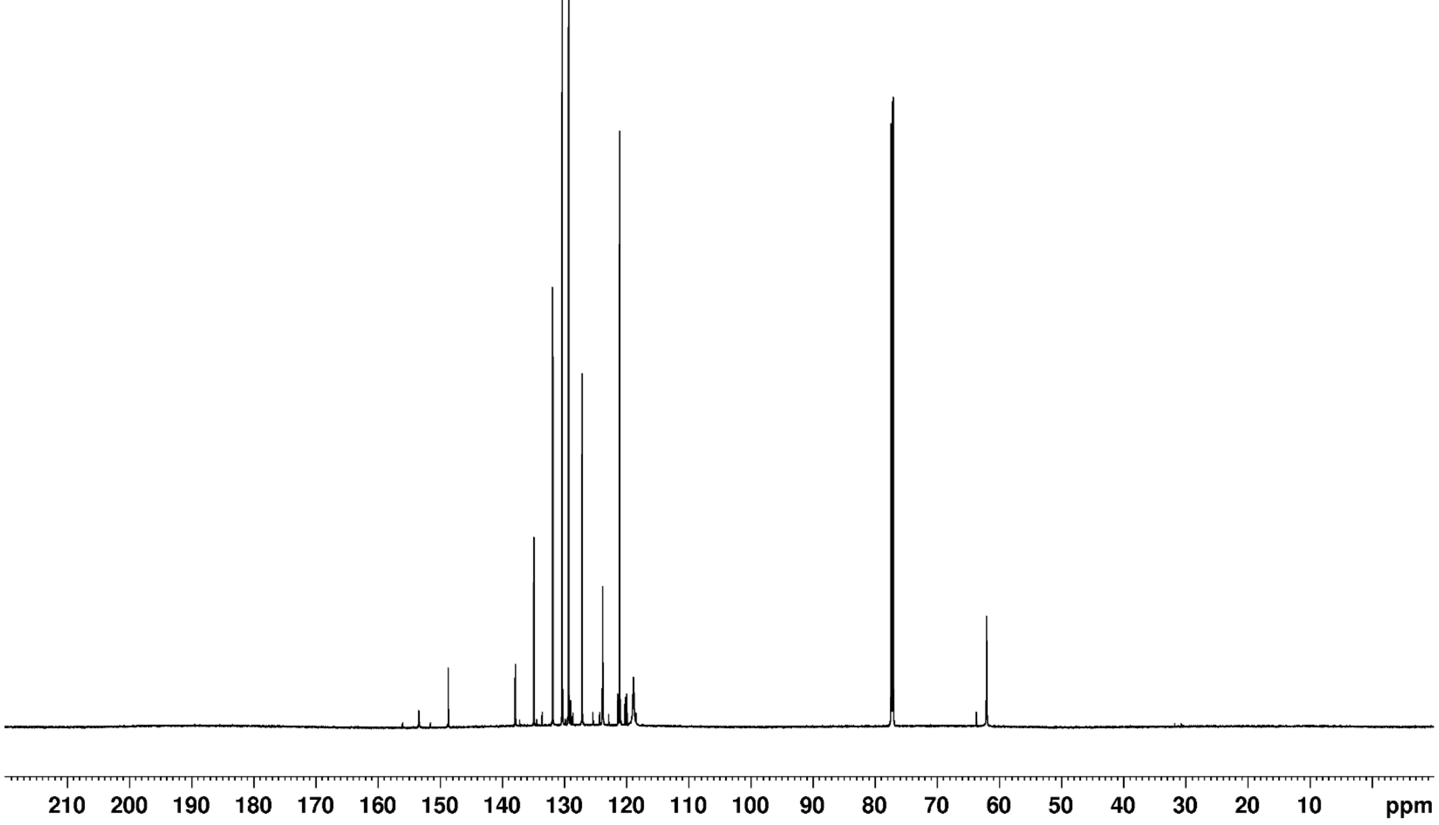

^{19}F NMR (376 MHz, $CDCl_3$) for (*Z*)-3-(4-(trifluoromethoxy)phenyl)allyl phenylcarbamate

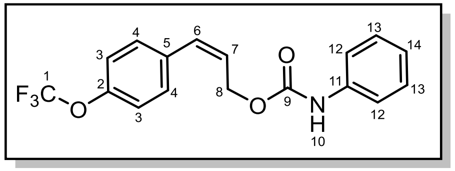

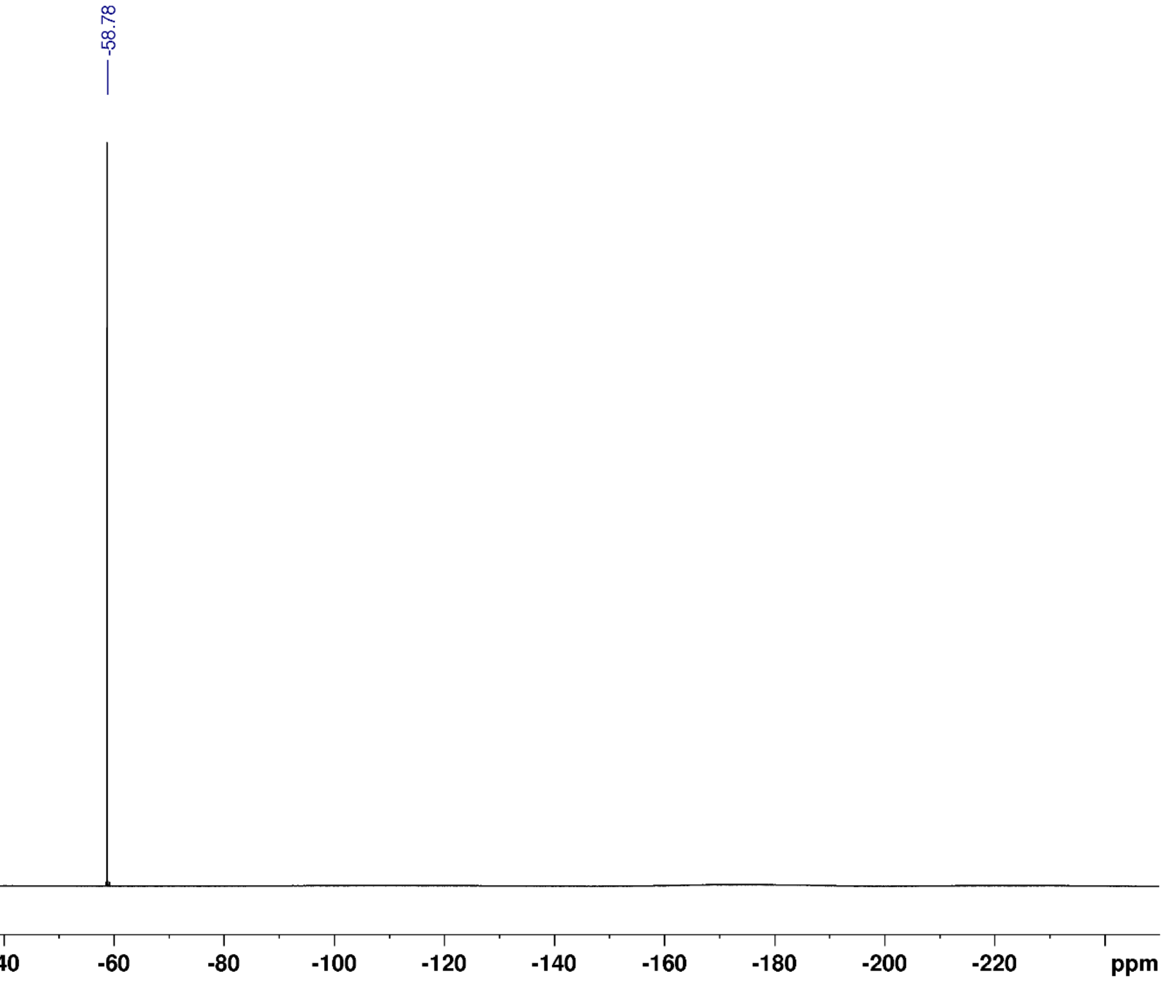

¹H NMR (700 MHz, CDCl$_3$) for (*Z*)-3-(4-chlorophenyl)allyl phenylcarbamate

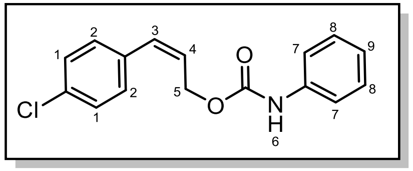

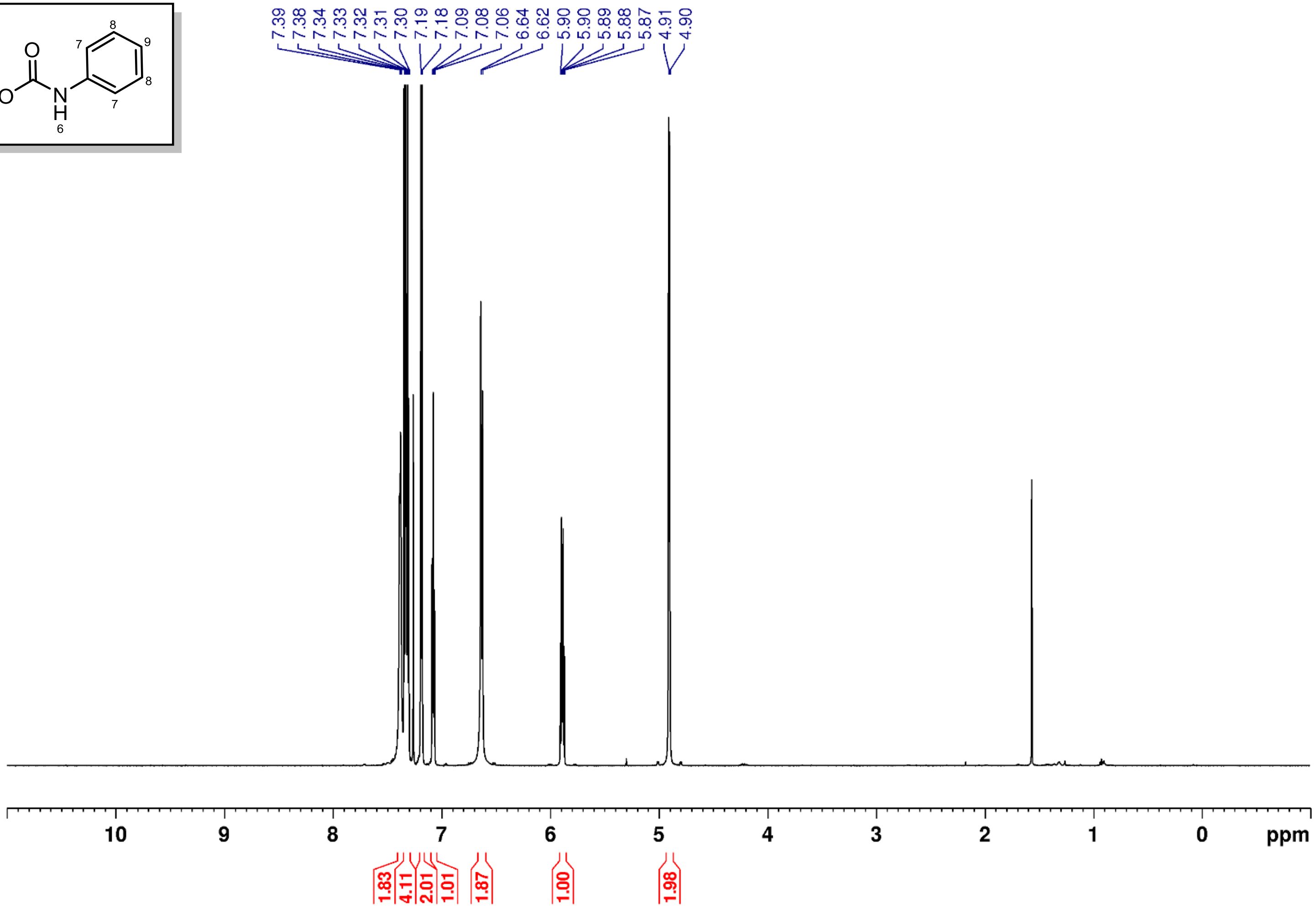

^{13}C NMR (176 MHz, CDCl$_3$) for (*Z*)-3-(4-chlorophenyl)allyl phenylcarbamate

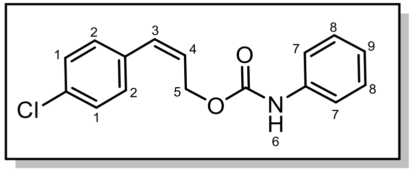

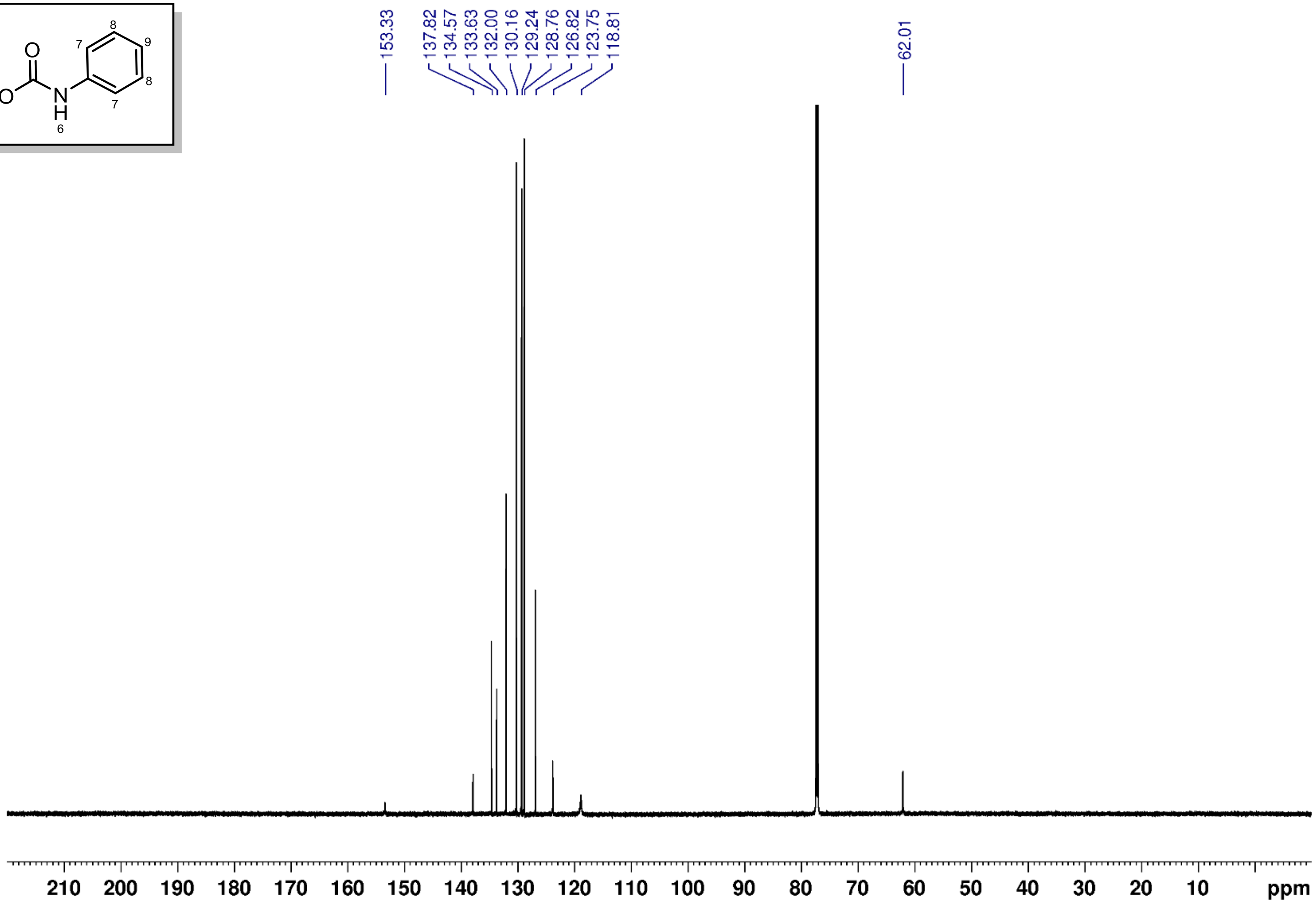

^{1}H NMR (700 MHz, CDCl$_3$) for (*Z*)-3-(4-(trifluoromethyl)phenyl)allyl phenylcarbamate

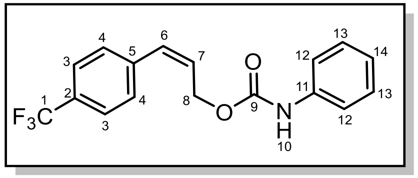

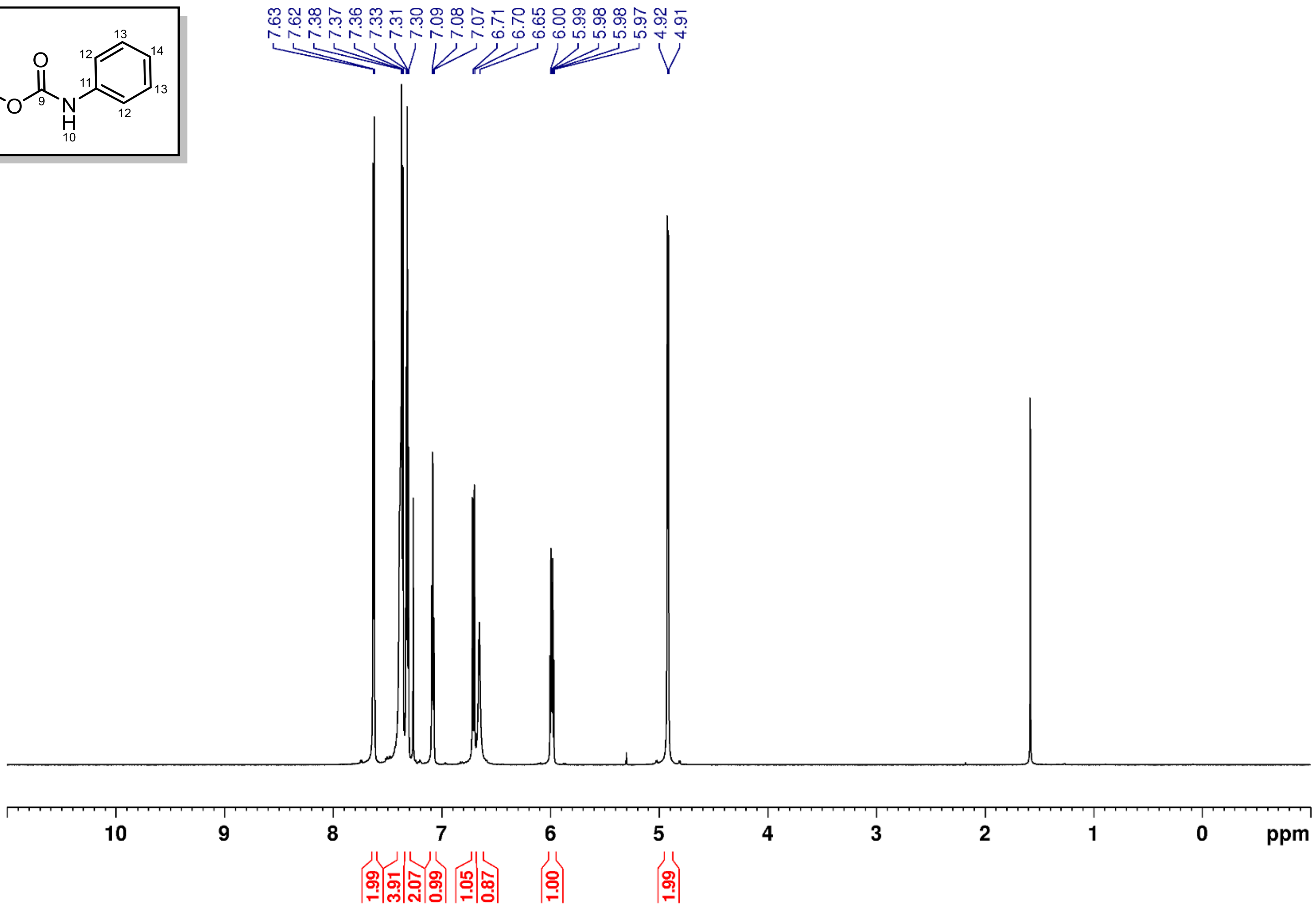

^{13}C NMR (176 MHz, $CDCl_3$) for (*Z*)-3-(4-(trifluoromethyl)phenyl)allyl phenylcarbamate

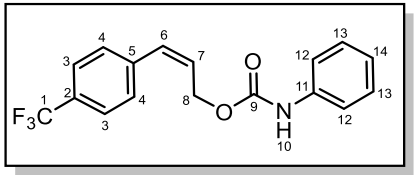

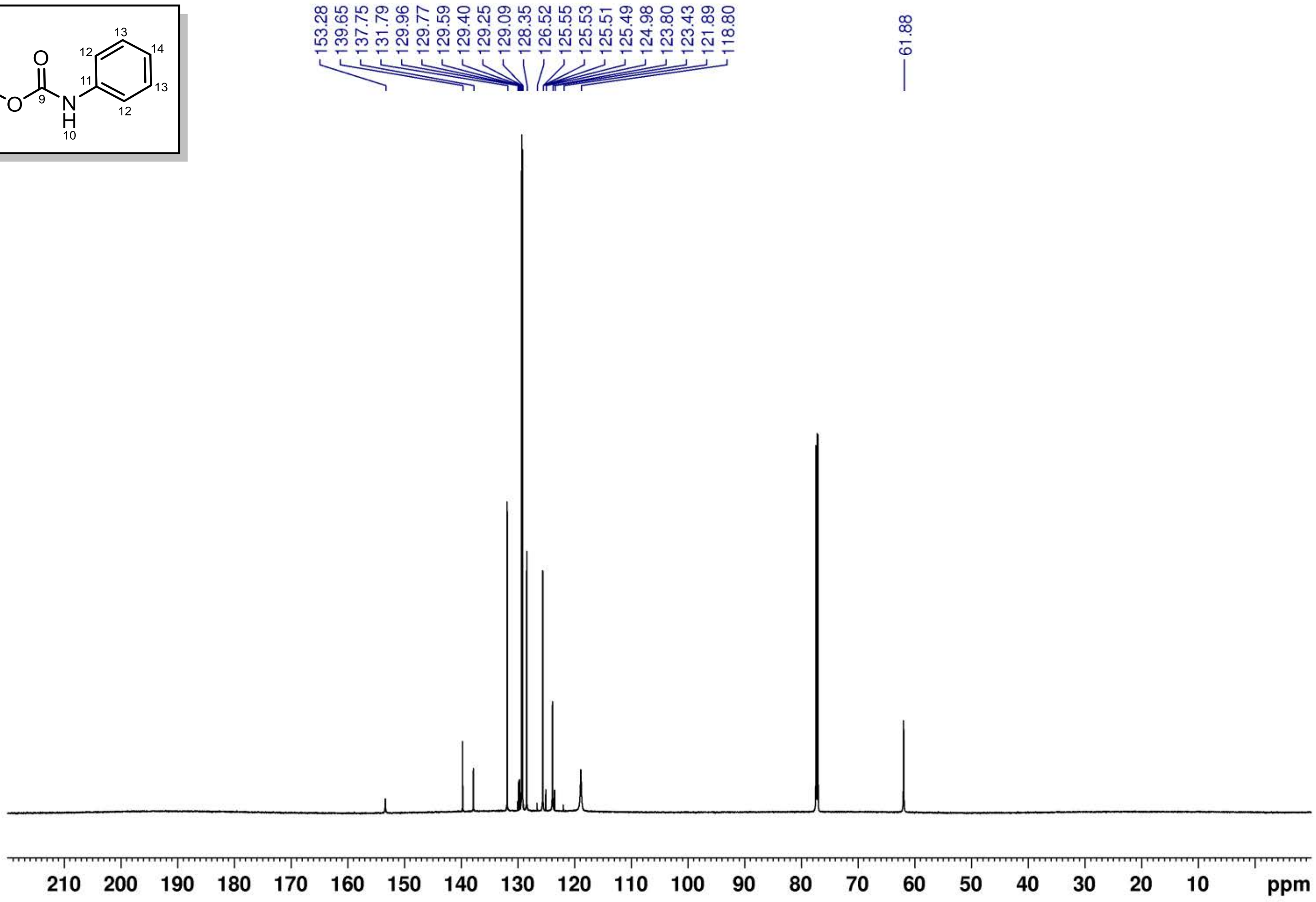

^{19}F NMR (376 MHz, CDCl$_3$) for (*Z*)-3-(4-(trifluoromethyl)phenyl)allyl phenylcarbamate

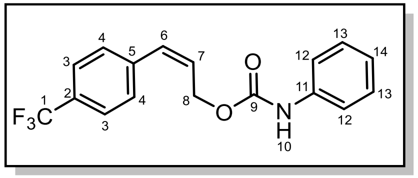

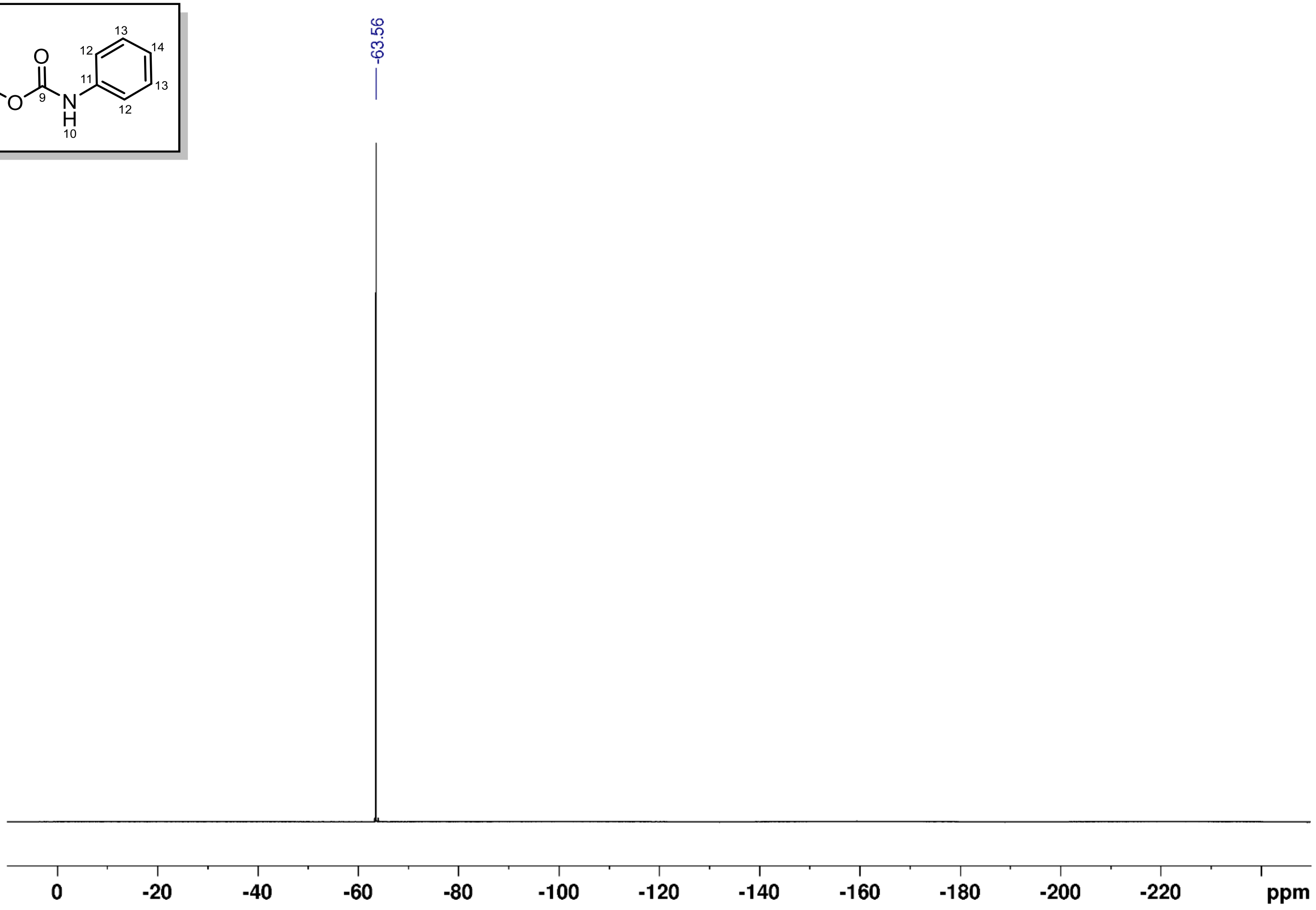

^{1}H NMR (400 MHz, CDCl$_3$) for (*Z*)-3-(naphthalen-1-yl)allyl phenylcarbamate

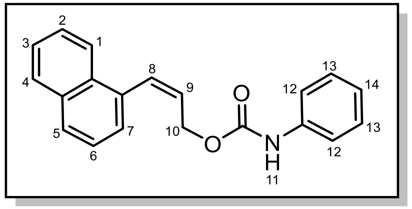

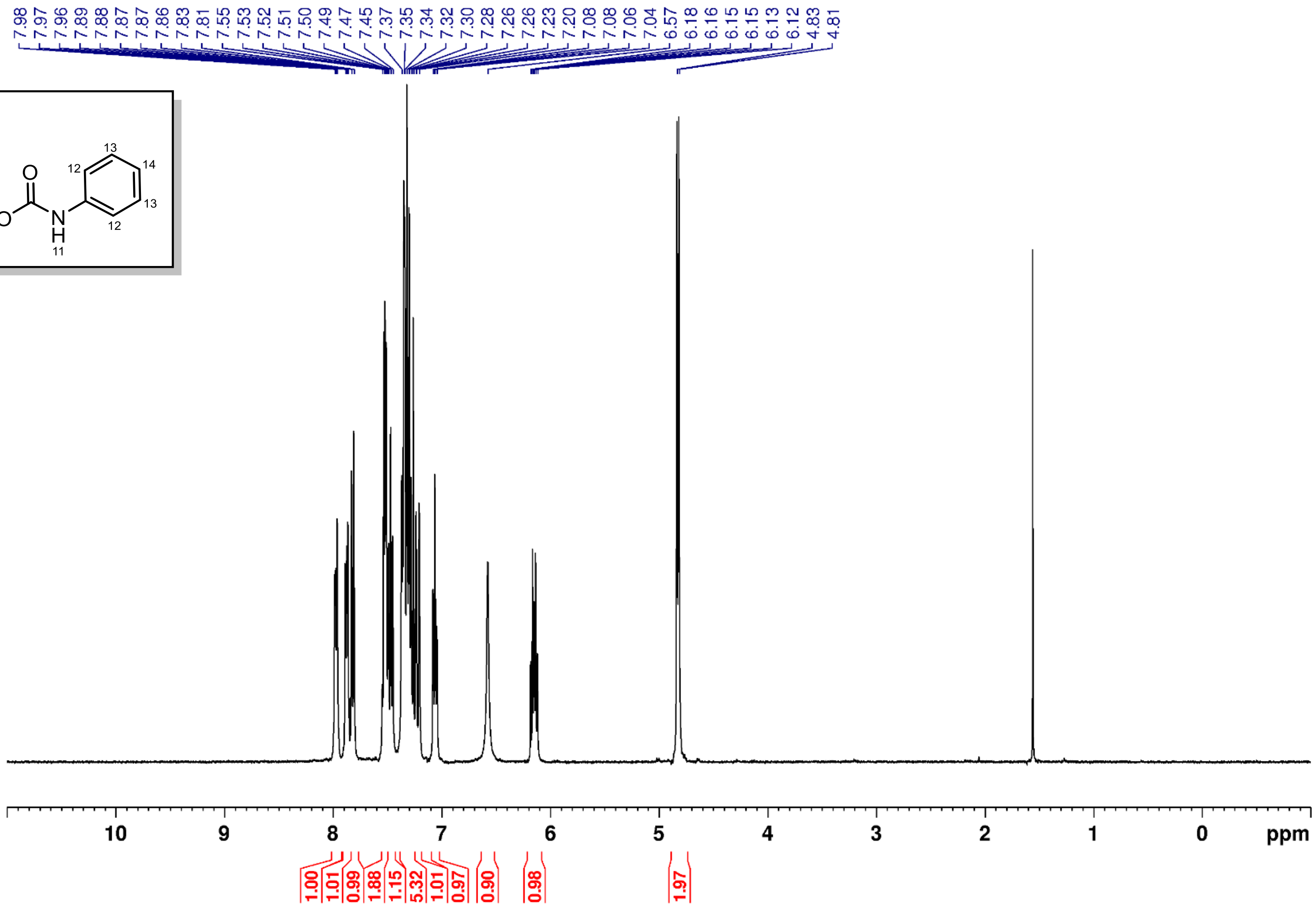

^{13}C NMR (101 MHz, CDCl$_3$) for (*Z*)-3-(naphthalen-1-yl)allyl phenylcarbamate

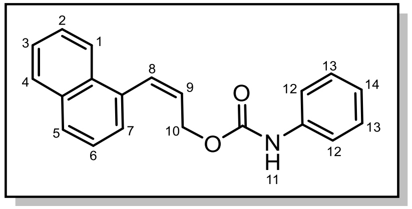

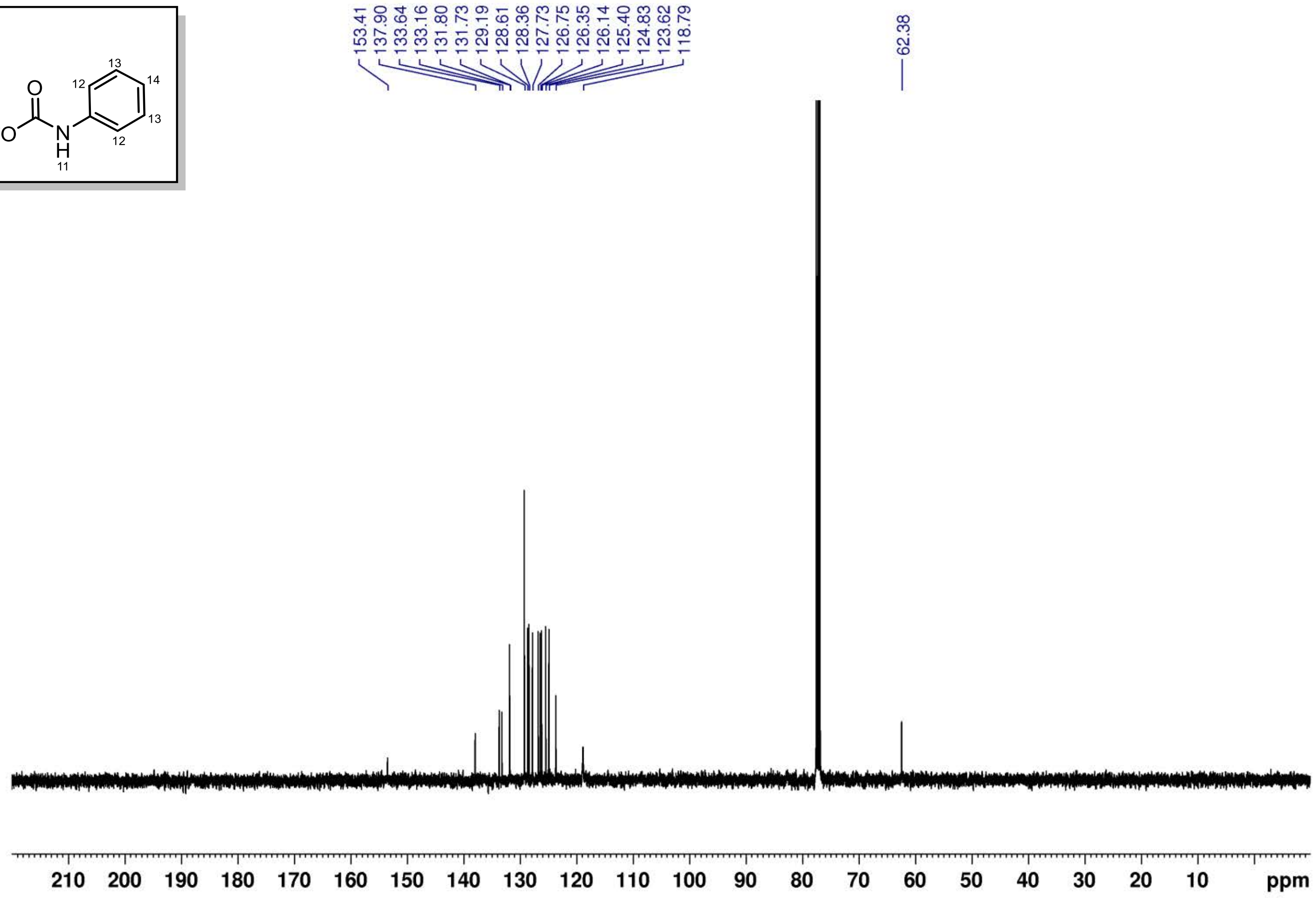

^{1}H NMR (500 MHz, $CDCl_3$) for (*Z*)-3-(naphthalen-2-yl)allyl phenylcarbamate

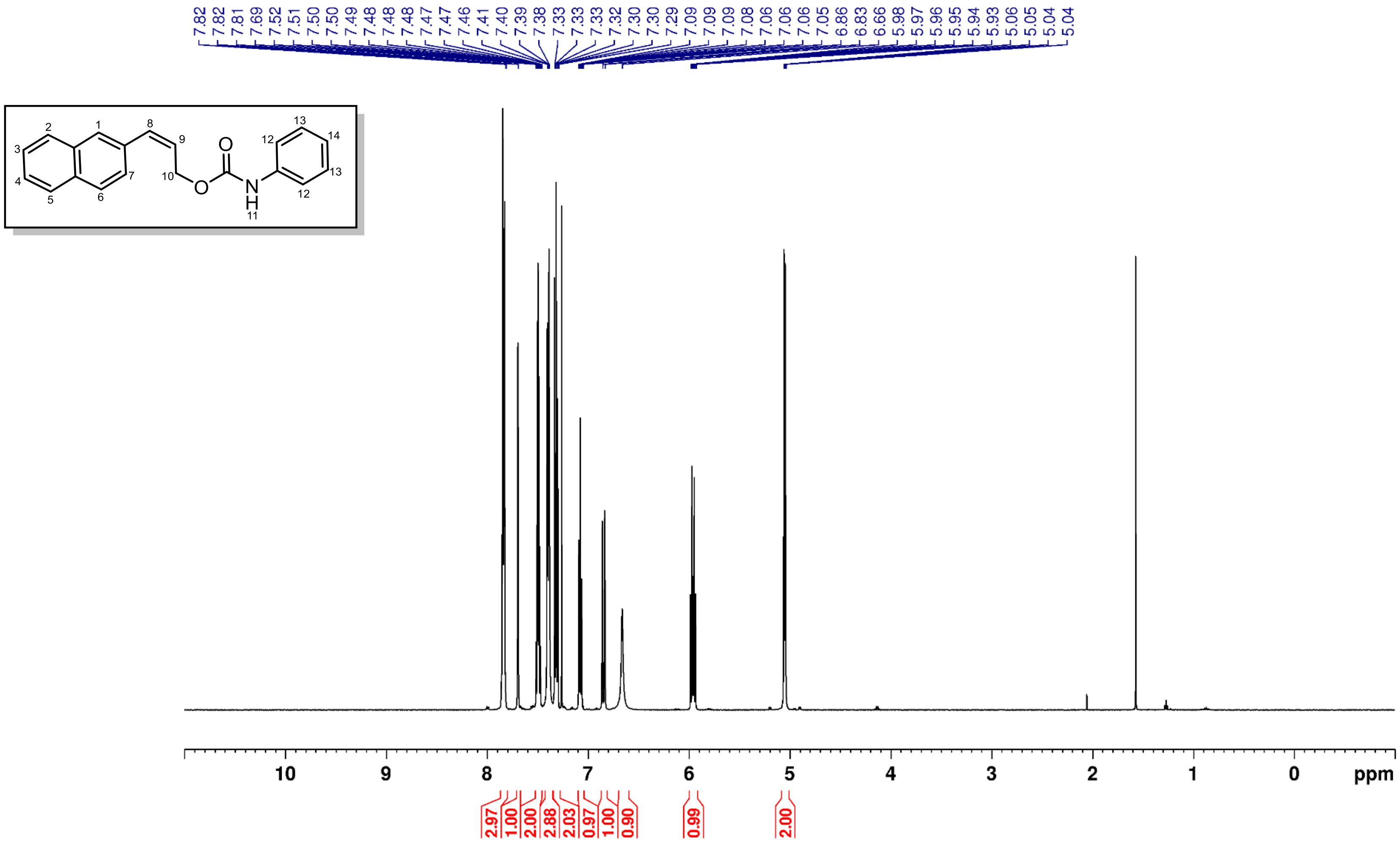

^{13}C NMR (126 MHz, CDCl$_3$) for (*Z*)-3-(naphthalen-2-yl)allyl phenylcarbamate

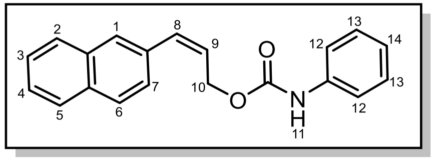

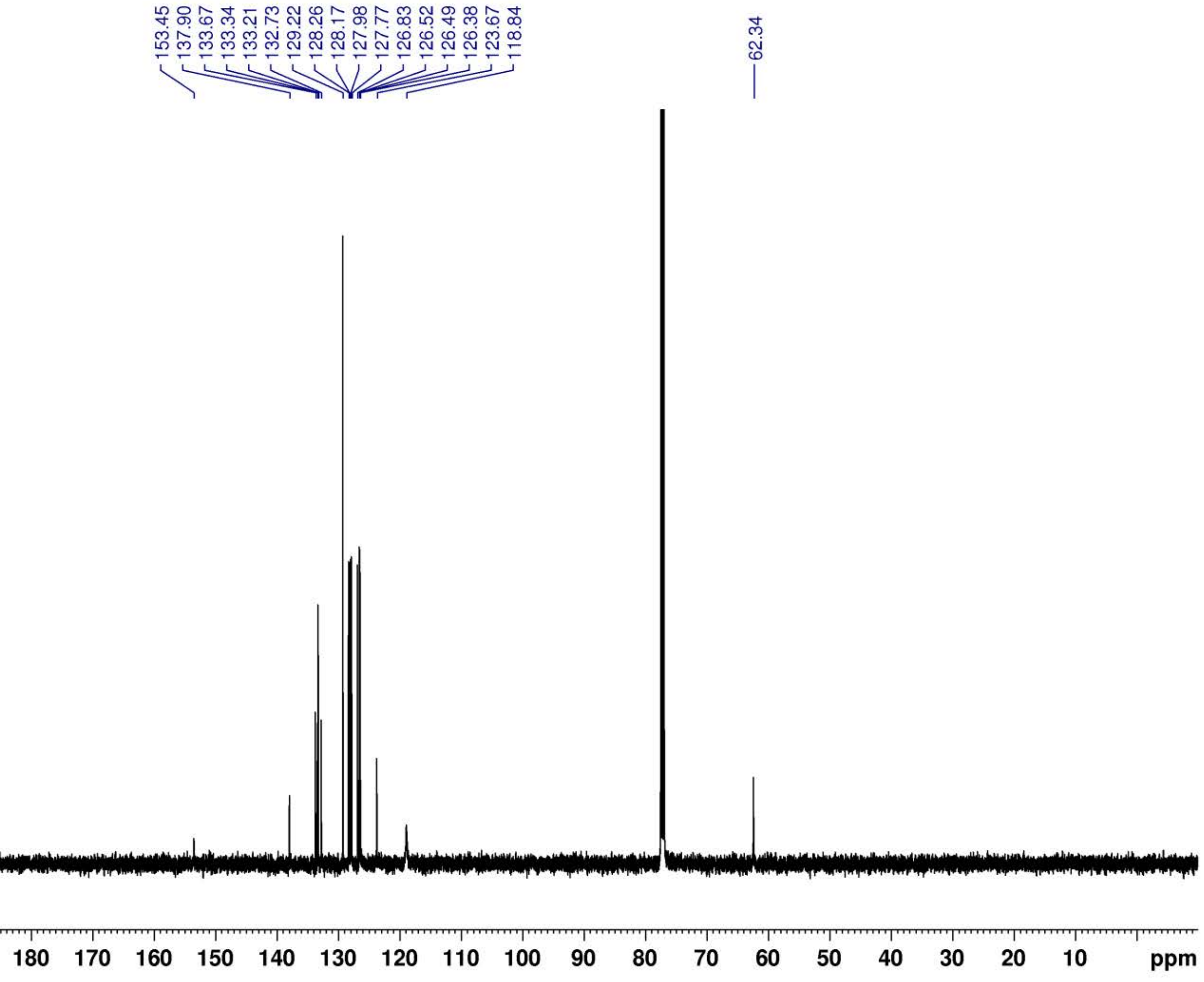

^{1}H NMR (400 MHz, CDCl$_3$) for (*Z*)-3-(thiophen-3-yl)allyl phenylcarbamate

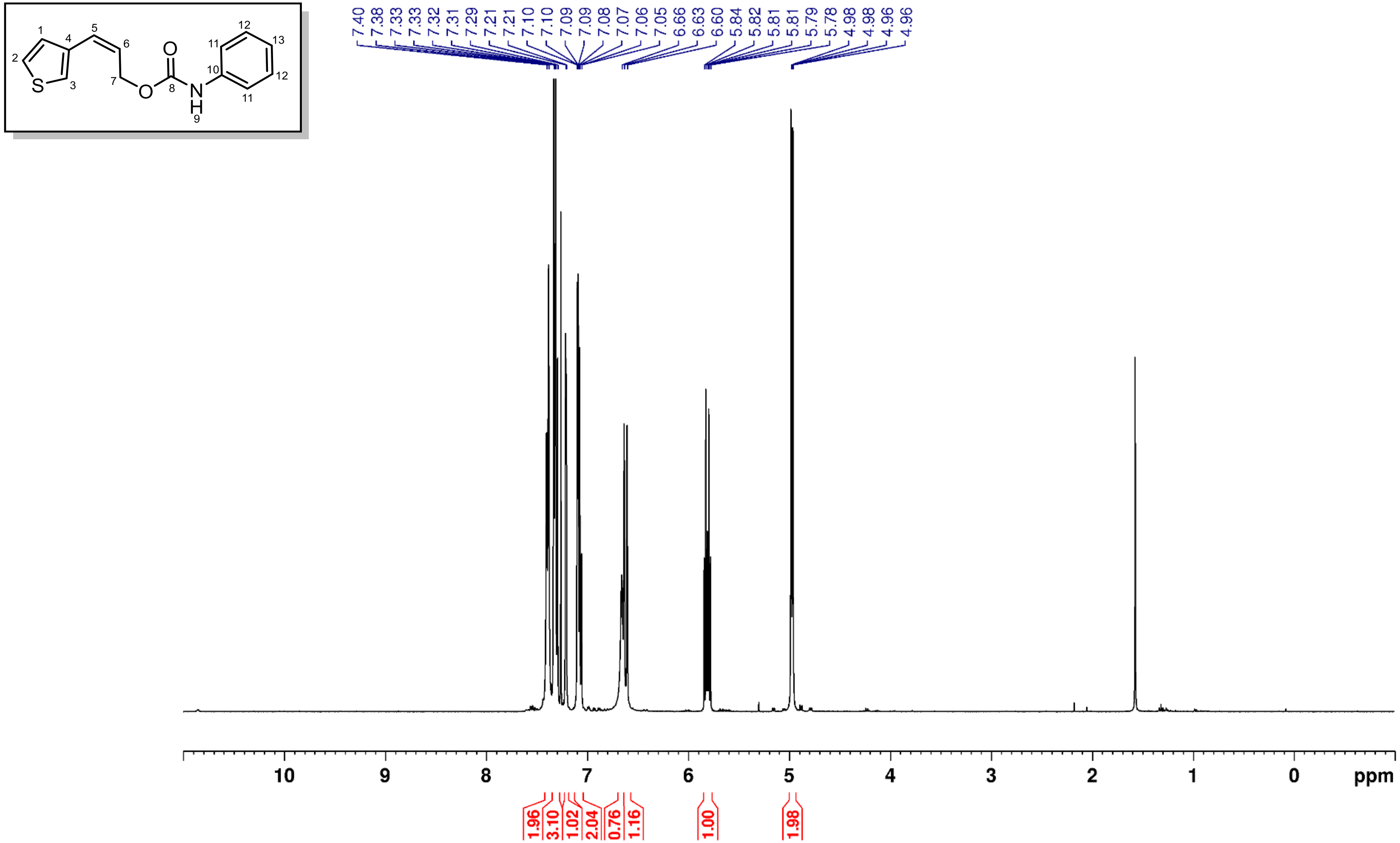

^{13}C NMR (101 MHz, CDCl$_3$) for (*Z*)-3-(thiophen-3-yl)allyl phenylcarbamate

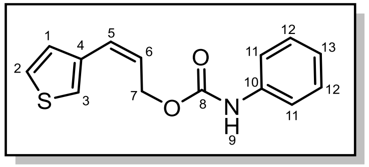

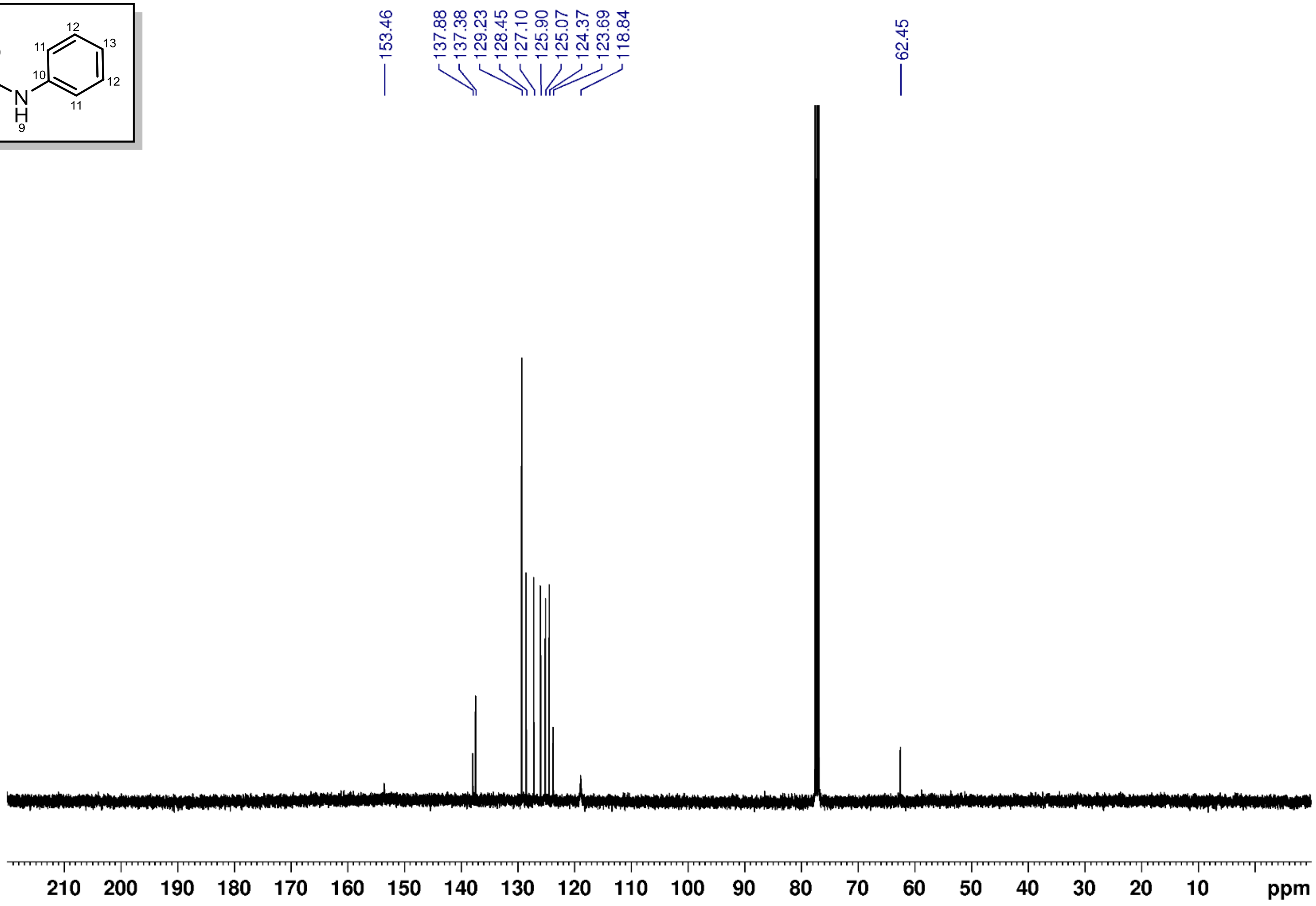

^{1}H NMR (400 MHz, CDCl$_3$) for (*Z*)-4-(4-(tert-butyl)phenyl)but-3-en-2-yl phenylcarbamate

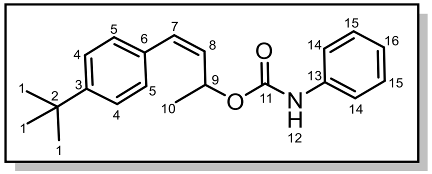

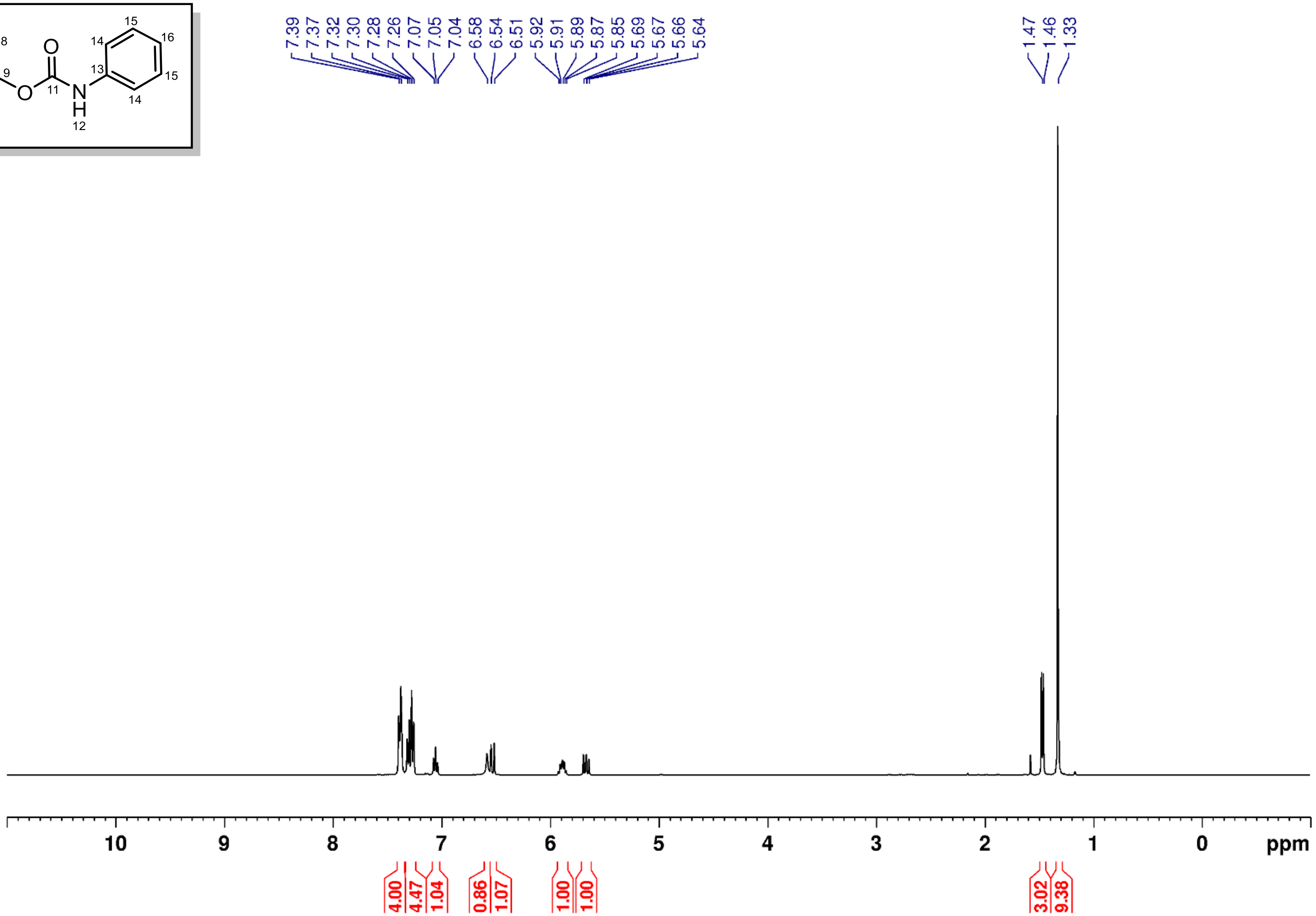

^{13}C NMR (101 MHz, CDCl$_3$) for (*Z*)-4-(4-(tert-butyl)phenyl)but-3-en-2-yl phenylcarbamate

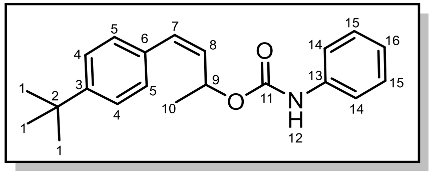

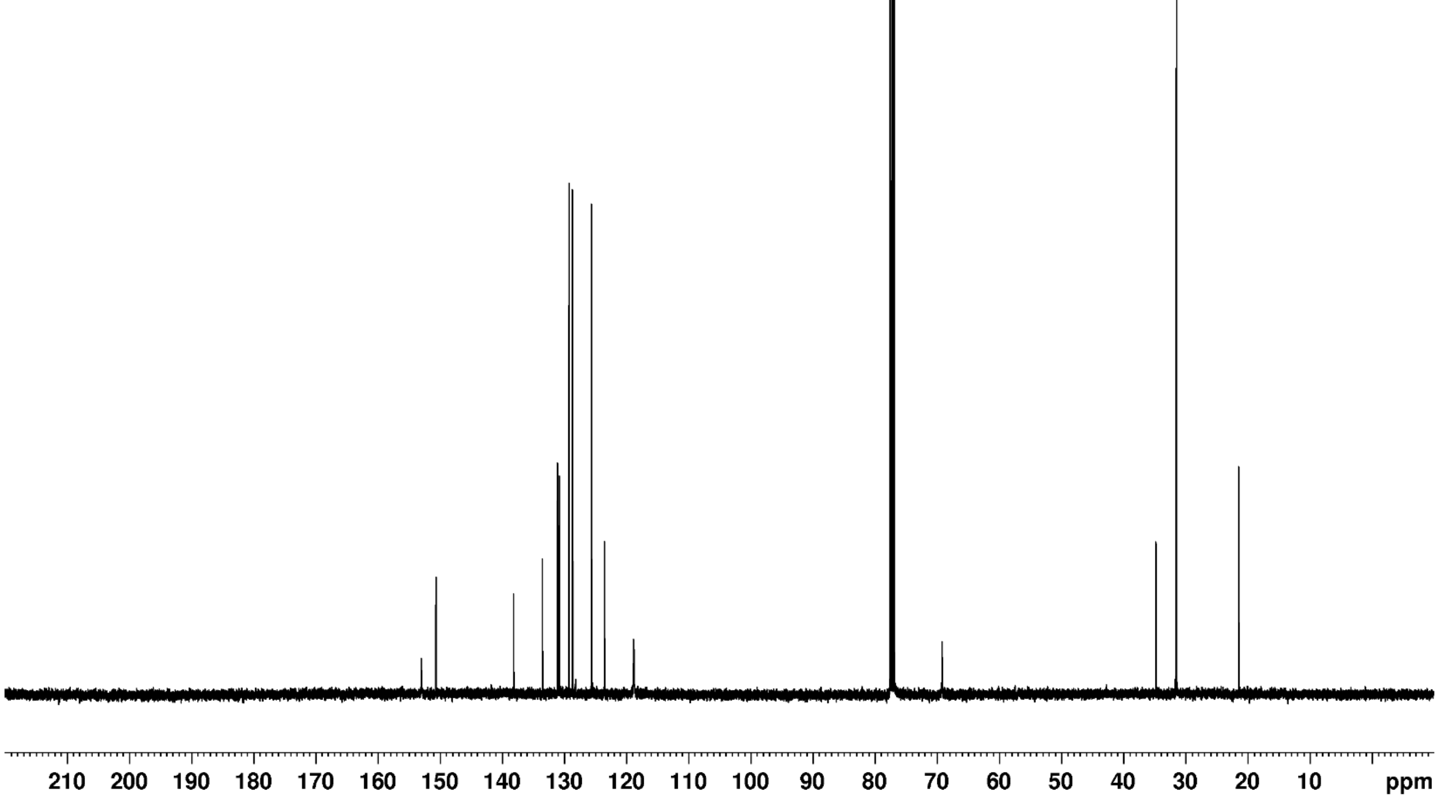

^{1}H NMR (400 MHz, CDCl$_3$) for (*Z*)-1-(4-(tert-butyl)phenyl)-4-methylpent-1-en-3-yl phenylcarbamate

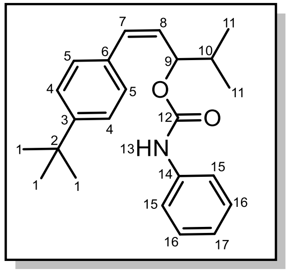

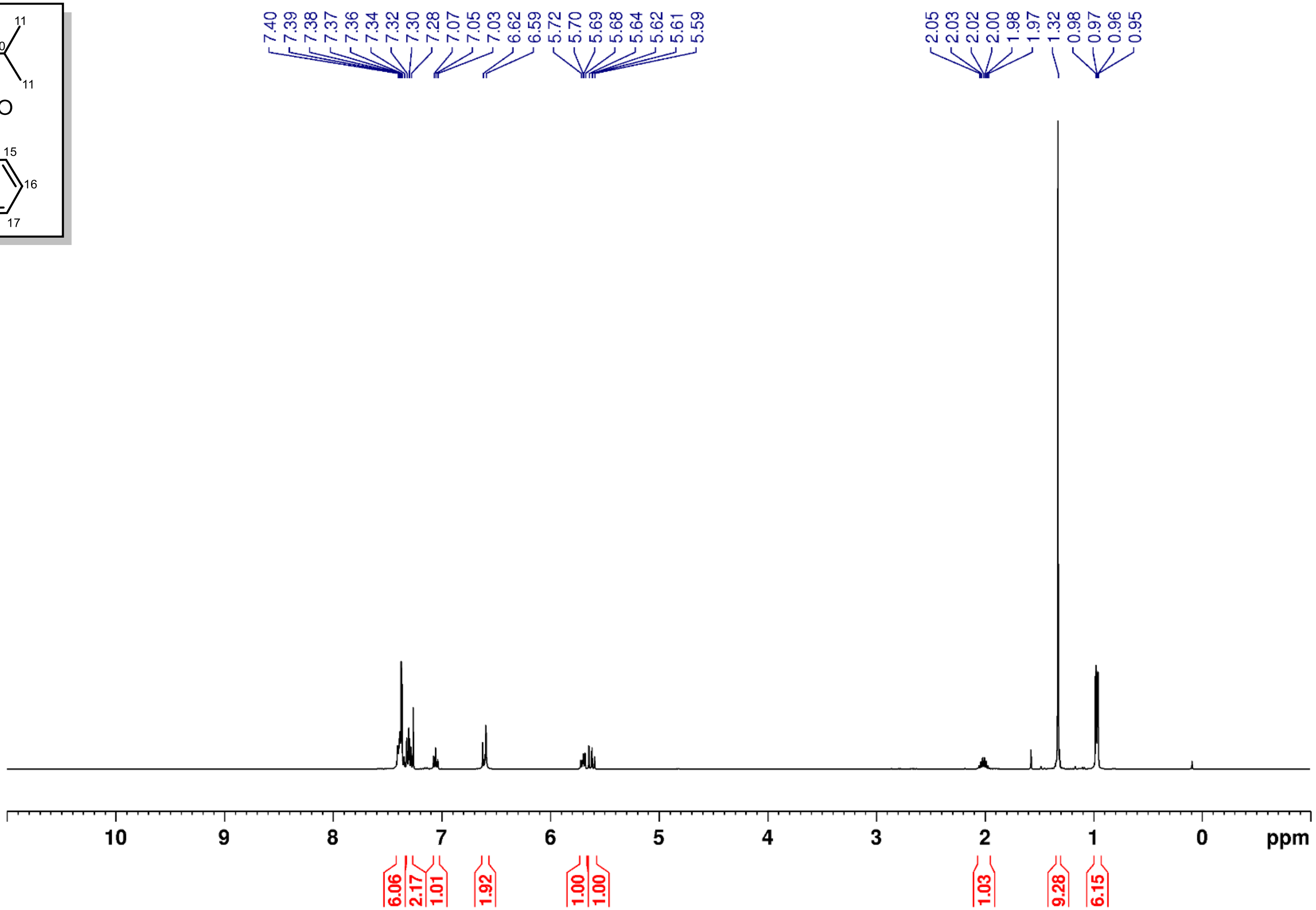

^{13}C NMR (101 MHz, CDCl$_3$) for (*Z*)-1-(4-(tert-butyl)phenyl)-4-methylpent-1-en-3-yl phenylcarbamate

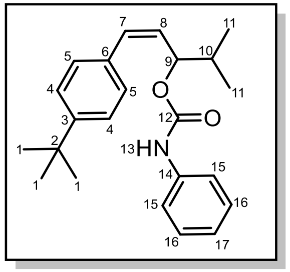

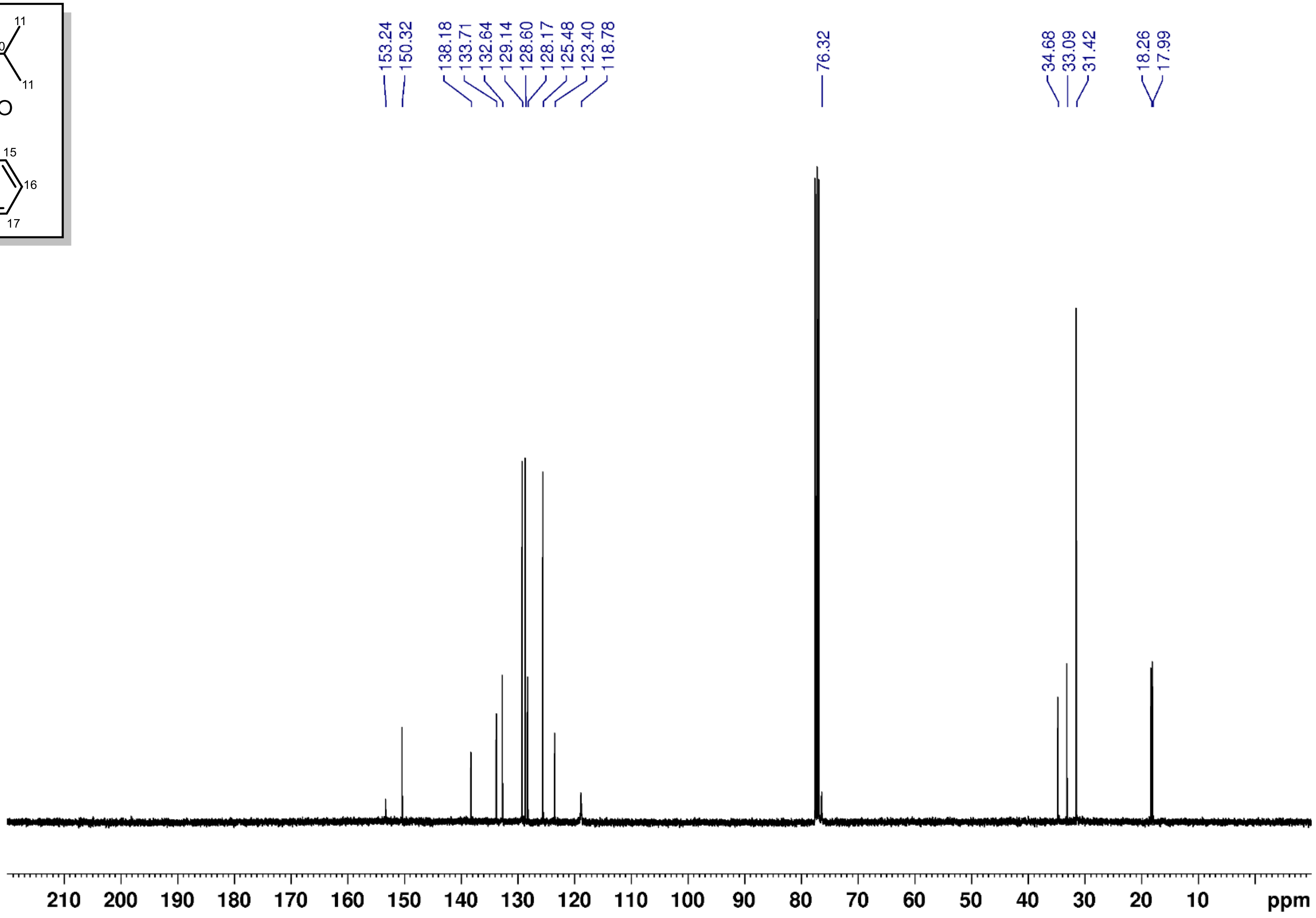

^{1}H NMR (400 MHz, CDCl$_3$) for 2-phenylallyl phenylcarbamate

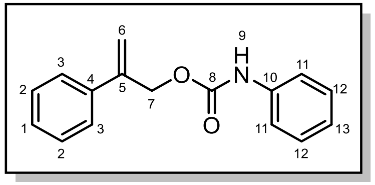

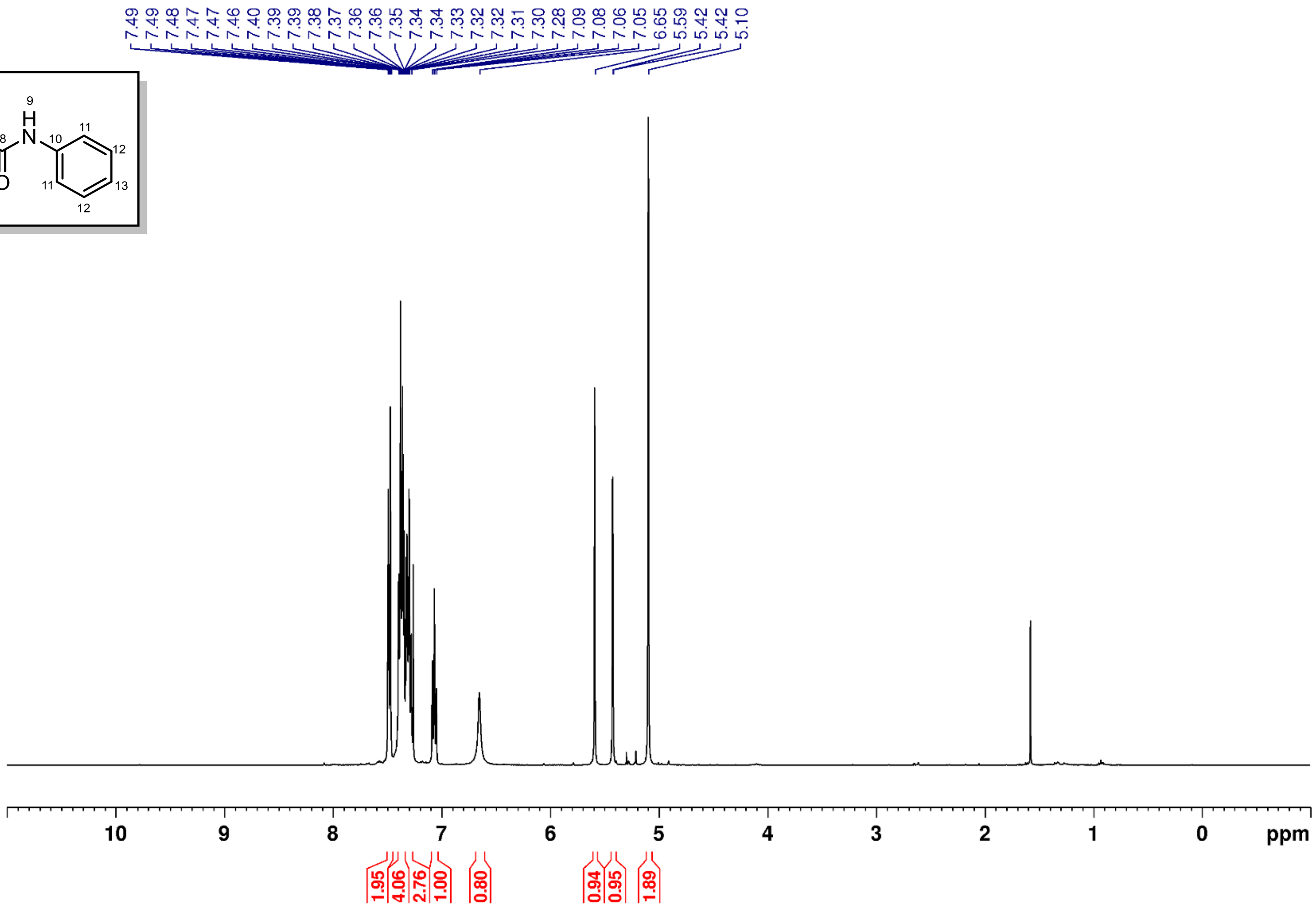

^{13}C NMR (101 MHz, CDCl$_3$) for 2-phenylallyl phenylcarbamate

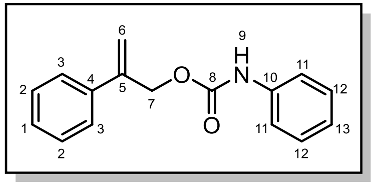

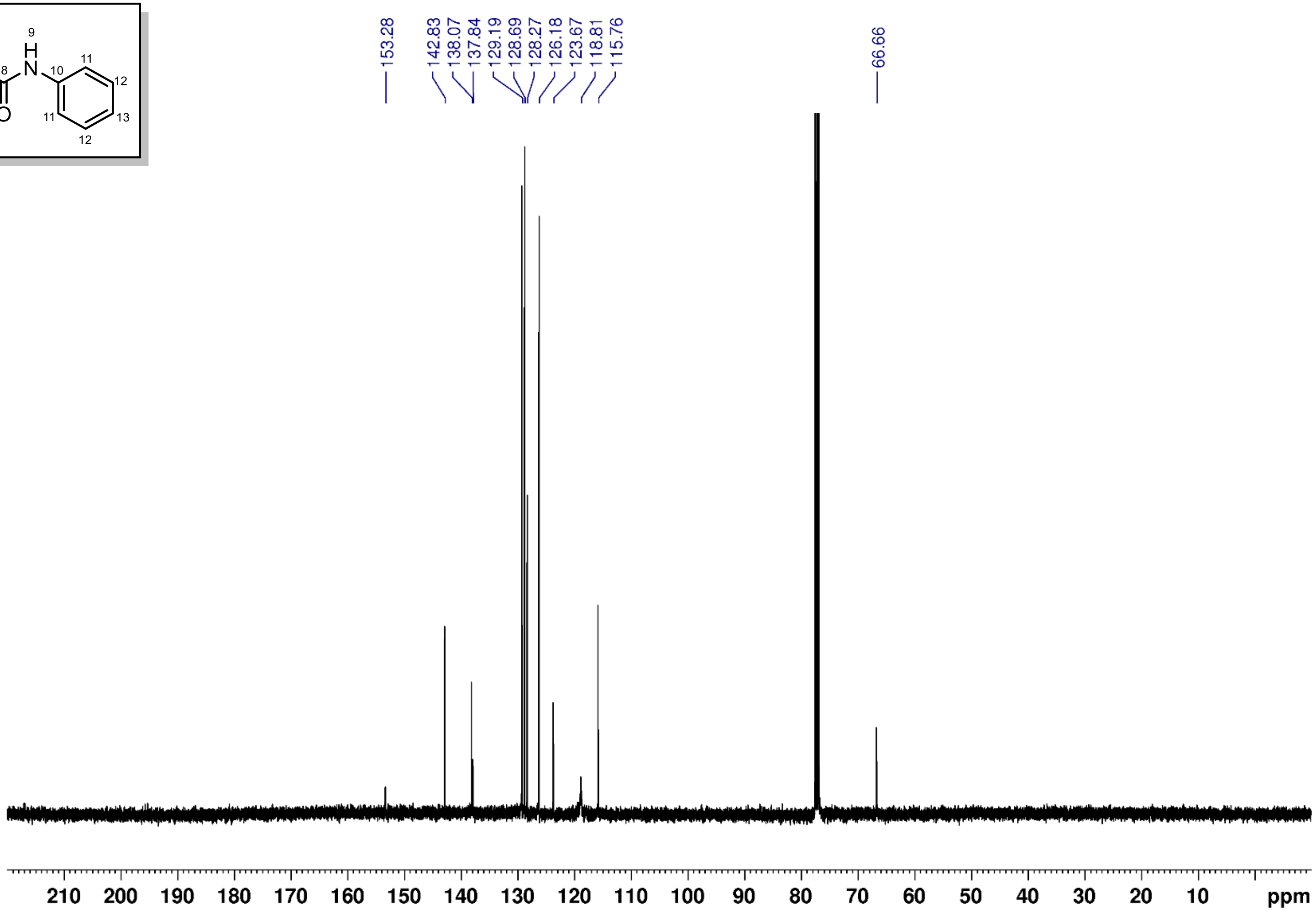

^{1}H NMR (400 MHz, $CDCl_3$) for 3-methylbut-2-en-1-yl phenylcarbamate

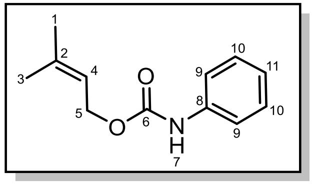

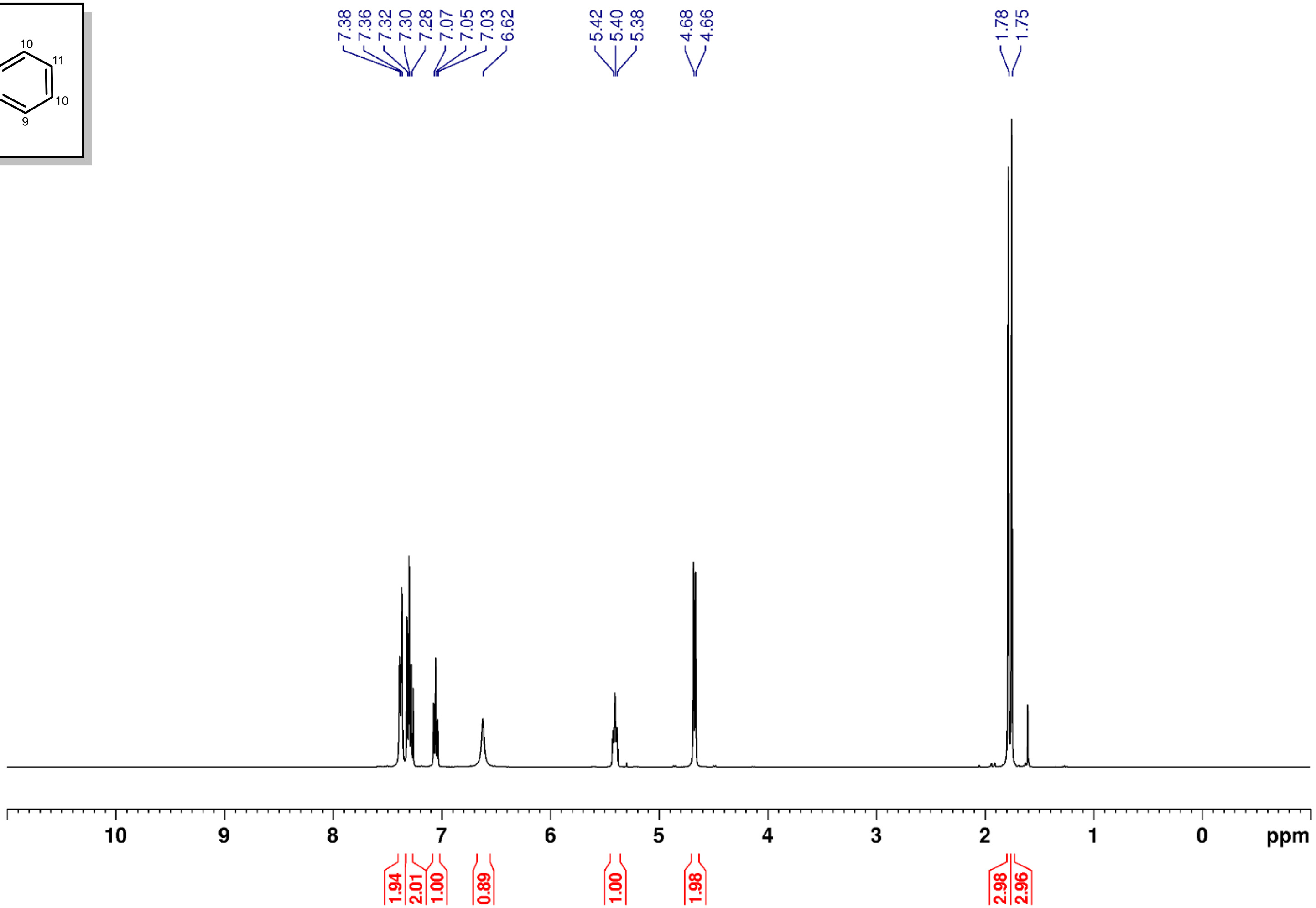

^{13}C NMR (101 MHz, $CDCl_3$) for 3-methylbut-2-en-1-yl phenylcarbamate

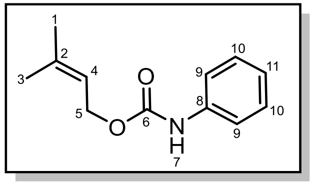

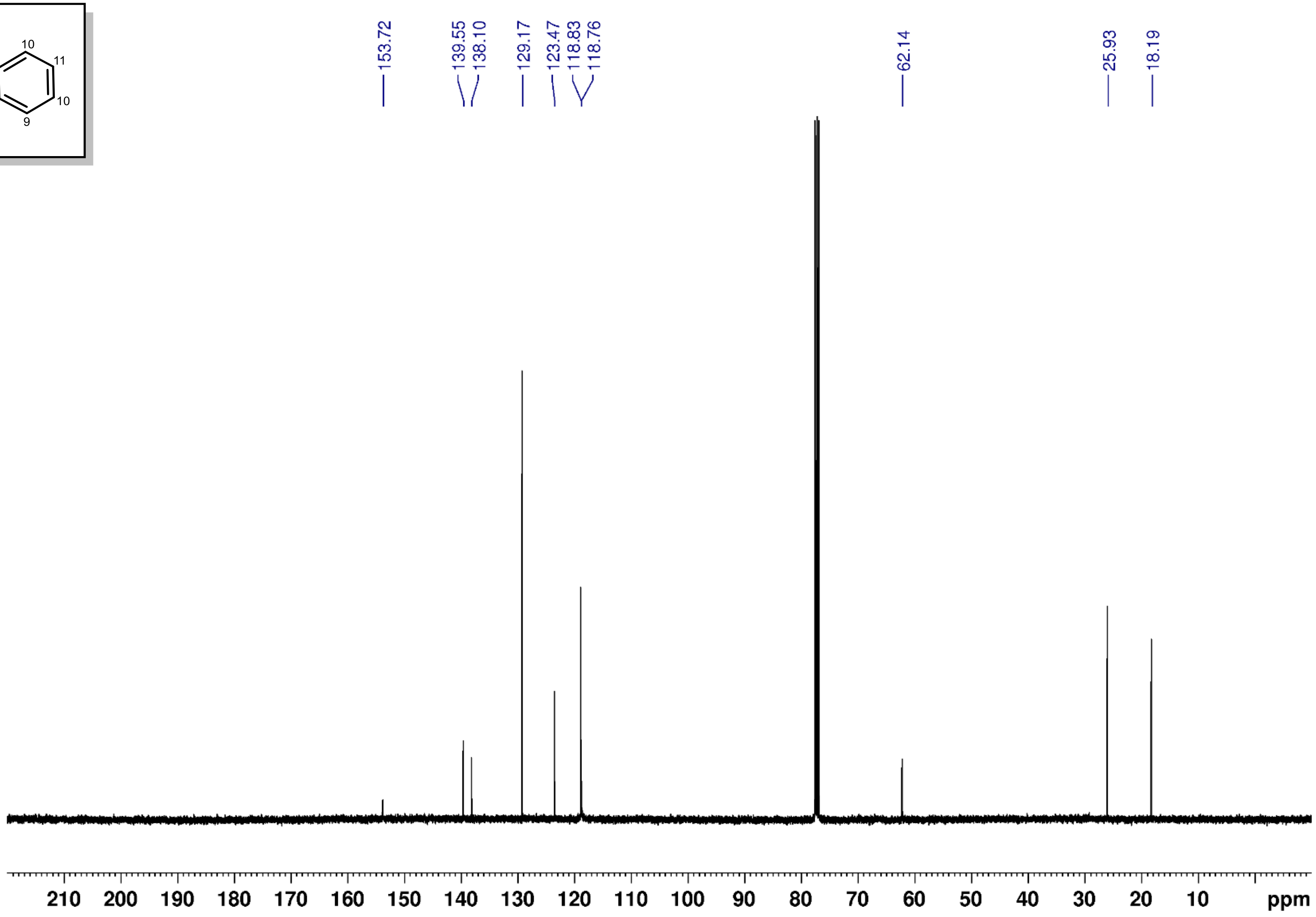

^{1}H NMR (400 MHz, CDCl$_3$) for (*Z*)-hex-2-en-1-yl phenylcarbamate

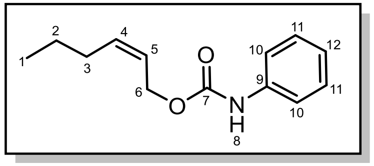

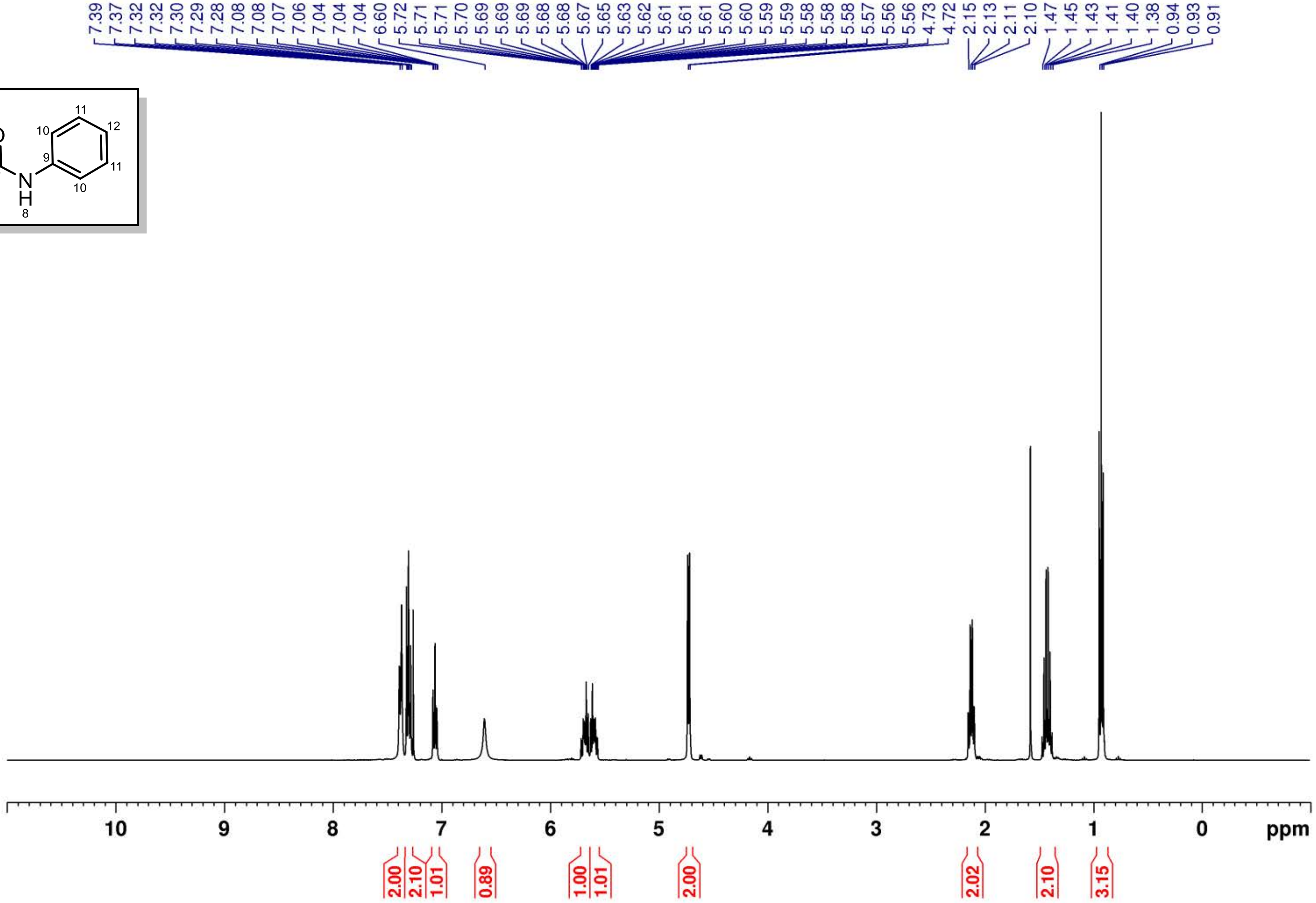

^{13}C NMR (101 MHz, $CDCl_3$) for (*Z*)-hex-2-en-1-yl phenylcarbamate

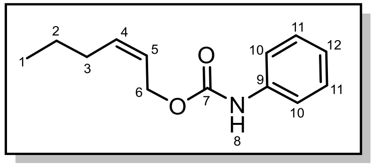

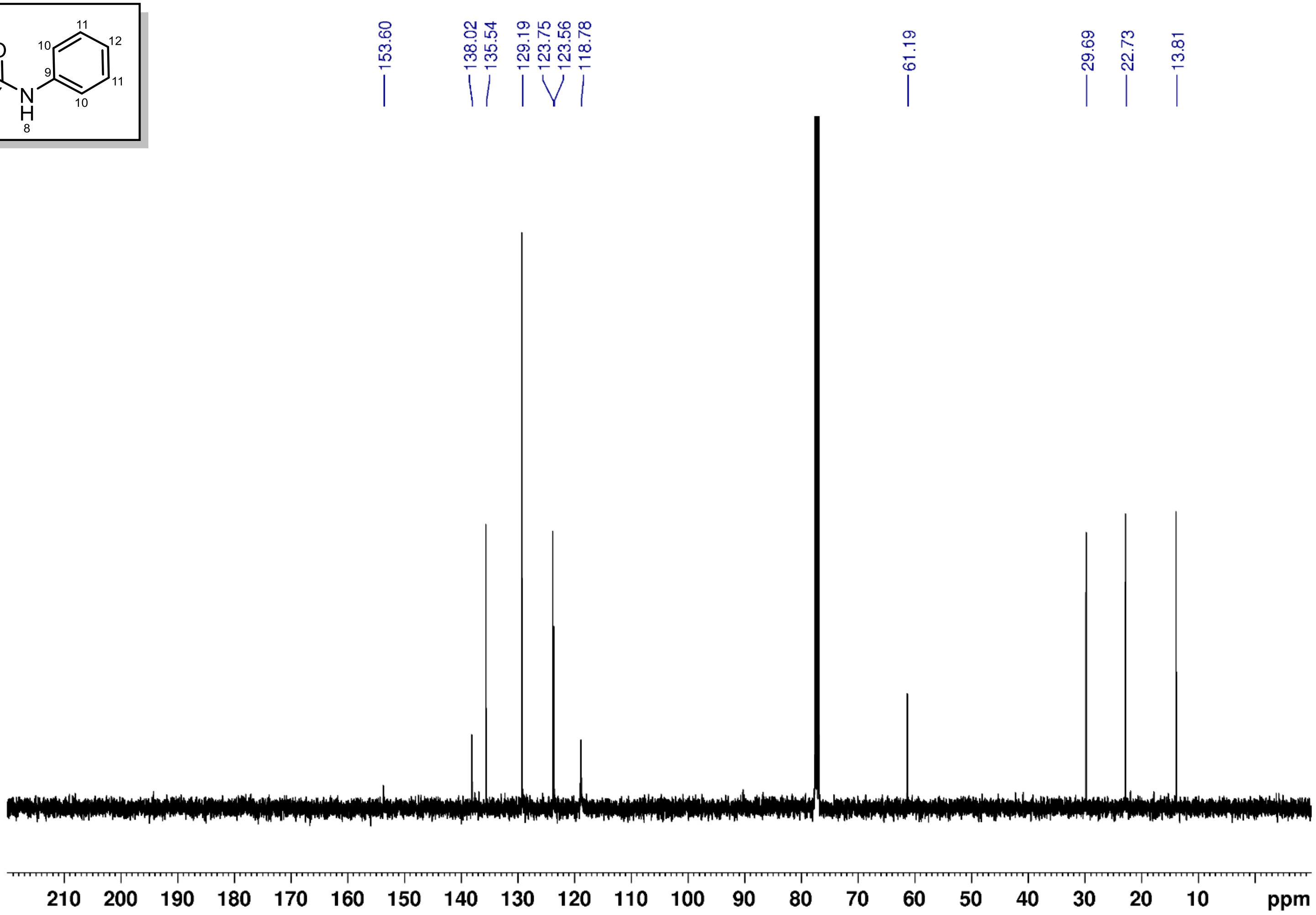

^{1}H NMR (400 MHz, CDCl$_3$) for cinnamyl phenylcarbamate

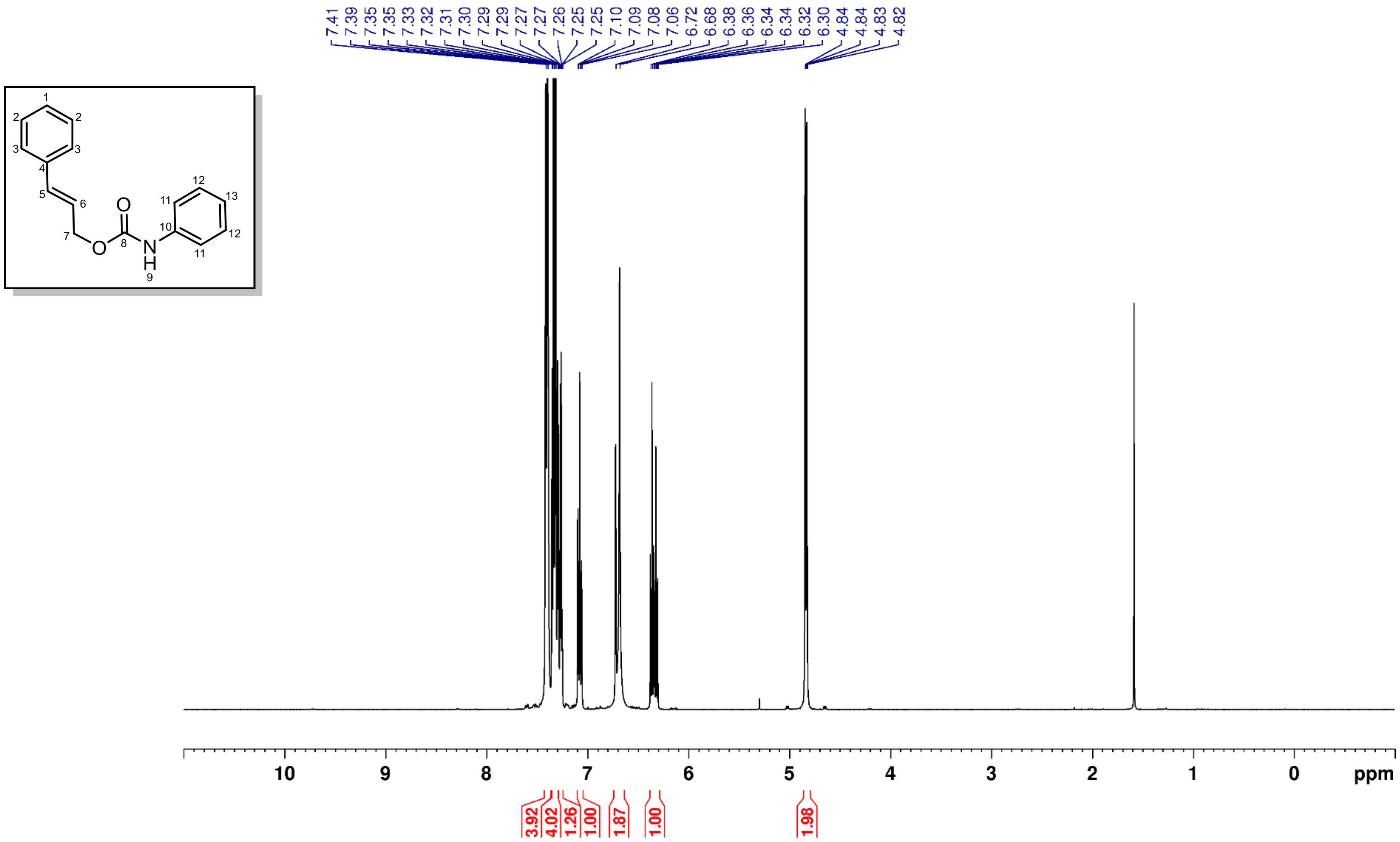

^{13}C NMR (101 MHz, CDCl$_3$) for cinnamyl phenylcarbamate

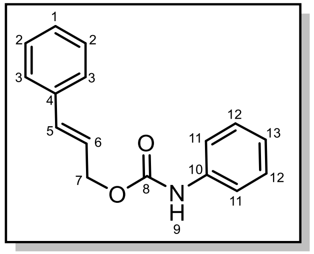

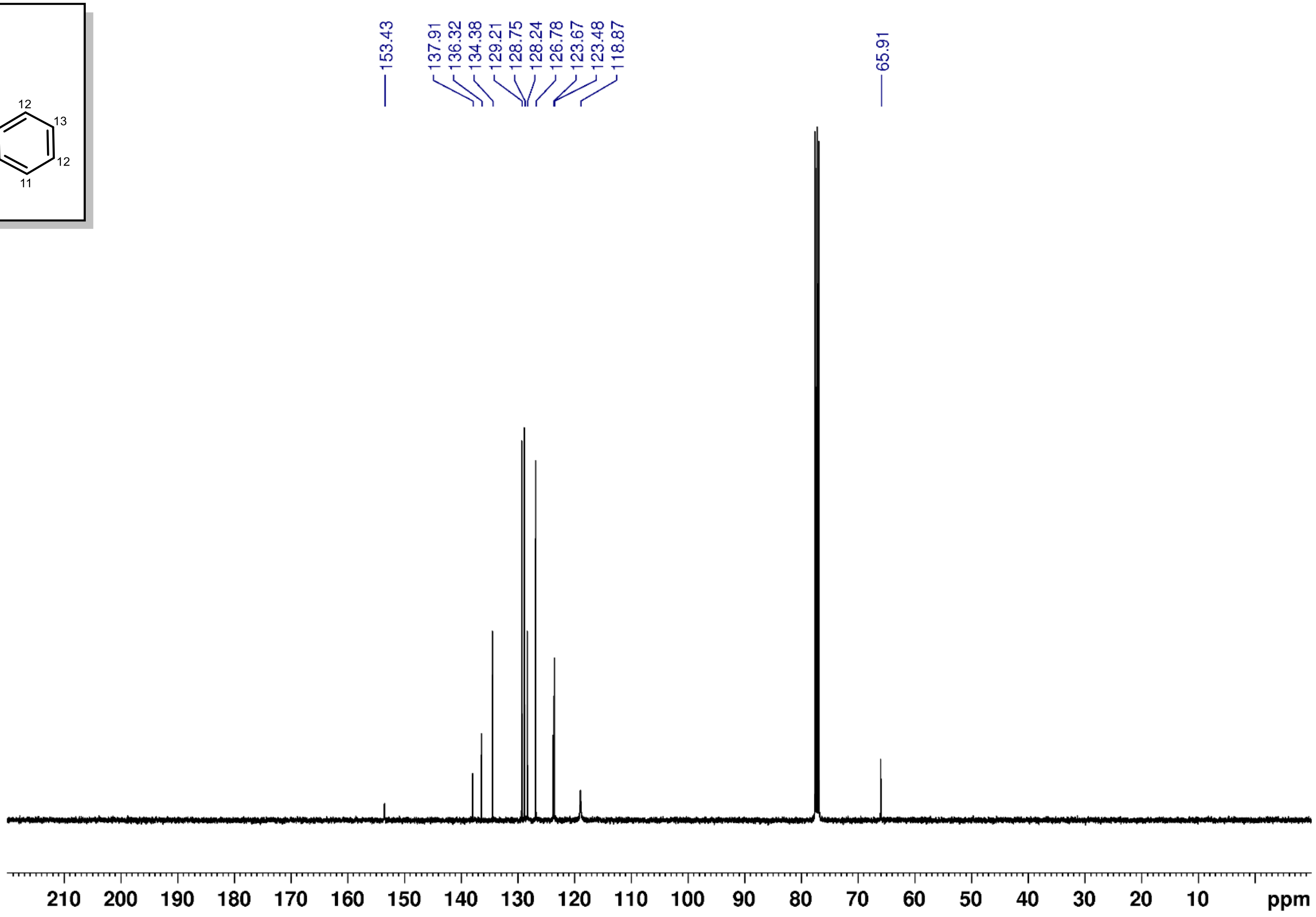

NMR Spectra for Substrates – N-Me Allylic Carbamates:

^{1}H NMR (400 MHz, CDCl$_3$) for (*Z*)-3-phenylallyl methyl(phenyl)carbamate (**1a**)

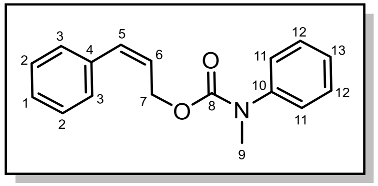

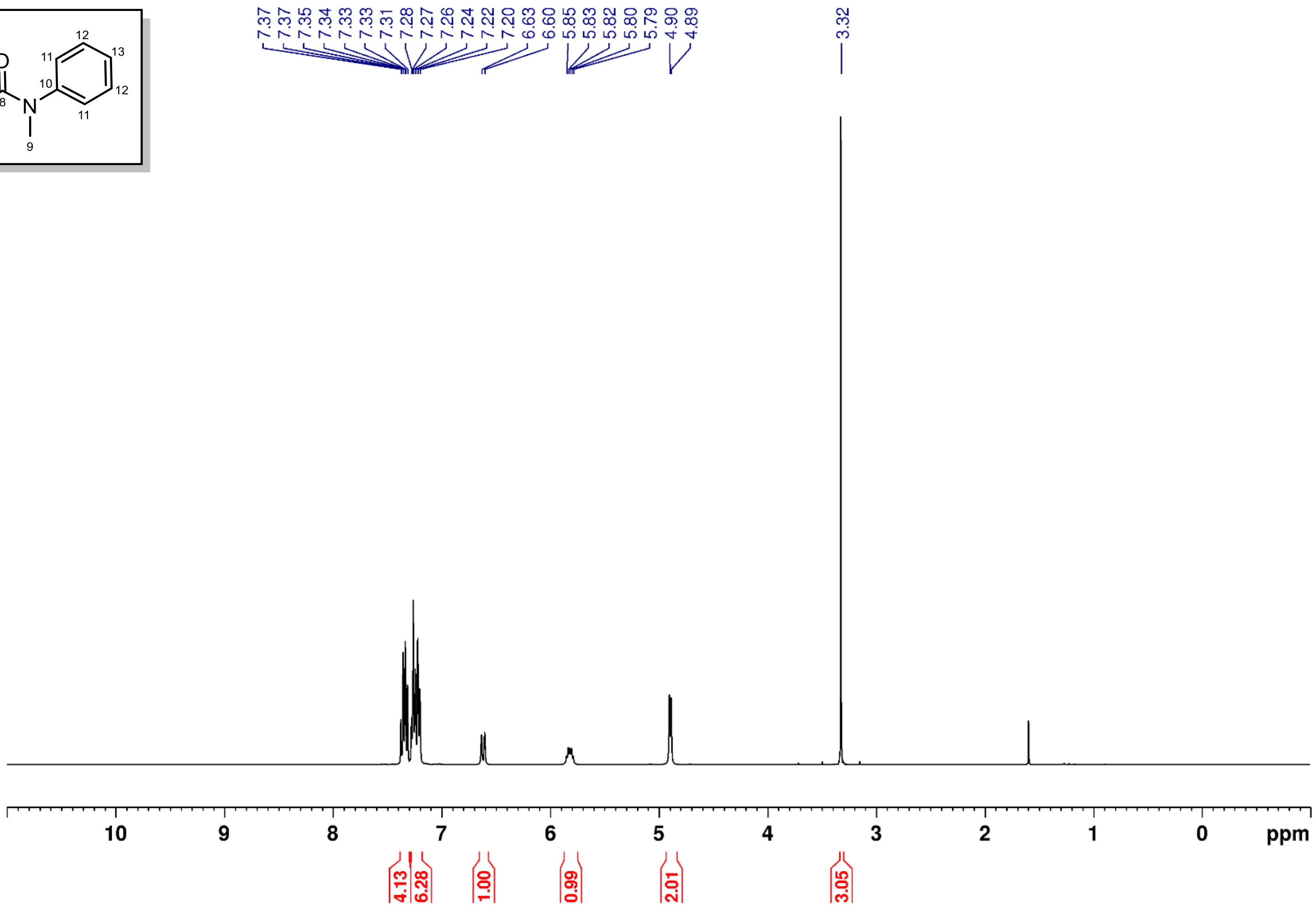

^{13}C NMR (101 MHz, $CDCl_3$) for (*Z*)-3-phenylallyl methyl(phenyl)carbamate (**1a**)

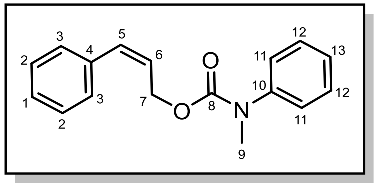

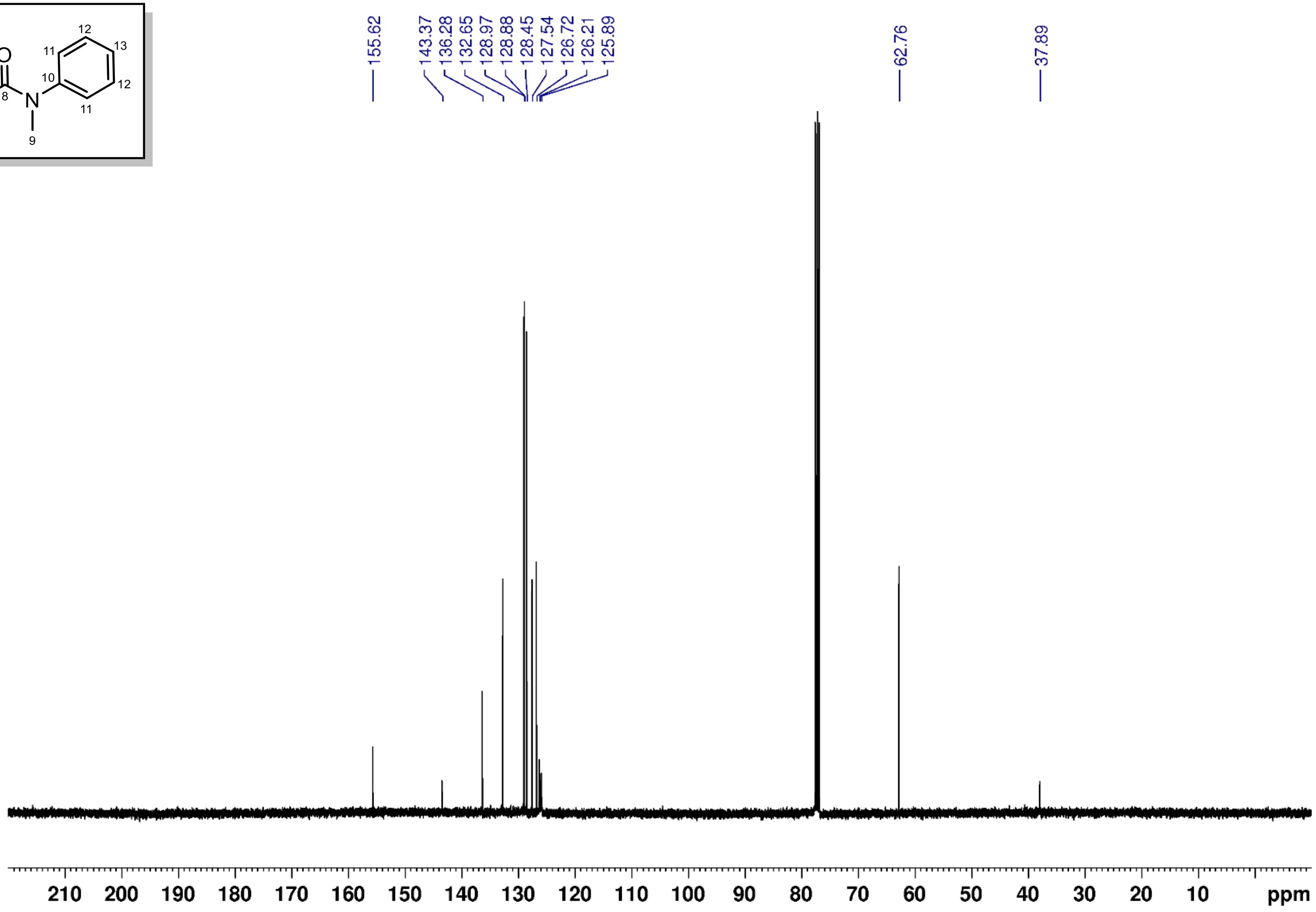

^{1}H NMR (400 MHz, $CDCl_3$) for (*Z*)-3-(2-isopropylphenyl)allyl methyl(phenyl)carbamate (**1b**)

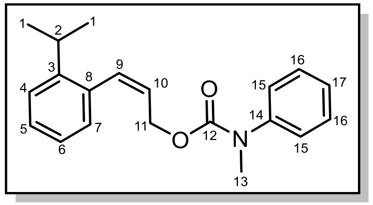

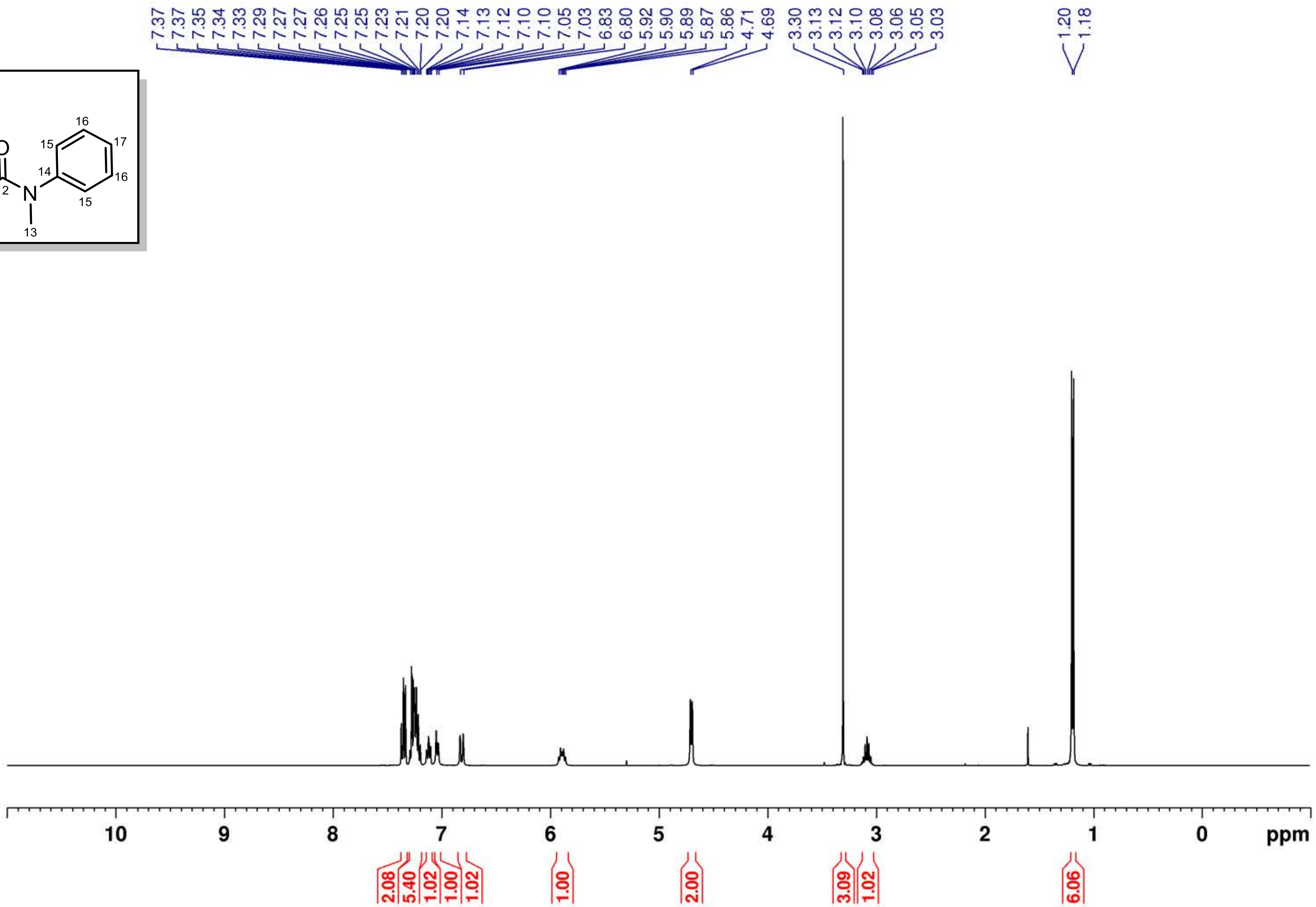

^{13}C NMR (101 MHz, CDCl$_3$) for (*Z*)-3-(2-isopropylphenyl)allyl methyl(phenyl)carbamate (**1b**)

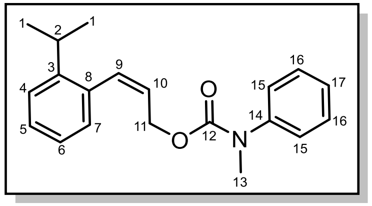

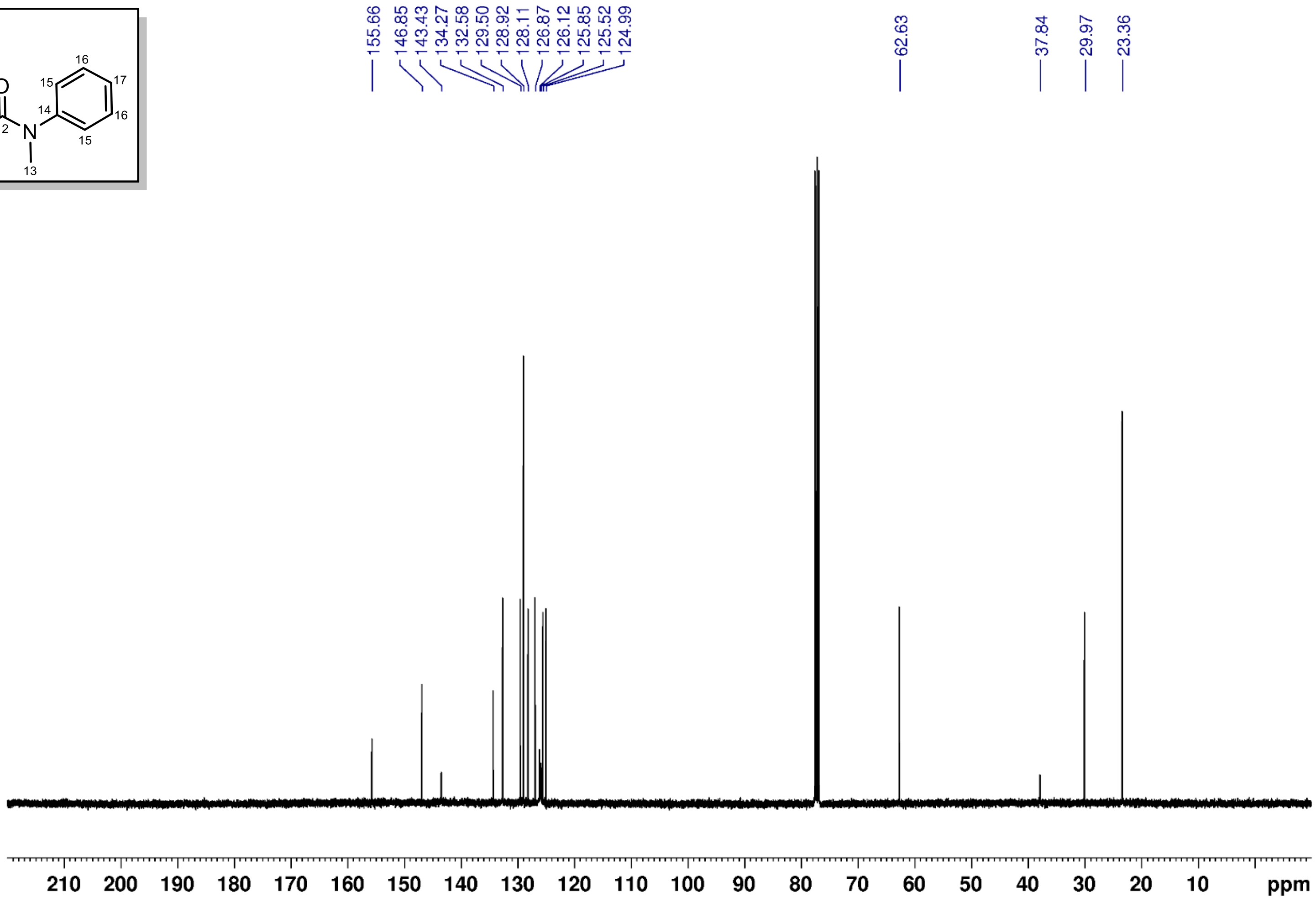

^{1}H NMR (400 MHz, $CDCl_3$) for (*Z*)-3-(2-methoxyphenyl)allyl methyl(phenyl)carbamate (**1c**)

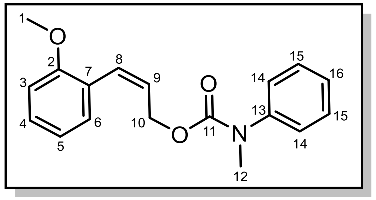

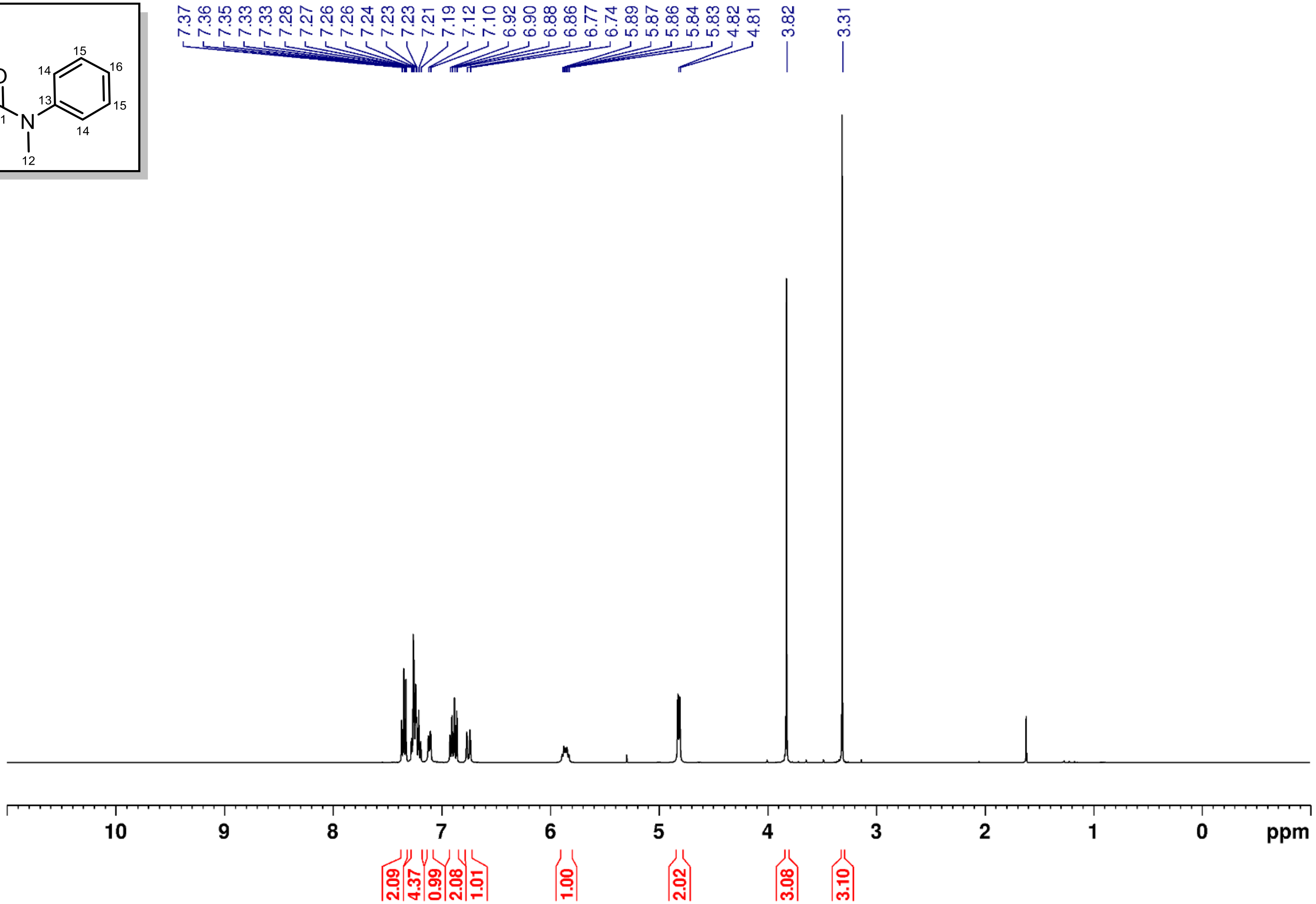

^{13}C NMR (101 MHz, CDCl$_3$) for (*Z*)-3-(2-methoxyphenyl)allyl methyl(phenyl)carbamate (**1c**)

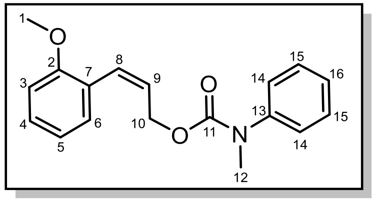

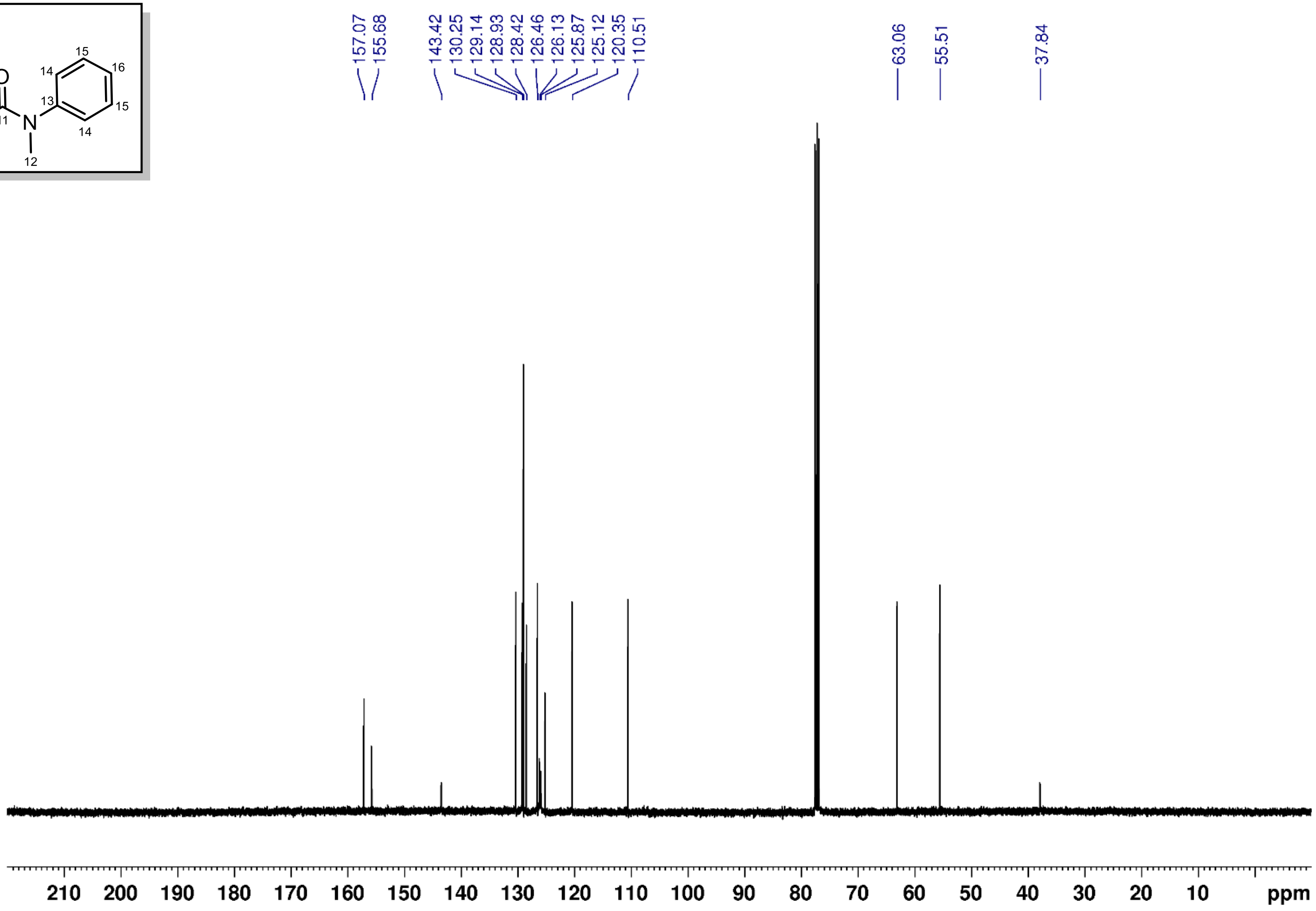

^{1}H NMR (400 MHz, CDCl$_3$) for (*Z*)-3-(2-fluorophenyl)allyl methyl(phenyl)carbamate (**1d**)

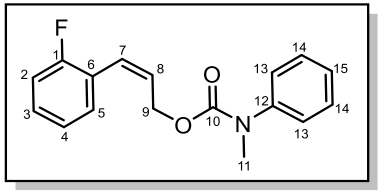

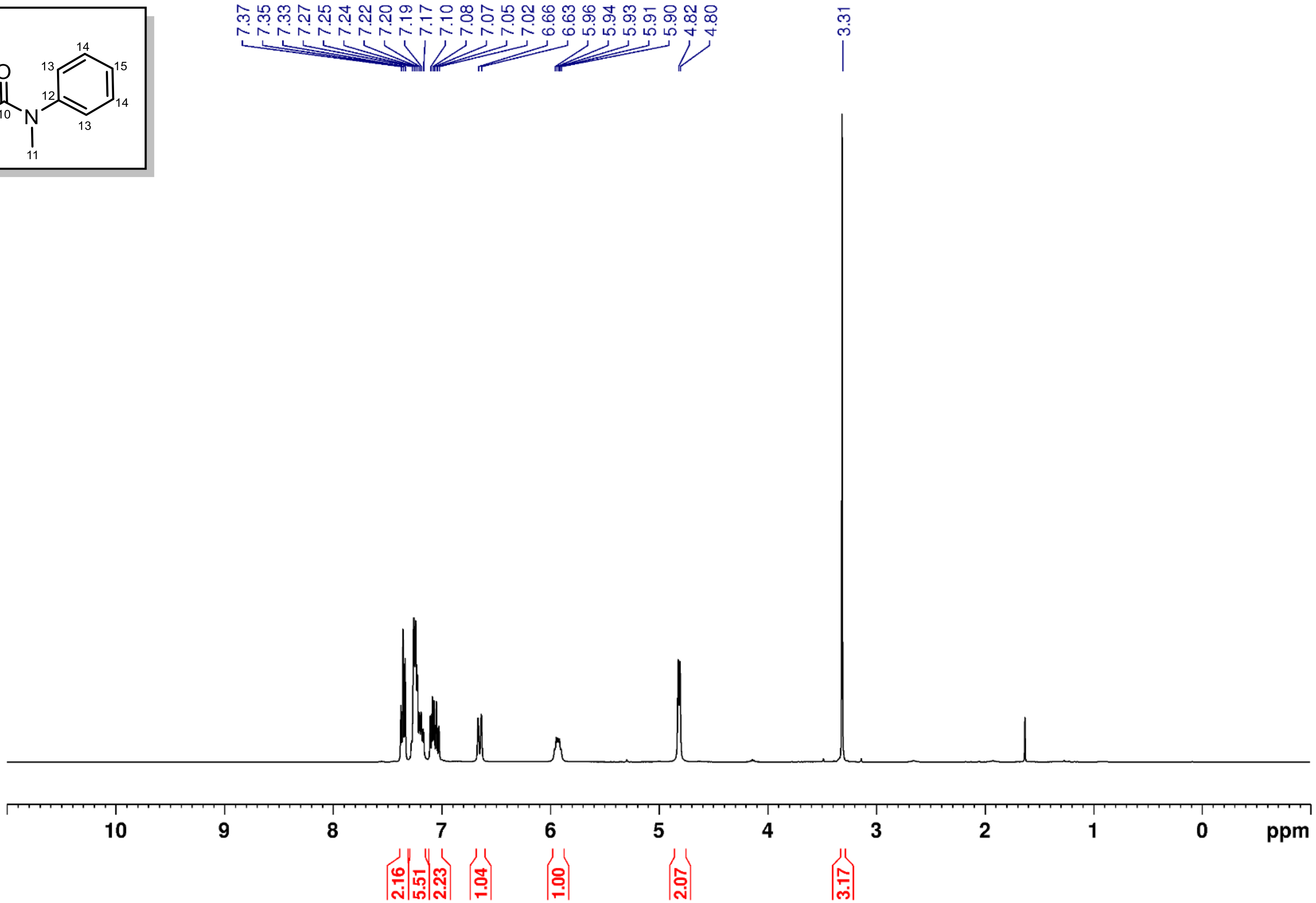

^{13}C NMR (101 MHz, CDCl$_3$) for (*Z*)-3-(2-fluorophenyl)allyl methyl(phenyl)carbamate (**1d**)

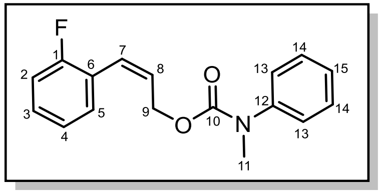

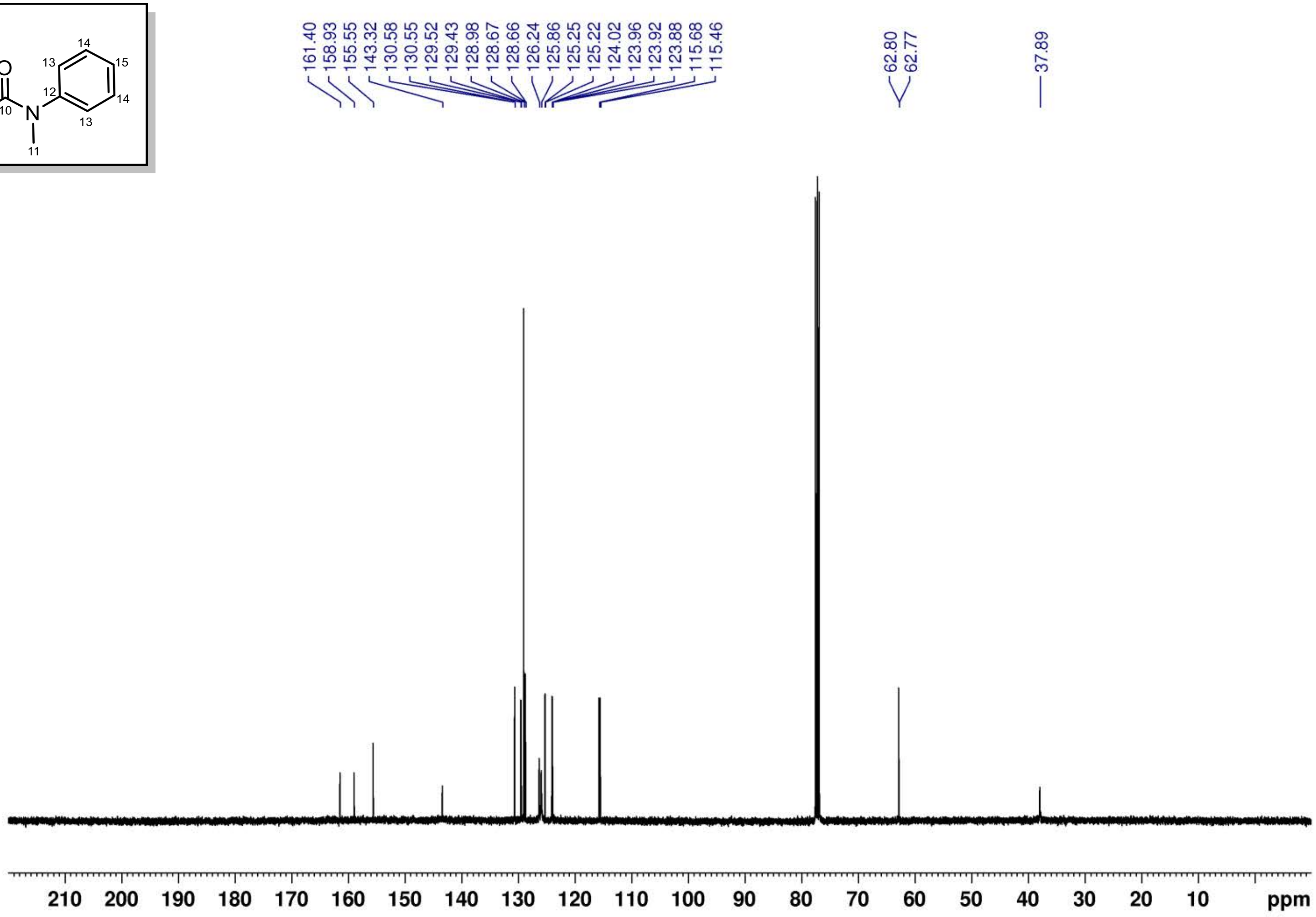

^{19}F NMR (376 MHz, $CDCl_3$) for (*Z*)-3-(2-fluorophenyl)allyl methyl(phenyl)carbamate (**1d**)

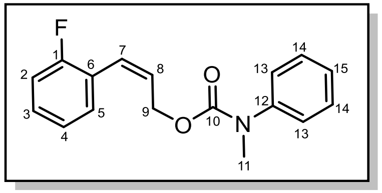

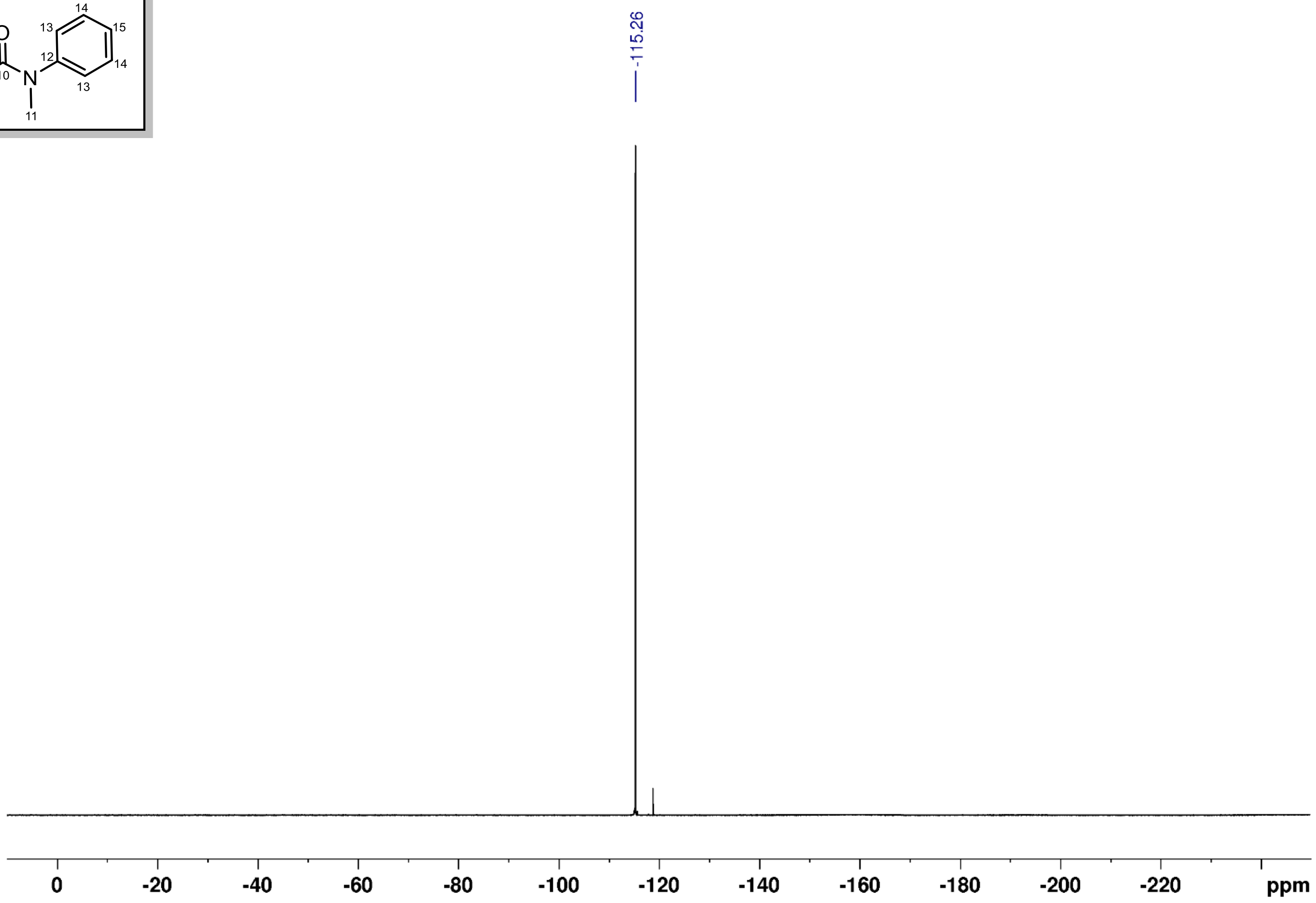

^{1}H NMR (400 MHz, $CDCl_3$) for prop-2-yn-1-yl methyl(phenyl)carbamate

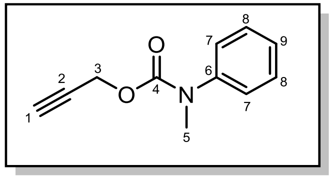

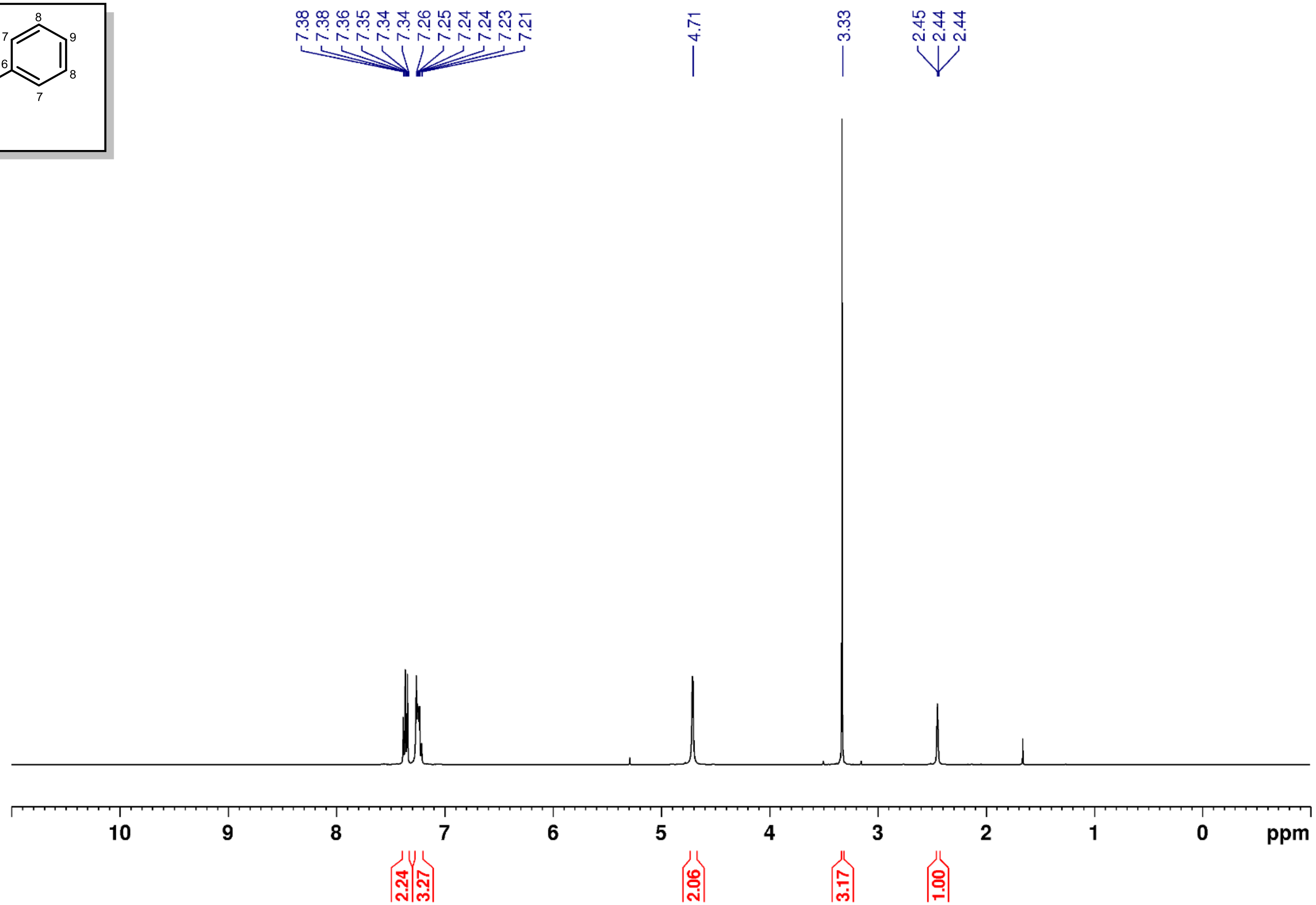

^{13}C NMR (101 MHz, $CDCl_3$) for prop-2-yn-1-yl methyl(phenyl)carbamate

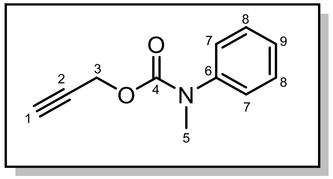

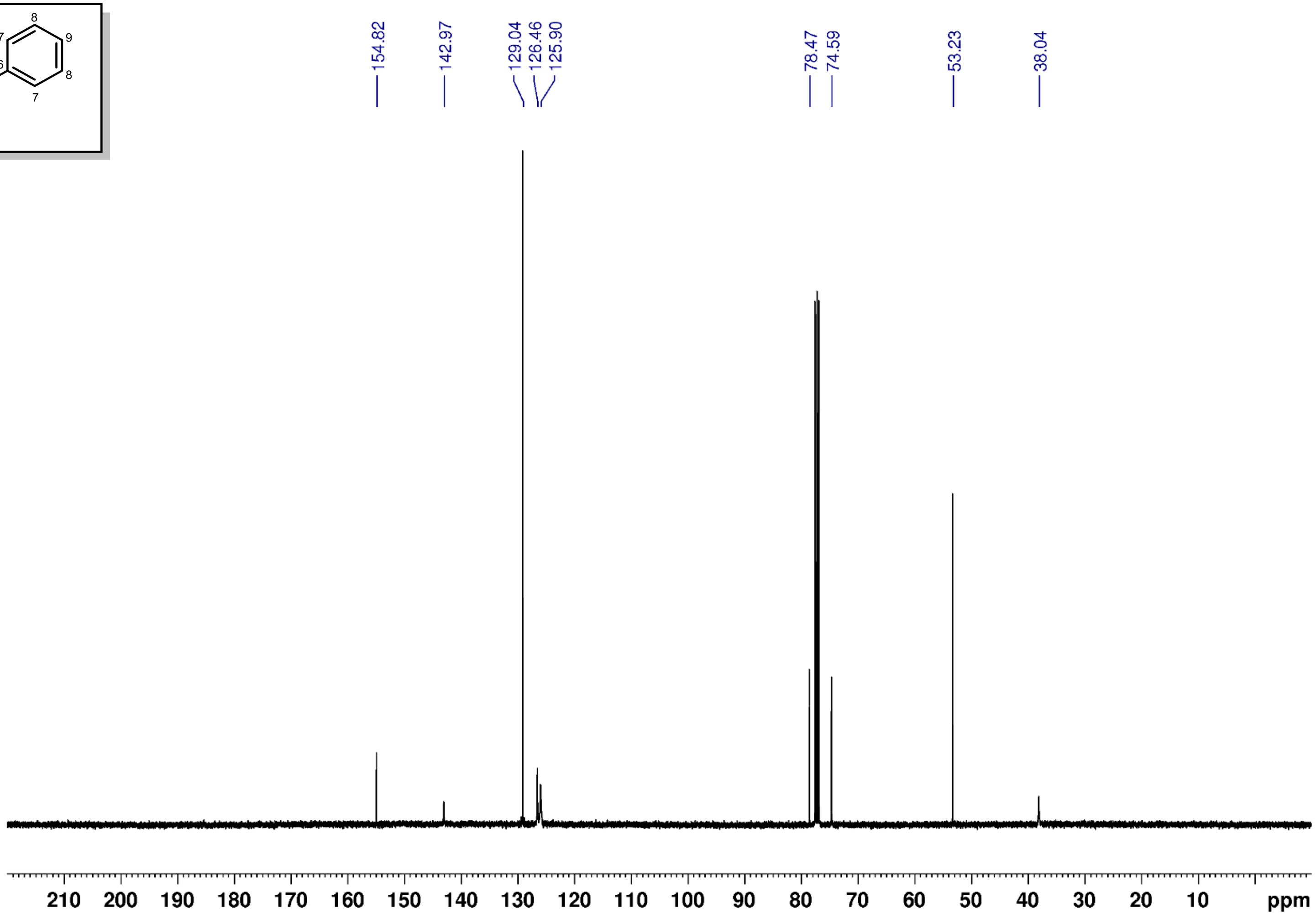

^{1}H NMR (400 MHz, $CDCl_3$) for 3-(*o*-tolyl)prop-2-yn-1-yl methyl(phenyl)carbamate

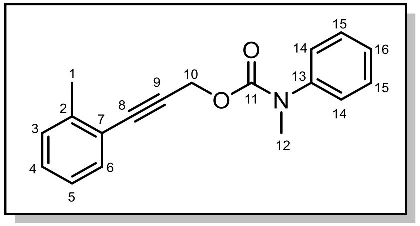

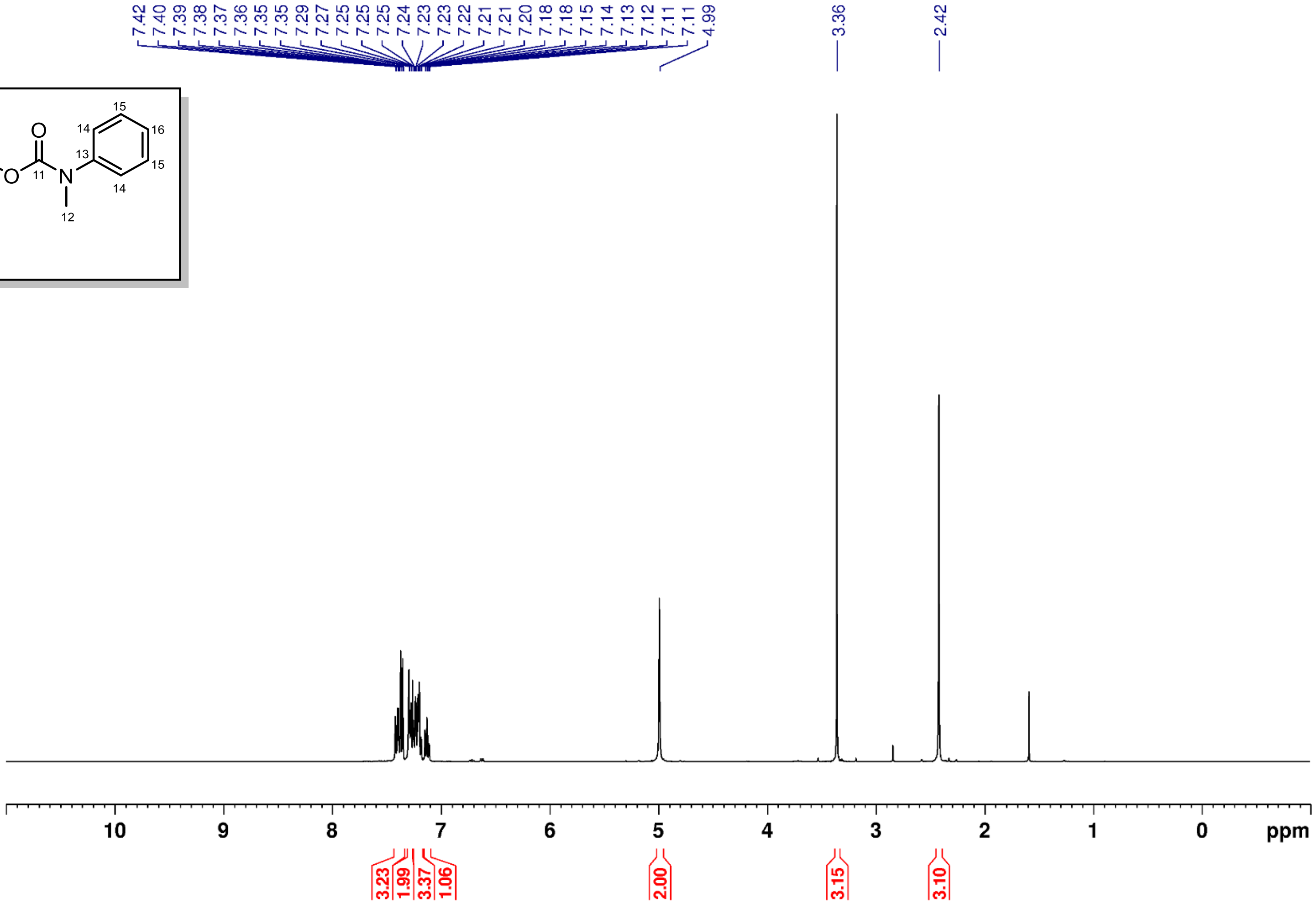

^{13}C NMR (101 MHz, $CDCl_3$) for 3-(*o*-tolyl)prop-2-yn-1-yl methyl(phenyl)carbamate

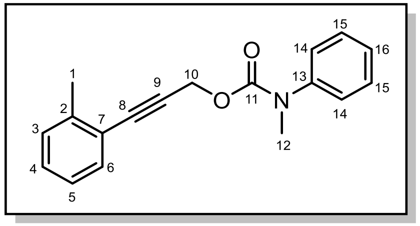

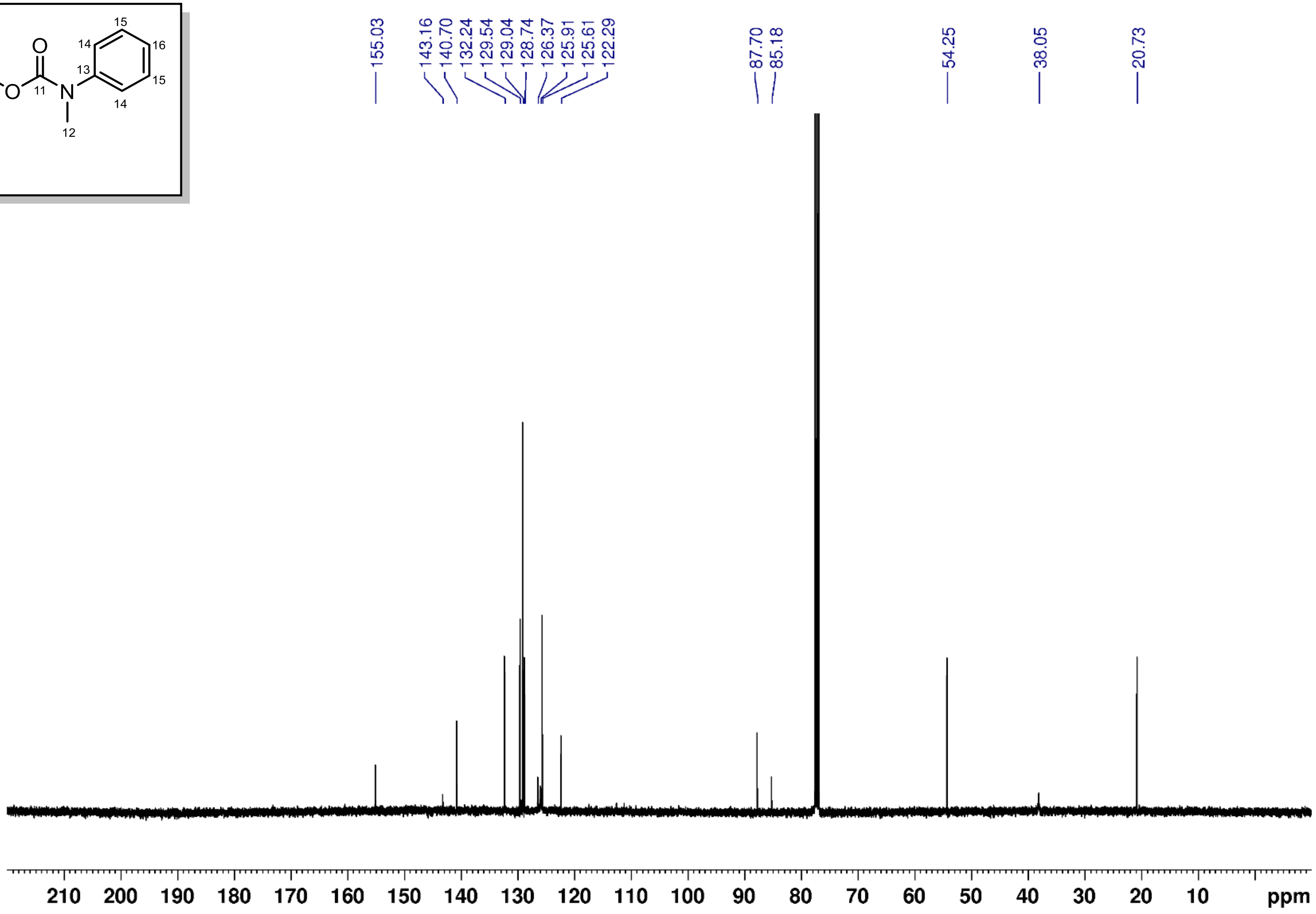

^{1}H NMR (400 MHz, $CDCl_3$) for (*Z*)-3-(*o*-tolyl)allyl methyl(phenyl)carbamate (**1e**)

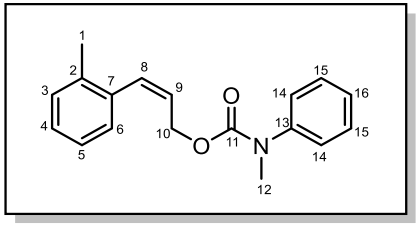

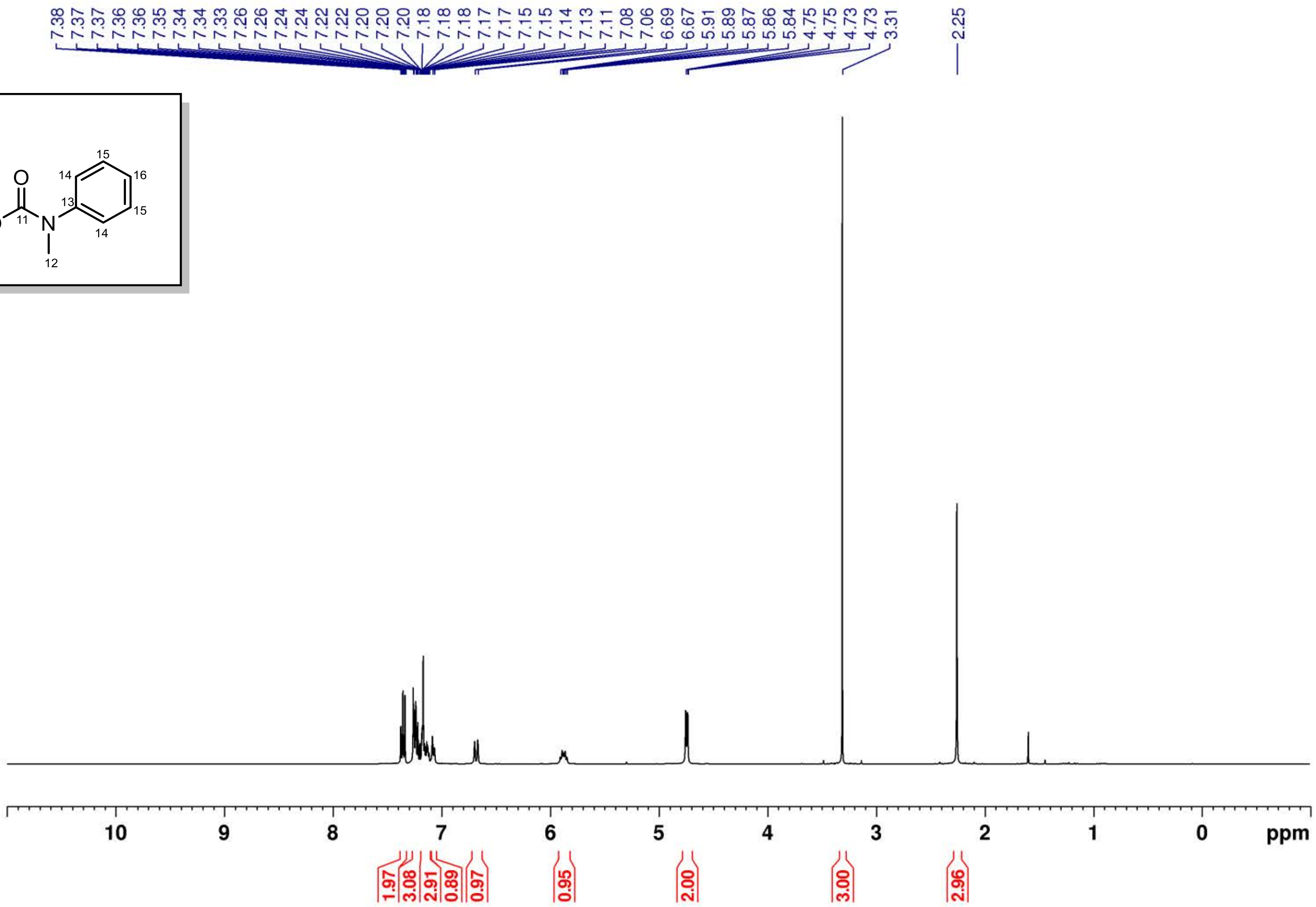

^{13}C NMR (101 MHz, $CDCl_3$) for (*Z*)-3-(*o*-tolyl)allyl methyl(phenyl)carbamate (**1e**)

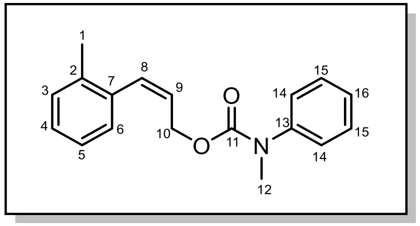

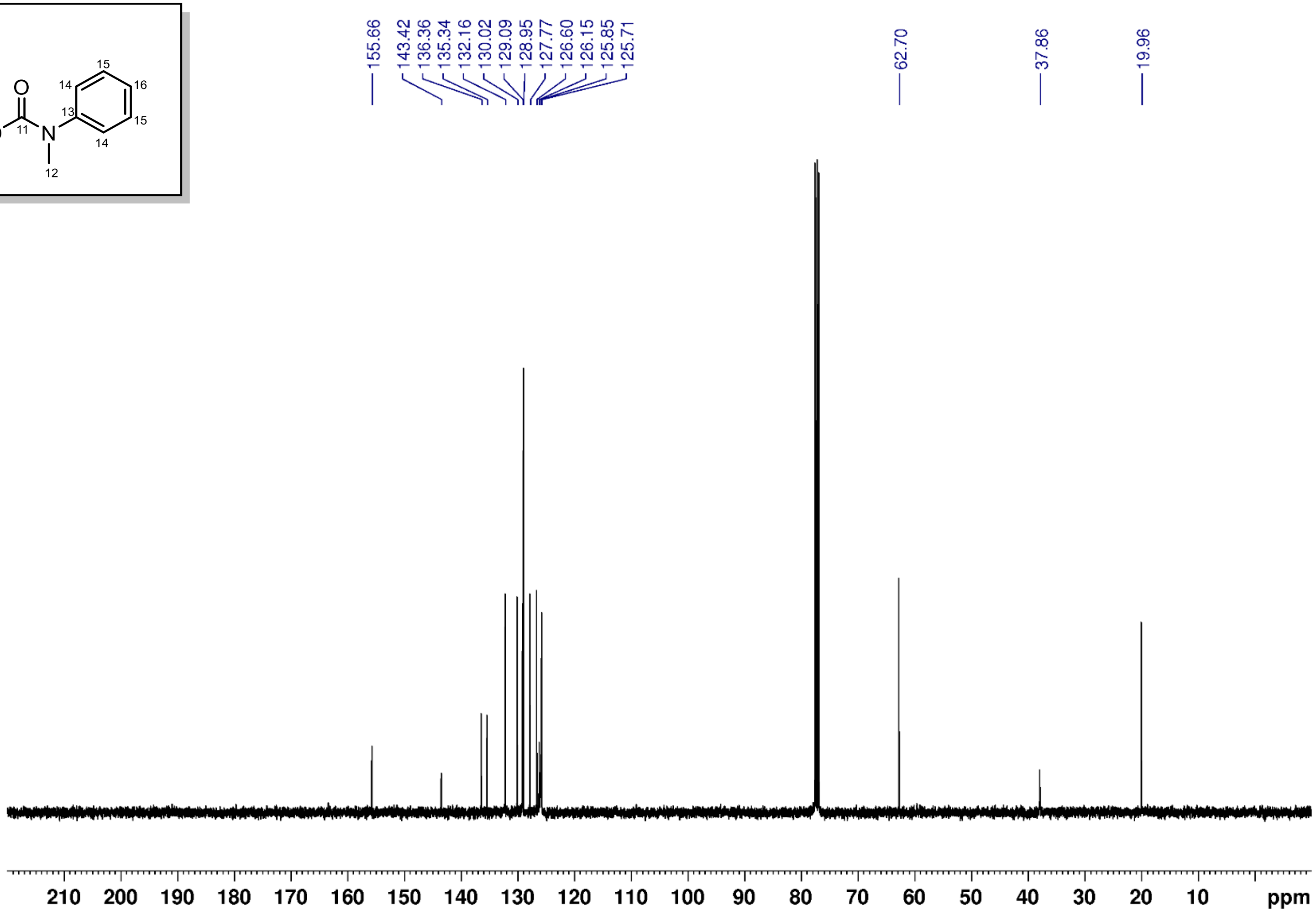

^{1}H NMR (700 MHz, CDCl$_3$) for *tert*-butyl (*Z*)-methyl(3-(3-((methyl(phenyl)carbamoyl)oxy)prop-1-en-1-yl)phenyl)carbamate (**1f**)

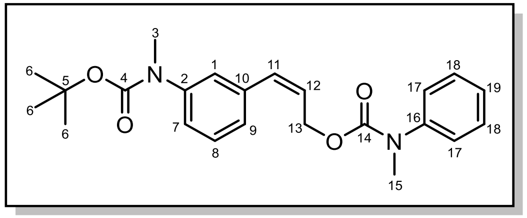

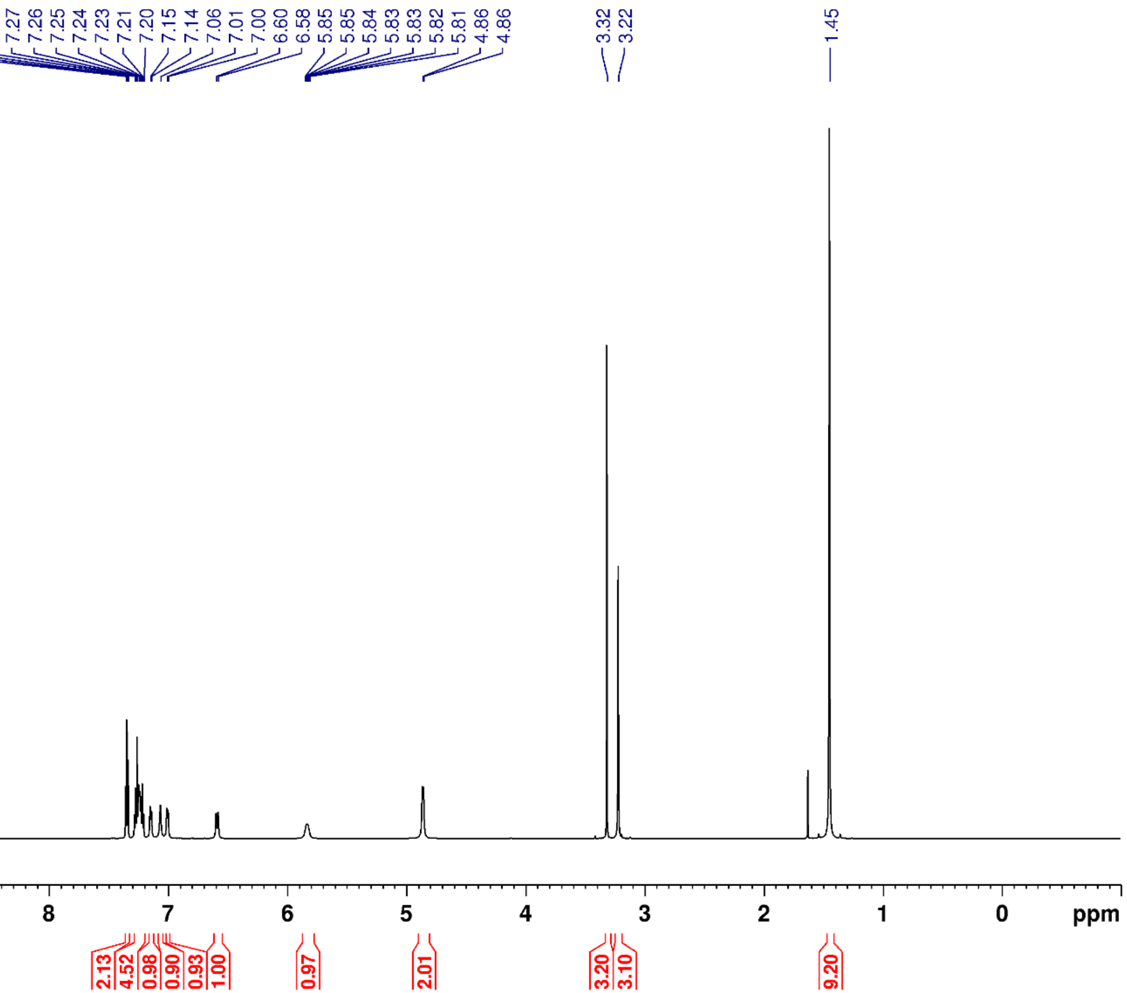

^{13}C NMR (176 MHz, CDCl$_3$) for *tert*-butyl (*Z*)-methyl(3-(3-((methyl(phenyl)carbamoyl)oxy)prop-1-en-1-yl)phenyl)carbamate (**1f**)

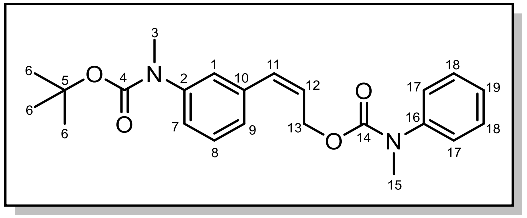

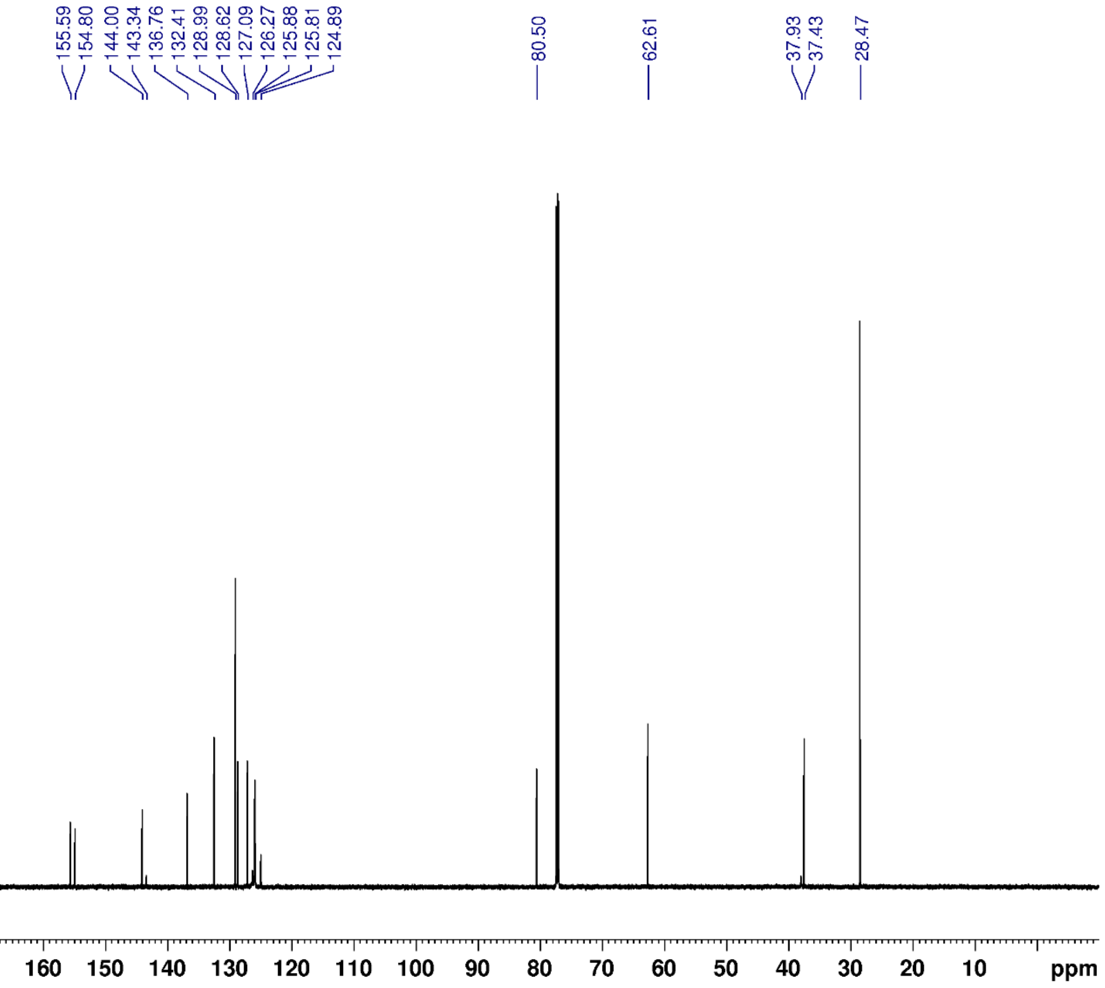

^{1}H NMR (700 MHz, $CDCl_3$) for (*Z*)-3-(3-(*tert*-butyl)phenyl)allyl methyl(phenyl)carbamate (**1g**)

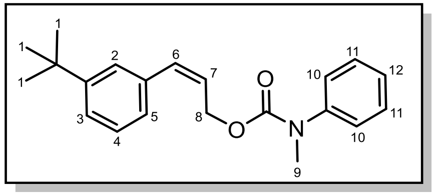

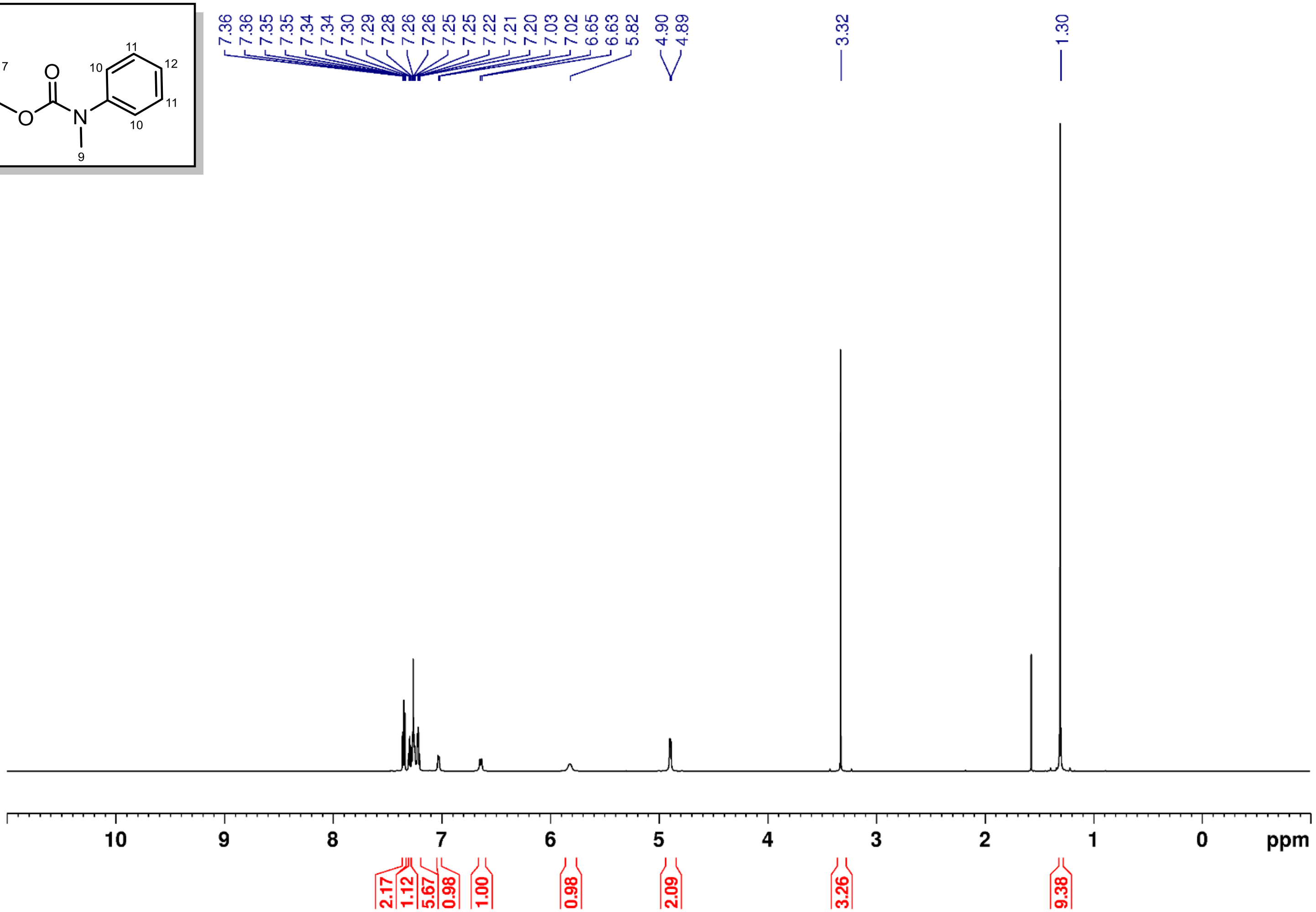

^{13}C NMR (176 MHz, $CDCl_3$) for (*Z*)-3-(3-(*tert*-butyl)phenyl)allyl methyl(phenyl)carbamate (**1g**)

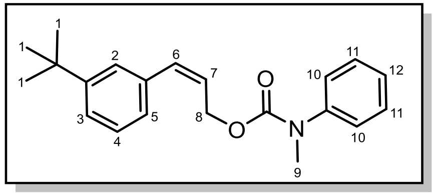

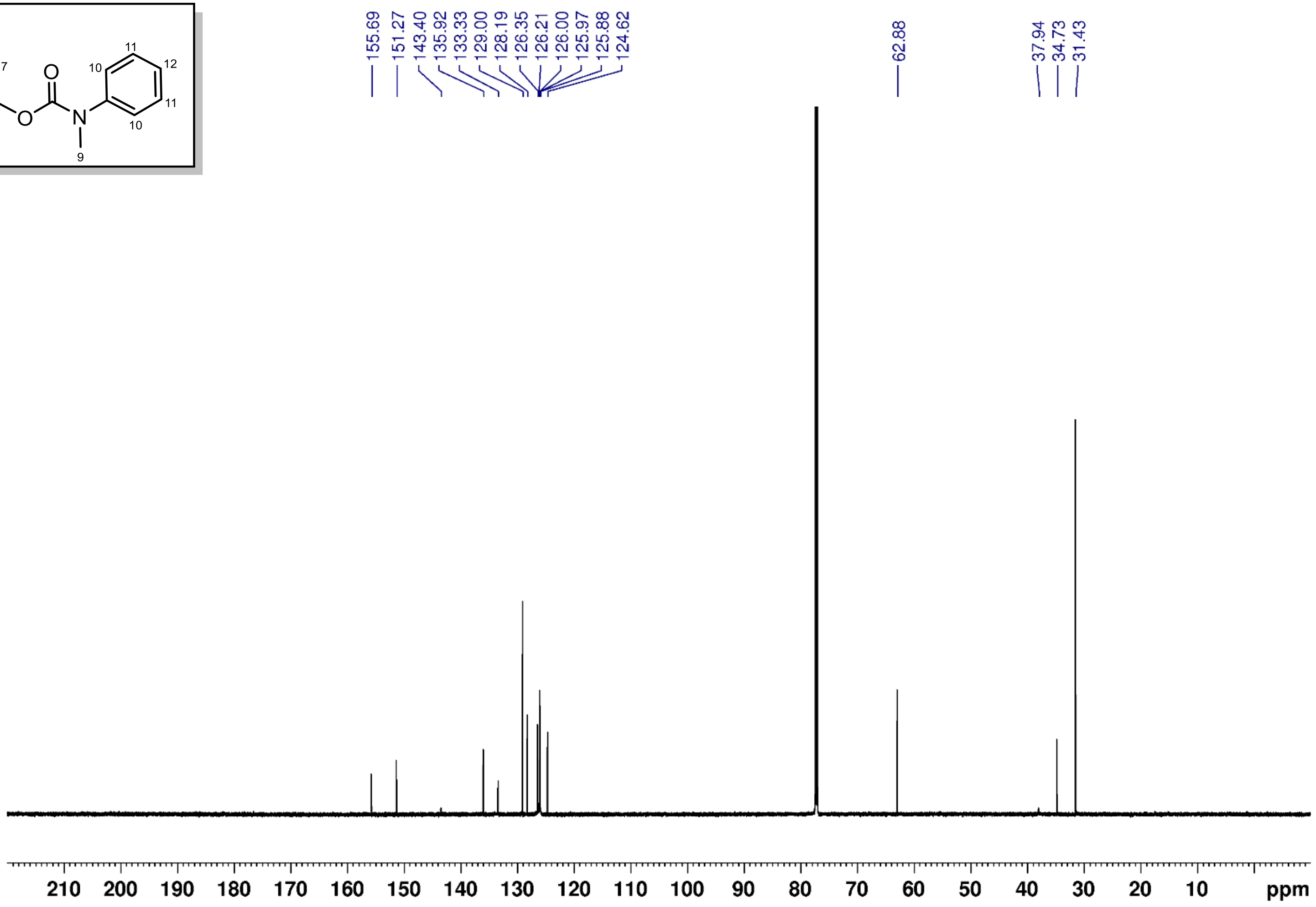

^{1}H NMR (400 MHz, $CDCl_3$) for (*Z*)-3-(3-methoxyphenyl)allyl methyl(phenyl)carbamate (**1h**)

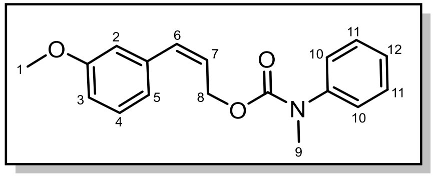

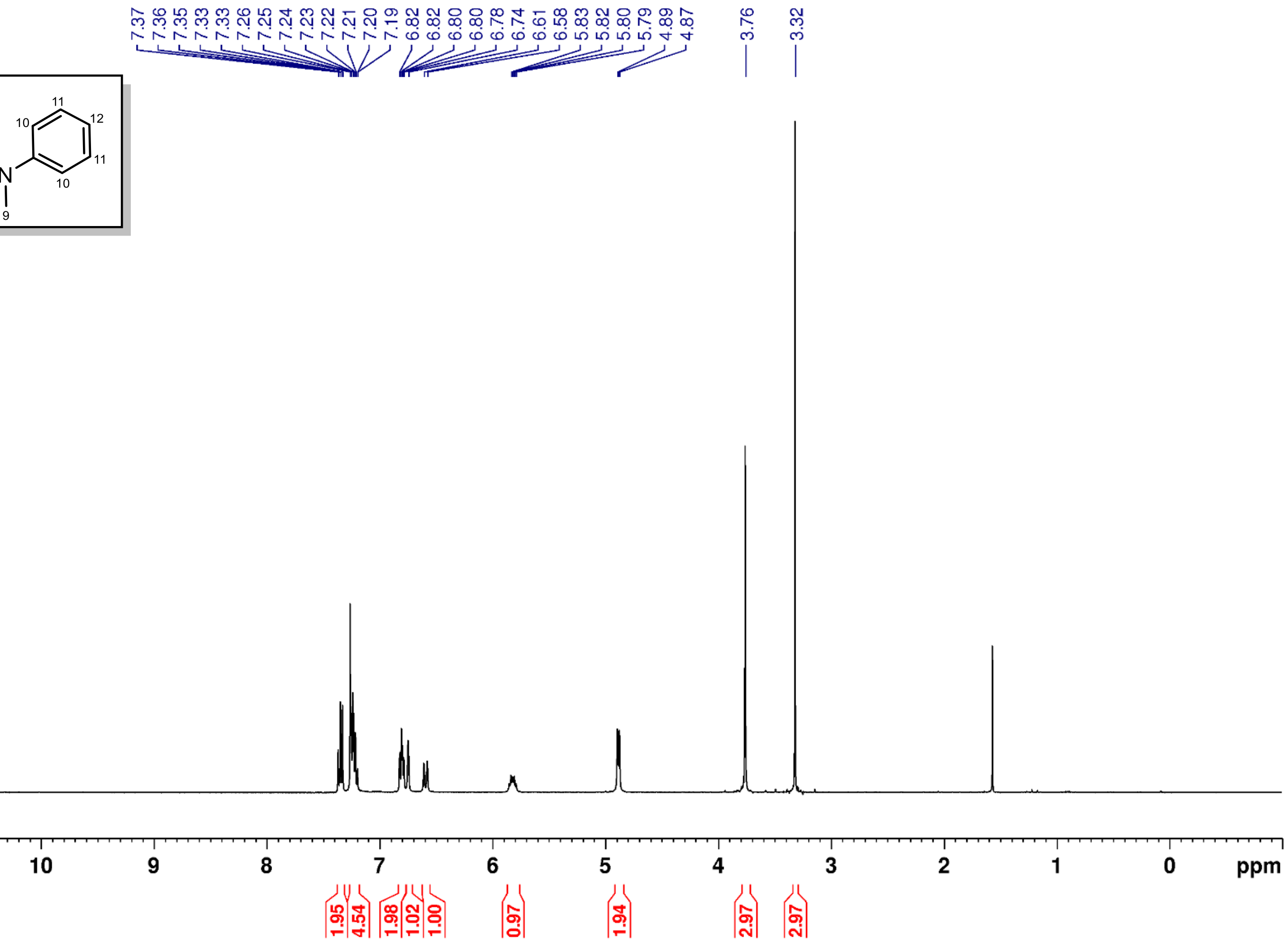

^{13}C NMR (101 MHz, $CDCl_3$) for (*Z*)-3-(3-methoxyphenyl)allyl methyl(phenyl)carbamate (**1h**)

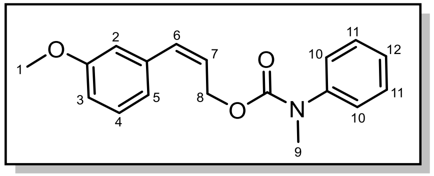

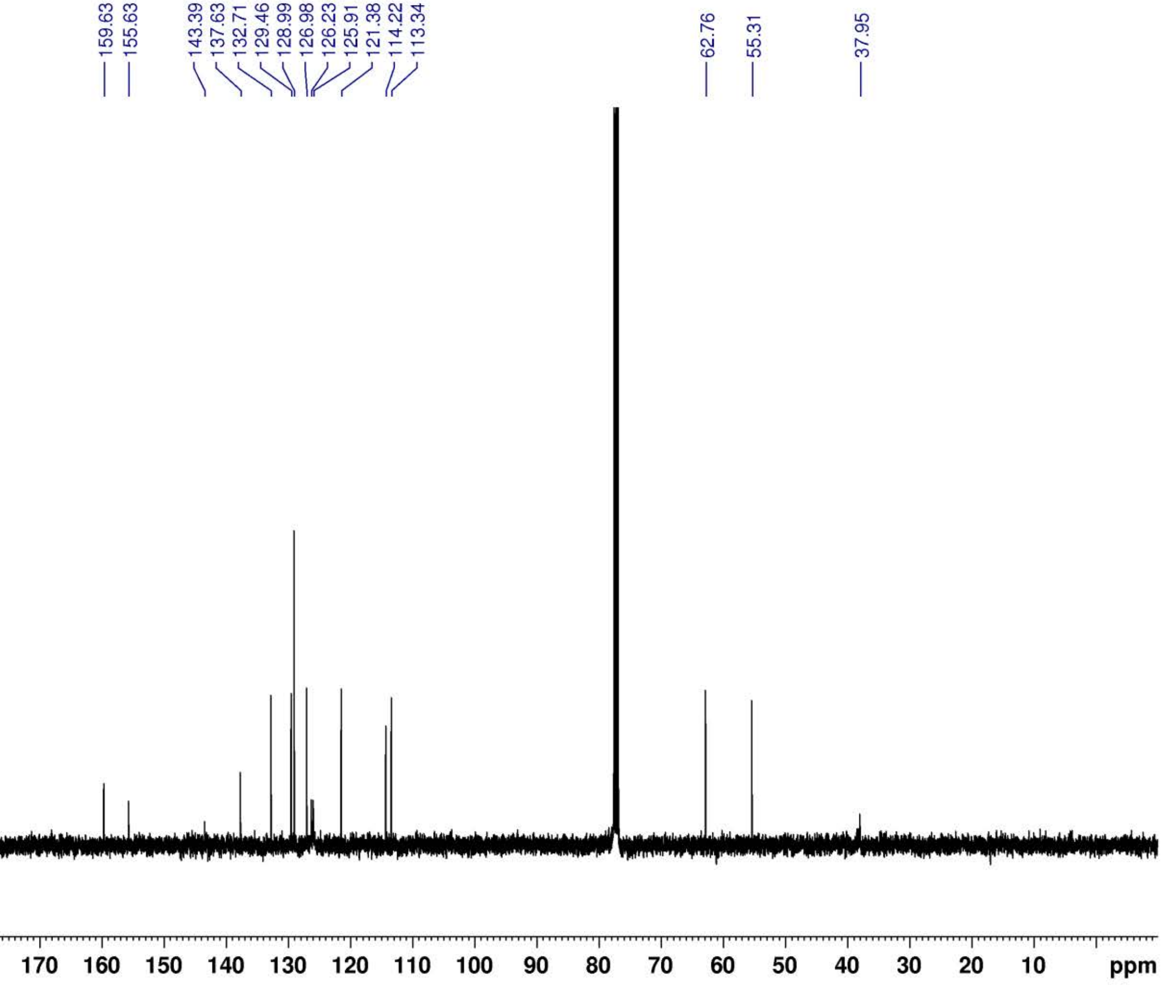

^{1}H NMR (400 MHz, CDCl$_3$) for (*Z*)-3-(3-bromophenyl)allyl methyl(phenyl)carbamate (**1i**)

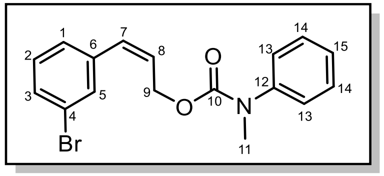

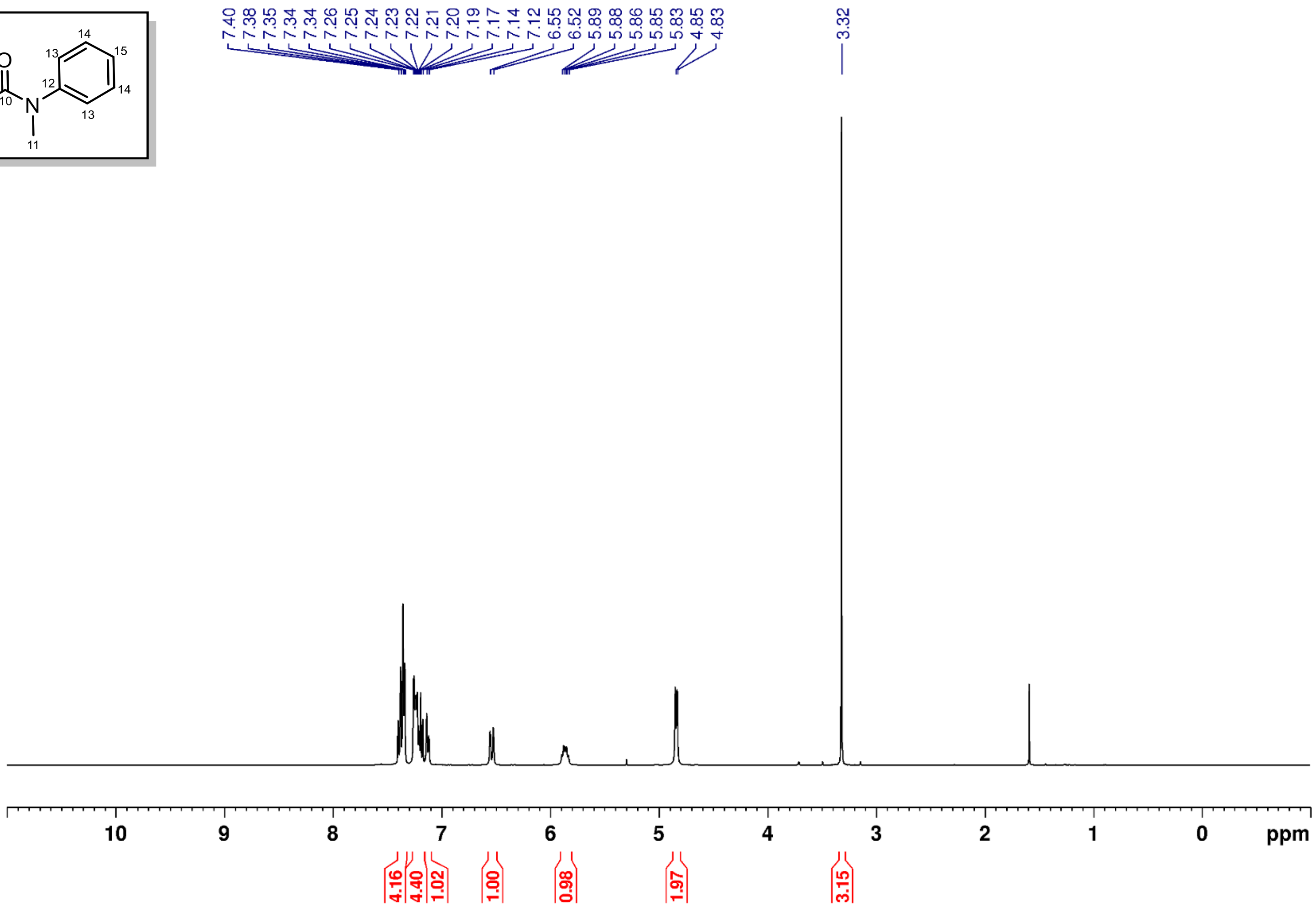

^{13}C NMR (101 MHz, CDCl₃) for (*Z*)-3-(3-bromophenyl)allyl methyl(phenyl)carbamate (**1i**)

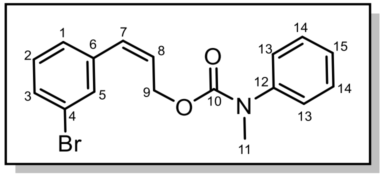

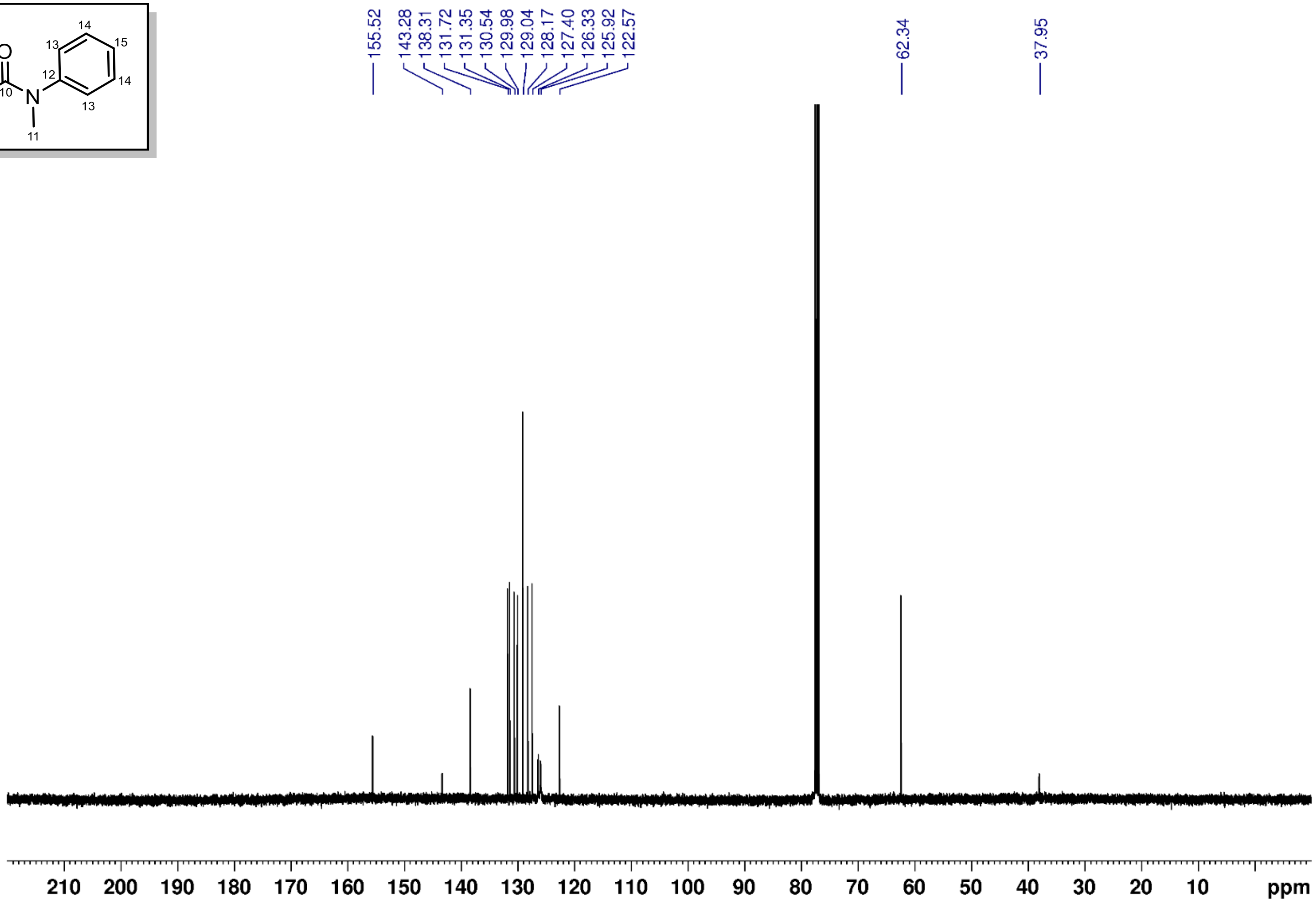

^{1}H NMR (400 MHz, CDCl$_3$) for ethyl (*Z*)-3-(3-((methyl(phenyl)carbamoyl)oxy)prop-1-en-1-yl)benzoate (**1j**)

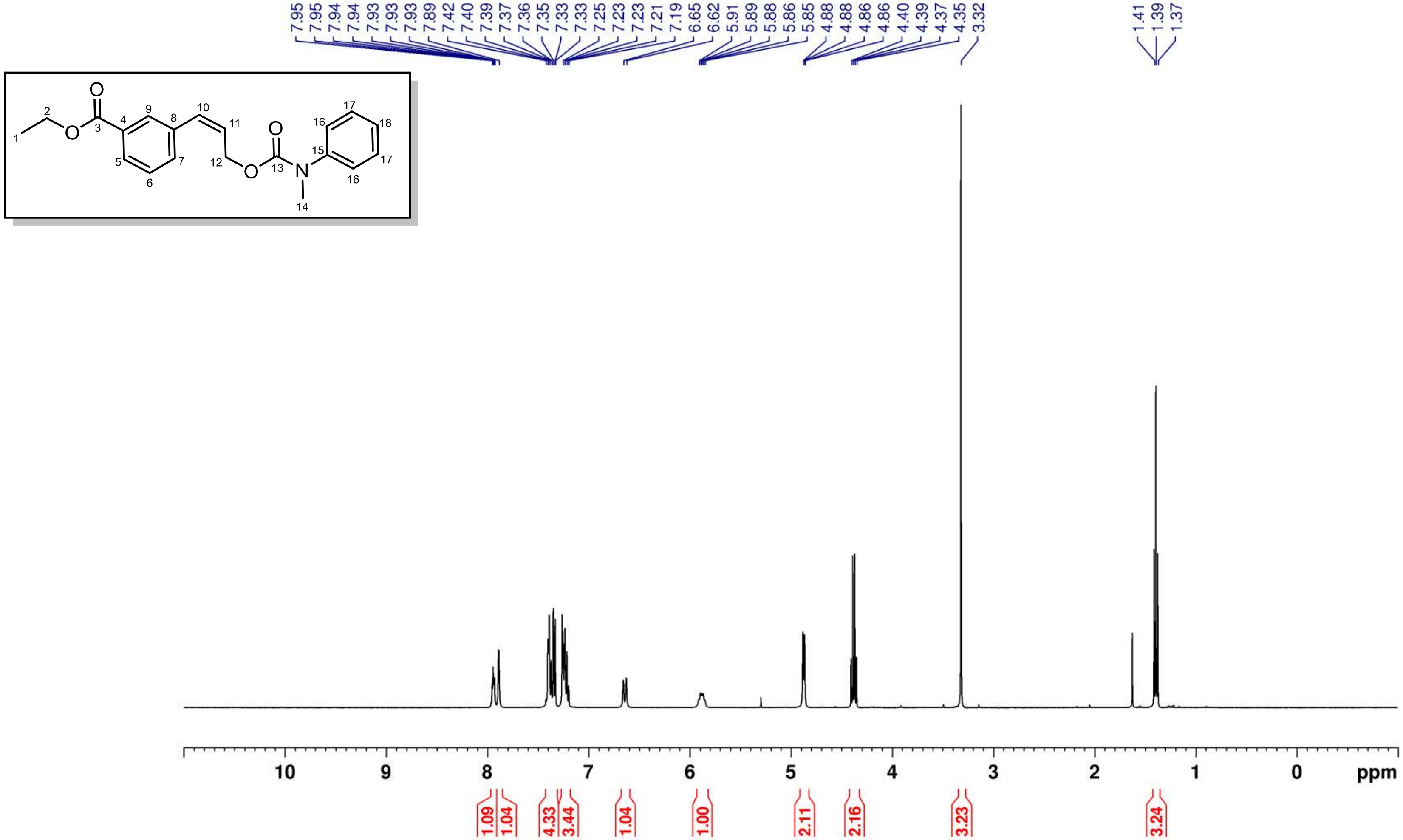

^{13}C NMR (101 MHz, $CDCl_3$) for ethyl (*Z*)-3-(3-((methyl(phenyl)carbamoyl)oxy)prop-1-en-1-yl)benzoate (**1j**)

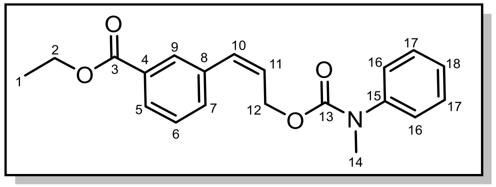

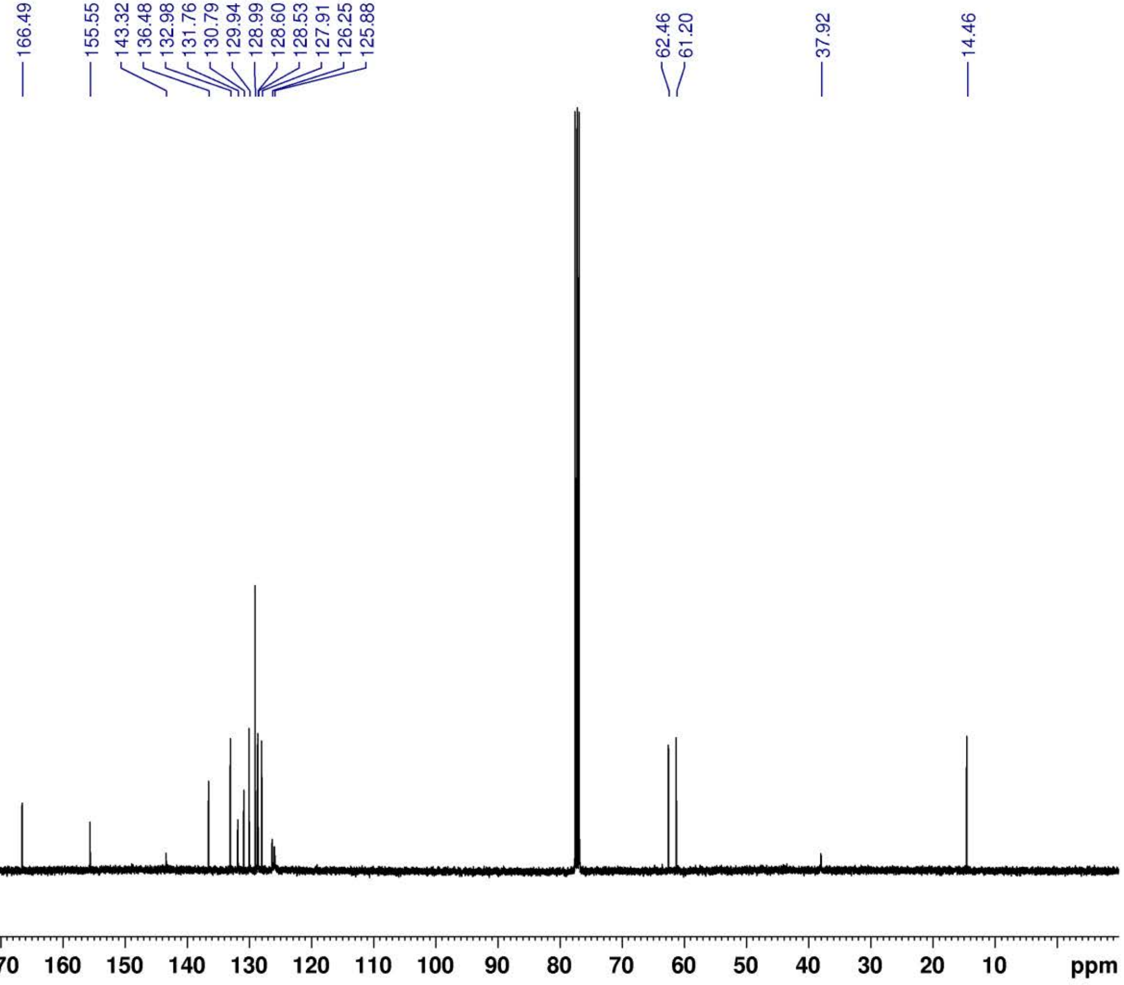

^{1}H NMR (700 MHz, CDCl$_3$) for (*Z*)-3-(3,4-dichlorophenyl)allyl methyl(phenyl)carbamate (**1k**)

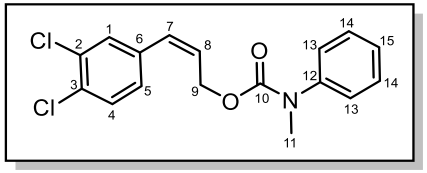

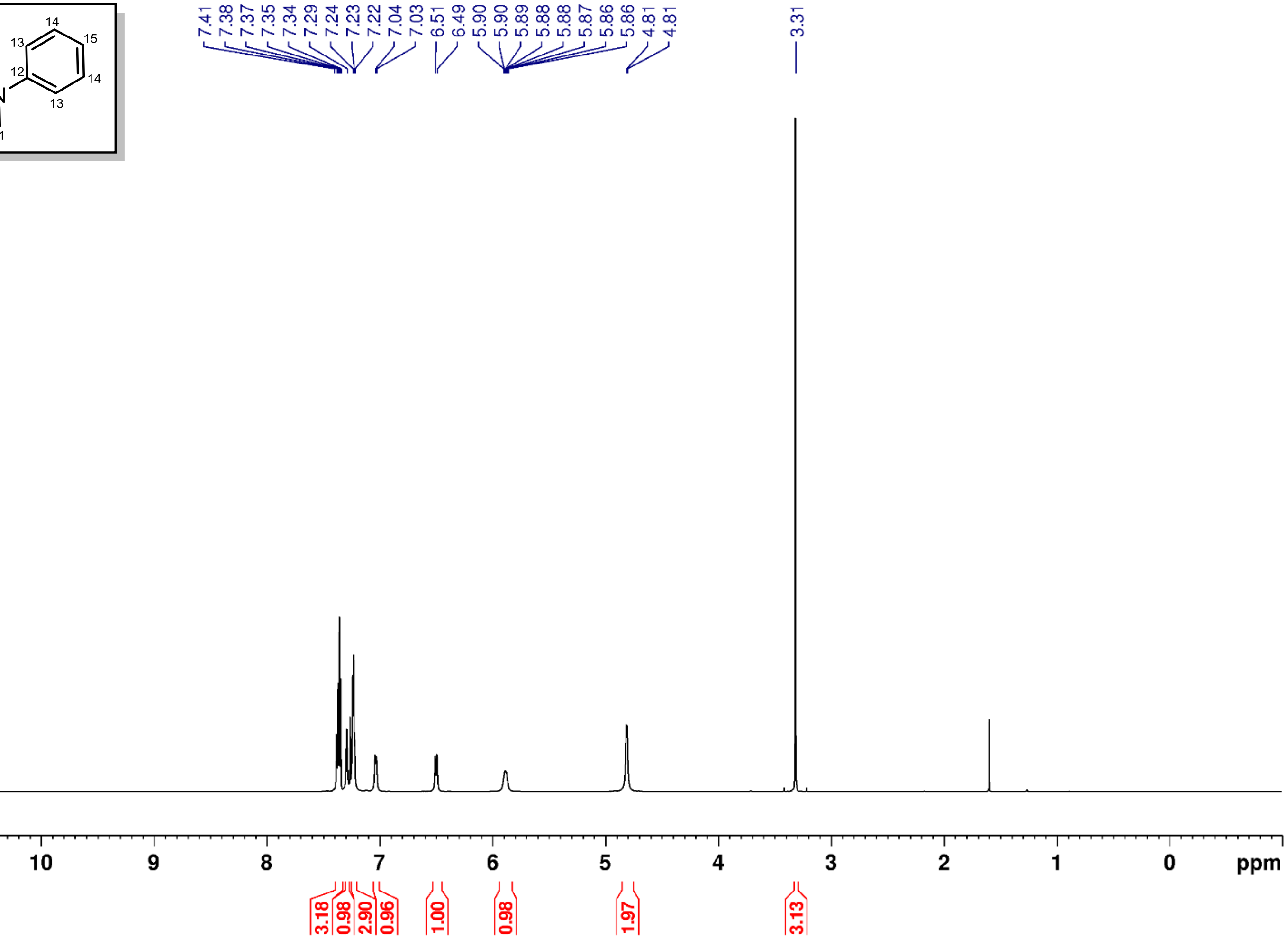

^{13}C NMR (176 MHz, CDCl$_3$) for (*Z*)-3-(3,4-dichlorophenyl)allyl methyl(phenyl)carbamate (**1k**)

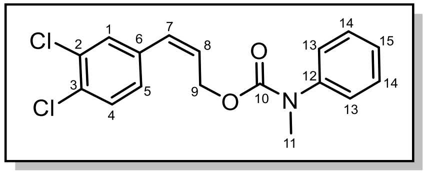

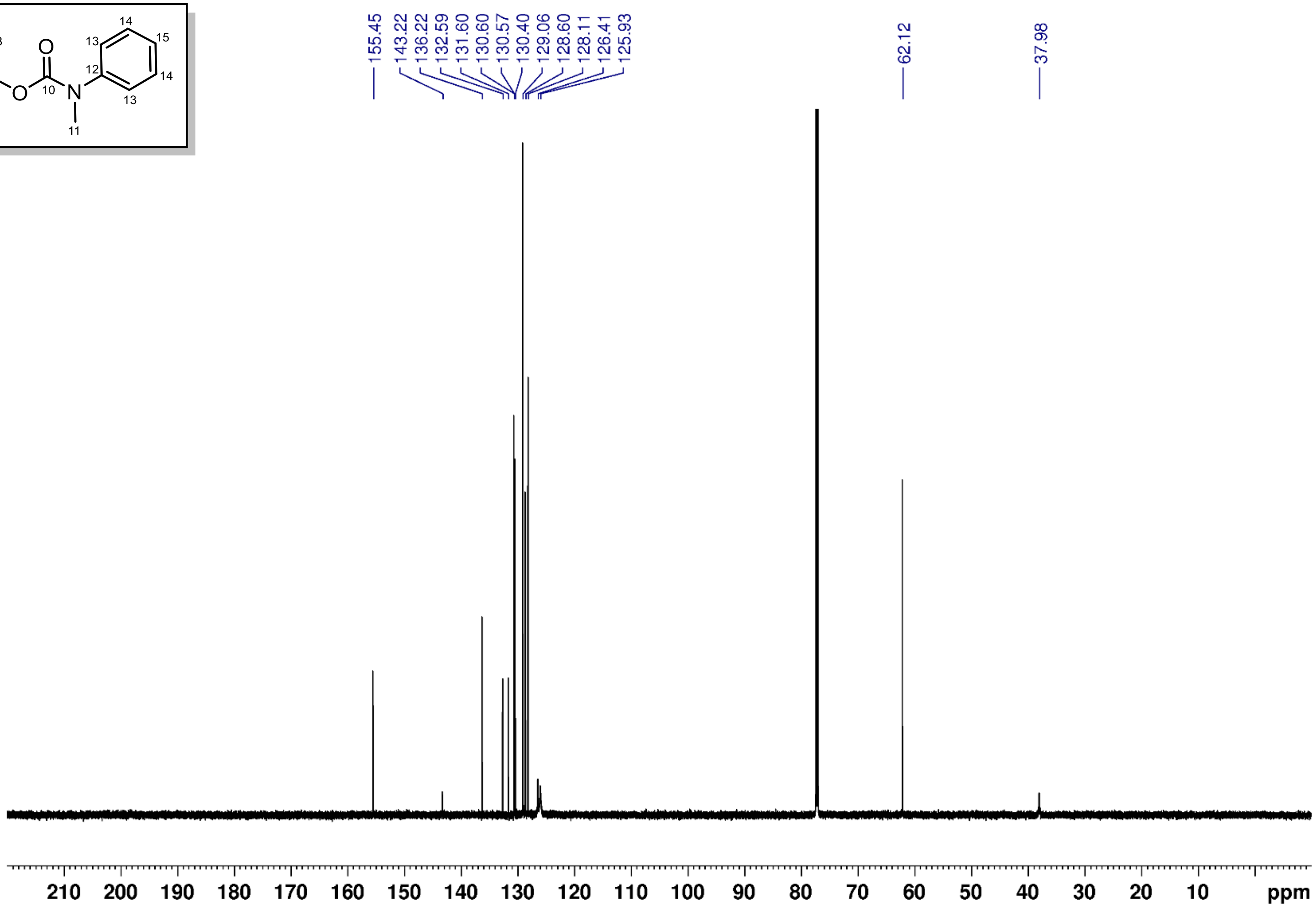

^{1}H NMR (500 MHz, $CDCl_3$) for (*Z*)-3-(4-(tert-butyl)phenyl)allyl methyl(phenyl)carbamate (**1l**)

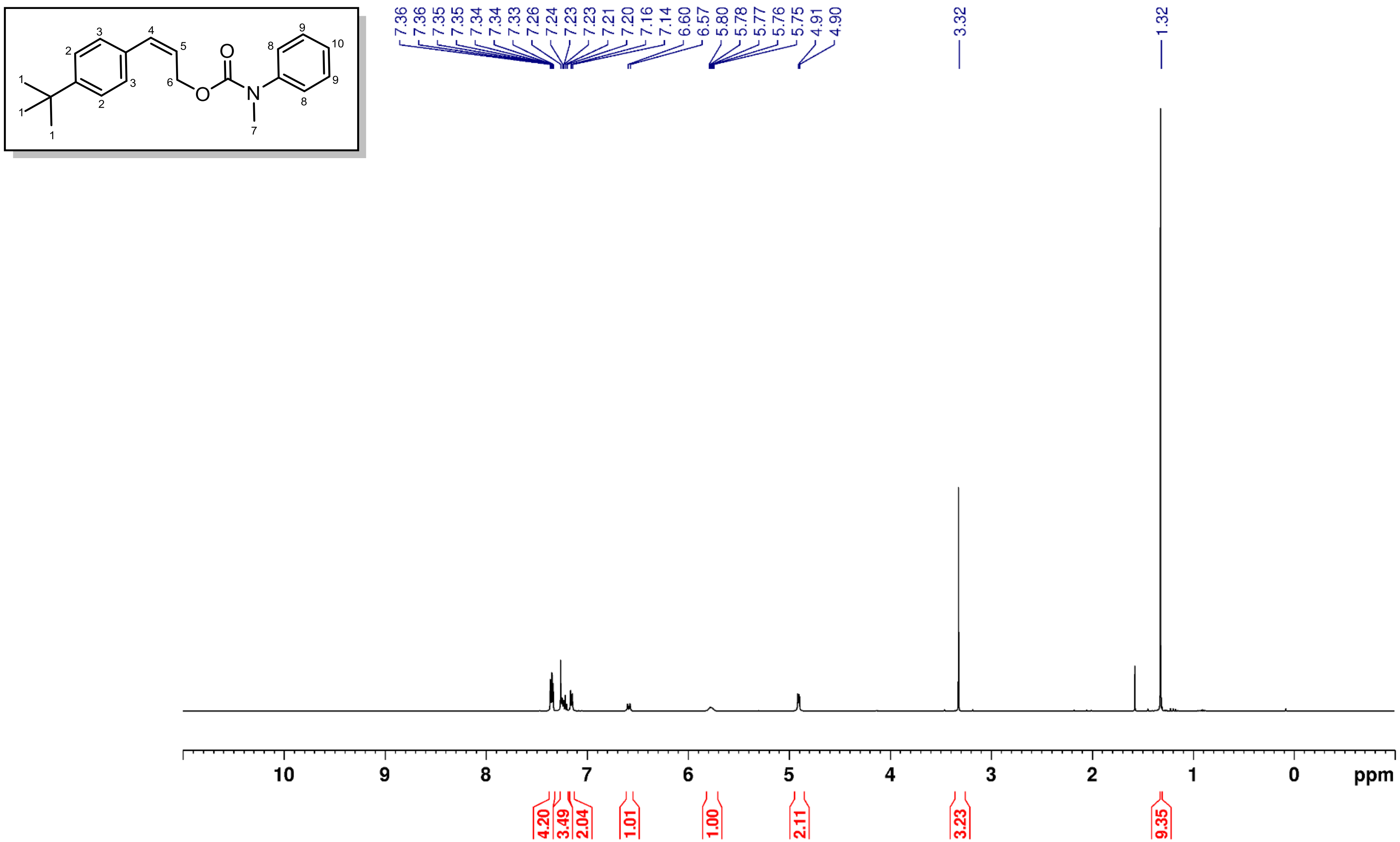

^{13}C NMR (126 MHz, $CDCl_3$) for (*Z*)-3-(4-(tert-butyl)phenyl)allyl methyl(phenyl)carbamate (**1l**)

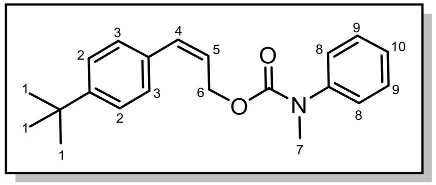

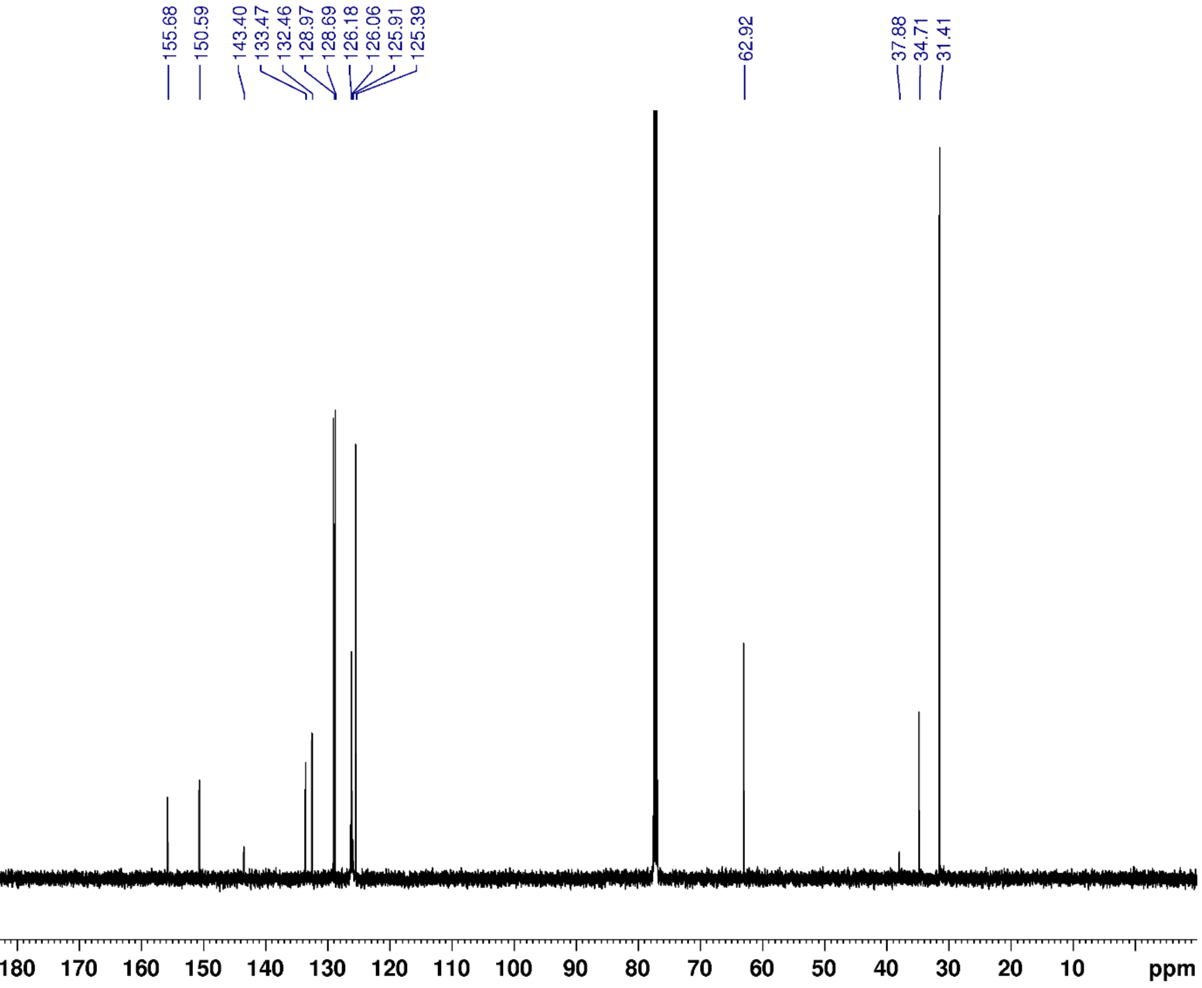

¹H NMR (700 MHz, CDCl₃) for (*Z*)-3-(4-(trifluoromethoxy)phenyl)allyl methyl(phenyl)carbamate (**1m**)

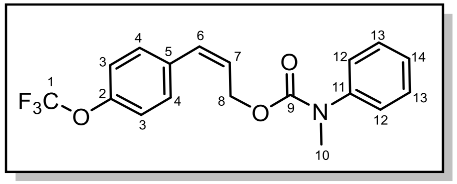

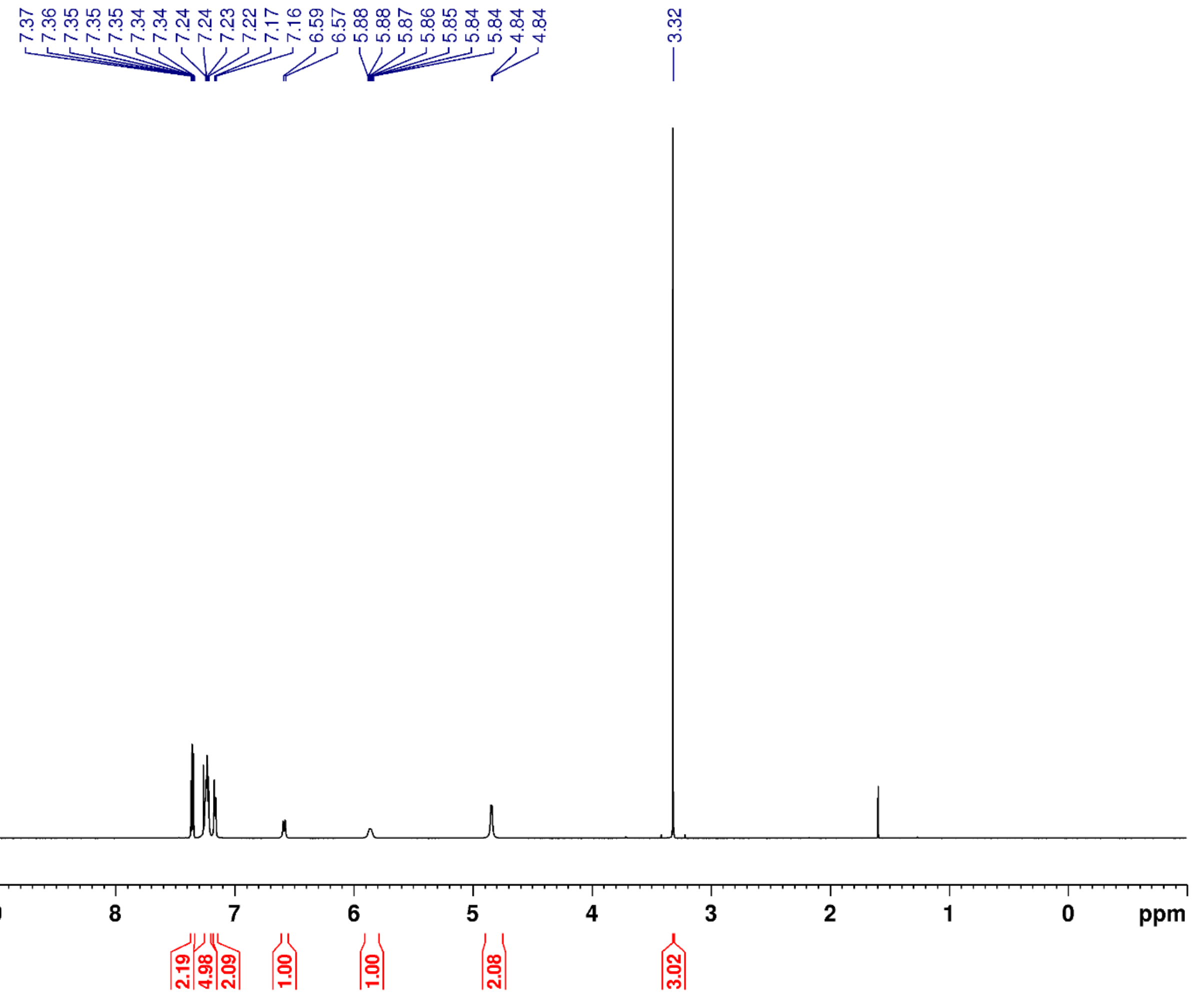

^{13}**C NMR (176 MHz, CDCl$_3$)** for (*Z*)-3-(4-(trifluoromethoxy)phenyl)allyl methyl(phenyl)carbamate (**1m**)

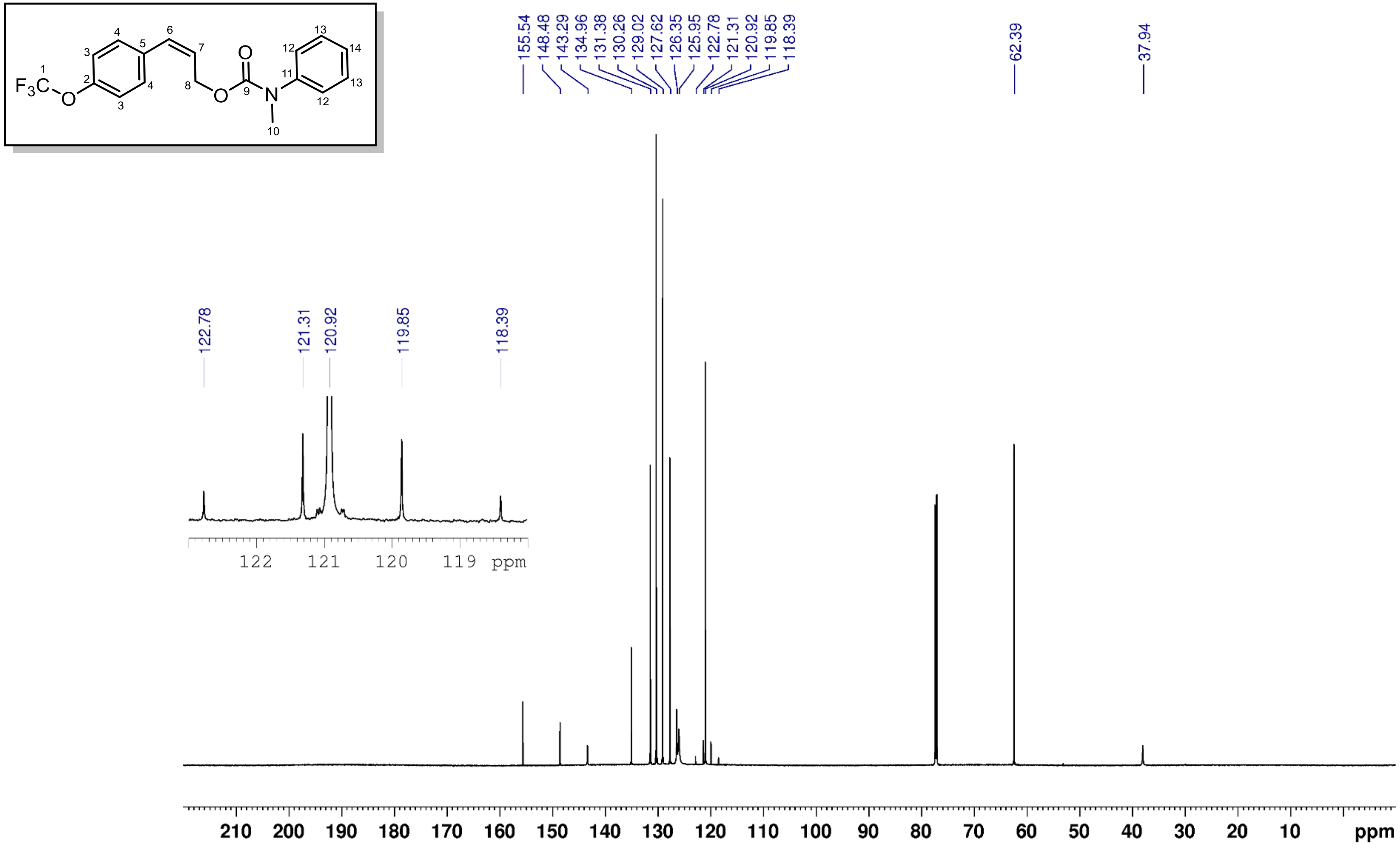

^{19}F NMR (376 MHz, CDCl$_3$) for (*Z*)-3-(4-(trifluoromethoxy)phenyl)allyl methyl(phenyl)carbamate (**1m**)

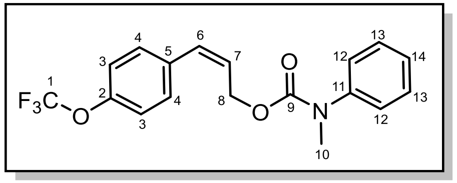

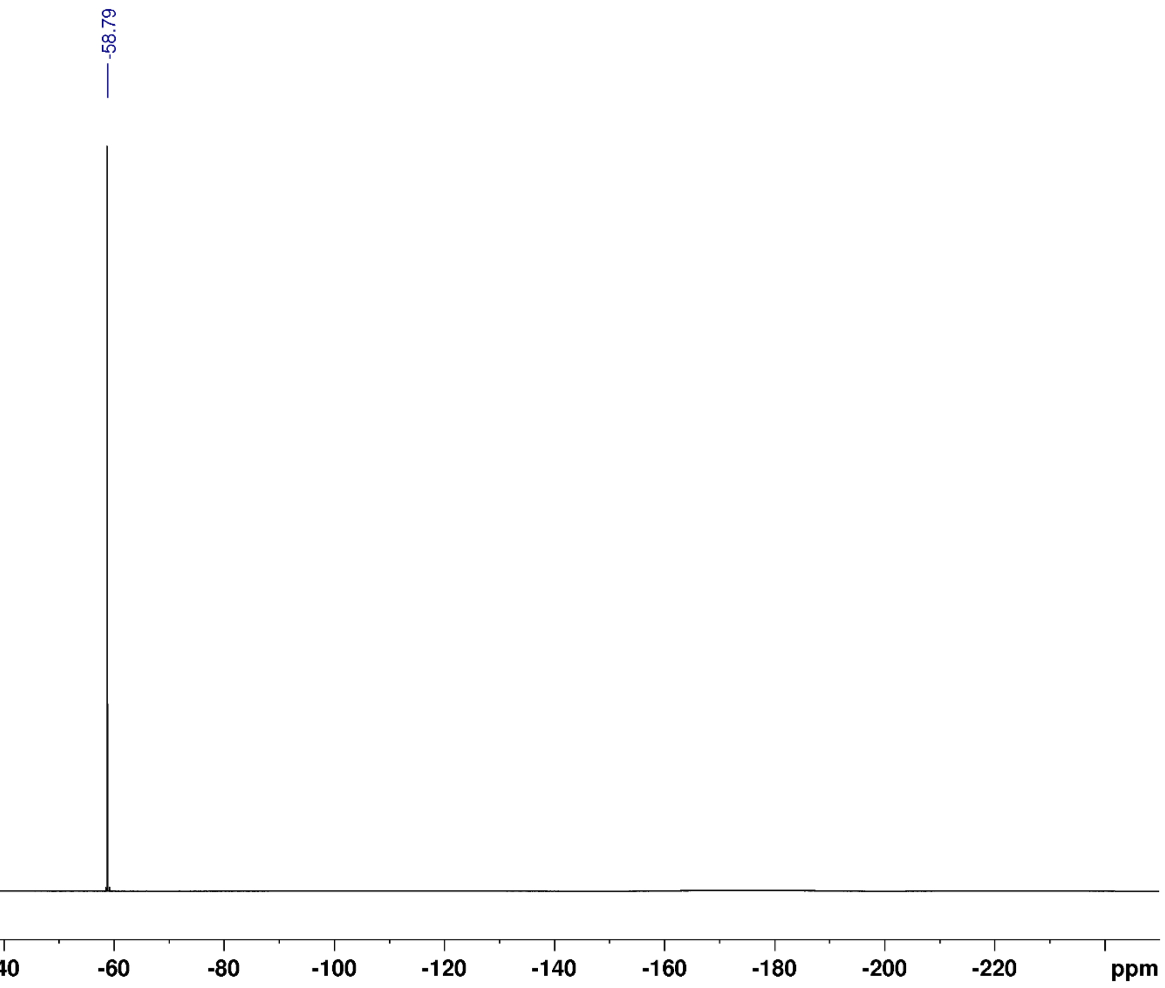

¹H NMR (500 MHz, $CDCl_3$) for (*Z*)-3-(4-chlorophenyl)allyl methyl(phenyl)carbamate (**1n**)

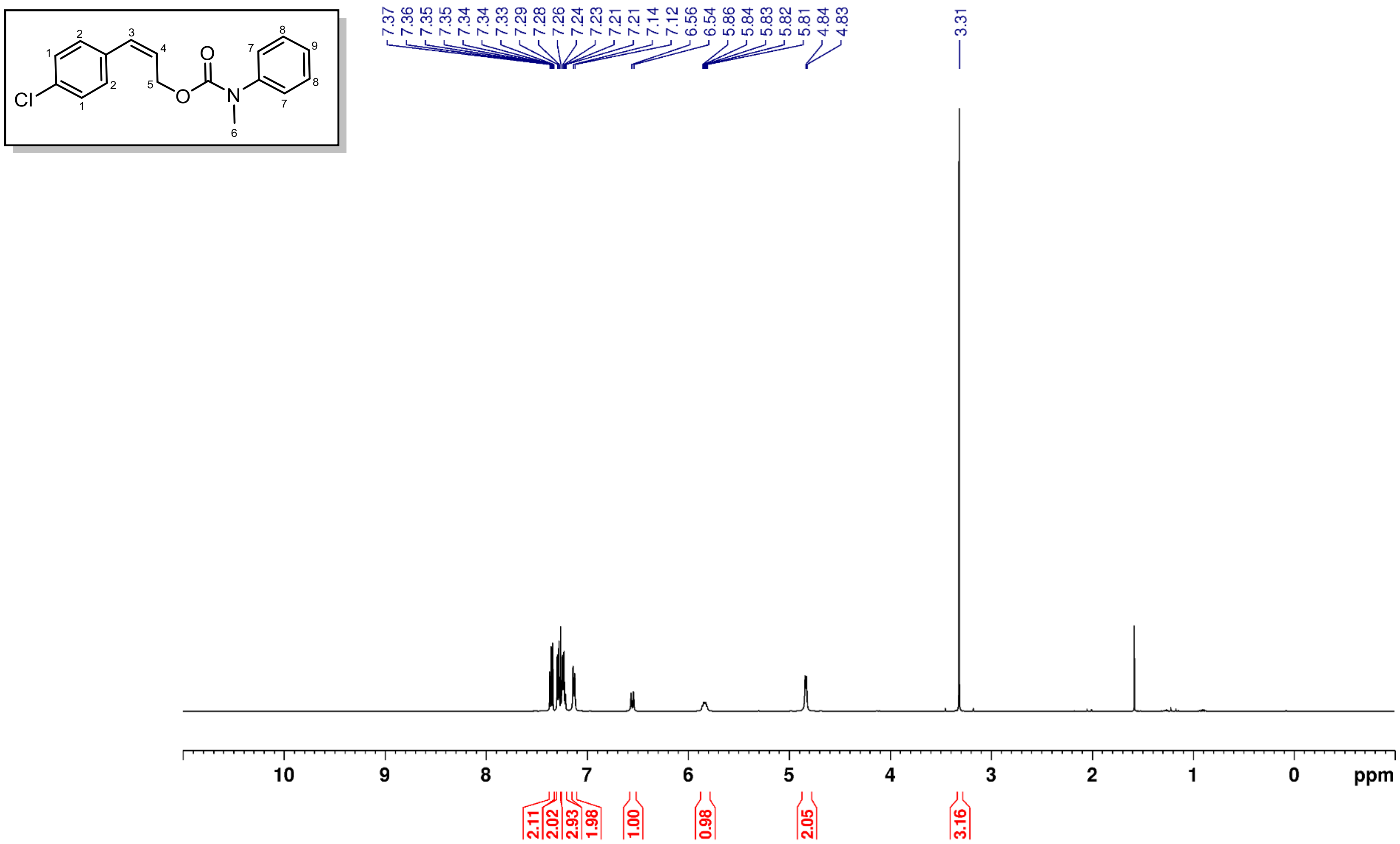

^{13}C NMR (126 MHz, CDCl$_3$) for (*Z*)-3-(4-chlorophenyl)allyl methyl(phenyl)carbamate (**1n**)

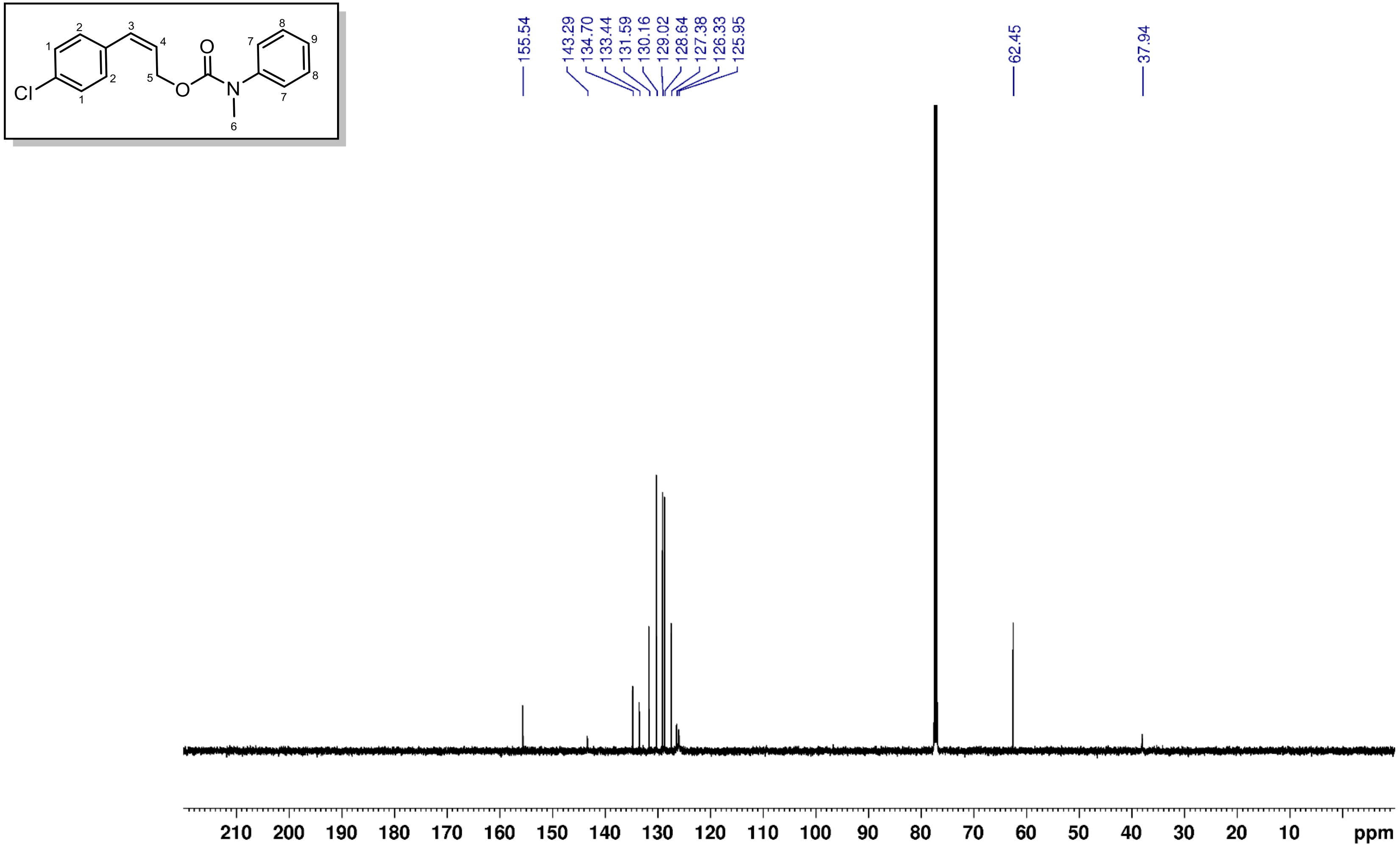

^{1}H NMR (700 MHz, $CDCl_3$) for (*Z*)-3-(4-(trifluoromethyl)phenyl)allyl methyl(phenyl)carbamate (**1o**)

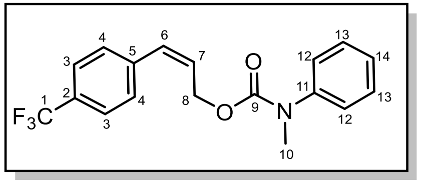

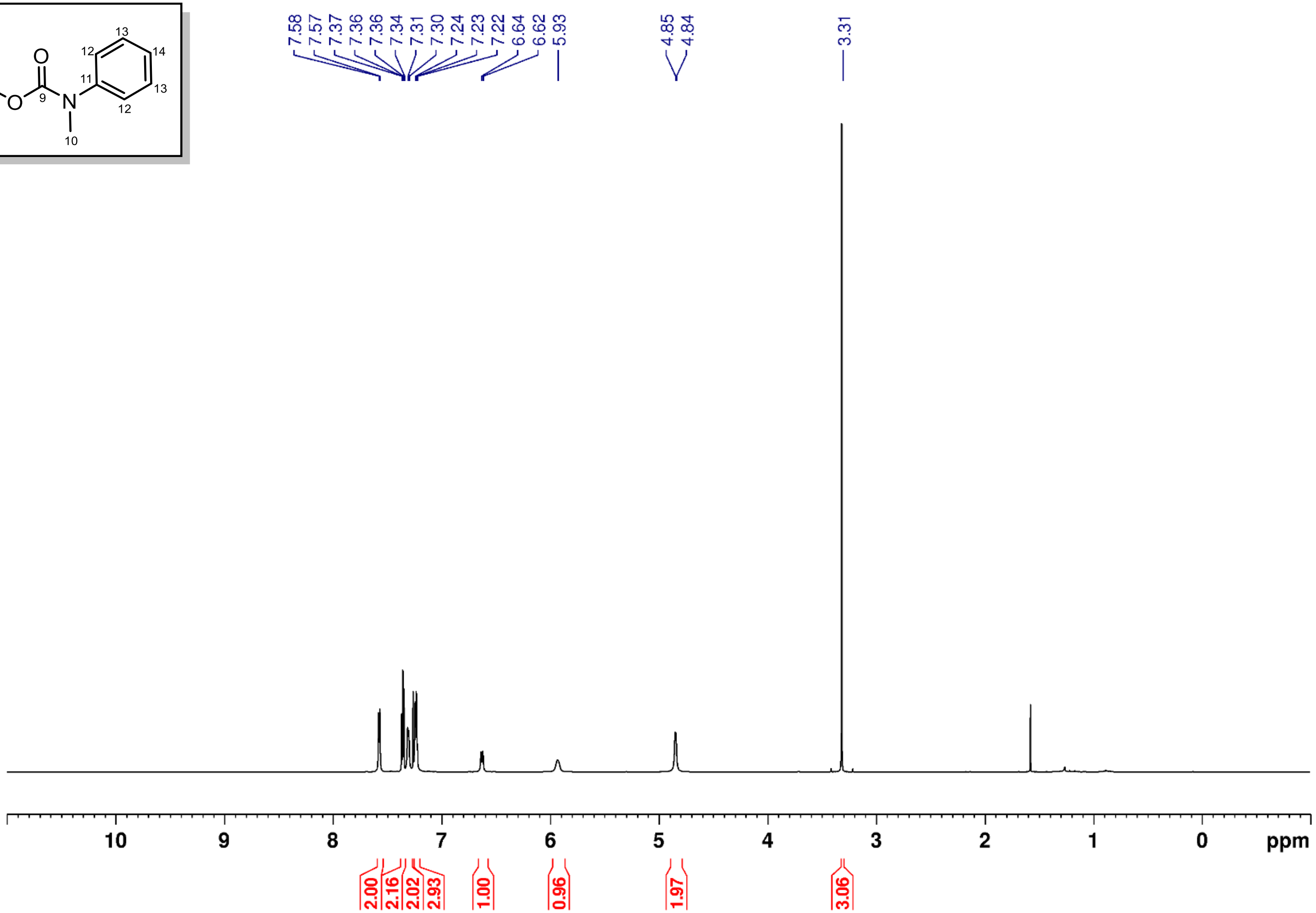

^{13}C NMR (176 MHz, CDCl$_3$) for (*Z*)-3-(4-(trifluoromethyl)phenyl)allyl methyl(phenyl)carbamate (**1o**)

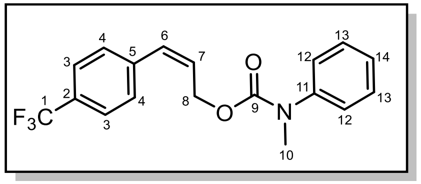

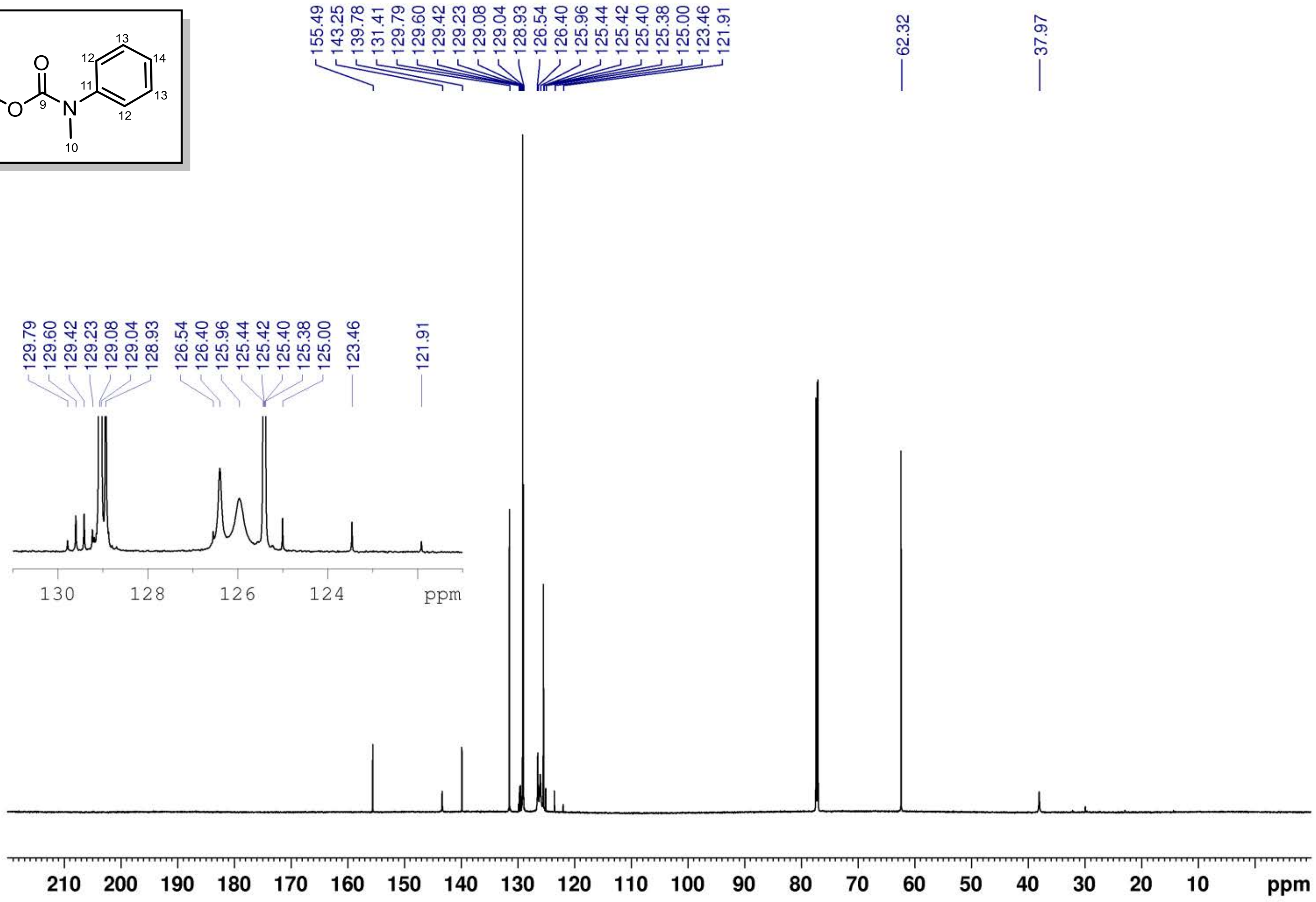

¹⁹F NMR (376 MHz, CDCl$_3$) for (*Z*)-3-(4-(trifluoromethyl)phenyl)allyl methyl(phenyl)carbamate (**1o**)

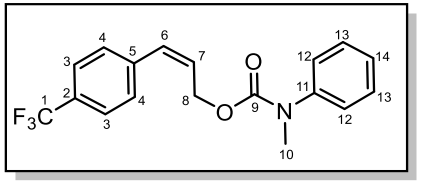

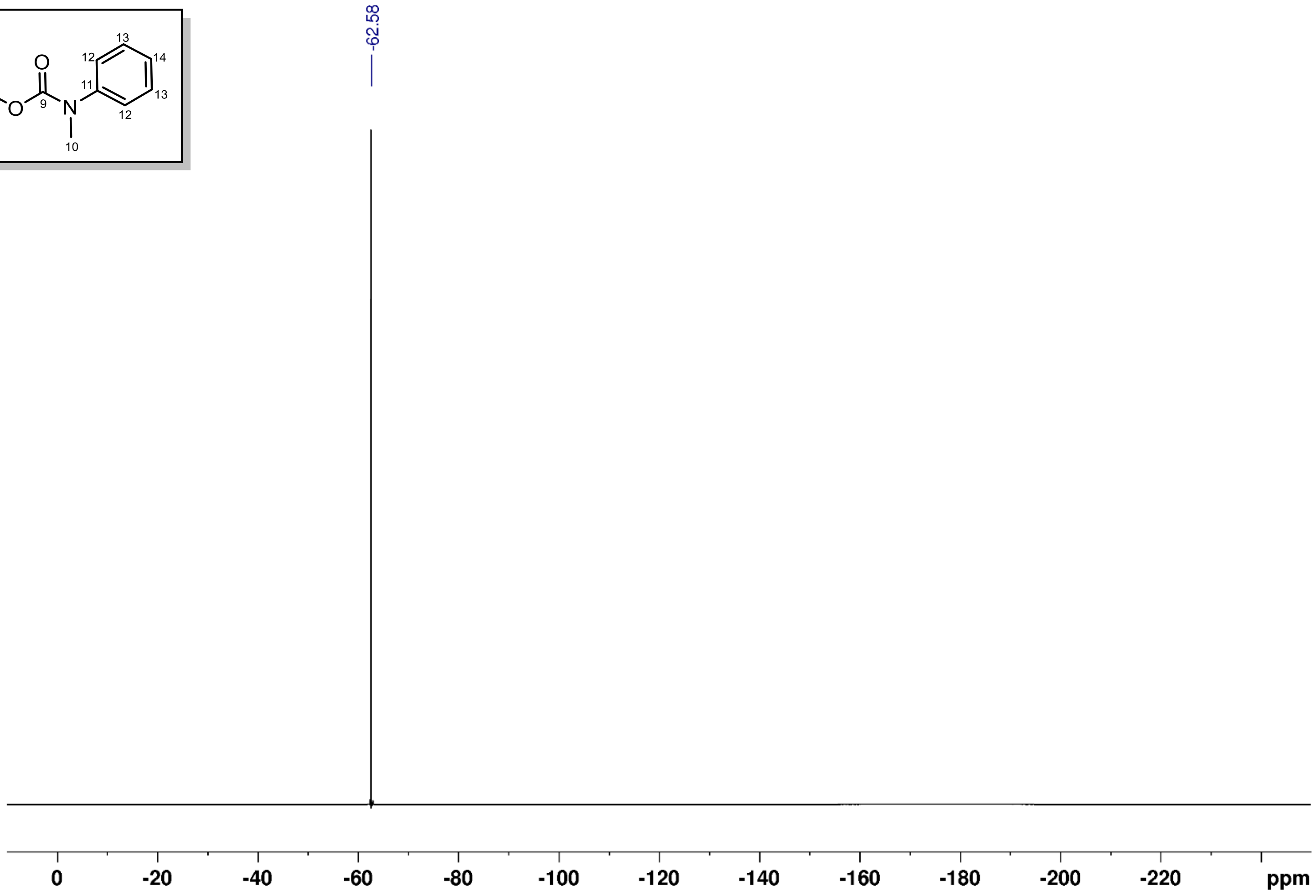

^{1}H NMR (500 MHz, CDCl$_3$) for (*Z*)-3-(naphthalen-1-yl)allyl methyl(phenyl)carbamate (**1p**)

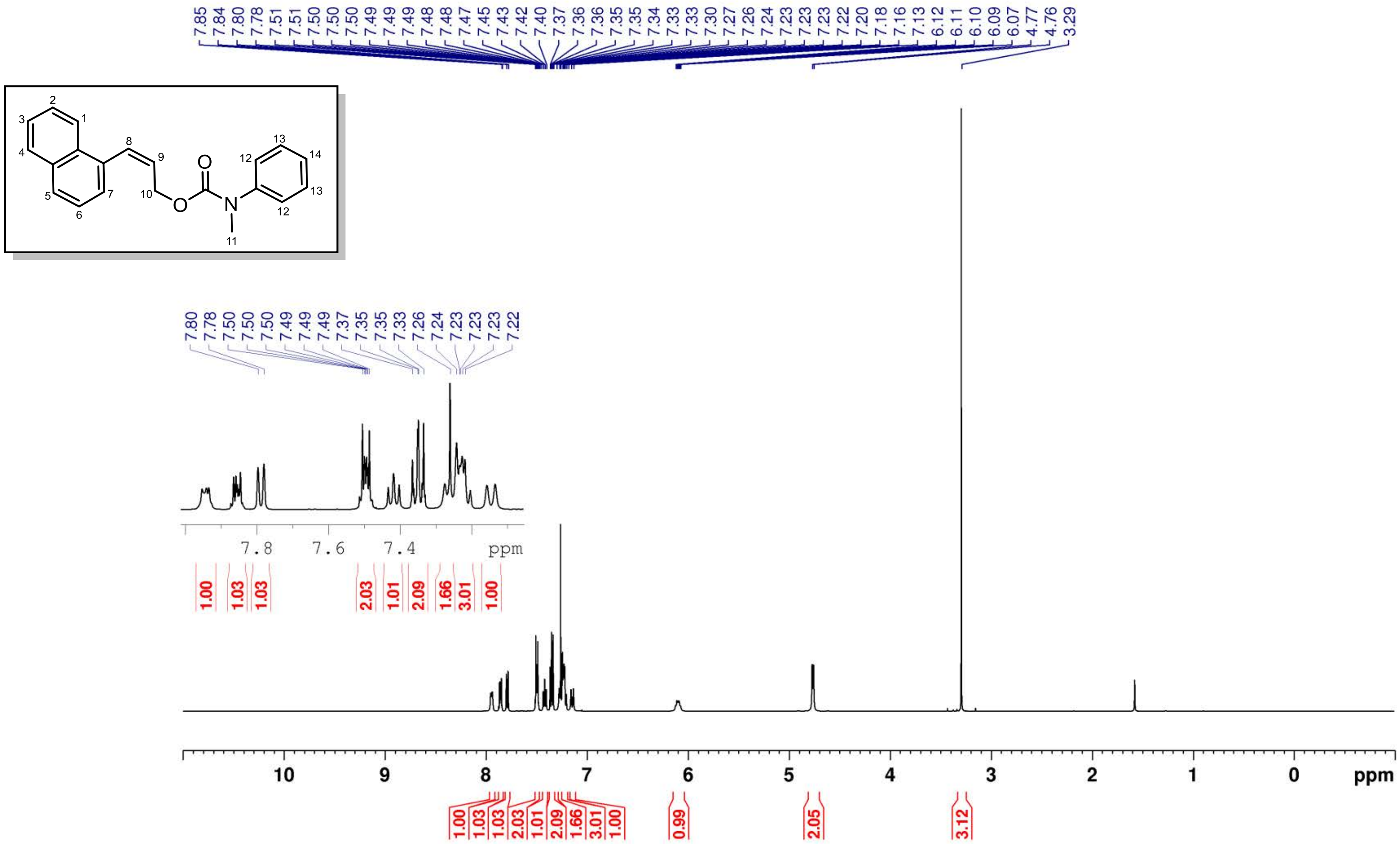

^{13}C NMR (126 MHz, $CDCl_3$) for (*Z*)-3-(naphthalen-1-yl)allyl methyl(phenyl)carbamate (**1p**)

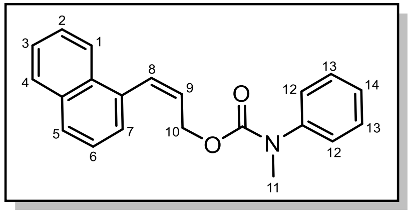

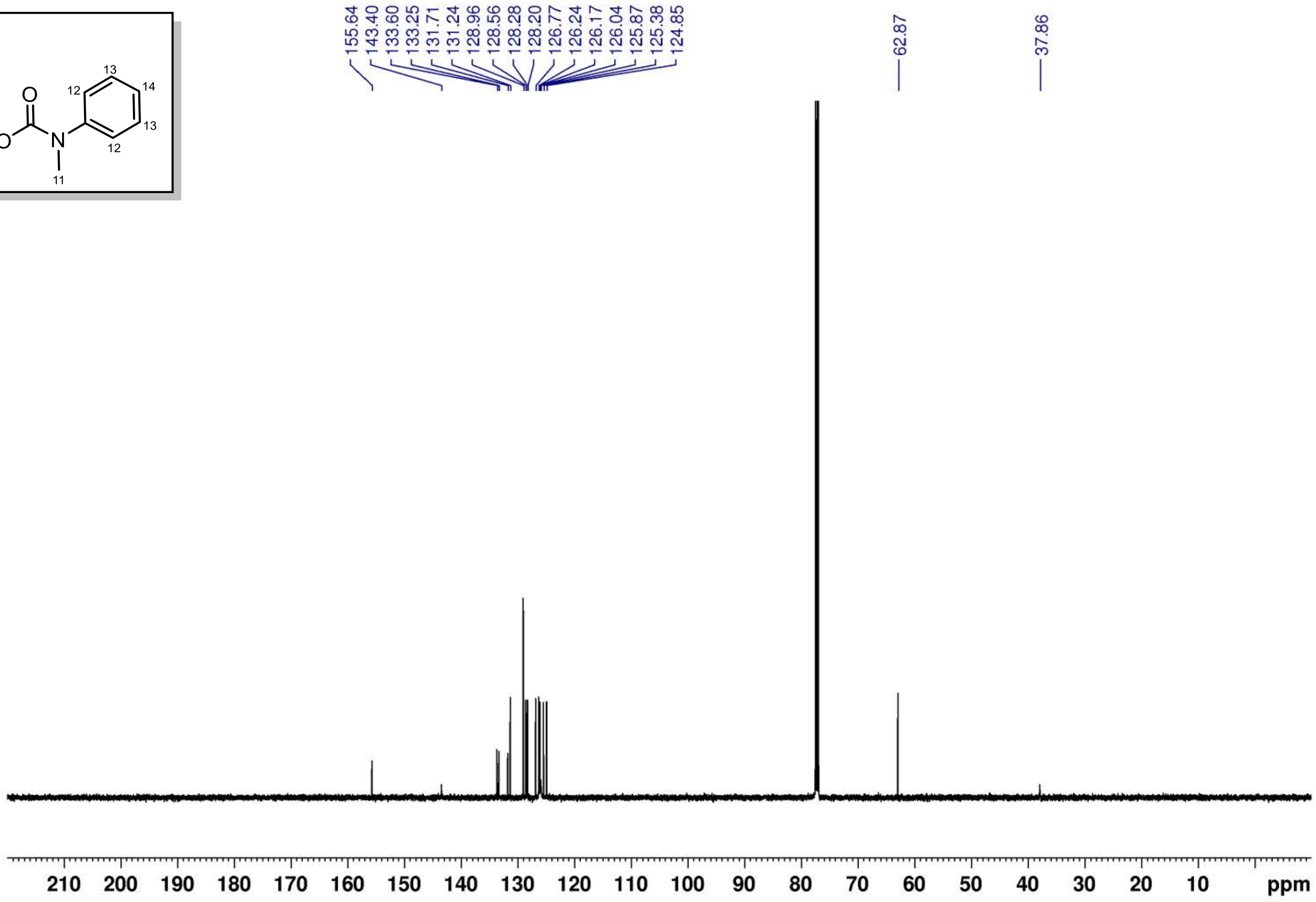

^{1}H NMR (500 MHz, $CDCl_3$) for (*Z*)-3-(naphthalen-2-yl)allyl methyl(phenyl)carbamate (**1q**)

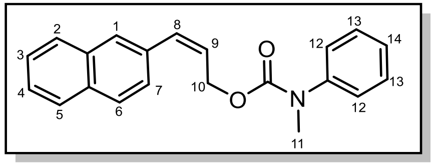

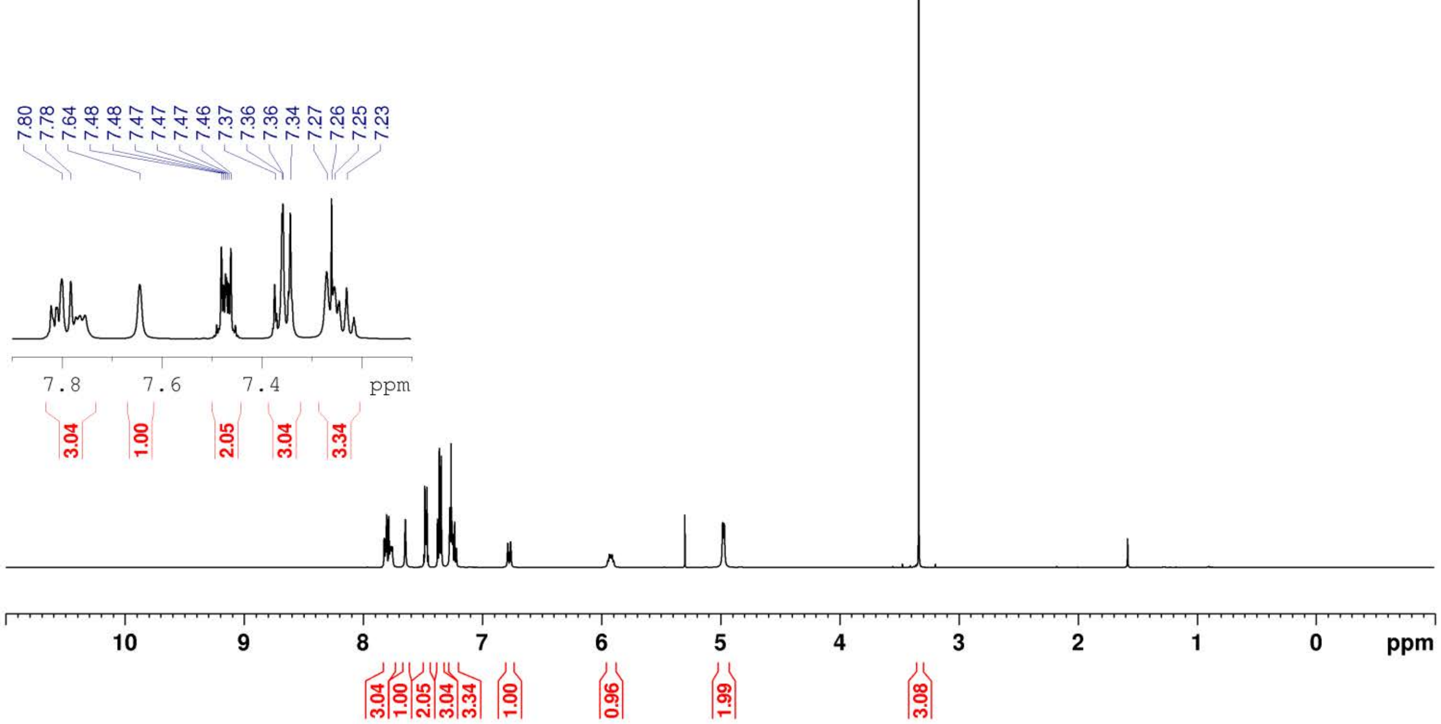

^{13}C NMR (126 MHz, CDCl$_3$) for (*Z*)-3-(naphthalen-2-yl)allyl methyl(phenyl)carbamate (**1q**)

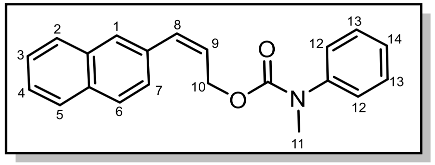

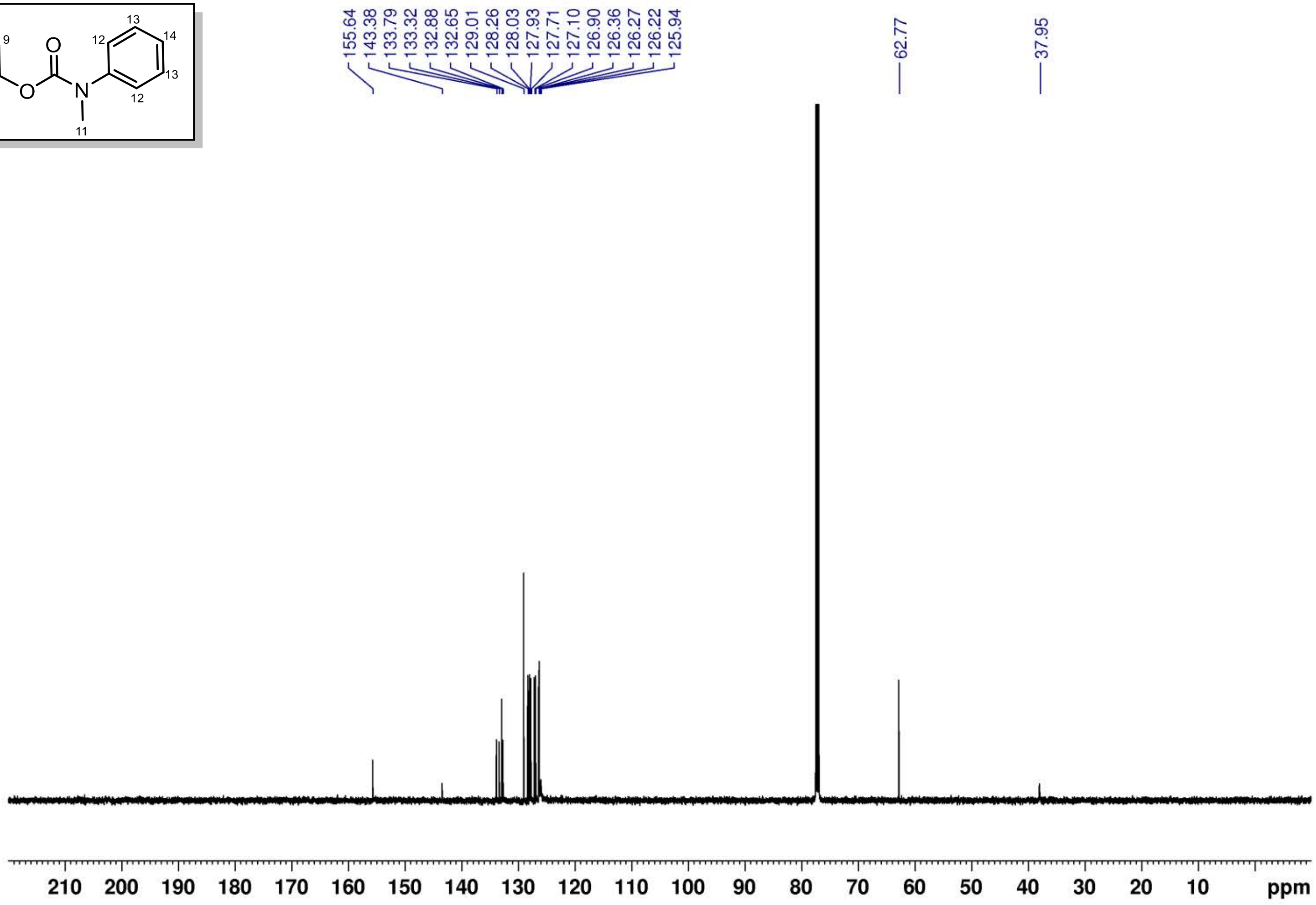

¹H NMR (400 MHz, CDCl₃) for (*Z*)-3-(thiophen-3-yl)allyl methyl(phenyl)carbamate (**1r**)

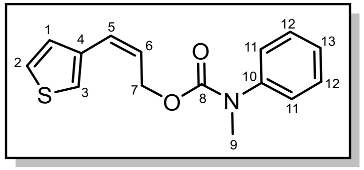

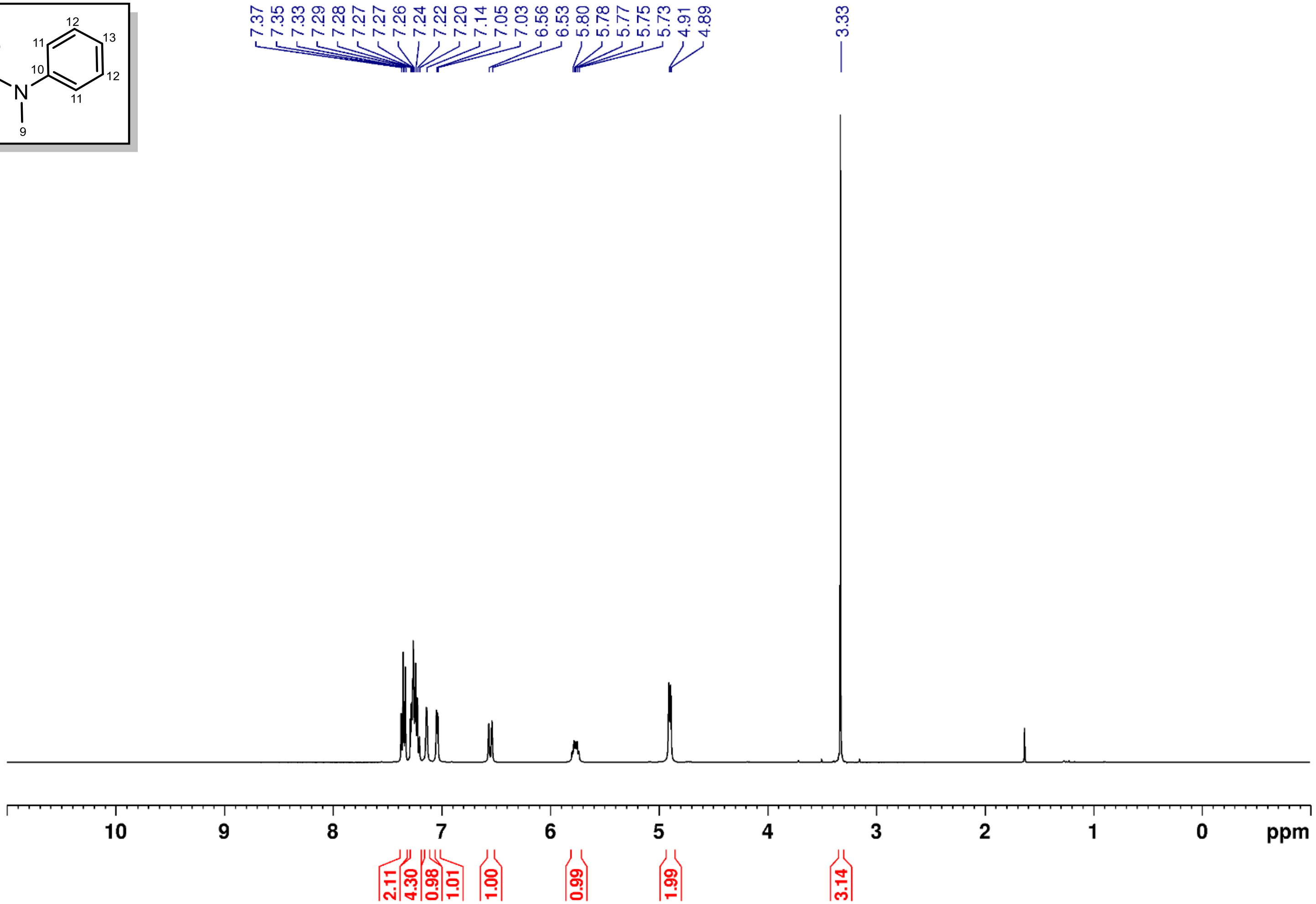

^{13}C NMR (101 MHz, CDCl$_3$) for (*Z*)-3-(thiophen-3-yl)allyl methyl(phenyl)carbamate (**1r**)

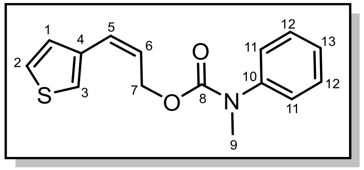

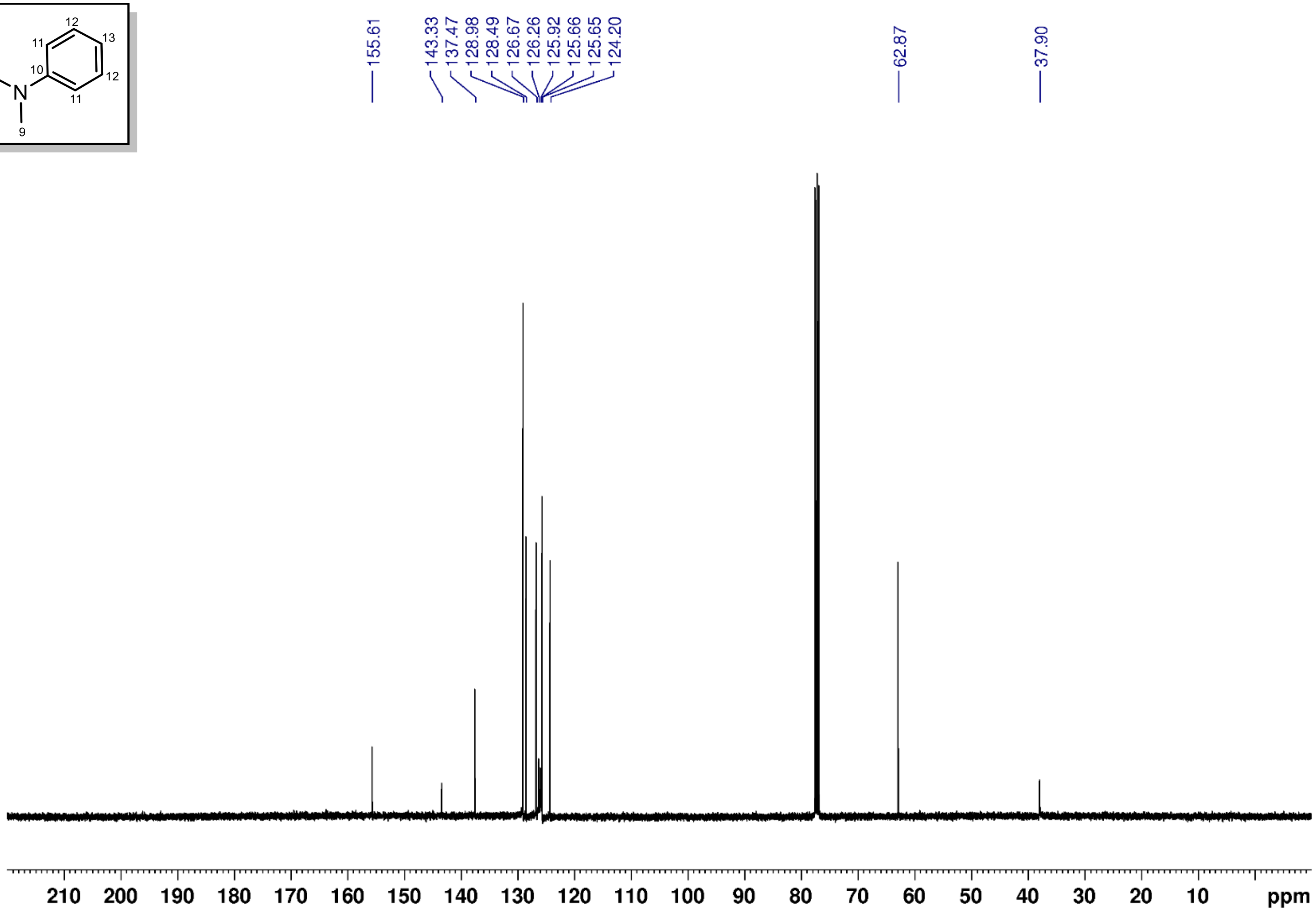

^{1}H NMR (700 MHz, CDCl$_3$) for methyl (*Z*)-5-(3-((methyl(phenyl)carbamoyl)oxy)prop-1-en-1-yl)-1-tosyl-1*H*-indole-3-carboxylate (**1s**)

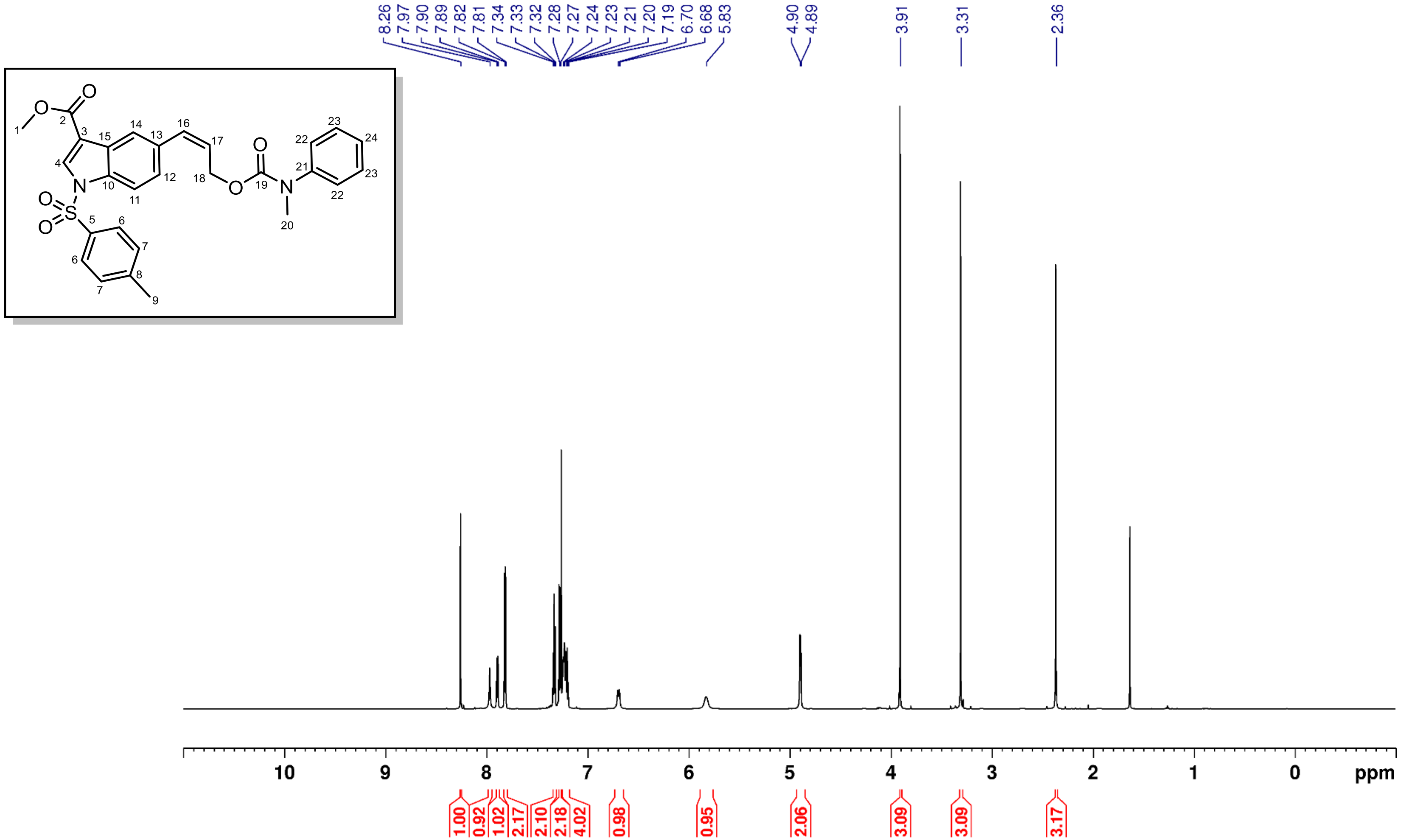

^{13}C NMR (176 MHz, $CDCl_3$) for methyl (*Z*)-5-(3-((methyl(phenyl)carbamoyl)oxy)prop-1-en-1-yl)-1-tosyl-1*H*-indole-3-carboxylate (**1s**)

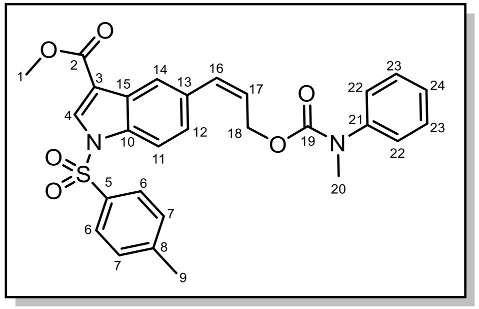

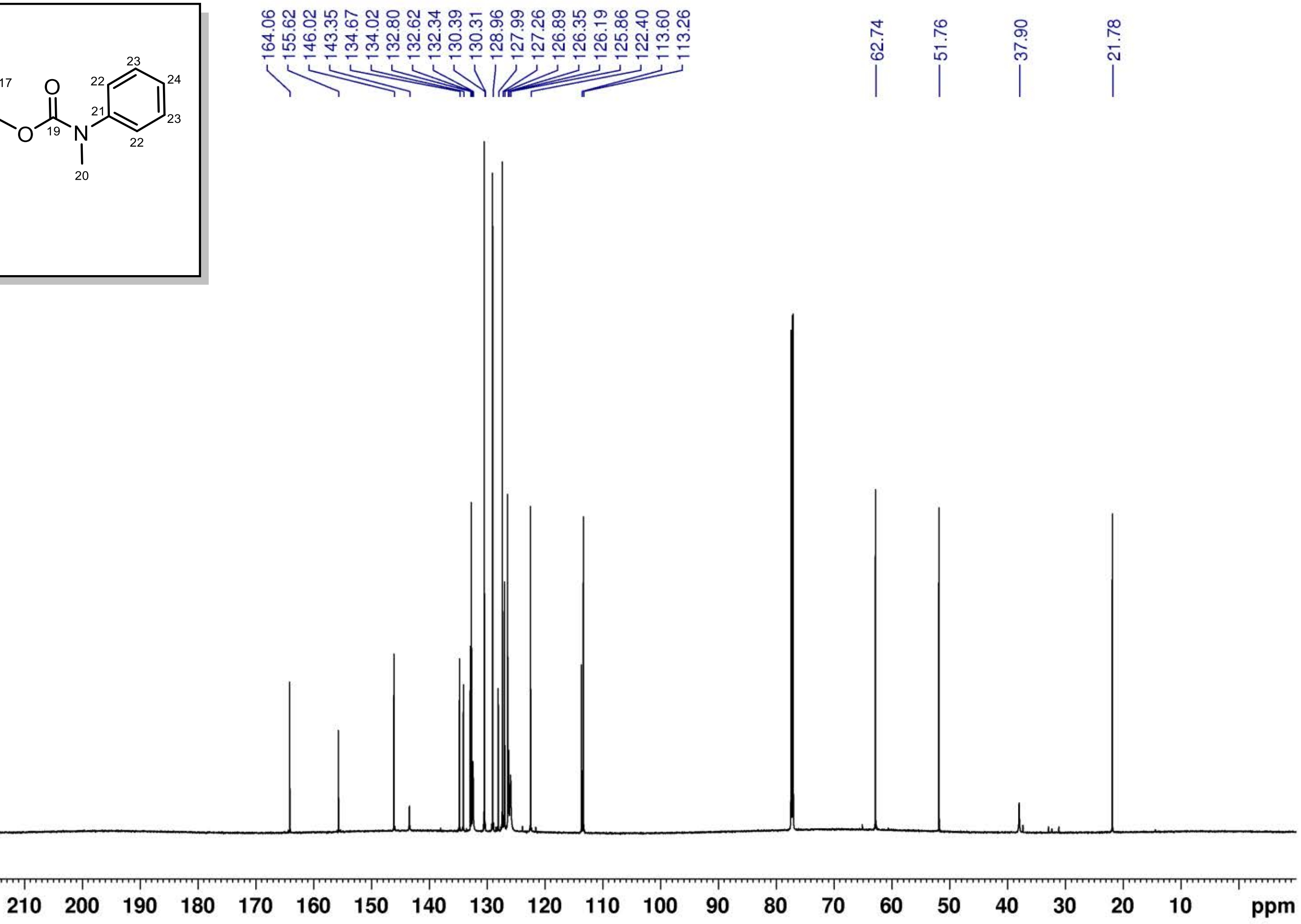

^{1}H NMR (400 MHz, CDCl$_3$) for (*Z*)-4-(4-(*tert*-butyl)phenyl)but-3-en-2-yl methyl(phenyl)carbamate ((***rac***)-**1t**))

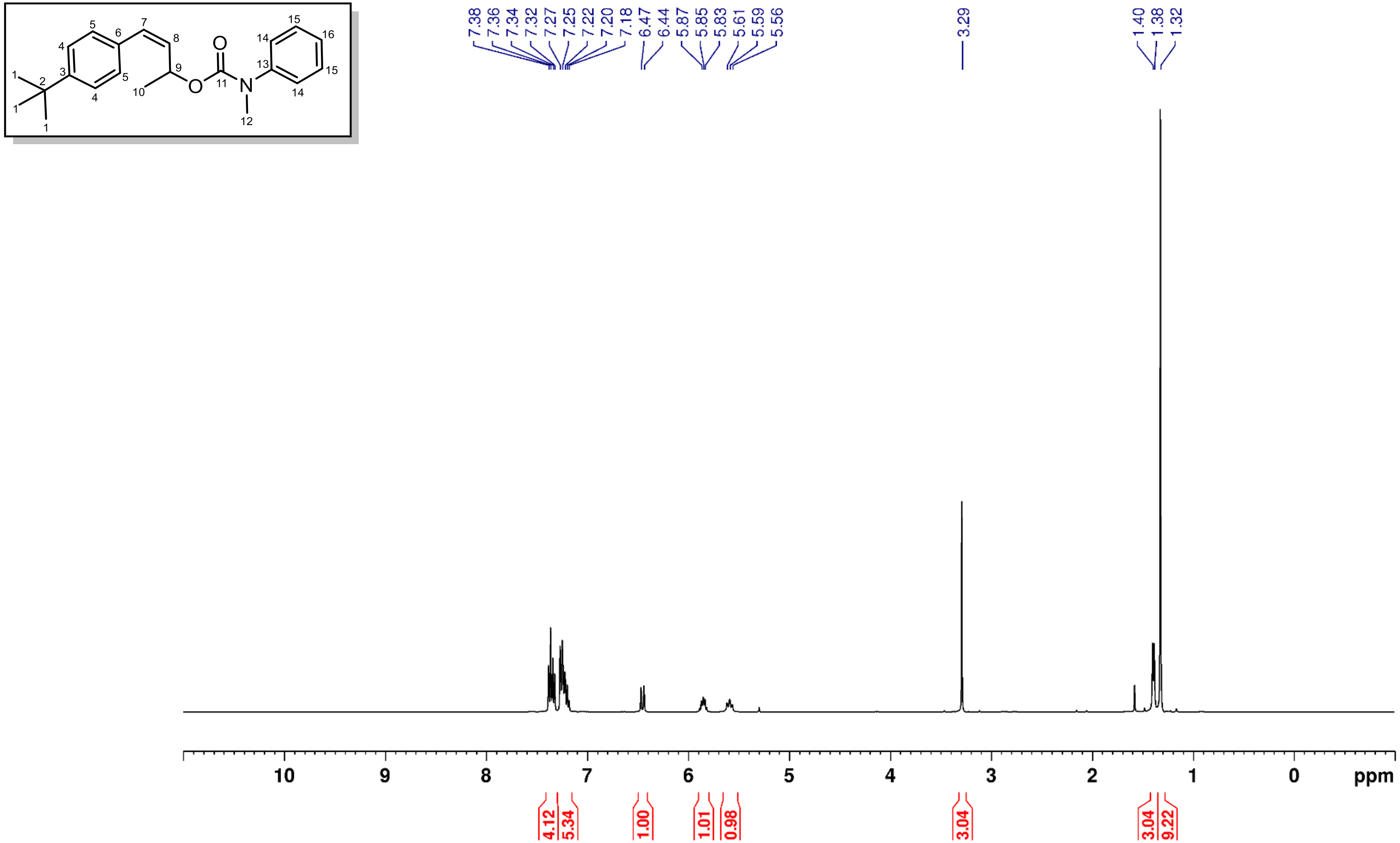

^{13}C NMR (101 MHz, CDCl$_3$) for (*Z*)-4-(4-(*tert*-butyl)phenyl)but-3-en-2-yl methyl(phenyl)carbamate ((***rac***)-**1t**))

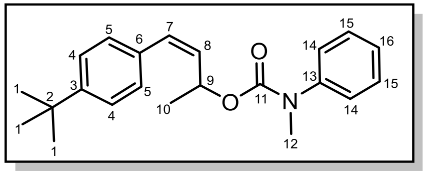

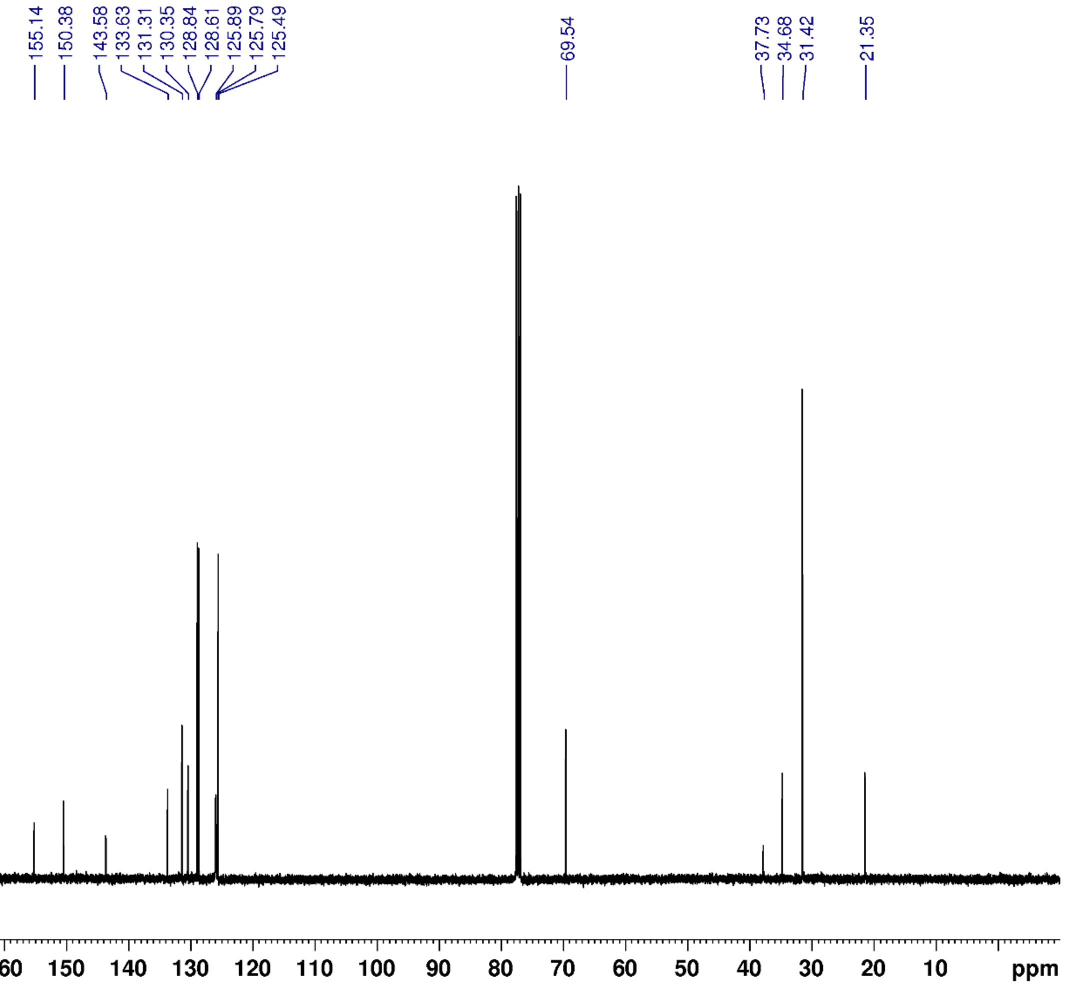

^{1}H NMR (700 MHz, $CDCl_3$) for (*Z*)-1-(4-(*tert*-butyl)phenyl)-4-methylpent-1-en-3-yl methyl(phenyl)carbamate ((***rac***)-**1u**))

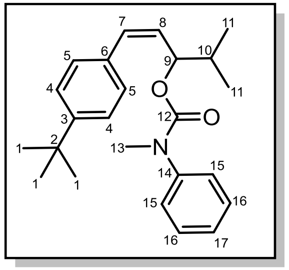

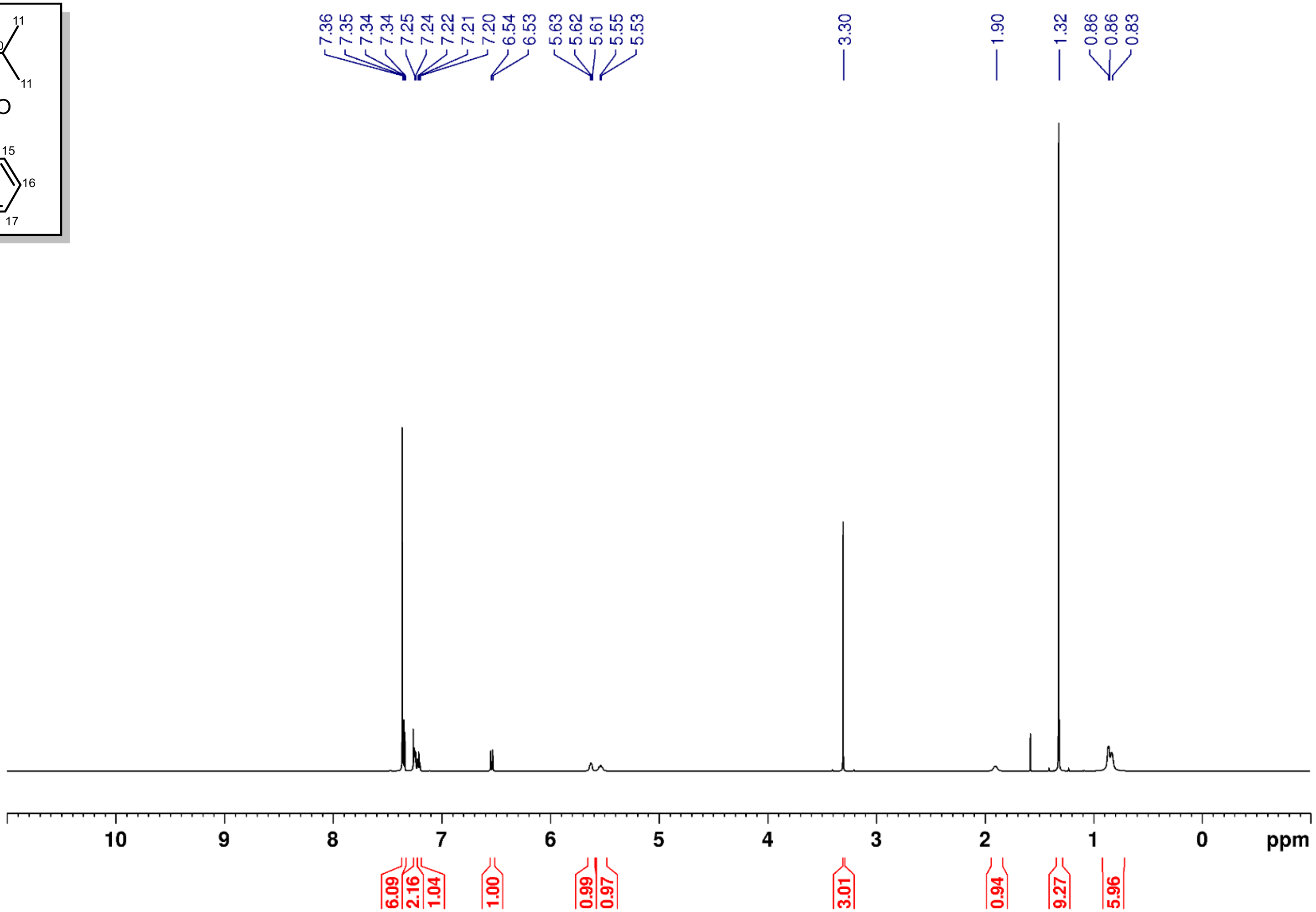

^{13}C NMR (176 MHz, CDCl$_3$) for (*Z*)-1-(4-(*tert*-butyl)phenyl)-4-methylpent-1-en-3-yl methyl(phenyl)carbamate ((***rac***)-**1u**))

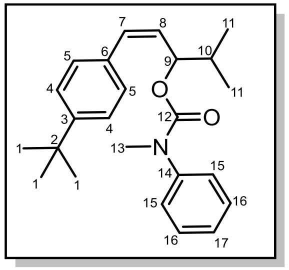

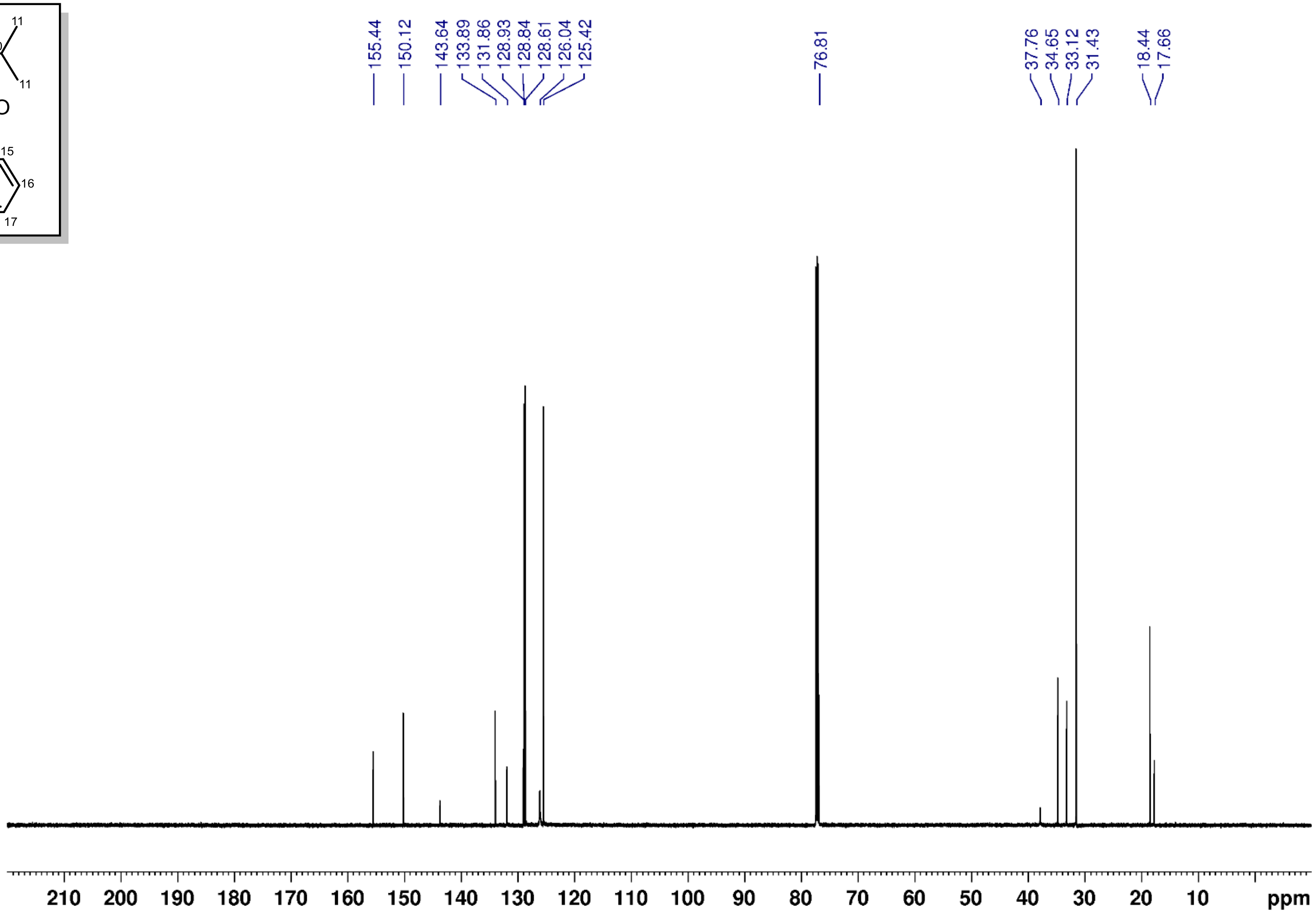

^{1}H NMR (400 MHz, $CDCl_3$) for 2-phenylallyl methyl(phenyl)carbamate (**15a**)

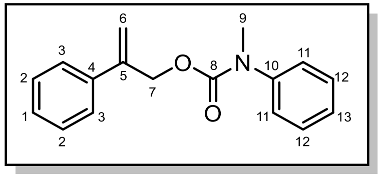

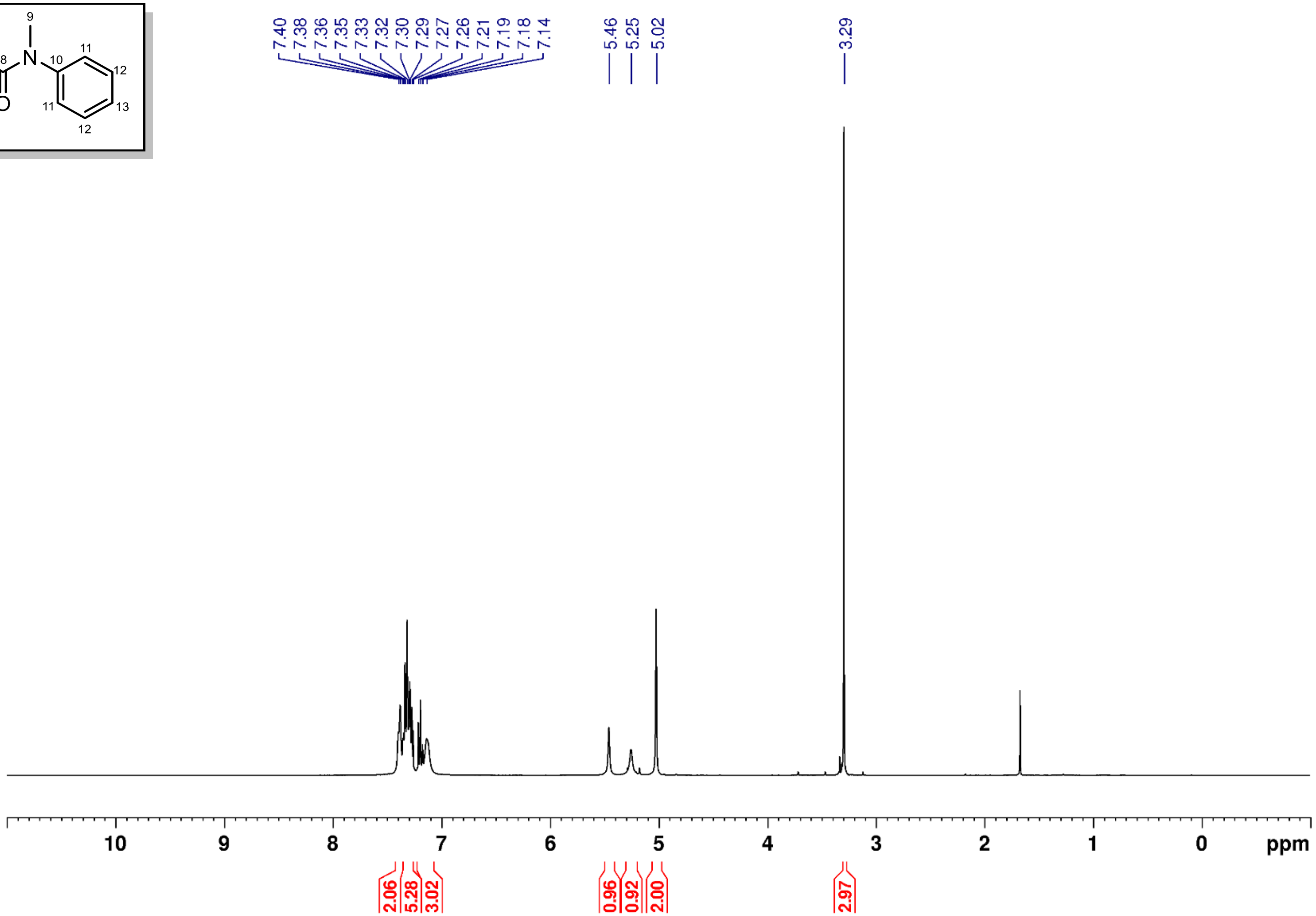

^{13}C NMR (101 MHz, CDCl$_3$) for 2-phenylallyl methyl(phenyl)carbamate (**15a**)

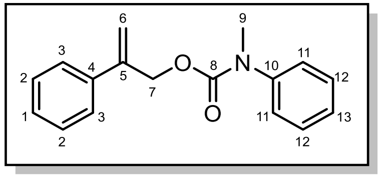

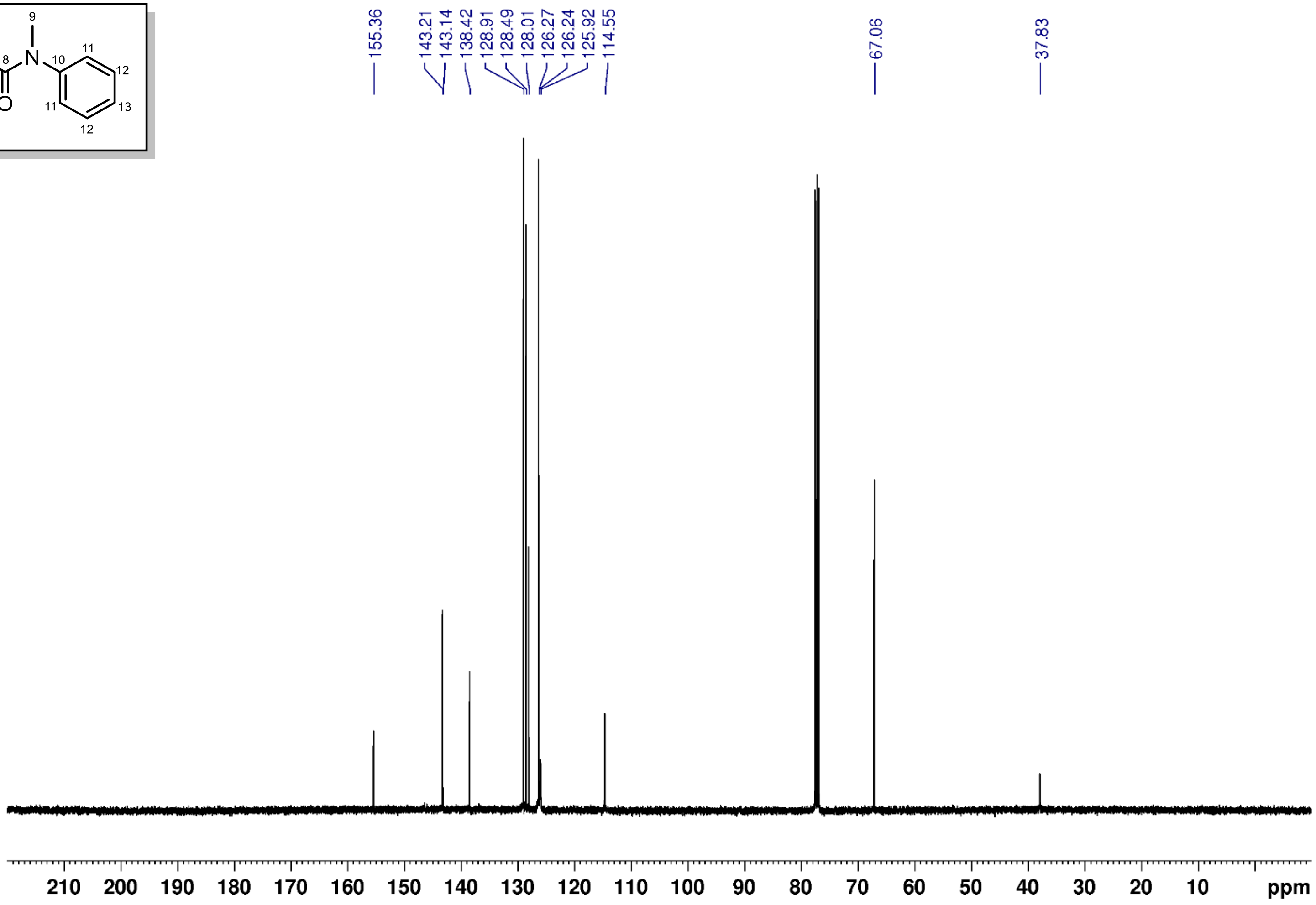

^{1}H NMR (400 MHz, $CDCl_3$) for 3-methylbut-2-en-1-yl methyl(phenyl)carbamate (**15b**)

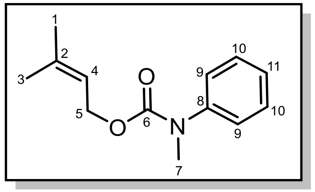

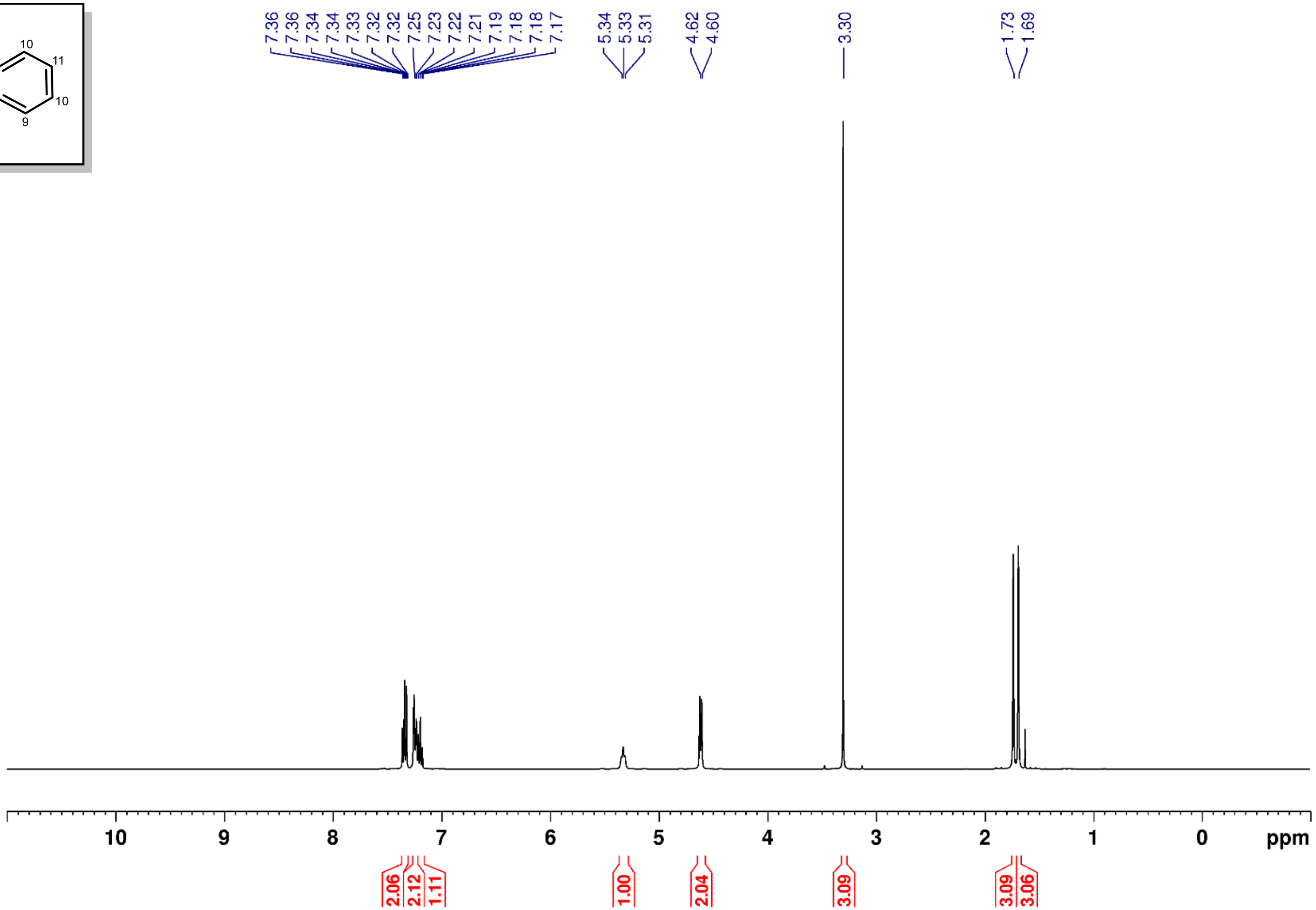

^{13}C NMR (101 MHz, $CDCl_3$) for 3-methylbut-2-en-1-yl methyl(phenyl)carbamate (**15b**)

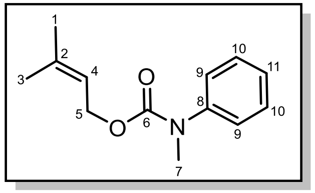

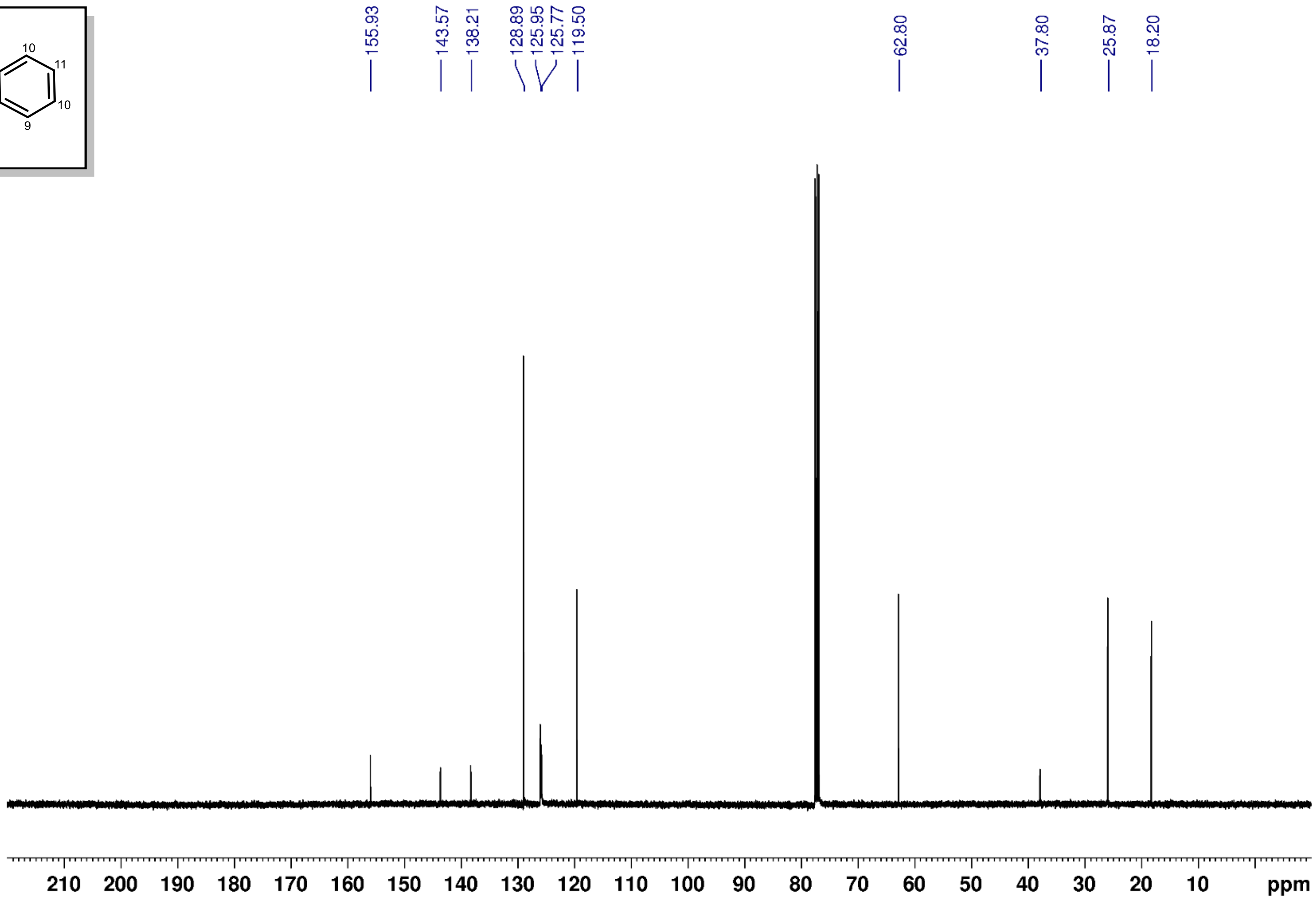

^{1}**H NMR (400 MHz, $CDCl_3$)** for (*Z*)-hex-2-en-1-yl methyl(phenyl)carbamate (**15c**)

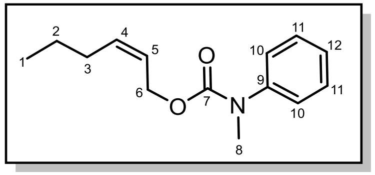

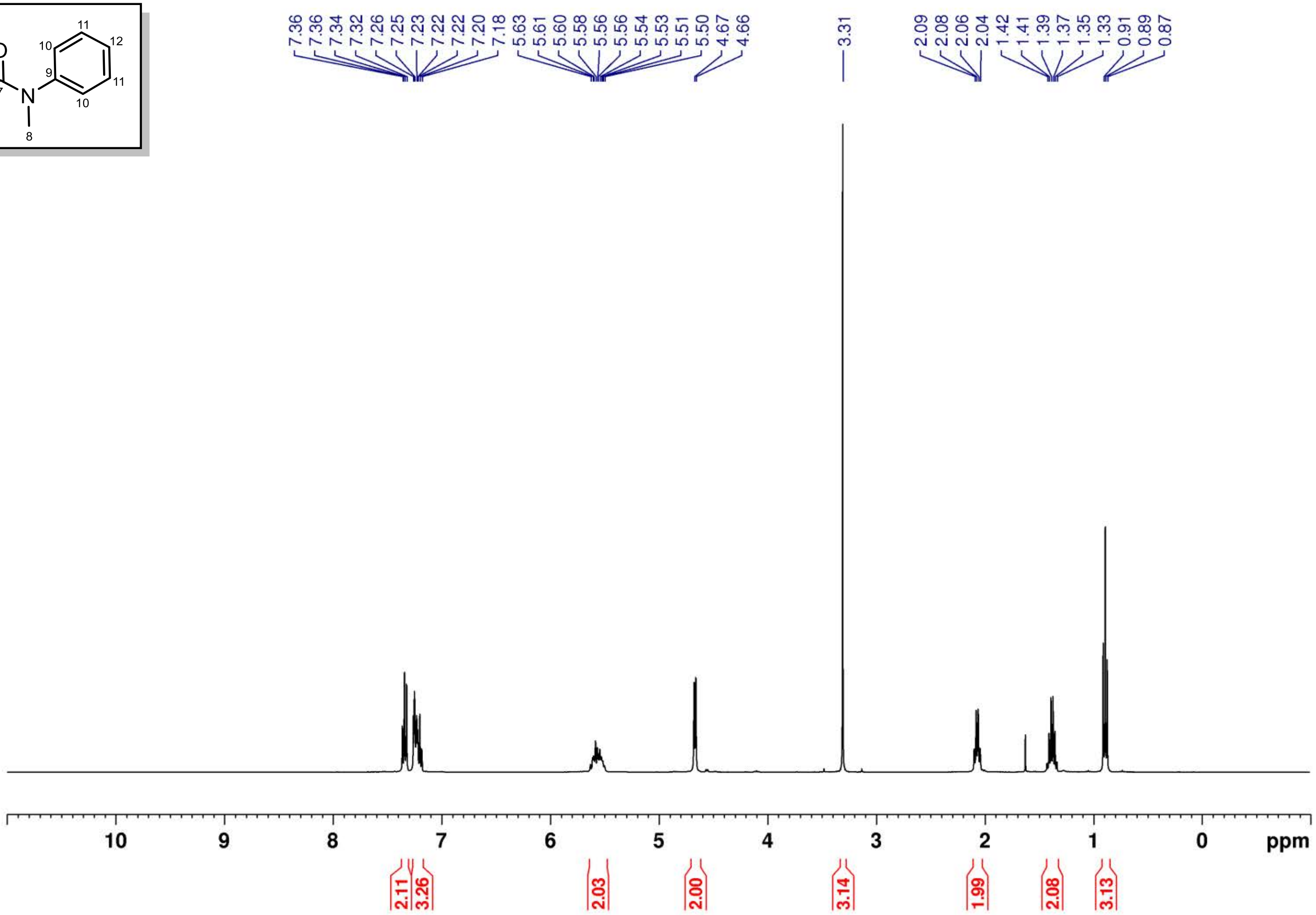

^{13}C NMR (101 MHz, $CDCl_3$) for (*Z*)-hex-2-en-1-yl methyl(phenyl)carbamate (**15c**)

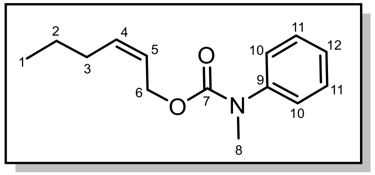

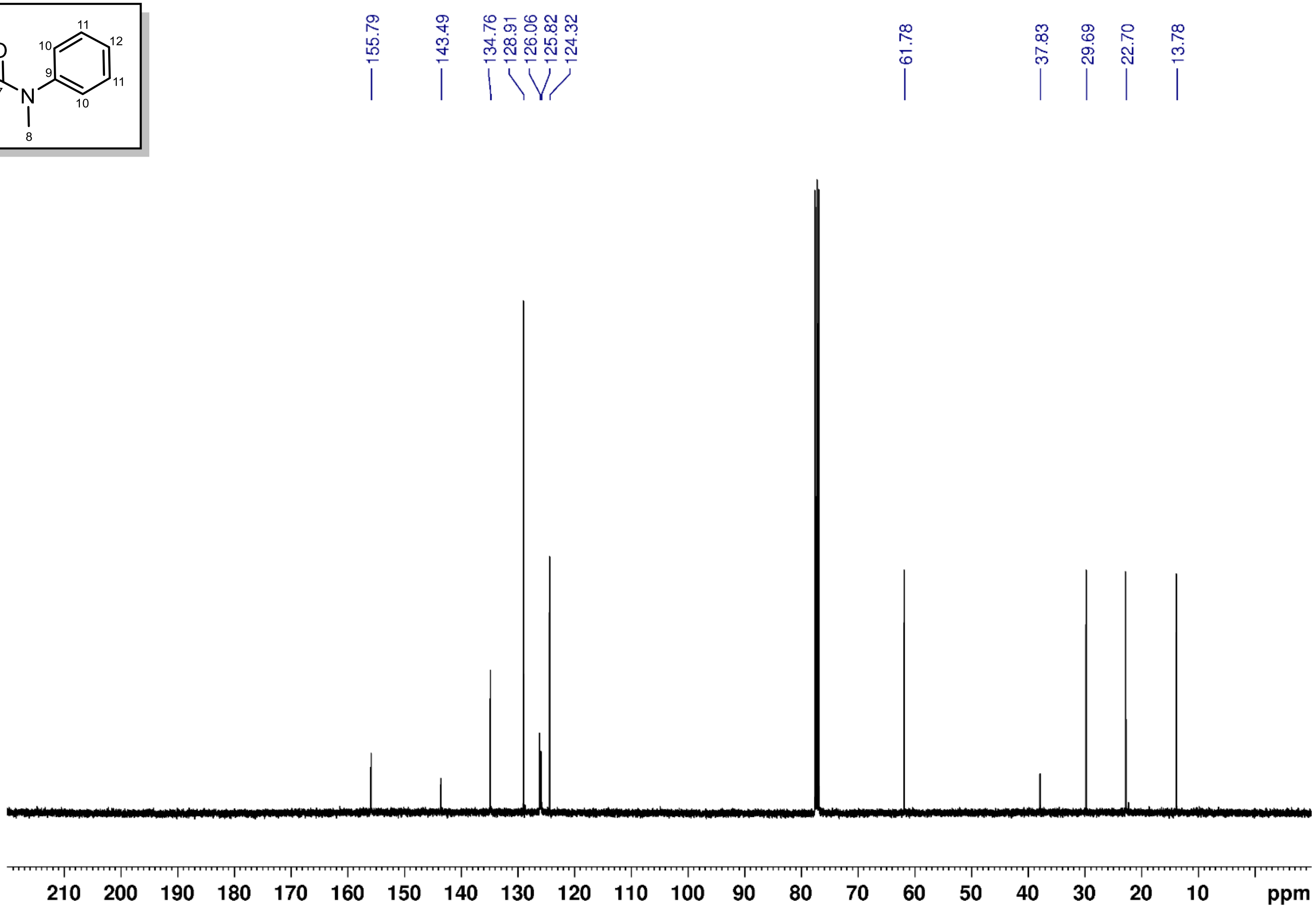

^{1}H NMR (400 MHz, $CDCl_3$) for cinnamyl methyl(phenyl)carbamate (**S2**)

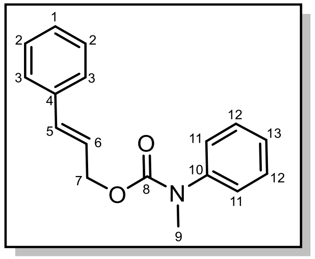

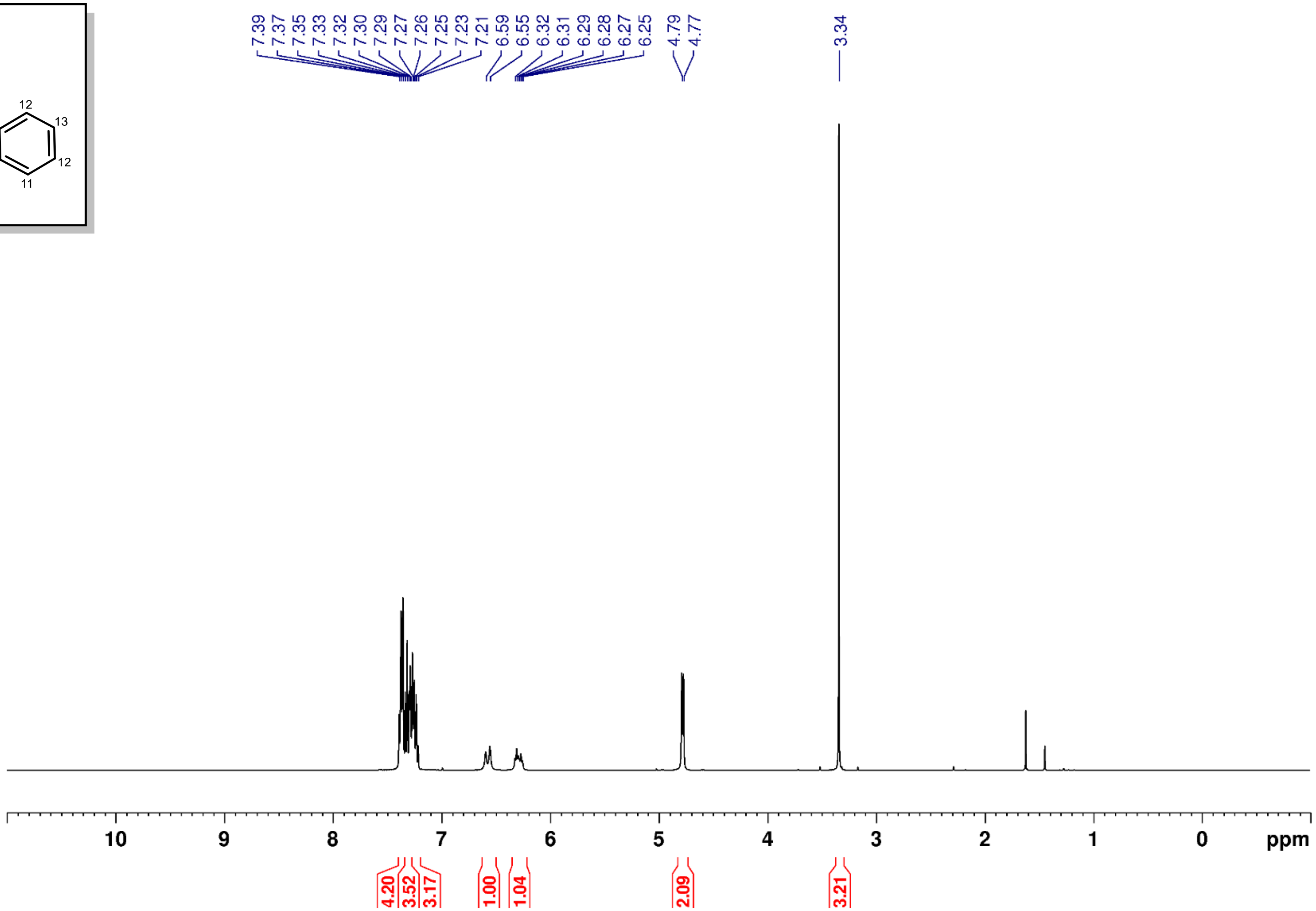

^{13}C NMR (101 MHz, $CDCl_3$) for cinnamyl methyl(phenyl)carbamate (**S2**)

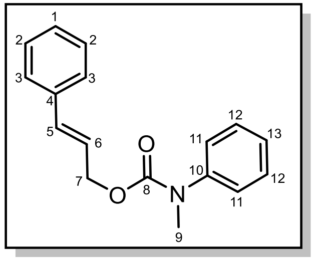

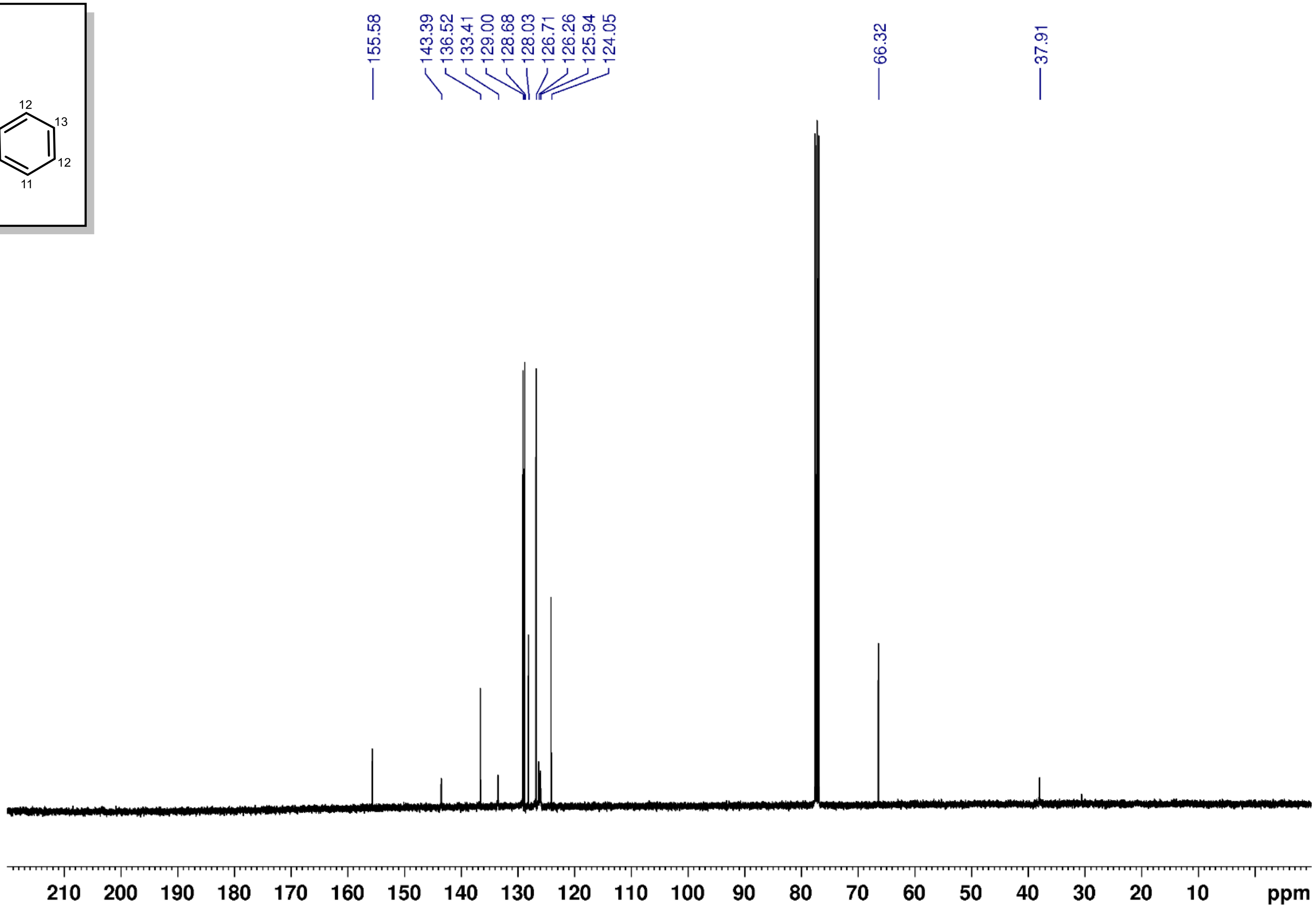

NMR Spectra for Substrates – N-Me Homoallylic Amide:

^{1}H NMR (400 MHz, CDCl$_3$) for *N*-methyl-*N*-phenylpent-4-ynamide

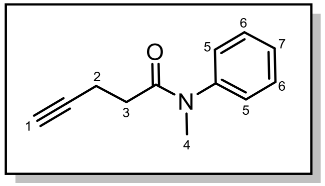

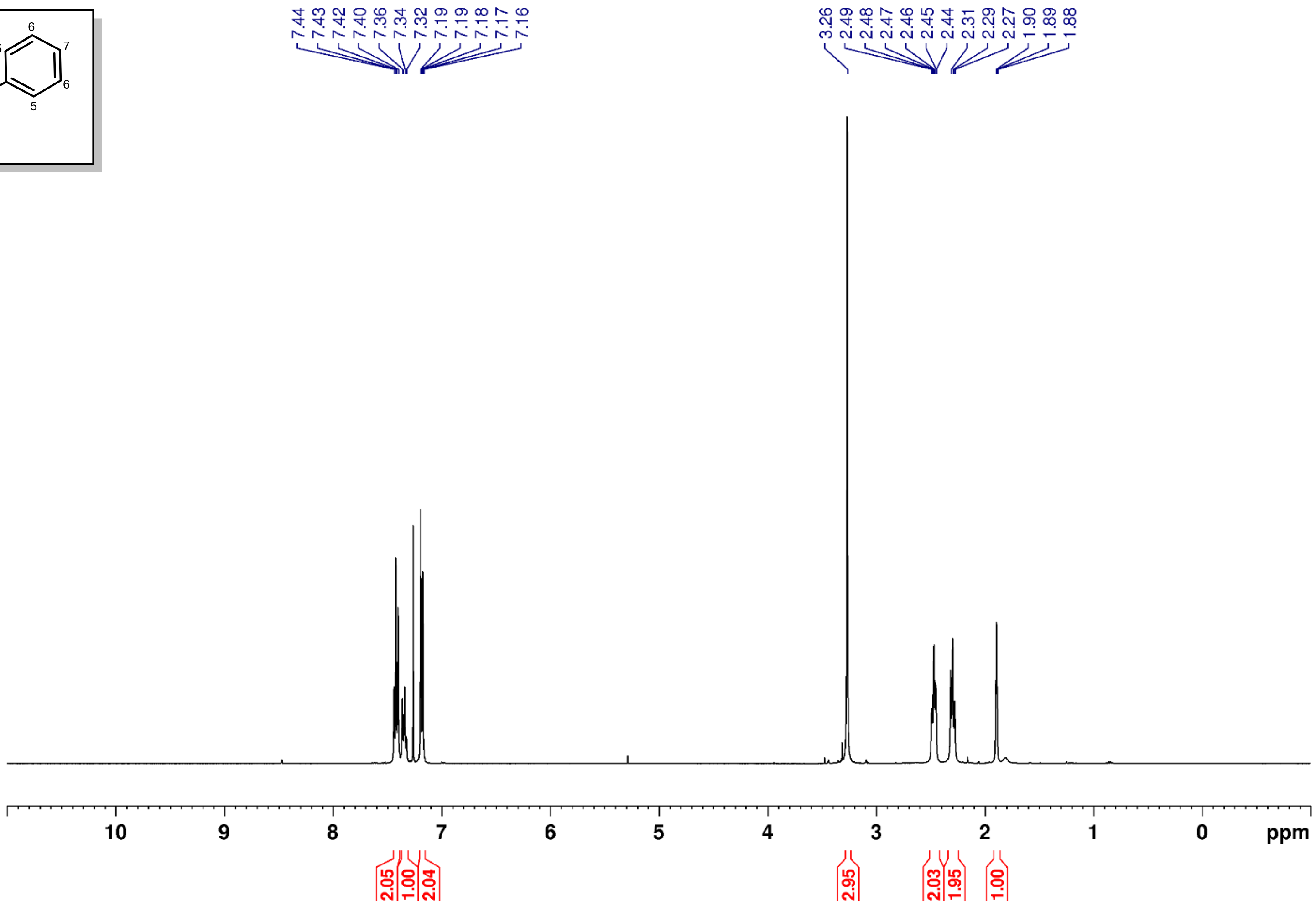

^{13}C NMR (101 MHz, $CDCl_3$) for *N*-methyl-*N*-phenylpent-4-ynamide

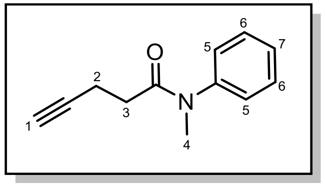

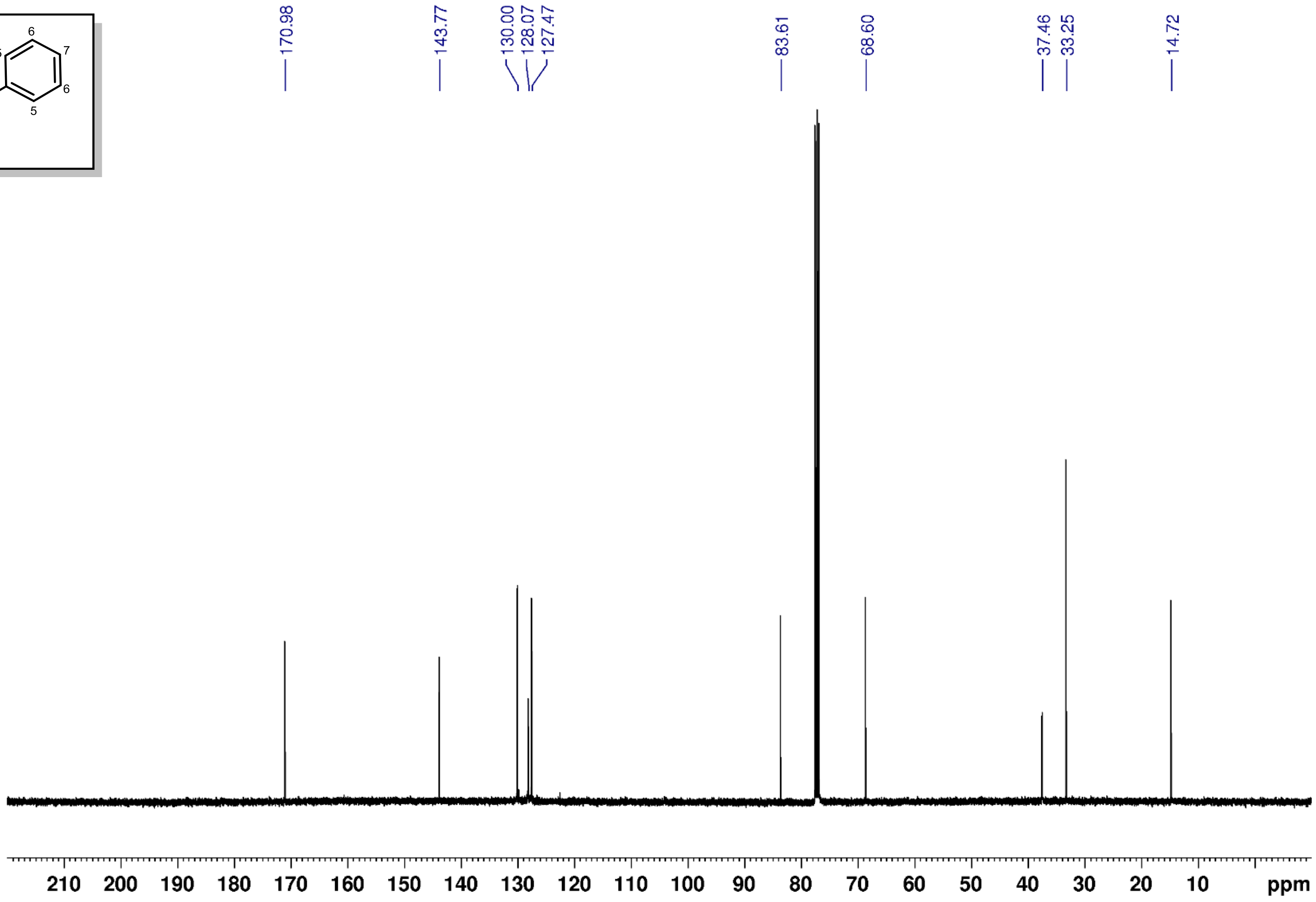

^{1}H NMR (400 MHz, CDCl$_3$) for *N*-methyl-*N*,5-diphenylpent-4-ynamide

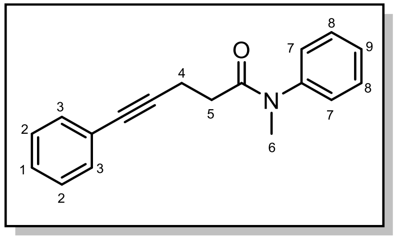

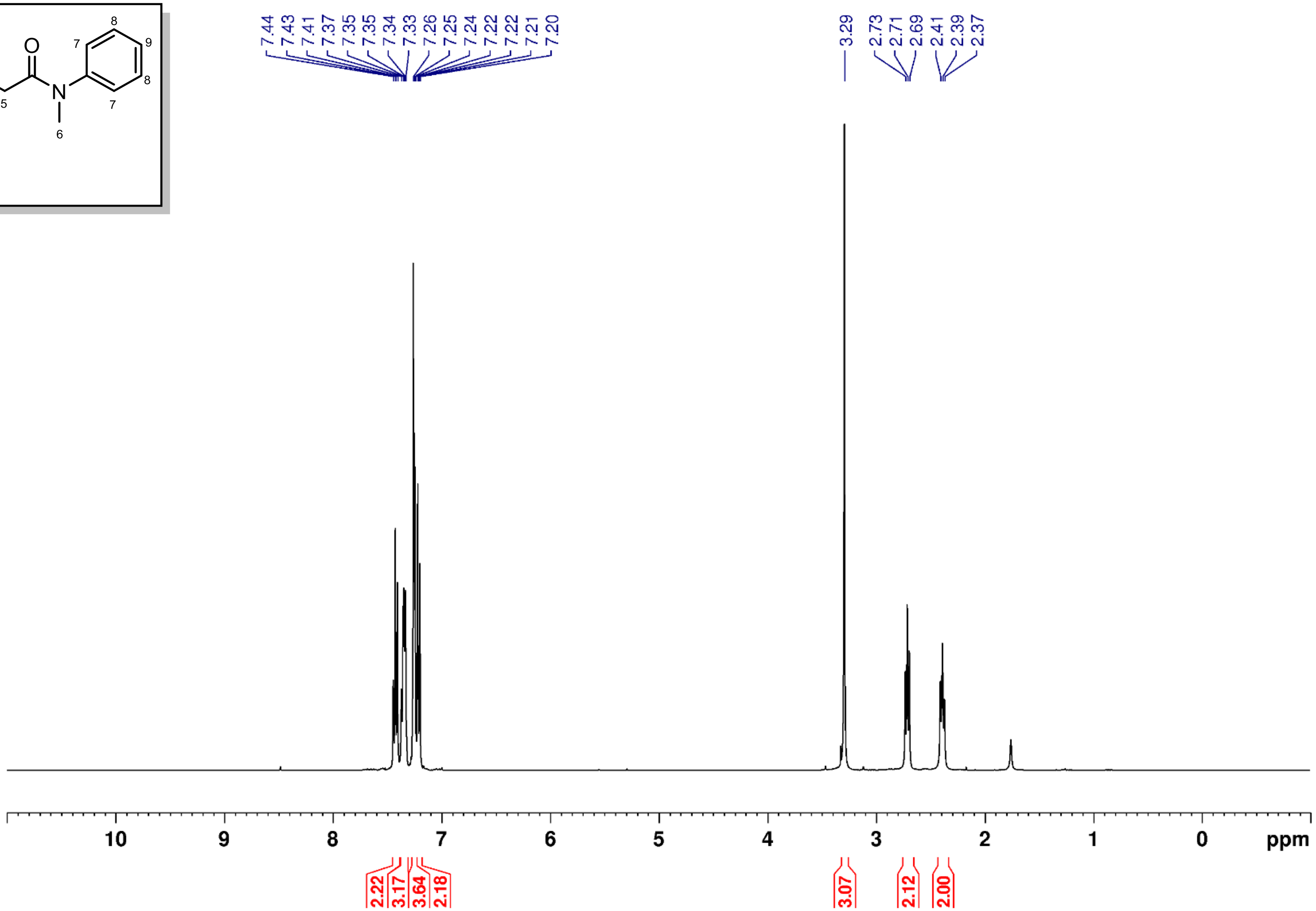

^{13}C NMR (101 MHz, CDCl$_3$) for *N*-methyl-*N*,5-diphenylpent-4-ynamide

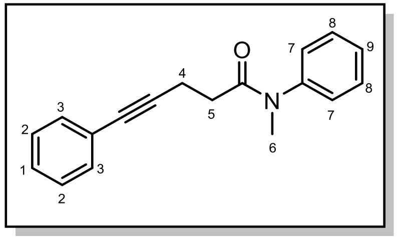

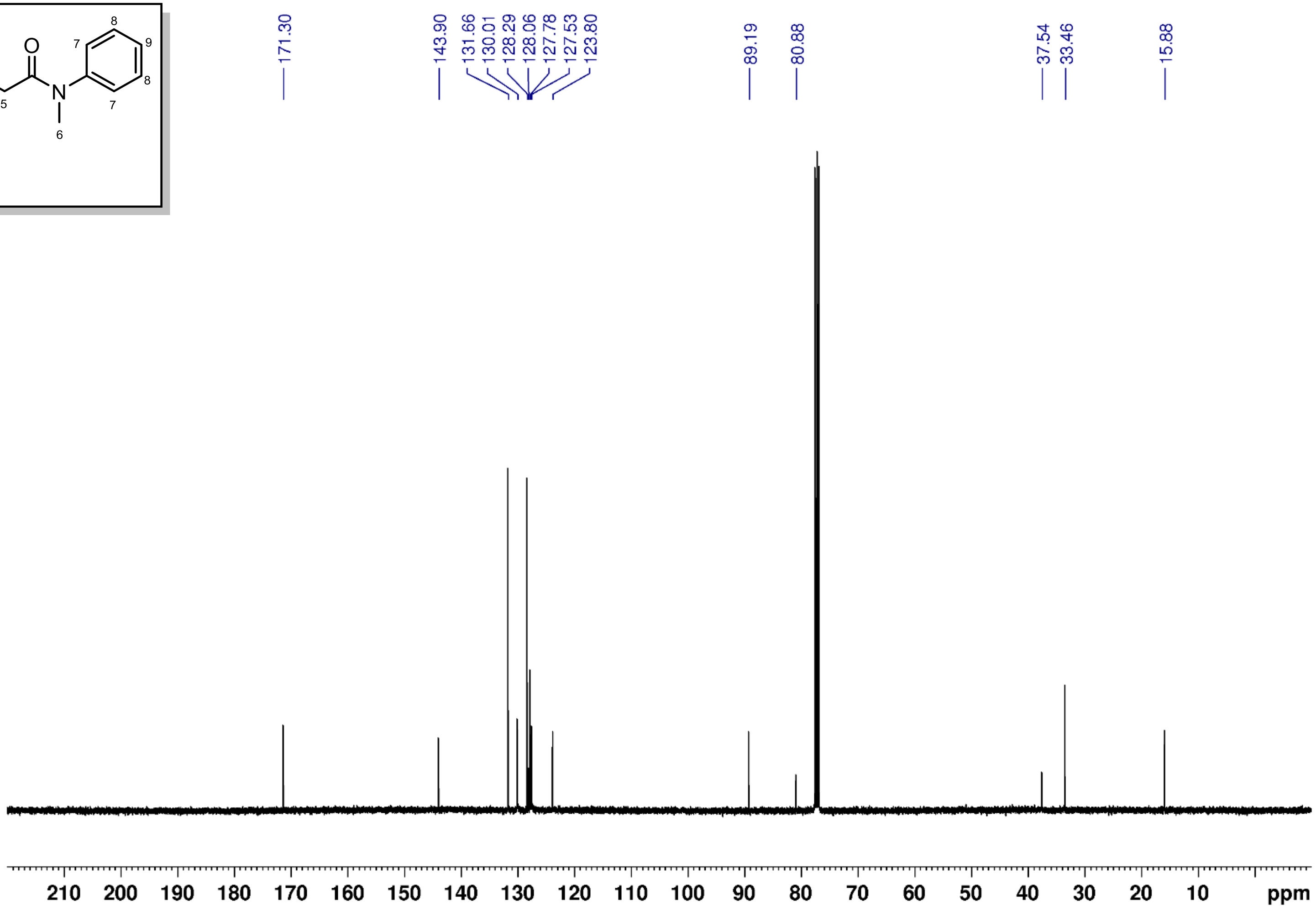

^{1}H NMR (700 MHz, CDCl$_3$) for (*Z*)-*N*-methyl-*N*,5-diphenylpent-4-enamide

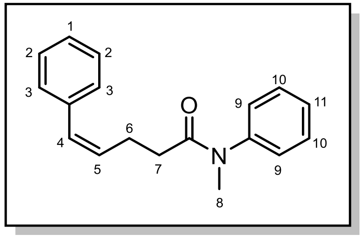

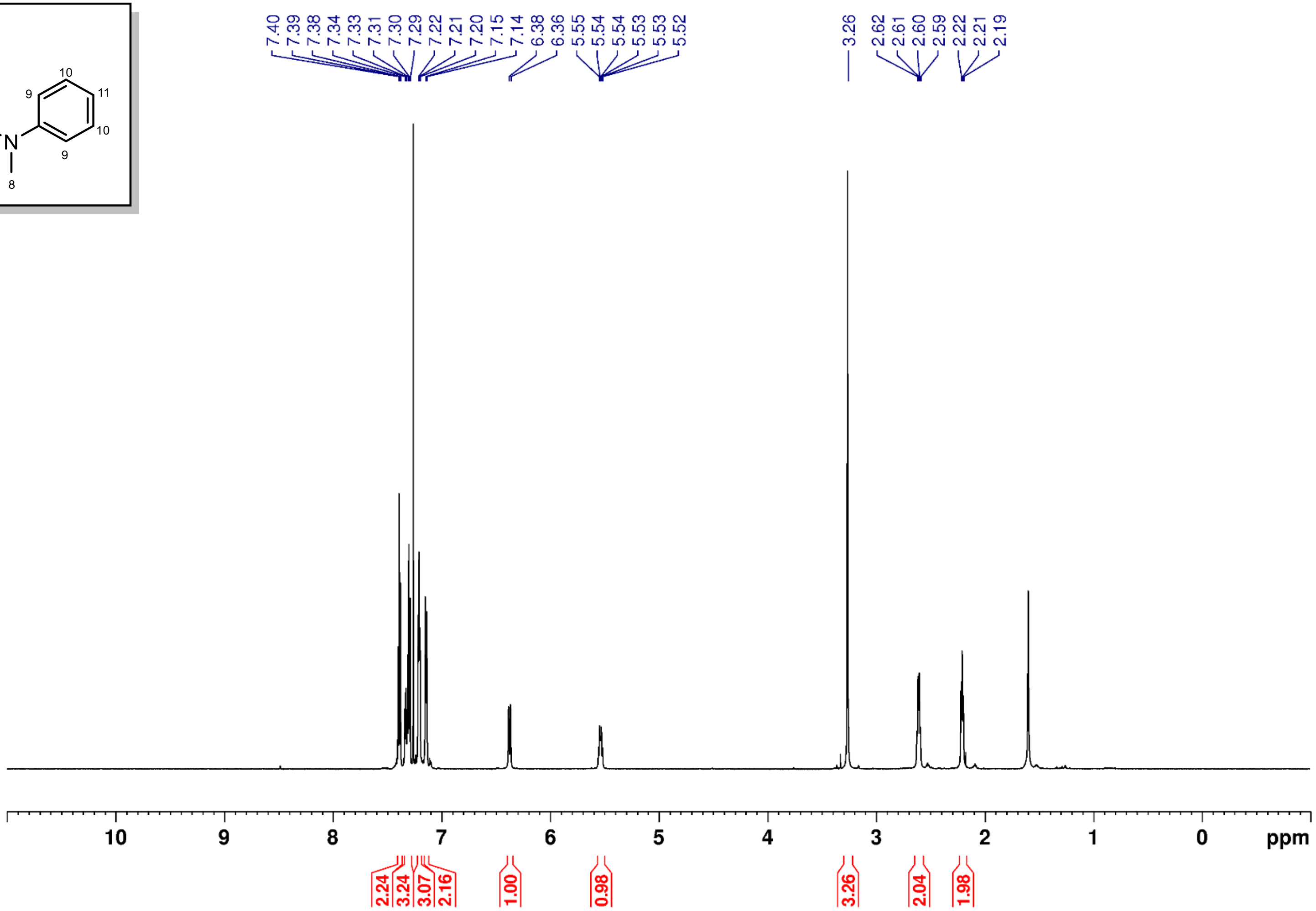

^{13}C NMR (176 MHz, CDCl$_3$) for (*Z*)-*N*-methyl-*N*,5-diphenylpent-4-enamide

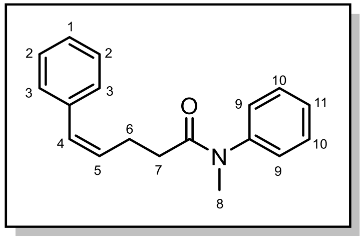

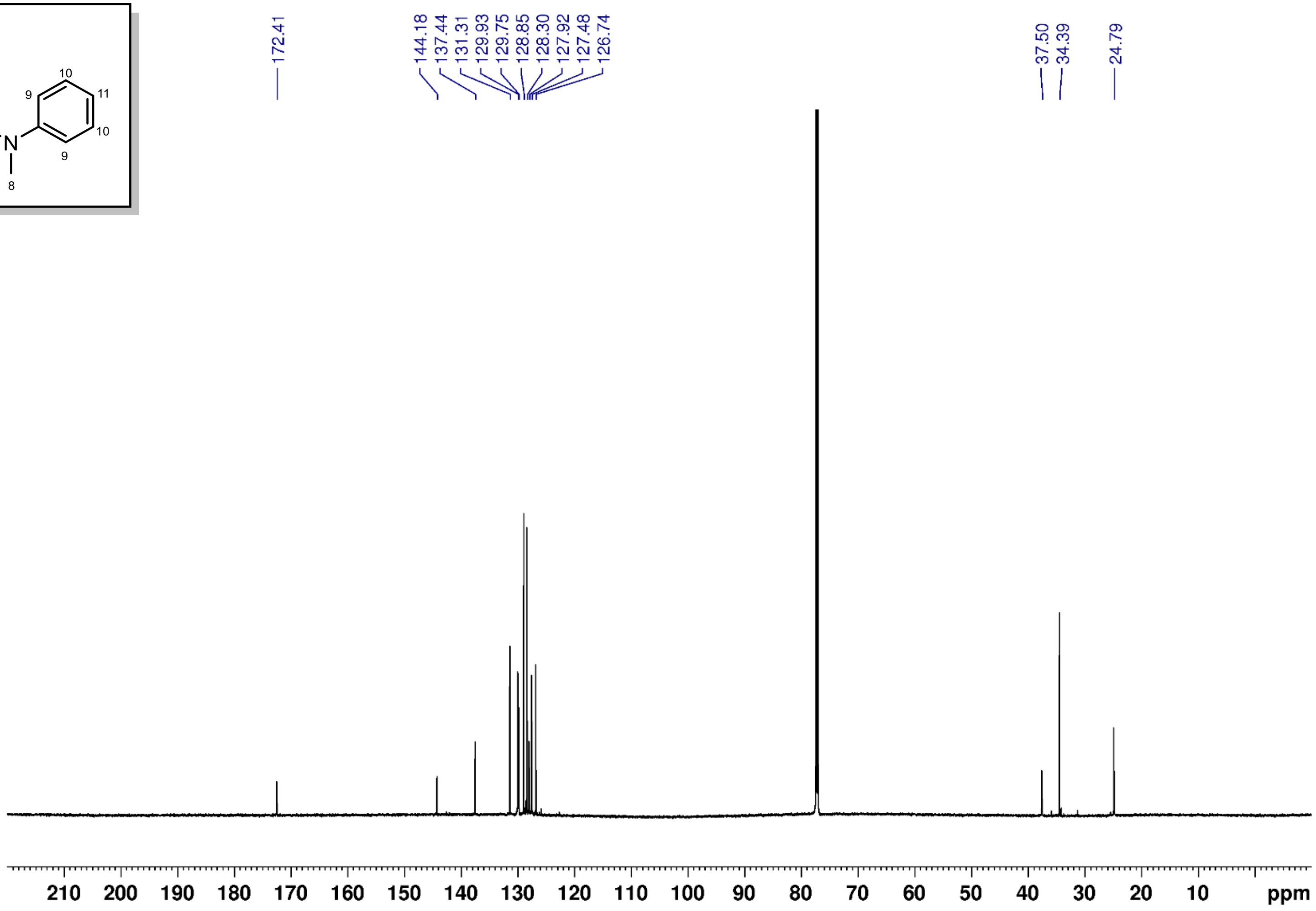

NMR Spectra for Enantioenriched Products – Aziridination:

^{1}H NMR (700 MHz, CD_3CN) for 2,2,3,3,4,4,4-heptafluorobutyl ((*S*)-((*R*)-2-oxo-1,3-dioxolan-4-yl)(phenyl)methyl)sulfamate (**4a**)

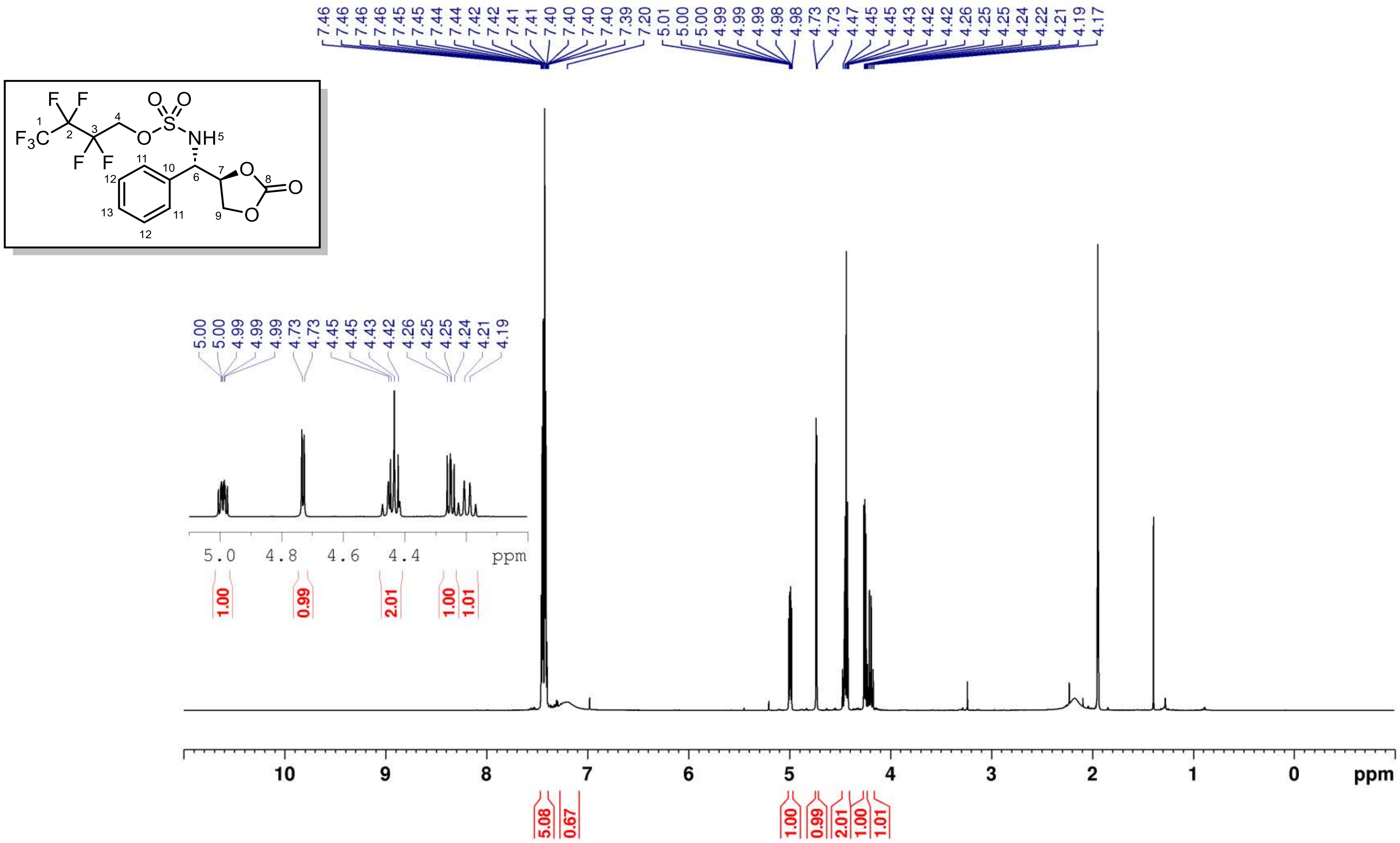

^{13}C NMR (176 MHz, CD_3CN) for 2,2,3,3,4,4,4-heptafluorobutyl ((*S*)-((*R*)-2-oxo-1,3-dioxolan-4-yl)(phenyl)methyl)sulfamate (**4a**)

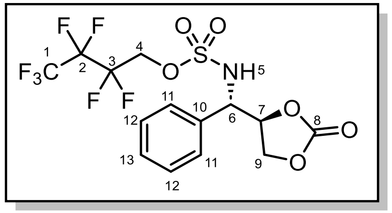

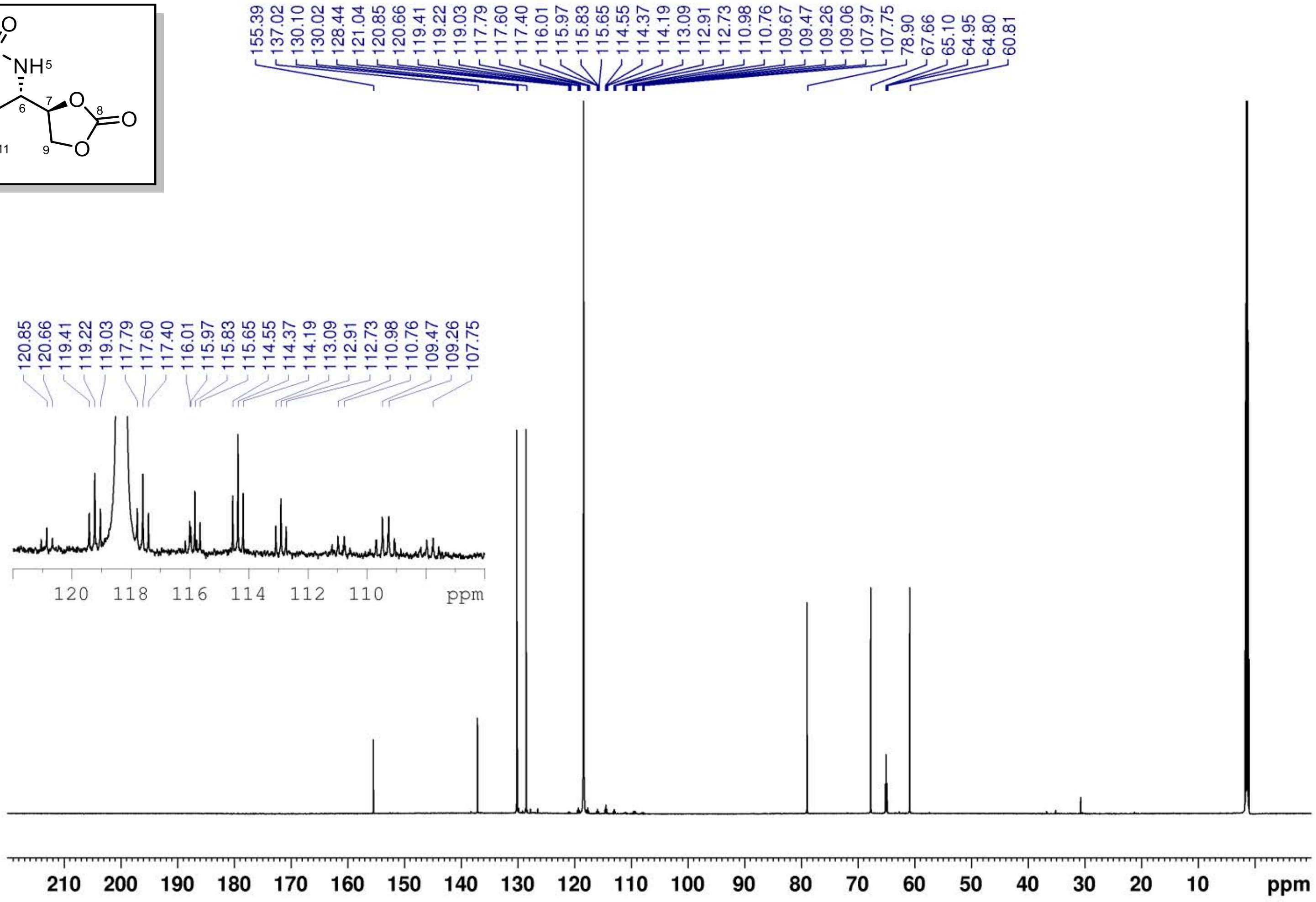

^{19}F NMR (376 MHz, CD_3CN) for 2,2,3,3,4,4,4-heptafluorobutyl ((*S*)-((*R*)-2-oxo-1,3-dioxolan-4-yl)(phenyl)methyl)sulfamate (**4a**)

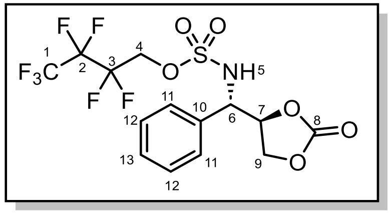

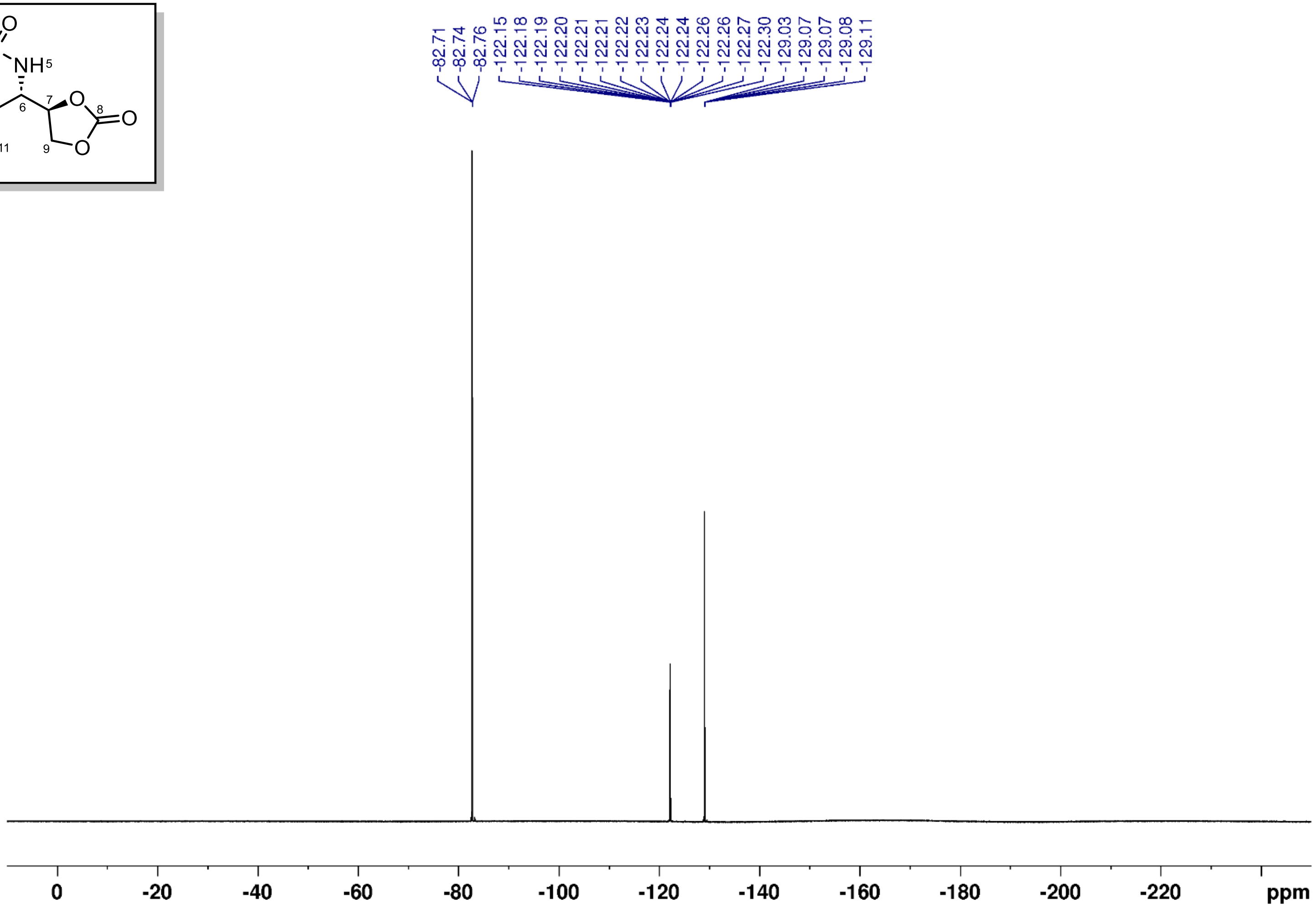

^{1}H NMR (400 MHz, CD$_3$CN) for 2,2,3,3,4,4,4-heptafluorobutyl ((*R*)-((*S*)-2-oxo-1,3-dioxolan-4-yl)(phenyl)methyl)sulfamate (***ent*-4a**)

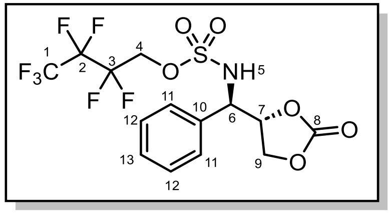

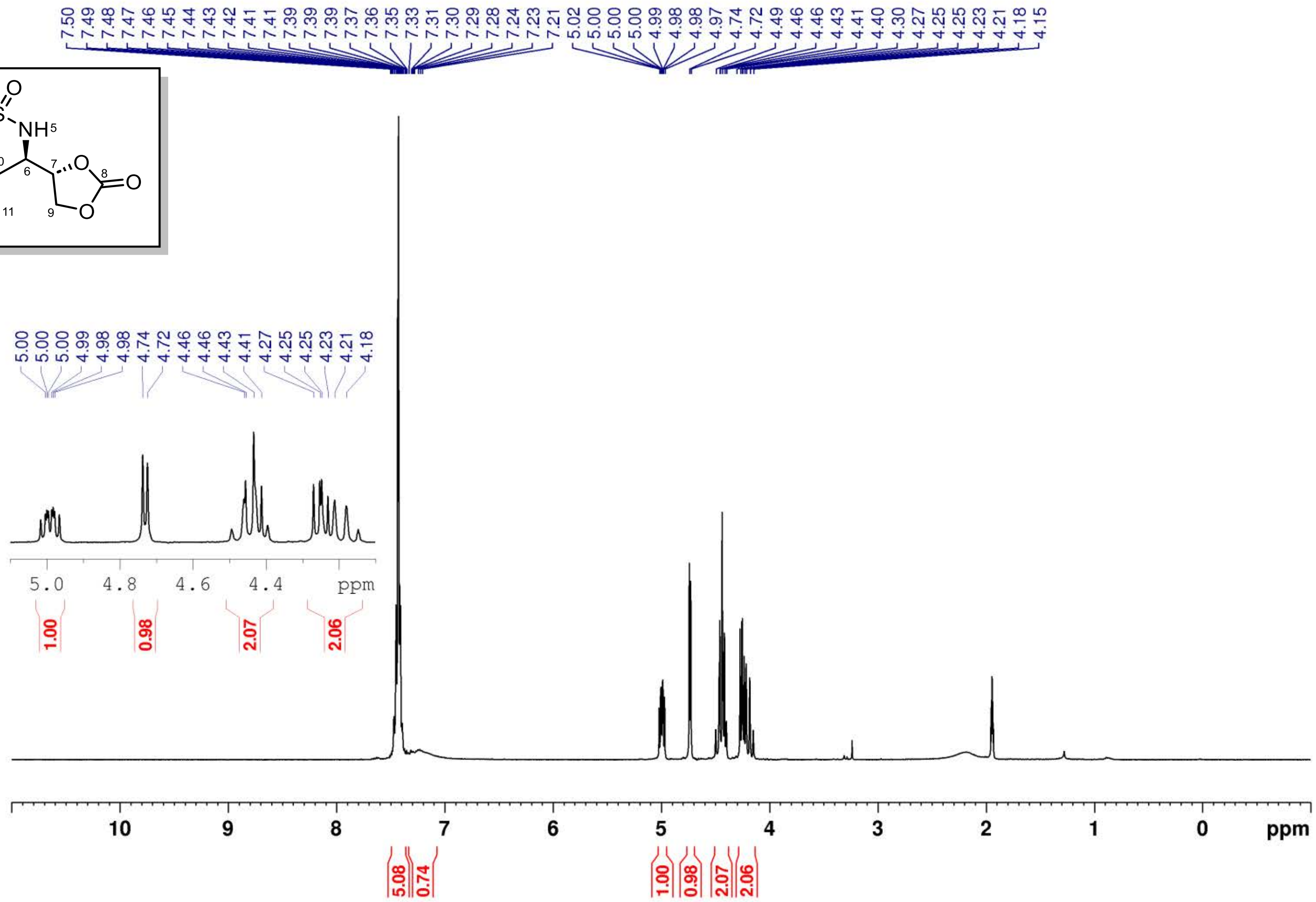

^{1}H NMR (500 MHz, CDCl$_3$) for 2,2,3,3,4,4,4-heptafluorobutyl ((*S*)-(2-isopropylphenyl)((*R*)-2-oxo-1,3-dioxolan-4-yl)methyl)sulfamate (**4b**)

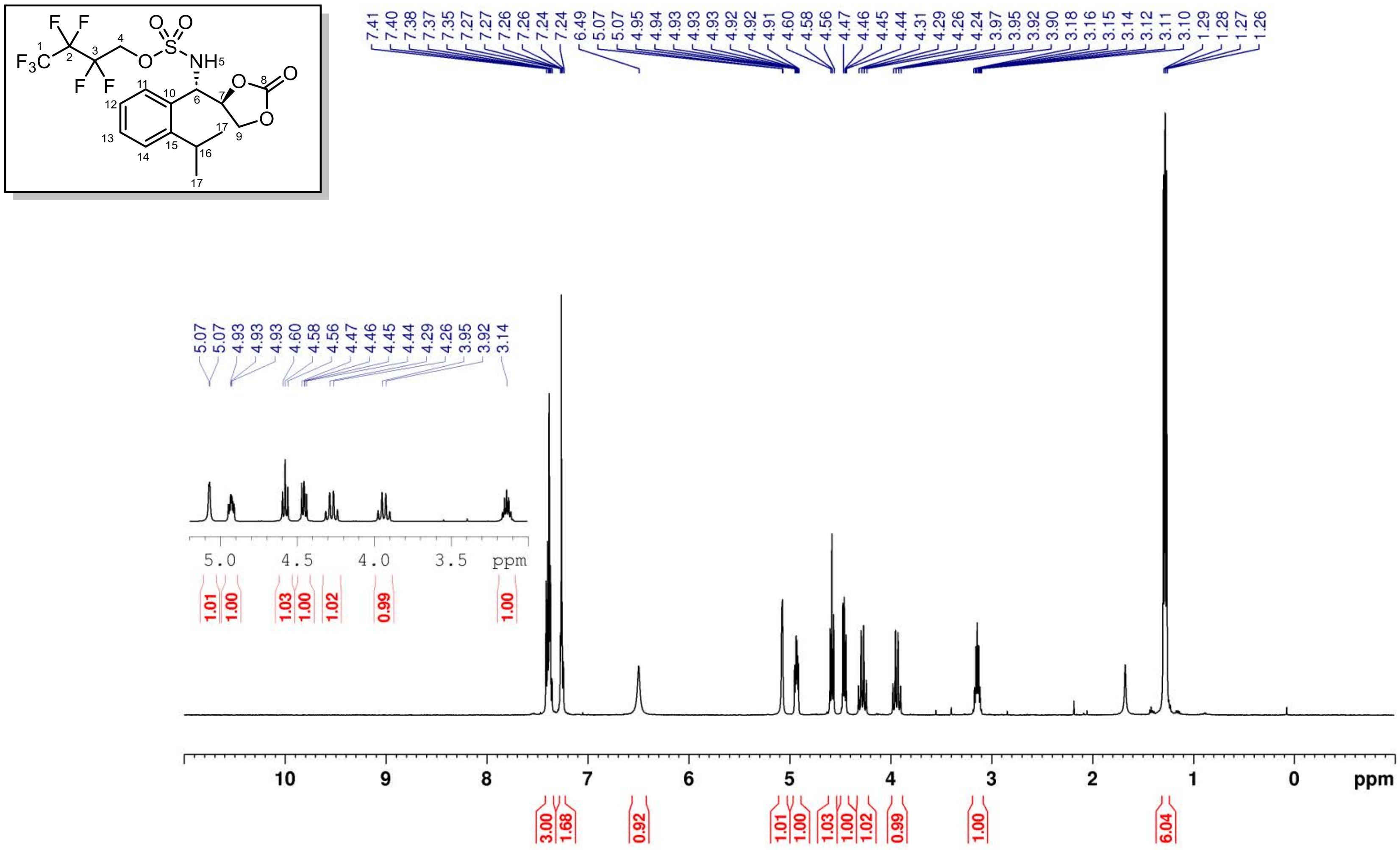

^{13}C NMR (126 MHz, CDCl$_3$) for 2,2,3,3,4,4,4-heptafluorobutyl ((*S*)-(2-isopropylphenyl)((*R*)-2-oxo-1,3-dioxolan-4-yl)methyl)sulfamate (**4b**)

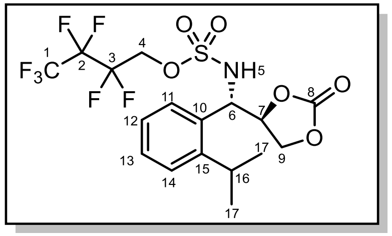

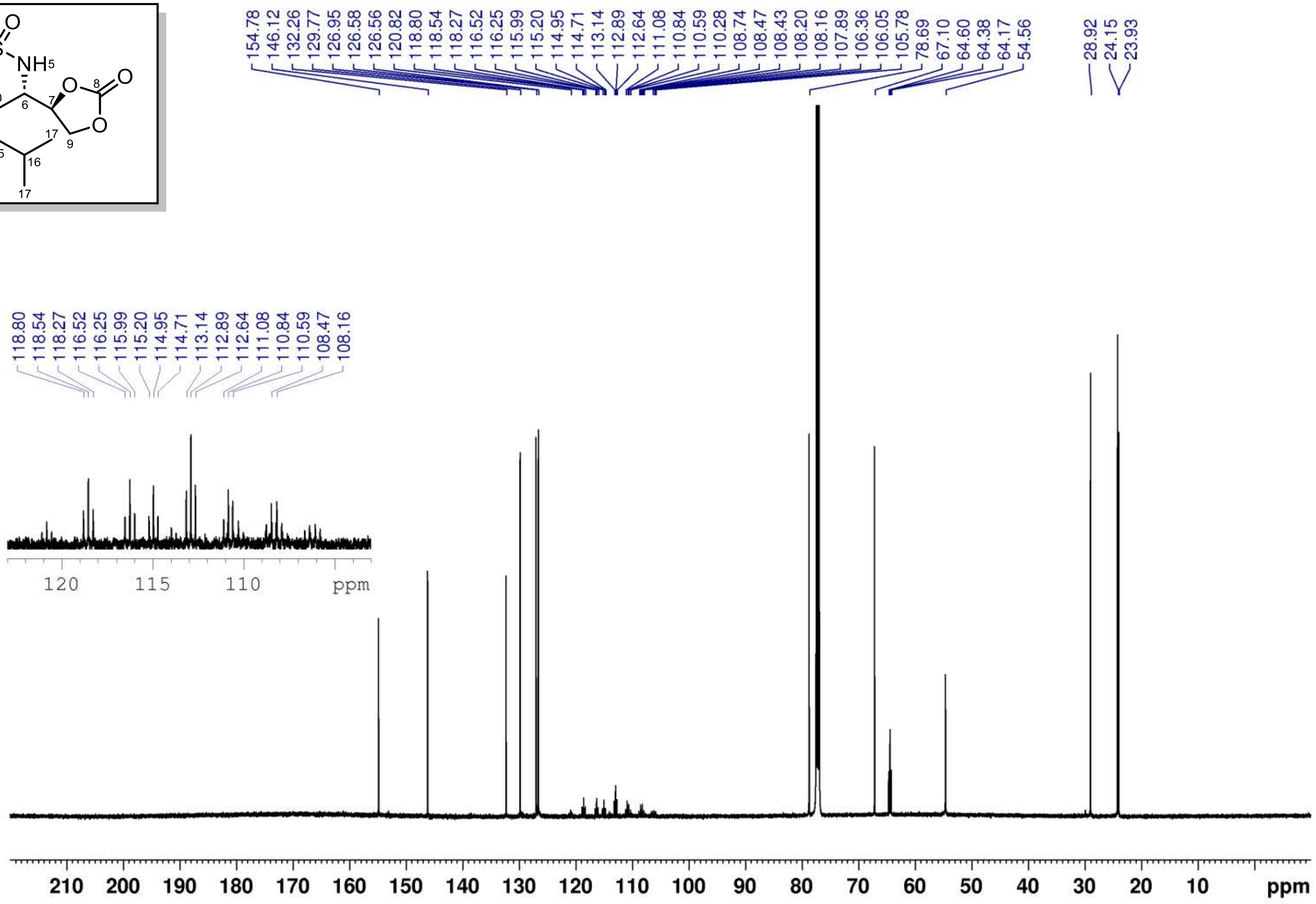

^{19}F NMR (376 MHz, $CDCl_3$) for 2,2,3,3,4,4,4-heptafluorobutyl ((*S*)-(2-isopropylphenyl)((*R*)-2-oxo-1,3-dioxolan-4-yl)methyl)sulfamate (**4b**)

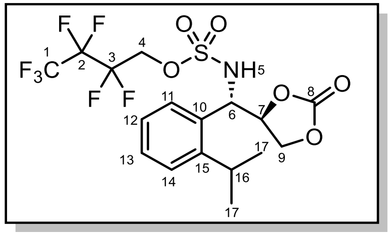

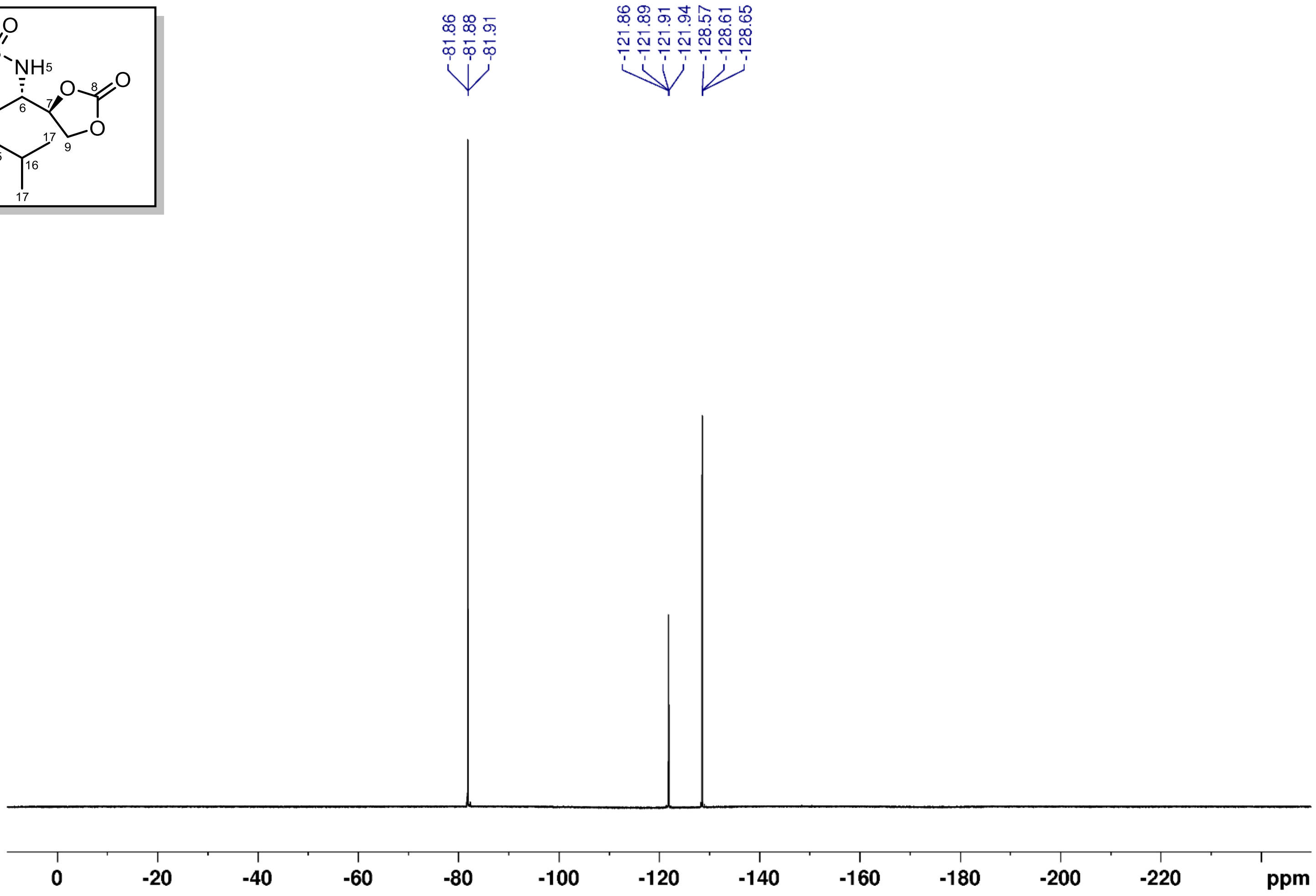

^{1}H NMR (700 MHz, $CDCl_3$) for 2,2,3,3,4,4,4-heptafluorobutyl ((*S*)-(2-methoxyphenyl)((*R*)-2-oxo-1,3-dioxolan-4-yl)methyl)sulfamate (**4c**)

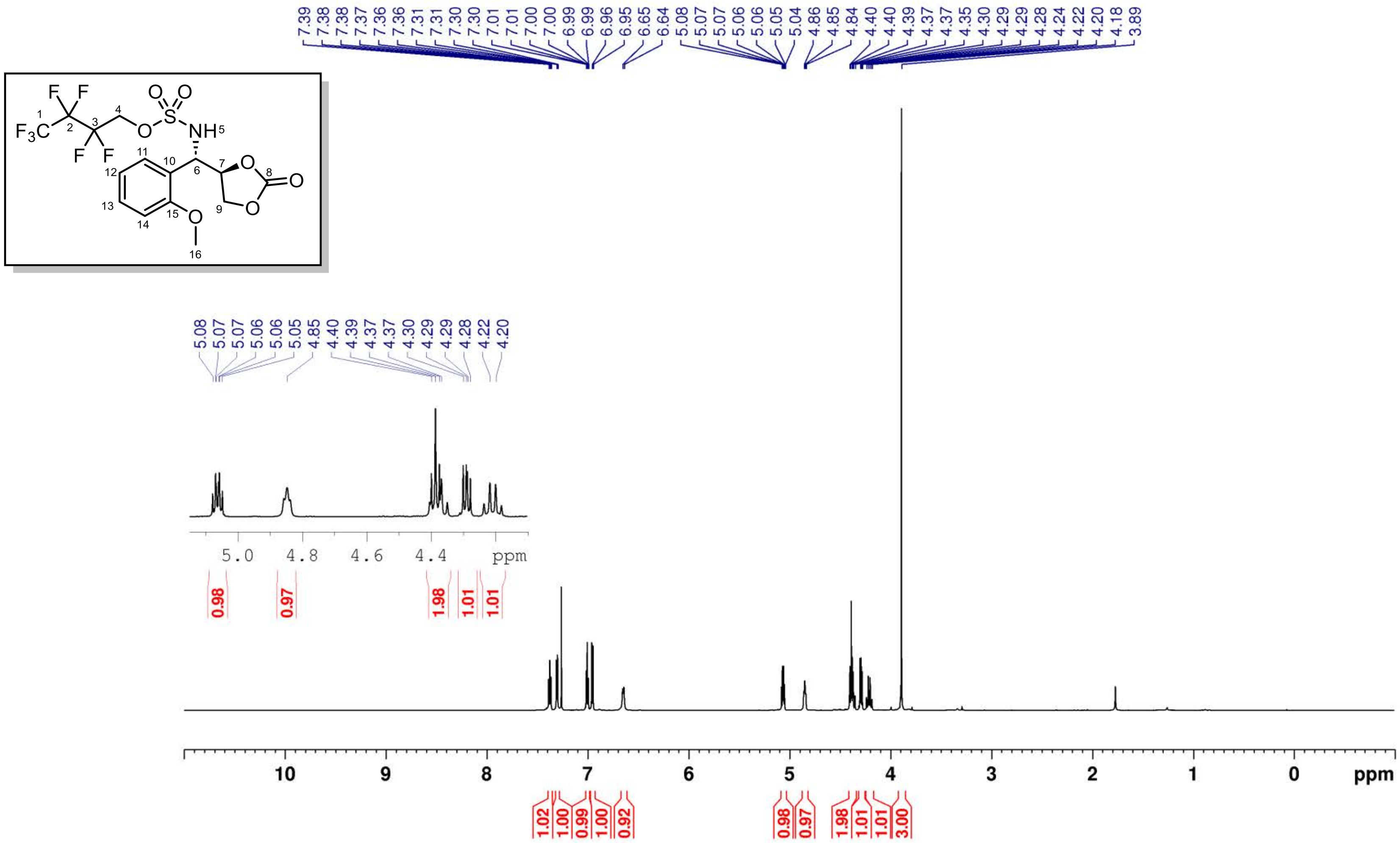

^{13}C NMR (176 MHz, CDCl$_3$) for 2,2,3,3,4,4,4-heptafluorobutyl ((*S*)-(2-methoxyphenyl)((*R*)-2-oxo-1,3-dioxolan-4-yl)methyl)sulfamate (**4c**)

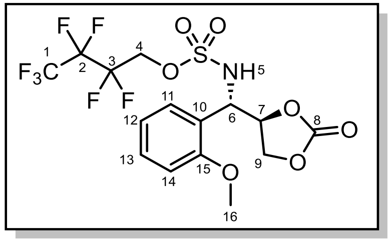

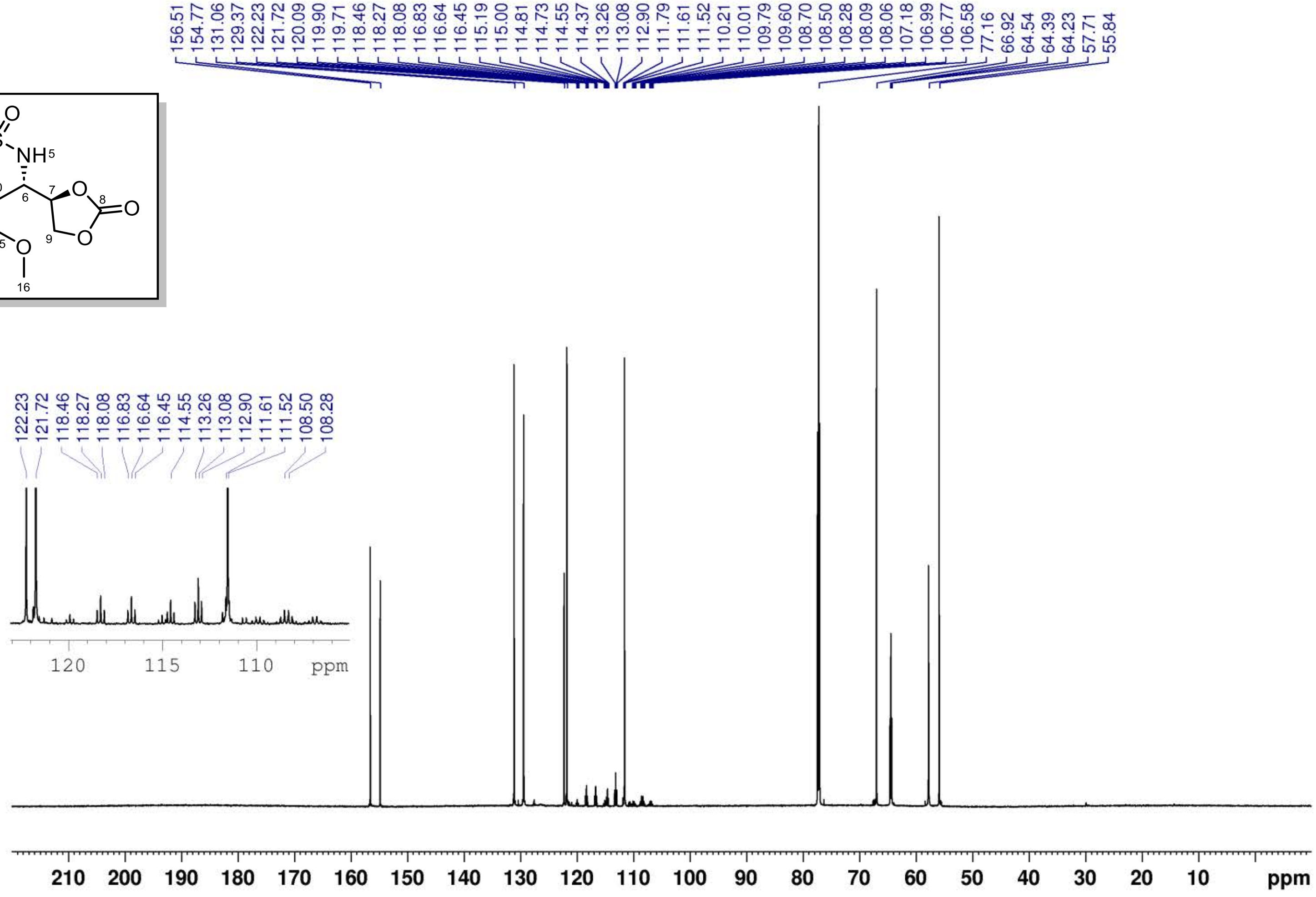

^{19}F NMR (471 MHz, $CDCl_3$) for 2,2,3,3,4,4,4-heptafluorobutyl ((*S*)-(2-methoxyphenyl)((*R*)-2-oxo-1,3-dioxolan-4-yl)methyl)sulfamate (**4c**)

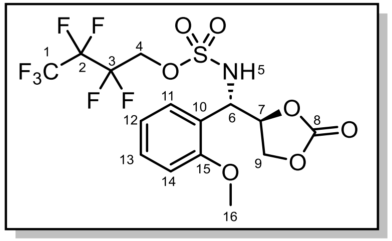

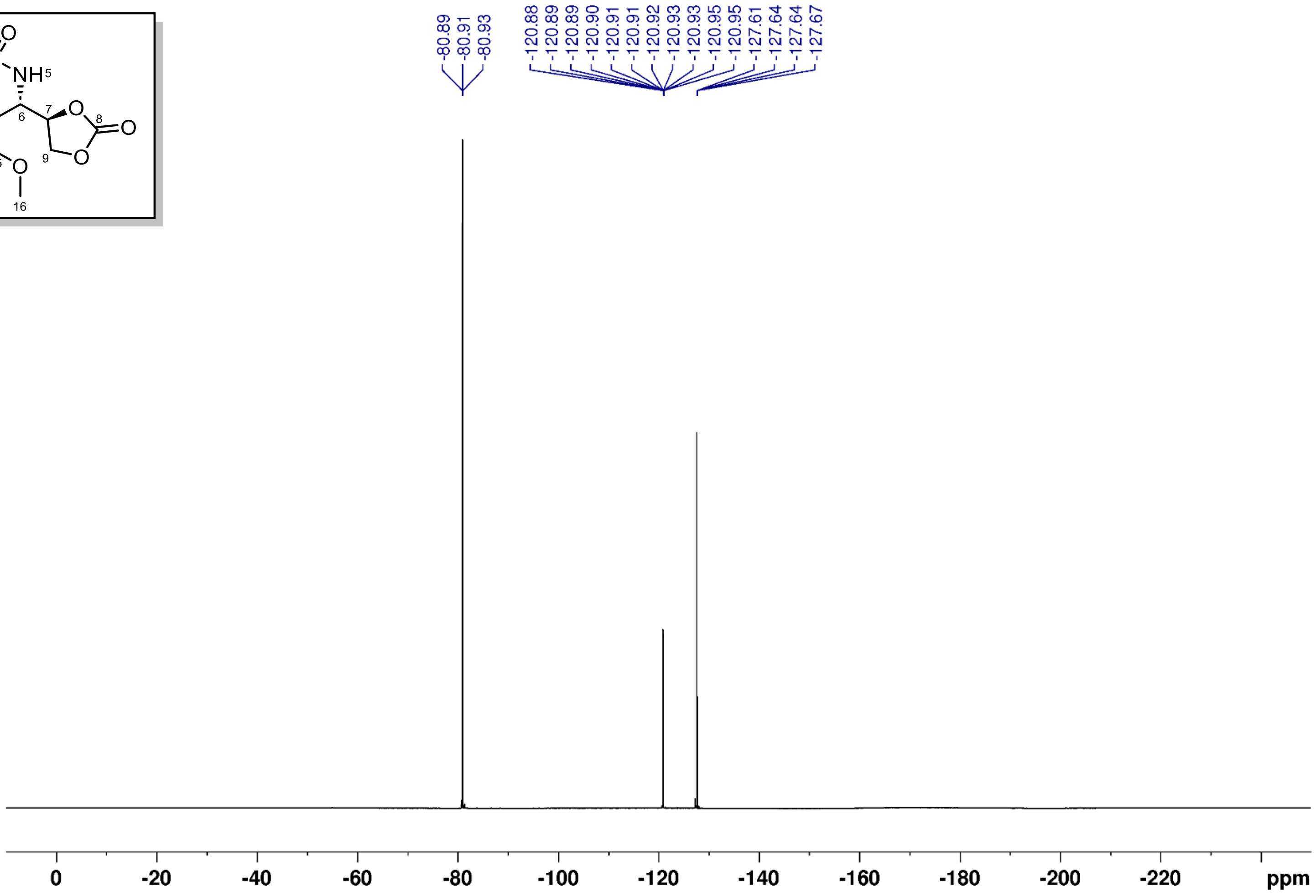

¹H NMR (700 MHz, $CDCl_3$) for 2,2,3,3,4,4,4-heptafluorobutyl ((*S*)-(2-fluorophenyl)((*R*)-2-oxo-1,3-dioxolan-4-yl)methyl)sulfamate (**4d**)

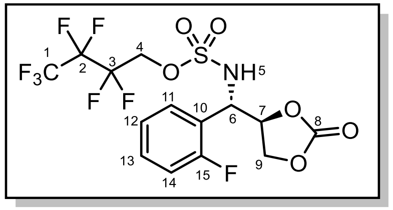

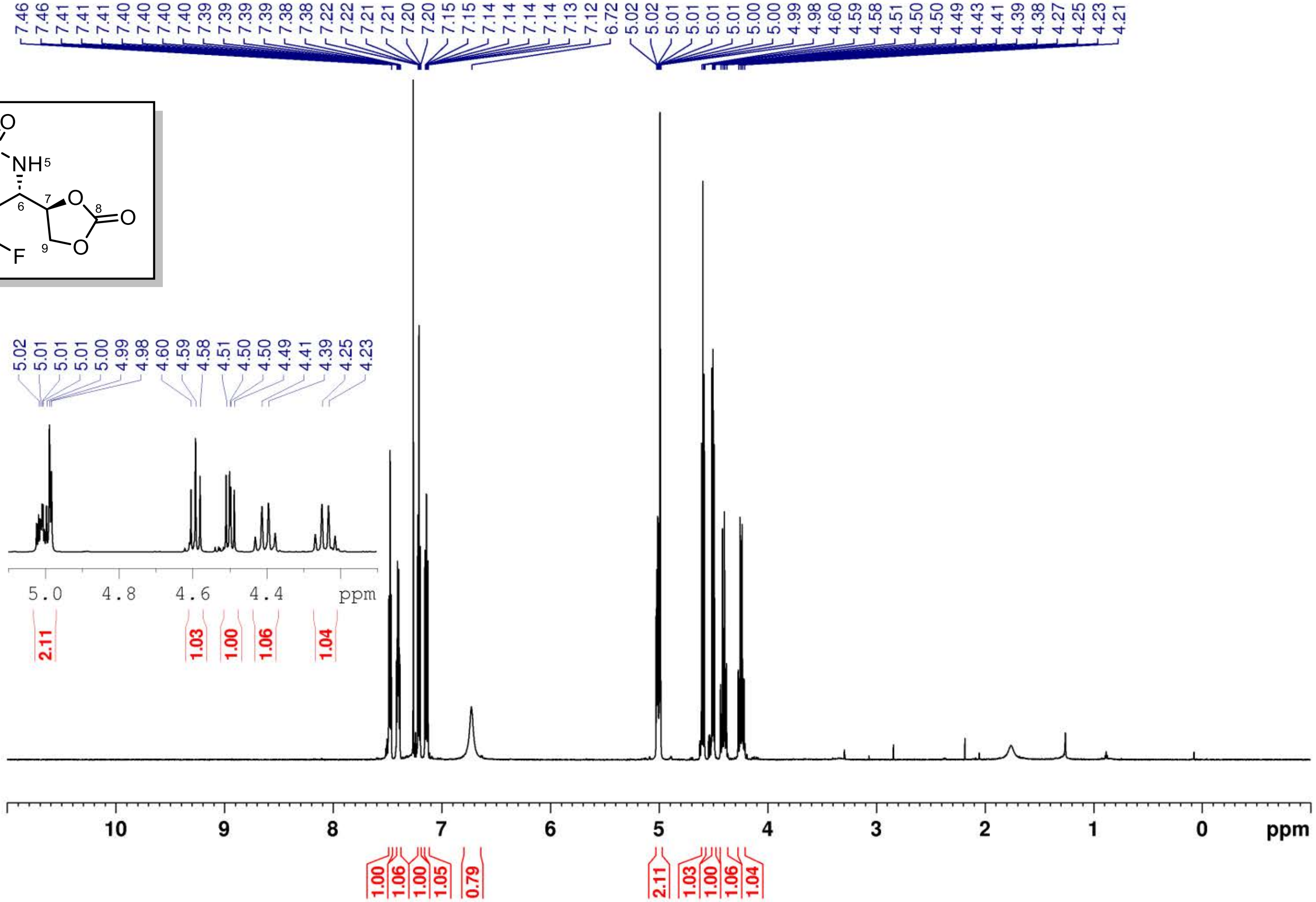

^{13}C NMR (176 MHz, CDCl$_3$) for 2,2,3,3,4,4,4-heptafluorobutyl ((*S*)-(2-fluorophenyl)((*R*)-2-oxo-1,3-dioxolan-4-yl)methyl)sulfamate (**4d**)

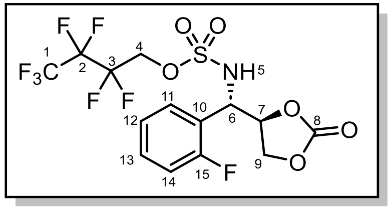

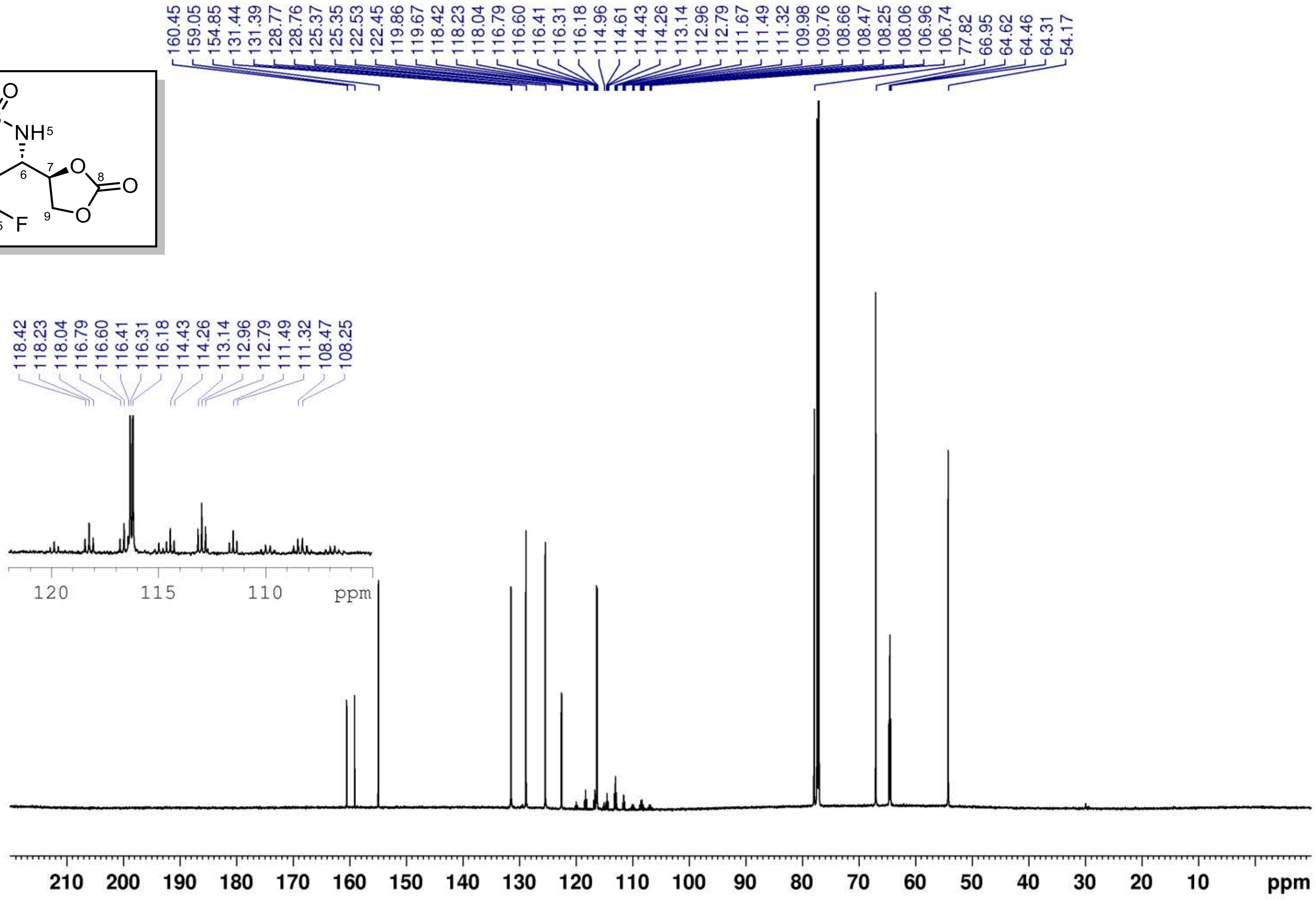

^{19}F NMR (376 MHz, CDCl$_3$) for 2,2,3,3,4,4,4-heptafluorobutyl ((*S*)-(2-fluorophenyl)((*R*)-2-oxo-1,3-dioxolan-4-yl)methyl)sulfamate (**4d**)

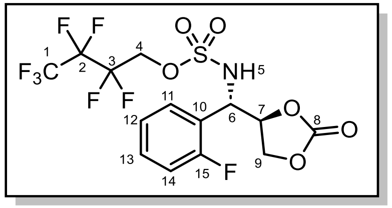

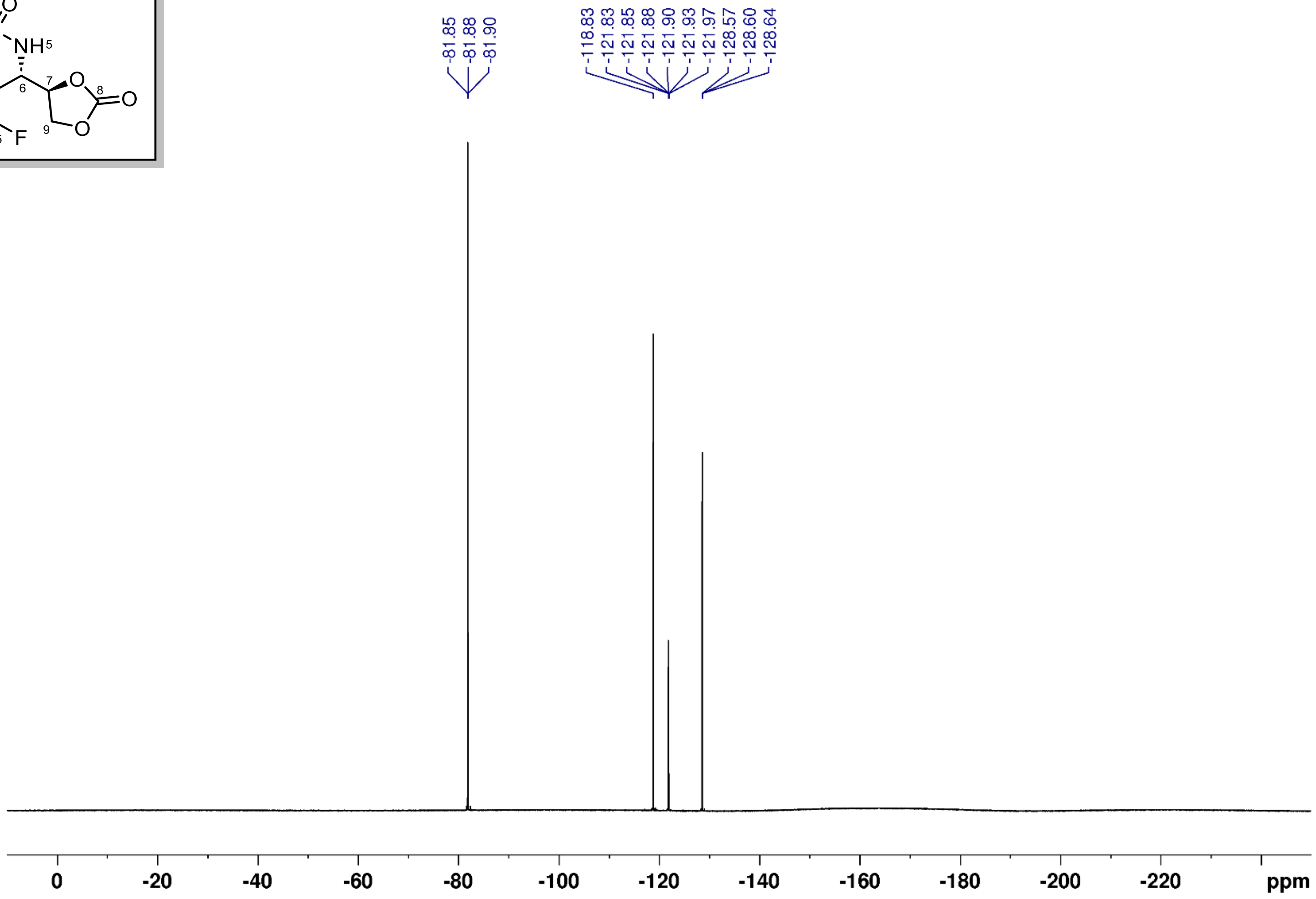

^{1}H NMR (700 MHz, CDCl3) for 2,2,3,3,4,4,4-heptafluorobutyl ((*S*)-((*R*)-2-oxo-1,3-dioxolan-4-yl)(o-tolyl)methyl)sulfamate (**4e**)

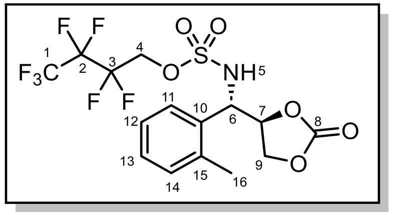

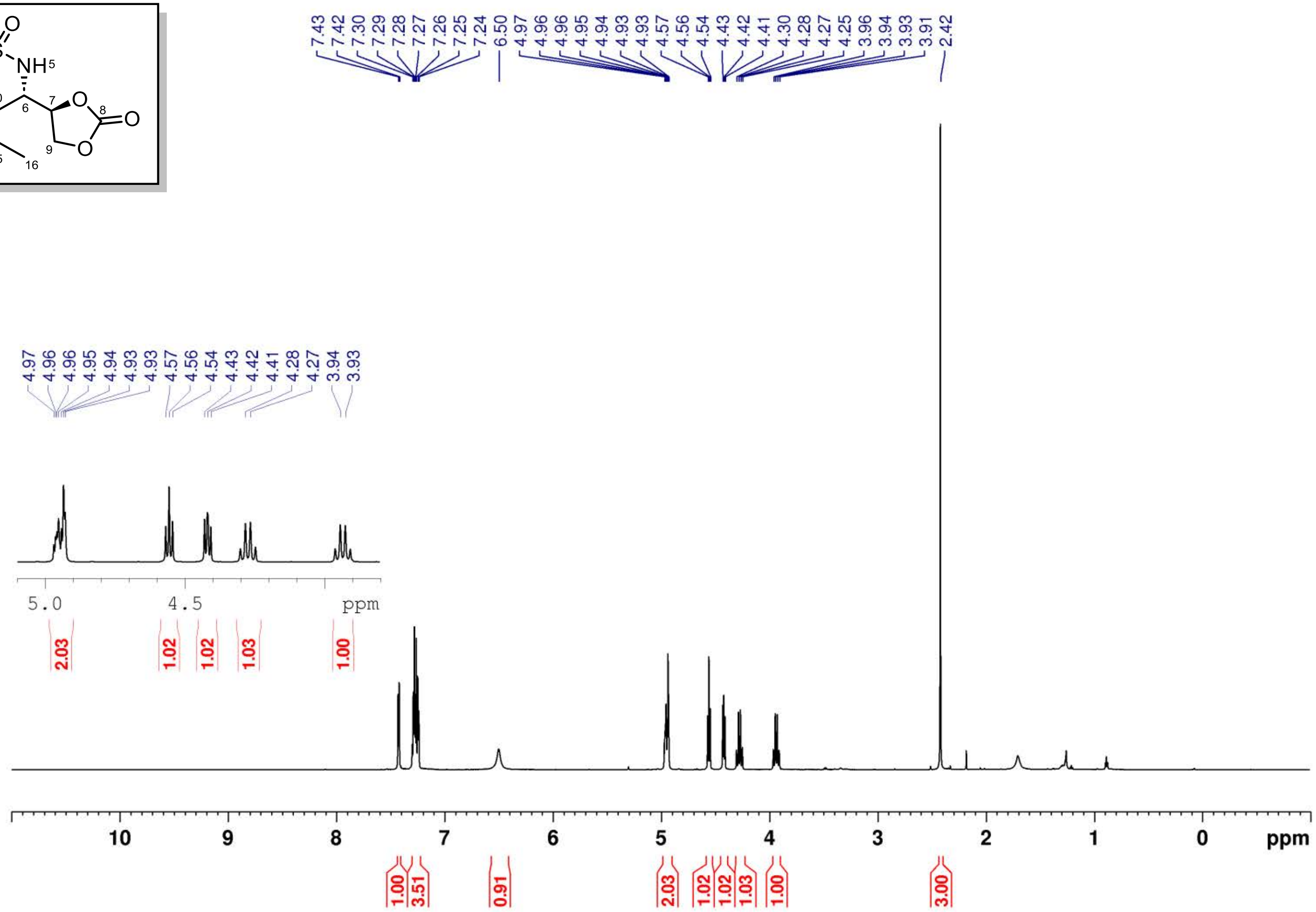

^{13}C NMR (176 MHz, CDCl$_3$) for 2,2,3,3,4,4,4-heptafluorobutyl ((*S*)-((*R*)-2-oxo-1,3-dioxolan-4-yl)(o-tolyl)methyl)sulfamate (**4e**)

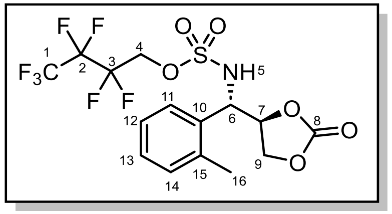

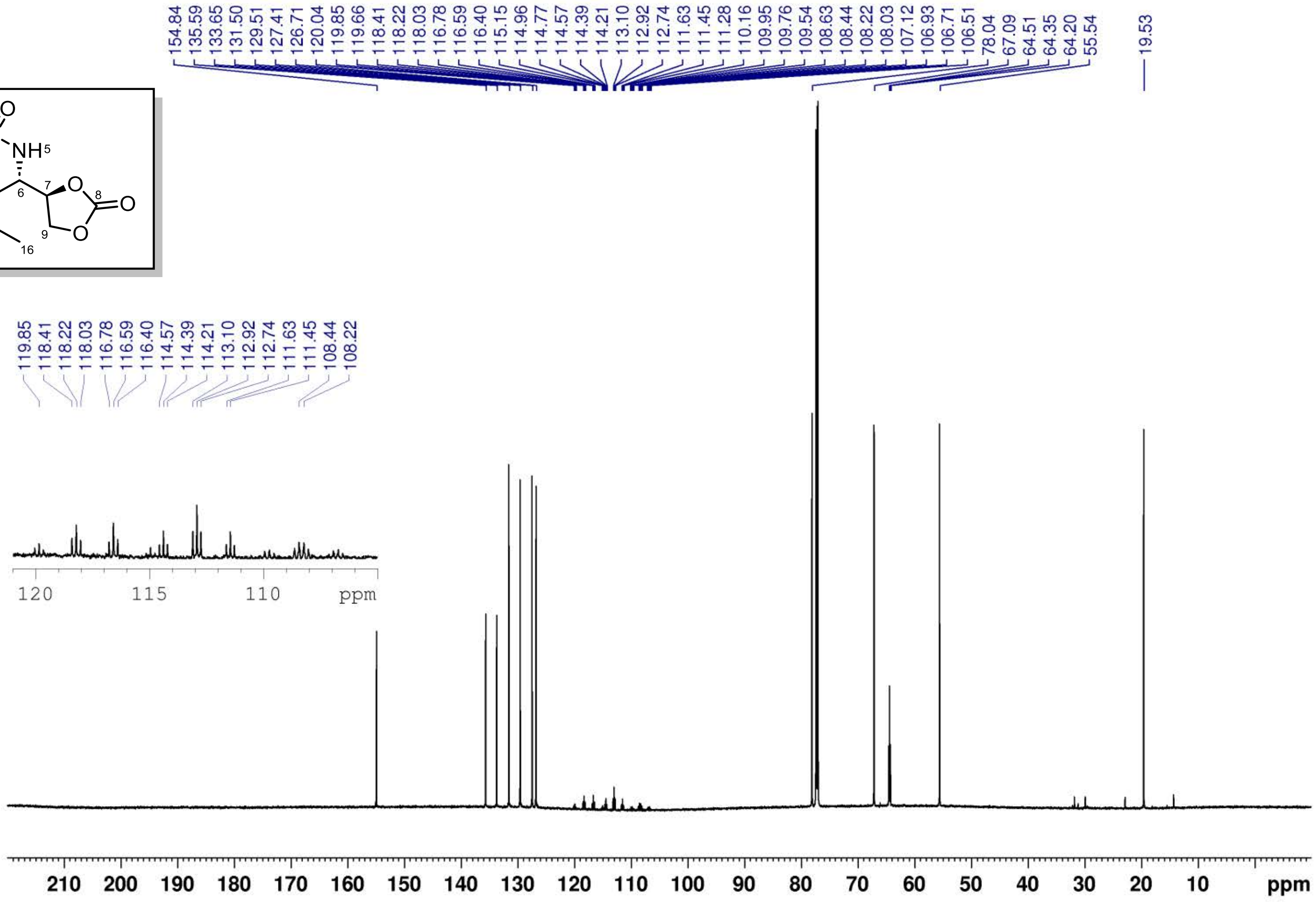

^{19}F NMR (376 MHz, CDCl$_3$) for 2,2,3,3,4,4,4-heptafluorobutyl ((*S*)-((*R*)-2-oxo-1,3-dioxolan-4-yl)(o-tolyl)methyl)sulfamate (**4e**)

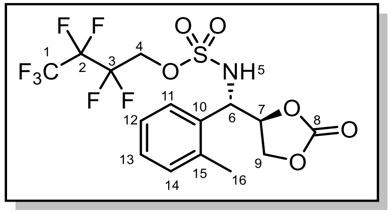

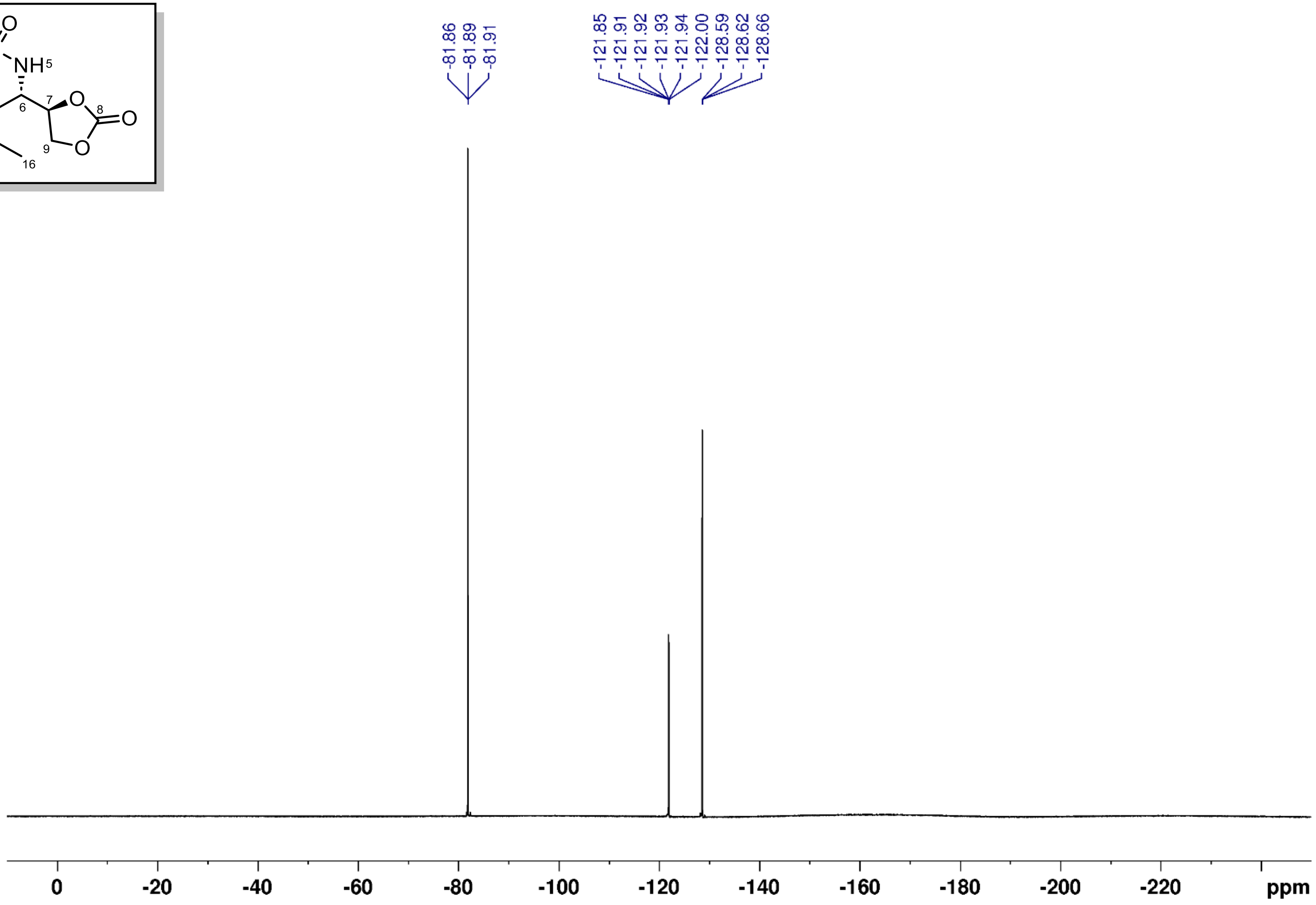

^{1}H NMR (700 MHz, CD_3CN) for 2,2,3,3,4,4,4-heptafluorobutyl ((*S*)-(3-((*tert*-butoxycarbonyl)(methyl)amino)phenyl)((*R*)-2-oxo-1,3-dioxolan-4-yl)methyl)sulfamate (**4f**)

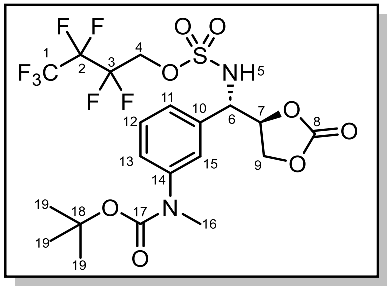

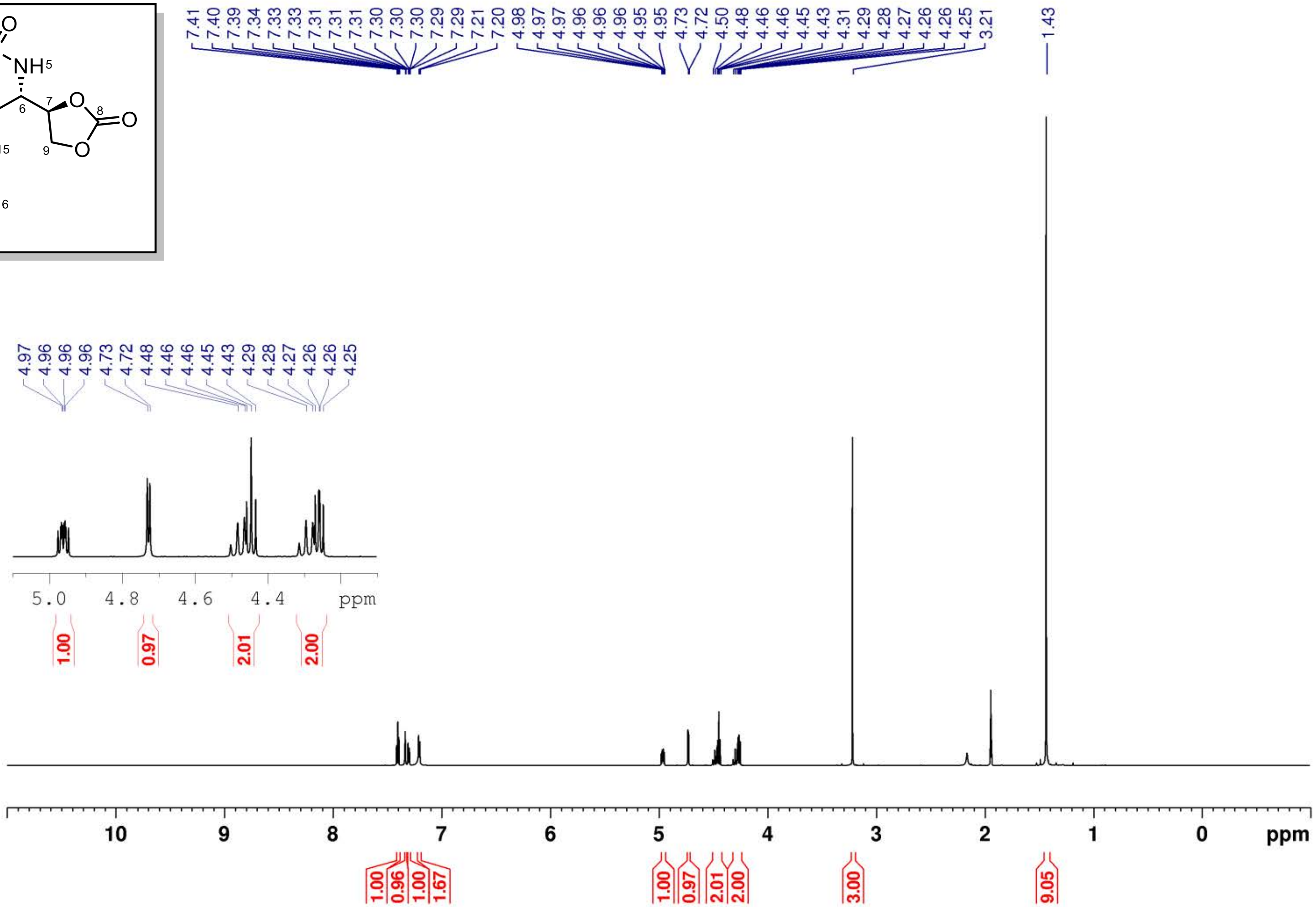

^{13}C NMR (176 MHz, CD_3CN) for 2,2,3,3,4,4,4-heptafluorobutyl ((*S*)-(3-((*tert*-butoxycarbonyl)(methyl)amino)phenyl)((*R*)-2-oxo-1,3-dioxolan-4-yl)methyl)sulfamate (**4f**)

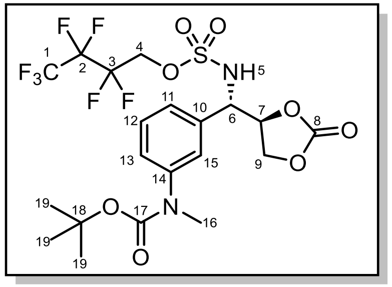

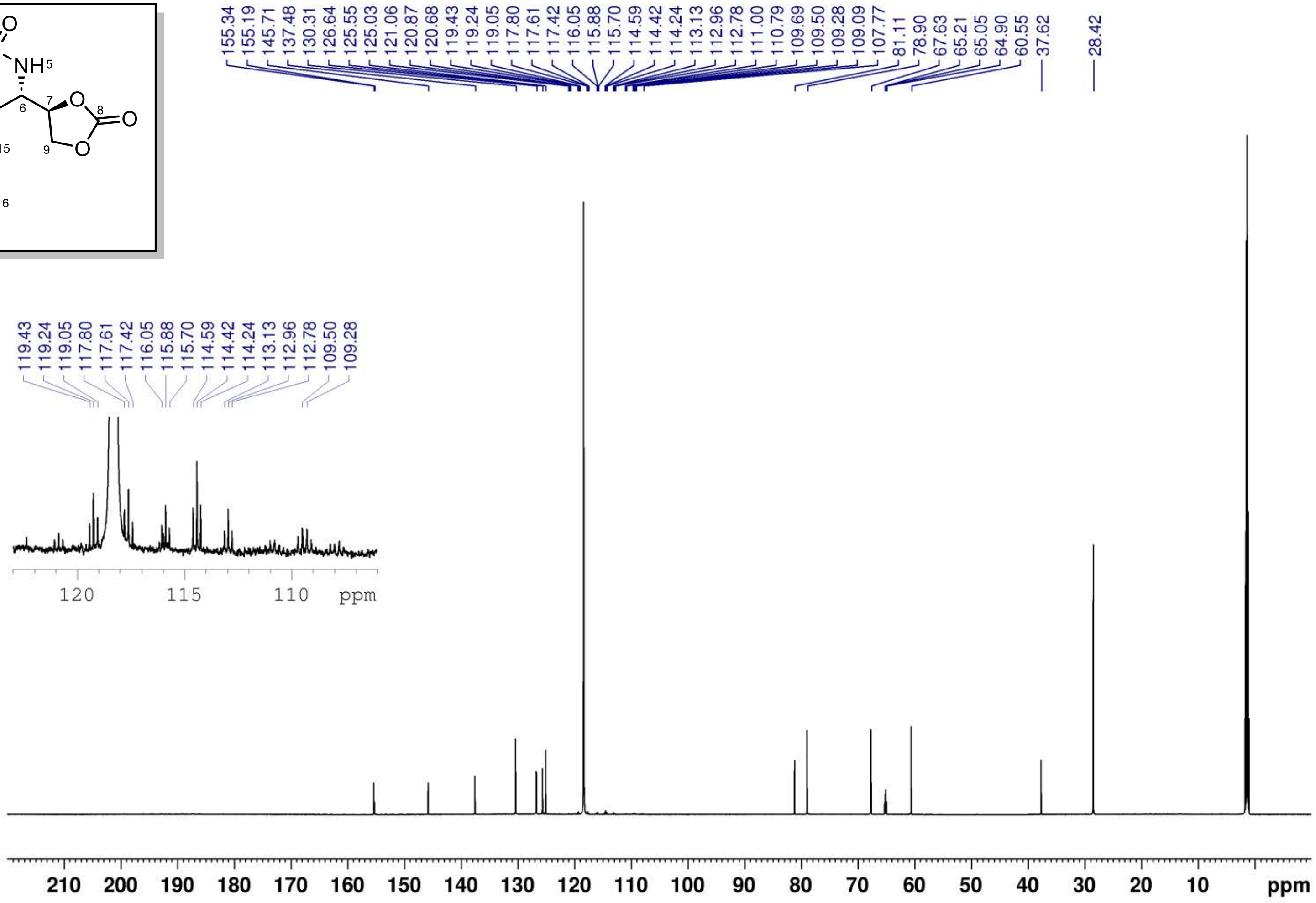

^{19}F NMR (376 MHz, CD$_3$CN) for 2,2,3,3,4,4,4-heptafluorobutyl ((*S*)-(3-((*tert*-butoxycarbonyl)(methyl)amino)phenyl)((*R*)-2-oxo-1,3-dioxolan-4-yl)methyl)sulfamate (**4f**)

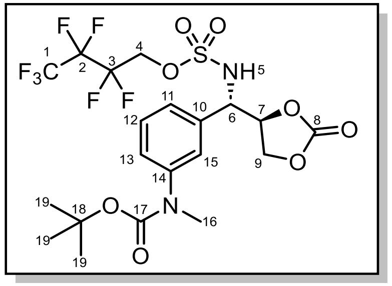

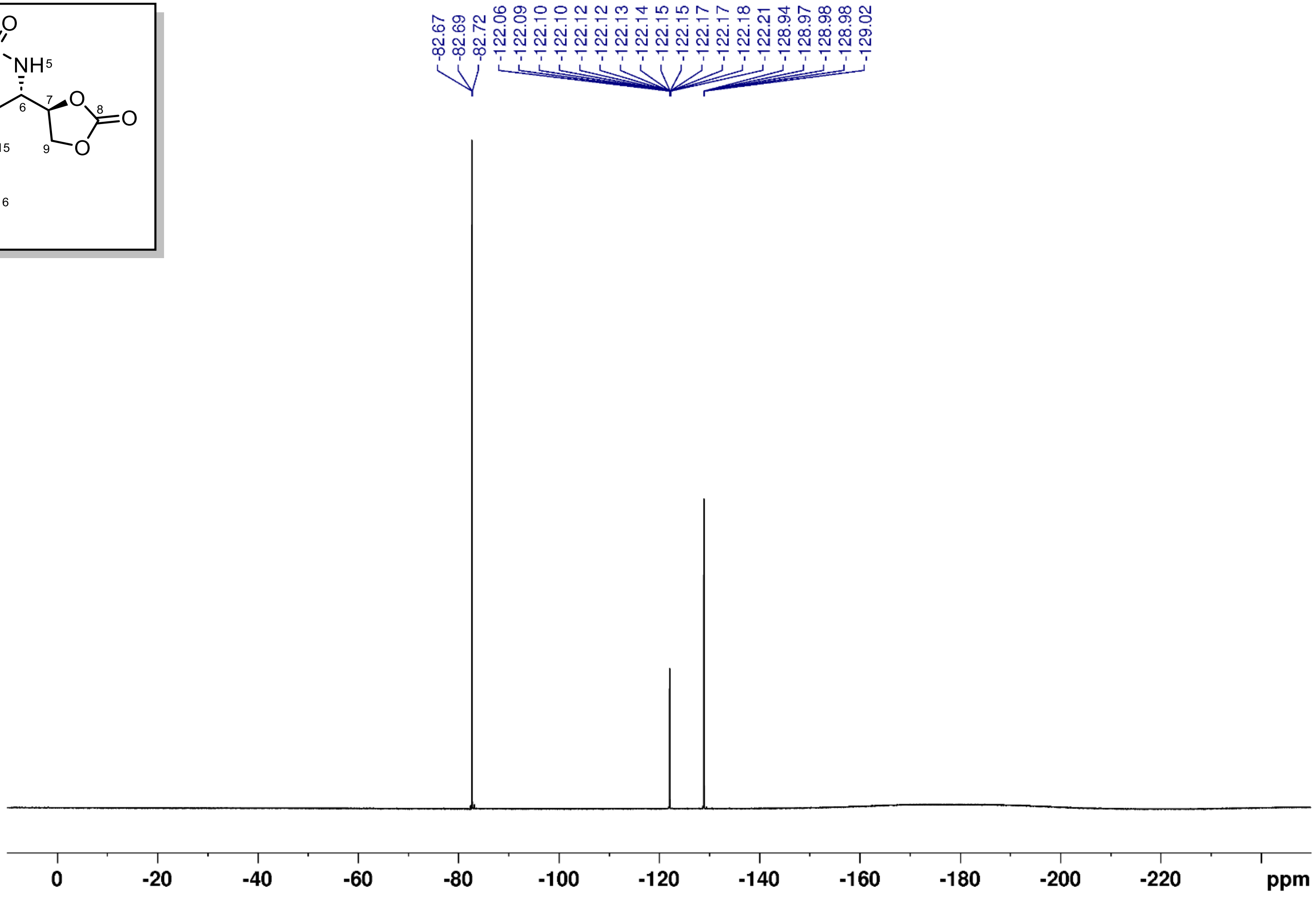

^{1}H NMR (700 MHz, $CDCl_3$) for 2,2,3,3,4,4,4-heptafluorobutyl ((*S*)-(3-(*tert*-butyl)phenyl)((*R*)-2-oxo-1,3-dioxolan-4-yl)methyl)sulfamate (**4g**)

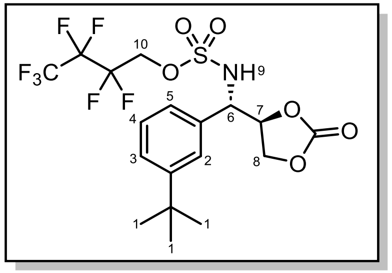

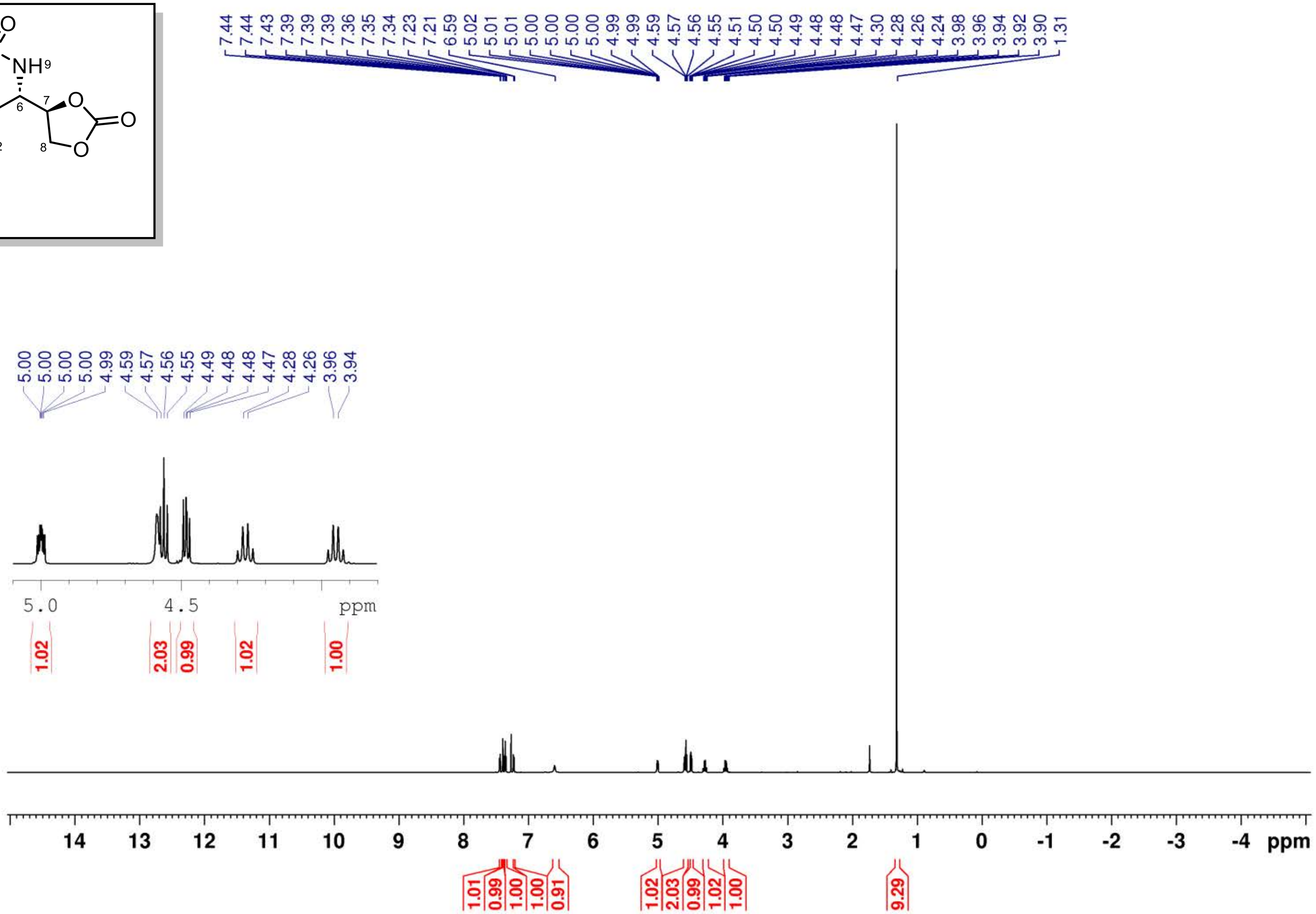

^{13}C NMR (176 MHz, CDCl$_3$) for 2,2,3,3,4,4,4-heptafluorobutyl ((*S*)-(3-(*tert*-butyl)phenyl)((*R*)-2-oxo-1,3-dioxolan-4-yl)methyl)sulfamate (**4g**)

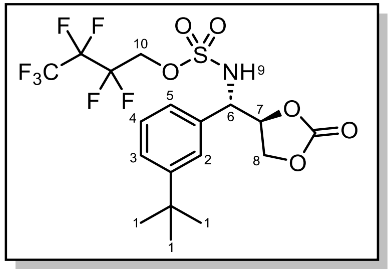

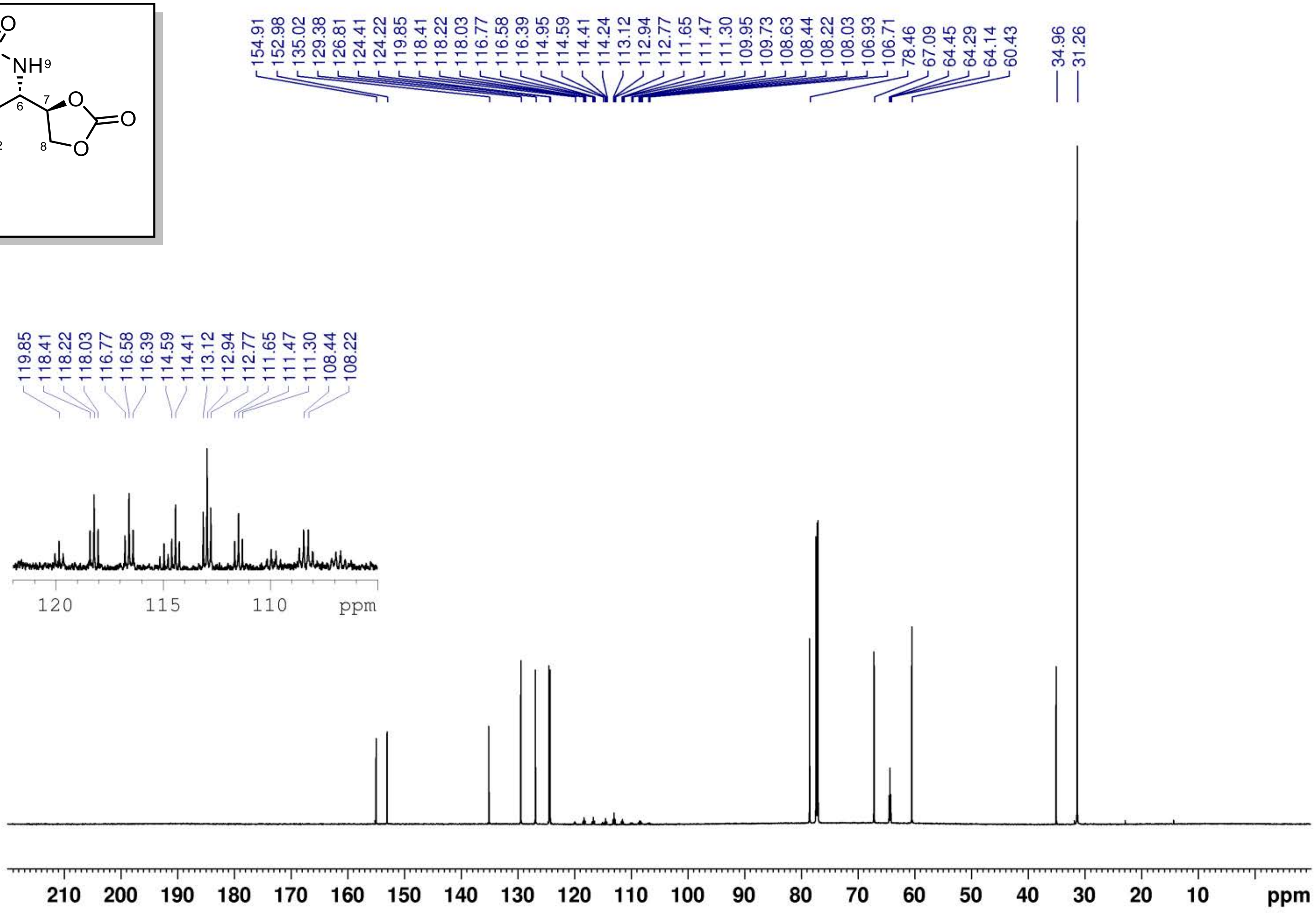

^{19}F NMR (376 MHz, CDCl$_3$) for 2,2,3,3,4,4,4-heptafluorobutyl ((*S*)-(3-(*tert*-butyl)phenyl)((*R*)-2-oxo-1,3-dioxolan-4-yl)methyl)sulfamate (**4g**)

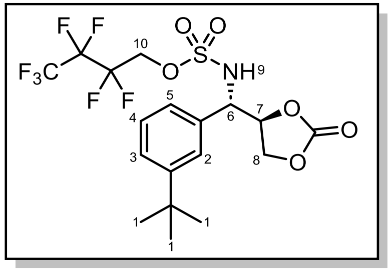

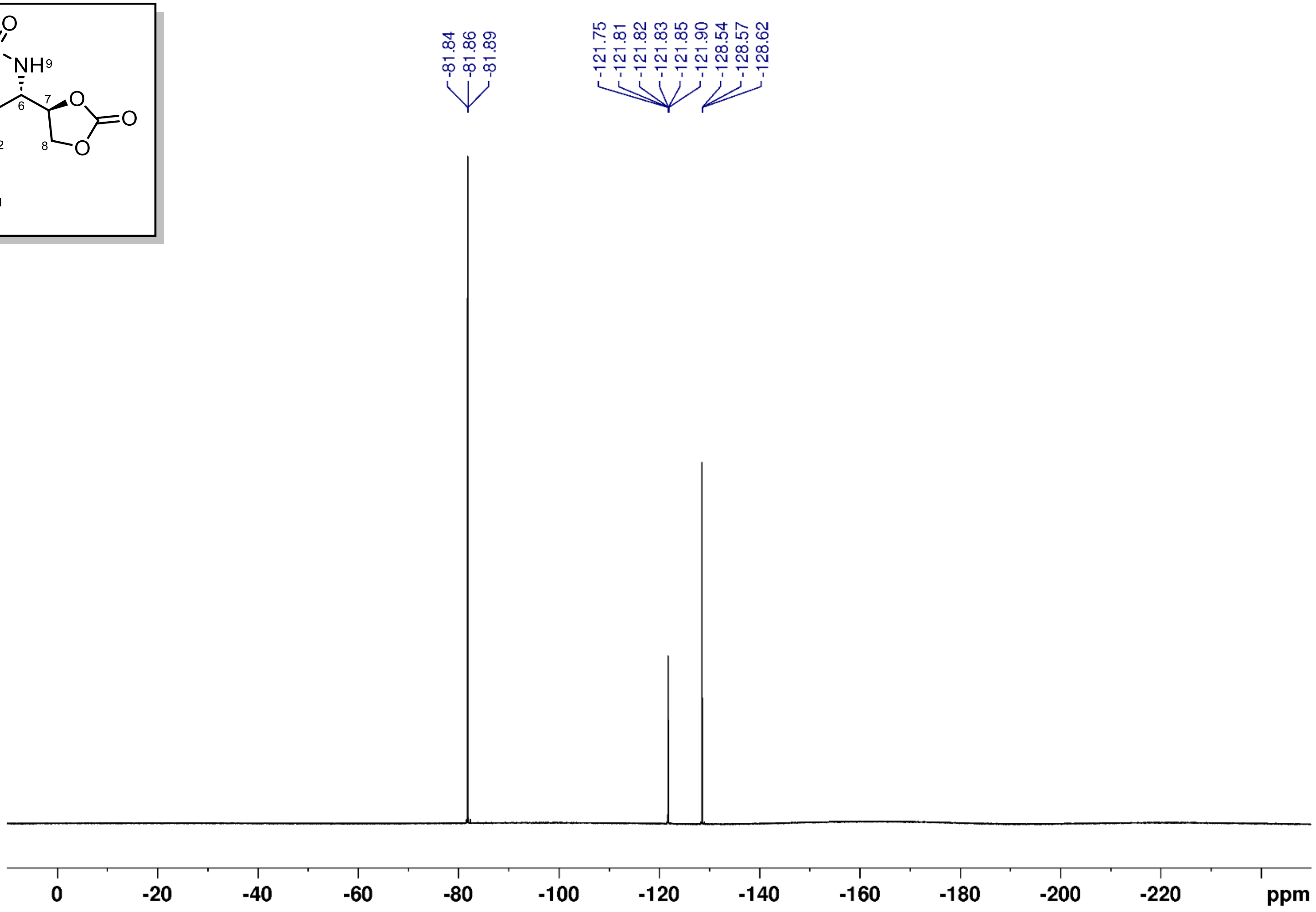

^{1}H NMR (700 MHz, CDCl$_3$) for 2,2,3,3,4,4,4-heptafluorobutyl ((*S*)-(3-methoxyphenyl)((*R*)-2-oxo-1,3-dioxolan-4-yl)methyl)sulfamate (**4h**)

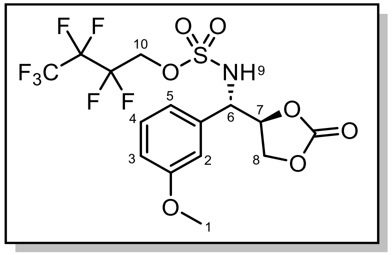

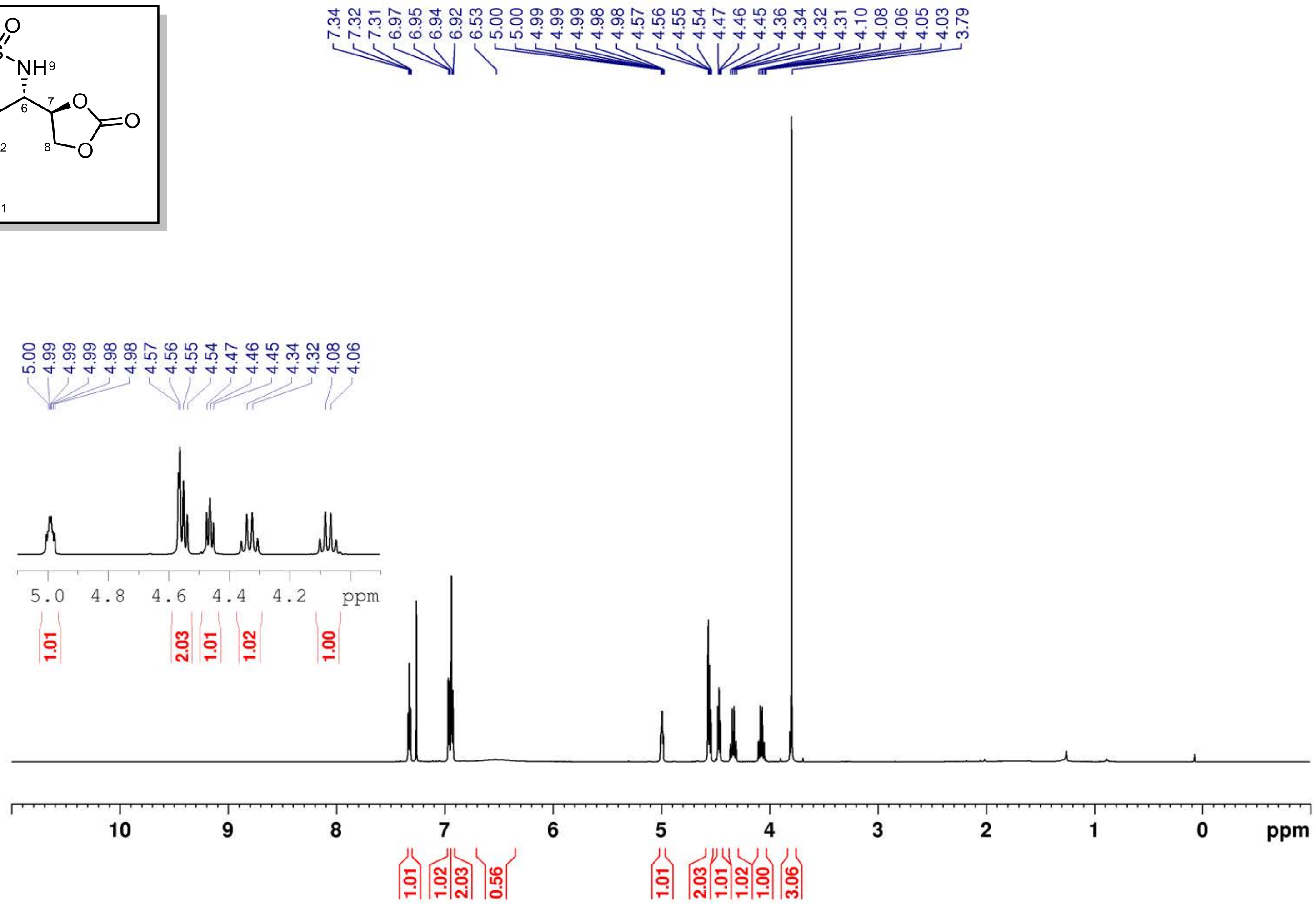

^{13}C NMR (176 MHz, $CDCl_3$) for 2,2,3,3,4,4,4-heptafluorobutyl ((*S*)-(3-methoxyphenyl)((*R*)-2-oxo-1,3-dioxolan-4-yl)methyl)sulfamate (**4h**)

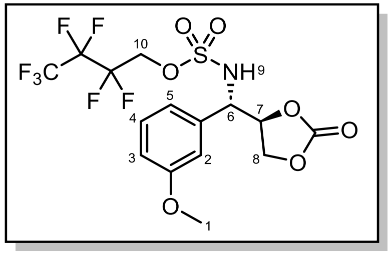

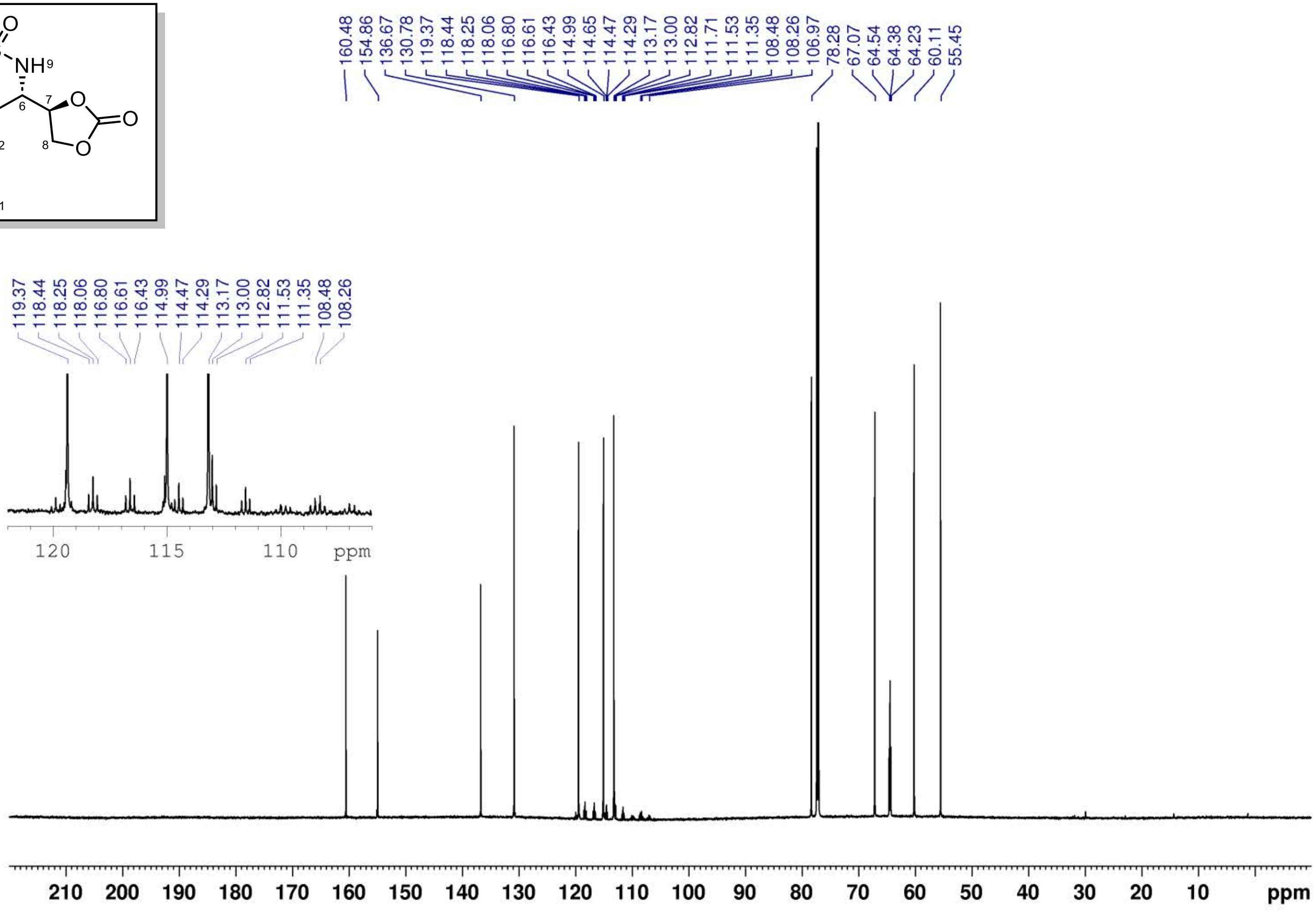

¹⁹F NMR (376 MHz, CDCl₃) for 2,2,3,3,4,4,4-heptafluorobutyl ((*S*)-(3-methoxyphenyl)((*R*)-2-oxo-1,3-dioxolan-4-yl)methyl)sulfamate (**4h**)

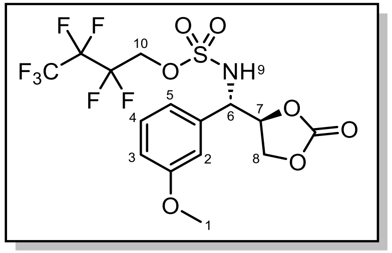

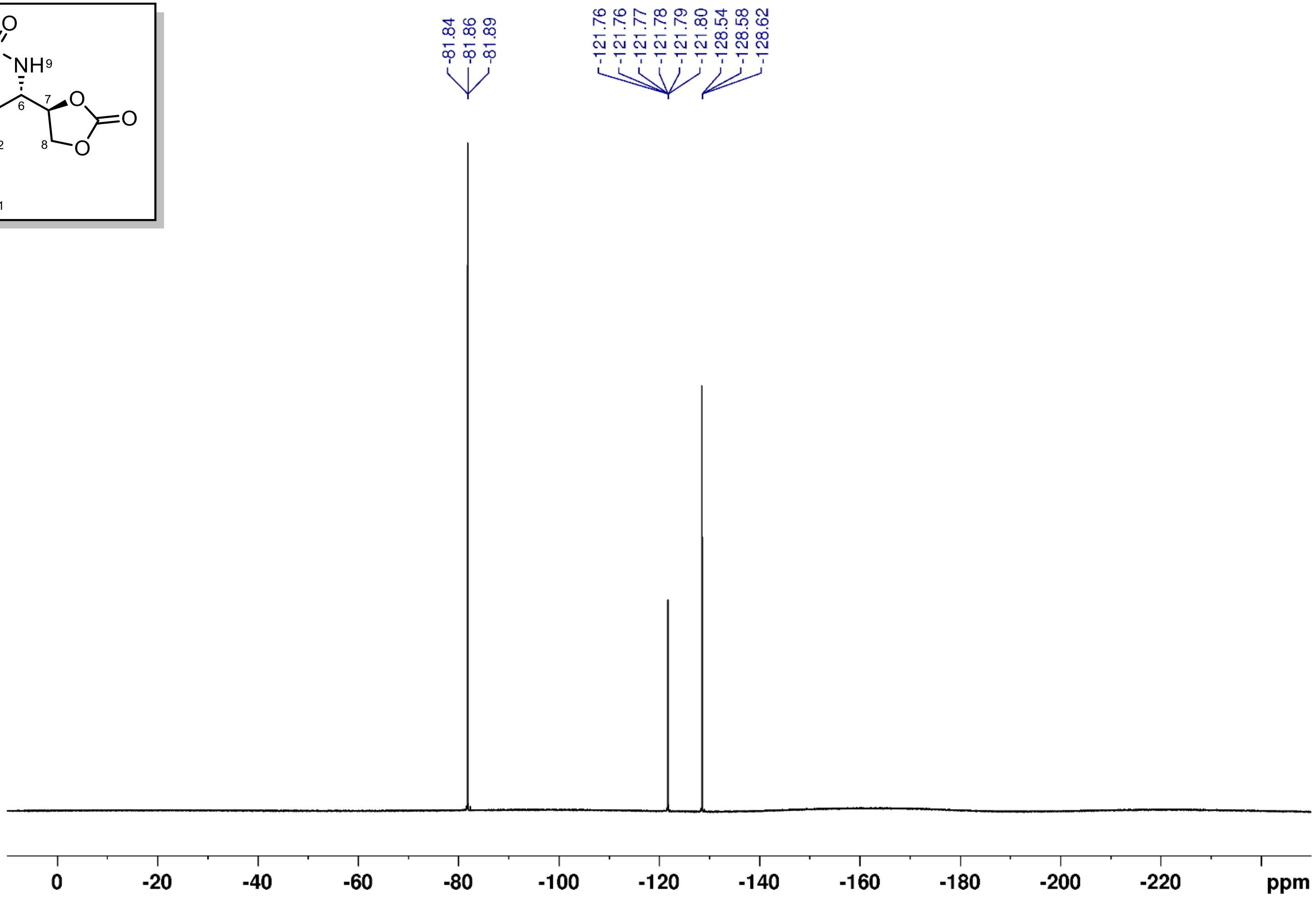

^{1}H NMR (700 MHz, CD_3CN) for 2,2,3,3,4,4,4-heptafluorobutyl ((*S*)-(3-bromophenyl)((*R*)-2-oxo-1,3-dioxolan-4-yl)methyl)sulfamate (**4i**)

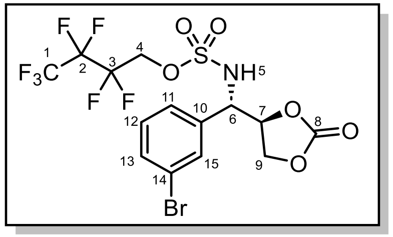

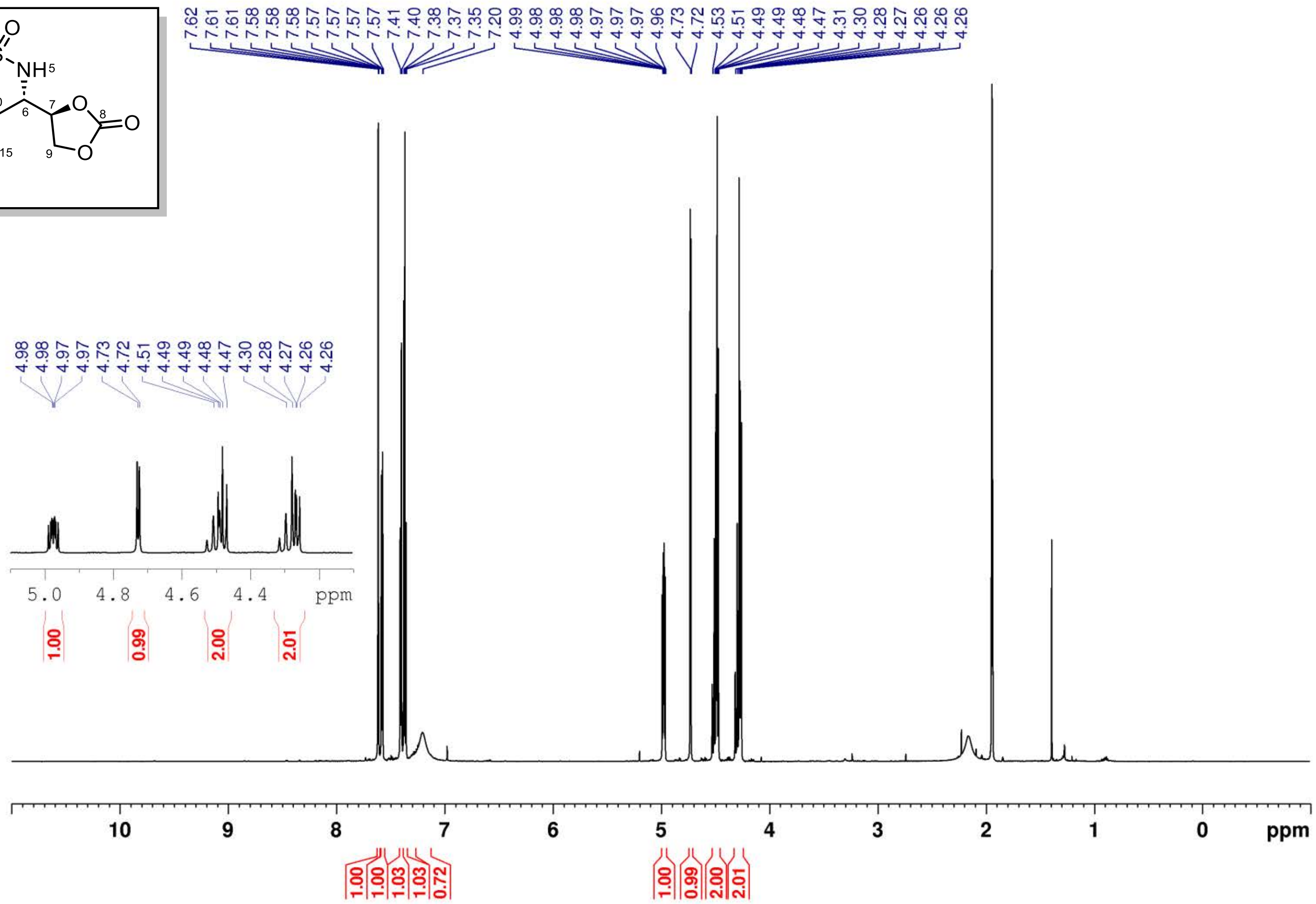

^{13}C NMR (176 MHz, CD_3CN) for 2,2,3,3,4,4,4-heptafluorobutyl ((*S*)-(3-bromophenyl)((*R*)-2-oxo-1,3-dioxolan-4-yl)methyl)sulfamate (**4i**)

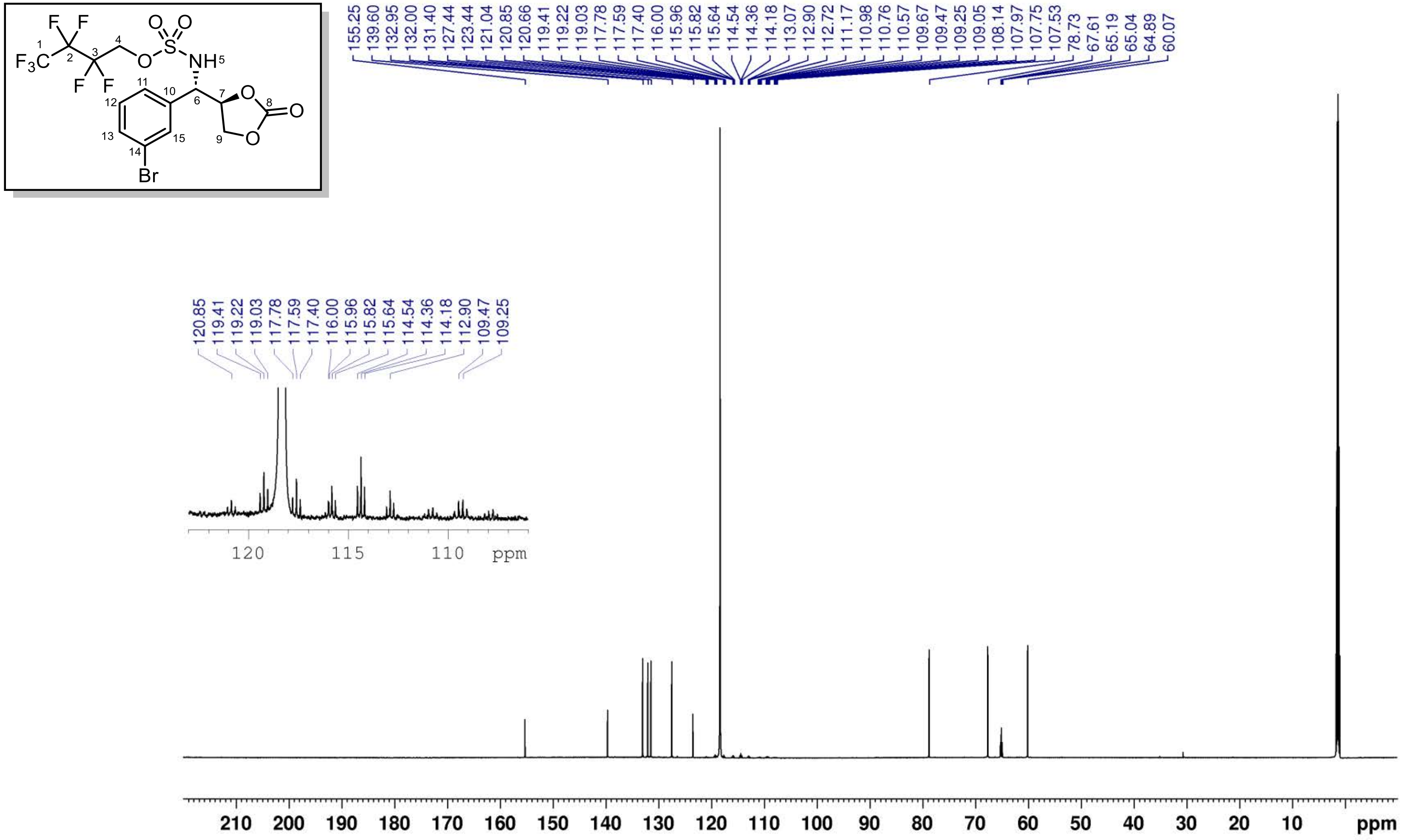

^{19}F NMR (376 MHz, CD_3CN) for 2,2,3,3,4,4,4-heptafluorobutyl ((*S*)-(3-bromophenyl)((*R*)-2-oxo-1,3-dioxolan-4-yl)methyl)sulfamate (**4i**)

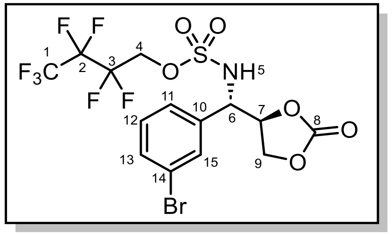

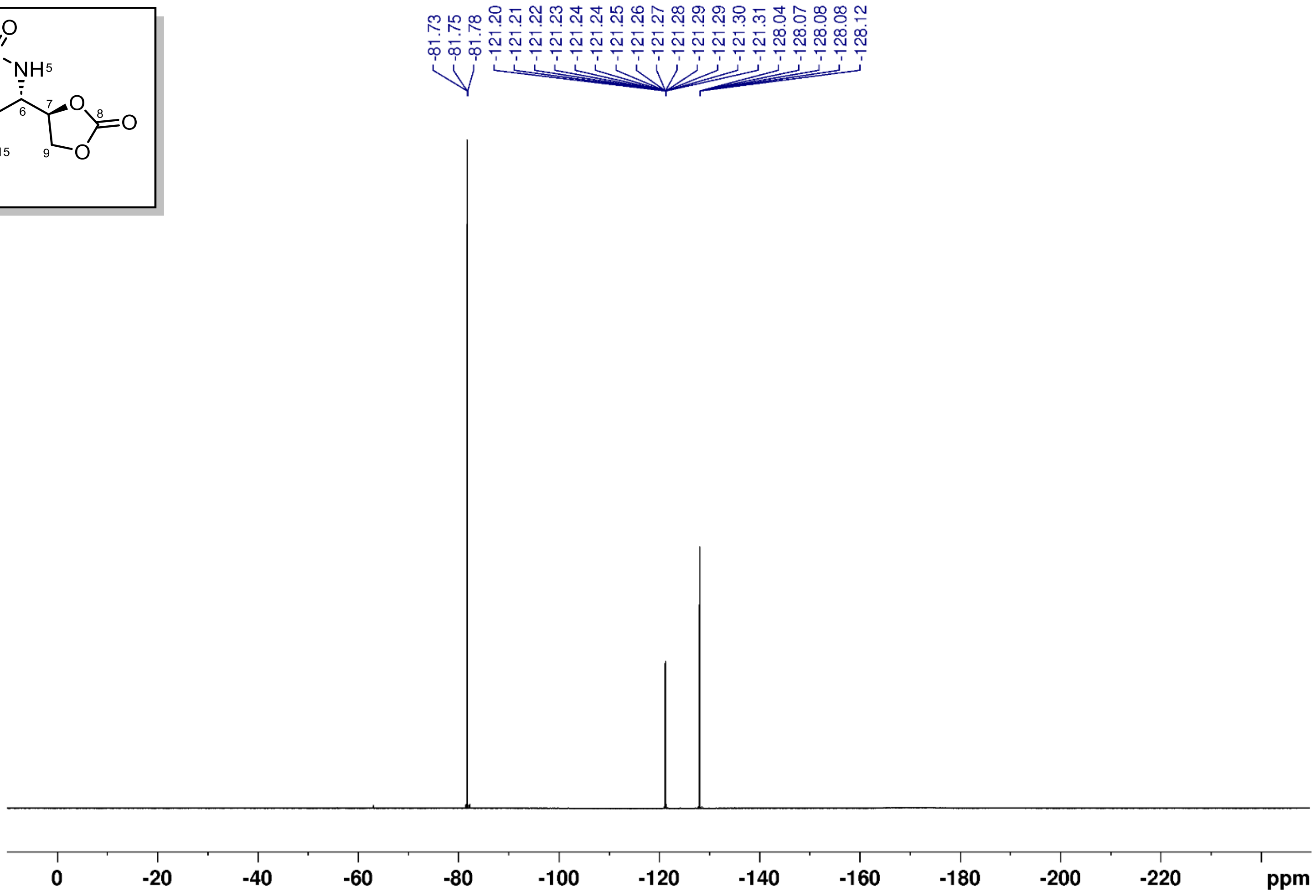

^{1}H NMR (700 MHz, CDCl$_3$) for ethyl 3-((*S*)-(((2,2,3,3,4,4,4-heptafluorobutoxy)sulfonyl)amino)((*R*)-2-oxo-1,3-dioxolan-4-yl)methyl)benzoate (**4j**)

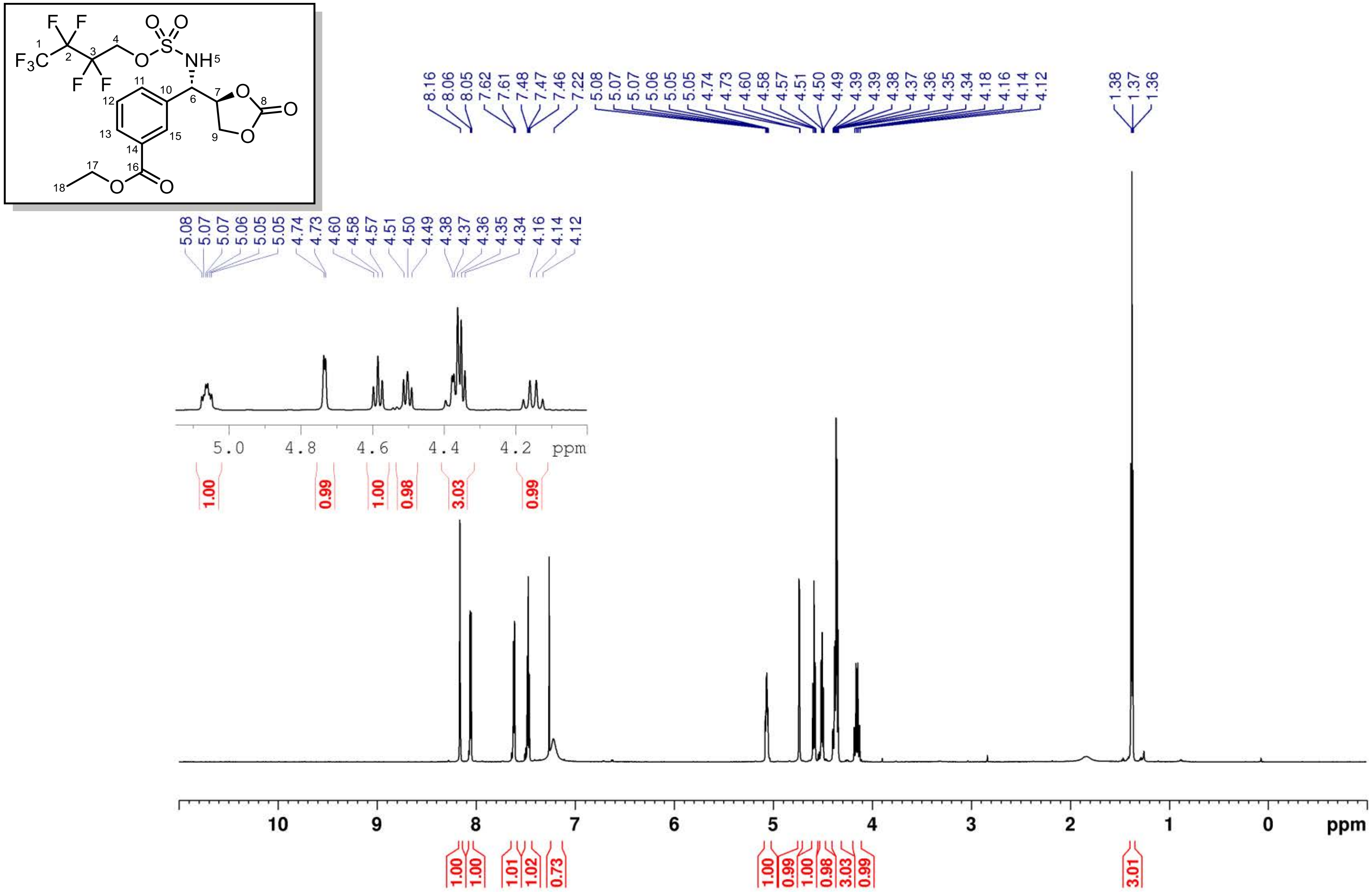

^{13}C NMR (176 MHz, CDCl$_3$) for ethyl 3-((*S*)-(((2,2,3,3,4,4,4-heptafluorobutoxy)sulfonyl)amino)((*R*)-2-oxo-1,3-dioxolan-4-yl)methyl)benzoate (**4j**)

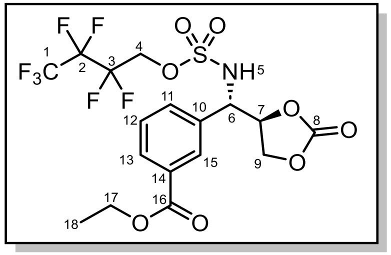

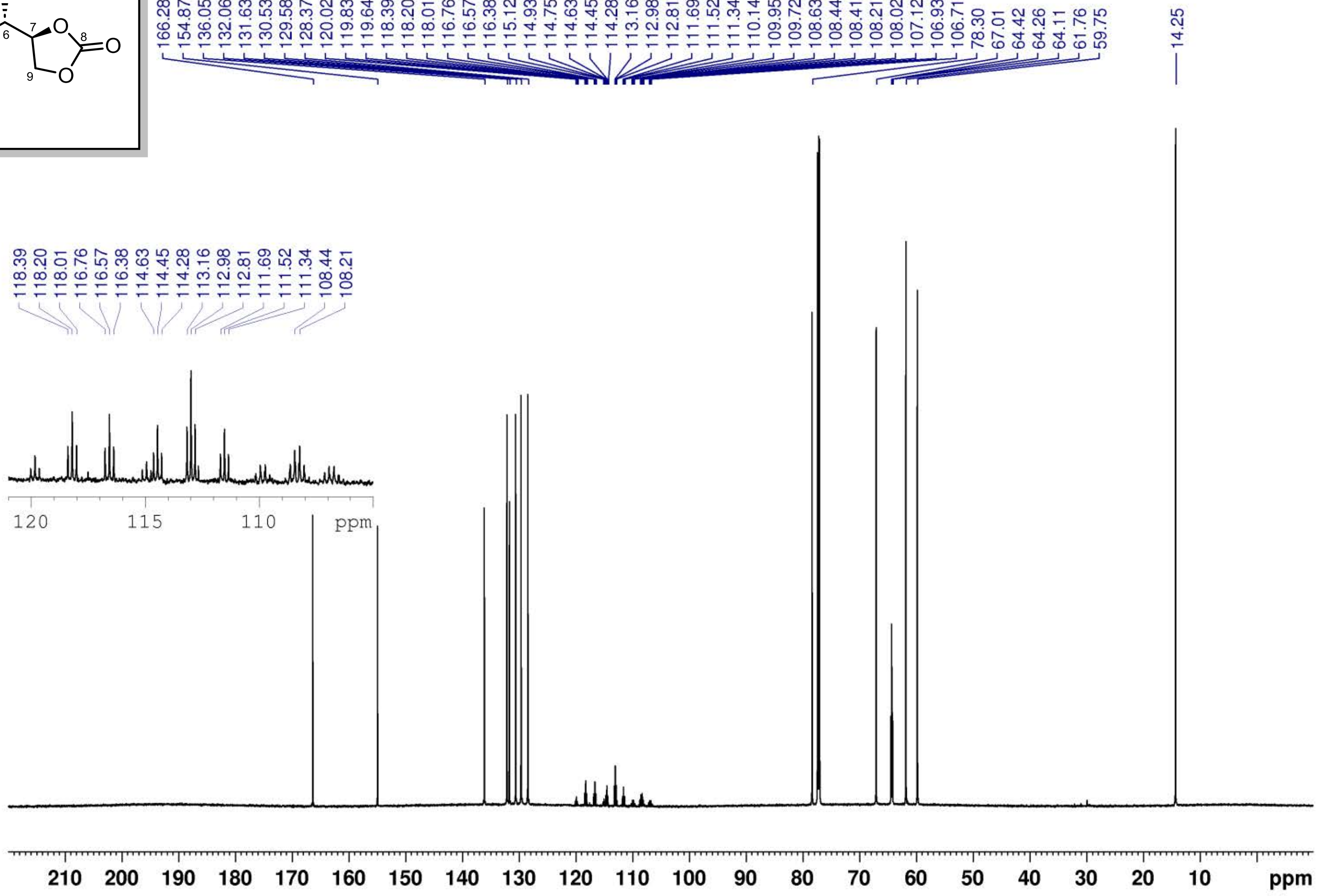

^{19}F NMR (376 MHz, $CDCl_3$) for ethyl 3-((*S*)-(((2,2,3,3,4,4,4-heptafluorobutoxy)sulfonyl)amino)((*R*)-2-oxo-1,3-dioxolan-4-yl)methyl)benzoate (**4j**)

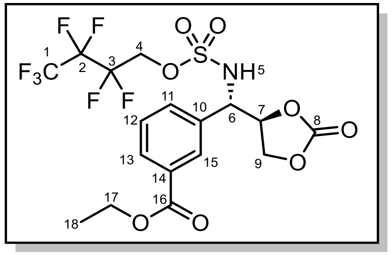

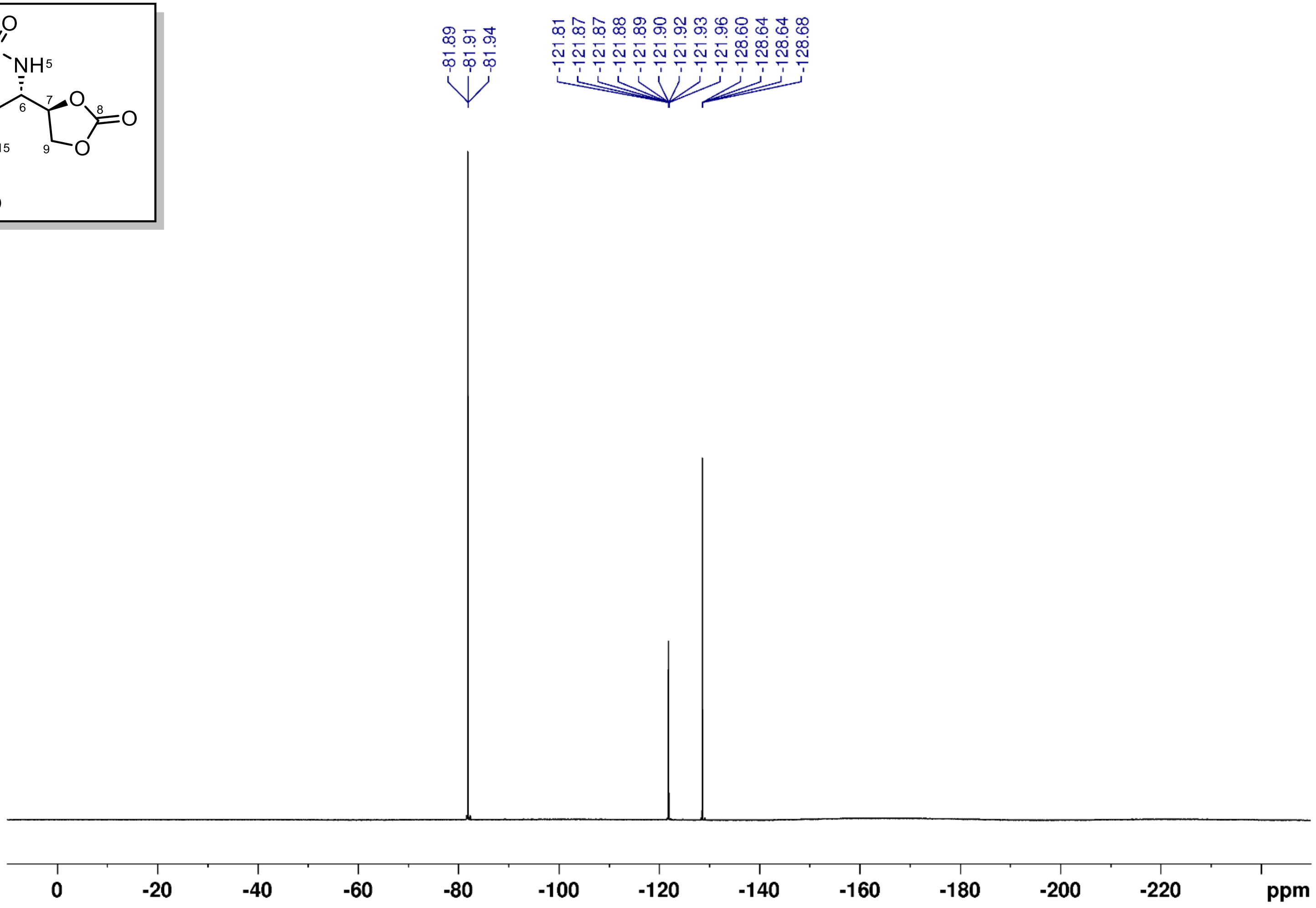

^{1}H NMR (700 MHz, CD_3CN) for 2,2,3,3,4,4,4-heptafluorobutyl ((*S*)-(3,4-dichlorophenyl)((*R*)-2-oxo-1,3-dioxolan-4-yl)methyl)sulfamate (**4k**)

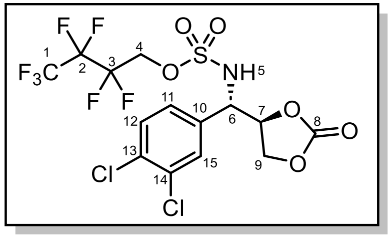

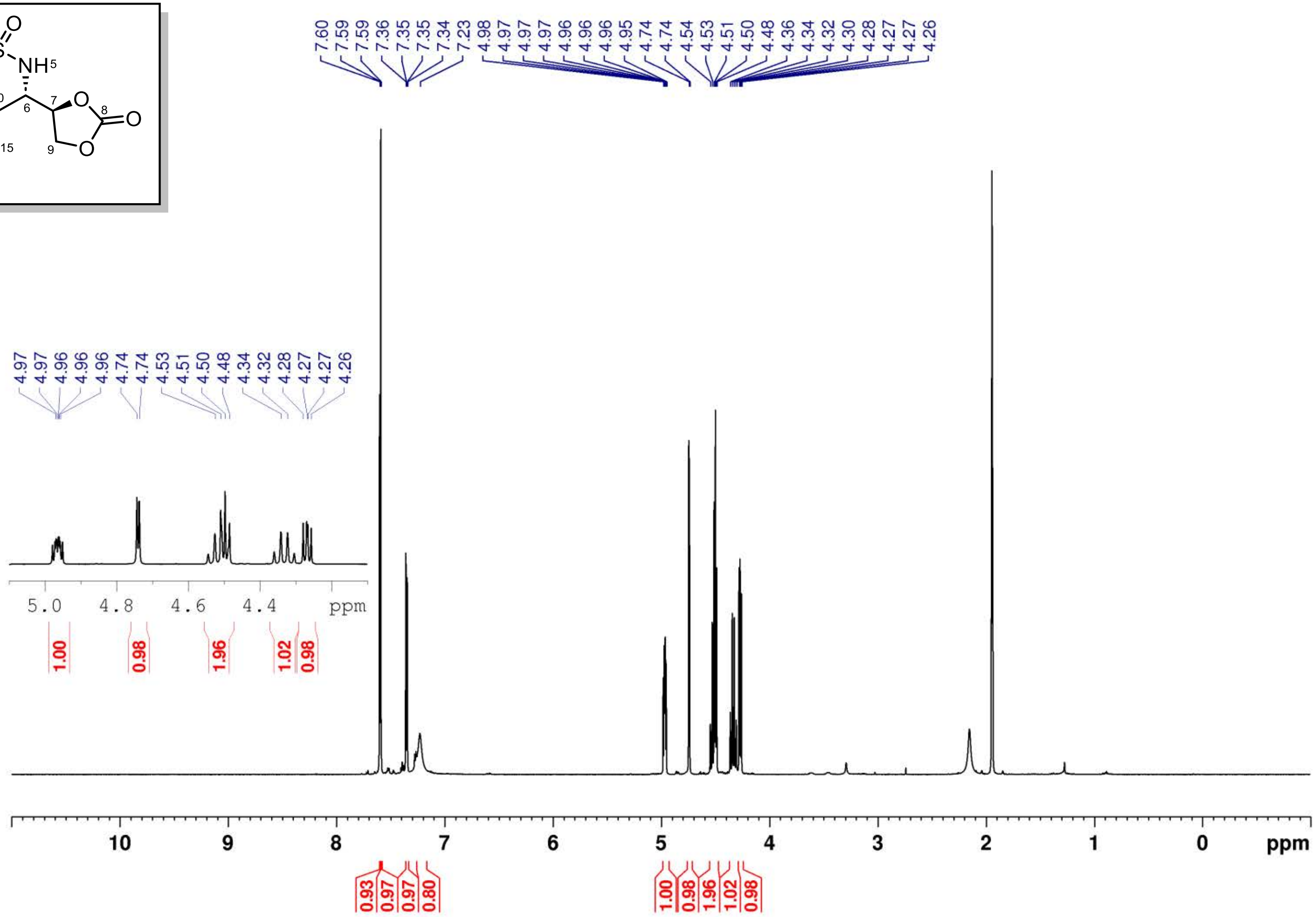

^{13}C NMR (176 MHz, CD_3CN) for 2,2,3,3,4,4,4-heptafluorobutyl ((*S*)-(3,4-dichlorophenyl)((*R*)-2-oxo-1,3-dioxolan-4-yl)methyl)sulfamate (**4k**)

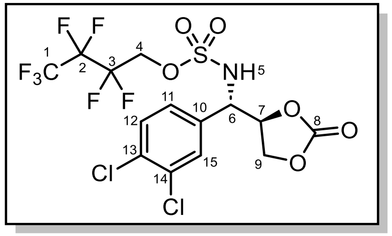

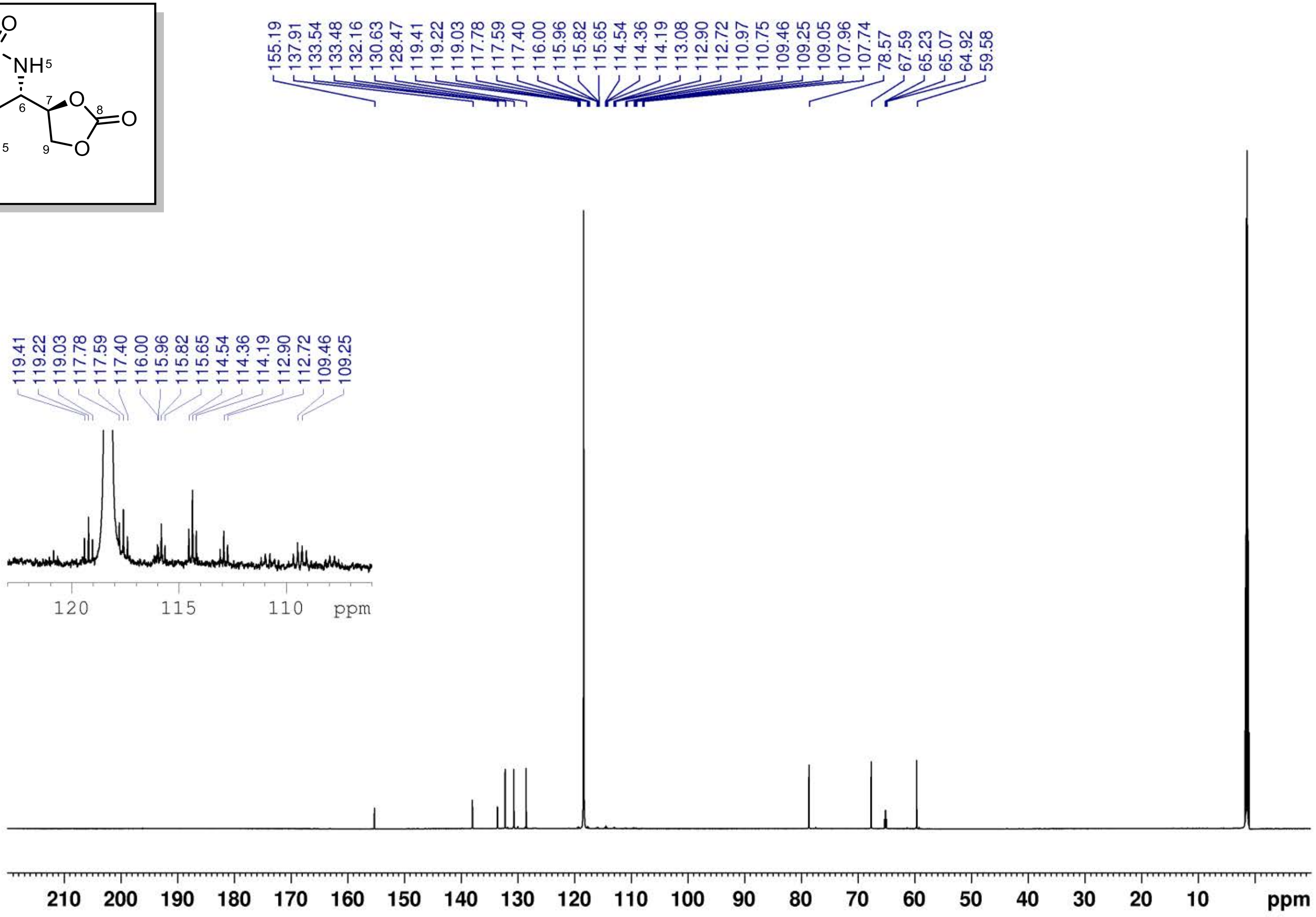

^{19}F NMR (376 MHz, CD_3CN) for 2,2,3,3,4,4,4-heptafluorobutyl ((*S*)-(3,4-dichlorophenyl)((*R*)-2-oxo-1,3-dioxolan-4-yl)methyl)sulfamate (**4k**)

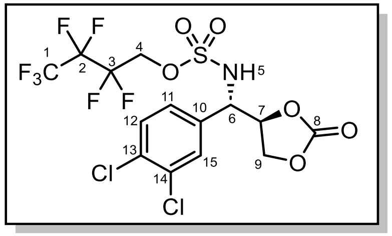

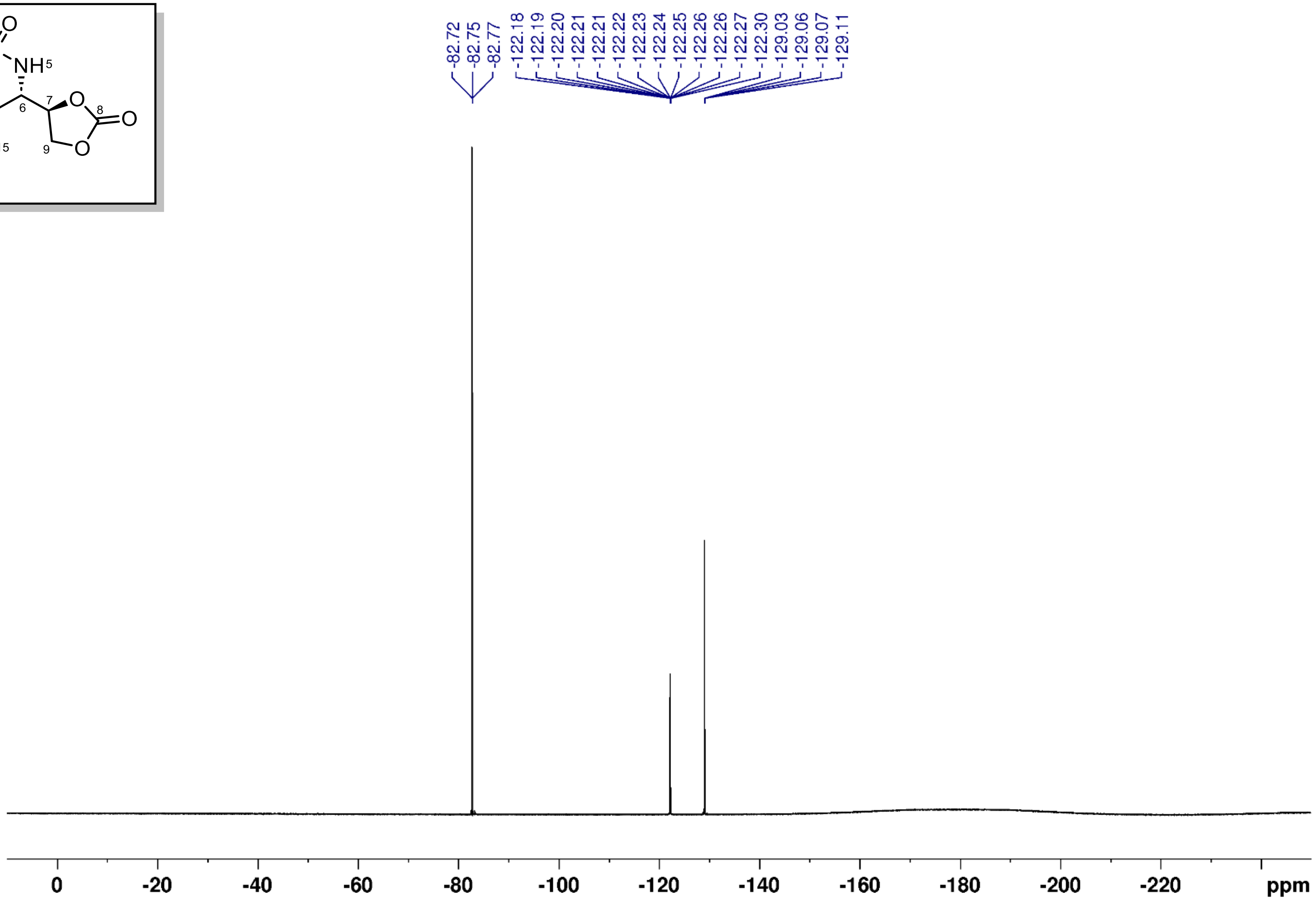

^{1}H NMR (700 MHz, CDCl$_3$) for 2,2,3,3,4,4,4-heptafluorobutyl ((*S*)-(4-(*tert*-butyl)phenyl)((*R*)-2-oxo-1,3-dioxolan-4-yl)methyl)sulfamate (**4l**)

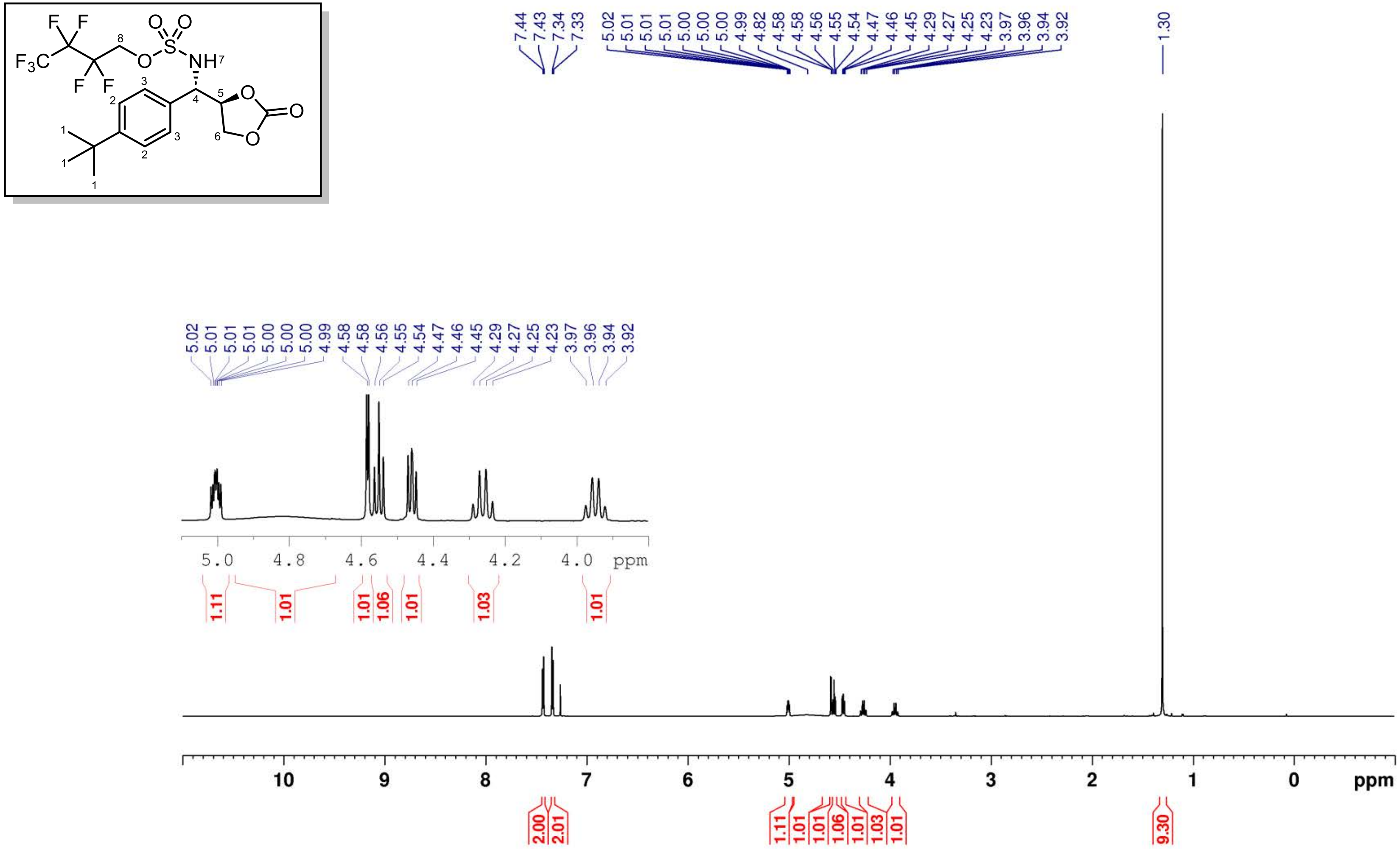

^{13}C NMR (176 MHz, $CDCl_3$) for 2,2,3,3,4,4,4-heptafluorobutyl ((*S*)-(4-(*tert*-butyl)phenyl)((*R*)-2-oxo-1,3-dioxolan-4-yl)methyl)sulfamate (**4l**)

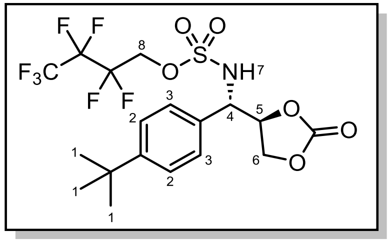

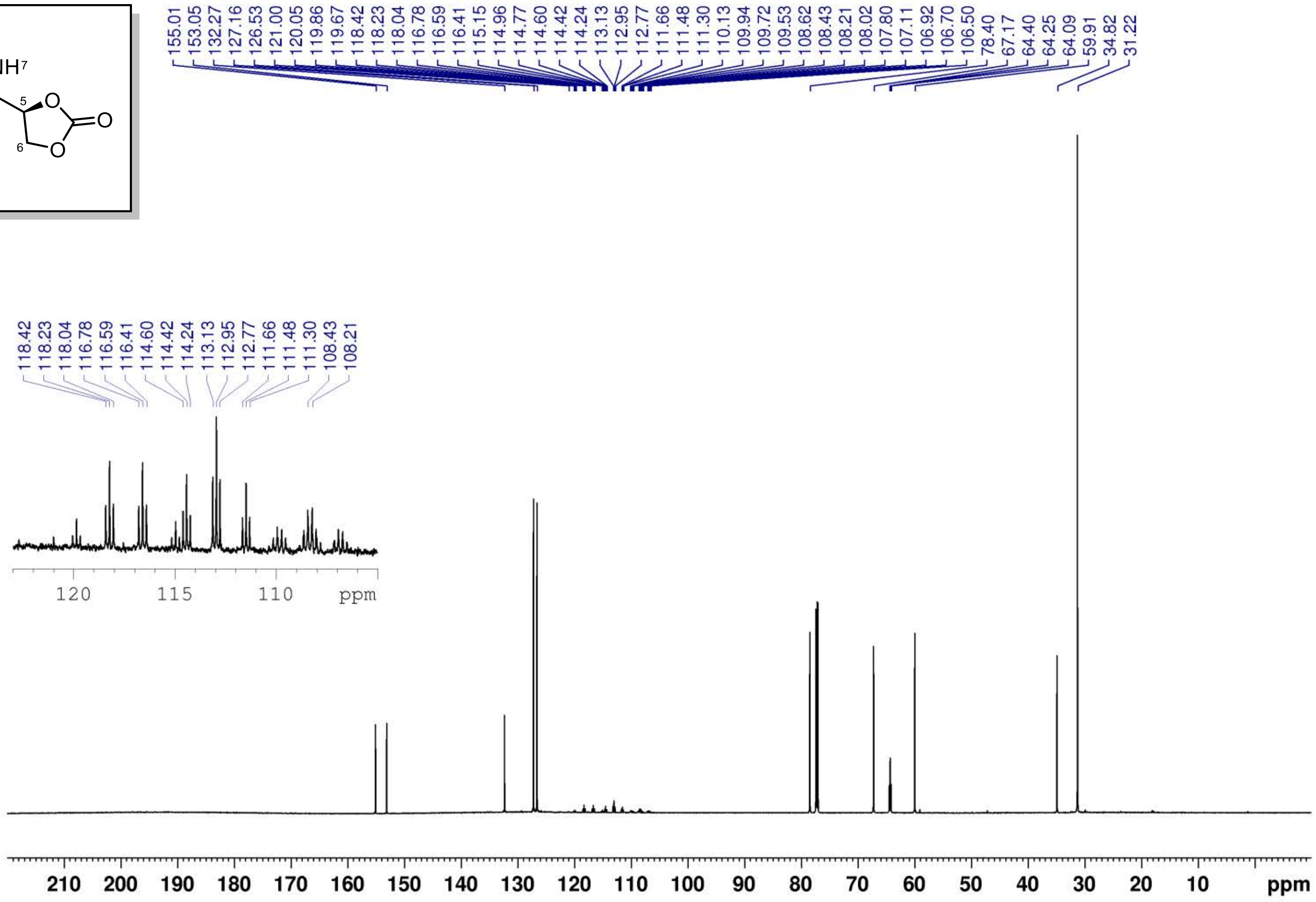

¹⁹F NMR (376 MHz, CDCl₃) for 2,2,3,3,4,4,4-heptafluorobutyl ((*S*)-(4-(*tert*-butyl)phenyl)((*R*)-2-oxo-1,3-dioxolan-4-yl)methyl)sulfamate (**4l**)

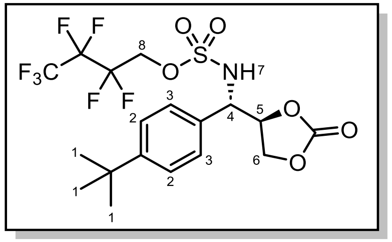

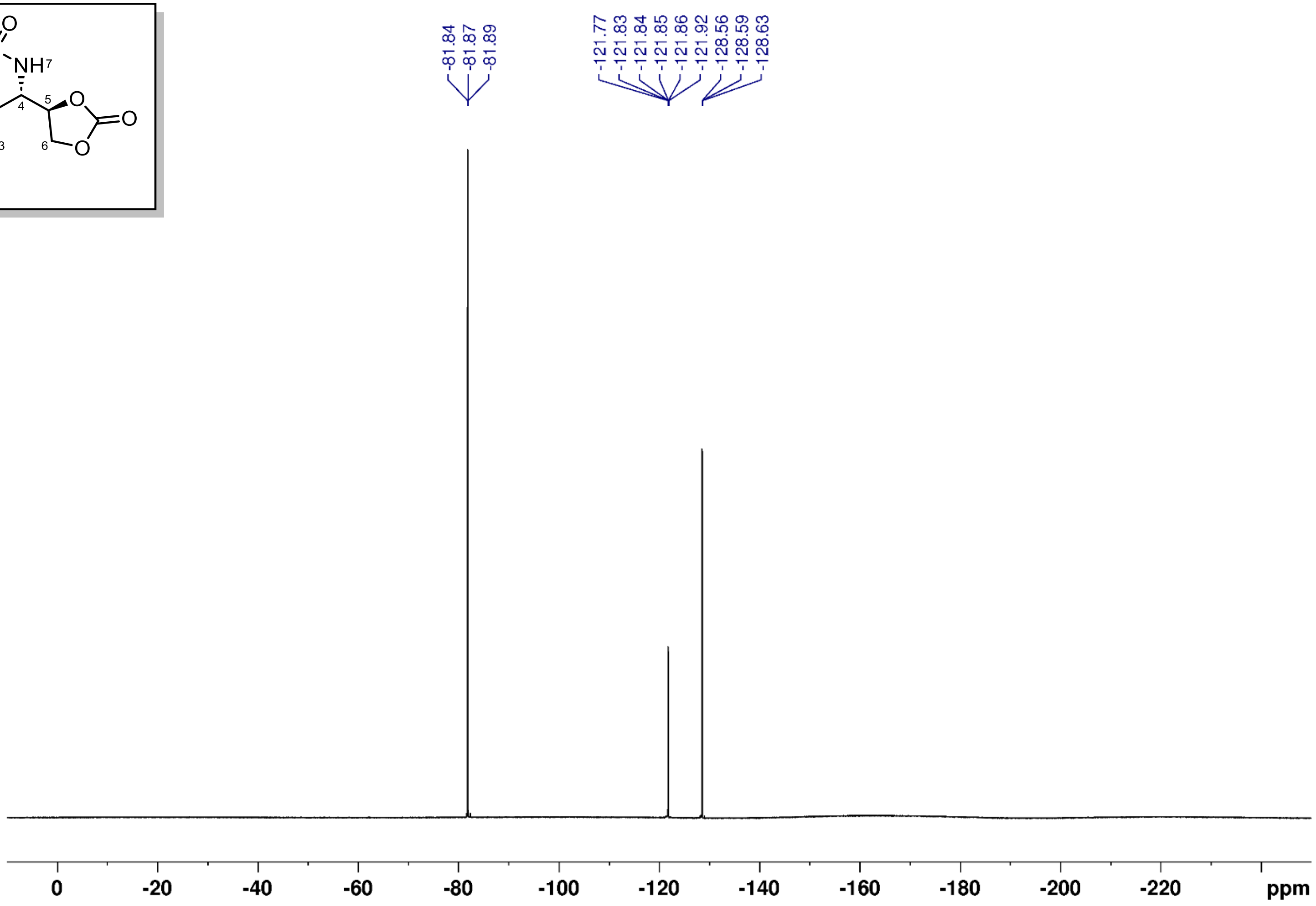

^{1}H NMR (700 MHz, CD_3CN) for 2,2,3,3,4,4,4-heptafluorobutyl ((*S*)-((*R*)-2-oxo-1,3-dioxolan-4-yl)(4-(trifluoromethoxy)phenyl)methyl)sulfamate (**4m**)

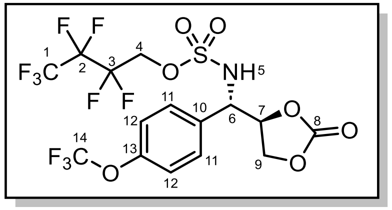

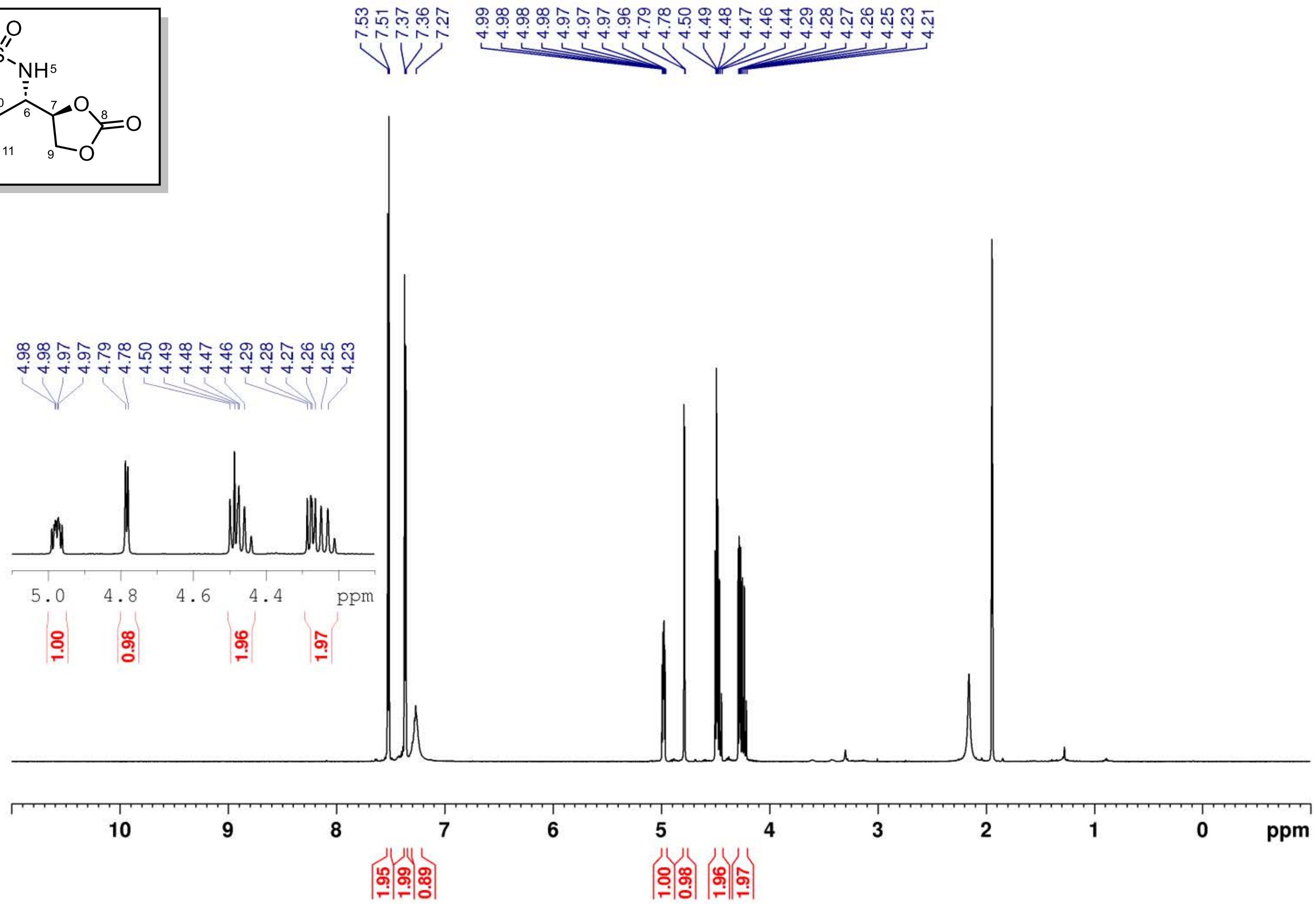

^{13}C NMR (176 MHz, CD_3CN) for 2,2,3,3,4,4,4-heptafluorobutyl ((*S*)-((*R*)-2-oxo-1,3-dioxolan-4-yl)(4-(trifluoromethoxy)phenyl)methyl)sulfamate (**4m**)

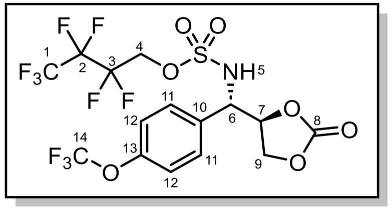

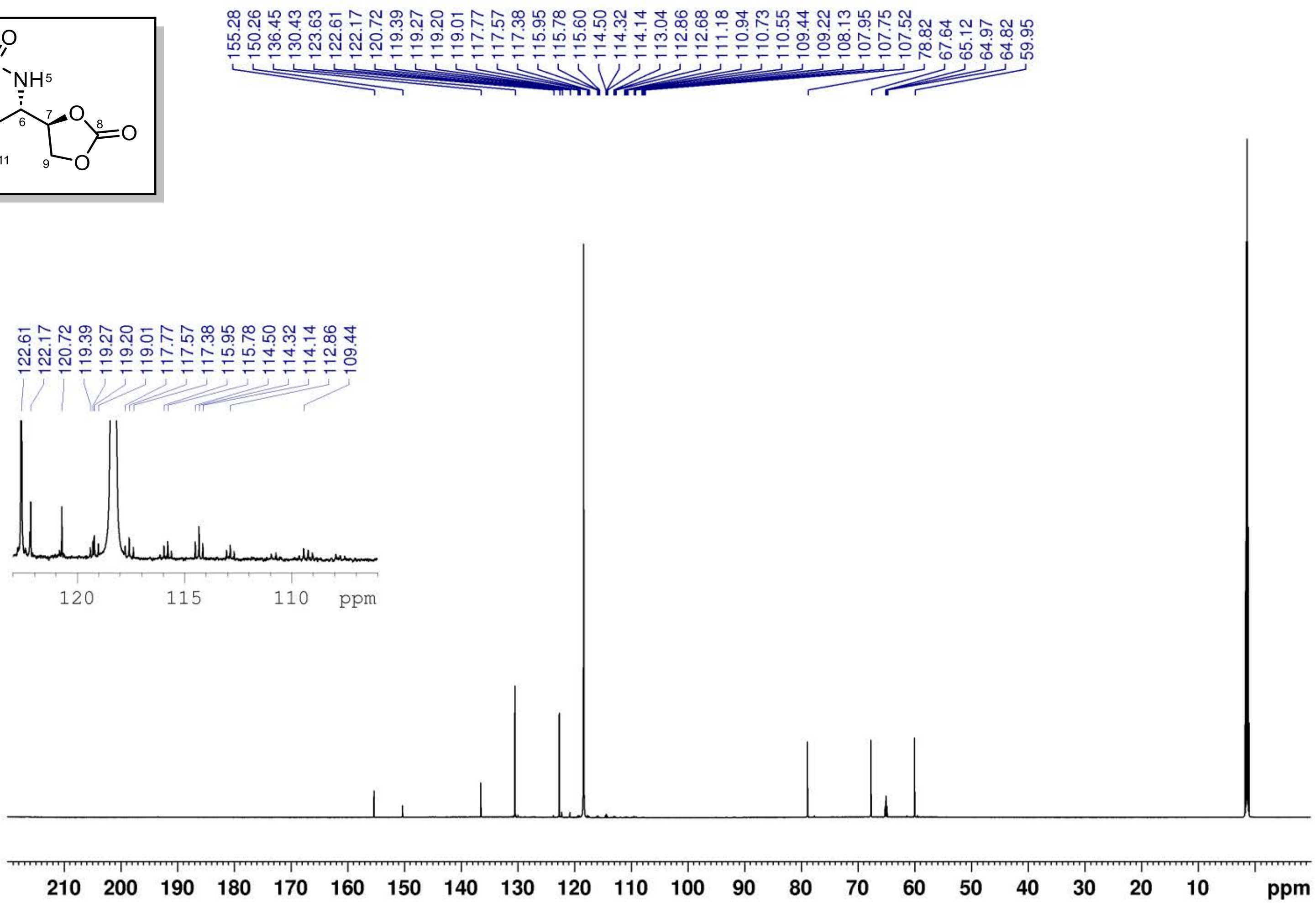

^{19}F NMR (376 MHz, CD_3CN) for 2,2,3,3,4,4,4-heptafluorobutyl ((*S*)-((*R*)-2-oxo-1,3-dioxolan-4-yl)(4-(trifluoromethoxy)phenyl)methyl)sulfamate (**4m**)

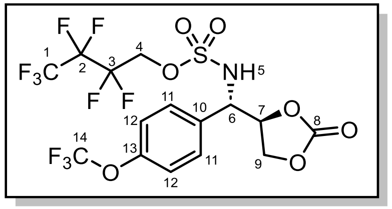

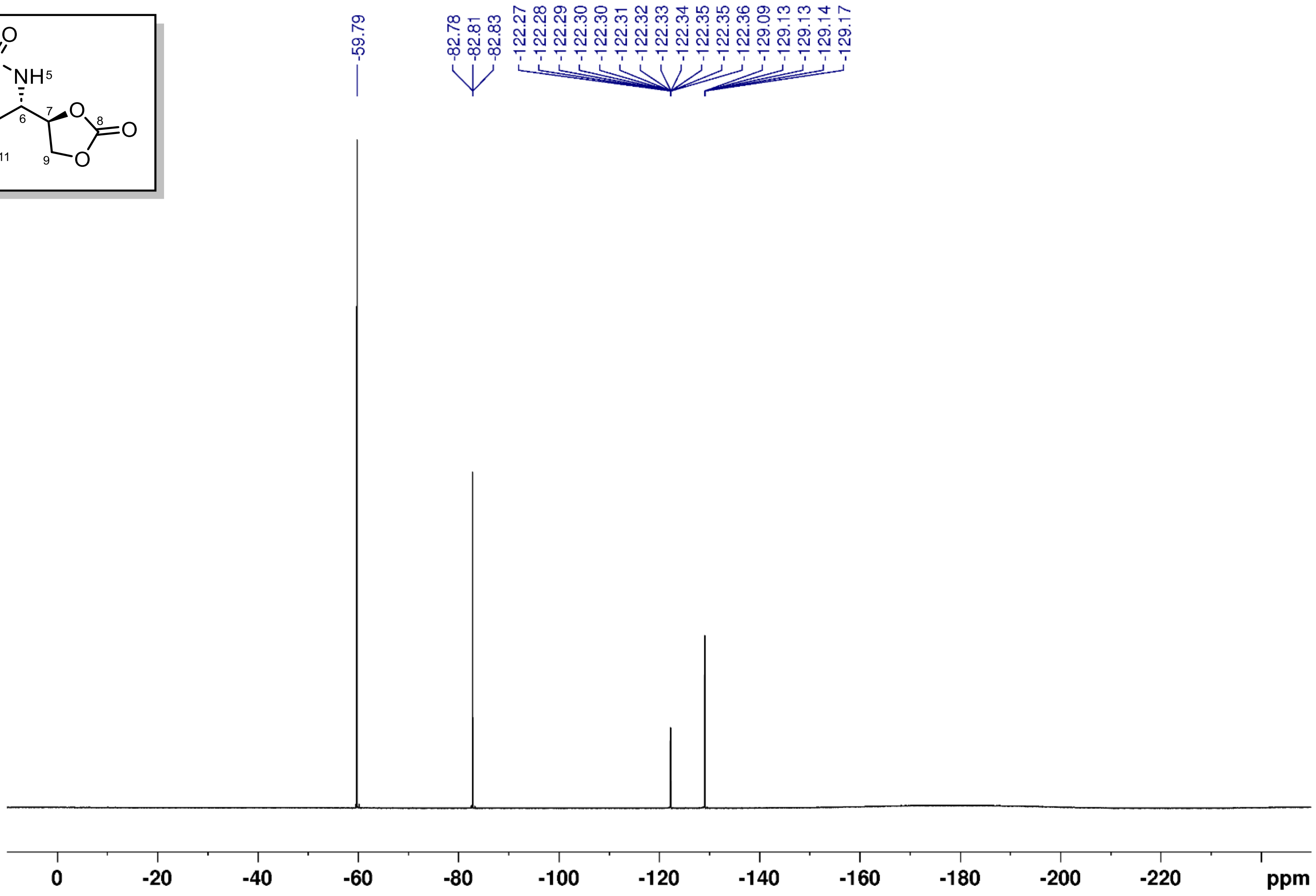

^{1}H NMR (700 MHz, $CDCl_3$) for 2,2,3,3,4,4,4-heptafluorobutyl ((*S*)-(4-chlorophenyl)((*R*)-2-oxo-1,3-dioxolan-4-yl)methyl)sulfamate (**4n**)

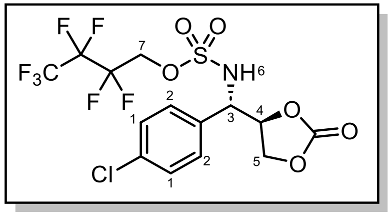

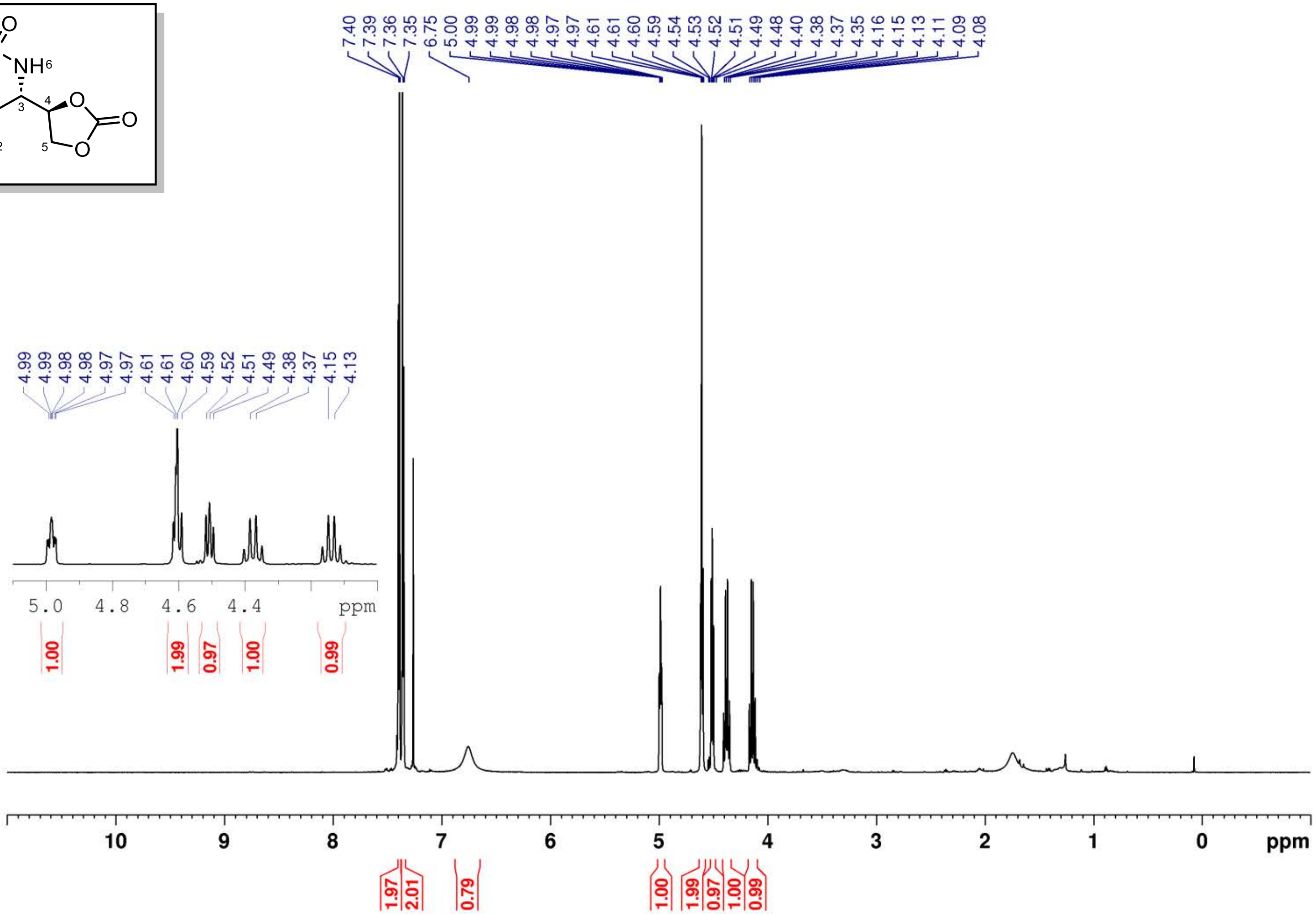

^{13}C NMR (176 MHz, CDCl$_3$) for 2,2,3,3,4,4,4-heptafluorobutyl ((*S*)-(4-chlorophenyl)((*R*)-2-oxo-1,3-dioxolan-4-yl)methyl)sulfamate (**4n**)

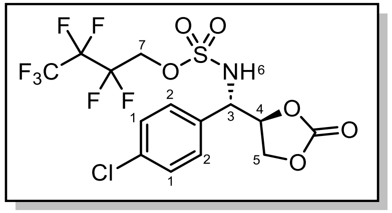

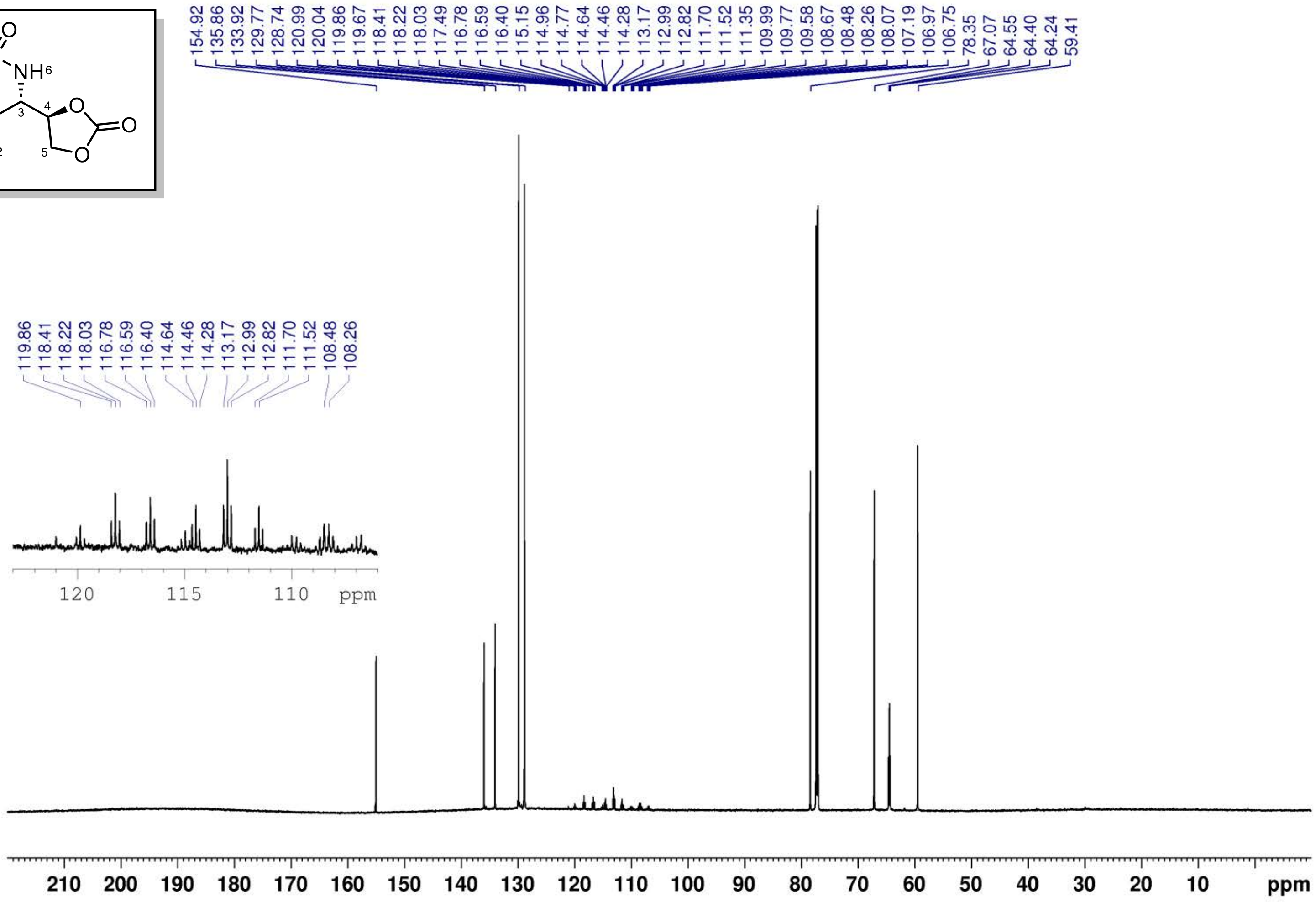

^{19}F NMR (376 MHz, CDCl$_3$) for 2,2,3,3,4,4,4-heptafluorobutyl ((*S*)-(4-chlorophenyl)((*R*)-2-oxo-1,3-dioxolan-4-yl)methyl)sulfamate (**4n**)

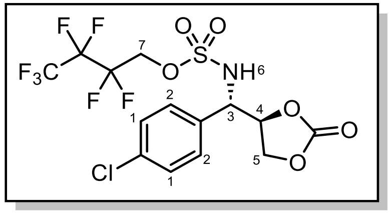

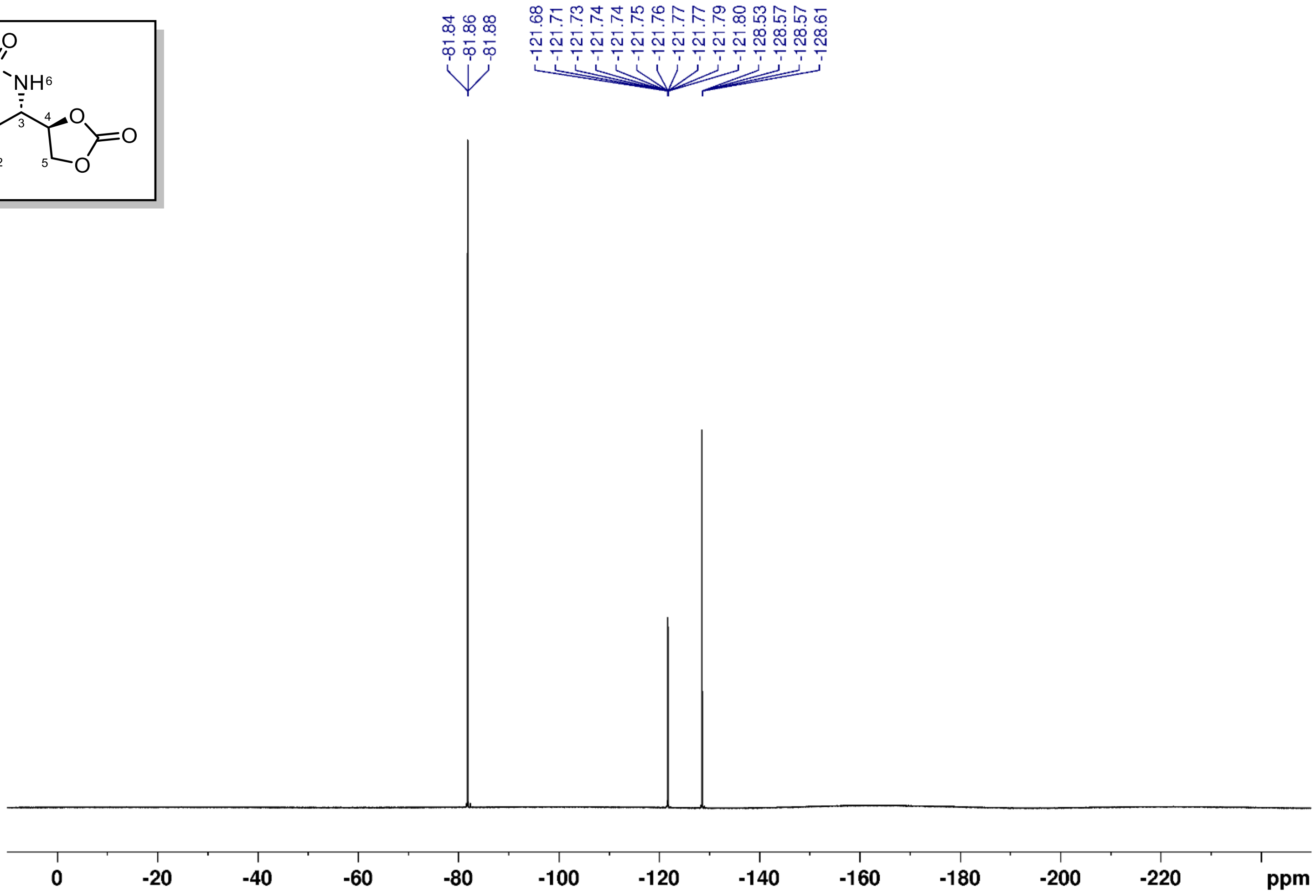

^{1}H NMR (700 MHz, CDCl$_3$) for 2,2,3,3,4,4,4-heptafluorobutyl ((*S*)-((*R*)-2-oxo-1,3-dioxolan-4-yl)(4-(trifluoromethyl)phenyl)methyl)sulfamate (**4o**)

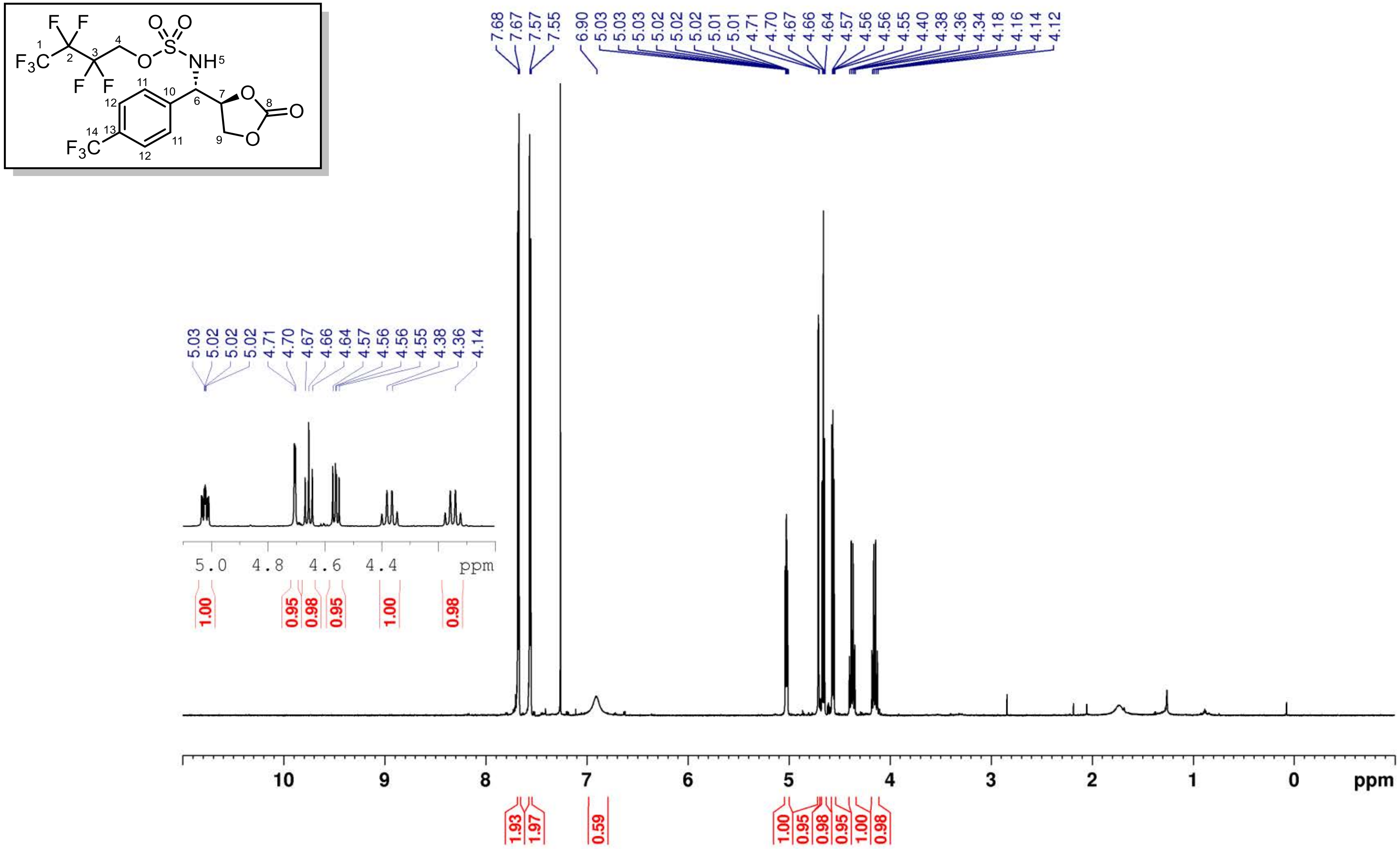

^{13}C NMR (176 MHz, CDCl$_3$) for 2,2,3,3,4,4,4-heptafluorobutyl ((*S*)-((*R*)-2-oxo-1,3-dioxolan-4-yl)(4-(trifluoromethyl)phenyl)methyl)sulfamate (**4o**)

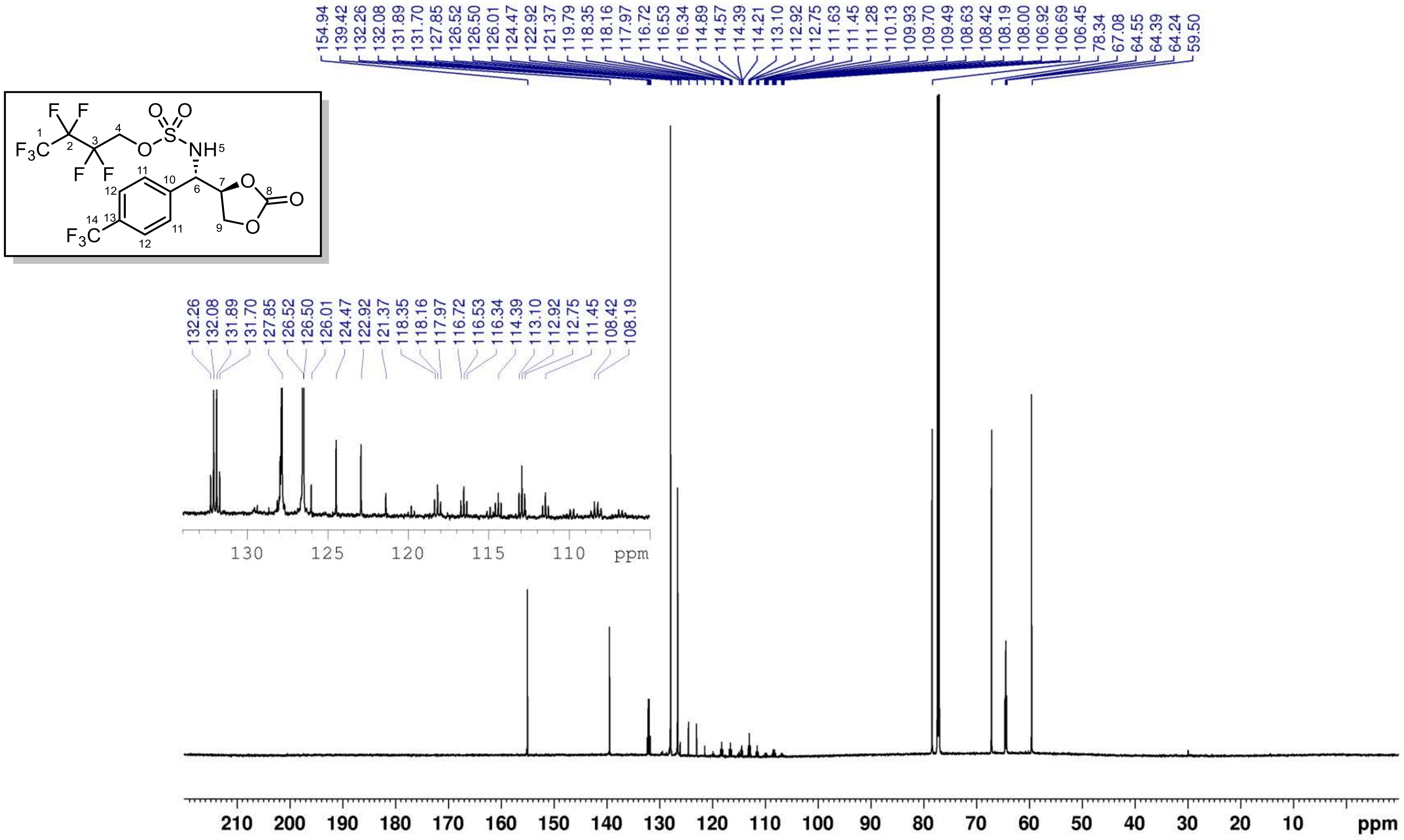

^{19}F NMR (376 MHz, $CDCl_3$) for 2,2,3,3,4,4,4-heptafluorobutyl ((*S*)-((*R*)-2-oxo-1,3-dioxolan-4-yl)(4-(trifluoromethyl)phenyl)methyl)sulfamate (**4o**)

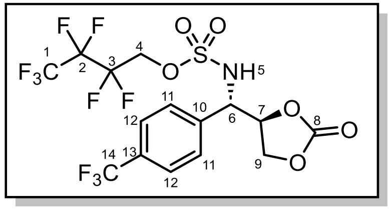

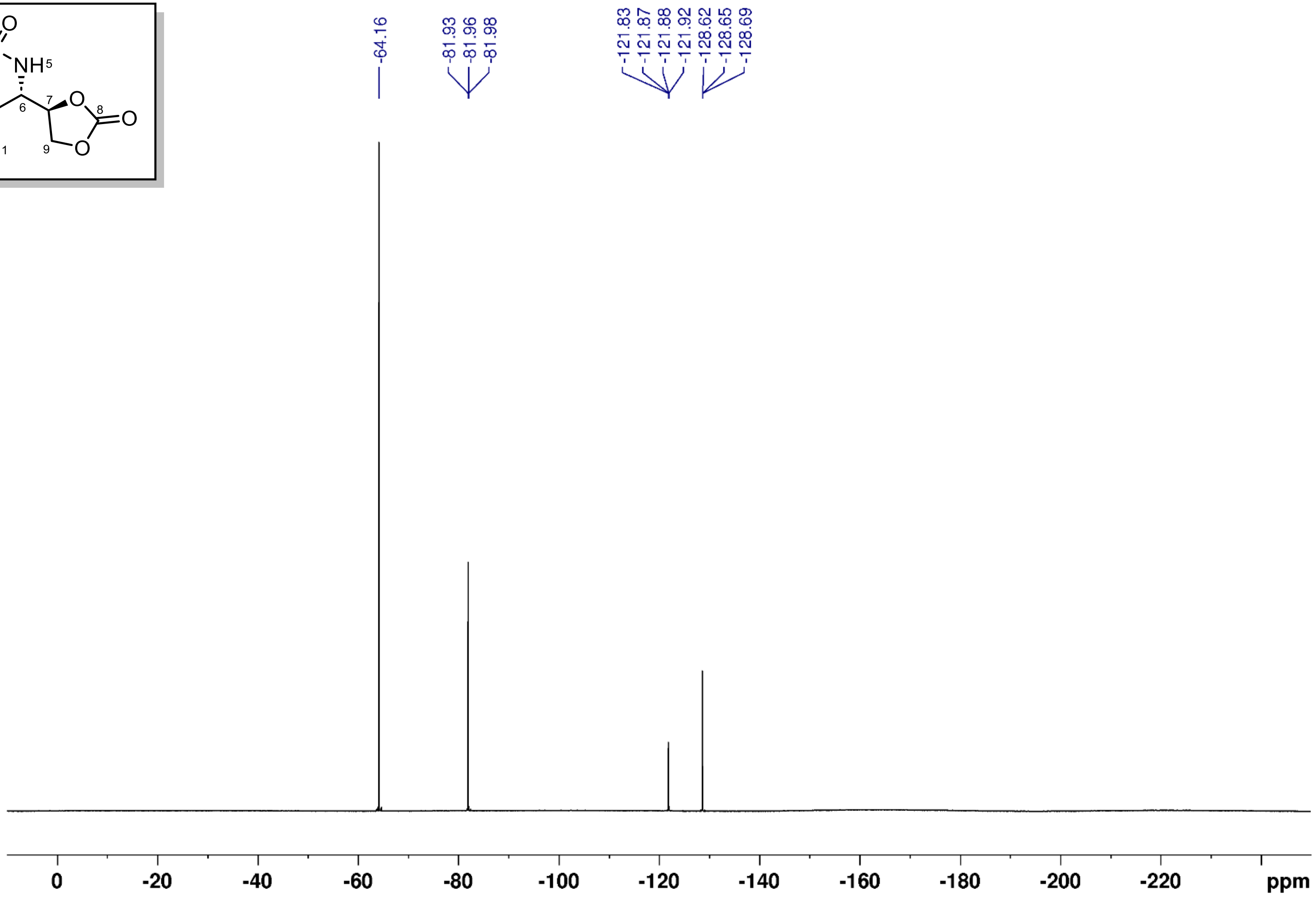

^{1}H NMR (700 MHz, CDCl$_3$) for 2,2,3,3,4,4,4-heptafluorobutyl ((*S*)-naphthalen-1-yl((*R*)-2-oxo-1,3-dioxolan-4-yl)methyl)sulfamate (**4p**)

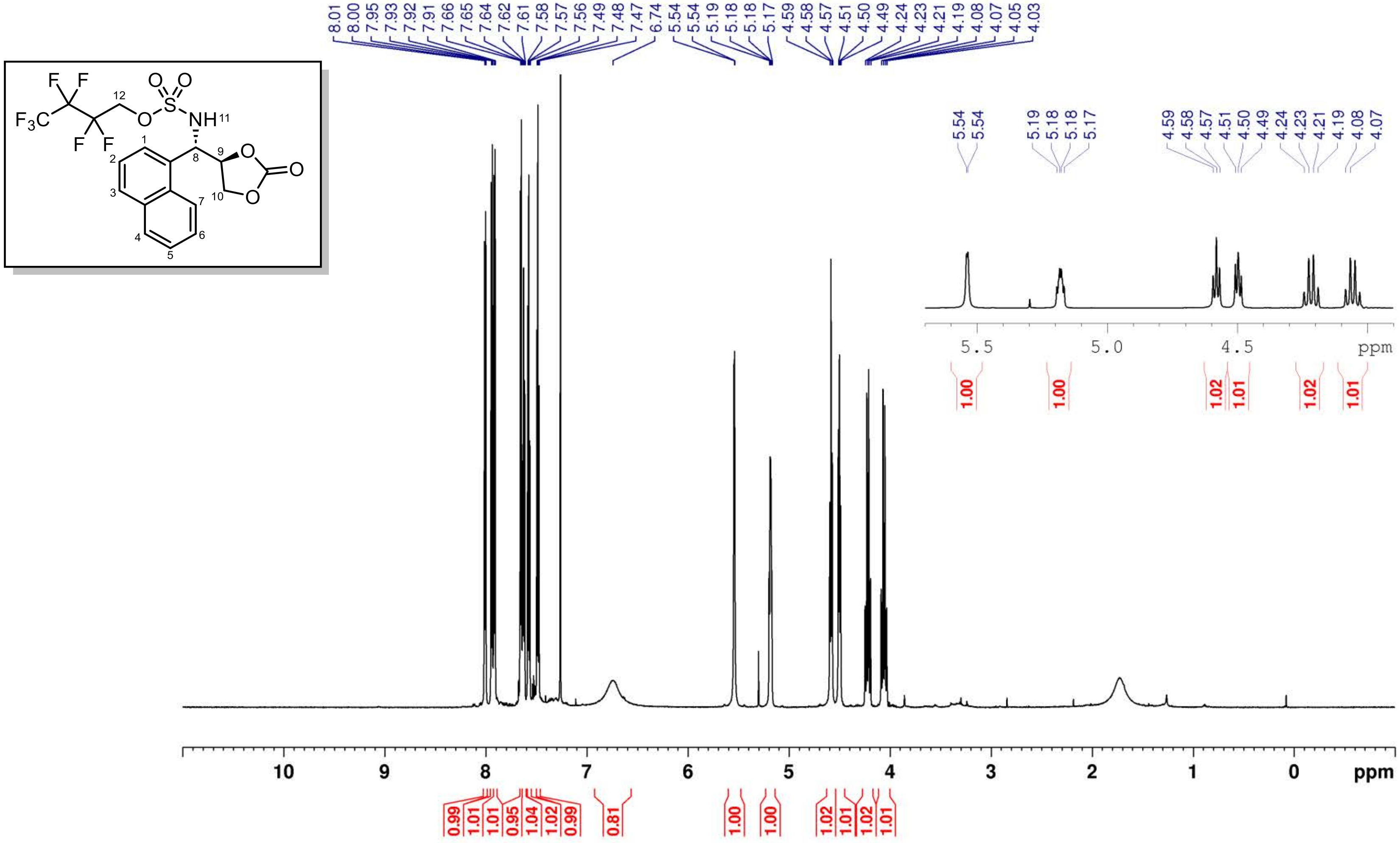

^{13}C NMR (176 MHz, CDCl$_3$) for 2,2,3,3,4,4,4-heptafluorobutyl ((*S*)-naphthalen-1-yl((*R*)-2-oxo-1,3-dioxolan-4-yl)methyl)sulfamate (**4p**)

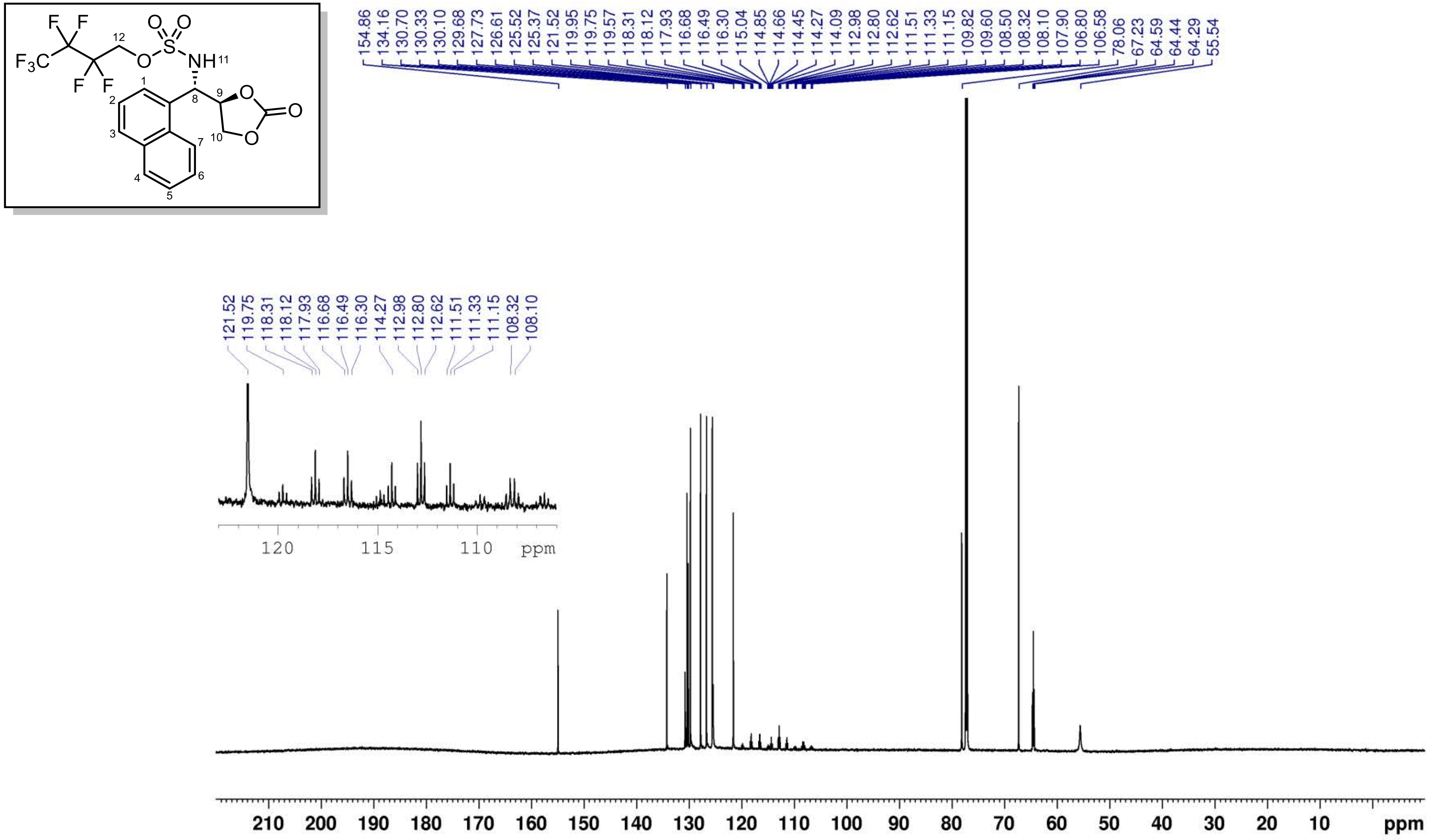

^{19}F NMR (376 MHz, $CDCl_3$) for 2,2,3,3,4,4,4-heptafluorobutyl ((*S*)-naphthalen-1-yl((*R*)-2-oxo-1,3-dioxolan-4-yl)methyl)sulfamate (**4p**)

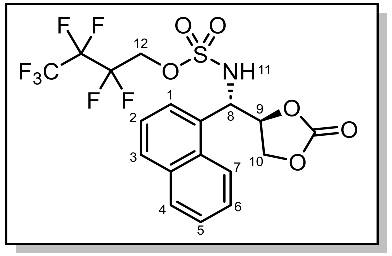

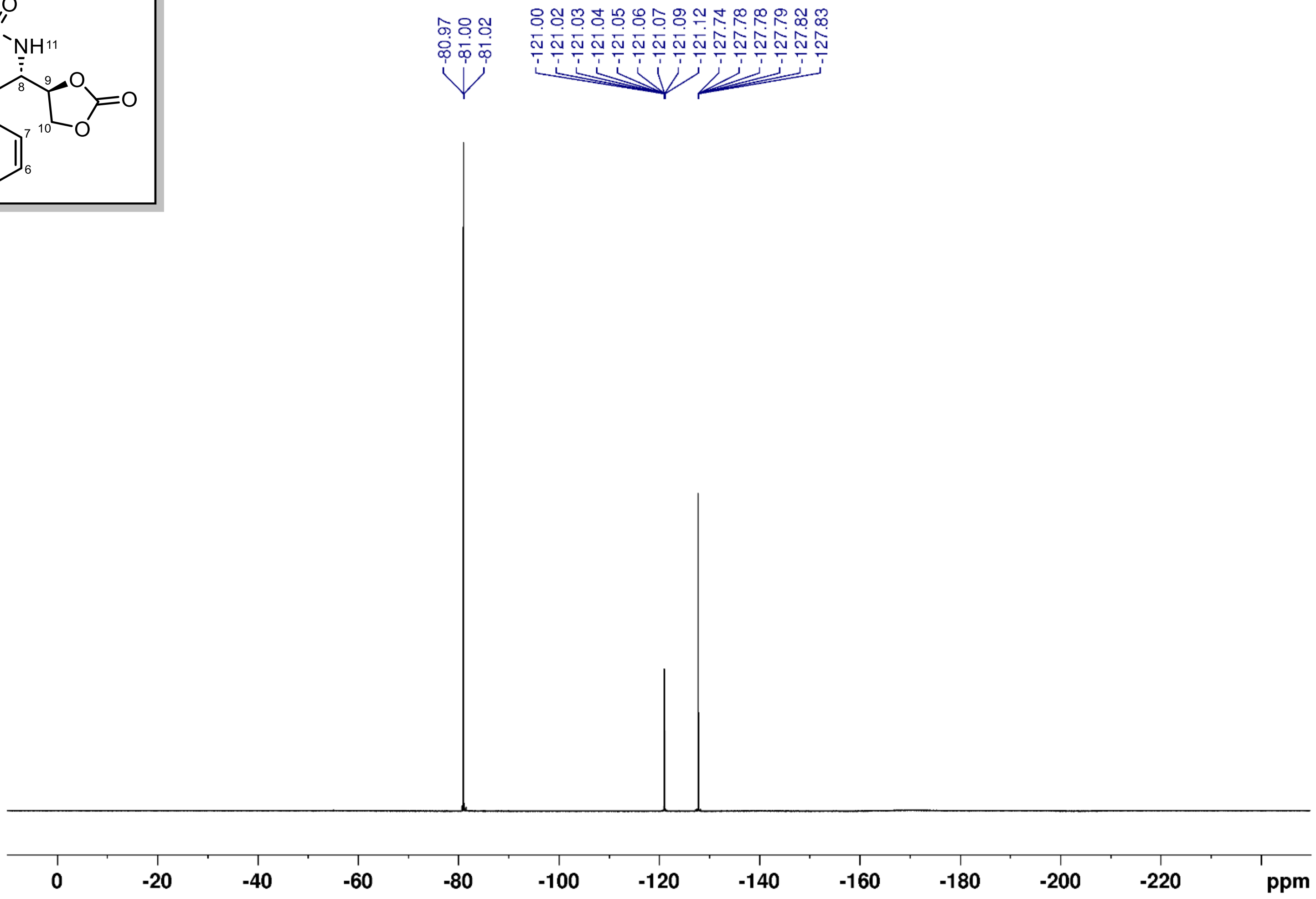

^{1}H NMR (700 MHz, $CDCl_3$) for 2,2,3,3,4,4,4-heptafluorobutyl ((*S*)-naphthalen-2-yl((*R*)-2-oxo-1,3-dioxolan-4-yl)methyl)sulfamate (**4q**)

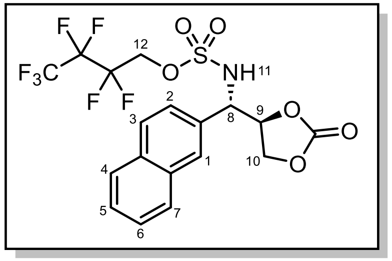

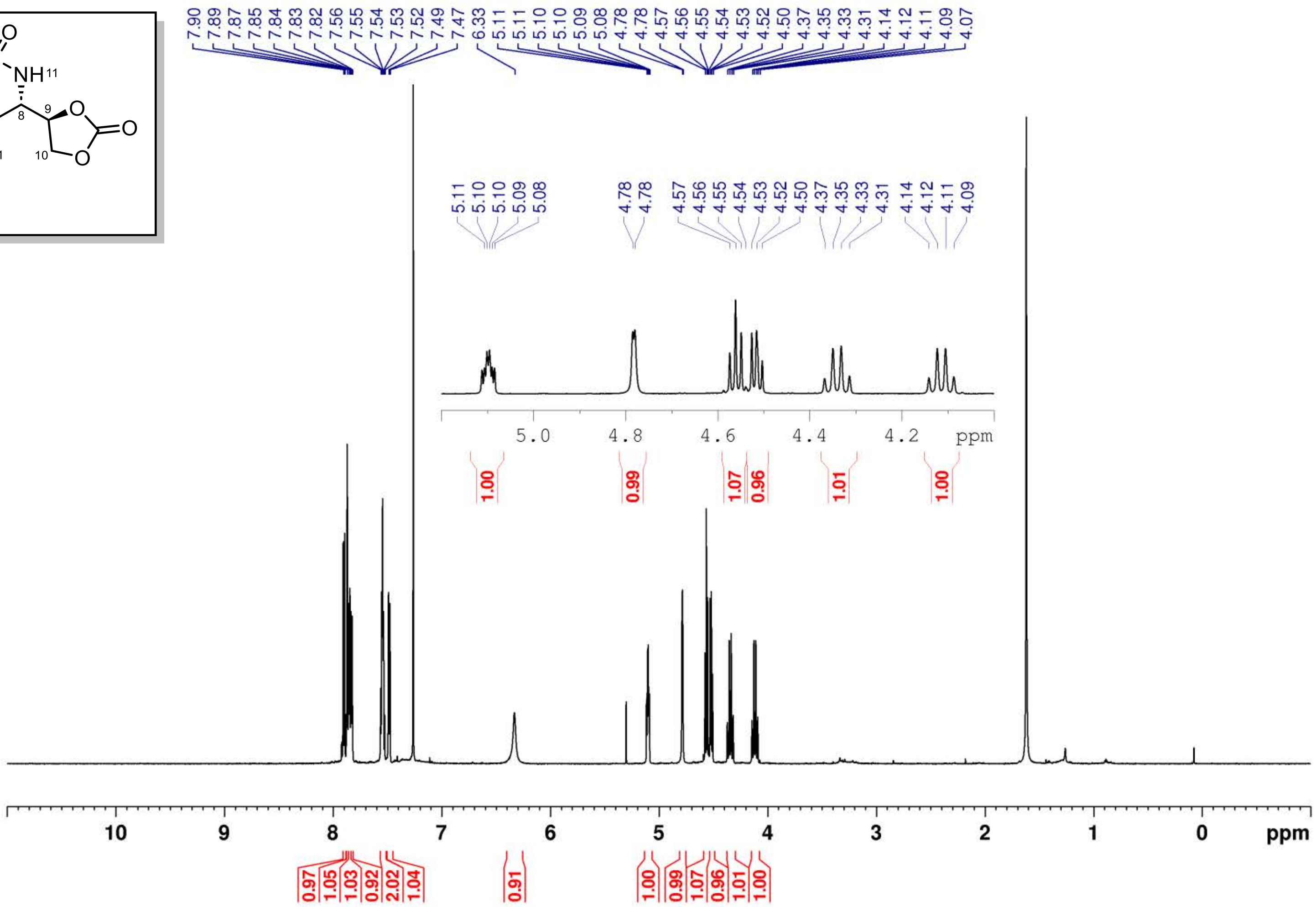

^{13}C NMR (176 MHz, CDCl$_3$) for 2,2,3,3,4,4,4-heptafluorobutyl ((*S*)-naphthalen-2-yl((*R*)-2-oxo-1,3-dioxolan-4-yl)methyl)sulfamate (**4q**)

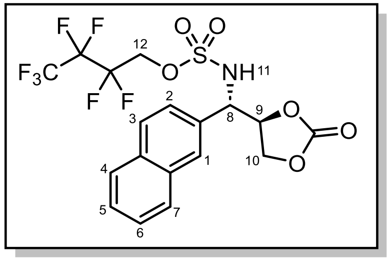

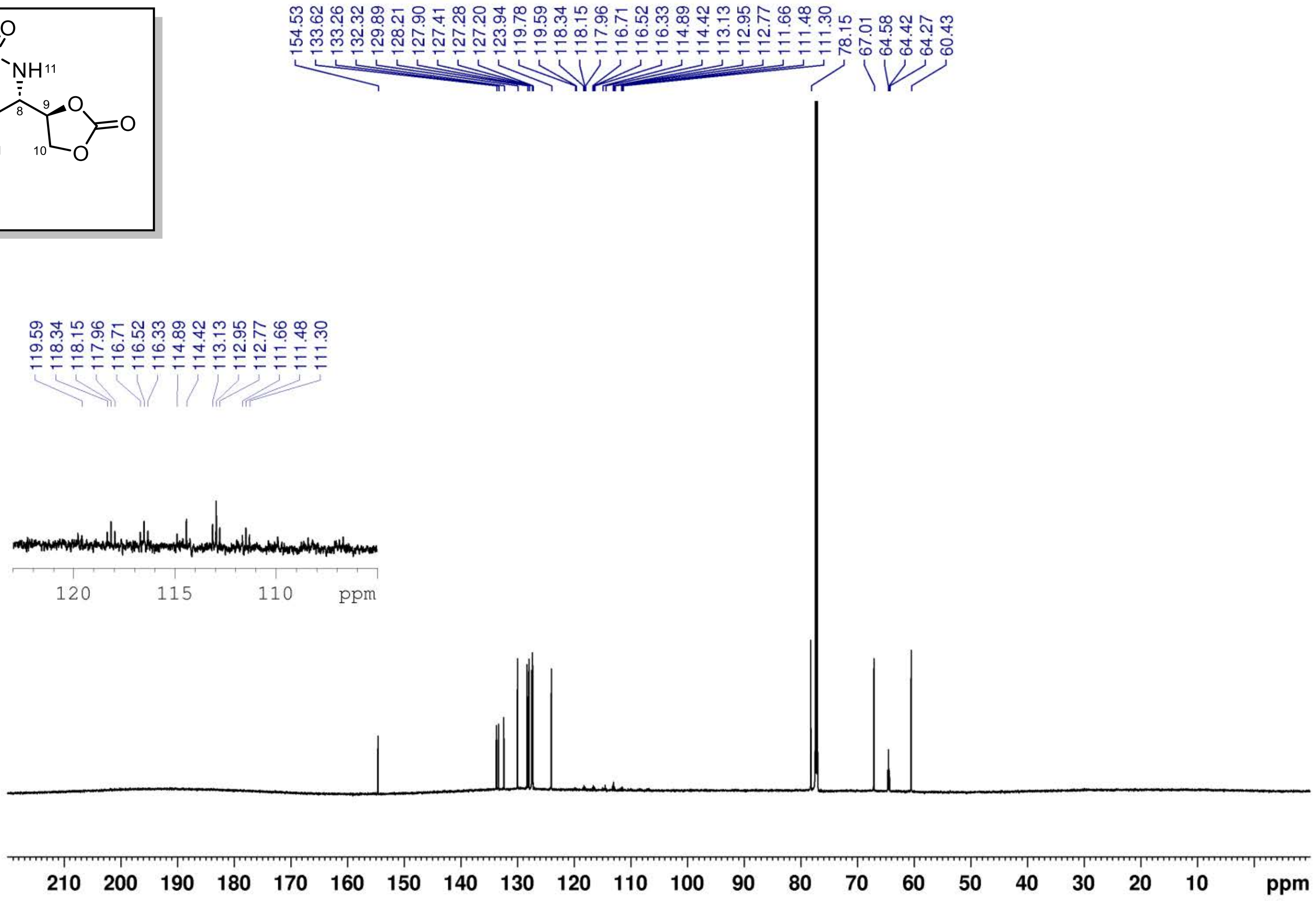

^{19}F NMR (376 MHz, CDCl$_3$) for 2,2,3,3,4,4,4-heptafluorobutyl ((*S*)-naphthalen-2-yl((*R*)-2-oxo-1,3-dioxolan-4-yl)methyl)sulfamate (**4q**)

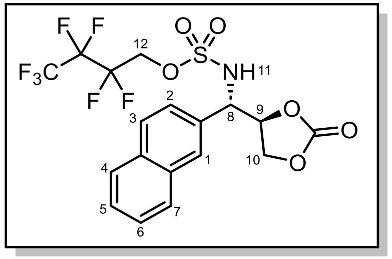

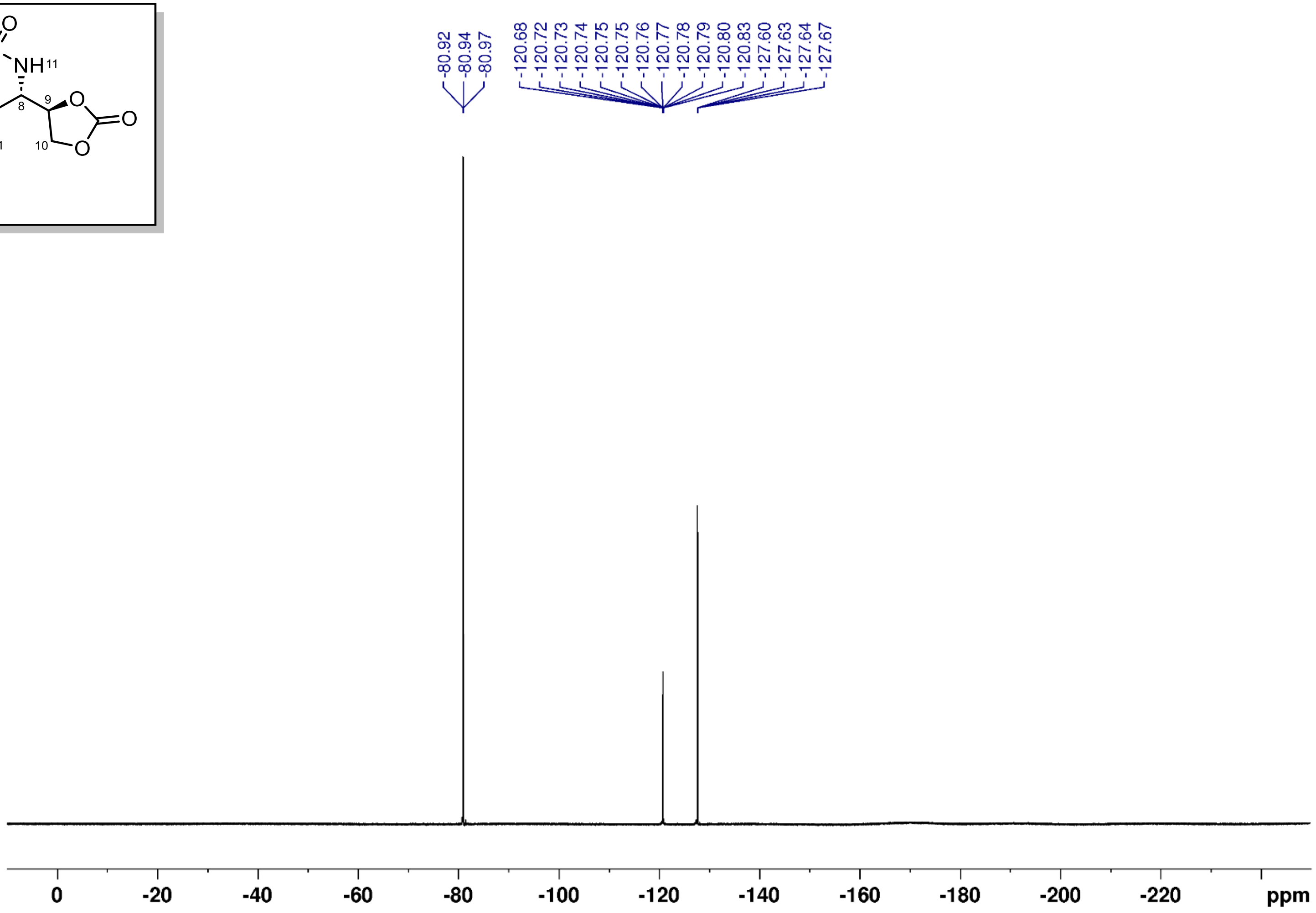

^{1}H NMR (700 MHz, CDCl$_3$) for 2,2,3,3,4,4,4-heptafluorobutyl ((*S*)-((*R*)-2-oxo-1,3-dioxolan-4-yl)(thiophen-3-yl)methyl)sulfamate (**4r**)

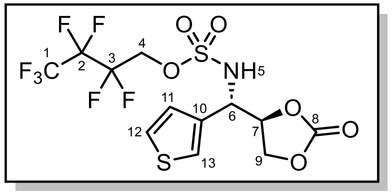

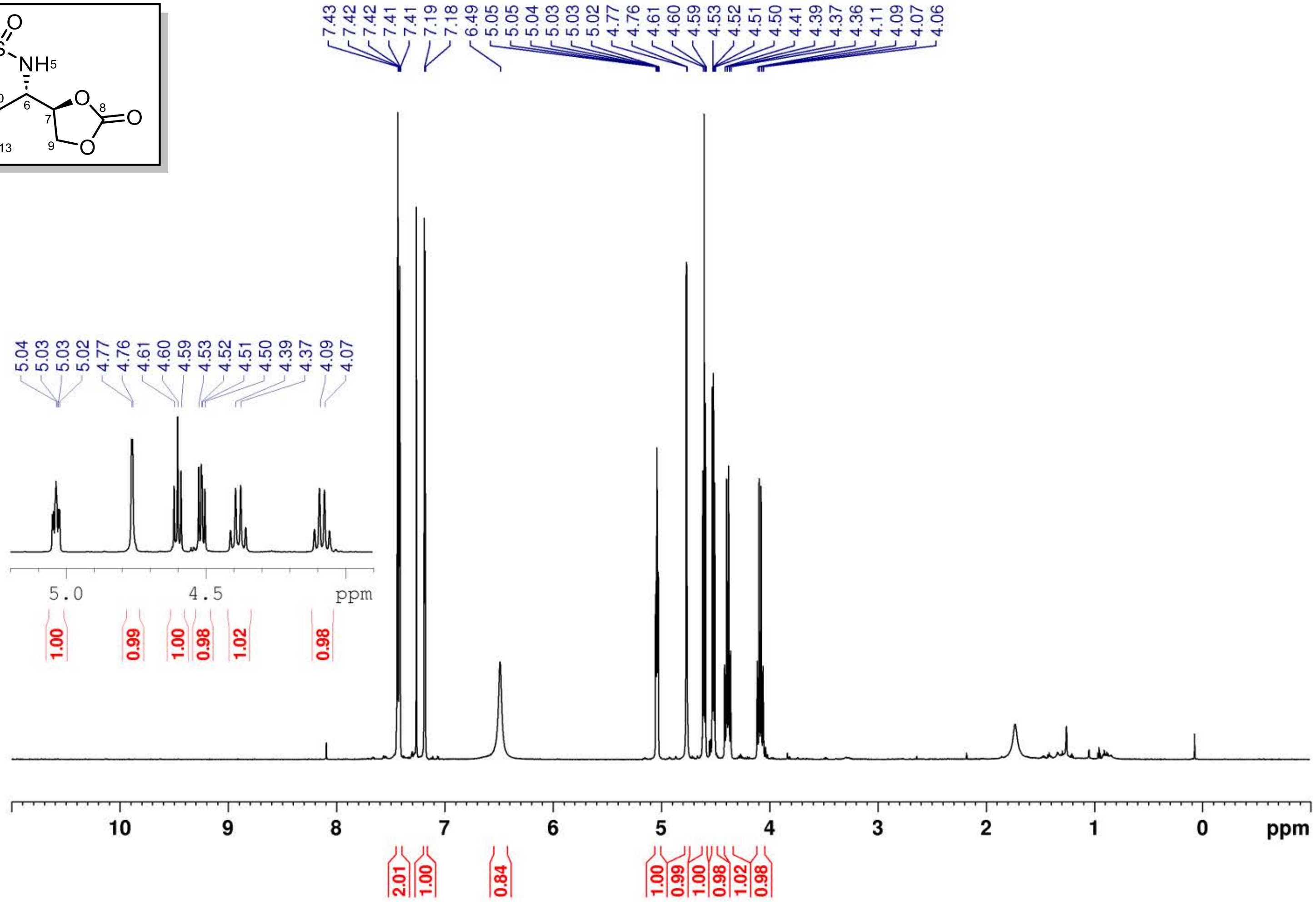

^{13}C NMR (176 MHz, $CDCl_3$) for 2,2,3,3,4,4,4-heptafluorobutyl ((*S*)-((*R*)-2-oxo-1,3-dioxolan-4-yl)(thiophen-3-yl)methyl)sulfamate (**4r**)

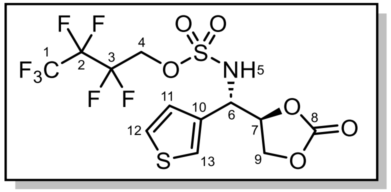

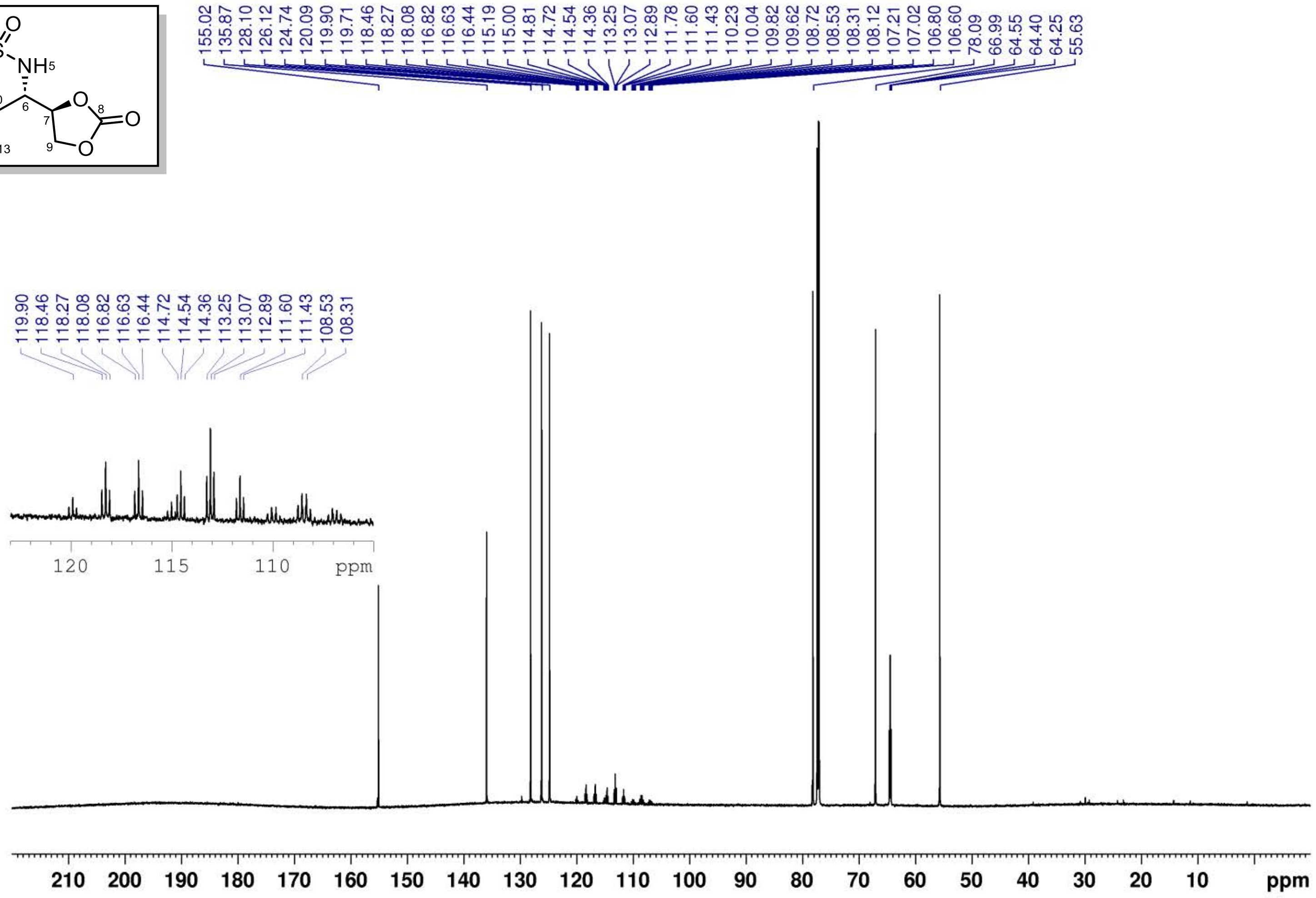

^{19}F NMR (376 MHz, CDCl$_3$) for 2,2,3,3,4,4,4-heptafluorobutyl ((*S*)-((*R*)-2-oxo-1,3-dioxolan-4-yl)(thiophen-3-yl)methyl)sulfamate (**4r**)

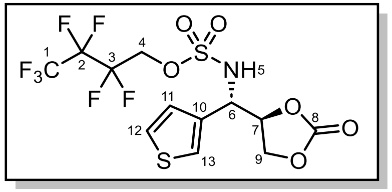

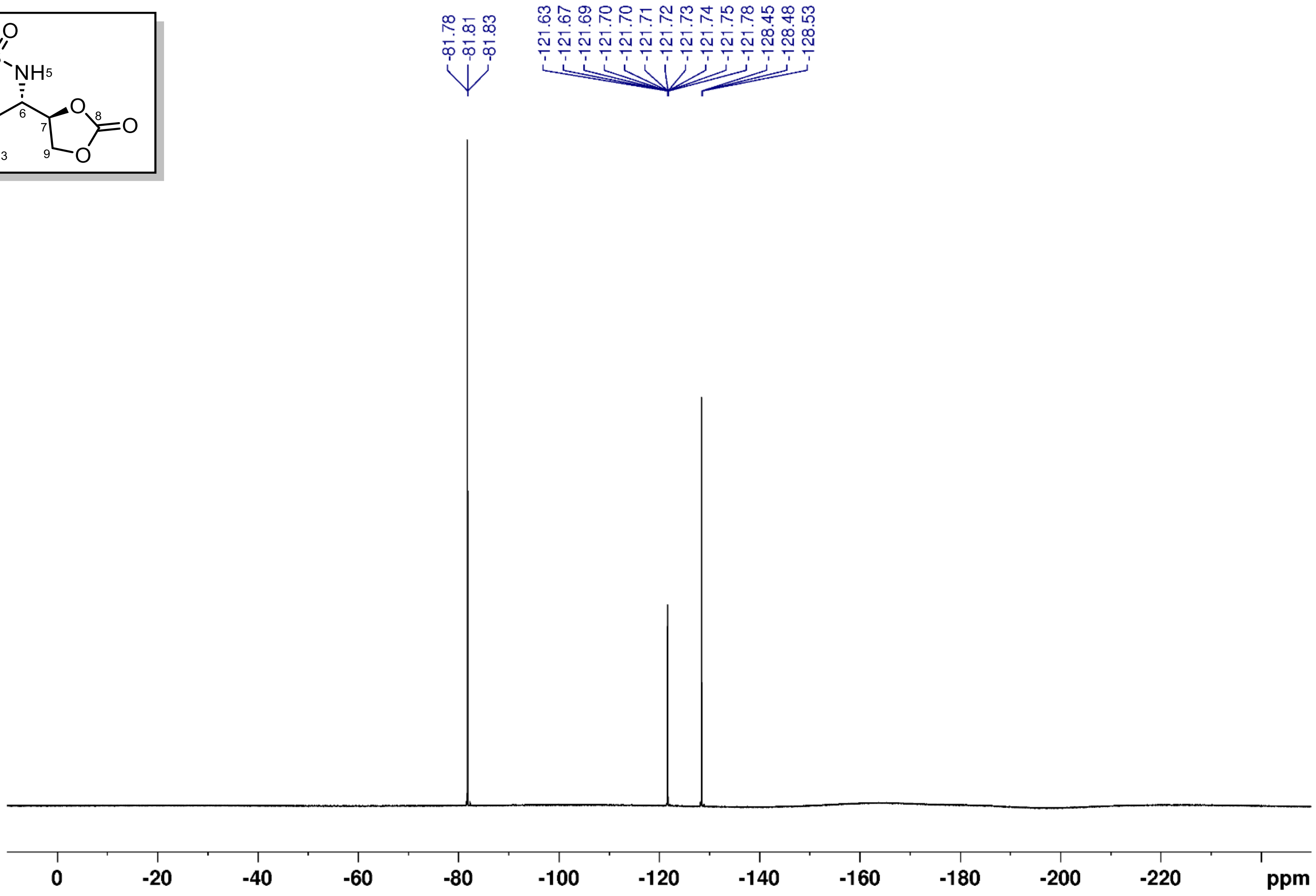

^{1}H NMR (700 MHz, CDCl$_3$) for methyl 5-((*S*)-(((2,2,3,3,4,4,4-heptafluorobutoxy)sulfonyl)amino)((*R*)-2-oxo-1,3-dioxolan-4-yl)methyl)-1-tosyl-1H-indole-3-carboxylate (**4s**)

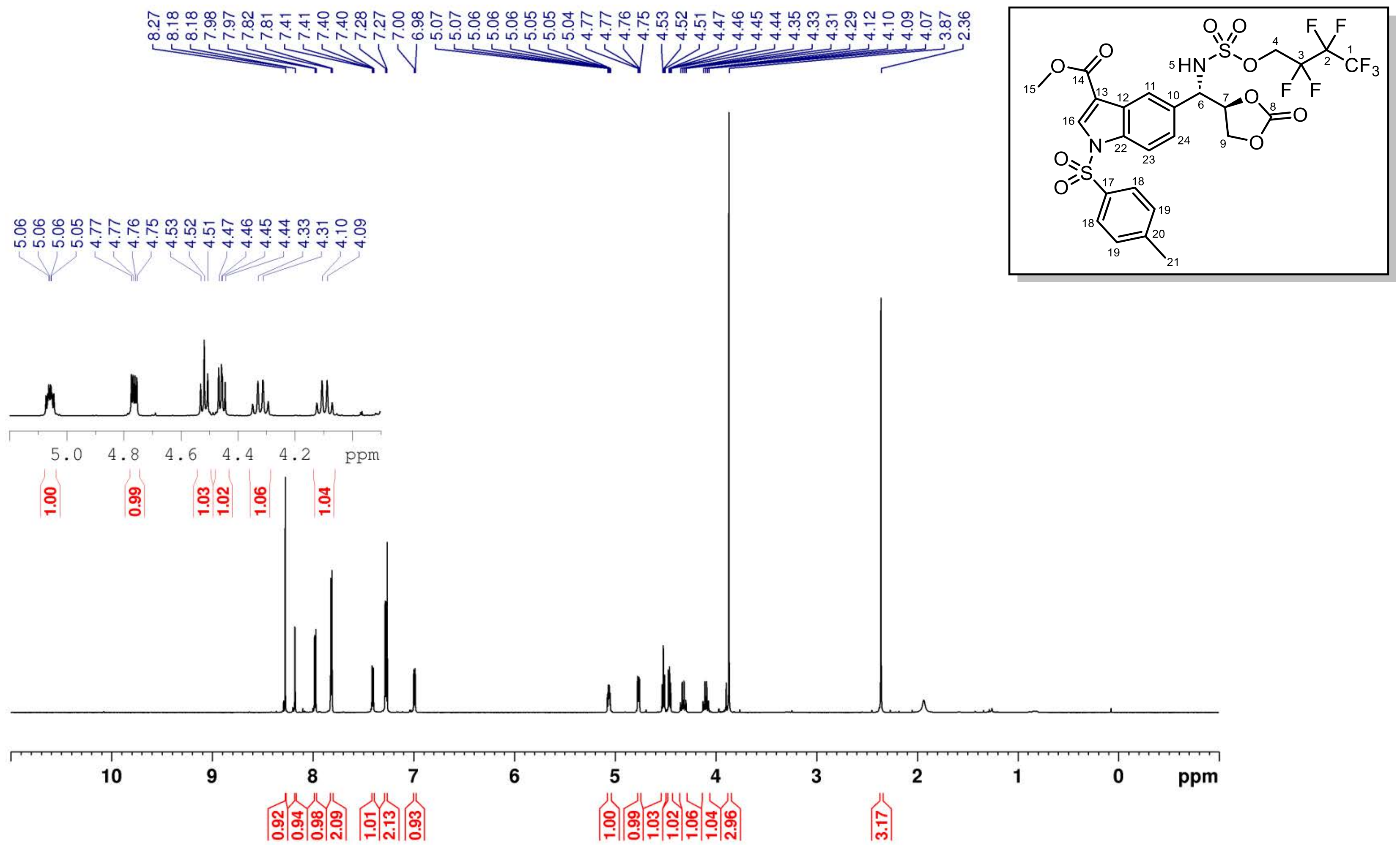

^{13}C NMR (176 MHz, CDCl$_3$) for methyl 5-((*S*)-(((2,2,3,3,4,4,4-heptafluorobutoxy)sulfonyl)amino)((*R*)-2-oxo-1,3-dioxolan-4-yl)methyl)-1-tosyl-1H-indole-3-carboxylate (**4s**)

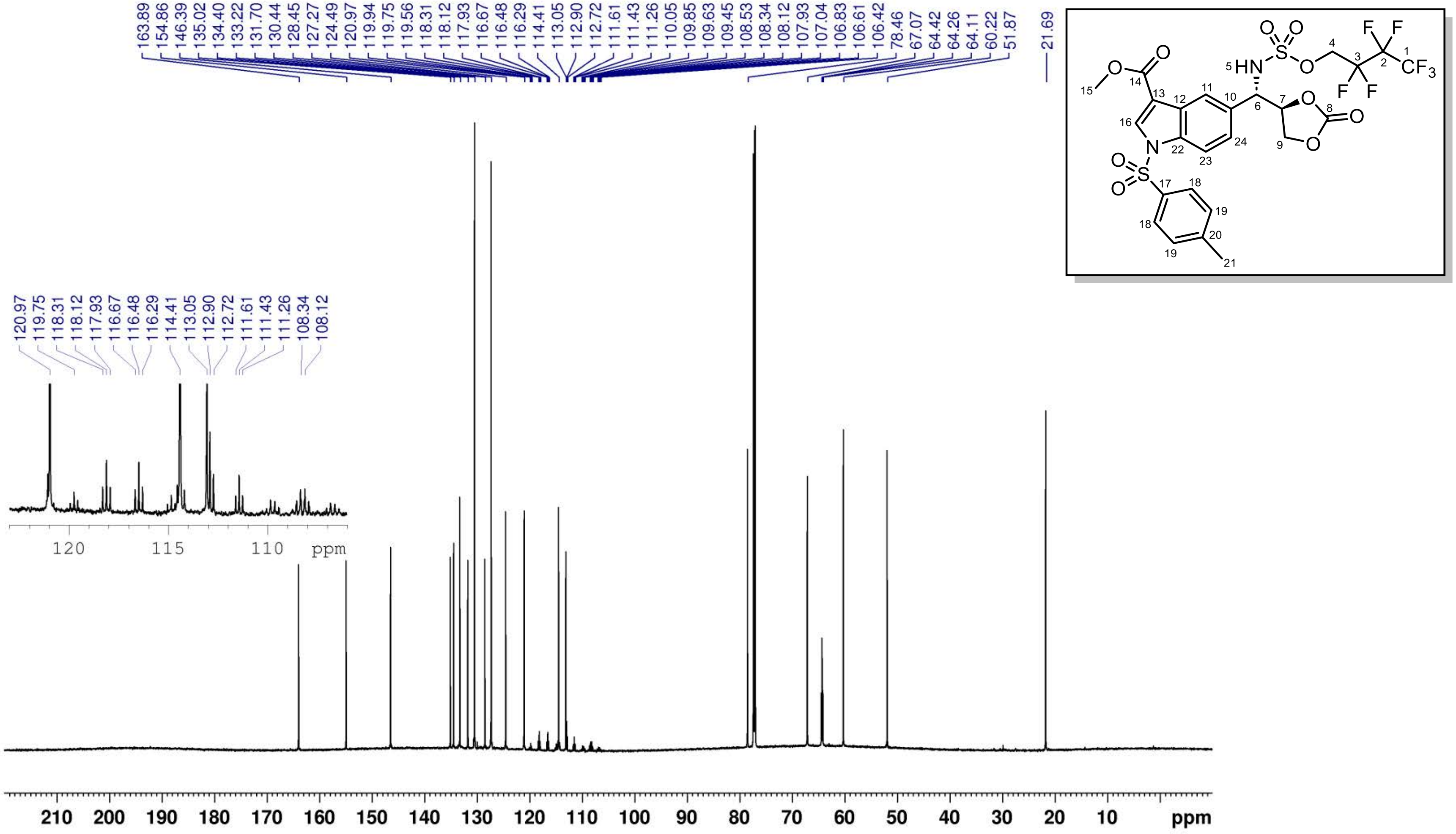

^{19}F NMR (376 MHz, CDCl$_3$) for methyl 5-((*S*)-(((2,2,3,3,4,4,4-heptafluorobutoxy)sulfonyl)amino)((*R*)-2-oxo-1,3-dioxolan-4-yl)methyl)-1-tosyl-1H-indole-3-carboxylate (**4s**)

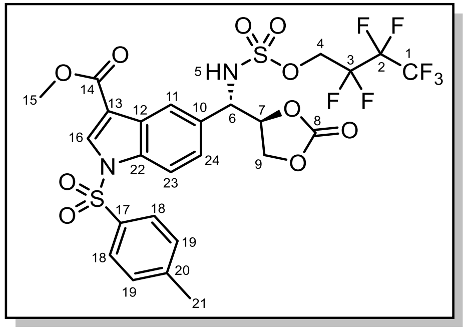

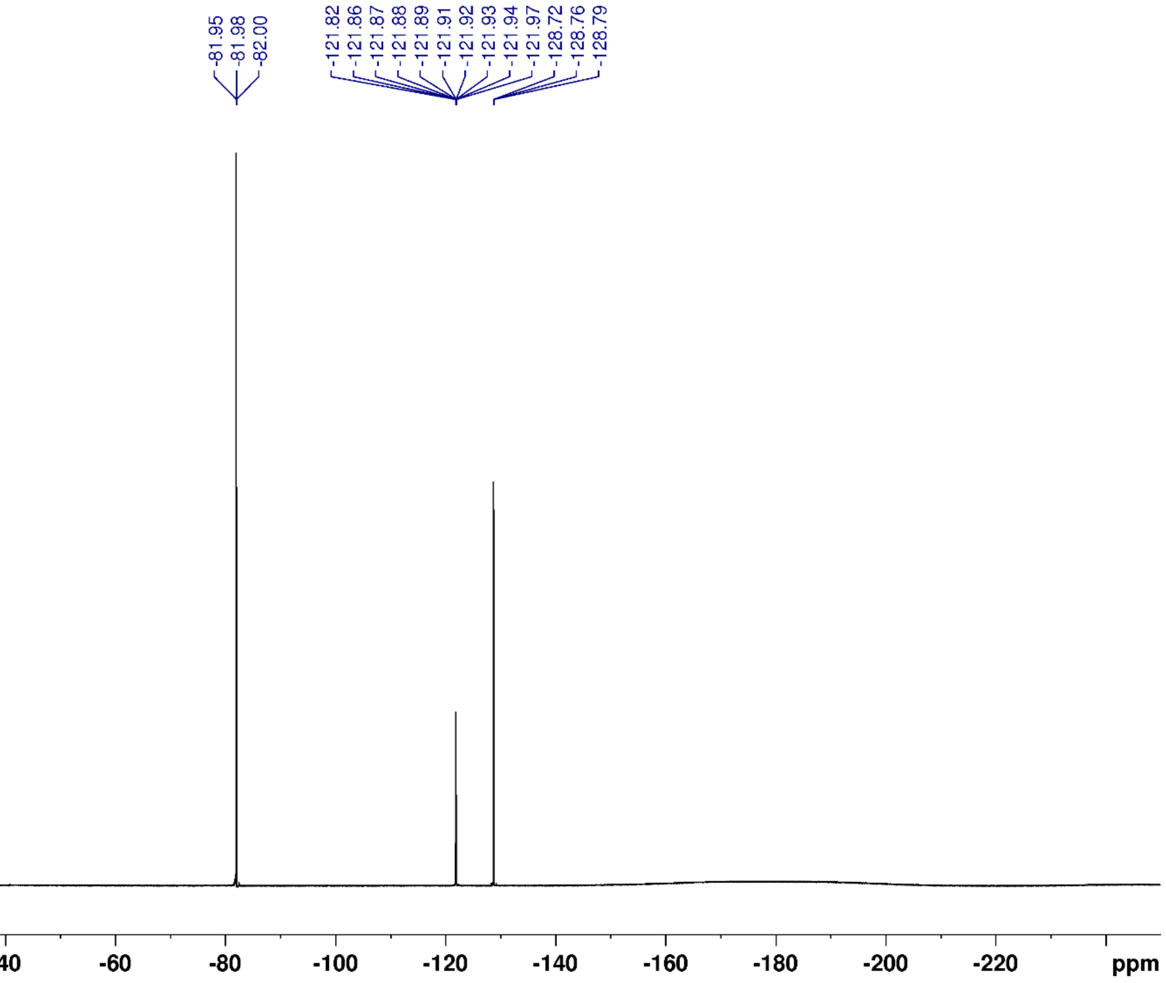

^{1}H NMR (400 MHz, CDCl$_3$) for (*R*,*Z*)-4-(4-(*tert*-butyl)phenyl)but-3-en-2-yl methyl(phenyl)carbamate ((***R***)-**1t**)

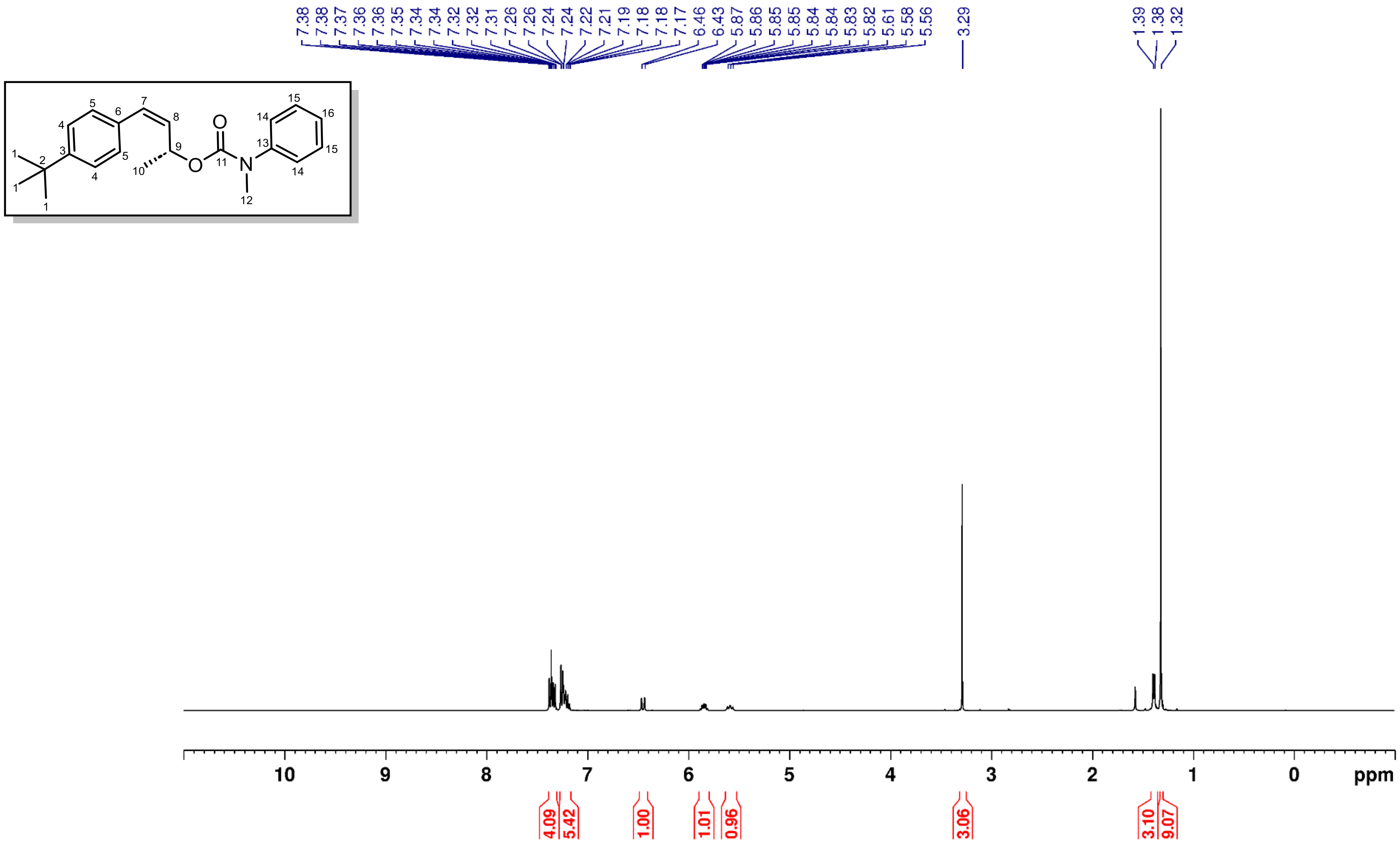

^{1}H NMR (500 MHz, CDCl$_3$) for 2,2,3,3,4,4,4-heptafluorobutyl ((*S*)-(4-(tert-butyl)phenyl)((4*R*,5*S*)-5-methyl-2-oxo-1,3-dioxolan-4-yl)methyl)sulfamate (**4t**)

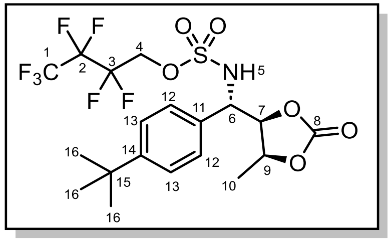

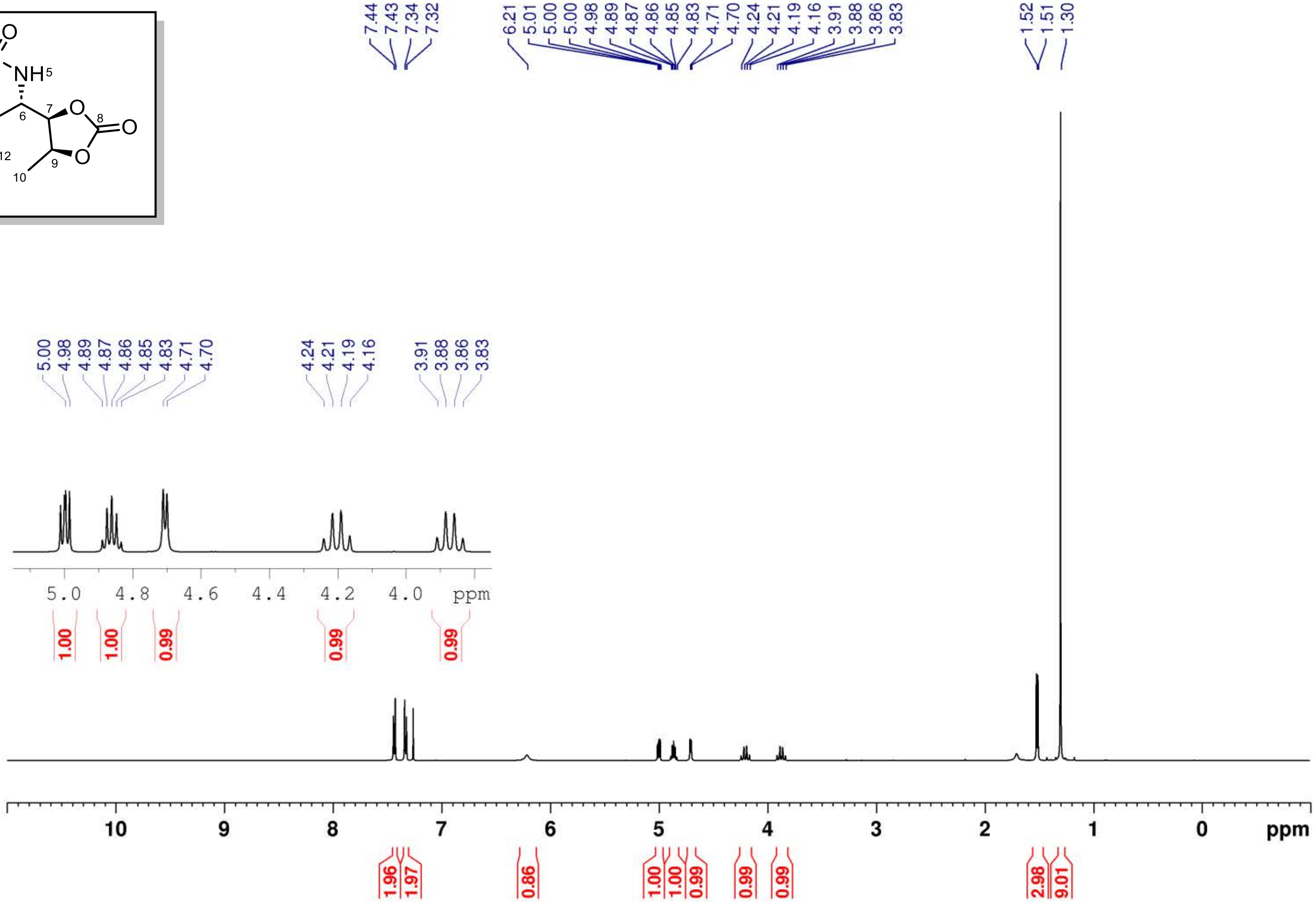

^{13}C NMR (126 MHz, CDCl$_3$) for 2,2,3,3,4,4,4-heptafluorobutyl ((*S*)-(4-(tert-butyl)phenyl)((4*R*,5*S*)-5-methyl-2-oxo-1,3-dioxolan-4-yl)methyl)sulfamate (**4t**)

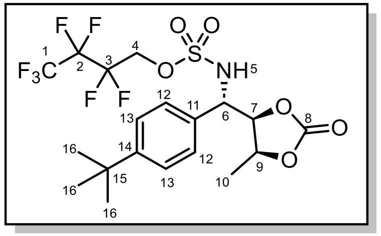

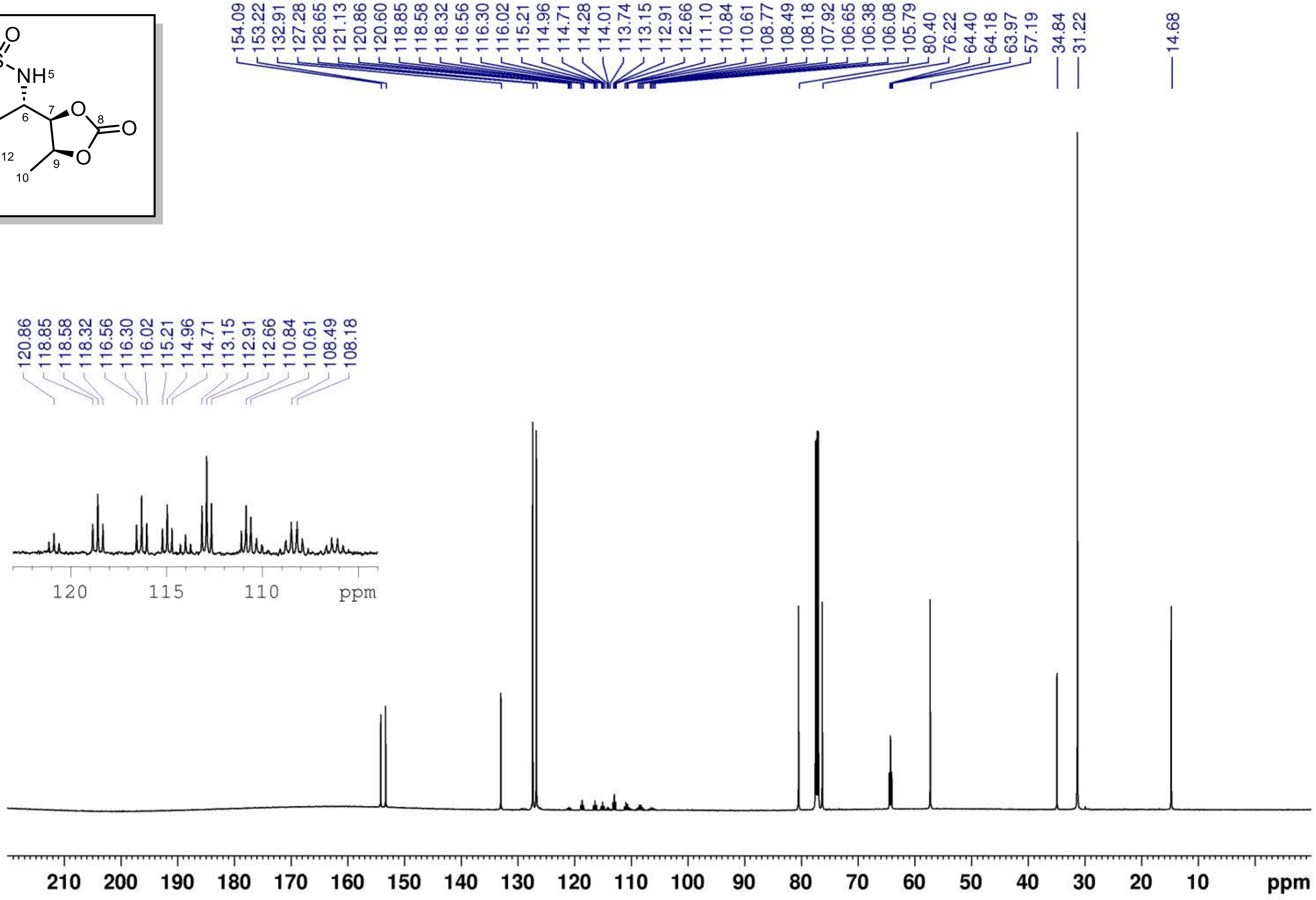

^{19}F NMR (376 MHz, CDCl$_3$) for 2,2,3,3,4,4,4-heptafluorobutyl ((*S*)-(4-(tert-butyl)phenyl)((4*R*,5*S*)-5-methyl-2-oxo-1,3-dioxolan-4-yl)methyl)sulfamate (**4t**)

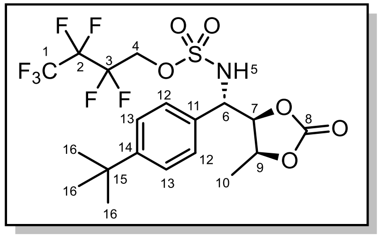

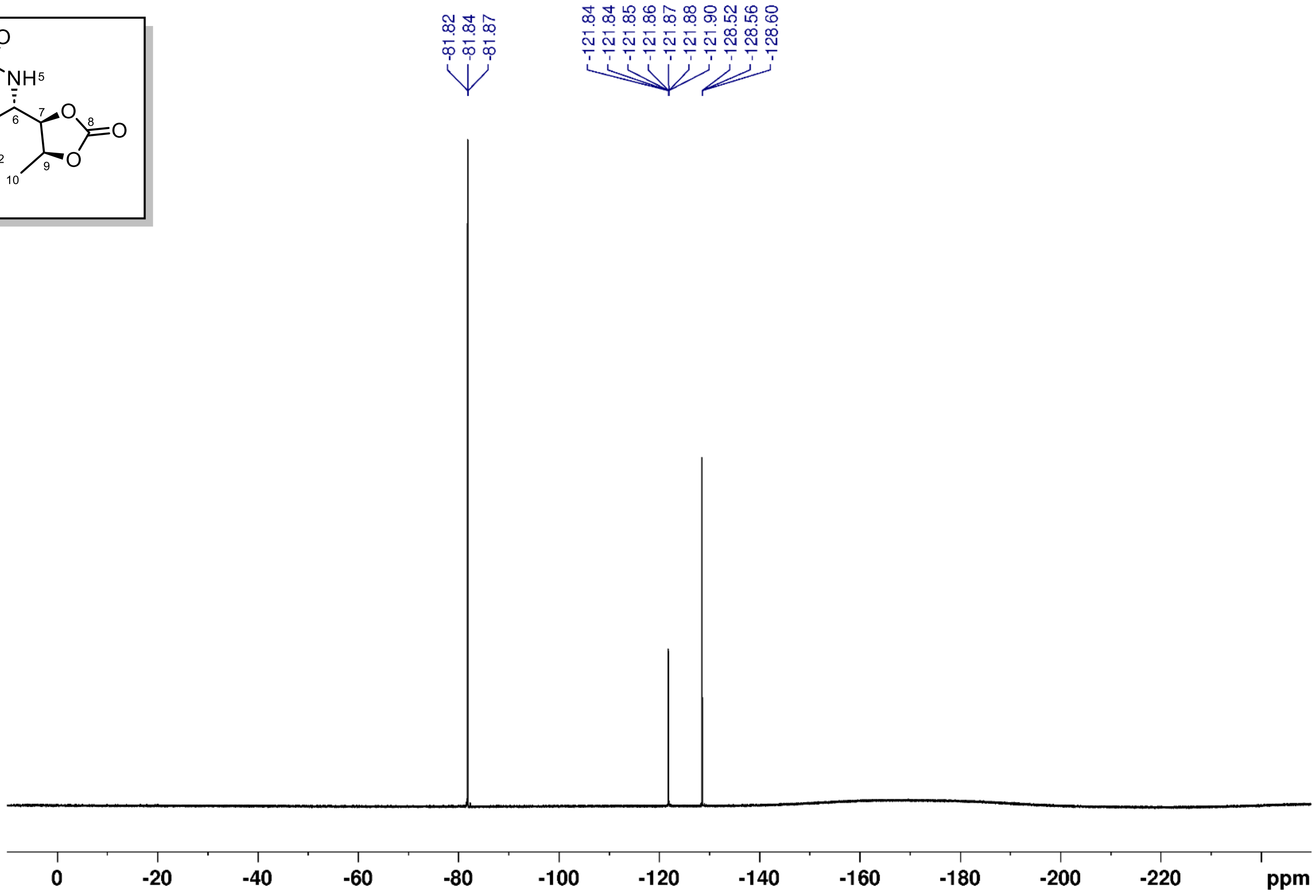

^{1}H-^{1}H NOESY (500 MHz, CDCl$_3$) for 2,2,3,3,4,4,4-heptafluorobutyl ((*S*)-(4-(*tert*-butyl)phenyl)((4*R*,5*S*)-5-methyl-2-oxo-1,3-dioxolan-4-yl)methyl)sulfamate (**4t**)

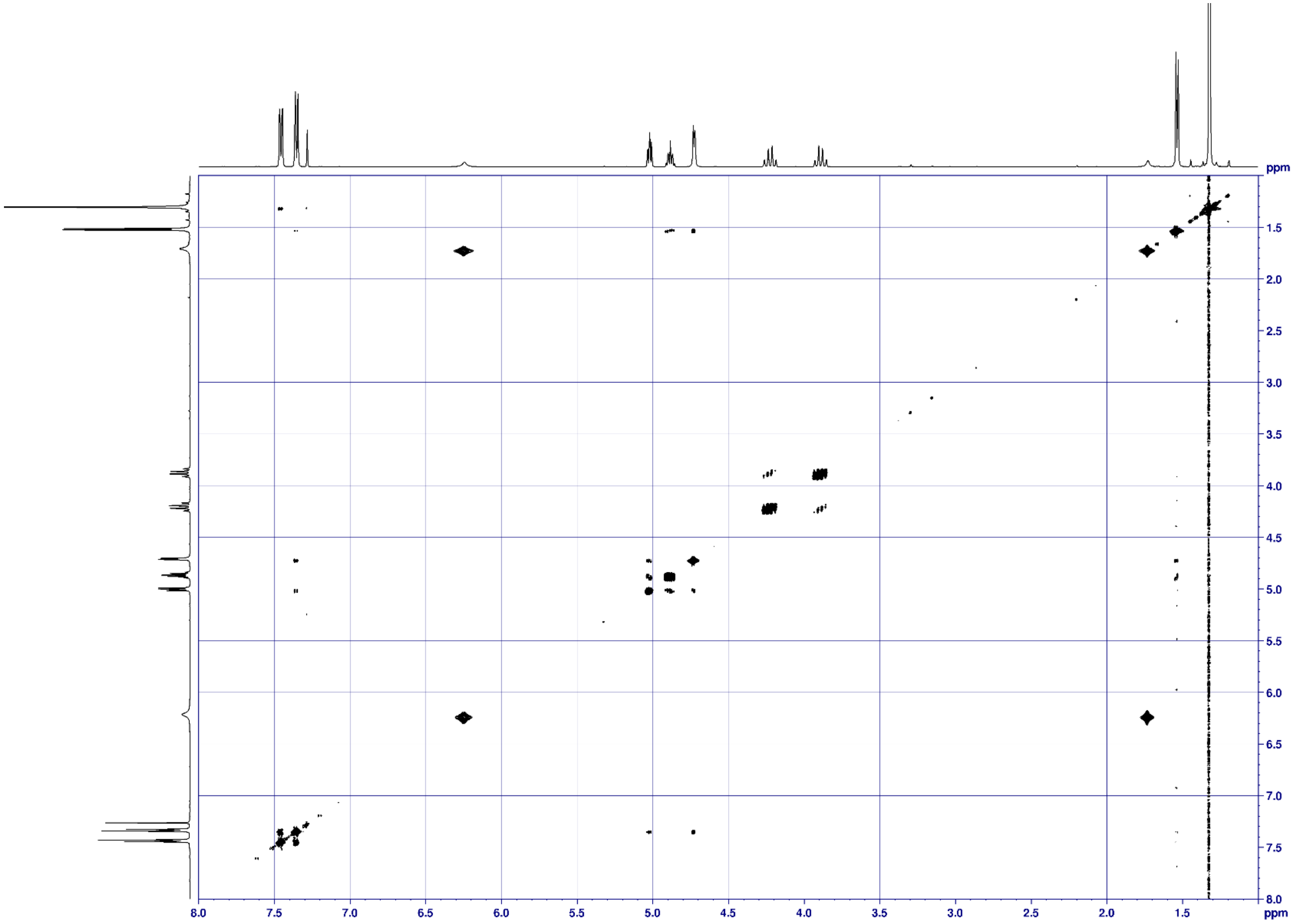

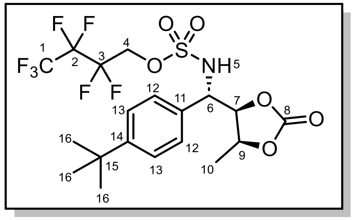

^{1}H-^{1}H NOESY (500 MHz, $CDCl_3$) for 2,2,3,3,4,4,4-heptafluorobutyl ((*S*)-(4-(*tert*-butyl)phenyl)((4*R*,5*S*)-5-methyl-2-oxo-1,3-dioxolan-4-yl)methyl)sulfamate (**4t**)

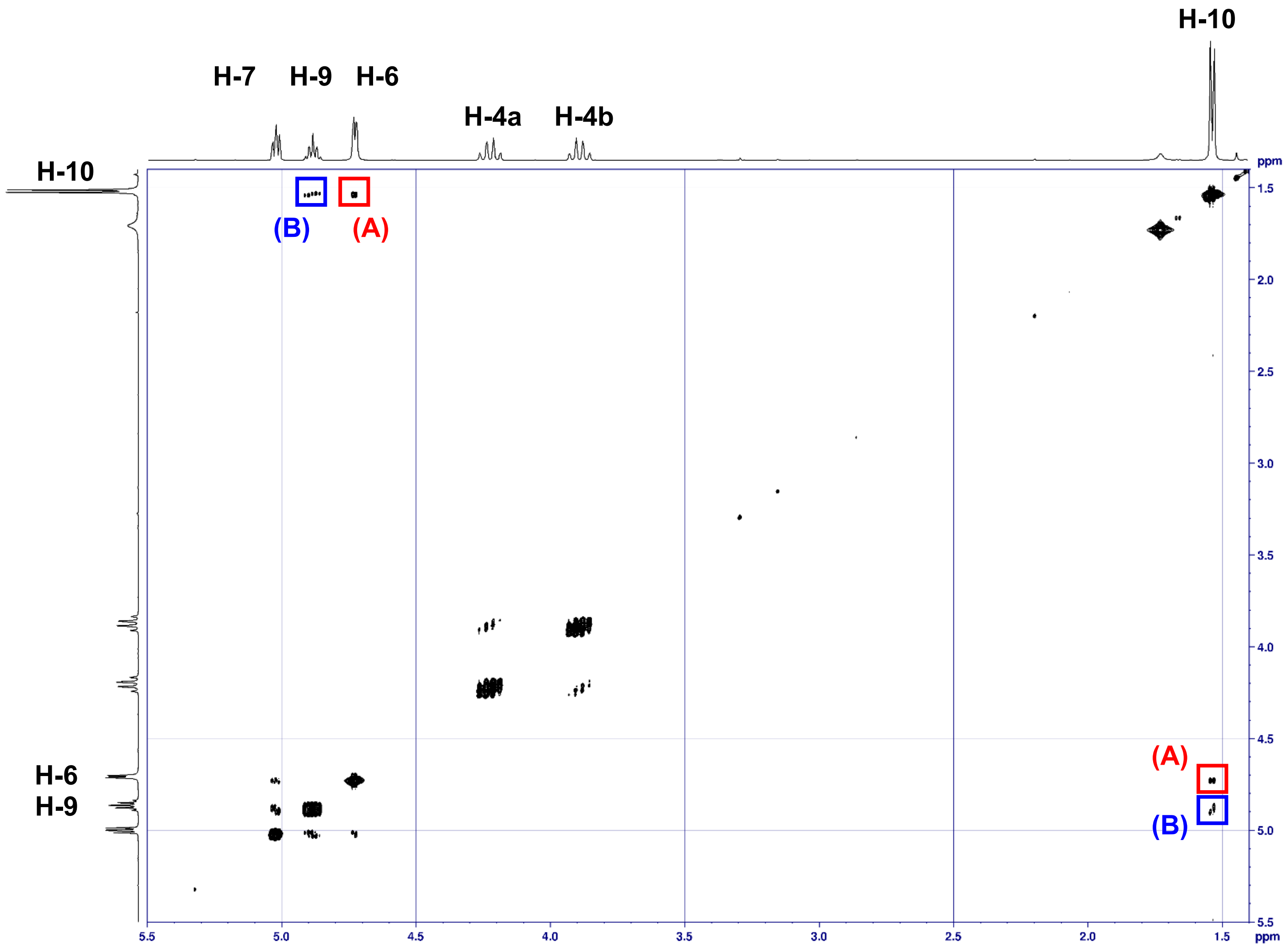

syn diastereomer
(assigned as this compound)

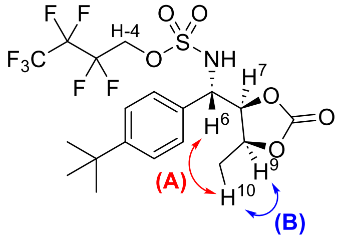

^{1}H NMR (400 MHz, CDCl$_3$) for (*R*,*Z*)-1-(4-(tert-butyl)phenyl)-4-methylpent-1-en-3-yl methyl(phenyl)carbamate ((***R***)-**1u**)

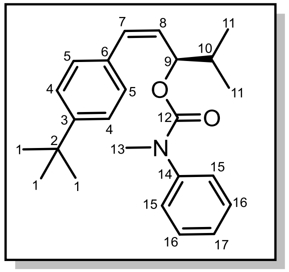

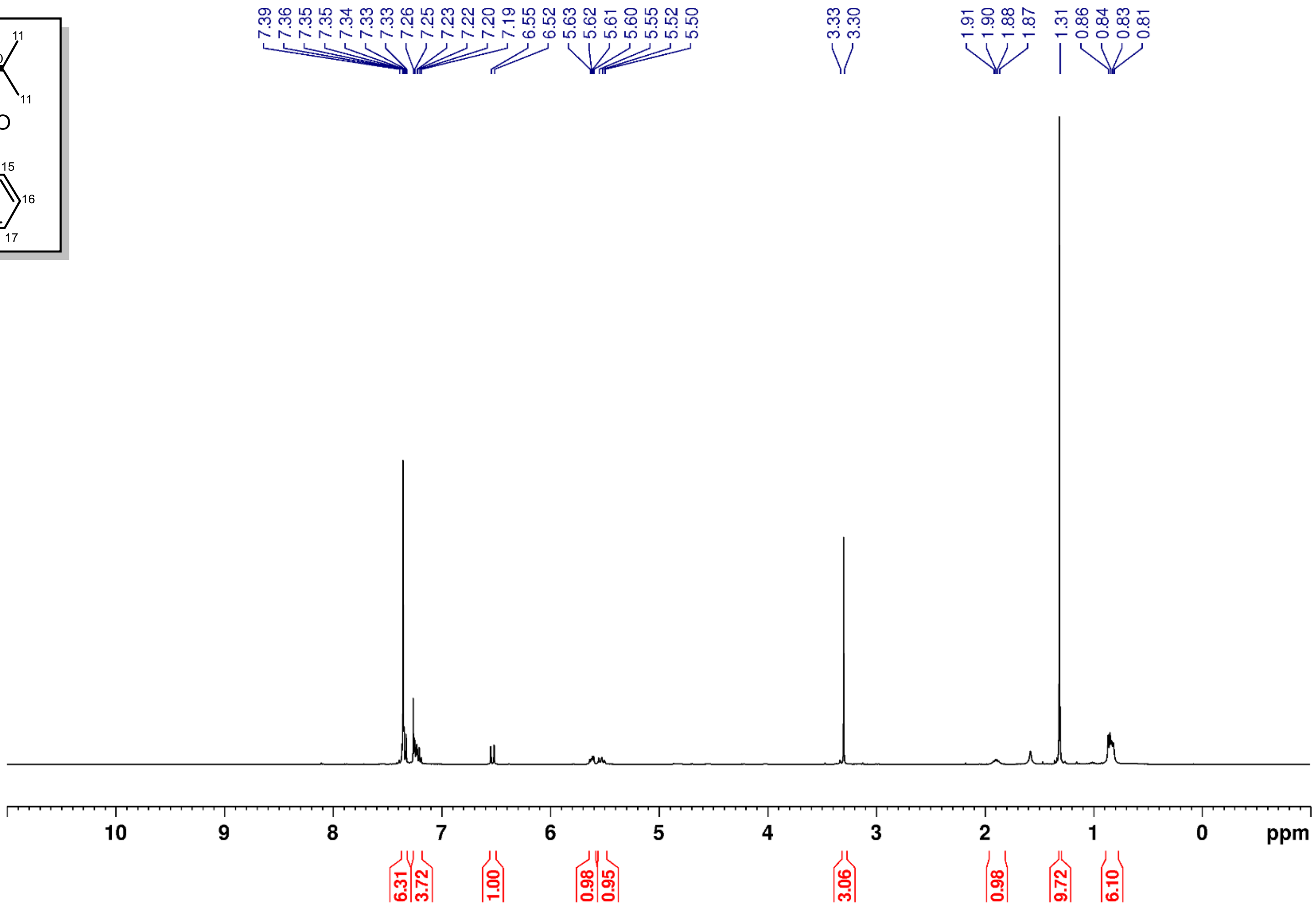

^{1}H NMR (700 MHz, CDCl$_3$) for 2,2,3,3,4,4,4-heptafluorobutyl ((*S*)-(4-(tert-butyl)phenyl)((4*R*,5*S*)-5-isopropyl-2-oxo-1,3-dioxolan-4-yl)methyl)sulfamate (**4u**)

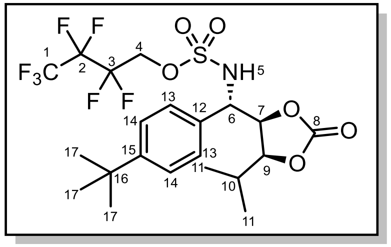

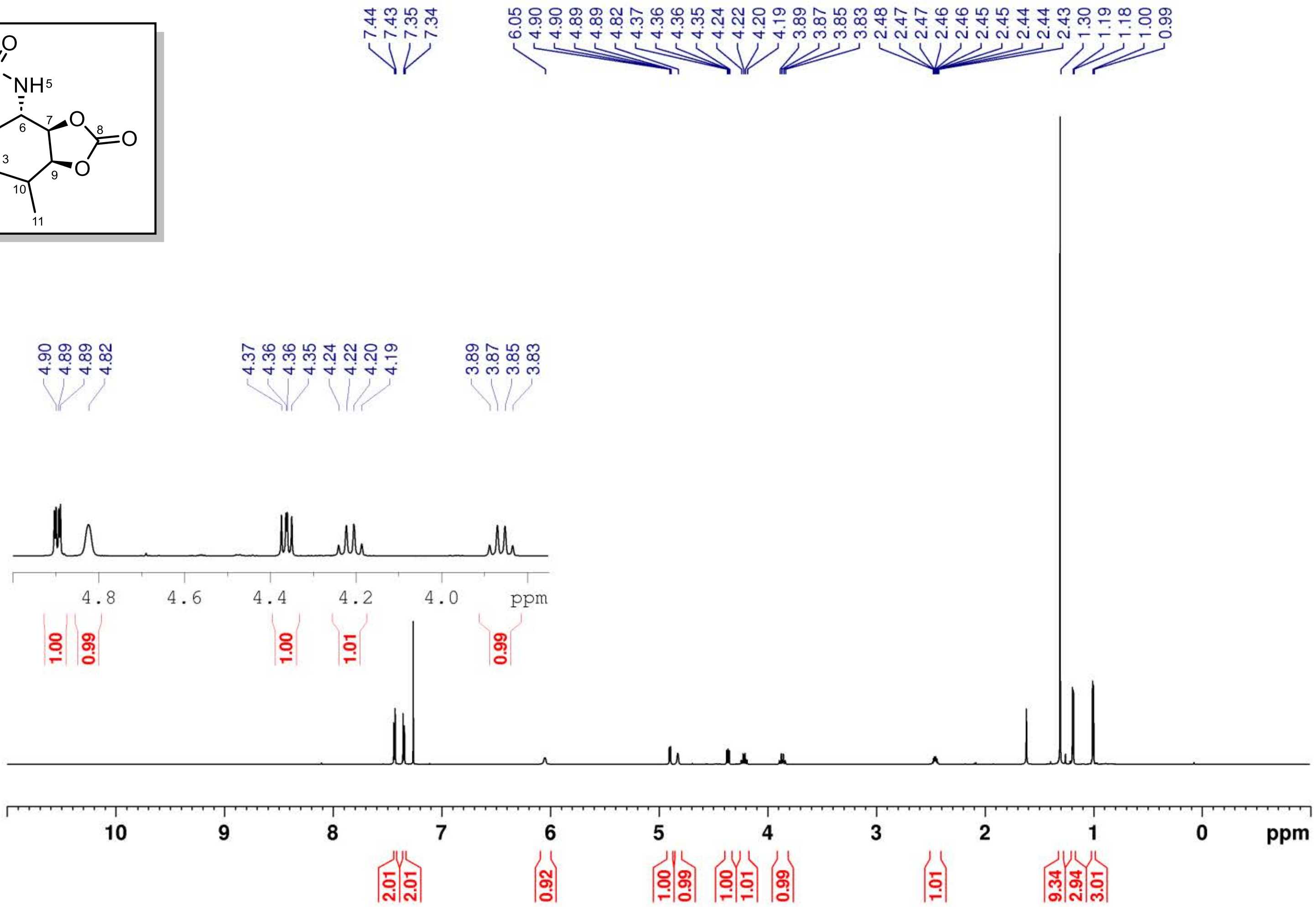

^{13}C NMR (176 MHz, CDCl$_3$) for 2,2,3,3,4,4,4-heptafluorobutyl ((*S*)-(4-(tert-butyl)phenyl)((4*R*,5*S*)-5-isopropyl-2-oxo-1,3-dioxolan-4-yl)methyl)sulfamate (**4u**)

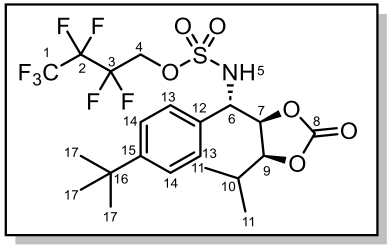

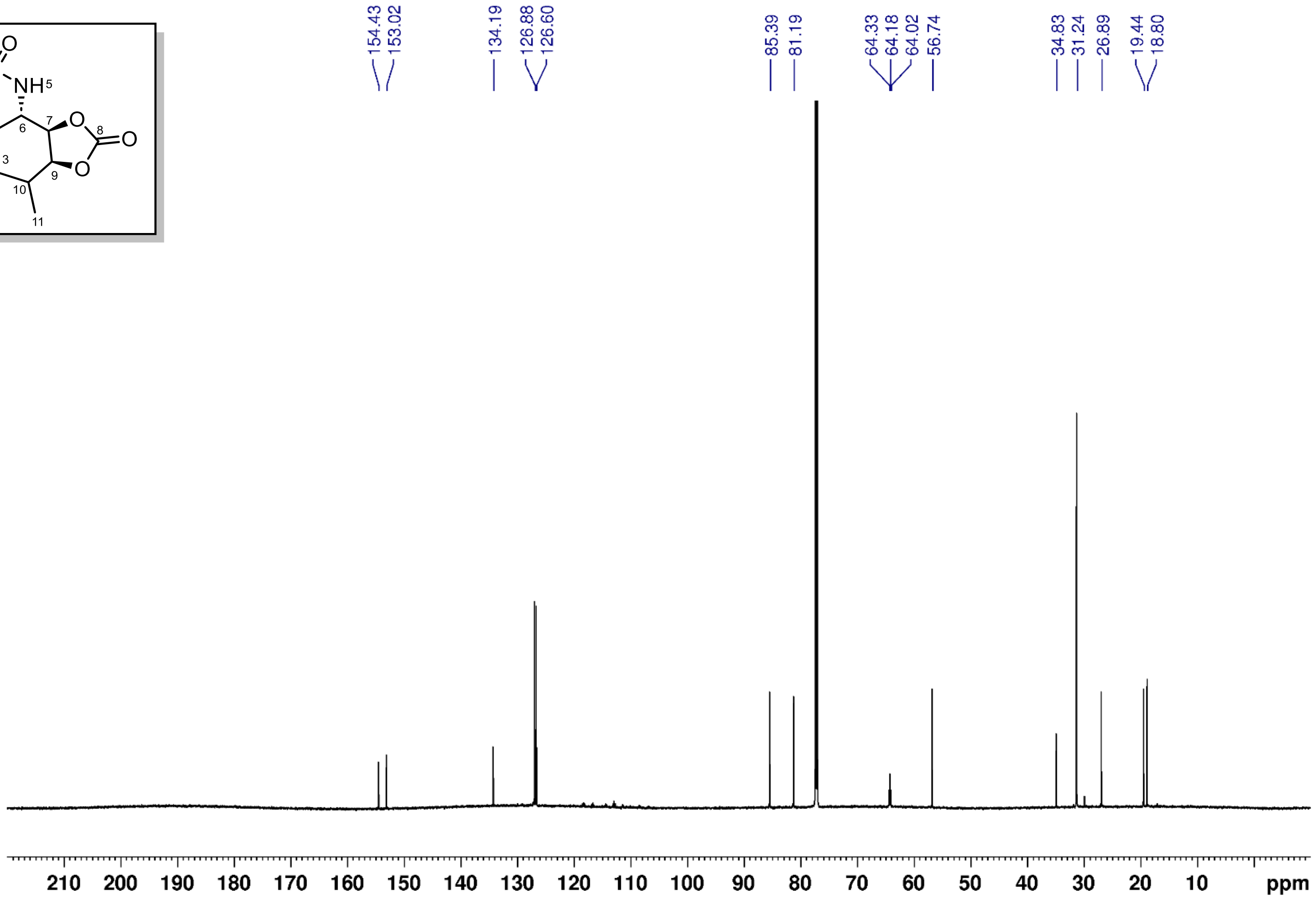

^{19}F NMR (376 MHz, CDCl$_3$) for 2,2,3,3,4,4,4-heptafluorobutyl ((*S*)-(4-(tert-butyl)phenyl)((4*R*,5*S*)-5-isopropyl-2-oxo-1,3-dioxolan-4-yl)methyl)sulfamate (**4u**)

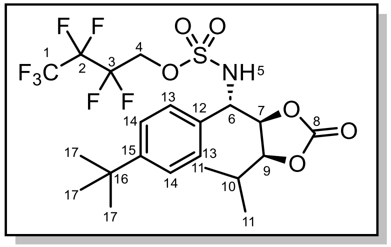

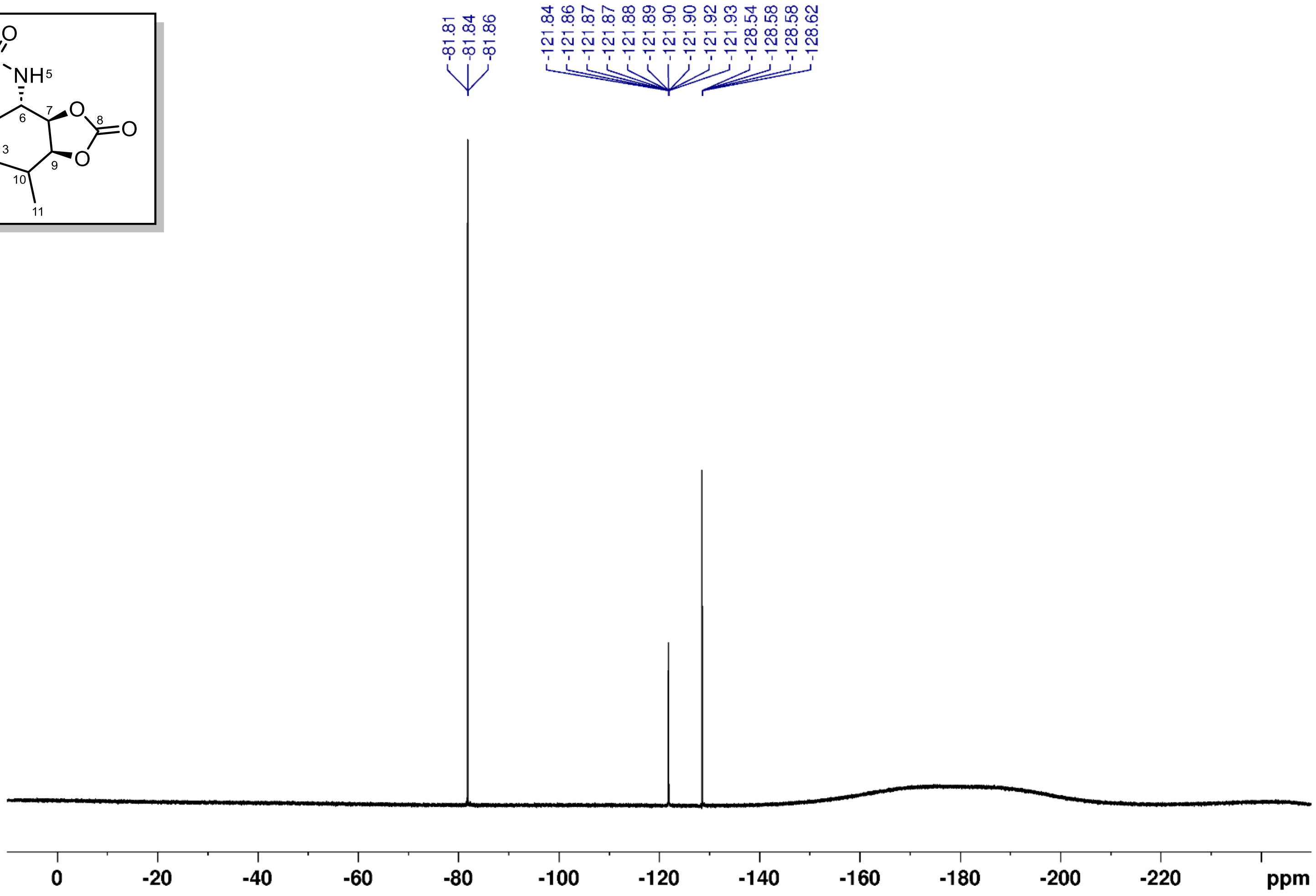

^{1}H NMR (700 MHz, CDCl$_3$) for 2,2,3,3,4,4,4-heptafluorobutyl ((*S*)-((*S*)-5-oxotetrahydrofuran-2-yl)(phenyl)methyl)sulfamate (**9**)

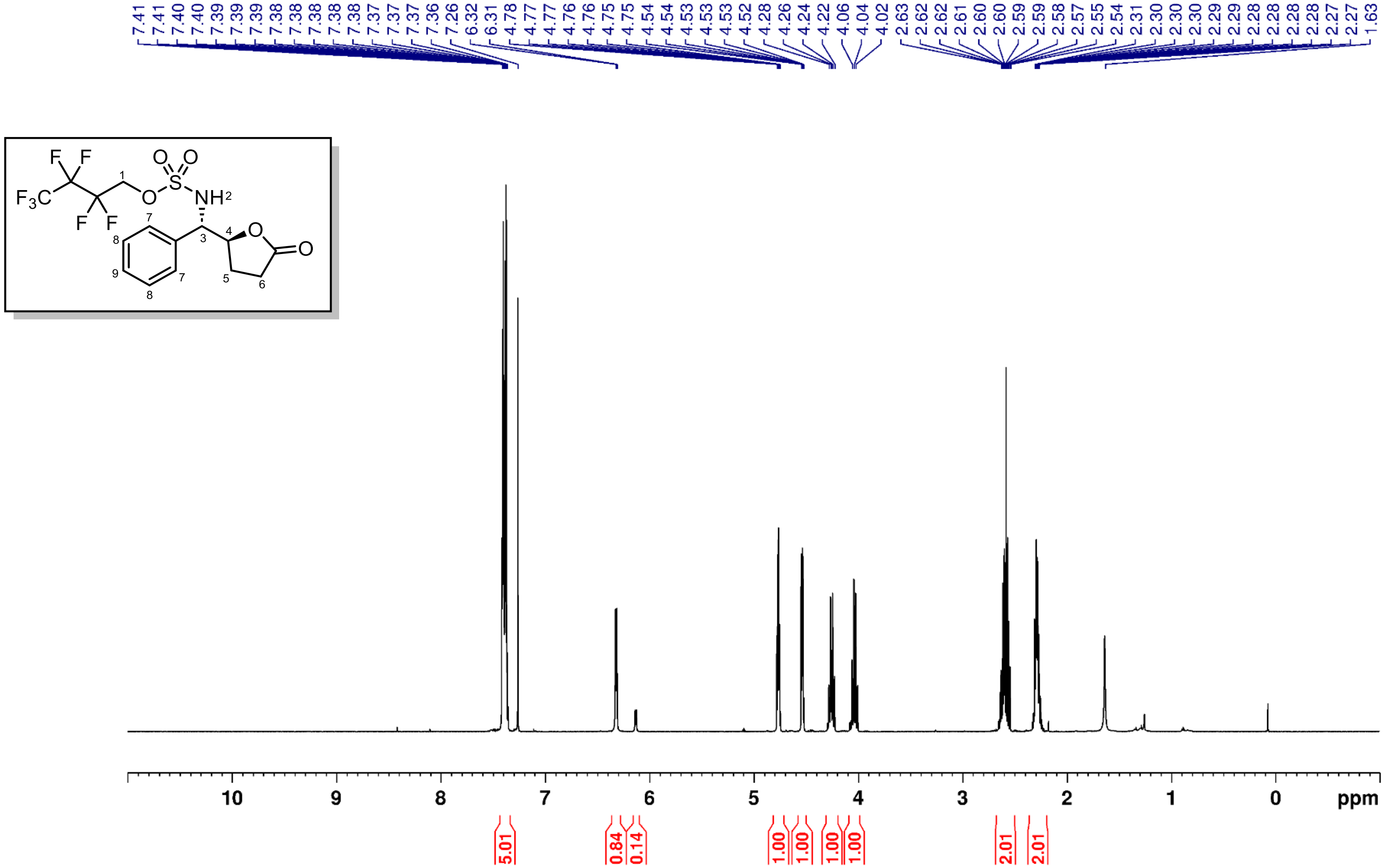

^{13}C NMR (176 MHz, CDCl$_3$) for 2,2,3,3,4,4,4-heptafluorobutyl ((*S*)-((*S*)-5-oxotetrahydrofuran-2-yl)(phenyl)methyl)sulfamate (**9**)

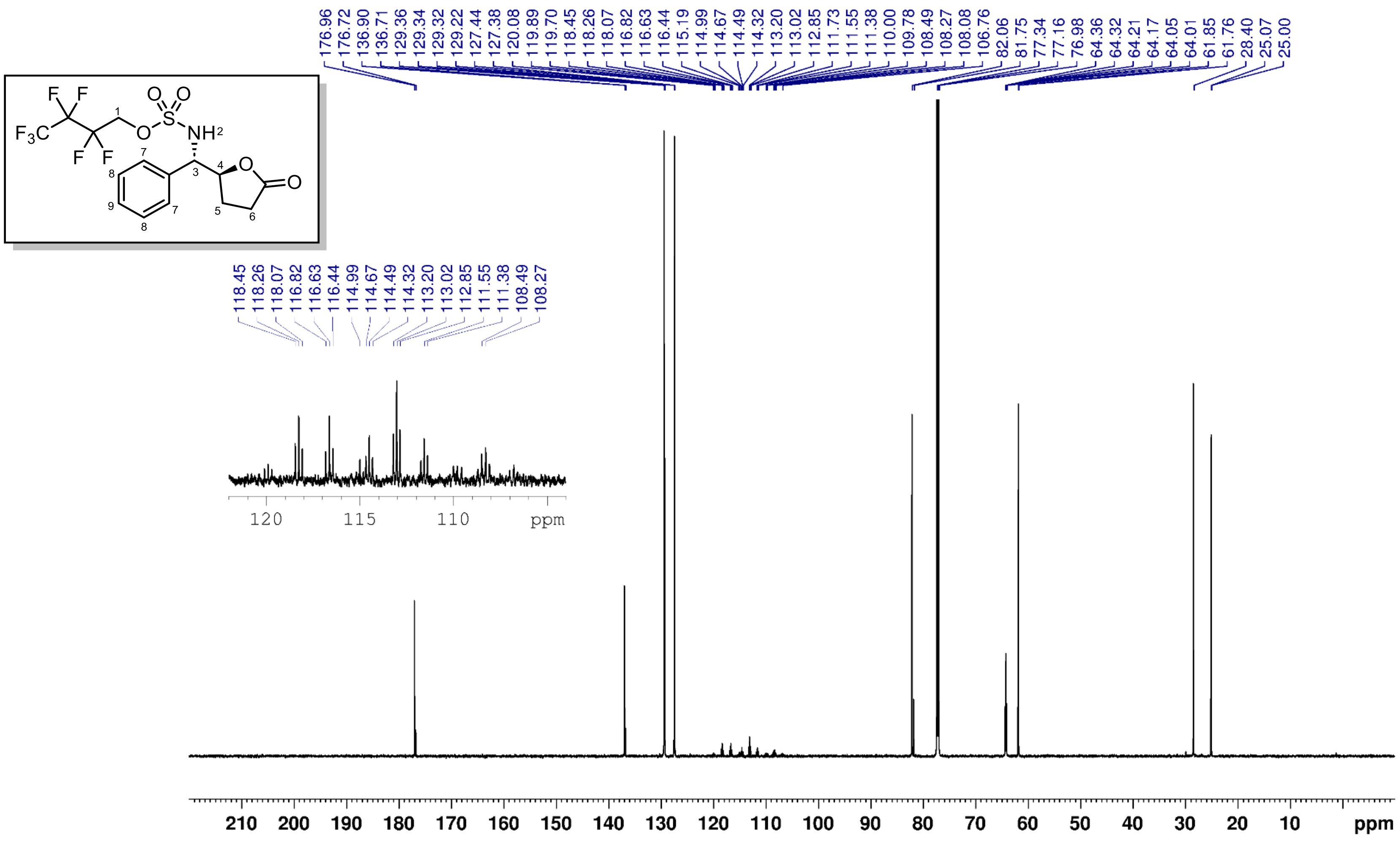

^{19}F NMR (376 MHz, $CDCl_3$) for 2,2,3,3,4,4,4-heptafluorobutyl ((*S*)-((*S*)-5-oxotetrahydrofuran-2-yl)(phenyl)methyl)sulfamate (**9**)

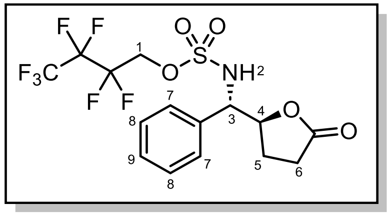

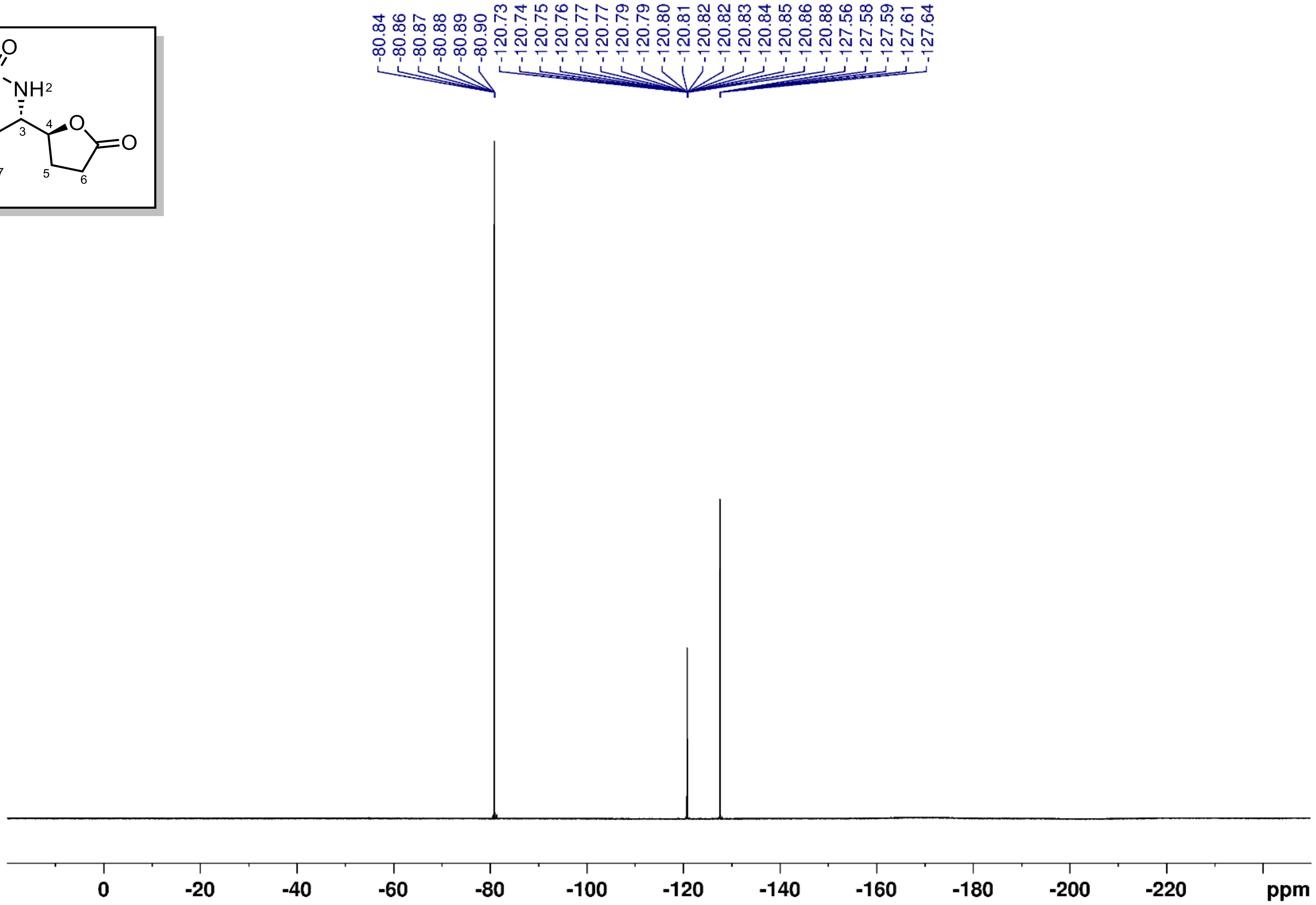

^{1}H NMR (700 MHz, $CDCl_3$) for 2,2,3,3,4,4,4-heptafluorobutyl ((2-oxo-4-phenyl-1,3-dioxolan-4-yl)methyl)sulfamate (**16a**)

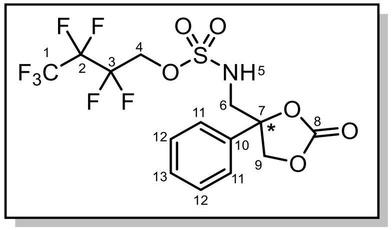

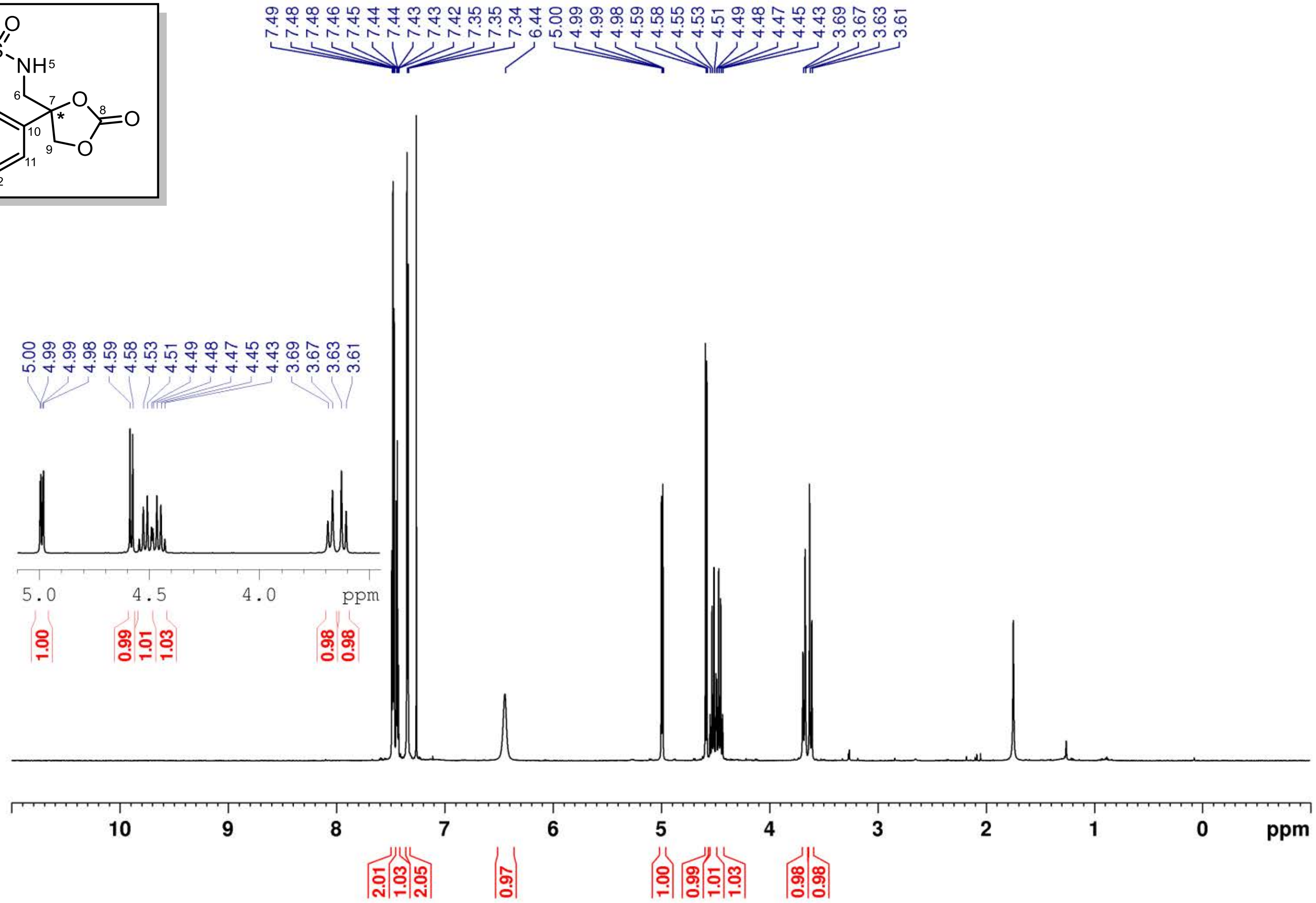

^{13}C NMR (176 MHz, CDCl$_3$) for 2,2,3,3,4,4,4-heptafluorobutyl ((2-oxo-4-phenyl-1,3-dioxolan-4-yl)methyl)sulfamate (**16a**)

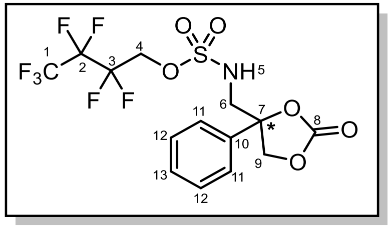

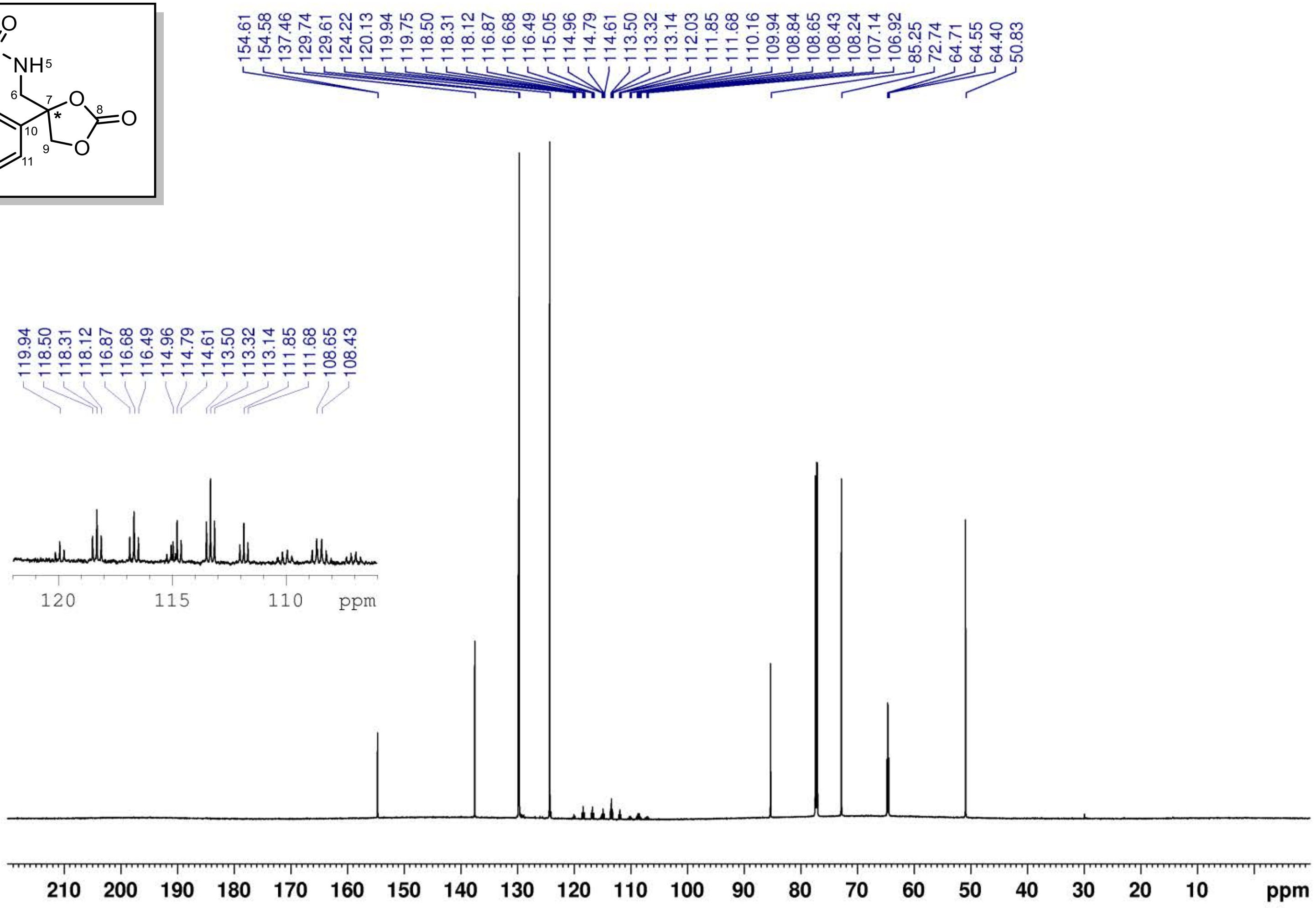

¹⁹F NMR (376 MHz, CDCl₃) for 2,2,3,3,4,4,4-heptafluorobutyl ((2-oxo-4-phenyl-1,3-dioxolan-4-yl)methyl)sulfamate (**16a**)

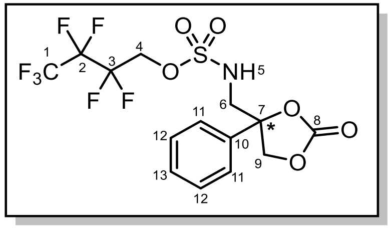

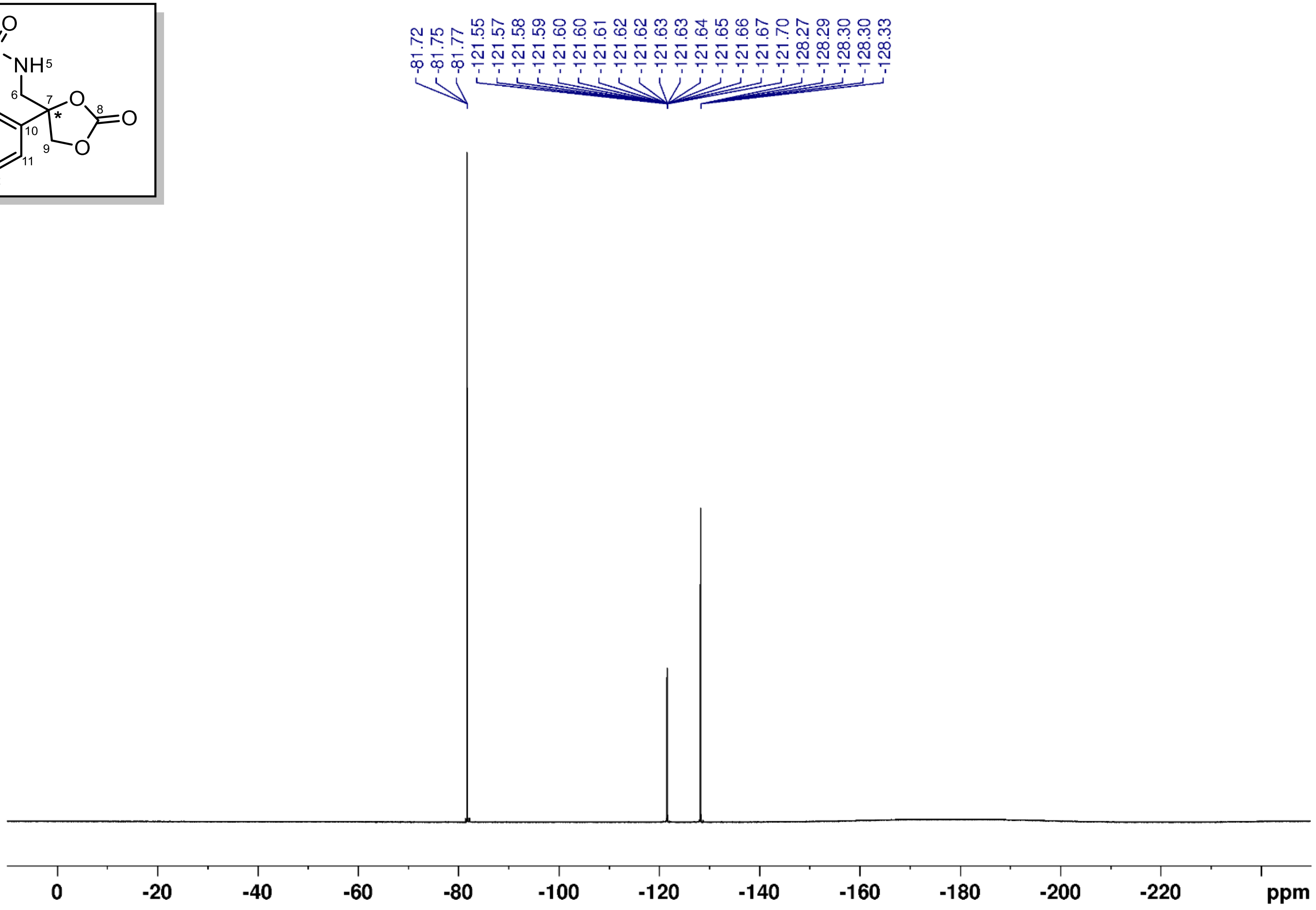

^{1}H NMR (500 MHz, $CDCl_3$) for 2,2,3,3,4,4,4-heptafluorobutyl (2-(2-oxo-1,3-dioxolan-4-yl)propan-2-yl)sulfamate (**16b**)

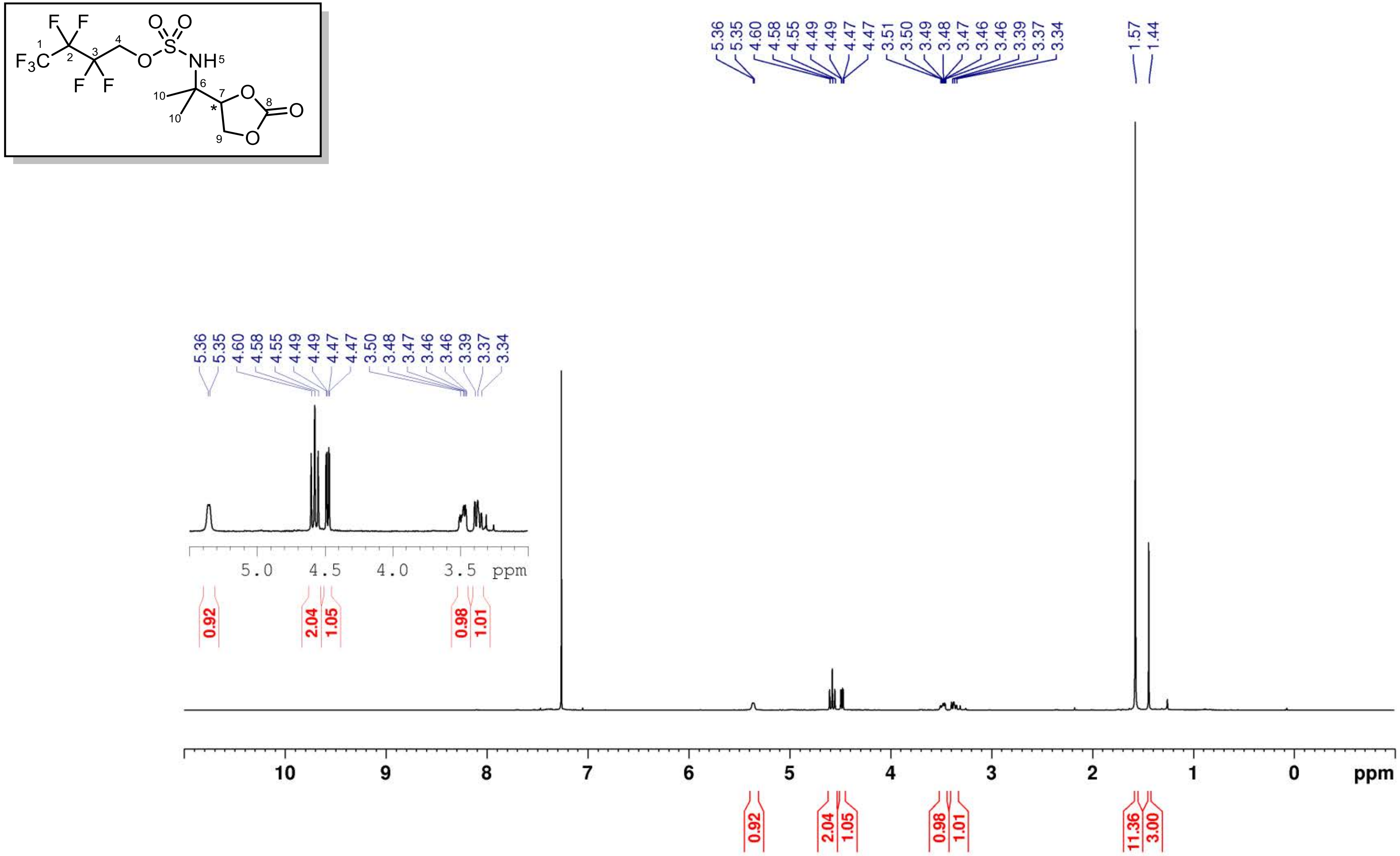

^{13}C NMR (126 MHz, CDCl$_3$) for 2,2,3,3,4,4,4-heptafluorobutyl (2-(2-oxo-1,3-dioxolan-4-yl)propan-2-yl)sulfamate (**16b**)

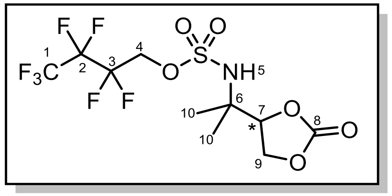

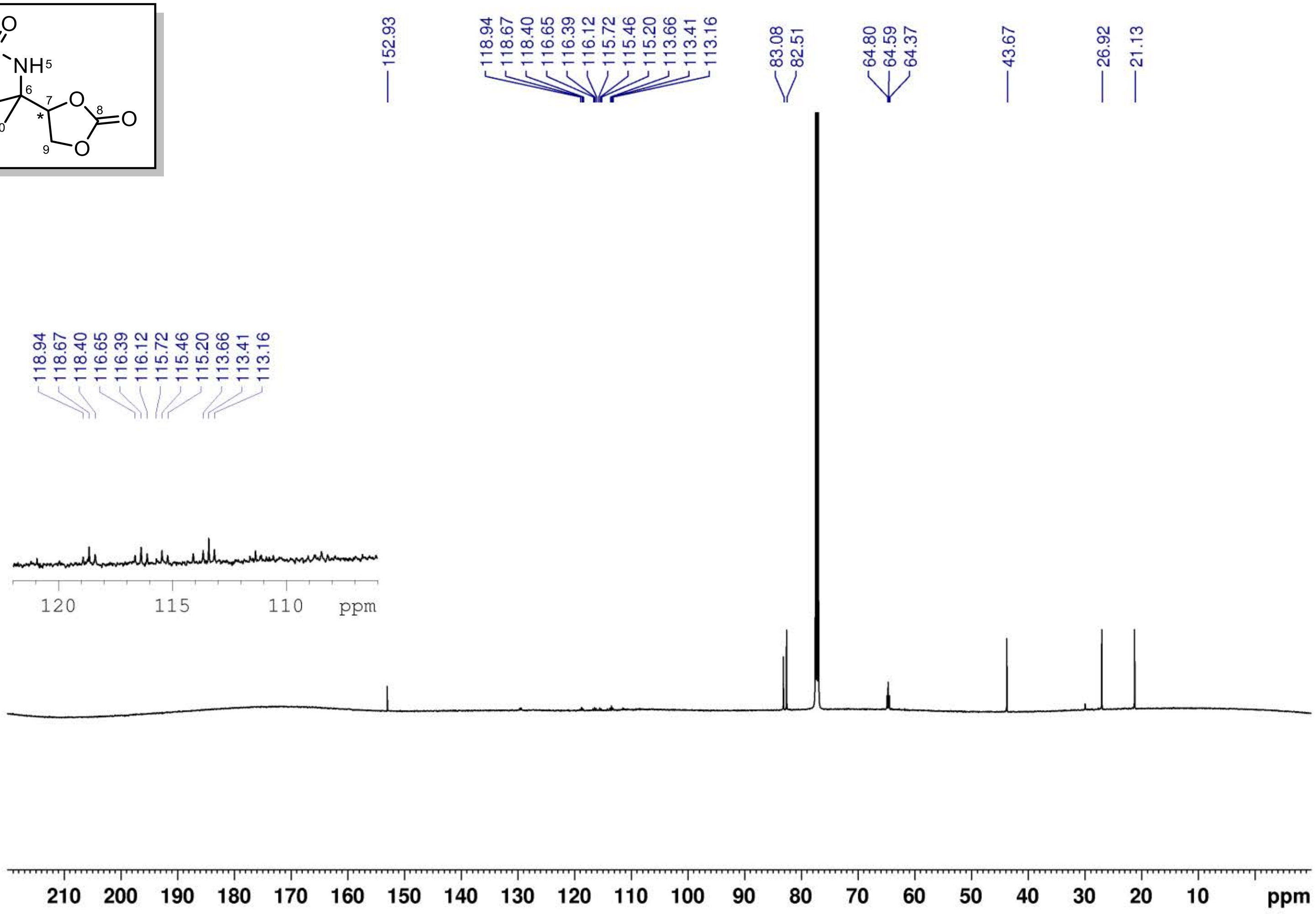

^{19}F NMR (376 MHz, $CDCl_3$) for 2,2,3,3,4,4,4-heptafluorobutyl ((2-oxo-4-phenyl-1,3-dioxolan-4-yl)methyl)sulfamate (**16b**)

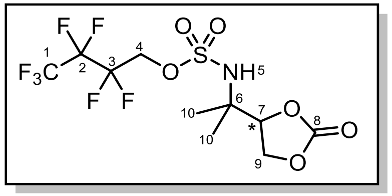

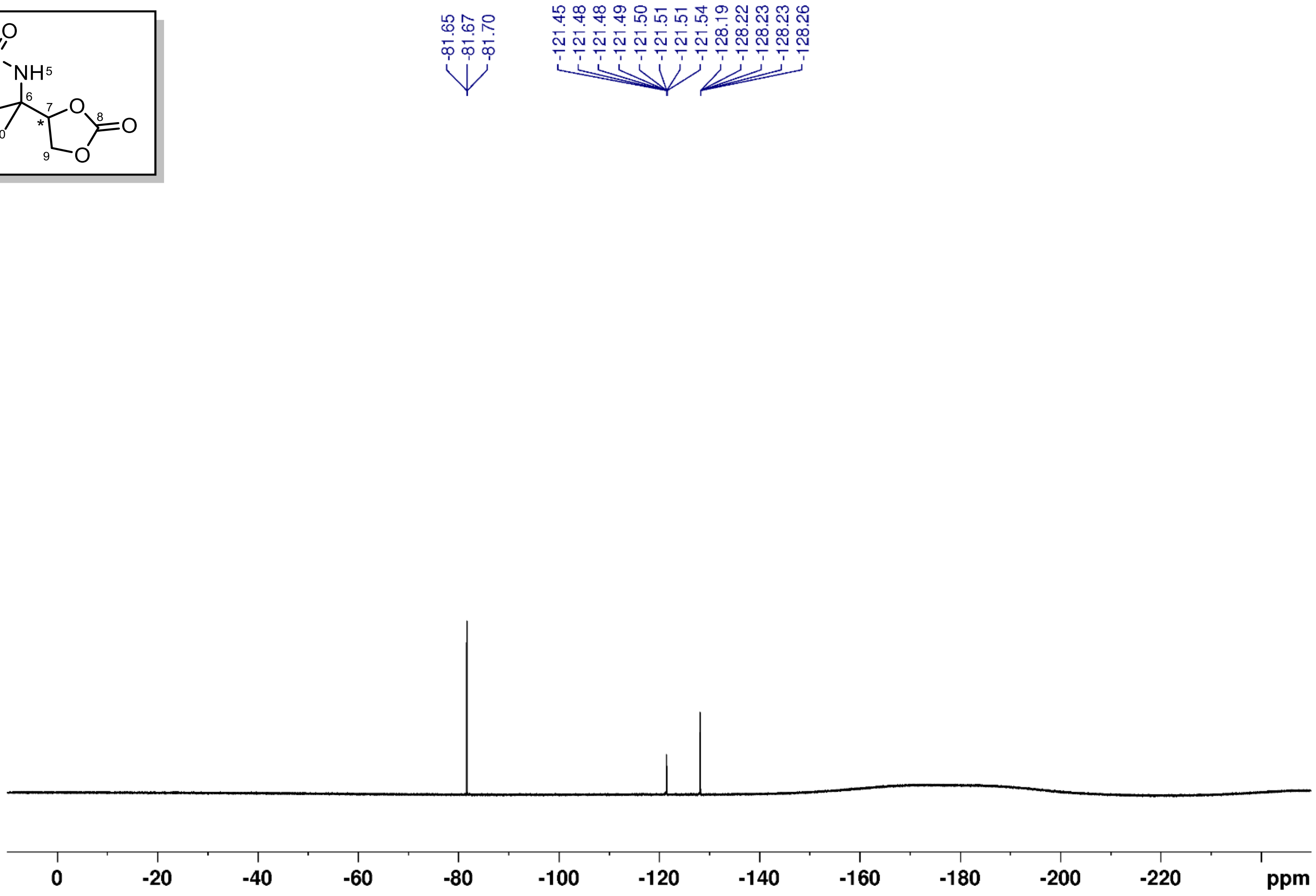

^{1}H NMR (500 MHz, $CDCl_3$) for 2,2,3,3,4,4,4-heptafluorobutyl (1-(2-oxo-1,3-dioxolan-4-yl)butyl)sulfamate (**16c**)

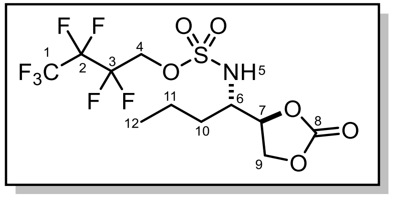

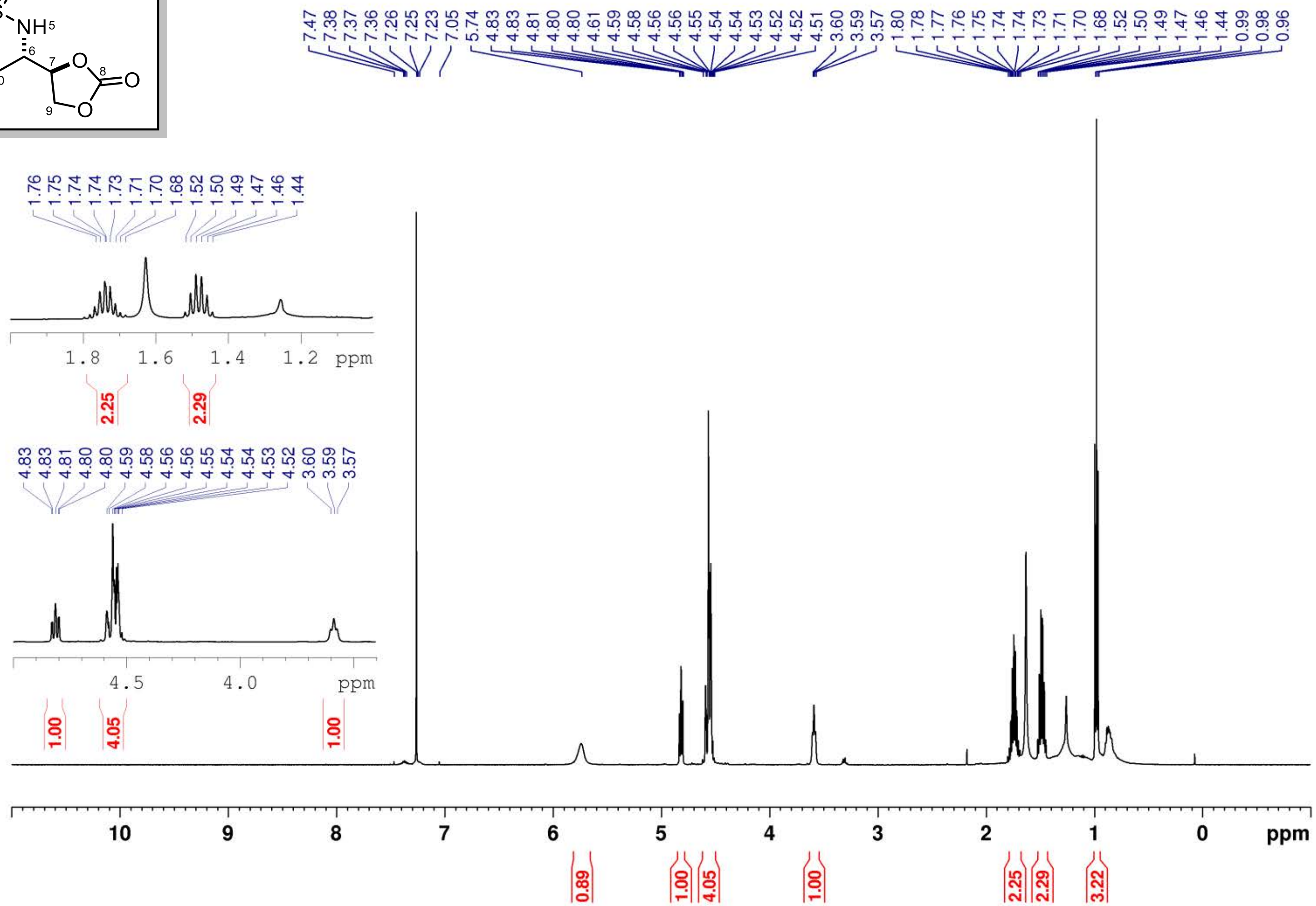

^{13}C NMR (126 MHz, CDCl$_3$) for 2,2,3,3,4,4,4-heptafluorobutyl (1-(2-oxo-1,3-dioxolan-4-yl)butyl)sulfamate (**16c**)

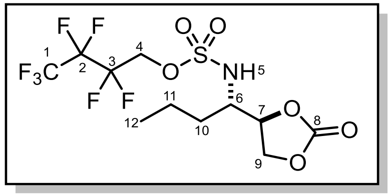

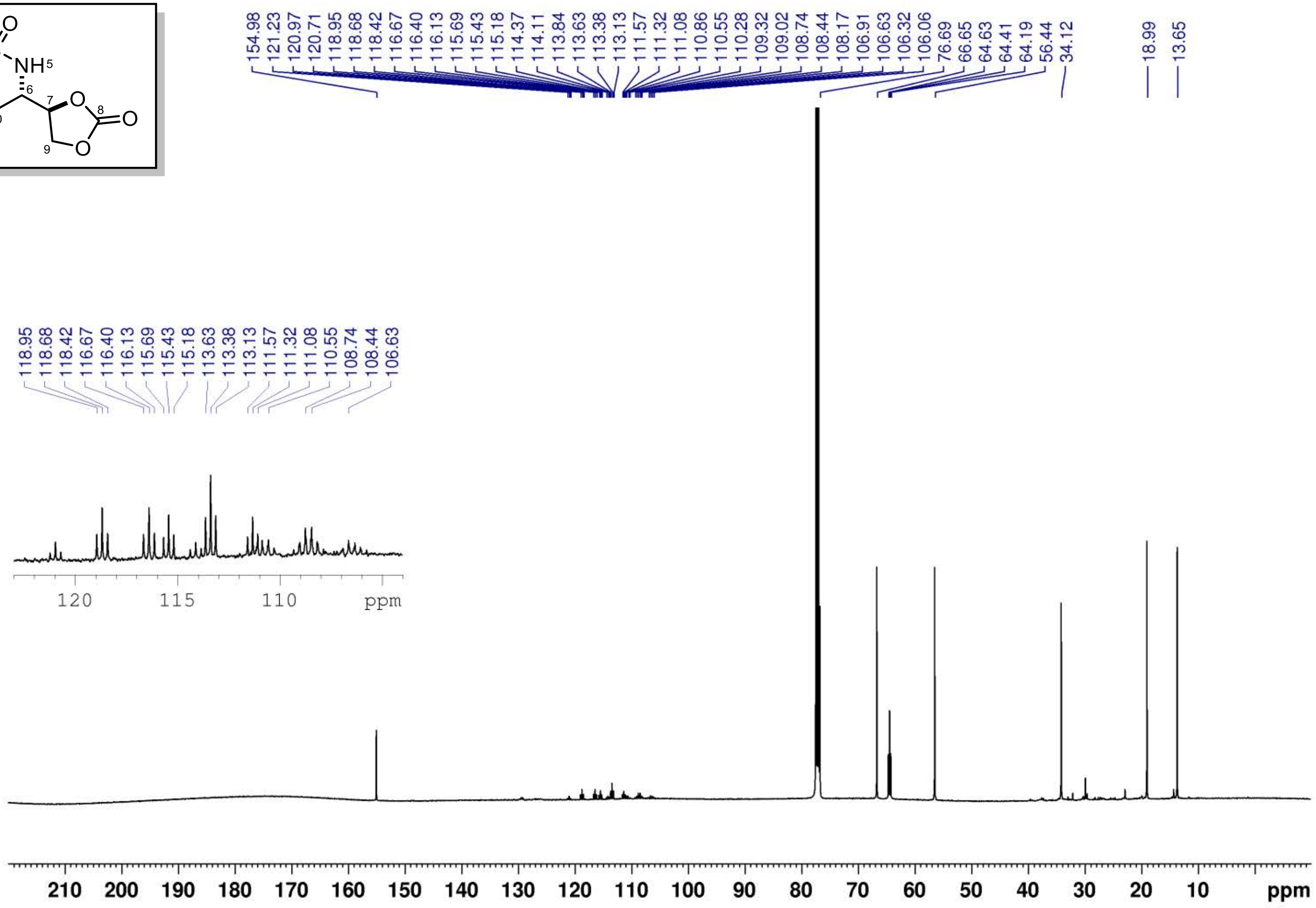

^{19}F NMR (376 MHz, CDCl$_3$) for 2,2,3,3,4,4,4-heptafluorobutyl (1-(2-oxo-1,3-dioxolan-4-yl)butyl)sulfamate (**16c**)

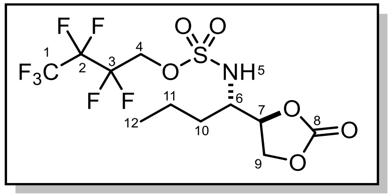

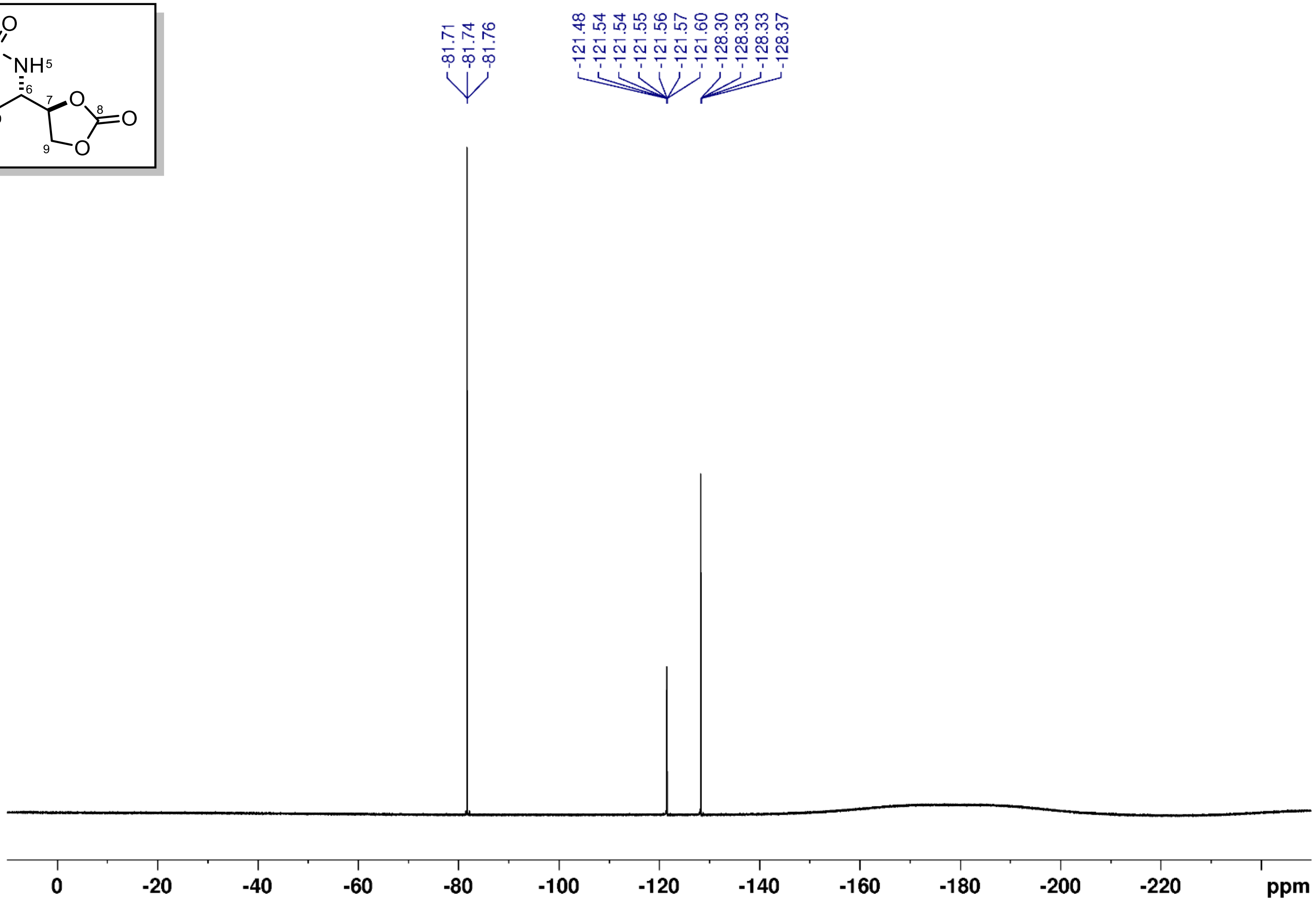

^{1}H NMR (500 MHz, $CDCl_3$) for 2,2,3,3,4,4,4-heptafluorobutyl (4-bromobenzyl)(1-(2-oxo-1,3-dioxolan-4-yl)butyl)sulfamate

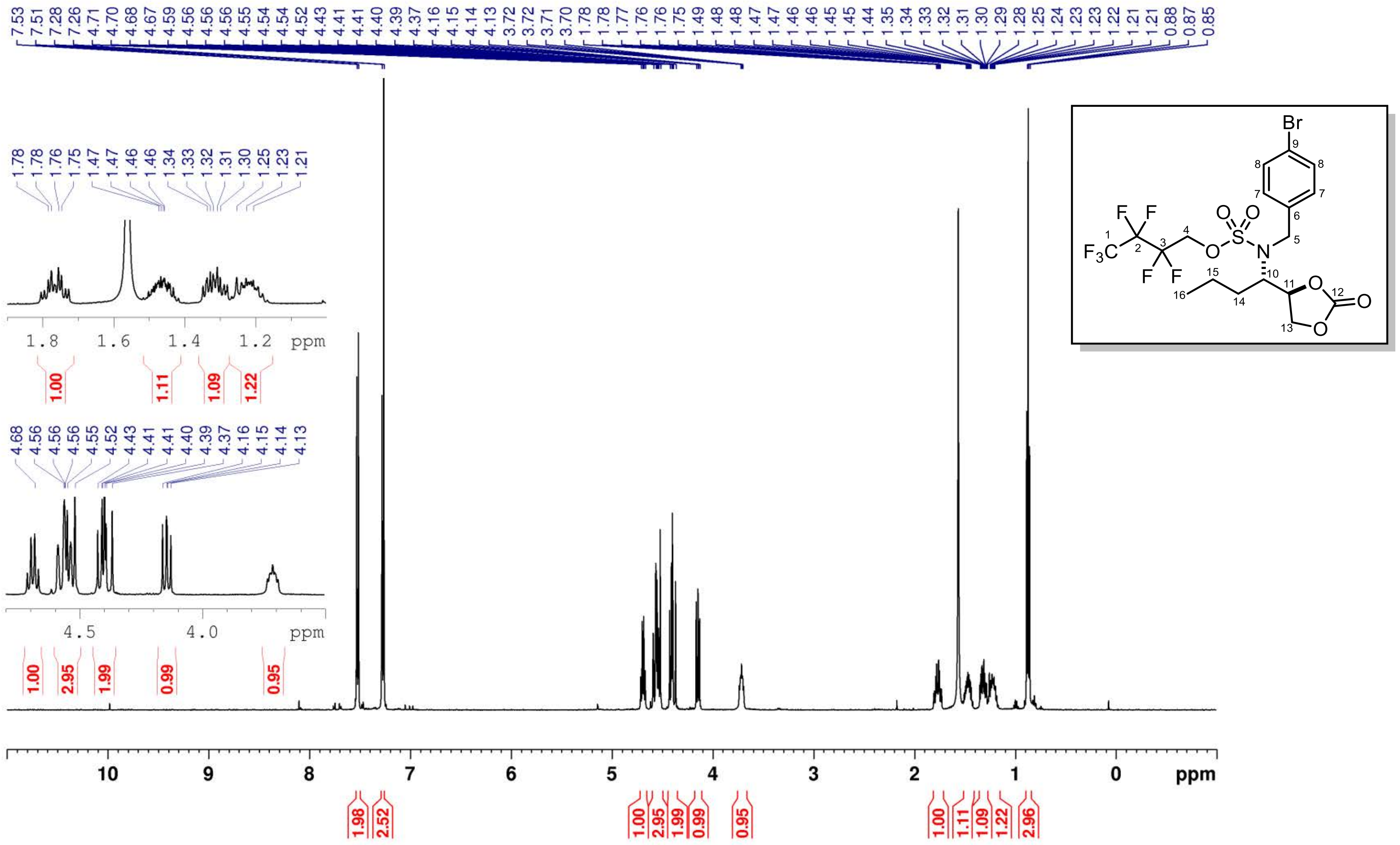

^{13}C NMR (126 MHz, CDCl$_3$) for 2,2,3,3,4,4,4-heptafluorobutyl (4-bromobenzyl)(1-(2-oxo-1,3-dioxolan-4-yl)butyl)sulfamate

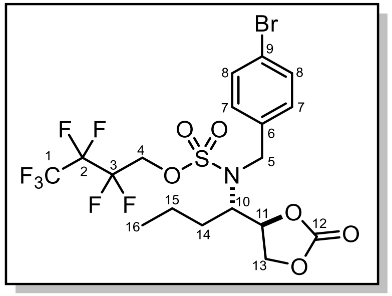

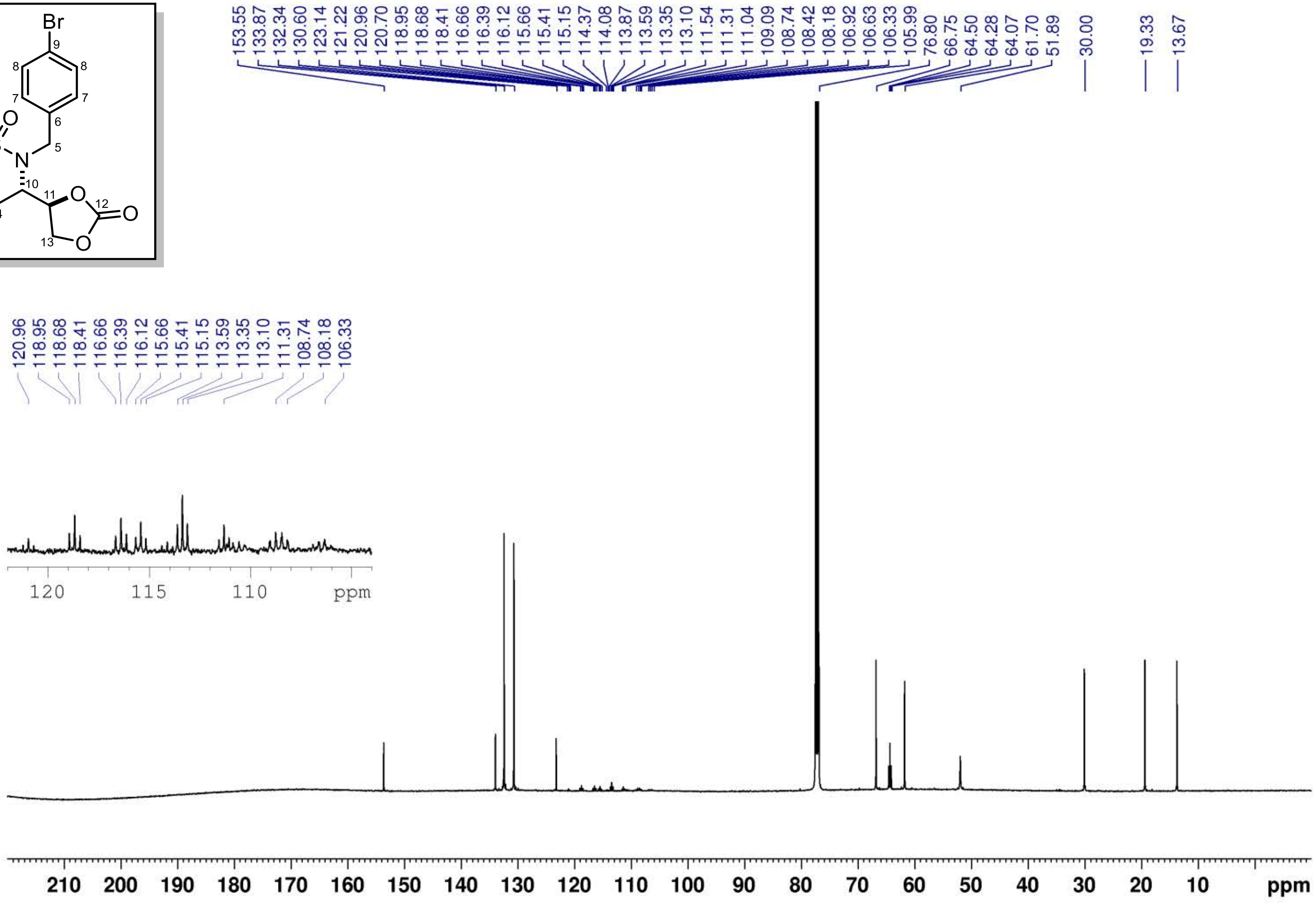

^{19}F NMR (376 MHz, CDCl₃) for 2,2,3,3,4,4,4-heptafluorobutyl (1-(2-oxo-1,3-dioxolan-4-yl)butyl)sulfamate

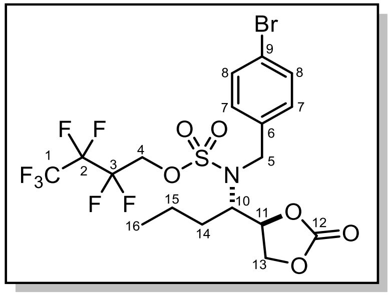

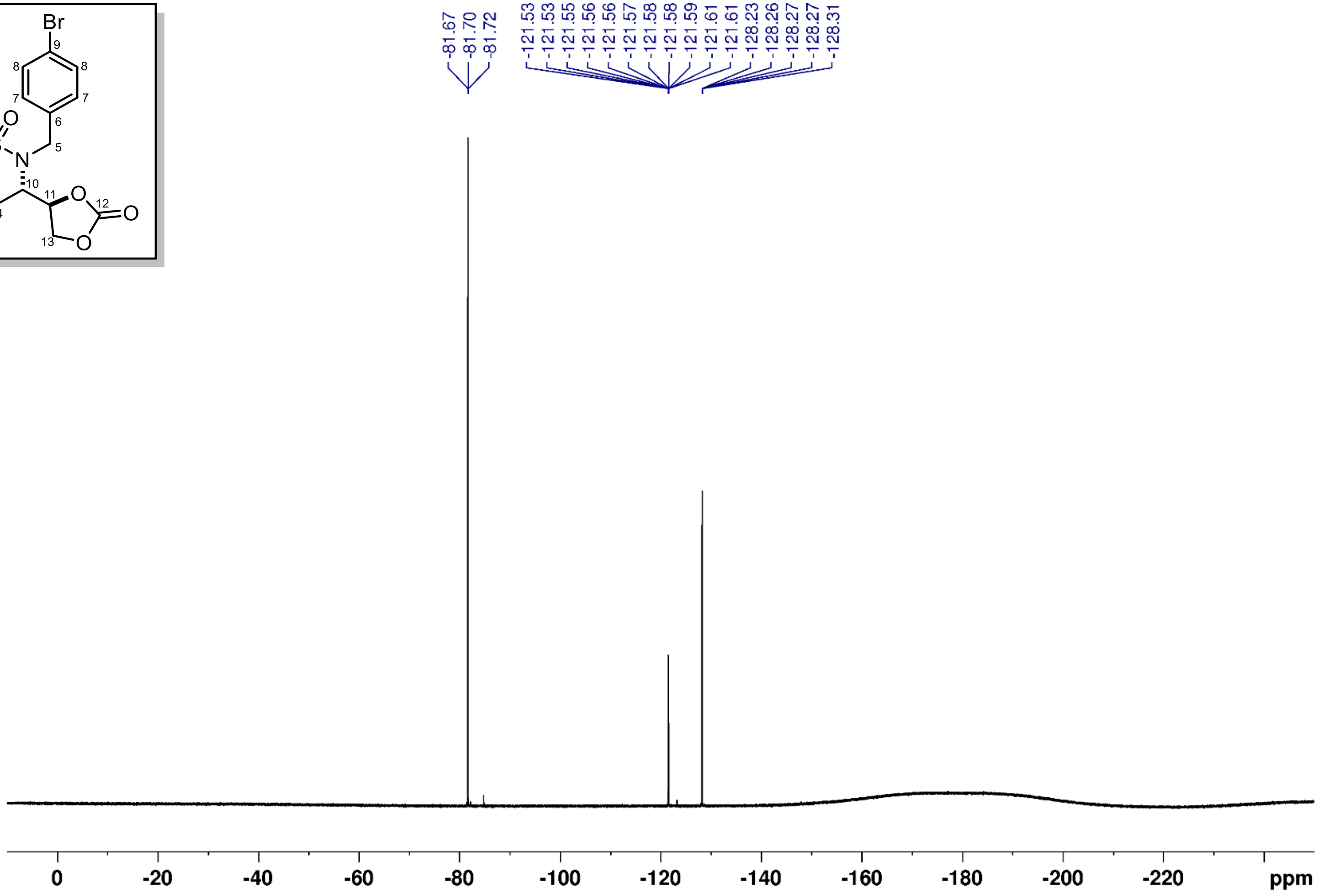

¹H NMR (700 MHz, CD$_3$CN) for 2,2,3,3,4,4,4-heptafluorobutyl ((2-oxo-1,3-dioxolan-4-yl)(phenyl)methyl)sulfamate (**S3**)

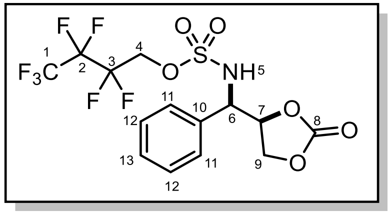

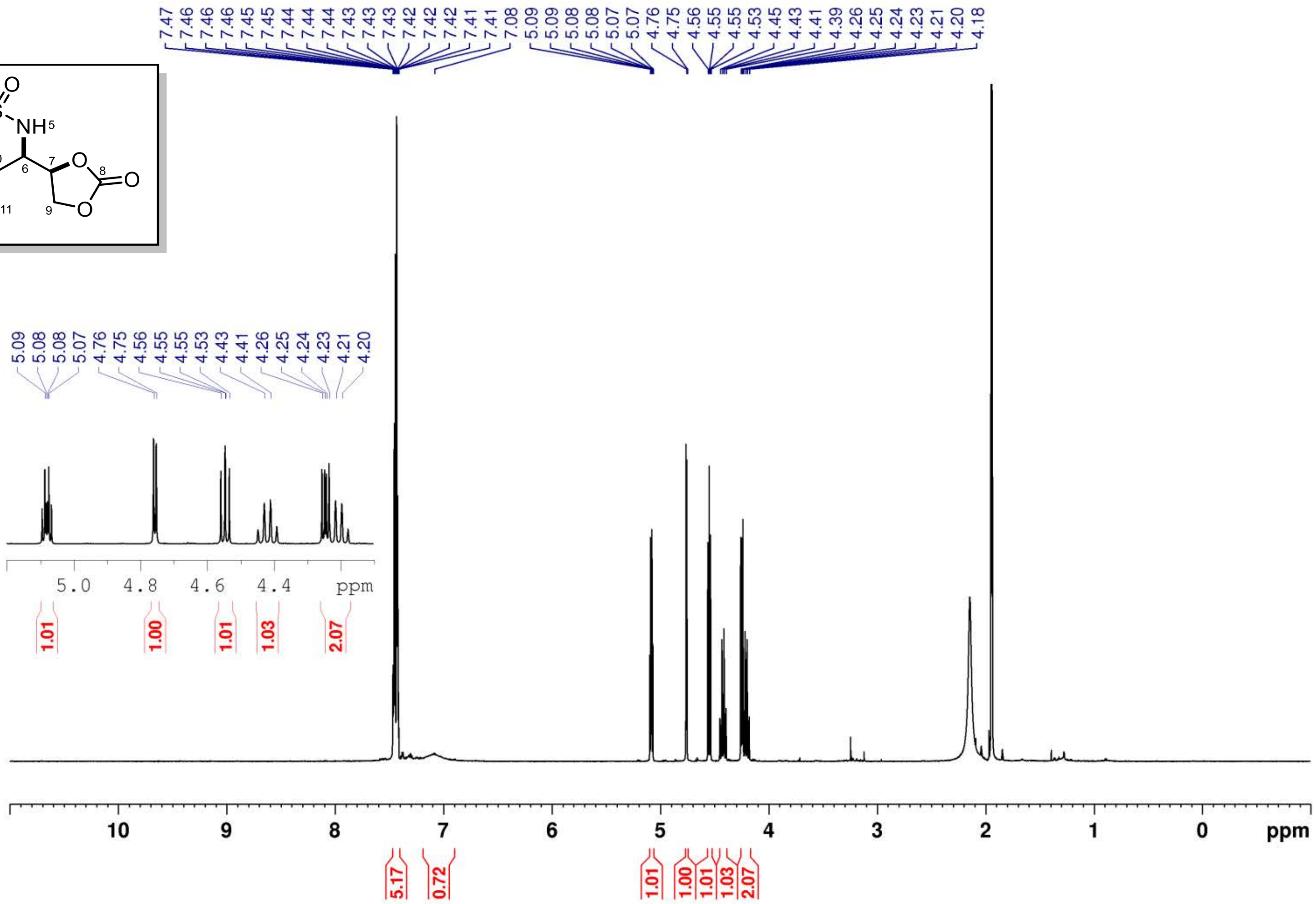

^{13}C NMR (176 MHz, CD_3CN) for 2,2,3,3,4,4,4-heptafluorobutyl ((2-oxo-1,3-dioxolan-4-yl)(phenyl)methyl)sulfamate (**S3**)

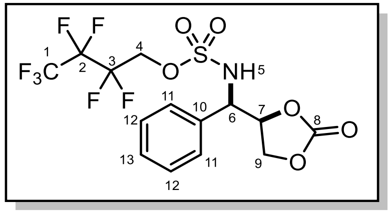

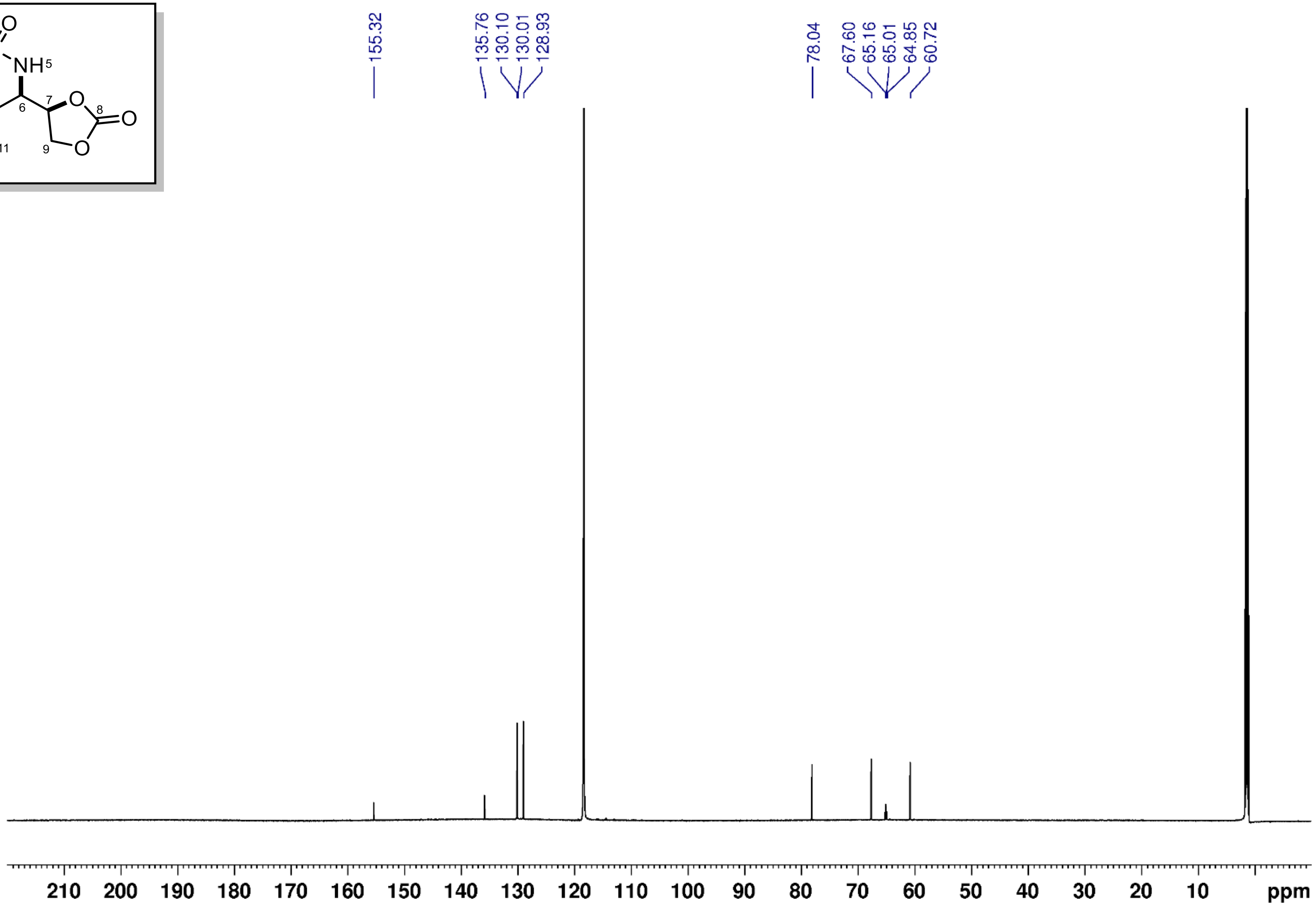

^{19}F NMR (376 MHz, CD_3CN) for 2,2,3,3,4,4,4-heptafluorobutyl ((2-oxo-1,3-dioxolan-4-yl)(phenyl)methyl)sulfamate (**S3**)

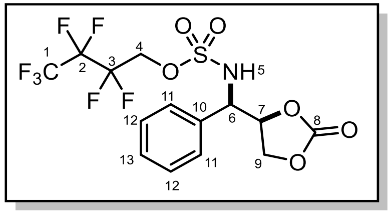

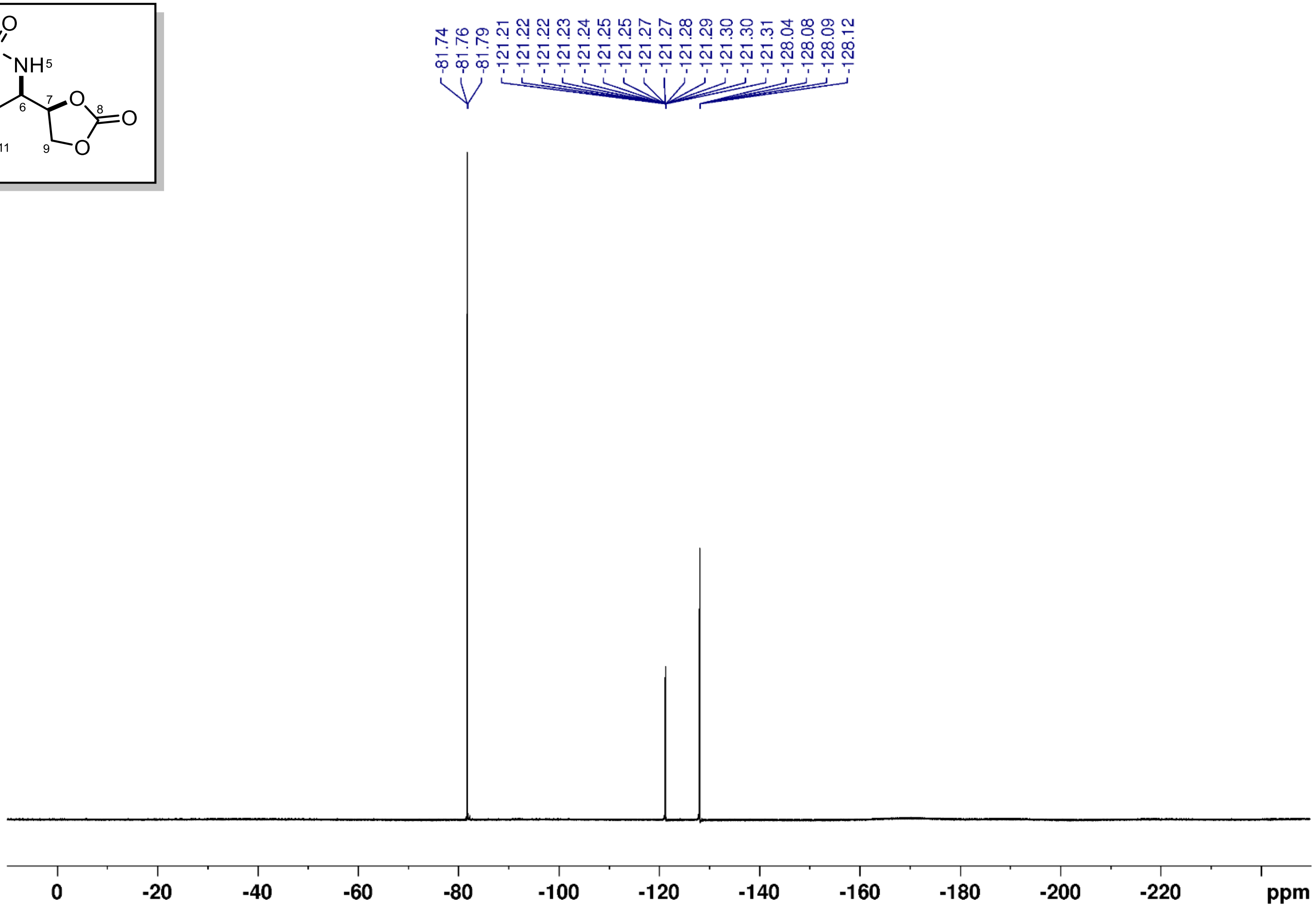

NMR Spectra for Post-Functionalisation Products from Aziridine

^{1}H NMR (700 MHz, CD_3CN) for 2,2,3,3,4,4,4-heptafluorobutyl (2*S*,3*S*)-2-(((methyl(phenyl)carbamoyl)oxy)methyl)-3-phenylaziridine-1-sulfonate (**3a**)

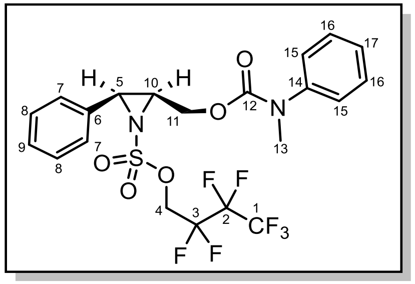

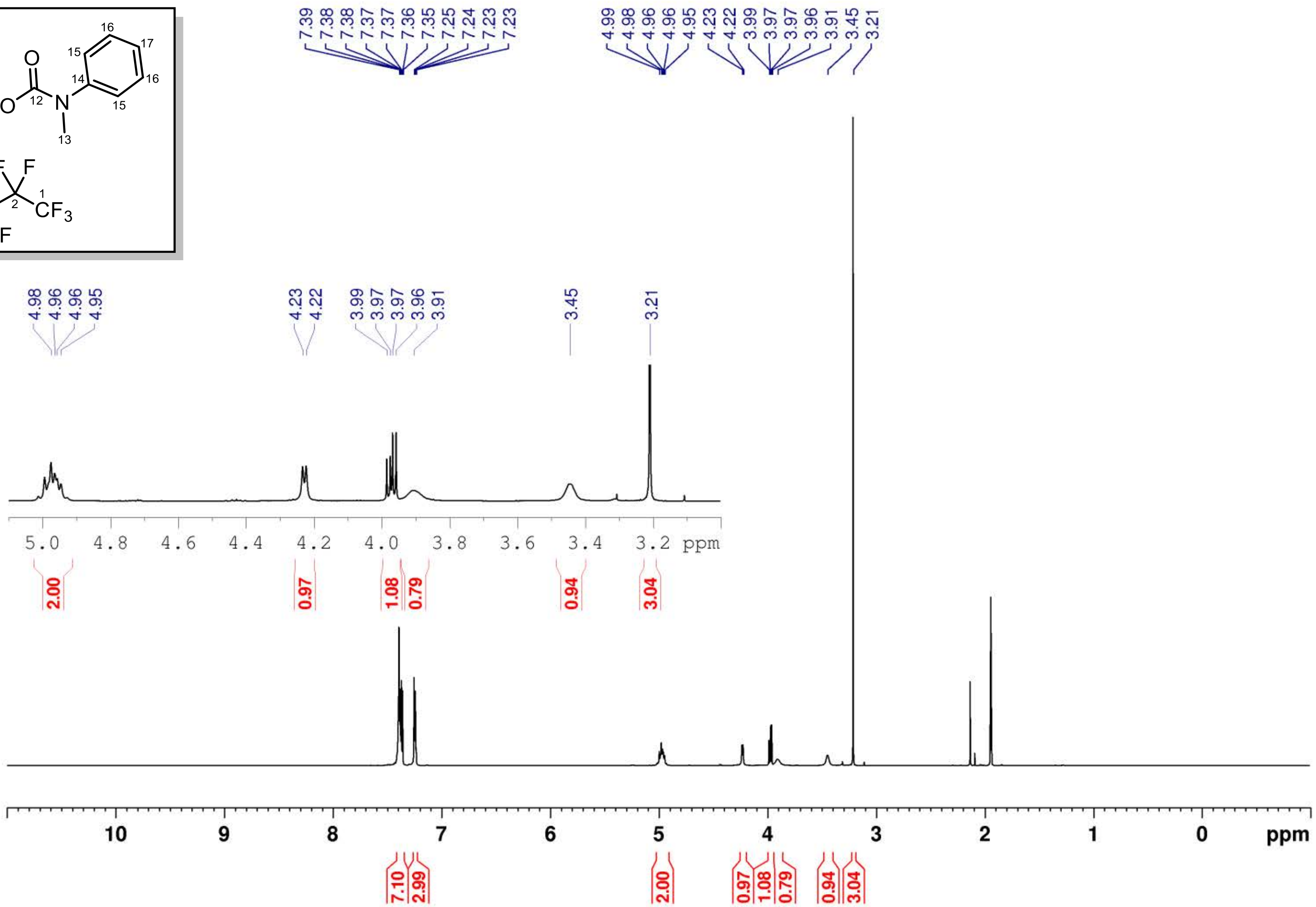

^{13}C NMR (176 MHz, CD_3CN) for 2,2,3,3,4,4,4-heptafluorobutyl (2*S*,3*S*)-2-(((methyl(phenyl)carbamoyl)oxy)methyl)-3-phenylaziridine-1-sulfonate (**3a**)

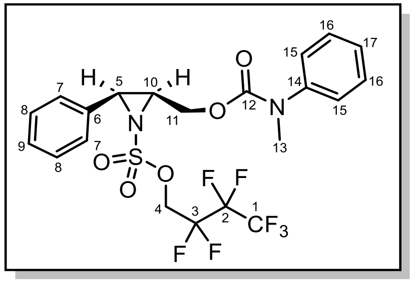

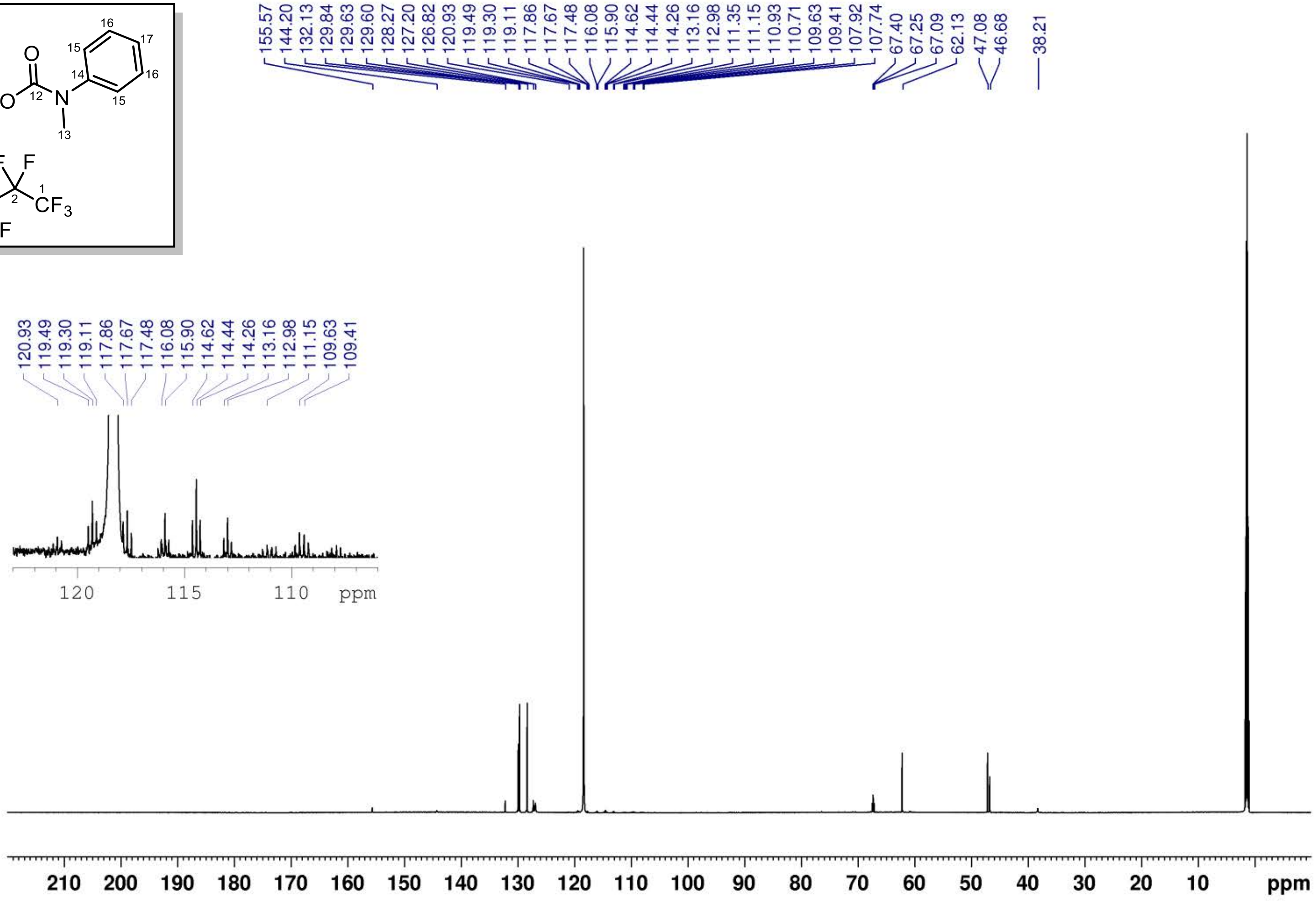

^{19}F NMR (176 MHz, CD_3CN) for 2,2,3,3,4,4,4-heptafluorobutyl (2*S*,3*S*)-2-(((methyl(phenyl)carbamoyl)oxy)methyl)-3-phenylaziridine-1-sulfonate (**3a**)

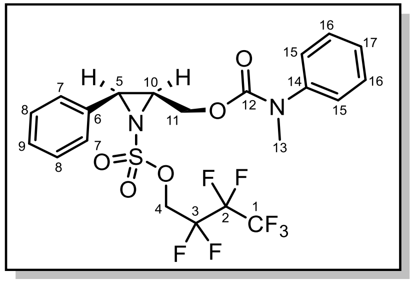

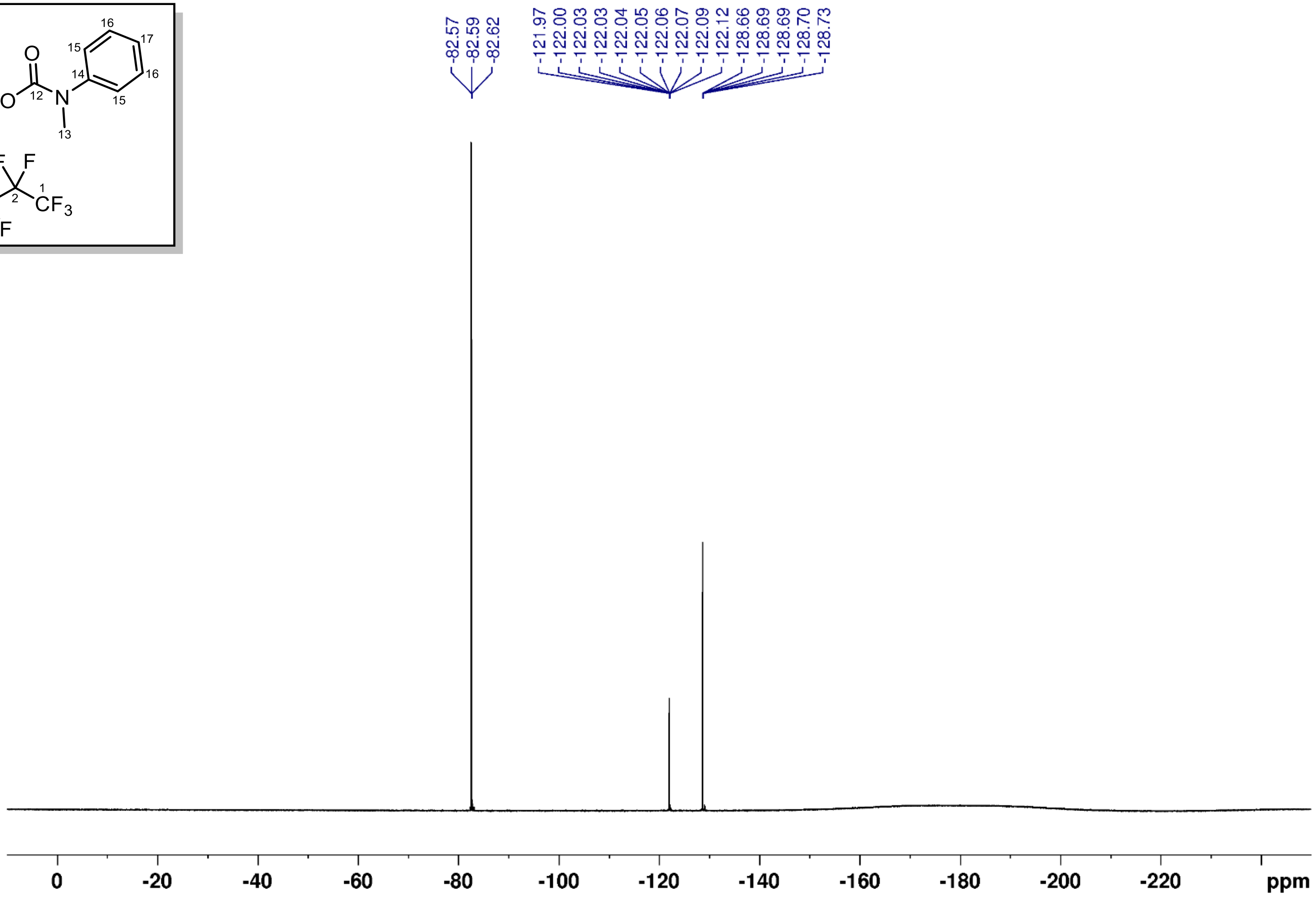

^{1}H NMR (400 MHz, $CDCl_3$) for 2,2,3,3,4,4,4-heptafluorobutyl 2-(4-chlorophenyl)-3-(((methyl(phenyl)carbamoyl)oxy)methyl)aziridine-1-sulfonate

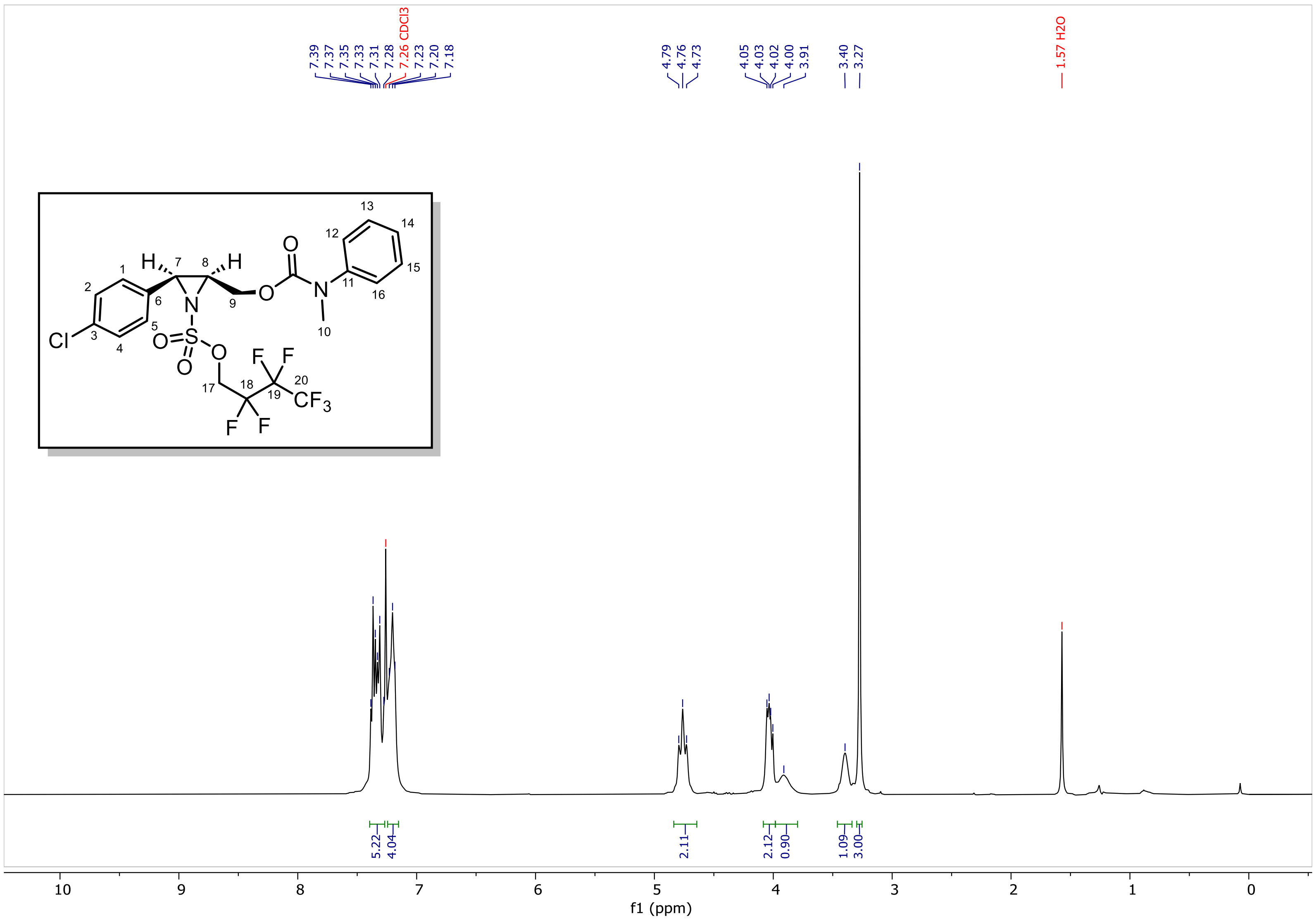

^{1}H NMR (400 MHz, CD_2Cl_2) for 2,2,3,3,4,4,4-heptafluorobutyl 2-(((methyl(phenyl)carbamoyl)oxy)methyl)-3-(4-(trifluoromethyl)phenyl)aziridine-1-sulfonate

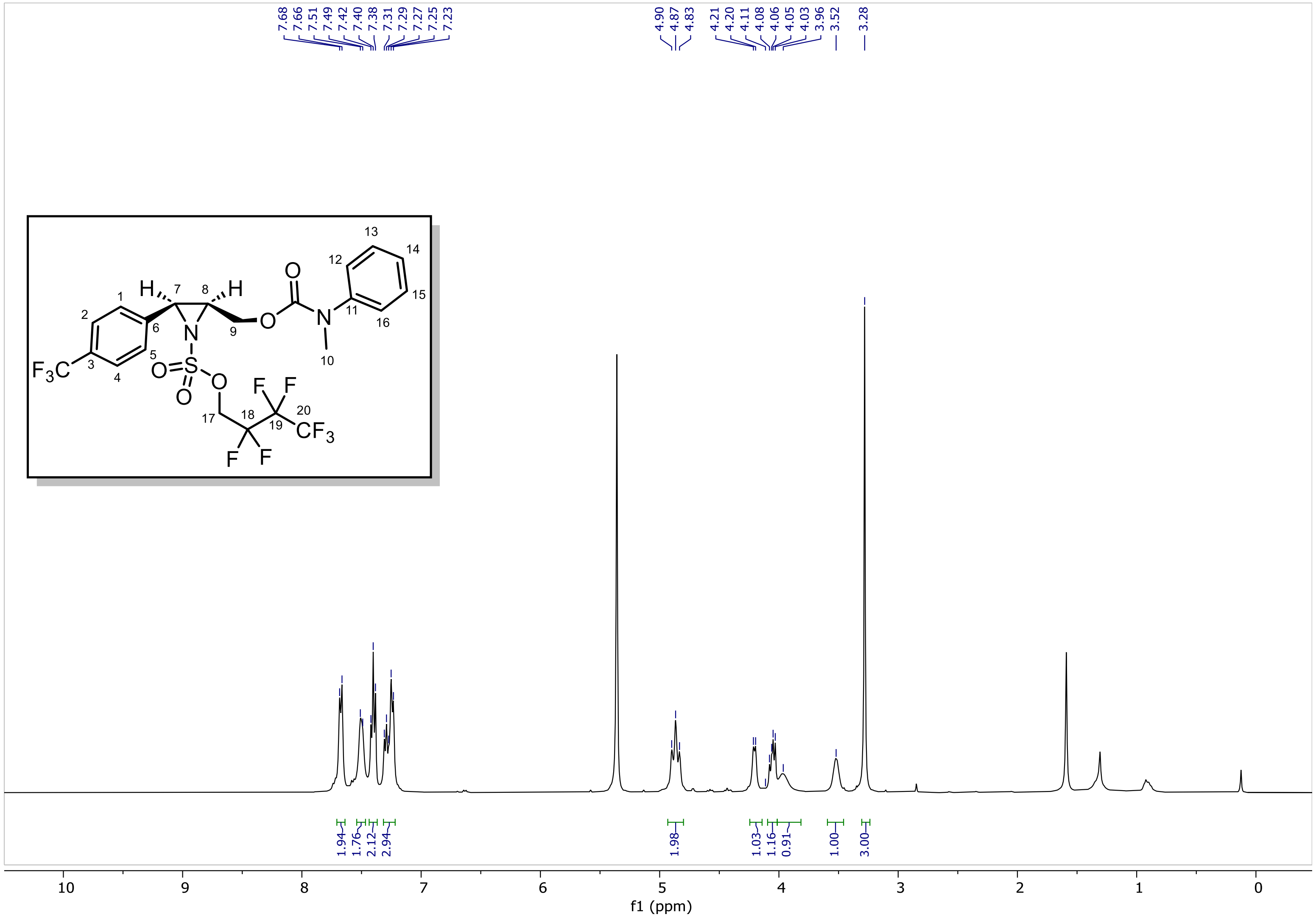

^{1}H NMR (700 MHz, CD_3CN) for 2,2,3,3,4,4,4-heptafluorobutyl ((1*R*,2*S*)-1-azido-3-((methyl(phenyl)carbamoyl)oxy)-1-phenylpropan-2-yl)sulfamate (**5**)

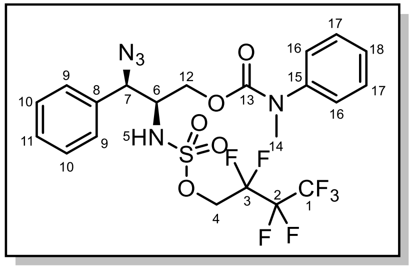

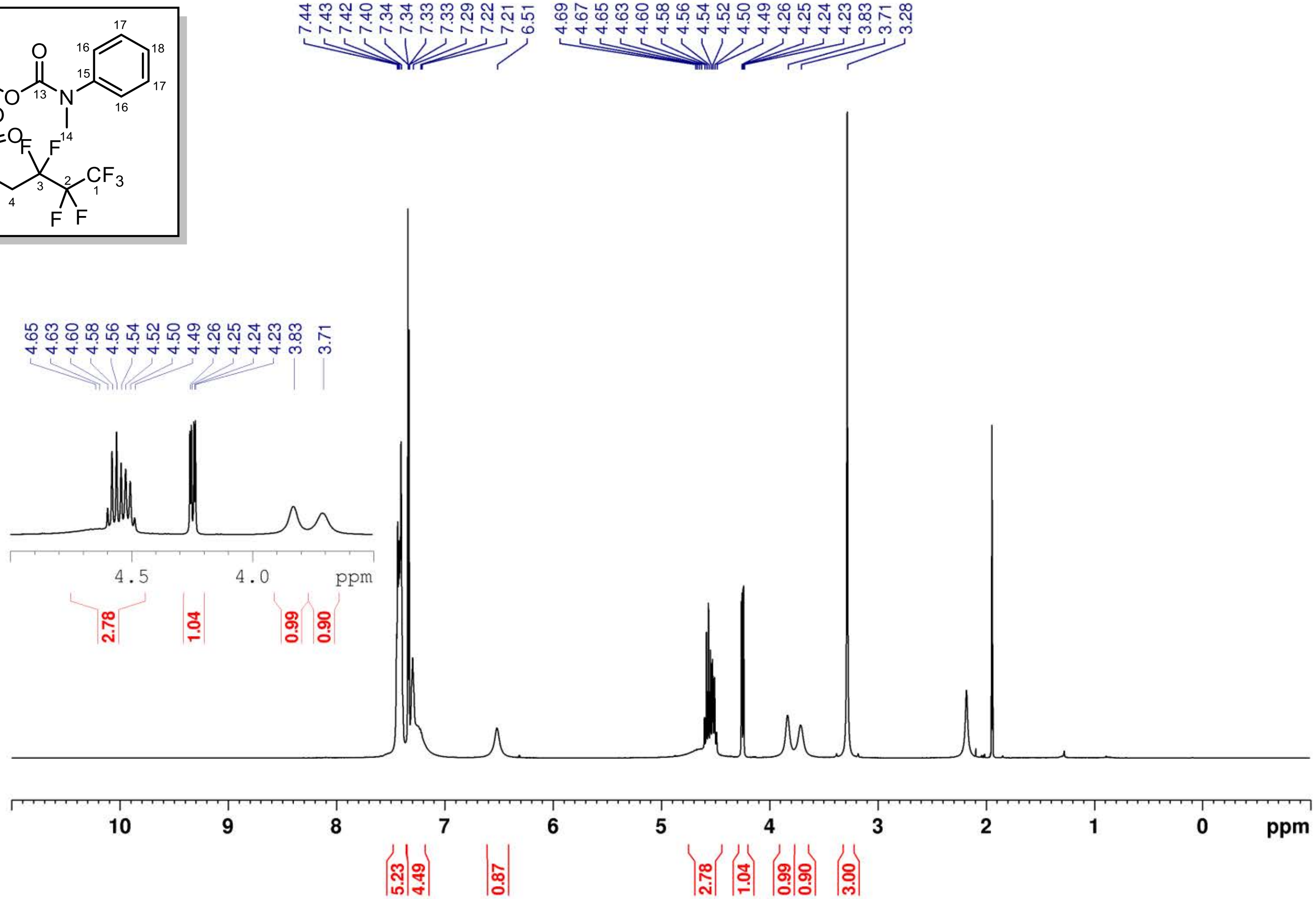

^{13}C NMR (176 MHz, CD_3CN) for 2,2,3,3,4,4,4-heptafluorobutyl ((1*R*,2*S*)-1-azido-3-((methyl(phenyl)carbamoyl)oxy)-1-phenylpropan-2-yl)sulfamate (**5**)

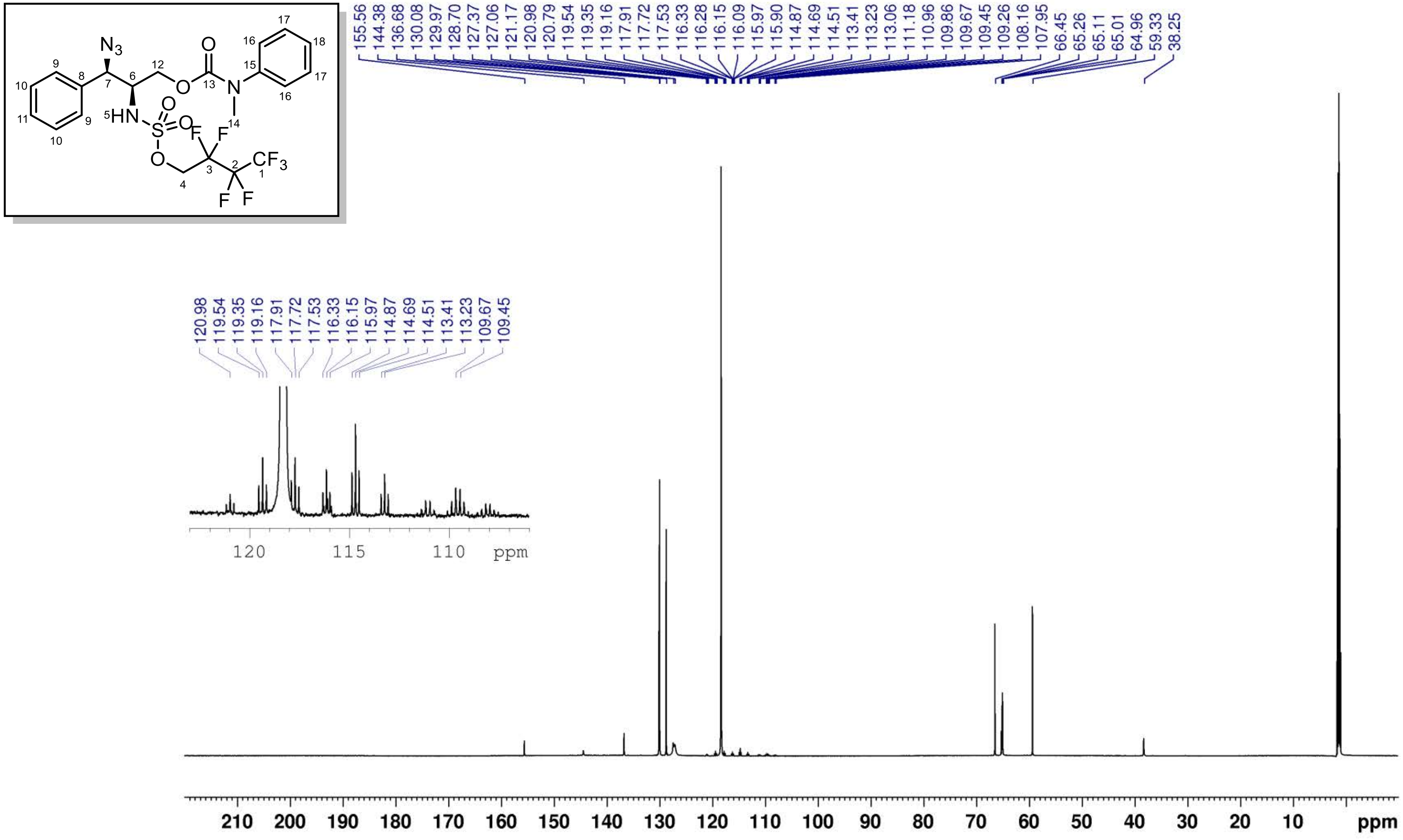

^{19}F NMR (376 MHz, CD_3CN) for 2,2,3,3,4,4,4-heptafluorobutyl ((1*R*,2*S*)-1-azido-3-((methyl(phenyl)carbamoyl)oxy)-1-phenylpropan-2-yl)sulfamate (**5**)

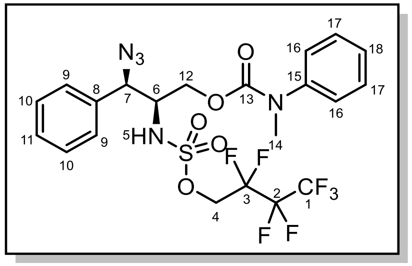

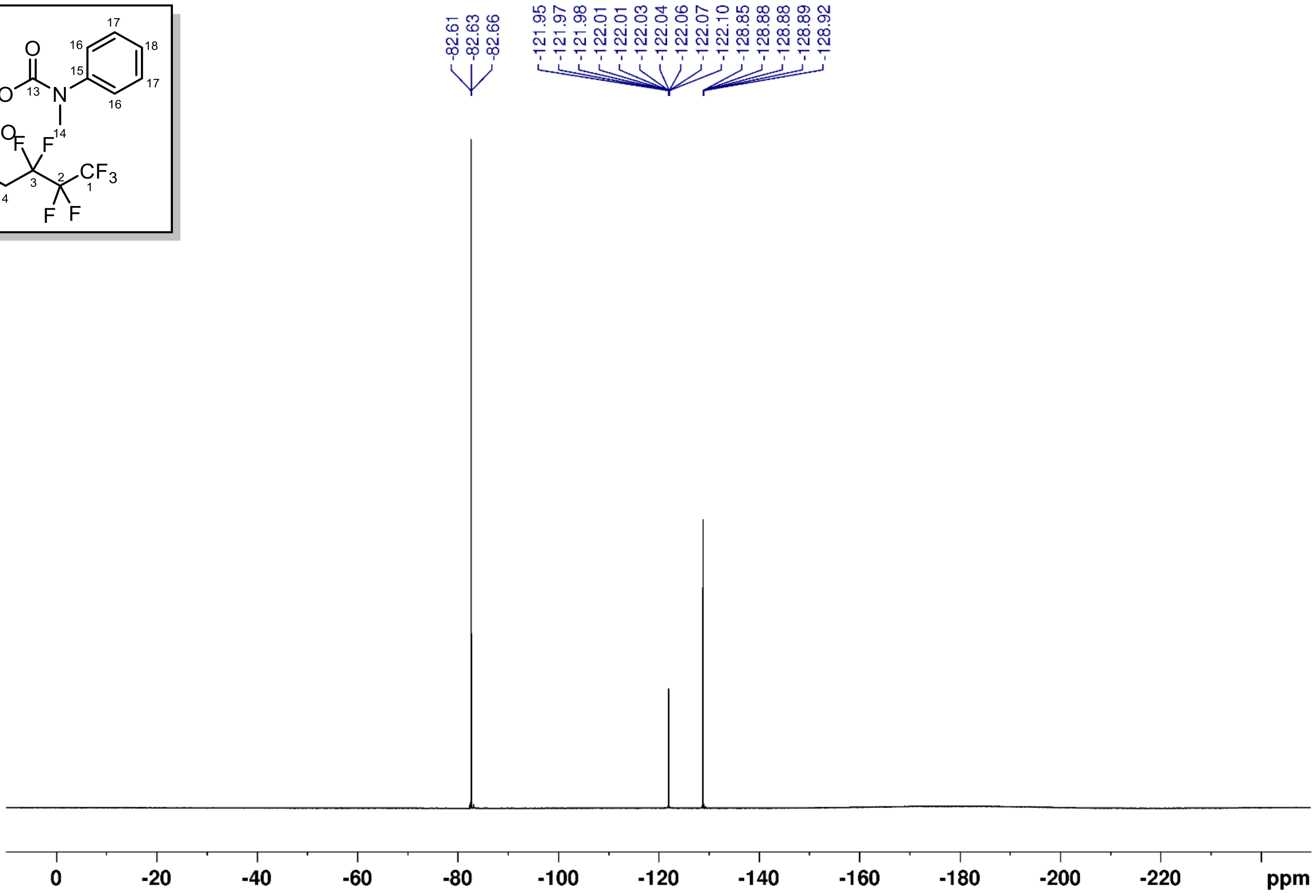

^{1}H NMR (700 MHz, CD_3CN) for 2,2,3,3,4,4,4-heptafluorobutyl ((1*S*,2*R*)-2-azido-3-((methyl(phenyl)carbamoyl)oxy)-1-phenylpropyl)sulfamate (**6**)

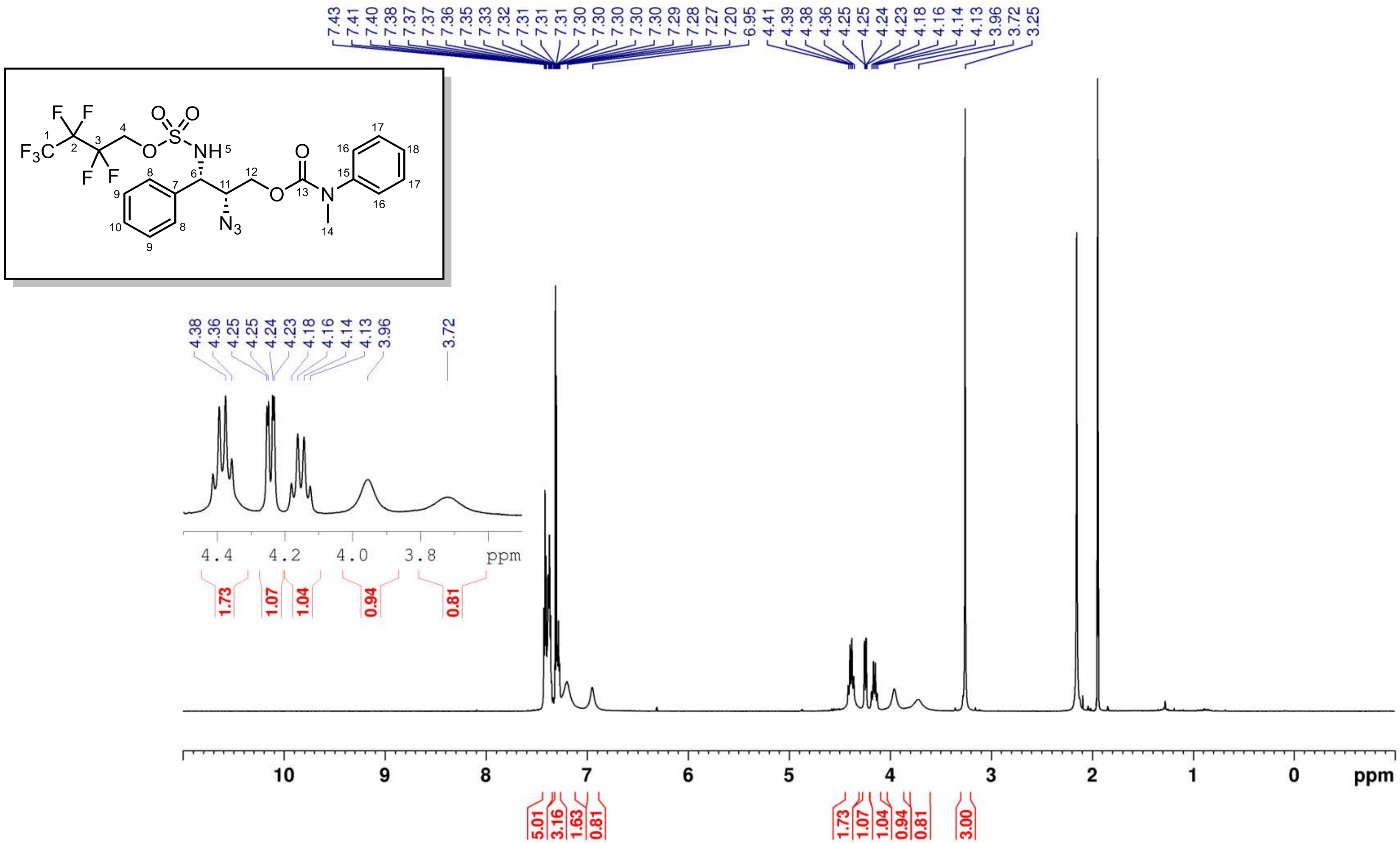

^{13}C NMR (176 MHz, CD_3CN) for 2,2,3,3,4,4,4-heptafluorobutyl ((1*S*,2*R*)-2-azido-3-((methyl(phenyl)carbamoyl)oxy)-1-phenylpropyl)sulfamate (**6**)

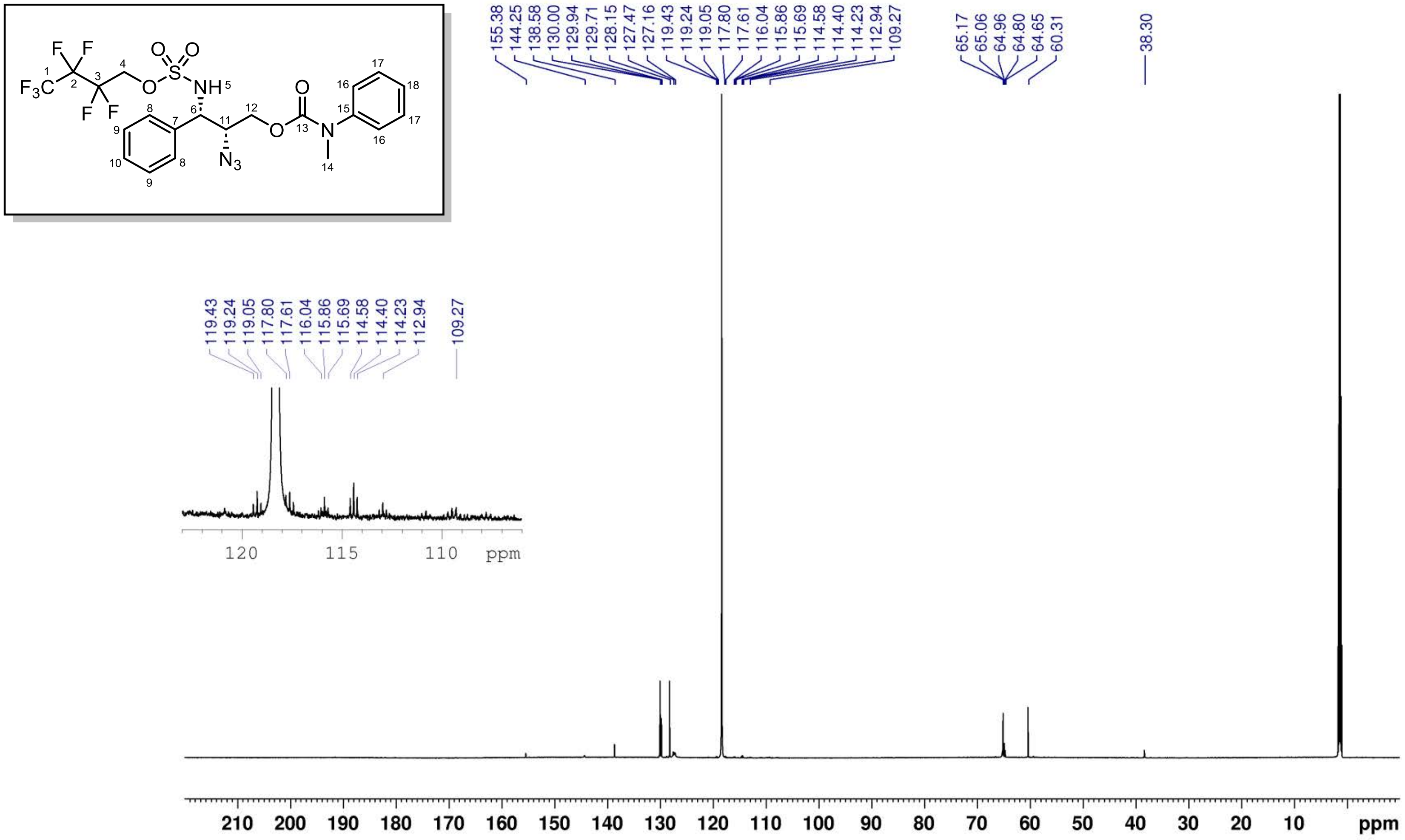

^{19}F NMR (376 MHz, CD_3CN) for 2,2,3,3,4,4,4-heptafluorobutyl ((1*S*,2*R*)-2-azido-3-((methyl(phenyl)carbamoyl)oxy)-1-phenylpropyl)sulfamate (**6**)

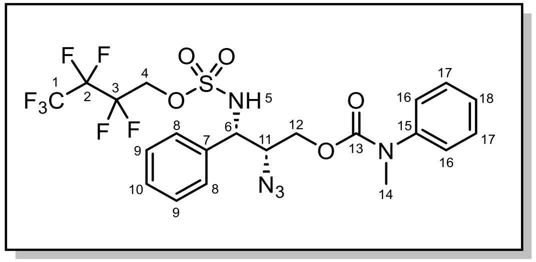

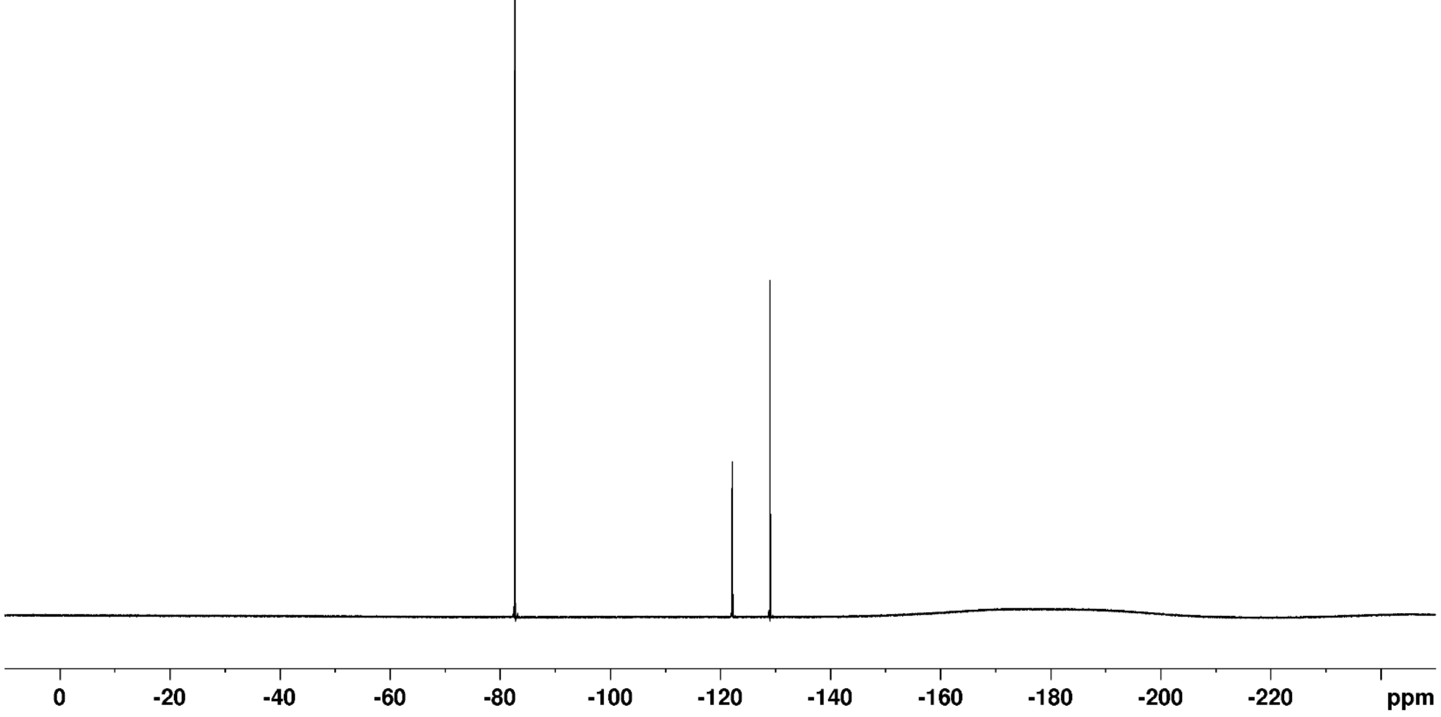

^{1}H NMR (700 MHz, CD_3CN) for 2,2,3,3,4,4,4-heptafluorobutyl (*S*)-(1-((methyl(phenyl)carbamoyl)oxy)-3-phenylpropan-2-yl)sulfamate (**7**)

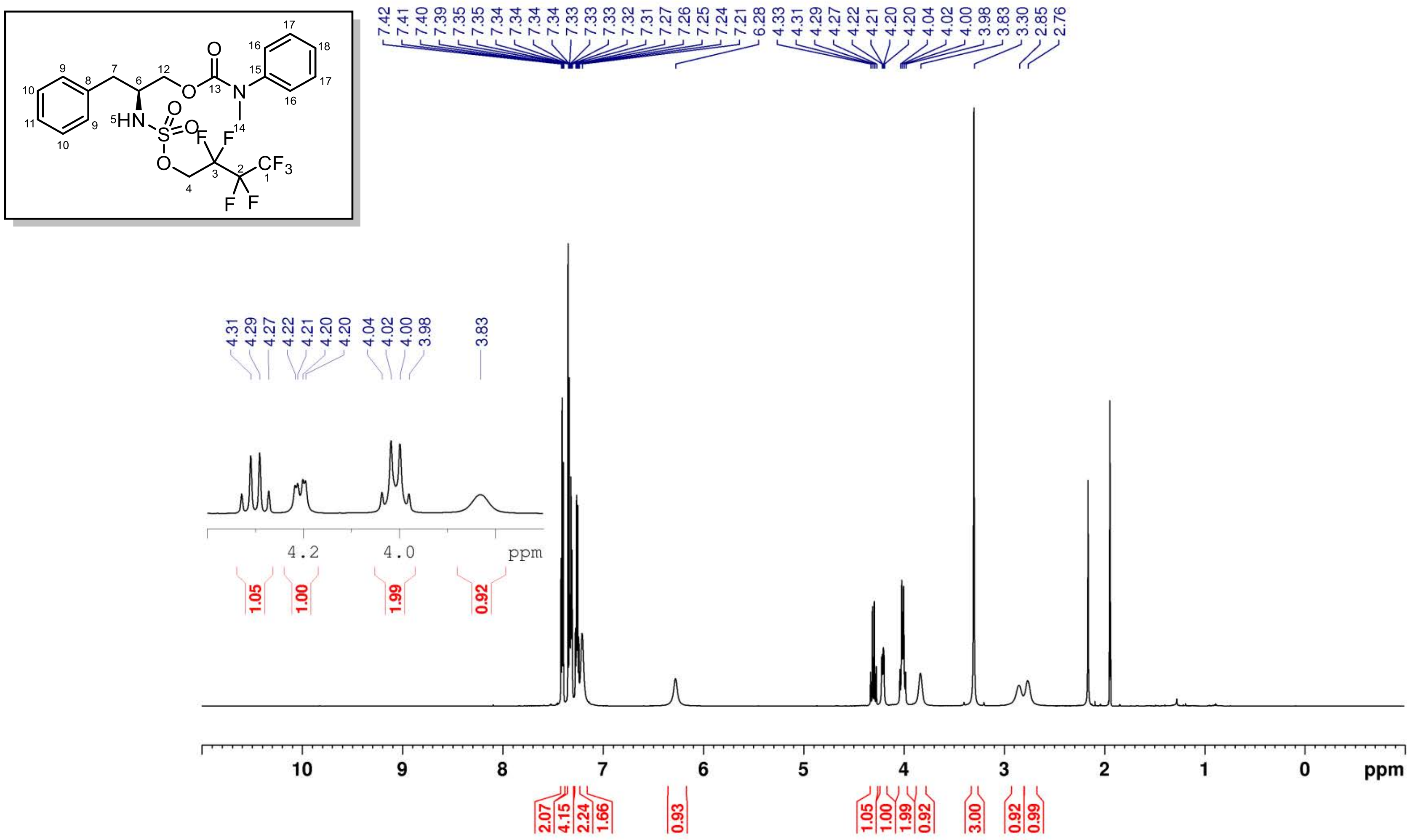

^{13}C NMR (176 MHz, CD_3CN) for 2,2,3,3,4,4,4-heptafluorobutyl (*S*)-(1-((methyl(phenyl)carbamoyl)oxy)-3-phenylpropan-2-yl)sulfamate (**7**)

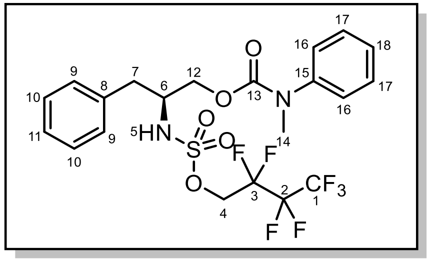

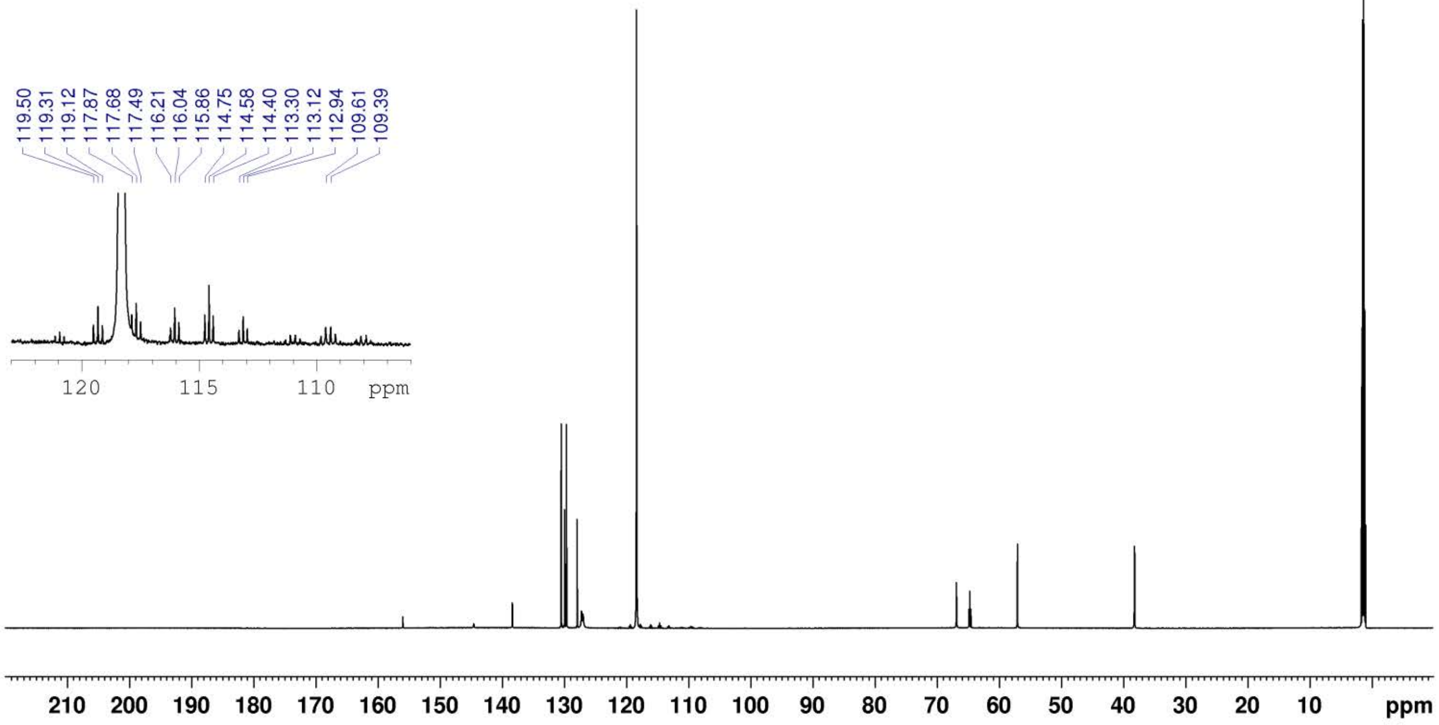

^{19}F NMR (376 MHz, CD_3CN) for 2,2,3,3,4,4,4-heptafluorobutyl (*S*)-(1-((methyl(phenyl)carbamoyl)oxy)-3-phenylpropan-2-yl)sulfamate (**7**)

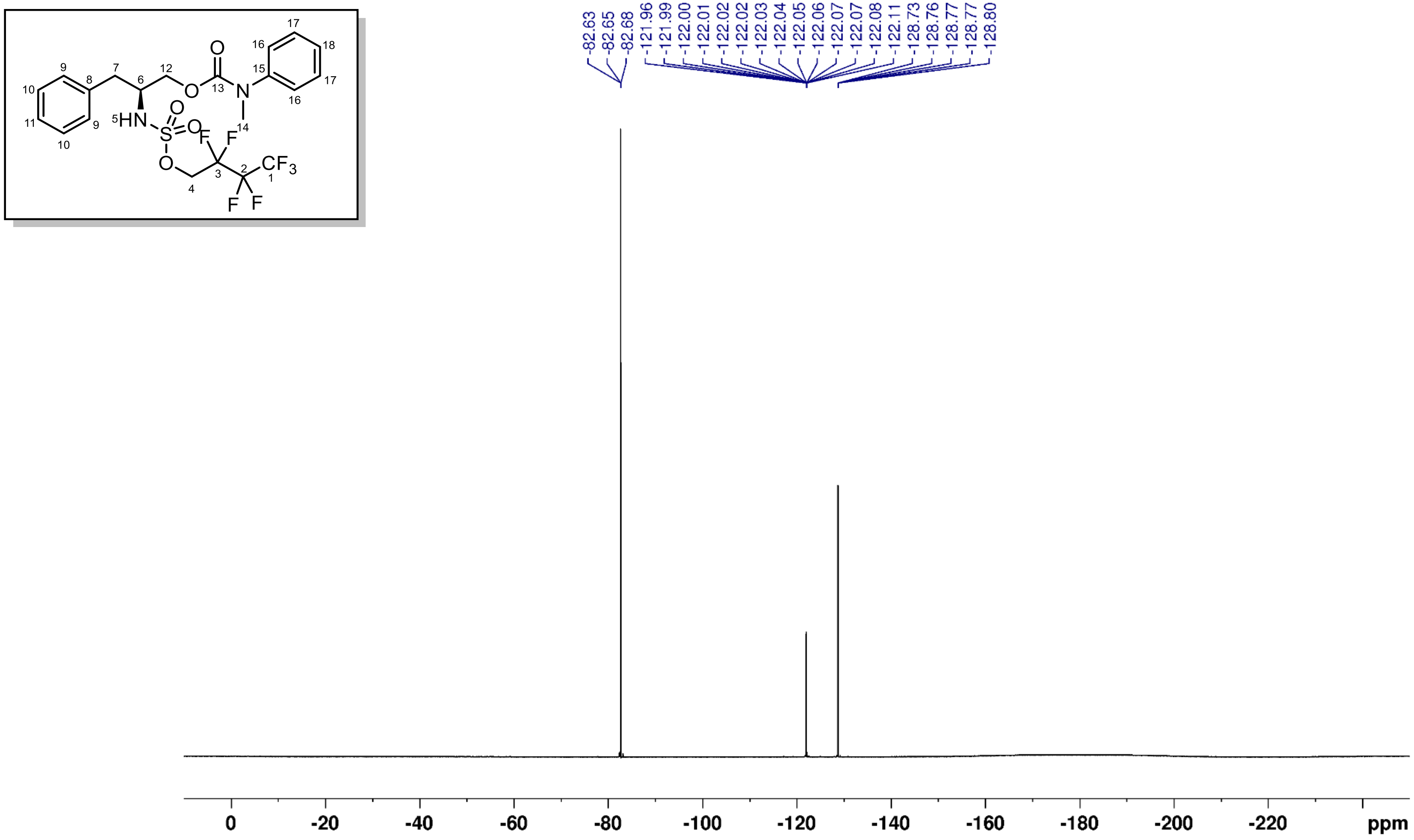

¹H NMR (500 MHz, CD_3CN) for 2,2,3,3,4,4,4-heptafluorobutyl ((1*S*,2*R*)-3-chloro-2-((methyl(phenyl)carbamoyl)oxy)-1-phenylpropyl)sulfamate (**8**)

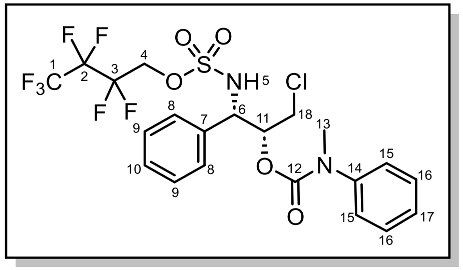

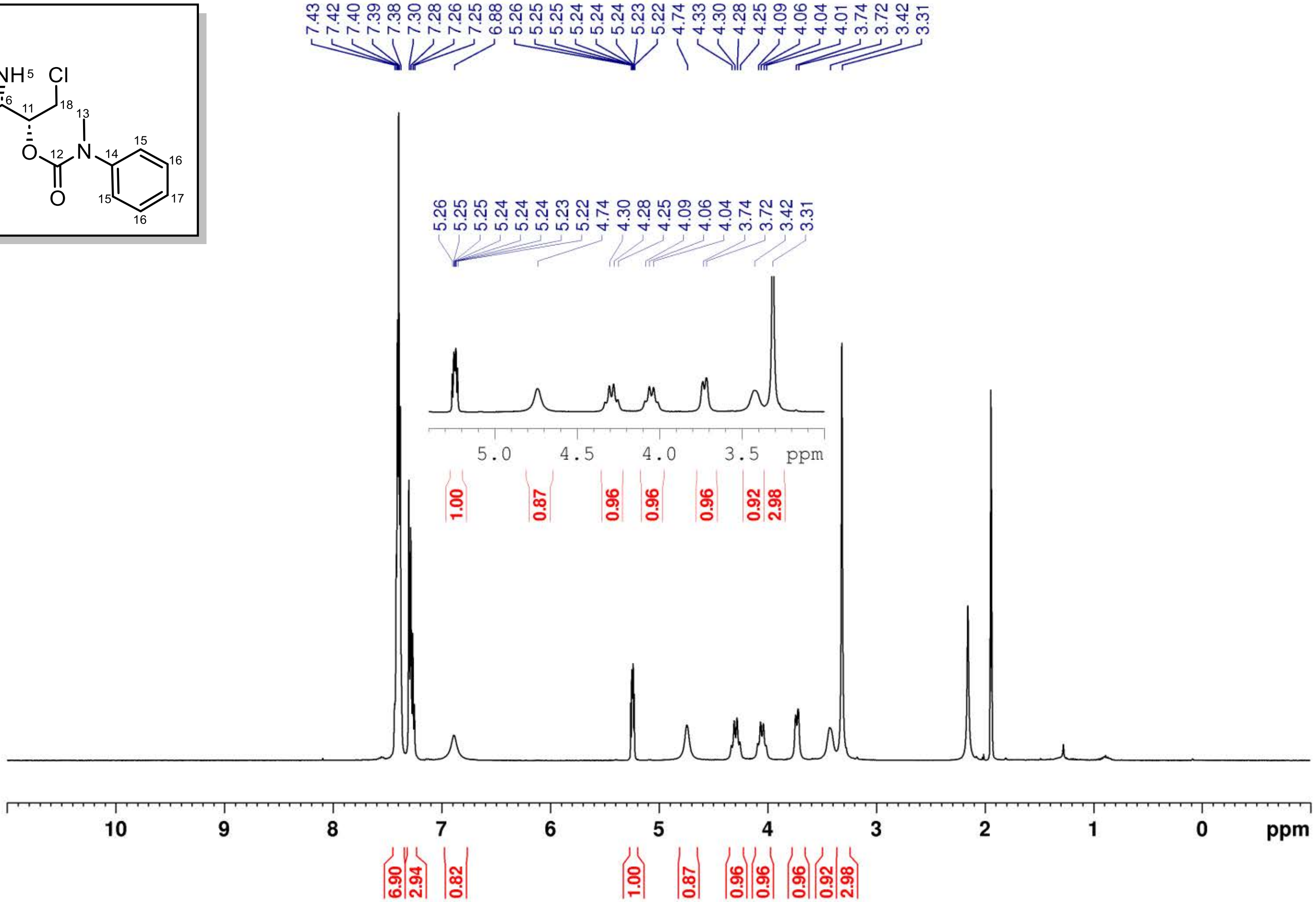

^{13}C NMR (176 MHz, CD_3CN) for 2,2,3,3,4,4,4-heptafluorobutyl ((1*S*,2*R*)-3-chloro-2-((methyl(phenyl)carbamoyl)oxy)-1-phenylpropyl)sulfamate (**8**)

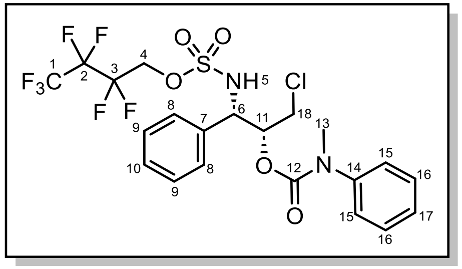

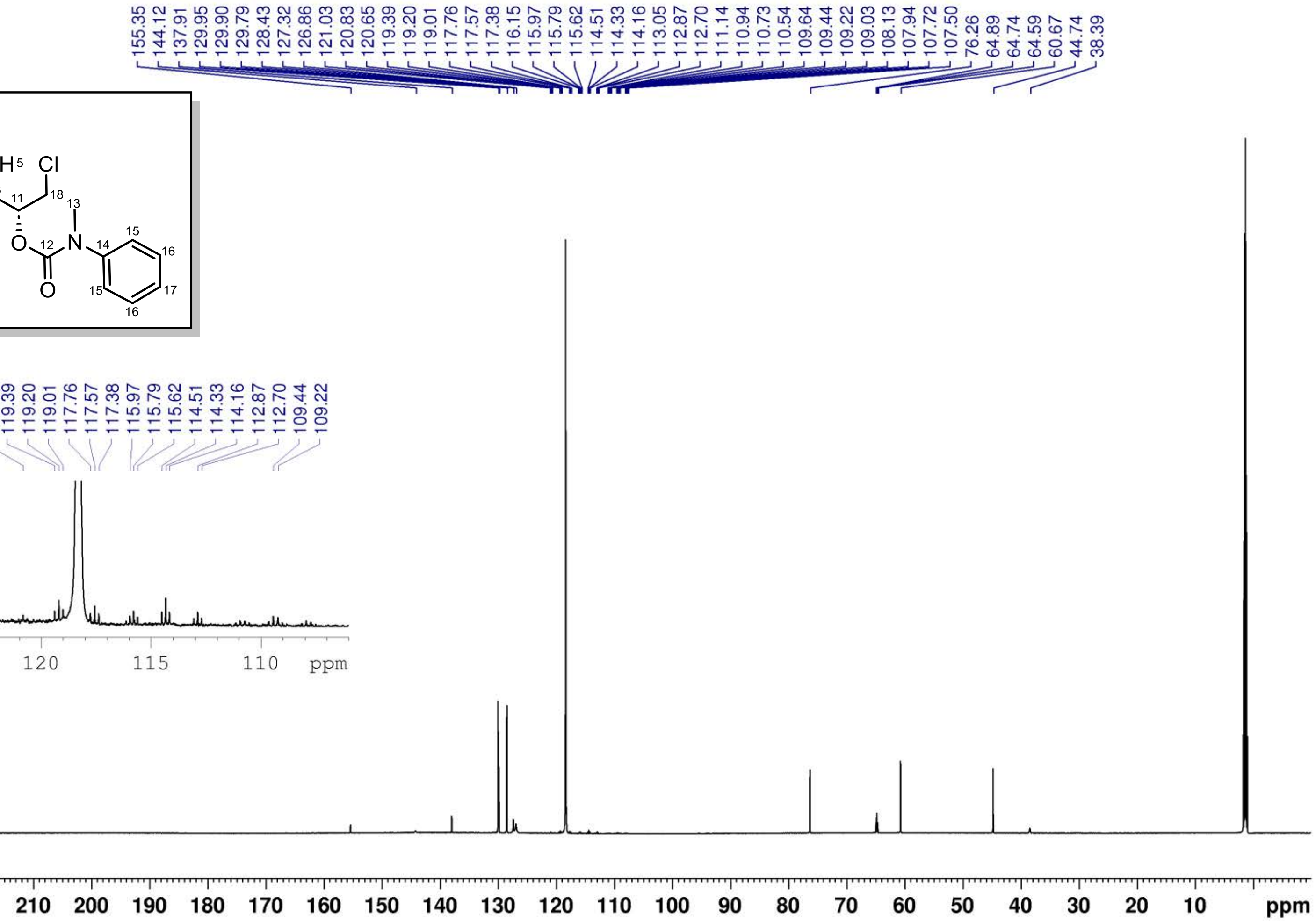

^{19}F NMR (471 MHz, CD_3CN) for 2,2,3,3,4,4,4-heptafluorobutyl ((1*S*,2*R*)-3-chloro-2-((methyl(phenyl)carbamoyl)oxy)-1-phenylpropyl)sulfamate (**8**)

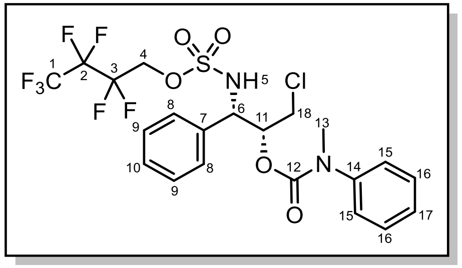

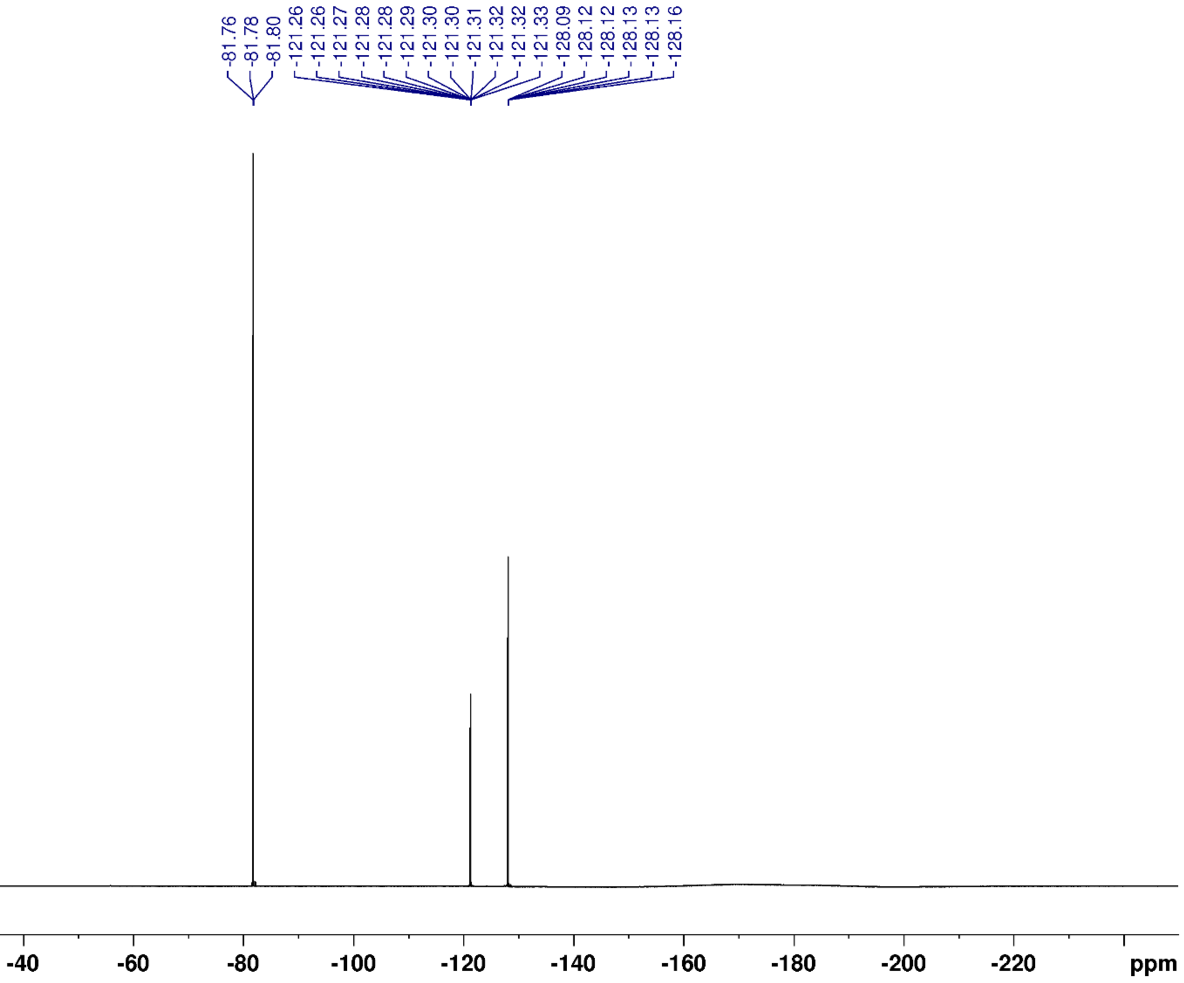

NMR Spectra for Substrates – N-H Carbamates for C-H Amination:

^{1}H NMR (700 MHz, CDCl$_3$) for phenethyl phenylcarbamate

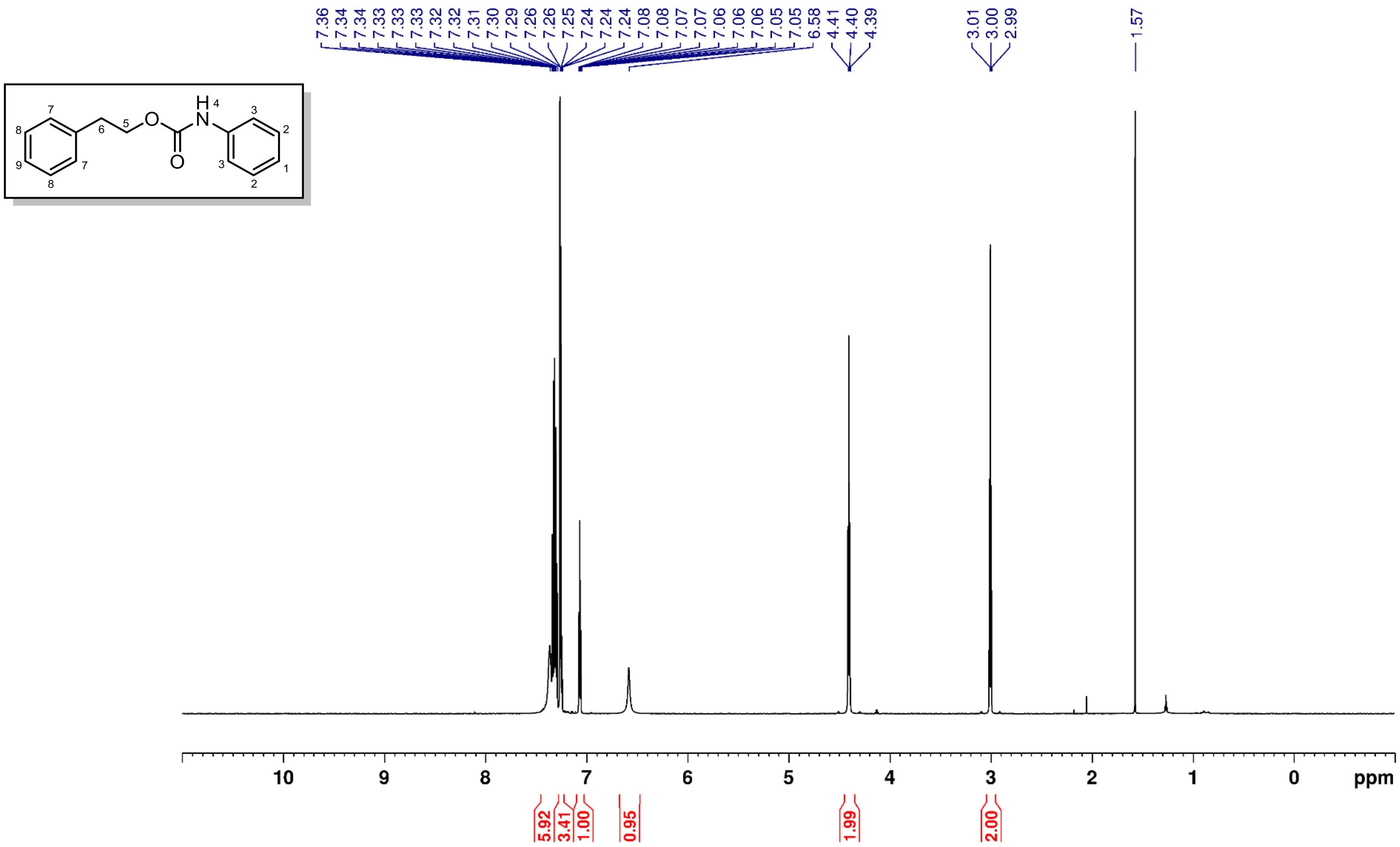

^{13}C NMR (176 MHz, CDCl$_3$) for phenethyl phenylcarbamate

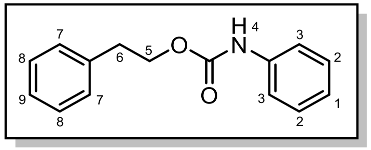

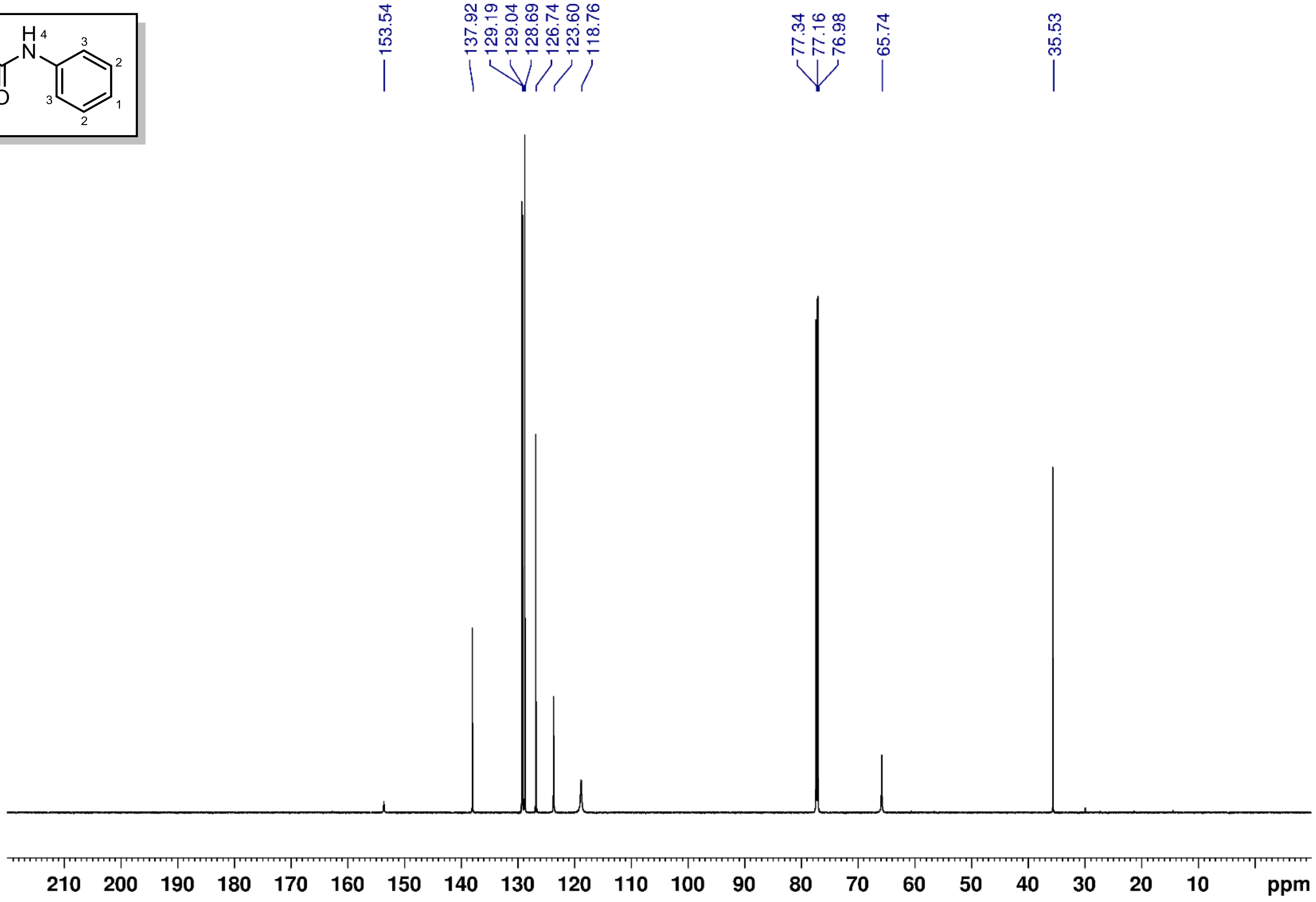

^{1}H NMR (700 MHz, $CDCl_3$) for pentyl phenylcarbamate

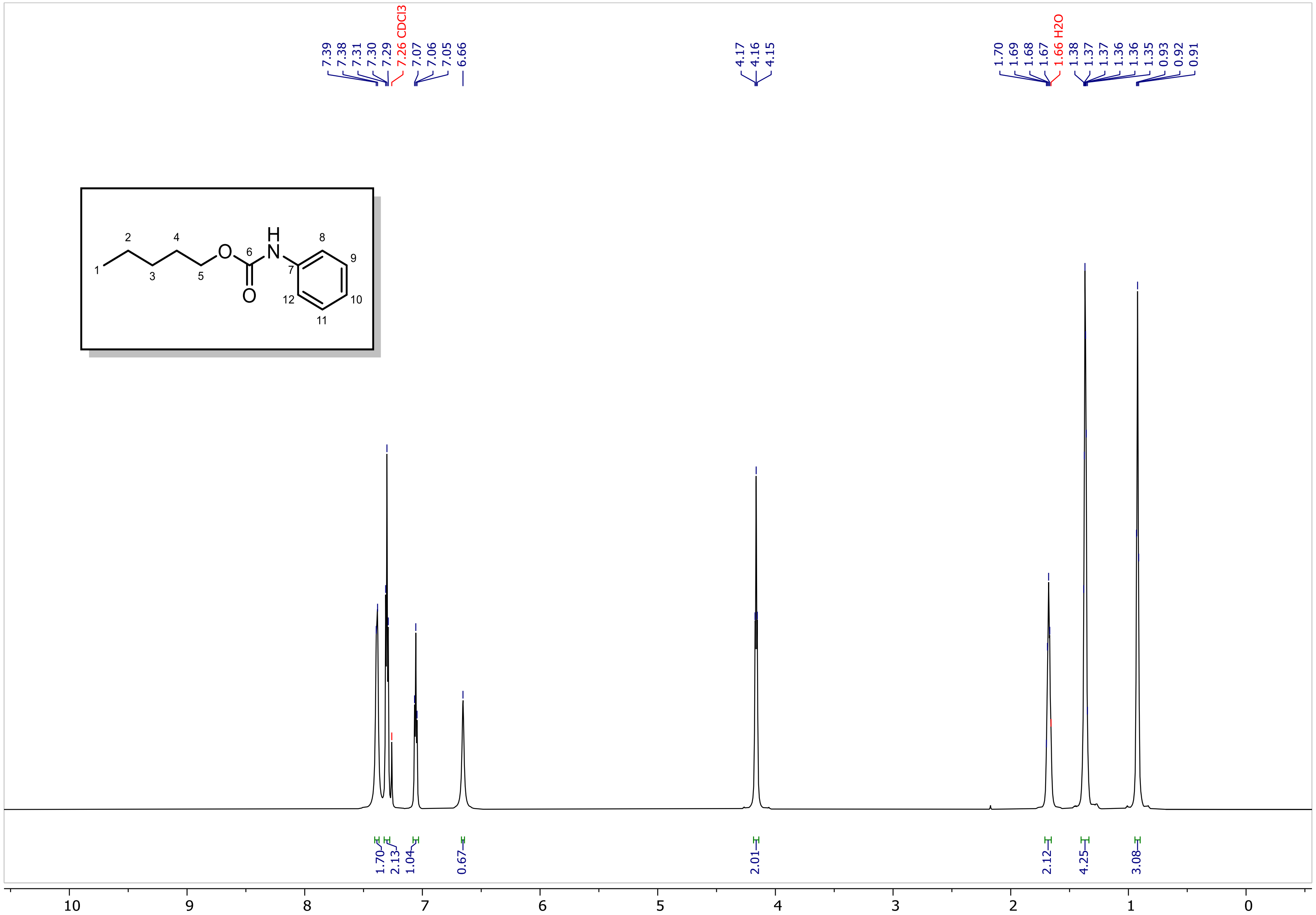

^{13}C NMR (176 MHz, $CDCl_3$) for pentyl phenylcarbamate

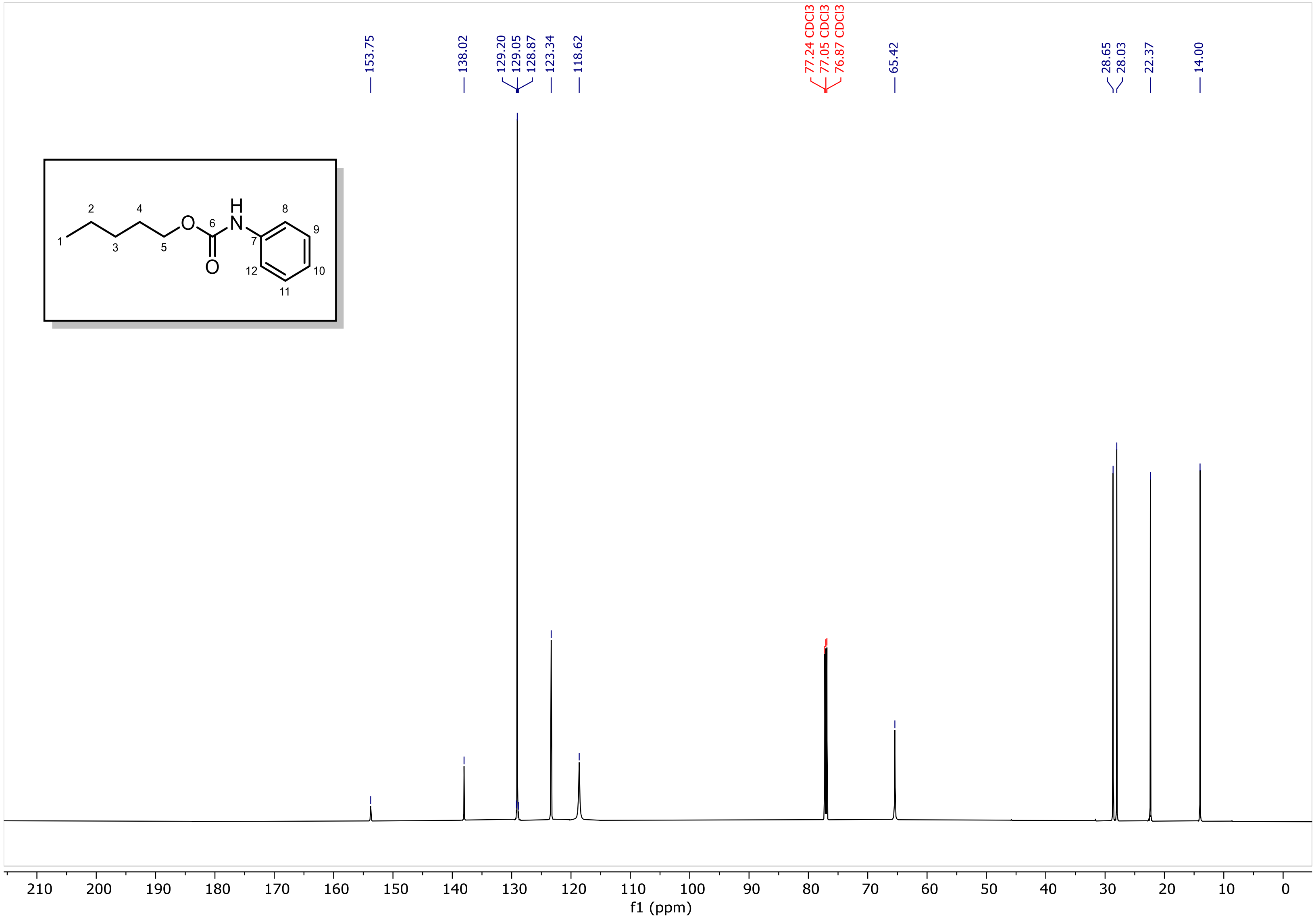

^{1}H NMR (700 MHz, CDCl3) for phenethyl morpholine-4-carboxylate

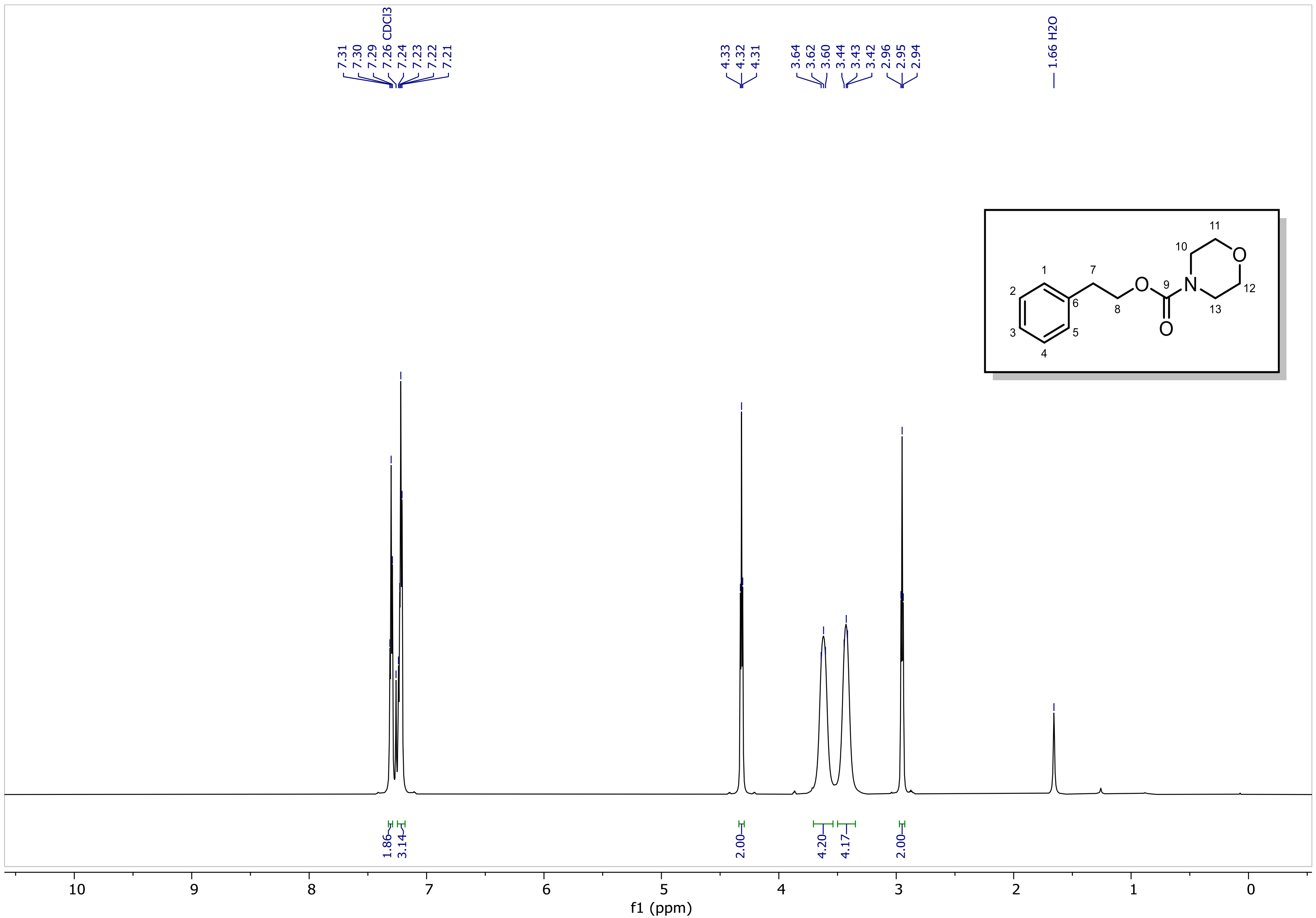

^{13}C NMR (176 MHz, $CDCl_3$) for phenethyl morpholine-4-carboxylate

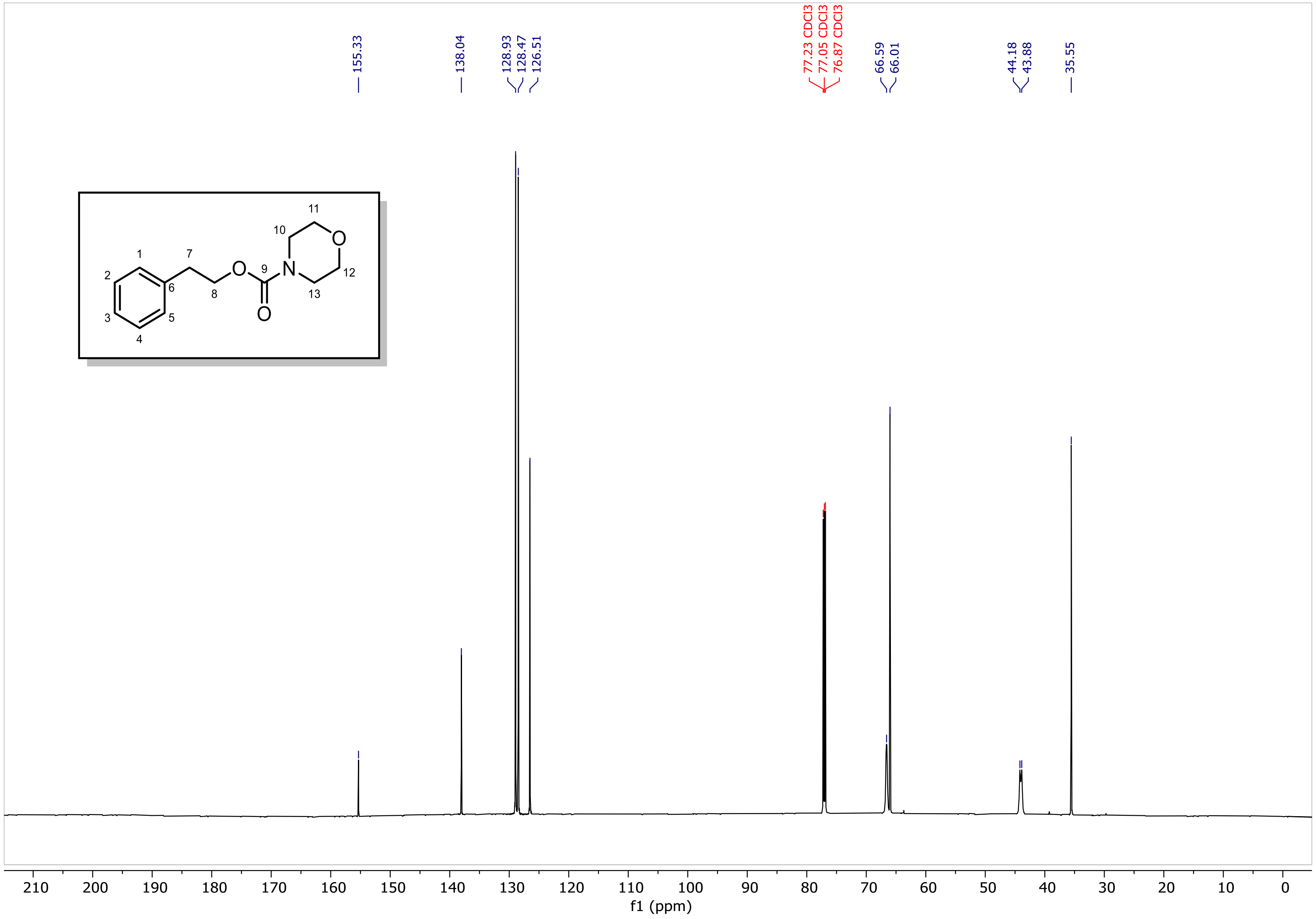

^{1}H NMR (700 MHz, CDCl$_3$) for 4-methoxyphenethyl phenylcarbamate

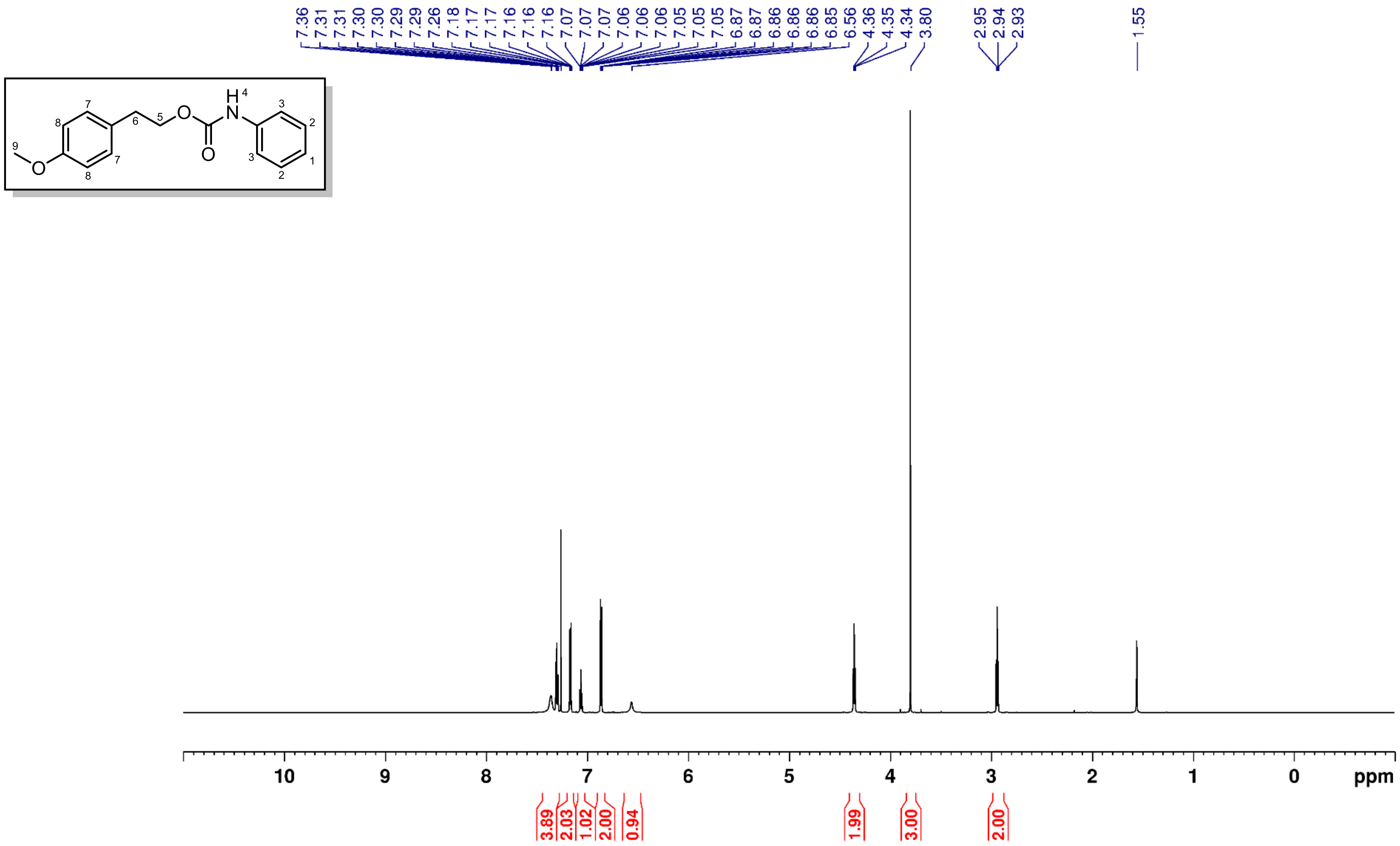

^{13}C NMR (176 MHz, $CDCl_3$) for 4-methoxyphenethyl phenylcarbamate

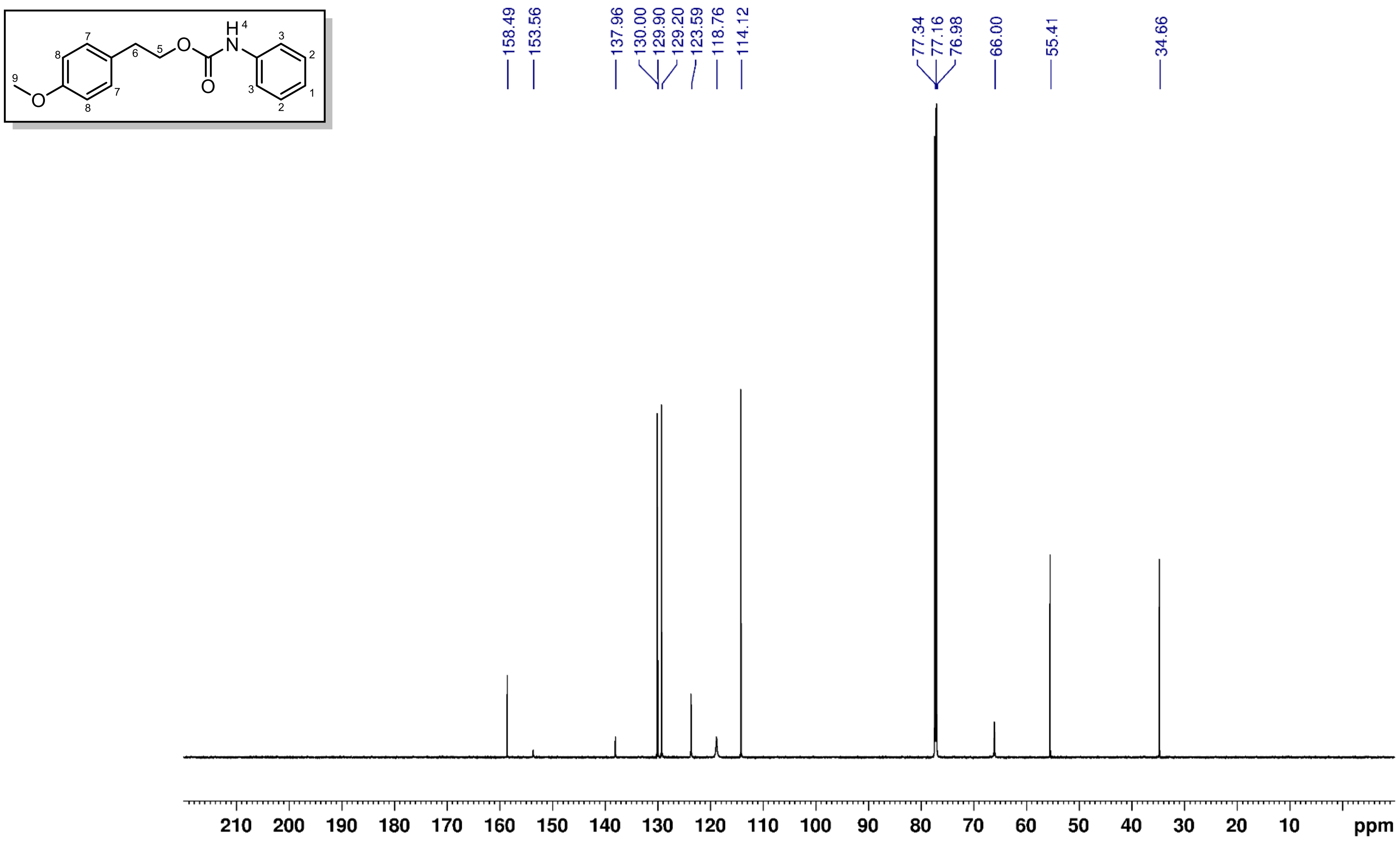

^{1}H NMR (700 MHz, $CDCl_3$) for 4-(*tert*-butyl)phenethyl phenylcarbamate

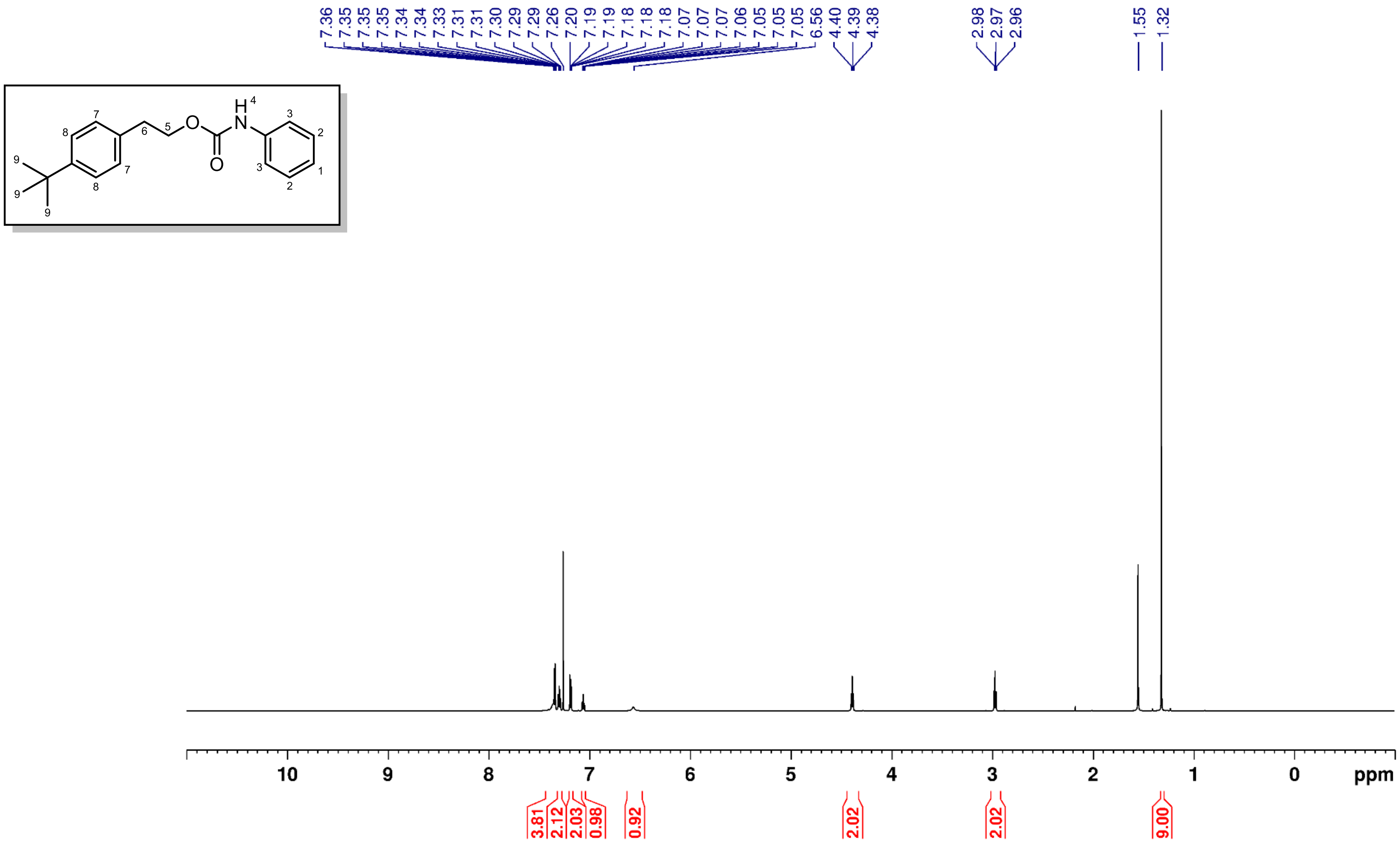

^{13}C NMR (176 MHz, $CDCl_3$) for 4-(*tert*-butyl)phenethyl phenylcarbamate

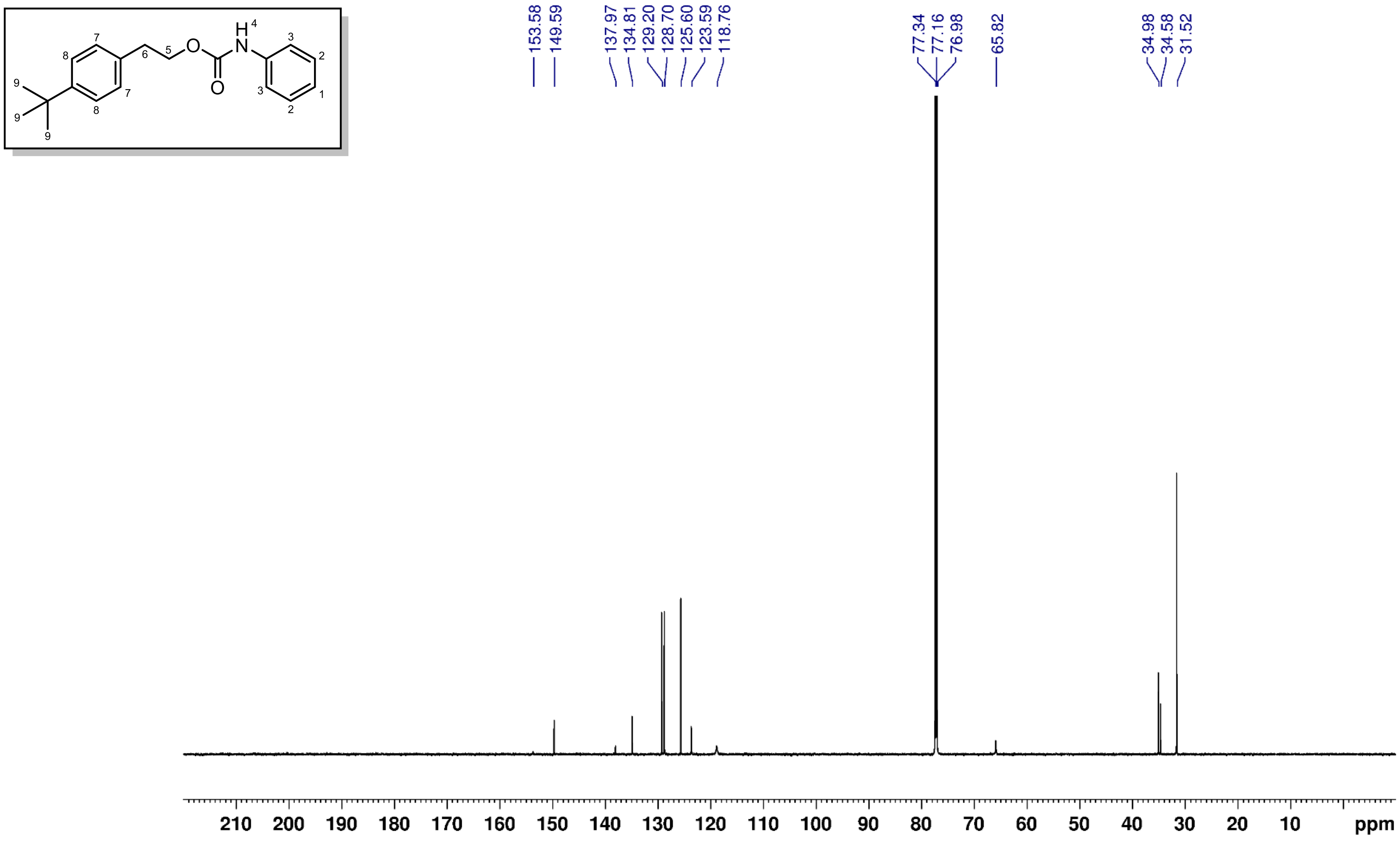

^{1}H NMR (700 MHz, CDCl$_3$) for 4-methylphenethyl phenylcarbamate

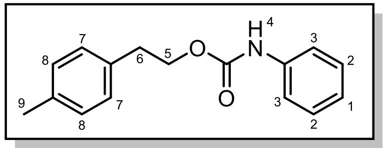

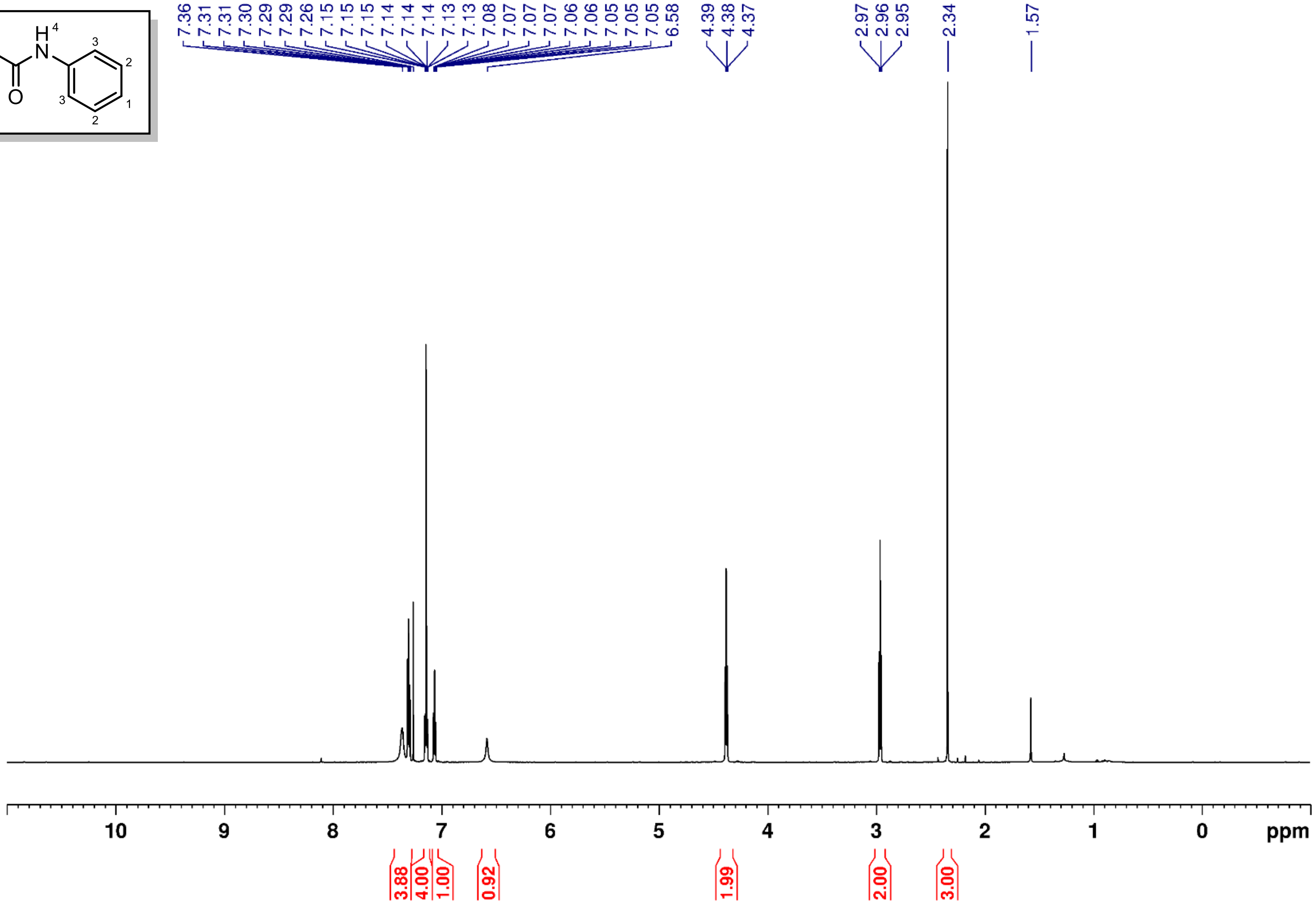

^{13}C NMR (176 MHz, CDCl$_3$) for 4-methylphenethyl phenylcarbamate

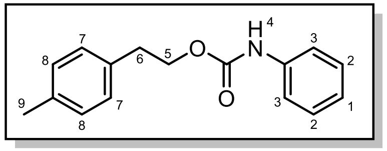

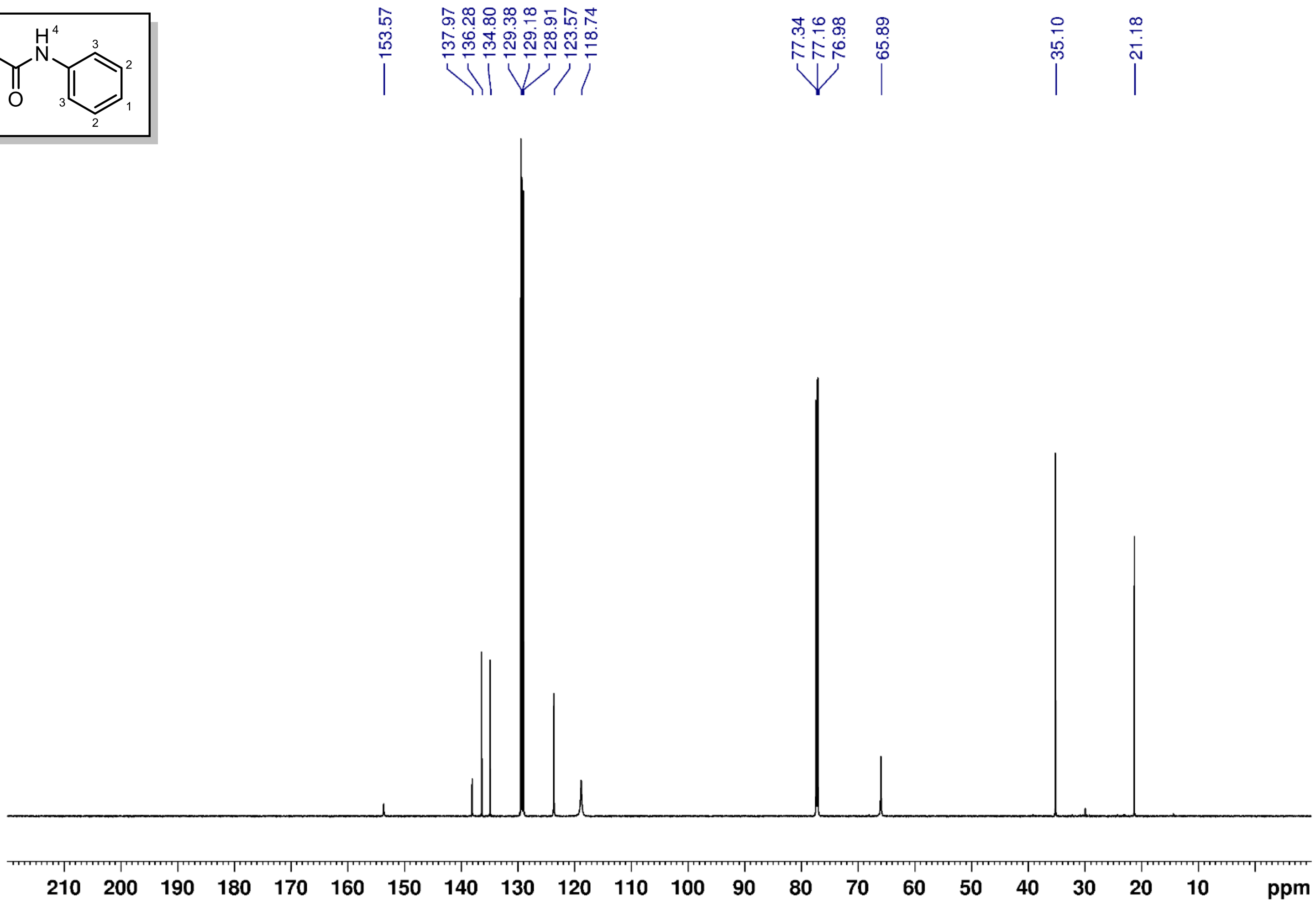

¹H NMR (700 MHz, CDCl₃) for 4-bromophenethyl phenylcarbamate

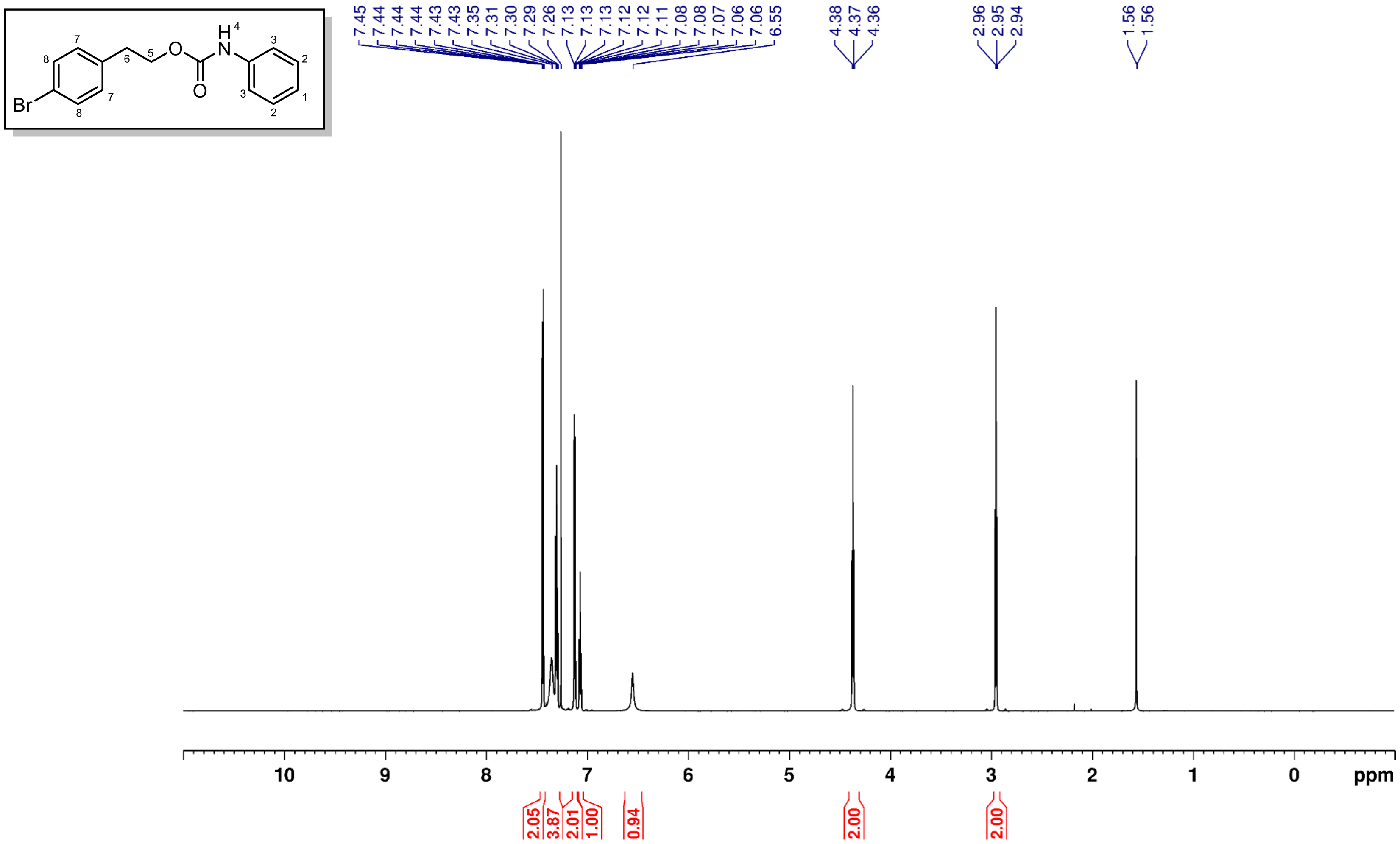

^{13}C NMR (176 MHz, $CDCl_3$) for 4-bromophenethyl phenylcarbamate

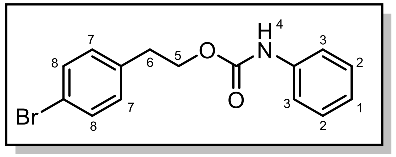

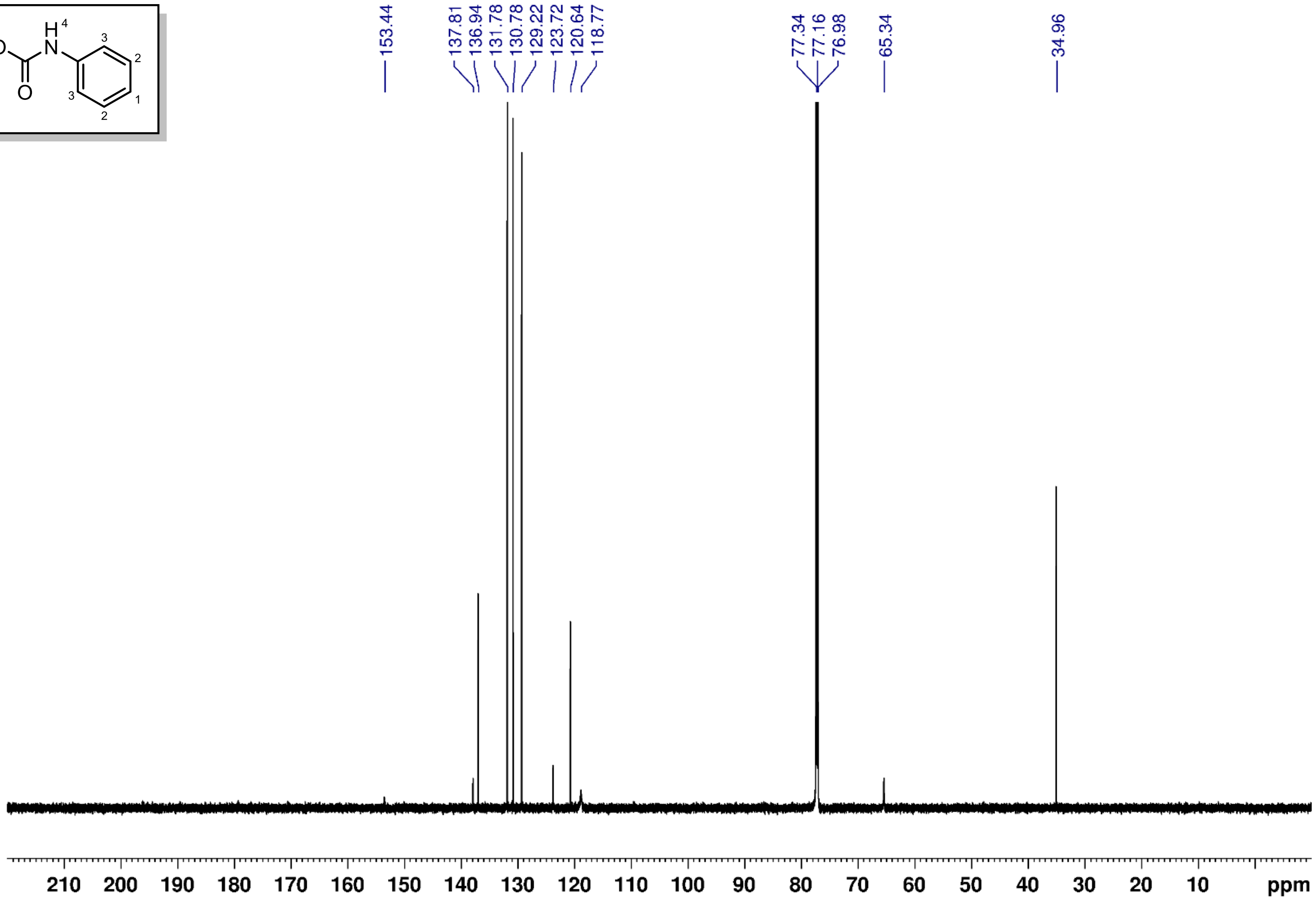

^{1}H NMR (700 MHz, CDCl$_3$) for 4-chlorophenethyl phenylcarbamate

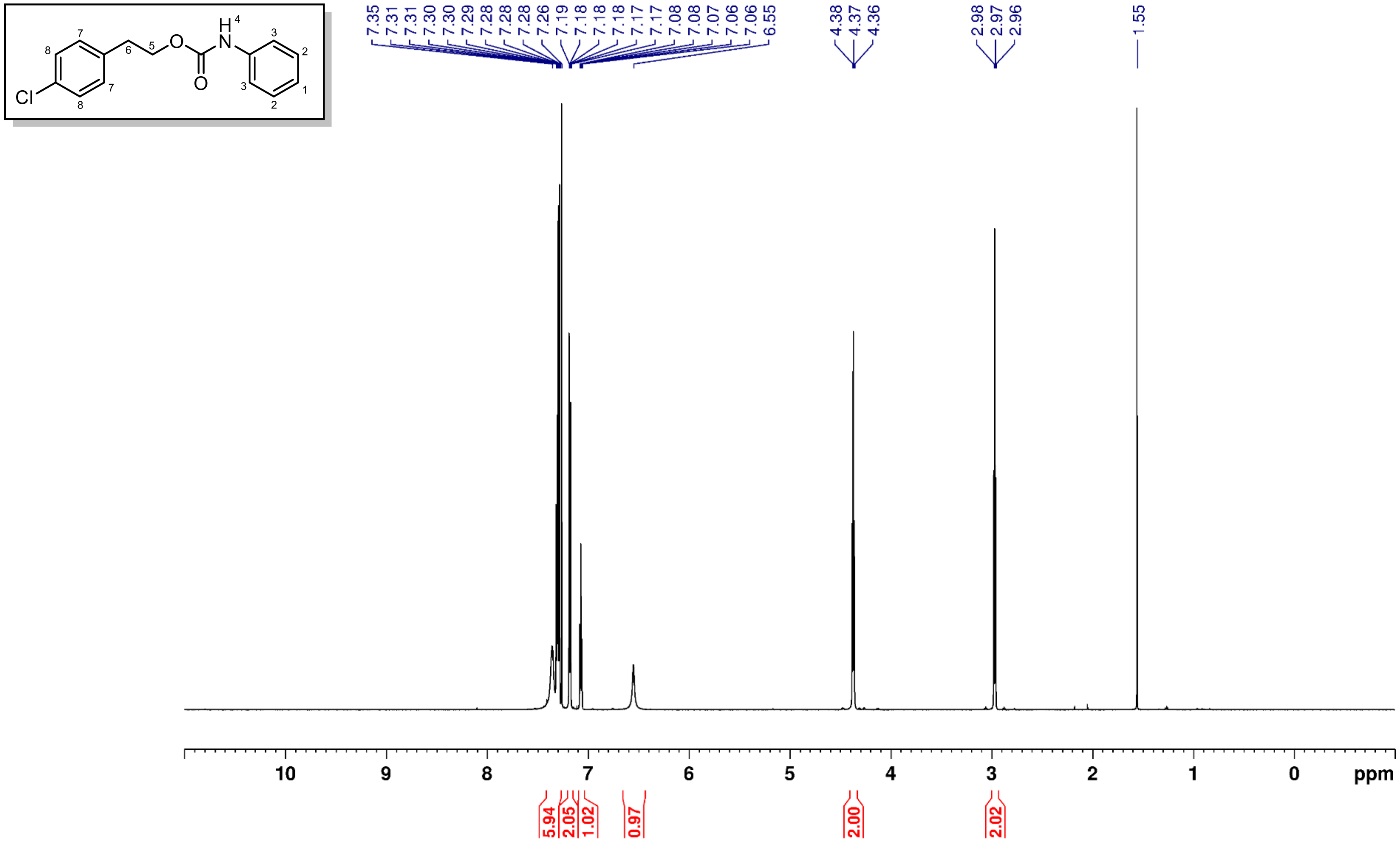

^{13}C NMR (176 MHz, $CDCl_3$) for 4-chlorophenethyl phenylcarbamate

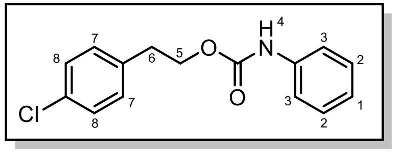

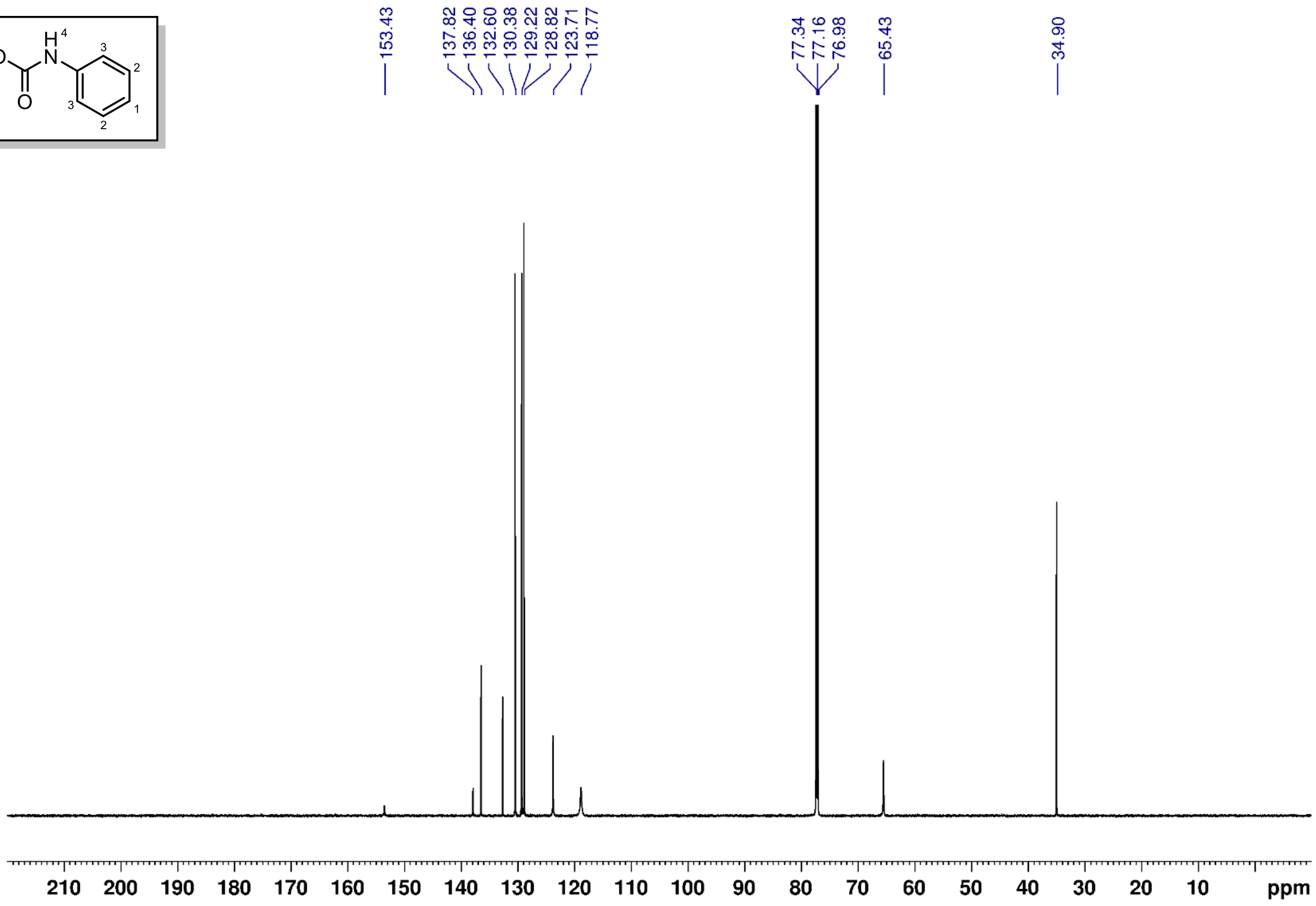

^{1}H NMR (700 MHz, $CDCl_3$) for 4-(trifluoromethyl)phenethyl phenylcarbamate

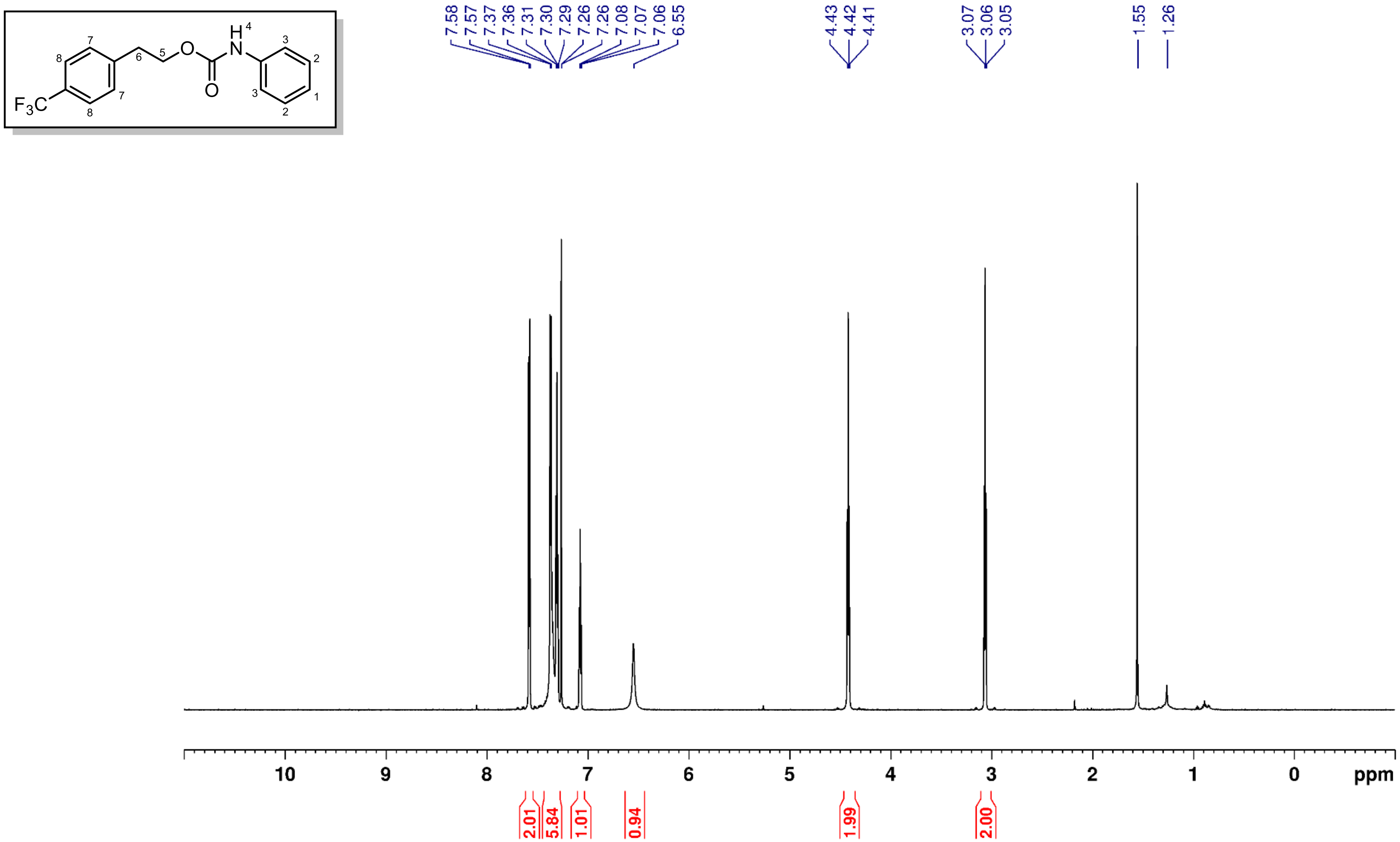

^{13}C NMR (176 MHz, CDCl$_3$) for 4-(trifluoromethyl)phenethyl phenylcarbamate

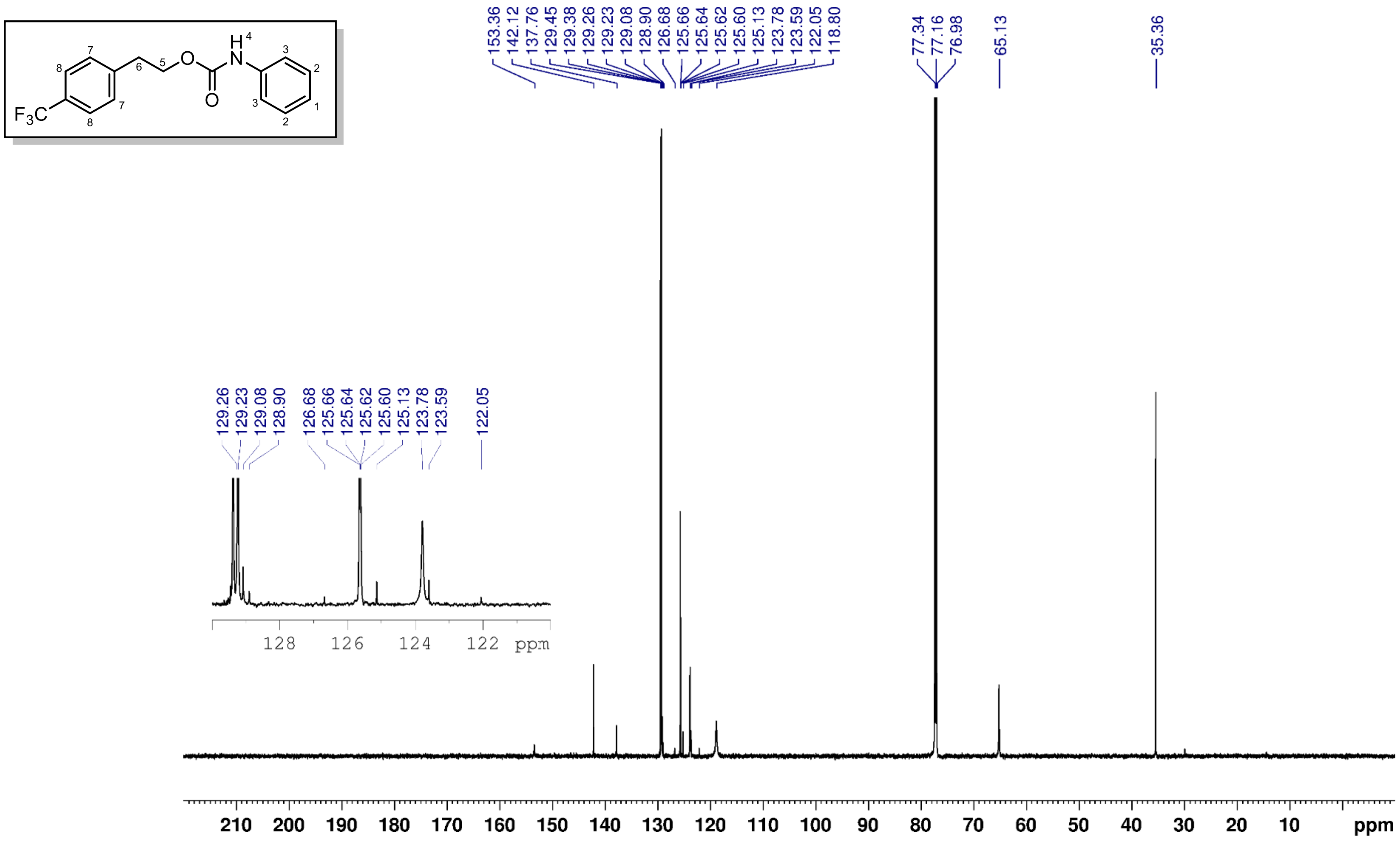

^{19}F NMR (376 MHz, CDCl$_3$) for 4-(trifluoromethyl)phenethyl phenylcarbamate

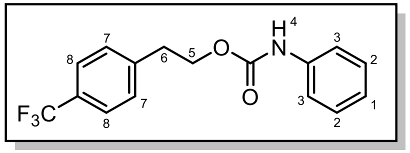

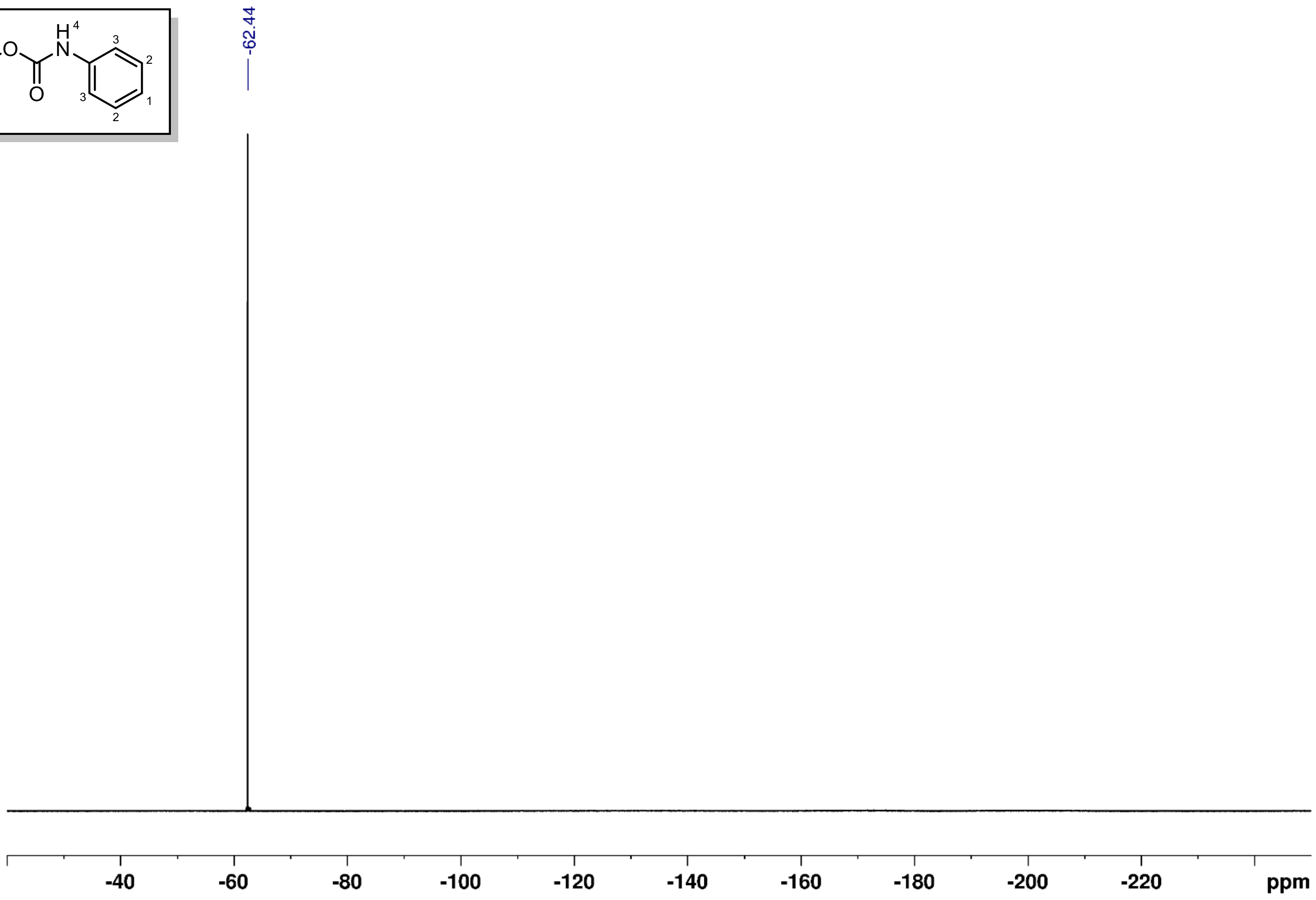

¹H NMR (700 MHz, CDCl₃) for 3-methoxyphenethyl phenylcarbamate

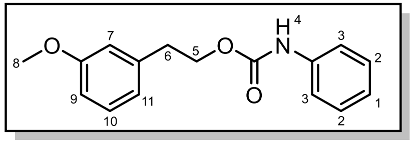

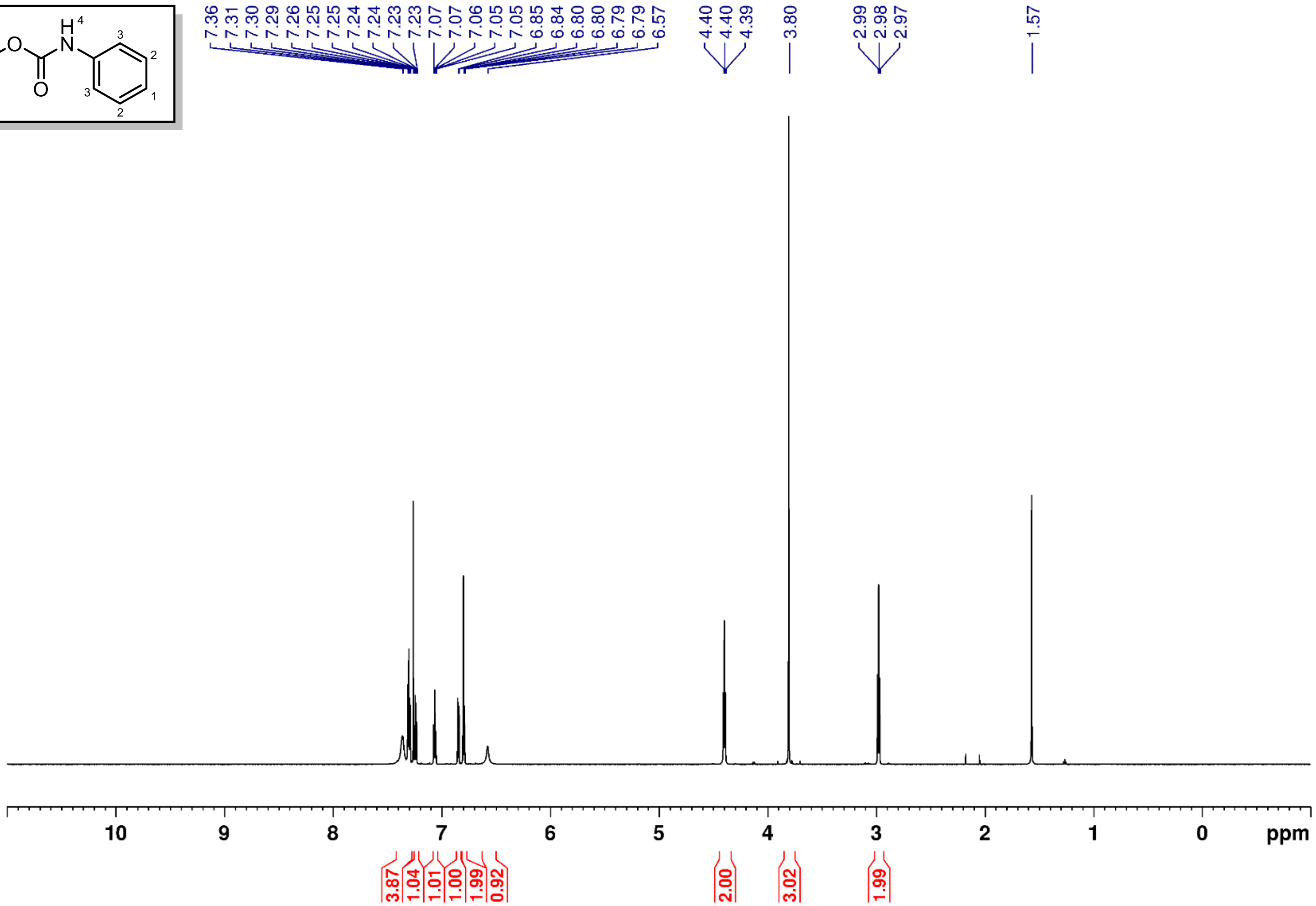

^{13}C NMR (176 MHz, CDCl$_3$) for 3-methoxyphenethyl phenylcarbamate

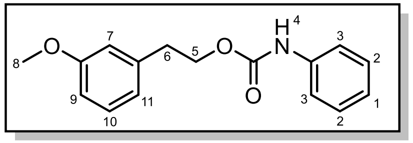

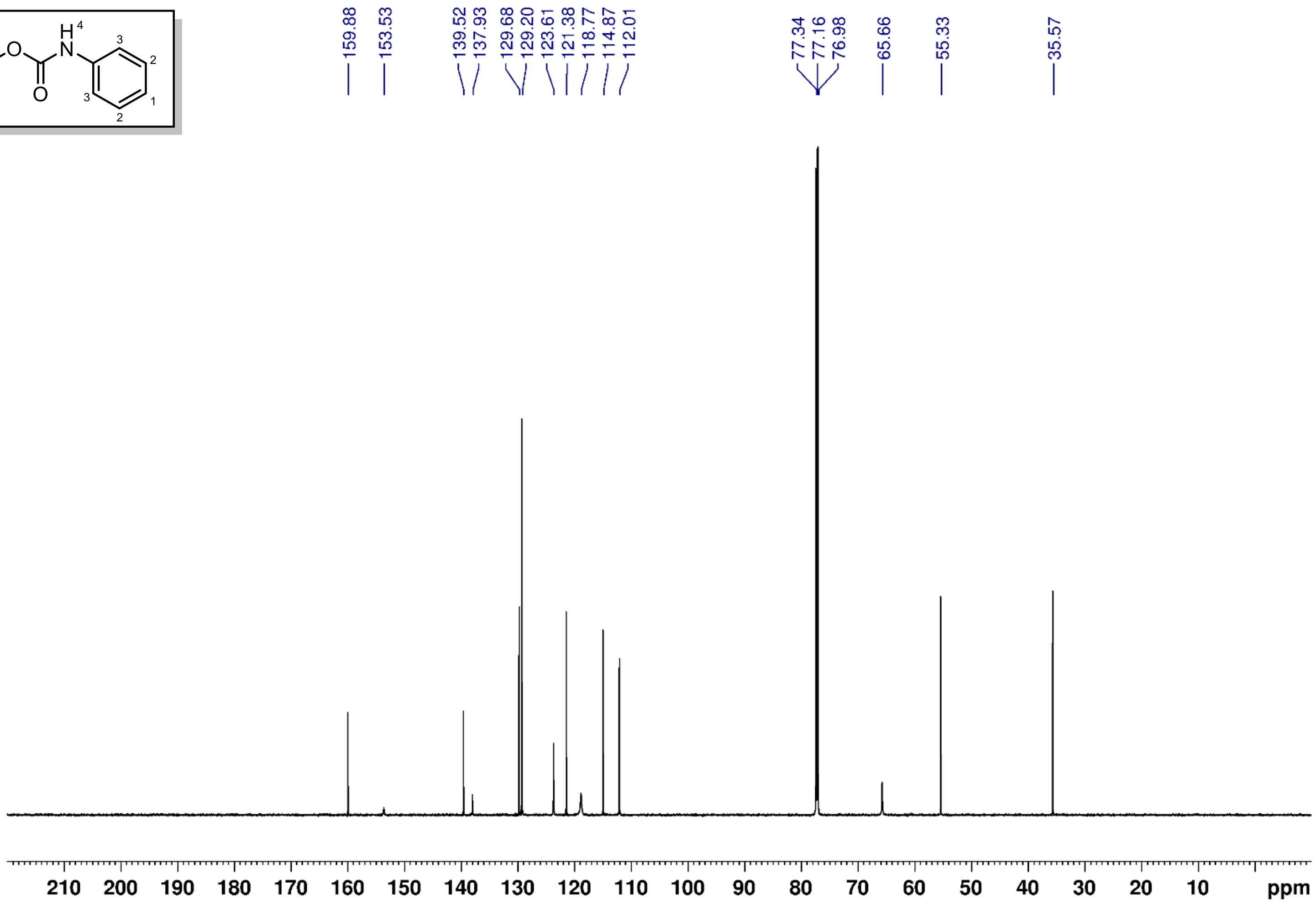

^{1}H NMR (700 MHz, $CDCl_3$) for 3-methylphenethyl phenylcarbamate

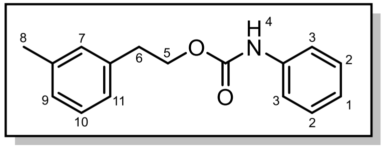

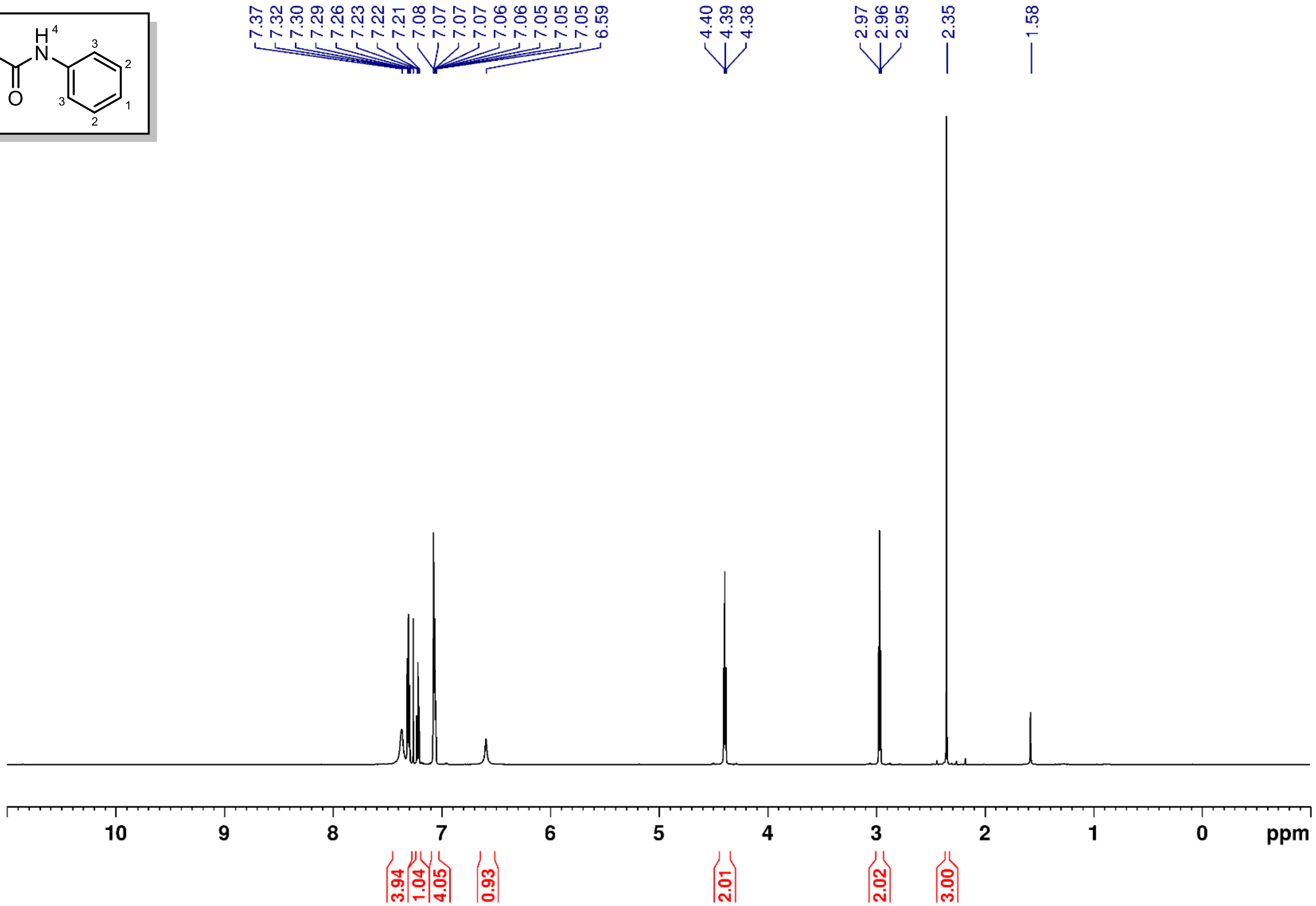

^{13}C NMR (176 MHz, CDCl$_3$) for 3-methylphenethyl phenylcarbamate

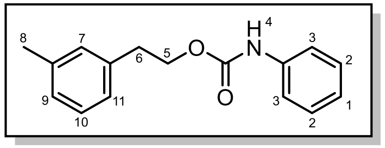

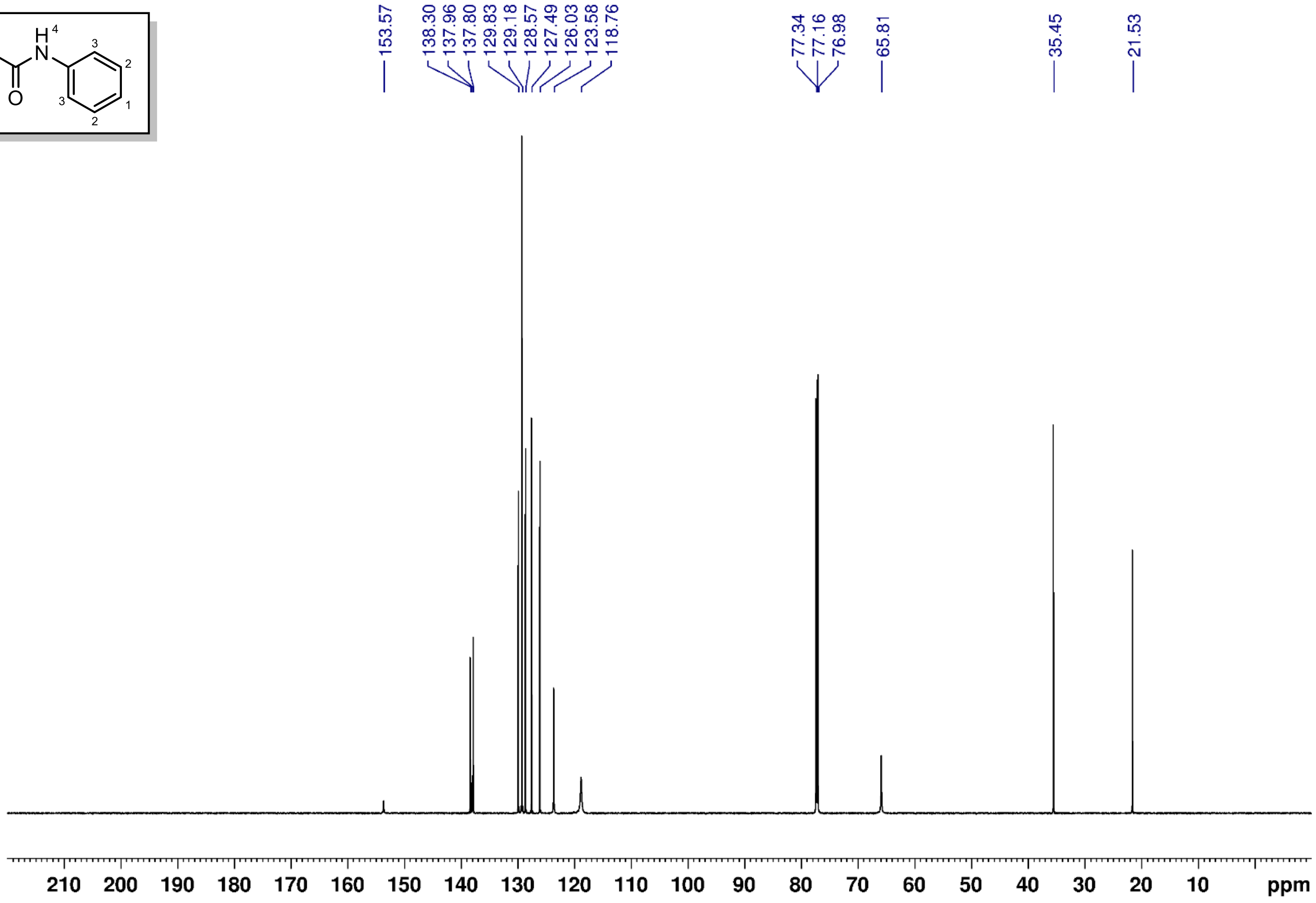

^{1}H NMR (700 MHz, CDCl$_3$) for 3-chlorophenethyl phenylcarbamate

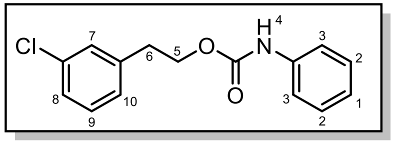

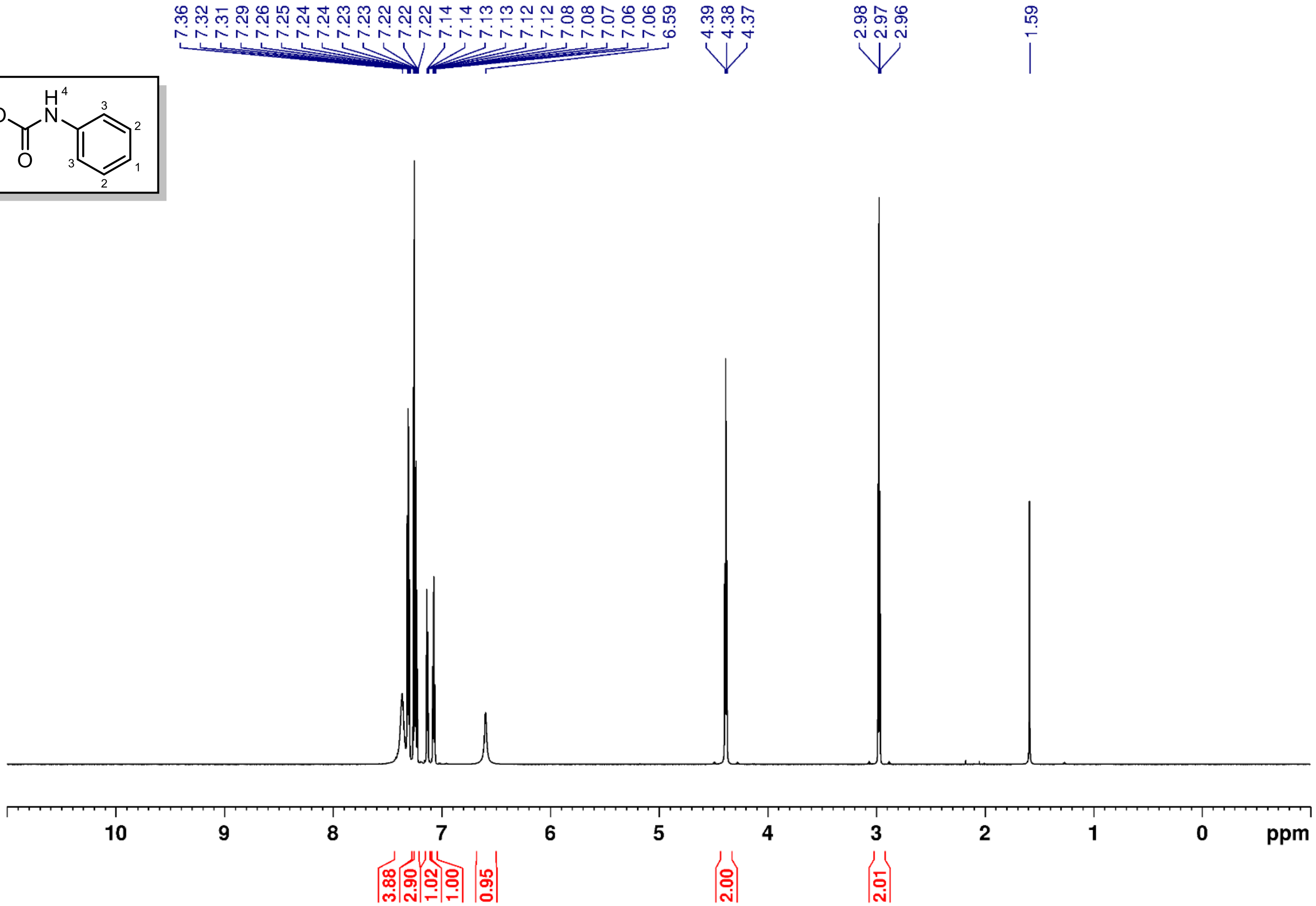

^{13}C NMR (176 MHz, $CDCl_3$) for 3-chlorophenethyl phenylcarbamate

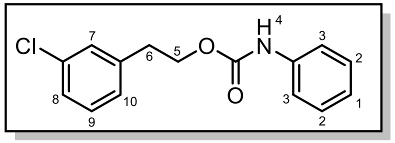

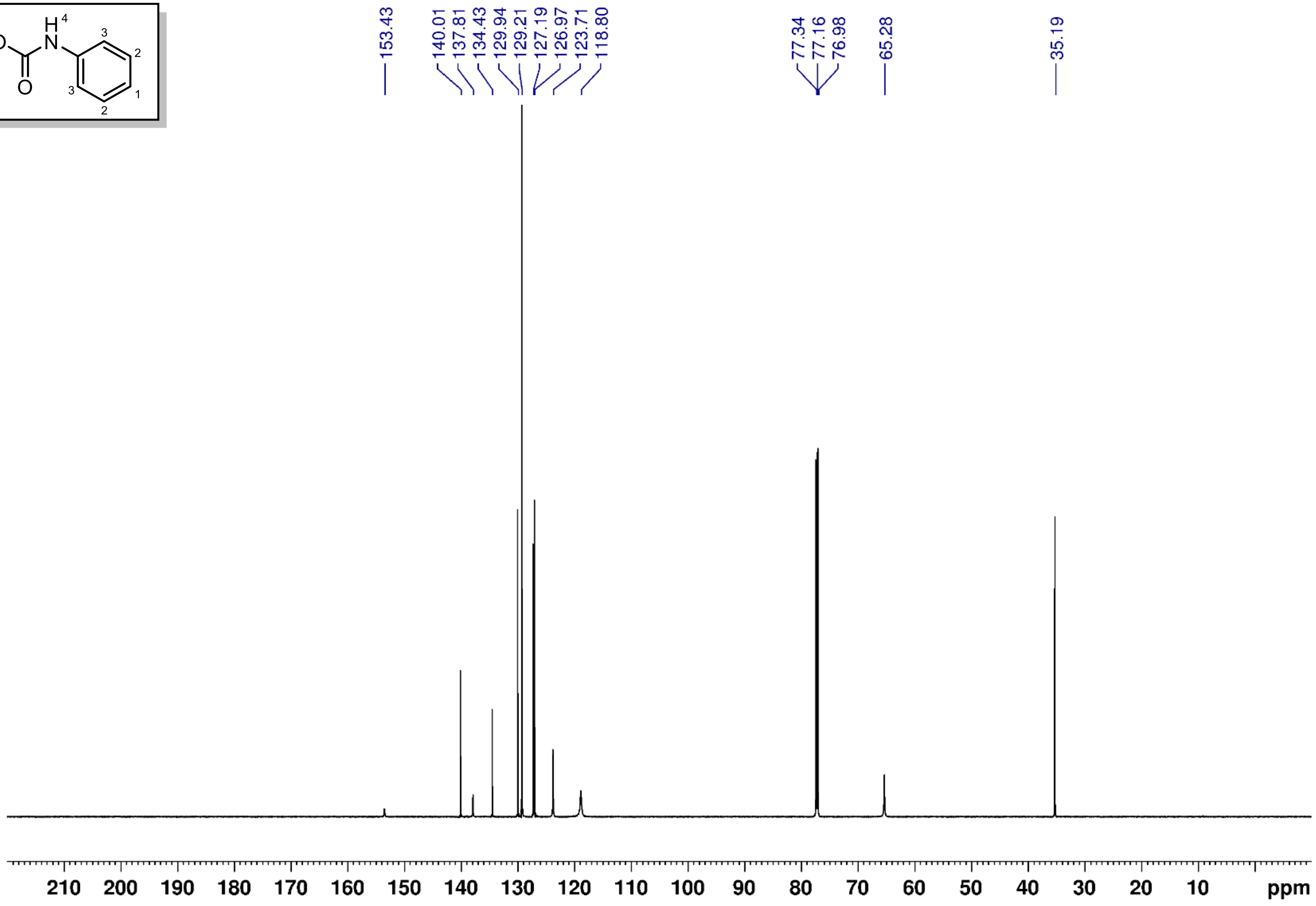

^{1}H NMR (700 MHz, CDCl$_3$) for 3-bromophenethyl phenylcarbamate

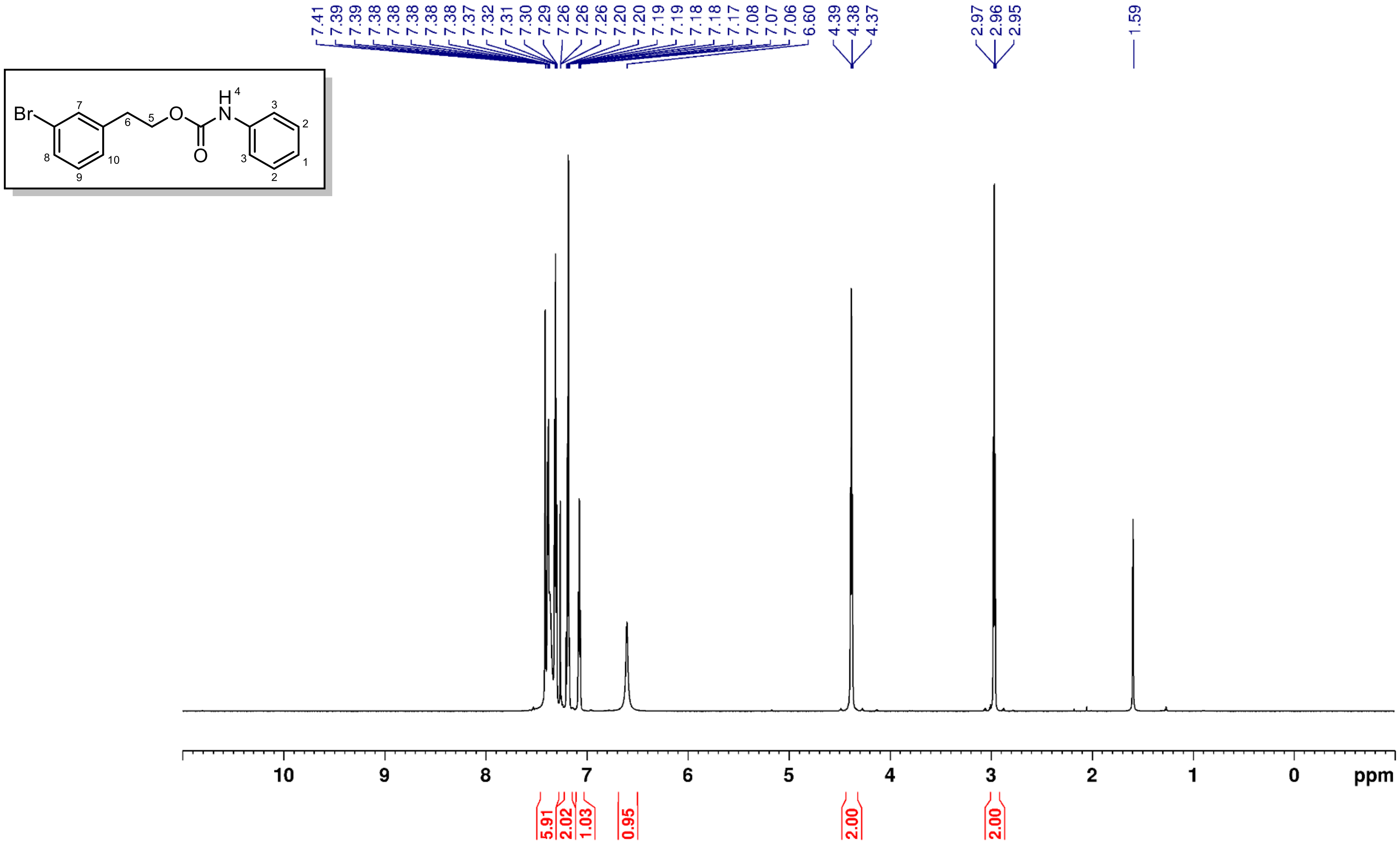

^{13}C NMR (176 MHz, $CDCl_3$) for 3-bromophenethyl phenylcarbamate

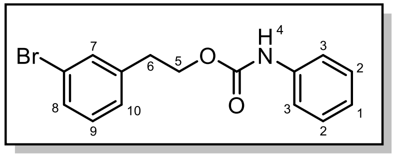

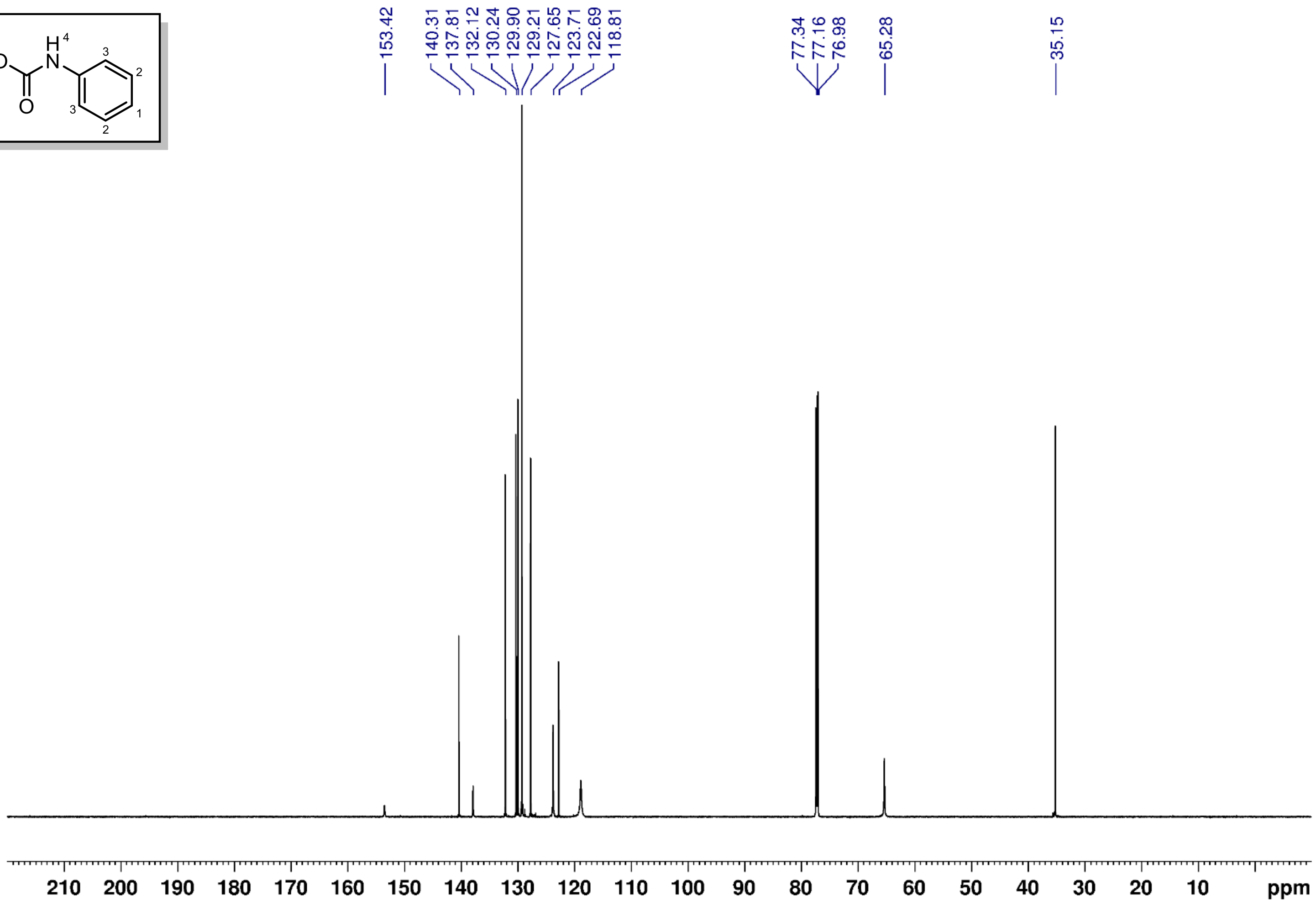

^{1}H NMR (700 MHz, $CDCl_3$) for 3-(trifluoromethyl)phenethyl phenylcarbamate

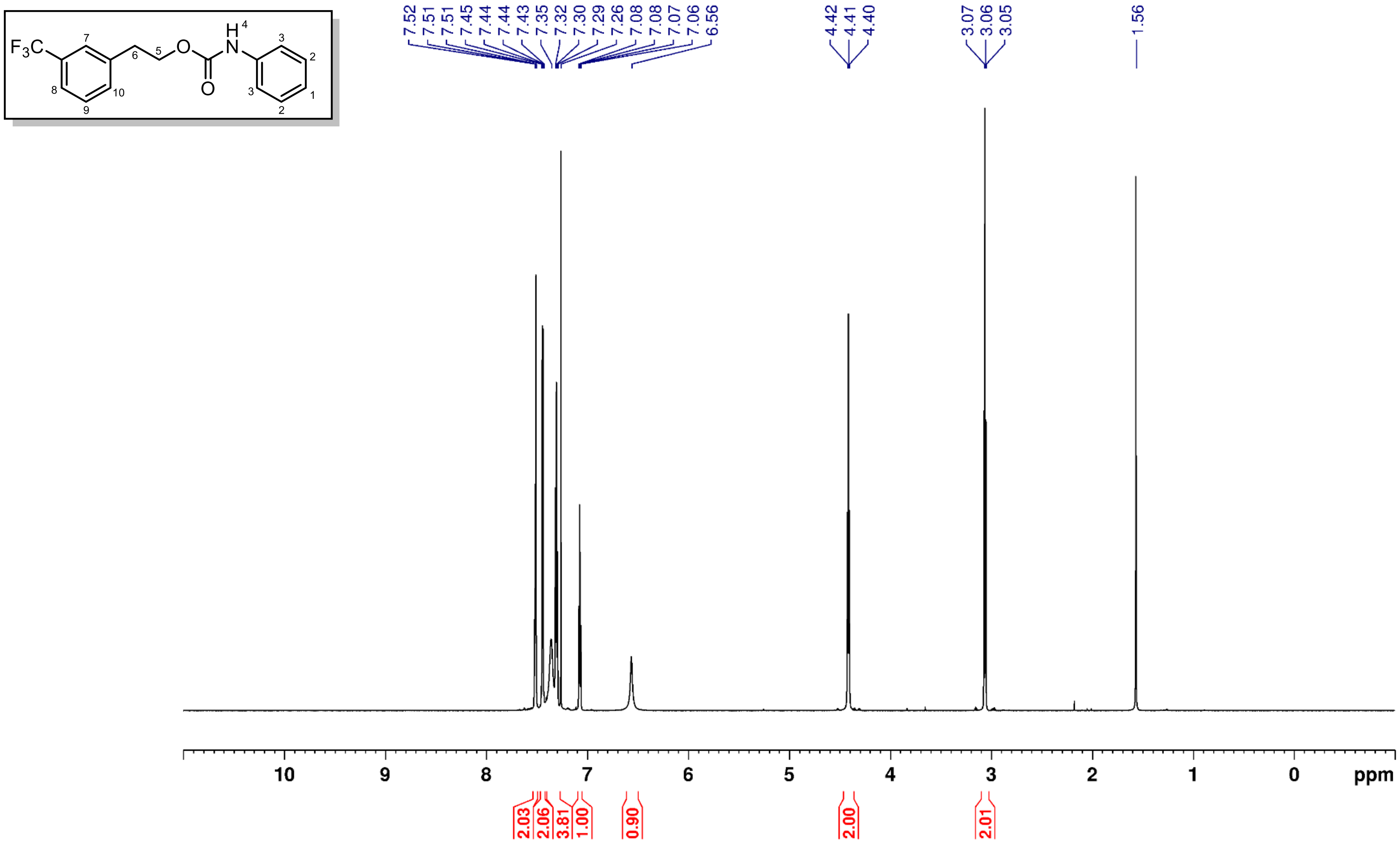

^{13}C NMR (176 MHz, $CDCl_3$) for 3-(trifluoromethyl)phenethyl phenylcarbamate

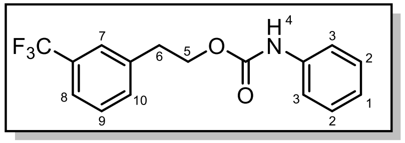

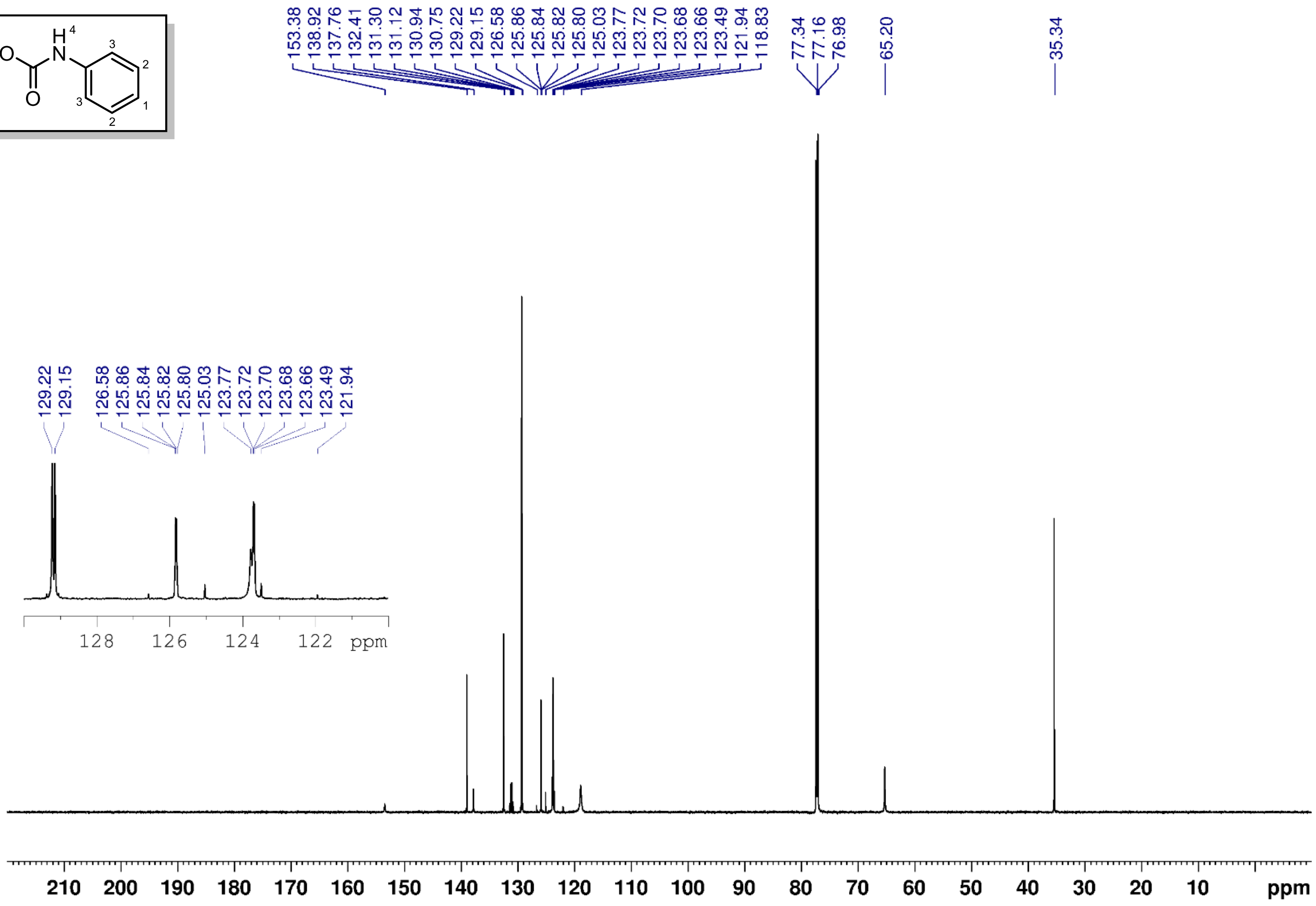

^{19}F NMR (376 MHz, CDCl$_3$) for 3-(trifluoromethyl)phenethyl phenylcarbamate

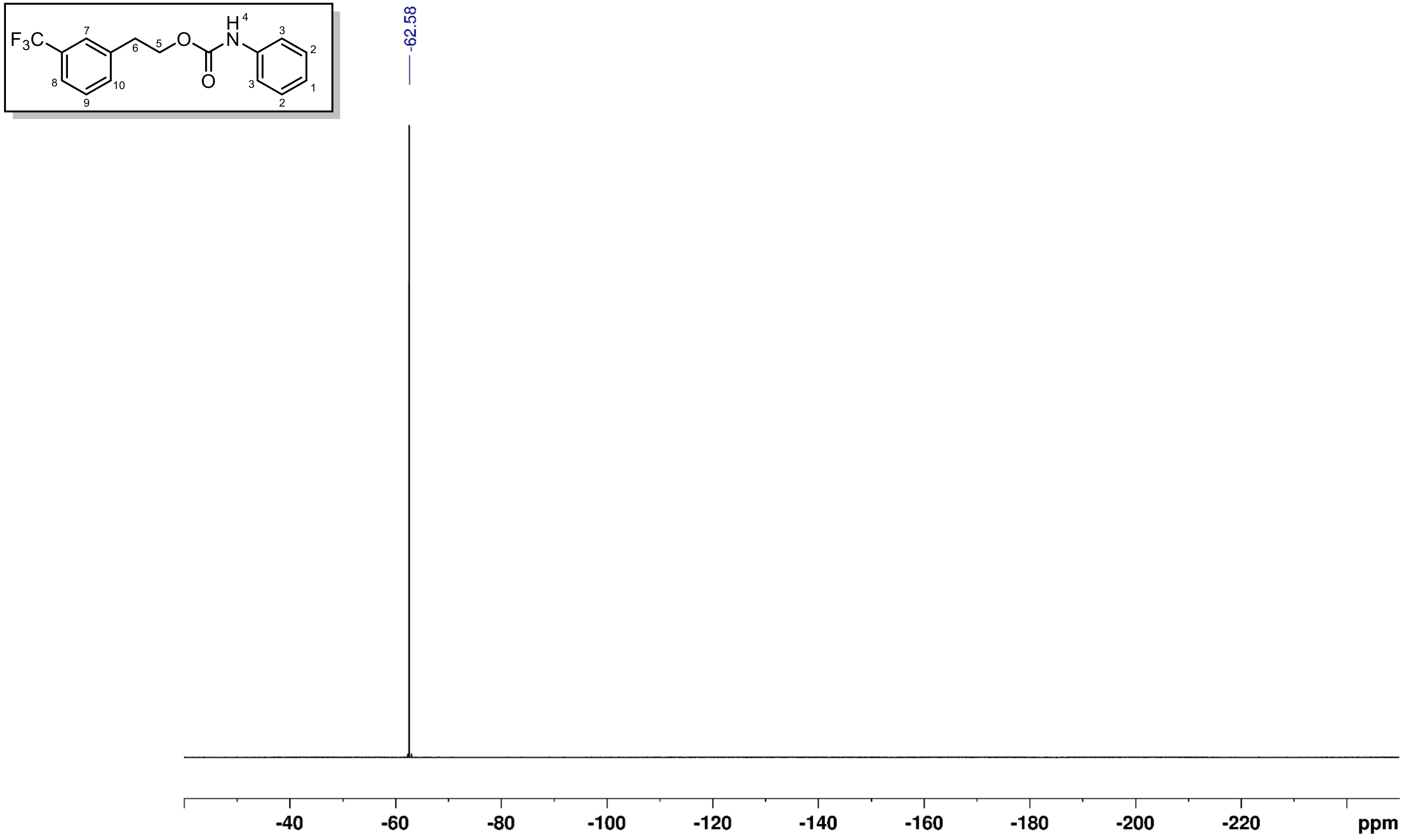

^{1}H NMR (700 MHz, CDCl$_3$) for 2-methylphenethyl phenylcarbamate

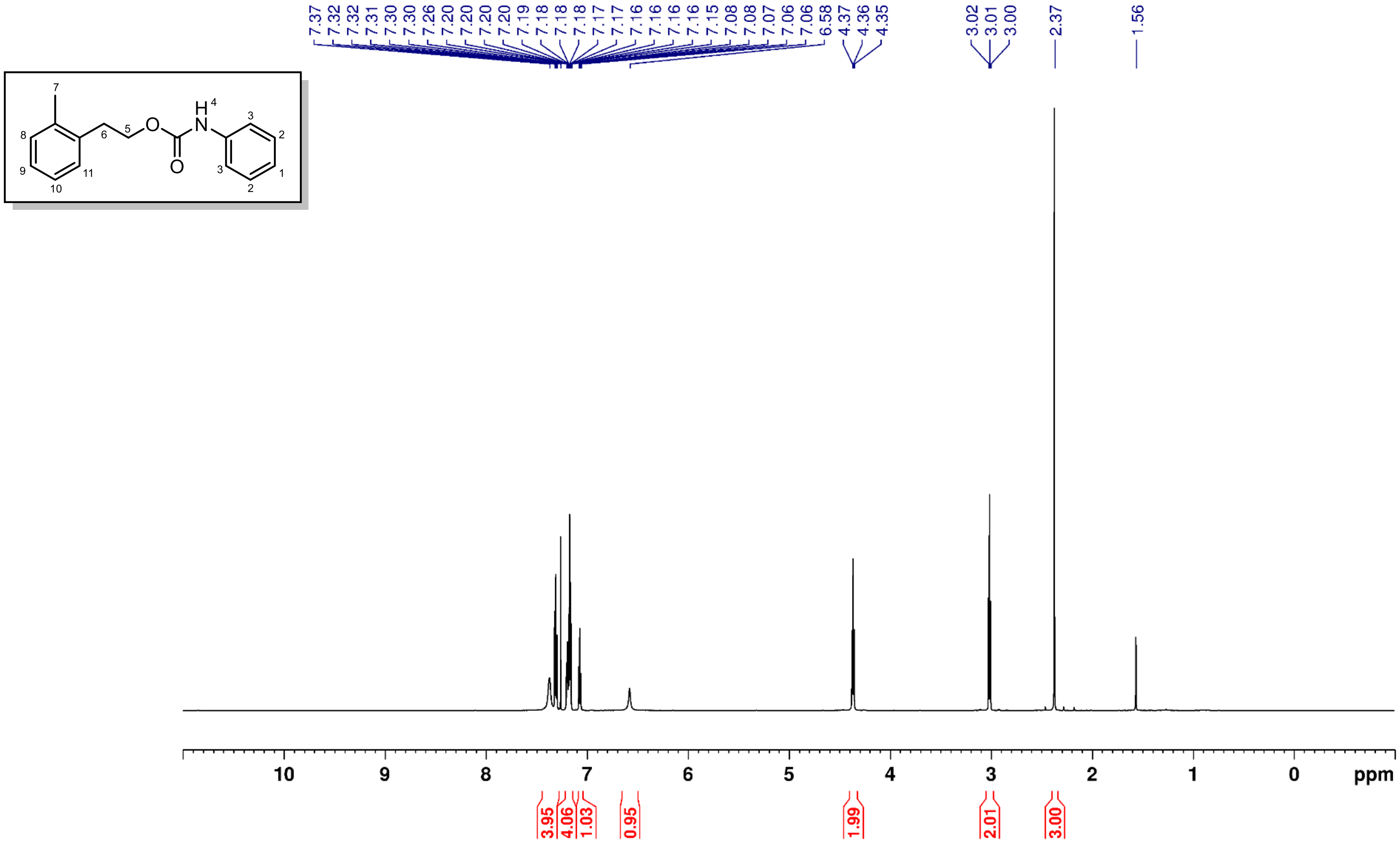

^{13}**C NMR (176 MHz, CDCl$_3$)** for 2-methylphenethyl phenylcarbamate

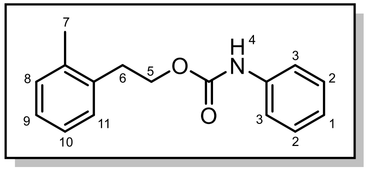

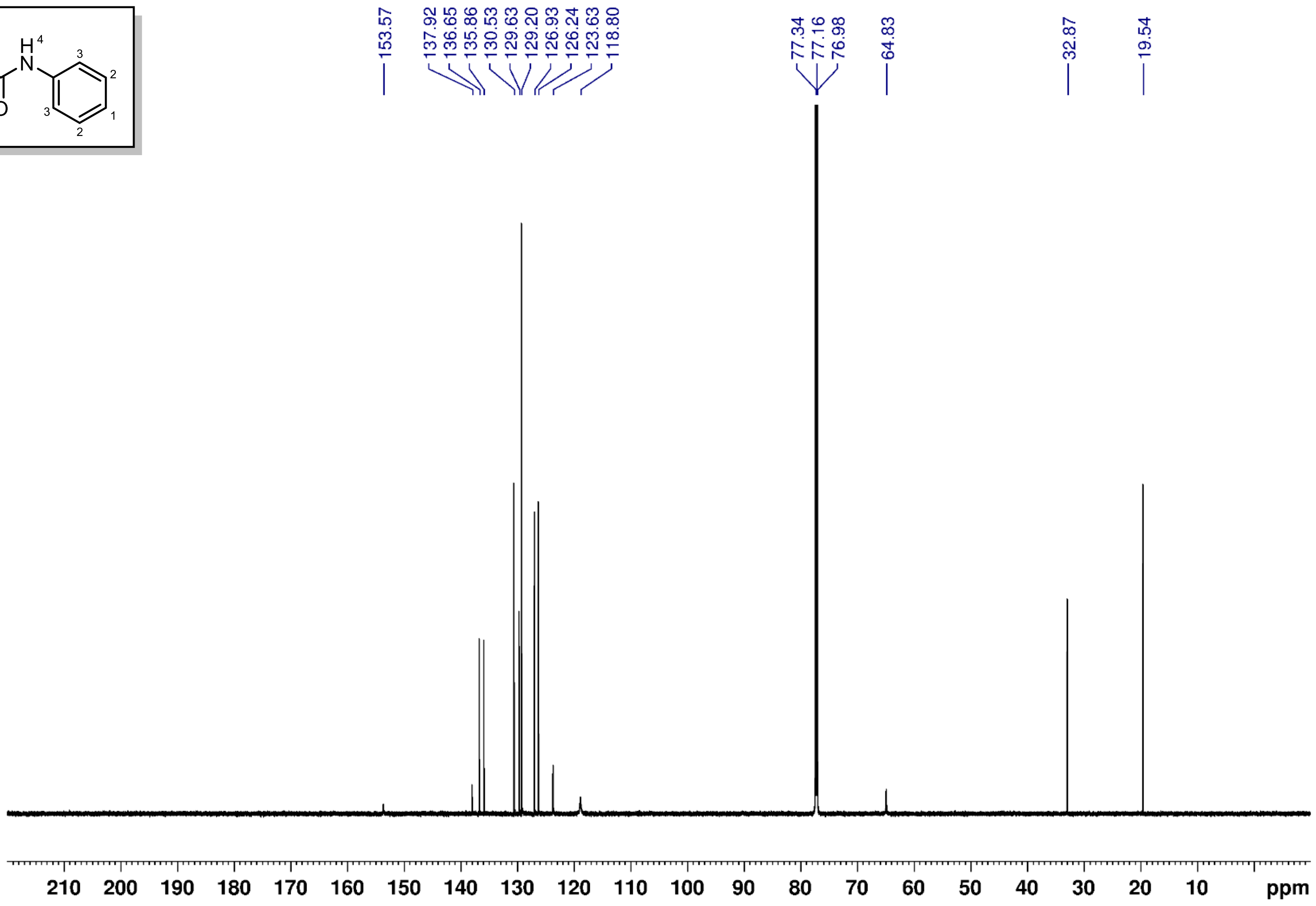

^{1}H NMR (700 MHz, CDCl$_3$) for 2-chlorophenethyl phenylcarbamate

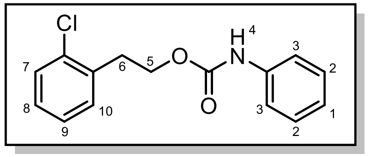

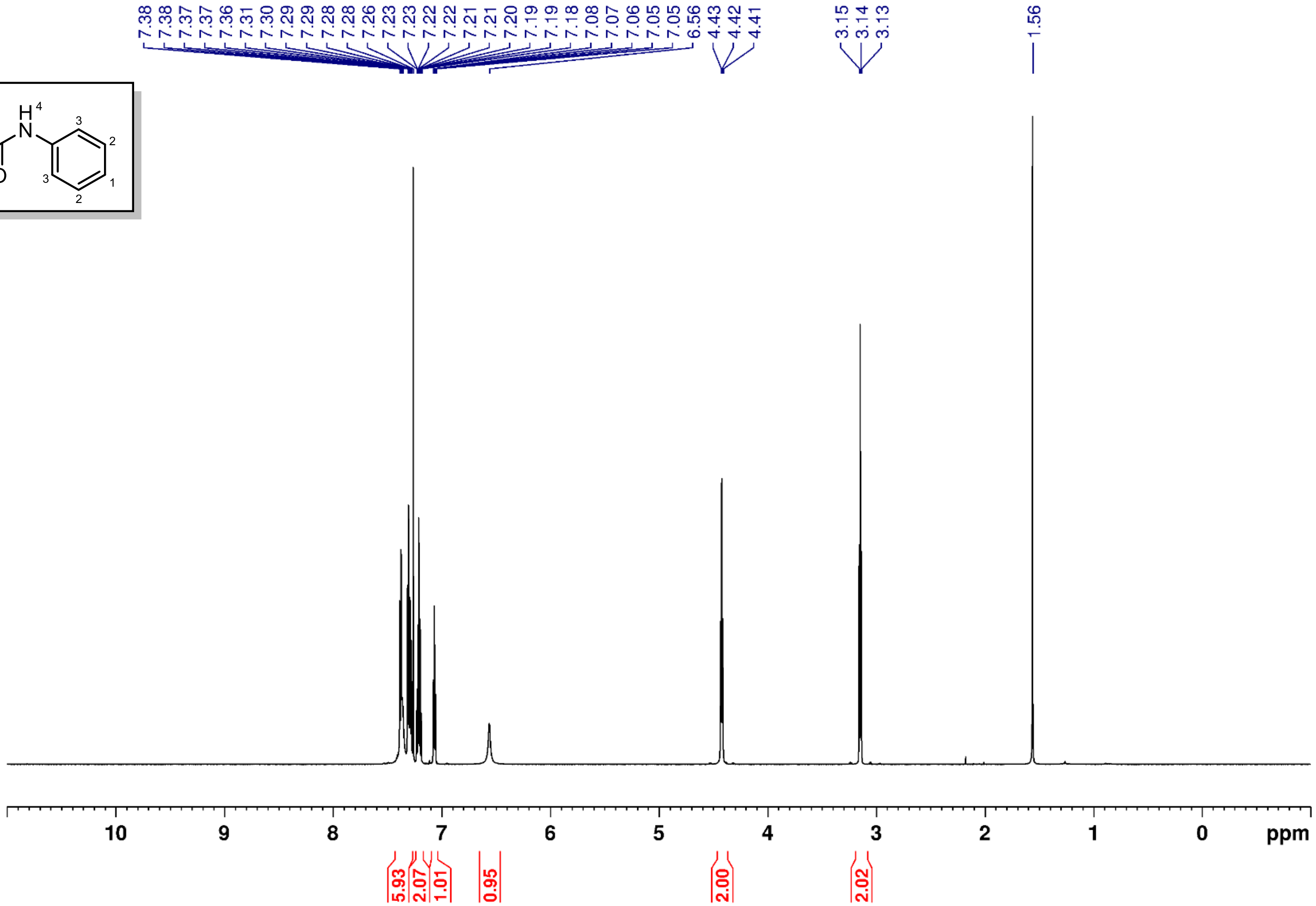

^{13}C NMR (176 MHz, CDCl$_3$) for 2-chlorophenethyl phenylcarbamate

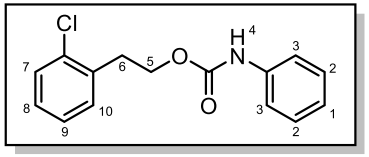

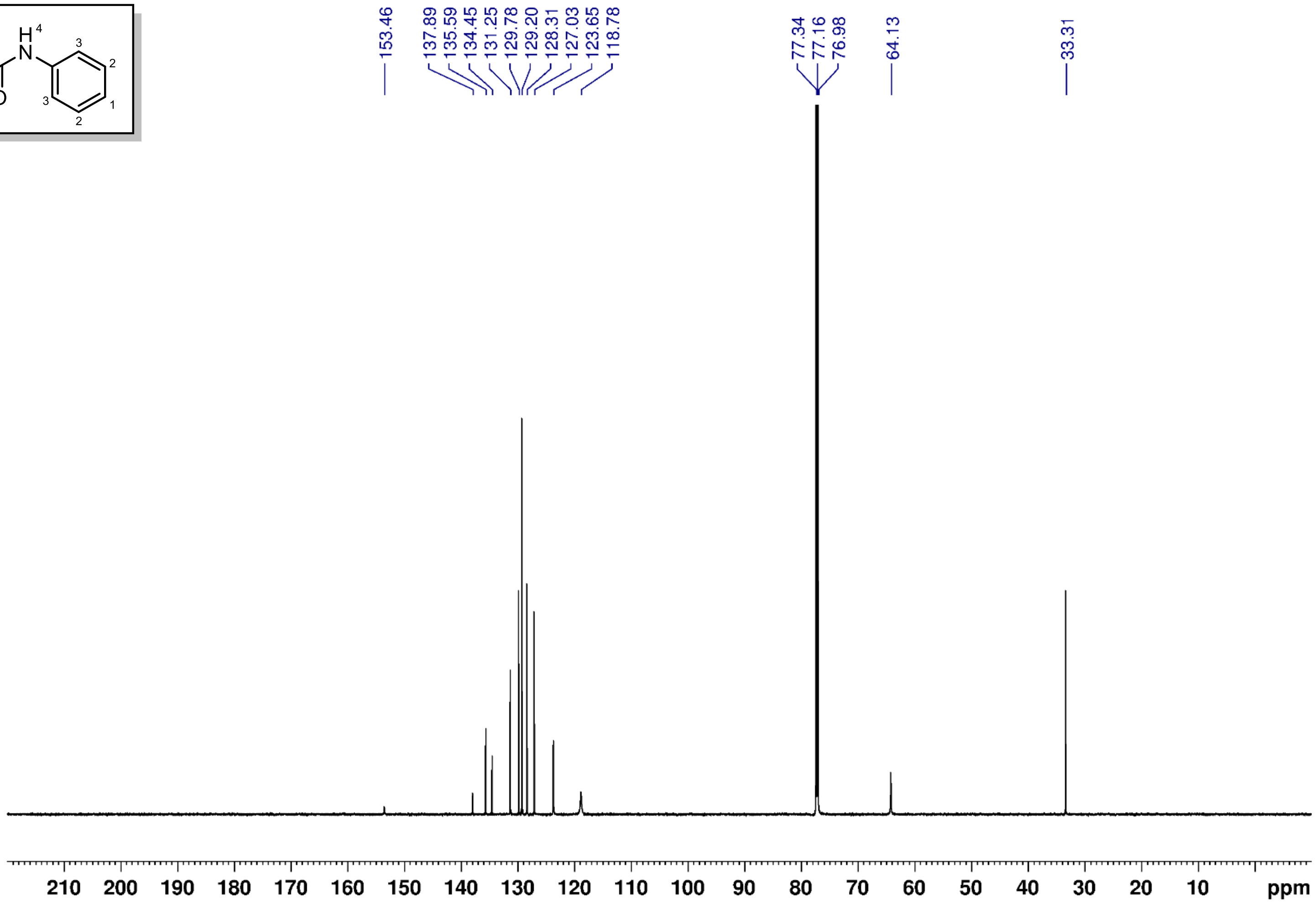

^{1}H NMR (700 MHz, CDCl$_3$) for 2-bromophenethyl phenylcarbamate

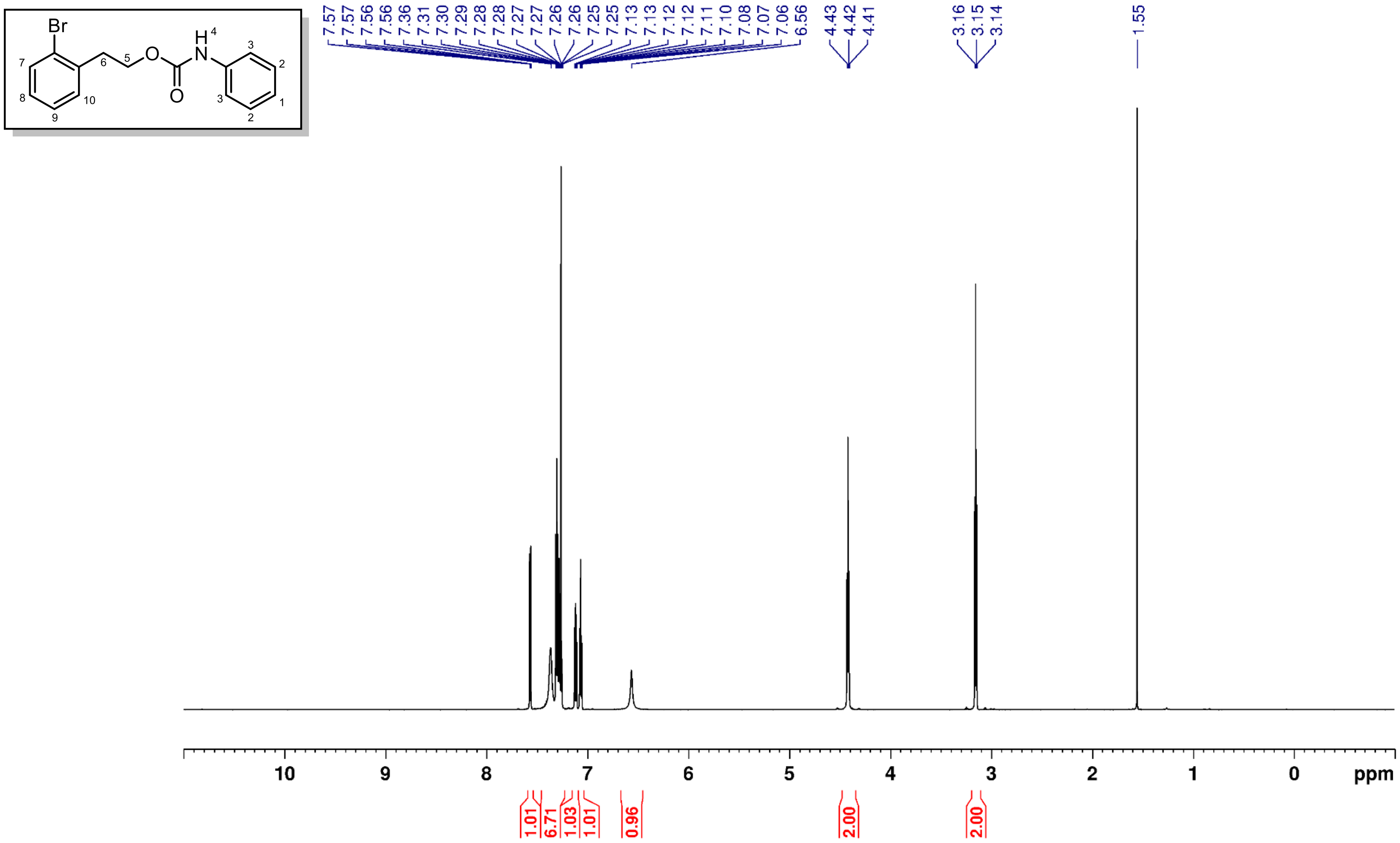

^{13}C NMR (176 MHz, $CDCl_3$) for 2-bromophenethyl phenylcarbamate

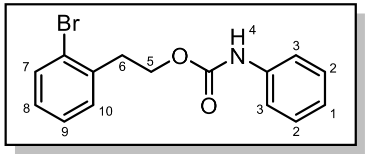

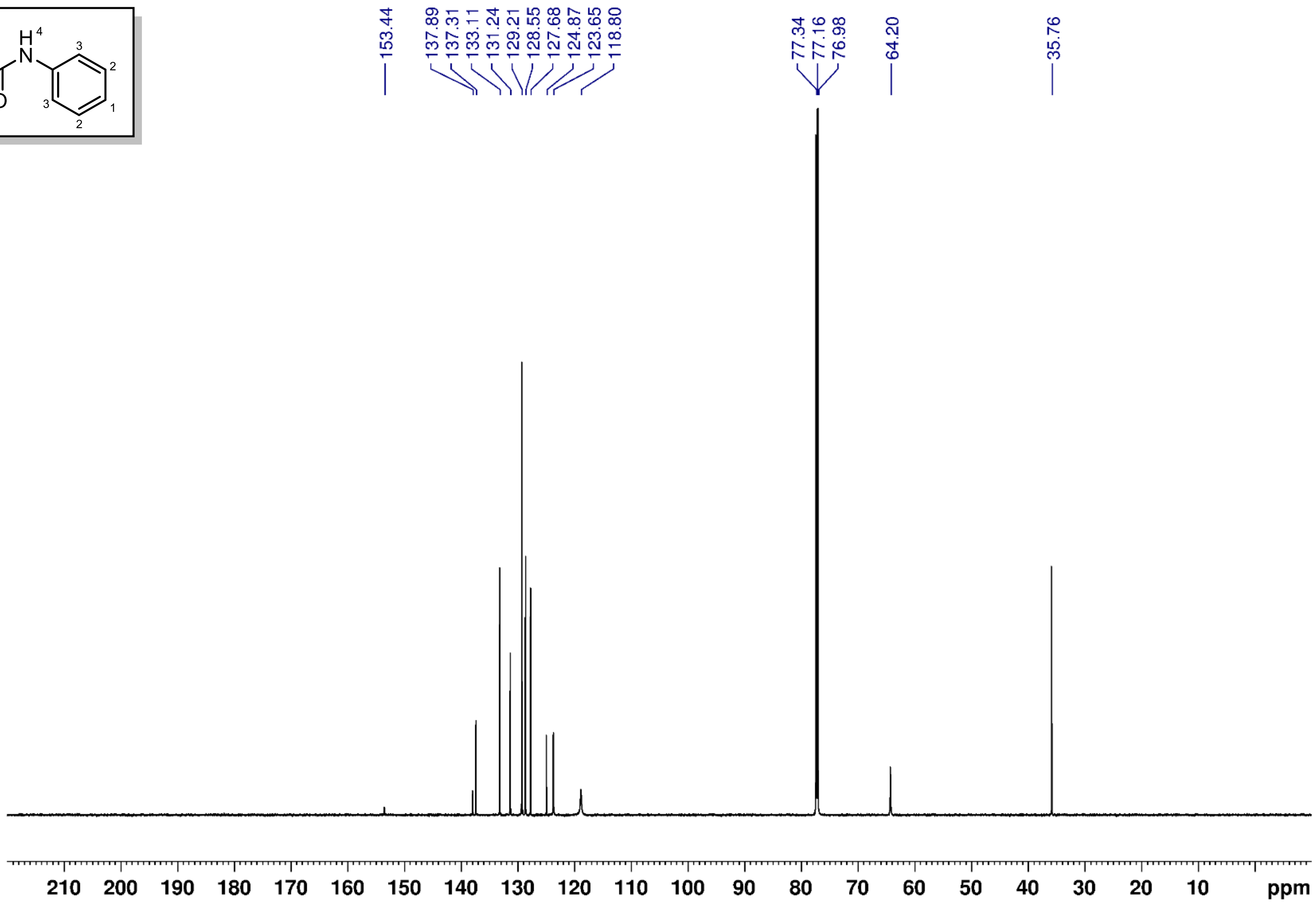

^{1}H NMR (700 MHz, $CDCl_3$) for 2-(thiophen-2-yl)ethyl phenylcarbamate

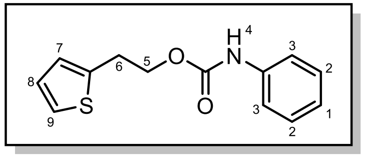

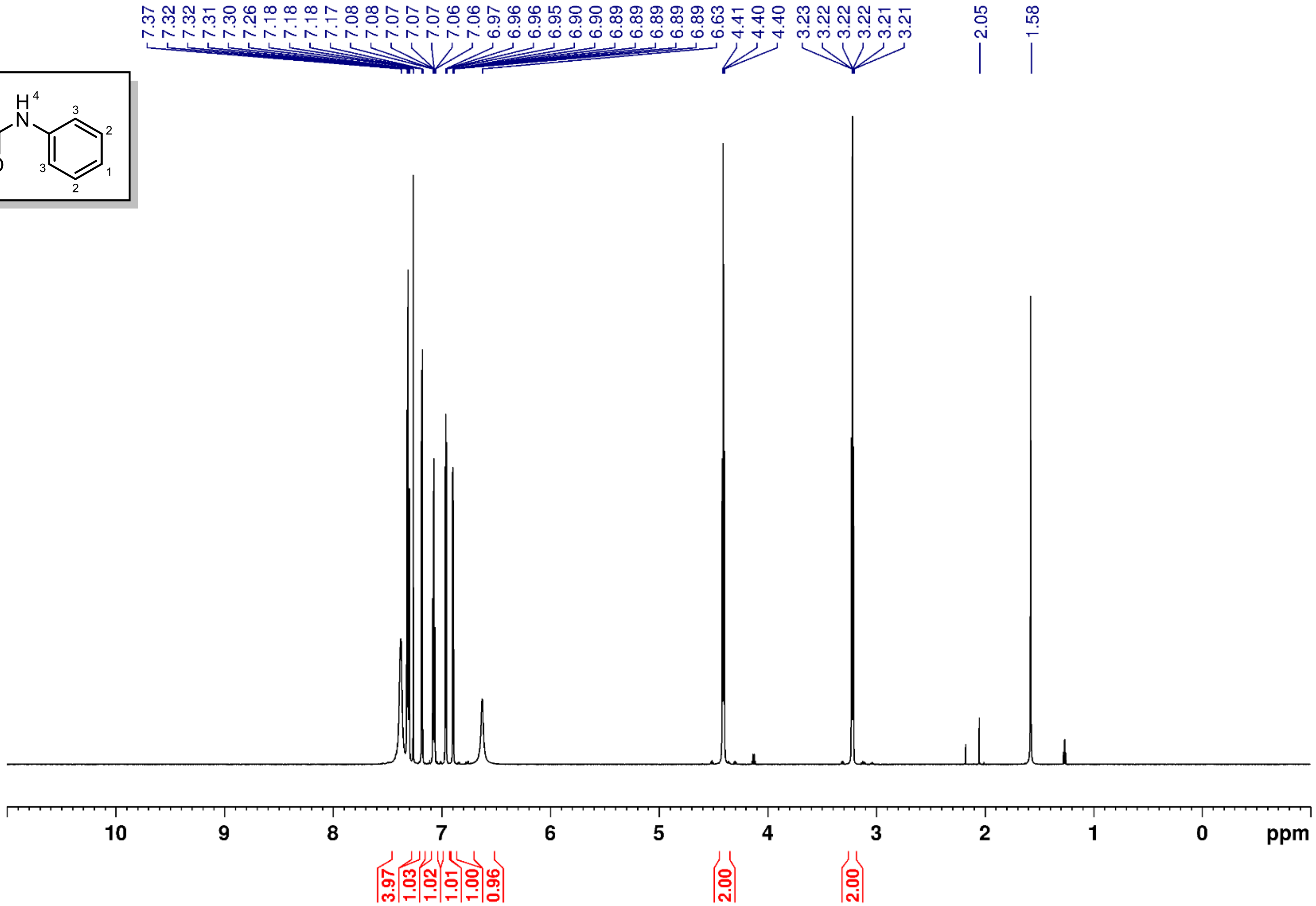

^{13}C NMR (176 MHz, CDCl$_3$) for 2-(thiophen-2-yl)ethyl phenylcarbamate

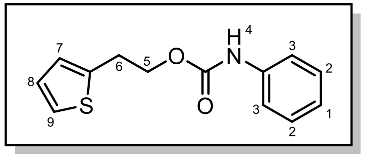

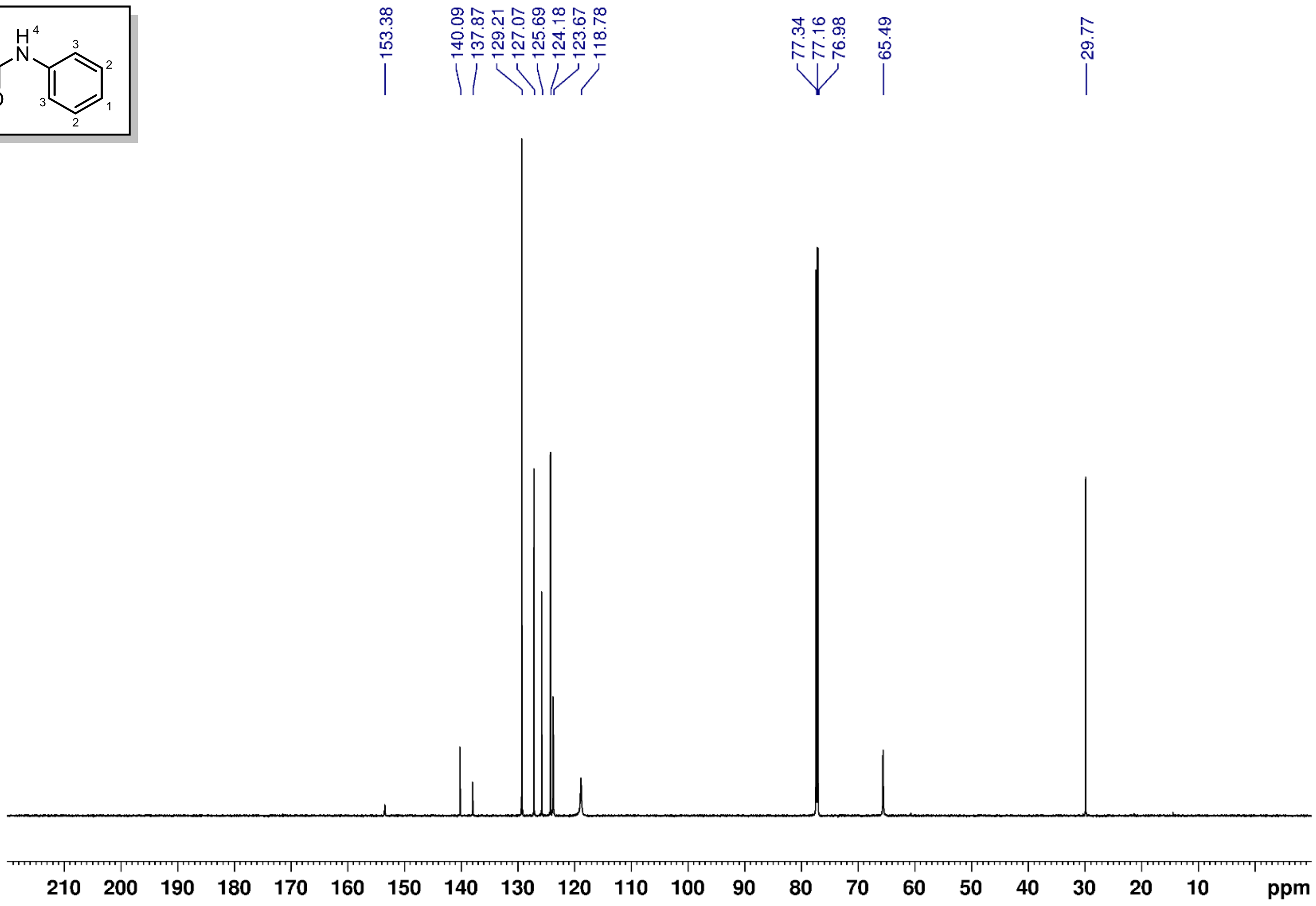

^{1}H NMR (700 MHz, $CDCl_3$) for 2-(thiophen-3-yl)ethyl phenylcarbamate

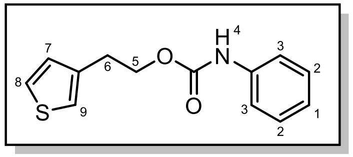

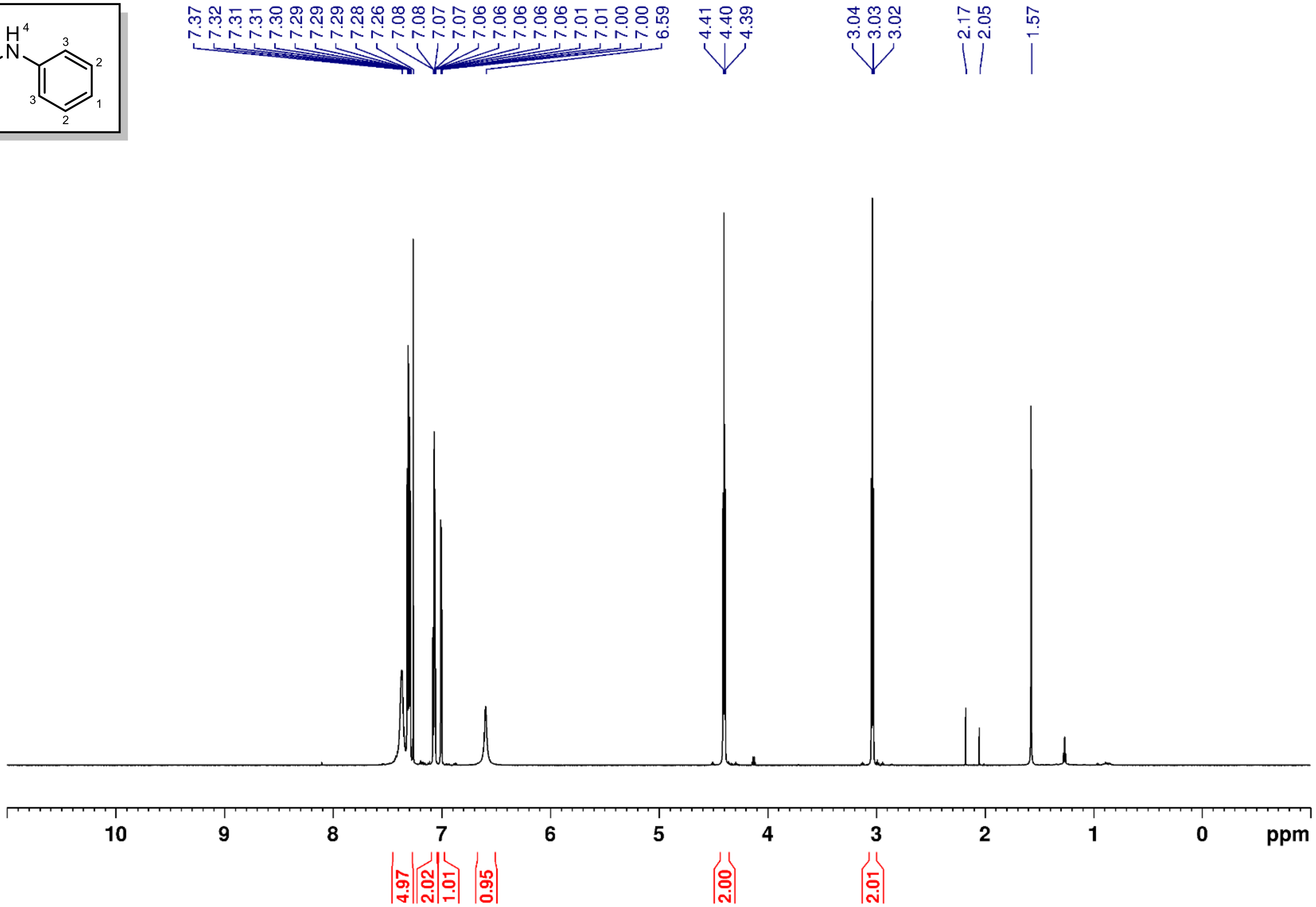

^{13}**C NMR (176 MHz, CDCl$_3$)** for 2-(thiophen-3-yl)ethyl phenylcarbamate

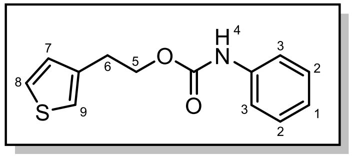

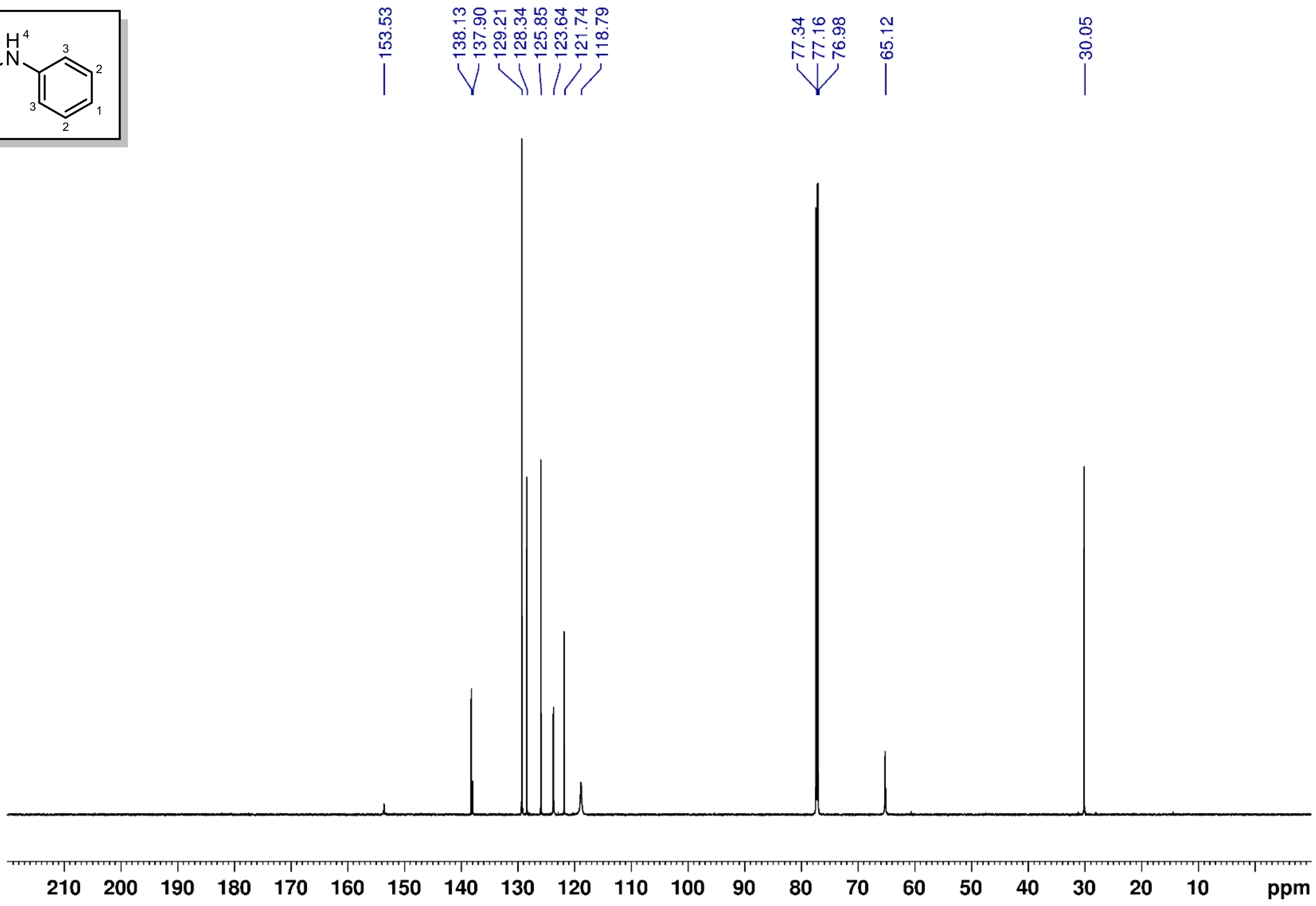

^{1}H NMR (500 MHz, $CDCl_3$) for 2,3-dihydro-1*H*-inden-2-yl phenylcarbamate

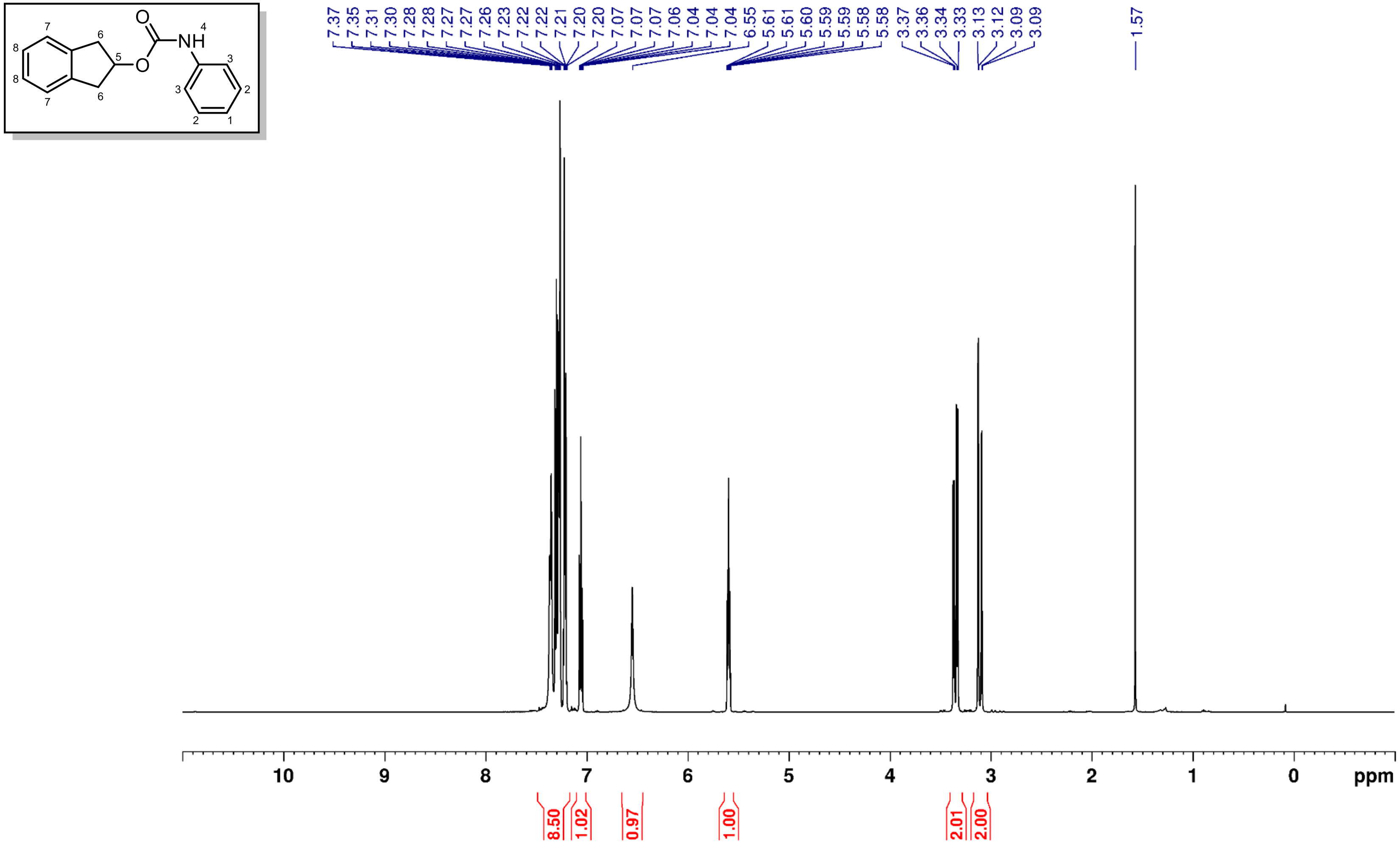

^{13}C NMR (126 MHz, $CDCl_3$) for 2,3-dihydro-1*H*-inden-2-yl phenylcarbamate

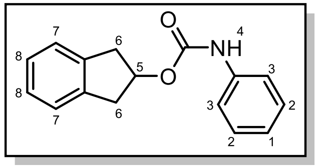

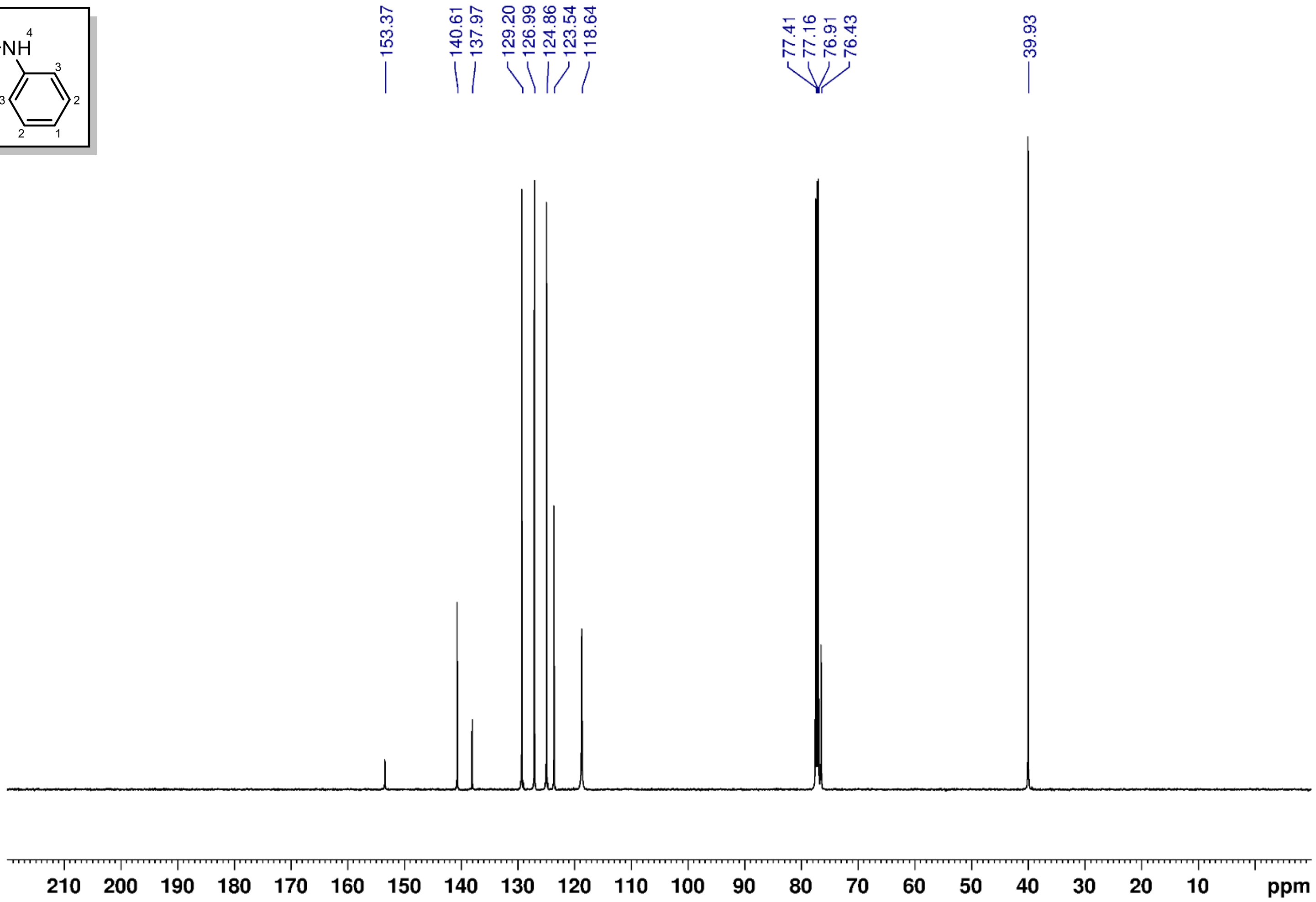

^{1}H NMR (700 MHz, $CDCl_3$) for 1-phenylpropan-2-yl phenylcarbamate

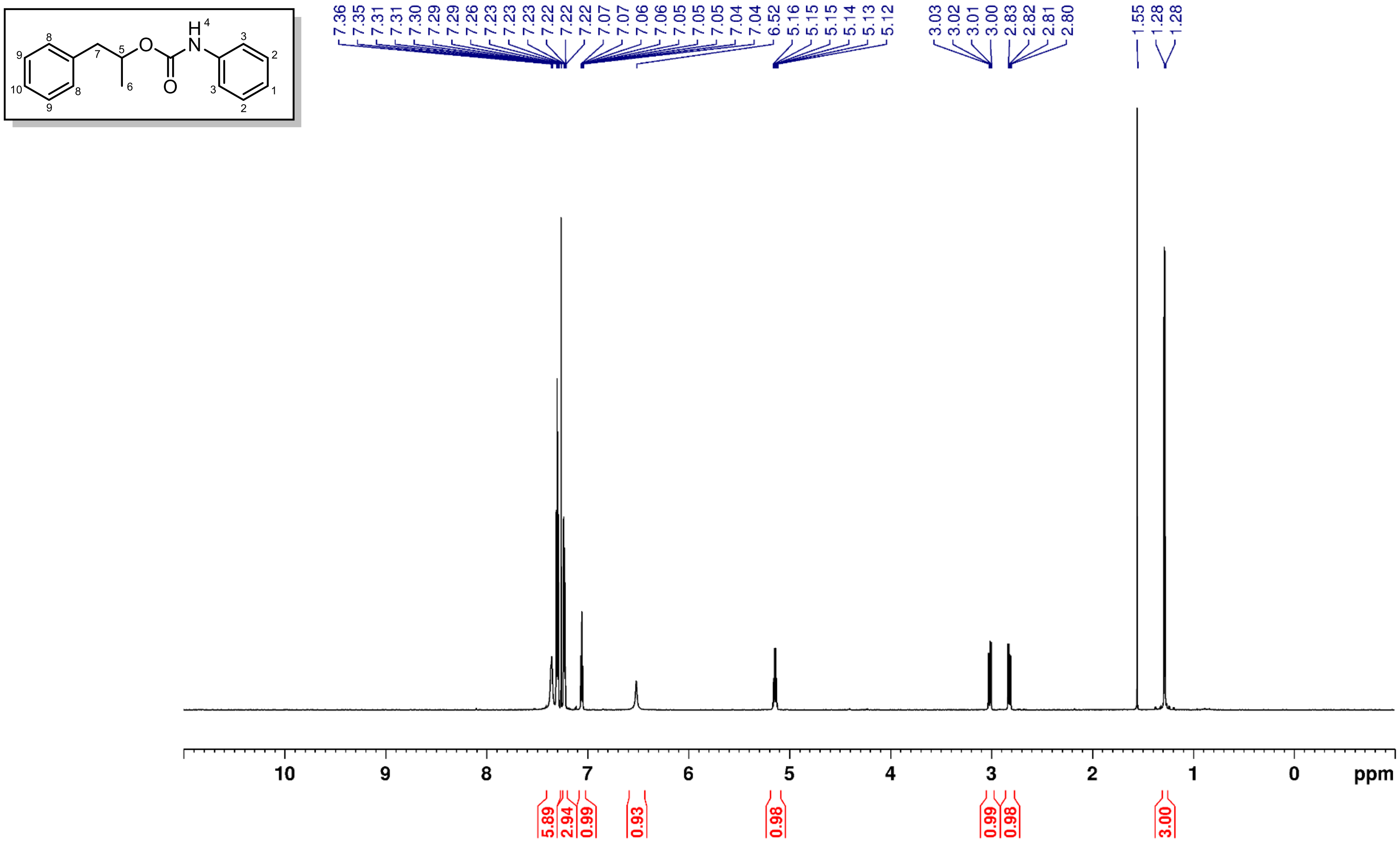

^{13}C NMR (176 MHz, CDCl$_3$) for 1-phenylpropan-2-yl phenylcarbamate

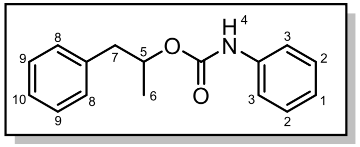

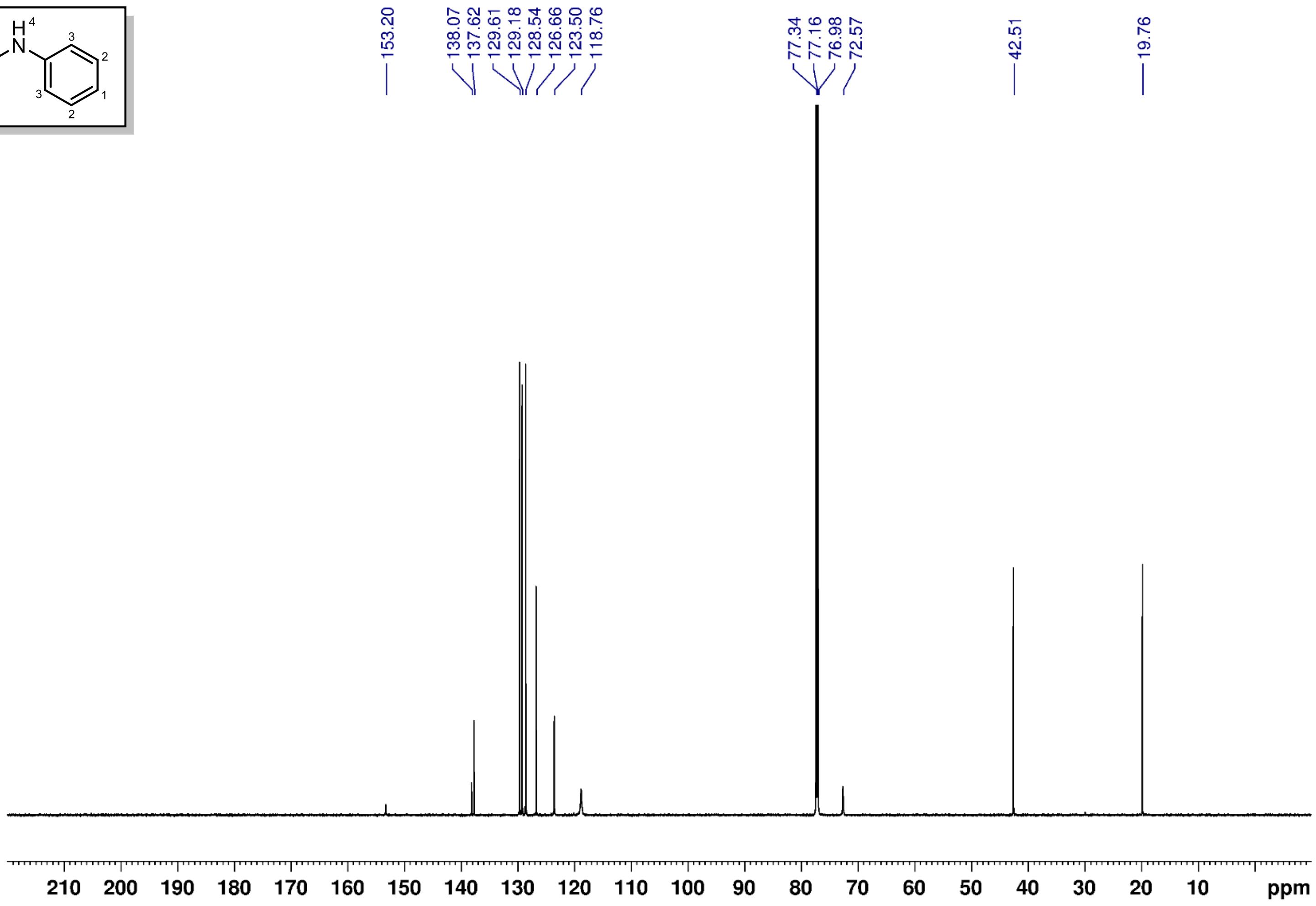

^{1}**H NMR (400 MHz, CDCl$_3$)** for phenethyl methyl(phenyl)carbamate (**S4**)

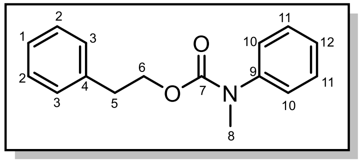

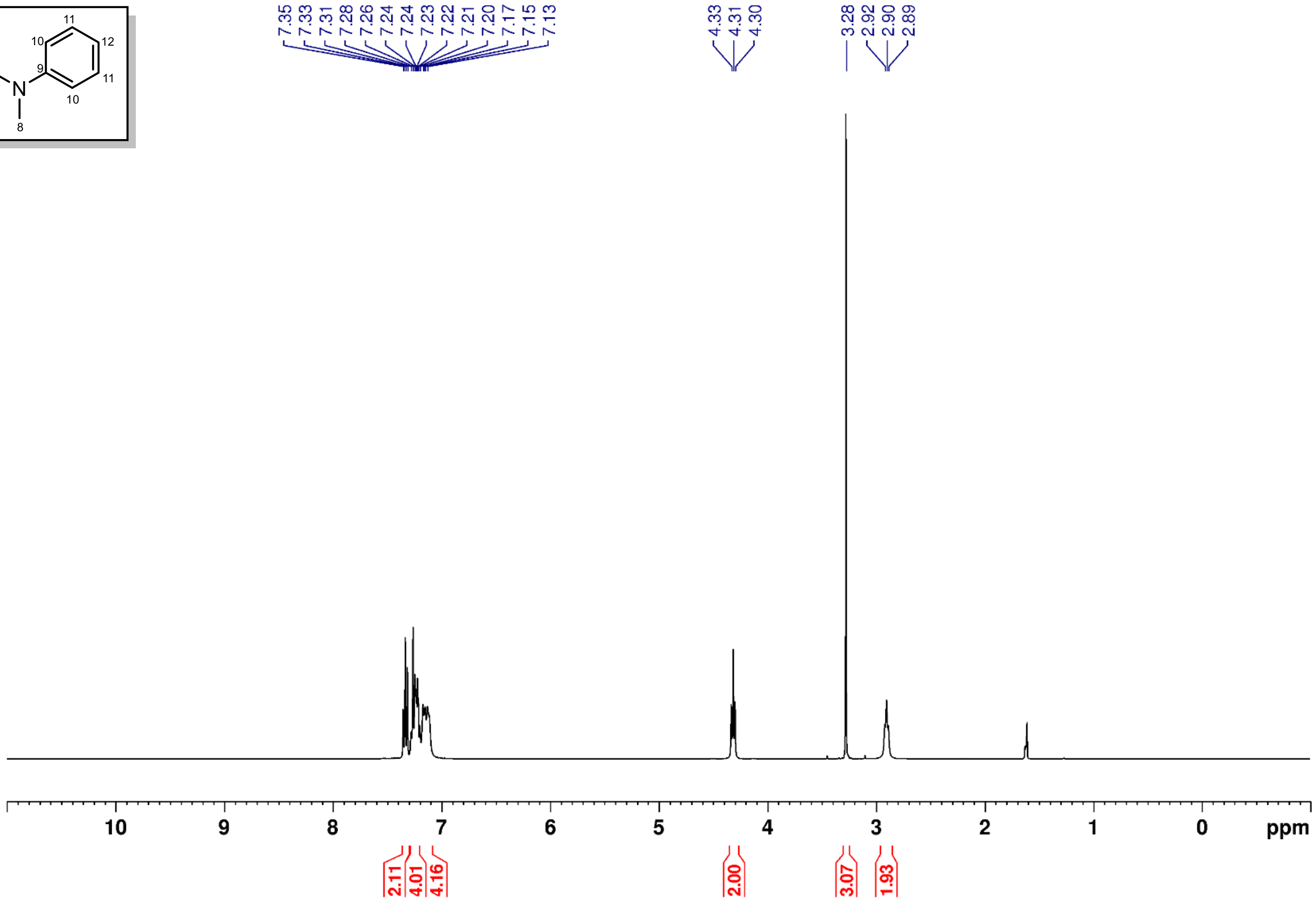

^{13}C NMR (101 MHz, CDCl$_3$) for phenethyl methyl(phenyl)carbamate (**S4**)

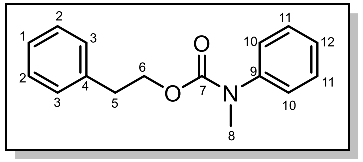

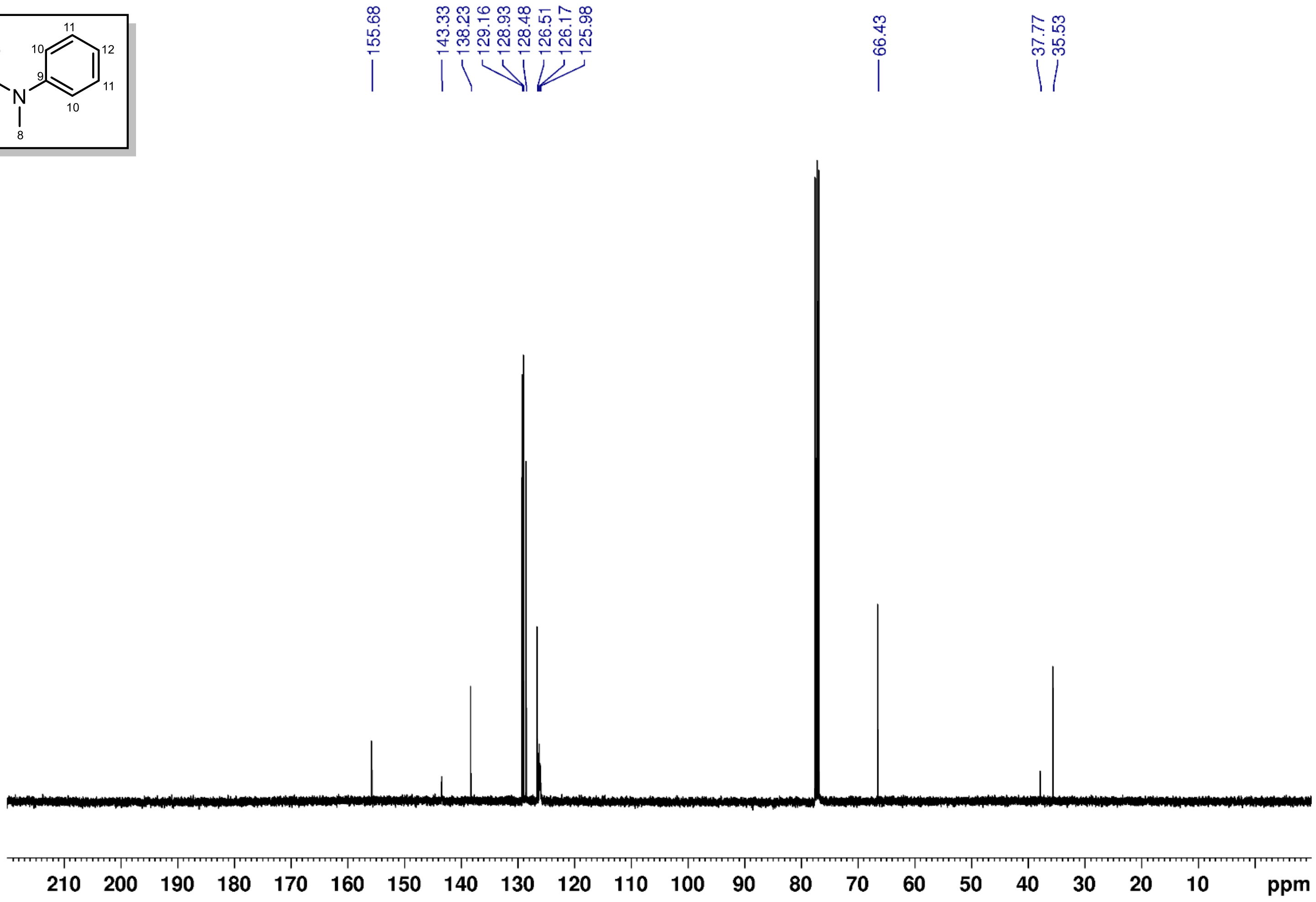

^{1}H NMR (700 MHz, CDCl$_3$) for pentyl methyl(phenyl)carbamate

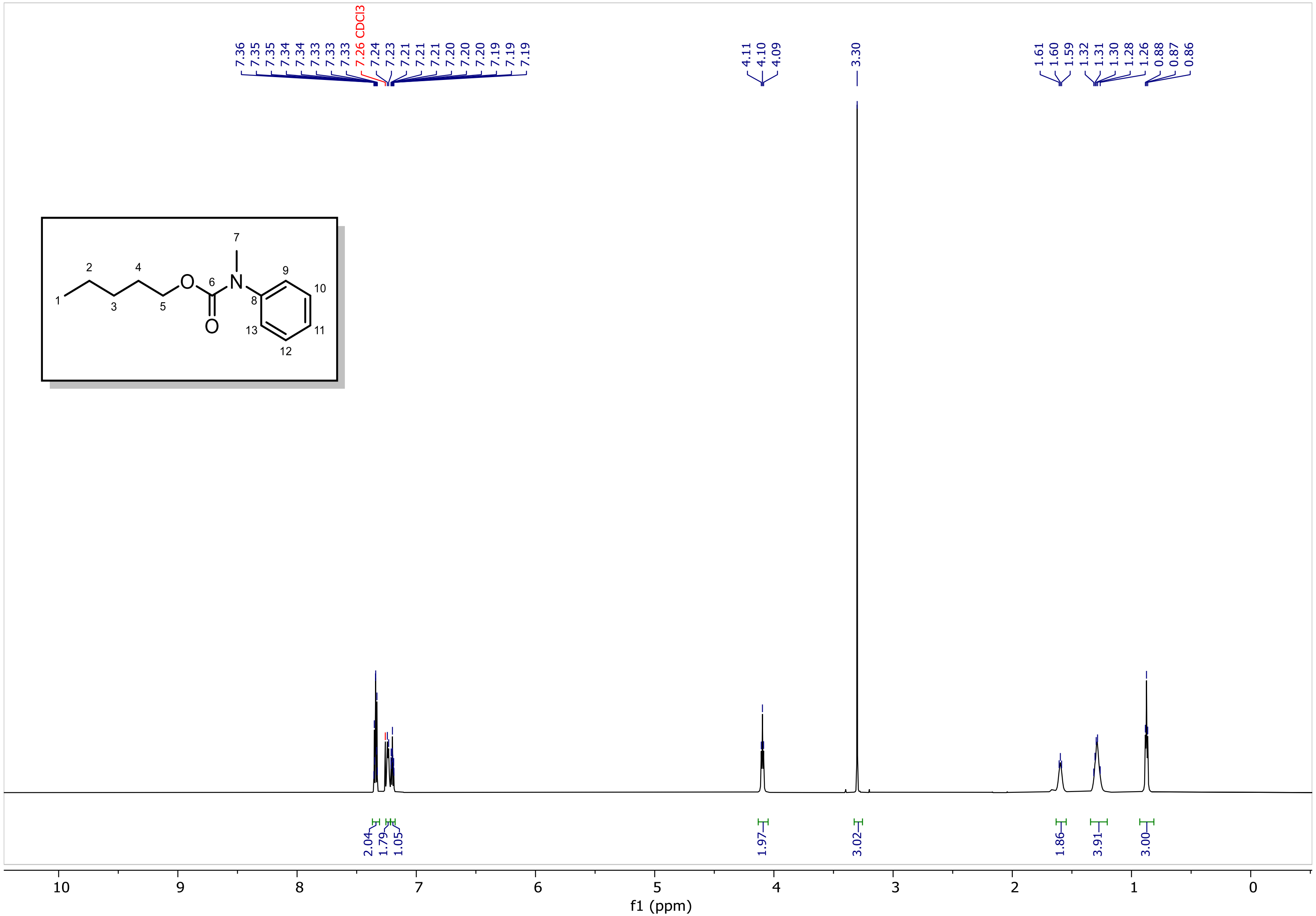

^{13}C NMR (176 MHz, CDCl$_3$) for pentyl methyl(phenyl)carbamate

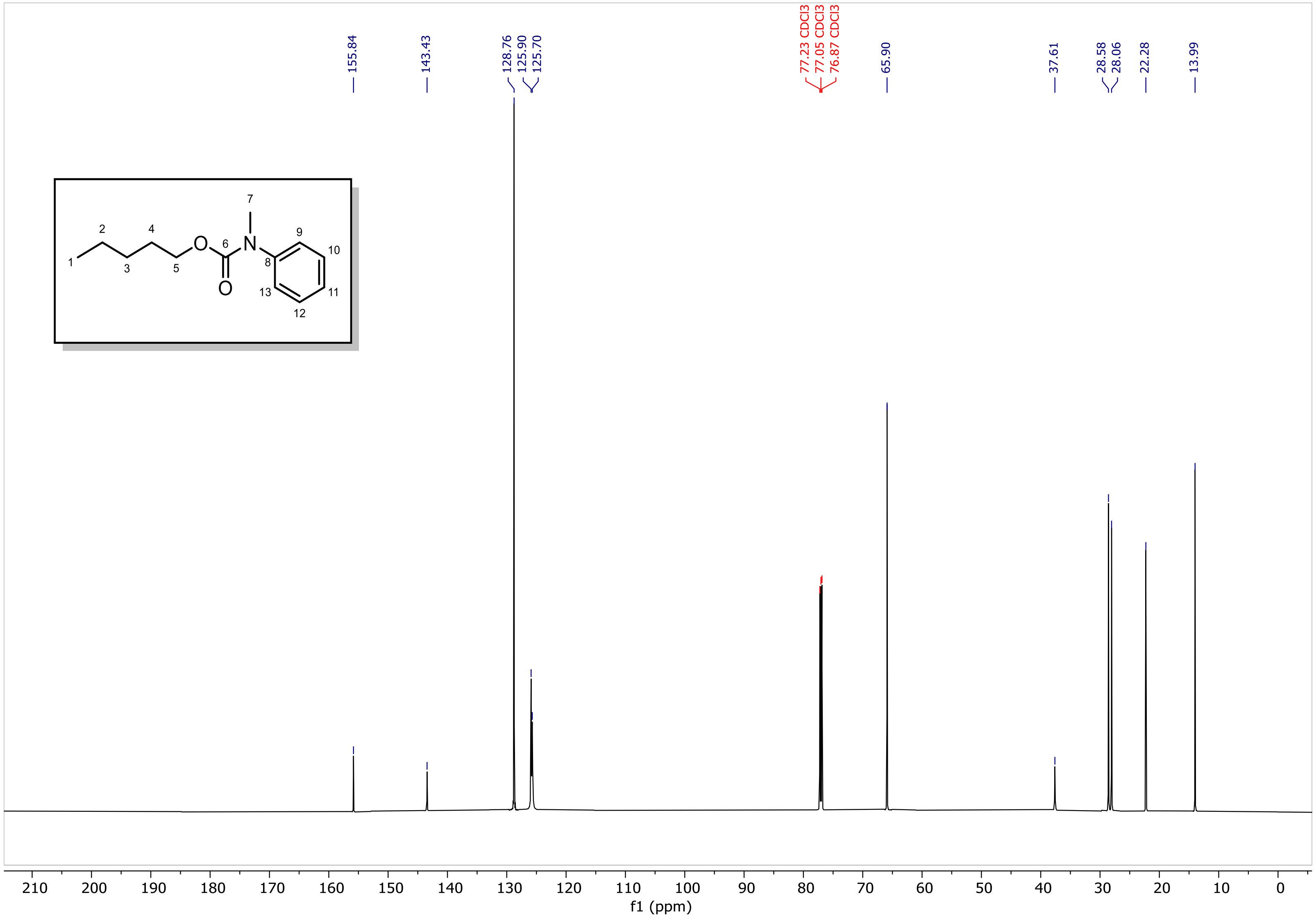

NMR Spectra for Substrates – N-Et Carbamates for C-H Amination:

^{1}H NMR (700 MHz, CD_3CN) for phenethyl ethyl(phenyl)carbamate (**10a**)

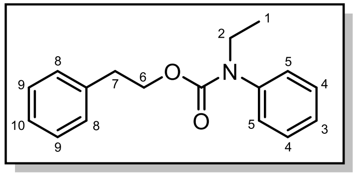

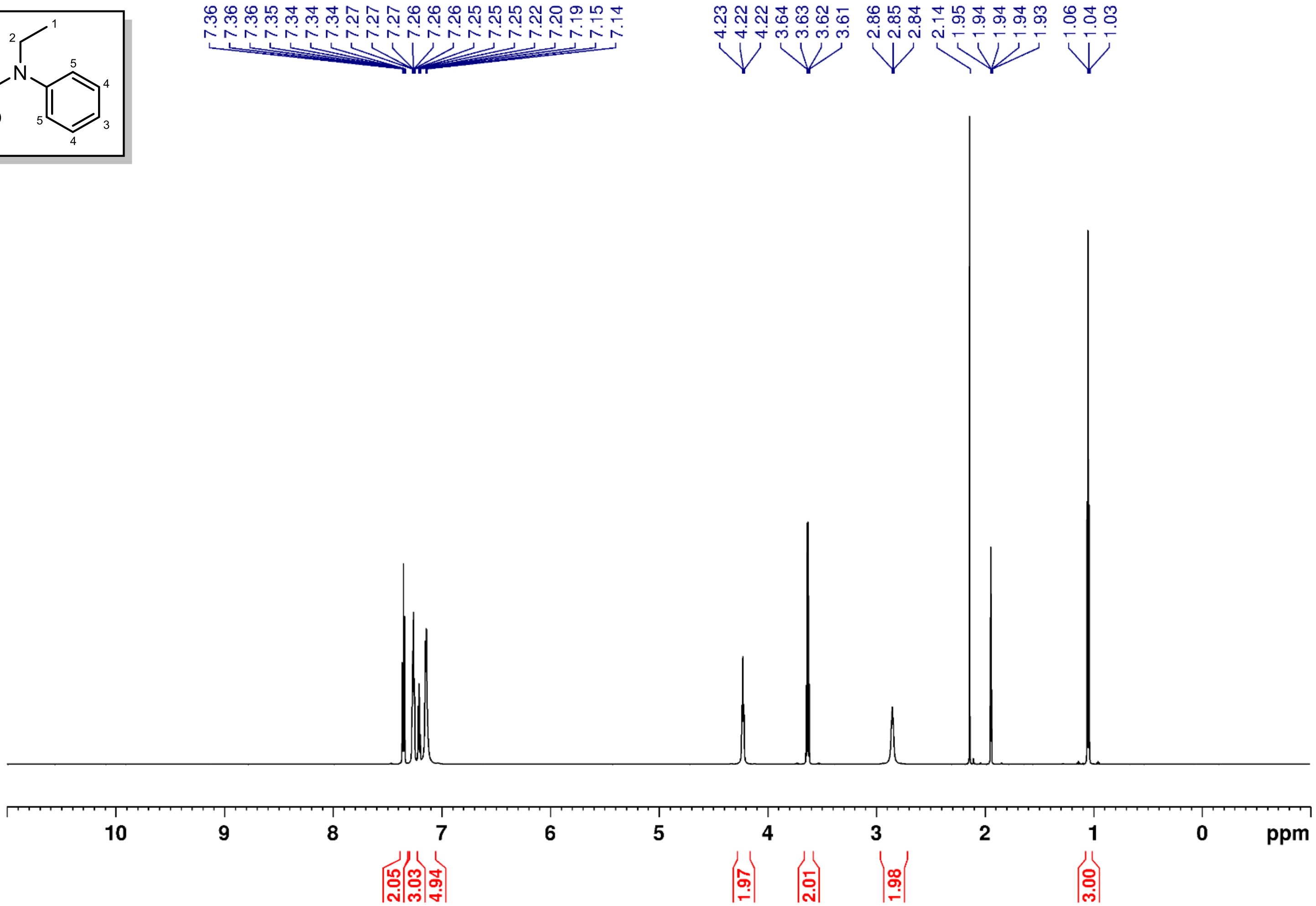

^{13}C NMR (176 MHz, CD_3CN) for phenethyl ethyl(phenyl)carbamate (**10a**)

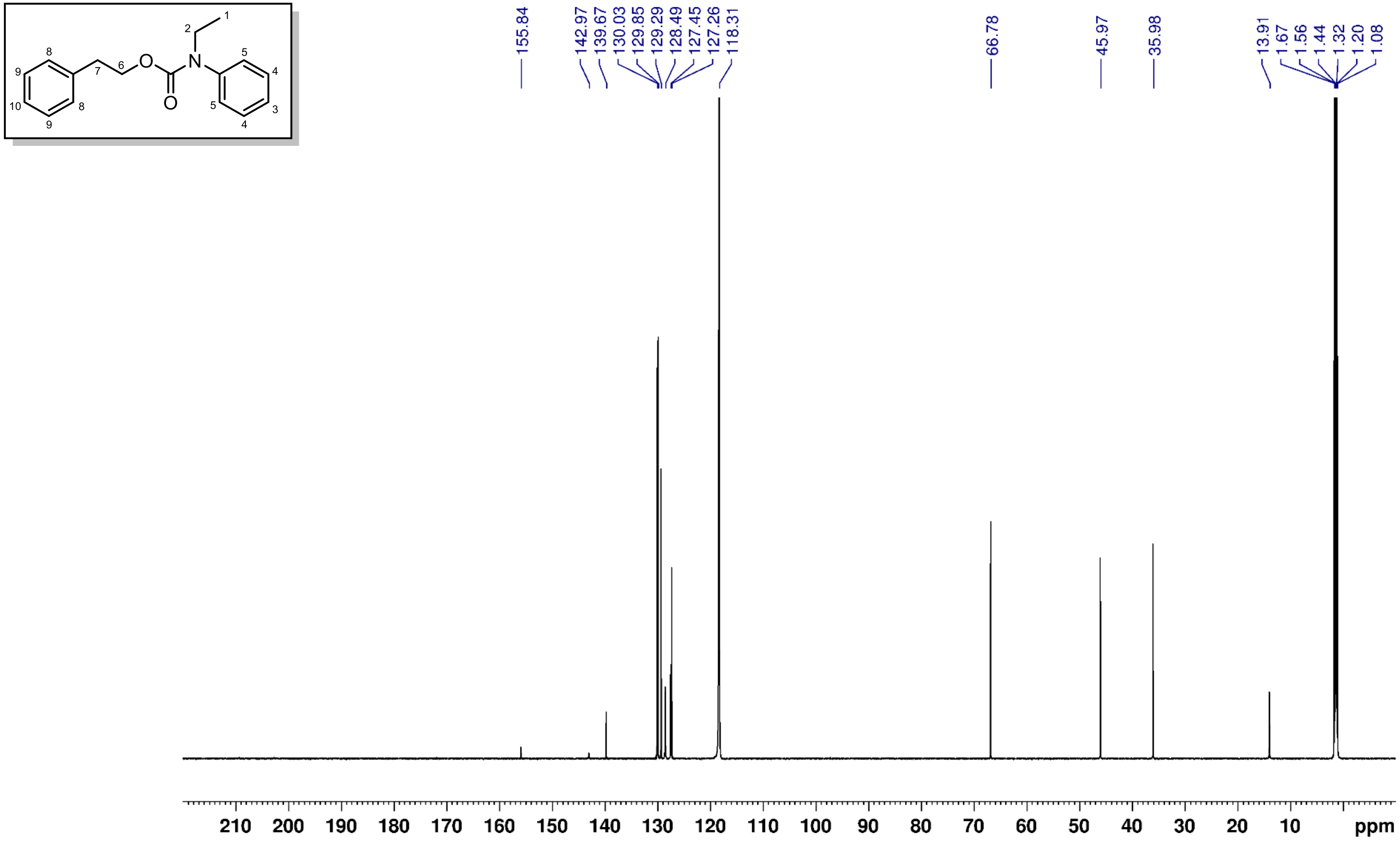

^{1}H NMR (700 MHz, CD_3CN) for 4-methoxyphenethyl ethyl(phenyl)carbamate (**10b**)

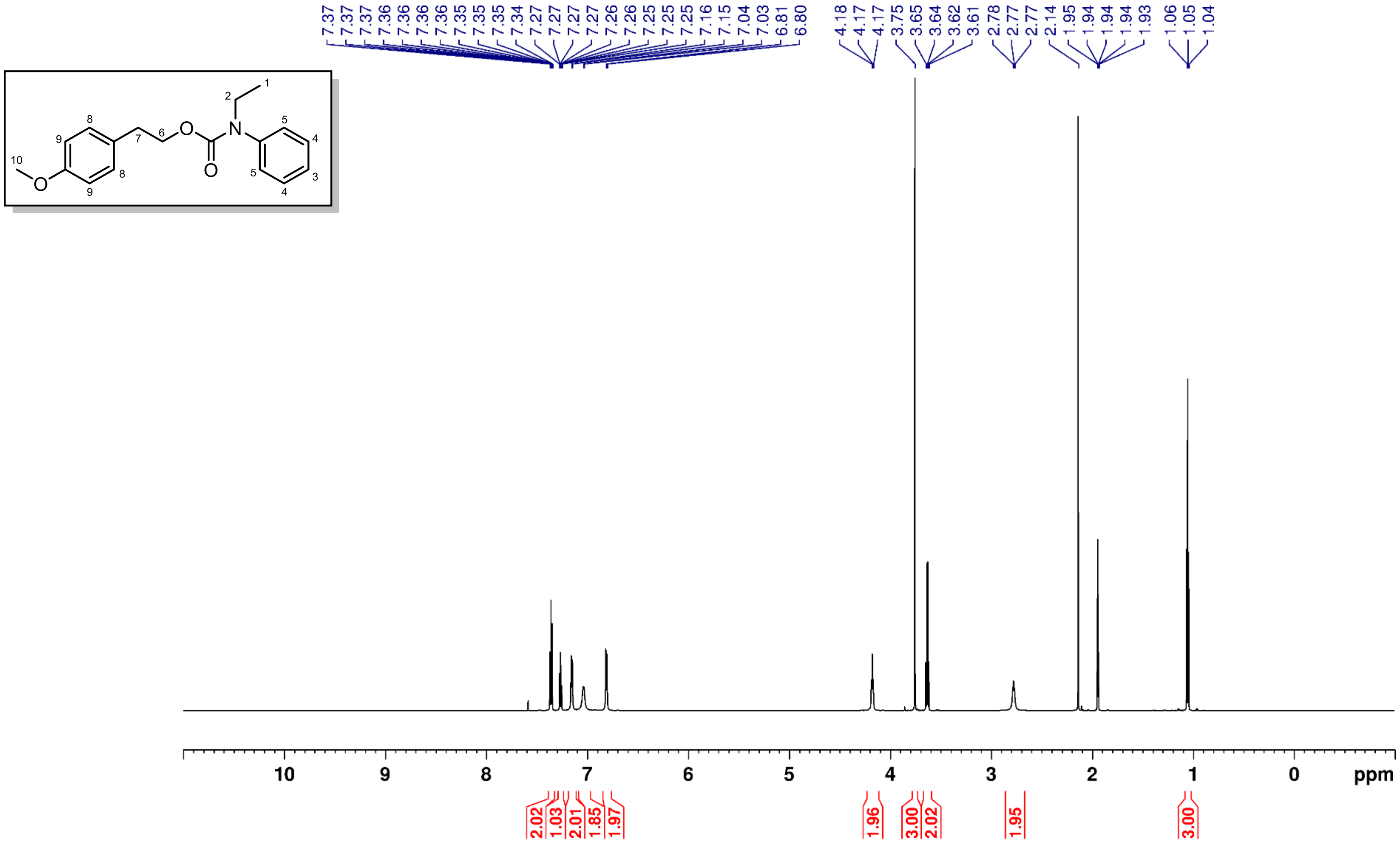

^{13}C NMR (176 MHz, CD_3CN) for 4-methoxyphenethyl ethyl(phenyl)carbamate (10b)

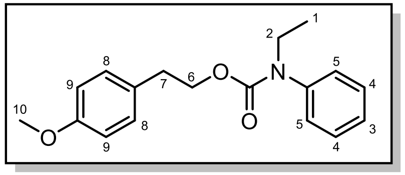

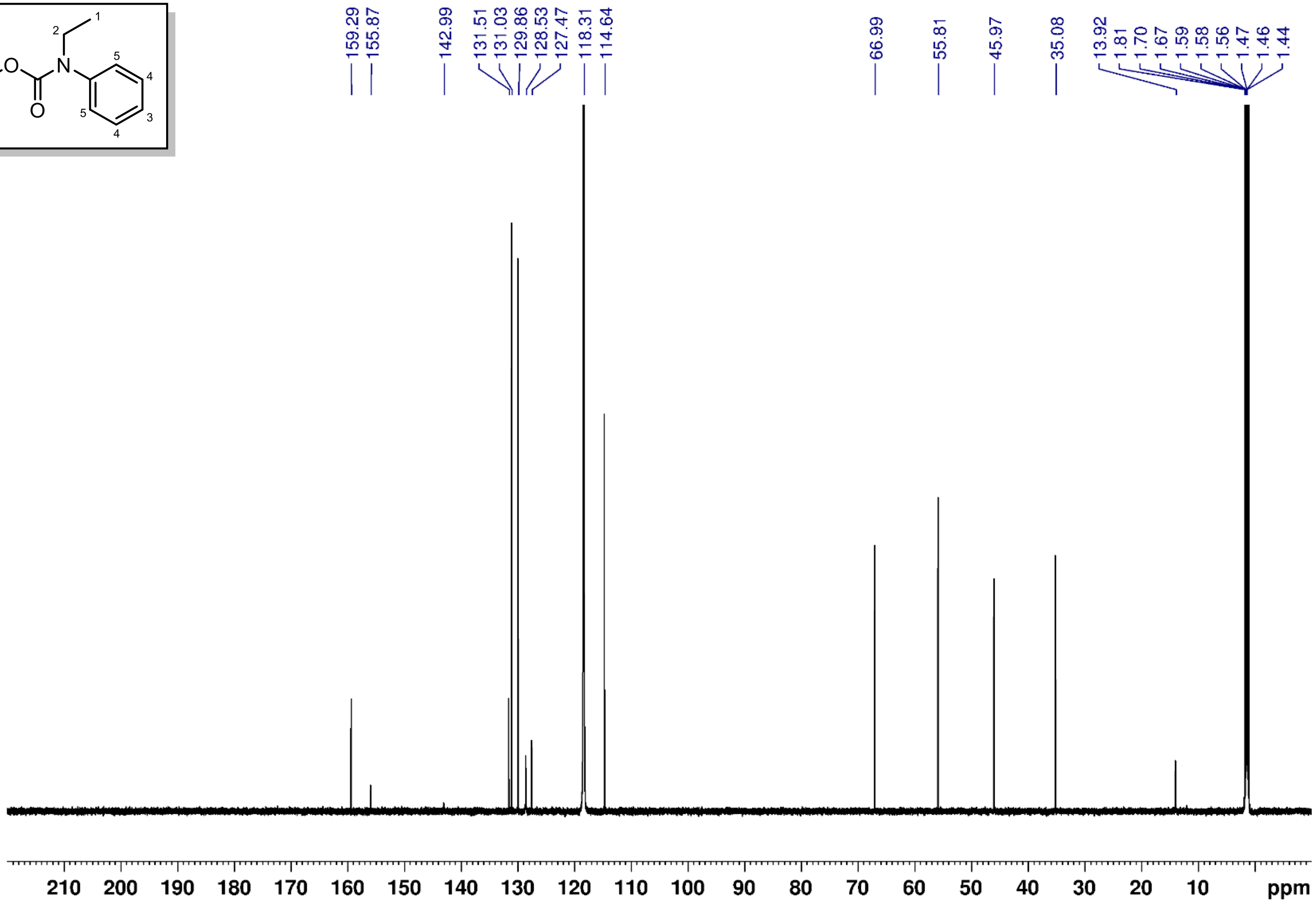

^{1}H NMR (700 MHz, CD_3CN) for 4-(*tert*-butyl)phenethyl ethyl(phenyl)carbamate (**10c**)

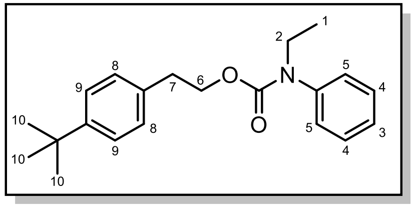

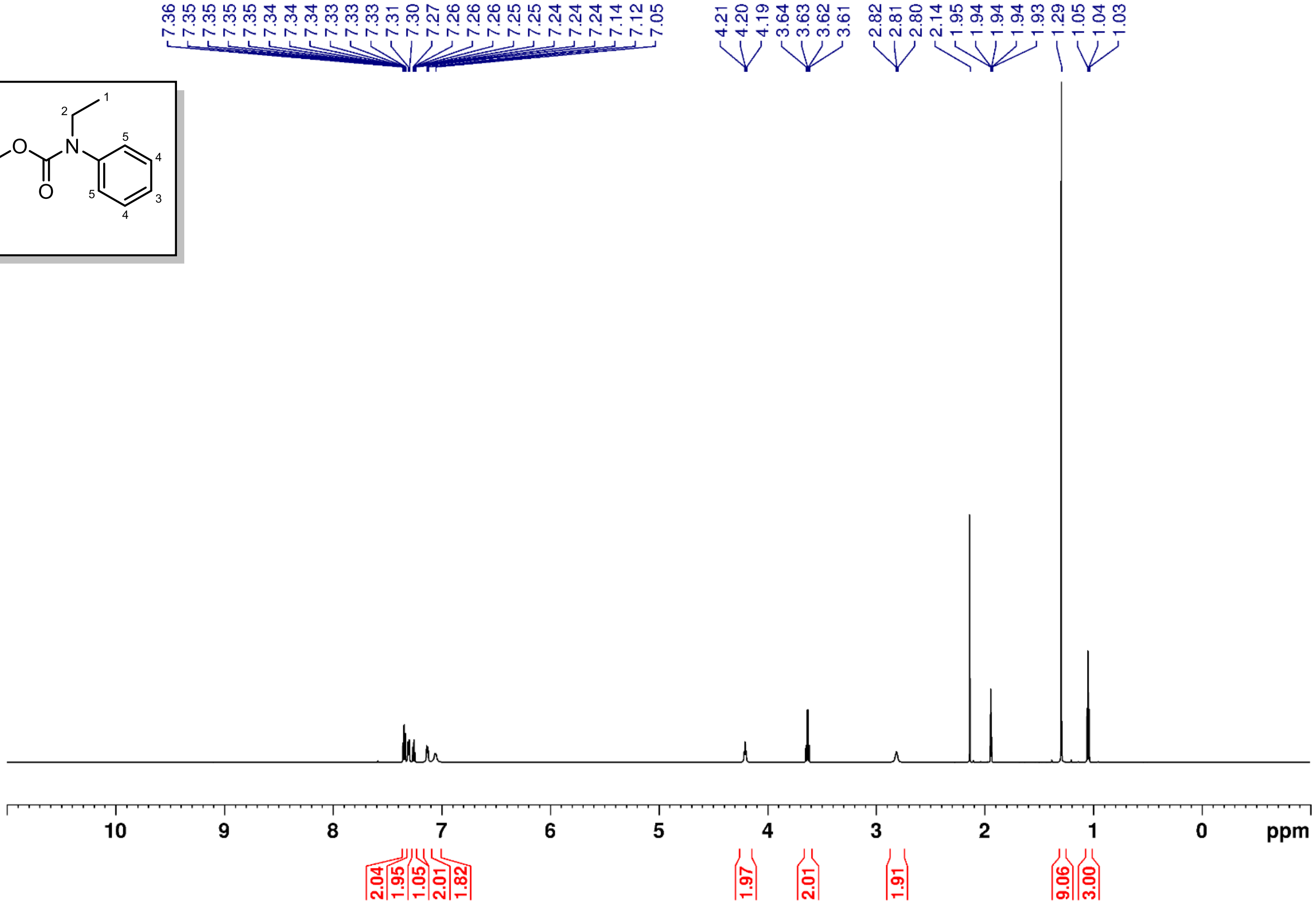

^{13}C NMR (176 MHz, CD_3CN) for 4-(*tert*-butyl)phenethyl ethyl(phenyl)carbamate (**10c**)

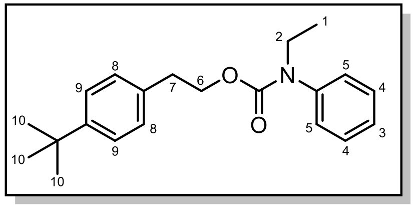

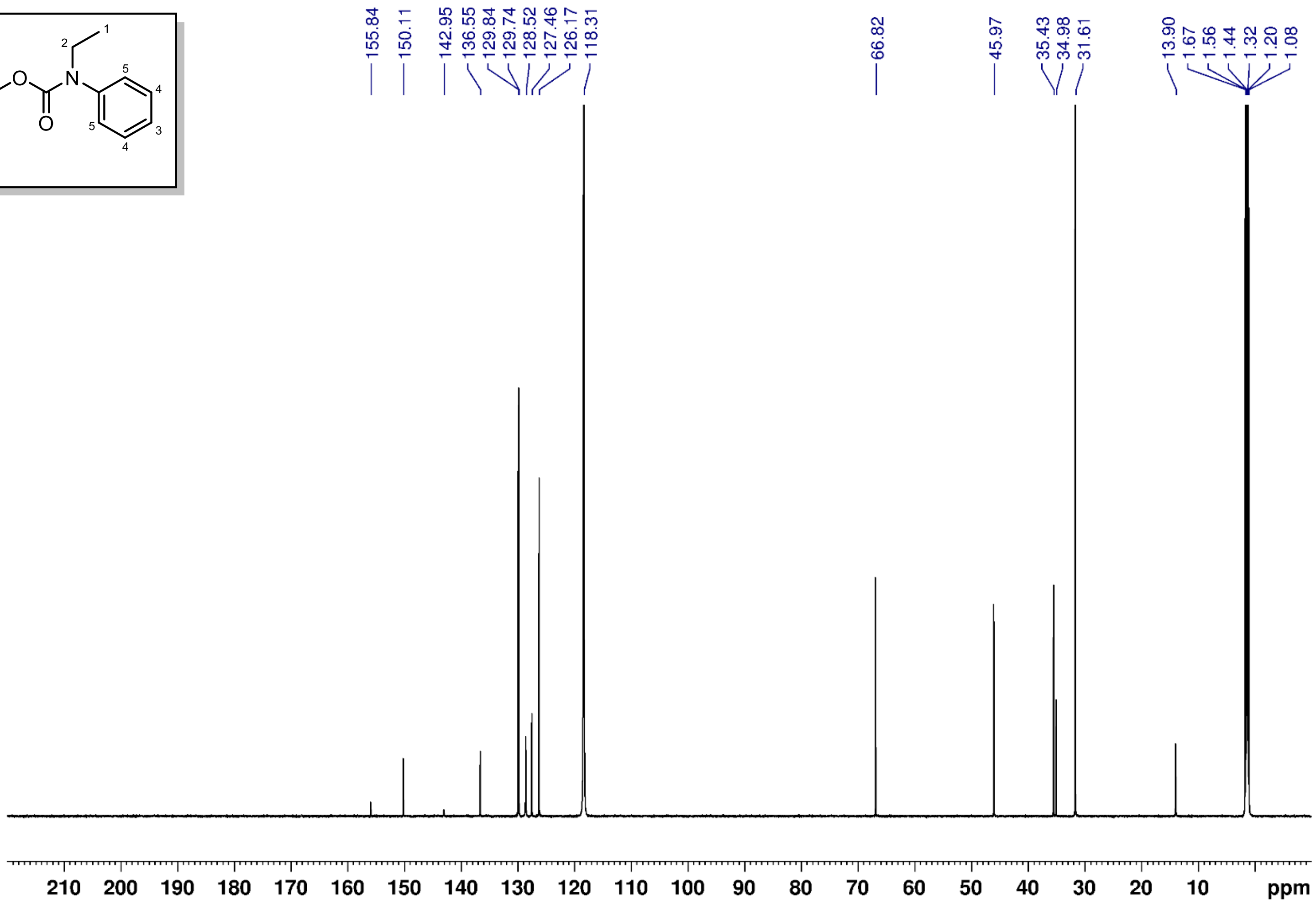

^{1}H NMR (700 MHz, CD_3CN) for 4-methylphenethyl ethyl(phenyl)carbamate (**10d**)

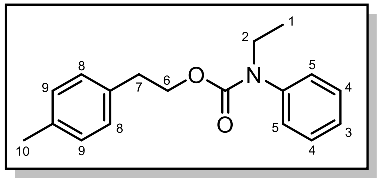

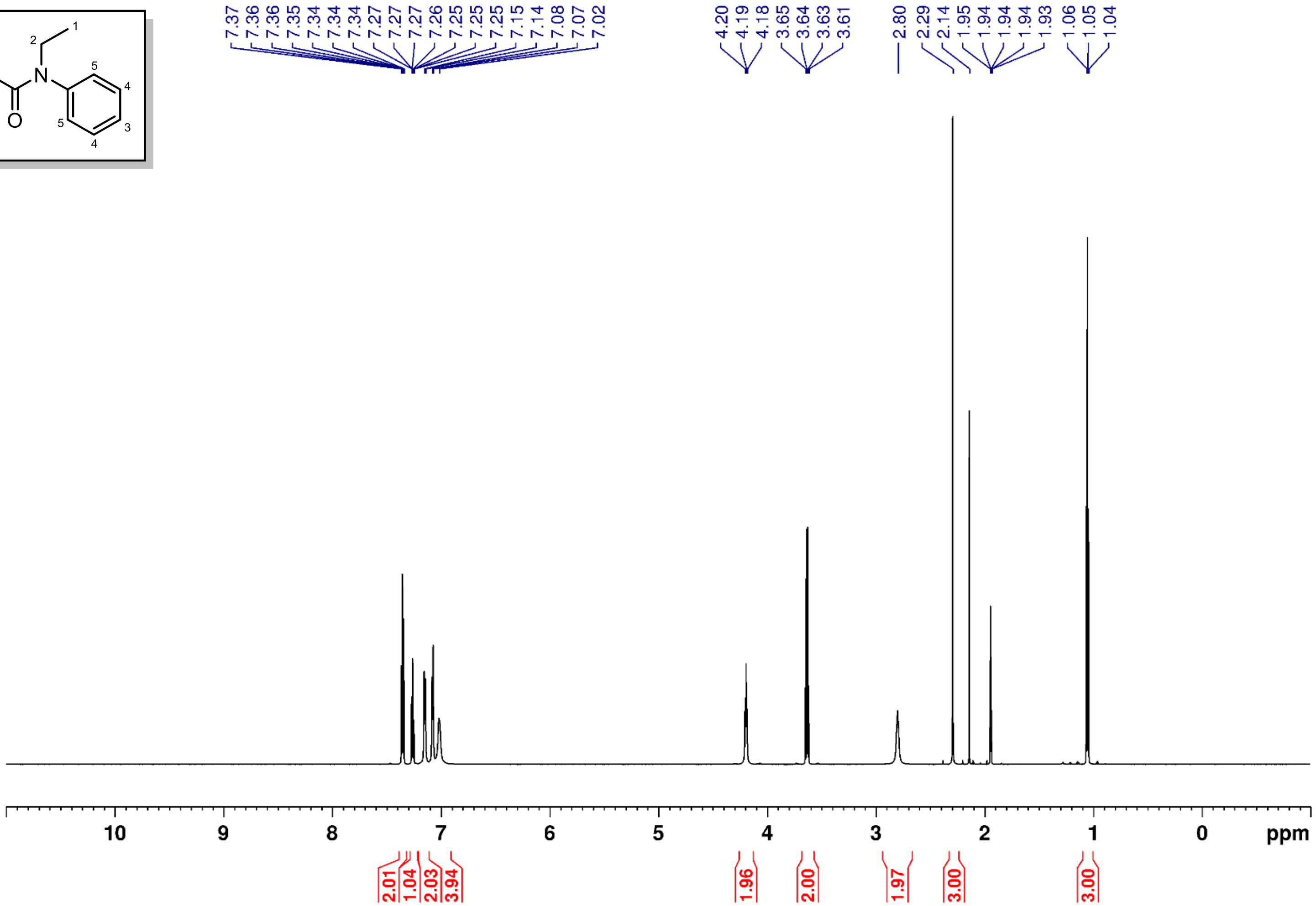

^{13}C NMR (176 MHz, CD_3CN) for 4-methylphenethyl ethyl(phenyl) carbamate (**10d**)

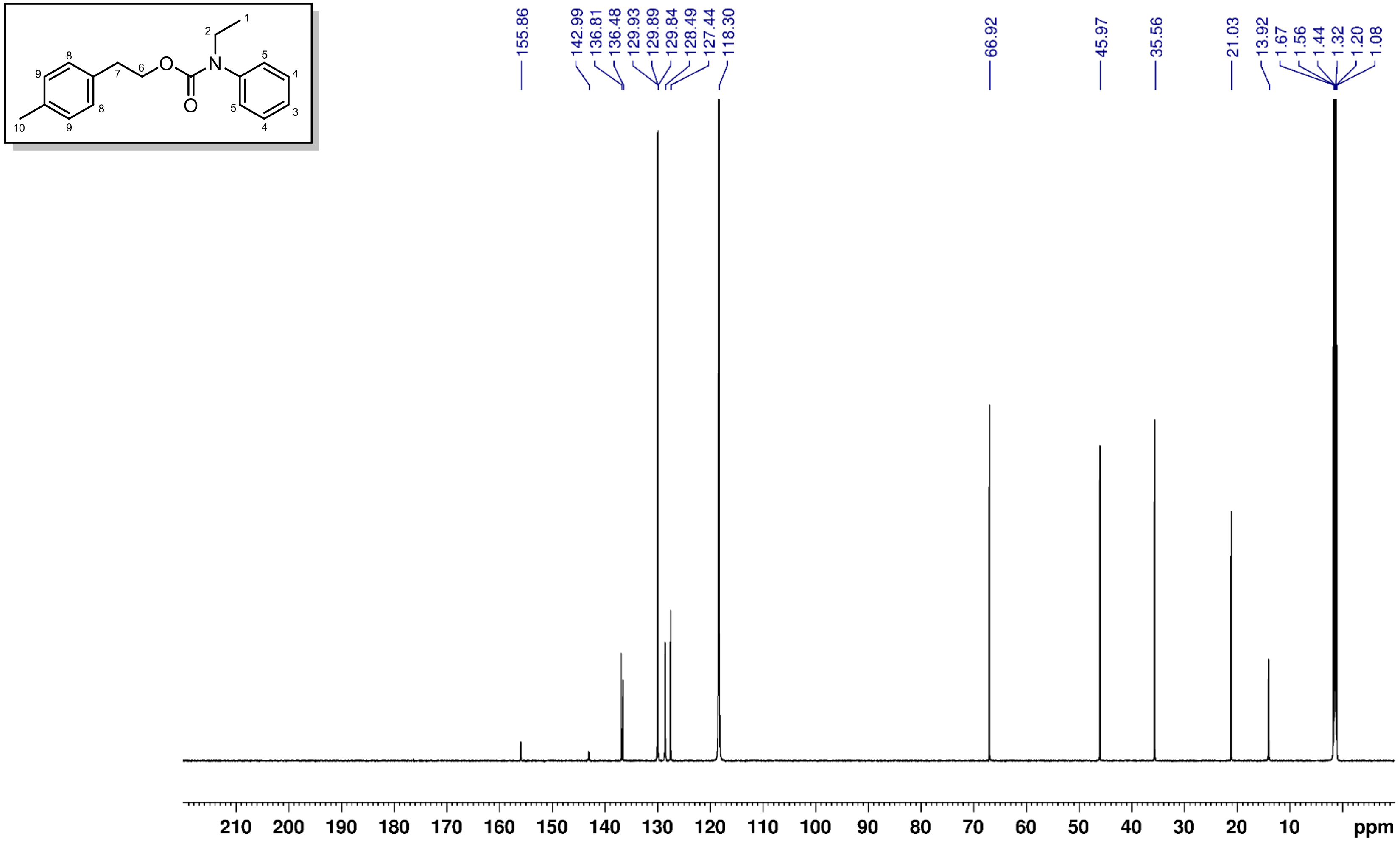

^{1}H NMR (700 MHz, CD$_3$CN) for 4-bromophenethyl ethyl(phenyl)carbamate (**10e**)

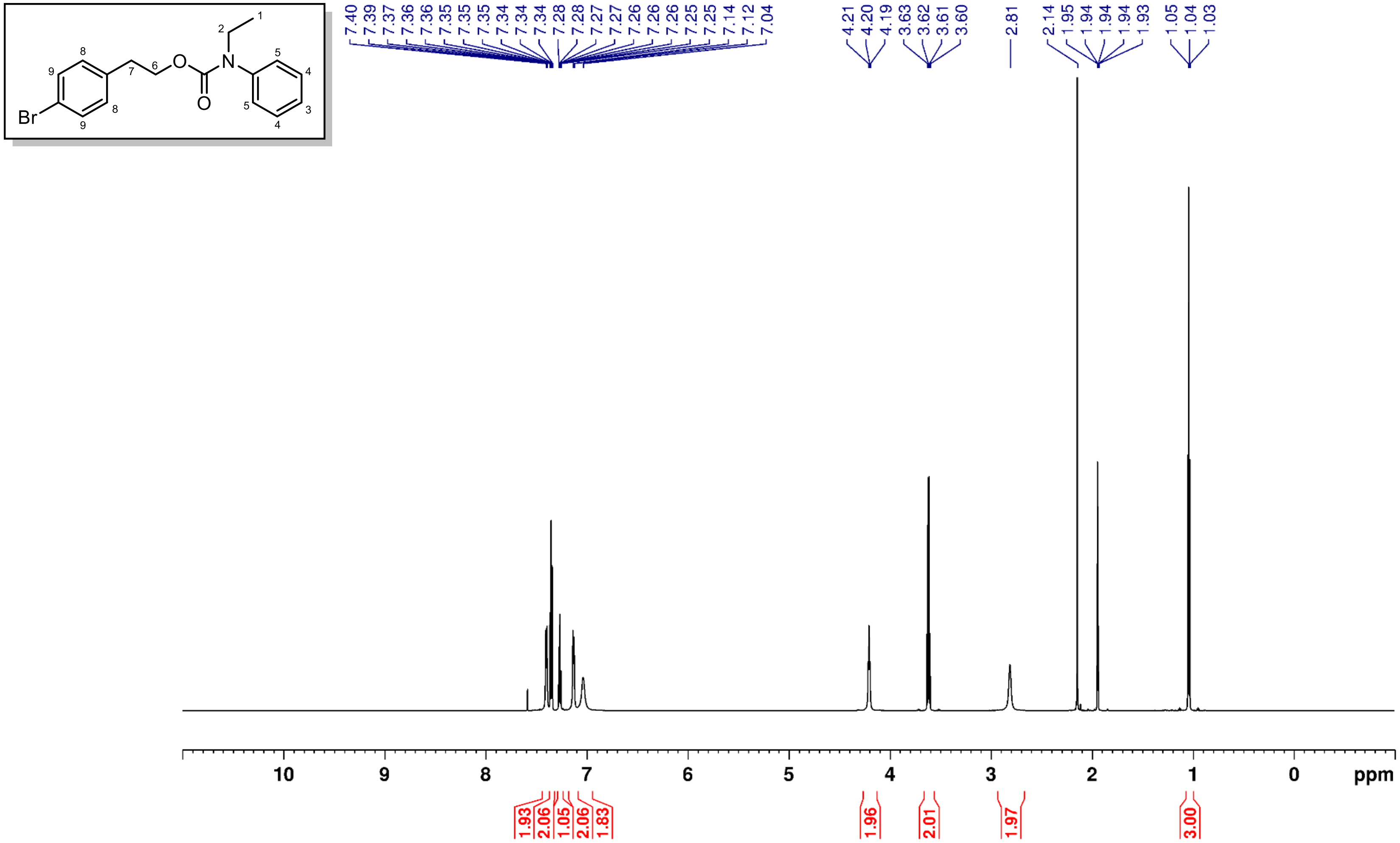

^{13}C NMR (176 MHz, CD_3CN) for 4-bromophenethyl ethyl(phenyl)carbamate (**10e**)

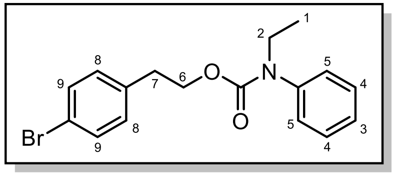

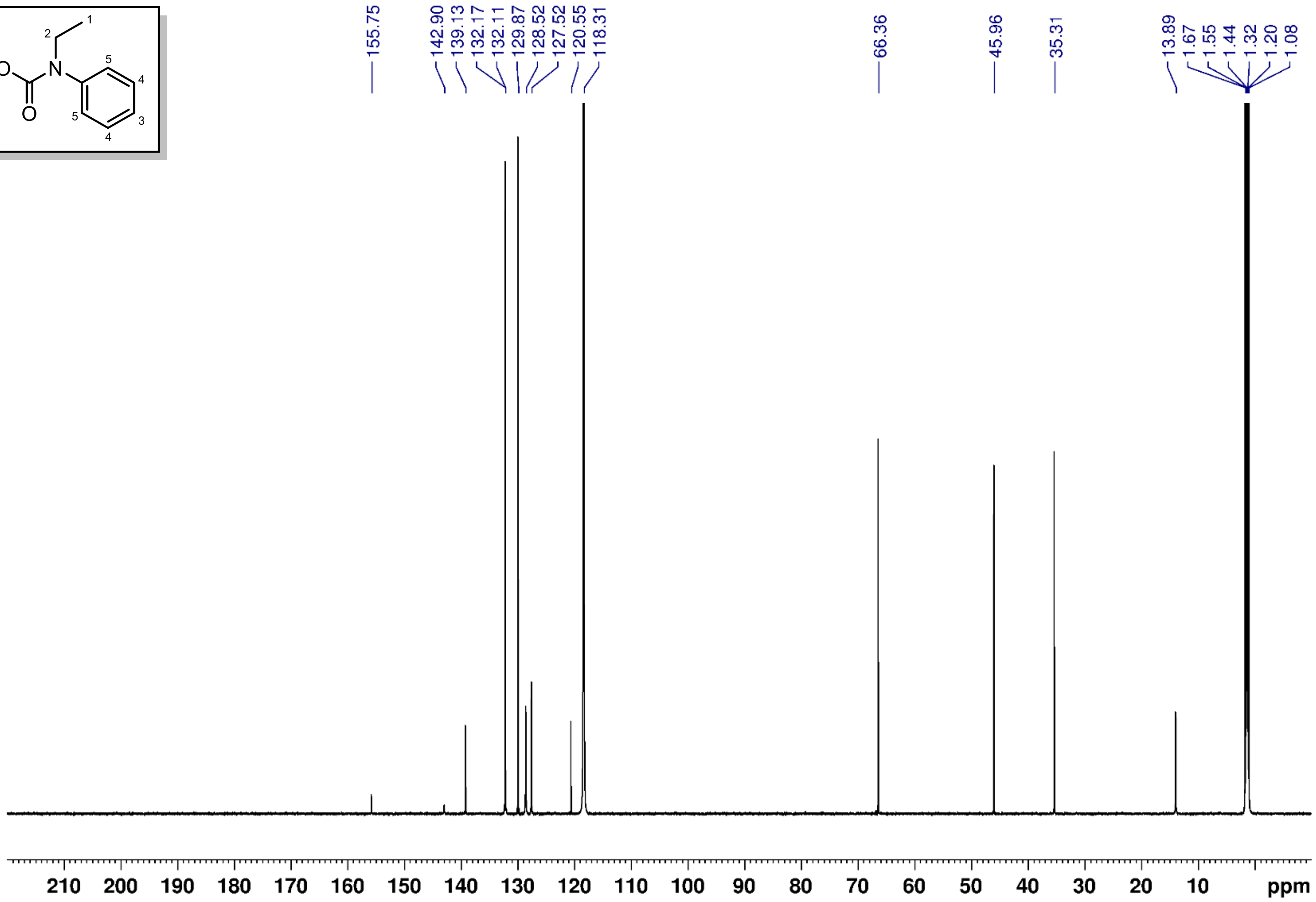

^{1}H NMR (700 MHz, CD_3CN) for 4-chlorophenethyl ethyl(phenyl)carbamate (**10f**)

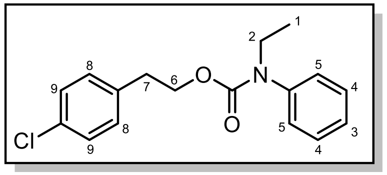

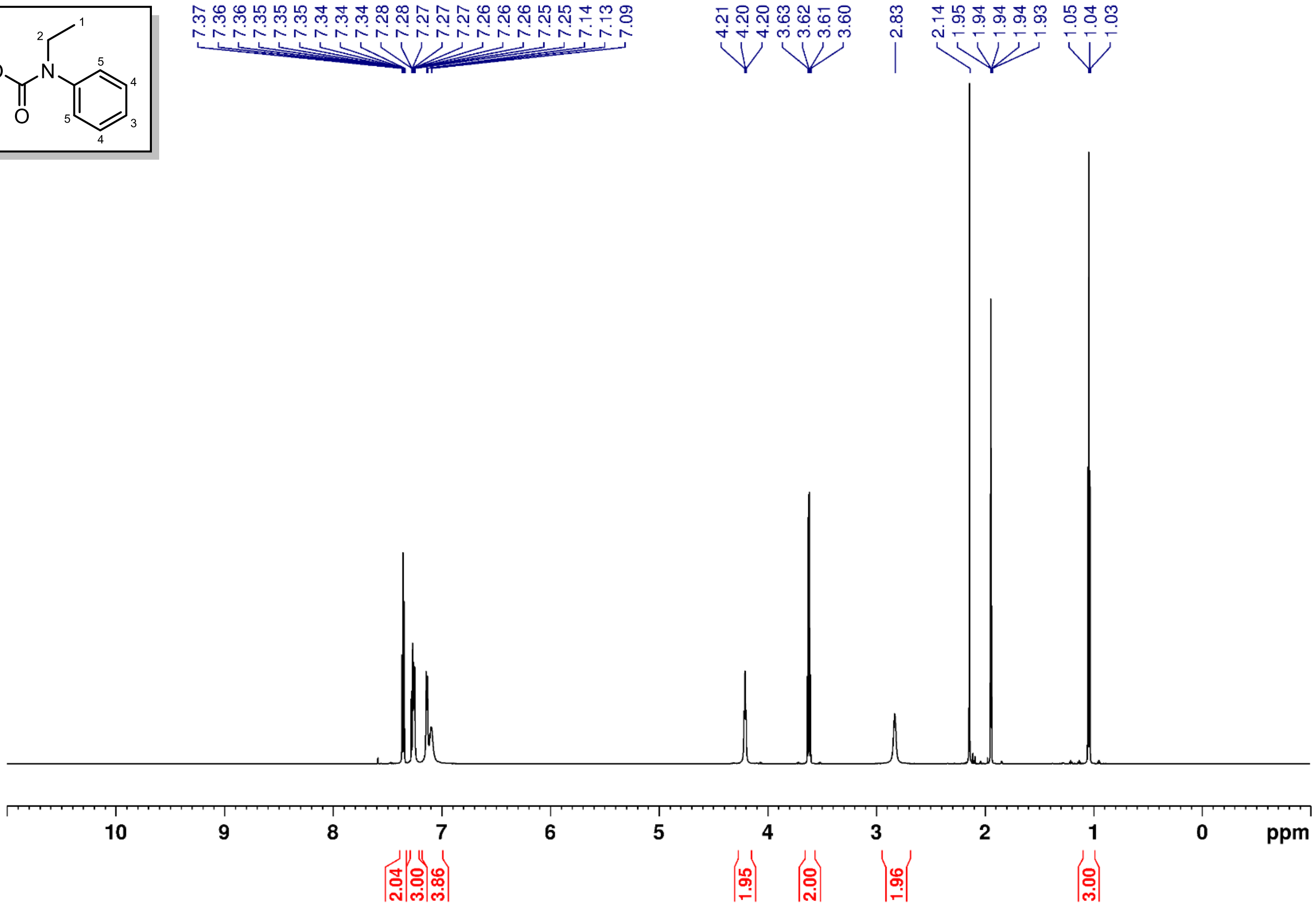

^{13}**C NMR (176 MHz, CD_3CN)** for 4-chlorophenethyl ethyl(phenyl)carbamate (**10f**)

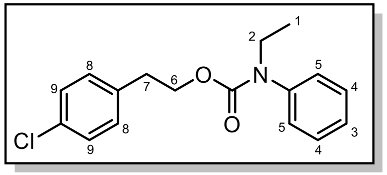

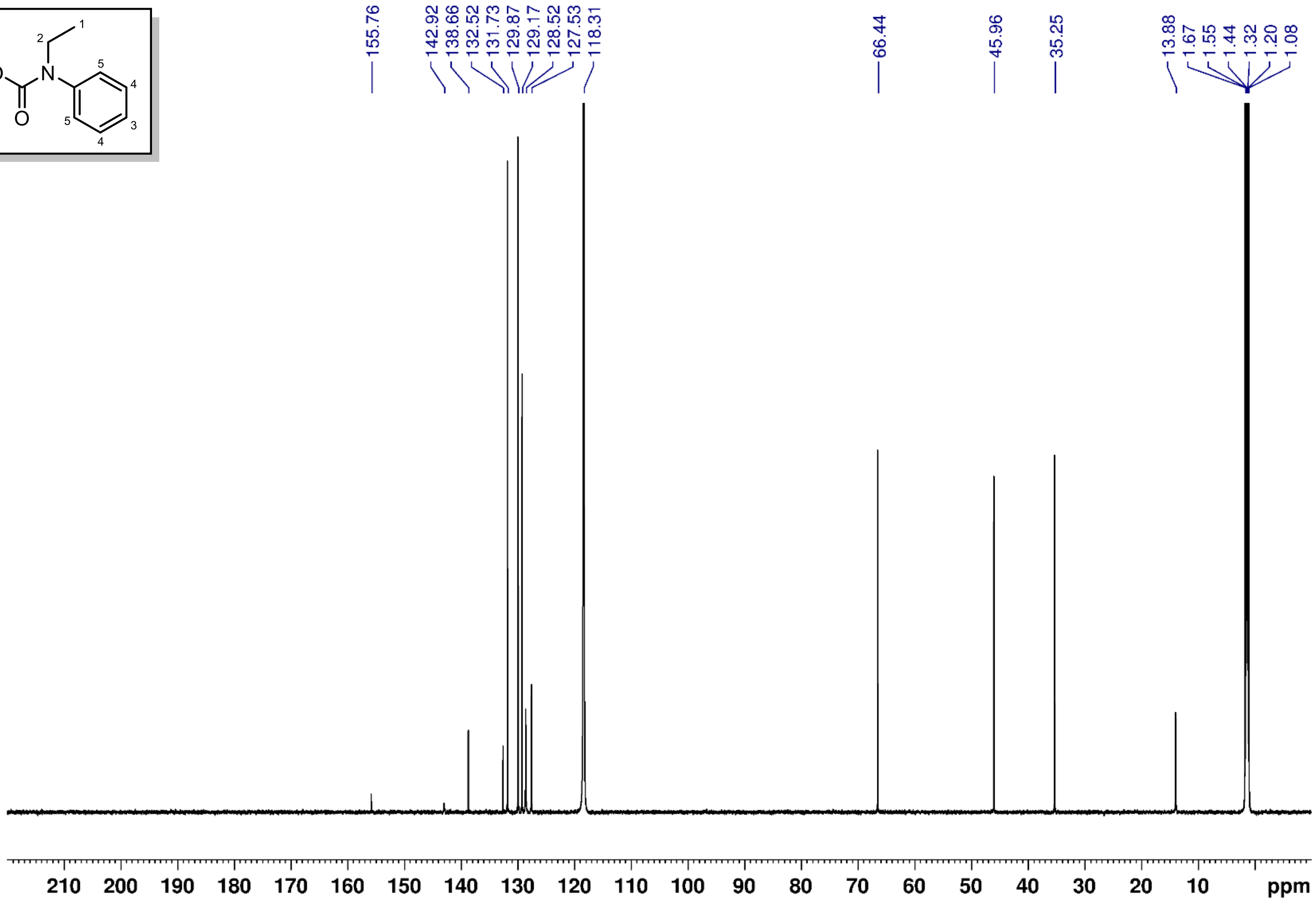

^{1}H NMR (700 MHz, CD_3CN) for 4-(trifluoromethyl)phenethyl ethyl(phenyl)carbamate (**10g**)

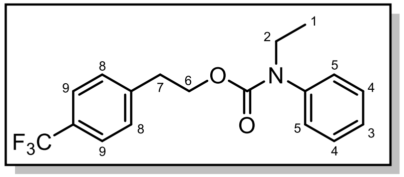

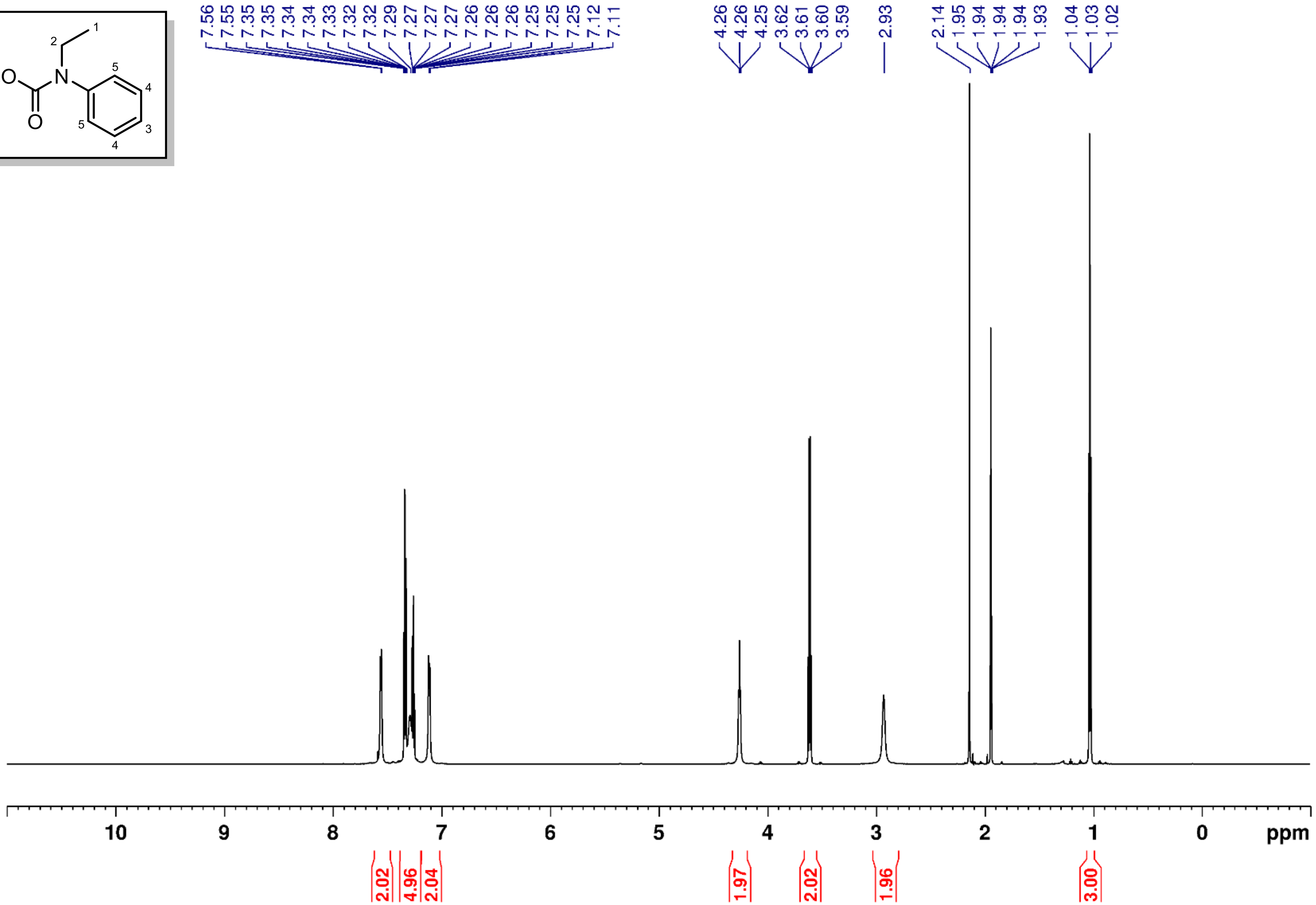

^{13}C NMR (176 MHz, CD_3CN) for 4-(trifluoromethyl)phenethyl ethyl(phenyl)carbamate (**10g**)

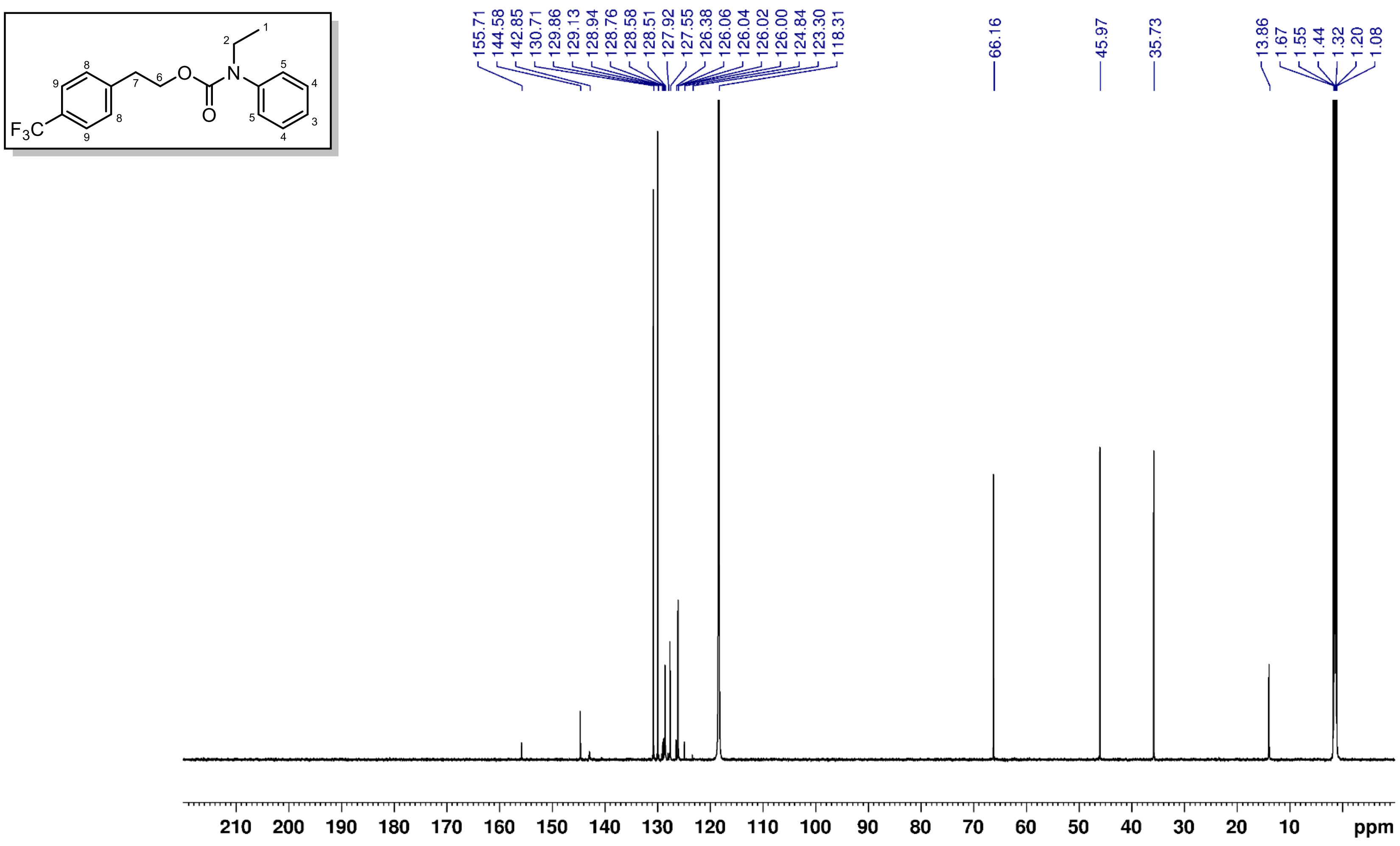

^{19}F NMR (376 MHz, CD_3CN) for 4-(trifluoromethyl)phenethyl ethyl(phenyl)carbamate (**10g**)

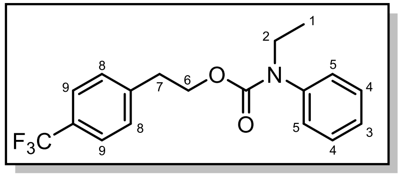

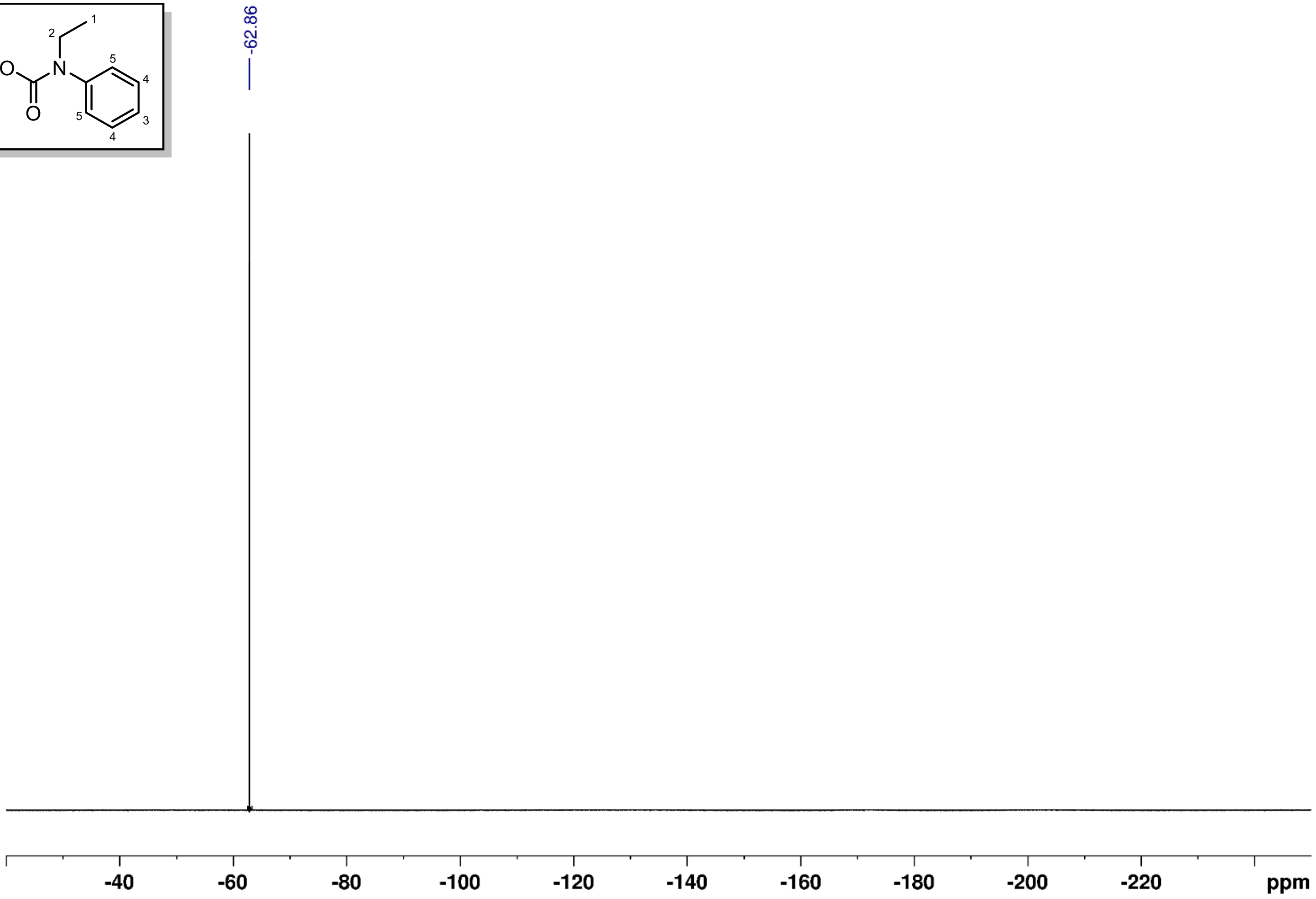

^{1}H NMR (700 MHz, CD_3CN) for 3-methoxyphenethyl ethyl(phenyl)carbamate (**10h**)

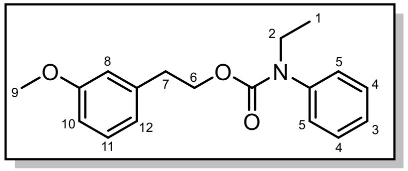

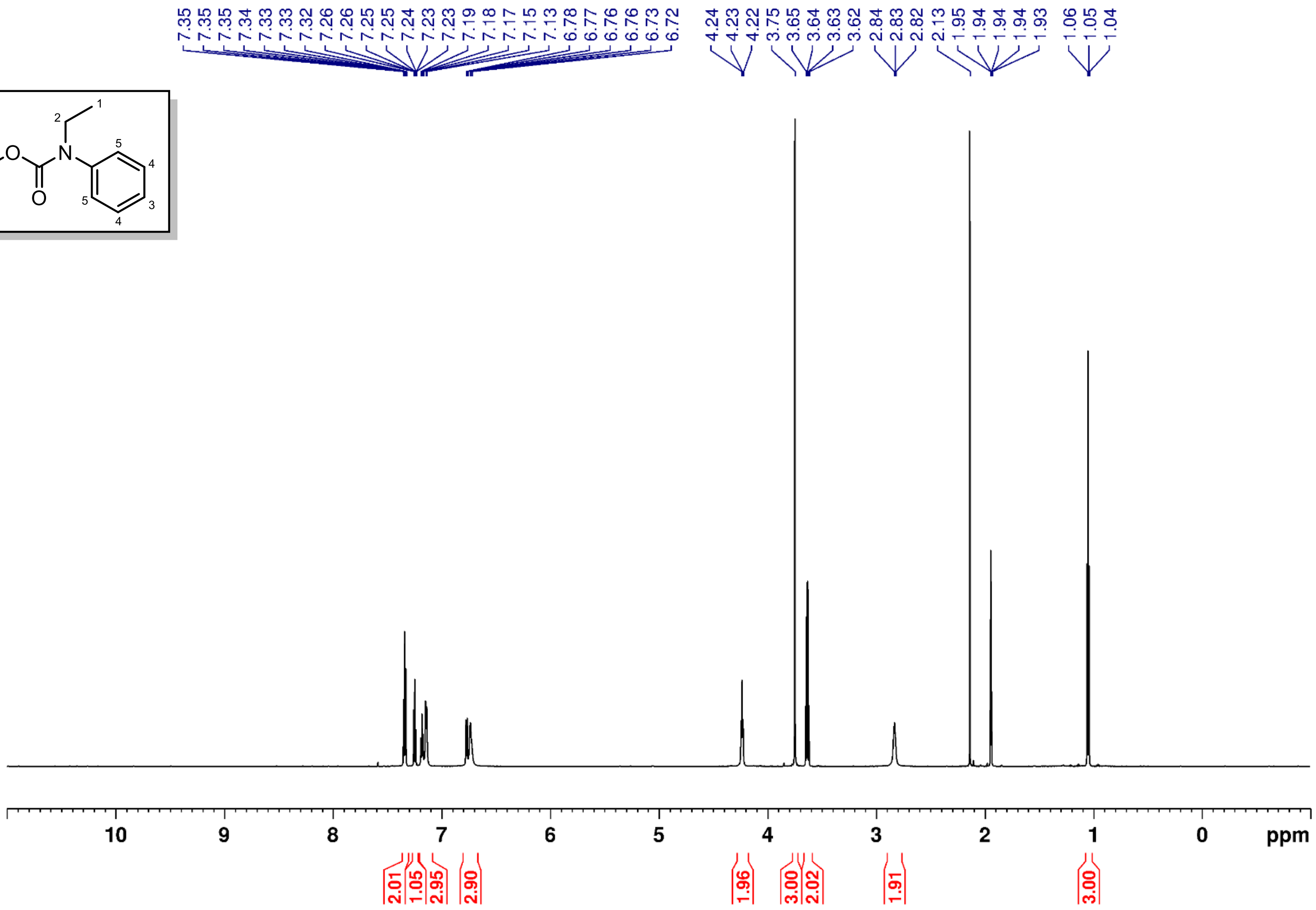

^{13}C NMR (176 MHz, CD_3CN) for 3-methoxyphenethyl ethyl(phenyl)carbamate (**10h**)

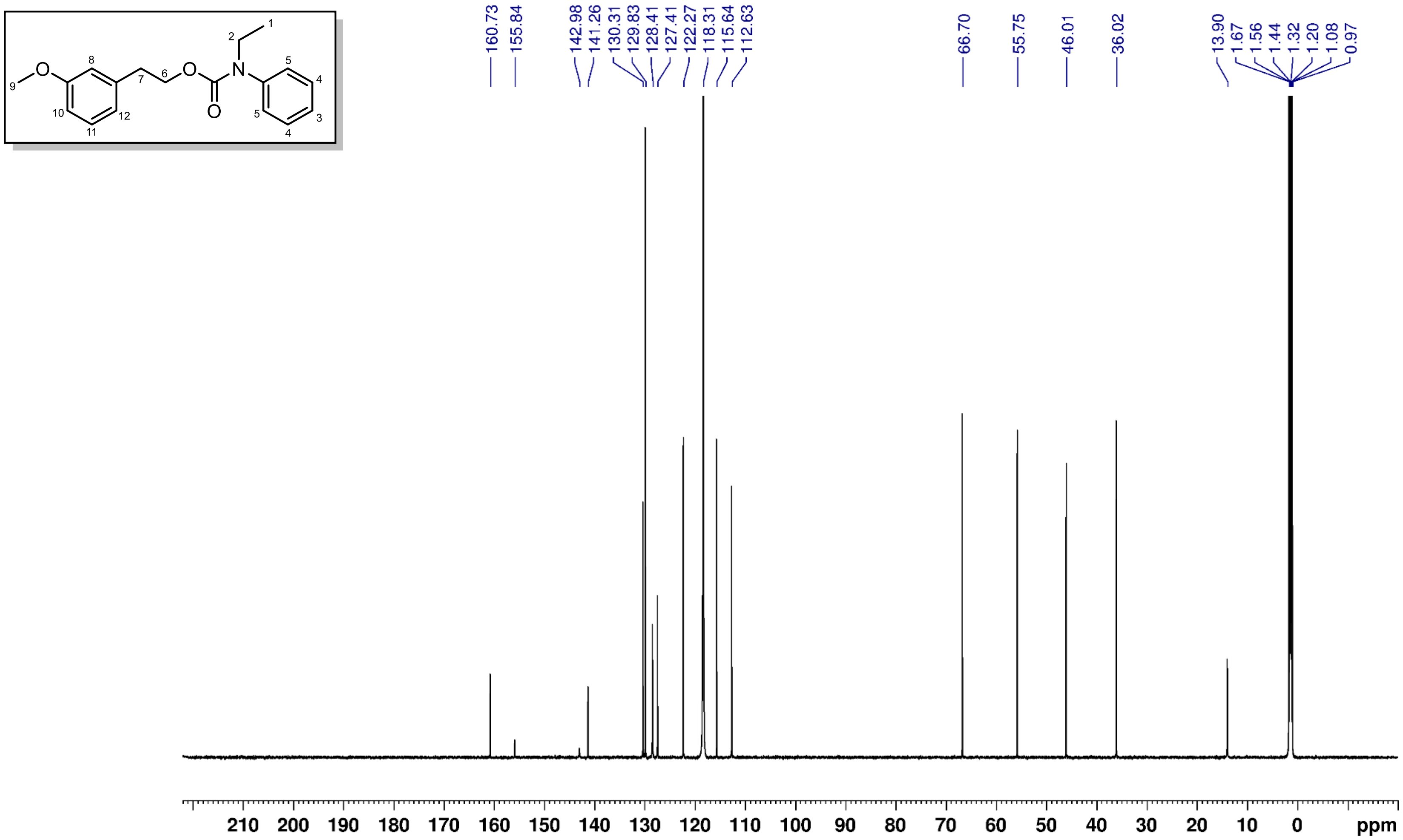

^{1}H NMR (700 MHz, CD_3CN) for 3-methylphenethyl ethyl(phenyl)carbamate (**10i**)

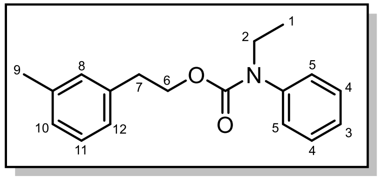

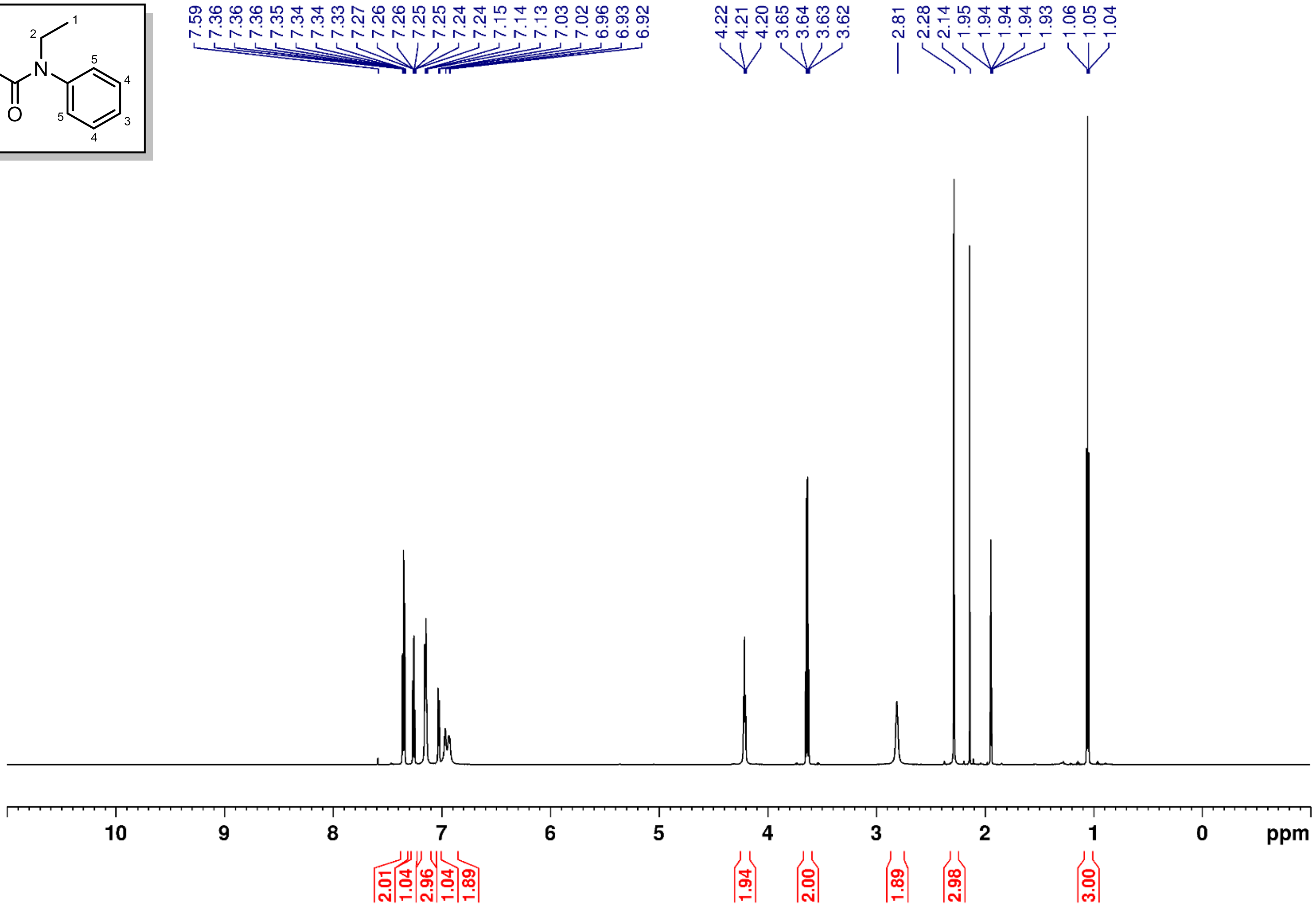

^{13}C NMR (176 MHz, CD_3CN) for 3-methylphenethyl ethyl(phenyl)carbamate (**10i**)

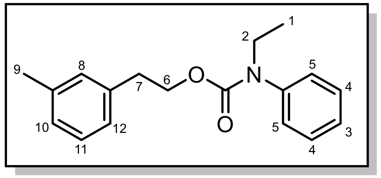

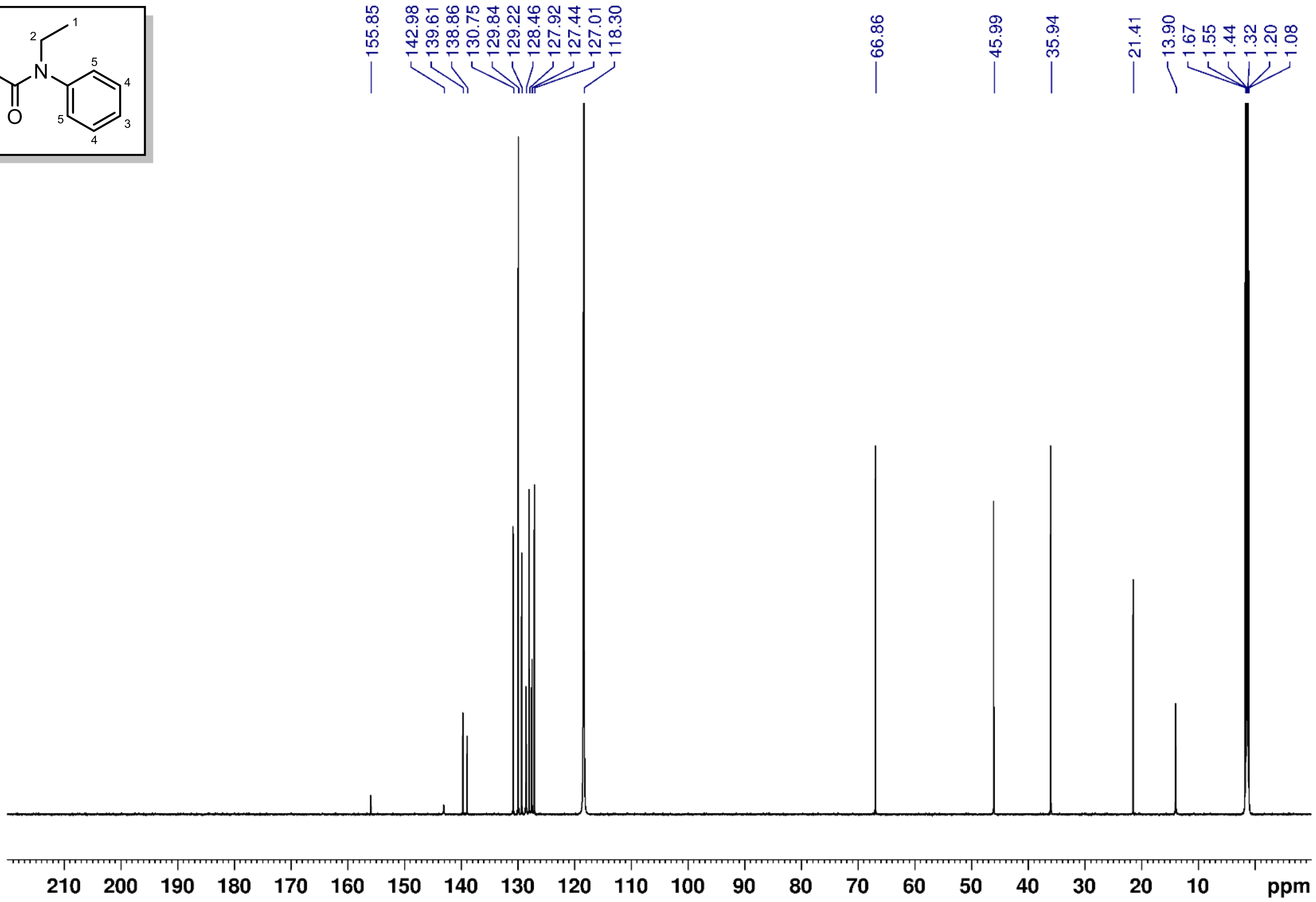

^{1}H NMR (700 MHz, CD_3CN) for 3-chlorophenethyl ethyl(phenyl)carbamate (**10j**)

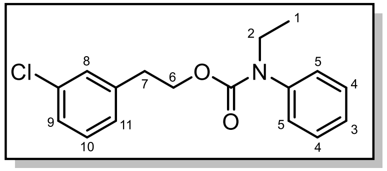

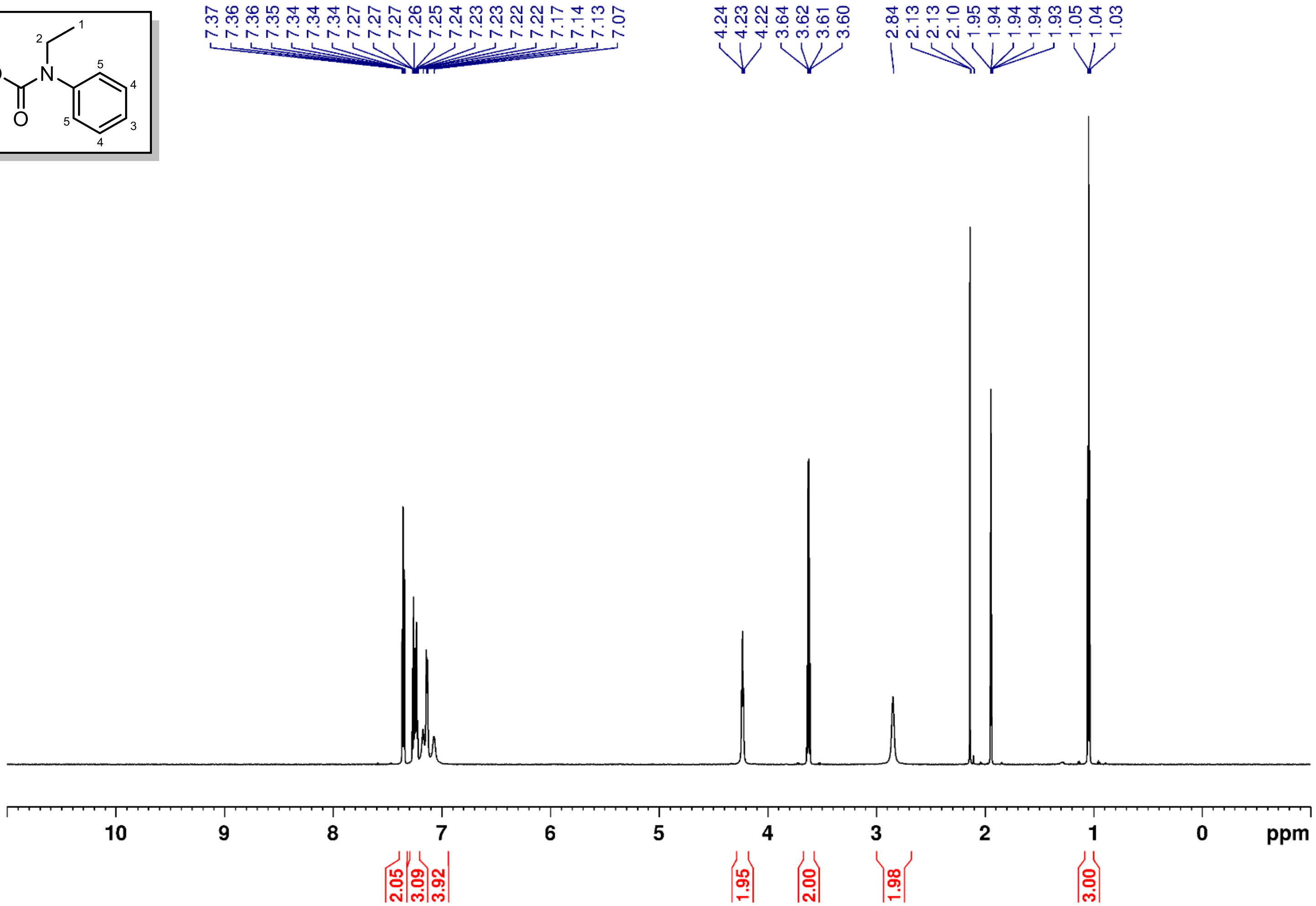

^{13}C NMR (176 MHz, CD_3CN) for 3-chlorophenethyl ethyl(phenyl)carbamate (**10j**)

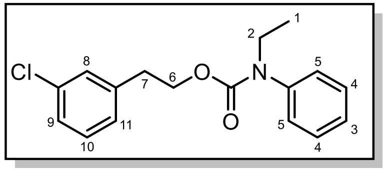

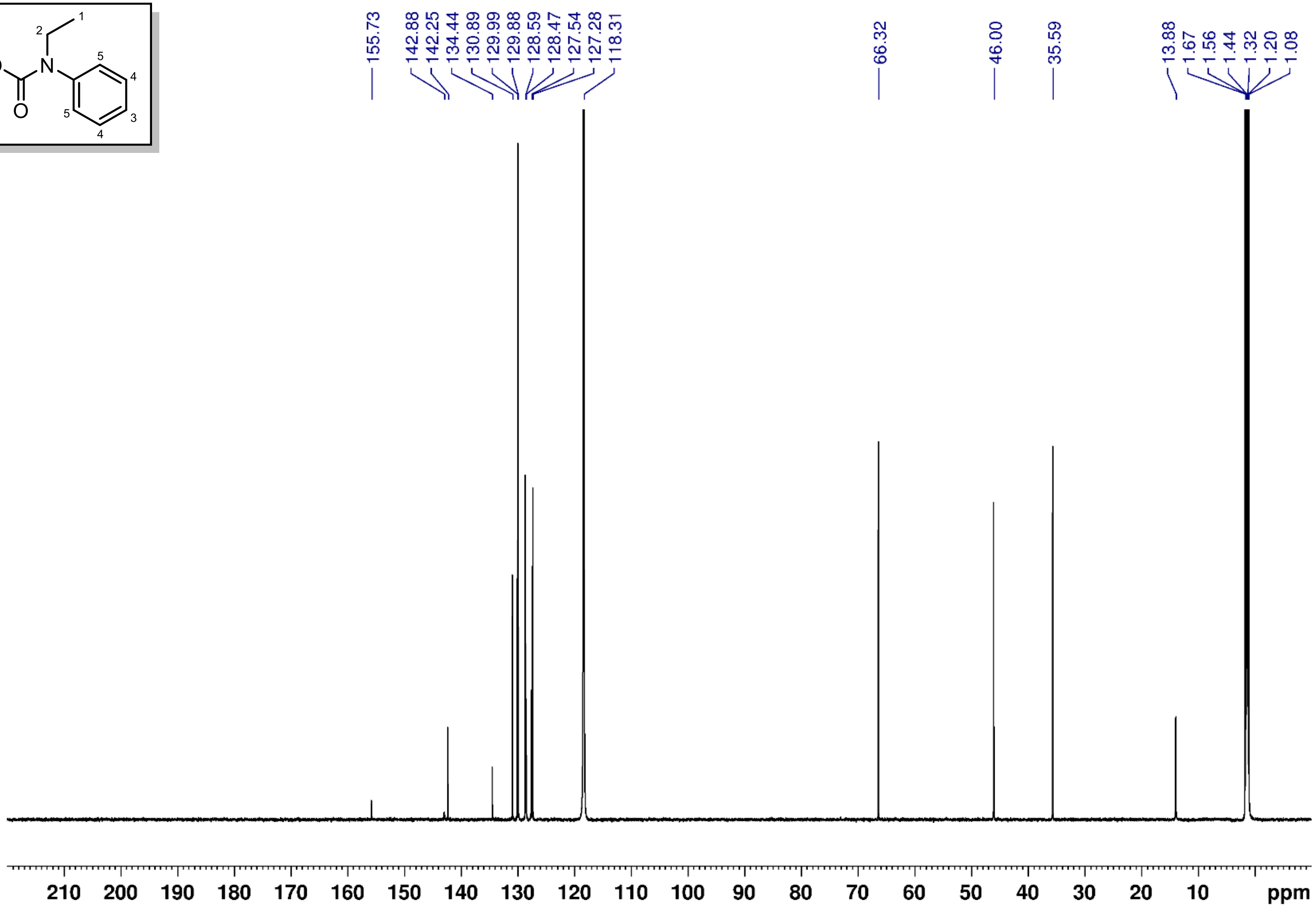

^{1}H NMR (700 MHz, CD_3CN) for 3-bromophenethyl ethyl(phenyl)carbamate (**10k**)

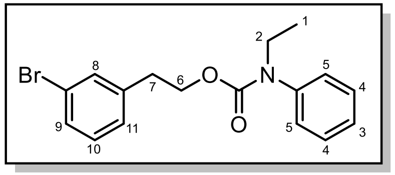

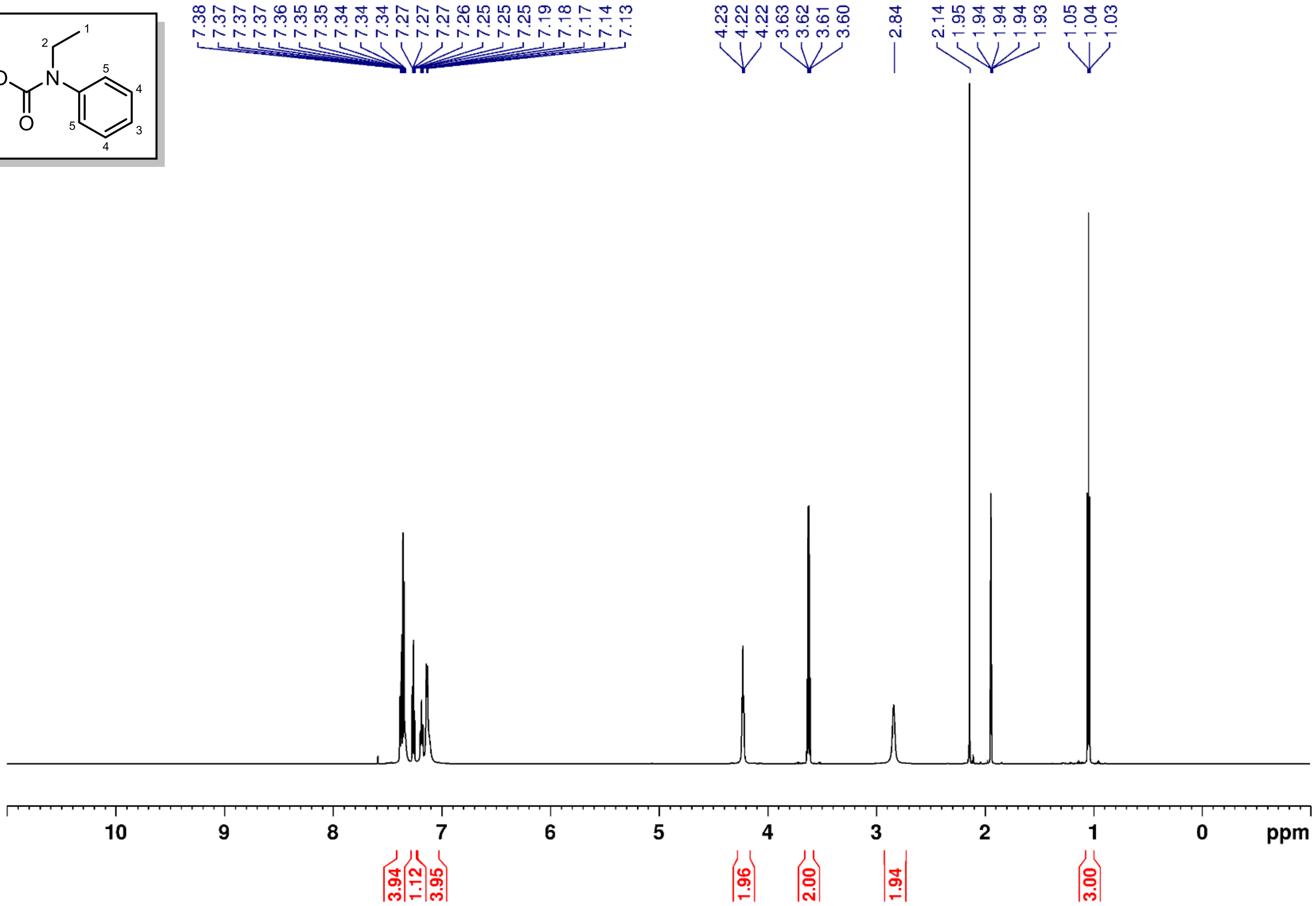

^{13}C NMR (176 MHz, CD_3CN) for 3-bromophenethyl ethyl(phenyl)carbamate (**10k**)

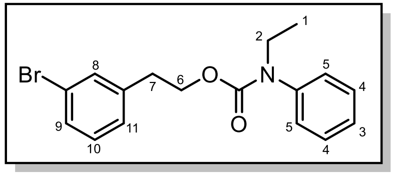

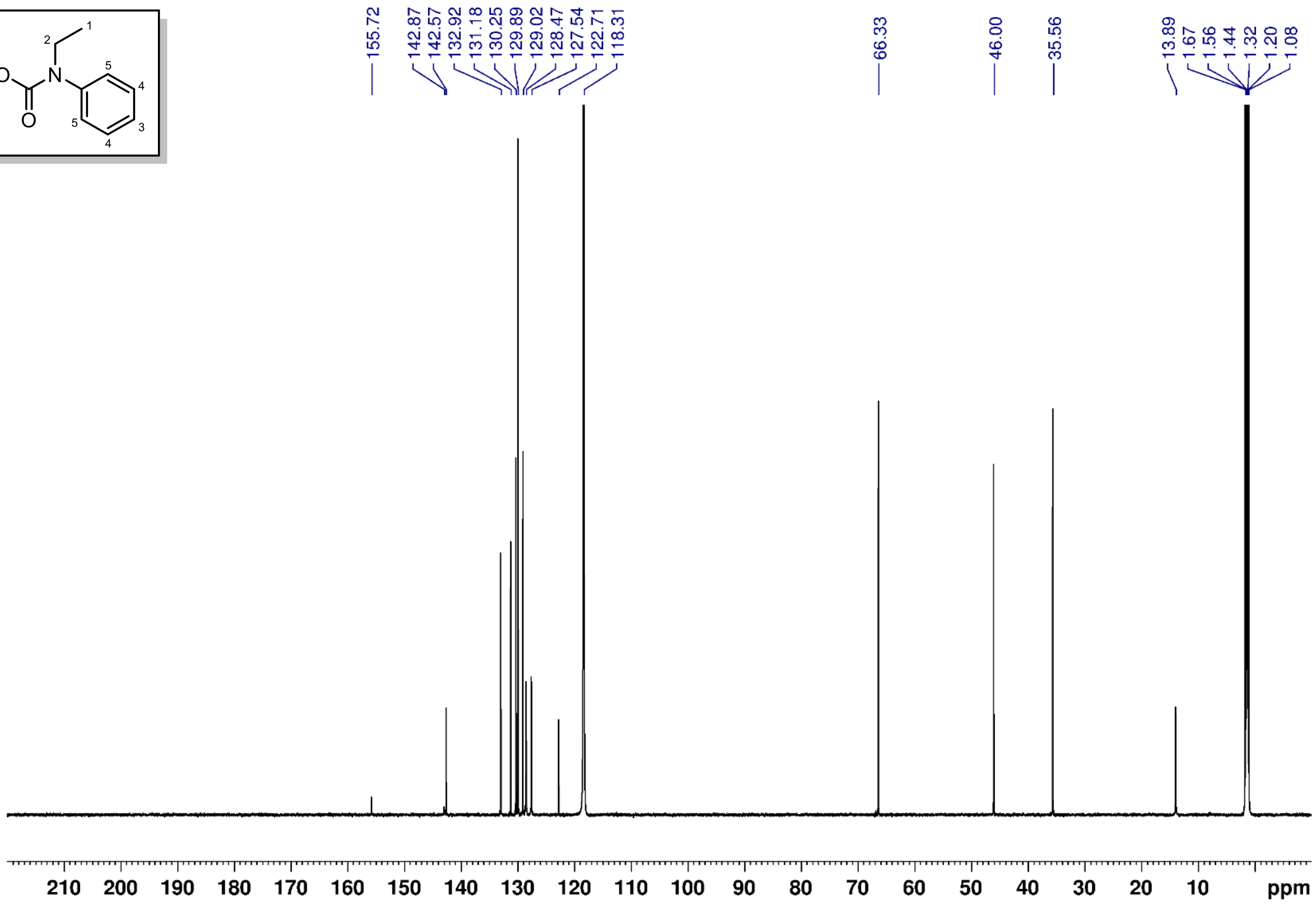

^{1}H NMR (700 MHz, CD_3CN) for 3-(trifluoromethyl)phenethyl ethyl(phenyl)carbamate (**10l**)

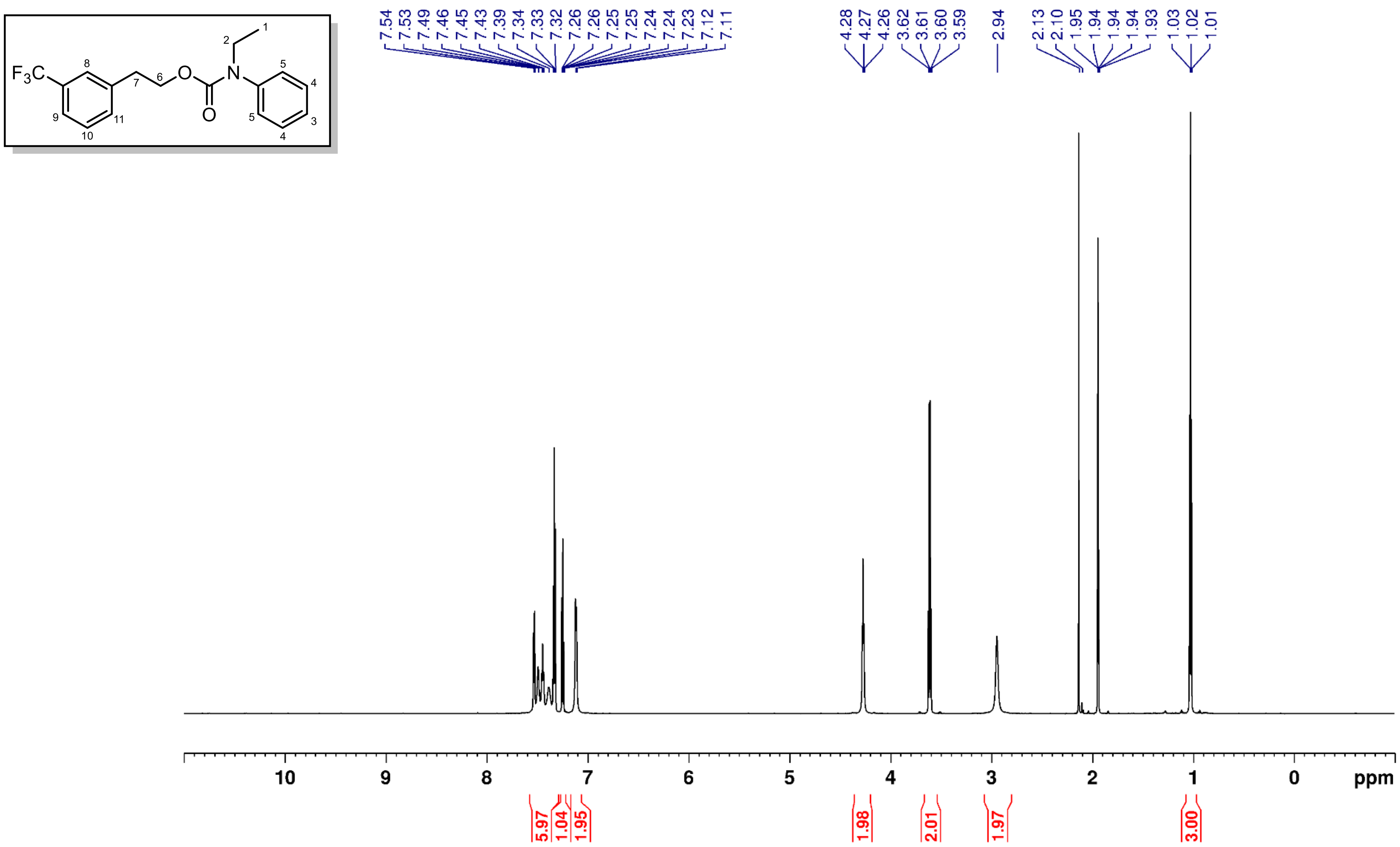

^{13}**C NMR (176 MHz, CD$_3$CN)** for 3-(trifluoromethyl)phenethyl ethyl(phenyl)carbamate (**10l**)

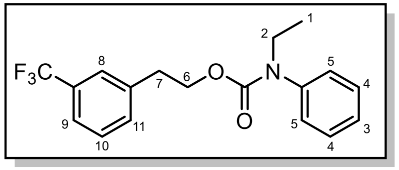

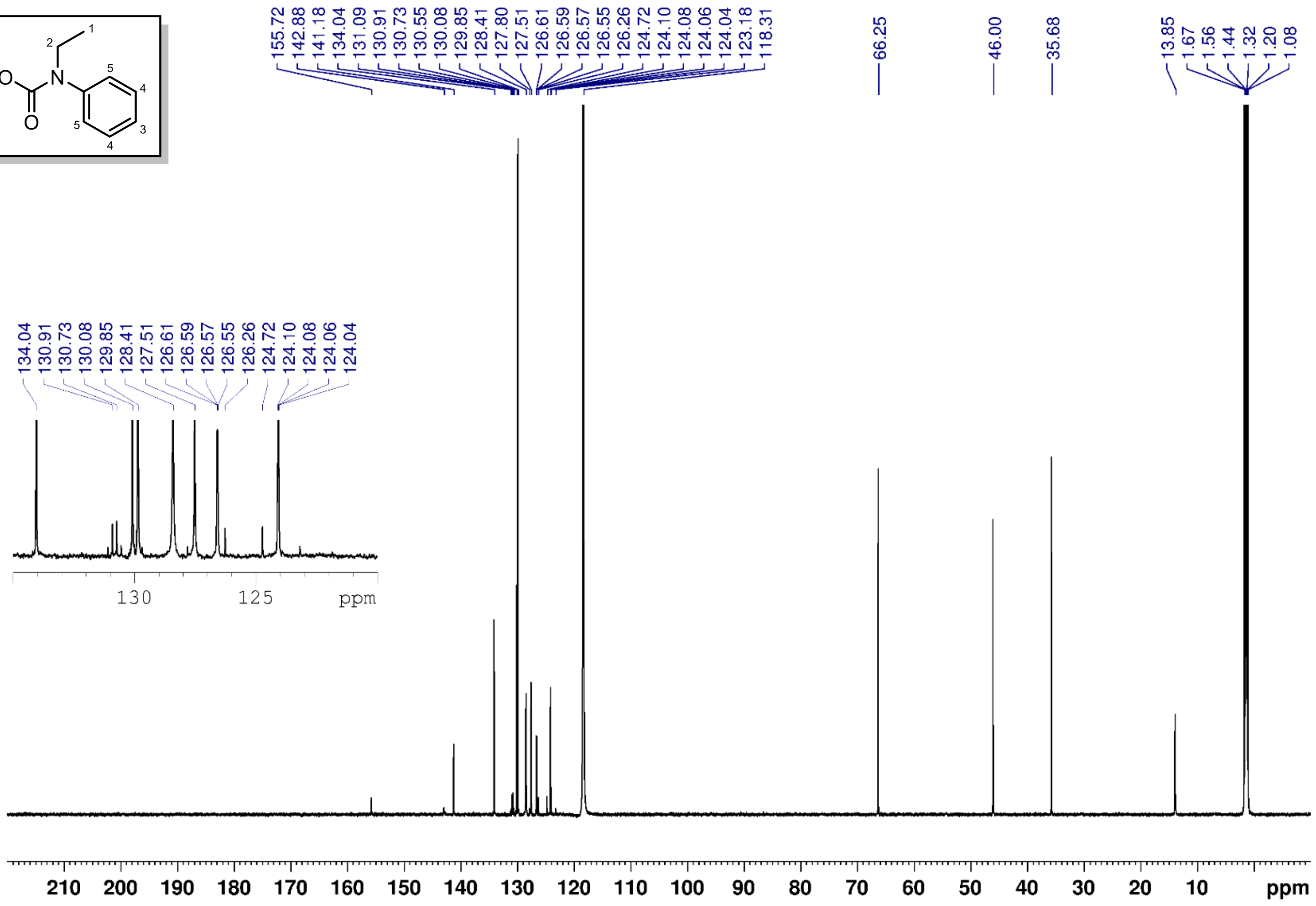

^{19}F NMR (376 MHz, CD_3CN) for 3-(trifluoromethyl)phenethyl ethyl(phenyl)carbamate (**10l**)

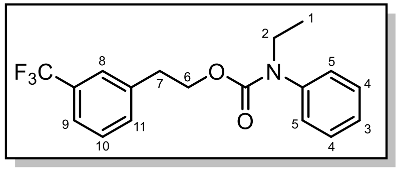

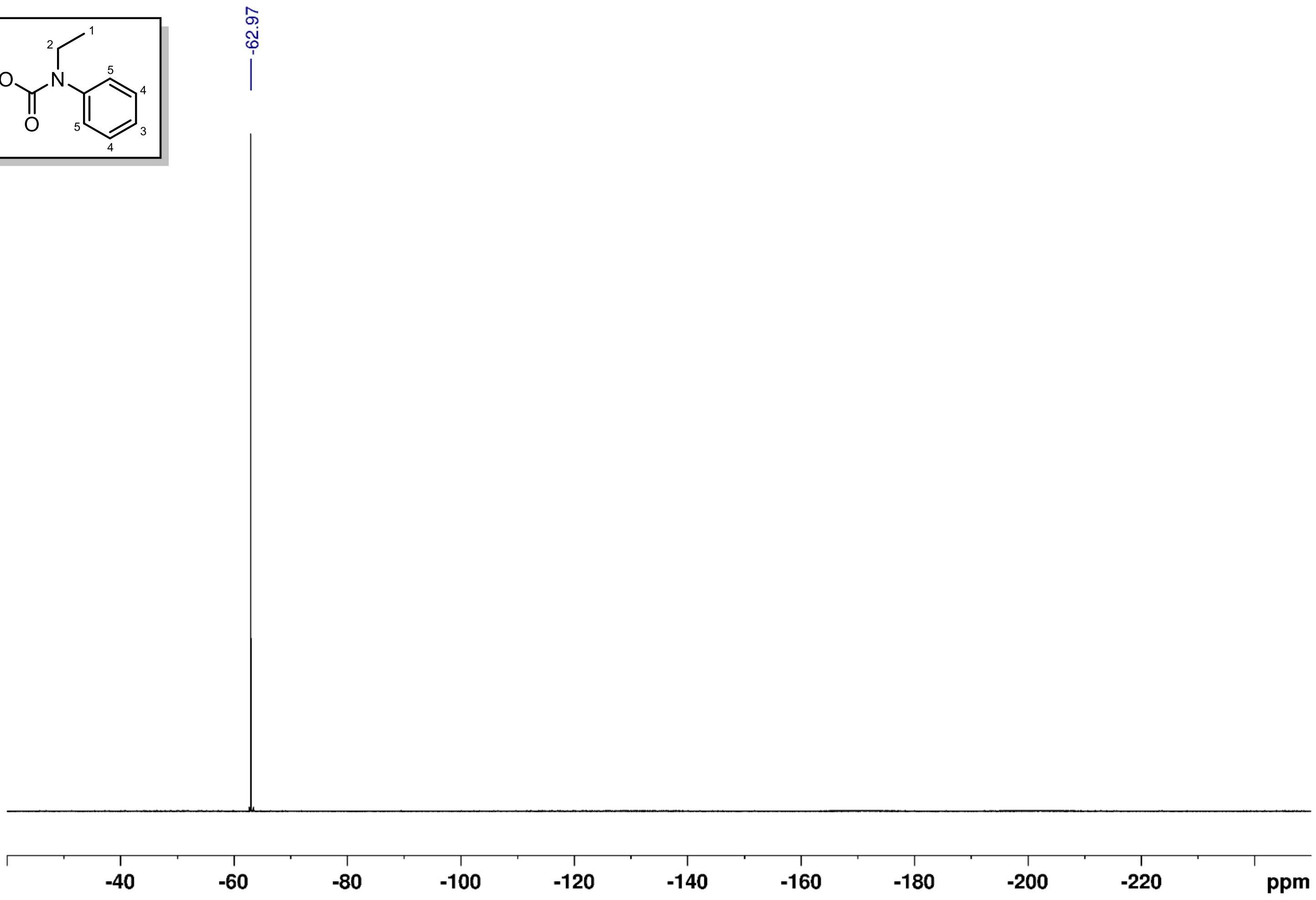

^{1}H NMR (700 MHz, CD_3CN) for 2-methylphenethyl ethyl(phenyl)carbamate (**10m**)

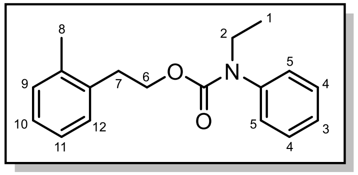

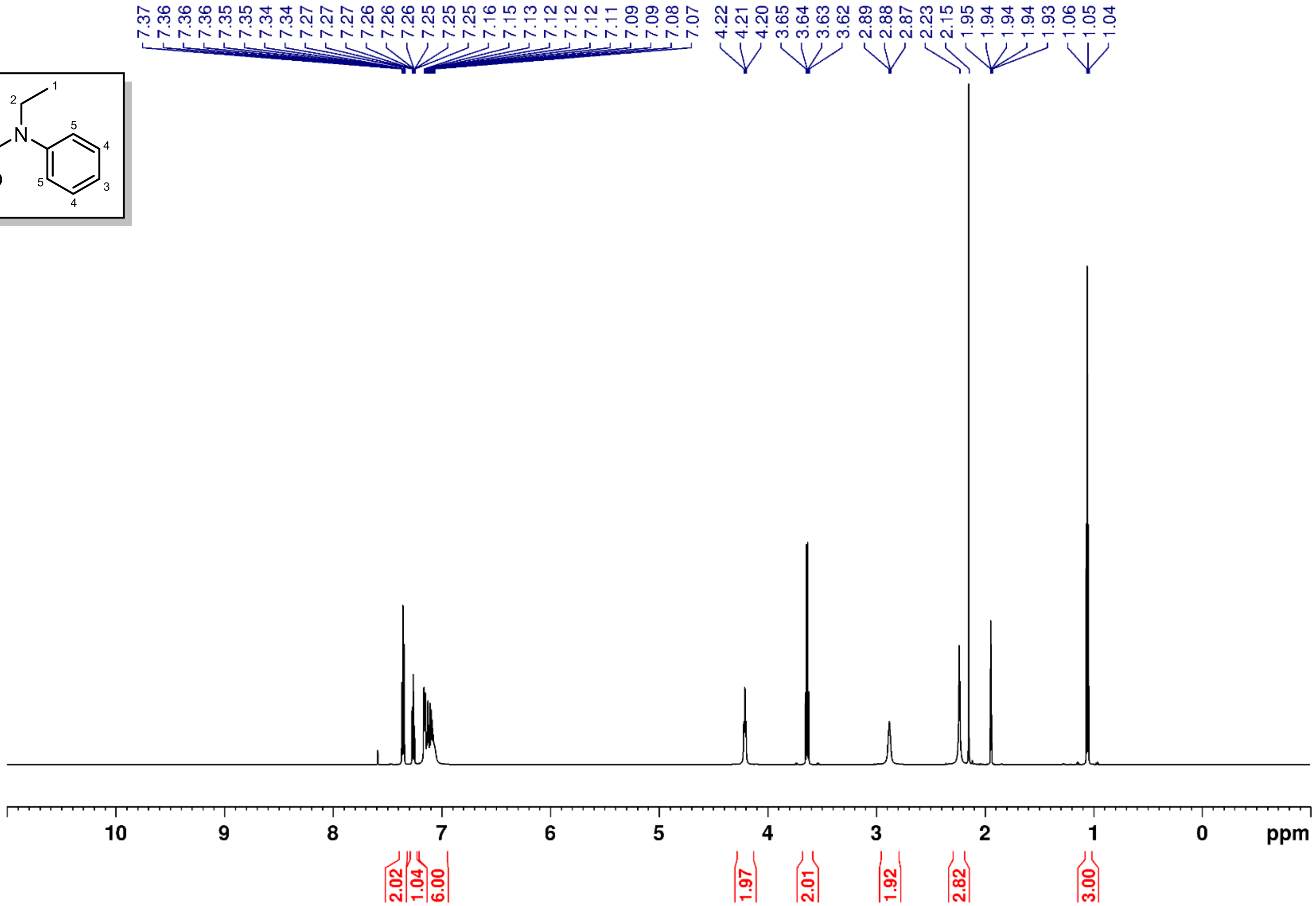

^{13}C NMR (176 MHz, CD_3CN) for 2-methylphenethyl ethyl(phenyl)carbamate (**10m**)

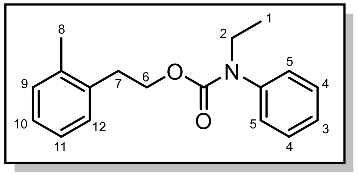

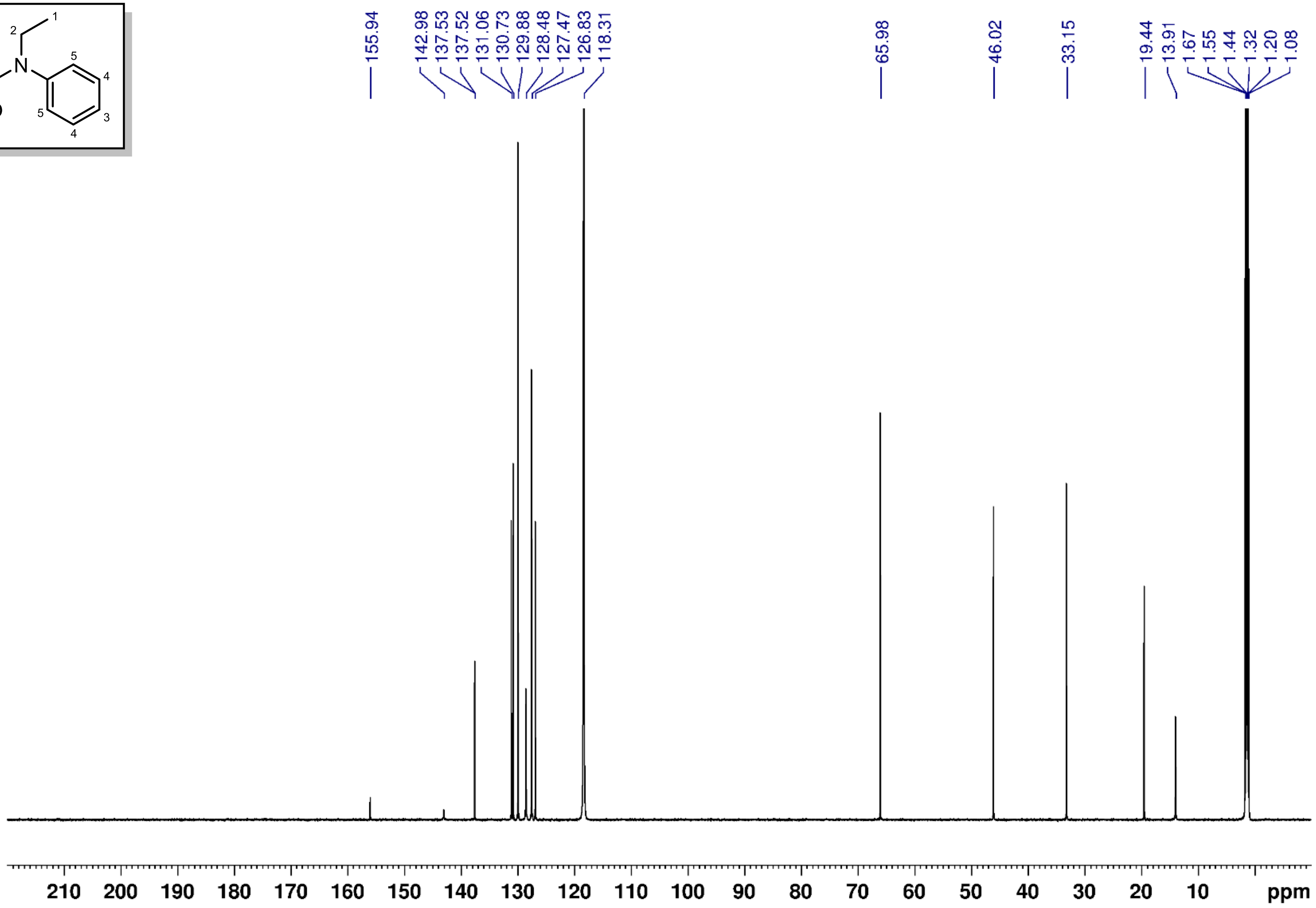

^{1}H NMR (700 MHz, CD_3CN) for 2-chlorophenethyl ethyl(phenyl)carbamate (**10n**)

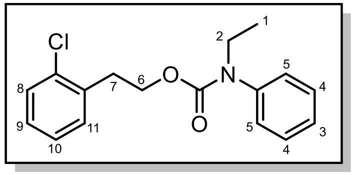

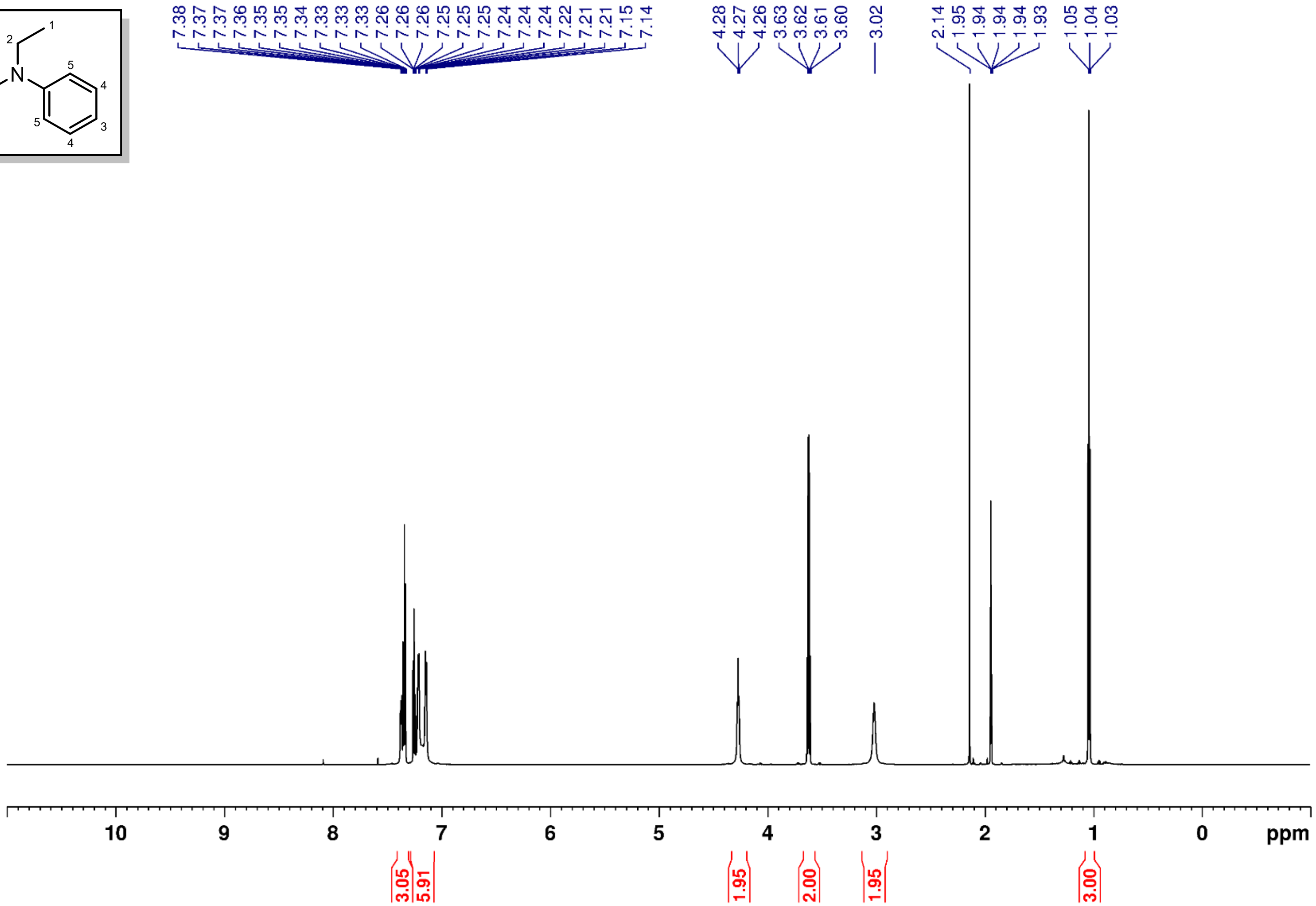

^{13}C NMR (176 MHz, CD_3CN) for 2-chlorophenethyl ethyl(phenyl)carbamate (**10n**)

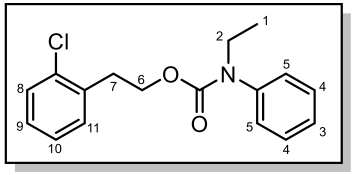

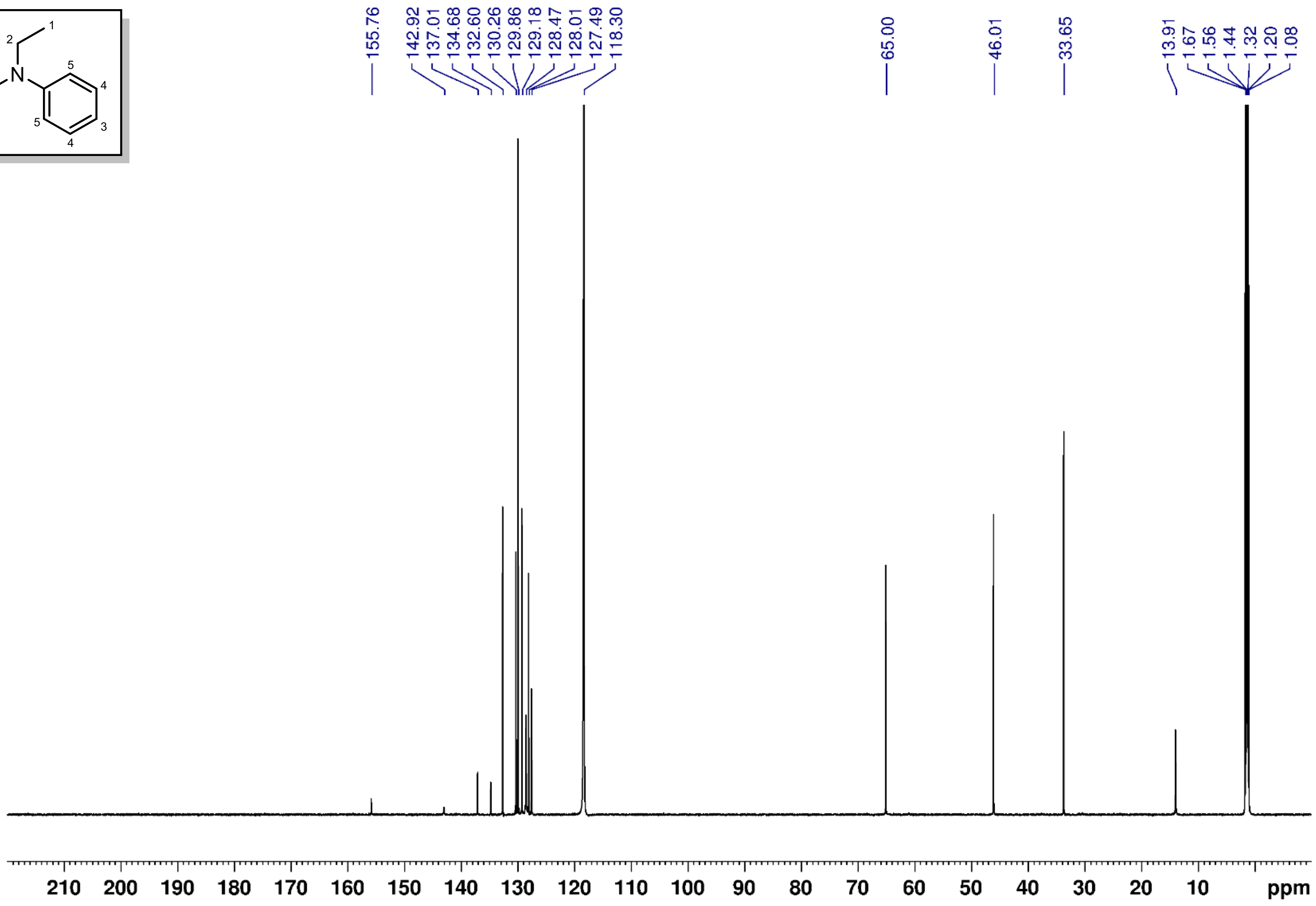

^{1}H NMR (700 MHz, CD_3CN) for 2-bromophenethyl ethyl(phenyl)carbamate (**10o**)

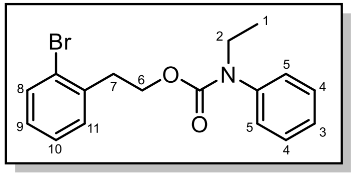

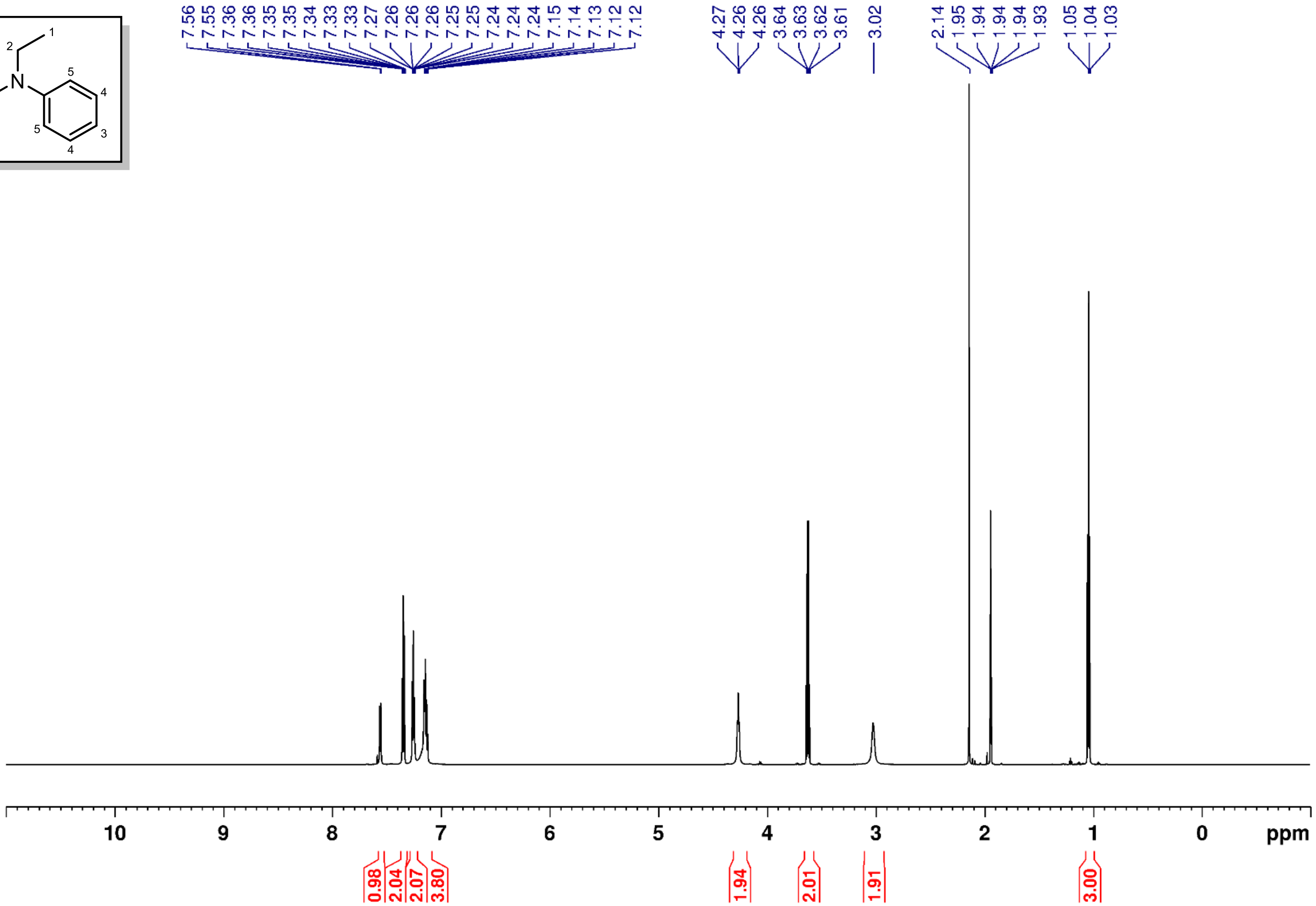

^{13}C NMR (176 MHz, CD_3CN) for 2-bromophenethyl ethyl(phenyl)carbamate (**10o**)

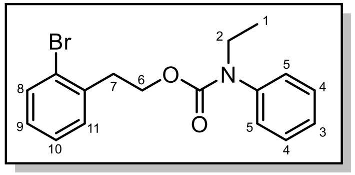

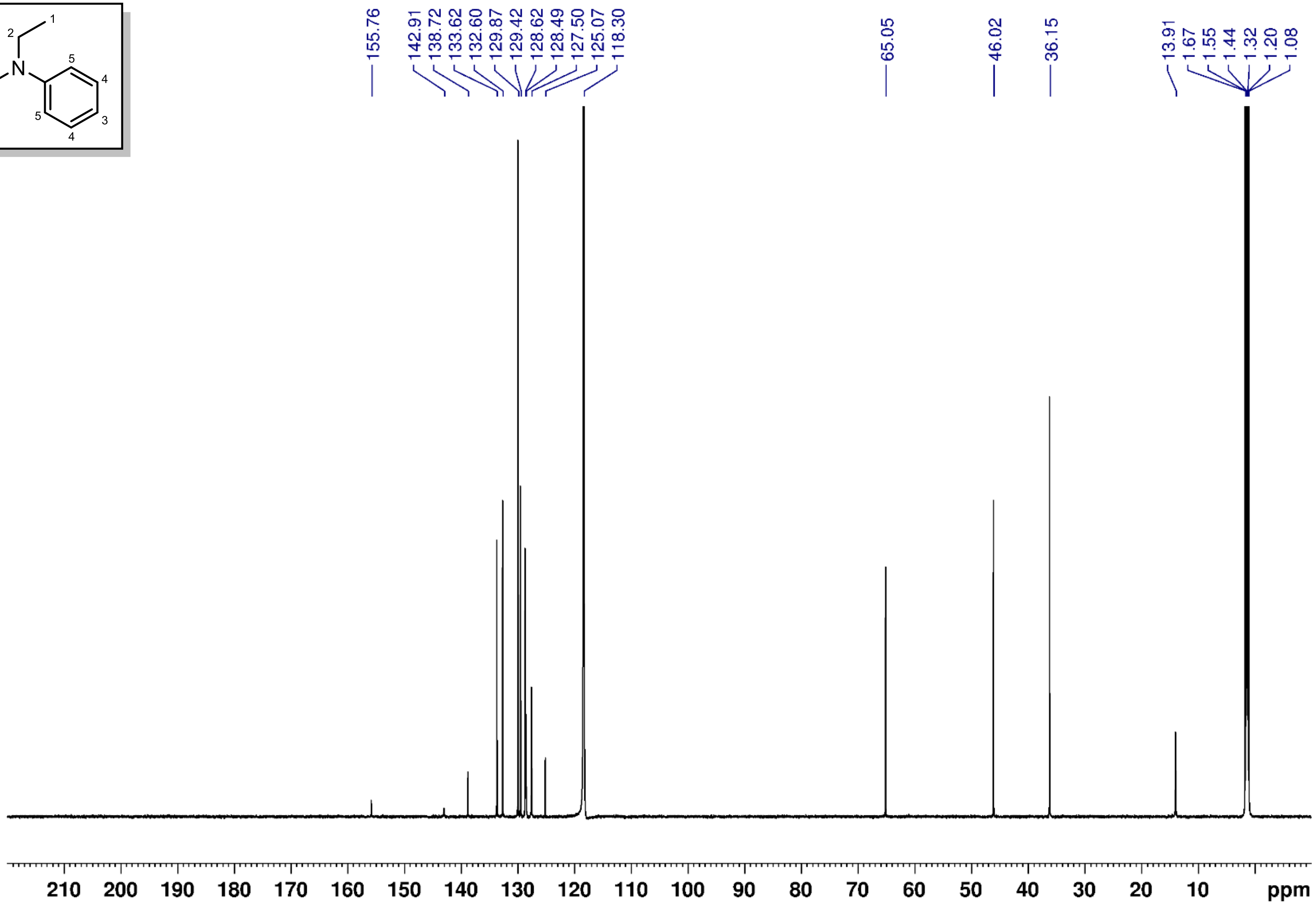

^{1}H NMR (700 MHz, CD_3CN) for 2-(thiophen-2-yl)ethyl ethyl(phenyl)carbamate (**10p**)

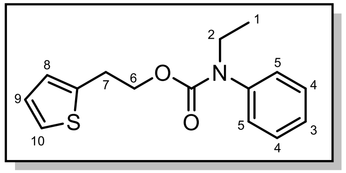

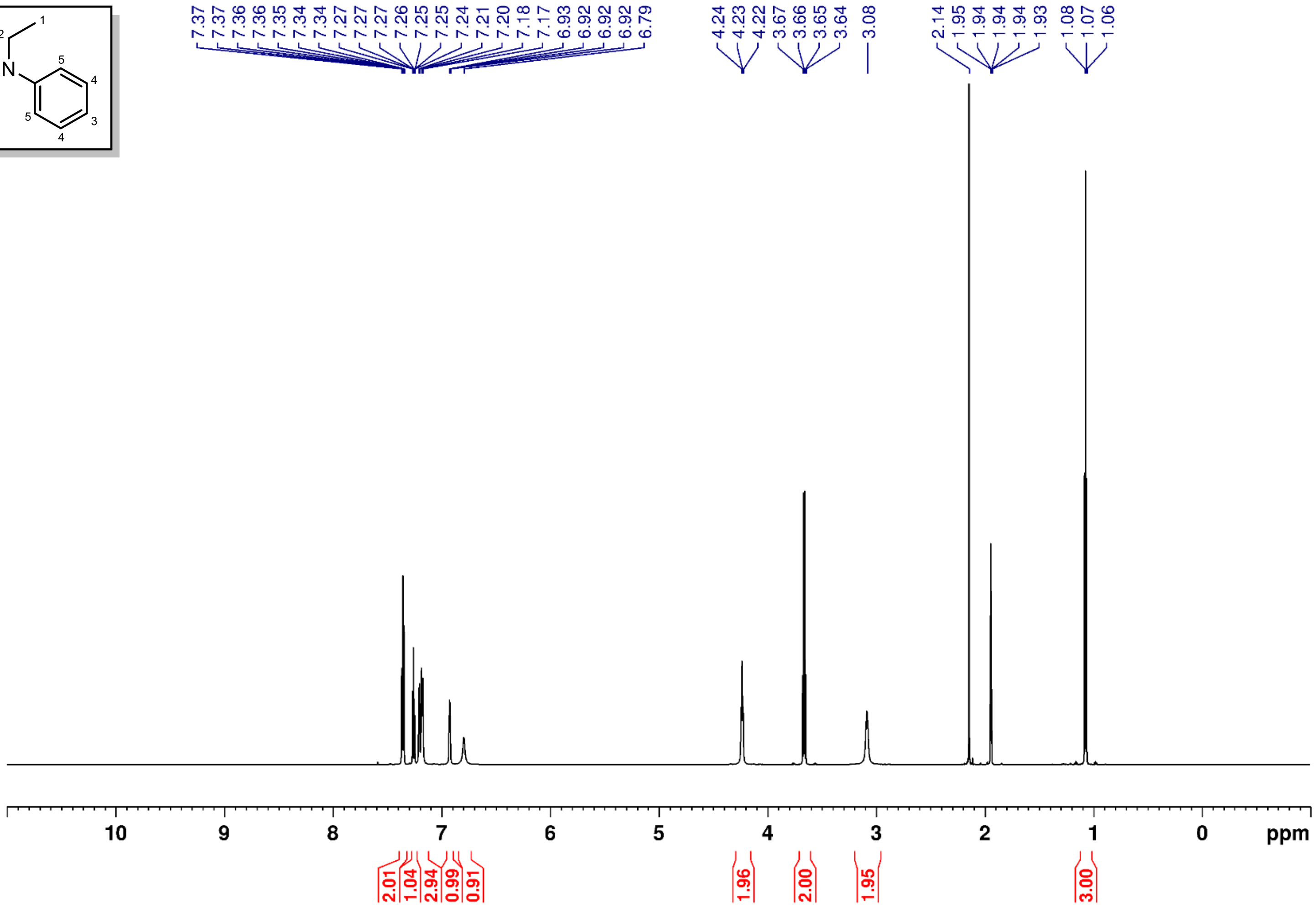

^{13}**C NMR (176 MHz, CD_3CN)** for 2-(thiophen-2-yl)ethyl ethyl(phenyl)carbamate (**10p**)

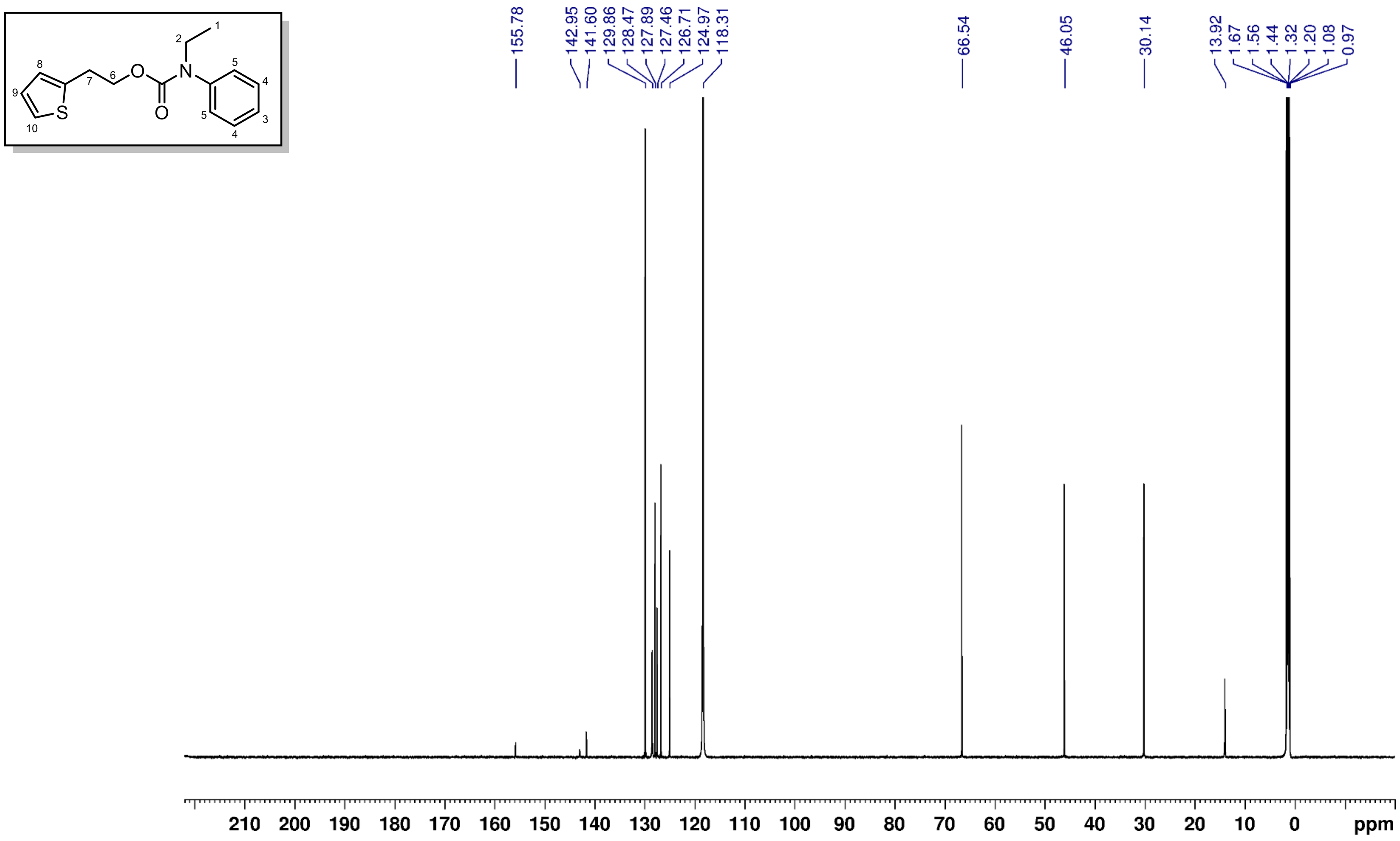

^{1}H NMR (700 MHz, CD_3CN) for 2-(thiophen-3-yl)ethyl ethyl(phenyl)carbamate (**10q**)

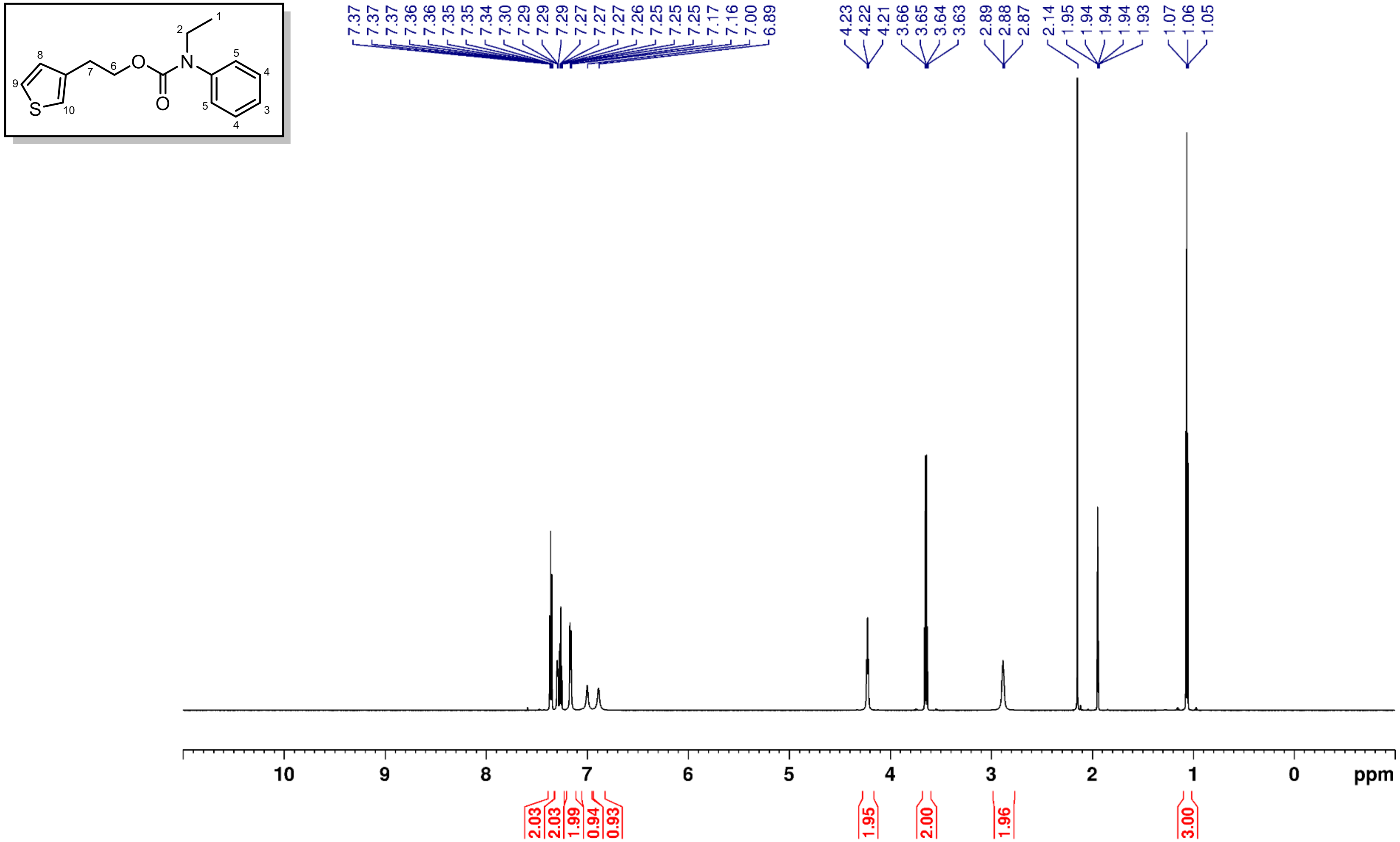

^{13}C NMR (176 MHz, CD_3CN) for 2-(thiophen-3-yl)ethyl ethyl(phenyl)carbamate (**10q**)

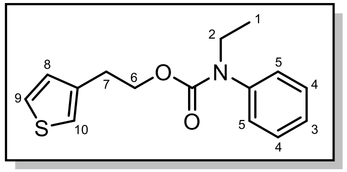

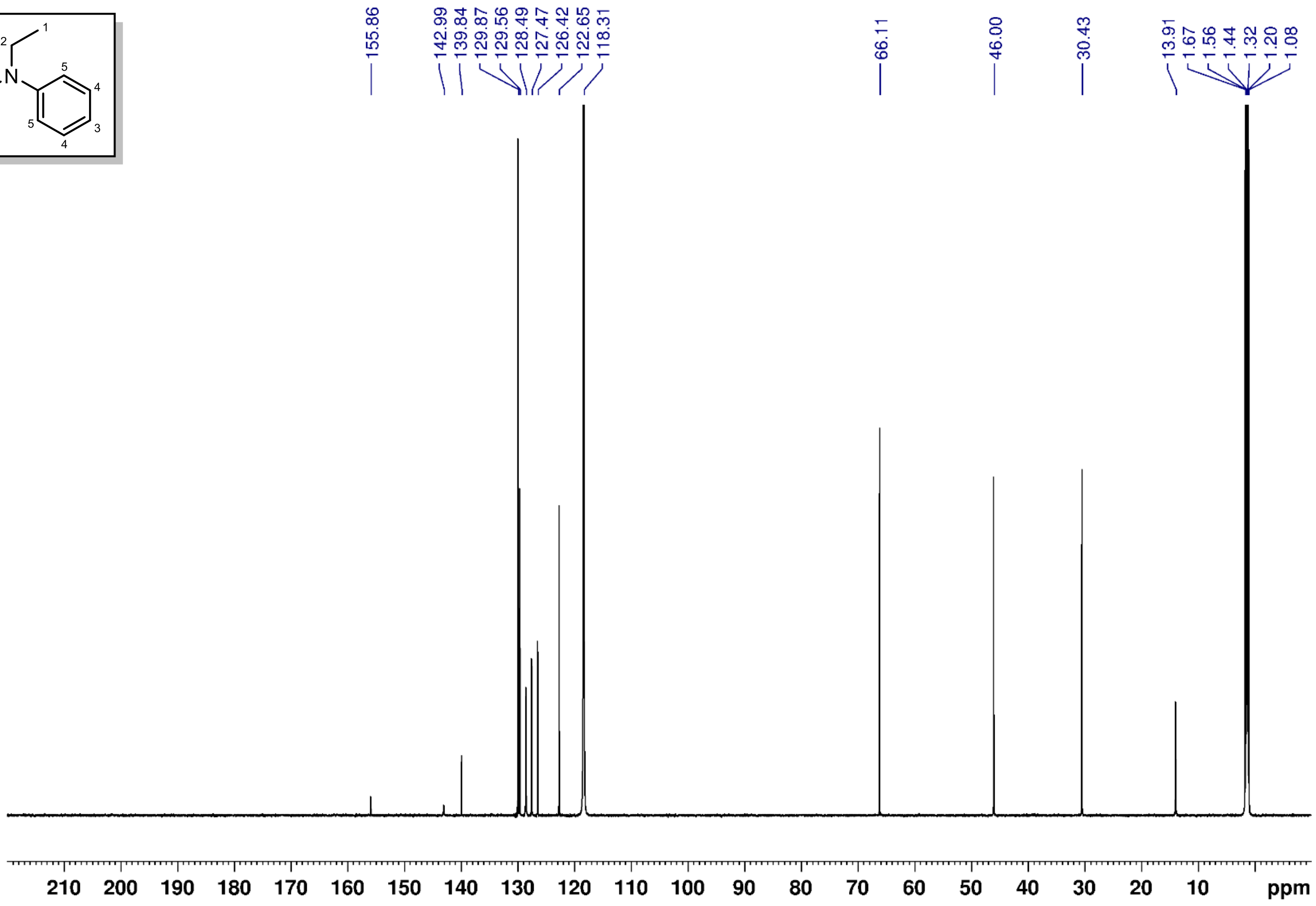

^{1}H NMR (500 MHz, CD_3CN) for 2,3-dihydro-1*H*-inden-2-yl ethyl(phenyl)carbamate (**10r**)

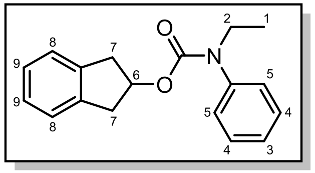

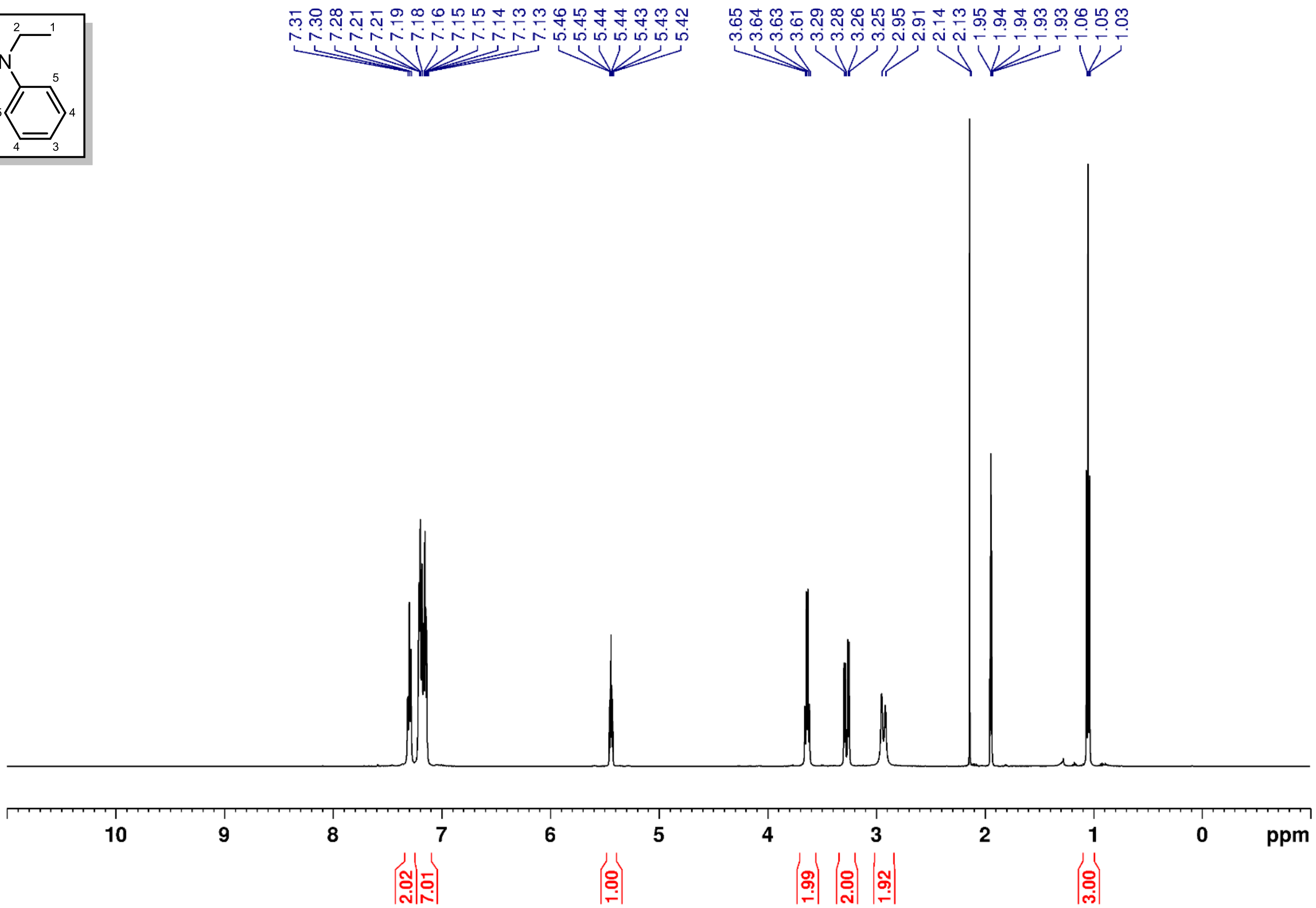

^{13}C NMR (126 MHz, CD_3CN) for 2,3-dihydro-1*H*-inden-2-yl ethyl(phenyl)carbamate (**10r**)

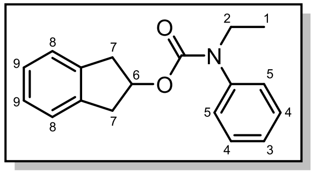

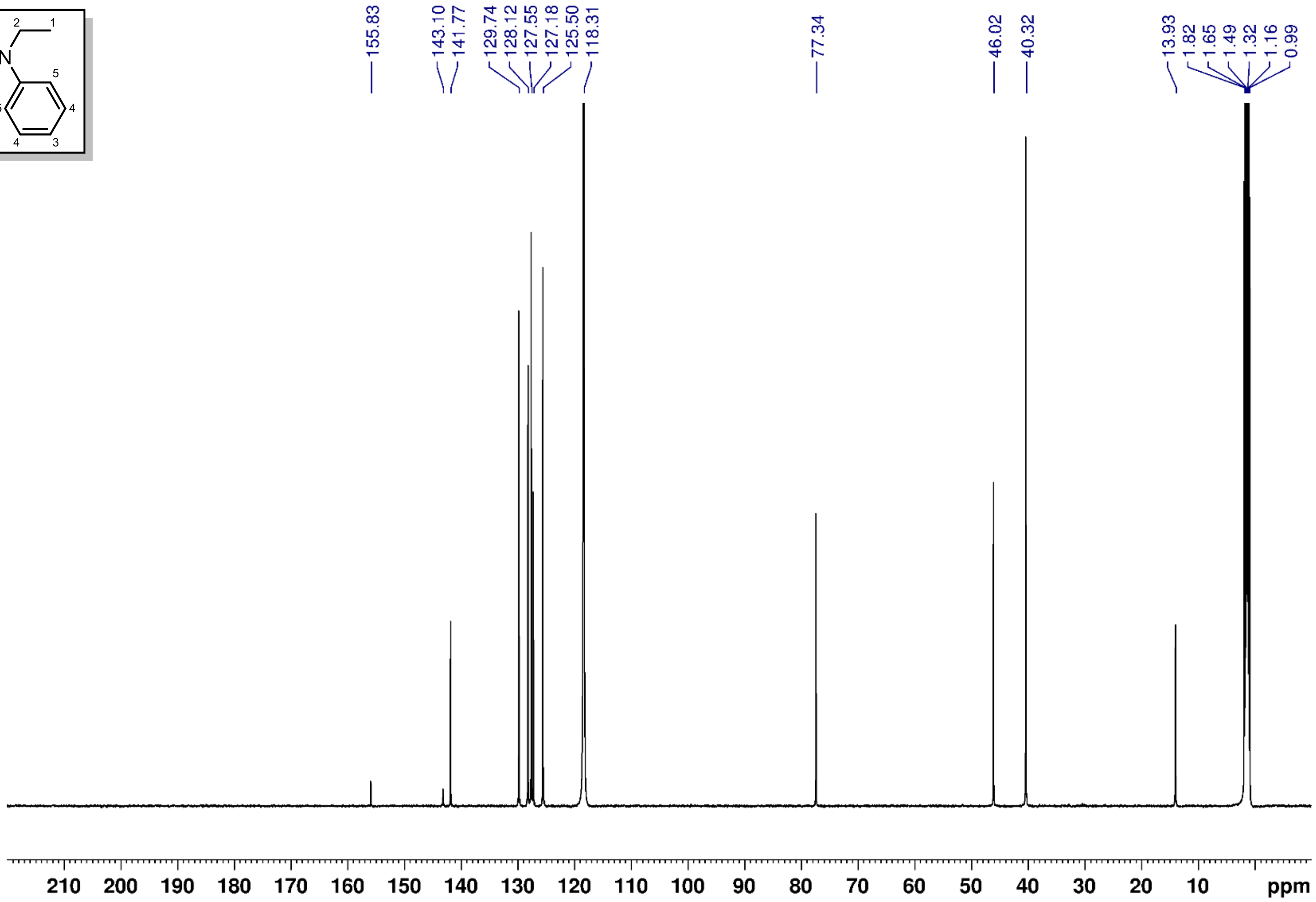

^{1}H NMR (700 MHz, CD_3CN) for 1-phenylpropan-2-yl ethyl(phenyl)carbamate (***(rac)*-10s**)

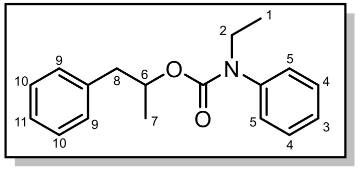

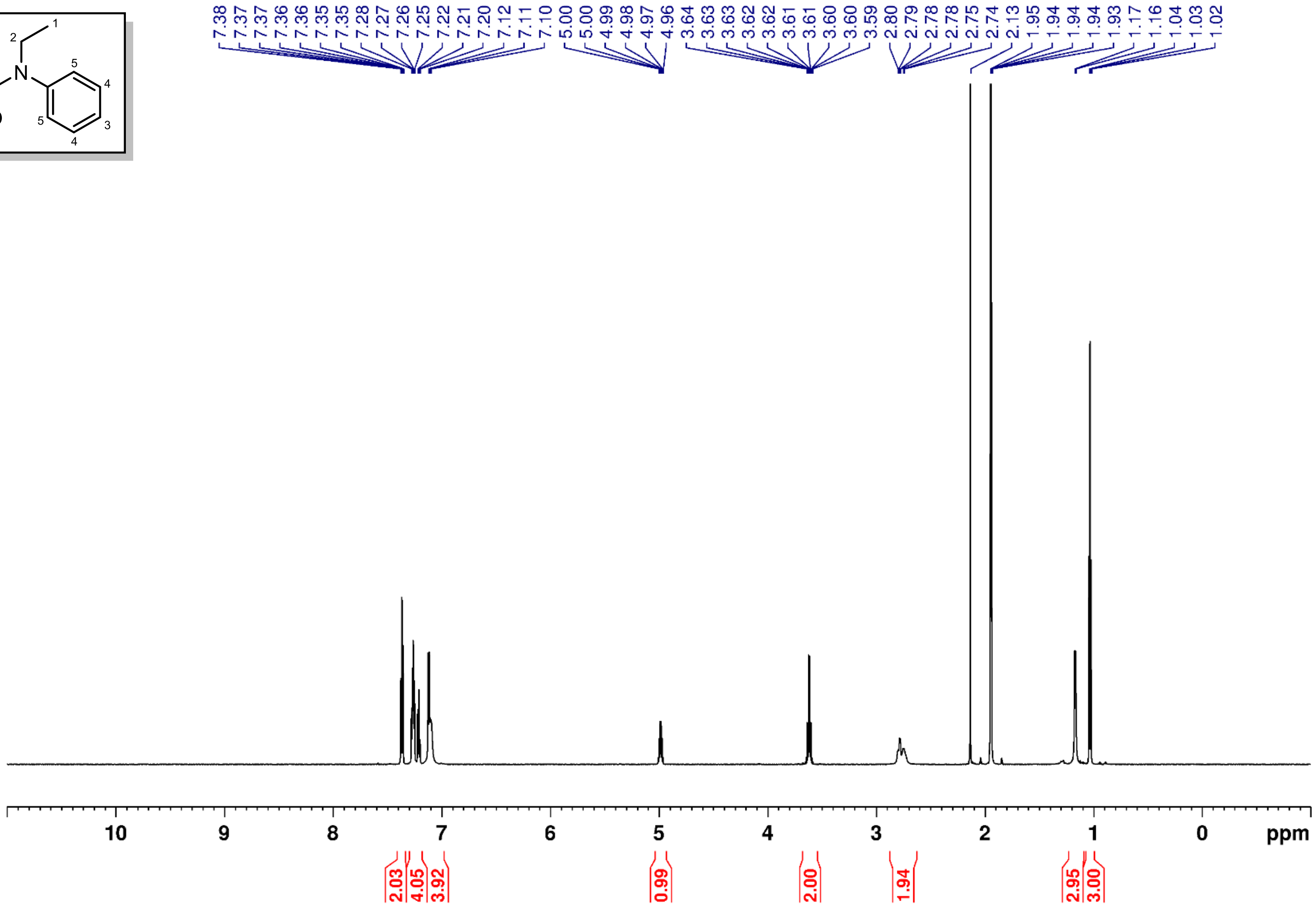

^{13}C NMR (176 MHz, CD_3CN) for 1-phenylpropan-2-yl ethyl(phenyl)carbamate (**(*rac*)-10s**)

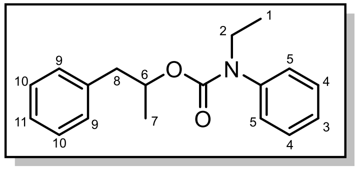

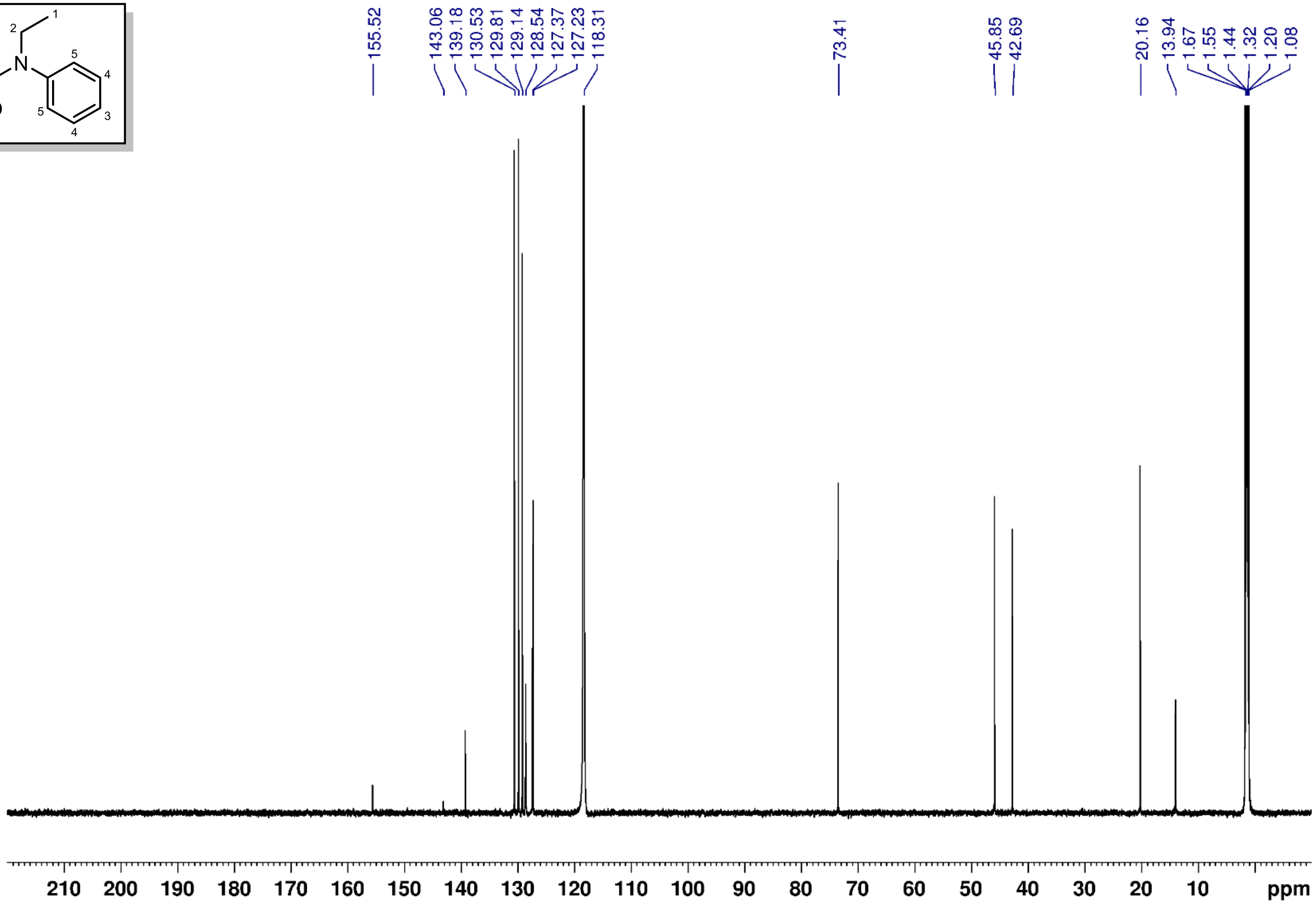

NMR Spectra for Enantioenriched Products – C-H Amination:

^{1}H NMR (700 MHz, CD_3CN) for 2,2,3,3,3-pentafluoropropyl (*S*)-(2-((ethyl(phenyl)carbamoyl)oxy)-1-phenylethyl)sulfamate (**11a**)

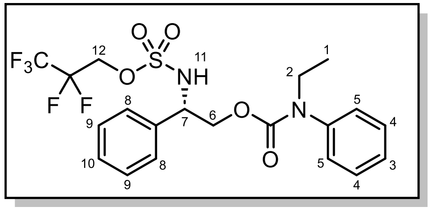

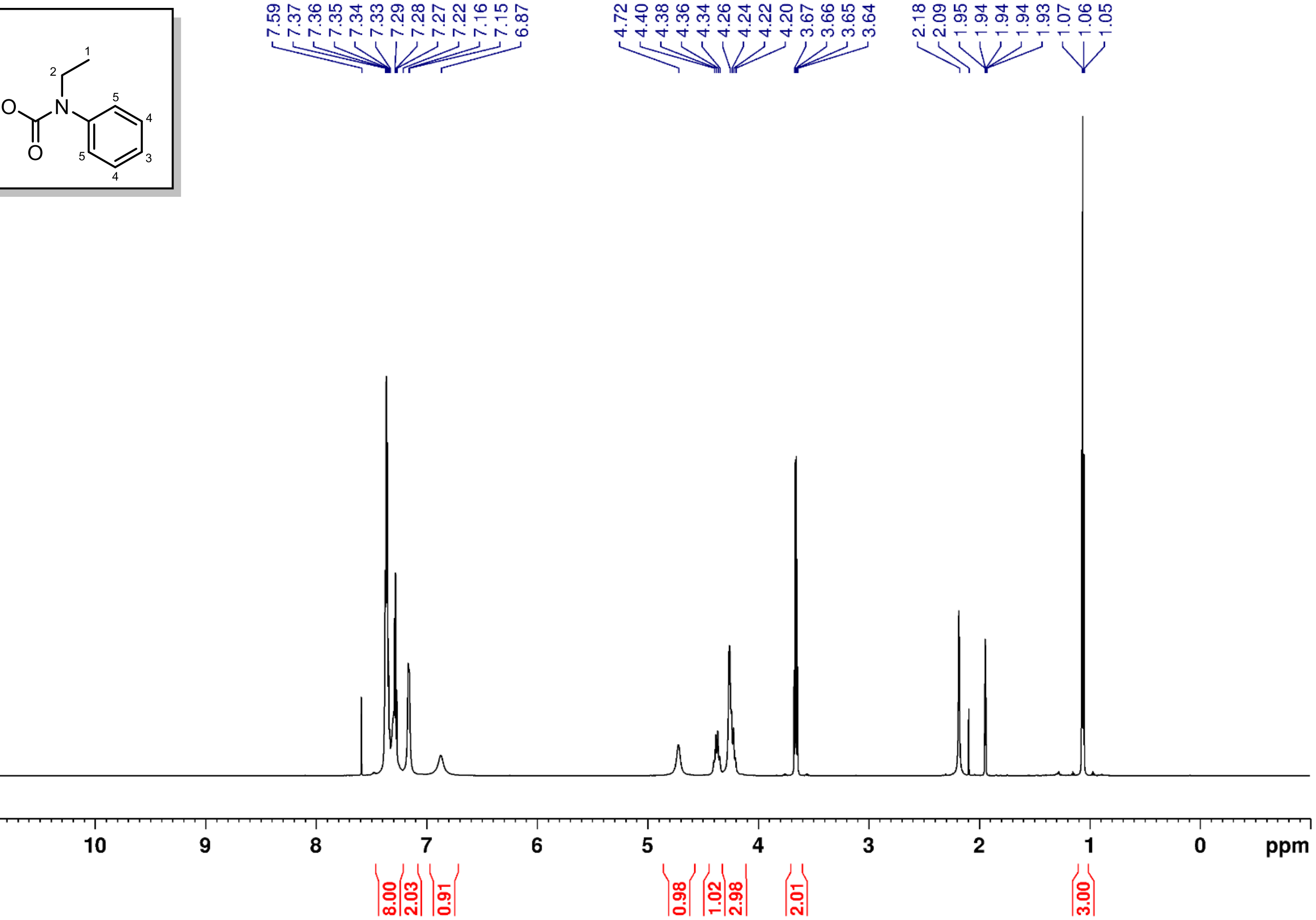

^{1}H NMR (700 MHz, CD_3CN) for 2,2,3,3,3-pentafluoropropyl (*R*)-(2-((ethyl(phenyl)carbamoyl)oxy)-1-phenylethyl)sulfamate (***ent*-11a**)

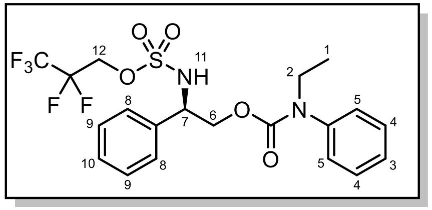

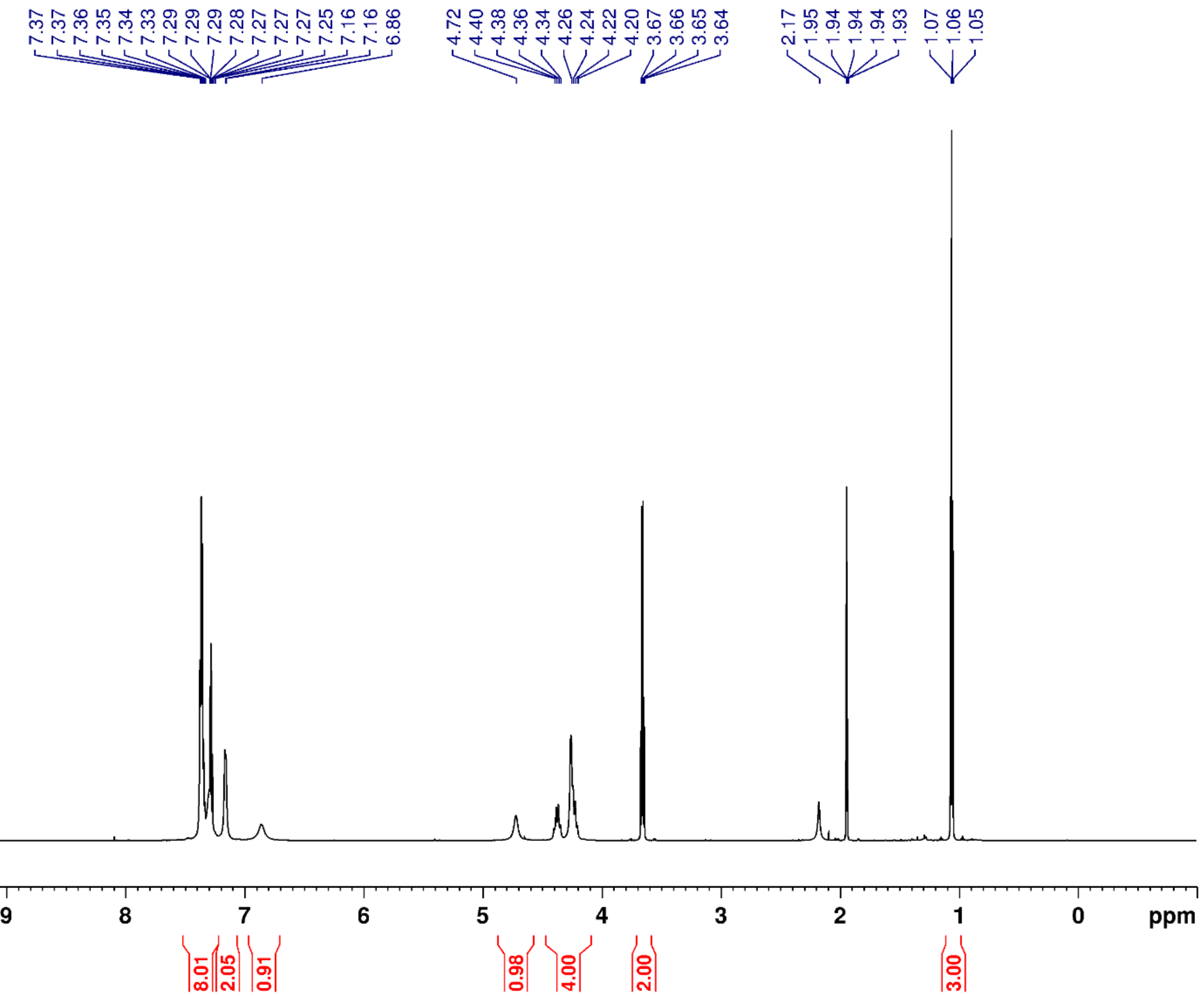

^{13}C NMR (176 MHz, CD_3CN) for 2,2,3,3,3-pentafluoropropyl (*S*)-(2-((ethyl(phenyl)carbamoyl)oxy)-1-phenylethyl)sulfamate (**11a**)

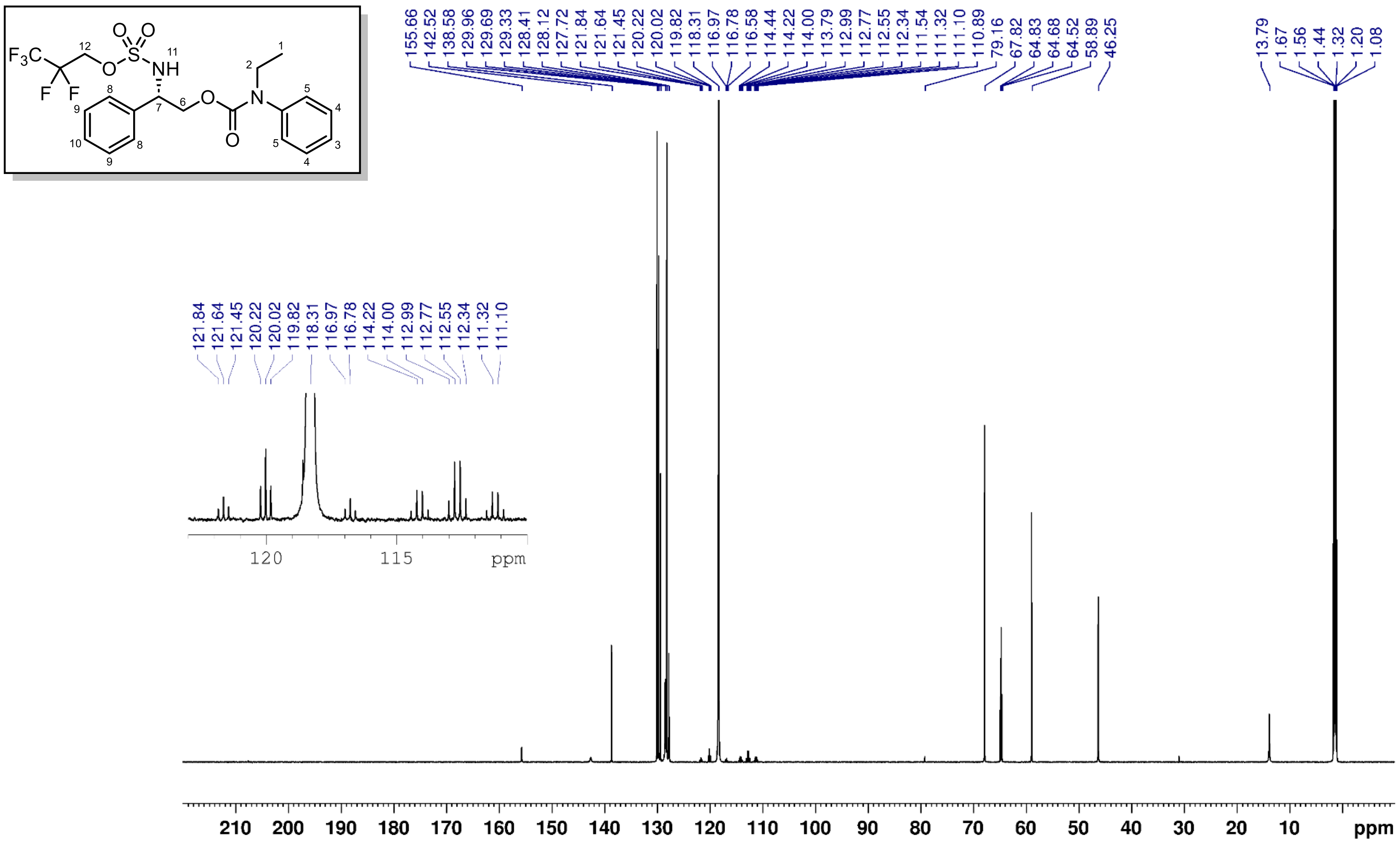

^{19}F NMR (376 MHz, CD_3CN) for 2,2,3,3,3-pentafluoropropyl (*S*)-(2-((ethyl(phenyl)carbamoyl)oxy)-1-phenylethyl)sulfamate (**11a**)

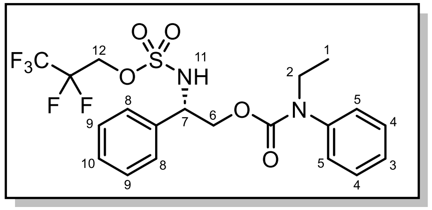

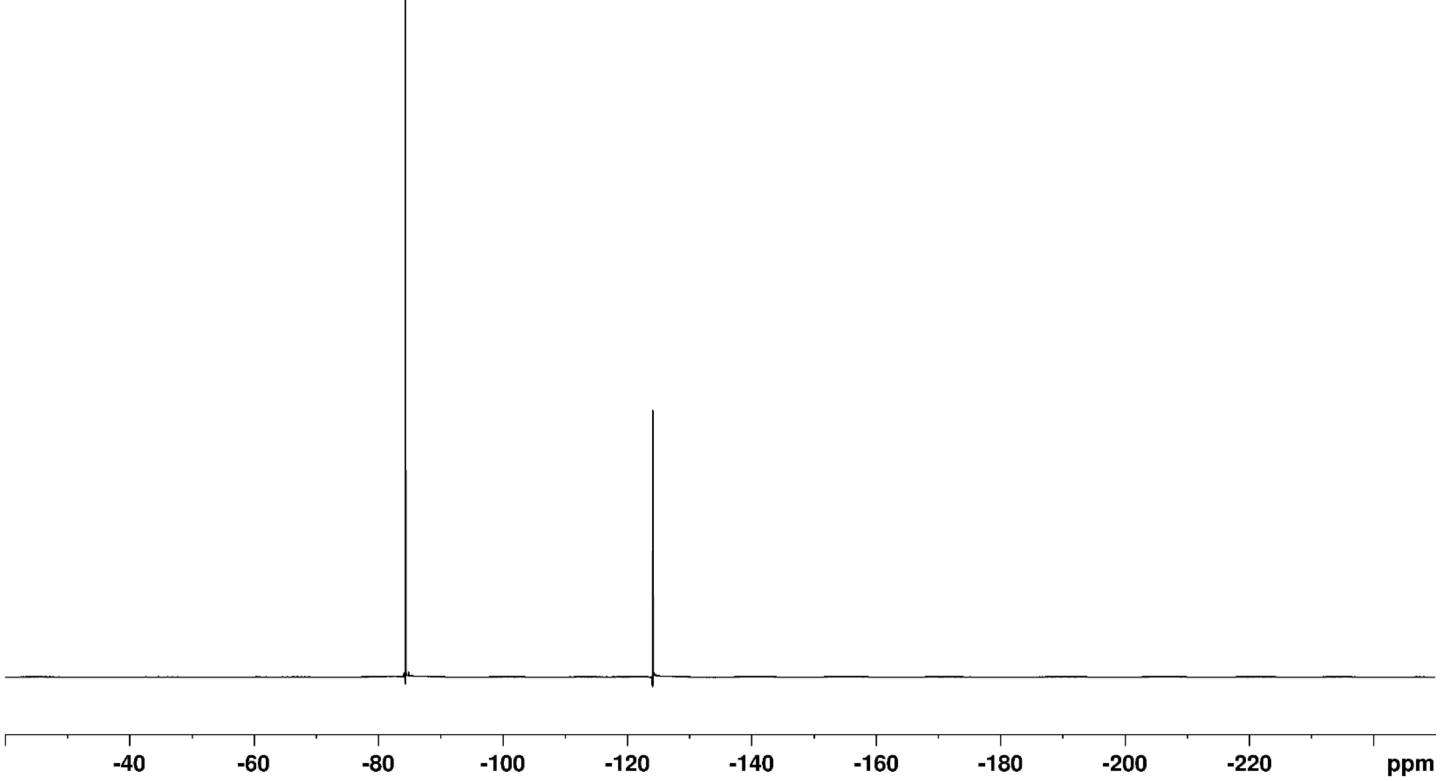

^{1}H NMR (700 MHz, CD_3CN) for 2,2,3,3,3-pentafluoropropyl (*S*)-(2-((ethyl(phenyl)carbamoyl)oxy)-1-(4-methoxyphenyl)ethyl)sulfamate (**11b**)

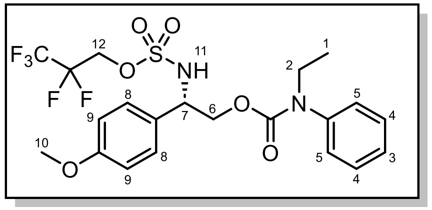

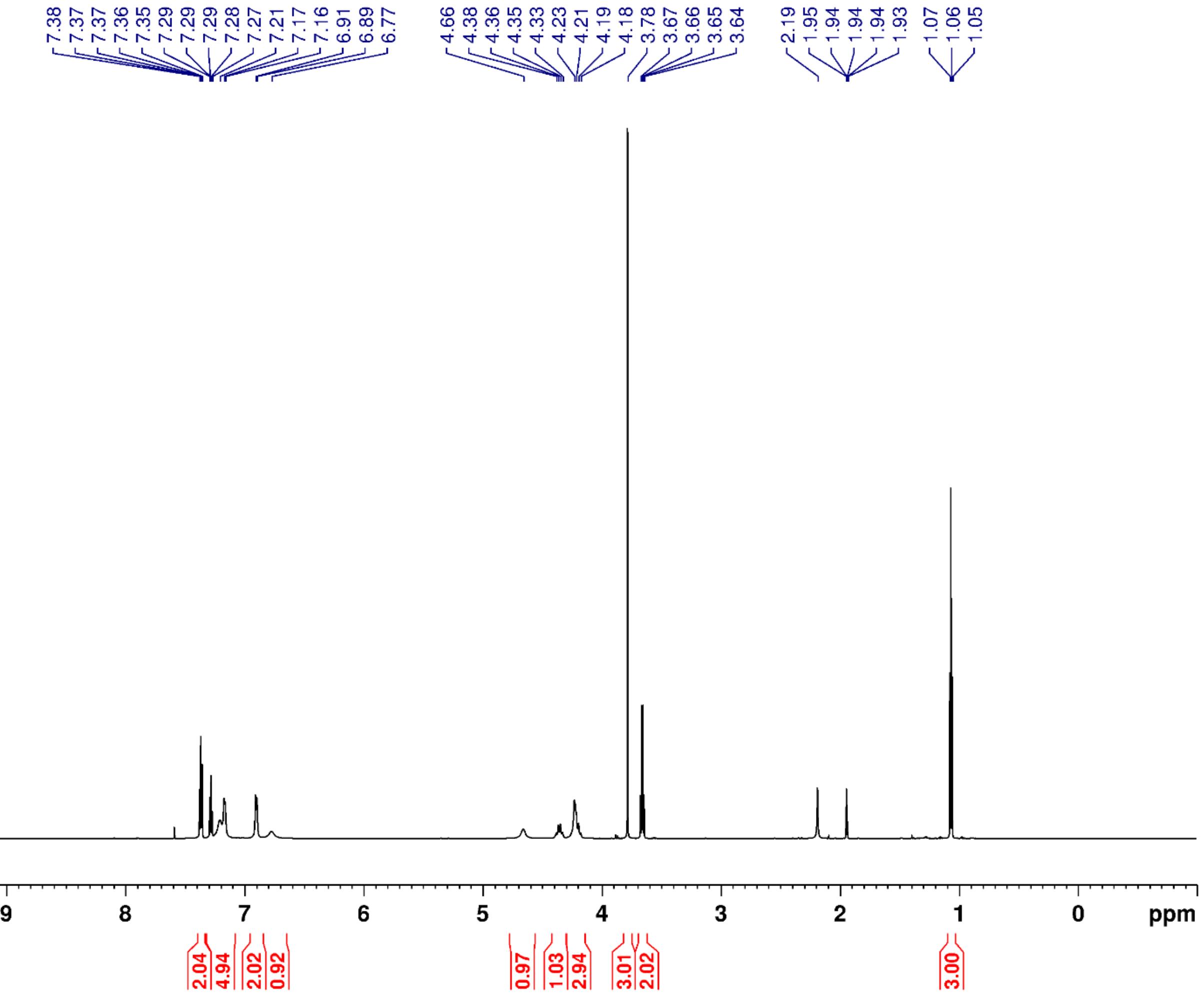

^{1}H NMR (700 MHz, CD_3CN) for 2,2,3,3,3-pentafluoropropyl (*R*)-(2-((ethyl(phenyl)carbamoyl)oxy)-1-(4-methoxyphenyl)ethyl)sulfamate (***ent*-11b**)

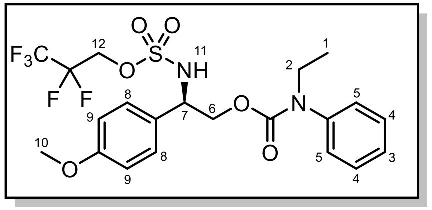

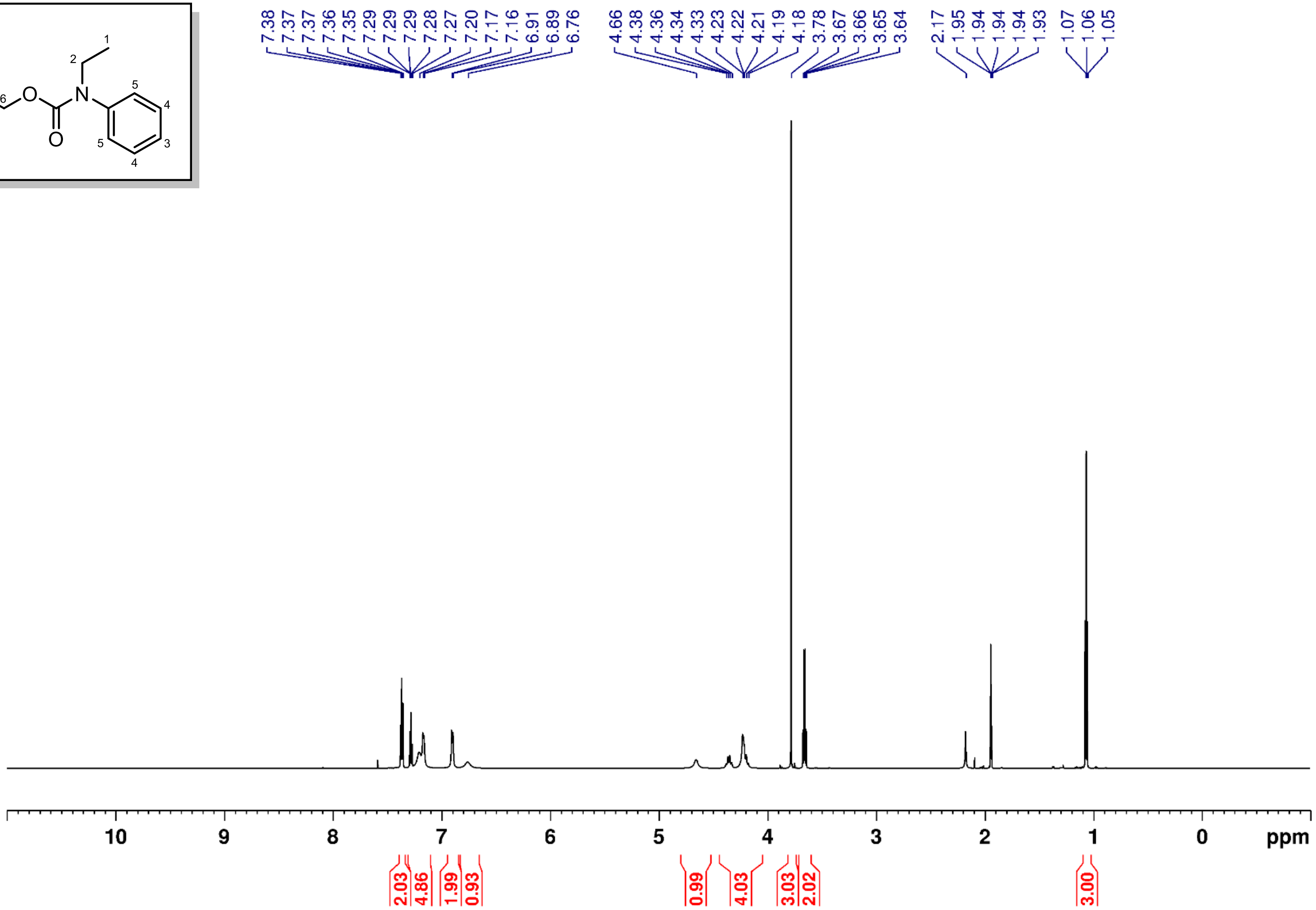

^{13}C NMR (176 MHz, CD_3CN) for 2,2,3,3,3-pentafluoropropyl (*S*)-(2-((ethyl(phenyl)carbamoyl)oxy)-1-(4-methoxyphenyl)ethyl)sulfamate (**11b**)

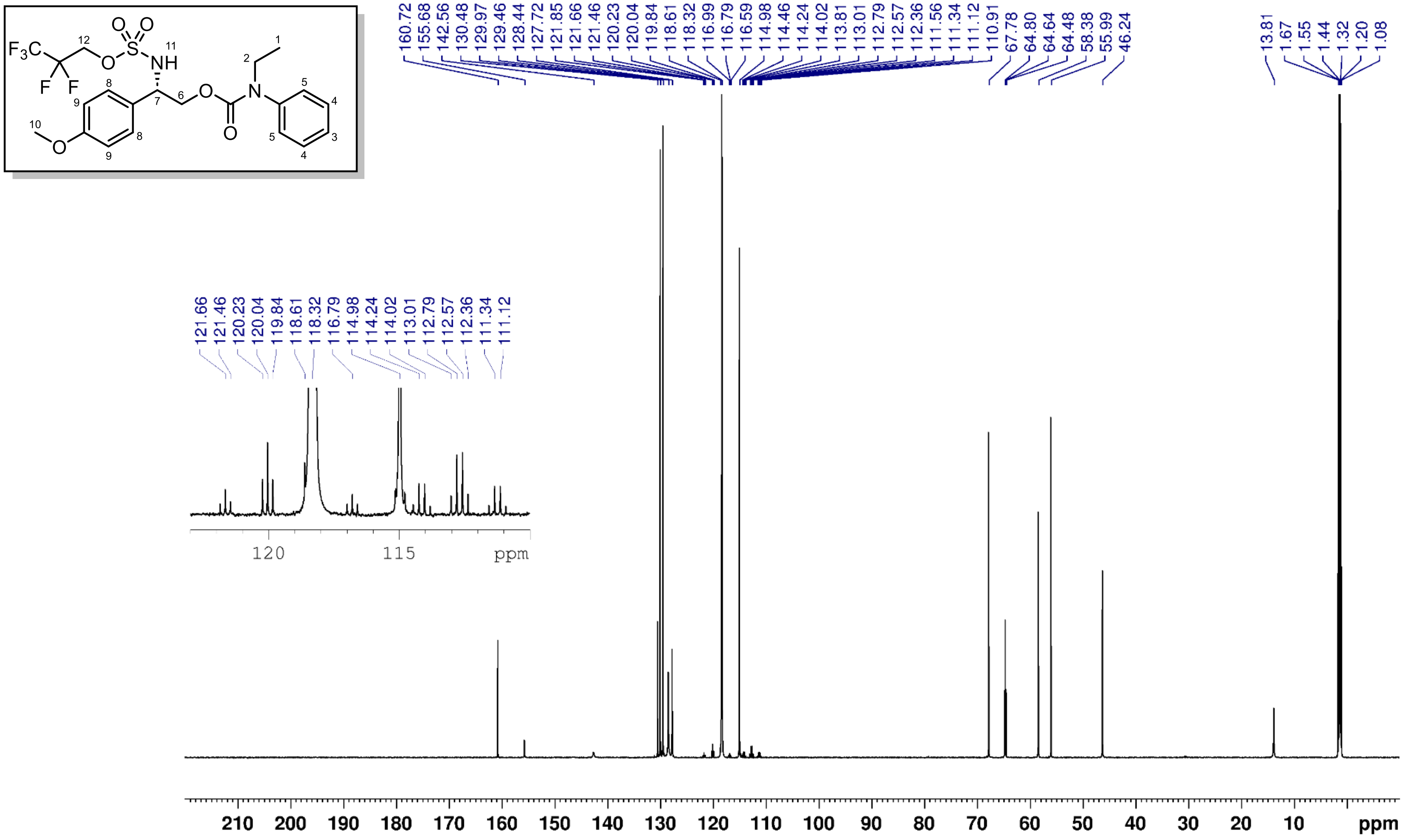

^{19}F NMR (376 MHz, CD_3CN) for 2,2,3,3,3-pentafluoropropyl (*S*)-(2-((ethyl(phenyl)carbamoyl)oxy)-1-(4-methoxyphenyl)ethyl)sulfamate (**11b**)

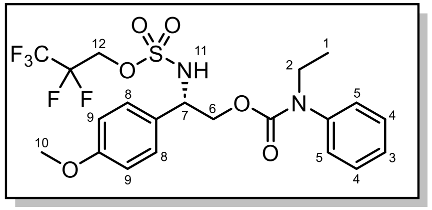

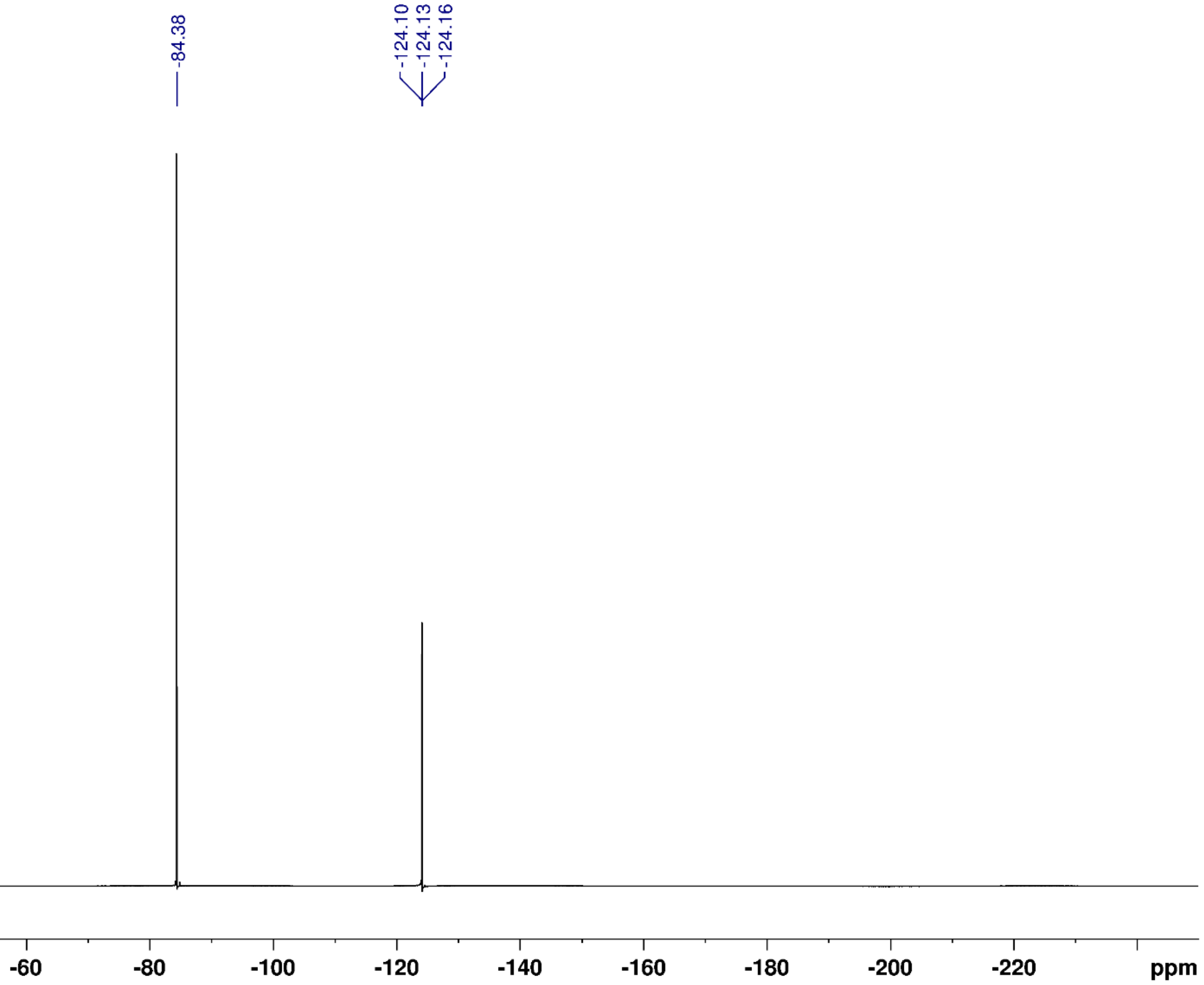

^{1}H NMR (700 MHz, CD_3CN) for 2,2,3,3,3-pentafluoropropyl (*S*)-(1-(4-(*tert*-butyl)phenyl)-2-((ethyl(phenyl)carbamoyl)oxy)ethyl)sulfamate (**11c**)

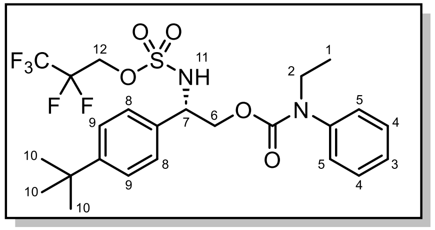

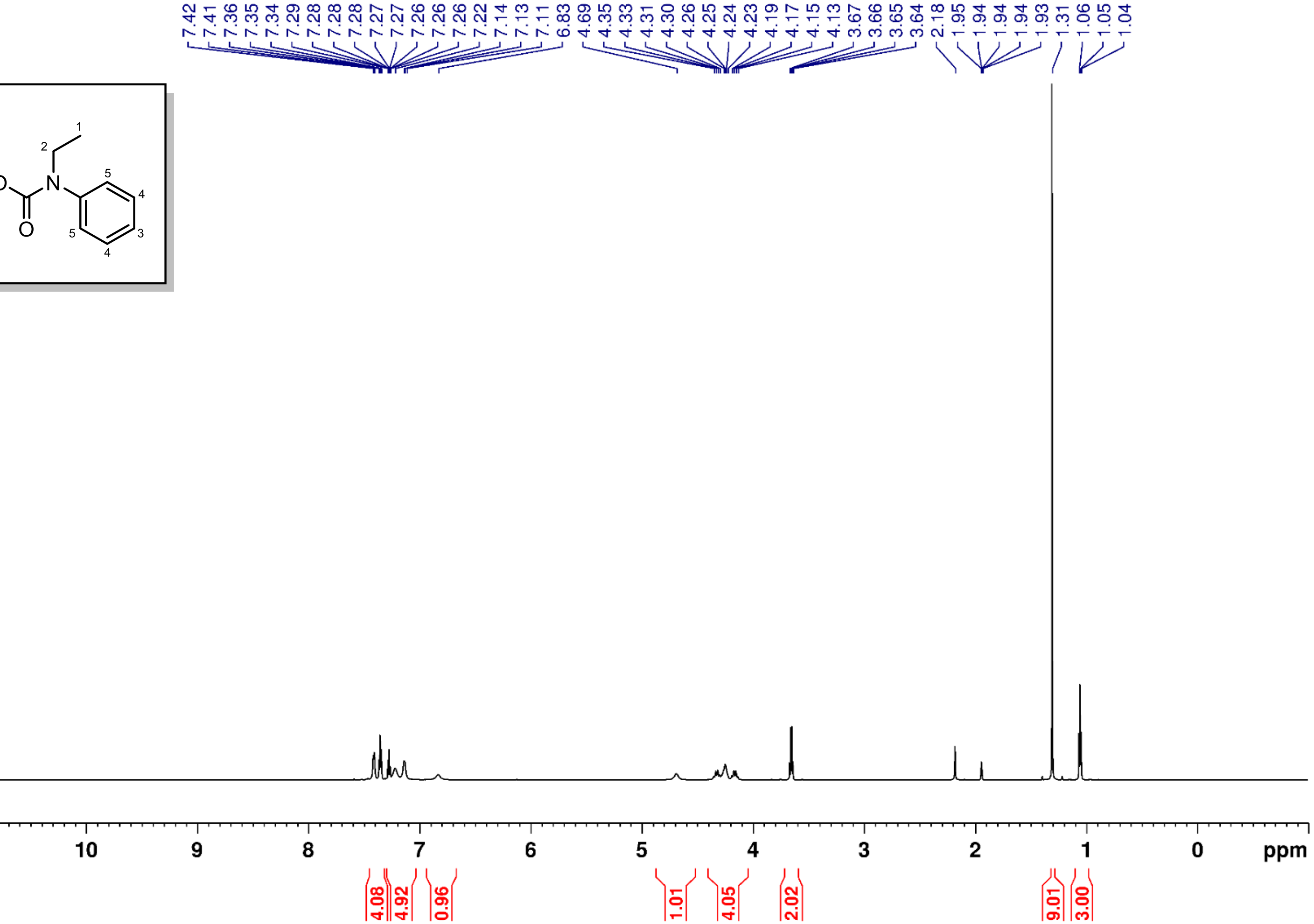

^{1}H NMR (700 MHz, CD_3CN) for 2,2,3,3,3-pentafluoropropyl (*R*)-(1-(4-(*tert*-butyl)phenyl)-2-((ethyl(phenyl)carbamoyl)oxy)ethyl)sulfamate (***ent*-11c**)

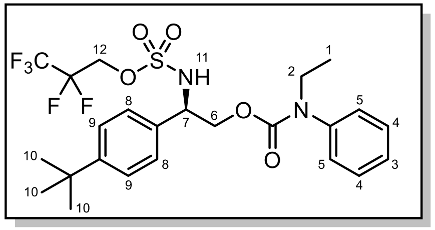

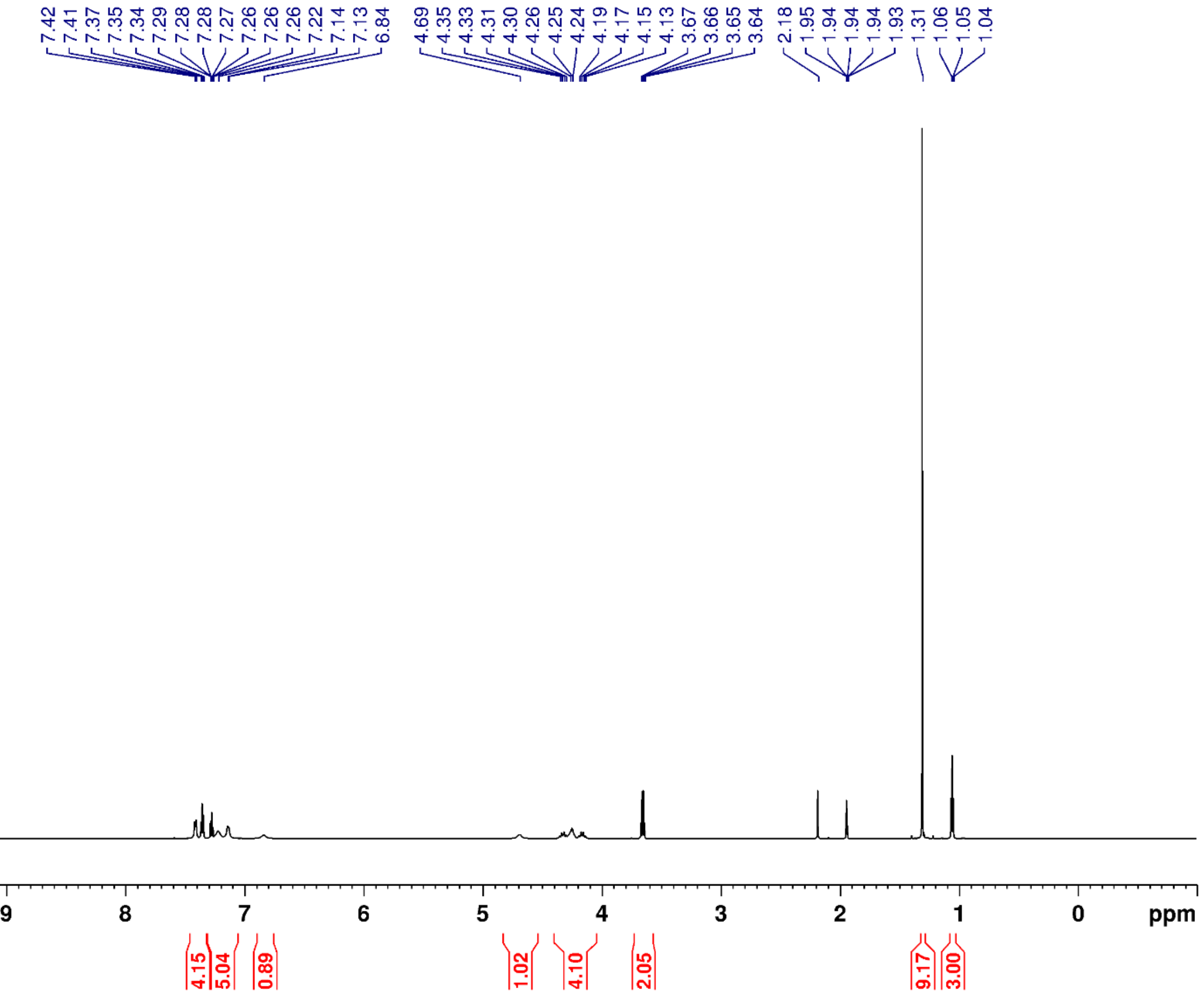

^{13}C NMR (176 MHz, CD_3CN) for 2,2,3,3,3-pentafluoropropyl (*S*)-(1-(4-(*tert*-butyl)phenyl)-2-((ethyl(phenyl)carbamoyl)oxy)ethyl)sulfamate (**11c**)

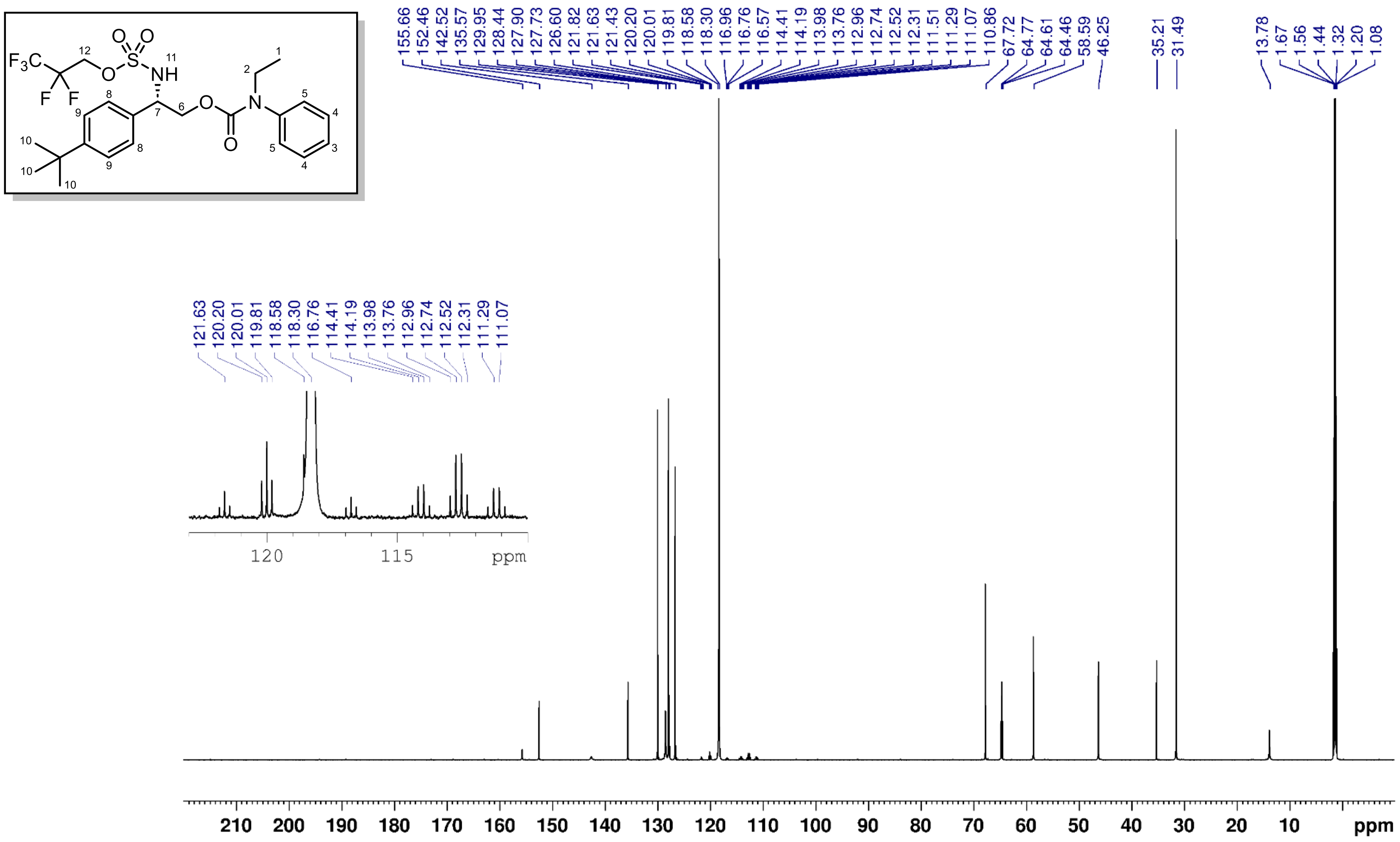

^{19}F NMR (376 MHz, CD_3CN) for 2,2,3,3,3-pentafluoropropyl (*S*)-(1-(4-(*tert*-butyl)phenyl)-2-((ethyl(phenyl)carbamoyl)oxy)ethyl)sulfamate (**11c**)

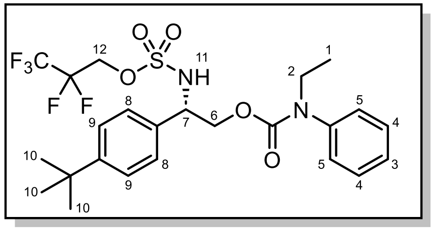

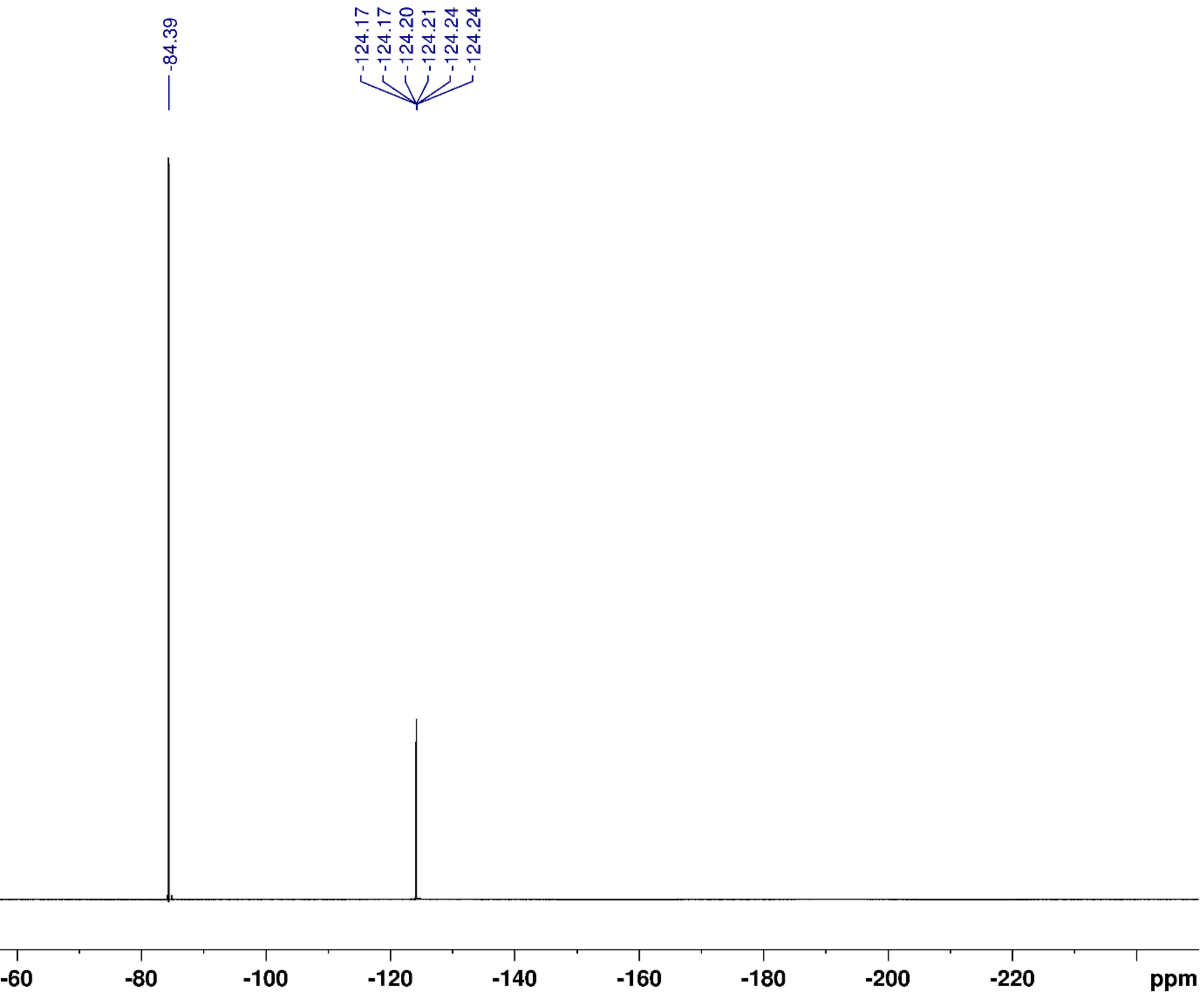

^{1}H NMR (700 MHz, CD_3CN) for 2,2,3,3,3-pentafluoropropyl (*S*)-(2-((ethyl(phenyl)carbamoyl)oxy)-1-(*p*-tolyl)ethyl)sulfamate (**11d**)

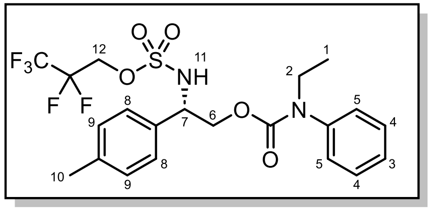

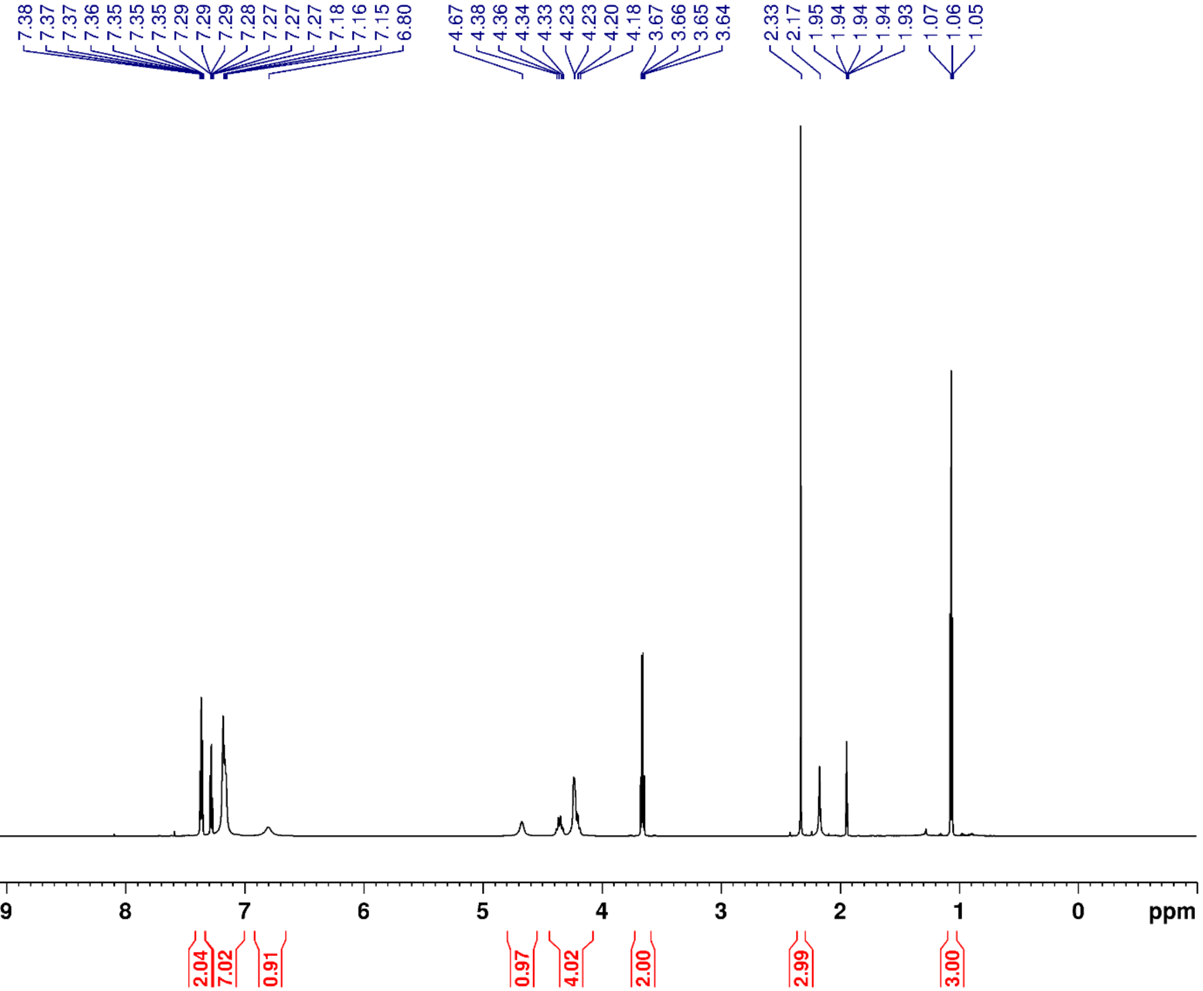

^{1}H NMR (700 MHz, CD_3CN) for 2,2,3,3,3-pentafluoropropyl (*R*)-(2-((ethyl(phenyl)carbamoyl)oxy)-1-(*p*-tolyl)ethyl)sulfamate (***ent*-11d**)

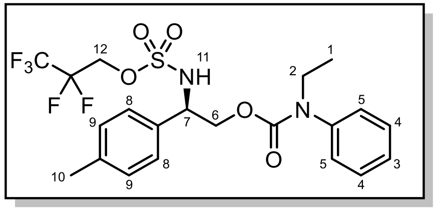

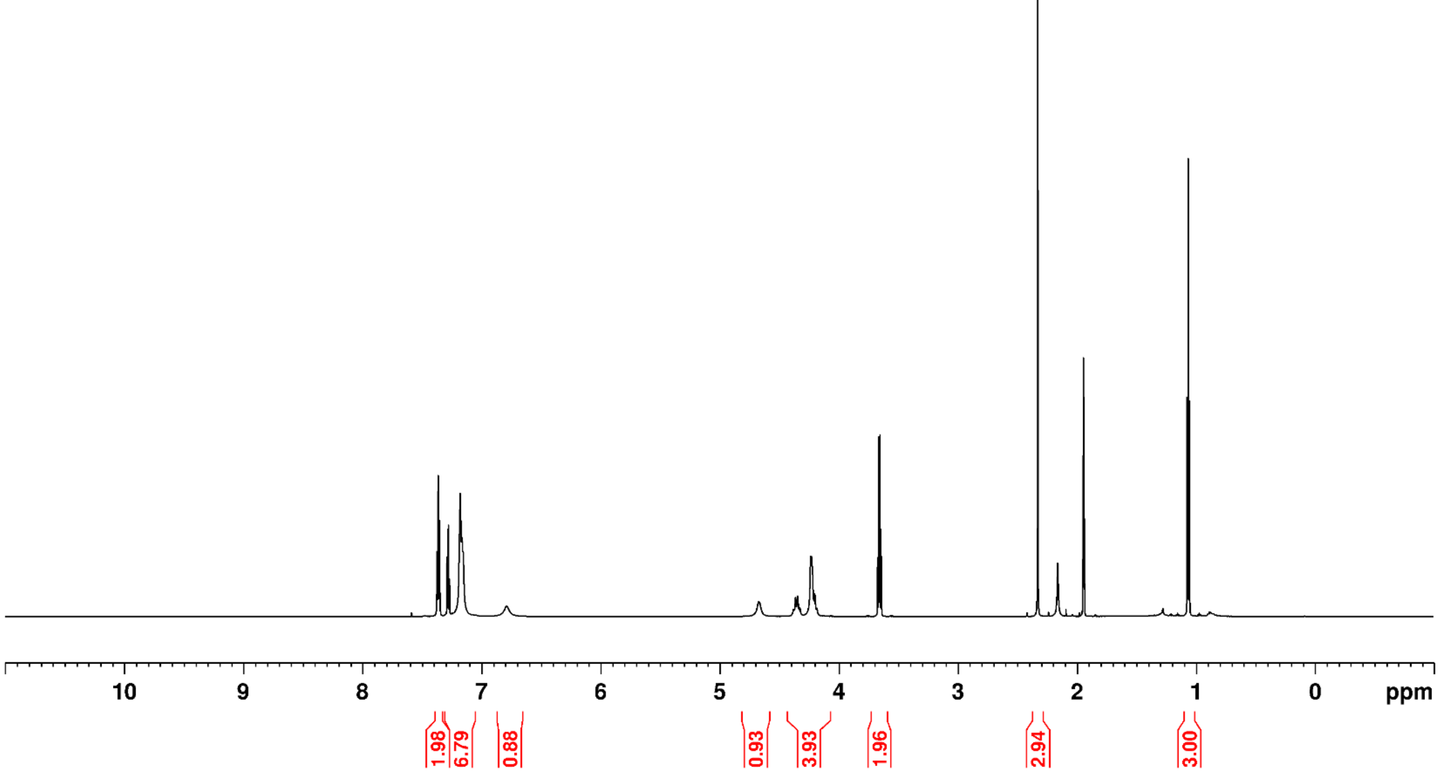

^{13}C NMR (176 MHz, CD_3CN) for 2,2,3,3,3-pentafluoropropyl (*S*)-(2-((ethyl(phenyl)carbamoyl)oxy)-1-(*p*-tolyl)ethyl)sulfamate (**11d**)

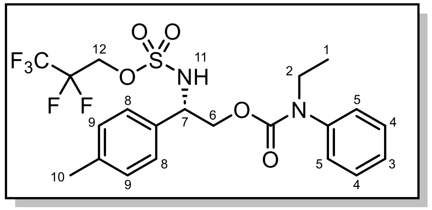

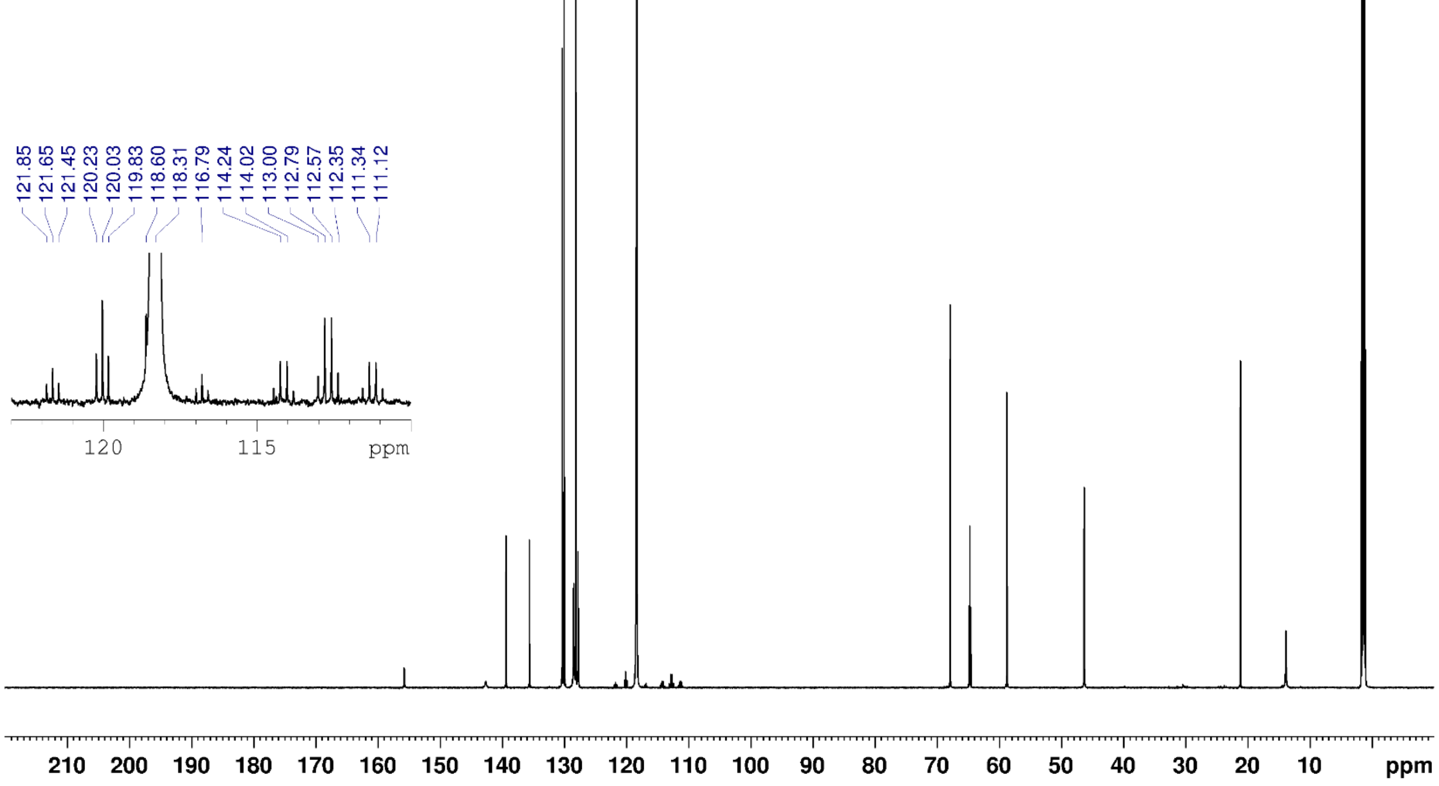

^{19}F NMR (376 MHz, CD_3CN) for 2,2,3,3,3-pentafluoropropyl (*S*)-(2-((ethyl(phenyl)carbamoyl)oxy)-1-(*p*-tolyl)ethyl)sulfamate (**11d**)

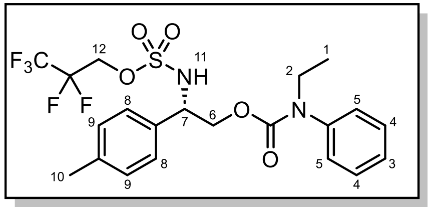

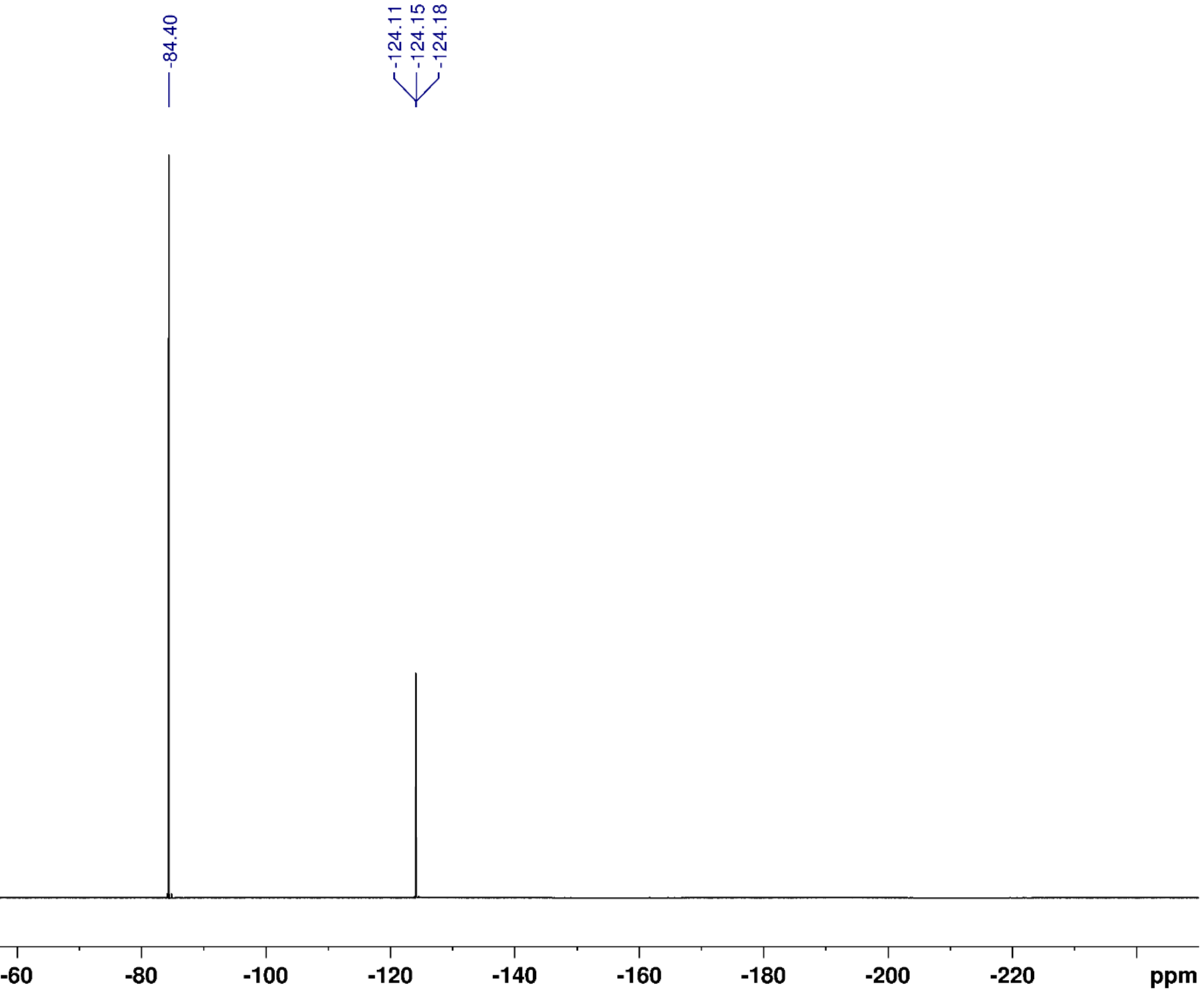

^{1}H NMR (700 MHz, CD_3CN) for 2,2,3,3,3-pentafluoropropyl (*S*)-(1-(4-bromophenyl)-2-((ethyl(phenyl)carbamoyl)oxy)ethyl)sulfamate (**11e**)

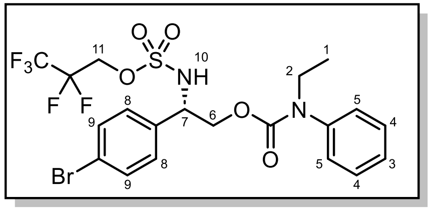

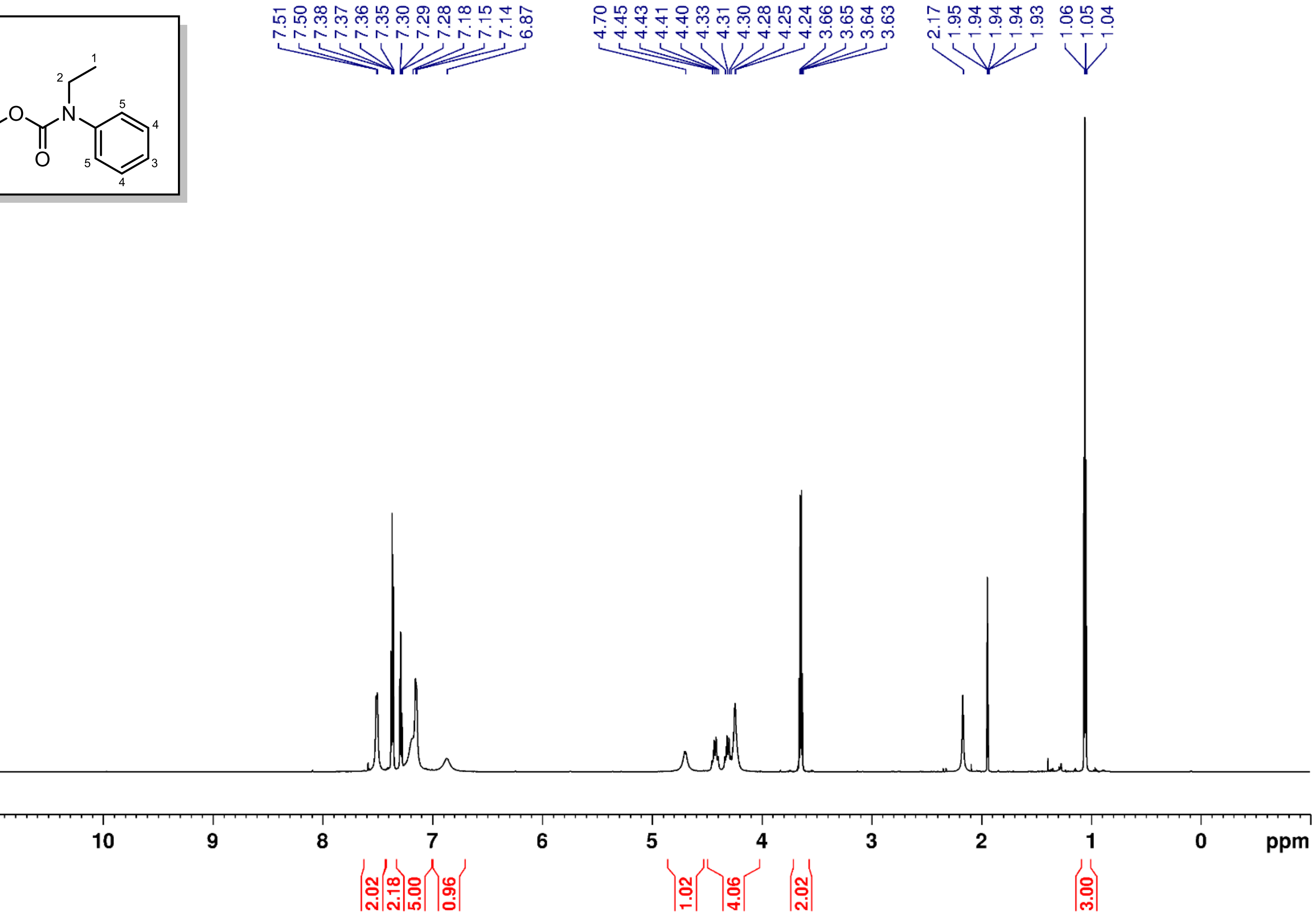

^{1}H NMR (700 MHz, CD_3CN) for 2,2,3,3,3-pentafluoropropyl (*R*)-(1-(4-bromophenyl)-2-((ethyl(phenyl)carbamoyl)oxy)ethyl)sulfamate (***ent*-11e**)

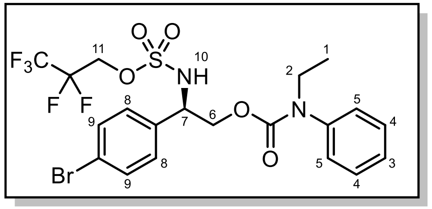

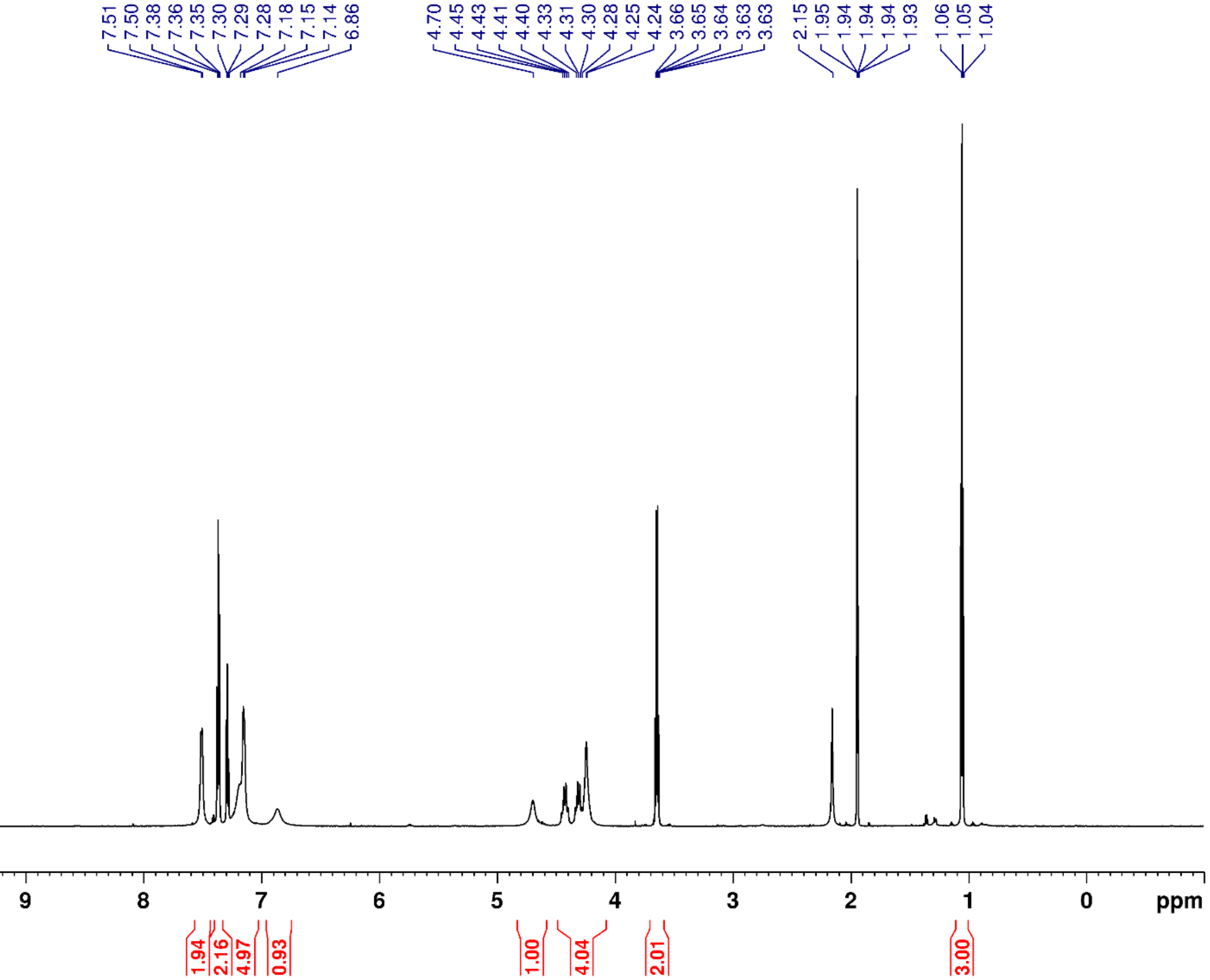

^{13}C NMR (176 MHz, CD_3CN) for 2,2,3,3,3-pentafluoropropyl (*S*)-(1-(4-bromophenyl)-2-((ethyl(phenyl) carbamoyl)oxy)ethyl)sulfamate (**11e**)

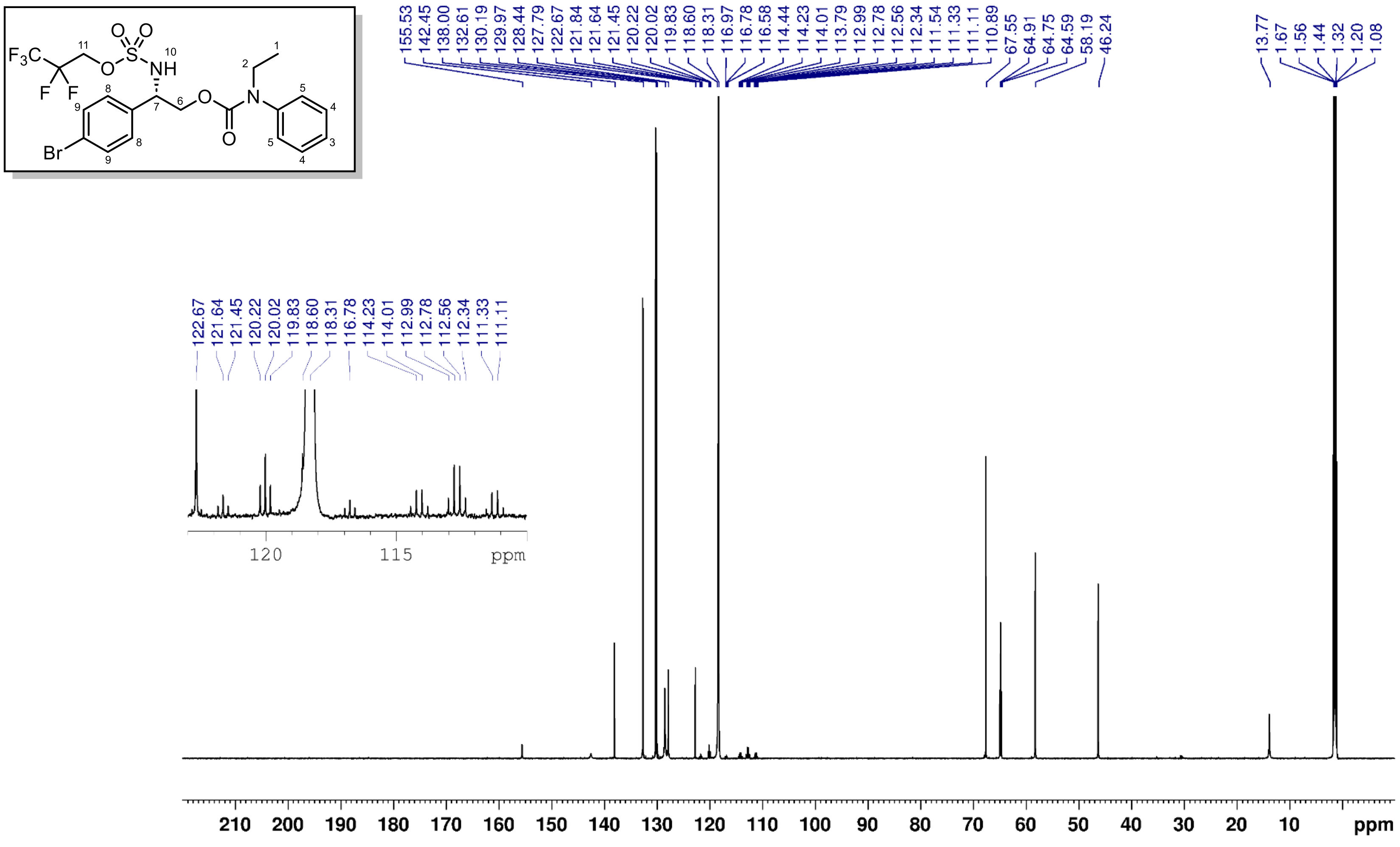

^{19}F NMR (376 MHz, CD_3CN) for 2,2,3,3,3-pentafluoropropyl (*S*)-(1-(4-bromophenyl)-2-((ethyl(phenyl) carbamoyl)oxy)ethyl)sulfamate (**11e**)

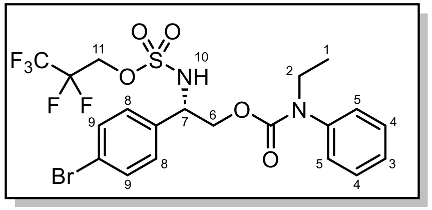

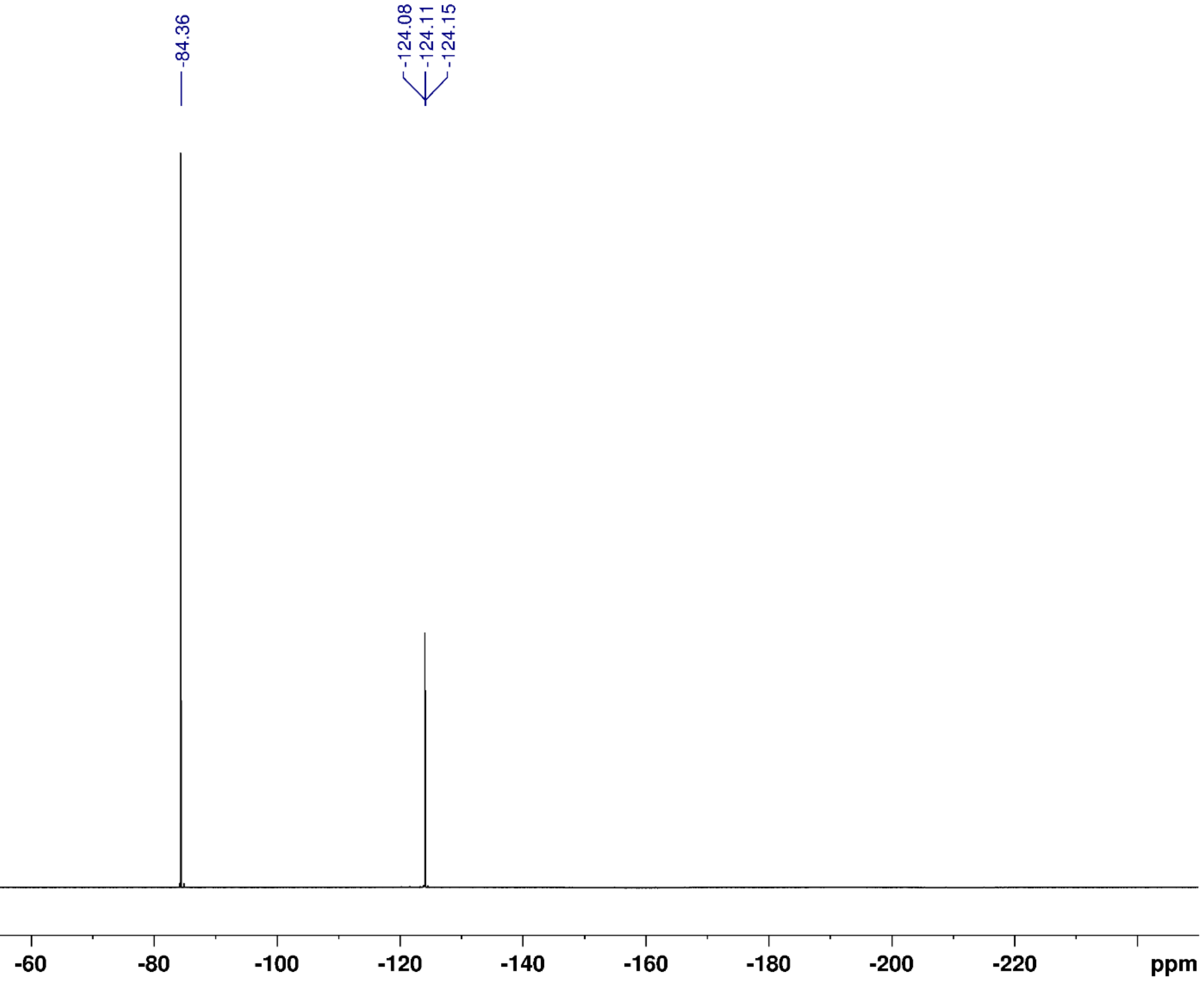

^{1}H NMR (700 MHz, CD_3CN) for 2,2,3,3,3-pentafluoropropyl (*S*)-(1-(4-chlorophenyl)-2-((ethyl(phenyl)carbamoyl)oxy)ethyl)sulfamate (**11f**)

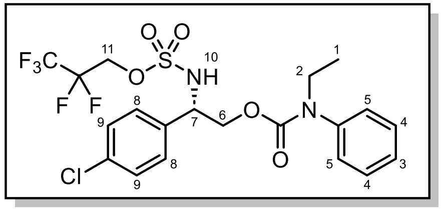

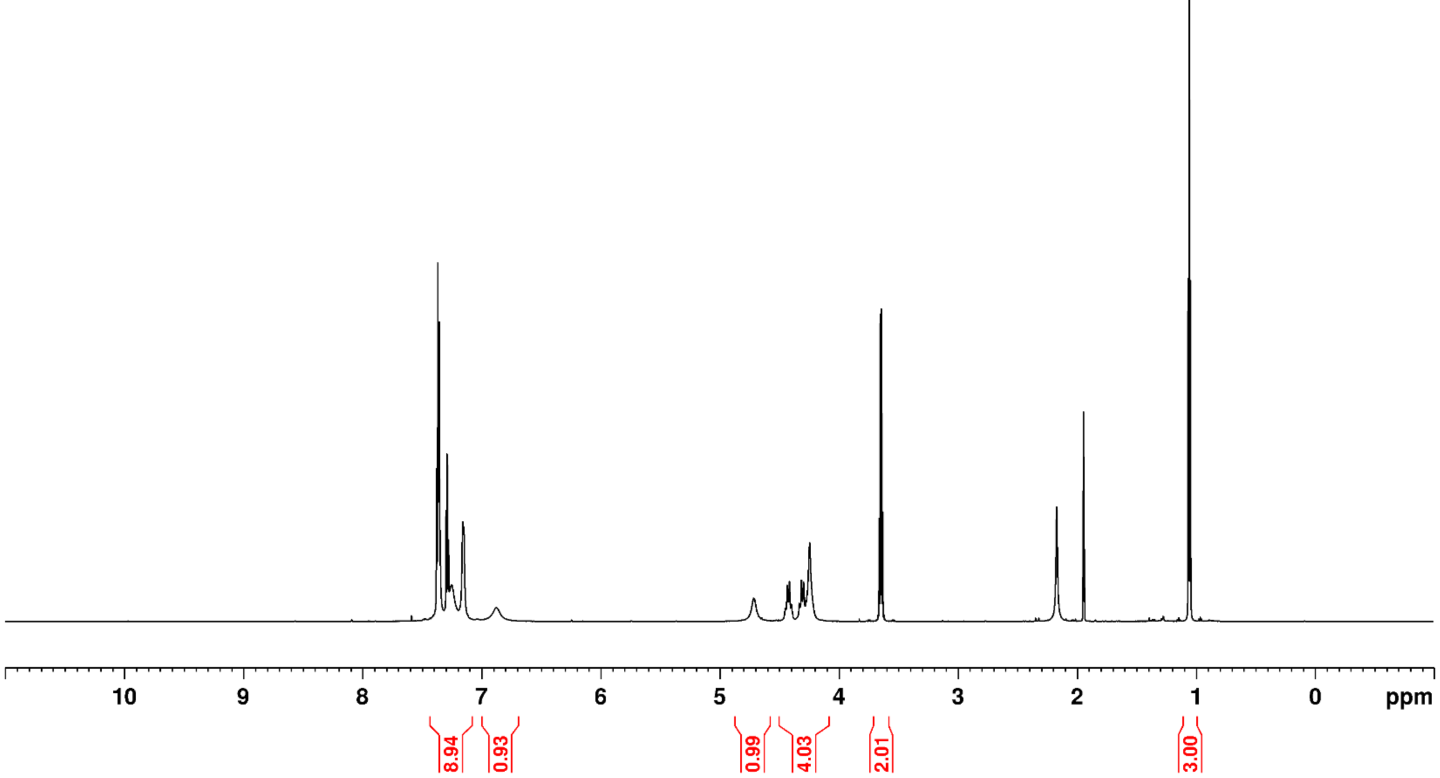

^{1}H NMR (700 MHz, CD_3CN) for 2,2,3,3,3-pentafluoropropyl (*R*)-(1-(4-chlorophenyl)-2-((ethyl(phenyl)carbamoyl)oxy)ethyl)sulfamate (***ent*-11f**)

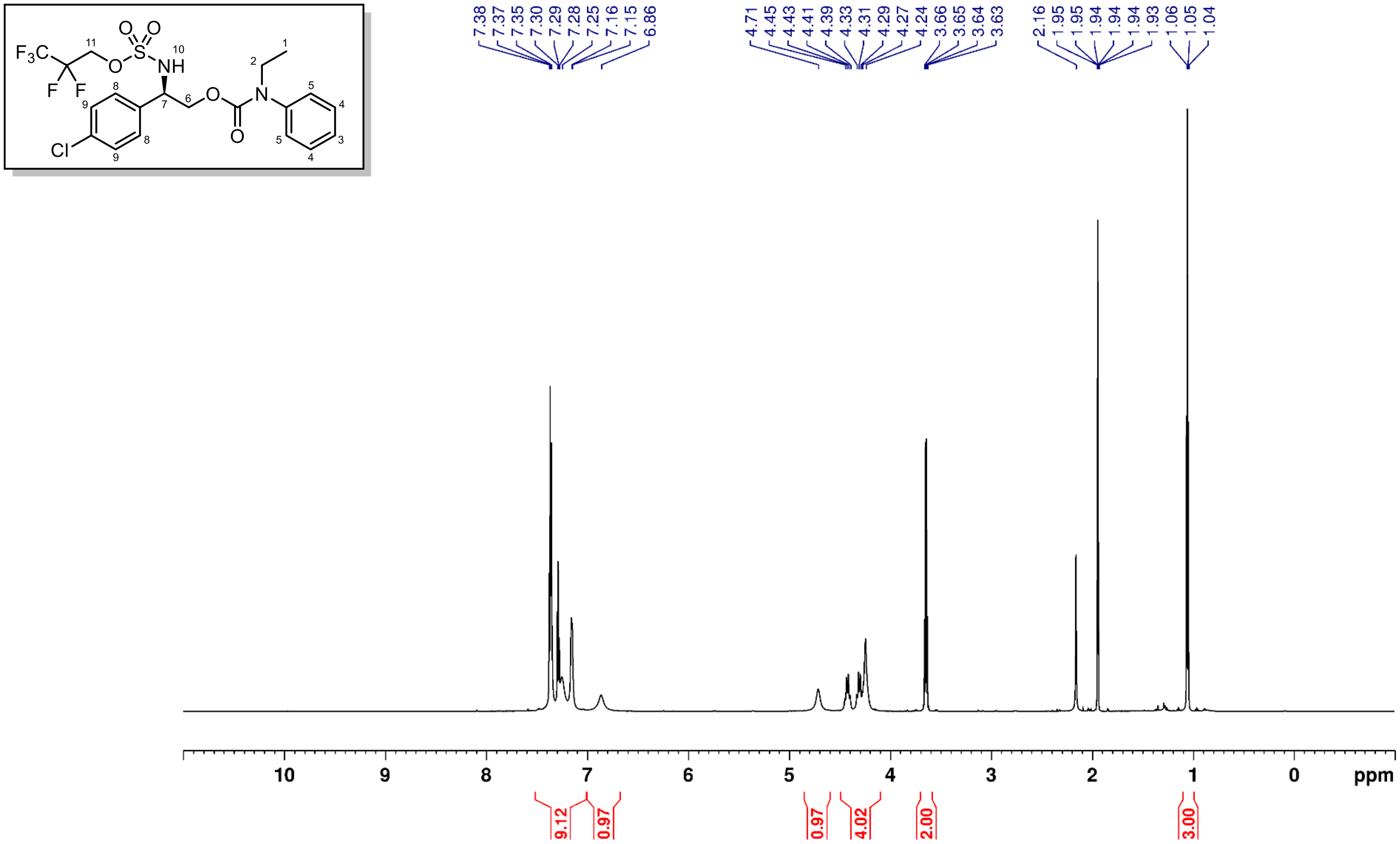

^{13}C NMR (176 MHz, CD_3CN) for 2,2,3,3,3-pentafluoropropyl (*S*)-(1-(4-chlorophenyl)-2-((ethyl(phenyl)carbamoyl)oxy)ethyl)sulfamate (**11f**)

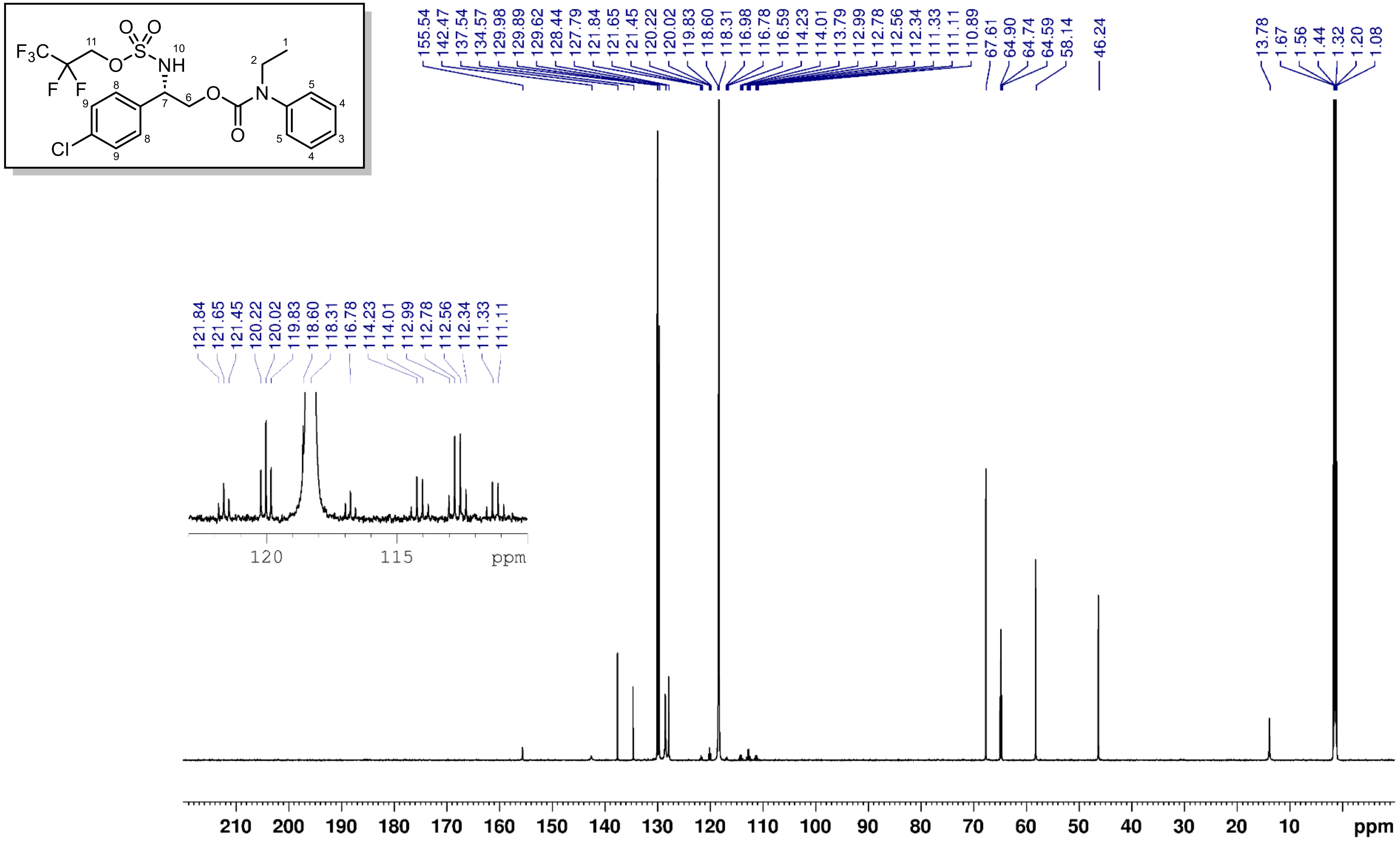

^{19}F NMR (376 MHz, CD_3CN) for 2,2,3,3,3-pentafluoropropyl (*S*)-(1-(4-chlorophenyl)-2-((ethyl(phenyl)carbamoyl)oxy)ethyl)sulfamate (**11f**)

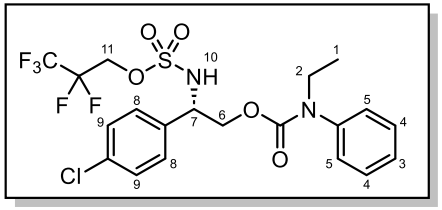

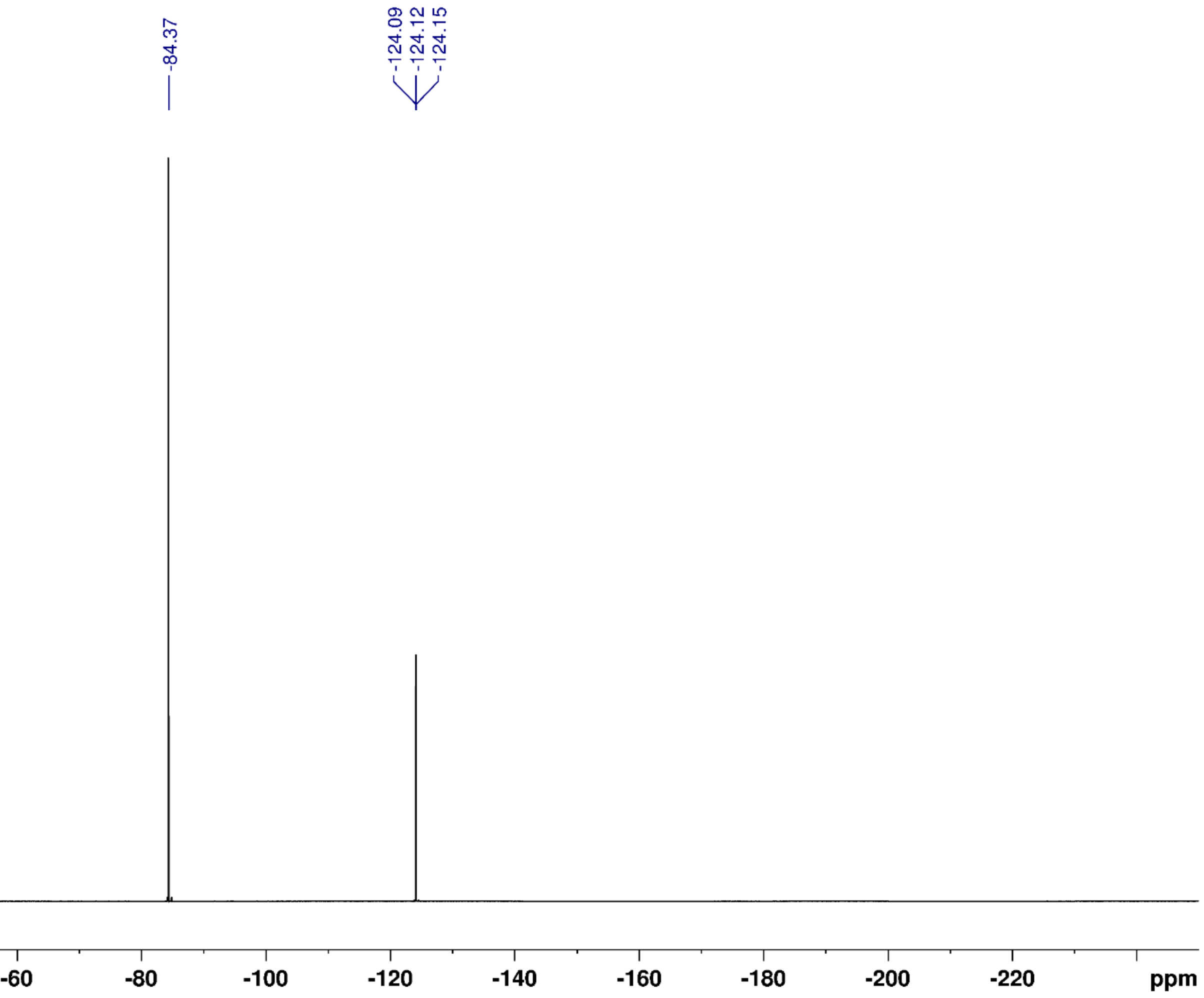

^{1}H NMR (700 MHz, CD_3CN) for 2,2,3,3,3-pentafluoropropyl (*S*)-(2-((ethyl(phenyl)carbamoyl)oxy)-1-(4-(trifluoromethyl)phenyl)ethyl)sulfamate (**11g**)

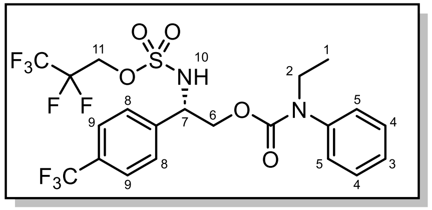

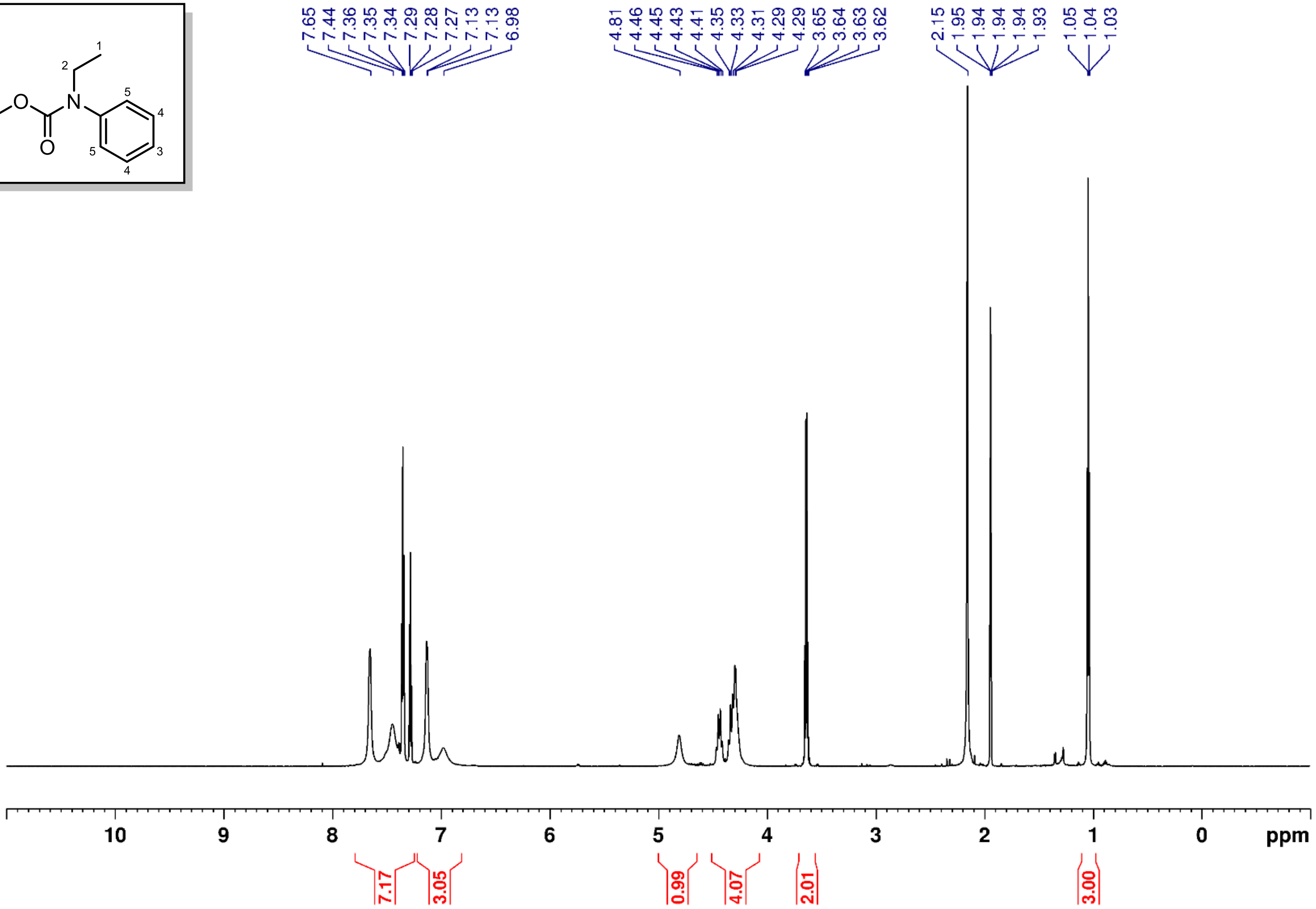

^{1}H NMR (700 MHz, CD_3CN) for 2,2,3,3,3-pentafluoropropyl (*R*)-(2-((ethyl(phenyl)carbamoyl)oxy)-1-(4-(trifluoromethyl)phenyl)ethyl)sulfamate (***ent*-11g**)

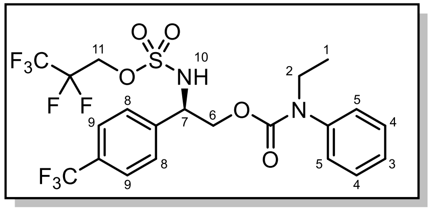

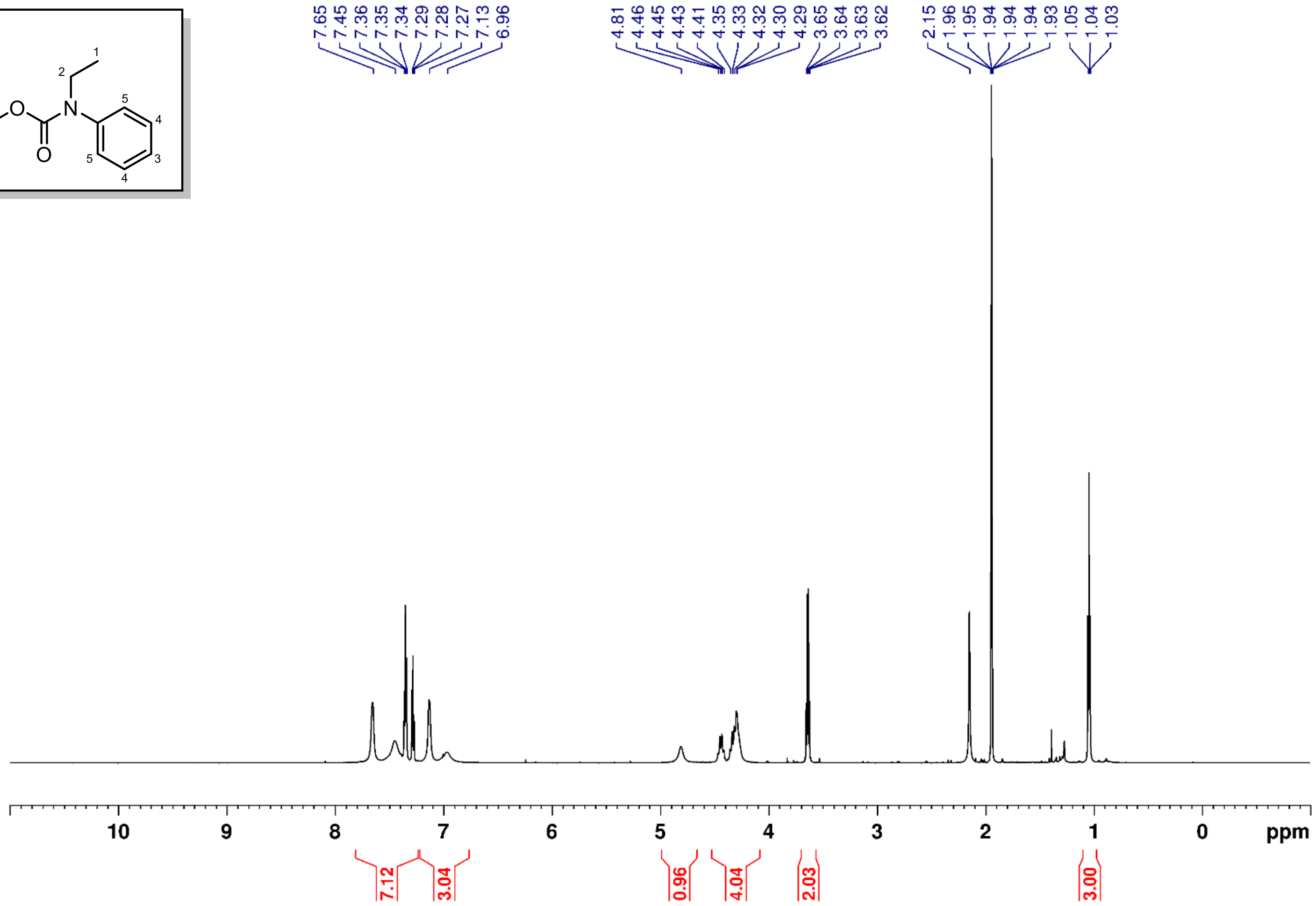

^{13}C NMR (176 MHz, CD_3CN) for 2,2,3,3,3-pentafluoropropyl (*S*)-(2-((ethyl(phenyl)carbamoyl)oxy)-1-(4-(trifluoromethyl)phenyl)ethyl)sulfamate (**11g**)

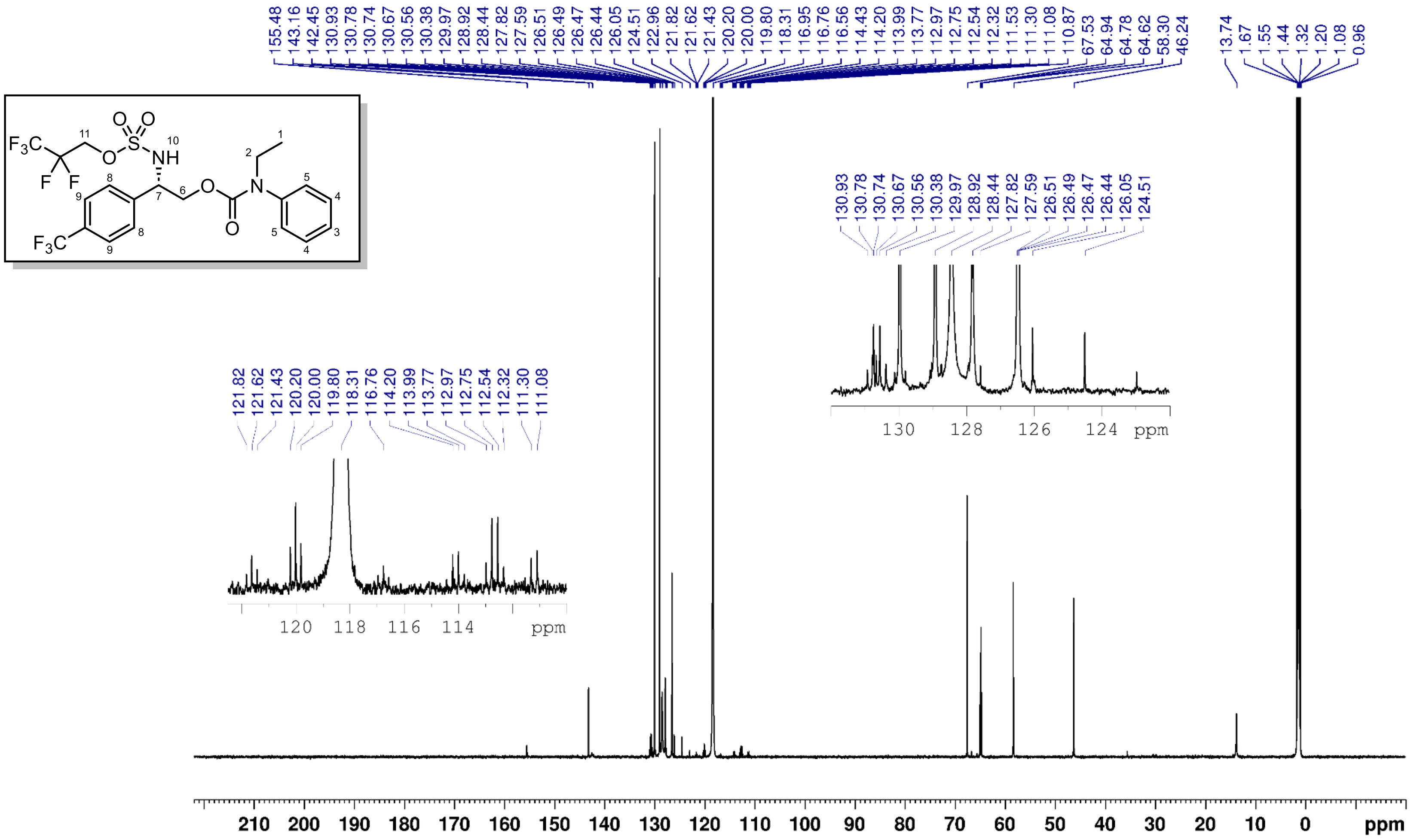

^{19}F NMR (376 MHz, CD_3CN) for 2,2,3,3,3-pentafluoropropyl (*S*)-(2-((ethyl(phenyl)carbamoyl)oxy)-1-(4-(trifluoromethyl)phenyl)ethyl)sulfamate (**11g**)

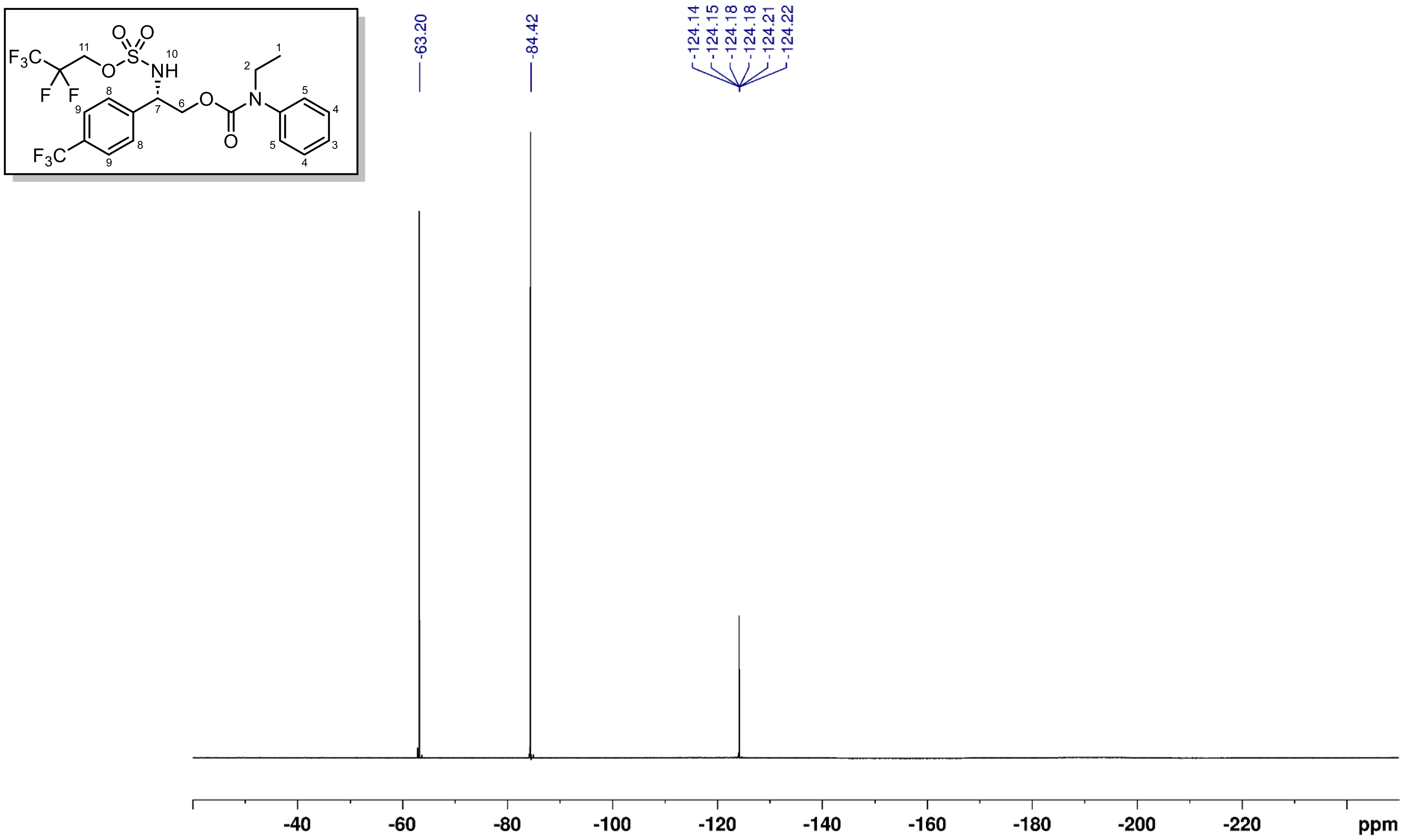

^{1}H NMR (700 MHz, CD_3CN) for 2,2,3,3,3-pentafluoropropyl (*S*)-(2-((ethyl(phenyl)carbamoyl)oxy)-1-(3-methoxyphenyl)ethyl)sulfamate (**11h**)

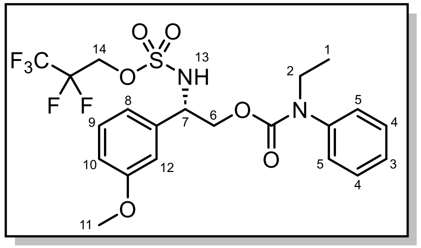

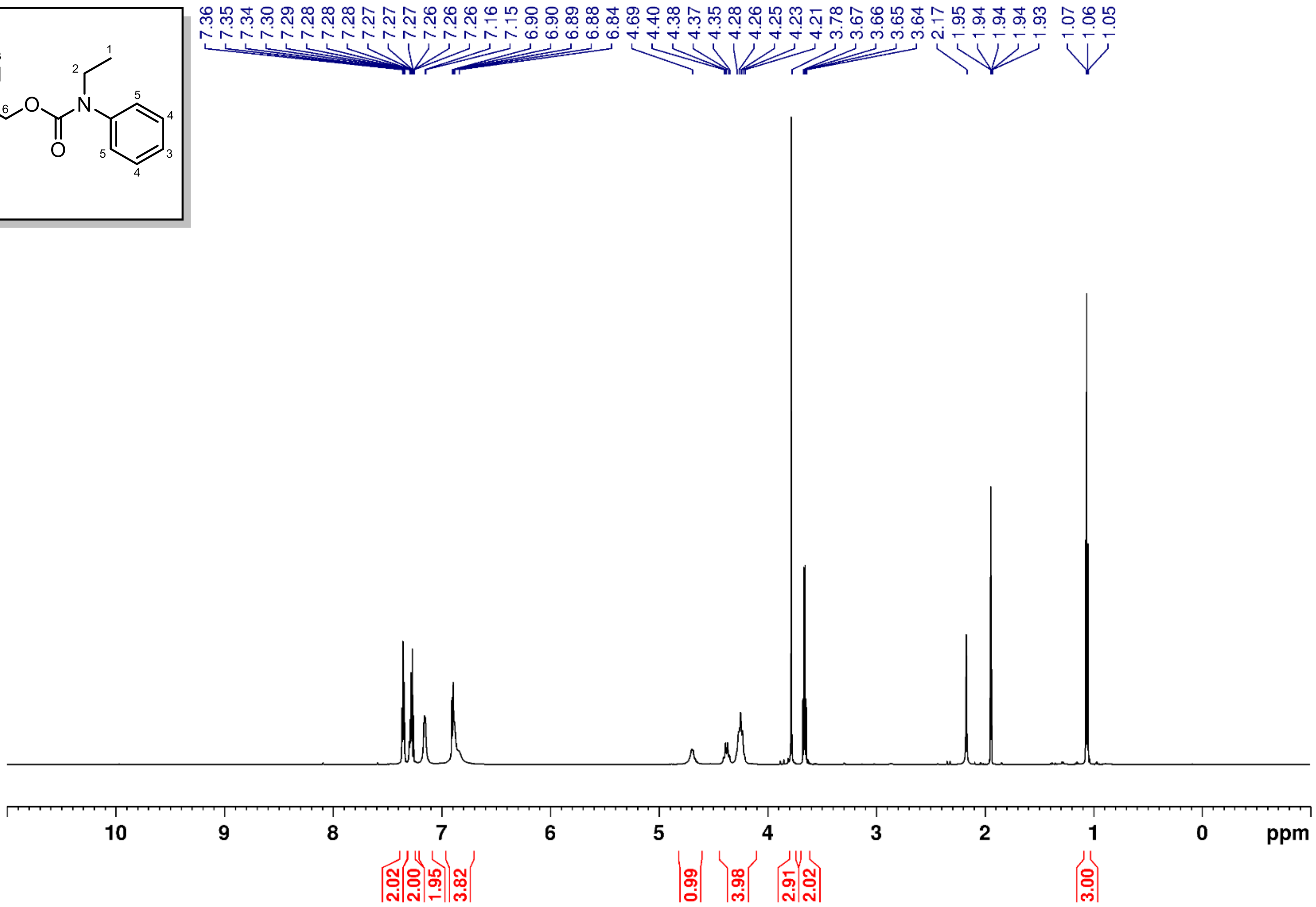

^{1}H NMR (700 MHz, CD_3CN) for 2,2,3,3,3-pentafluoropropyl (*R*)-(2-((ethyl(phenyl)carbamoyl)oxy)-1-(3-methoxyphenyl)ethyl)sulfamate (***ent*-11h**)

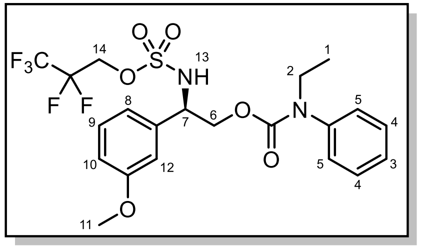

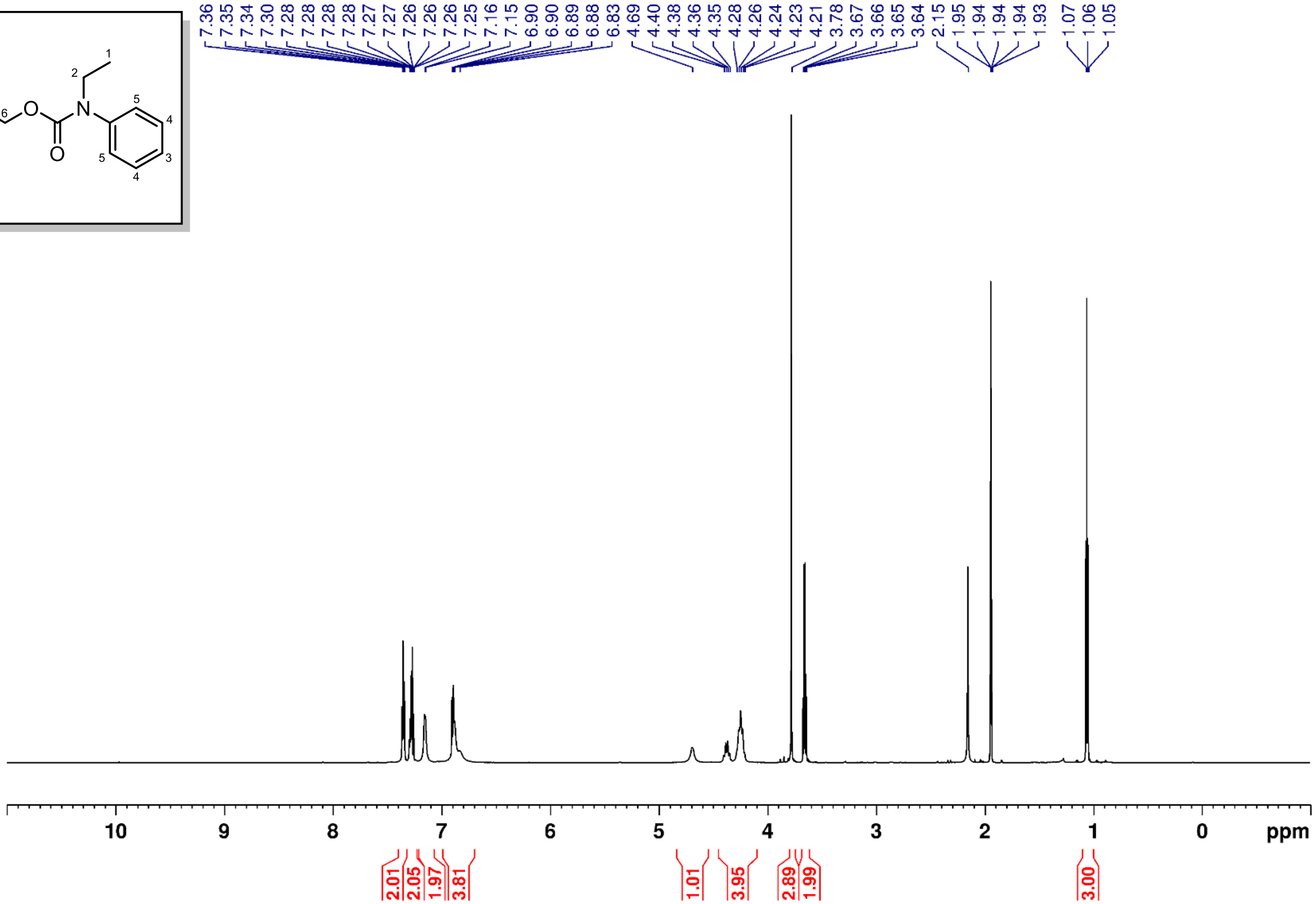

^{13}C NMR (176 MHz, CD_3CN) for 2,2,3,3,3-pentafluoropropyl (*S*)-(2-((ethyl(phenyl)carbamoyl)oxy)-1-(3-methoxyphenyl)ethyl)sulfamate (**11h**)

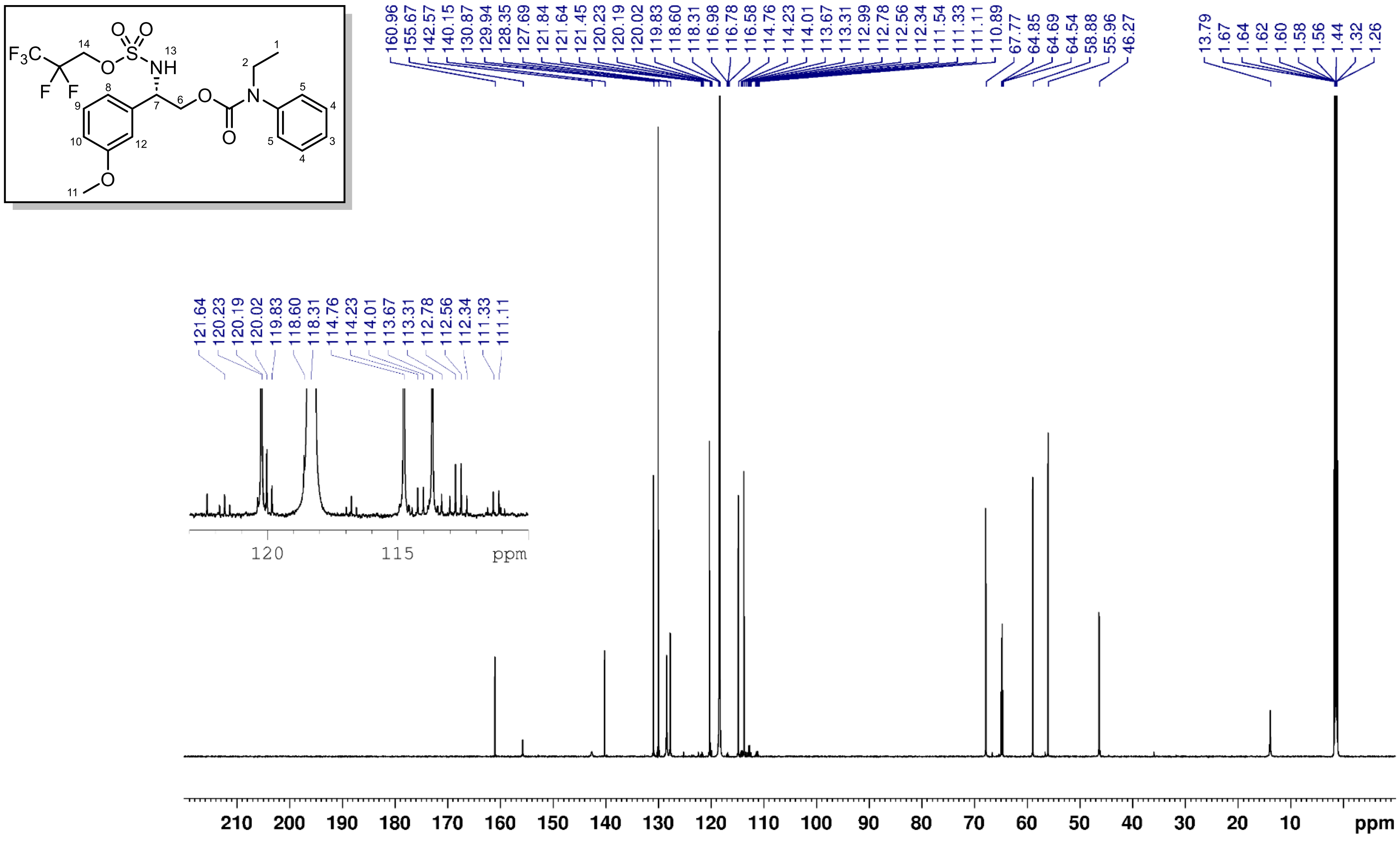

^{19}F NMR (376 MHz, CD_3CN) for 2,2,3,3,3-pentafluoropropyl (*S*)-(2-((ethyl(phenyl)carbamoyl)oxy)-1-(3-methoxyphenyl)ethyl)sulfamate (**11h**)

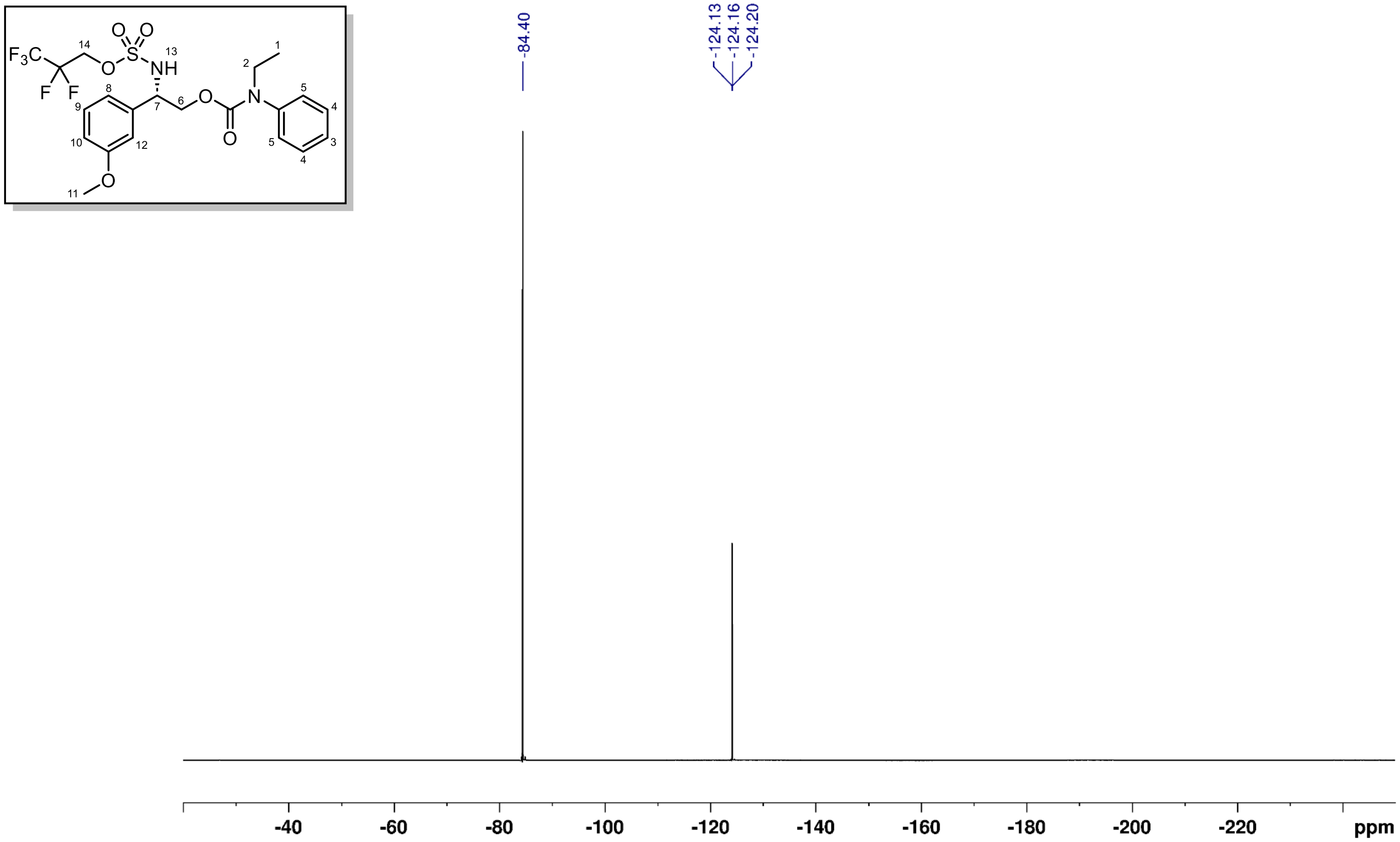

^{1}H NMR (700 MHz, CD_3CN) for 2,2,3,3,3-pentafluoropropyl (*S*)-(2-((ethyl(phenyl)carbamoyl)oxy)-1-(*m*-tolyl)ethyl)sulfamate (**11i**)

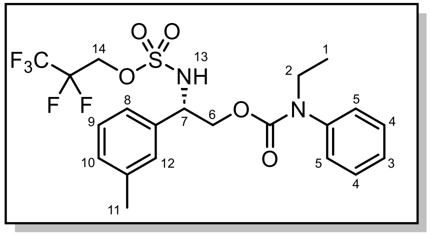

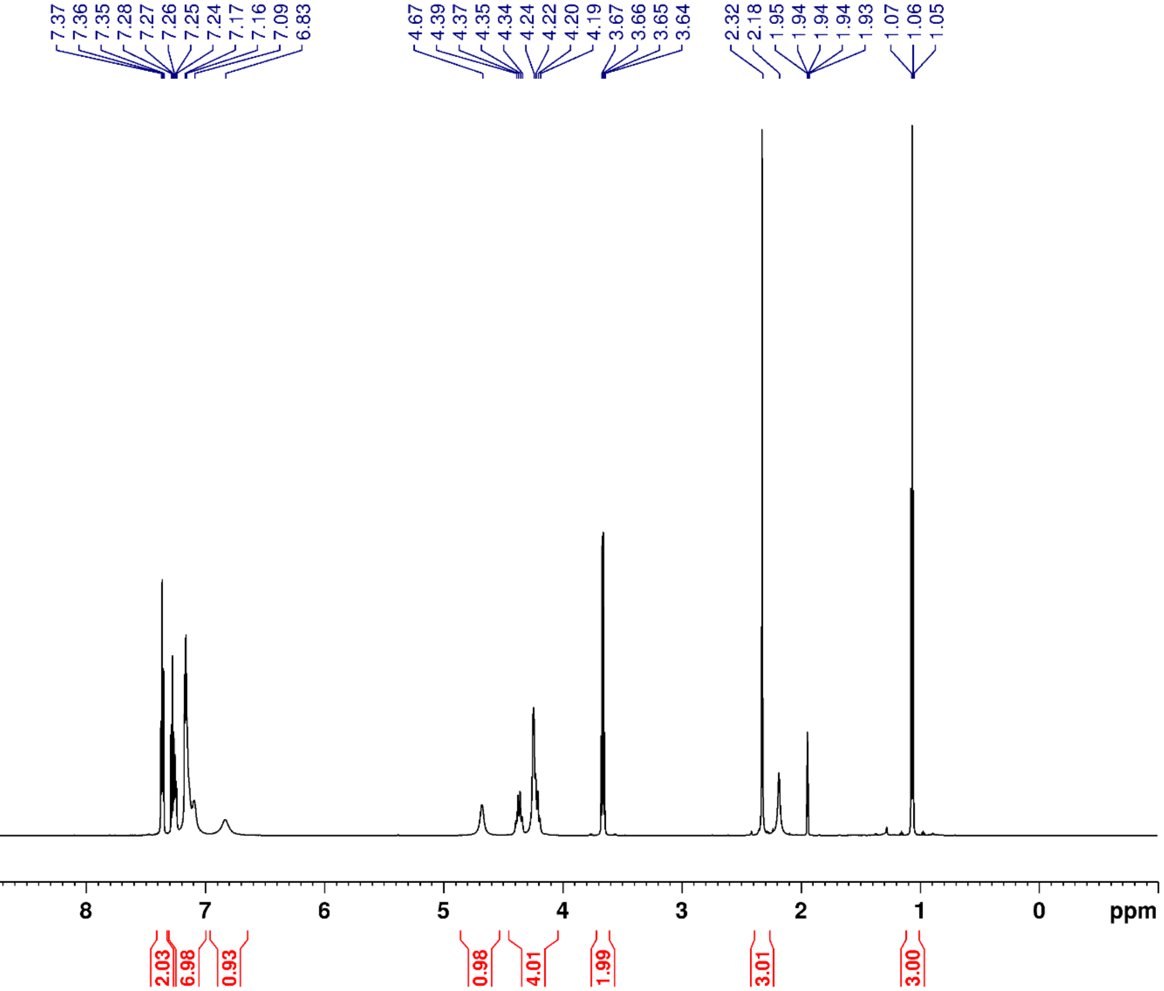

^{1}H NMR (700 MHz, CD_3CN) for 2,2,3,3,3-pentafluoropropyl (*R*)-(2-((ethyl(phenyl)carbamoyl)oxy)-1-(*m*-tolyl)ethyl)sulfamate (***ent*-11i**)

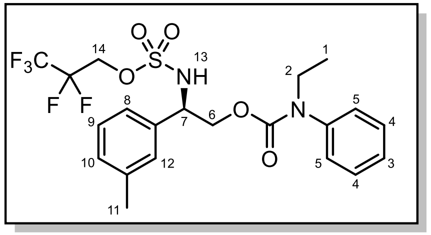

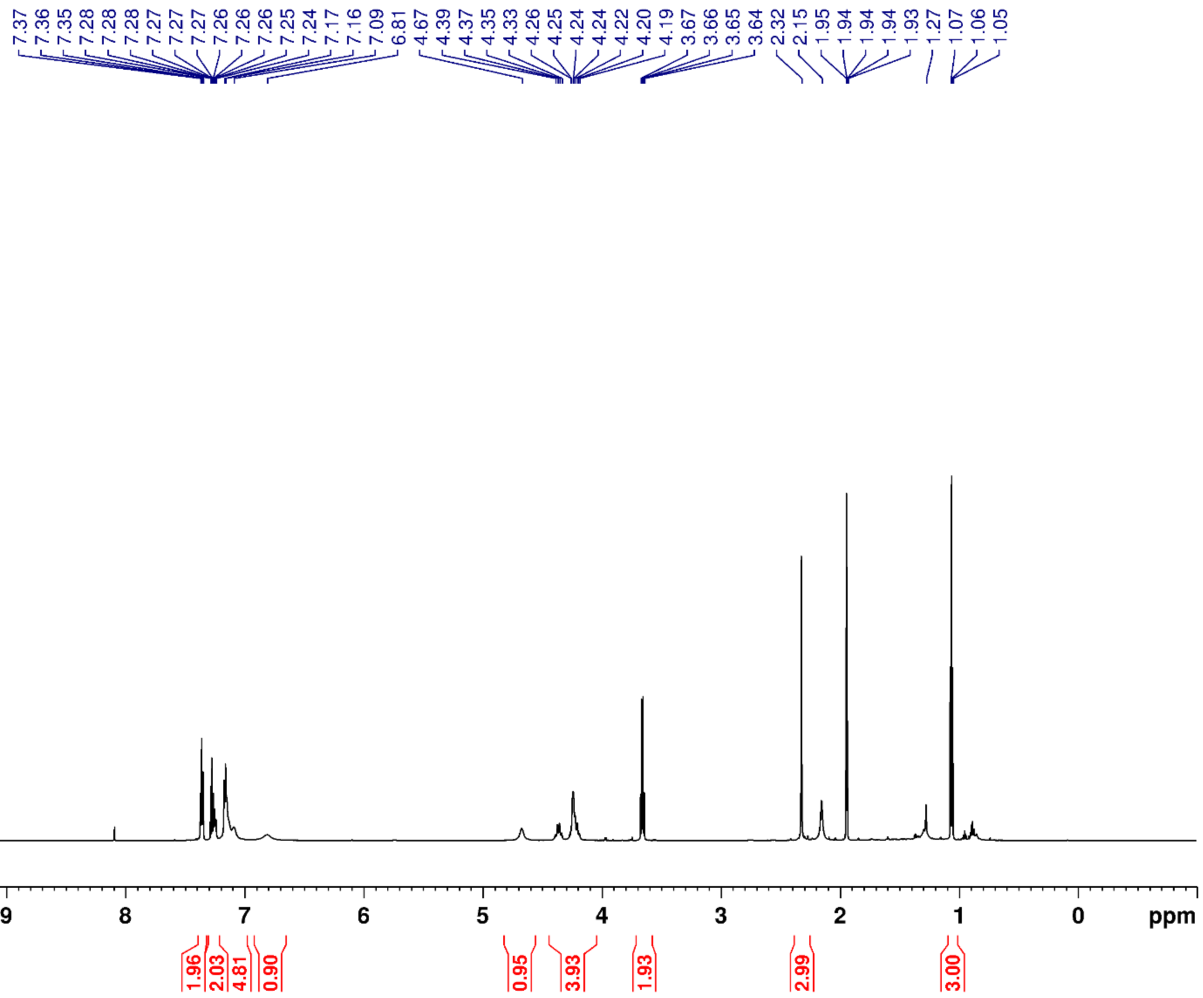

^{13}C NMR (176 MHz, CD_3CN) for 2,2,3,3,3-pentafluoropropyl (*S*)-(2-((ethyl(phenyl)carbamoyl)oxy)-1-(*m*-tolyl)ethyl)sulfamate (**11i**)

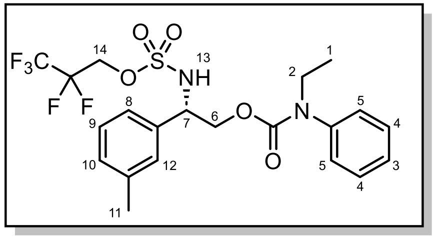

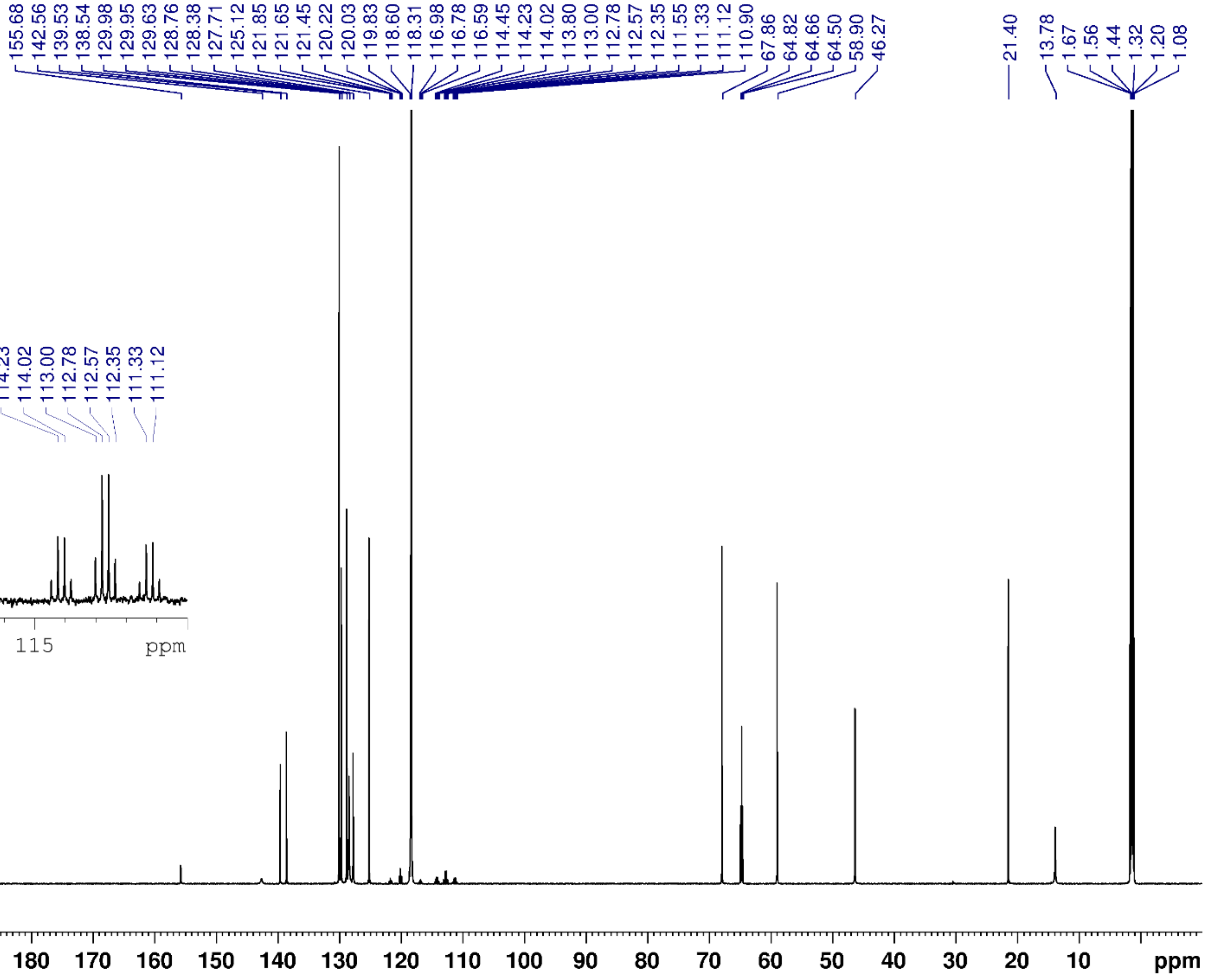

^{19}F NMR (376 MHz, CD_3CN) for 2,2,3,3,3-pentafluoropropyl (*S*)-(2-((ethyl(phenyl)carbamoyl)oxy)-1-(3-methoxyphenyl)ethyl)sulfamate (**11i**)

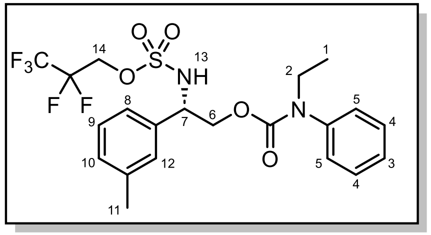

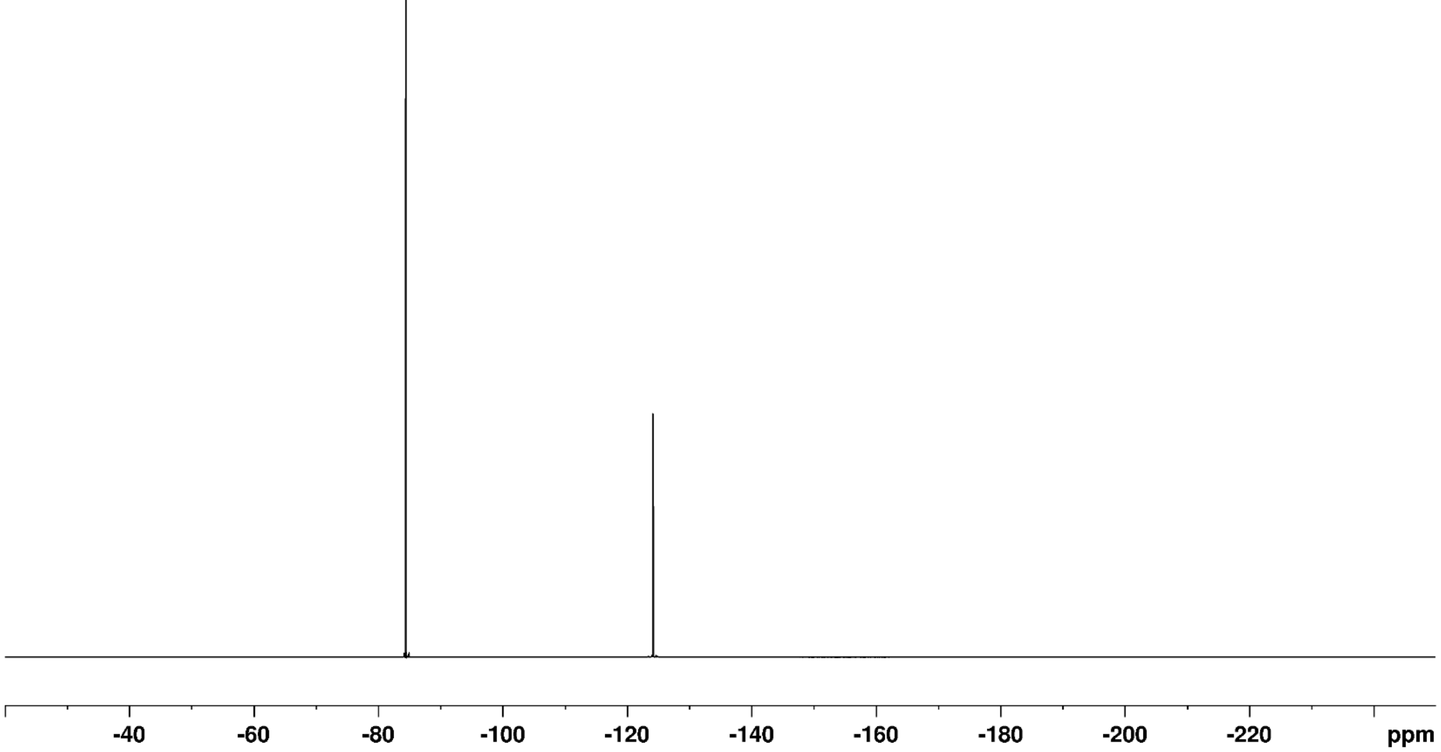

^{1}H NMR (700 MHz, CD_3CN) for 2,2,3,3,3-pentafluoropropyl (*S*)-(1-(3-chlorophenyl)-2-((ethyl(phenyl)carbamoyl)oxy)ethyl)sulfamate (**11j**)

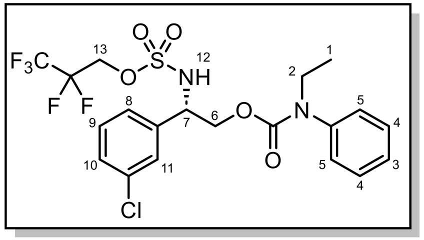

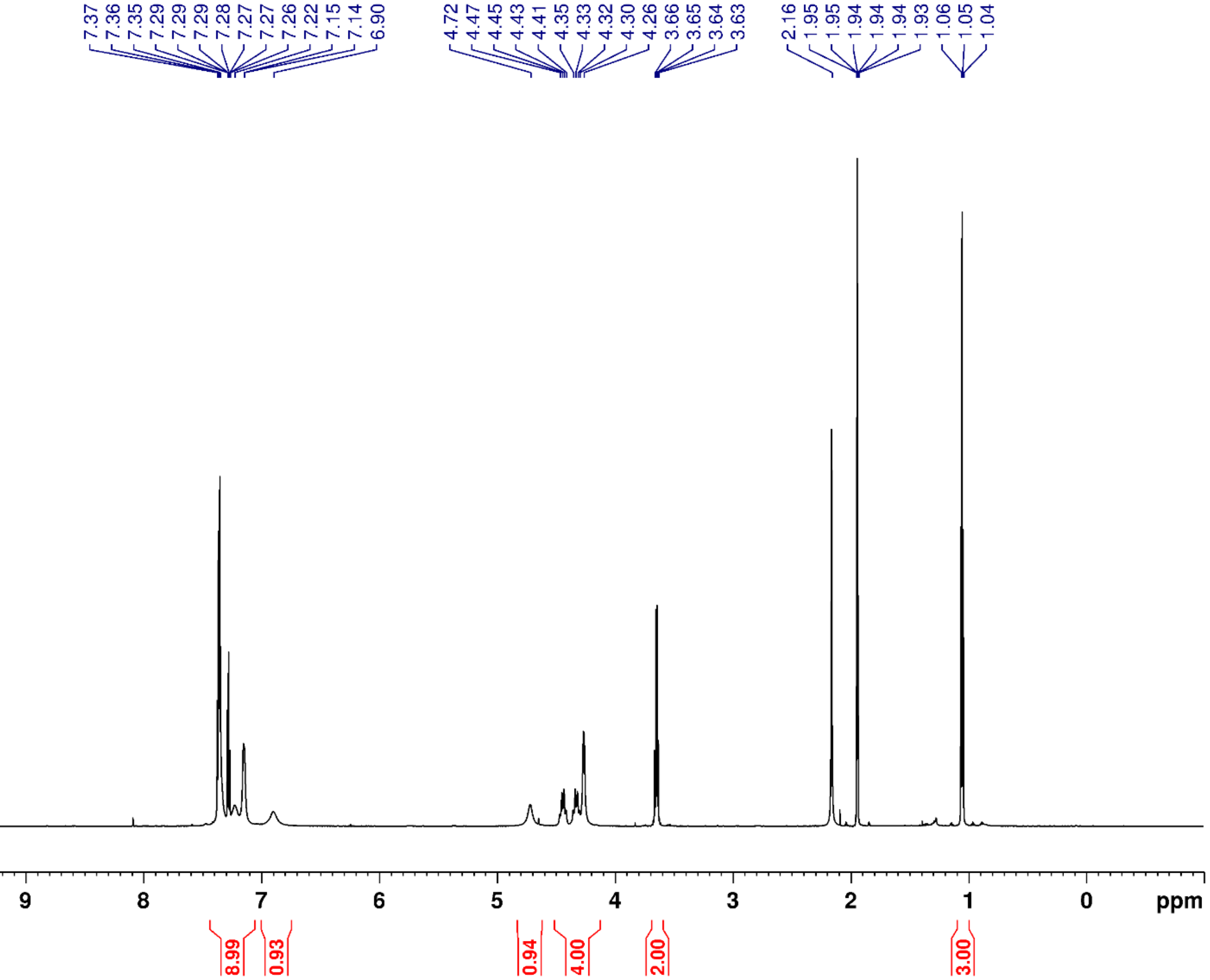

^{1}H NMR (700 MHz, CD_3CN) for 2,2,3,3,3-pentafluoropropyl (*R*)-(1-(3-chlorophenyl)-2-((ethyl(phenyl)carbamoyl)oxy)ethyl)sulfamate (***ent*-11j**)

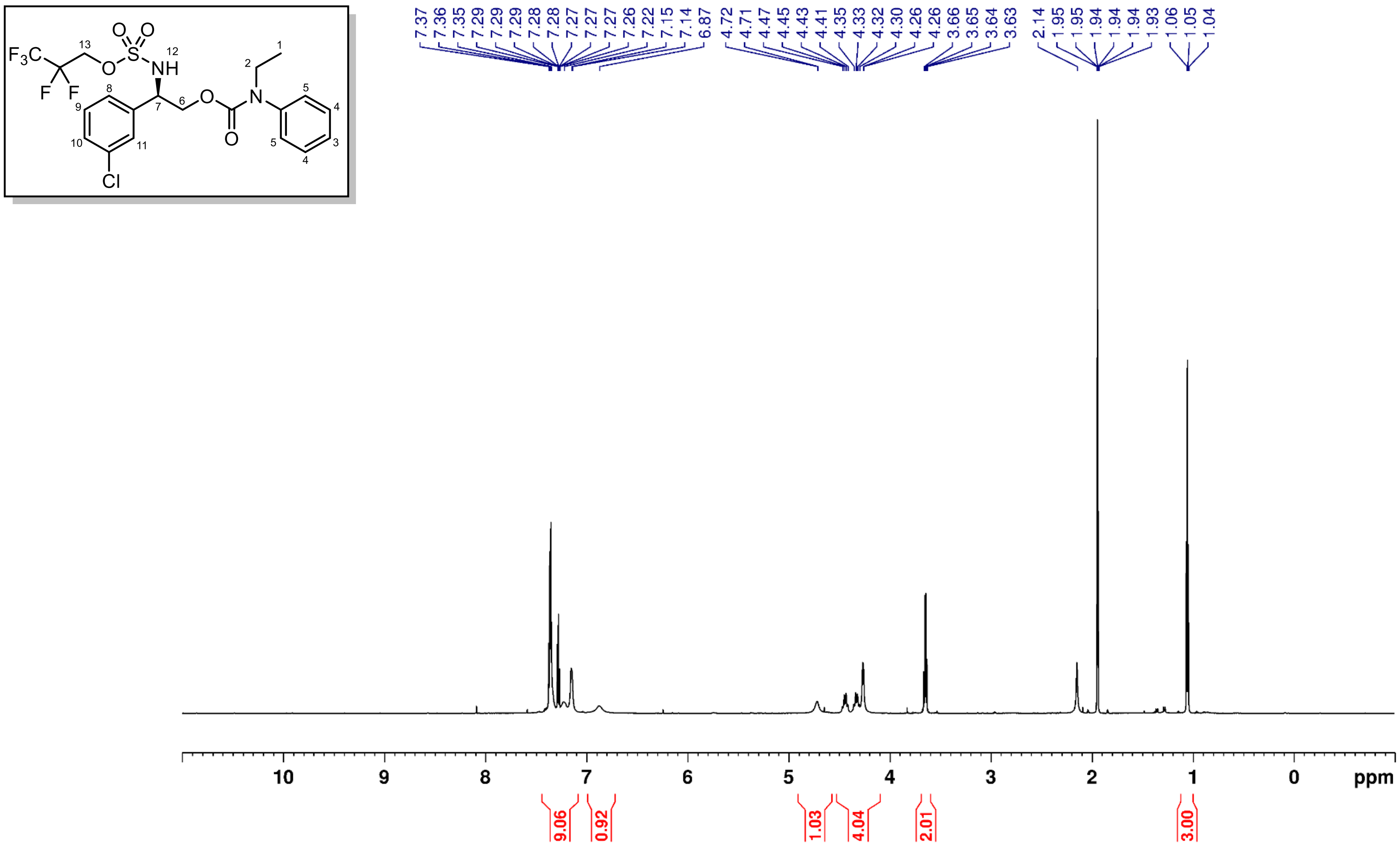

^{13}C NMR (176 MHz, CD_3CN) for 2,2,3,3,3-pentafluoropropyl (*S*)-(1-(3-chlorophenyl)-2-((ethyl(phenyl)carbamoyl)oxy)ethyl)sulfamate (**11j**)

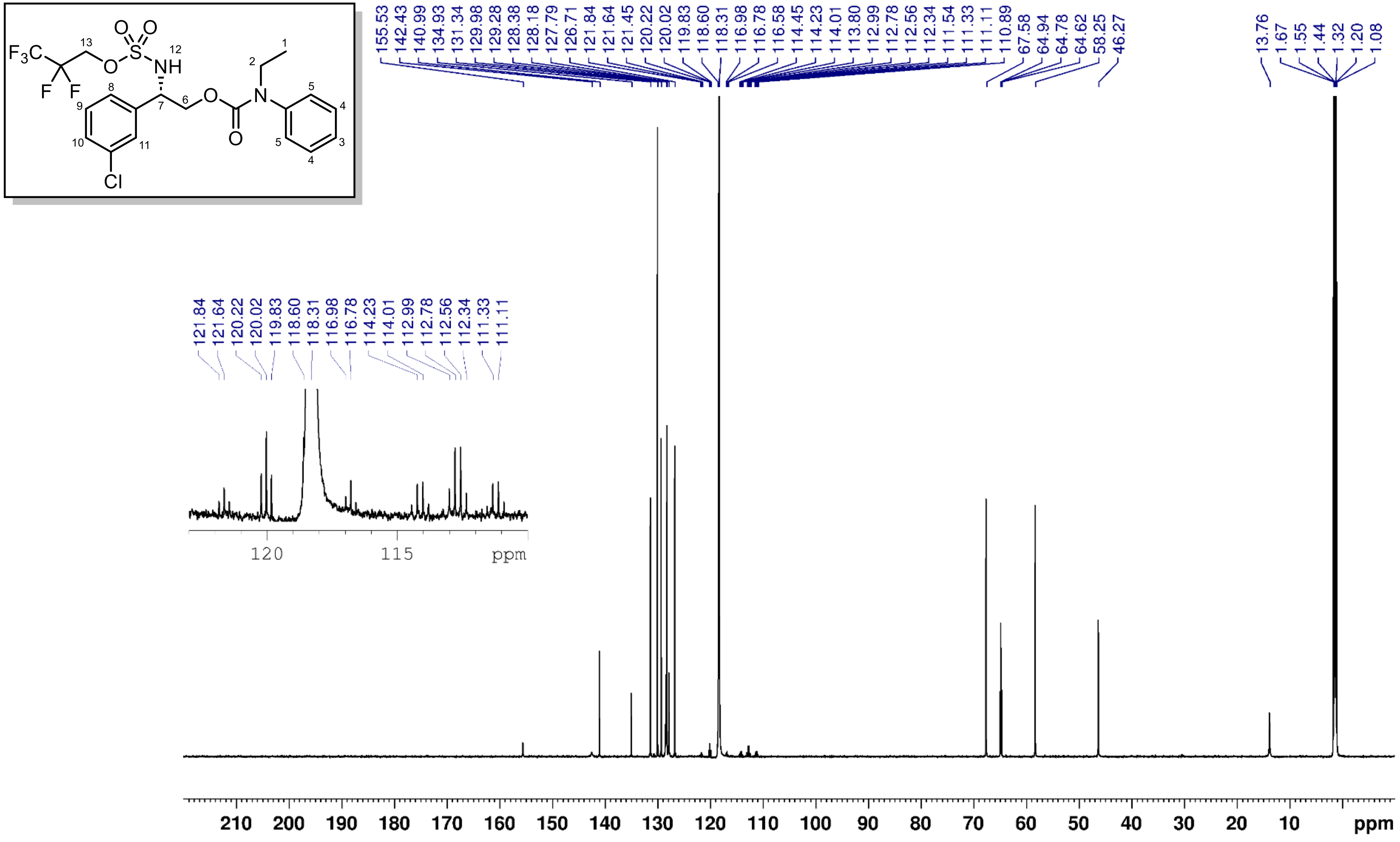

^{19}F NMR (376 MHz, CD_3CN) for 2,2,3,3,3-pentafluoropropyl (*S*)-(1-(3-chlorophenyl)-2-((ethyl(phenyl)carbamoyl)oxy)ethyl)sulfamate (**11j**)

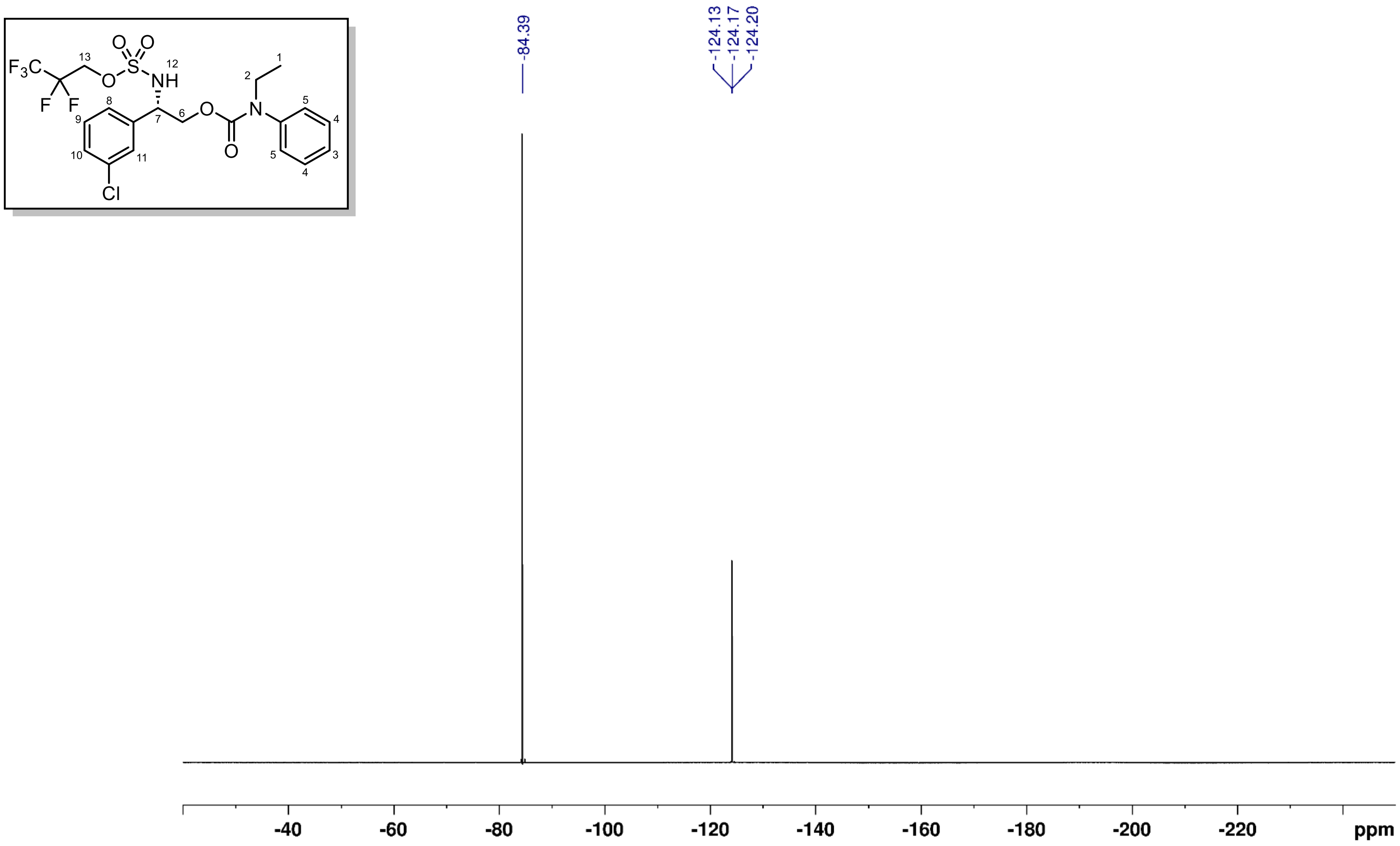

^{1}H NMR (700 MHz, CD_3CN) for 2,2,3,3,3-pentafluoropropyl (*S*)-(1-(3-bromophenyl)-2-((ethyl(phenyl)carbamoyl)oxy)ethyl)sulfamate (**11k**)

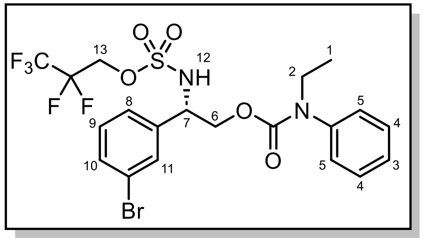

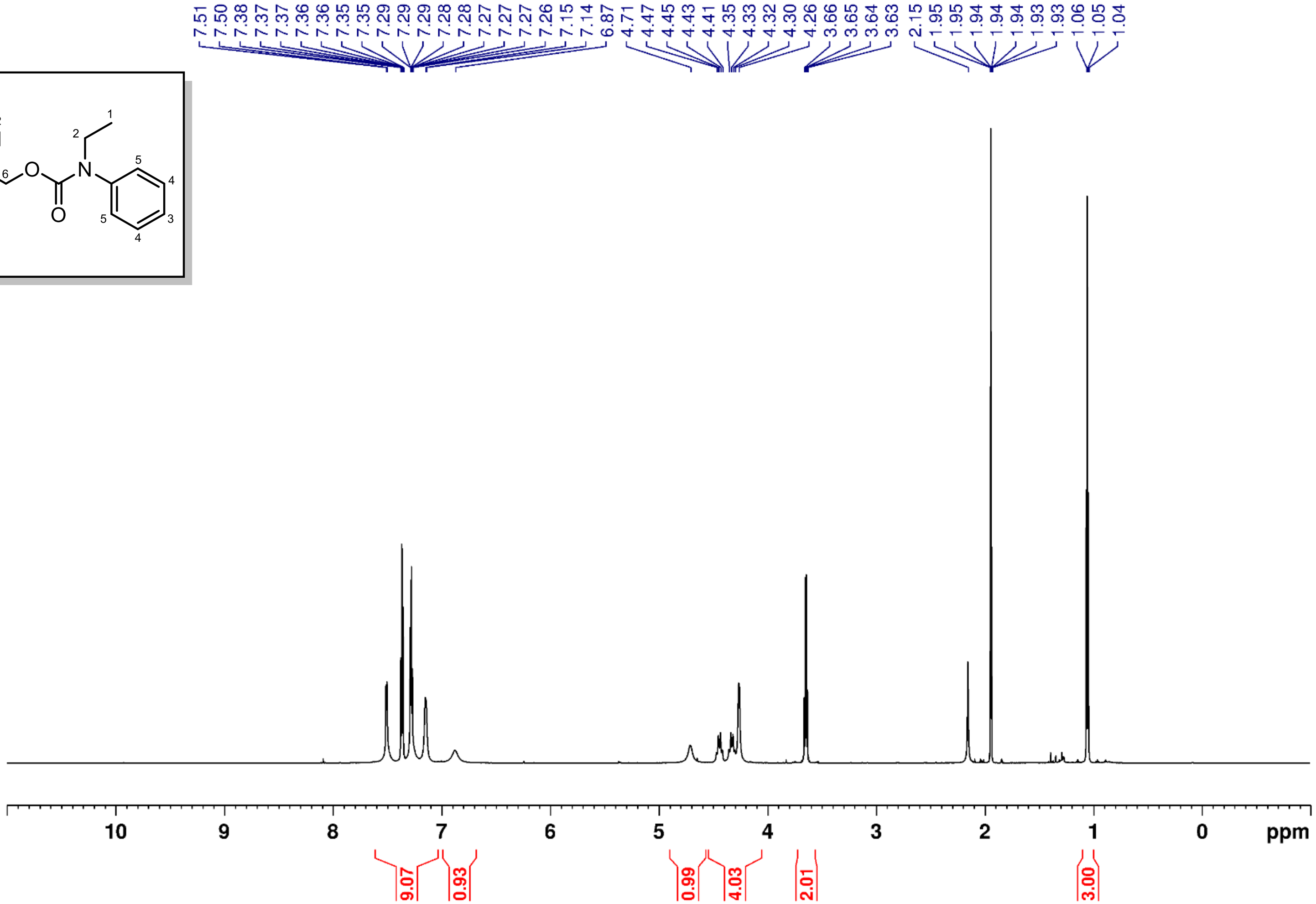

^{1}H NMR (700 MHz, CD_3CN) for 2,2,3,3,3-pentafluoropropyl (*R*)-(1-(3-bromophenyl)-2-((ethyl(phenyl)carbamoyl)oxy)ethyl)sulfamate (***ent*-11k**)

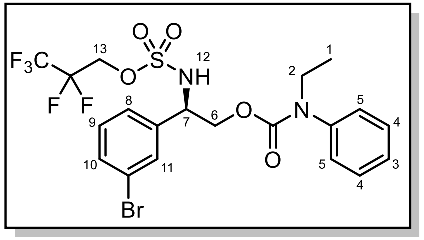

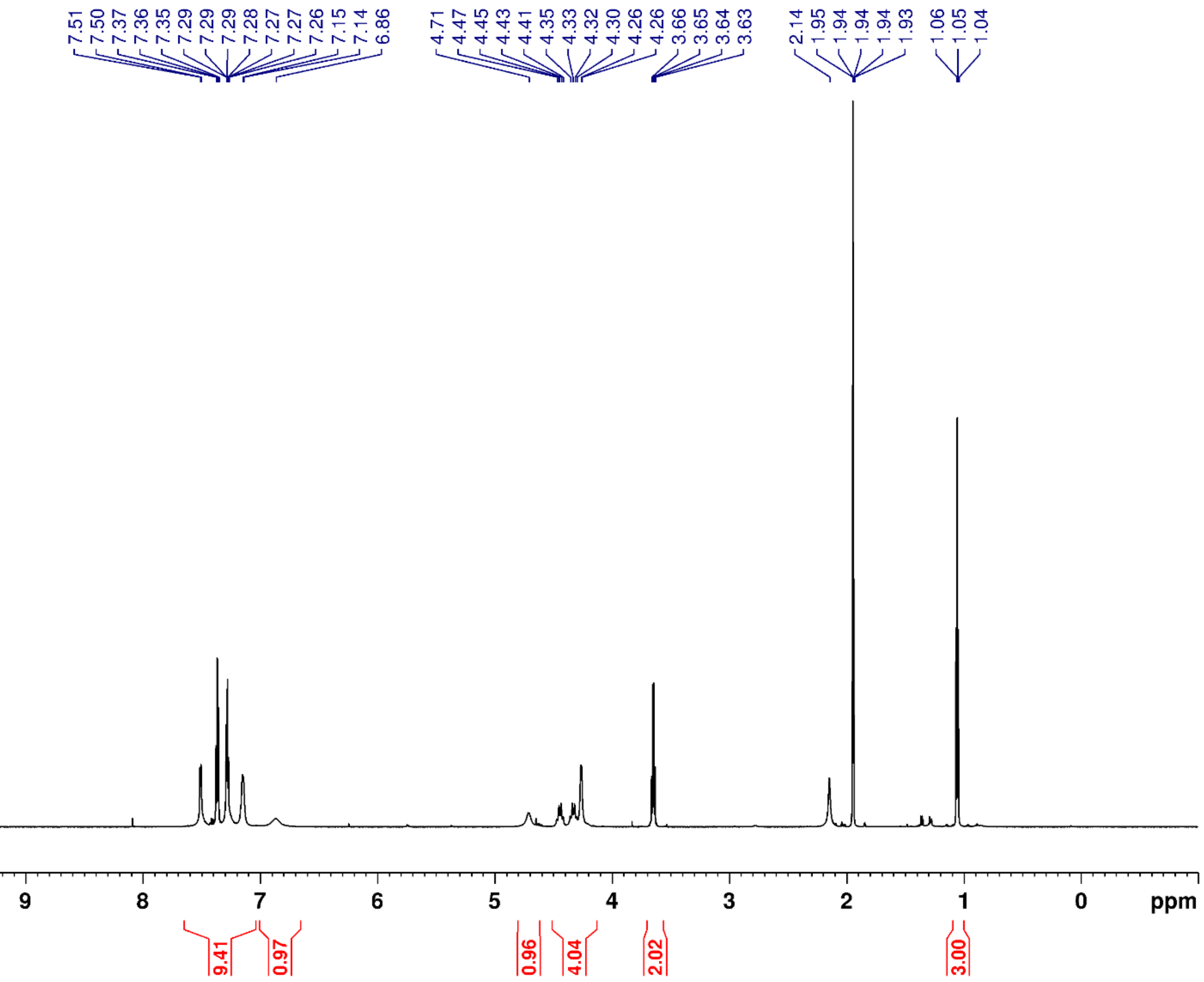

^{13}C NMR (176 MHz, CD_3CN) for 2,2,3,3,3-pentafluoropropyl (*S*)-(1-(3-bromophenyl)-2-((ethyl(phenyl)carbamoyl)oxy)ethyl)sulfamate (**11k**)

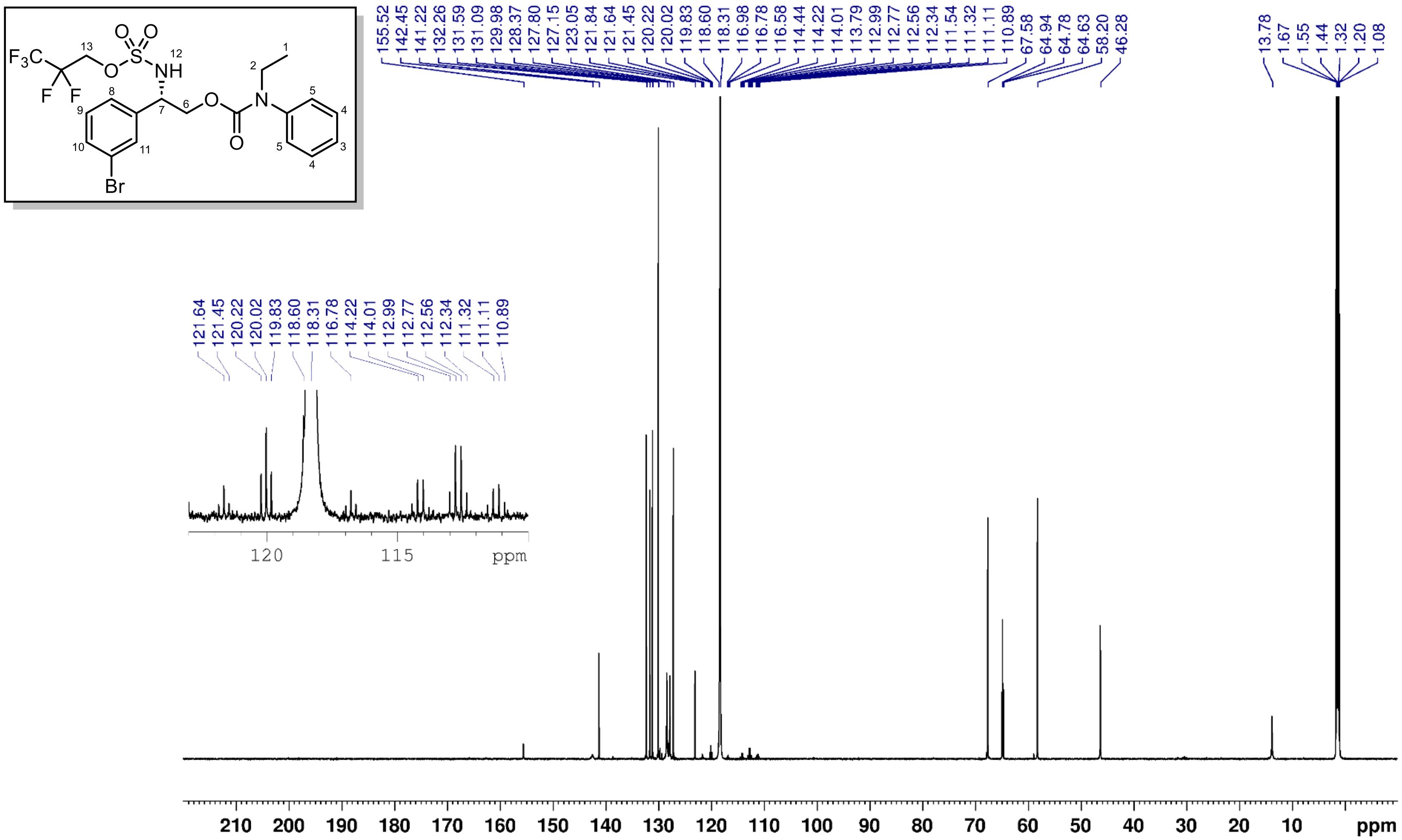

^{19}F NMR (376 MHz, CD_3CN) for 2,2,3,3,3-pentafluoropropyl (*S*)-(1-(3-bromophenyl)-2-((ethyl(phenyl)carbamoyl)oxy)ethyl)sulfamate (**11k**)

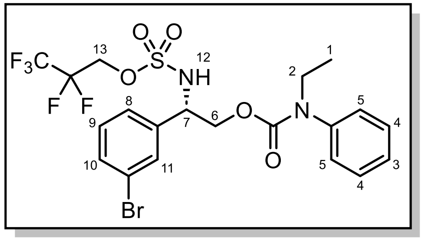

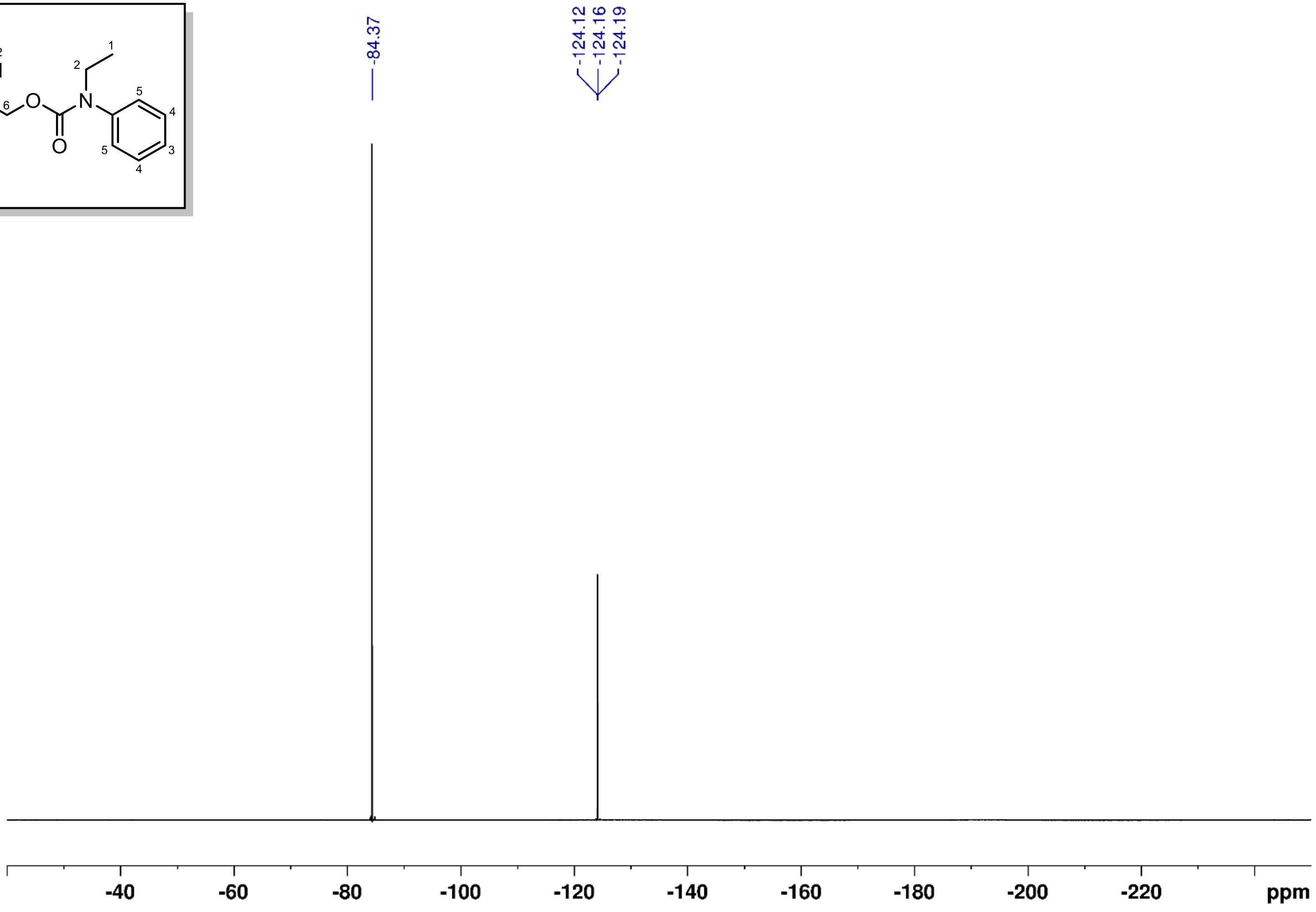

^{1}H NMR (700 MHz, CD_3CN) for 2,2,3,3,3-pentafluoropropyl (*S*)-(2-((ethyl(phenyl)carbamoyl)oxy)-1-(3-(trifluoromethyl)phenyl)ethyl)sulfamate (**11l**)

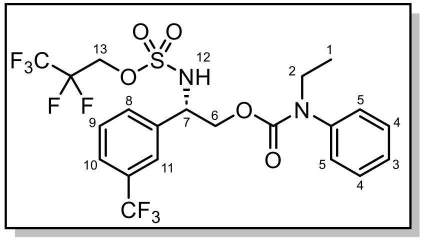

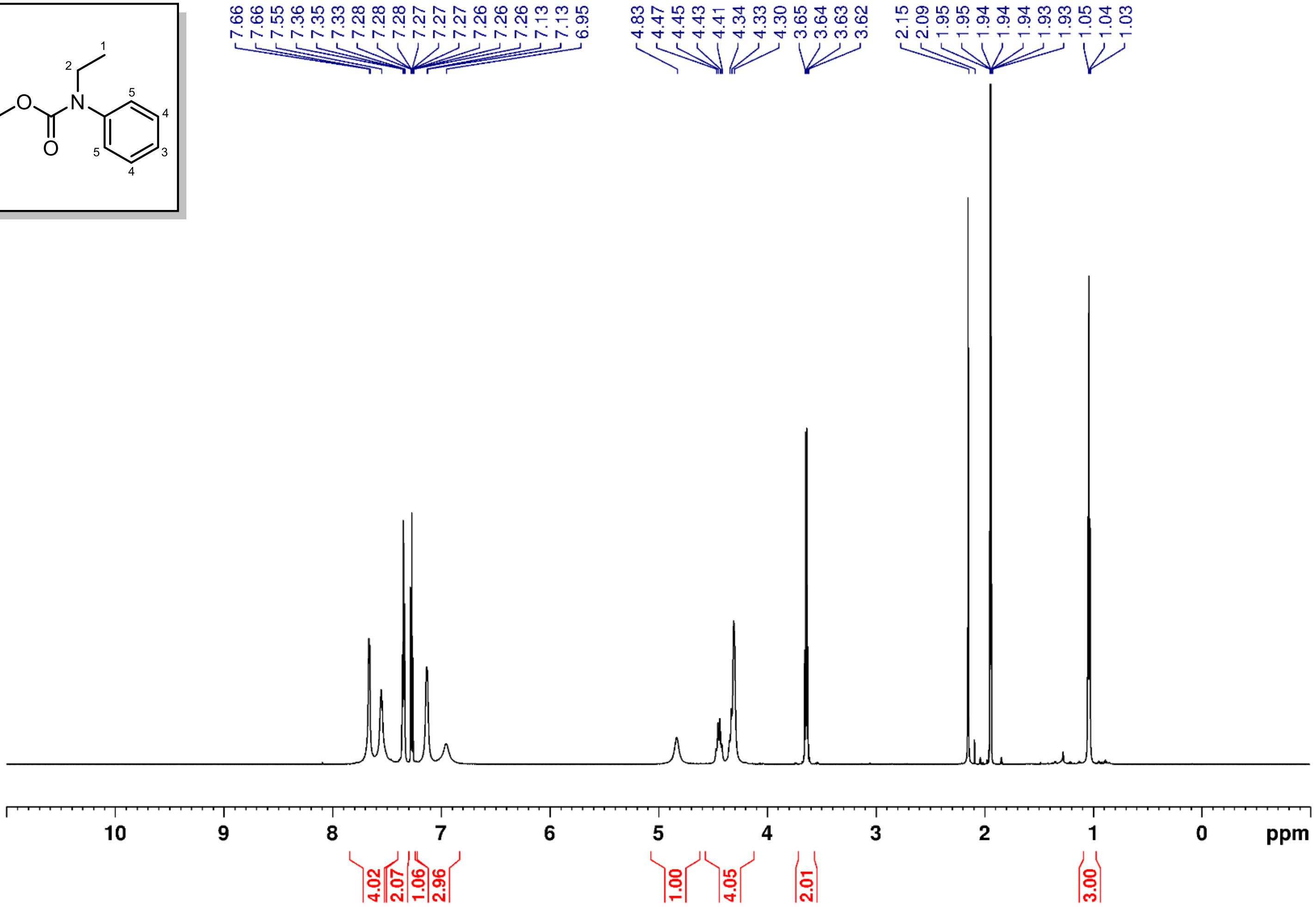

¹H NMR (700 MHz, CD_3CN) for 2,2,3,3,3-pentafluoropropyl (*R*)-(2-((ethyl(phenyl)carbamoyl)oxy)-1-(3-(trifluoromethyl)phenyl)ethyl)sulfamate (***ent*-11l**)

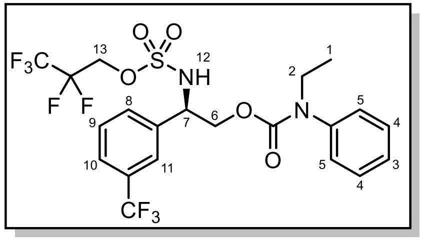

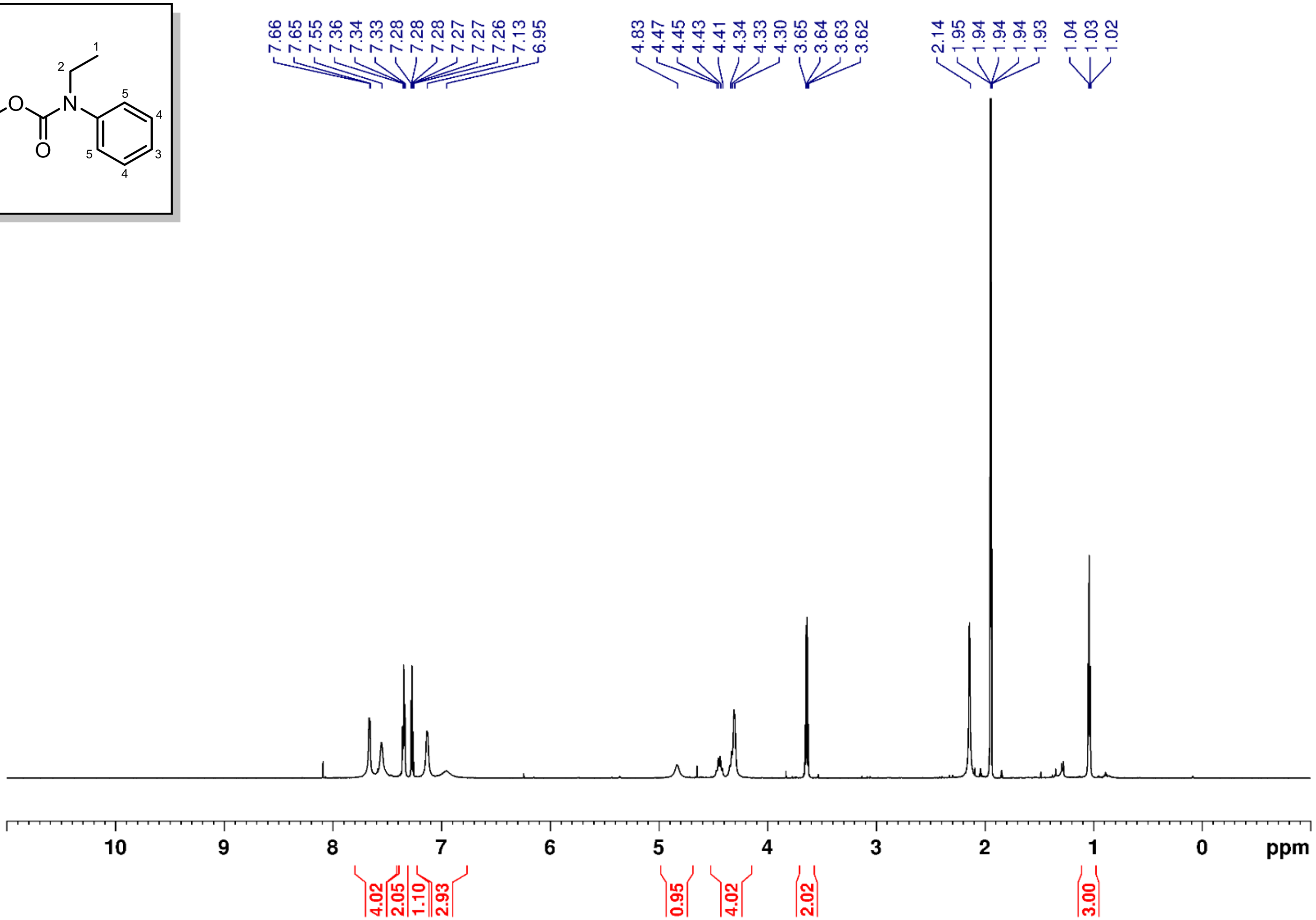

^{13}C NMR (176 MHz, CD_3CN) for 2,2,3,3,3-pentafluoropropyl (*S*)-(2-((ethyl(phenyl)carbamoyl)oxy)-1-(3-(trifluoromethyl)phenyl)ethyl)sulfamate (**11l**)

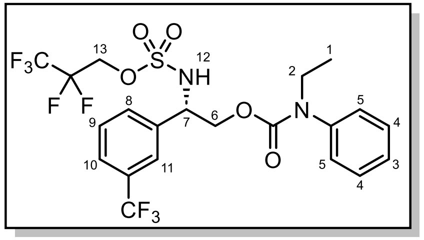

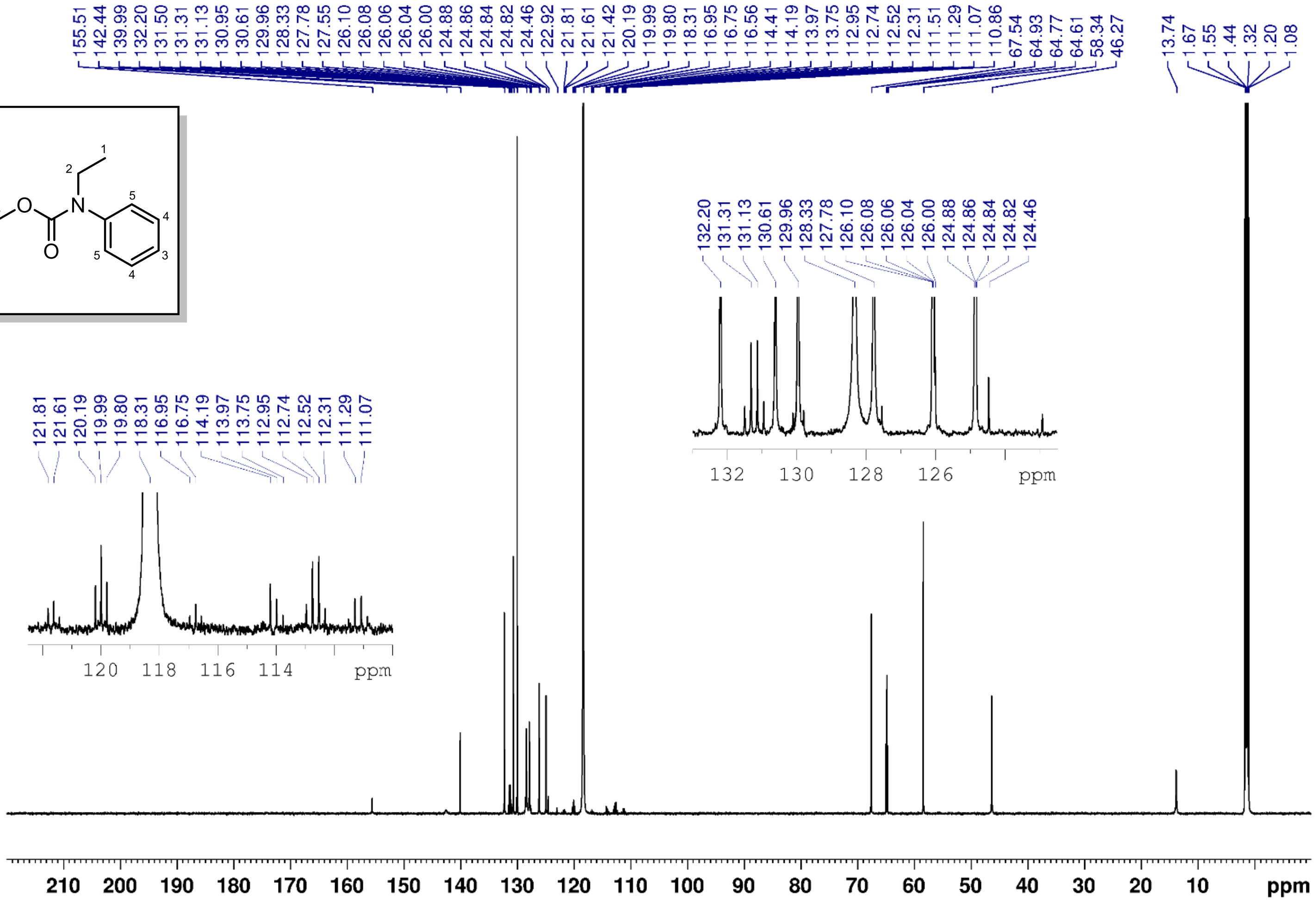

^{19}F NMR (376 MHz, CD_3CN) for 2,2,3,3,3-pentafluoropropyl (*S*)-(2-((ethyl(phenyl)carbamoyl)oxy)-1-(3-(trifluoromethyl)phenyl)ethyl)sulfamate (**11l**)

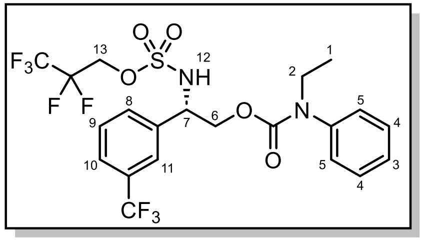

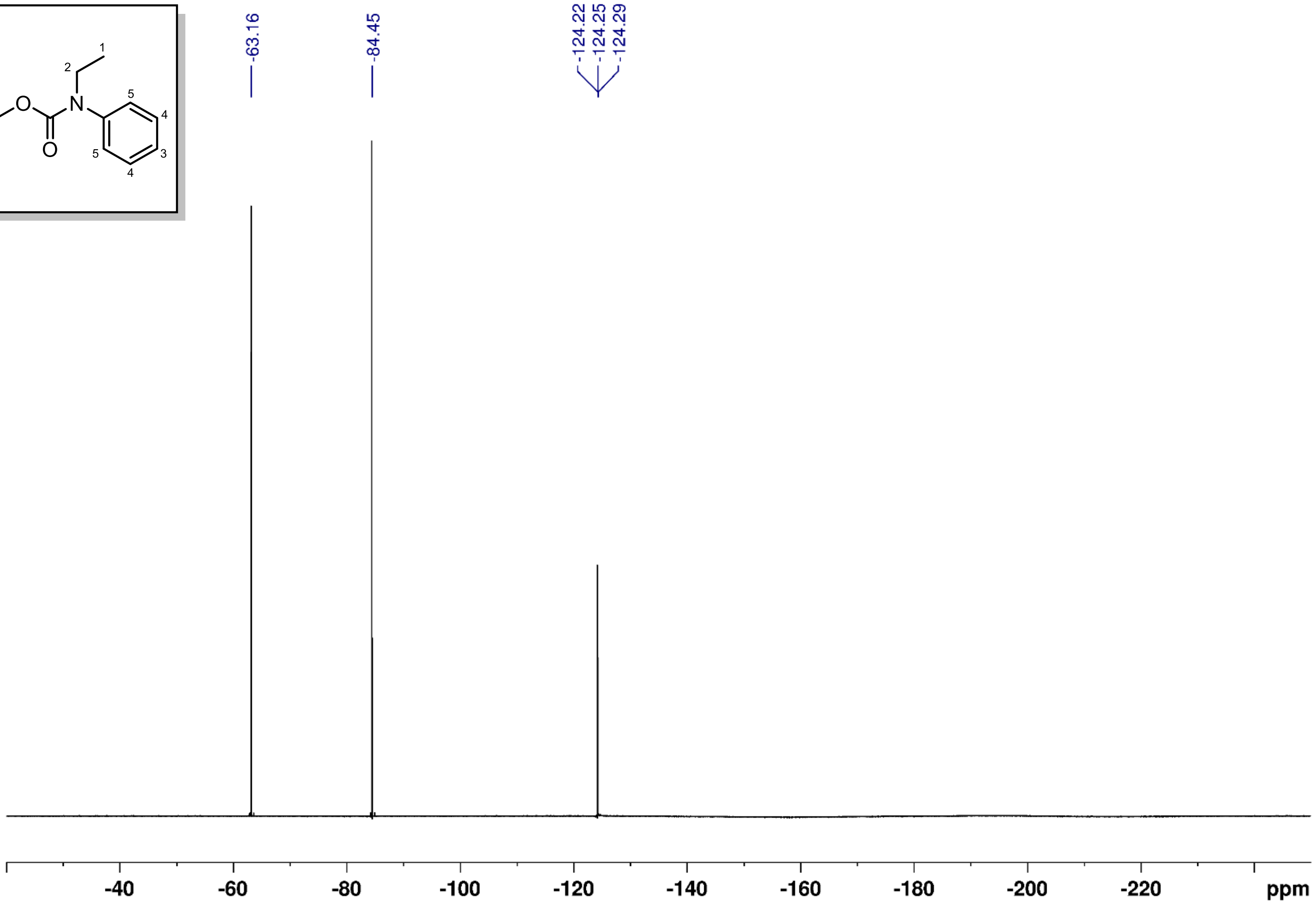

¹H NMR (700 MHz, CD_3CN) for 2,2,3,3,3-pentafluoropropyl (*S*)-(2-((ethyl(phenyl)carbamoyl)oxy)-1-(*o*-tolyl)ethyl)sulfamate (**11m**)

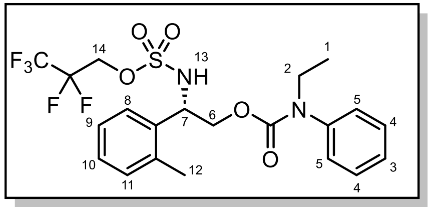

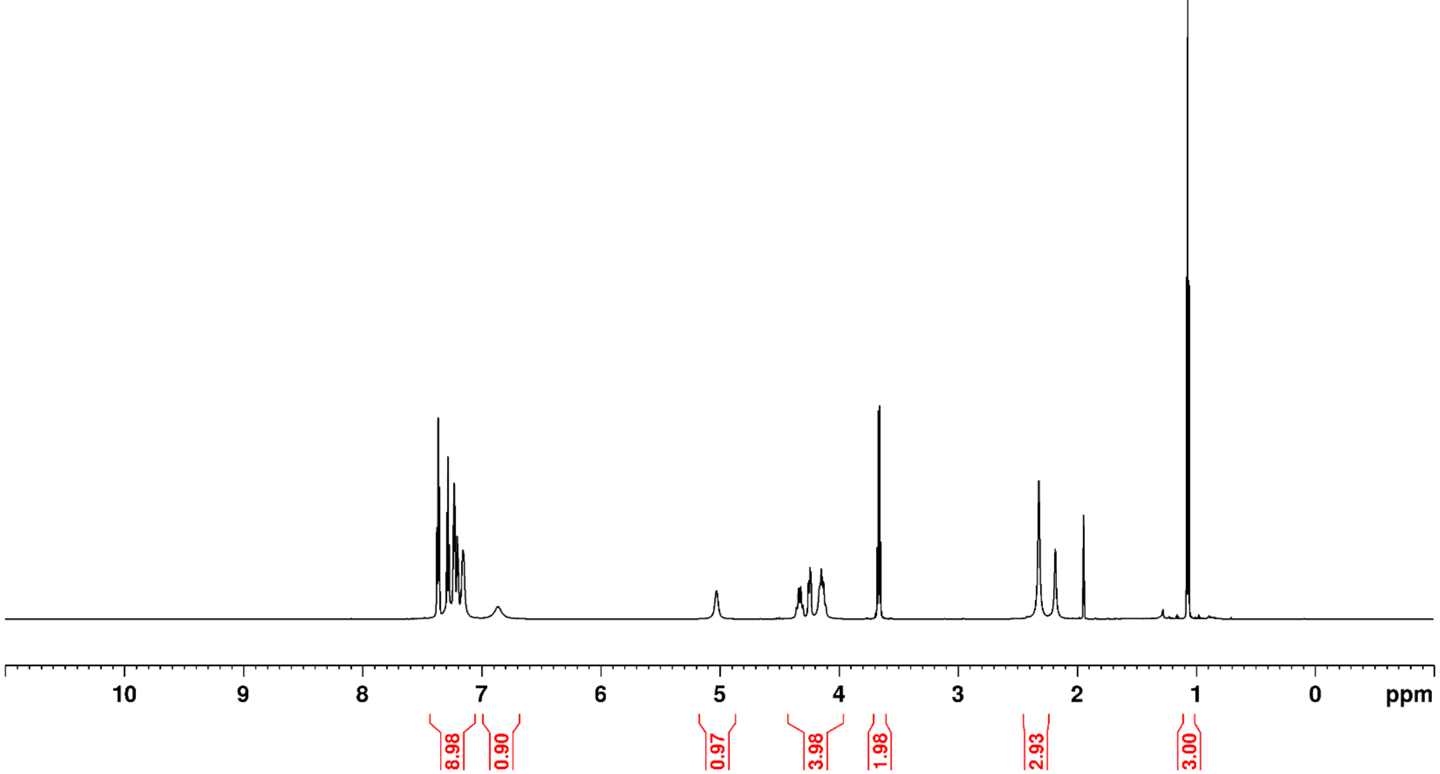

^{1}H NMR (700 MHz, CD_3CN) for 2,2,3,3,3-pentafluoropropyl (*R*)-(2-((ethyl(phenyl)carbamoyl)oxy)-1-(*o*-tolyl)ethyl)sulfamate (***ent*-11m**)

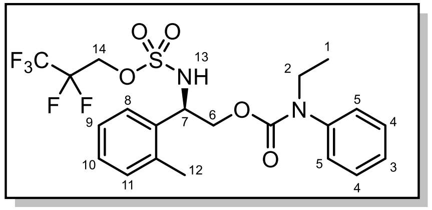

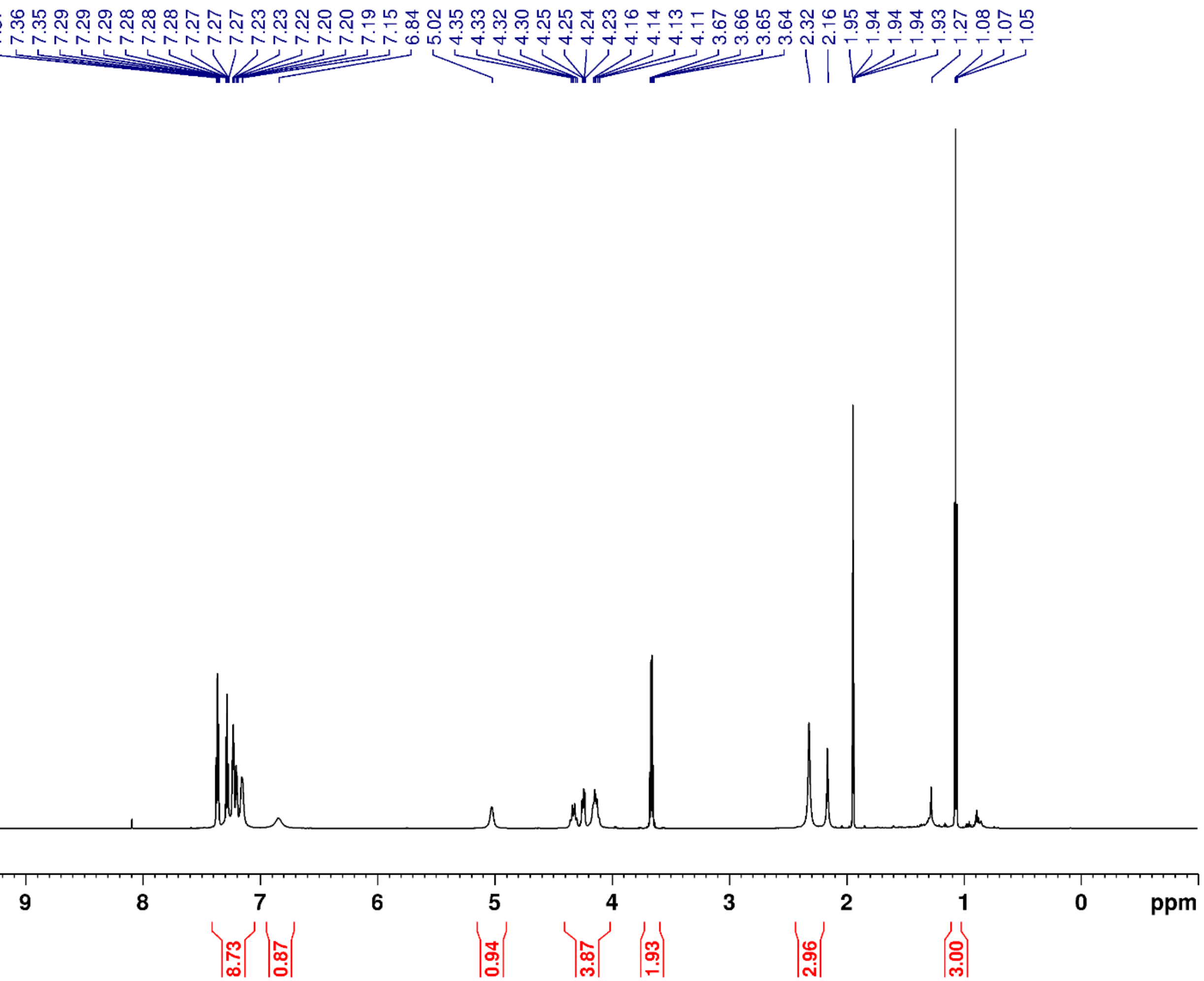

^{13}C NMR (176 MHz, CD_3CN) for 2,2,3,3,3-pentafluoropropyl (*S*)-(2-((ethyl(phenyl)carbamoyl)oxy)-1-(*o*-tolyl)ethyl)sulfamate (**11m**)

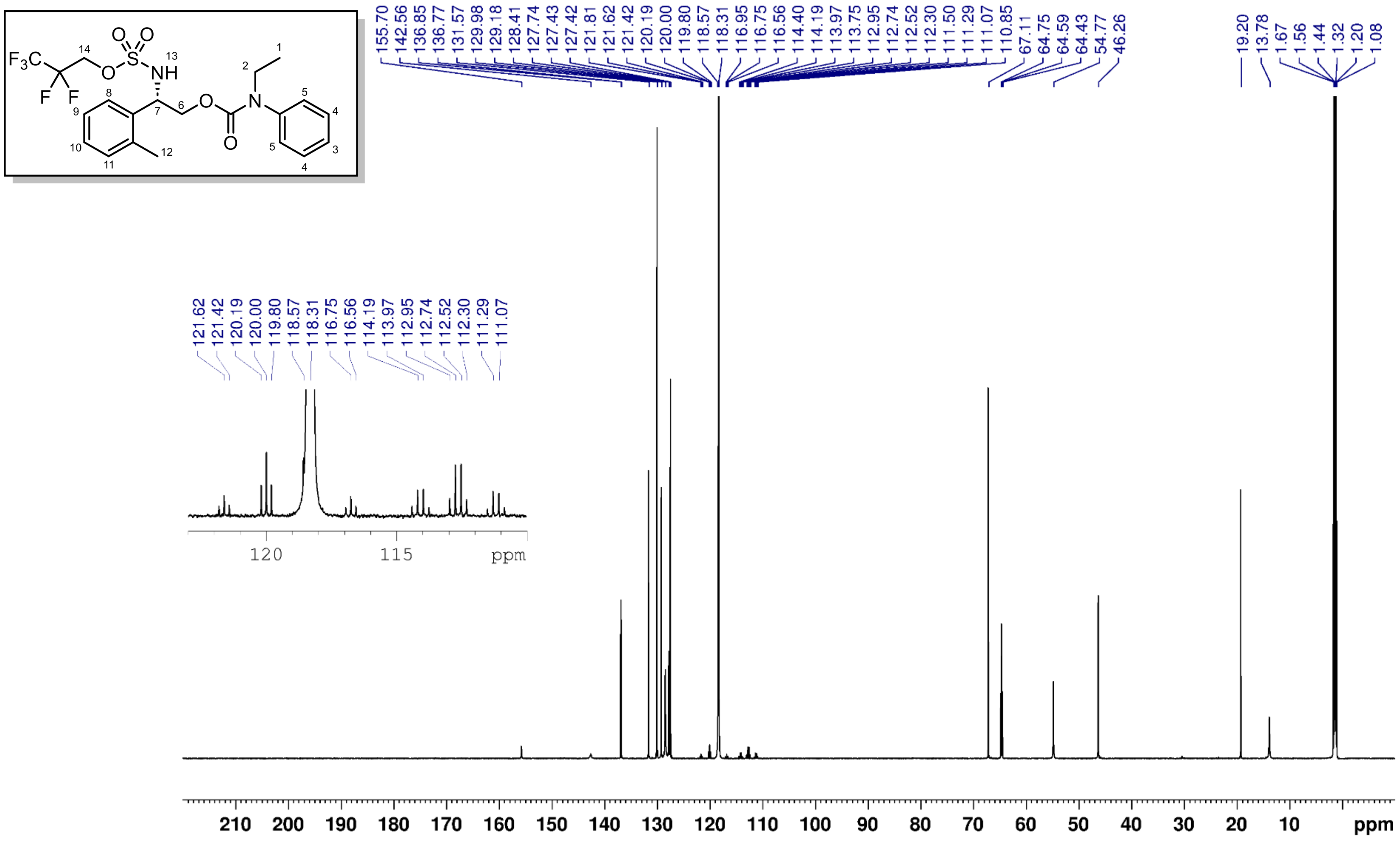

^{19}F NMR (376 MHz, CD_3CN) for 2,2,3,3,3-pentafluoropropyl (*S*)-(2-((ethyl(phenyl)carbamoyl)oxy) -1-(*o*-tolyl)ethyl)sulfamate (**11m**)

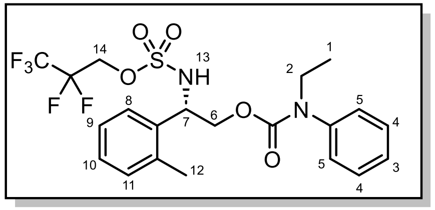

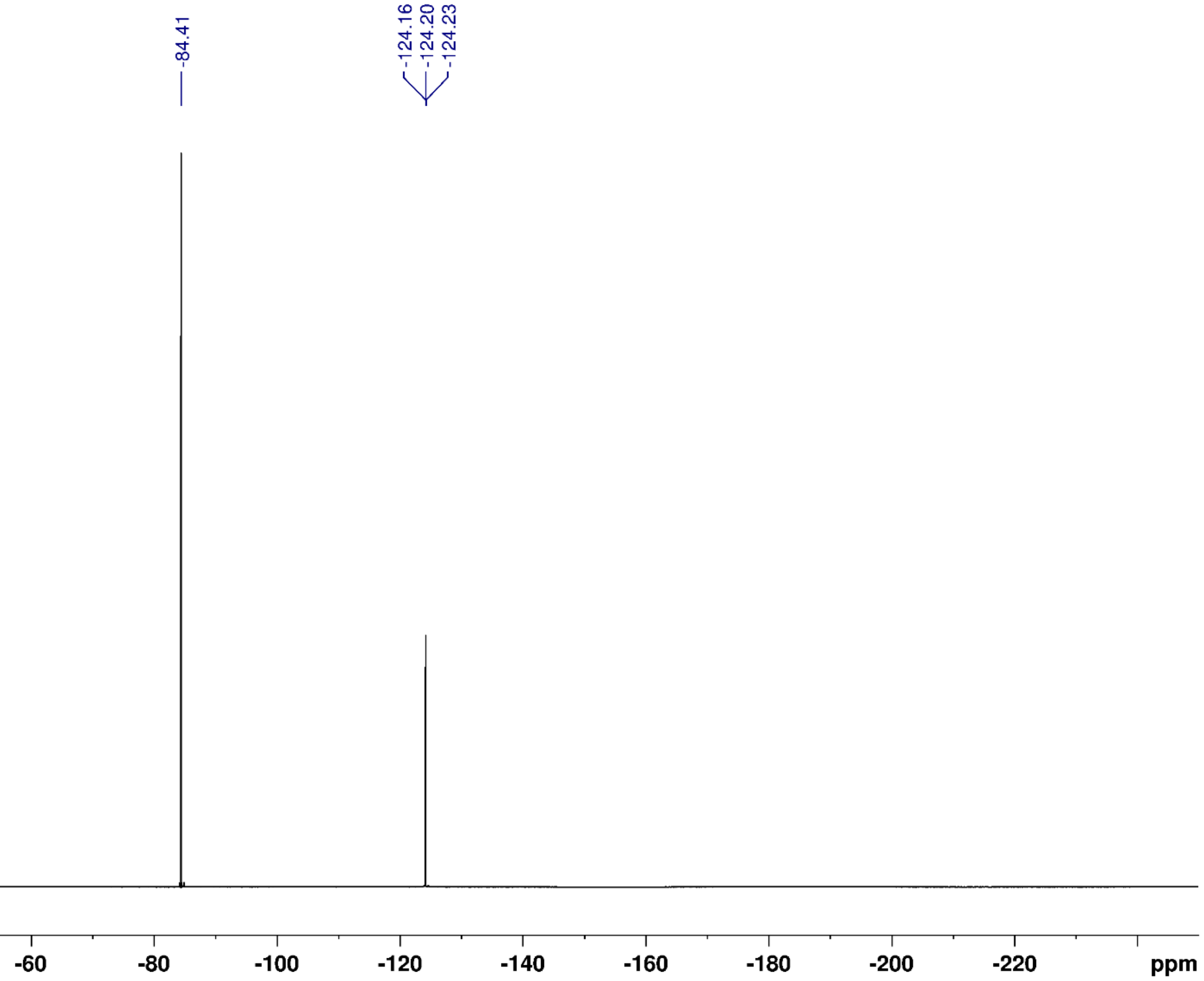

^{1}H NMR (700 MHz, CD_3CN) for 2,2,3,3,3-pentafluoropropyl (*S*)-(1-(2-chlorophenyl)-2-((ethyl(phenyl)carbamoyl)oxy)ethyl)sulfamate (**11n**)

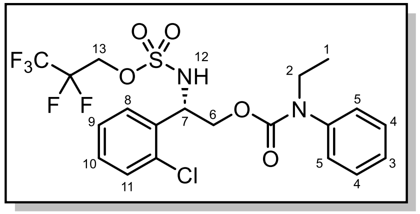

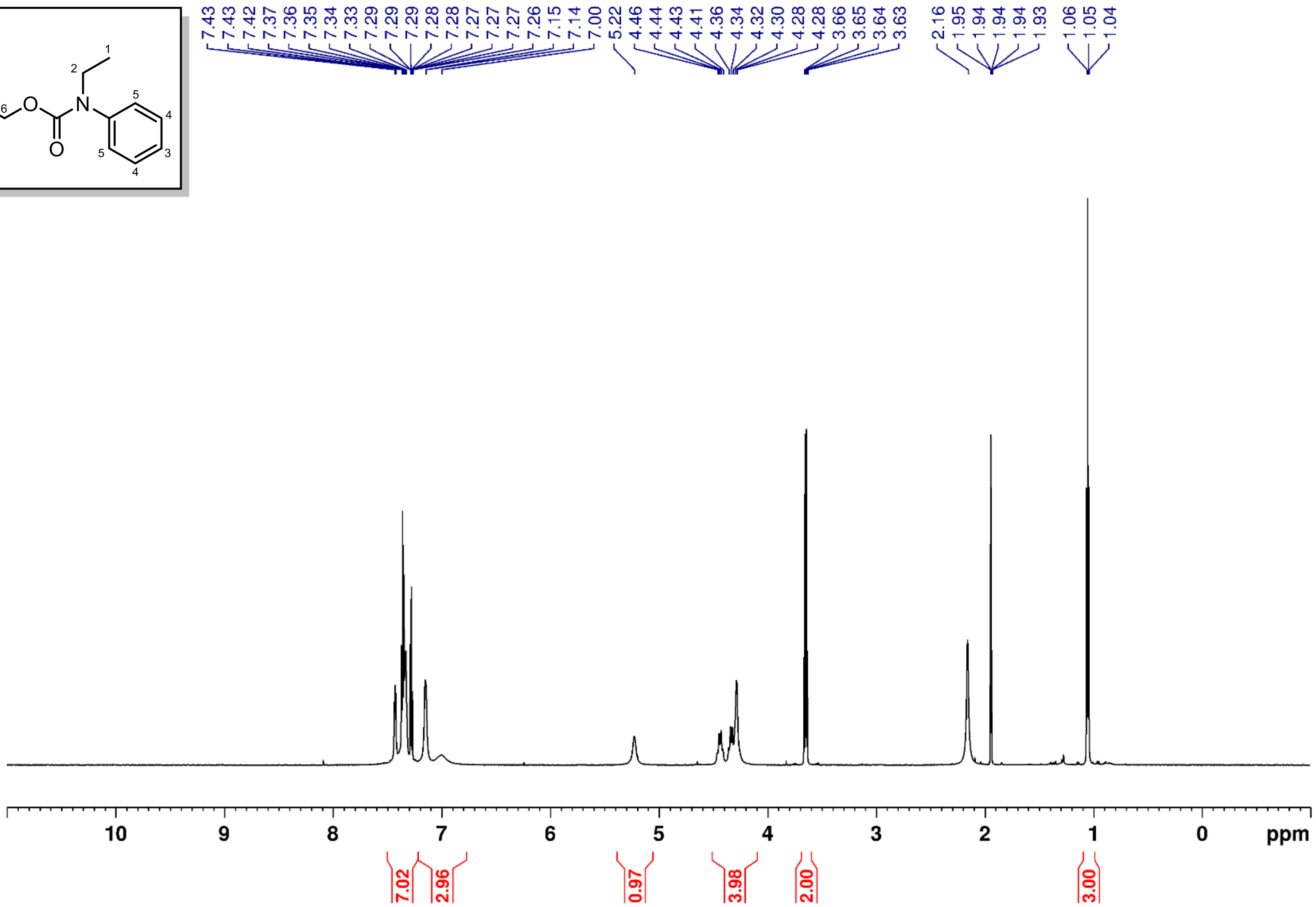

^{1}H NMR (700 MHz, CD_3CN) for 2,2,3,3,3-pentafluoropropyl (*R*)-(1-(2-chlorophenyl)-2-((ethyl(phenyl)carbamoyl)oxy)ethyl)sulfamate (***ent*-11n**)

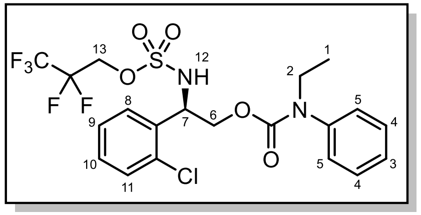

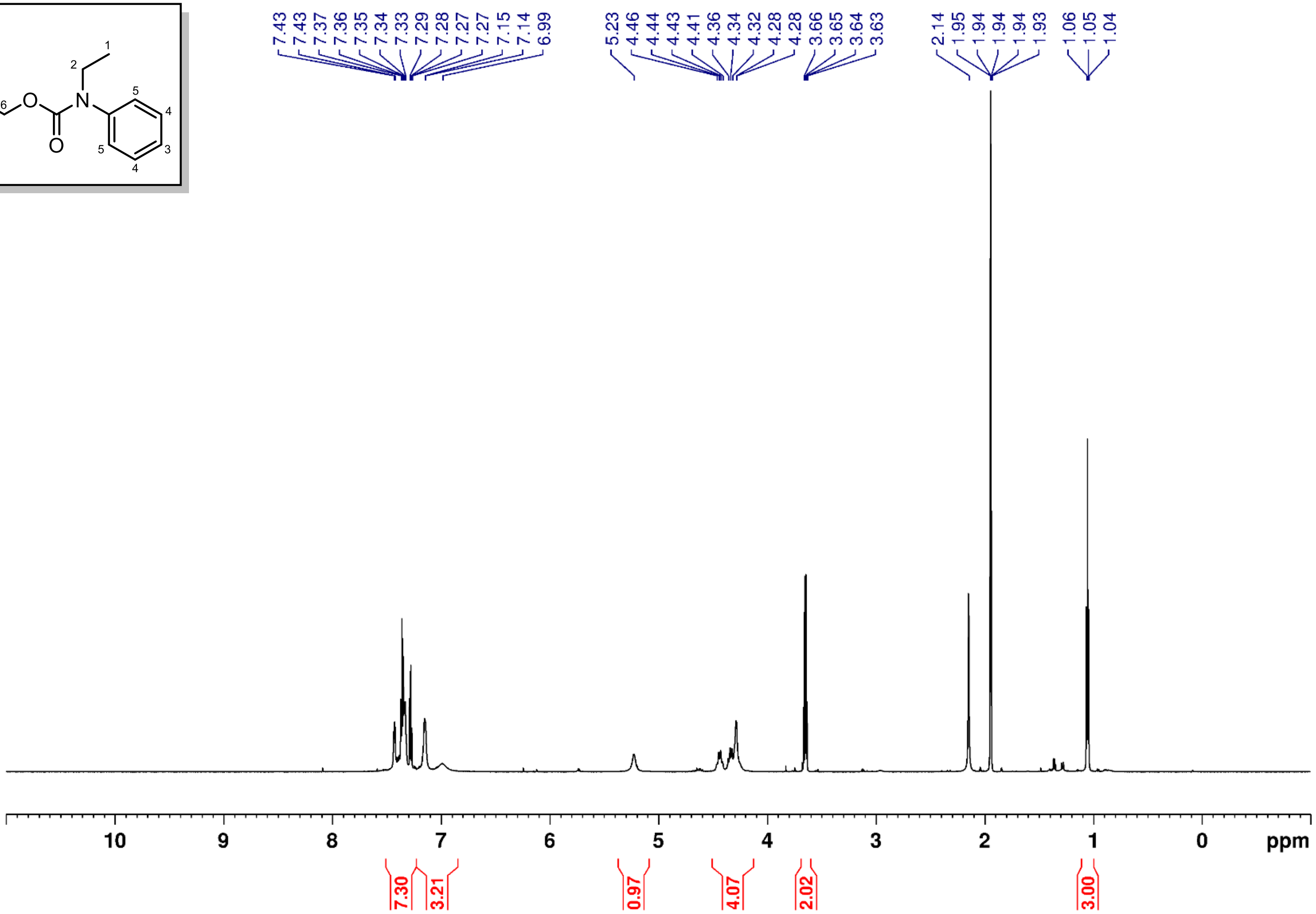

^{13}C NMR (176 MHz, CD_3CN) for 2,2,3,3,3-pentafluoropropyl (*S*)-(1-(2-chlorophenyl)-2-((ethyl(phenyl)carbamoyl)oxy)ethyl)sulfamate (**11n**)

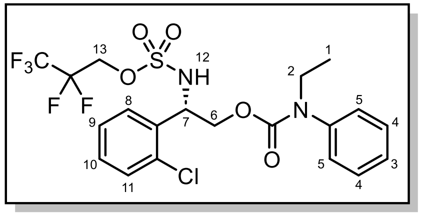

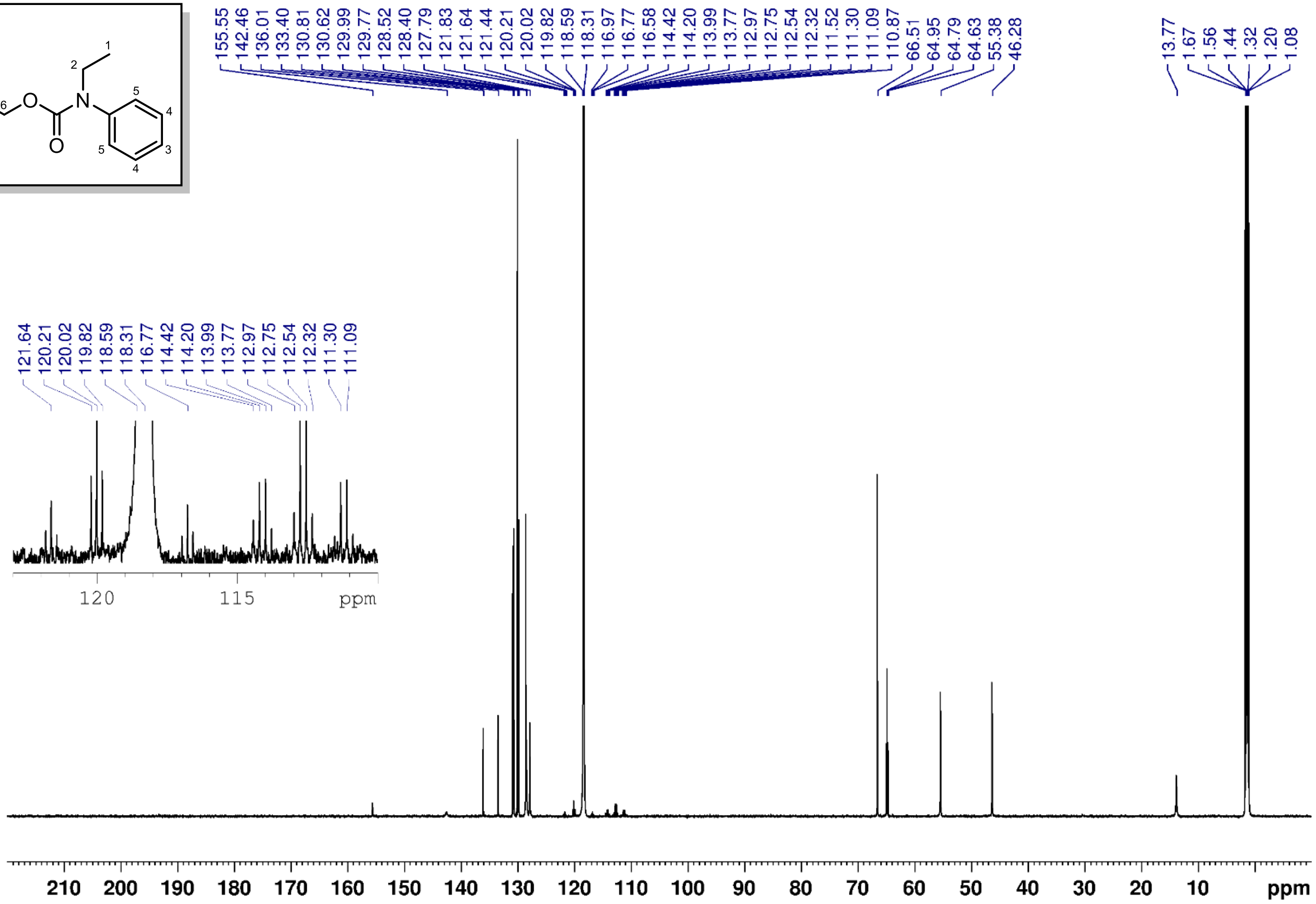

^{19}F NMR (376 MHz, CD_3CN) for 2,2,3,3,3-pentafluoropropyl (*S*)-(1-(2-chlorophenyl)-2-((ethyl(phenyl)carbamoyl)oxy)ethyl)sulfamate (**11n**)

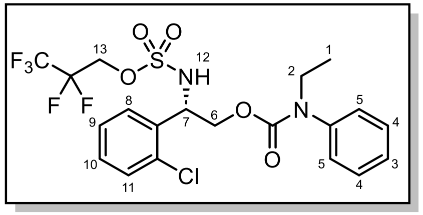

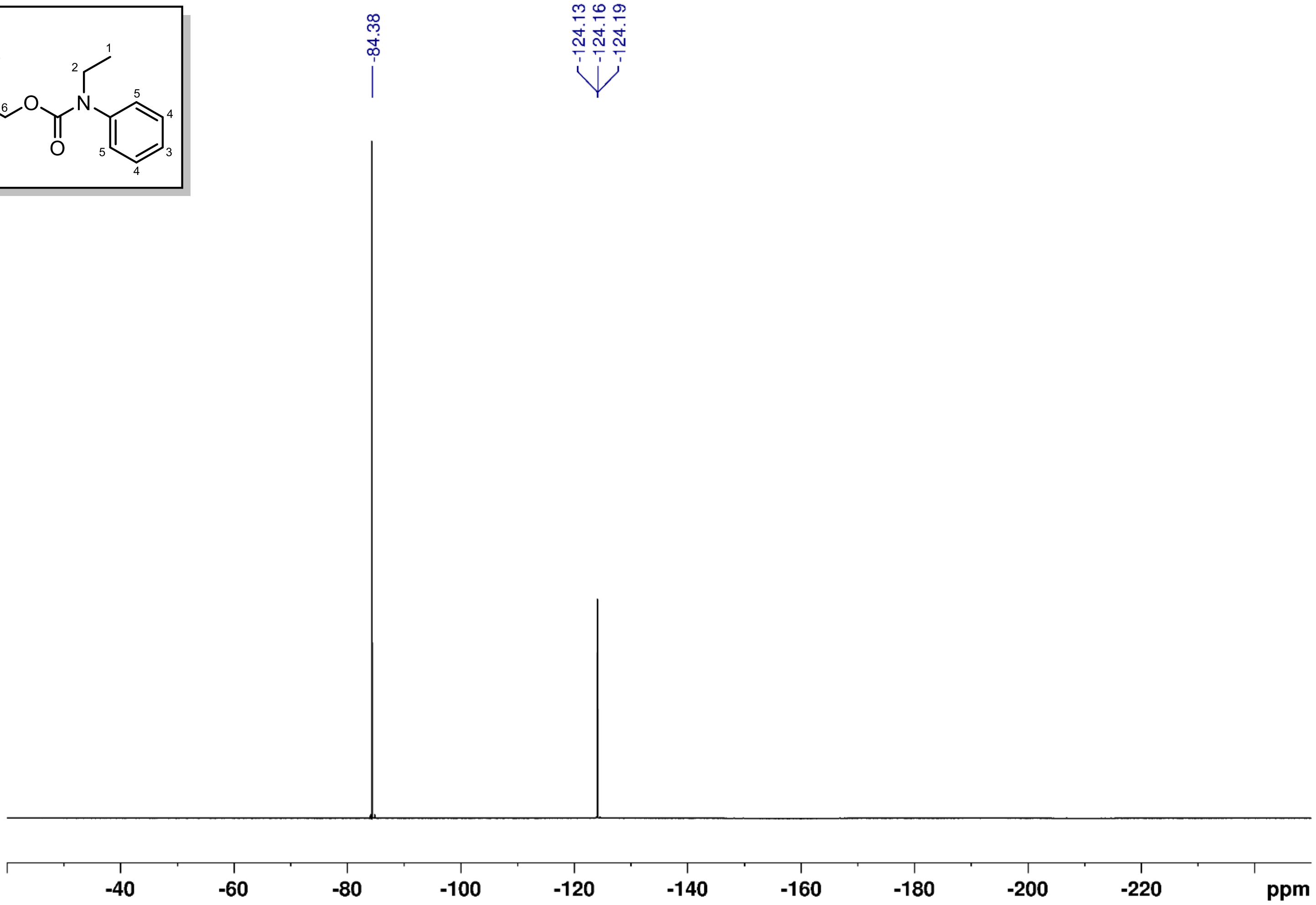

^{1}H NMR (700 MHz, CD_3CN) for 2,2,3,3,3-pentafluoropropyl (*S*)-(1-(2-bromophenyl)-2-((ethyl(phenyl)carbamoyl)oxy)ethyl)sulfamate (**11o**)

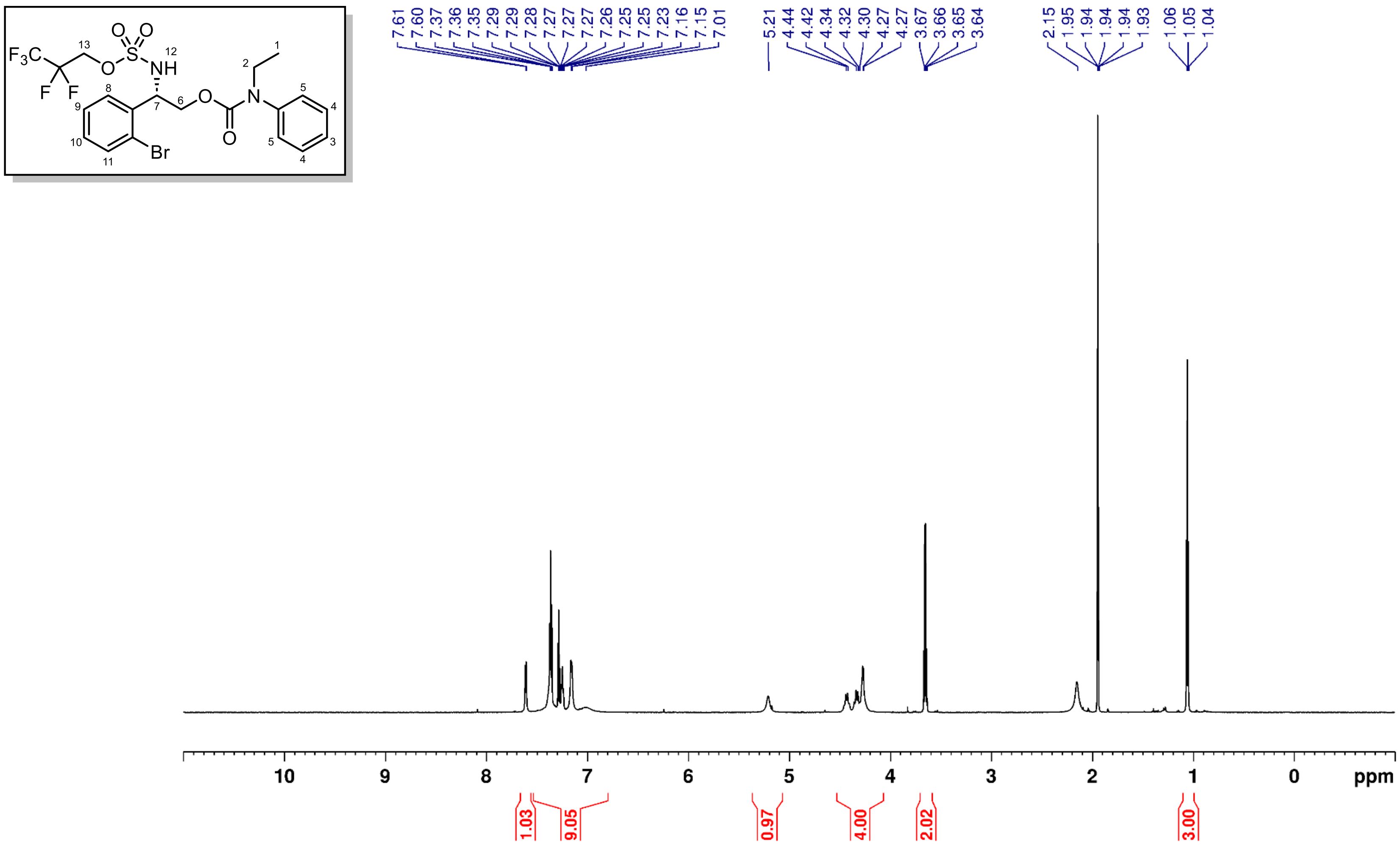

^{1}H NMR (700 MHz, CD_3CN) for 2,2,3,3,3-pentafluoropropyl (*R*)-(1-(2-bromophenyl)-2-((ethyl(phenyl)carbamoyl)oxy)ethyl)sulfamate (***ent*-11o**)

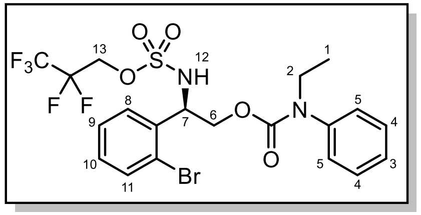

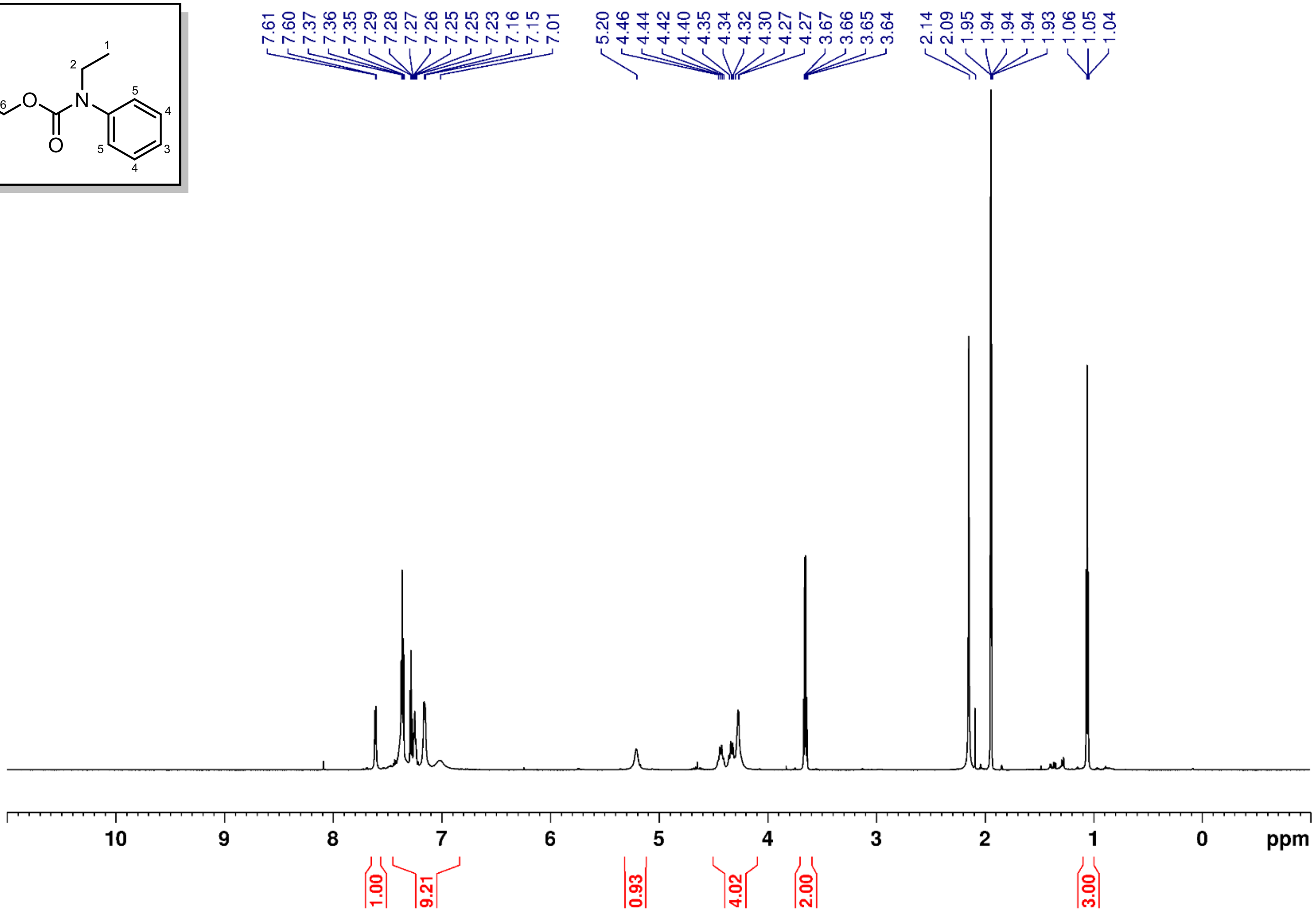

^{13}C NMR (176 MHz, CD_3CN) for 2,2,3,3,3-pentafluoropropyl (*S*)-(1-(2-bromophenyl)-2-((ethyl(phenyl)carbamoyl)oxy)ethyl)sulfamate (**11o**)

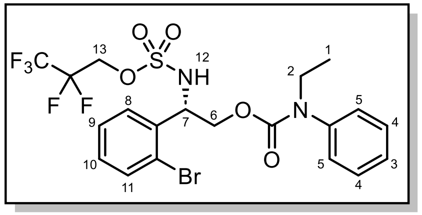

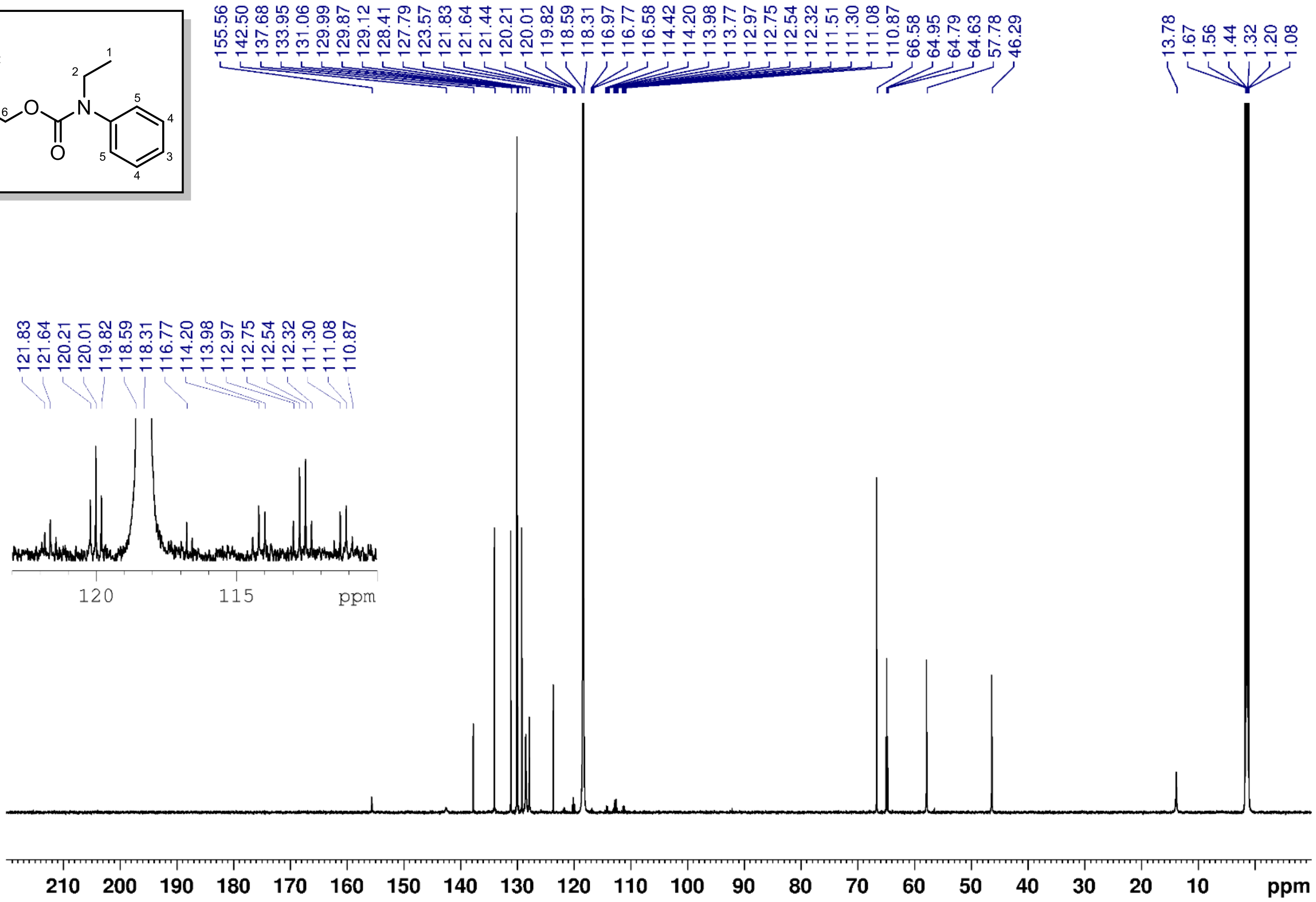

^{19}F NMR (376 MHz, CD_3CN) for 2,2,3,3,3-pentafluoropropyl (*S*)-(1-(2-bromophenyl)-2-((ethyl(phenyl)carbamoyl)oxy)ethyl)sulfamate (**11o**)

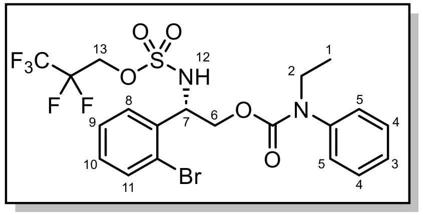

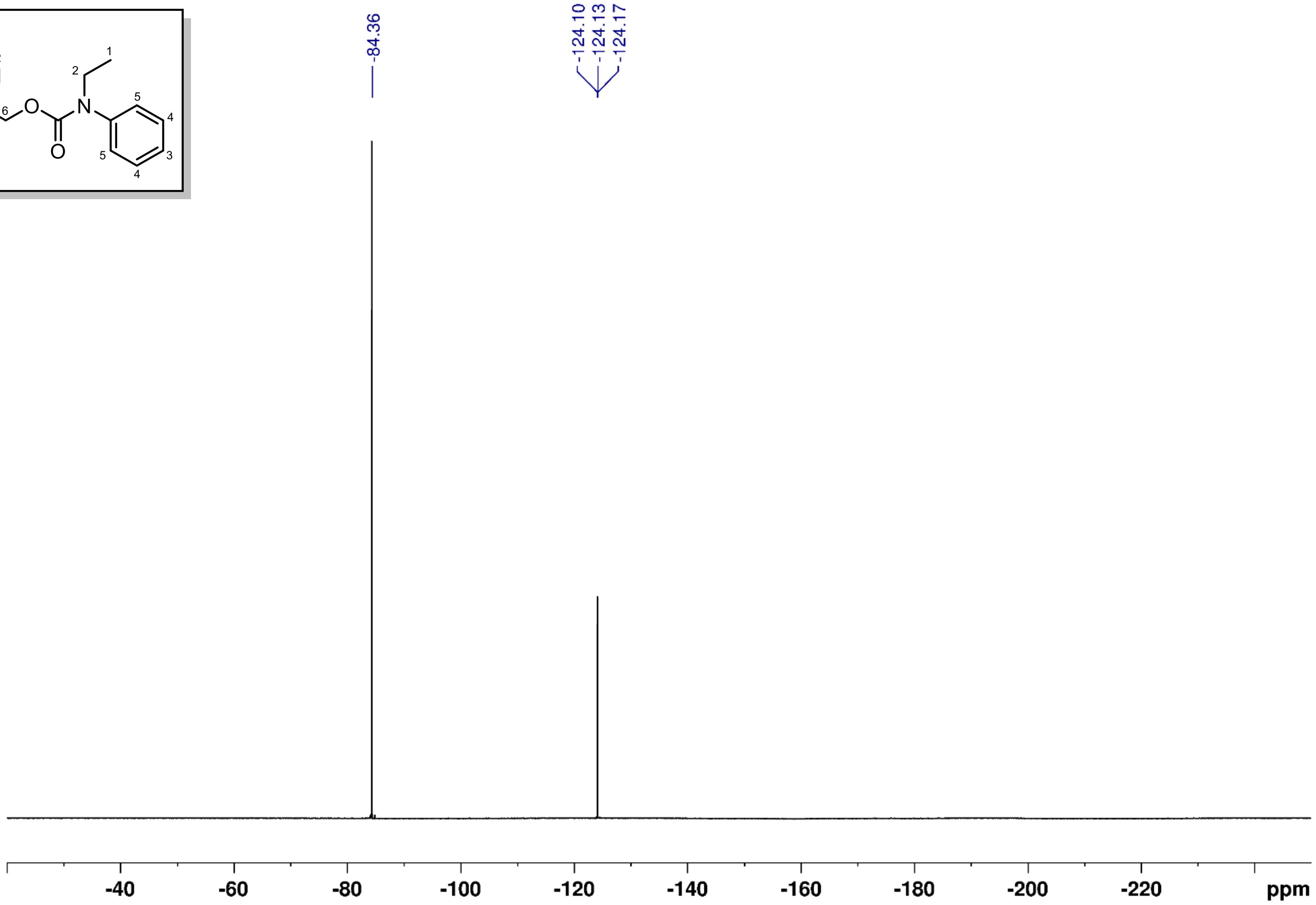

^{1}H NMR (700 MHz, CD_3CN) for 2,2,3,3,3-pentafluoropropyl (*S*)-(2-((ethyl(phenyl)carbamoyl)oxy)-1-(thiophen-2-yl)ethyl)sulfamate (**11p**)

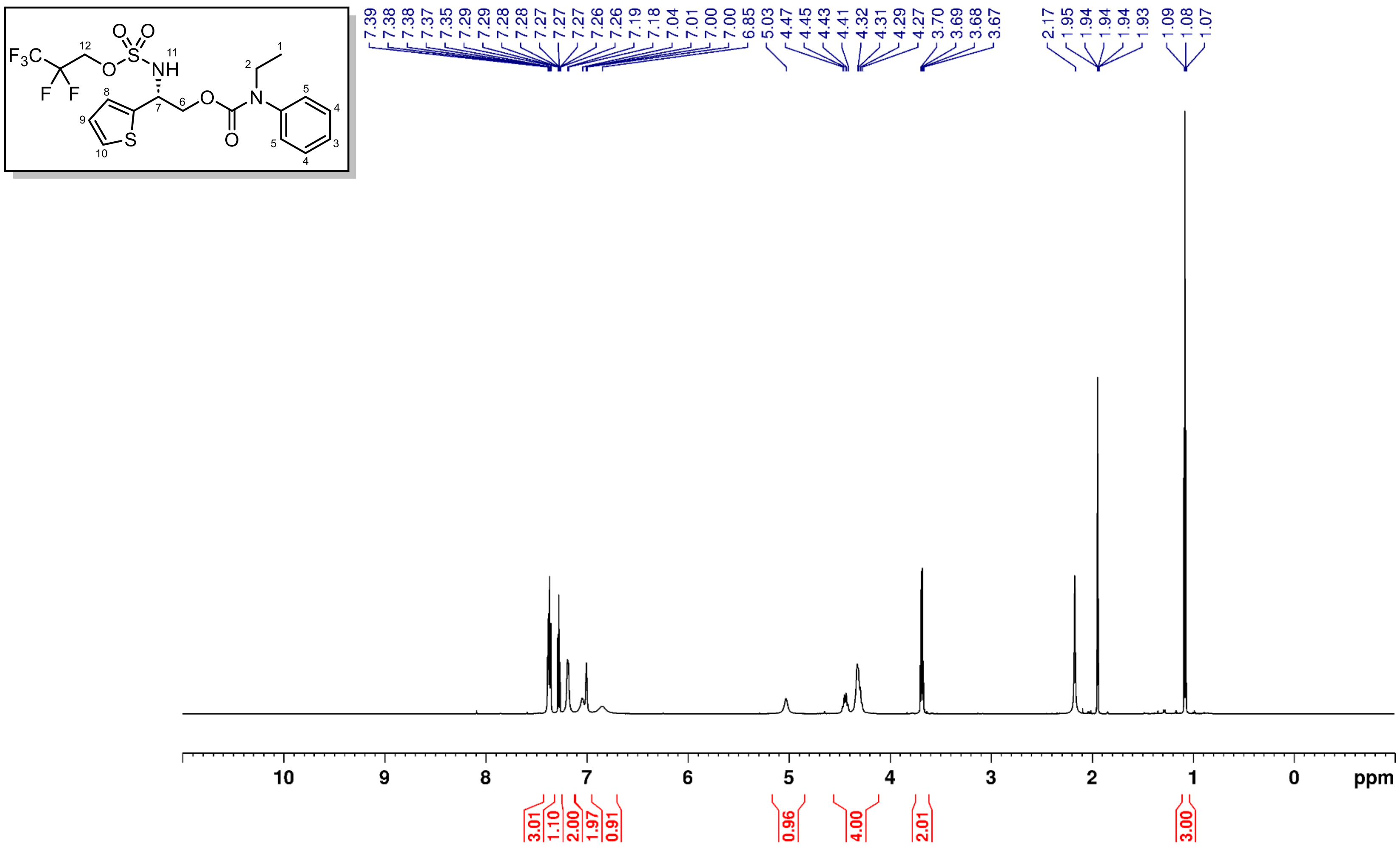

^{1}H NMR (700 MHz, CD_3CN) for 2,2,3,3,3-pentafluoropropyl (*R*)-(2-((ethyl(phenyl)carbamoyl)oxy)-1-(thiophen-2-yl)ethyl)sulfamate (***ent*-11p**)

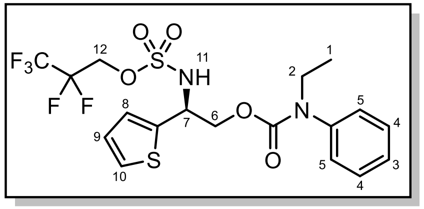

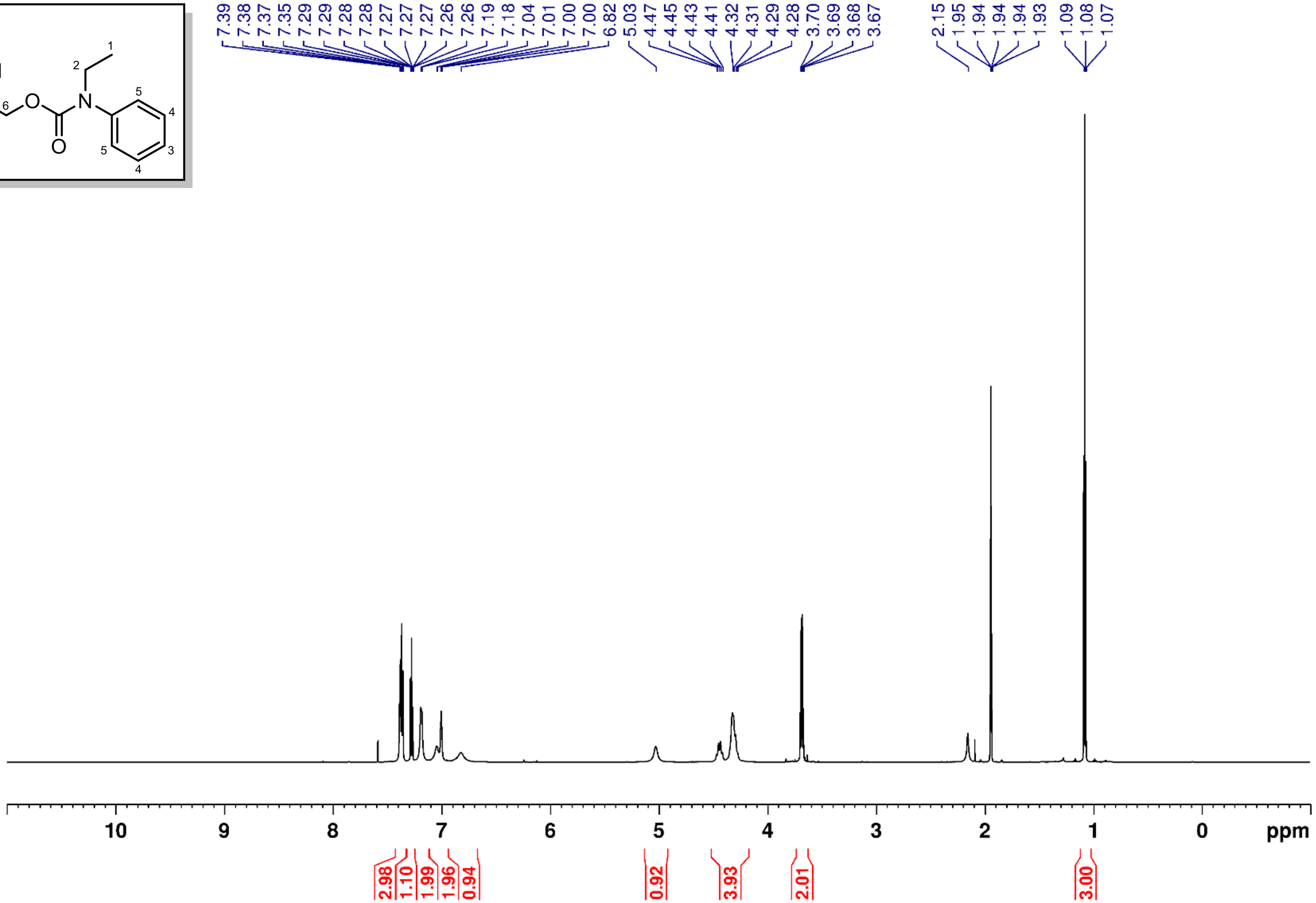

^{13}C NMR (176 MHz, CD_3CN) for 2,2,3,3,3-pentafluoropropyl (*S*)-(2-((ethyl(phenyl)carbamoyl)oxy)-1-(thiophen-2-yl)ethyl)sulfamate (**11p**)

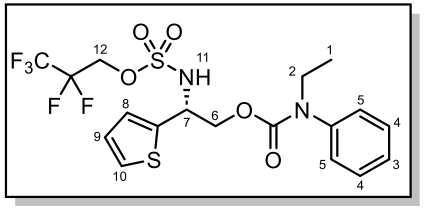

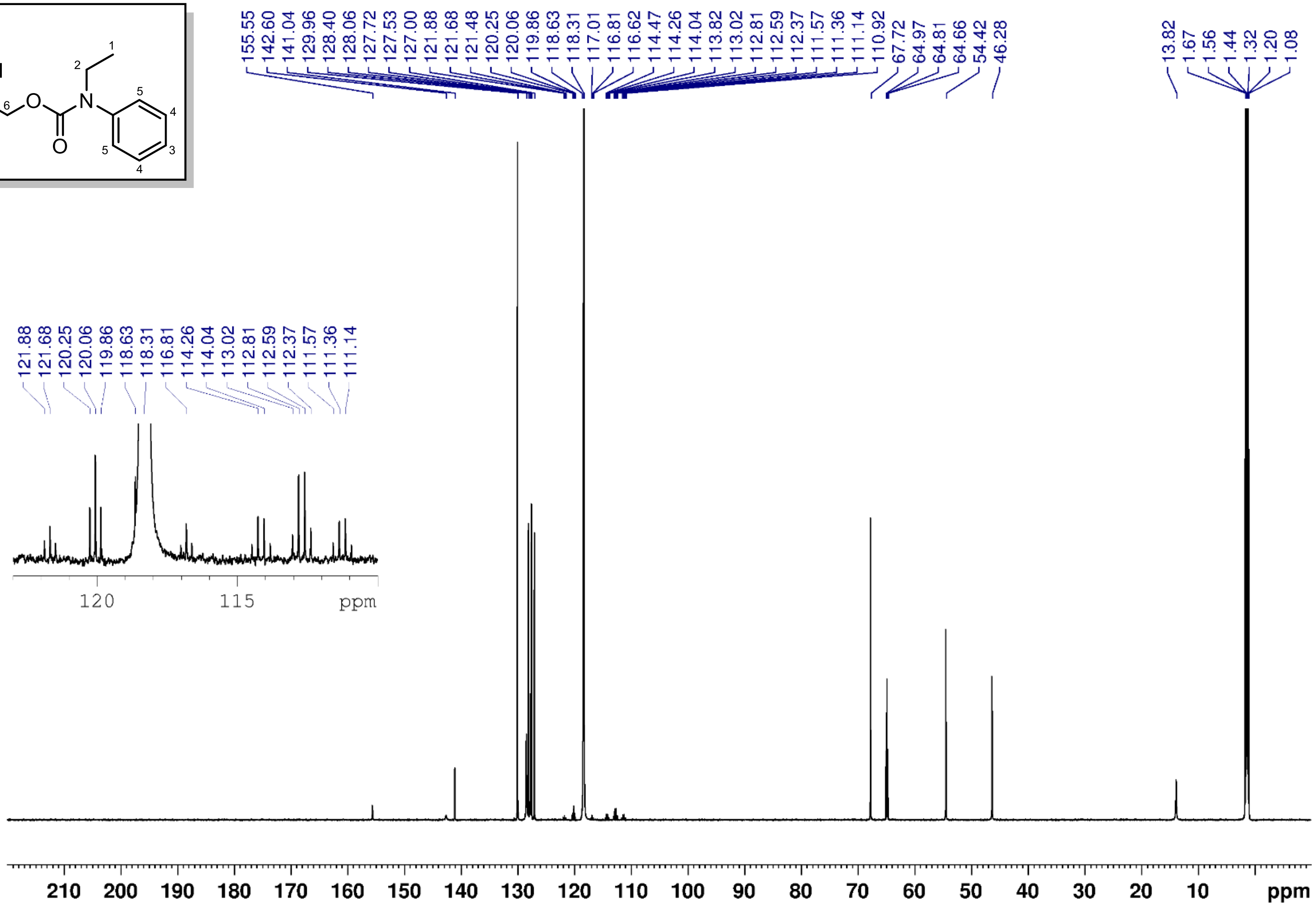

^{19}F NMR (376 MHz, CD_3CN) for 2,2,3,3,3-pentafluoropropyl (*S*)-(2-((ethyl(phenyl)carbamoyl)oxy)-1-(thiophen-2-yl)ethyl)sulfamate (**11p**)

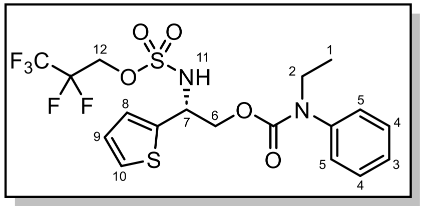

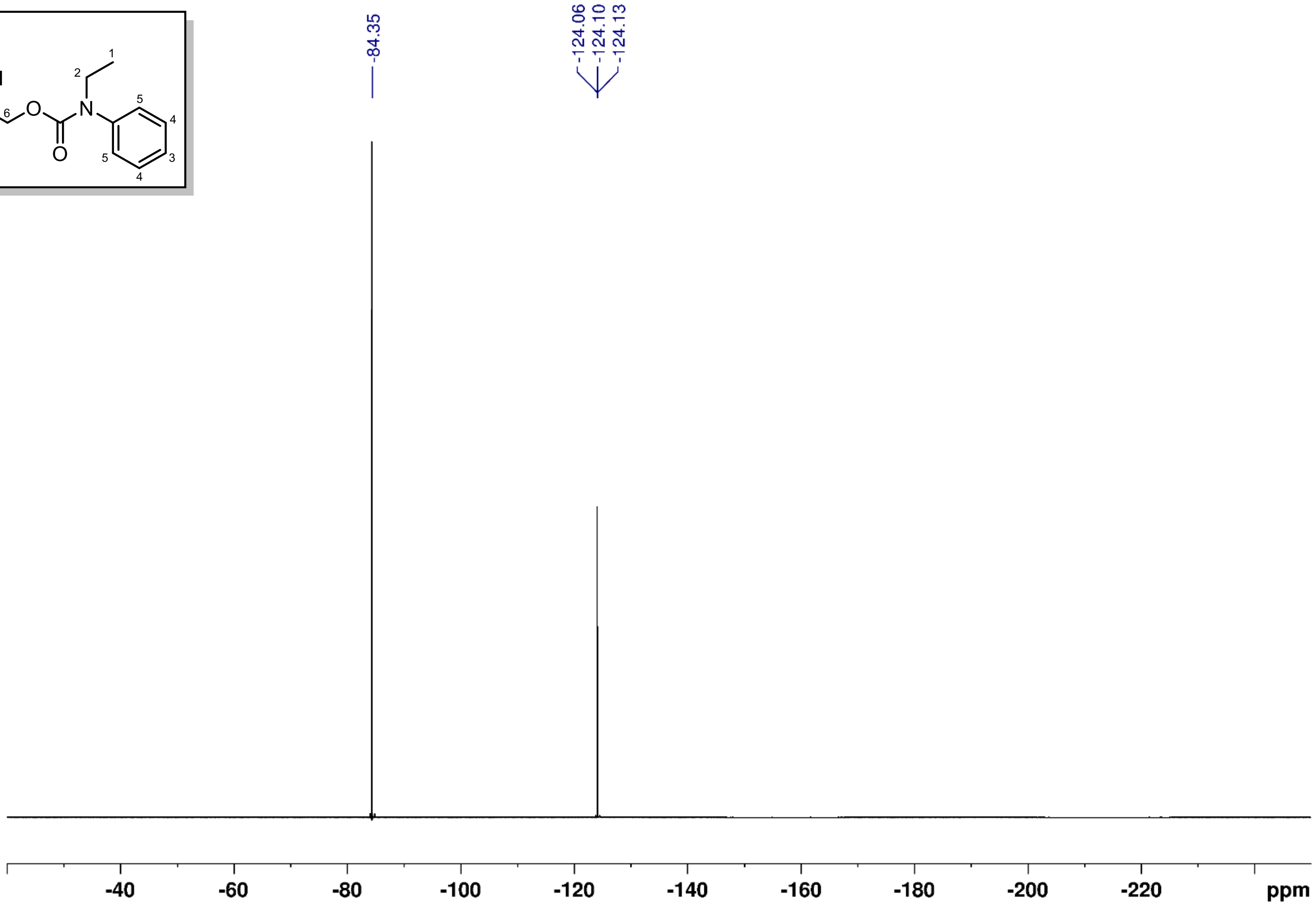

^{1}H NMR (700 MHz, CD_3CN) for 2,2,3,3,3-pentafluoropropyl (*S*)-(2-((ethyl(phenyl)carbamoyl)oxy)-1-(thiophen-3-yl)ethyl)sulfamate (**11q**)

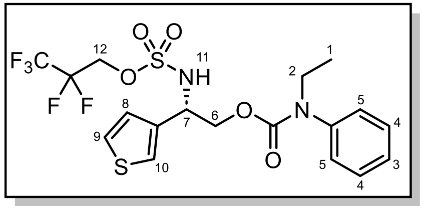

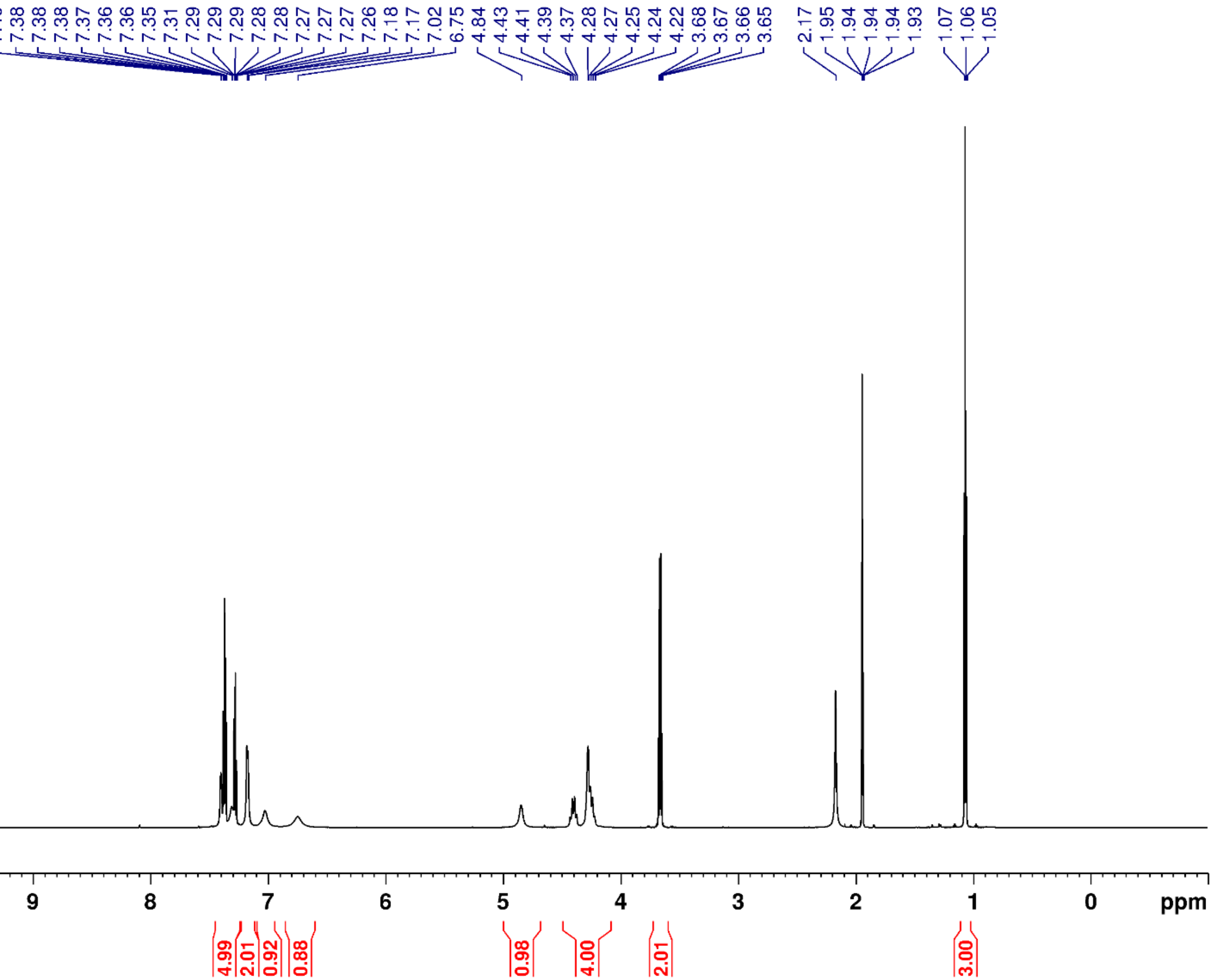

^{1}H NMR (700 MHz, CD_3CN) for 2,2,3,3,3-pentafluoropropyl (*R*)-(2-((ethyl(phenyl)carbamoyl)oxy)-1-(thiophen-3-yl)ethyl)sulfamate (***ent*-11q**)

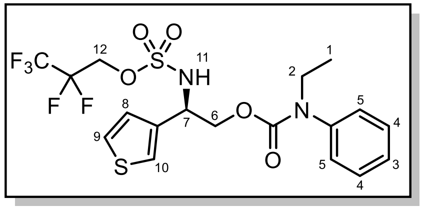

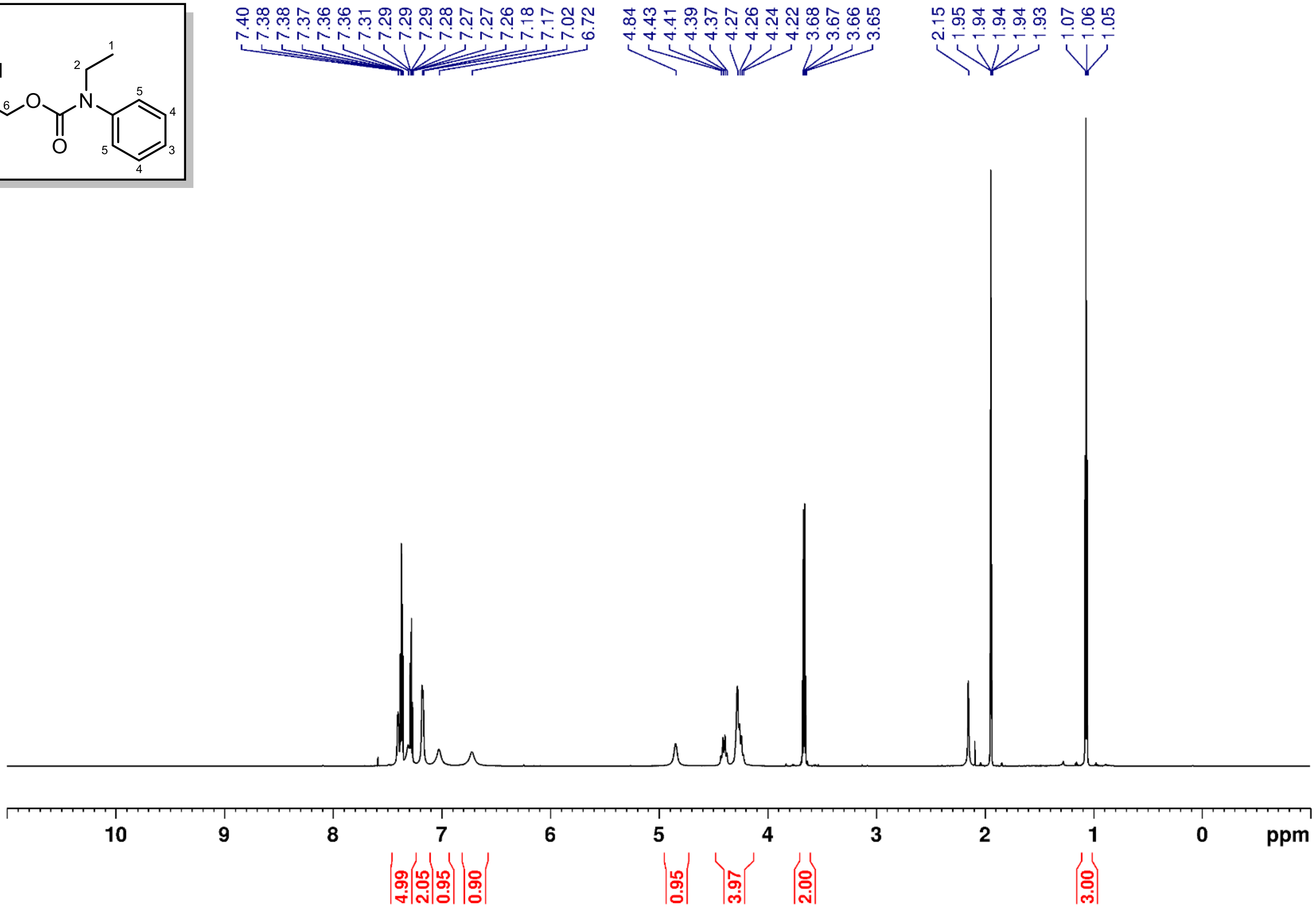

^{13}C NMR (176 MHz, CD_3CN) for 2,2,3,3,3-pentafluoropropyl (*S*)-(2-((ethyl(phenyl)carbamoyl)oxy)-1-(thiophen-3-yl)ethyl)sulfamate (**11q**)

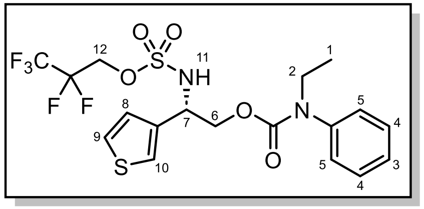

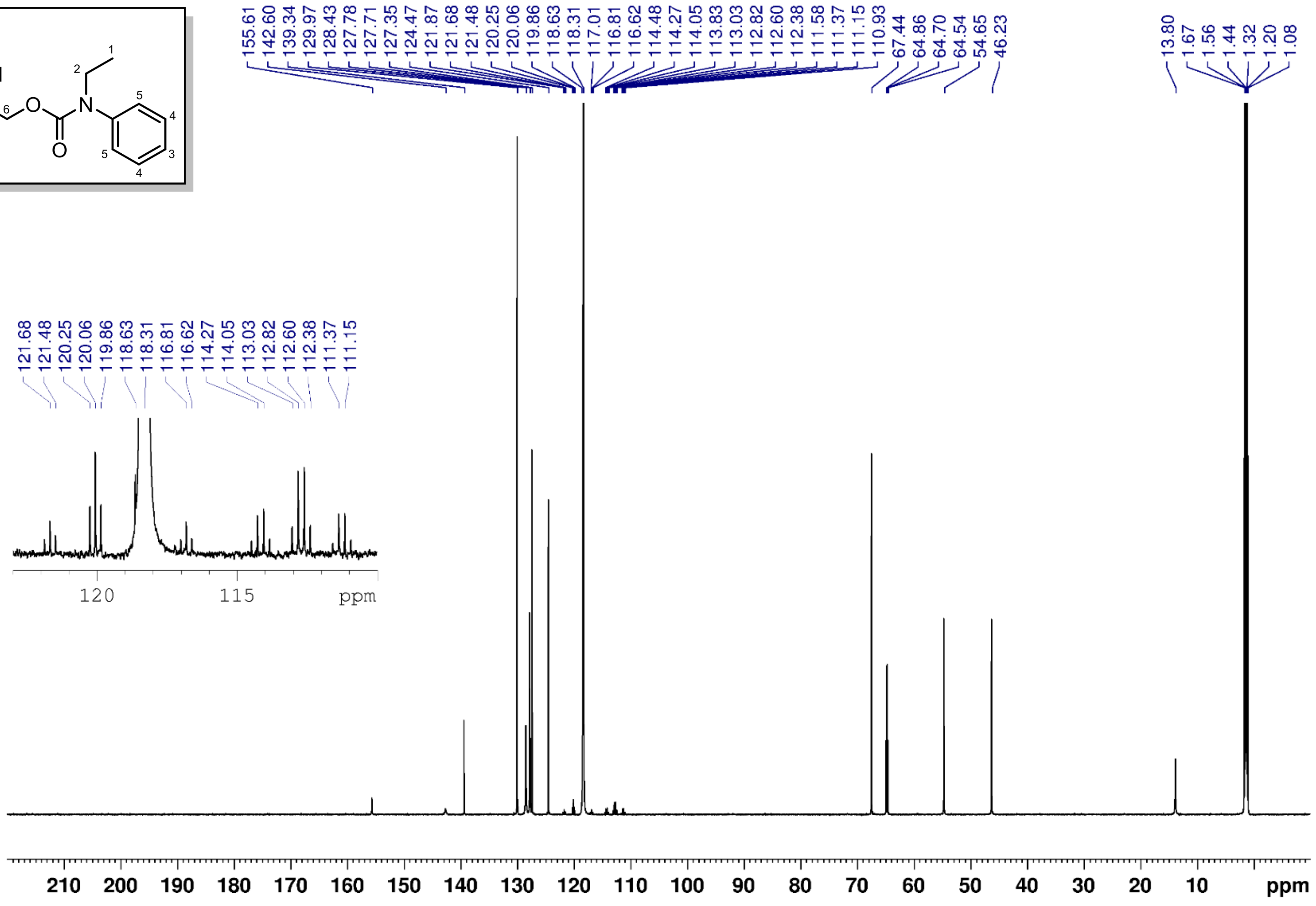

^{19}F NMR (376 MHz, CD_3CN) for 2,2,3,3,3-pentafluoropropyl (*S*)-(2-((ethyl(phenyl)carbamoyl)oxy)-1-(thiophen-3-yl)ethyl)sulfamate (**11q**)

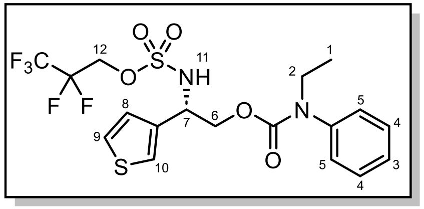

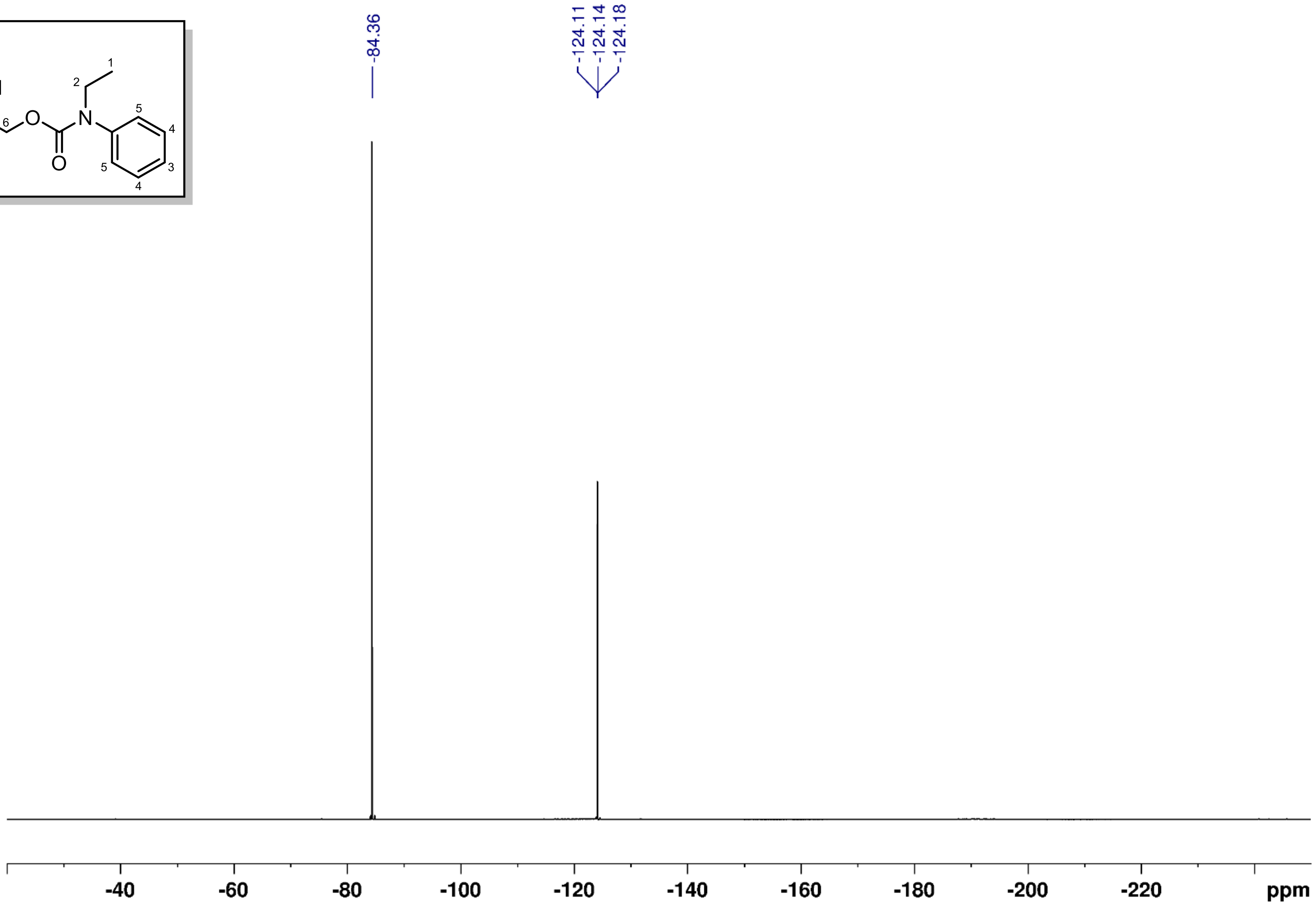

^{1}H NMR (500 MHz, CD_3CN) for 2,2,3,3,3-pentafluoropropyl ((1*S*,2*S*)-2-((ethyl(phenyl)carbamoyl)oxy)-2,3-dihydro-1*H*-inden-1-yl)sulfamate (**11r**)

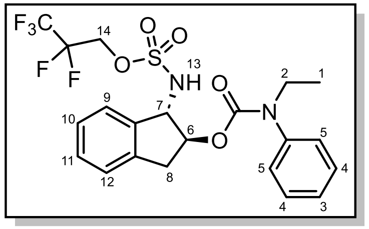

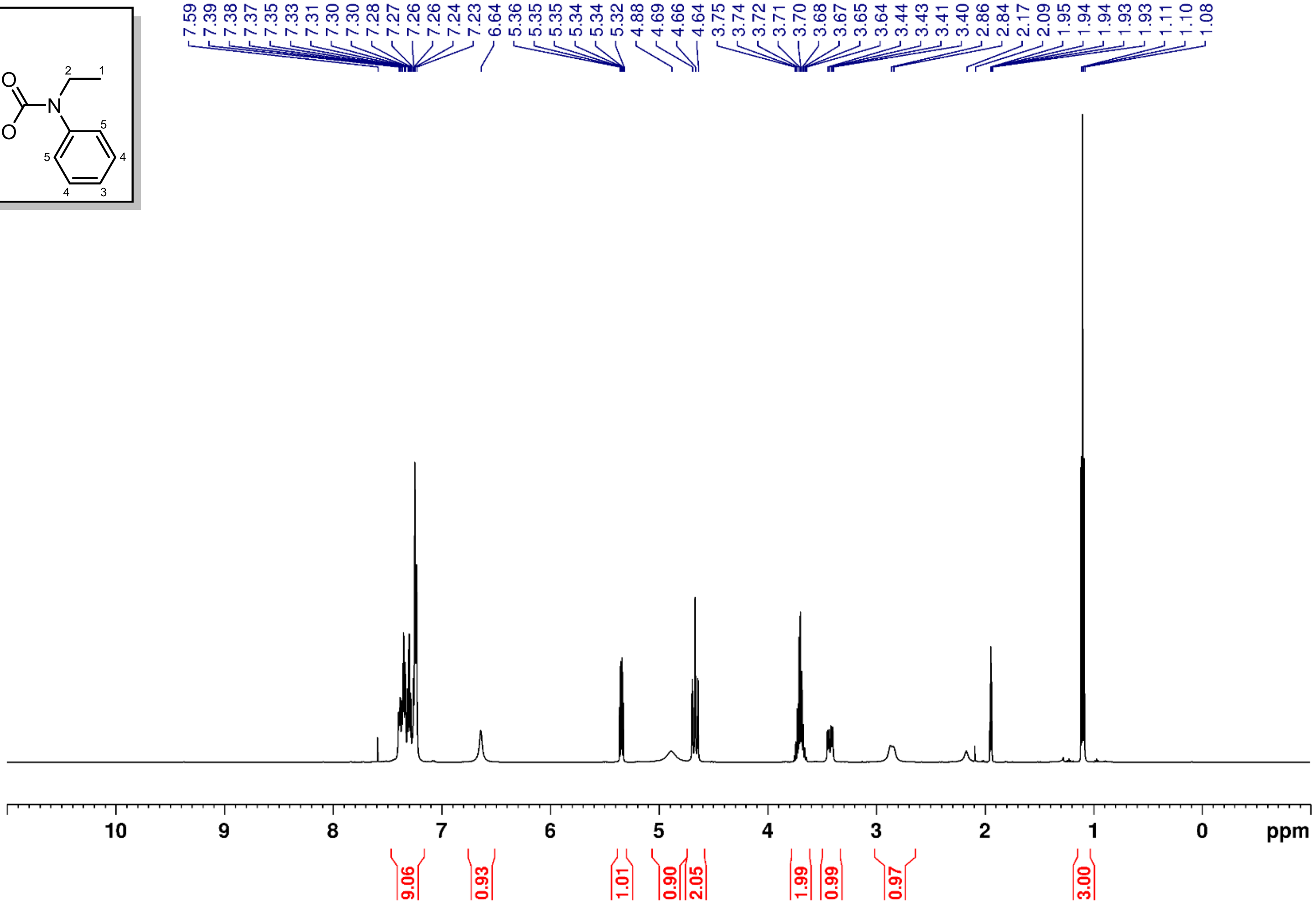

¹H NMR (500 MHz, CD_3CN) for 2,2,3,3,3-pentafluoropropyl ((1*R*,2*R*)-2-((ethyl(phenyl)carbamoyl)oxy)-2,3-dihydro-1*H*-inden-1-yl)sulfamate (***ent*-11r**)

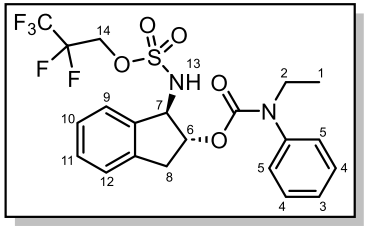

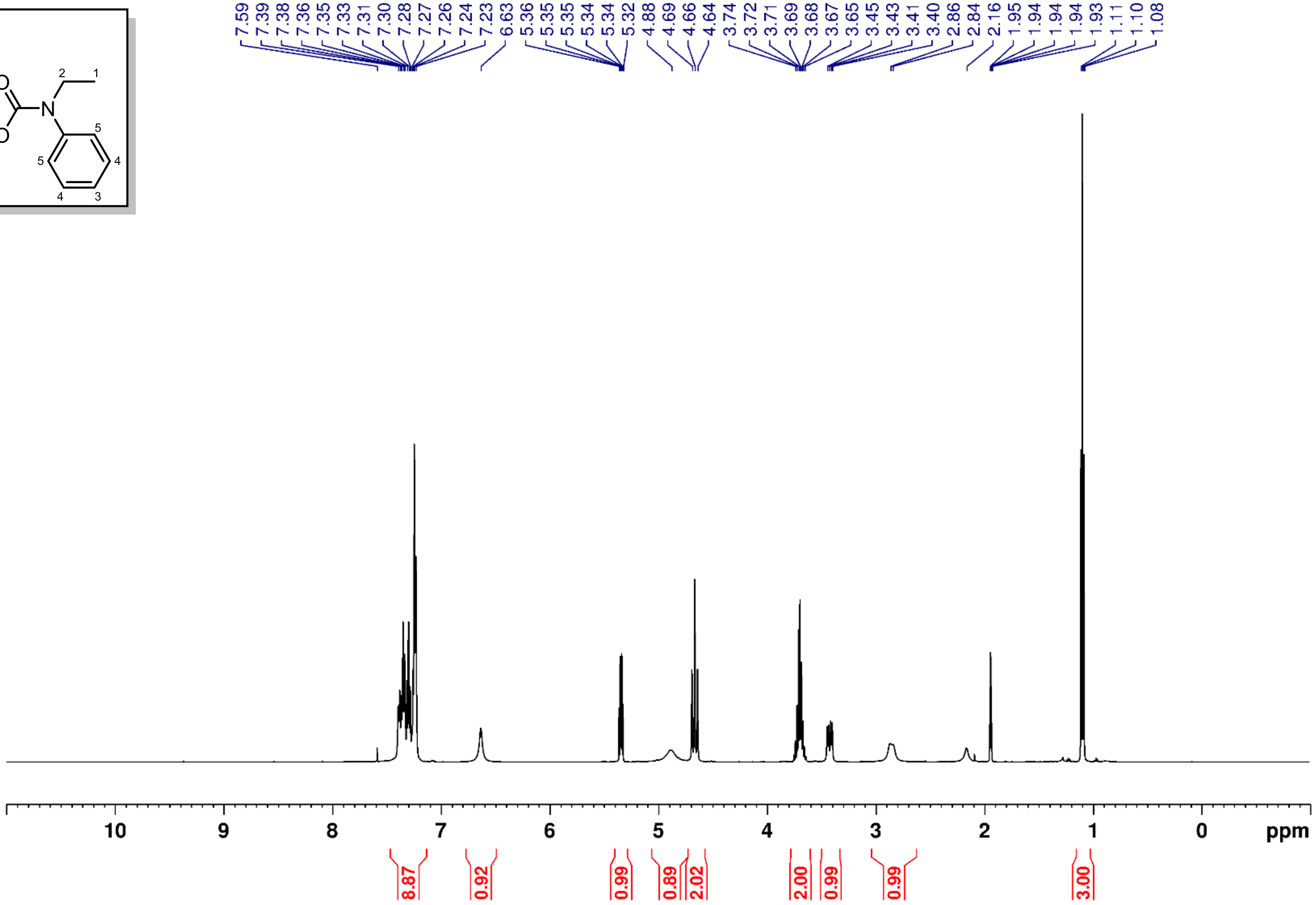

^{13}C NMR (126 MHz, CD_3CN) for 2,2,3,3,3-pentafluoropropyl ((1*S*,2*S*)-2-((ethyl(phenyl)carbamoyl)oxy)-2,3-dihydro-1*H*-inden-1-yl)sulfamate (**11r**)

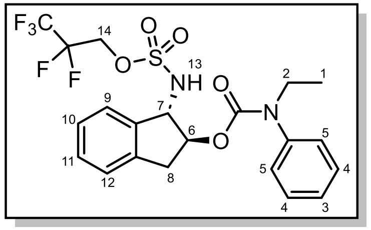

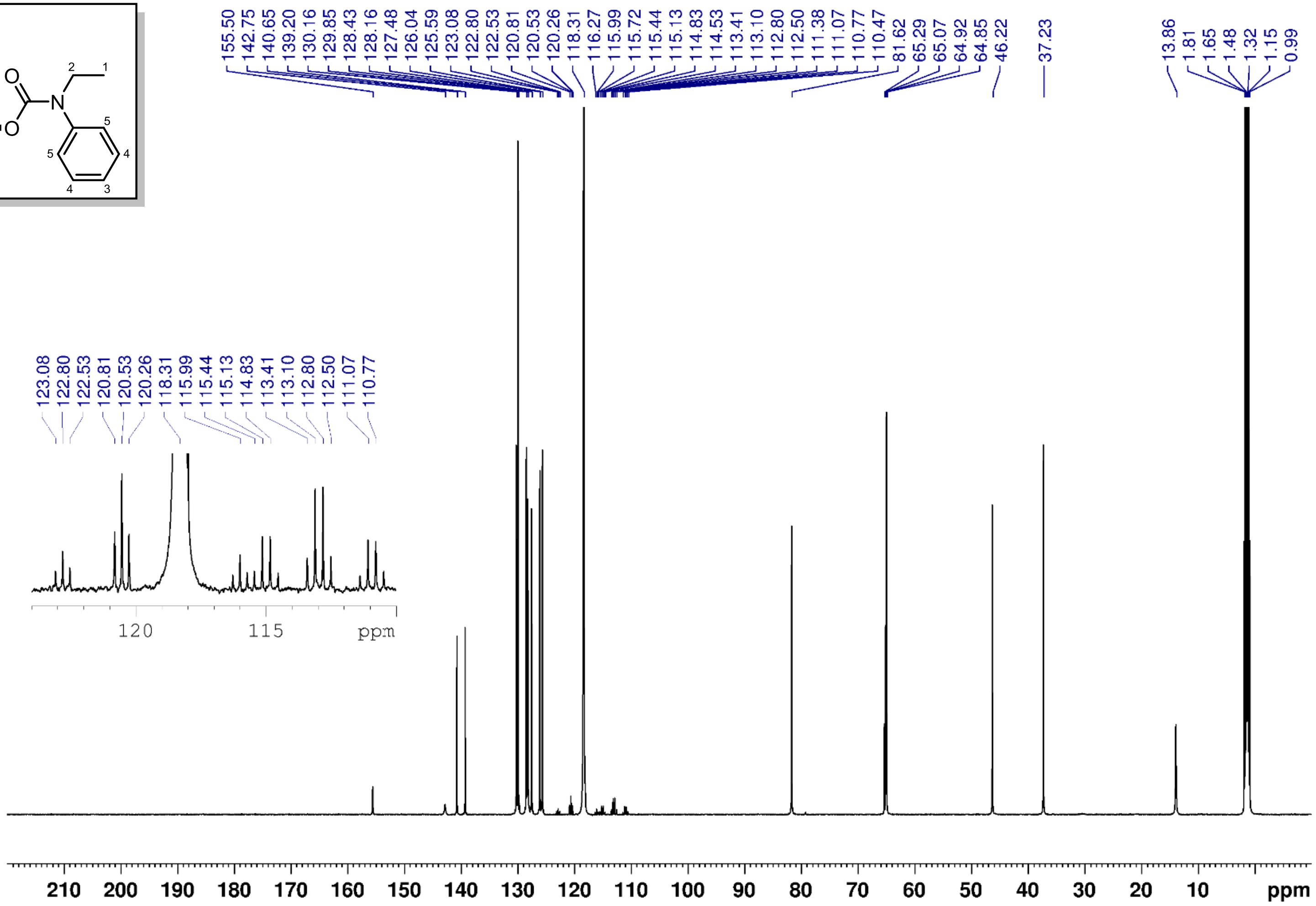

^{19}F NMR (376 MHz, CD_3CN) for 2,2,3,3,3-pentafluoropropyl ((1*S*,2*S*)-2-((ethyl(phenyl)carbamoyl)oxy)-2,3-dihydro-1*H*-inden-1-yl)sulfamate (**11r**)

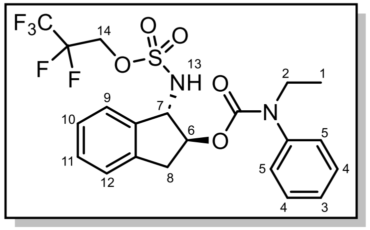

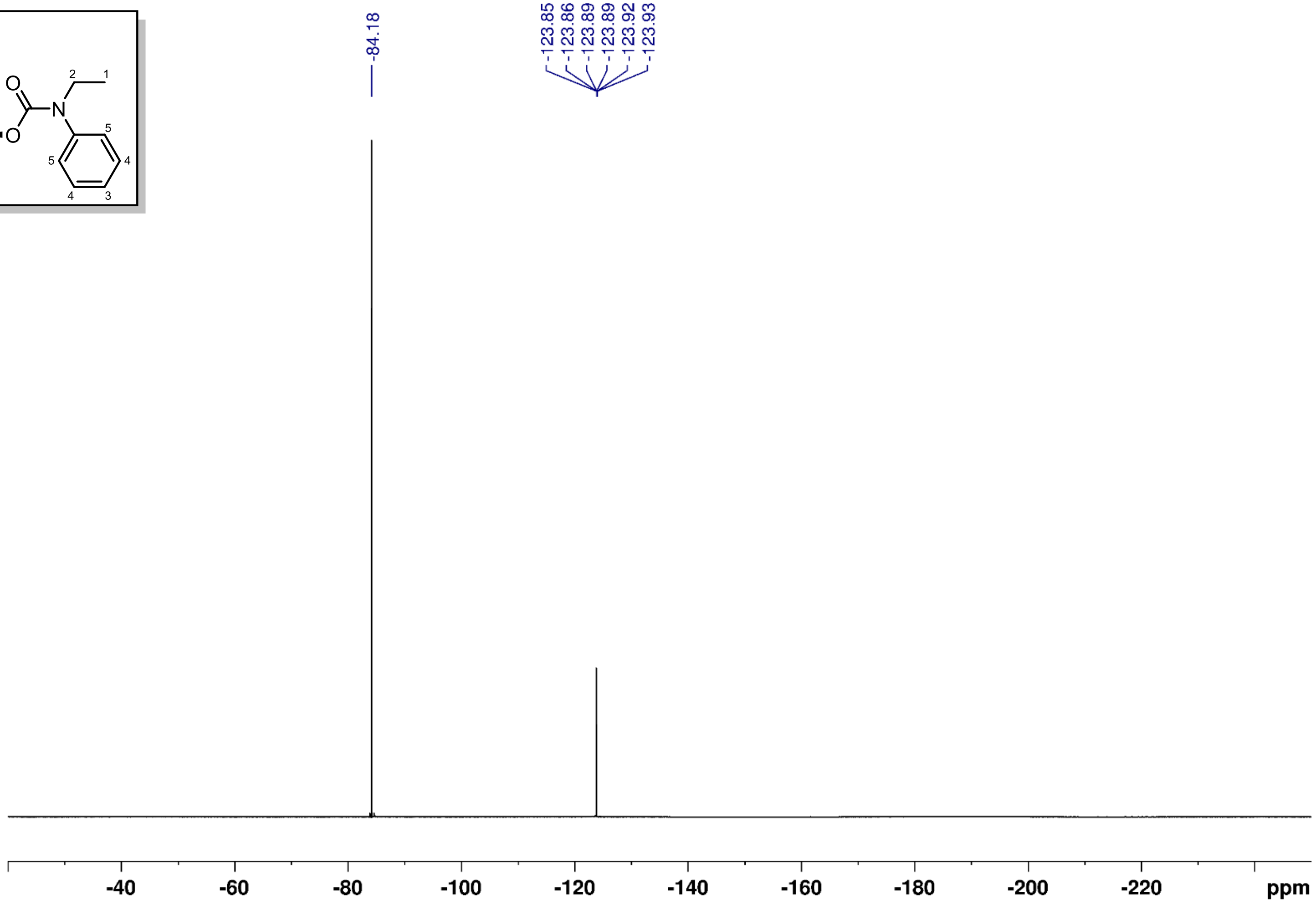

^{1}H NMR (700 MHz, CD_3CN) for 2,2,3,3,4,4,4-heptafluorobutyl (*S*)-(2-((methyl(phenyl)carbamoyl)oxy)-1-phenylethyl)sulfamate (**S5a**)

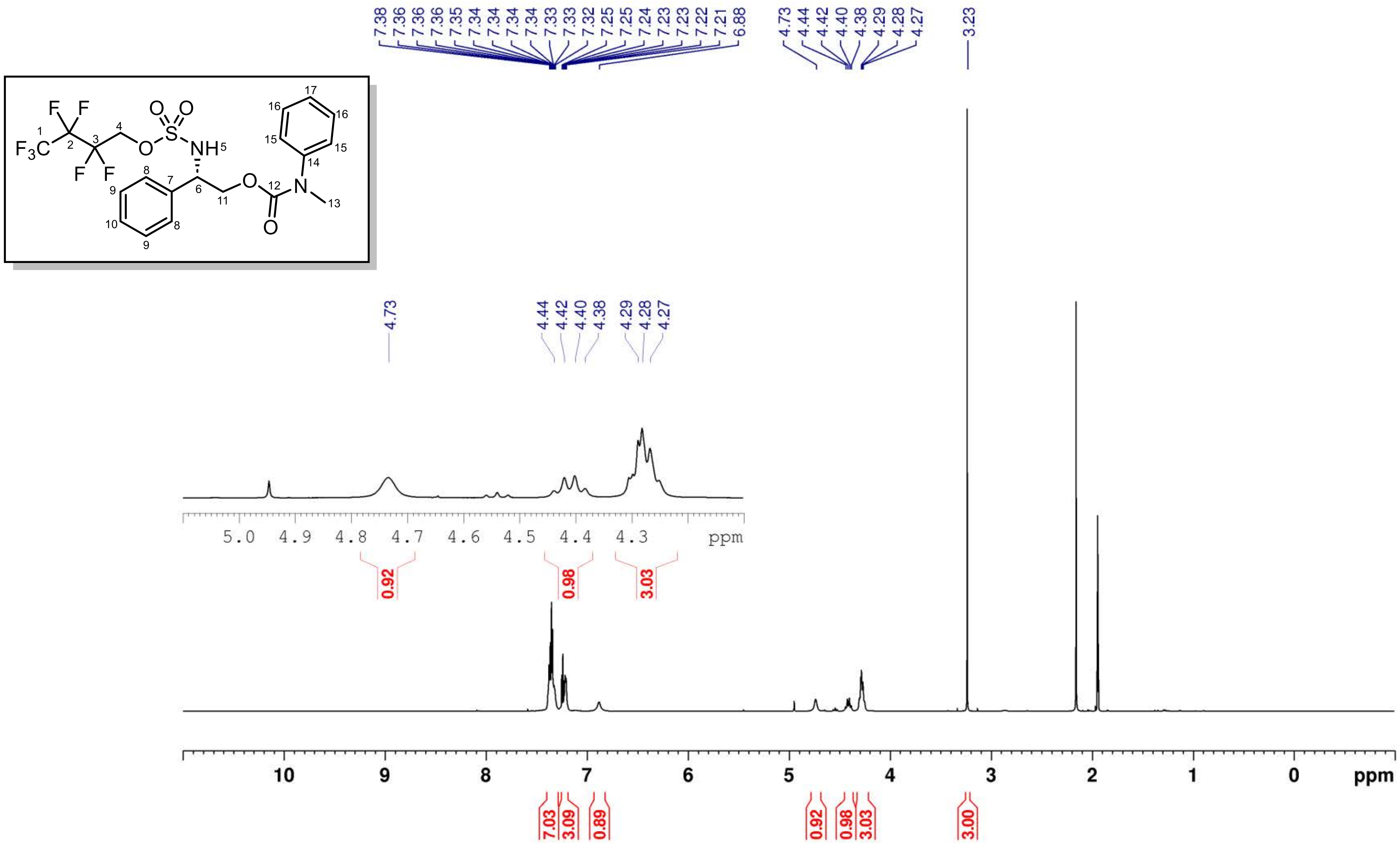

^{13}C NMR (176 MHz, CD_3CN) for 2,2,3,3,4,4,4-heptafluorobutyl (*S*)-(2-((methyl(phenyl)carbamoyl)oxy)-1-phenylethyl)sulfamate (**S5a**)

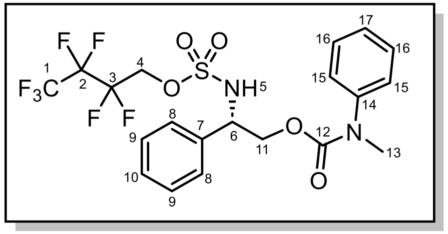

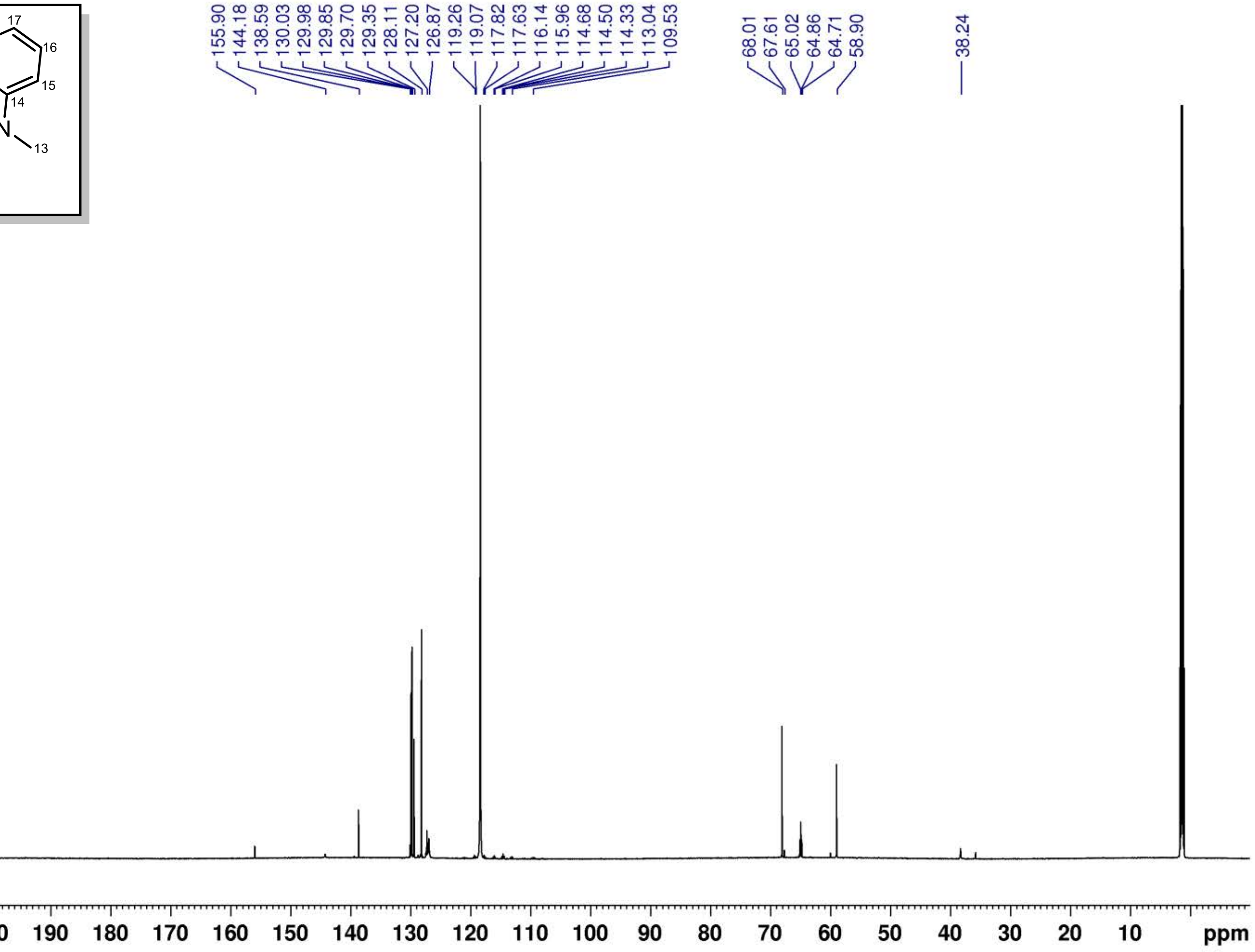

^{19}F NMR (376 MHz, CD_3CN) for 2,2,3,3,4,4,4-heptafluorobutyl (*S*)-(2-((methyl(phenyl)carbamoyl)oxy)-1-phenylethyl)sulfamate (**S5a**)

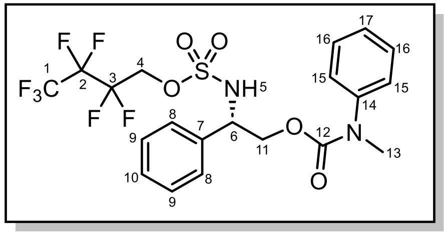

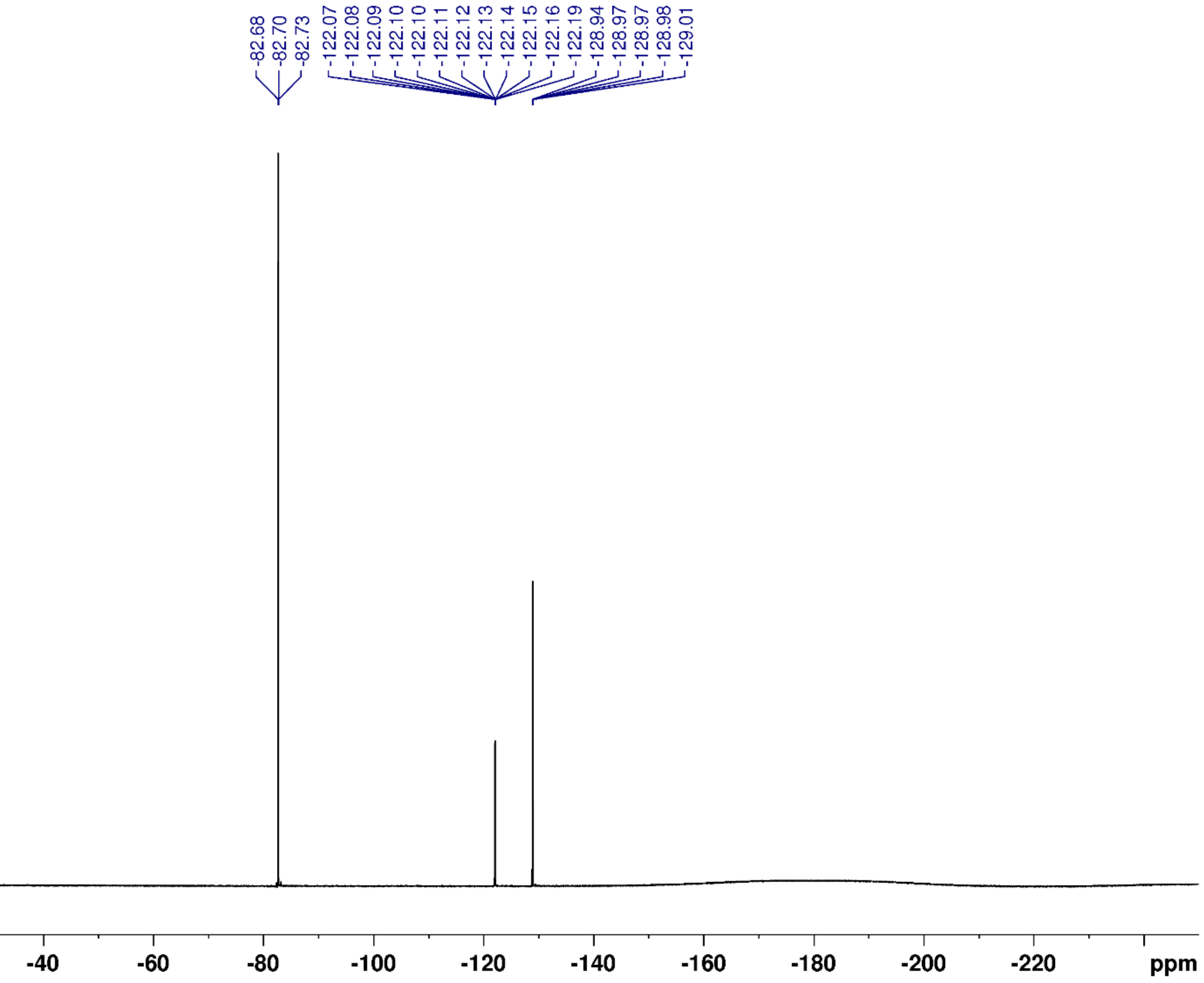

^{1}H NMR (700 MHz, CD_3CN) for 2,2,3,3,4,4,4-heptafluorobutyl (((phenethoxycarbonyl)(phenyl)amino)methyl)sulfamate (**S6a**)

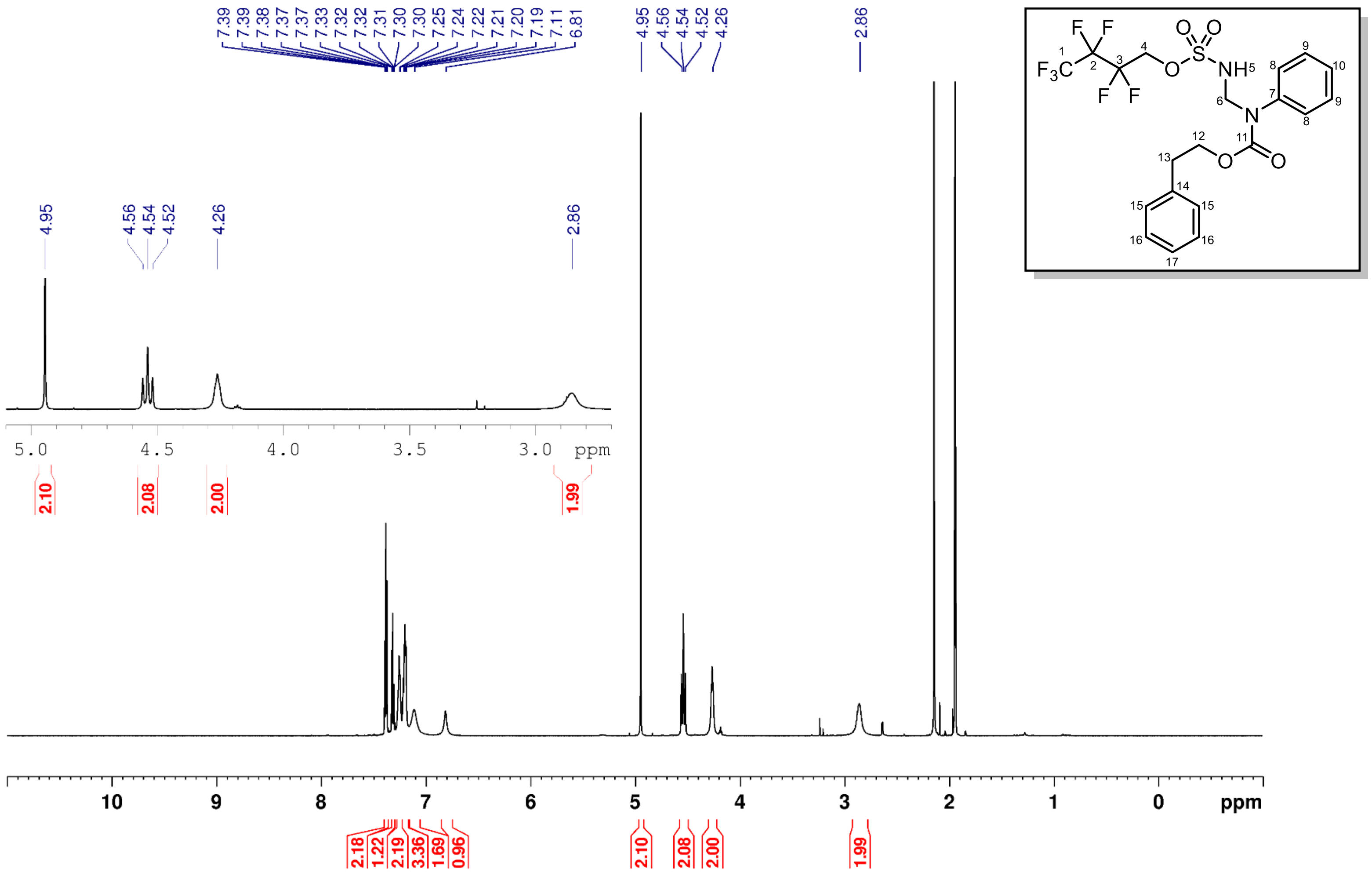

^{13}C NMR (176 MHz, CD_3CN) for 2,2,3,3,4,4,4-heptafluorobutyl (((phenethoxycarbonyl)(phenyl)amino)methyl)sulfamate (**S6a**)

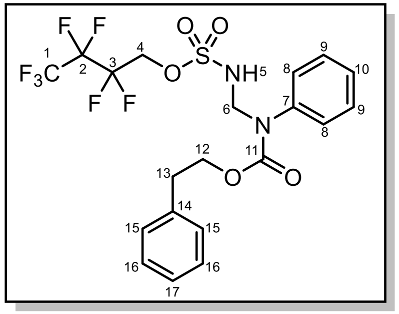

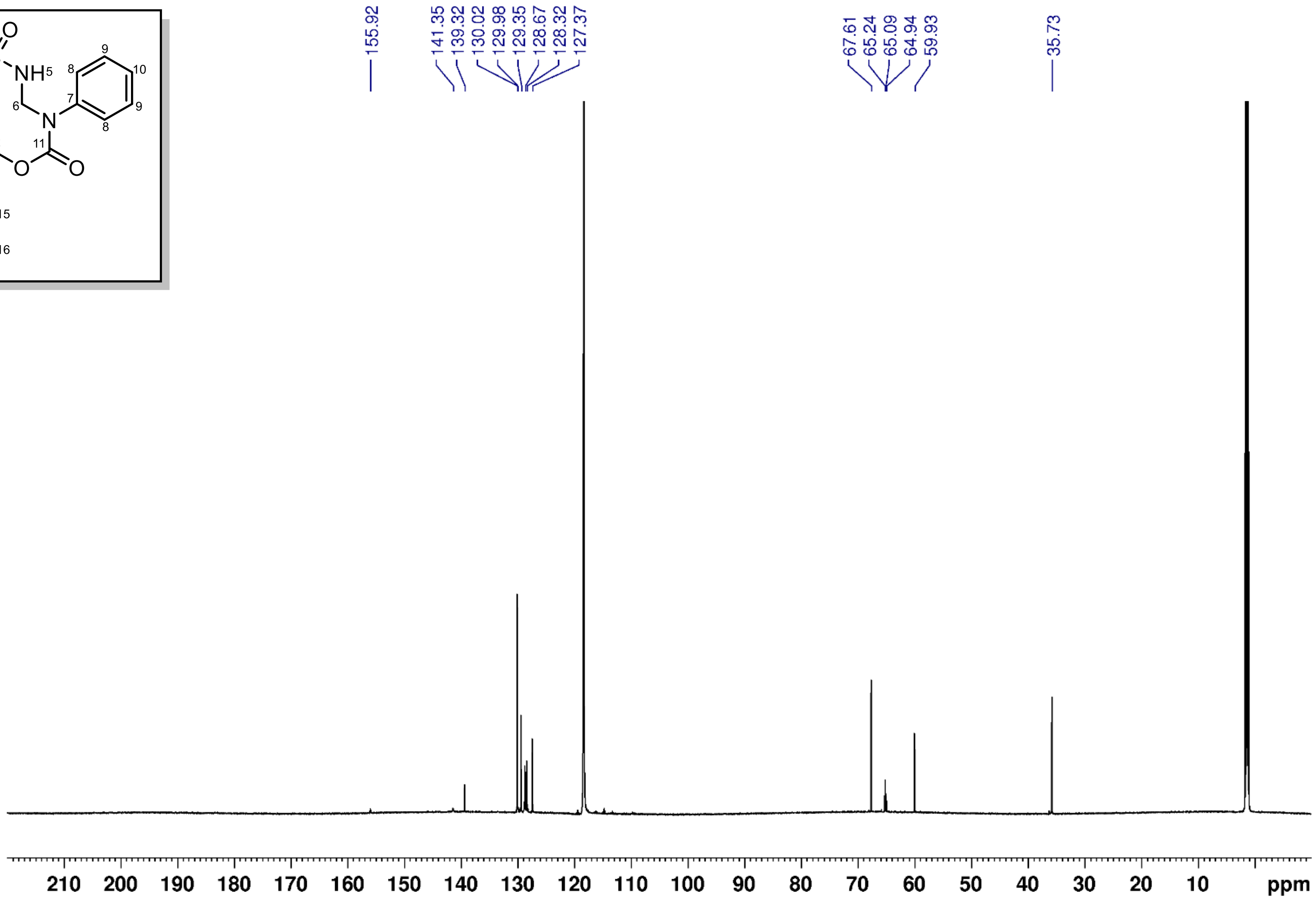

^{19}F NMR (376 MHz, CD_3CN) for 2,2,3,3,4,4,4-heptafluorobutyl (((phenethoxycarbonyl)(phenyl)amino)methyl)sulfamate (**S6a**)

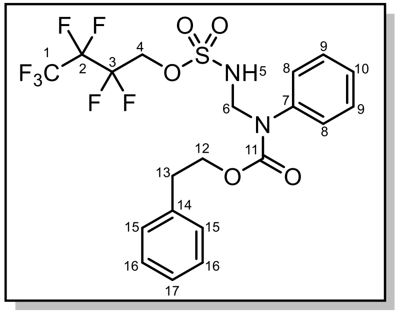

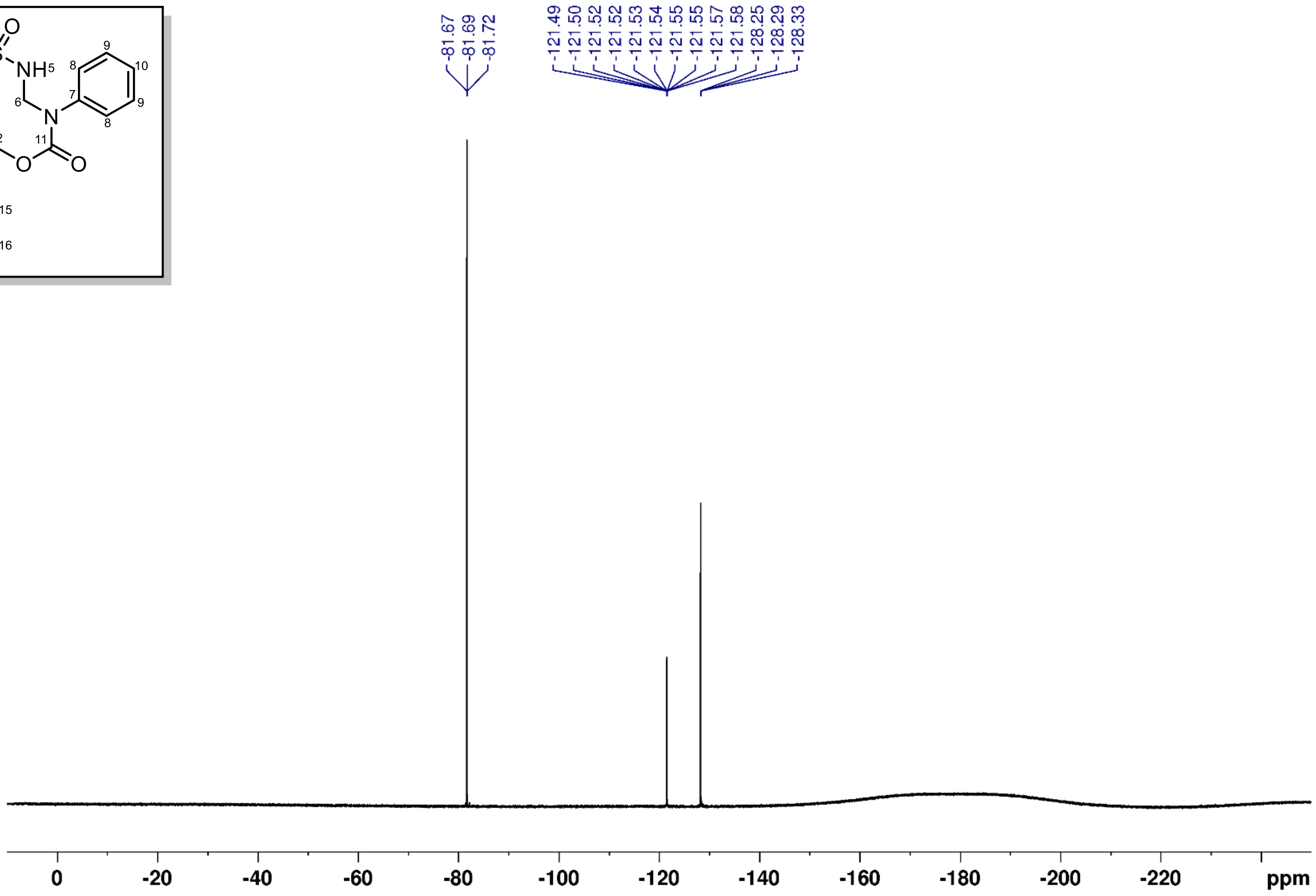

^{1}H NMR (700 MHz, $CDCl_3$) for 2,2,3,3,3-pentafluoropropyl ((((pentyloxy)carbonyl)(phenyl)amino)methyl)sulfamate

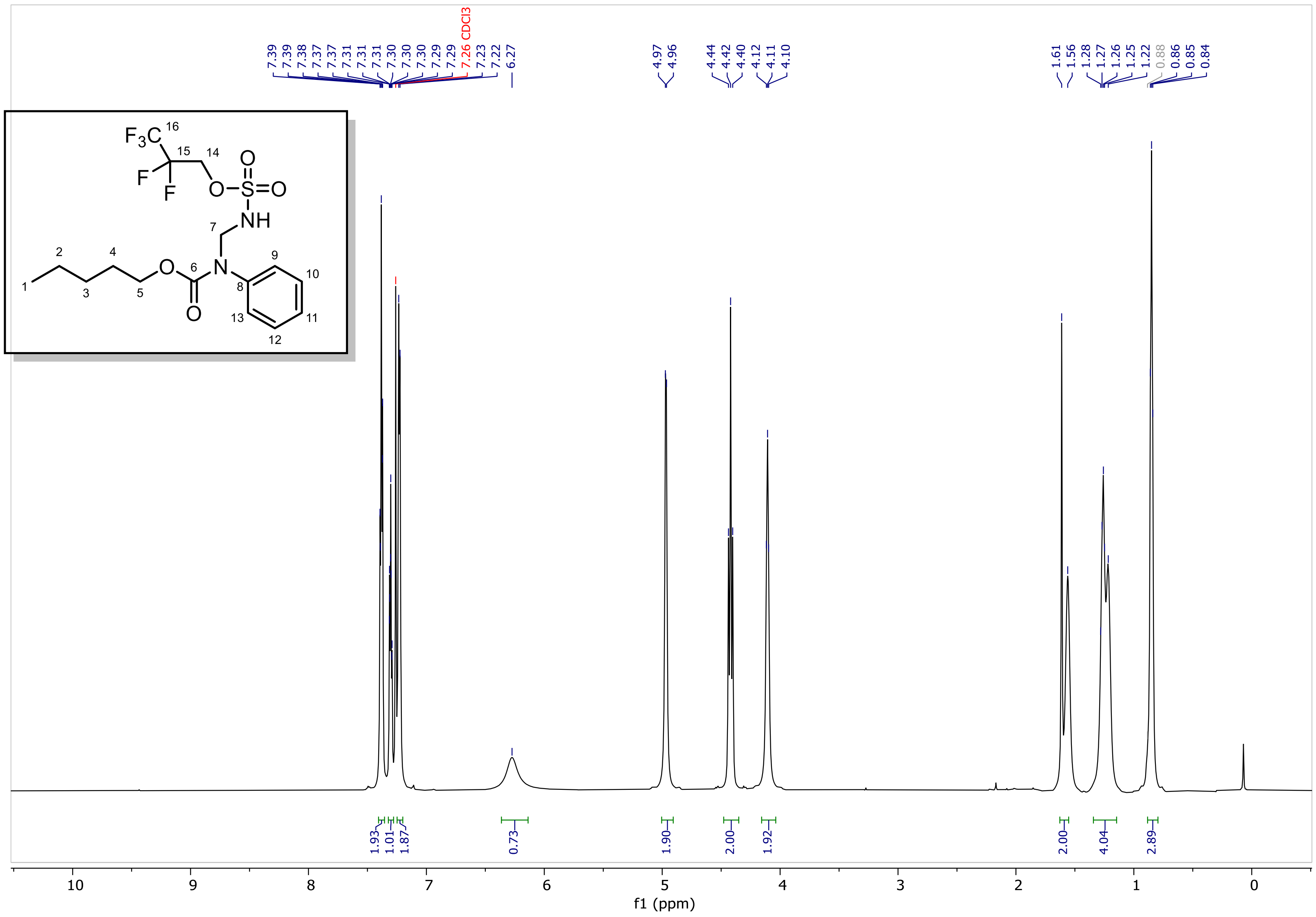

^{13}C NMR (176 MHz, CDCl$_3$) for 2,2,3,3,3-pentafluoropropyl ((((pentyloxy)carbonyl)(phenyl)amino)methyl)sulfamate

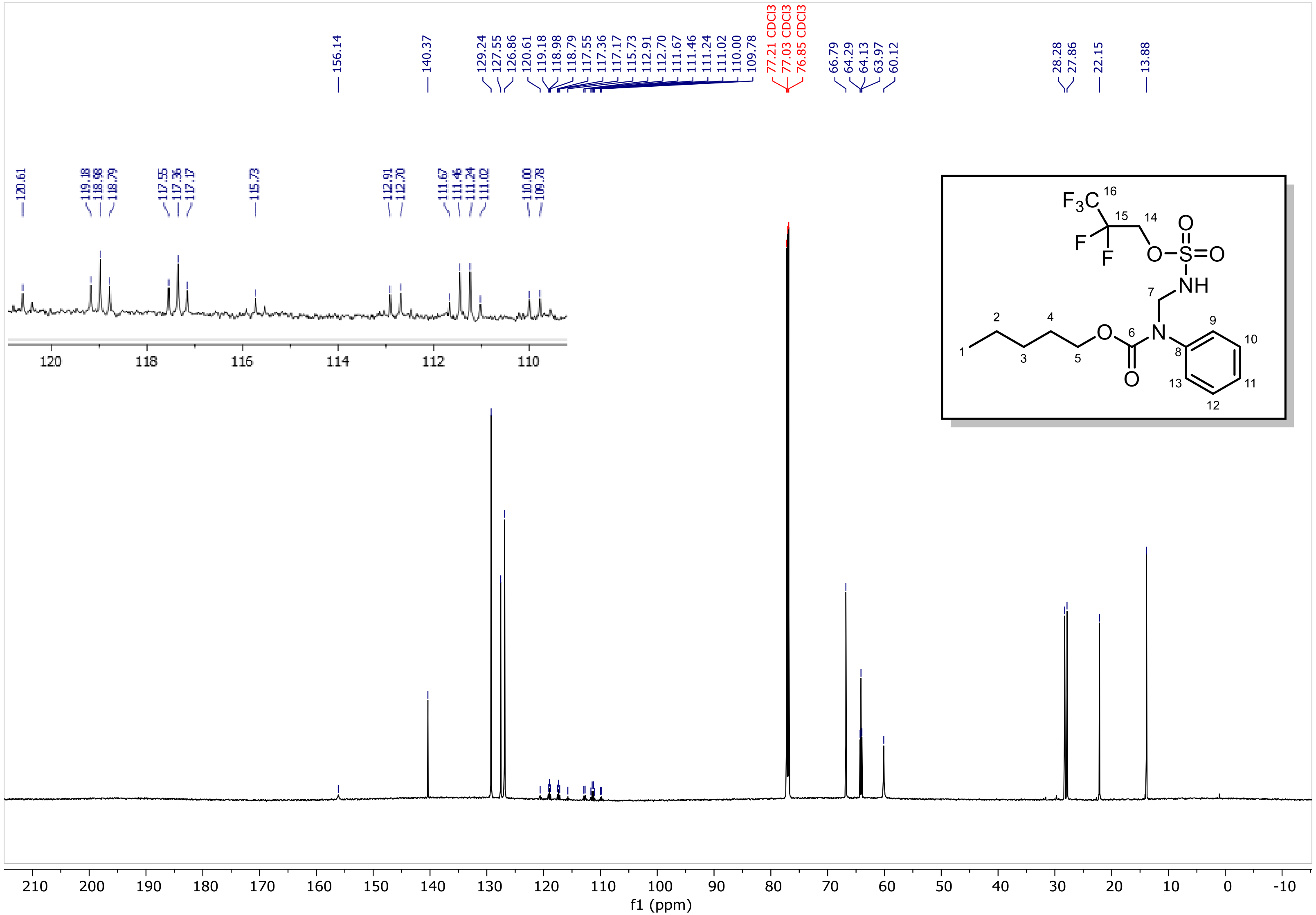

^{19}F NMR (376 MHz, $CDCl_3$) for 2,2,3,3,3-pentafluoropropyl ((((pentyloxy)carbonyl)(phenyl)amino)methyl)sulfamate

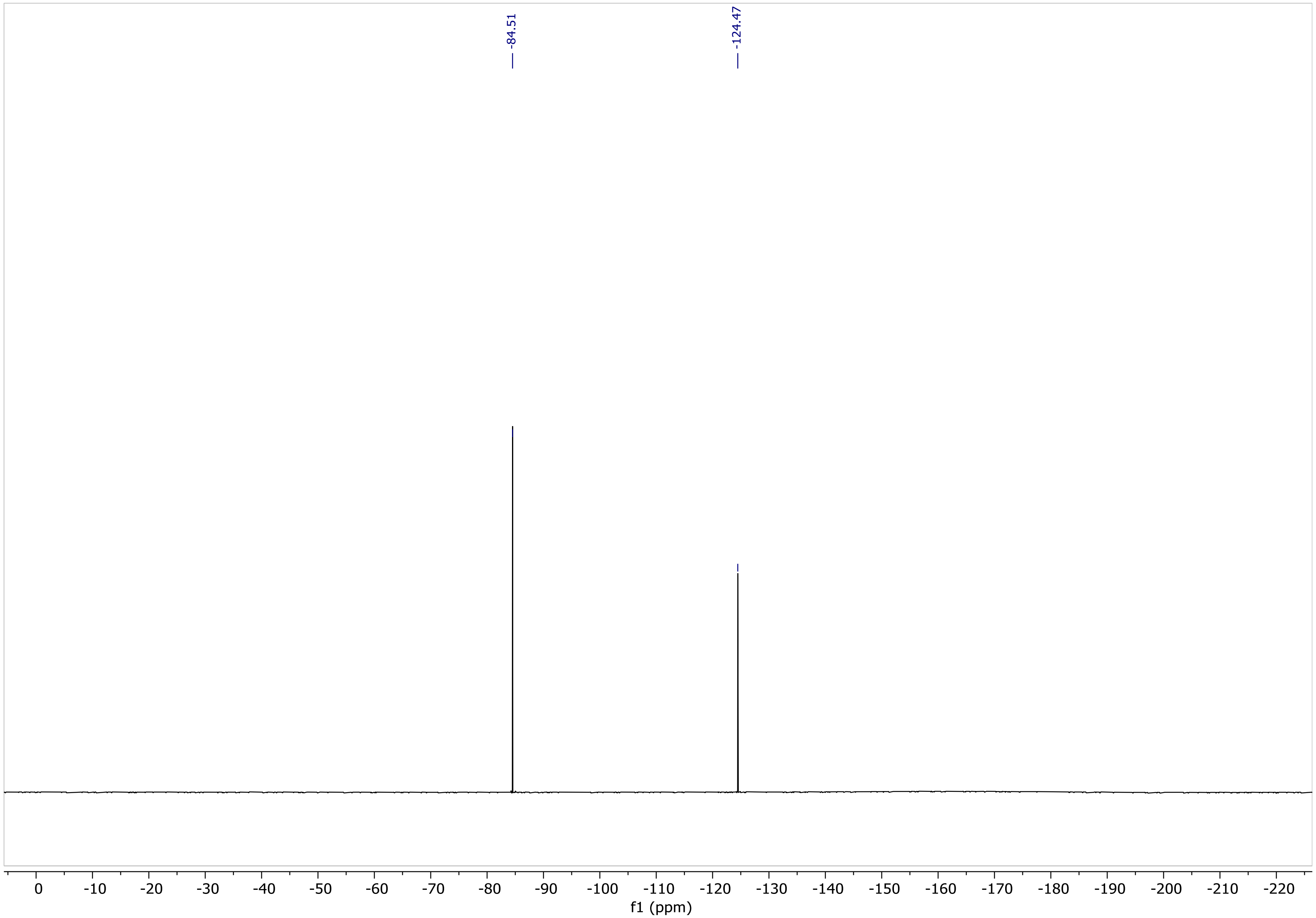

^{1}H NMR (700 MHz, CD_3CN) for 2,2,2,3,3-pentafluoropropyl (1-((phenethoxycarbonyl)(phenyl)amino)ethyl)sulfamate (**S7**)

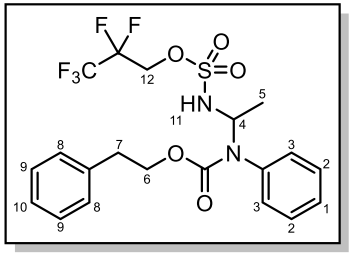

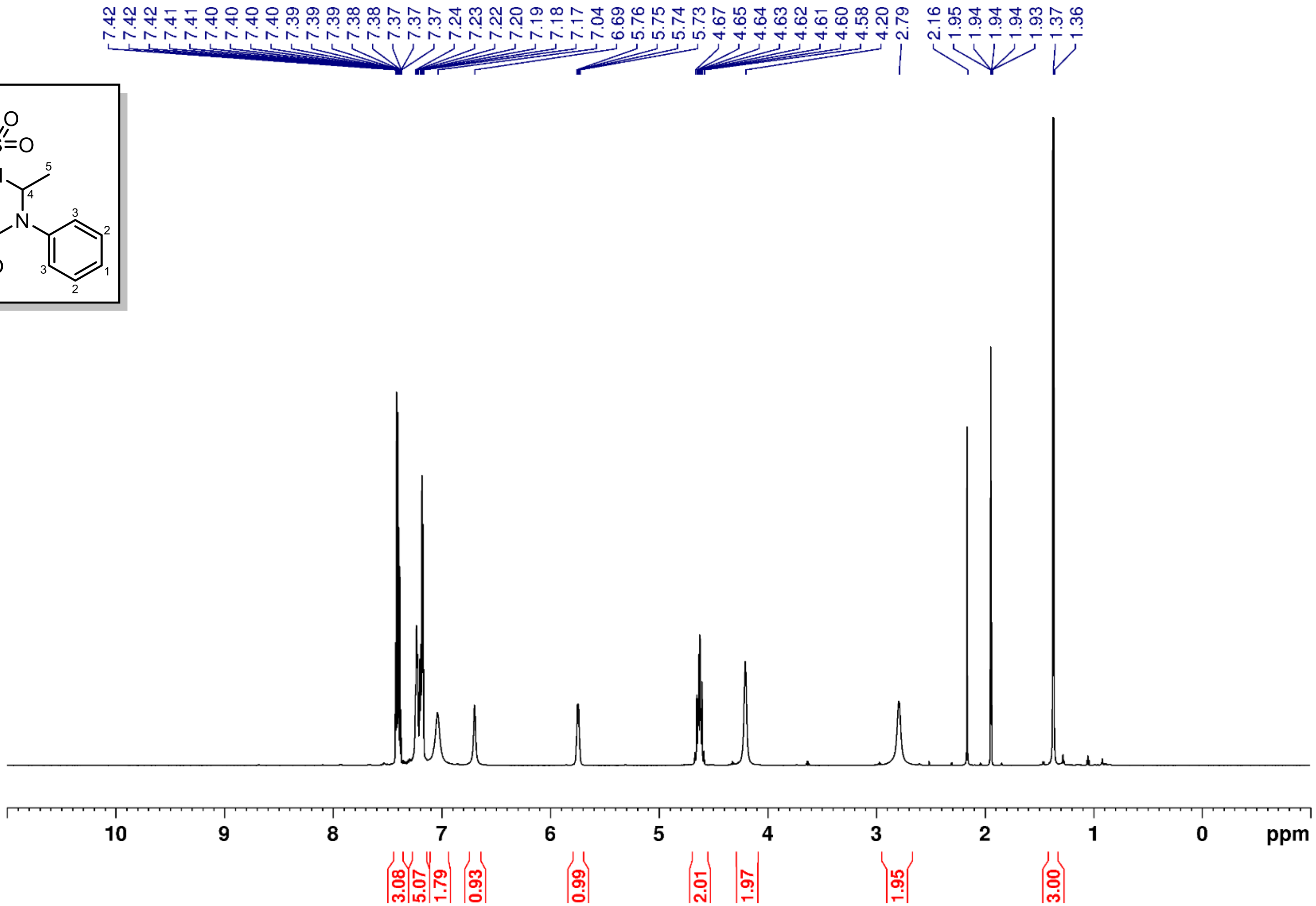

^{13}C NMR (176 MHz, CD_3CN) for 2,2,3,3,3-pentafluoropropyl (1-((phenethoxycarbonyl)(phenyl) amino)ethyl)sulfamate (**S7**)

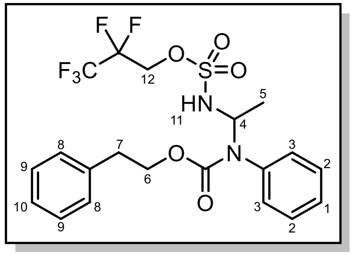

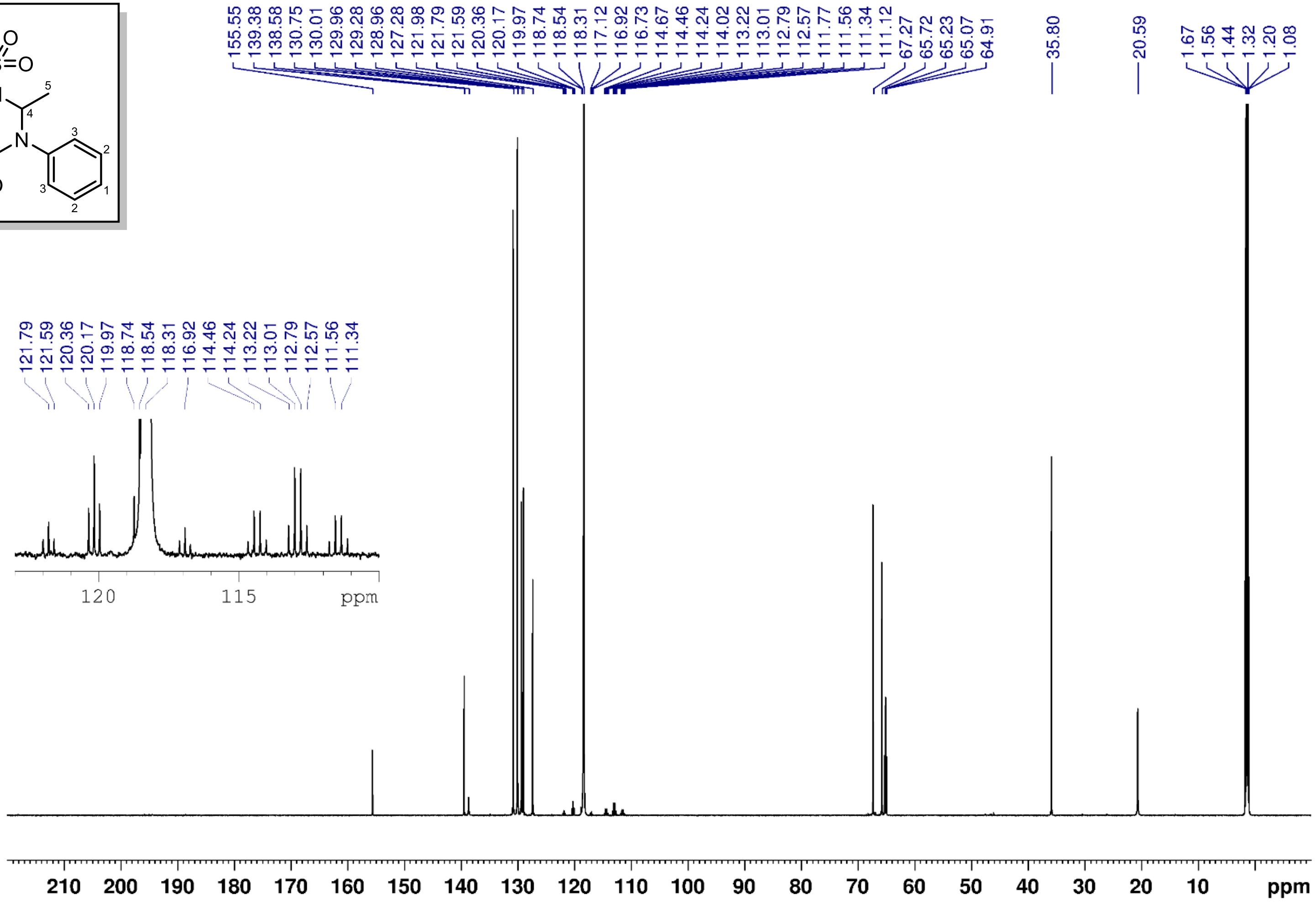

^{19}F NMR (376 MHz, CD_3CN) for 2,2,3,3,3-pentafluoropropyl (1-((phenethoxycarbonyl)(phenyl) amino)ethyl)sulfamate (**S7**)

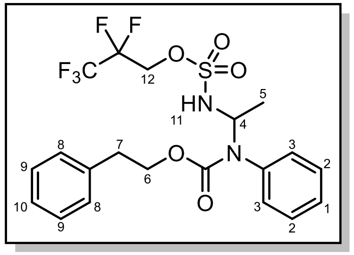

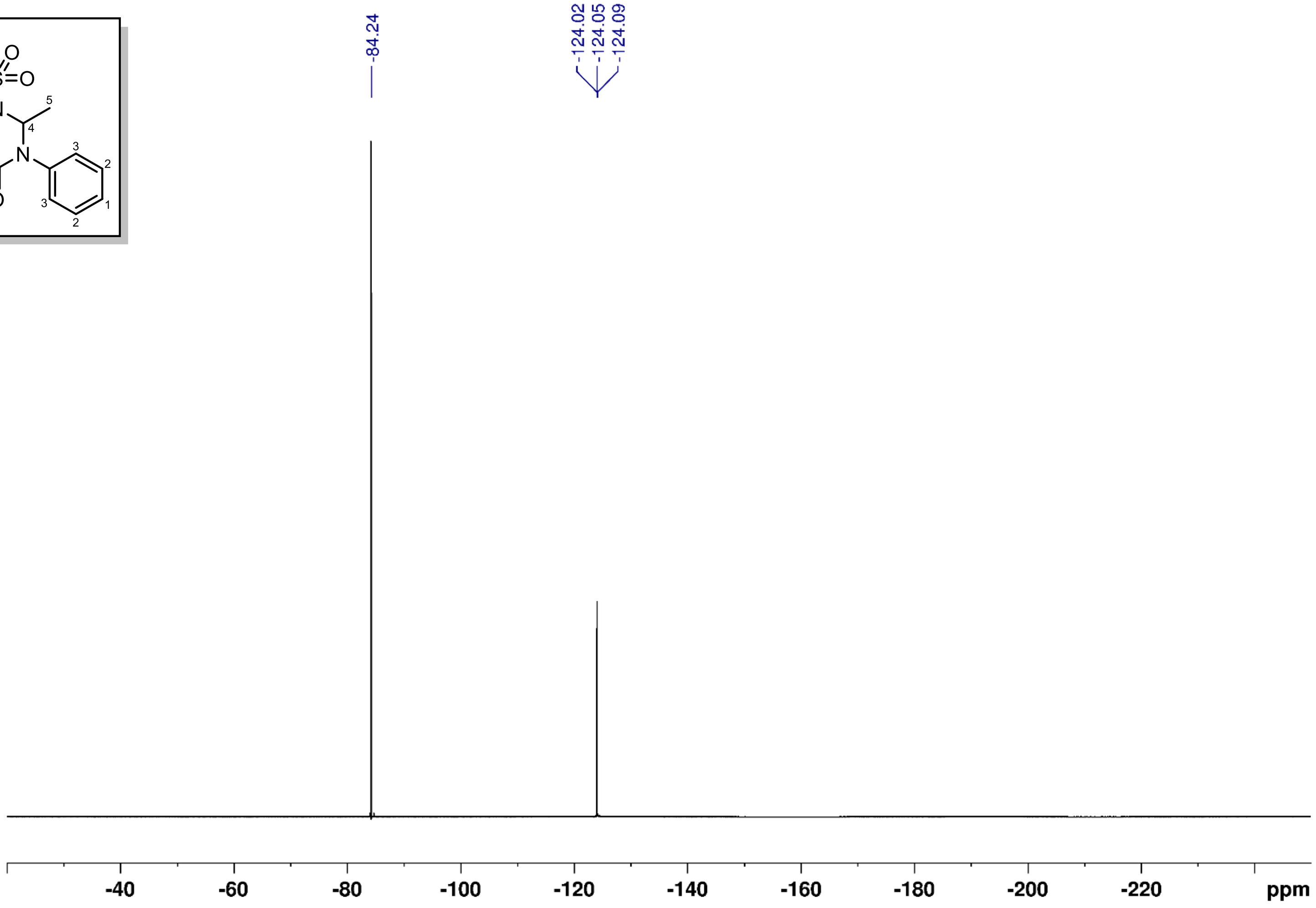

^{1}H NMR (700 MHz, CD_3CN) for phenethyl 3-(((2,2,3,3,3-pentafluoropropoxy)sulfonyl)amino)morpholine-4-carboxylate

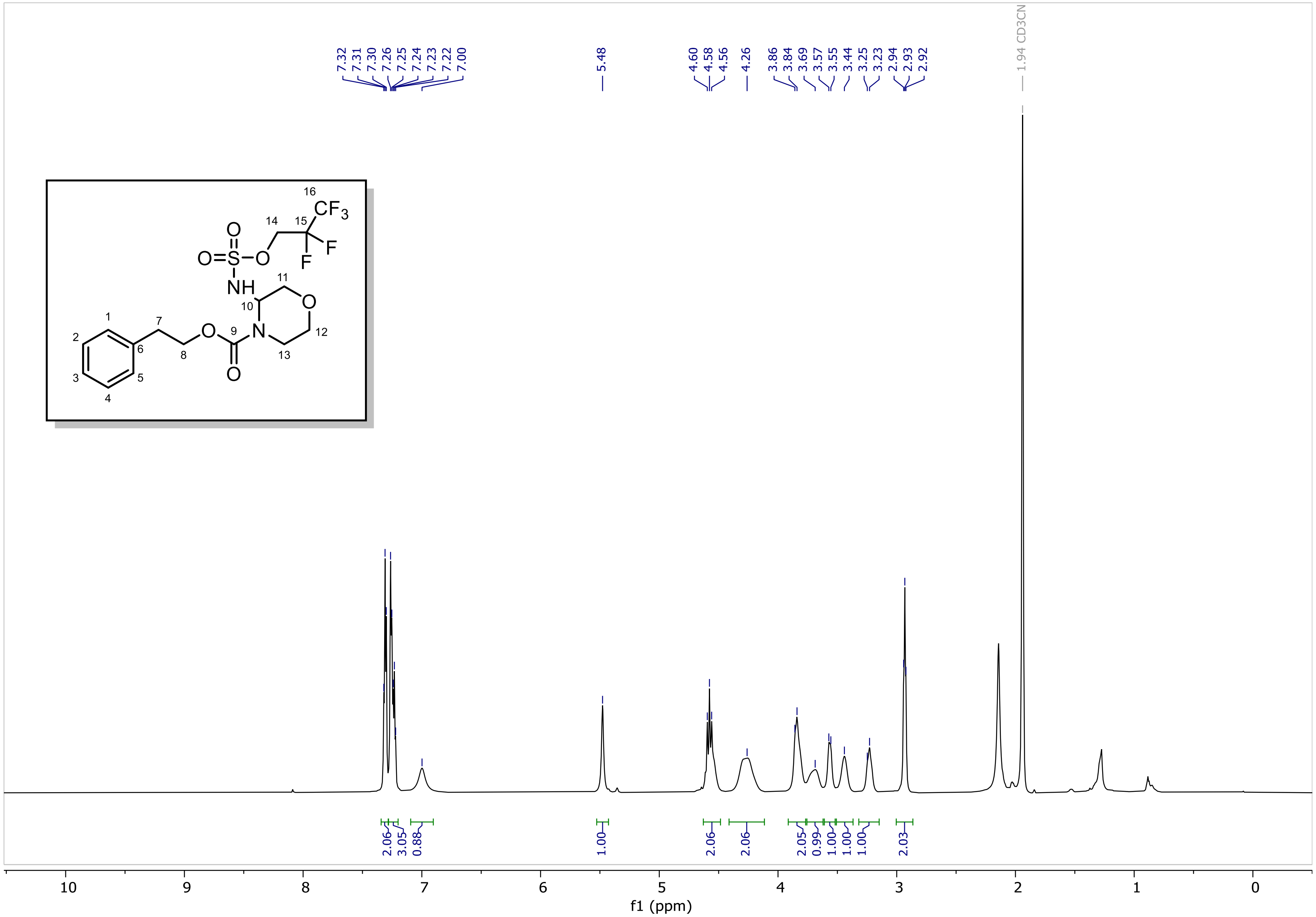

^{13}C NMR (176 MHz, CD_3CN) for phenethyl 3-(((2,2,3,3,3-pentafluoropropoxy)sulfonyl)amino)morpholine-4-carboxylate

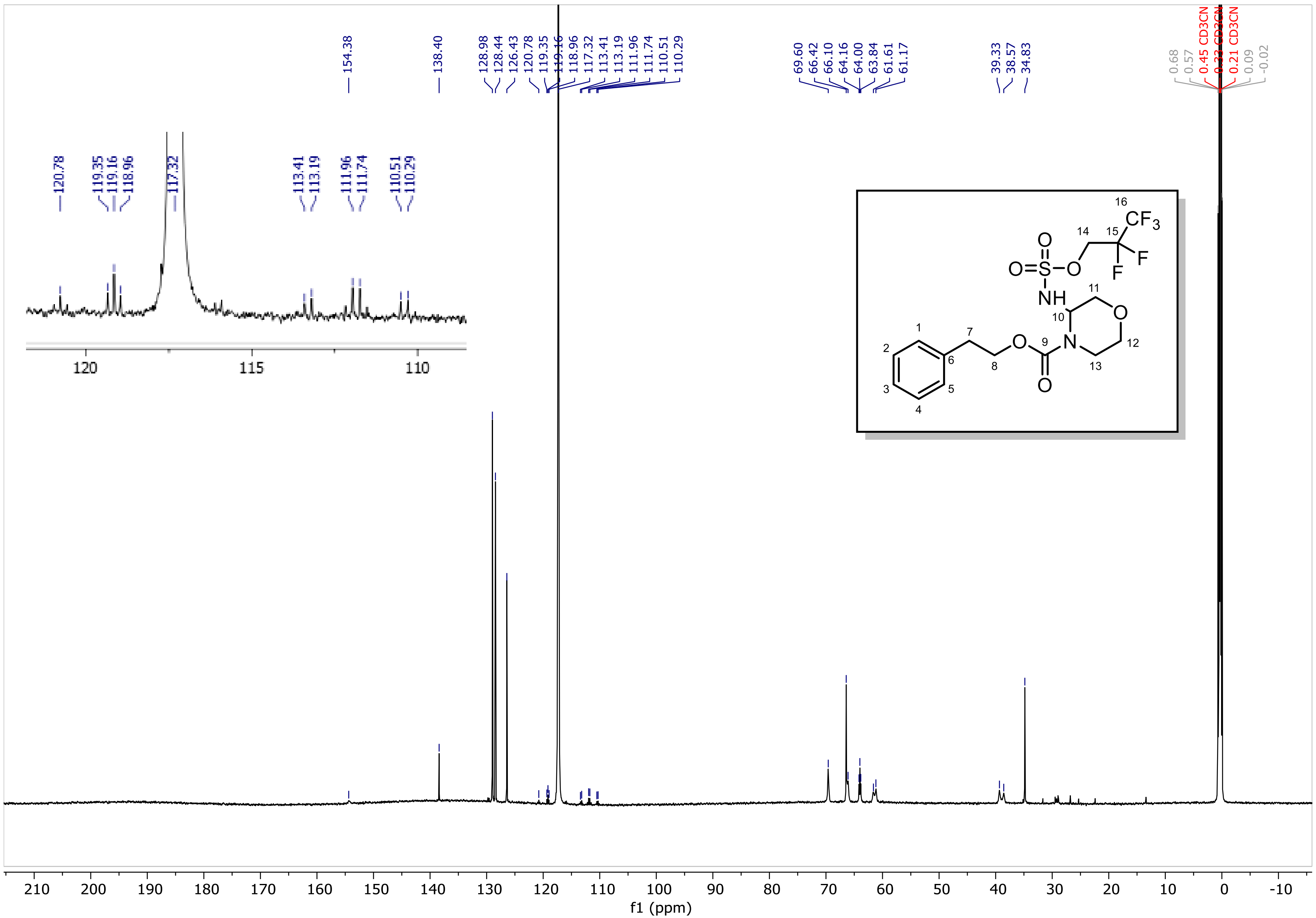

^{19}F NMR (376 MHz, CD_3CN) for phenethyl 3-(((2,2,3,3,3-pentafluoropropoxy)sulfonyl)amino)morpholine-4-carboxylate

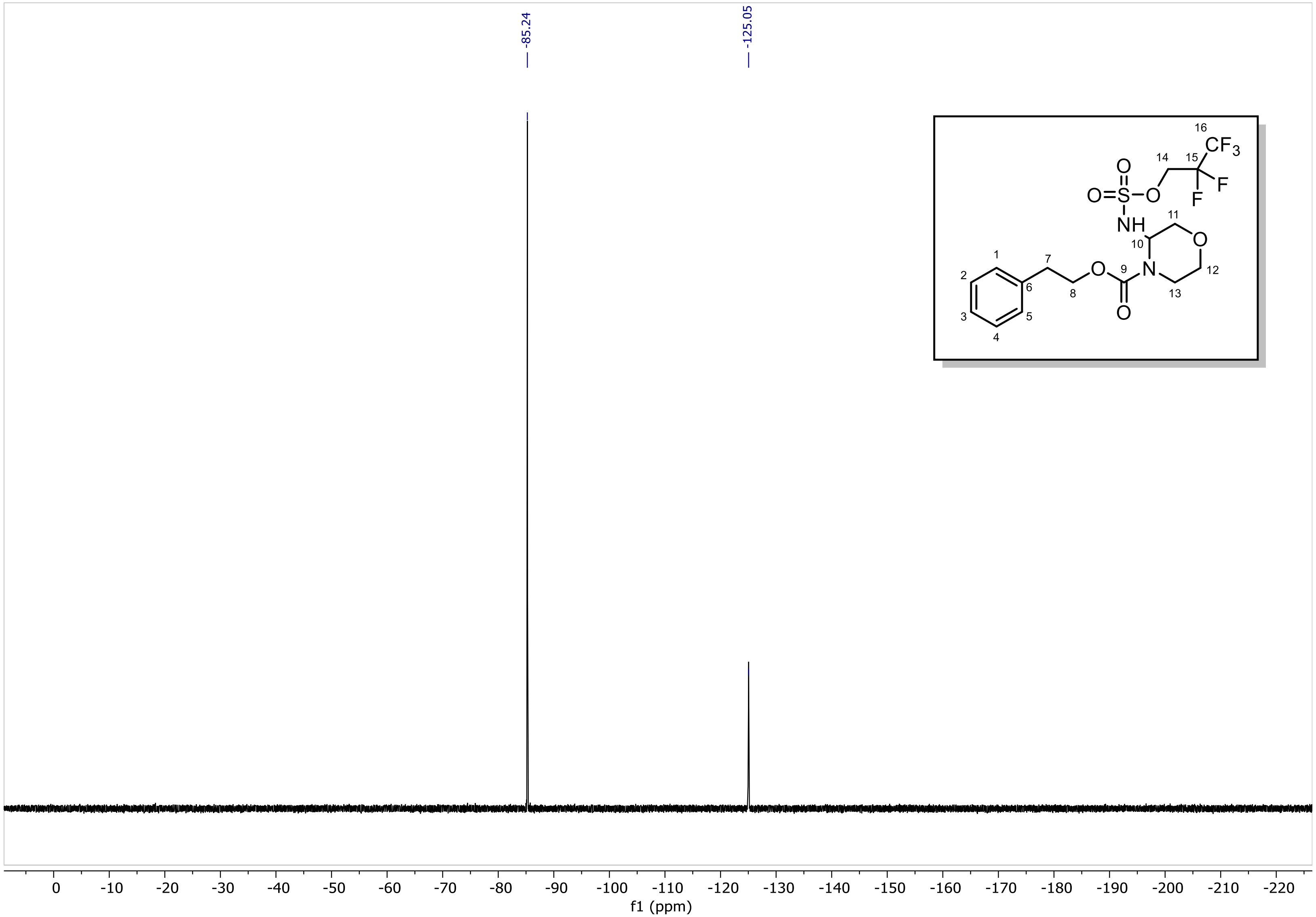

^{1}H NMR (500 MHz, CD_3CN) for (*R*)-1-phenylpropan-2-yl ethyl(phenyl)carbamate (**(*R*)-10s**)

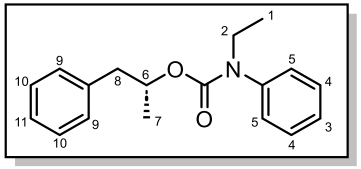

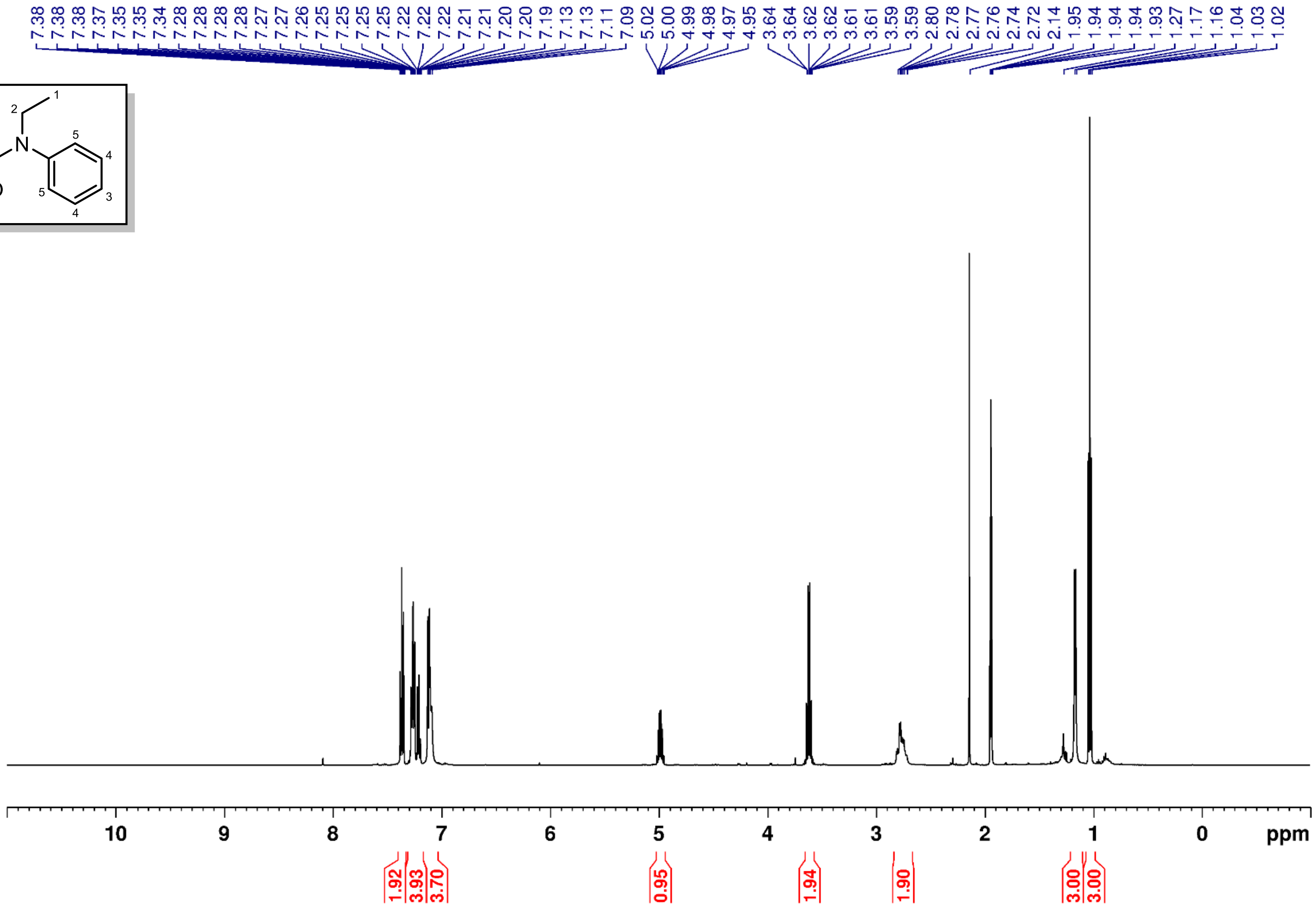

^{1}H NMR (700 MHz, CD_3CN) for 2,2,3,3,3-pentafluoropropyl ((1*S*,2*S*)-2-((ethyl(phenyl)carbamoyl)oxy)-1-phenylpropyl)sulfamate (**11s**)

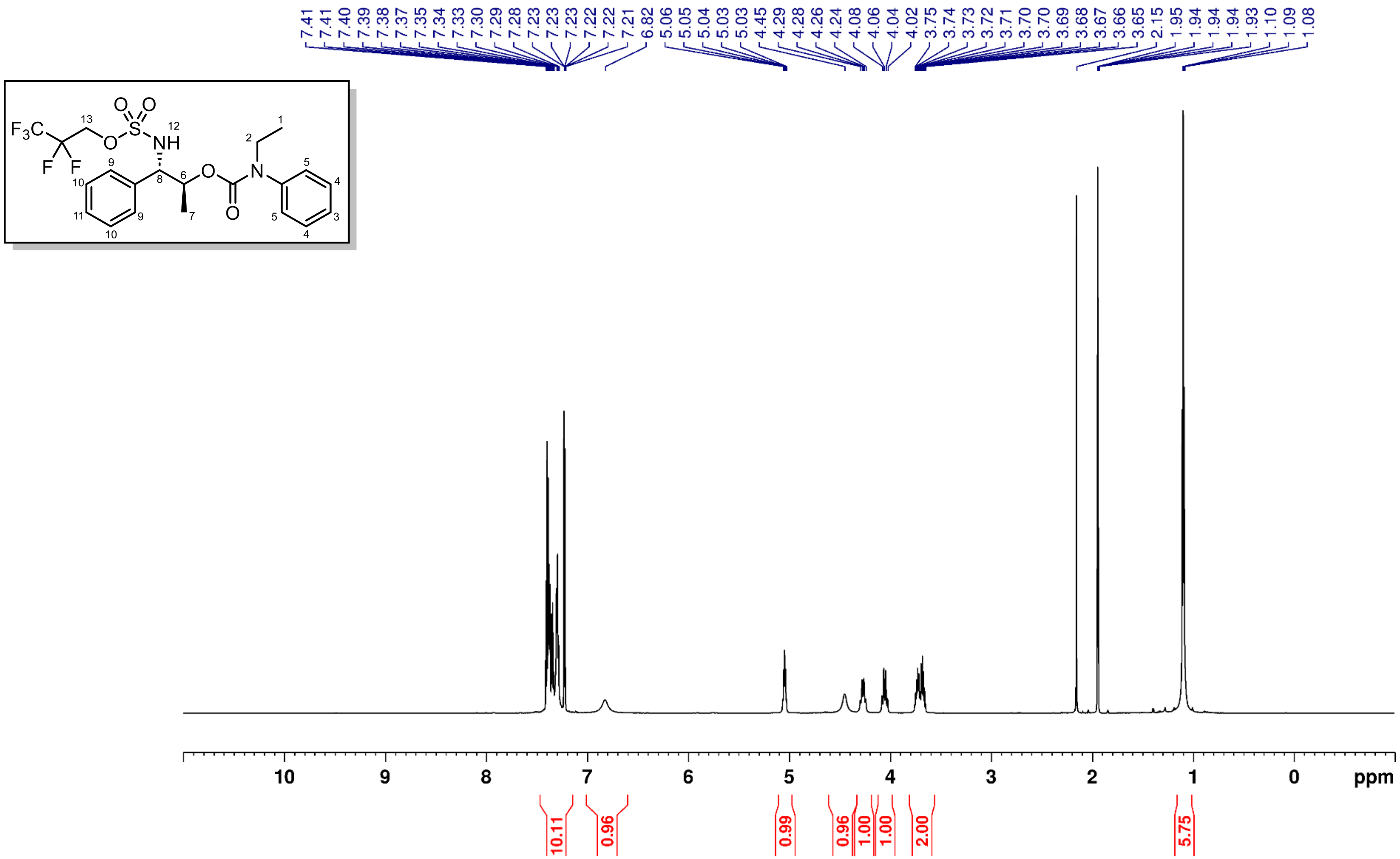

^{1}H NMR (700 MHz, CD_3CN) for 2,2,3,3,3-pentafluoropropyl ((1*R*,2*R*)-2-((ethyl(phenyl)carbamoyl)oxy)-1-phenylpropyl)sulfamate (***ent*-11s**)

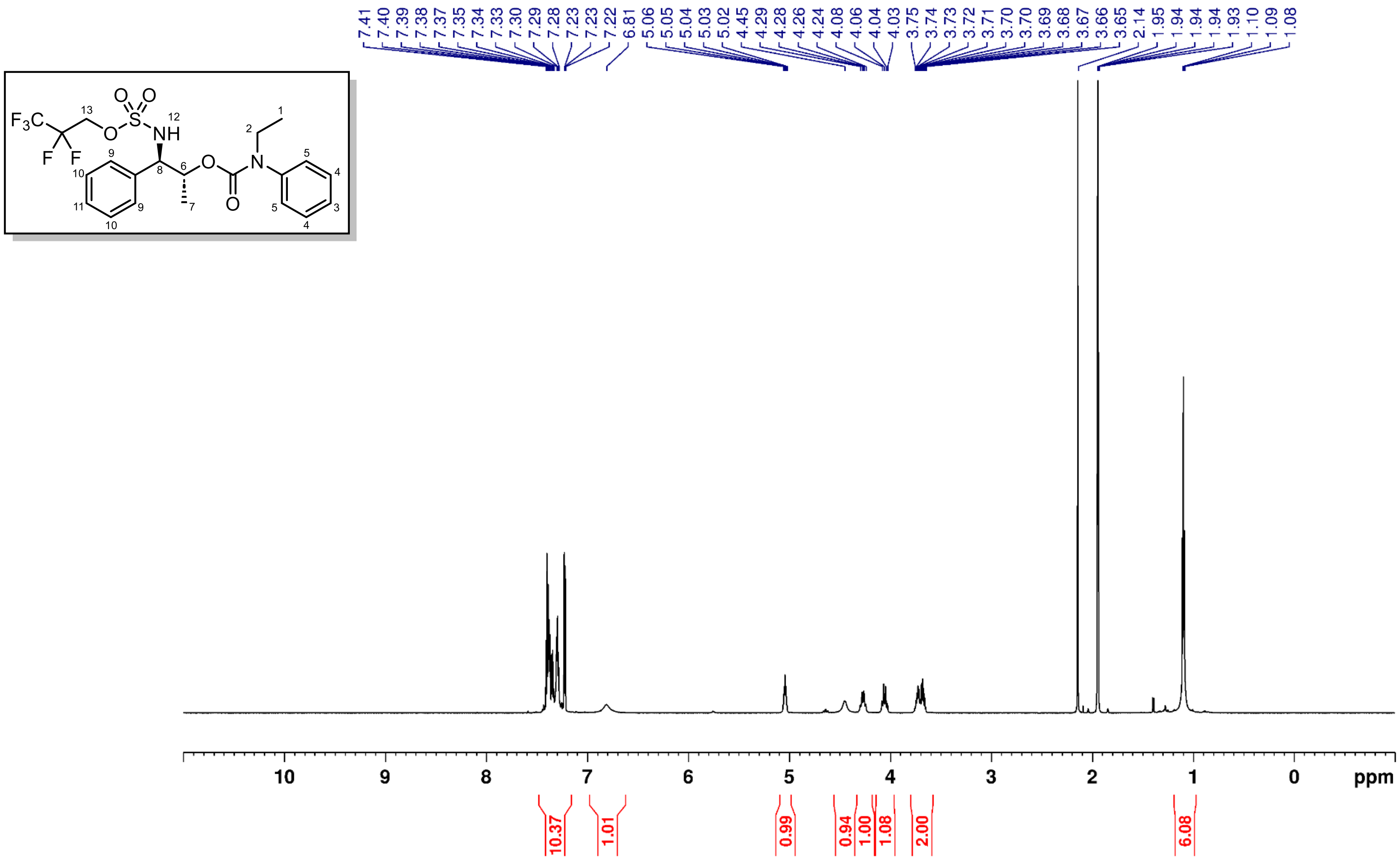

^{13}C NMR (176 MHz, CD_3CN) for 2,2,3,3,3-pentafluoropropyl ((1*S*,2*S*)-2-((ethyl(phenyl)carbamoyl)oxy)-1-phenylpropyl)sulfamate (**11s**)

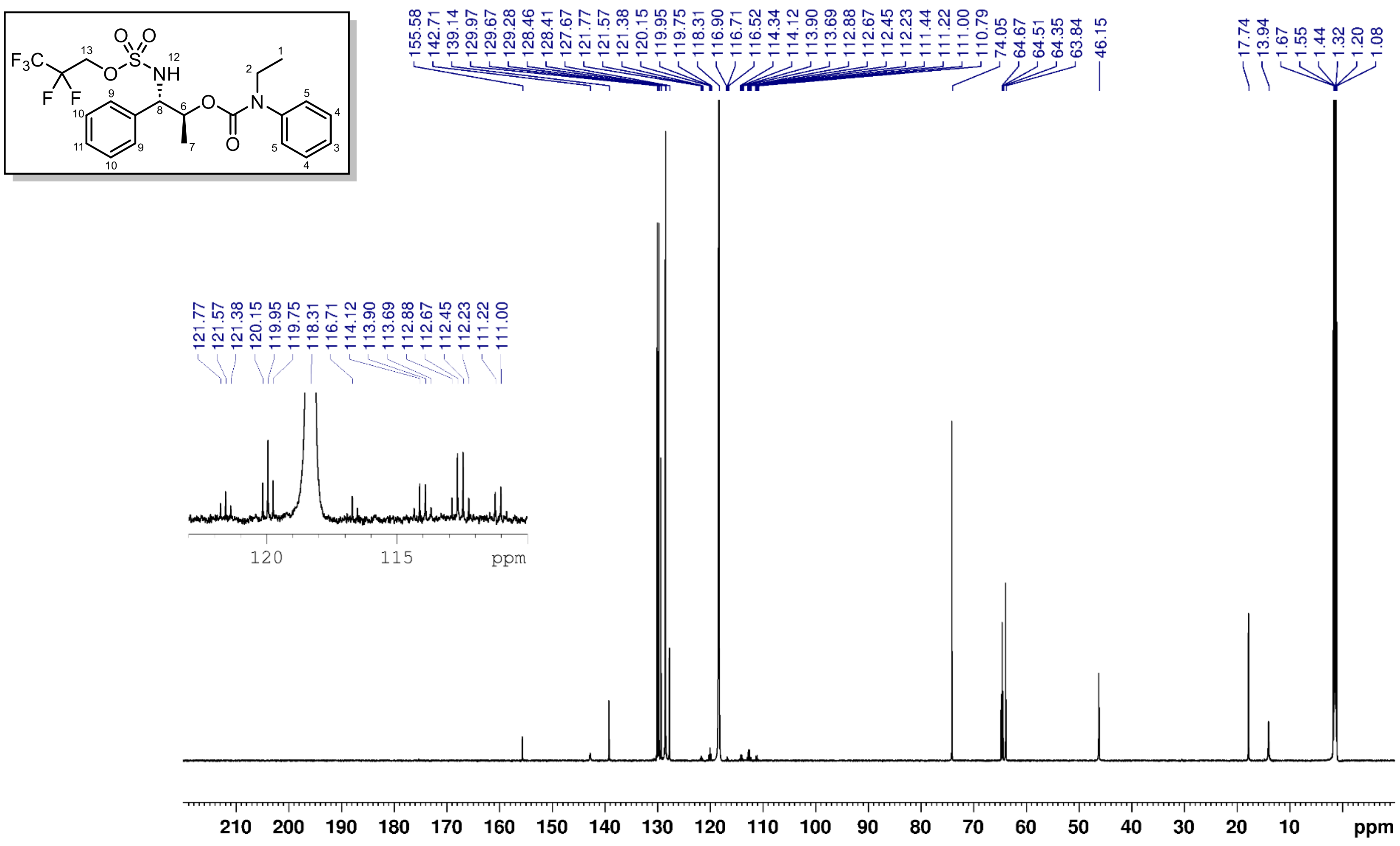

^{19}F NMR (376 MHz, CD_3CN) for 2,2,3,3,3-pentafluoropropyl ((1*S*,2*S*)-2-((ethyl(phenyl)carbamoyl)oxy)-1-phenylpropyl)sulfamate (**11s**)

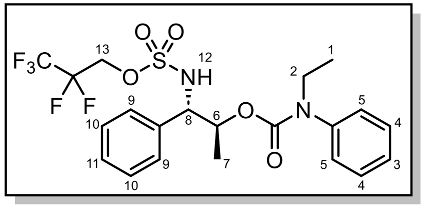

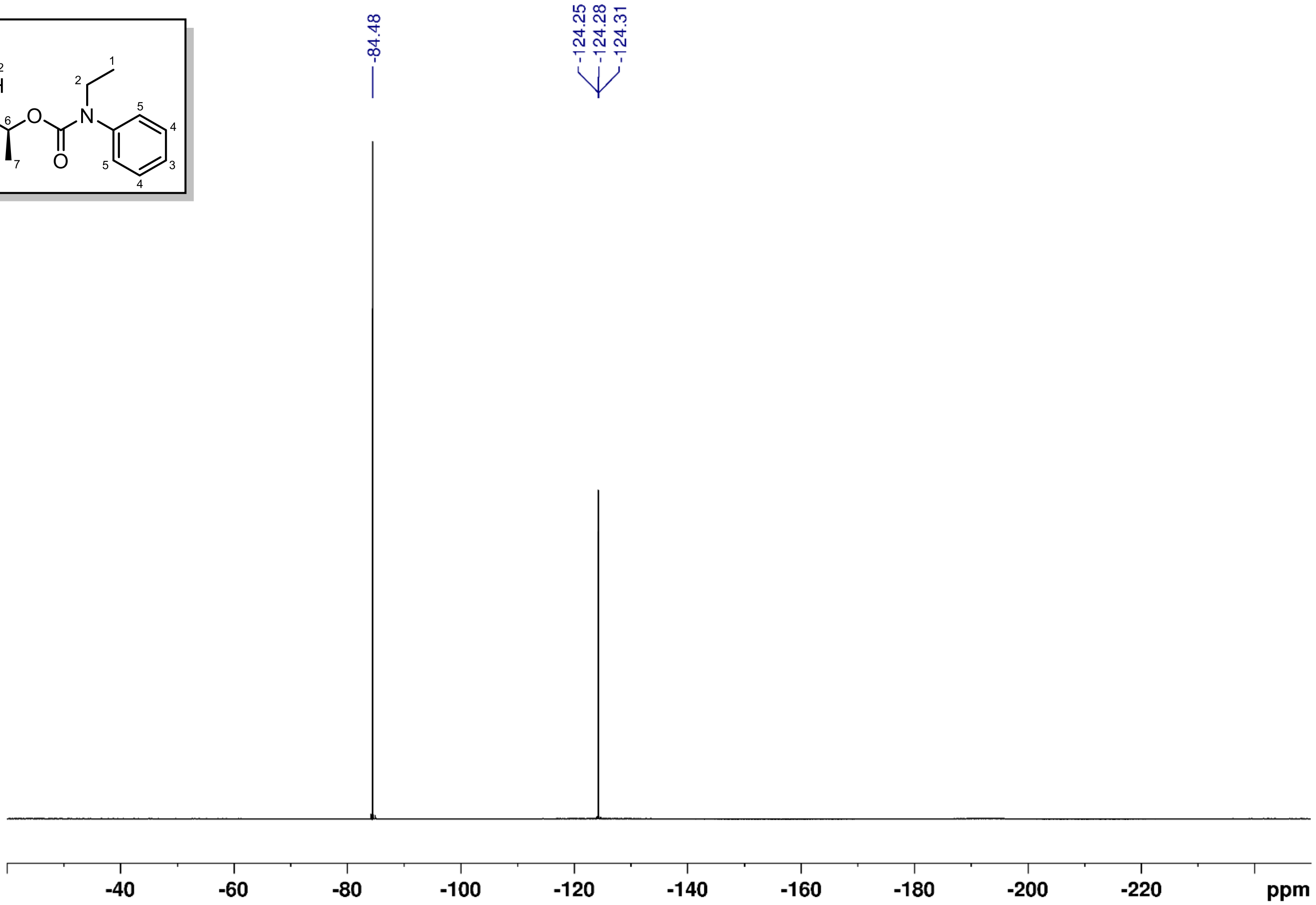

^{1}H NMR (700 MHz, CD_3CN) for 2,2,3,3,3-pentafluoropropyl (4*S*,5*S*)-5-methyl-2-oxo-4-phenyloxazolidine-3-sulfonate (**S8**)

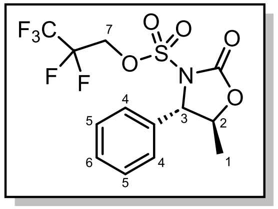

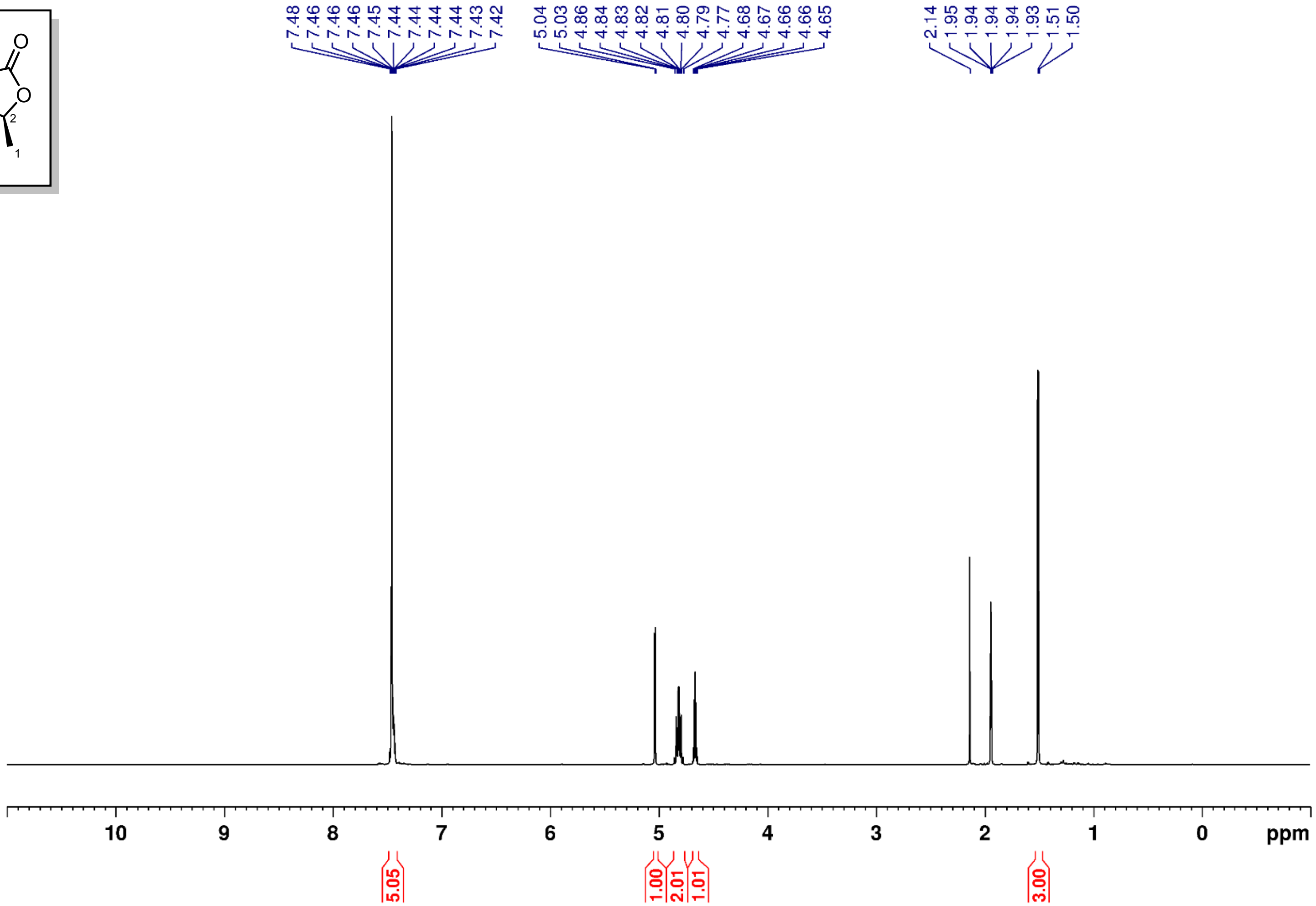

^{13}C NMR (176 MHz, CD_3CN) for 2,2,3,3,3-pentafluoropropyl (4*S*,5*S*)-5-methyl-2-oxo-4-phenyloxazolidine-3-sulfonate (**S8**)

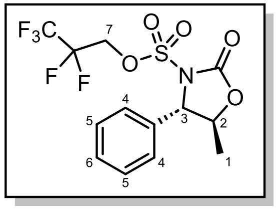

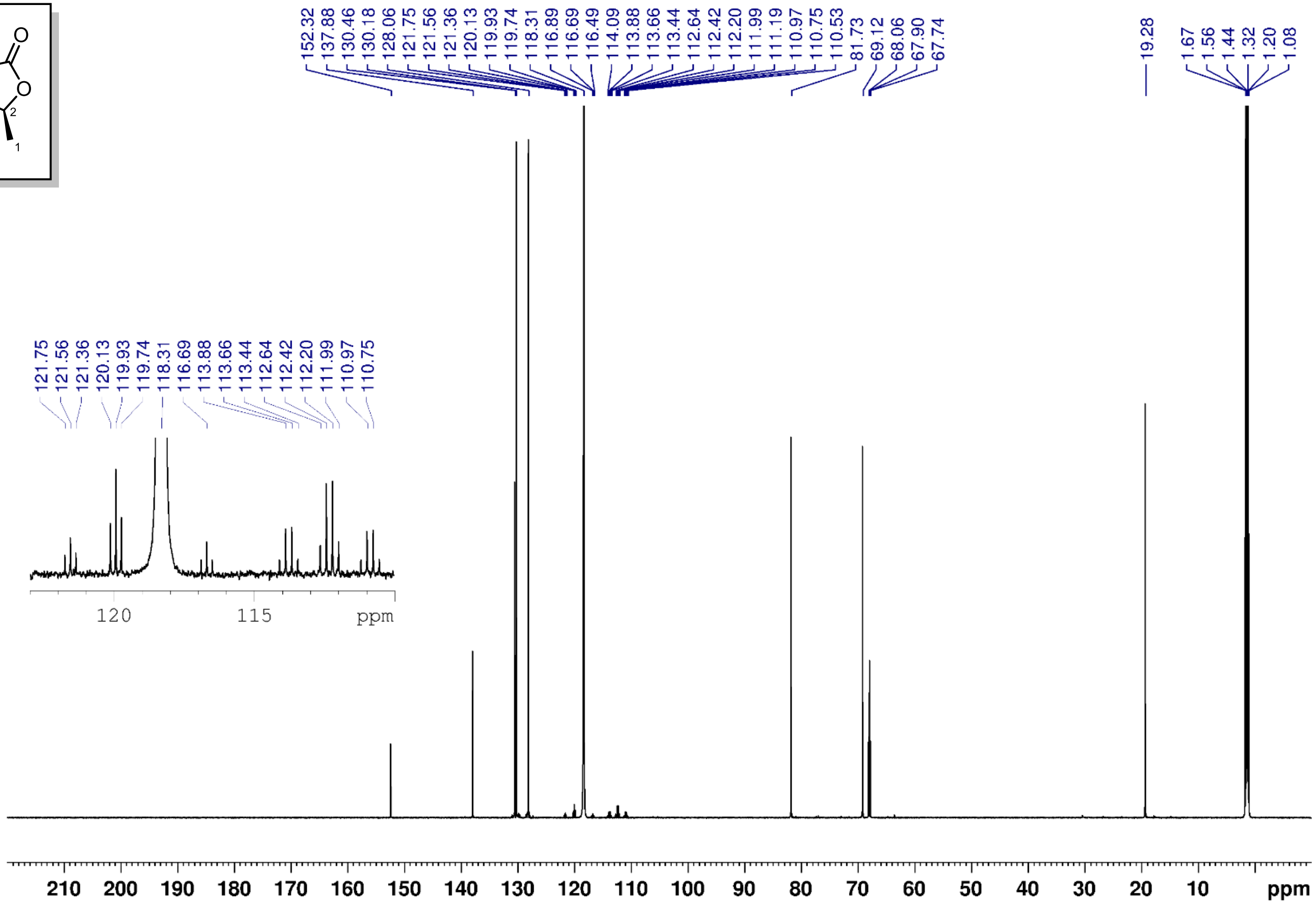

^{19}F NMR (376 MHz, CD_3CN) for 2,2,3,3,3-pentafluoropropyl (4*S*,5*S*)-5-methyl-2-oxo-4-phenyloxazolidine-3-sulfonate (**S8**)

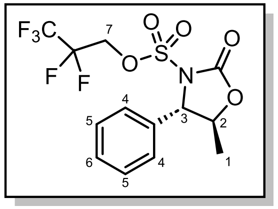

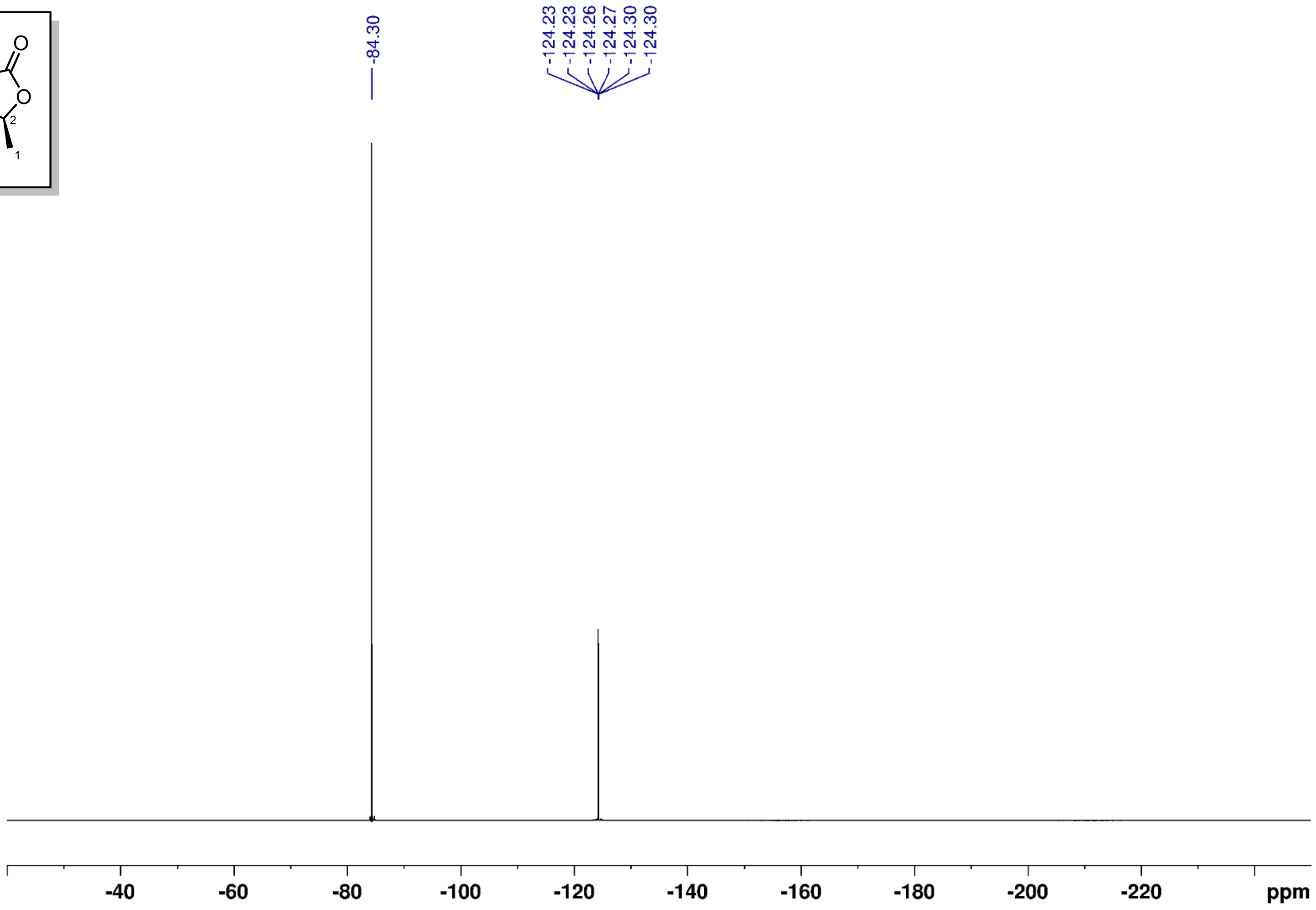

^{1}H-^{1}H NOESY (500 MHz, CD_3CN) for 2,2,3,3,3-pentafluoropropyl (4*S*,5*S*)-5-methyl-2-oxo-4-phenyloxazolidine-3-sulfonate (**S8**)

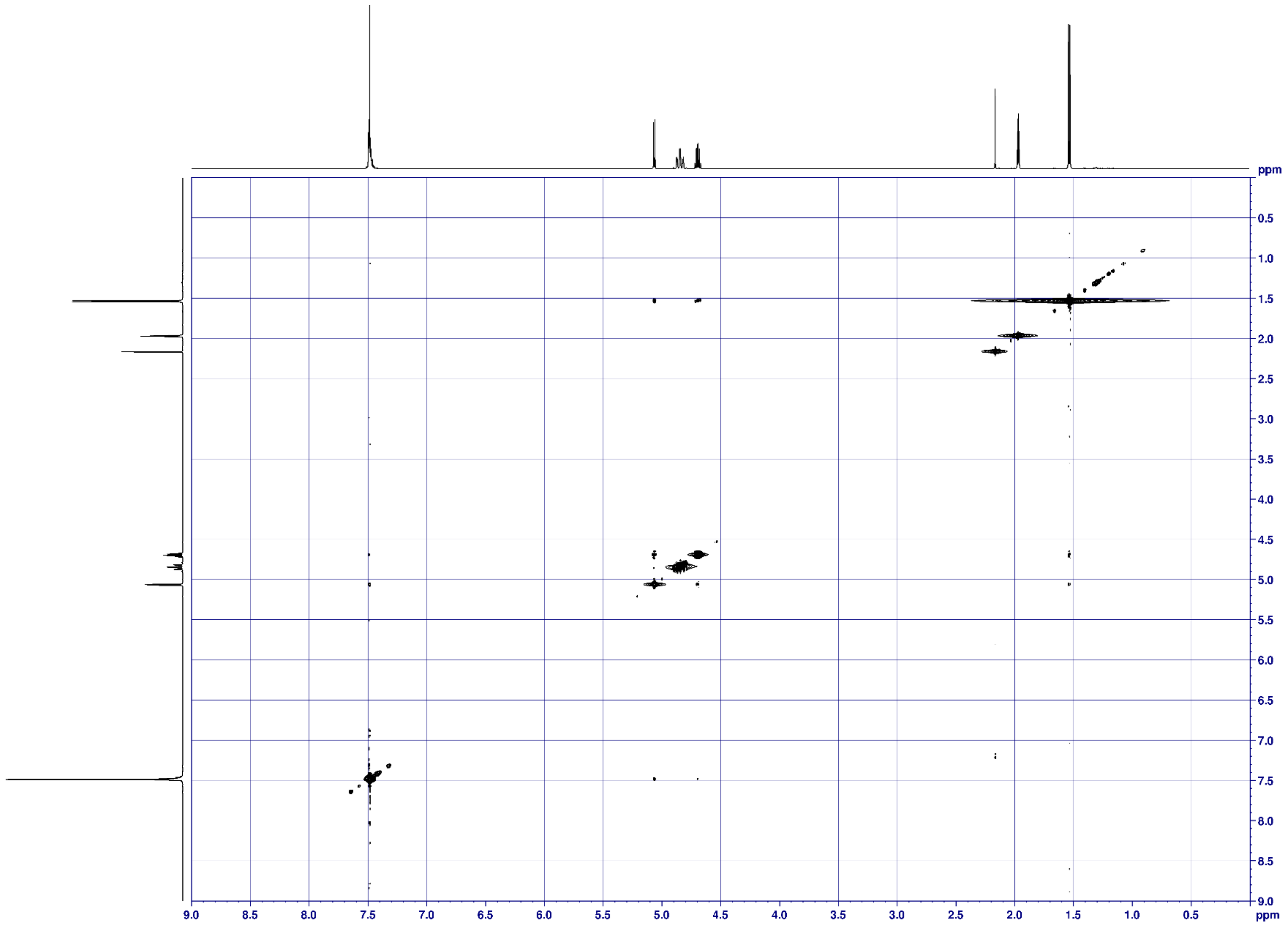

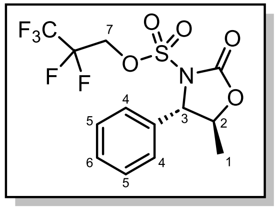

^{1}H-^{1}H NOESY (500 MHz, CD_3CN) for 2,2,3,3,3-pentafluoropropyl (4*S*,5*S*)-5-methyl-2-oxo-4-phenyloxazolidine-3-sulfonate (**S8**)

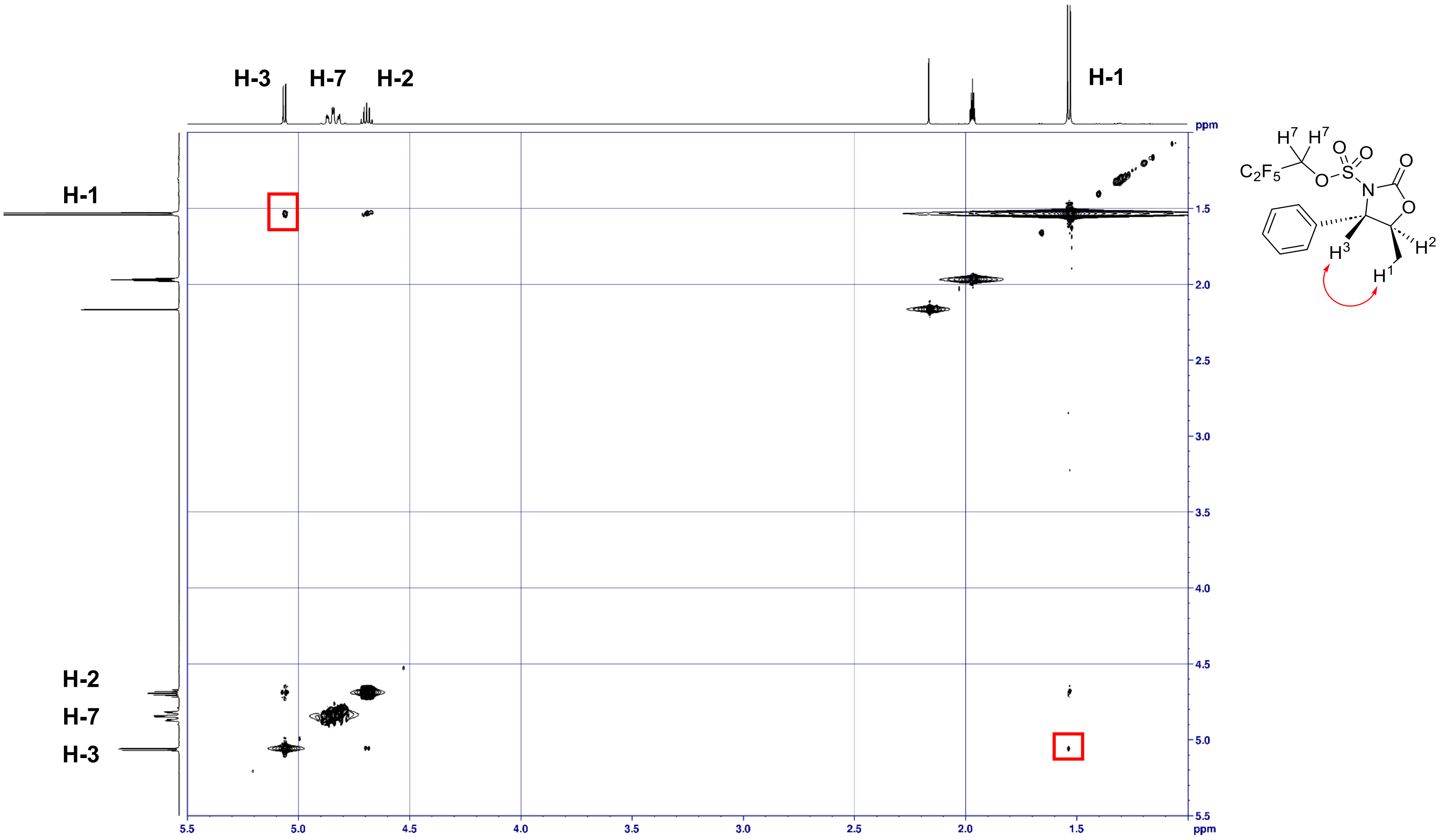

NMR Spectra for Post-Functionalisation Products from C-H Amination

^{1}H NMR (700 MHz, CD_3CN) for 2,2,3,3,3-pentafluoropropyl (*S*)-(2-hydroxy-1-phenylethyl)sulfamate (**12**)

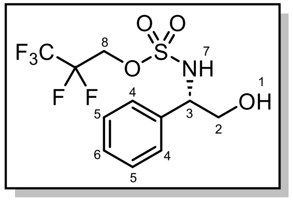

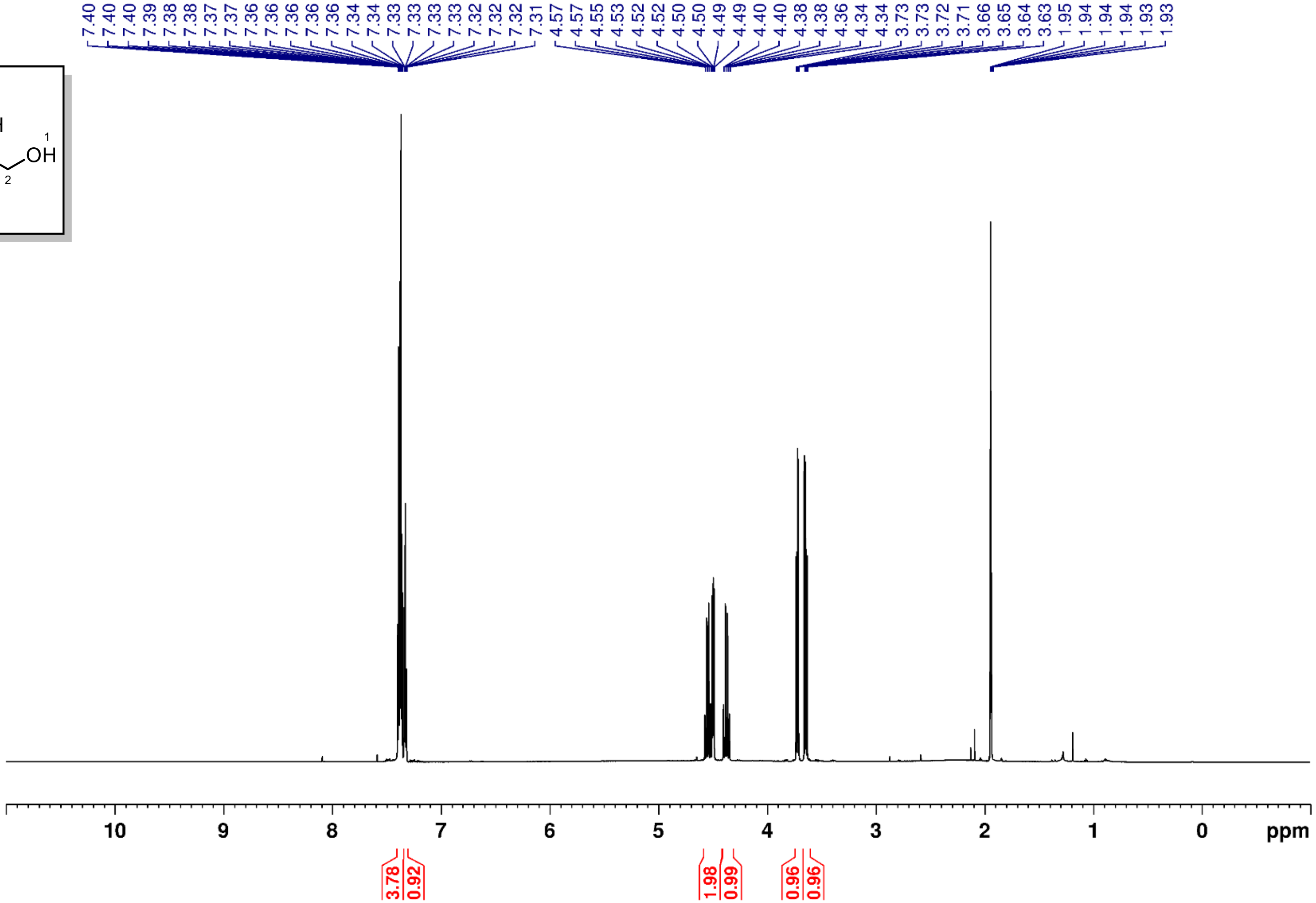

^{1}H NMR (700 MHz, CD_3CN) for 2,2,3,3,3-pentafluoropropyl (*R*)-(2-hydroxy-1-phenylethyl)sulfamate (***ent*-12**)

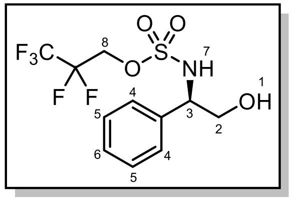

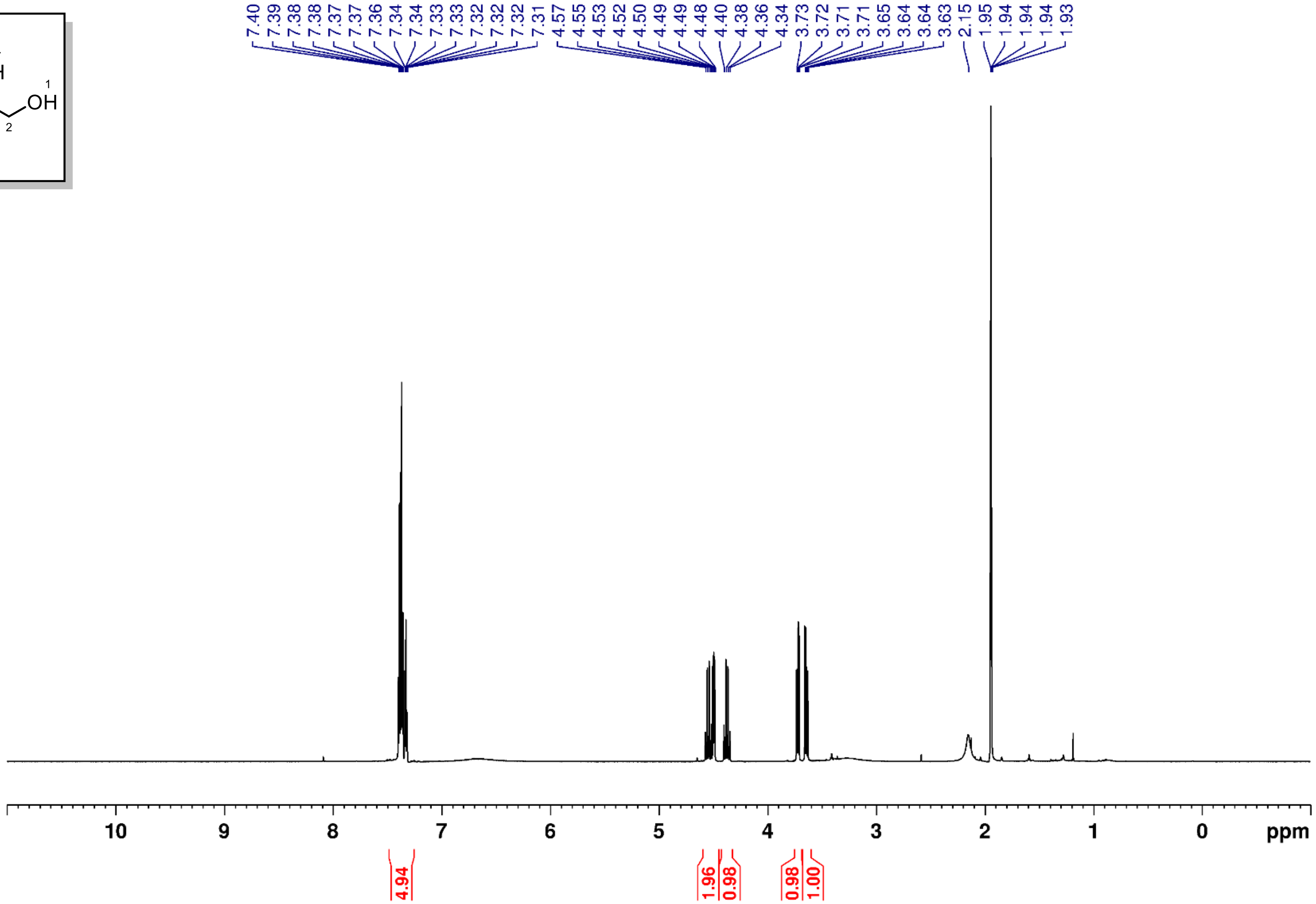

^{13}C NMR (176 MHz, CD_3CN) for 2,2,3,3,3-pentafluoropropyl (*S*)-(2-hydroxy-1-phenylethyl)sulfamate (**12**)

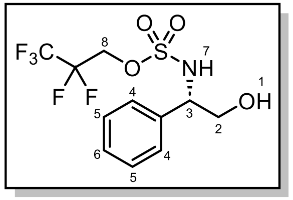

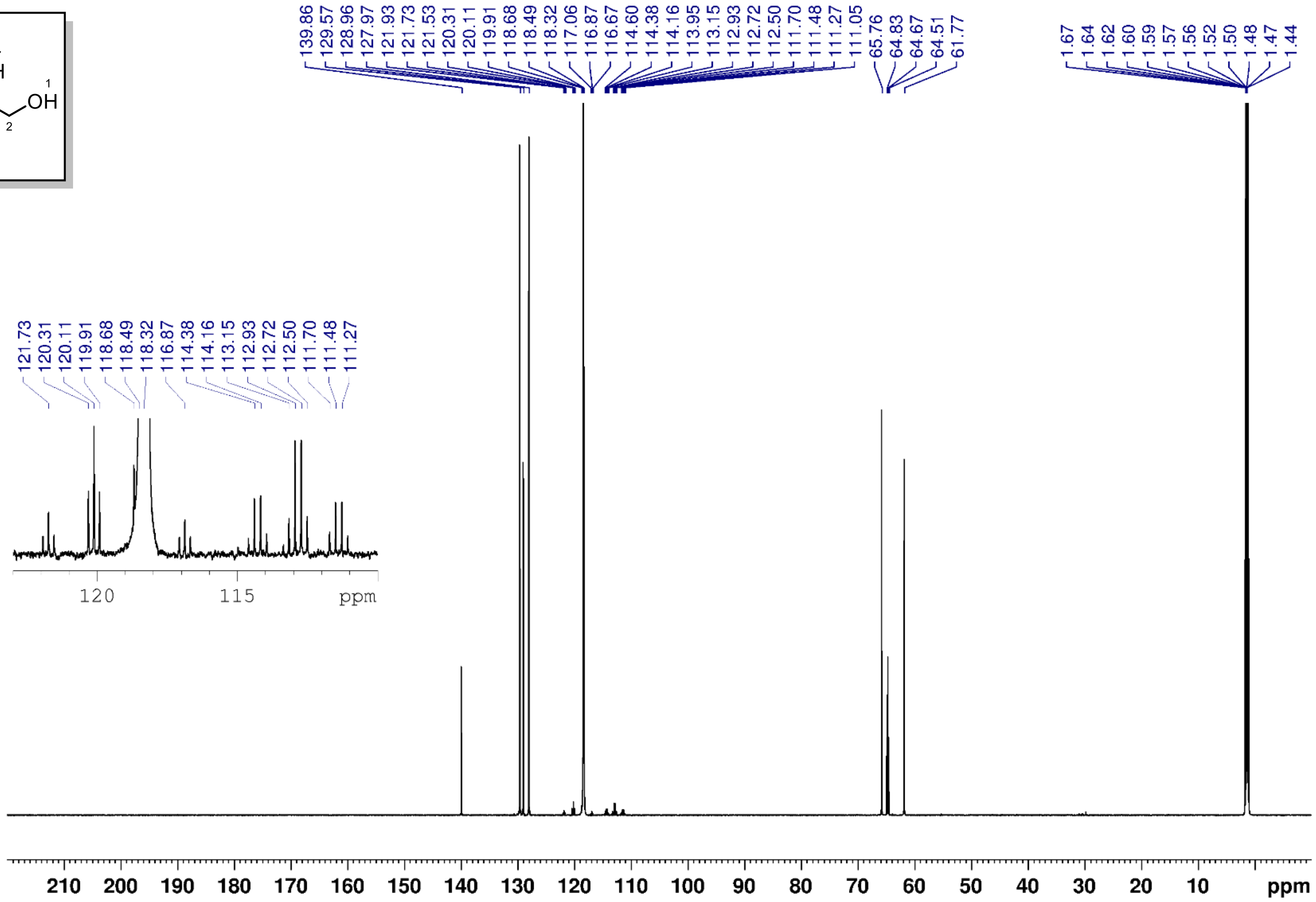

^{19}F NMR (376 MHz, CD_3CN) for 2,2,3,3,3-pentafluoropropyl (*S*)-(2-hydroxy-1-phenylethyl)sulfamate (**12**)

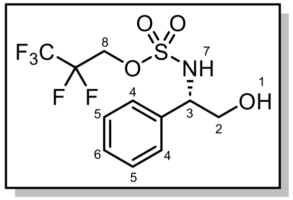

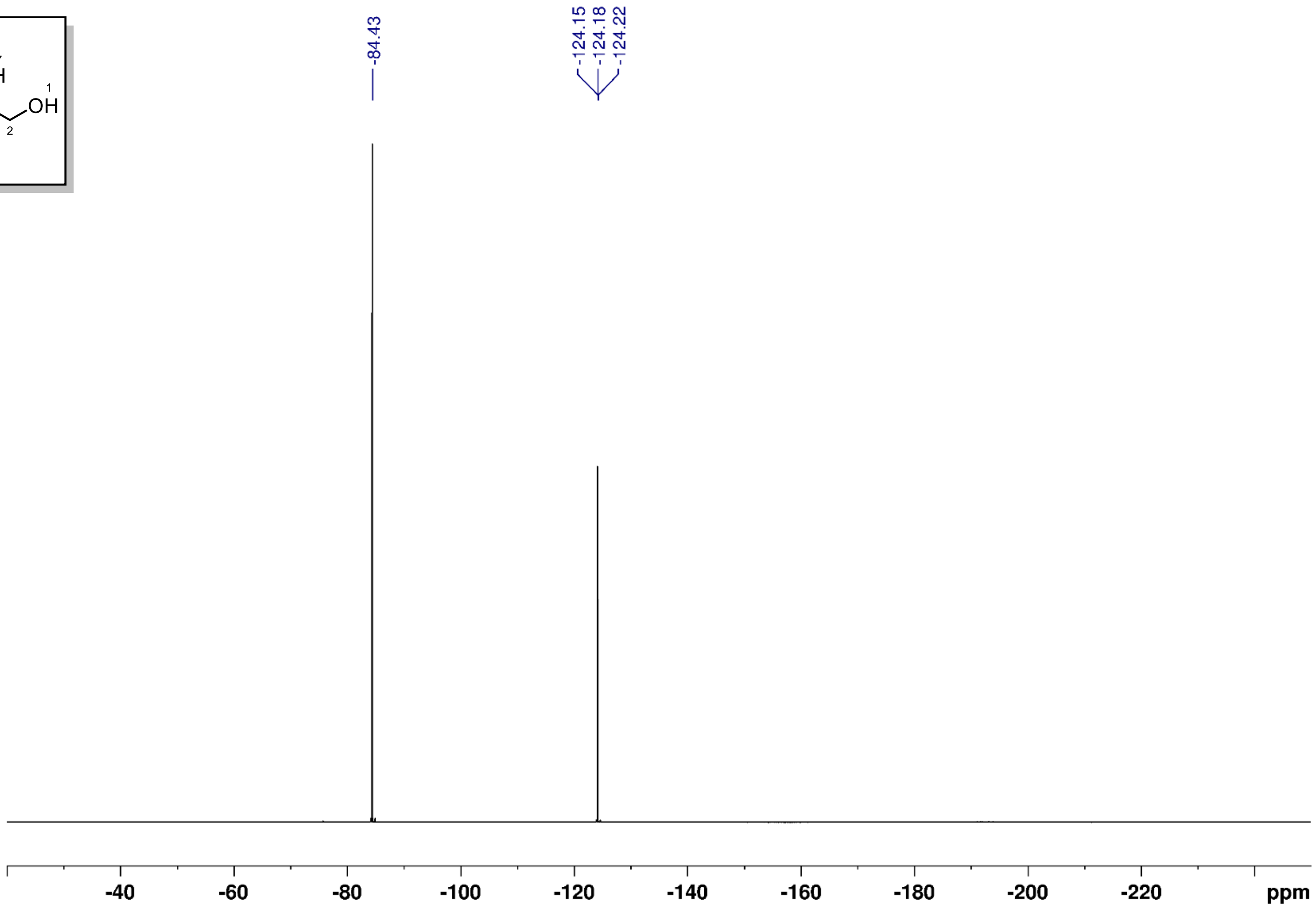

^{1}H NMR (700 MHz, CD_3CN) for (*S*)-2-amino-2-phenylethyl ethyl(phenyl)carbamate (**13**)

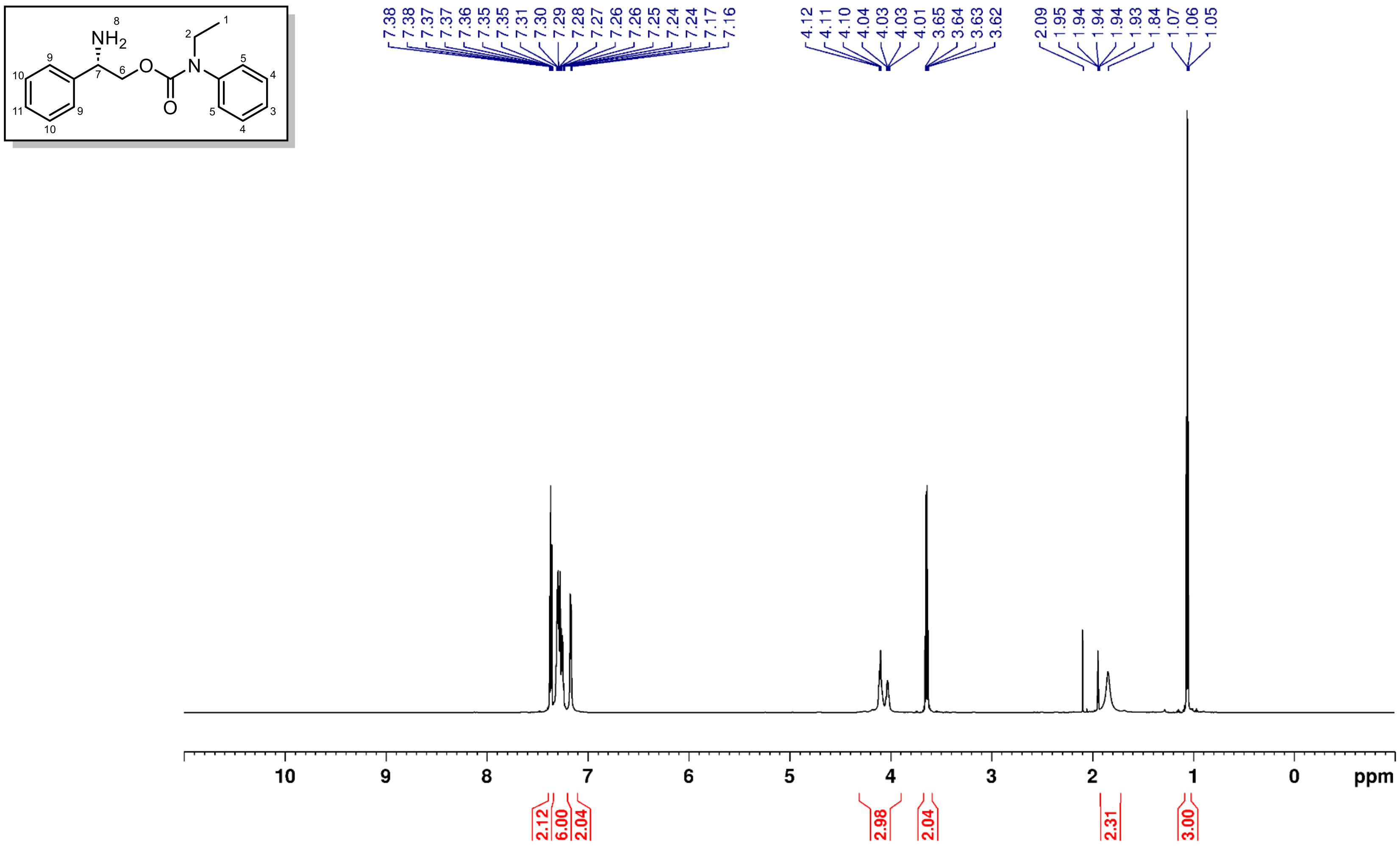

^{13}C NMR (176 MHz, CD_3CN) for (*S*)-2-amino-2-phenylethyl ethyl(phenyl)carbamate (**13**)

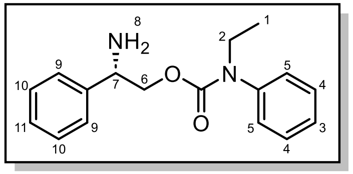

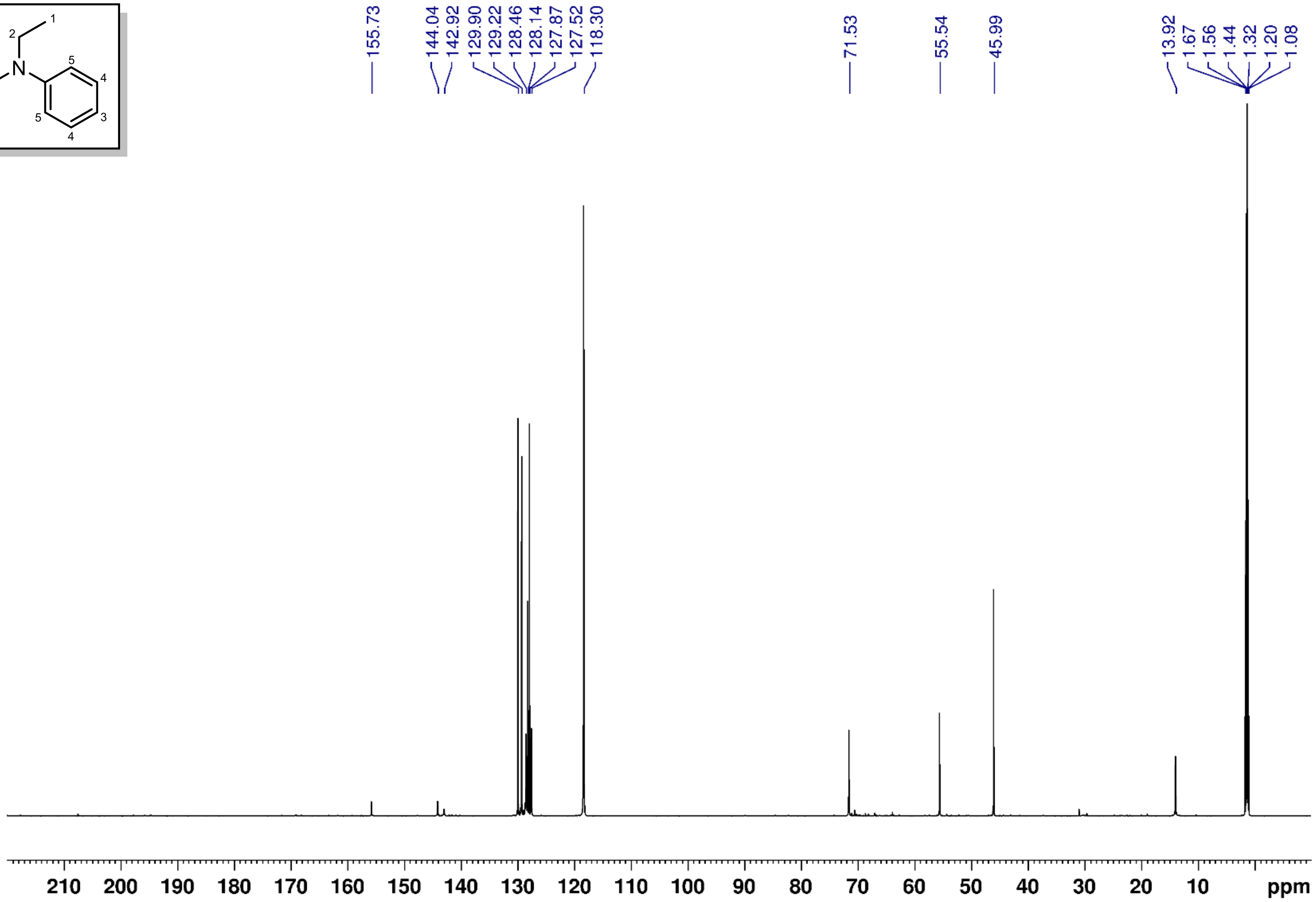

^{1}H NMR (700 MHz, CD_3CN) for (*S*)-2-((tert-butoxycarbonyl)amino)-2-phenylethyl ethyl(phenyl)carbamate (**14**)

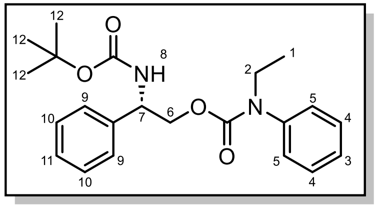

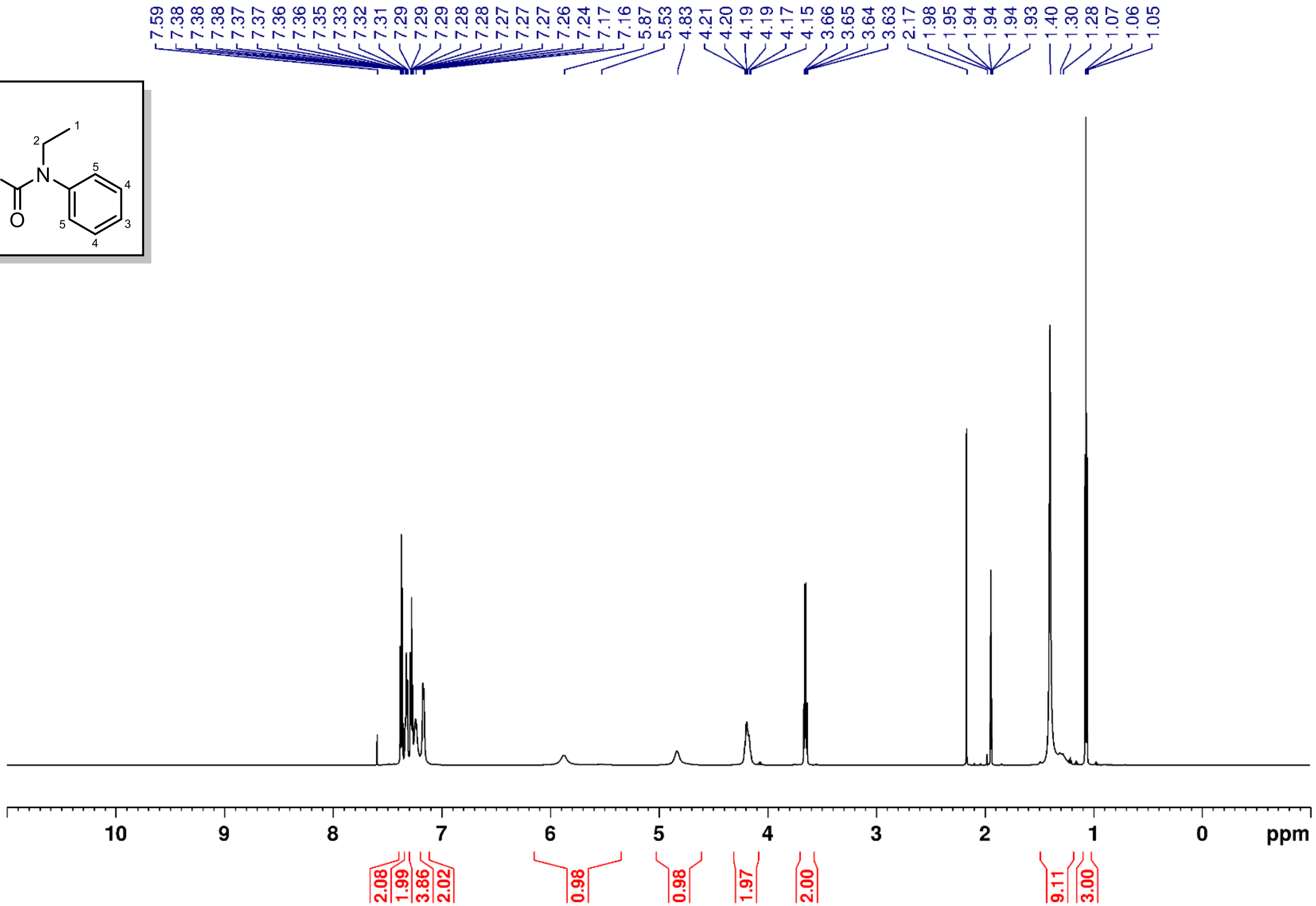

^{1}H NMR (700 MHz, CD_3CN) for (*R*)-2-((tert-butoxycarbonyl)amino)-2-phenylethyl ethyl(phenyl)carbamate (***ent*-14**)

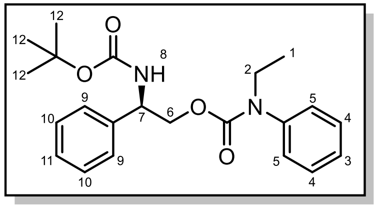

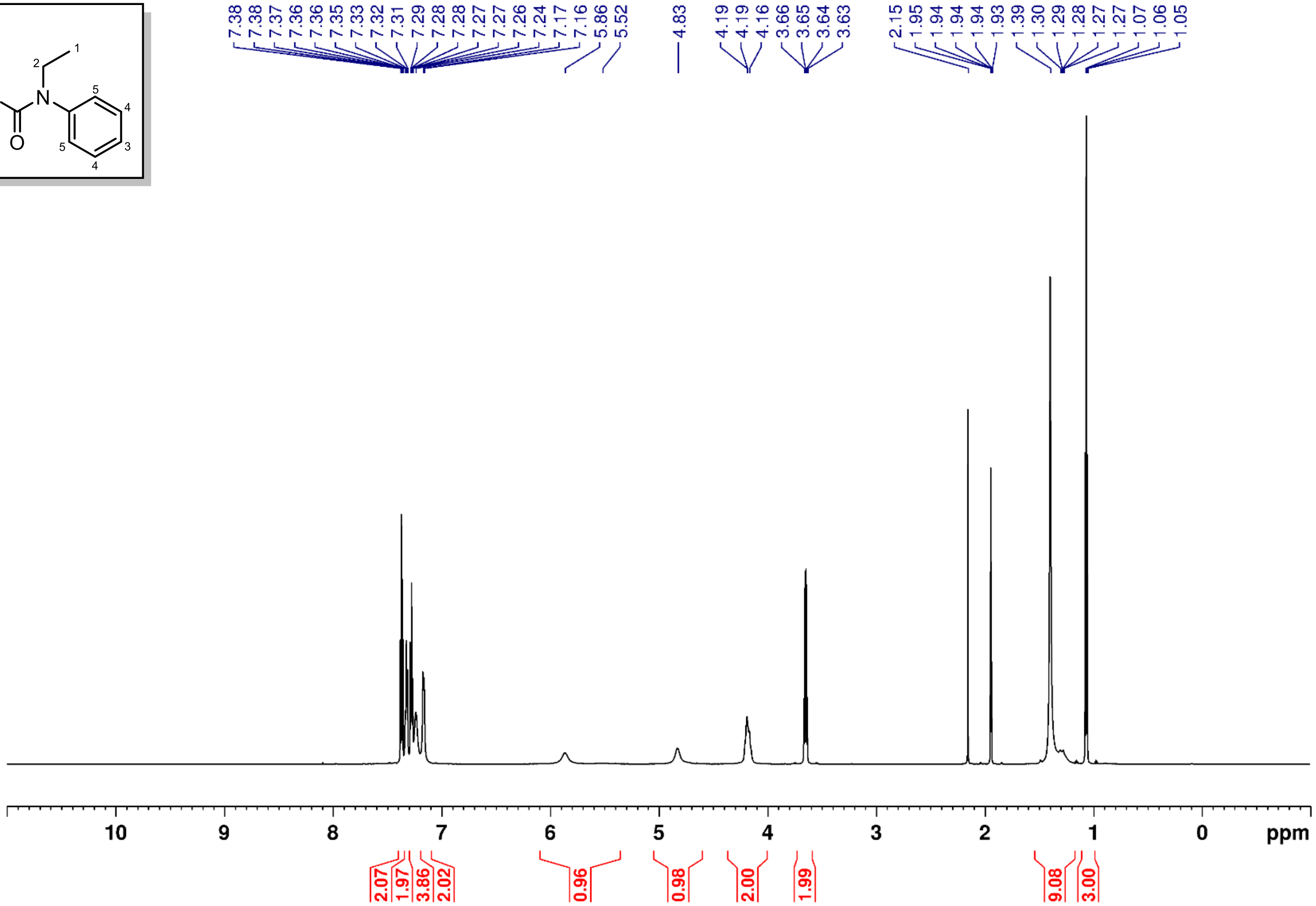

^{13}C NMR (176 MHz, CD_3CN) for (*S*)-2-((tert-butoxycarbonyl)amino)-2-phenylethyl ethyl(phenyl)carbamate (**14**)

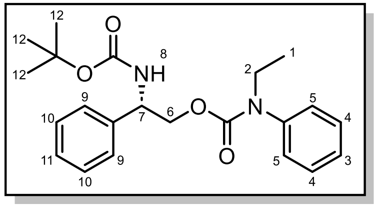

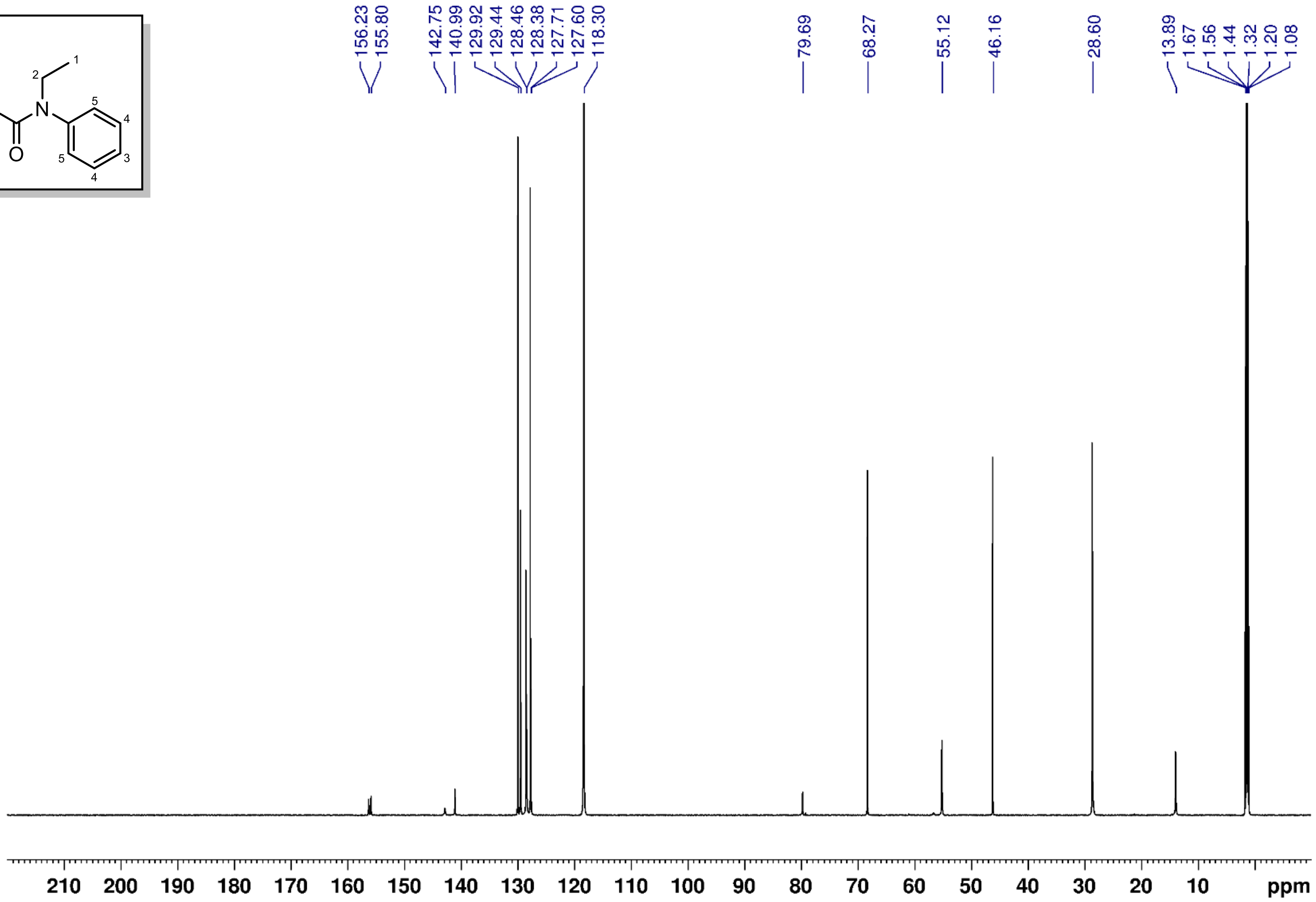

NMR Spectra for Products to Determine Absolute Stereochemistry

^{1}H NMR (700 MHz, CD_3OD) for (4*S*,5*R*)-5-(hydroxymethyl)-4-phenyloxazolidin-2-one

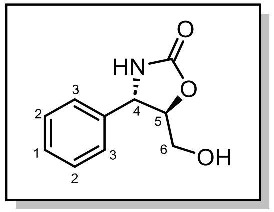

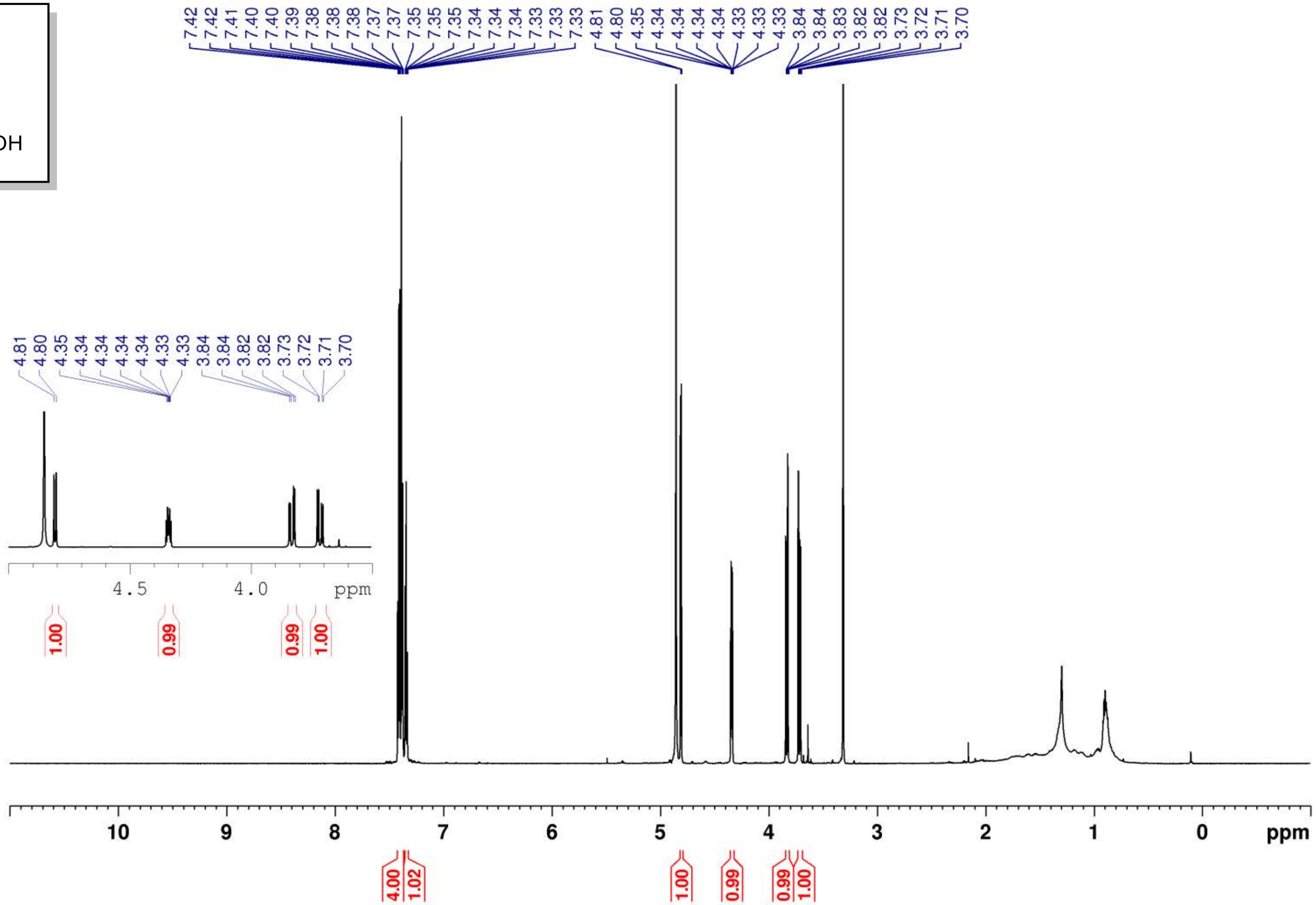

^{13}C NMR (176 MHz, CD$_3$OD) for (4*S*,5*R*)-5-(hydroxymethyl)-4-phenyloxazolidin-2-one

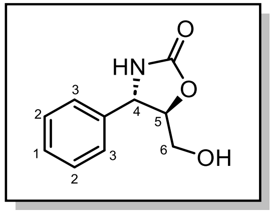

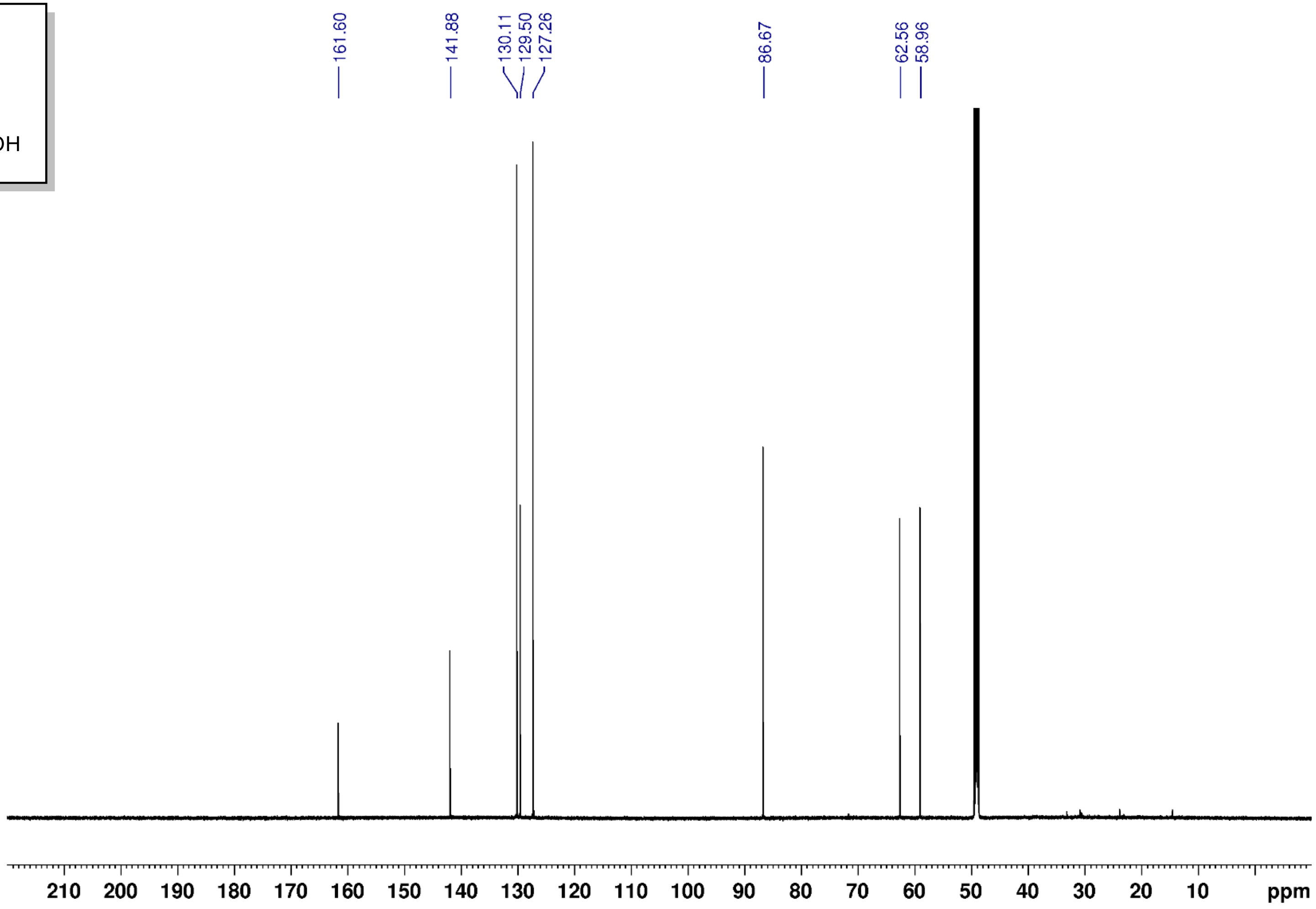

^{1}H NMR (500 MHz, CD_3CN) for 2,2,3,3,3-pentafluoropropyl ((1*S*,2*S*)-2-hydroxy-2,3-dihydro-1*H*-inden-1-yl)sulfamate (**S9**)

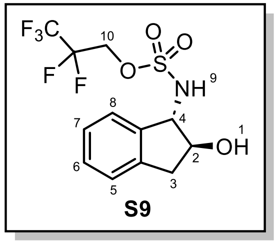

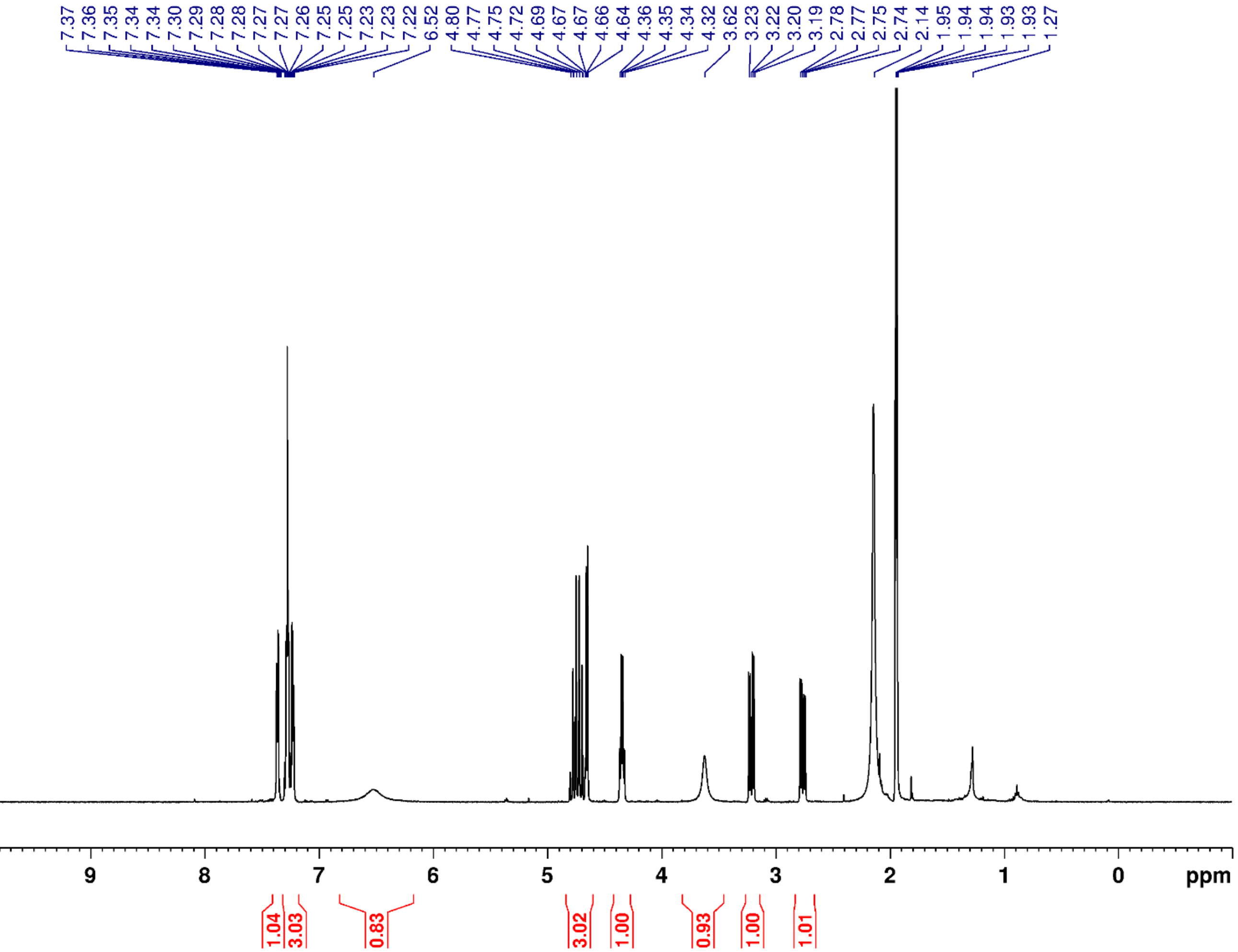

^{1}H NMR (700 MHz, CD_3CN) for 2,2,3,3,3-pentafluoropropyl ((1*R*,2*R*)-2-hydroxy-2,3-dihydro-1*H*-inden-1-yl)sulfamate (***ent*-S9**)

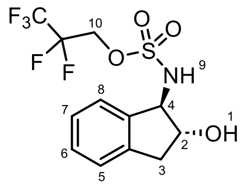

***ent*-S9**

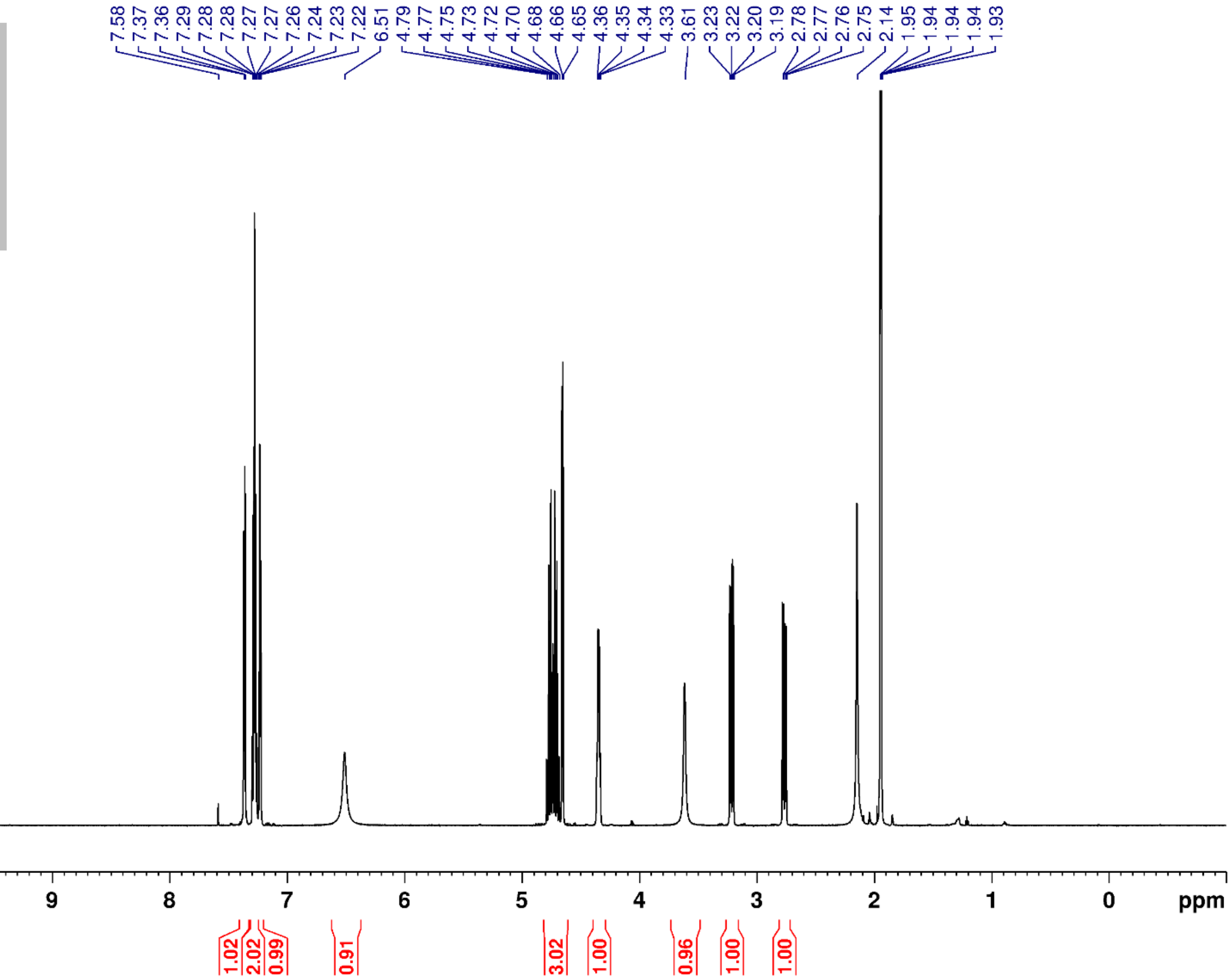

^{13}**C NMR (126 MHz, CD_3CN)** for 2,2,3,3,3-pentafluoropropyl ((1*S*,2*S*)-2-hydroxy-2,3-dihydro-1*H*-inden-1-yl)sulfamate (**S9**)

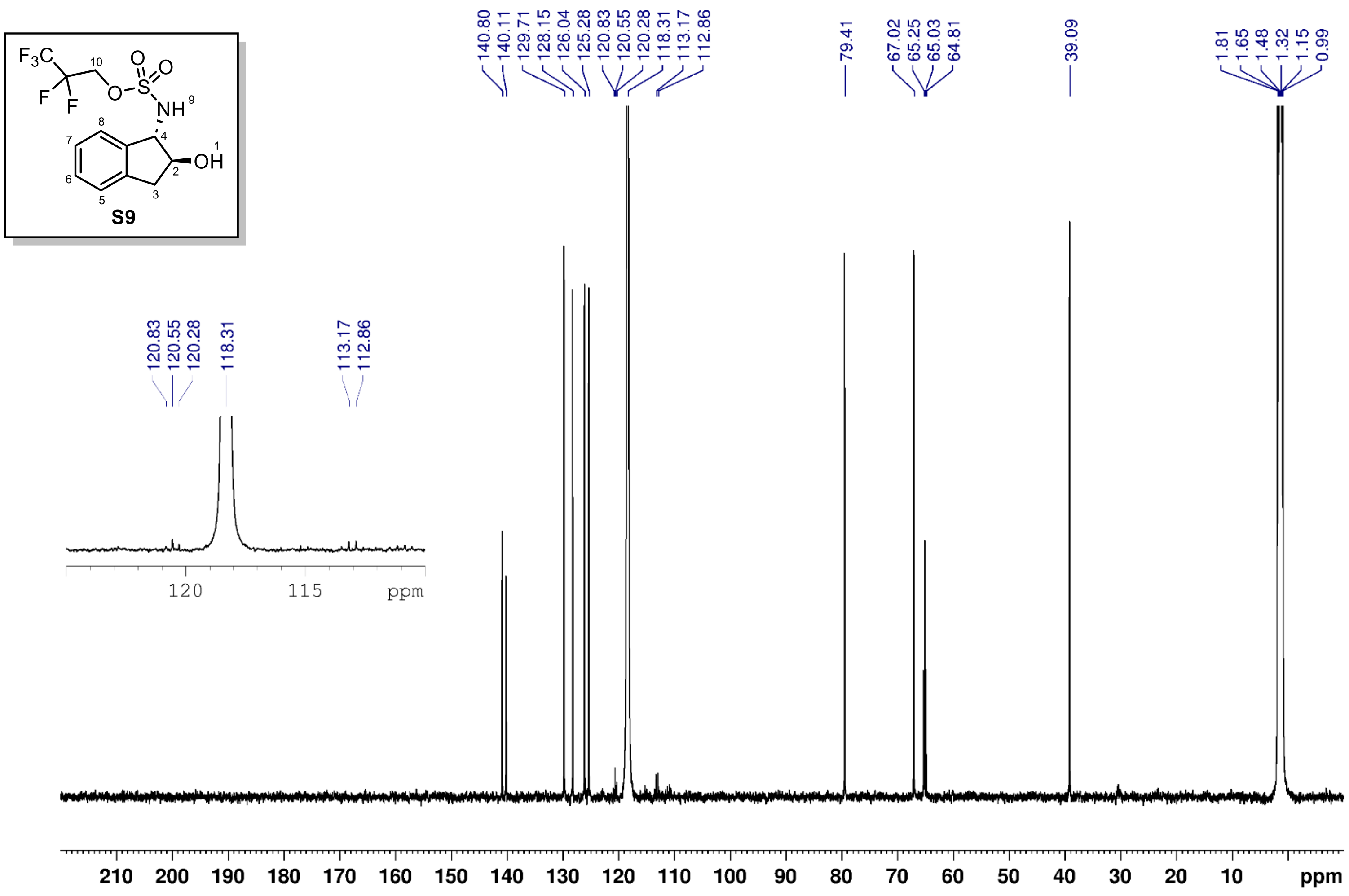

^{13}C NMR (176 MHz, CD_3CN) for 2,2,3,3,3-pentafluoropropyl ((1*R*,2*R*)-2-hydroxy-2,3-dihydro-1*H*-inden-1-yl)sulfamate (***ent*-S9**)

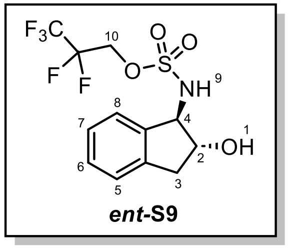

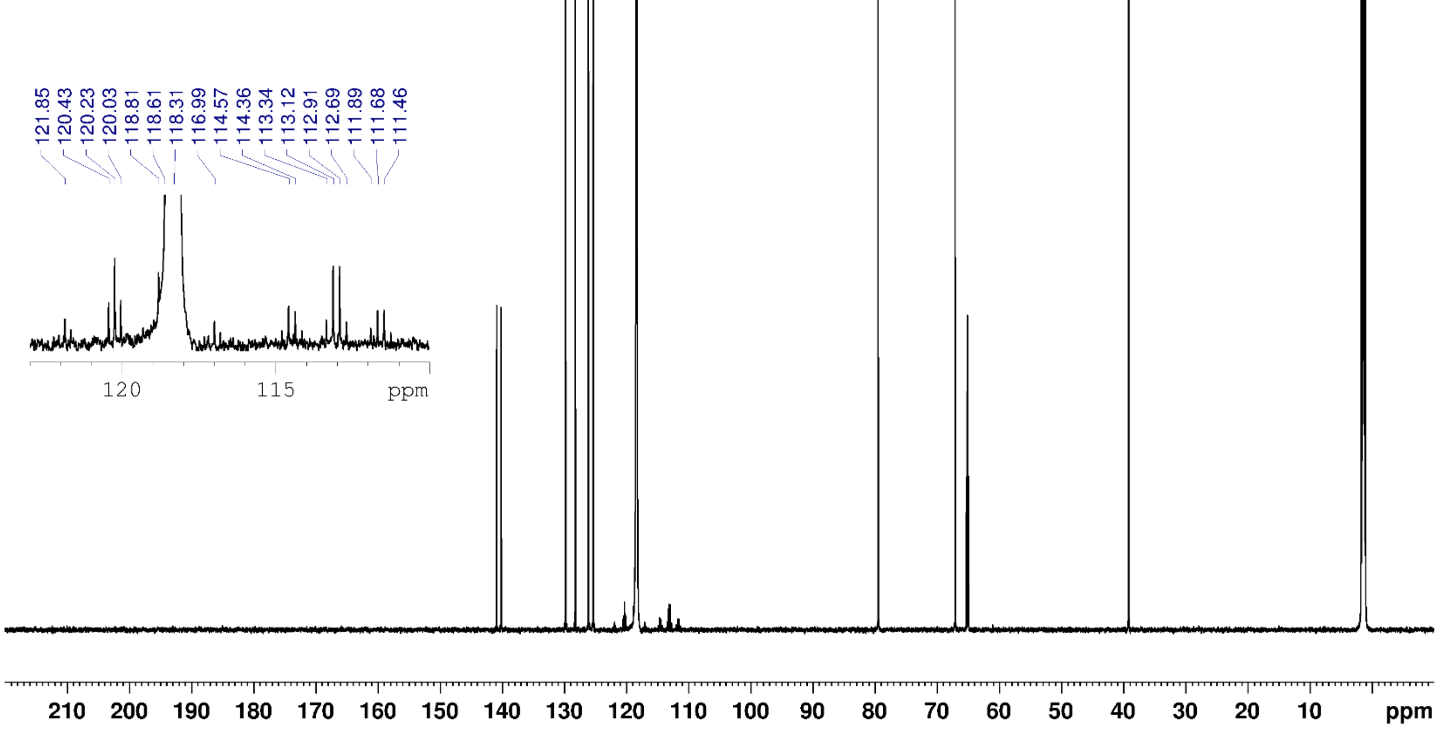

^{19}F NMR (376 MHz, CD_3CN) for 2,2,3,3,3-pentafluoropropyl ((1*S*,2*S*)-2-hydroxy-2,3-dihydro-1*H*-inden-1-yl)sulfamate (**S9**)

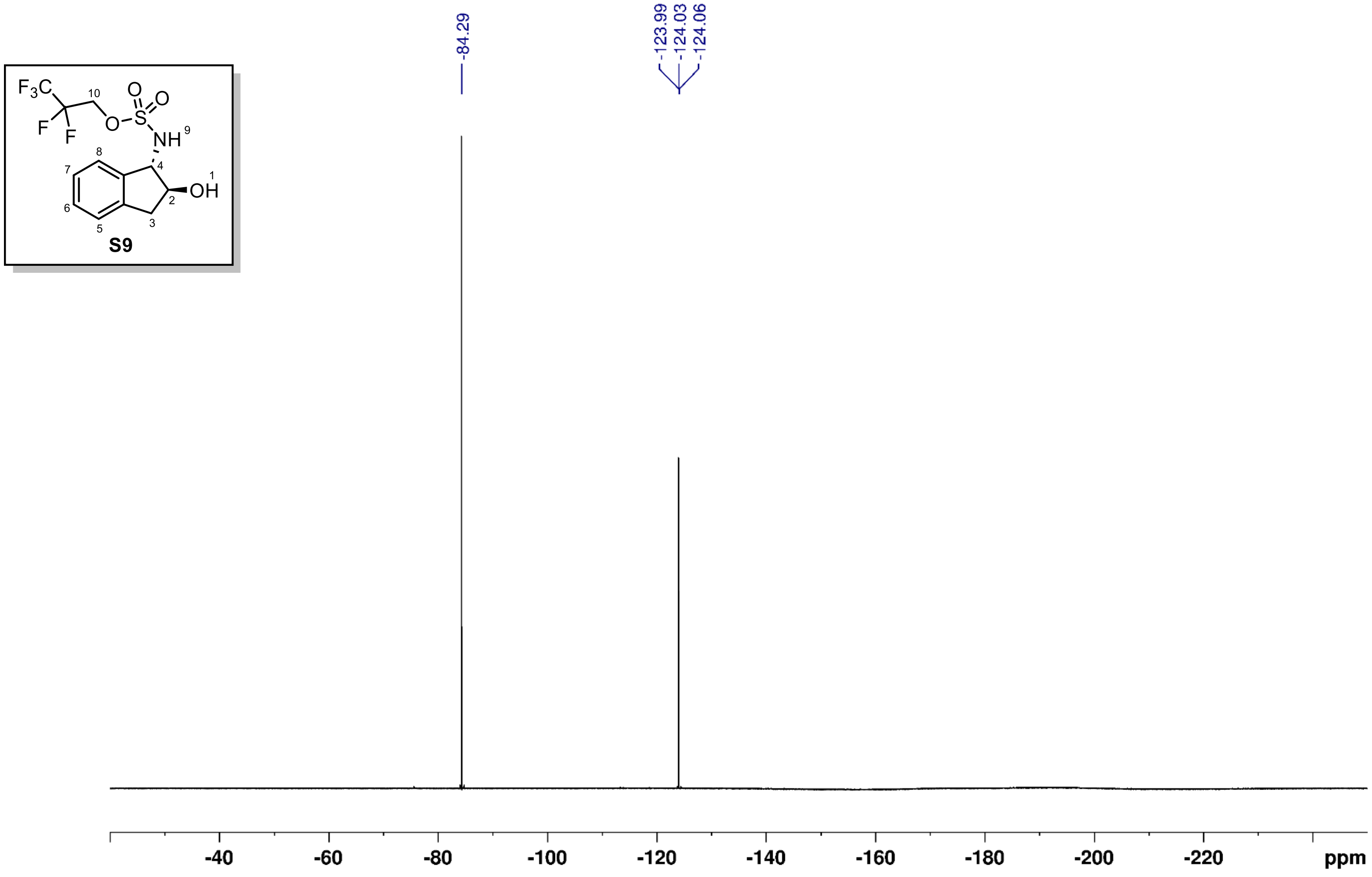

^{19}F NMR (376 MHz, CD_3CN) for 2,2,3,3,3-pentafluoropropyl ((1*R*,2*R*)-2-hydroxy-2,3-dihydro-1*H*-inden-1-yl)sulfamate (***ent*-S9**)

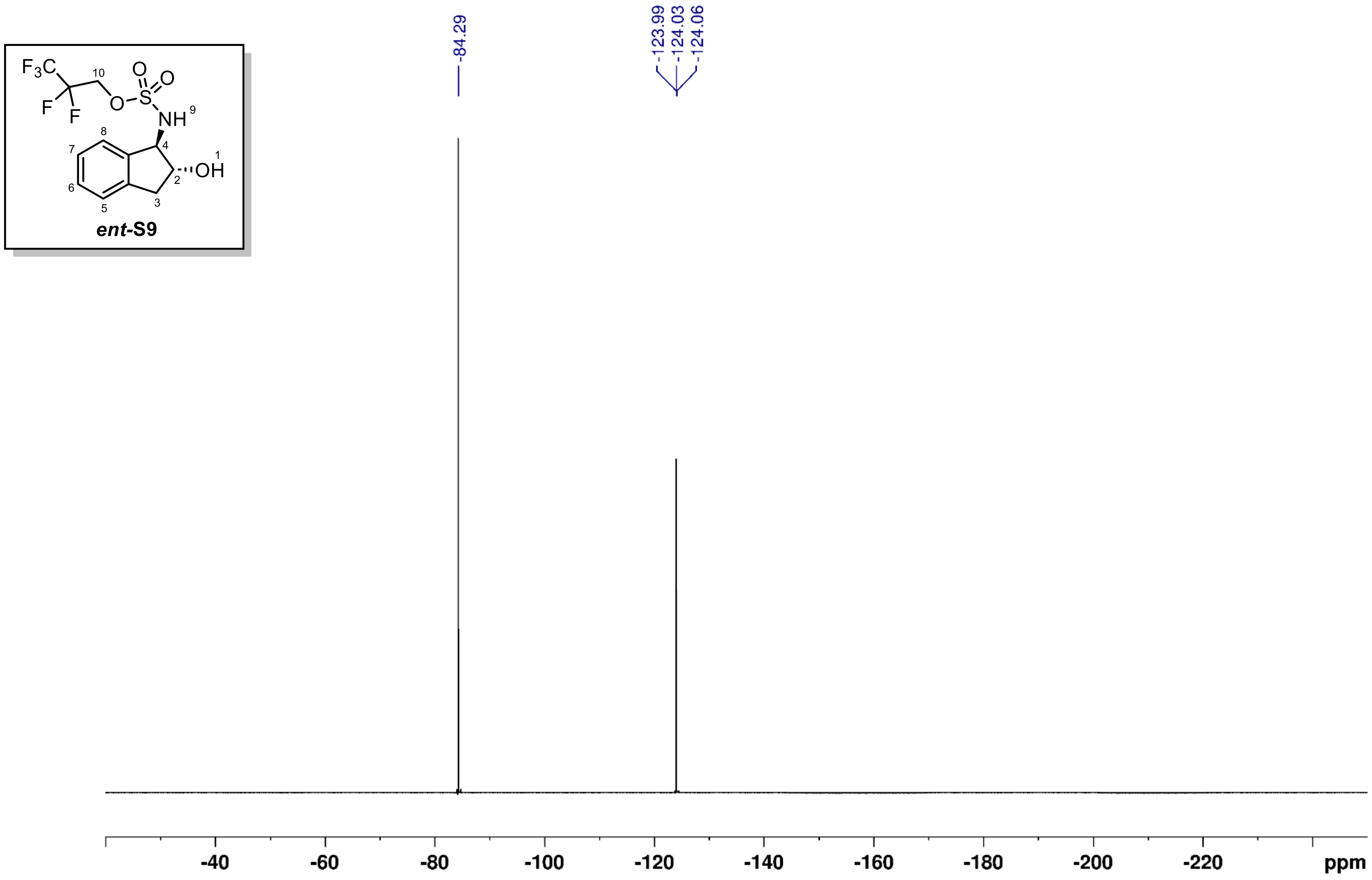

¹H NMR (700 MHz, CD_3CN) for 2,2,3,3,3-pentafluoropropyl ((1*S*,2*R*)-2-hydroxy-2,3-dihydro-1*H*-inden-1-yl)sulfamate (**S10**): 95% purity

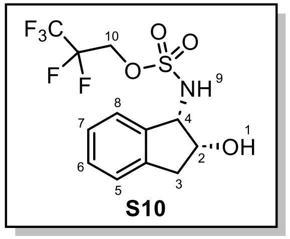

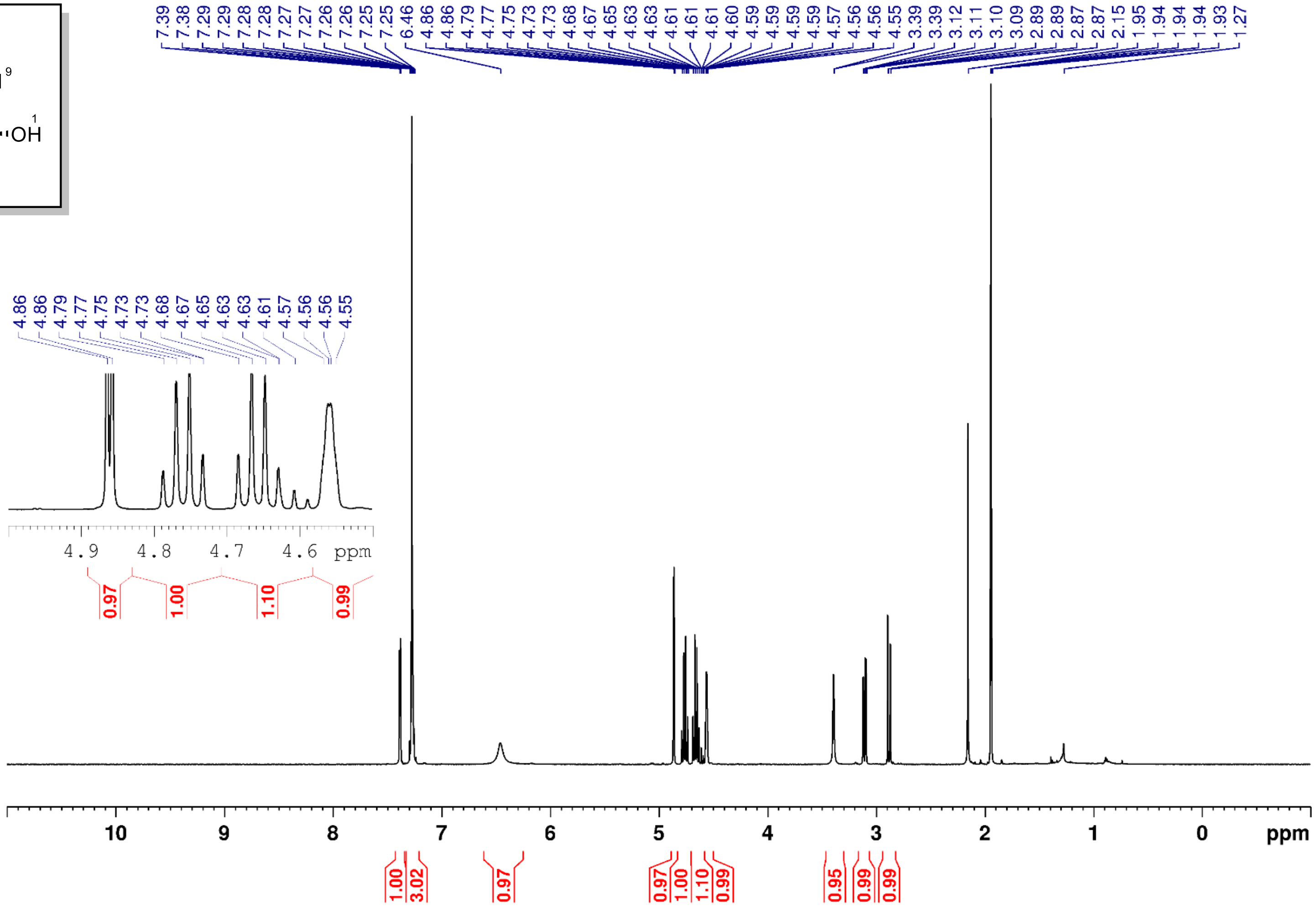

^{13}C NMR (176 MHz, CD_3CN) for 2,2,3,3,3-pentafluoropropyl ((1*S*,2*R*)-2-hydroxy-2,3-dihydro-1*H*-inden-1-yl)sulfamate (**S10**): 95% purity

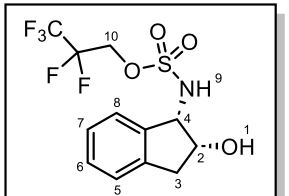

S10

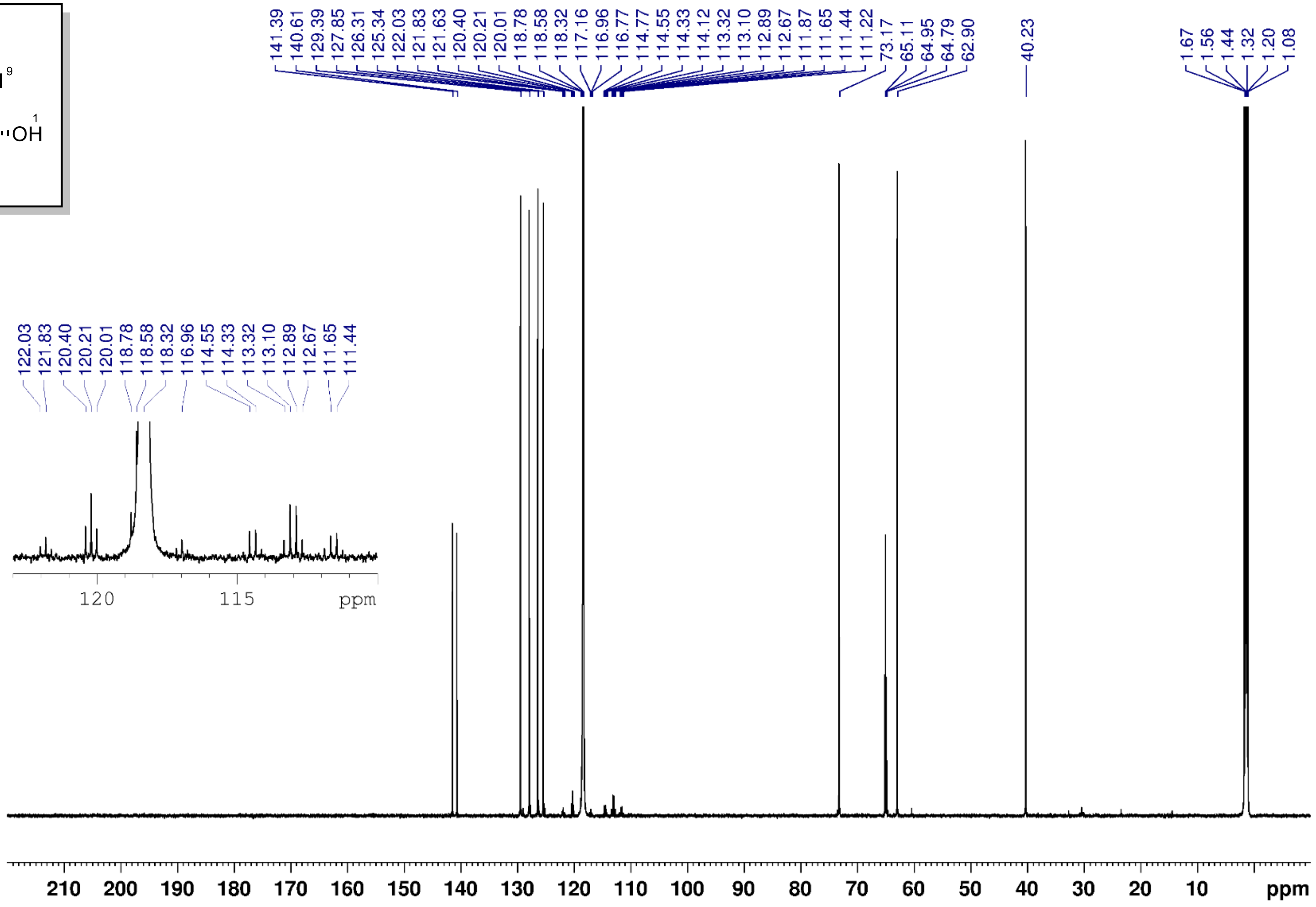

^{19}F NMR (376 MHz, CD_3CN) for 2,2,3,3,3-pentafluoropropyl ((1*S*,2*R*)-2-hydroxy-2,3-dihydro-1*H*-inden-1-yl)sulfamate (**S10**): 95% purity

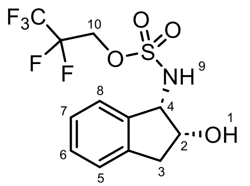

S10

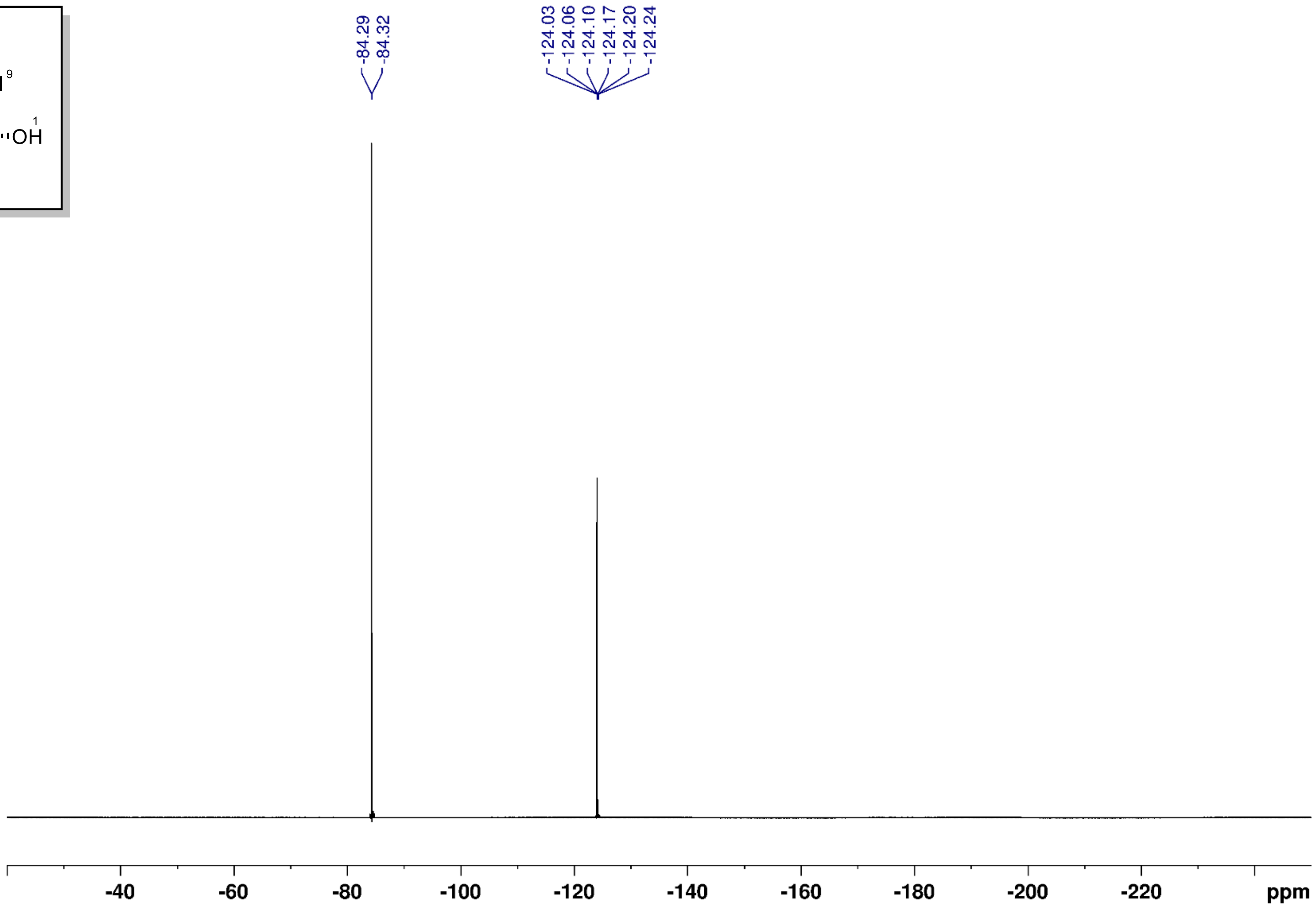

Supplement: Supplementary file 1 — Supporting Information [file ANIE-64-e202507532-s001.pdf]
